# A systematic review and meta-analysis of the association between e-cigarette use among non-tobacco users and initiating smoking of combustible cigarettes

**DOI:** 10.1186/s12954-024-01013-x

**Published:** 2024-05-22

**Authors:** Mimi M. Kim, Isabella Steffensen, Red Thaddeus D. Miguel, Tanja Babic, Julien Carlone

**Affiliations:** Thera-Business, Kanata, ON Canada

**Keywords:** E-cigarettes, Cigarette smoking, Smoking initiation, Regular smoking, Progression to smoking, Meta-anlaysis

## Abstract

**Introduction:**

The rapid increase in e-cigarette use over the past decade has triggered an important public health question on the potential association between e-cigarette use and combustible cigarette smoking. Following AMSTAR 2 and PRISMA guidelines, this evidence synthesis sought to identify and characterize any associations between e-cigarette use among individuals not smoking cigarettes and initiation of cigarette smoking.

**Methods:**

The protocol was registered on September 24, 2018 (PROSPERO 2018 CRD42018108540). Three databases were queried from January 01, 2007 to April 26, 2023. Search results were screened using the PICOS review method.

**Results:**

Among 55 included studies (40 “good” and 15 “fair”; evidence grade: “high”) that adjusted for gender, age, and race/ethnicity between groups, generally, there was a significant association between non-regular e-cigarette use and initiation of cigarette smoking, further supported by the meta-analytic results (AOR 3.71; 95% CI 2.86–4.81). However, smoking initiation was most often measured as ever/current cigarette smoking. Two studies (quality: 2 “good”) evaluated progression to regular cigarette smoking among individuals with regular use of e-cigarettes, and generally found no significant associations. One study (“good”) evaluated smoking initiation among individuals with regular use of e-cigarettes, finding an increasing probability of ever smoking cigarettes with increased e-cigarette use. Twelve studies (10 “good” and two “fair”) examining progression to regular smoking among individuals with non-regular use of e-cigarettes reported inconsistent findings.

**Conclusions:**

Numerous methodological flaws in the body of literature limit the generalizability of these results to all individuals who are not smoking cigarettes with few studies measuring established/regular use/smoking of e-cigarettes and cigarettes. Further, studies did not control adequately for specific confounding variables representing common liabilities between e-cigarette use and cigarette smoking, nor did they account for sufficient follow-up durations. Collectively, these flaws limit the generalizability of findings to the question of an association between e-cigarette use and cigarette smoking initiation.

**Supplementary Information:**

The online version contains supplementary material available at 10.1186/s12954-024-01013-x.

## Implications

In order to support robust determinations regarding e-cigarette use and the initiation of—or progression to—cigarette smoking, future research should apply measures of e-cigarette and cigarette use in a manner consistent with examining true initiation (i.e., established and/or regular use, as opposed to ever or current use), increase follow-up durations to adequately evaluate progression to regular smoking, and sufficiently account for known or suspected confounding variables that would represent common liabilities between e-cigarette use and cigarette smoking.

**Supplementary Information:**

The online version contains supplementary material available at 10.1186/s12954-024-01013-x.

## Introduction

Empirical evidence suggests e-cigarette aerosol does not contain most of the approximately 7000 chemicals present in cigarette smoke [1, 2]. However, with the decline in cigarette smoking prevalence, there has been a parallel increasing prevalence in electronic cigarette (e-cigarette) use [3–6].

The potential association between e-cigarette use and cigarette smoking is an important public health issue [7–9]. Understanding the individual and population level impact of e-cigarettes requires an objective synthesis of the empirical evidence that informs on the potential association between e-cigarette use and subsequent cigarette smoking and the inherent risks to health presented by e-cigarettes themselves [2]. Among the public health concerns of the use of e-cigarettes is the question of youth who may transition from e-cigarettes to cigarette smoking [2]. Hence, an assessment of causality is central to understanding the public health effect of e-cigarettes.

The Common Liability model is an important consideration when assessing causality between e-cigarette and cigarette smoking, particularly among tobacco non-users [10, 11]. Specifically, the common liability model posits that risks associated with using different substances can be explained by identifying common predisposing factors that also influence use behaviors [10, 11]. According to this model, where risk-taking propensities and psychosocial processes can be factors that link patterns of multiple addictions, common liability can provide a parsimonious explanation of substance use and addiction co-occurrence [11]. Thus, narrowly focusing on the association between e-cigarette use and subsequent cigarette smoking without consideration of potential common liability factors limits an inference of causality [12].

The current systematic review and meta-analysis evaluated potential associations between e-cigarette use among tobacco non-users and cigarette smoking initiation, applying a level of methodological rigor not previously reported in other reviews. Based on a general understanding of the available published literature on e-cigarette use and cigarette smoking, a priori outcome measures included: age at initiation of smoking combustible cigarettes; percent who initiated smoking combustible cigarettes; and initiation and progression to regular smoking of combustible cigarettes. Study design was not limited in the inclusion criteria. While previous systematic reviews have examined the relationship between e-cigarette use and the onset of cigarette smoking in youth and young adults [3, 13–17], as well as in the general population [18, 19], this review specifically focused on initiation of and progression to regular cigarette smoking—an outcome measure unique to this systematic review. Furthermore, given the rapid rate of emerging evidence on e-cigarette use, this review provides an important timely evidence synthesis to previous reviews.

## Methods

### Overview

The methods and results reported here correspond to a larger systematic review addressing the key research question, “Are there any potential associations between e-cigarette use among non-tobacco users and intention to smoke combustible cigarettes or initiating smoking of combustible cigarettes?” The focus of the findings reported here is the identification and characterization of any potential associations between e-cigarette use among non-tobacco users and the *initiation* of cigarette smoking.

The review protocol was registered with PROSPERO (The International Prospective Register of Systematic Reviews) on September 24, 2018 (PROSPERO 2018 CRD42018108540; http://www.crd.york.ac.uk/PROSPERO/display_record.php?ID=CRD42018108540).

This review strictly followed standards of systematic review methodology (“high” overall rating by A MeaSurement Tool to Assess systematic Reviews [AMSTAR] 2) [20] and reporting (Preferred Reporting Items for Systematic Reviews and Meta-Analyses [PRISMA]) [21].

### Terminology

Specific terminology in this review are fully reported in Supplemental Section 1: Terminology.

### Literature search methods

MEDLINE (Medical Literature Analysis and Retrieval System Online), EMBASE (Excerpta Medica Database), and PsycINFO were the database sources for the literature search. Applying search terms developed using medical subject headings (MeSH) and text words related to the associations between e-cigarette use and cigarette smoking intention and initiation, a full literature search was executed by an information specialist. Search dates were restricted to 2007 onwards due to the mass market introduction of e-cigarettes in the US [1, 2] (Supplemental Section 2: Literature Search Strategy).

The screening process was executed according to the PICOS (Population or participants and conditions of interest, Interventions or exposures, Comparisons or control groups, Outcomes of interest, and Study designs) review method (Supplemental Section 3: Inclusion/Exclusion Criteria) [22]. The population of interest—tobacco non-users—without restriction by age. The interventions and controls were individuals using e-cigarettes and non-users, respectively. Outcome measures identified a priori included: age of initiation for cigarette smoking, initiation of cigarette smoking, and initiation and progression to regular cigarette smoking (not included in previously published systematic reviews [3, 13]). Given the limited available evidence from randomized controlled trials (RCTs), this review was not limited by study design. The search strategy included: published peer-reviewed literature; theses and dissertations; government and industry documents; clinical trial registries (clinicaltrials.gov); gray literature in Google Scholar; consideration of reference lists across included studies; and content expert consultation. Studies were restricted to English-only publications.

Although the established/regular e-cigarette use provides the strongest evidence measure of sustained use behaviors, this review did not restrict use criteria. Additionally, studies were not restricted to those controlling for specific confounding variables that would represent common liabilities between e-cigarette use and cigarette smoking. The current review focused on studies that adjusted for *at least* the confounders of age, gender, and race/ethnicity.

### Evidence synthesis

Two reviewers independently screened articles based on the inclusion/exclusion criteria at the title/abstract level and then, full-text for studies not excluded based on the title/abstract alone. Data extraction was first conducted by one reviewer and then checked by a second reviewer. Across all levels of review and data extraction, discrepancies were resolved through discussion between the two reviewers and included a third team member when adjudication was necessary. All data were extracted and recorded in the DistillerSR platform (Evidence Partners, Ottawa, Canada) [23].

Estimates of the difference between individuals using e-cigarettes and individuals who are not using e-cigarettes are presented with the best measures of precision (i.e., 95% confidence intervals [CIs]) and/or statistical significance (i.e., *p* value) reported in the included studies. Reporting references to “significant” and/or “significantly” are only used to indicate statistical significance (i.e., *p* < 0.05 and/or CI excludes 1.0). The DerSimonian–Laird method was used to conduct random-effects meta-analyses where included studies were weighted by the inverse of the sum of within-study variance plus between-study variance [24]. The Cochran’s Q statistic assessed heterogeneity across pooled studies which was then quantified using the inconsistency index (I^2^).

Study authors were contacted to obtain missing data. All meta-analytic data were analyzed through Review Manager version 5.3 [25], in Windows 10 Pro version 22H2.

### Sensitivity analyses

Data permitting, sensitivity analyses were planned to include stratification of results (or removal of data inputs) from: studies that did not adjust for meso- and macro-level variables in addition to age, race/ethnicity, and gender; studies that did not define e-cigarette use or regular cigarette smoking; and studies with a questionable definition of e-cigarette use and/or regular cigarette smoking. Additionally, data permitting, stratification by age group, and a sensitivity analysis of age, was planned. A sub-group analysis for the meta-analysis based on the country where the study was implemented, and a sensitivity analysis excluding studies graded as “Fair,” was likewise planned.

### Assessment of confounding

This review applied the Socio-Ecological Model as defined by McLeroy et al. [26] to guide consideration of the interrelationships between individuals and their social (micro-), physical (meso-), and policy (macro-) environments (further detail reported in Supplemental Section 4: Conceptual Framework).

Evaluation of confounding factors was followed according to Cochrane guidelines for systematic reviews [27]; specifically, during protocol writing, a list of potential confounding factors was identified a priori based on evidence and expert opinion from members of the research team and external advisors; and during the systematic review process, the variables that individual study authors considered were recorded for additional post hoc consideration.

### Outcomes and related psychometrics

Recognizing that not all the outcome measures are equally valid and reliable, this review examined the Contextual Question (CQ): “Have measures used to examine initiation and progression to regular cigarette smoking been psychometrically assessed as reliable and valid?” Specific criteria were applied to assess reliability and validity across the outcome measures [28] (full reporting in Supplemental Section 5: Contextual Questions).

### Study quality assessment

Two reviewers independently appraised study quality using the Downs and Black checklist. Individual studies were graded as either “excellent,” “good,” “fair,” or “poor” [29] (Full reporting in Supplemental Section 6: Study Quality Assessment). A funnel plot was planned to test for the risk of publication bias if 10 or more studies provided estimates pooled in the meta-analysis.

### Strength of evidence evaluation

Strength of evidence (SOE) was assessed for studies that controlled for age, gender, and race/ethnicity and those that did not control for key confounders. The overall SOE was graded as “high,” “moderate,” “low,” or “insufficient” using the Agency for Healthcare Research and Quality (AHRQ) Evidence Based Practice (EPC) grading system [30] (full reporting in Supplemental Section 7: Strength of Evidence).

### Consideration of industry funding bias

The potential impact of funding bias on results and conclusions has been a topic addressed in the evidence base [31–33]. As indicated in the conflict-of-interest disclosure for this review, and given the recent increase of peer-reviewed systematic reviews and meta-analyses, this topic with potential industry and public health impact may have a heightened importance as a methodological issue. To specifically address any potential concerns of funding bias in this reported evidence synthesis, this review was executed with the highest standards of the systematic review methodology including: *a* priori protocol registration (PROSPERO 2018 CRD42018108540; http://www.crd.york.ac.uk/PROSPERO/display_record.php?ID=CRD42018108540); strict adherence to the PICOS throughout the execution of this review; a transparent and replicable search strategy executed by an information specialist with corresponding literature research results (Supplemental Section 8: Literature Search Output, Studies Reviewed at the Full-Text Level); full reporting of excluded studies including reason for exclusion (Supplemental Section 9: List of Excluded Studies); full reporting details on quantitative methods; and the expected details, per AMSTAR-2 and PRISMA guidelines, to disseminate a fully transparent and replicable evidence synthesis. Overall, the methodological rigor of this review with fully transparent and replicable reporting can also serve as a measure to minimize publication bias with systematic reviews.

## Results

### Overview

The initial database search (January 1, 2007 to August 31, 2018) yielded 2526 articles, with four additional articles identified through other sources [3, 34–36], resulting in 2530 articles. The first updated literature search (January 1, 2018 to August 30, 2019) yielded 1525 articles with 307 duplicate articles due to applied overlapping timeframes between the two searches. This overlapping timeframe conducted searches from the first of the year; therefore, overlapping search timeframes were unavoidable. Additionally, two articles were identified through other sources [37, 38], resulting in 1220 unique articled retrieved. A second updated literature search for the timeframe of January 1, 2019 to October 7, 2020 yielded 2211 articles, of which 595 were duplicate articles with the previous database search, resulting in 1616 unique articles retrieved. A third updated search for the January 1, 2020 to November 24, 2021 timeframe yielded 3245 articles, of which 935 were duplicate articles with the previous database search, resulting in 2310 unique articles retrieved. Finally, a fourth updated search for the January 1, 2021 to April 26, 2023 period yielded 3925 articles, of which 1420 were duplicate articles with the previous database search, resulting in 2505 unique articles retrieved.

**Fig. 1:**
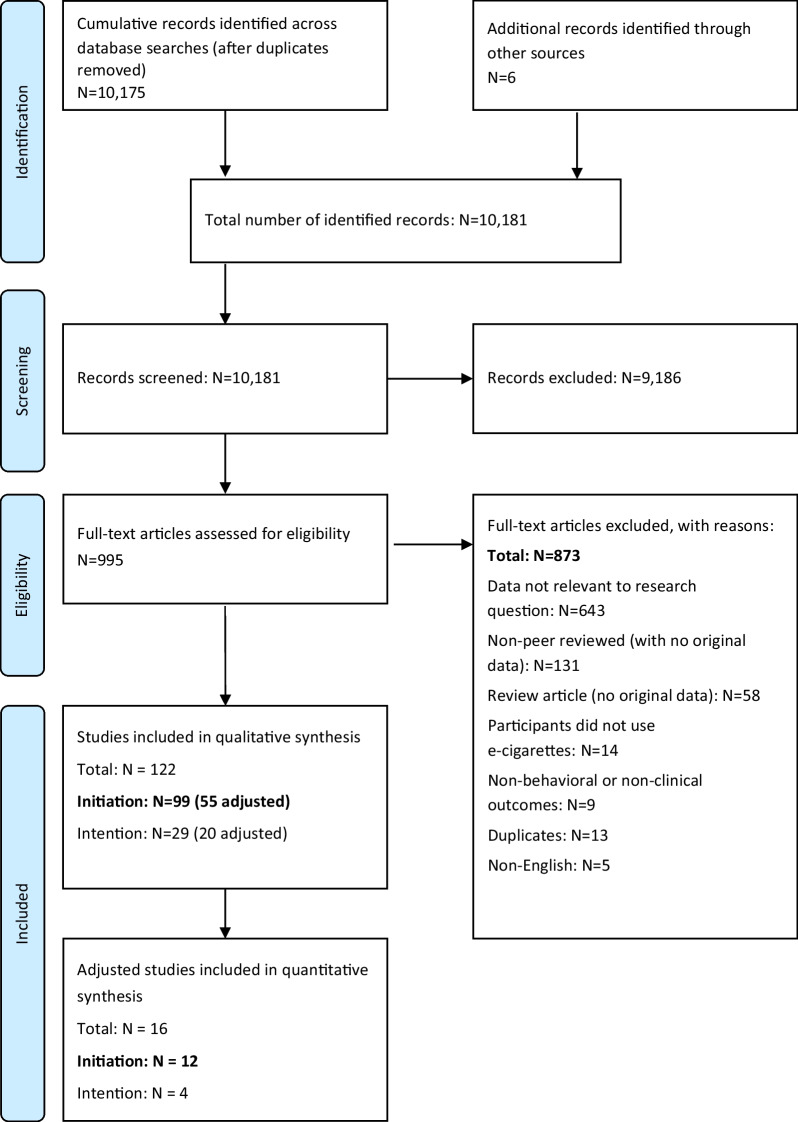
PRISMA flowchart

A cumulative total of 10,175 articles were retrieved from the specified databases, with an additional six additional articles identified from other sources (total: 10,181). Of the 10,181 potentially relevant articles, 9186 were excluded at the title/abstract level, resulting in 995 articles eligible for review at full-text level (Supplemental Section 8: Literature Search Output, Studies Reviewed at the Full-Text Level). Subsequently, a further 873 articles were excluded (Supplemental Section 9: List of Excluded Studies), resulting in 122 studies eligible for inclusion in the larger systematic review (Supplemental Section 10: List of Included Studies). Inter-rater reliability at Level 2 screening was considered substantial or near perfect agreement [39] across all literature searches with a weighted overall kappa ranging from 0.72 to 0.95 (refer to Fig. 1 for each level of screening).

Of the 122 studies identified in the systematic review, 99 studies reported on cigarette smoking initiation or progression and were eligible for the qualitative and quantitative evidence. Of these 99 studies, 55 reported results that were adjusted for gender, age, and race/ethnicity between groups. For each included study, data were extracted on: study characteristics (Supplemental Section 11: Study and Sample Characteristics, Adjusted Studies), demographic and baseline characteristics (Supplemental Section 12: Demographic and Baseline Characteristics, Adjusted Studies), and study outcomes (Supplemental Section 13: Evidence Tables, Adjusted Studies). Studies reporting unadjusted results are presented in Supplemental Section 14 (Study and Sample Characteristics, Unadjusted Studies), Supplemental Section 15 (Study and Sample Characteristics, Unadjusted Studies), and Supplemental Section 16 (Evidence Tables, Unadjusted Studies), but are not included in the qualitative or quantitative synthesis of evidence.

The highest number of studies (10 studies) were published in both 2020 [40–49] and 2018 [50–59]; followed by 7 studies in each of 2021 [60–66], 2019 [37, 67–72], and 2017 [34, 36, 73–77]; six studies in 2022 [78–83]; three studies in each of 2023 [84–86] and 2015 [87–89]; and two studies in 2016 [90, 91]. Studies were predominantly longitudinal in design and were from registered surveys. Of the 55 included studies, 41 were conducted in the US [36, 40, 41, 43–49, 51–54, 56, 58, 59, 62–65, 67–72, 75–79, 81–83, 86–91], five in the UK [34, 37, 61, 66, 85], two in Canada [60, 73], one study in each of Mexico [74], Netherlands [57], Netherlands and Belgium [84], Romania [55], South Korea [42], Switzerland [50], and Thailand [80]. In terms of the study population, four studies defined their study population as “adults” [40, 48, 69, 72], one study stratified their results by youth and adult populations [43]; three studies defined their participants as 12 years or older [59, 81, 86]. For the remaining 47 studies that defined participants, respondents were categorized as “youth,” “adolescents,” or “young adults” (participants defined as “students” were between grade 6 and college level) [34, 36, 37, 41, 42, 44–47, 49–58, 60–68, 70, 71, 73–80, 82–85, 87–91].

In addition to age, sex, and race/ethnicity, most studies included further adjustments with varying combinations of other micro, meso, and macro covariates. However, none of the studies sufficiently adjusted for potential confounding variables that would represent common liabilities between e-cigarette use and cigarette smoking [92]—meaning that a bias for those predisposing elements would exist among individuals using e-cigarettes that would likely be unadjusted for in the included studies.

Initiation of cigarette smoking was evaluated by the largest number of included studies (49 adjusted studies) [34, 36, 37, 41–44, 46, 47, 49–64, 66–68, 70–74, 76–91], followed by initiation and progression to regular cigarette smoking (12 adjusted studies) [37, 40, 45, 48, 54, 61, 65, 66, 69, 72, 73, 86]. One adjusted study examined the potential relationship between e-cigarette use and age of initiation for cigarette smoking [75].

The reliability and validity of each outcome measure were evaluated according to the CQ with a comprehensive but not systematic review of the literature. The objective in doing so was to provide fuller context for the interpretation of findings from the evidence synthesis. All measures were single-item measures related to the initiation and/or progression of cigarette smoking. All three measures of initiation were supported by empirical data regarding their reliability and/or validity, and therefore qualified as “acceptable”—including initiation of cigarette smoking, age of initiation for cigarette smoking, and initiation and progression to regular cigarette smoking (full reporting in Supplemental Section 5: Contextual Questions).

Quality appraisal for each included study was conducted by two reviewers according to the Downs and Black checklist [29]. Forty (73%) were rated “good” quality [34, 37, 40, 41, 44–49, 51–62, 64–68, 72–78, 84, 86–88, 90, 91], 15 (27%) were rated “fair,” [36, 42, 43, 50, 63, 69–71, 79–83, 85, 89] and no studies were rated “excellent” or “poor” (Supplemental Section 6: Study Quality Assessment). Publication bias was assessed using funnel plots, and no publication bias was detected.

The overall SOE among the adjusted data regarding the association between e-cigarette use and age of initiation of cigarette smoking was graded “moderate”; the body of evidence specific to e-cigarette use and initiation of cigarette smoking was graded “high”; and the body of evidence specific to initiation and progression to regular cigarette smoking was graded “moderate.” The SOE domain score table and the SOE and CQ ratings summary table for both the adjusted and unadjusted data are presented in Supplemental Section 7: Strength of Evidence.

### Definitions of e-cigarette use by outcome measure

Among the 55 included studies, one evaluated age of cigarette smoking initiation [75], 42 evaluated initiation of cigarette smoking [34, 36, 41–44, 46, 47, 49–53, 55–60, 62–64, 67, 68, 70, 71, 74, 76–85, 87–91], five evaluated progression to regular smoking [40, 45, 48, 65, 69], and seven studies evaluated both initiation of cigarette smoking and progression to regular smoking [37, 54, 61, 66, 72, 73, 86].

Among the 49 studies that examined initiation of cigarette smoking, only one evaluated the association between regular e-cigarette use and initiation of cigarette smoking. Wills et al. [77] defined e-cigarette use on a frequency scale (1–2 times ever use, 3–4 times ever use, yearly/monthly, and weekly/daily), with the initiation of cigarette smoking defined as having “ever smoked a whole cigarette”. For the remaining studies that examined initiation of cigarette smoking among individuals with non-regular use of e-cigarettes, ever use was the most common measure of both e-cigarette use (39 studies) [34, 37, 42–44, 46, 47, 49, 52, 54–57, 59, 61, 63, 64, 67, 68, 70–72, 74, 76–81, 84–90, 93] and cigarette use (33 studies) [34, 37, 42–44, 46, 47, 49, 52, 55–58, 61, 63, 64, 66–68, 70–74, 76–80, 84, 87, 89, 91], with current or past 30-day use being the second most common measure (16 studies for e-cigarette use [36, 41, 52–54, 58, 60, 62, 67, 72, 73, 78–80, 86, 91] and 23 studies for cigarette use [36, 41, 43, 47, 52–54, 58–60, 62–64, 68, 74, 76, 78–81, 85, 90, 93]). The most commonly evaluated relationship for these two tobacco use behaviors was between ever use of e-cigarettes and ever use of cigarettes (30 studies) [34, 37, 42–44, 46, 47, 49, 52, 55–57, 61, 63, 64, 66–68, 70–72, 74, 76–80, 84, 87, 88]. Ever use of e-cigarettes and current use of cigarettes was the second most commonly evaluated relationship (17 studies) [43, 47, 52, 54, 59, 63, 64, 68, 74, 76, 78–81, 85, 90, 93], followed by current use of e-cigarettes and current use of cigarettes (11 studies) [36, 41, 52–54, 58, 60, 62, 78–80].

Twelve studies examined the association between e-cigarette use and initiation of and progression to regular cigarette smoking [37, 45, 48, 54, 61, 65, 66, 69, 72, 73, 86, 94]. All of these 12 studies evaluated the association between non-regular e-cigarette use and progression to regular cigarette smoking. Additionally, two of the 12 studies also evaluated the association between regular e-cigarette use and progression to regular cigarette smoking [40, 48]. Azagba et al. [94] defined e-cigarette use as either every day (current daily use and having ever used fairly regularly), some day (current use and having ever used fairly regularly), or experimental (current use and never having used fairly regularly), with progression to regular cigarette smoking defined as transitioning from either current non-established to current-established cigarette smoking, current non-established to current daily-established cigarette smoking, or current-established to current daily-established cigarette smoking [40]. Among individuals with established (having ever used fairly regularly) e-cigarette use, Wei et al. [48] evaluated transitions from non-current, non-established cigarette smoking to either exclusive current-established cigarette smoking or current-established dual use of cigarettes and e-cigarettes.

For the 12 studies that used definitions of non-regular e-cigarette use, e-cigarette use was defined as follows: current or past-30-day use in two studies [45, 73]; e-cigarette experimentation, defined as non-established use (less than 100 times during lifetime) in one study [69]; and ever use of e-cigarettes in four studies [37, 61, 65, 66]. Three studies applied multiple definitions of non-regular e-cigarette use: Chaffee et al. [54] included ever, past 30-day, and former e-cigarette use; Sun et al. [86] included ever and past 30-day use, while McMillen et al. [72] included ever and past 30-day e-cigarette use. Two studies that evaluated regular e-cigarette use also evaluated non-regular use defined as experimental use [40, 48].

In the one study that evaluated age of initiation of cigarette smoking, e-cigarette use was defined as current use [75].

### Qualitative synthesis of best available evidence

Fifty-five studies adjusted for three main confounders (gender, age, and race/ethnicity) between groups, and were analyzed in the qualitative review and quantitative syntheses reported below. Results for each outcome measure in the qualitative analysis were stratified by regular versus non-regular e-cigarette use.

Adjusted data for age of initiation, initiation of cigarette smoking, and progression to regular smoking are provided in Supplemental Section 13: Evidence Tables, Adjusted Studies. Unadjusted data for age of initiation of cigarette smoking, initiation of cigarette smoking, and initiation and progression to regular cigarette smoking are provided in Supplemental Section 16: Evidence Tables, Unadjusted Studies; however, unadjusted data are not included in qualitative analysis.

#### Age of initiation of cigarette smoking (regular e-cigarette use)

No studies provided adjusted analyses of *age of initiation of cigarette smoking* among individuals with regular use of e-cigarettes.

#### Age of initiation of cigarette smoking (non-regular e-cigarette use: 1 study)

One adjusted study was identified that investigated the association between non-regular e-cigarette use and age of initiation of cigarette smoking [75] (Summary characteristics of this study are provided in Table 1). In a cross-sectional analysis, McCabe et al. [75] reported that the adjusted odds of smoking the first cigarette at an earlier age (Grade 8 or below) were significantly higher among individuals using e-cigarettes (current [past-30-day]) versus individuals who are not using e-cigarettes (adjusted odds ratio [AOR] 4.12, 95% CI 2.56–6.62). Further, the odds of an earlier age of onset of daily cigarette smoking (before 8th grade level) were not significantly different between individuals currently using e-cigarettes (past-30-day) and individuals who are not using e-cigarettes (AOR 1.67, 95% CI 0.385–7.25) [75].

**Table 1:** Summary characteristics of adjusted studies for age of initiation of cigarette smoking among individuals with non-regular use of e-cigarettes (n = 1)

Citation	Study design	Definition of individuals who use e-cigarettes	Definition of individuals who do not use e-cigs	Definition of initiation of cigarette smoking
McCabe et al. [75]	Cross-sectional analytic	Individuals not smoking cigarettes with current e-cig use defined as response of ≥ 1 to question, “During the last 30 days, on how many occasions (if any) have you used e-cigs?”	Individuals not smoking cigarettes with response of 0 to question, “During the last 30 days, on how many occasions (if any) have you used e-cigs?”	Age in yrs given in response to the question “When, if ever did you smoke your first cigarette?” Age in yrs given in response to question, “When, if ever, did you smoke cigarettes on a daily basis?”

*e-cigs* electronic cigarettes, *yrs* years

#### Initiation of cigarette smoking (regular e-cigarette use: 1 study)

One adjusted study was identified that investigated the association between regular e-cigarette use and odds of initiation of cigarette smoking among individuals not smoking cigarettes at baseline[77] (Summary characteristics of this study are provided in Table 2).

**Table 2:** Summary characteristics of adjusted studies for initiation of cigarette smoking among individuals using e-cigarettes regularly (n = 1)

Citation	Study design	Definition of individuals who use e-cigarettes	Definition of individuals who do not use e-cigs	Definition of initiation of cigarette smoking
Wills et al. [77]	Longitudinal panel	Answered “Yes” when asked if having ‘ever used’ an e-cig and giving the use frequency ‘I have smoked e-cigs 1–2 times’, ‘I have smoked e-cigs 3–4 times’, ‘I usually smoke a few e-cigs a year’, ‘I usually smoke a few e-cigs a month’, ‘I usually smoke a few e-cigs each week’, and ‘I usually smoke e-cigs everyday’ who were also individuals who never smoked cigarettes (“Not having smoked a cigarette in lifetime”)	Answered “I have never smoked an e-cigarette in my life” to the question “which of the following is most true for you about smoking electronic cigarettes?”	Individuals not smoking cigarettes at Time 1 who reported smoking ≥ 1 cigarette at Time 2 by answering “I have smoked cigarettes 1–2, 3–4 times,” “usually smoke every week,” “usually smoke every day” to question, “which of the following is most true for you about smoking?”

*e-cigs* electronic cigarettes, *yrs* years

In their study of 1070 individuals who never smoked cigarettes at baseline, Wills et al. [77] examined the association between e-cigarette use and initiation of cigarette smoking by stratifying the probability of smoking onset by frequency of e-cigarette use at baseline, including a measure of regular (weekly/daily) e-cigarette use. Compared with individuals who are not using e-cigarettes, all individuals who have used e-cigarettes had significantly higher adjusted odds of initiating cigarette smoking: individuals who ever used e-cigarettes (1–2 times): AOR 2.88 (95% CI 1.96–4.22); individuals who ever used e-cigarettes (3–4 times): AOR 2.29 (95% CI 1.35–3.87); weekly/daily users: AOR 4.09 (95% CI 2.43–6.88); and yearly/monthly users: AOR 4.17 (95% CI 2.03–8.57).

#### Initiation of cigarette smoking (non-regular e-cigarette use: 49 studies)

Forty-nine adjusted studies examined the association between non-regular e-cigarette use and *initiation of cigarette smoking* among individuals not smoking cigarettes at baseline [34, 36, 37, 41–44, 46, 47, 49–64, 66–68, 70–74, 76–91]. Summary characteristics of these 49 studies are provided in Table 3.

**Table 3:** Summary characteristics of adjusted studies for initiation of cigarette smoking among individuals using e-cigarettes regularly (n = 49)

Citation	Study design	Definition of individuals who use e-cigarettes	Definition of individuals who do not use e-cigs	Definition of initiation of cigarette smoking
Akre et al. [50]	Longitudinal panel	Individuals who use e-cigarettes: any e-cig use in the past 12 months	Individuals who have not used e-cigs: no e-cig use in the past 12 months	Individuals who never smoked cigarettes at Time 0 reporting smoking cigarettes at Time 1 (12 months)
Aleyan et al. [60]	Prospective cohort	Individuals who never smoked cigarettes at baseline reporting any past 30-day use of e-cigs	Individuals who never smoked cigarettes at baseline reporting no past 30-day use of e-cigs	Individuals who never smoked cigarettes at Wave 1 initiating cigarette smoking at Wave 3:
				Past 30-day e-cig use at Wave 1 or Wave 2 and past 30-day cigarette smoking at Wave 3; Past 30-day e-cig use at Wave 1 or Wave 2 and past 30-day dual use at Wave 3.
Auf et al. [67]	Retrospective cohort	E-cig initiators: Response of “yes” to a question inquiring whether “e-cigarette was the first tried tobacco product” (i.e., individuals who never smoked cigarettes prior to first e-cig use) Individuals who use e-cigarettes: Ever users: self-reported any lifetime use of e-cig by individuals who never smoked cigarettes Current users: self-reported past-30-day use of e-cig by individuals who never smoked cigarettes;	Individuals who have not used e-cigs: Self-reported never use of e-cig (and any other tobacco product) by individuals who never smoked cigarettes	Self-reported initiation of cigarettes via e-cig: Individuals who ever smoked cigarettes: any lifetime use Current cigarette users: past-30-day use
Barrington-Trimis et al. [52]	Prospective cohort [NB: Three pooled prospective cohorts]	Individuals who use e-cigarettes: Individuals reporting either an age of first e-cig use, or an ever use (“even one or two puffs”) were classified as ever use of e-cig Individuals who use e-cigarette exclusively: Individuals self-reporting past-30-day e-cig use at baseline, and no past-30 day cig use at baseline Neither product user: Individuals self-reporting no past-30-day use of either e-cig or cigarettes	Individuals who have not used e-cigs: Individuals that reported to have never tried (not “even one or two puffs”) an e-cig	Self-reported cigarette use:
				Experimental: Ever use, but not in the past 30 days Infrequent users: 1–2 days in the past 30 days Frequent users: 3 or more days in the past 30 days Exclusive cigarette user: individuals self-reporting past-30-day cigarette use, and no past-30 day e-cig use Individuals using e-cigs and smoking cigarettes: Individuals self-reporting past-30-day use of both e-cig and cigarettes Neither product user: Individuals self-reporting no past-30-day use of either e-cig or cigarettes
Barrington-Trimis et al. [52]	Prospective cohort	Ever users: any who have reported an “age of first use” of e-cigs who are individuals who never smoked cigarettes	Never use of e-cigs	Baseline never-smokers reporting smoking at follow up
Barrington-Trimis et al. [90]	Prospective cohort	Individuals who use e-cigarettes: Individuals not smoking cigarettes reporting an age at first use of e-cigs were classified as “ever users”	Individuals who have not used e-cigs: Individuals not smoking cigarettes who had never tried an e-cig (“not even 1 or 2 puffs”)	Reporting at 2015 follow-up the use of cigarettes among individuals not smoking cigarettes at baseline
Berry et al. [68]	Prospective cohort	Individuals who use e-cigarettes in the past: Tobacco-naïve individuals at Wave 1 reported ever use of e-cigs between Waves 1 and 3, which preceded ever use of any other tobacco product	Individuals who have not used e-cigs: Tobacco-naïve individuals at Wave 1 reported never use of e-cigs (or any other non-cigarette tobacco product) between Waves 1 and 3	Self-reported cigarette use at Wave 3: Individuals who ever smoked cigarettes: any lifetime use (even 1 or 2 puffs)
				Current cigarette users: past-30-day use
				*Youth reporting any of the following were considered intermediate or high risk: ever alcohol use, ever marijuana use, prescription drug abuse, enjoying frightening things, liking new and exciting experiences, preferring unpredictable friends, willingness to smoke in next yr, curiosity about cigarettes, or susceptibility to cigarette peer pressure from friends. Youths who did not exhibit any risk behaviors, sensation-seeking personality traits, or cigarette susceptibility were considered low risk
Best et al. [34]	Prospective cohort	Individuals not smoking cigarettes who answered, “Tried once or twice,” “More than once a month but less than a week,” or “More than once a week” to question, “Which one of the following is closest to describing your experience of e-cigarettes/vapourisers/shisha pens?”	Individuals not smoking cigarettes who answered, “Never tried, not even once” to question, “Which of the following is closest to describing your experience of e-cigarettes/vapourisers/shisha pens?”	Smoking ≥ 1 puff of a cigarette at 1 yr follow-up
Bold et al. [53]	Longitudinal panel	Individuals not smoking cigarettes who answered any use to, “How many days out of the past 30 days did you use e-cigarettes?” (open-ended response, 0–30)	Individuals not smoking cigarettes who answered “0” days to, “How many days out of the past 30 days did you use e-cigarettes?”	Individuals not smoking cigarettes having ever tried a cigarette, even 1 or 2 puffs in the past 30 days, at follow up
Chaffee et al. [54]	Prospective cohort	Ever user: Individuals who never smoked cigarettes and lifetime use of e-cigs Past-30-day users: individuals who never smoked cigarettes and use of e-cigs in the past 30 days Former user: individuals who never smoked cigarettes and ever use of e-cigs but no use in the past 30 days	Never use of e-cigs	Individuals reporting one of the following outcomes at 1 yr follow-up: 1. Smoking during the past 30 days, 2. having smoked 100 cigarettes at 1 yr follow-up, 3. both outcomes together
Conner et al. [37]	Prospective cohort	Individuals who use e-cigarettes: Individuals who never smoked cigarettes at baseline reporting ever use of e-cigs (“I have tried them once or twice,” “I use them sometimes [more than once a month but less than once a week],” or “I use them often [more than once a week]”);	Individuals who have not used e-cigs: Individuals who never smoked cigarettes reporting never having used e-cigs at baseline	Ever smoker: self-reported ever-use of cigarettes (even once) at follow-up
Conner et al. [61]	Prospective cohort	*Individuals with early e-cig use:* Baseline (Wave 3) individuals who never smoked cigarettes who reported ever use of e-cigs *Individuals with late e-cig use:* Baseline (Wave 3) individuals who never smoked cigarettes and individuals who never used e-cigarettes, who reported never smoking and ever e-cig use at Wave 4	Individuals who never smoked cigarettes reporting never having used e-cigs at baseline	Baseline (Wave 3) individuals who never smoked cigarettes who reported cigarette ever use at follow-up (Wave 4 or Wave 5)
Duan et al. [62]	Prospective cohort	Individuals who never smoked cigarettes at baseline reporting past 30-day e-cig use	Individuals who never smoked cigarettes at baseline reporting no past 30-day e-cig use	Individuals who never smoked cigarettes at baseline who self-reported any past 30-day smoking at follow-up; Individuals who never smoked cigarettes at baseline who ever smoked cigarettes, even one or two puffs at follow-up
Epstein et al. [49]	Prospective cohort	Individuals who do not smoke cigarettes at baseline who responded “1 and higher occasions” to “on how many occasions have you used electronic cigarettes or e-cigs (‘Vapes’), such as Ruyan or NJOY in the past 12 months?	Individuals who do not smoke cigarettes at baseline who responded “0 occasions” to “on how many occasions have you used electronic cigarettes or e-cigs (‘Vapes’), such as Ruyan or NJOY in the past 12 months?”	Individuals who do not smoke cigarettes at baseline (who never used cigarettes more than “once or twice” and never used 100 cigarettes in their lifetime) at any Wave through age 21, who used cigarettes on 3 or more occasions at age 23
Evans-Polce et al. [41]	Longitudinal panel	Individuals who use e-cigarettes: Individuals who never smoked cigarettes reporting past 30 day e-cig use	Non-e-cig user: Individuals who never smoked cigarettes reporting no past 30 day e-cig use	Individuals who never smoked cigarettes at baseline reporting past 30 day or past 12 month cigarette use at follow-up
Hair et al. [63]	Prospective cohort	*Individuals who have ever used e-cigs:* Individuals who never smoked cigarettes at baseline who responded “yes” to “have you ever used or tried any e-cigs?” *Ever JUUL user:* Individuals who never smoked cigarettes at baseline who responded “yes” to “have you ever used or tried any e-cigs?”, and “yes” to “have you ever used a JUUL vape?” *Ever e-cig (non-JUUL) user:* Individuals who never smoked cigarettes at baseline who responded “yes” to “have you ever used or tried any e-cigs?”, and “no” to “have you ever used a JUUL vape?”	Individuals who never smoked cigarettes at baseline who reported never use of e-cigs	Individuals who never smoked cigarettes at baseline (spring 2017) reporting cigarette smoking in 2019.
				Individuals who ever smoked cigarettes: ever tried cigarettes, even 1 or 2 puffs;
				Current cigarette users: any past-30-day use of cigarettes
Hammond et al. [73]	Prospective cohort	Users at baseline (use of e-cig products in the last 30 days) and cigarette never-smokers (“Never having tried cigarette smoking”)	Never used e-cigs at baseline and never-smokers	Never smoked a whole cigarette at baseline and smoked a whole cigarette at 1-yr follow up
Harlow et al. [78]	Prospective cohort	Individuals who have ever used e-cigs: Individuals who never smoked cigarettes at Wave 2 and e-cig never-users at Wave 1 who reported ever e-cig use (even once or twice) at Waves 2, 3, and 4 Individuals who have ever used e-cigs were divided into the following groups: Individuals currently using e-cigs: Individuals who have ever used e-cigs who reported current (i.e., past 30-day) e-cig use at a follow-up wave Former e-cig users: Individuals who have ever used e-cigs who did not use e-cigs in the past 30 days at a follow-up wave Tobacco flavored e-cig users: Individuals who have ever used e-cigs who initiated e-cig use with tobacco-flavored e-cigs Nontobacco flavored e-cig users: Individuals who have ever used e-cigs who initiated e-cig use with nontobacco flavored e-cig, such as menthol, mint, clove, spice, fruit, chocolate, alcoholic drinks, candy, or other sweets	Individuals who never smoked cigarettes at baseline who never used e-cigs	Reported having ever smoked a cigarette, even one or two puffs at Waves 3, 4, and 5
Kang et al. [42]	Cross-sectional analytic	Individuals who never smoked cigarettes who reported ever using an e-cig at baseline	Individuals who never smoked cigarettes who reported never using an e-cig at baseline	Individuals who never smoked at baseline reporting ever cigarette ever use (one or two puffs) at end state
Kasza et al. [43]	Longitudinal panel	Individuals who use e-cigarettes: Individuals who never smoked cigarettes reporting ever ENDS use	Individuals who have not used e-cigs: Individuals who never smoked cigarettes reporting no ever use of ENDS	Individuals who never smoked cigarettes at baseline reporting past 30 day cigarette use at any follow-up
				Individuals who never smoked cigarettes at baseline reporting ever cigarette use at any follow-up
Keller-Hamilton et al. [64]	Prospective cohort	Individuals who responded “yes” to “have you ever used an e-cig, such as Smoking Everywhere, NJOY, Blu, or Vapor King, even one or two times?”	Never used e-cigs at baseline	Individuals who ever smoked cigarettes: ever tried cigarettes smoking even 1 or 2 puffs;
				Current cigarette users: smoked a cigarettes, even one or two puffs in the past 30 days; *Complete case analysis:* Individuals who were compliant at all five time points and were not missing data for variables in the propensity score model
Kintz et al. [44]	Prospective cohort	Individuals who use e-cigarettes: Individuals who never smoked cigarettes at baseline who reported an age of first use of e-cigs	Individuals who have not used e-cigs: Individuals who never smoked cigarettes at baseline and who reported having never used an e-cig	Individuals who report an age at first use of cigarettes at follow up
Kong et al. [70]	Prospective cohort [NB: Three pooled prospective cohorts]	Individuals who use e-cigarettes: Individuals who never smoked cigarettes at baseline reporting either an age of first e-cig use or an ever use (“even one or two puffs”) were classified as ever users of e-cig	Individuals who have not used e-cigs: Individuals who never smoked cigarettes at baseline reporting never having tried (not “even one or two puffs”) an e-cig	Individuals who reported a valid age in response to a question asking about the age of ever cigarette use (“even one or two puffs”) at follow-up* Follow-up for CHS and YASS was approximately 18 months; follow-up for H&H was approximately 12 months
Lee and Fry [71]	Prospective cohort	Individuals who use e-cigarettes: Individuals who never smoked cigarettes at baseline who reported baseline e-cig ever use	Non-e-cig user: Individuals who never smoked cigarettes at baseline who reported never use of e-cigs at baseline	Never use of cigarettes at Wave 1 followed by ever-use of cigarettes by Wave 2
Leventhal et al. [87]	Prospective cohort	Individual using e-cigs at baseline (lifetime and past 6-month use of e-cigs) and never tried cigarettes (“No, not even a few puffs”)	Individuals who never used e-cigs use and never tried cigarettes	Becoming an individual who ever smoked cigarettes was described as responses of “No” at baseline and “Yes” at follow-up periods to question “Have you ever used combustible cigarette” at 6- and 12-month follow-up
Loukas et al. [79]	Longitudinal	Individuals currently using e-cigs (ENDS): E-cig (ENDS) use on at least one day in the past 30 days Individuals who have ever used e-cigs (ENDS): Ever used an e-cig (ENDS) product as intended, even one or two puffs	Individuals not smoking cigarettes who were not “individuals who have ever used e-cigs” and were “non-current ENDS users”	Individuals who currently smoke: Smoked at least one day in the past 30 days
				Individuals who did not currently smoke: Reported ever smoking, but did not use cigarettes in the past 30-days
Lozano et al. [74]	Prospective cohort	Tried at least once in their lifetime and who are individuals not smoking cigarettes (“No” to question, “Have you ever tried or experimented with cigarette smoking, even one or two puffs?”)	Did not try e-cigs even once in their lifetime and who are individuals not smoking cigarettes	Trial of smoking at follow up: “Yes” to question, “Have you ever tried or experimented with cigarette smoking, even one or two puffs?”
				Current cigarette smoking: ≥ 1 cigarette in the past 30 days
Martinelli et al. [84]	Longitudinal panel	Reported ever using an e-cig	Reported never using an e-cig	Individuals who never smoked at baseline reporting ever use of cigarettes at 6-month and 12-month follow-ups
McMillen et al. [72]	Longitudinal panel	Individuals who have ever used e-cigs: Individuals who do not smoke cigarettes responding “yes” to question “Have you ever used an e-cigarette, such as NJOY, Blu, or Smoking Everywhere, even 1 or 2 times?” and responding “not at all” to question “Do you now use e-cigarettes every day, some days, or not at all?” Past 30-day e-cig users: Individuals who do not smoke cigarettes responding “yes” to question “Have you ever used an e-cigarette, such as NJOY, Blu, or Smoking Everywhere, even 1 or 2 times?” and responding “every day” or “some days” to question “Do you now use e-cigarettes every day, some days, or not at all?”	Individuals who have not used e-cigs: Individuals who have ever used e-cigs: Individuals who do not smoke cigarettes responding “no” to question “Have you ever used an e-cigarette, such as NJOY, Blu, or Smoking Everywhere, even 1 or 2 times?”	Individuals who never smoked cigarettes at baseline reporting having smoked a cigarette in the past 12 months at follow-up
Miech et al. [36]	Longitudinal panel	Individuals who use e-cigarettes; Individuals who never smoked cigarettes who answered “1 or more days” to question, “During the last 30 days, how many days (if any) have you used e-cigs?”	Individuals who have not used e-cigs: Individuals who never smoked cigarettes who answered “0” to question, “During the last 30 days, how many days (if any) have you used e-cigs?”	Reporting cigarette use at follow-up by responding with “smoked once or twice,” or more, to the question “what best describes your cigarette smoking in the last 12 months?”
Owotomo et al. [46]	Prospective cohort	Individuals who never smoked cigarettes at baseline who reported ever use of e-cigs (even one or two times)	Individuals who never smoked cigarettes at baseline who reported never use of e-cigs	Individuals who never smoked cigarettes at baseline who ever smoked cigarettes, even one or two puffs, in the past 12 months
Parnham et al. [85]	Longitudinal	Individuals who answered ‘Only tried once or twice’, ‘In the past but not now’, ‘Less than once a month’, ‘At least once a month but less than once a week’ and ‘At least once a week’ (and yes to ‘ever used e-cigs’ for wave 7) to the questions “have you ever used e-cigs” or “do you ever use e-cigs”	Individuals who answered ‘I have never’ to the questions “have you ever used e-cigs” or “do you ever use e-cigs”	Transition from ever having used an e-cig and never smoked, to currently smoking (past 30 days)
Patanavanich et al. [80]	Longitudinal cohort	Individuals currently using e-cigs: Individuals who used e-cigs during the last 30 days Individuals who have ever used e-cigs: Individuals who ever tried e-cigs Experimenters: Ever users who were not current users	Individuals who had never used e-cigs at baseline	Transition from never smoking cigarettes to combustible cigarette smoking and dual product use at the 12-month follow-up
Penzes et al. [55]	Prospective cohort	Individuals not smoking cigarettes and “Yes” to the question “have you ever tried e-cigs?”	Individuals not smoking cigarettes and “No” to question “Have you ever tried e-cigs?”	Individual who never smoked at baseline reporting at least 1 puff of cigarette at 6-months
Primack et al. [56]	Prospective cohort	Individuals using e-cigs at baseline (no frequency/intensity recorded) who are also individuals who never smoked cigarettes (no history of smoking) and non-susceptible to smoking (answered “Definitely no” to the questions “If one of your friends offered you a cigarette, would you try it?” and “Do you think you will smoke a cigarette sometime in the next year?”	No e-cig use at baseline who are also individuals who never smoked cigarettes and non-susceptible to smoking	Individuals who never smoked cigarettes at baseline with ≥ 1 puff of cigarette by follow-up
Primack et al. [88]	Longitudinal panel	E-cig use at baseline who are also never-smokers	No reported e-cig use at baseline among individuals who never smoked cigarettes	Individuals who smoked cigarettes regularly at 1 yr follow-up (no further details)
Primack et al. [89]	Prospective cohort	Individuals using e-cigs at baseline (no frequency/intensity recorded) who are also individuals who never smoked cigarettes (no history of smoking) and non-susceptible to smoking (answered “Definitely no” to the questions “If one of your friends offered you a cigarette, would you try it?” and “Do you think you will smoke a cigarette sometime in the next year?”	No e-cig use at baseline who are also individuals who never smoked cigarettes and non-susceptible to smoking	Individuals who never smoked cigarettes at baseline with ≥ 1 puff of cigarette at 18-month follow-up
Spindle et al. [76]	Longitudinal panel	Individuals who have ever used e-cigs: had used e-cigs at least “only once, even on one occasion” who are individuals who never smoked cigarettes Individuals currently using e-cigs: indicated use of e-cig in past 30 days who are individuals who never smoked cigarettes	No use of e-cigs with never smoking	Lifetime smoking follow-up: (“How many cigarettes have you smoked in your life?”);
				Current smoking at follow up: (“How many cigarettes have you smoked in the past 30 days?”) at 1-yr follow-up
Staff et al. [66]	Prospective cohort	Youth who, at age 14, never smoked cigarettes and reported ever trying an e-cigarette or vaping device	Youth who, at age 14, never smoked cigarettes and reported never trying an e-cigarette or vaping device	Individuals who never smoked cigarettes at age 14 who reported ever smoking by age 17
Stokes et al. [47]	Longitudinal panel	Individuals who use e-cigarettes: Cigarette naïve individuals reporting ever e-cig use, even one or two puffs	Individuals who have not used e-cigs: Cigarette naïve individuals reporting no ever e-cig use, even one or two puffs	Individuals reporting cigarette ever use (one or two puffs) or past 30-day use (any use of a cigarette within 30 days prior to outcome wave) at any follow-up
Sun et al. [81]	Longitudinal panel	Individuals who never smoked cigarettes at baseline reporting ever use of e-cigs (≥ 1) in the initial Wave in each pair of Waves	Individuals who never smoked cigarettes reporting never having used e-cigs at baseline	Individuals who never smoked cigarettes at baseline Wave reporting smoking behaviors (even one puff of cigarette) at the subsequent Wave
Sun et al. [86]	Longitudinal panel	Individuals who have ever used e-cigs: Cigarette-naïve individuals at baseline (Wave 3) who self-reported ever use of e-cigs Individuals currently using e-cigs: Cigarette-naïve individuals at baseline (Wave 3) who self-reported past 30 days e-cigs use	Cigarette-naïve individuals at baseline (Wave 3) who never used e-cigs	Individuals who never smoked cigarettes at baseline (Wave 3) who self-reported ever use of cigarettes in Wave 4 and continued cigarette use at Wave 5 among Wave 4 individuals who reported initiating cigarette smoking;
				Continued smoking measure (CSM) was defined as:
				CSM-I: Past 12-month cigarette use at wave 4 and past 12-month use at wave 5;
				CSM-II: Past 12-month cigarette use at Wave 4 and past 30-day use at wave 5
Treur et al. [57]	Cross-sectional analytic and prospective cohort	Ever-use of e-cigs at least once in their lifetime, with or without nicotine, and who have never smoked, or only tried once or twice	Never use of e-cigs in their lifetime, and who have never smoked, or only tried once or twice	Individuals who never smoked at baseline (Time 0) (“I have never smoked”) reporting smoking behavior at Time 1 with answers “I have smoked once or twice to try,” “I smoke once in a while, but not every day,” “I smoke every day”
Watkins et al. [58]	Prospective cohort	Ever use of at least once in the past 30 days, and never smoked 1 cigarette at baseline	Never use of e-cigs, and never smoked 1 cigarette at baseline	Individuals who never smoked at baseline reporting ever-use of cigarettes (≥ 1 cigarette) or ≥ 1 cigarette use in the past 30 days at 12–24 month follow-up
Wills et al. [77]	Longitudinal panel	Answered “Yes” when asked if having ‘ever used’ an e-cig and giving the use frequency ‘I have smoked e-cigs 1–2 times’, ‘I have smoked e-cigs 3–4 times’, ‘I usually smoke a few e-cigs a year’, ‘I usually smoke a few e-cigs a month’, ‘I usually smoke a few e-cigs each week’, and ‘I usually smoke e-cigs everyday’ who were also individuals who never smoked cigarettes (“Not having smoked a cigarette in their lifetime”)	Answered “I have never smoked an e-cigarette in my life” to the question “which of the following is most true for you about smoking electronic cigarettes?”	Individuals not smoking cigarettes at Time 1 who reported smoking ≥ 1 cigarette at Time 2 by answering “I have smoked cigarettes 1–2, 3–4 times,” “usually smoke every week,” “usually smoke every day” to question, “which of the following is most true for you about smoking?”
Wills et al. [91]	Longitudinal panel	Answered “Yes” when asked if having ‘ever used’ an e-cig and giving the use frequency ‘I have smoked e-cigs 1–2 times’, ‘I have smoked e-cigs 3–4 times’, ‘I usually smoke a few e-cigs a year’, ‘I usually smoke a few e-cigs a month’, ‘I usually smoke a few e-cigs each week’, and ‘I usually smoke e-cigs everyday’ who were also individuals who never smoked cigarettes (“Not having smoked a cigarette in their lifetime”)	Never use of e-cigs (“I have never smoked e-cigs in my life”)	“No” at Time 1 (baseline) and “Yes” at Time 2 to statements: “I have smoked a cigarette once or twice,” or “I have smoked 3–4 times” at 1-yr follow-up
Xu et al. [82]	Longitudinal panel	Baseline (Wave 1) individuals who are tobacco naïve who reported e-cigs use at Wave 2	Baseline (Wave 1) individuals who are tobacco naïve who did not use e-cigs at Wave 2	Individuals who initiated smoking at Wave 3 after e-cig use at Wave 2
Yang et al. [83]	Longitudinal panel	Individuals not smoking cigarettes who used e-cigs in the past 6 months	Individuals not smoking cigarettes who did not use e-cig in the past 6 months	Subsequent use of cigarette smoking by prior use of e-cig
Young-Wolff et al. [59]	Retrospective cohort	At least 1 “e-cig user” entry in electronic health records who are also individuals who never smoked cigarettes (0 entries as “smoker” in electronic health records)	No “e-cig user” entry in electronic health records	Change in smoking status from never use to smoking cigarettes (≥ 1 cigarette) at 12-month follow-up

*e-cigs* electronic cigarettes, *yrs* years

As discussed in the search results of the meta-analysis, 12 studies met the inclusion criteria of the meta-analysis [34, 43, 52, 56, 59, 63, 66, 76, 77, 80, 81, 84]. These studies are included in Table 3, but are not discussed in qualitative synthesis. For a variety of reasons, 37 studies did not meet the criteria to be included in the quantitative synthesis (Supplemental Section 17: Meta-Analytic Results); however, these studies contained information important to the research question and are described below.

Twenty-four studies—15 prospective cohort studies [37, 46, 49, 55, 58, 61, 62, 68, 70, 71, 73, 83, 87, 89, 90], eight longitudinal panel studies [41, 47, 50, 53, 72, 82, 86, 88], and one retrospective cohort study [67]—all reported statistically significant AORs, showing a higher likelihood of individuals who have used e-cigarettes (non-regular use: ever, ever in the past 12 months, and current) initiating smoking compared with individuals who are not using e-cigarettes. Their AORs ranged from 1.75 (95% CI 1.10–2.77) in a prospective cohort of Grade 9 individuals who never smoked cigarettes at baseline reporting any cigarette use at follow-up (either 6 or 12 months) [87] to 8.3 (95% CI 1.2–58.6) in a prospective cohort of 16–26 year old non-susceptible individuals who never smoked a cigarette reporting ever cigarette use (at least one puff) at 18-month follow-up [89].

Four studies calculated the adjusted relative risk (ARR) of individuals who have used e-cigarettes (ever and current [past-30-day]) smoking cigarettes compared with individuals who are not using e-cigarettes [36, 64, 74, 78]. Lozano et al. [74] found a statistically significantly higher risk for trying smoking (ARR 1.40, 95% CI 1.22–1.60), however, no significant difference was reported for current smoking (≥ 1 cigarette in the past 30 days; ARR 1.43, 95% CI 0.94–2.16). Miech et al. [36] also found a statistically significantly higher risk for current smoking (ARR 4.78, 95% CI 1.91–11.96).

Keller-Hamilton et al. [64] reported that individuals who have used e-cigarettes at baseline were more than twice as likely to report ever (ARR 2.71, 95% CI 1.89–3.87) and current (i.e., past 30 day) smoking (ARR 2.20, 95% CI 1.33–3.64) at follow-up compared to individuals who are not using e-cigarettes. Similar results were reported in a propensity score-matched analysis (ever cigarette use ARR 2.22; 95% CI 0.90–5.47; past 30-day cigarette use ARR 1.25; 95% CI 0.41–3.82). Using data from Waves 1–5 of the PATH study, Harlow et al. [78] showed that, among baseline never-smokers, ever e-cigarette use at Wave 2 was associated with a higher likelihood of ever smoking at Waves 3, 4, and 5 (ARR 2.7, 95% CI 2.4–3.0). This association was present for all sub-categories of e-cigarette ever-use, namely former use (ARR 2.5, 95% CI 2.2–2.9), current (i.e., past 30-day) use (ARR 3.5, 95% CI 2.9–4.1), use of tobacco-flavored (ARR 2.5, 95% CI 1.8–3.5), and nontobacco-flavored (ARR 2.8, 95% CI 2.5–3.1) e-cigarettes. In a marginal structural model that accounted for time-dependent confounding, ever e-cigarette use was similarly associated with a higher likelihood of ever smoking at follow-up waves (ARR 2.4, 95% CI 2.1–2.7), regardless of the sub-category of ever use (former use ARR 2.2, 95% CI 2.0–2.5; current use ARR 3.1, 95% CI 2.6–3.7), or e-cigarette flavor (tobacco flavored ARR 2.4, 95% CI 1.7–3.3; nontobacco flavored ARR 2.4, 95% CI 2.2–2.7) [78]. The study also reported that the likelihood of being an individual who currently smoked (i.e., past 30-day) at Waves 3–5 was higher among individuals who have ever used e-cigarettes at baseline (ARR 2.9, 95% CI 2.5–3.3), quit e-cigarette use (ARR 2.6, 95% CI 2.2–3.1), currently used (ARR 3.8, 95% CI 3.1–4.6), used tobacco-flavored (ARR 2.6, 95% CI 1.7–3.9), and non-tobacco-flavored (ARR 3.0, 95% CI 2.6–3.4) e-cigarettes [78]. Similarly, in the marginal structural model, the likelihood of past 30-day cigarette use at Waves 3–5 was associated with ever (ARR 2.5, 95% CI 2.2–2.9), former (ARR 2.3, 95% CI 1.9–2.7), current (ARR 3.4, 95% CI 2.8–4.2), tobacco-flavored (ARR 2.3, 95% CI 1.5–3.5), and nontobacco-flavored (ARR 2.6, 95% CI 2.2–3.0) [78] e-cigarette use.

A study by Aleyan et al. [60] calculated regression coefficients to estimate the association between past 30-day e-cigarette use at Wave 1 and initiation of cigarette smoking at Wave 3. Past-30-day e-cigarette use at Wave 1 was significantly associated with past 30-day cigarette smoking (β = 1.06; SE = 0.28; 95% CI 0.52–1.60; *p* < 0.001), and dual use at Wave 3 (β = 1.31; SE = 0.24; 95% CI 0.84–1.79; *p* < 0.001). Further, the association between past 30-day e-cigarette use at Wave 1 and cigarette smoking at Wave 3 remained significant after adjustment for having one or more friends who smoked at Wave 1.

Kintz et al. [44] calculated a phi-coefficient for the relationship between ever use of e-cigs at baseline and subsequent cigarette initiation (self-reported first use) at follow-up, and found that baseline ever e-cigarette use was significantly associated with cigarette smoking initiation at follow-up (phi coefficient = 0.141, *p* < 0.001).

Two studies applied a multistate Markov model to evaluate the probability of transitioning to cigarette smoking [42, 85]. A study by Kang et al. [42] applied a multistate Markov model to show that individuals who have ever used e-cigarettes at baseline had a 9.52% (95% CI 6.57–13.85) probability of transitioning to dual e-cigarette and cigarette use, whereas individuals who are not using e-cigarettes at baseline had a 1.39% (95% CI 1.29–1.49) probability of transitioning to exclusive cigarette use. Parnham et al. [85] examined transition probabilities between e-cigarette use and smoking in UK adolescents and young adults. In an analysis that adjusted for age, wave of data collection, sex, ethnicity, and tertiles of household income, adjusted probability of transition from ever e-cigarette use to smoking ranged from 14% (95% CI 13–16) in Year 1 to 27% (95% CI 25–29) in Year 5, while the probability of transitioning from e-cigarette never use to smoking ranged from 2% (95% CI 2–2) to 10% (95% CI 9–10) [85].

The study by Loukas et al. [79] reported hazard ratios for the association between past 30-day and ever e-cigarette use and transition from never to current cigarette smoking. After adjusting for covariates, both past 30-day (HR 2.69, 95% CI 1.95–3.72) and ever (HR 2.16, 95% CI 1.79–2.62) e-cigarette use were associated with a higher likelihood of transition to smoking.

Conner et al. [61] evaluated cigarette smoking initiation (ever use) among individuals who have used e-cigarettes “early” and “late”, defined as reporting ever e-cigarette use at either Wave 3 (early) or Wave 4 (late), respectively. The authors found that the adjusted odds of individuals using e-cigarettes early, compared to individuals who never used e-cigarettes, initiating cigarette smoking was statistically significant both at Wave 4 (AOR 1.39, 95% CI 1.29–1.50) and at Wave 5 (AOR 3.55, 95% CI 2.82–4.49). Similarly, individuals using e-cigarettes late were significantly more likely to initiate cigarette smoking at Wave 5 compared to individuals who have never used e-cigarettes (AOR 2.87, 95% CI 2.33–3.53) [61].

Chaffee et al. [54] calculated the AORs for initiating smoking in three different groups of individuals who have used e-cigarettes (versus individuals who have not used e-cigarettes) and found the following: a non-significant AOR of 1.57 (95% CI 0.99–2.49) for individuals who have ever used e-cigarettes and have smoked at least 100 + cigarettes; a non-significant AOR of 1.69 (95% CI 0.93–3.05) for individuals who have used e-cigarettes in the past-30-days who have smoked at least 100 cigarettes; a non-significant AOR for individuals who have smoked at least 100 cigarettes but have quit e-cigarette use (AOR 1.55, 95% CI 0.94–2.56); a non-significant AOR for individuals who have ever used e-cigarettes and smoked a cigarette in the past 30 days (AOR 1.32, 95% CI 0.99–1.76); a significant AOR for individuals who have used e-cigarettes in the past 30 days and smoked a cigarette in the past 30 days (AOR 1.64, 95% CI 1.12–2.41); and, a non-significant AOR for individuals who quit e-cigarette use and smoked in the past 30 days (AOR 1.20, 95% CI 0.86–1.68) [54].

In additional to their overall analysis, Owotomo et al. [46] reported AORs for cigarette smoking initiation among subgroups of adolescents according to their baseline cigarette smoking intentions. Overall, the authors found ever e-cigarette use to be significantly associated with ever cigarette smoking (AOR 2.58, 95% CI 1.73–3.85). The association remained significant in a subgroup analysis of adolescents with no baseline intention to smoke (AOR 4.62, 95% CI 2.87–7.42); however, among the subgroup of adolescents with baseline cigarette smoking intentions, the association between ever e-cigarette use and cigarette smoking initiation was nonsignificant (AOR 1.57, 95% CI 0.94–2.63). The AOR for the interaction between smoking intention and ever e-cigarette use with regards to smoking initiation was statistically significant (AOR 0.34, 95% CI 0.18–0.64), suggesting the association between e cigarette use and ever cigarette smoking was dependent on previous smoking intention status.

Three of the 37 studies not included in the meta-analysis evaluated initiation of cigarette smoking and either susceptibility or propensity to smoke cigarettes among individuals using e-cigarettes versus individuals who are not using e-cigarettes [52, 57, 91]. Barrington-Trimis et al. [52] evaluated the association between susceptibility and initiation of cigarette smoking in either individuals who have ever used e-cigarettes or individuals who are not using e-cigarettes and found a statistically significant difference between the two groups. The authors found that among individuals who are not using e-cigarettes, susceptibility to cigarette use was associated with over three times the odds of subsequent initiation of cigarette smoking compared with non-susceptible individuals who are not using e-cigarettes (AOR 3.47, 95% CI 2.38–5.07); however, only a small, non-statistically significant association was observed between susceptible and non-susceptible individuals who have ever used e-cigarettes and initiation of cigarette smoking (AOR 1.57, 95% CI 0.80–3.05) [52]. Thus, susceptibility only statistically significantly affected the subsequent initiation of cigarette smoking in individuals who are not using e-cigarettes (*p*_interaction_ = 0.04).

Findings from a 2016 study by Wills et al. indicated that the effect of e-cigarette for cigarette smoking onset decreased as propensity increased—the AOR for smoking onset for individuals currently using e-cigarettes (past-30-day) versus individuals who are not using e-cigarettes was 2.23 (95% CI 1.57–3.17) for those in the bottom 10th percentile for propensity to smoke, and 1.32 (95% CI 1.19–1.47) for those in the top 10th percentile for propensity to smoke [91].

In a 2018 study, Treur et al. provided AORs for low-propensity- and high-propensity-to-smoke groups for ever e-cigarette versus individuals who are not using e-cigarettes, both with and without nicotine [57]. The investigators found that, for e-cigarettes containing nicotine, the AOR for initiating conventional smoking was 7.80 (95% CI 1.90–32.04) in the low-propensity-to-smoke group, and 2.89 (95% CI 1.47–5.68) in the high-propensity-to-smoke group; for e-cigarettes containing no nicotine, the AOR for initiating conventional smoking was 6.07 (95% CI 2.18–16.90) in the low-propensity-to-smoke group, and 3.30 (95% CI 2.33–4.67) in the high-propensity-to-smoke group.

Treur et al. also compared the effects of e-cigarette use with nicotine and e-cigarette use without nicotine in individuals using e-cigarettes versus individuals who have never used e-cigarettes [57]. The study reported an AOR for initiation of 5.36 (95% CI 2.73–10.52) for individuals who ever used e-cigarettes without nicotine compared with individuals who are not using e-cigarettes, and an AOR of 11.90 (95% CI 3.36–42.11) for individuals who ever used e-cigarettes with nicotine compared with individuals not using e-cigarettes.

Three studies evaluated initiation in susceptible subgroups [34, 68, 90], two of which were included in the meta-analysis for initiation of cigarette smoking [34, 90]. The association between ever e-cigarette use and susceptibility to smoking was evaluated in a 2016 prospective cohort study by Barrington-Trimis et al. [90]. The study found that ever e-cigarette use had less of an effect in individuals classified as being susceptible to smoking, as demonstrated by a lower odds of initiation of cigarette smoking in that group (AOR 2.12, 95% CI 0.79–5.74), compared with individuals using e-cigarettes initially classified as non-susceptible to smoking (AOR 9.69, 95% CI 4.02–23.4) (*p*_interaction_ = 0.025) [90]. Interestingly, the effect of e-cigarette use in the susceptible group on initiation of cigarette smoking was not statistically significant.

Berry et al. [68] reported similar outcomes, both in terms of ever and current cigarette use. In terms of ever cigarette use, the authors demonstrated lower odds of initiation among individuals who had used e-cigarettes in the past versus individuals who are not using e-cigarettes (AOR 3.51, 95% CI 2.52–4.89) among individuals classified as intermediate/high risk for smoking, compared with those classified as low risk (AOR 8.57, 95% CI 3.87–18.97). Similarly, in terms of current cigarette use, odds of initiation were lower among individuals classified as intermediate/high risk (AOR 2.16, 95% CI 1.23–3.79) compared with those classified as low risk (AOR 10.36, 95% CI 3.11–34.54). In both cases, this indicates that e-cigarette use had less of an effect on initiation among those individuals considered intermediate/high risk.

Best et al. [34], also included in the meta-analysis, found that there was an interaction between susceptibility to smoking and ever e-cigarette use with regards to initiation of cigarette smoking (AOR for e-cigarette use and susceptibility interaction of 0.42, 95% CI 0.19–0.94). In other words, there would be greater interaction between e-cigarette use and non-susceptible populations compared with susceptible populations in terms of initiation of cigarette smoking. It is worth noting that although Best et al. refer in their study to susceptibility and not the intent, the questions that respondents answered, i.e., “Do you think you will smoke cigarettes or hand-rolled cigarettes at any time during the next year” and “If one of your friends offered you a cigarette or hand-rolled cigarettes (roll-ups), would you smoke it?” were questions that measured intent.

Lastly, one study by Barrington-Trimis et al. [51, 52] investigating initiation of cigarette smoking, with analyses of switching and dual-use, found that the adjusted odds of reporting dual use (at follow-up) among individuals who had ever used e-cigarettes exclusively at baseline (versus individuals who had never used e-cigarettes at baseline) were higher than the odds of reporting switching from baseline exclusive e-cigarette use to exclusive cigarette smoking at follow-up (AOR 7.16, 95% CI 4.47–11.5 vs. AOR 2.67, 95% CI 1.53–4.65, respectively). In another analysis, the authors also found that the odds of reporting dual use among current (past 30-day) e-cigarette users (versus non-current users) were similarly higher than the odds of reporting switching from exclusive e-cigarette use to exclusive cigarette smoking (AOR 8.86, 95% CI 5.08–15.4 vs. AOR 3.84, 95% CI 1.80–8.19, respectively [52].

#### Initiation of and progression to regular cigarette smoking (regular e-cigarette use: 2 studies)

Two adjusted studies were identified that provided adjusted analyses of *initiation of and progression to regular cigarette smoking* in individuals with regular use of e-cigarettes [40, 48]. Summary characteristics of these two studies are provided in Table 4.

**Table 4:** Summary characteristics of adjusted studies for initiation and progression to regular cigarette smoking among individuals using e-cigarettes regularly (n = 2)

Citation	Study design	Definition of individuals who use e-cigarettes	Definition of individuals who do not use e-cigs	Definition of initiation of cigarette smoking
Azagba et al. [40]	Prospective cohort	Individuals who use e-cigs every day: Individuals who reported having ever used e-cigs fairly regularly and currently using e-cigs every day Individuals who use e-cigs somedays: Individuals who reported having ever used e-cigs fairly regularly and currently using e-cigs some days	Individuals who do not use e-cigs: Those who answered no to “Have you used an e-cigarette, such as NJOY, Blu, or Smoking Everywhere, even one or two times?”	Individuals smoking cigarettes experimentally (< 100-lifetime cigarettes and currently smoking some days or every day) at baseline who reported someday (≥ 100-lifetime cigarettes and currently smoking cigarettes somedays) or every day (≥ 100 cigarettes in their lifetime and currently smoked cigarettes every day) smoking at follow up.
				Individuals who smoked some-days (≥ 100-lifetime cigarettes and currently smoking cigarettes somedays) at baseline who reported everyday smoking (≥ 100 cigarettes in their lifetime and currently smoked cigarettes every day) at follow up
Wei et al. [48]	Prospective cohort	Individuals with established e-cig use: Individuals not current (not using “every day” or “somedays”) and not established (< 100-lifetime cigarettes) with cigarette smoking reporting current e-cig use, and having used e-cigs “fairly regularly”	Individuals using e-cigs experimentally: Individuals not current (not using “every day” or “somedays”) and not established (< 100-lifetime cigarettes) with cigarette smoking reporting current e-cig use, but not having used e-cigs “fairly regularly”	Individuals smoking cigarettes exclusively: Wave 1 Individuals not current (not using “every day” or “somedays”) and not established (< 100-lifetime cigarettes) with cigarette smoking reporting current (“every day” or “somedays”), established (≥ 100-lifetime cigarettes) cigarette use at Wave 2
				Dual-use: Wave 1 Individuals not current (not using “every day” or “somedays”) and not established (< 100-lifetime cigarettes) with cigarette smoking reporting current (“every day” or “somedays”), established (≥ 100-lifetime cigarettes) cigarette use and current (“every day” or “somedays”), established having used fairly regularly) e-cig use

*e-cigs* electronic cigarettes, *yrs* years

Azagba et al. [40] defined regular e-cigarette use as either every day or someday use. In terms of the transition from experimental to some-day cigarette smoking, no significant association was found between individuals using e-cigarettes every day and individuals who have never used e-cigarettes (AOR 1.31, 95% CI 0.20–8.58), nor between individuals using e-cigarettes some day and individuals who have never used e-cigarettes (AOR 0.48, 95% CI 0.13–1.78). Similarly, no significant associations were found between individuals using e-cigarettes every day and individuals who have never used e-cigarettes (AOR 0.58, 95% CI 0.09–3.93) and individuals using e-cigarettes some day and individuals who have never used e-cigarettes (AOR 1.14, 95% CI 0.42–3.05) in terms of the transition from experimental to daily cigarette smoking. Likewise, in terms of the transition from some-day to daily cigarette smoking, no significant association was found between individuals using e-cigarettes every day and individuals who have never used e-cigarettes (AOR 1.89, 95% CI 0.98–3.66), nor between individuals using e-cigarettes some day and individuals who have never used e-cigarettes (AOR 1.41, 95% CI 0.84–2.39).

Wei et al. [48] evaluated transitions from non-current, non-established cigarette smoking to either exclusive current-established cigarette smoking or current-established dual use of cigarettes and e-cigarettes, among baseline individuals using e-cigarettes exclusively. The authors found that individuals who have established e-cigarette use were significantly less likely to transition to exclusive current-established cigarette smoking than individuals who have non-established e-cigarette use (AOR 0.13, 95% CI 0.02–0.87); however, no significant association was found between e-cigarette use (established versus non-established) and transitioning to dual use of cigarettes and e-cigarettes (AOR 0.53, 95% CI 0.05–6.25).

#### Initiation of and progression to regular cigarette smoking (non-regular e-cigarette use: 11 studies)

Eleven adjusted studies examined the potential association between e-cigarette use and *initiation and progression to regular cigarette smoking* among individuals with non-regular use of e-cigarettes [37, 40, 45, 54, 61, 65, 66, 69, 72, 73, 86]. Study characteristics for the 11 included studies are presented in Table 5.

**Table 5:** Summary characteristics of adjusted studies for initiation and progression to regular cigarette smoking among individuals with non-regular use of e-cigarettes (n = 11)

Citation	Study design	Definition of individuals who use e-cigarettes	Definition of individuals who do not use e-cigs	Definition of initiation of cigarette smoking
Azagba et al. [40]	Prospective cohort	Individuals using e-cigs experimentally: Individuals who reported having never used e-cigs fairly regularly but were currently using e-cigs every day or some days	Individuals who do not use e-cigs: Those who answered no to “Have you used an e-cigarette, such as NJOY, Blu, or Smoking Everywhere, even one or two times?”	Individuals smoking cigarettes experimentally (< 100-lifetime cigarettes and currently smoking some days or every day) at baseline who reported someday (≥ 100-lifetime cigarettes and currently smoking cigarettes somedays) or every day (≥ 100 cigarettes in their lifetime and currently smoked cigarettes every day) smoking at follow up.
				Individuals smoking cigarettes some days (≥ 100-lifetime cigarettes and currently smoking cigarettes somedays) at baseline who reported everyday smoking (≥ 100 cigarettes in their lifetime and currently smoked cigarettes every day) at follow up
Chaffee et al. [54]	Prospective cohort	Individuals who have ever used: individuals who never smoked cigarettes and lifetime use of e-cigs, Past-30-day users: individuals who never smoked cigarettes and use of e-cigs in the past 30 days	Never use of e-cigs	Smoking during the past 30 days and having smoked ≥ 100 cigarettes at 1 yr follow up (current established smoking)
Conner et al. [37]	Prospective cohort	*Individuals using e-cigs*: Individuals who never smoked cigarettes at baseline reporting ever use of e-cigs (“I have tried them once or twice,” “I use them sometimes [more than once a month but less than once a week],” or “I use them often [more than once a week]”)	*Individuals who have not used e-cigs*: Individuals who never smoked cigarettes reporting never having used e-cigs at baseline	Individuals who smoked cigarettes regularly: self-reported regular use of cigarettes at follow-up (1 or more cigarettes a week)
Conner et al. [61]	Prospective cohort	*Individuals with early e-cig use:* Baseline (Wave 3) individuals who never smoked cigarettes who reported ever use of e-cigs *Individuals with late e-cig use:* Baseline (Wave 3) individuals who never smoked cigarettes and individuals who never used e-cigarettes, who reported never smoking and ever e-cig use at Wave 4	Individuals who never smoked cigarettes reporting never having used e-cigs at baseline	Individuals who never smoked cigarettes at baseline (Wave 3) who reported ever smoking cigarettes regularly (≥ 1 cigarette per week) at follow-up (Wave 4 or Wave 5)
Friedman et al. [69]	Cross-sectional analytic	Individuals using e-cigs: Individuals reporting either exclusive e-cig use or e-cig experimentation prior to cigarette use	Individuals who have not used e-cigs: Individuals reporting no e-cig use	Self-reported established (≥ 100 cigarettes smoked and past-30-day use) or daily smoking status
Hammond et al. [73]	Prospective cohort	Individuals using e-cigss at baseline (use of e-cig products in the last 30 days) and individuals who never smoked a cigarette (“Never having tried cigarette smoking”)	Never used e-cigs at baseline and never-smoked	Smoked every day for 7 days at 1-yr follow-up
McMillen et al. [72]	Longitudinal panel	Individuals everusing e-cigs: Individuals who do not smoke cigarettes responding “yes” to question “Have you ever used an e-cigarette, such as NJOY, Blu, or Smoking Everywhere, even 1 or 2 times?” and responding “not at all” to question “Do you now use e-cigarettes every day, some days, or not at all?” individuals using e-cigs in the past 30 days: Individuals who do not smoke cigarettes responding “yes” to question “Have you ever used an e-cigarette, such as NJOY, Blu, or Smoking Everywhere, even 1 or 2 times?” and responding “every day” or “some days” to question “Do you now use e-cigarettes every day, some days, or not at all?”	Individuals who have not used e-cigs: Individuals who do not smoke cigarettes responding “no” to question “Have you ever used an e-cigarette, such as NJOY, Blu, or Smoking Everywhere, even 1 or 2 times?”	Individuals who never smoked cigarettes at baseline reporting current (past 30 day) smoking, and having smoked ≥ 100 cigarettes at follow-up (became individuals with established smoking cigarettes)
Osibogun et al. [45]	Longitudinal panel	Individuals using e-cigs: Individuals who do not smoke cigarettes reporting current e-cig use in the past 30 days	Individuals who have not used e-cigs: Individuals who do not smoke cigarettes reporting no current e-cig use in the past 30 days	1-yr progression outcome: Individuals with no past 30 day cigarette smoking at baseline waves (Wave 1 or Wave 2) reporting cigarette smoking in at least 20 of the past 30 days at follow-up waves (Wave 2 or Wave 3);
				2-yr progression outcome: Individuals with no past 30 day cigarette smoking at Wave 1 reporting cigarette smoking in at least 20 of past 30 days at Wave 3
Pierce et al. [65]	Longitudinal panel	Individuals who responded “yes” to whether they had ever used the e-cig even 1 or 2 times?”	Never used e-cigs at baseline	Daily use of cigarettes (at least 25 days in the past 30 days)
Staff et al. [66]	Prospective cohort	Youth who, at age 14, never smoked cigarettes and reported ever trying an e-cigarette or vaping device	Youth who, at age 14, never smoked cigarettes and reported never trying an e-cigarette or vaping device	Individuals who never smoked cigarettes at age 14 who report frequent smoking (> 6 cigarettes per week) by age 17
Sun et al. [86]	Longitudinal panel	Individuals who have ever used e-cigs: Cigarette-naïve individuals at baseline (Wave 3) who self-reported ever use of e-cigs Individuals currently using e-cigs: Cigarette-naïve individuals at baseline (Wave 3) who self-reported past 30 days e-cigs use	Cigarette-naïve individuals at baseline (Wave 3) who never used e-cigs	Individuals who never smoked cigarettes at baseline (Wave 3) who reported cigarette smoking in the past 12 months at Wave 4 and continued established* use at Wave 5 among Wave 4 individuals who reported initiating cigarette smoking.
				* Established use was defined as ≥ 100 lifetime cigarettes and currently smoking
				CSM was defined as: CSM-V—Past 12-month use at Wave 4, established use and ≥ 20 days use in the past 30 days at Wave 5

*CSM* continued smoking measure, *e-cig* electronic cigarette, *smoking* cigarette smoking, *yr(s)* year(s)

Sun et al. [86] used data from Waves 3–5 of the PATH study to investigate the association between e-cigarette use and the progression into regular cigarette smoking—defined as past 12-month use at Wave 4 with established use and at least 20 days use in the past 30 days at Wave 5. The authors show that the association between ever e-cigarette user and progression into regular smoking is non-significant with baseline e-cigarette ever-users having a lower risk of progressing into established regular smoking 0.13% (95% CI − 0.31 to 0.58) versus 0.17% (95% CI − 0.30 to 0.64) for baseline e-cigarette never-users (ARD − 0.03, 95% CI − 0.33 to 0.27; AOR 0.80, 95% CI 0.10–6.49). Similarly, e-cigarette current use was not associated with progression into established regular smoking as evidenced by the absolute risk of 0.47% (95% CI − 1.46 to 2.39) for individuals currently using e-cigs versus 0.15% (95% CI − 0.27 to 0.58) for e-cig non-users (ARD 0.31, 95% CI − 1.36 to 1.99; AOR 3.14, 95% CI 0.13–74.96) [86].

In addition to applying measures of regular e-cigarette use described previously, Azagba et al. [40] also applied a non-regular definition of experimental e-cigarette use. Consistent with their findings from their analyses of regular e-cigarette use, no significant associations were found between experimental and e-cigarette never-users in terms of: transitioning from experimental to someday cigarette smoking (AOR 0.98, 95% CI 0.44–2.20); transitioning from experimental to daily cigarette smoking (AOR 0.59, 95% CI 0.26–1.31); and transitioning from some day to daily cigarette smoking (AOR 1.03, 95% CI 0.61–1.75) [40].

A longitudinal panel study by McMillen et al. [72] reported inconsistent findings, depending on the measure of e-cigarette use applied. When evaluating ever e-cigarette use (versus e-cigarette non-use), no significant association with progression to current established cigarette smoking was found (AOR 2.5, 95% CI 0.6–10.9); however, current e-cigarette users were found to be significantly more likely to progress to current established cigarette smoking compared to individuals who are not using e-cigarettes (AOR 8.0, 95% CI 2.8–22.7). Another longitudinal panel study by Pierce et al. [65] evaluated rate of progression to daily cigarette smoking at Wave 4 among ever (but not daily) tobacco product users at Wave 3 of the PATH survey. The authors found that the adjusted risk difference between individuals who have ever used e-cigarettes versus e-cigarette never-users for progression to daily cigarette smoking was 7% (95% CI 6–9%) higher for individuals using e-cigarettes, although statistical significance was not assessed [65].

Findings from a prospective cohort study by Chaffee et al. [54] suggested the AOR of progressing to regular smoking (i.e., smoked ≥ 100 cigarettes and smoked in the past 30 days) was statistically significantly higher in individuals who have ever used e-cigarettes compared with individuals who are not using e-cigarettes (AOR 1.80, 95% CI 1.04–3.12); however, no such association was shown for past-30-day e-cigarette users (AOR 1.76, 95% CI 0.92–3.37). A second prospective cohort study by Hammond et al. [73] reported that progression to regular cigarette smoking was statistically significantly higher in past-30-day e-cigarette users compared with individuals who are not using e-cigarettes (AOR 1.79, 95% CI 1.41–2.28), while findings from a third prospective cohort study by Conner et al. [37] suggested statistically significantly higher odds of progressing to regular smoking (≥ 1 cigarette per week) at 2 years among individuals who have ever used e-cigarettes compared with individuals who are not using e-cigarettes (AOR 1.27, 95% CI 1.17–1.39). A fourth prospective cohort study, also by Conner et al. [61], reported statistically significantly higher odds of regular smoking (defined as smoking at least 1 cigarette per week) at Wave 5 among adolescents who first reported e-cigarette use at 13–14 years old (i.e., early users; AOR 1.25, 95% CI 1.16–1.34), and those who first reported e-cigarette use at 14–15 years (i.e., late users; AOR 1.12, 95% CI 1.08–1.16). The final prospective cohort study by Staff et al. [66] reported that the adjusted odds of reporting frequent smoking by age 17 were significantly higher for individuals using e-cigarettes compared with individuals who are not using e-cigarettes at baseline (AOR 2.91, 95% CI 1.56–5.4). The odds of frequent smoking remained significantly higher for individuals using e-cigarettes when the samples were matched on risk factors using propensity score matching.

Osibogun et al. [45] evaluated progression to regular cigarette smoking at both 1 and 2 years from baseline, finding that progression at 1 year was significantly associated with e-cigarette use (AOR 5.0, 95% CI 1.9–12.8). However, progression at 2 years was not significantly associated with e-cigarette use (AOR 3.4, 95% CI 1.0–11.5) [45].

The one cross-sectional study by Friedman et al. [69] reported statistically significantly lower odds of current established (≥ 100-lifetime cigarettes and past-30-day use) (AOR 0.22 95% CI 0.10–0.50) or daily (AOR 0.22 95% CI 0.06–0.77) cigarette use among individuals who experimented exclusively with e-cigarettes (experimenting before the age of 18 years) compared with individuals who did not experiment with e-cigarettes. Findings from this study also suggested statistically significantly higher odds of reporting current established cigarette smoking among individuals who first experimented with e-cigarettes and then with cigarettes, compared with individuals who did not experiment with e-cigarettes (AOR 1.89 95% CI 1.09–3.27); however, no significant difference in the odds of daily smoking was shown (AOR 0.73 95% CI NR).

### Quantitative synthesis of best available evidence

Meta-analyses were performed by calculating pooled ORs from studies presenting AORs on *initiation of cigarette smoking* among naïve (individuals who never smoked cigarettes) cigarette smokers who either ever used or never used e-cigarettes. A meta-analysis evaluating the association between regular e-cigarette use and initiation of cigarette smoking was not possible, given that only one study reported adjusted outcomes for this association. Additionally, a meta-analysis evaluating e-cigarette use and initiation and progression to regular smoking was not possible, due to differences in definitions of e-cigarette use and/or outcome measures between studies (full results in Supplemental Section 17: Meta-Analytic Results; all relevant code is publicly available [DOI:10.5281/zenodo.10927677]).

Twelve studies met all the inclusion criteria and were included in the meta-analysis for initiation of cigarette smoking [34, 43, 52, 56, 59, 63, 66, 76, 77, 80, 81, 84]. All 12 studies included individuals who never smoked cigarettes who were evaluated for initiation of cigarette smoking (minimum inclusion criteria = 1 puff). The studies compared an e-cigarette use group (regardless of frequency, volume, and duration) to a control group of e-cigarette never-users. The results from each study controlled for age, gender, race/ethnicity, and other covariates. All studies were longitudinal in design and had a combined analytic sample of 57,730 respondents.

**Fig. 2:**
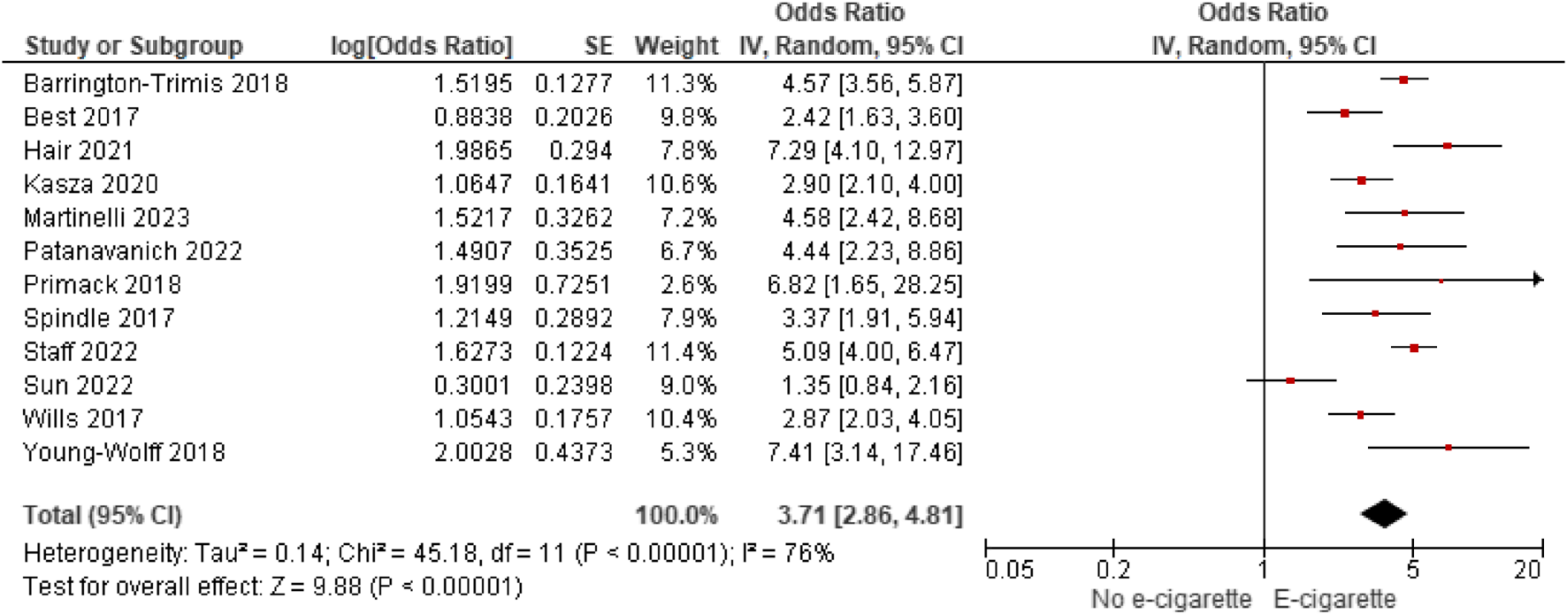
Meta-analysis of odds of initiation of cigarette smoking among individuals who never smoked cigarettes who used e-cigarettes

**Fig. 3:**
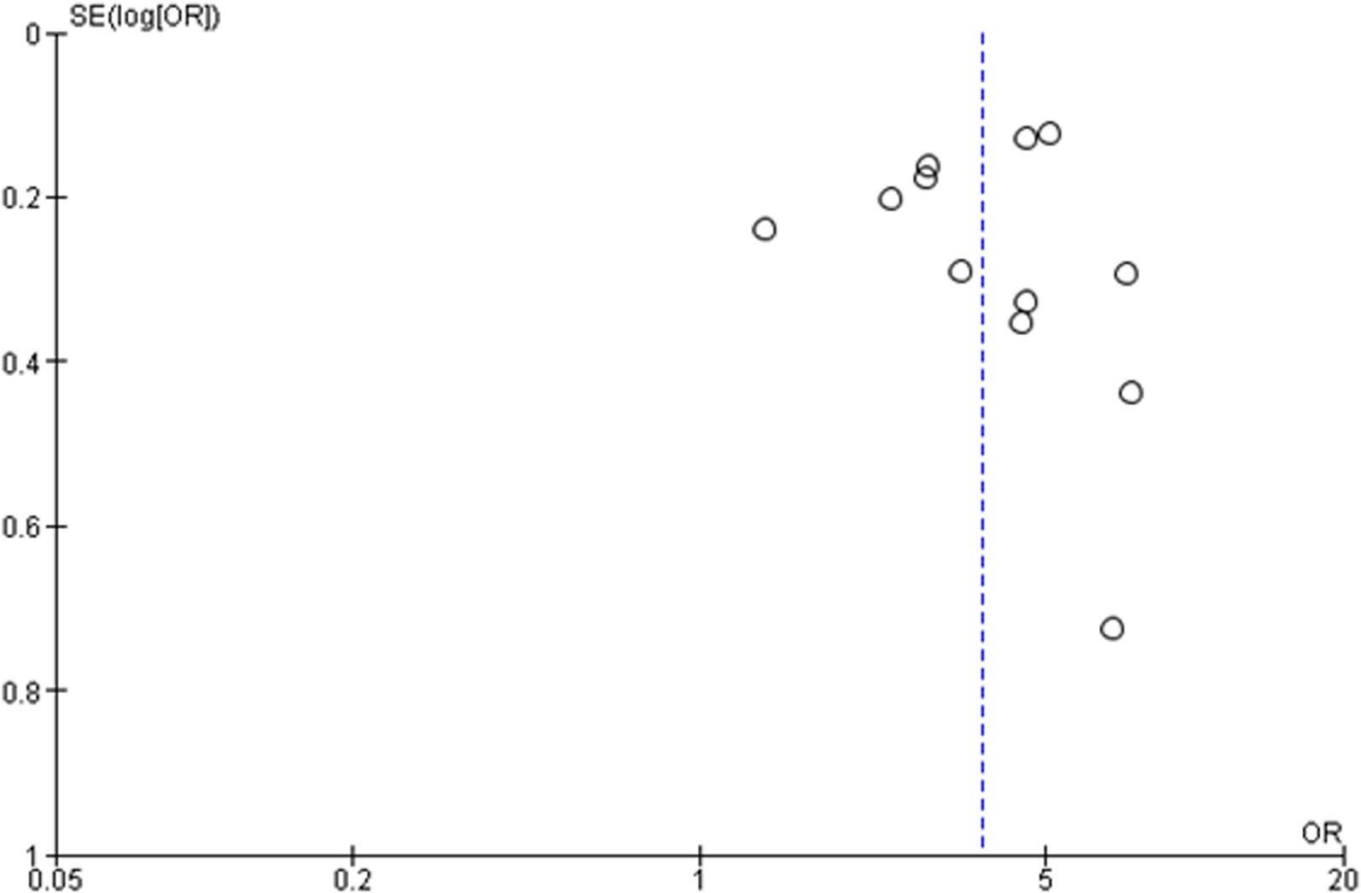
Funnel plot for publication bias

For the 12 studies, the AORs ranged from 1.35 to 7.41. Pooling their results, the overall OR was 3.71 (95% CI 2.86–4.81). The test for the overall effect of the model was noted to be statistically significant (*p* < 0.00001). Heterogeneity tests revealed an I^2^ of 76% and a *X*^*2*^ of 45.18 (*p* < 0.00001) (Fig. 2). An assessment of publication bias—via the development of a funnel plot—was generally symmetrical, suggesting an absence of publication bias (Fig. 3).

**Fig. 4:**
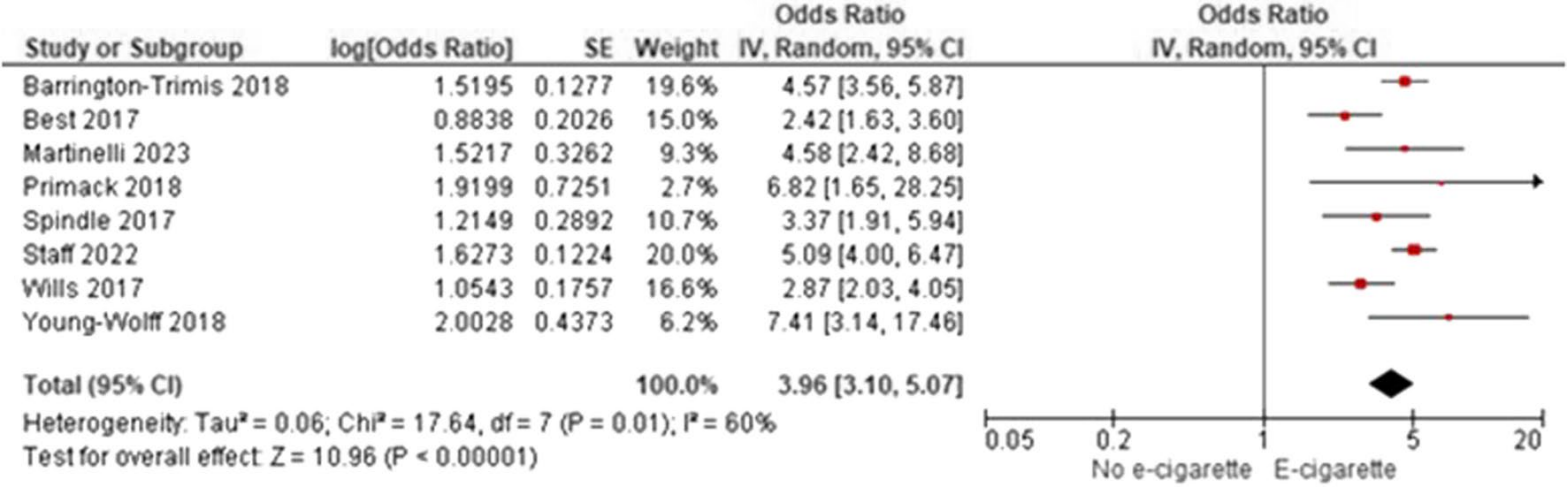
Sensitivity analysis of odds of initiation of cigarette smoking among individuals who never smoked cigarettes who used e-cigarettes—excluding studies rated as “fair” quality

Additionally, a sensitivity analysis excluding studies with a “Fair” quality rating was conducted—resulting in the exclusion of four studies [43, 63, 80, 81]. Results of the eight studies with “Good” rating, presented a pooled OR of 3.96 (95% CI 3.10–5.07), with an I^2^ of 60% and a *X*^*2*^ of 17.64 (*p* < 0.00001) (Fig. 4).

**Fig. 5:**
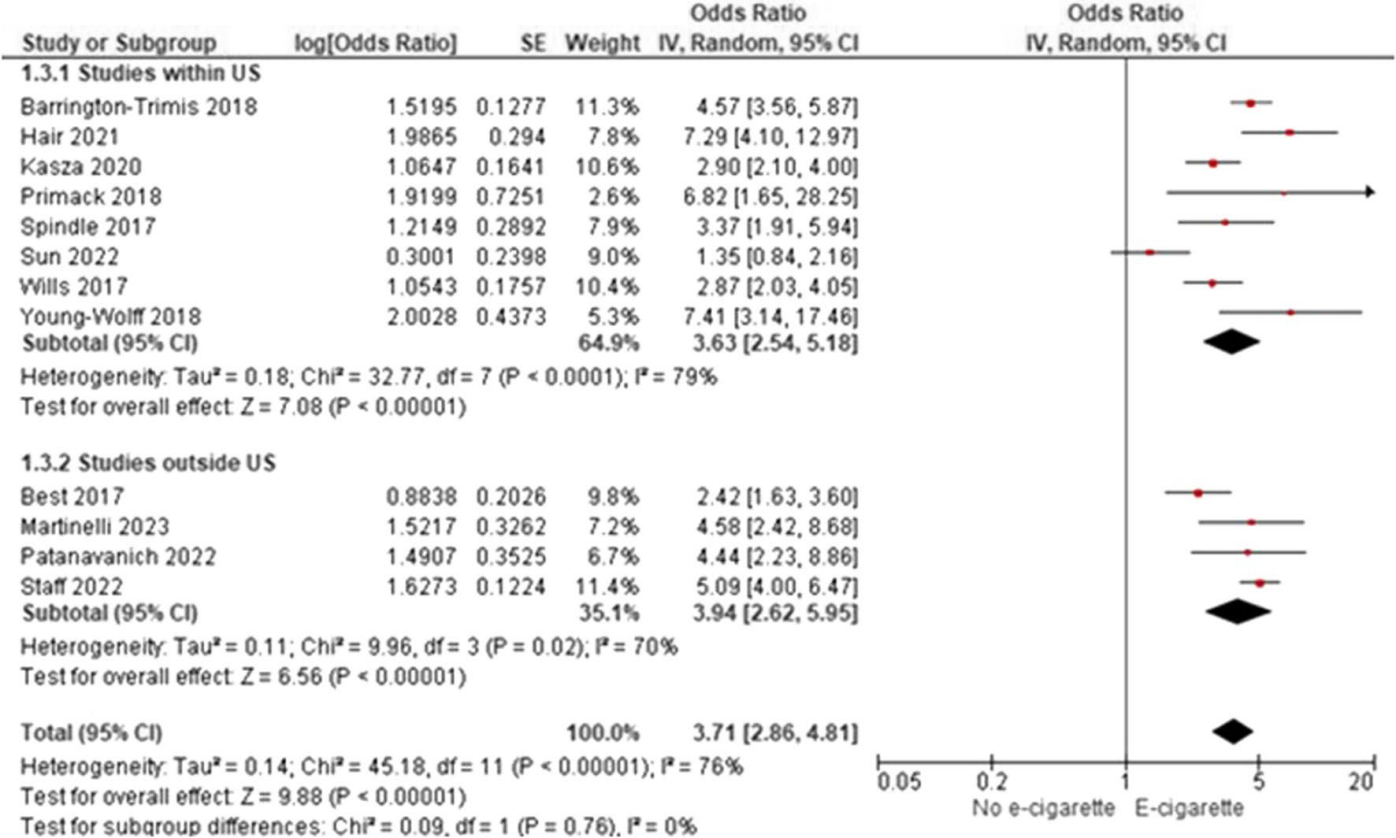
Sub-group meta-analysis of odds of initiation of cigarette smoking among individuals who never smoked cigarettes who used e-cigarettes from studies conducted in the US and outside the US

A sub-group analysis was conducted based on the country where the study was implemented (US-based or outside the US). The sub-group analysis stratified the results of eight studies conducted in the US [43, 52, 56, 59, 63, 76, 77, 81] and four studies conducted outside of the US [34, 66, 80, 84]. In the eight US studies, AORs ranged from 1.35 to 7.41, and the pooled overall OR was 3.63 (95% CI 2.54–5.18). The test for overall effect revealed that the results were significant (*p* < 0.00001), while heterogeneity was noted with I^2^ of 79% and *X*^*2*^ of 32.77 (*p* < 0.0001). In the studies outside the US the AORs ranged from 2.42 to 5.09 and the pooled OR was 3.94 (95% CI 2.62–5.95), with a significant test for overall effect (*p* < 0.00001), and I^2^ of 70% and *X*^*2*^ of 9.96 (*p* < 0.00001). The test for subgroup difference presented an I^2^ of 0% and *X*^*2*^ of 0.09 (*p* = 0.76) (Fig. 5).

**Fig. 6:**
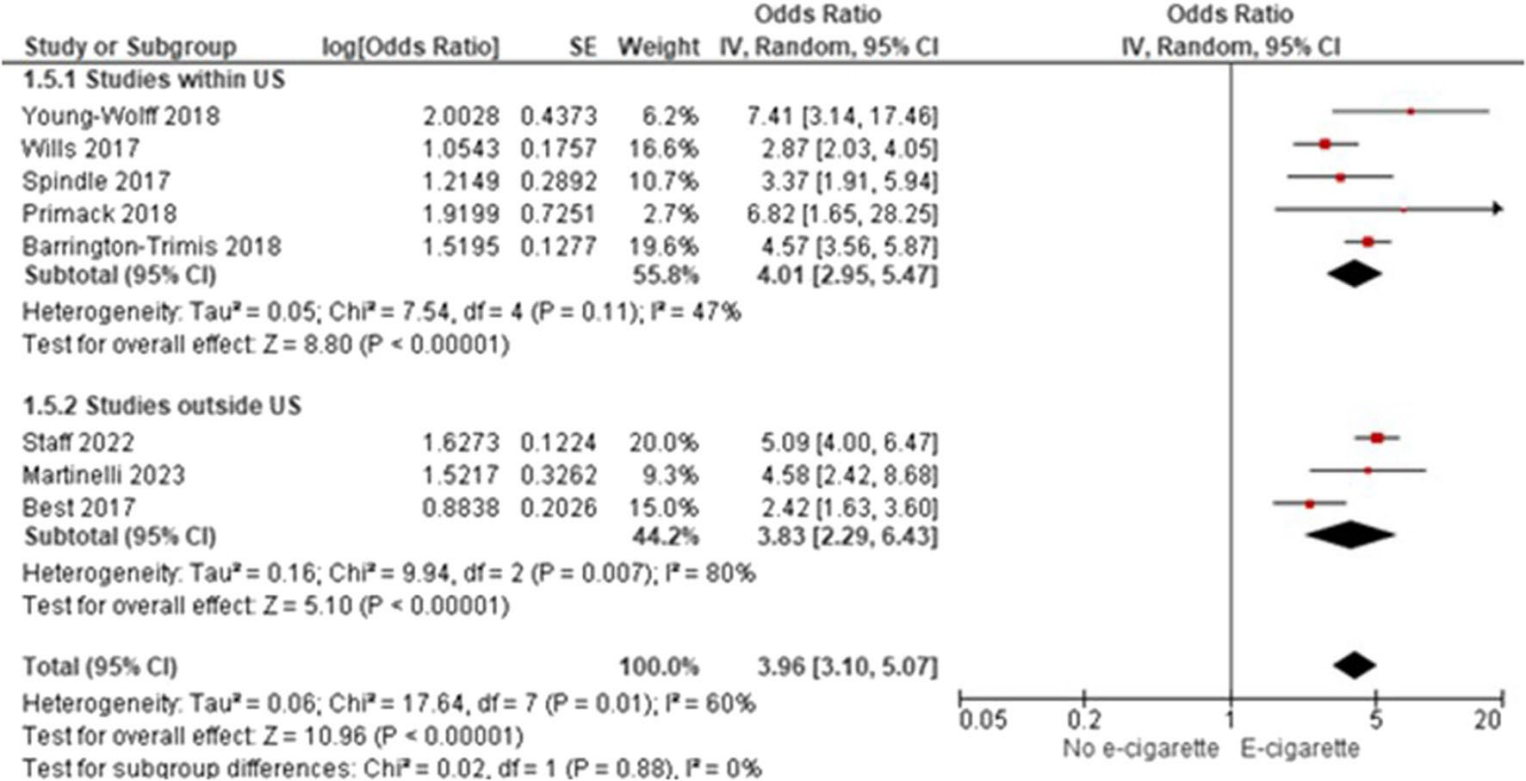
Sub-group meta-analysis of odds of initiation of cigarette smoking among individuals who never smoked cigarettes who used e-cigarettes from studies conducted in the US and outside the US—excluding studies rated as “fair” quality

As with the main analysis, a sensitivity analysis of the subgroup analysis based on country was performed, excluding studies graded as “Fair” quality, which resulting in the exclusion of three US-based studies [43, 63, 81], and one study from outside the US [80]. Pooled results from the remaining five US studies revealed a statistically significant pooled OR of 4.01 (95% CI 2.95–5.47; *p* < 0.00001) with an I^2^ of 47% and a *X*^*2*^ of 7.54 (*p* = 0.11). In the remaining studies outside the US the AORs ranged from 2.42 to 5.09 and the pooled OR was 3.83 (95% CI 2.29–5.07), with a significant test for overall effect (*p* < 0.00001), and I^2^ of 80% and *X*^*2*^ of 9.94 (*p* < 0.00001). The test for subgroup difference presented an I^2^ of 0% and *X*^*2*^ of 0.02 (*p* = 0.88) (Fig. 6).

## Discussion

The current systematic review identified a number of “good” quality studies (according to the Downs and Black quality metrics [29]) that evaluated the association between e-cigarette use and initiation of cigarette smoking, and initiation of and progression to regular cigarette smoking. Over half of the included studies controlled for age, gender, and race/ethnicity and reported adjusted results to provide a higher level of evidence. This review focused on such studies in the quantitative and qualitative synthesis of results.

A meta-analysis of 12 studies evaluating initiation of cigarette smoking indicated an increased odds (3.7 times higher) for individuals who have ever used e-cigarettes compared with individuals who are not using e-cigarettes and no indication of publication bias among the studies was observed [34, 43, 51, 56, 59, 63, 66, 76, 77, 80, 81, 84]. These findings are consistent with previously-conducted meta-analyses, all of which reported increased odds of initiation associated with e-cigarettes: O’Brien et al. [16] reported 4.06 times higher odds among teenagers; Soneji et al. [13] reported 3.5 times higher odds among a study population of adolescents and young adults; Chan et al. [14] and Khouja et al. [15] both reported 2.9 times higher odds, in populations of youth and youth-young adults, respectively; and Baenziger et al. [18] and Adermark et al. [19] reported 3.2 and 3.3 times higher odds, respectively, in samples from the general population.

Only one study, also included in the meta-analysis, reported on initiation of cigarette smoking in individuals with regular use of e-cigarettes, providing outcome data for initiation of cigarette smoking based on the frequency of e-cigarette use at baseline (from 1–2 uses/day to everyday use) [77]. Wills et al. [77] found an upward trend for the probability of initiation of cigarette smoking and increased e-cigarette use. Thirty-seven adjusted studies not included in the meta-analysis showed a similar trend, with a higher probability or incidence of initiation of cigarette smoking in the e-cigarette user group [36, 37, 41, 42, 44, 46, 47, 49, 50, 52–55, 57, 58, 60–62, 64, 67, 68, 70–74, 78, 79, 82, 83, 85–91]. These studies had similar definitions for e-cigarette use, with any or ever use at baseline, any e-cigarette use in the past 12 months, or any use in the past 30 days. All but one of these studies defined cigarette use as any cigarette use at follow-up, while the remaining study evaluated regular smoking, although definition of regular smoking was not provided.

Six studies compared initiation of cigarette smoking with e-cigarette use between study groups that were susceptible or not susceptible to cigarette smoking [34, 52, 57, 68, 90, 91]. E-cigarette use was either not associated with an increase in smoking initiation in individuals using e-cigarettes susceptible to cigarette smoking [52, 90], or the effect of e-cigarette use on initiation of cigarette smoking was less in individuals using e-cigarettes susceptible to cigarette smoking [34, 57, 68, 91].

The limited data from one study evaluating e-cigarettes with or without nicotine pointed to a higher probability of initiating cigarette smoking with nicotine-containing e-cigarettes [57]. With regards to “switching” or “dual-use” following initiation of cigarette smoking, two studies found that the odds of reporting dual use among exclusive e-cigarette ever users (versus never users) were higher than the odds of reporting switching from exclusive e-cigarette use to exclusive current cigarette smoking [52, 80]. In both studies, analyses of current (past 30-day) e-cigarette users reported similarly higher odds of dual-use compared with switching.

Twelve adjusted studies evaluated initiation of and progression to regular cigarette smoking for individuals using e-cigarettes versus individuals who are not using e-cigarettes [37, 40, 45, 48, 54, 61, 65, 66, 69, 72, 73, 86], two of which applied measures of regular e-cigarette use [40, 48]. Both studies generally found no significant associations between regular e-cigarette use and progression to regular cigarette smoking; however, one result suggested that established e-cigarette users were significantly less likely to transition to exclusive cigarette smoking than non-established e-cigarette users [48]. In terms of studies applying definitions of non-regular e-cigarette use, based on the variability in the results, and variations in the definition of a “regular” cigarette smoker, the current data regarding initiation of and progression to regular cigarette smoking does not support drawing conclusions. This is illustrated in the study by Friedman et al. [69], which reported statistically lower odds of both current established and daily cigarette use among individuals who experimented exclusively with e-cigarettes(non-established use prior to the age of 18 years old) compared with individuals who did not experiment with e-cigarettes. Conversely, this study also found statistically significantly higher odds of current established cigarette use among individuals who experimented with e-cigarettes first, then with cigarettes, compared with individuals who did not experiment with e-cigarettes; however, no significant difference in the odds of daily smoking was found between e-cigarette-then-cigarette experimenters compared with individuals who did not experiment with e-cigarettes.

Finally, only one adjusted study evaluated age of initiation of cigarette smoking [75]. Notably, although McCabe et al. [75] reported a significantly lower age among current e-cigarette users, age of regular (daily) cigarette smoking was not significantly different between current and non-current e-cigarette users.

The current systematic review exhibited three major strengths. Firstly, its comprehensive search methodology yielded a large number of studies for review. Secondly, the current review had a clearly defined PICOS, which assured the identification of the strongest evidence relevant to the research question. Thirdly, guidelines for this review ensured that only demographically adjusted and methodologically consistent studies were included in the quantitative syntheses. Finally, the strict adherence to AMSTAR-2 and PRISMA guidelines ensured the transparency and replicability of this review while minimizing any risk of various forms of bias (e.g. individual study design; industry sponsorship) to provide an unbiased and comprehensive synthesis of this evidence base. Collectively, these strengths support the robustness of this review in terms of comprehensiveness and methodological rigor.

Although the meta-analysis indicated a higher odds for initiation of cigarette smoking among individuals using e-cigarettes—a result generally supported by the studies included in the qualitative synthesis—interpretation of the results is limited for many critical reasons. Specifically, the definition of e-cigarette use was not restricted to regular use. While doing so would have provided the strongest evidence on potential associations with the initiation of cigarette smoking, such a restriction would have yielded too few studies. Instead, the review was broadened to include any measure of e-cigarette use, with most studies measuring ever or current (past-30-day) use. Also, few studies examined initiation and/or progression to regular cigarette smoking, instead applying definitions of cigarette smoking that were more consistent with temporary experimentation and not true initiation, such as ever or current (past-30-day) smoking. Further, included studies were not restricted by specific confounding variables representing common liabilities between e-cigarette use and cigarette smoking, as this would have critically reduced the number of included studies in this review. The common-liability model considers the sequencing of drug use initiation, addiction, and addiction severity and posits that there are common sources of variation in the risk for specific addictions [11]. This model is critical for consideration given the empirical mixed signals that support or contradict the gateway hypothesis. However, the limited number of studies controlling for confounding variables related to common liability highlights the need for more robust studies to effectively measure the causal relationship between e-cigarette use and the initiation of cigarette smoking.

The majority of studies looked at how an e-cigarette-using population, individuals who never smoked cigarettes at baseline, developed cigarette smoking practices at follow-up. Though this information is indeed fundamental, it is equally important to understand the concepts of switching and dual-use. There are two possible trajectories that lead to an outcome of cigarette smoking among individuals using e-cigarettes. Between the baseline and follow-up surveys, (1) the e-cigarette user could begin cigarette smoking simultaneous with his/her e-cigarette use (dual use); or, (2) the e-cigarette user could eventually stop using the e-cigarette and after some time start smoking cigarettes (switchers). Information regarding whether individuals switched or dual used was limited, with only one adjusted study presenting specific data regarding single or dual use [52].

Further, 49 of 55 included studies reported on “youth”, “adolescent” and/or “young adult” populations, limiting the utility of the conclusions, as studies in youth and/or young adults are not necessarily translatable to older adults. Indeed, there is evidence that cigarette and e-cigarette smoking behaviors differ in different age groups. For example, one study determined that young adults (18–29 years of age) were more likely to be occasional smokers and reported lower daily consumption compared with older individuals who smoke cigarettes (30 years of age or older) [95]. Moreover, different age groups may vary in terms of their perceptions of and willingness to take risks, views of smoking addiction, perception of relative cigarette and e-cigarette health risks and/or benefits, and responses to behavioral interventions [96], which may differentially influence smoking behaviors and inherently, smoking cessation.

Finally, the duration of follow-up for the available studies was generally limited with most studies limited to 12 months in duration. This introduces the potentially limitation to observe whether cigarette smoking behavior actually persisted after initiation, i.e., true initiation and not simply temporary experimentation [2]. This may explain why so few of the included studies evaluated progression to regular cigarette smoking.

In conclusion, more robust studies are required to determine whether there is an association between e-cigarette use and initiation of cigarette smoking and progression to regular smoking. Based on findings from this review, the available studies neither sufficiently measure e-cigarette use—or cigarette smoking—in a manner consistent with examining causality, nor sufficiently account for known or suspected confounding variables to support robust determinations regarding e-cigarette use and cigarette smoking behaviors. Thus, the utility of the evidence base for policymakers, healthcare providers, and researchers is limited.

### Supplementary Information

Supplementary file1 

## Data Availability

All data and materials considered in this review are publicly available.
