# Supplementary material for: A systematic review and meta-analysis of the association between e-cigarette use among non-tobacco users and initiating smoking of combustible cigarettes
Source: Harm Reduct J. 2024 May 22;21:99. doi: 10.1186/s12954-024-01013-x (PMC11110305; doi:10.1186/s12954-024-01013-x)
Supplement: Supplementary file 1 — Supplementary file1 [file 12954_2024_1013_MOESM1_ESM.pdf]

Contents

1 Supplemental Section 1: Terminology 3

Table 1-1: Glossary of Key Terminology 3

2 Supplemental Section 2: Literature Search Strategy 6

3 Supplemental Section 3: Inclusion/Exclusion Criteria 13

4 Supplemental Section 4: Conceptual Framework 15

Figure 4-1: The Socio-Ecological Model 16

5 Supplemental Section 5: Contextual Questions 17

Table 5-1: Reliability and/or Validity Data Supporting Study Outcome Measures 21

6 Supplemental Section 6: Study Quality Assessment 28

Table 6-1: Downs and Black Checklist of Included Studies 28

Table 6-2: Overall Distribution of Downs & Black Ratings Among the Included Studies 32

7 Supplemental Section 7: Strength of Evidence 33

Table 7-1: SOE Grades and Definitions (Berkman et al., 2015) 33

Table 7-2: SOE Domain Scores for Adjusted Data 34

Table 7-3: SOE and Contextual Questions Ratings for Adjusted Data 34

Table 7-4: SOE Domain Scores for Unadjusted Data 35

Table 7-5: SOE and Contextual Questions Ratings for Unadjusted Data 35

8 Supplemental Section 8: Literature Search Output, Studies Reviewed at the Full-Text Level (n=995) 36

9 Supplemental Section 9: List of Excluded Studies (n=873) 516

10 Supplemental Section 8: Literature Search Output, Studies Reviewed at the Full-Text Level (n=995) .... **Error! Bookmark not defined.**

11 Supplemental Section 9: List of Excluded Studies (n=873) .... **Error! Bookmark not defined.**

12 Supplemental Section 10: List of Included Studies (n=122) 623

Characteristics of National Survey Datasets 651

13 Supplemental Section 13: Evidence Tables, Adjusted Studies 728

Table 13-1: Adjusted Results for Age of Cigarette Smoking Initiation Outcome Measures (n=1) .825

Table 13-2: Adjusted Results for Cigarette Smoking Initiation Outcome Measures (n=49)...........826
Table 13-3: Adjusted Results for Progression to Regular Cigarette Smoking Outcome Measures (n=12) 870
14 Supplemental Section 14: Study and Sample Characteristics, Unadjusted Studies (n=80) 878
Characteristics of National Survey Datasets 906
15 Supplemental Section 15: Demographic and Baseline Characteristics, Unadjusted Studies (n=80) 909
16 Supplemental Section 16: Evidence Tables, Unadjusted Studies 1040
Table 16-1: Unadjusted Results for Cigarette Smoking Initiation Outcome Measures (n=71) 1040
Table 16-2: Unadjusted Results for Cigarette Smoking Progression to Regular Cigarette Smoking Outcome Measures (n=18)........1076
17 Supplemental Section 17: Meta-Analytic Results 1085
Data Synthesis and Analysis........1085
Search Results 1088
Results 1091
Table 17-2: Covariates Controlled for in Studies Included in the Meta-Analysis of Initiation of Cigarette Smoking (n=12) 1095
Figure 17-2: Mean Age (SD) for Studies Included in the Meta-Analysis of Initiation of Cigarette Smoking 1098
Figure 17-3: Duration between Baseline and Follow-Up for Studies Included in the Meta-Analysis for Initiation of Cigarette Smoking 1099
Study Quality........1099
Table 17-3: Quality of Studies Included in Meta-Analysis for Initiation of Cigarette Smoking 1100
REFERENCES........1101

1 Supplemental Section 1: Terminology

A glossary of key terminology used in this report is provided in Error! Reference source not found..

Table 1-1: Glossary of Key Terminology

Key Term	Definition
Adjusted estimate	A measure of effect that has been altered, by stratification or regression modeling, in order to account for the effect of confounding factors (such as age, gender, and race/ethnicity).
Association	Statistical relationship between two or more events, characteristics, or other variables; in epidemiology, association implies a correlation between two variables.
Bias	Any tendency to influence the results of a trial (or their interpretation) other than the experimental intervention; a systematic error in the design or conduct of a study that results in conclusions that are different from the truth; for example, due to error in selecting the study population or in measuring the outcome or exposure (e.g., selection bias, information bias, interviewer bias).
Cohort study	Prospective cohort studies identify a group of people who are already receiving a particular treatment or have an exposure, follow them forward over time, and then compare their outcomes with a similar group that has not been affected by the treatment or exposure being studied. Like prospective cohort studies, retrospective cohort studies classify study participants on the basis of whether or not they were exposed to the factor under investigation. However, in retrospective studies, the research is initiated after both the exposure and the outcome have already occurred.
Confidence Interval	Range of likely values around the point estimate for the “true” population parameter; if one was to take 100 different samples and compute a 95% CI for each sample, then approximately 95 times out of 100 the CIs will contain the true value. Conveys 95% confidence that the true effect size lies within this range.
Confounder	A factor that can explain (entirely or partially) the observed association between ‘exposure’ and ‘outcome’. Confounders distort the association between ‘exposure’ and ‘outcome.’ By definition, to be considered a confounder, a variable must meet three criteria: (1) be associated with exposure, (2) be associated with the outcome, and (3) not be in the path between the exposure and the outcome, nor influenced directly by the exposure.
Cross-sectional analytic study	Studies in which the presence or absence of variables are determined in each member of the study population or in a representative sample at one particular time to compare the differences between exposure and non-exposure.
Heterogeneity	In systematic reviews, the amount of incompatibility between trials included in the review, whether clinical (i.e., the studies are clinically different) or statistical (i.e., the results are different from one another).
Incidence	Proportion of new cases developed during a given time period.
Longitudinal panel study	Studies in which the unit of analysis is followed at specified intervals, by collecting repeated measures from the same sample at different points in time.

Key Term	Definition
Meta-analysis	A statistical technique for combining and analyzing the results of a number of different studies on the same topic to enable identification of trends and patterns and more accurate estimation of significant effects.
Multivariate analysis	Used to investigate the relationship between independent variables and outcome by simultaneously allowing adjustment for confounding. Multivariate (also known as multivariable) models assess the effects of several variables simultaneously. The choice of an appropriate multivariable model (e.g., logistic regression, multiple linear regression, Cox proportional hazard analysis) is foremost dependent on the study design and the nature of the outcome variable.
Observational study	A family of studies in which investigators compare people who take an intervention with those who do not. The investigators neither allocate patients to receive the intervention nor administer the intervention. Instead, they compare records of patients who had taken an intervention and been treated in routine practice with similar patients who had not taken the intervention. The most common observational designs are case-studies, case-series, case-control studies, cohort studies, and historically controlled studies.
Odds Ratio	Measure of association between an exposure and an outcome; compares the odds (i.e., probability or likelihood) that an outcome will occur in the exposed group with the odds that the outcome will occur in the non-exposed group.
Point Estimate	Statistical measure of the association between an independent and a dependent variable. Often expressed as a relative effect (see Odds Ratio and Relative Risk).
Precision	Refers to the width of the CI around a point estimate or sample mean; conveys the level of certainty around the point estimate; precision is largely influenced by sample size.
Prevalence	Number of affected individuals (i.e., cases) alive at a point in time (point prevalence) or during a period of time (period prevalence).
p-value	The probability of seeing an effect equal to or more extreme than the one observed (e.g., point estimate) when in fact no true effect exists. A small p-value conveys that the observed effect is very unlikely to have arisen purely by chance. P-values less than 0.05 are generally reported as “statistically significant.”
Reliability	Extent to which a test will produce the same result if it is repeated at different periods or by different observers; consistency of the test.
Regression Analysis	Statistical methodology that allows the estimation of the effect of one or more variables on an outcome; different types of regression analyses exist for data analysis in epidemiology depending upon the study design and the outcome measurement. Linear regression is used for continuous outcomes, and logistic regression is used for binary outcomes.
Risk	Probability that an event will occur; risk, probability and likelihood are used interchangeably; there are various measures of risk in epidemiology.
Relative Risk	Relative measure of association between an exposure and an outcome; compares the incidence of an outcome in the exposed group to the incidence of the outcome in the non-exposed group.

Key Term	Definition
Statistically Significant	A phrase that implies that a statistical hypothesis test was calculated, and the resulting p-value was below the set threshold level (typically alpha=0.05) or the CI around the point estimate does not include the null value of 1.00.
Systematic Review	The application of strategies that limit bias in the assembly, critical appraisal, and synthesis of all relevant studies on a specific topic. Systematic reviews focus on peer-reviewed publications about a specific health problem and use rigorous, standardized methods for selecting and assessing articles. A systematic review may or may not include a meta-analysis**,** which is a quantitative summary of the results.
Trend	A finding observed across multiple studies; often refers to the direction or magnitude of an effect regardless of statistical hypothesis testing.
Unadjusted Estimate	A measure of effect that has not taken into account the effect of confounding factors.
Validity	The extent to which a measure or instrument assesses what it is intended to assess.

2 Supplemental Section 2: Literature Search Strategy

INITIAL DATABASE SEARCH – JANUARY 1 2007 to AUGUST 31, 2018

Database(s): **EMBASE** 1974 to 2018 August 31**, Ovid MEDLINE(R) ALL** 1946 to August 31, 2018**, PsycINFO** 1806 to August Week 4 2018

Search Strategy:

#	Searches	Results
1	(ecig$ or e-cig$ or e-voke$ or electronic nicotine or electronic cigarette$ or vape$ or vaping or vapourized nicotine or vaporized nicotine or vaporised nicotine).ti,ab.	8,458
2	(electronic adj3 cig$).ti,ab.	4,649
3	vaping/	665
4	electronic cigarettes/	5,863
5	electronic nicotine delivery systems/	5,521
6	1 or 2	8,483
7	or/3-5	6,560
8	6 or 7	9,214
9	tobacco/	69,962
10	nicotine/	75,935
11	(smok$ or tobacco$ or nicotin$ or cigar$ or cigs).ti,ab.	896,495
12	(mediat$ or moderat$ or incline$ or link$ or start$ or facilitat$ or promot$ or begin$ or began or initiat$ or progress$ or gateway or uptake$ or susceptibil$ or trajectory$ or transition$ or pathway$ or catalyst or sequence or likelihood or likely or onset or renormal$ or normali$ or prevalence or expectanc$ or consequenc$ or continu$ or "dual use$" or "dual usage" or "dual product use$" or "dual product usage" or perpetuat$ or contribut$ or "associated with" or "associates with" or "associate with" or "associated between" or "associates between" or "associate between" or "relationship with" or "relationship between" or intent or intension or tendanc$ or correlat$ or continu$ or open$ or willing$).mp.	29,465,972
13	or/9-11	920,265
14	12 and 13	665,102
15	14 and 8	5,653
16	limit 15 to english language	5,502
17	limit 16 to human	4,399
18	limit 17 to yr="2007 -Current"	4,398
19	remove duplicates from 18	2,526

DATABASE SEARCH UPDATE– JANUARY 1 2018 to AUGUST 30, 2019

Database(s): **Embase** 1974 to 2019 August 30**, Ovid MEDLINE(R) ALL** 1946 to August 30, 2019**, PsycINFO** 1806 to August Week 4 2019

Search Strategy:

#	Searches	Results
1	(ecig$ or e-cig$ or e-voke$ or electronic nicotine or electronic cigarette$ or vape$ or vaping or vapourized nicotine or vaporized nicotine or vaporised nicotine).ti,ab.	11535
2	(electronic adj3 cig$).ti,ab.	6000
3	vaping/	1398
4	electronic cigarettes/	8212
5	electronic nicotine delivery systems/	7549
6	1 or 2	11572
7	or/3-5	9232
8	6 or 7	12555
9	tobacco/	73828
10	nicotine/	79034
11	(smok$ or tobacco$ or nicotin$ or cigar$ or cigs).ti,ab.	959886
12	(mediat$ or moderat$ or incline$ or link$ or start$ or facilitat$ or promot$ or begin$ or began or initiat$ or progress$ or gateway or uptake$ or susceptibil$ or trajectory$ or transition$ or pathway$ or catalyst or sequence or likelihood or likely or onset or renormal$ or normali$ or prevalence or expectanc$ or consequenc$ or continu$ or "dual use$" or "dual usage" or "dual product use$" or "dual product usage" or perpetuat$ or contribut$ or "associated with" or "associates with" or "associate with" or "associated between" or "associates between" or "associate between" or "relationship with" or "relationship between" or intent or intension or tendanc$ or correlat$ or continu$ or open$ or willing$).mp.	31569855
13	or/9-11	984604
14	12 and 13	717638
15	14 and 8	8041
16	limit 15 to english language	7819
17	limit 16 to human	6209
18	limit 17 to last year	2257
19	remove duplicates from 18	1525

DATABASE SEARCH UPDATE– JANUARY 1 2019 to OCTOBER 7, 2020

Database(s): **Embase** 1974 to 2020 Oct 07**, Ovid MEDLINE(R) ALL** 1946 to October 02, 2020**, PsycINFO** 1806 to September Week 4 2020

Search Strategy:

#	Searches	Results
1	(ecig$ or e-cig$ or e-voke$ or electronic nicotine or electronic cigarette$ or vape$ or vaping or vapourized nicotine or vaporized nicotine or vaporised nicotine or nicotine vapor or nicotine vapour).ti,ab.	16582
2	(electronic adj3 cig$).ti,ab.	7961
3	vaping/	3402
4	electronic cigarettes/	11454
5	electronic nicotine delivery systems/	10332
6	1 or 2	16630
7	or/3-5	13386
8	6 or 7	18015
9	tobacco/	77308
10	nicotine/	82551
11	(smok$ or tobacco$ or nicotin$ or cigar$ or cigs).ti,ab.	1032704
12	(mediat$ or moderat$ or incline$ or link$ or start$ or facilitat$ or promot$ or begin$ or began or initiat$ or progress$ or gateway or uptake$ or susceptibil$ or trajectory$ or transition$ or pathway$ or catalyst or sequence or likelihood or likely or onset or renormal$ or normali$ or prevalence or expectanc$ or consequenc$ or continu$ or "dual use$" or "dual usage" or "dual product use$" or "dual product usage" or perpetuat$ or contribut$ or "associated with" or "associates with" or "associate with" or "associated between" or "associates between" or "associate between" or "relationship with" or "relationship between" or intent or intension or tendanc$ or correlat$ or continu$ or open$ or willing$).mp.	34136705
13	or/9-11	1058347
14	12 and 13	777361
15	14 and 8	11668
16	limit 15 to english language	11368
17	limit 16 to human	9026
18	limit 17 to yr="2019 -Current"	3367
19	remove duplicates from 18	2211

DATABASE SEARCH UPDATE– JANUARY 1 2020 to NOVEMBER 24, 2021

Databases: APA PsycInfo®, Embase®, MEDLINE®

Set#	Searched for	Results
S1	(AB,TI(ecig* or e-cig* or e-voke* or electronic nicotine or electronic cigarette* or vape* or vaping or vapourized nicotine or vaporized nicotine or vaporised nicotine or nicotine vapor or nicotine vapour))	24246
S2	AB,TI(electronic near/3 cig*)	9710
S3	S2 OR S1	24259
S4	MESH(vaping)	2842
S5	EMB(vaping)	3773
S6	MESH("electronic cigarettes")	225
S7	EMB ("electronic cigarettes")	0
S8	MESH("electronic nicotine delivery systems")	6247
S9	EMB("electronic nicotine delivery systems")	1
S10	S9 OR S8 OR S7 OR S6 OR S5 OR S4	10883
S11	S10 OR S3	25141
S12	MESH(tobacco)	83959
S13	EMB(tobacco)	123620
S14	MESH(nicotine)	37805
S15	EMB(nicotine)	62463
S16	AB,TI(smok* or tobacco* or nicotin* or cigar* or cigs)	1121031
S17	S16 OR S15 OR S14 OR S13 OR S12	1164387
S18	(mediat* or moderat* or incline* or link* or start*or facilitat* or promot* or begin* or began or initiat* or progress* or gateway or uptake* or susceptibil* or trajectory* or transition* or pathway* or catalyst or sequence or likelihood or likely or onset or renormal* or normali* or prevalence or expectanc* or consequenc* or continu* or "dual use*" or "dual usage" or "dual product use*" or "dual product usage" or perpetuat* or contribut* or "associated with" or "associates with" or "associate with" or "associated between" or "associates between" or "associate between" or "relationship with" or "relationship between" or intent or intension or tendanc* or correlat* or continu* or open* or willing*)	37056963
S19	S18 AND S17	846086
S20	S19 AND S11	17342

Set#	Searched for	Results
S21	(S20) and (pd(20200101-20211124)) and (human(yes) AND human(yes)) and (la.exact("English"))	5356*
S22	S20 and (human(yes) AND human(yes)) and (la.exact("English"))	14289*
S23	S22 and py(=2020)	1675°
S24	S22 and py(=2021)	1570°

* Duplicates are removed from the search, but included in the result count.

° Duplicates are removed from the search and from the result count.

DATABASE SEARCH UPDATE– JANUARY 1 2021 to APRIL 26, 2023

Databases: **Embase** 1974 to 2023 April 26, **Ovid MEDLINE(R) ALL** 1946 to April 26, 2023, **APA PsycInfo** 1806 to April Week 3 2023

Search Strategy:

#	Searches	Results
1	(ecig* or e-cig* or e-voke* or electronic nicotine or electronic cigarette* or vape* or vaping or vapourized nicotine or vaporized nicotine or vaporised nicotine or nicotine vapor or nicotine vapour).ti,ab.	28240
2	(electronic adj3 cig*).ti,ab.	11721
3	1 or 2	28316
4	vaping/	9469
5	electronic nicotine delivery systems/	19054
6	electronic cigarette/	21771
7	or/4-6	25112
8	7 or 3	30891
9	tobacco/	90620
10	nicotine/	91872
11	..nlpx "query=(smok* or tobacco* or nicotin* or cigar* or cigs).ti,a.b","desiredResults=10000","minHitsDivisor=7","permitHyponyms=NO","lowestVocabularySearchLevel=none","phrasesBroken=NO","speedWanted=Fastest","comment=No Related Terms","elimEnable=NO","constraintMinTerms=2"	576
12	(smok* or tobacco* or nicotin* or cigar* or cigs).ti,ab.	1194246
13	or/9-12	1222476
14	(mediat* or moderat* or incline* or link* or start*or facilitat* or promot* or begin* or began or initiat* or progress* or gateway or uptake* or susceptibil* or trajectory* or transition* or pathway* or catalyst or sequence or likelihood or likely or onset or renormal* or normali* or prevalence or expectanc* or consequenc* or continu* or "dual use*" or "dual usage" or "dual product use*" or "dual product usage" or perpetuat* or contribut* or "associated with" or "associates with" or "associate with" or "associated between" or "associates between" or "associate between" or "relationship with" or "relationship between" or intent or intension or tendanc* or correlat* or continu* or open* or willing*).ti,ab.	37263672
15	14 and 13	878064
16	15 and 8	20239
17	limit 16 to english language	19810
18	limit 17 to human	16837
19	limit 18 to yr="2021"	2781
20	remove duplicates from 19	1519
21	limit 17 to yr=2022-2023	4407

#	Searches	Results
22	remove duplicates from 21	2406

3 Supplemental Section 3: Inclusion/Exclusion Criteria

Inclusion and exclusion criteria were developed with review and input from external subject matter experts in tobacco research and systematic reviews. Search results were screened with inclusion/exclusion criteria developed using the PICOS (Population or participants and conditions of interest, Interventions or exposures, Comparisons or control groups, Outcomes of interest, and Study designs) review method. Outcome measures identified *a priori* included: intention to smoke cigarettes, susceptibility to smoke cigarettes, willingness/openness/curiosity to smoke cigarettes, age of initiation for cigarette smoking, initiation of cigarette smoking, and initiation and progression to regular cigarette smoking (not included in previous published systematic reviews and/or meta-analyses (1-3).

The following inclusion criteria were used:

**P**opulation or participants and conditions of interest	Youth, young adults, and adults who are non-tobacco users (includes never, former, or ever [experimenters/triers] users of tobacco products)
**I**nterventions or exposures	Nicotine free and/or nicotine containing e-cigarettes (also referred to as vapor products)
**C**omparisons or control groups	Never, former, ever, or current users of tobacco products who use or do not use e-cigarettes.
**O**utcomes of interest	• Willingness to smoke combustible cigarettes<br>• Intentions to smoke combustible cigarettes<br>• Openness to smoke combustible cigarettes.<br>• Susceptibility to smoke combustible cigarettes.<br>• Age at initiation of smoking combustible cigarettes<br>• Percent who initiated smoking combustible cigarettes<br>• Initiation and progression to regular smoking of combustible cigarettes
**S**tudy designs	Randomized/non-randomized controlled trials (although interventional studies are not expected), observational studies (e.g., cohort, case-control, and cross-sectional), systematic reviews, and meta-analyses. Narrative review articles, letters to the editors, government reports, conference abstracts, and commentaries will be considered if they contain original data.

The following studies were excluded from the systematic review:

1. Non-clinical study, such as laboratory research, animal study, *in vitro* or *ex vivo* study, post-mortem study, or biomechanical study.
2. Review article, with no original data relevant to the research question.
3. Letters to the editor, opinions, editorials, press releases, manufacturer's advertisement and other non-peer reviewed publications, unless publication contained original data relevant to the research question.
4. Study whereby participants did not use e-cigarettes.

5. Study whereby participants used e-cigarettes, but the study did not contain data relevant to the research question.
6. Article in which the abstract and full text was non-English.
7. Duplicate article or article with the exact same study outcome data as another published article.

4 Supplemental Section 4: Conceptual Framework

It is widely recognized that there are many levels of influence on an individual's health status and health behavior. Beyond individual-level variables (e.g., age, gender, and race/ethnicity), socio-cultural beliefs and practices, the economic environment, an individual's physical environment, and the larger legal and policy environment all have the potential to affect individual smoking behaviors. Therefore, a model that considers the influence of factors at various levels is useful in evaluating studies for their ability to adequately measure and control for confounding and to generate optimally valid results.

Significant variation in cigarette smoking behaviors has been found among different population subgroups, suggesting that factors that facilitate cigarette smoking exist in the complex individual and environmental influences on behavior. It is difficult to isolate the effects that e-cigarette use may have on cigarette smoking behavior from socio-cultural and other environmental influences. For example, having family members who smoke can have a strong influence on individual cigarette smoking behaviors, particularly among youth (4, 5). As a result, epidemiological studies that adjust for such factors are able to more rigorously isolate the independent effect of e-cigarette use on initiation and progression to regular cigarette smoking.

It is essential that the factors that influence cigarette smoking behaviors be considered to the greatest extent possible based on available data. The Socio-Ecological Model created by McLeroy et al. (1988) focuses on the interrelationships between individuals and their social, physical, and policy environments (6). As applied to this review, the socio-ecological model includes three main levels of factors that influence an individual's cigarette smoking behaviors: characteristics of the individual ("micro"); characteristics of the individual's social environment ("meso"); and characteristics of the systems-level environment in which the individual exists ("macro") (**Figure 4-1**: The Socio-Ecological Model. Examples of micro-level factors are age, race/ethnicity, gender, and education. Meso-level factors include living in a home where someone smokes, having friends who smoke, and whether someone lives in an urban or rural area, among others. Examples of macro-level factors include cigarette taxes, workplace cigarette smoking policies, and local or state tobacco control activities (e.g., cigarette smoking bans).

By applying this model, reviewers were able to (1) recognize the strong influence of individual and environmental factors on health behavior and prioritize studies and models that accounted for these factors, (2) consider appropriate adjustment for the assessment of confounding due to those variables as part of the evaluation of study quality, and (3) conduct appropriate sensitivity analyses that consider the impact of limiting analyses to studies that were able to adequately measure and control for confounding and the potential influence of variables at all relevant levels.

Based on the available empirical evidence and expertise of the review team, potential confounders were identified and categorized as micro-, meso- or macro-level variables, according to the socio-ecological model (6) (see **Figure 7-1**). Key confounders were identified as race/ethnicity, age, and gender based on the magnitude and strength of their association with cigarette smoking behaviors (7-9). A study in this review was considered to adequately control for confounding bias if it adjusted for age, gender, and race/ethnicity (if the study did not adjust for race/ethnicity, it was considered acceptable if the study was conducted in a non-diverse ethnic geographic region). Studies that also adjusted for meso- (e.g., living with a smoker) or macro-level factors (e.g., cigarette taxes) were flagged for possible inclusion in sensitivity analyses.

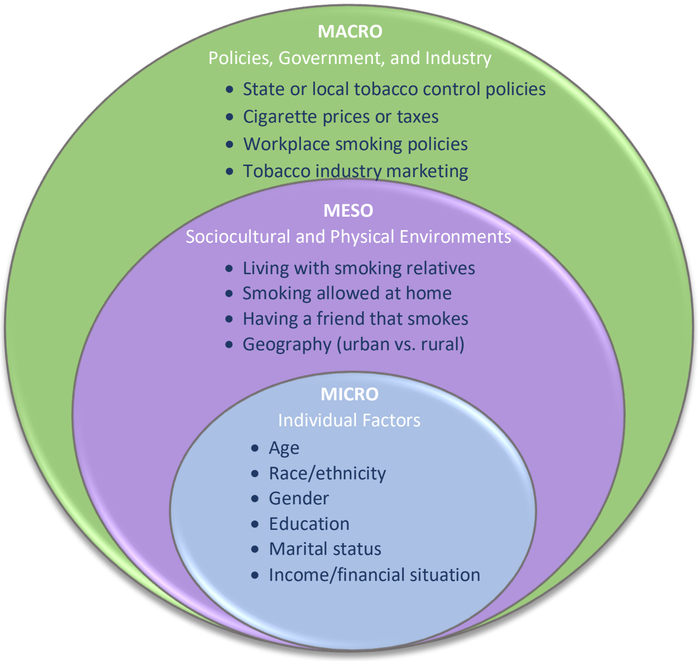

Figure 4-1: The Socio-Ecological Model

5 Supplemental Section 5: Contextual Questions

[NB: The reliability and validity data for each outcome measure included in this systematic review can be found in

Table 5-1: Reliability and/or Validity Data Supporting Study Outcome Measures]

Smoking behaviors are complicated constructs that are represented differently among epidemiological studies (10, 11). Single-item and multi-item measures (sometimes also referred to as "scales," "instruments," or "indicators"), capture different aspects of these constructs. Heterogeneity in results across studies reflects, in part, differences in how each construct was measured. However, less attention has been paid to the marked variation in psychometric properties of commonly used measures and the concept that not all measures are equally valid and reliable. This report addresses this evidence gap by examining the following contextual question (CQ): Have measures used to examine initiation and progression to regular cigarette smoking been psychometrically assessed as reliable and valid? CQs summarize additional information that can provide context for the findings of the key research question (12). Information regarding CQs may also inform the degree to which the results from individual measures contribute to the overall SOE for a particular outcome.

Reliability. Reliability is the overall consistency of a measure (scale), over time and between individuals, and can be measured in a number of ways depending upon the type of measurement scale. Not all indicators of reliability are relevant to this review. One of the most relevant dimensions of reliability is the extent to which a measure yields the same score each time it is administered, under the assumption that true change has not occurred between measurement time points (13).

For single-item measures (such as "how old at first cigarette use") and multi-item measures (such as the Fagerström Test for Nicotine Dependence [FTND]), temporal consistency is often assessed by estimating test-retest reliability (i.e., completing the test twice using the same instrument at two different times and comparing results, often through an appropriate correlation statistic or 'percent agreement'). A kappa (κ) statistic, for example, may be calculated. Kappa quantifies the extent of agreement between the two measurements across a time interval that is greater than the extent expected by chance alone (14, 15). This assessment yields an assessment of the "repeatability" of the scale and is affected by, among other things, the scale's length, the amount of time between measurements, and factors related to the study population (16).

Additionally, multi-item scales that are used to measure a complex construct are expected to be "unidimensional," such that each of the underlying items, or groups of items (subscales), assesses a single underlying (or "latent") construct (e.g., cigarette smoking initiation). For multi-item scales, reliability is most often defined as the extent to which the items or subscales of the instrument are a consistent measure of a single, unidimensional "construct." Unidimensionality of multi-item scales is sometimes assessed using principal components (factor) analysis. Cronbach's alpha (α) is a common statistic used to measure the strength of such consistency of the internal interrelationships, or average correlation, or the strength of the inter-item relationships, of its component items (17). Numerous factors influence the value of alpha, including the number of items included in the scale (smaller numbers of items limit the maximum value of alpha that is achievable, even in the best of circumstances) and the level of interrelatedness of the items or subscales. Importantly, the degree of an instrument's reliability can also be affected by how closely the use of that instrument mirrored the way it was intended to be used. For this reason, there is no single set of agreed-upon threshold values that allow for categorization of reliability.

In keeping with published tradition, and using liberal thresholds, reliability estimates (i.e., values of Cronbach's alpha and kappa or other appropriate estimators of test-retest correlation) equal to or greater than 0.70 were considered to be "acceptable" indicators of reliability, and values below 0.70 were considered of "insufficient strength" for the purpose of this review (16, 18-22). Note that an alpha threshold of 0.80 (and sometimes 0.90) is commonly cited as the minimum level of consistency for use of a scale in a clinical setting. Thus, given the research (as opposed to clinical) context of this systematic review, the threshold for "acceptable" internal consistency and temporal reliability was set at the lower, less conservative, level of 0.70 (23).

Validity. Validity is at the core of understanding whether the results of a research study, which are based upon the measurement of one or more relevant underlying behavioral constructs or 'outcomes', are representative of what

the research was designed to investigate. The International Agency for Research on Cancer (IARC) has provided an excellent overview, through 2008, of scientific evaluation of the validity of multi-item scales for numerous cigarette smoking behaviors and outcomes (24). The methods of the current CQ review are aligned with the general approach of that publication.

There are two types of validity: internal and external. Internal validity is defined as the extent to which a measure reflects what it is supposed to be measuring (23). Internal validity has been assessed as the degree of correlation with other established multi-item scales or single-item measures (24, 25). There are also different types of indicators of internal validity. Specifically, criterion and construct validity are often considered in the evaluation of the psychometric properties of a measure. *Criterion validity* assesses the extent to which a measure agrees with another accepted measure or predicts outcomes of interest. *Construct (or convergent) validity* assesses the extent to which a measure captures the theoretical concept being measured. Construct validity is often assessed through convergent evidence, or demonstration that the measure correlates highly (e.g., correlation coefficients ≥0.50 or ≥0.70) with other measures of the same, or clearly related, constructs. In contrast, external validity is demonstrated by obtaining similar results when the same instrument is used in different, relevant population samples; i.e., the results can be generalized beyond the immediate study.

Although the constructs of cigarette smoking behaviors are measured differently, the behaviors are interconnected. A number of studies have found that ever-use (that is, cigarette smoking, even a few puffs) is a necessary precursor to progression to regular cigarette smoking (26). Thus, as an indicator of criterion validity, ever-use cigarette smoking measures can be further evaluated by testing whether they predict (or are associated with) cigarette smoking progression.

Relationship between reliability and validity. For research results to be informative for decision-making, all study methods must be rigorous, as well as both reliable and valid. A scale may be reliable (i.e., measured consistently) but not necessarily be valid (i.e., measuring what it is designed to measure). However, a measure cannot be valid unless it is reliable.

Unlike the systematic review performed to inform the study research question, the CQ was not assessed using systematic review methodology. Instead, a search was conducted in PubMed and Google Scholar for studies that informed the reliability and validity of the cigarette smoking-related outcome measures included in the systematic review. One team member extracted relevant information, and a senior author verified its accuracy and completeness during the preparation of this report.

The following criteria were used to assess the reliability and validity of the cigarette smoking-related outcome measures assessed in this review:

Criteria to Assess Reliability:

1. Criterion One: A statistical indication of consistency, with estimates ≥0.70, evidenced by one or more of the following:
    a. Internal consistency reliability: Cronbach's alpha (α) or the Kuder-Richardson Formula 20 (KR-20)
    b. Temporal (test-retest) reliability or 'repeatability': Cohen's kappa (κ), intraclass correlation coefficient (ICC), or other statistically appropriate correlation (e.g., Pearson's *r* or Spearman's *rho*)

2. Criterion Two: Demonstration of reliability (internal consistency and/or temporal) in critical population subgroups (i.e., males and females/youth and adults) as appropriate for the specific instrument (e.g., if the scale or measure was intentionally designed for use among youth, the standard for that instrument will be demonstration of reliability and validity among youth).

Criteria to Assess Validity:

1. Criterion One:
    a. Demonstration of construct or criterion validity through statistically significant ($p<0.05$) relationship (or association) between the scale (or measure) and at least one of following:
        i. Behavioral measures related to cigarette smoking (e.g., self-reported age at first daily smoking)
        ii. Biochemical measures related to cigarette smoking (e.g., expired carbon monoxide, salivary cotinine)
    b. Statistically significant ($p<0.05$) evidence of any of these types of validity through one of the following indicators:
        i. Correlation (such as Pearson's product-moment coefficient *r* or Spearman's *rho*) ≥±0.50
        ii. An OR, or other indicator of relative risk (whose 95% confidence bounds do not include the null value, or 1.0)
        iii. An effect size (e.g., Cohen's *d*) ≥0.2

2. Criterion Two: Demonstration of external (namely, population) validity in critical population subgroups (i.e., males and females/youth and adults) as appropriate for the specific instrument (e.g., if the scale or measure was intentionally designed for use among youth, the standard for that instrument will be demonstration of reliability and validity among youth).

The following scoring approach was informed by the IARC Handbook on Cancer Prevention (24):

"Acceptable" – measures for which available empirical findings were identified in the reviewed studies demonstrating at least one criterion for reliability and at least one criterion for validity in both sexes; or

"Inconclusive" – measures either for which available empirical findings in the reviewed studies did not meet the required criteria above or for which insufficient empirical findings were identified by the methods employed herein.

Table 5-1: Reliability and/or Validity Data Supporting Study Outcome Measures

Measure	Final Grade	Reliability<br><br>(Study, statistical method, test-retest interval, study population)	Validity<br><br>(Study, behavioral/biochemical measure, statistical method, result, study population)
Age at initiation of cigarette smoking	**ACCEPTABLE**	(Brigham et al., 2008)(27)<br><br>Self-administered questionnaire<br>κ=0.82 (self-reported age first smoked at least once a month)<br>2 years<br><br>*236 adults age 17 to 82, mainly white women US population*<br><br>(Brigham et al., 2009)(28)<br><br>Self-administered questionnaire<br><br>ICC=0.82 (self-reported age of onset of daily cigarette use)<br>ICC=0.85 (self-reported age of onset of weekly cigarette use)<br><br>2 months<br><br>*1,229 Adult smokers, mostly white, 18-78 years*<br><br>(Brigham et al., 2010)(29)<br>Study 1 (Oregon)<br>In-person interviews<br><br>ICC=0.33 (self-reported age at first cigarette)<br><br>19.5 years<br><br>$r_{s=}$0.01 (self-reported age at first daily smoking)	(Hwang and Park, 2014)(34)<br>*Age at smoking initiation and subsequent smoking levels (frequent smoker, daily smoker):*<br><br>OR for age at smoking initiation becoming a frequent smoker vs Grade 10-11:<br>Grade 9: 1.82, 95% CI: 1.16-2.85<br>Grade 8: 2.65, 95% CI: 1.64-4.29<br>Grade 7: 3.79, 95% CI: 2.02-7.12<br>Grade 1-6 or before: 3.14, 95% CI: 1.83-5.40<br><br>OR for age at smoking initiation becoming a daily smoker vs Grades 10-11:<br>Grade 9: 1.43, 95% CI: 0.92-2.20<br>Grade 8: 2.65, 95% CI: 1.29-3.21<br>Grade 7: 3.30, 95% CI: 1.85-5.90<br>Grades 1-6 or before: 2.94, 95% CI: 1.76-4.92<br><br>*743 students in Grade 10-11 in South Korea who are current smokers*<br><br>(Kendler et al., 2013)(35)<br>Mailed questionnaires, with subsequent face-to-face or telephone interviews

Measure	Final Grade	**Reliability**<br><br>**(Study, statistical method, test-retest interval, study population)**	**Validity**<br><br>**(Study, behavioral/biochemical measure, statistical method, result, study population)**
		346 child/adolescents, age 10 to 18<br><br>Study 2 (Pittsburgh)<br>In-person interviews<br>ICC=0.58 (age at first cigarette)<br>ICC=0.52 (self-reported age at first daily smoking)<br>3.9 years<br>*294 child/adolescents, age 10 to 18*<br><br>(Colby et al., 2012)(30)<br>In-person, telephone, or videotaped interviews<br>ICC=0.89 (self-reported age of onset, first puff)<br>ICC=0.74 (self-reported age of onset, daily smoking)<br>ICC=0.94 (self-reported age of onset, weekly smoking)<br>4-8 weeks<br>*220 Adult men and women (ages 34-44), mainly white (83.5%) from a distinct geographical region*<br><br>(Grant et al., 2003)(31)<br>In-person interviews	*Age at onset of regular smoking and level of nicotine dependence as measured by the Fagerström Test for Nicotine Dependence (FTND) and craving*:<br><br>FTND:<br>For male pairs: Cohen's d=0.20, p=0.004<br>For female pairs: Cohen's d=0.27, p=0.04<br><br>Craving:<br>For male pairs: Cohen's d=0.38, p<0.0001<br>For female pairs: Cohen's d=0.25, p=0.06<br>*175 male-male and 69 female-female monozygotic twin pairs who differed by at least 2 years in age at onset of regular smoking. For the male-male pairs, the early-onset smoking member had a mean current age of 36.1 years (SD=8.6). For the later-onset smoking member the mean age at interview was 36.3 years (SD=8.5). For the female-female pairs, the mean age at interview was 36.1 years (SD=7.8) for the early-onset member and 35.9 years (SD=7.8) for the late-onset member.*

Measure	Final Grade	**Reliability**<br><br>**(Study, statistical method, test-retest interval, study population)**	**Validity**<br><br>**(Study, behavioral/biochemical measure, statistical method, result, study population)**
		ICC=0.91 (self-reported age at onset of smoking)<br><br>ICC=0.89 (self-reported age at onset of daily use)<br><br>3 to 20 weeks<br><br>*Nationally representative sample of 2,657 participants, age 18-24 years, varying ethnicities*<br><br><br>(Johnson and Mott, 2001)(32)<br><br>Online survey questionnaire<br><br>ICC=0.82 (self-reported age of first cigarette use, adults 1992-1994); 0.64 (self-reported age of first cigarette use, youth 1990-1992); 0.67 (self-reported age of first cigarette use, youth 1994-1996); 0.52 (self-reported age of first cigarette use, youth 1994-1998); 0.64 (self-reported age of first cigarette use, youth 1996-1998)<br><br>2 to 4 years<br><br>*12,686 adults, 10,042 children, racially diverse*<br><br><br>(Soulakova et al., 2012)(33)<br><br>In-person and telephone interviews<br><br>ICC=0.78 (Self-reported for age of initiation of fairly regular smoking)	

Measure	Final Grade	Reliability<br><br>(Study, statistical method, test-retest interval, study population)	Validity<br><br>(Study, behavioral/biochemical measure, statistical method, result, study population)
		ICC=0.70 (Proxy reporting for age of initiation of fairly regular smoking)<br><br>ICC=0.48 (Mixed self- and proxy reporting for age of initiation of fairly regular smoking)<br><br>1 year ± 2 weeks<br><br>*6,783 racially diverse youth and adult former and current smokers*	
% who initiated cigarette smoking	**ACCEPTABLE**	(Colby et al., 2012)(30)<br><br>In-person, telephone, or videotaped interviews<br><br>κ=0.92 (% ever puffed cigarettes) κ=0.78 (% smoked again) 4 to 8 months<br><br>*220 Adult men and women (ages 34-44), mainly white (83.5%) from a distinct geographical region*<br><br>(36)<br><br>Classroom pen-and-paper self-administered questionnaire<br><br>KR-20=0.96 at Time 1 and 0.98 at Time 2 (self-reporting for trying smoking)<br><br>1 year	(37)<br><br>In-person interviews<br><br>*Correlation between self-reported smoking status and salivary cotinine levels:*<br><br>*r*=0.75, df=859, p<0.0001<br><br>*707 participants from a birth cohort in an Australian health care institution (15-18 yrs of age)*

Measure	Final Grade	Reliability<br><br>(Study, statistical method, test-retest interval, study population)	Validity<br><br>(Study, behavioral/biochemical measure, statistical method, result, study population)
		1,184 students from 3rd to 5th grade in a single school district in North Carolina, 81% Caucasian, 17% African American, 2% other ethnic groups<br><br>(Stanton et al., 1996)(37)<br><br>In-person interviews<br><br>κ=0.29 (self-reported recall of smoking initiation)<br><br>Comparison of recall of 18 year-olds of their smoking status at age 15 with responses given at age 15.<br><br>*707 participants from a birth cohort in an Australian health care institution (15-18 yrs of age)*	
Initiation and progression to regular cigarette smoking	**ACCEPTABLE**	(Colby et al., 2012)(30)<br>In-person, telephone, or videotaped interviews<br>κ=0.95 (% ever smoked daily)<br>κ=0.92 (% smoked weekly)<br>4 to 8 months<br>*220 participants from a birth cohort aged 34-44 years old from a distinct geographical region in the United States*<br><br>(Eppel et al., 2006)(38)<br><br>Classroom pen-and-paper self-administered questionnaire.<br><br>Test–retest reliability for smoking frequency:	(Eppel et al., 2006)(38)<br><br>Classroom pen-and-paper self-administered questionnaire. 1-month vs. 3-month recall for smoking frequency and intensity<br><br>*Recall of smoking frequency, intensity and number of cigarettes in ages 14-16 vs ages 12-13:*<br><br>OR initiating recalls at ages 14-16 against those at ages 12-13=3.9, 95% CI: 1.3-11.3 (recall of smoking frequency)

Measure	Final Grade	**Reliability**<br><br>**(Study, statistical method, test-retest interval, study population)**	**Validity**<br><br>**(Study, behavioral/biochemical measure, statistical method, result, study population)**
		κ=0.78 (0.66–0.91)<br><br>Test-retest reliability for smoking intensity:<br><br>κ=0.75 (0.61–0.89);<br><br>1 and 3 months<br><br>*63 novice smokers in Montreal, aged 12-13 years at baseline*<br><br><br>(Grant et al., 2003)(31)<br><br>In-person interviews<br><br>ICC=0.83 (self-reported number of days/months smoking daily)<br><br>3-20 weeks<br><br>*Nationally representative sample of 2,657 participants, age 18-24 years, varying ethnicities*<br><br><br>(Huerta et al., 2005)(39)<br><br>In-person interviews<br><br>ICC=0.73 males, 0.76 females (self-reported current smoking history)<br><br>1 to 4 years (from recruitment to discharge)	OR initiating recalls at ages 14-16 against those at ages 12-13=3.7, 95% CI: 1.2-11.2 (recall of smoking intensity)<br><br>OR initiating recalls at ages 14-16 against those at ages 12-13=3.2, 95% CI: 1.1-9.8 (recall of total number of cigarettes/day)<br><br>*63 novice smokers in Montreal, aged 12-13 years at baseline*<br><br><br>(Stanton et al., 1996)(37)<br><br>In-person interviews<br><br>*Correlation between self-reported smoking status and cotinine levels in saliva*<br><br>r=0.75, df=859, p<0.0001<br><br>*707 participants from a birth cohort from an Australian health care institution (15-18 yrs of age)*

Measure	Final Grade	**Reliability**<br><br>**(Study, statistical method, test-retest interval, study population)**	**Validity**<br><br>**(Study, behavioral/biochemical measure, statistical method, result, study population)**
		7,276 members of the Israeli Defense Force, ages 18-22, recruited from 1986 to 2000<br><br><br>(Stanton et al., 1996)(37)<br><br>In-person interviews<br>κ=0.29 (self-reported smoking status)<br><br>Comparison of recall of 18 year-olds of their smoking status at age 15 with responses given at age 15.<br><br>*707 participants from a birth cohort from an Australian health care institution (15-18 yrs of age)*	

*Predictive validity

AOR=adjusted odds ratio; CI=confidence interval; FTND=Fagerström Test for Nicotine Dependence; ICC=intraclass correlation coefficient; KR-20=Kuder-Richardson Formula 20; MTF=Monitoring the Future; NPV=negative predictive value; OR=odds ratio; PPV=positive predictive value; SD=standard deviation; UK=United Kingdom; US=United States; yr/yrs=year/years

6 Supplemental Section 6: Study Quality Assessment

The quality of included studies was assessed with the Downs and Black checklist, one of the most rigorous instruments for evaluating observational studies(40). The Downs and Black checklist is a comprehensive instrument that includes 27 items divided into the following five domains: (1) study reporting [10 items]; (2) external validity [3 items]; (3) internal validity – related to measurement bias (outcome and exposure) [7 items]; (4) internal validity – related to confounding bias [6 items]; and (5) power [1 item]. The instrument was used as reported in the original study, with the power question being simplified to a checklist as to whether the study was adequately powered. The maximum achievable score for a study was 28, and score ranges were grouped into the following four quality levels: “excellent” (26-28); “good” (20-25); “fair” (15-19); and “poor” (≤14).

Individual studies’ quality assessments by domain, as well as their overall rating, are provided in **Table 6-1**: Downs and Black Checklist of Included Studies. The overall distribution of ratings among all of the included studies is provided in **Table 6-2**: Overall Distribution of Downs & Black Ratings Among the Included Studies.

Table 6-1: Downs and Black Checklist of Included Studies

Study	Reporting (Q1-10)	External Validity (Q11-13)	Internal Validity/ Bias (Q14-20)	Internal Validity/ Confounding (Q21-26)	Power (Q27)	Total	Rating
(Akre et al., 2018)(41)	6	3	4	3	0	16	FAIR
(Aleyan et al., 2021)(42)	9	3	4	4	1	21	GOOD
(Auf et al., 2019)(43)	8	3	4	4	1	20	GOOD
(Azagba et al. 2020)(44)	9	3	4	3	1	20	GOOD
(Barrington-Trimis et al., 2016)(45)	10	3	4	4	1	22	GOOD
(Barrington-Trimis et al., 2018a)(46)	10	3	4	4	1	22	GOOD
(Barrington-Trimis et al., 2018b)(47)	10	3	4	4	1	22	GOOD
(Barrington-Trimis et al., 2019)(48)	8	3	4	4	1	20	GOOD
(Bennet et al., 2022)(49)	10	3	4	3	1	21	GOOD
(Berry et al., 2019)(50)	9	3	4	4	1	21	GOOD
(Best et al., 2017)(51)	9	3	4	3	1	20	GOOD
(Bold et al., 2018)(52)	9	3	4	4	1	21	GOOD
(Chaffee et al., 2018)(53)	9	3	4	4	1	21	GOOD
(Cheng et al., 2020)(54)	7	3	4	3	1	18	FAIR
(Chien et al., 2019)(55)	9	3	4	4	1	21	GOOD
(Coleman et al., 2019)(56)	8	3	4	3	1	19	FAIR
(Conner et al., 2017)(57)	9	3	4	3	1	20	GOOD

Study	Reporting (Q1-10)	External Validity (Q11-13)	Internal Validity/ Bias (Q14-20)	Internal Validity/ Confounding (Q21-26)	Power (Q27)	Total	Rating
(Conner et al., 2019a)(58)	9	3	5	3	1	21	GOOD
(Conner et al., 2019b)(59)	7	3	5	1	1	17	FAIR
(Conner et al., 2021)(60)	9	3	4	4	1	21	GOOD
(Dai et al., 2022)(61)	10	3	4	3	1	21	GOOD
(Duan et al., 2021)(62)	10	3	4	3	1	21	GOOD
(East et al., 2018)(63)	9	3	4	2	1	19	FAIR
(Epstein et al., 2020)(64)	8	3	4	4	1	20	GOOD
(Evans-Polce et al., 2019)(65)	9	3	4	4	1	21	GOOD
(Evans-Polce et al., 2020)(66)	9	3	4	4	1	21	GOOD
(Fearon et al., 2023)(67)	8	3	4	2	1	18	FAIR
(Friedman et al., 2019)(68)	7	3	4	3	1	18	FAIR
(Gallus et al., 2022)(69)	8	3	4	3	1	19	FAIR
(Gmel et al., 2016)(70)	9	3	4	3	1	20	GOOD
(Gravely et al., 2020)(71)	7	3	4	3	1	18	FAIR
(Hair et al., 2021)(72)	8	3	4	2	1	18	FAIR
(Hammond et al., 2017)(73)	10	3	4	3	1	21	GOOD
(Hansen et al., 2020)(74)	10	3	4	3	1	21	GOOD
(Harlow et al., 2022)(75)	9	3	4	4	1	21	GOOD
(Jankowski et al., 2019)(76)	7	3	4	2	0	16	FAIR
(Kang et al., 2020)(77)	8	3	4	3	1	19	FAIR
(Kasza et al., 2018)(78)	6	3	4	3	1	17	FAIR
(Kasza et al., 2020)(79)	8	3	4	3	1	19	FAIR
(Keller-Hamilton et al., 2021)(80)	9	3	4	4	1	21	GOOD
(Kinnunen et al., 2019)(81)	8	3	4	4	1	20	GOOD
(Kintz et al., 2020)(82)	8	3	4	4	1	20	GOOD
(Kong et al., 2019)(83)	8	3	4	3	1	19	FAIR
(Kowitt et al., 2019)(84)	7	3	4	2	1	17	FAIR
(Kulak et al., 2018)(85)	8	3	4	2	1	18	FAIR
(Lee & Fry, 2019)(86)	8	3	4	3	1	19	FAIR
(Legleye et al., 2021) (87)	8	3	4	3	1	19	FAIR

Study	Reporting (Q1-10)	External Validity (Q11-13)	Internal Validity/ Bias (Q14-20)	Internal Validity/ Confounding (Q21-26)	Power (Q27)	Total	Rating
(Leventhal et al., 2015)(88)	10	3	4	4	1	22	GOOD
(Leventhal et al., 2016)(89)	9	3	4	2	1	19	FAIR
(Liu et al., 2020)(90)	7	3	4	3	1	18	FAIR
(Loukas et al., 2018b)(91)	6	3	4	3	1	17	FAIR
(Loukas et al., 2018a)(92)	9	3	4	3	1	20	GOOD
(Loukas et al., 2022)(93)	8	3	4	4	0	19	FAIR
(Lozano et al., 2017)(94)	9	3	4	4	1	21	GOOD
(Martinelli et al., 2023)(95)	10	3	4	3	1	21	GOOD
(McCabe et al., 2017)(96)	9	3	4	3	1	20	GOOD
(McCabe et al., 2019)(97)	6	3	4	2	1	16	FAIR
(McMillen et al., 2019)(98)	9	3	4	3	1	20	GOOD
(Melka et al., 2020)(99)	10	3	4	4	1	22	GOOD
(Miech et al., 2017)(100)	7	3	4	4	1	19	FAIR
(Morgenstern et al., 2018)(101)	9	3	4	4	1	21	GOOD
(Mus et al., 2023)(102)	7	3	4	3	0	17	FAIR
(Odani et al., 2018)(103)	6	3	4	3	1	17	FAIR
(Osibogun et al., 2020)(104)	9	3	4	3	1	20	GOOD
(Osibogun et al., 2022)(105)	9	3	4	3	0	19	FAIR
(Owotomo et al., 2020)(106)	9	3	4	4	1	21	GOOD
(Parnham et al., 2023)(107)	8	3	4	3	0	18	FAIR
(Patanavanich et al., 2022)(108)	8	3	4	3	0	18	FAIR
(Penzes et al., 2018)(109)	9	3	3	4	1	20	GOOD
(Perez et al., 2021)(110)	7	3	4	2	1	17	FAIR
(Pierce et al., 2021)(111)	9	3	4	4	1	21	GOOD
(Primack et al., 2015a)(112)	7	3	4	4	1	19	FAIR

Study	Reporting (Q1-10)	External Validity (Q11-13)	Internal Validity/ Bias (Q14-20)	Internal Validity/ Confounding (Q21-26)	Power (Q27)	Total	Rating
(Primack et al., 2015b)(113)	10	3	4	4	1	22	GOOD
(Primack et al., 2018)(114)	10	3	4	4	1	22	GOOD
(Selya et al., 2018)(115)	9	3	4	4	1	21	GOOD
(Shafie-Khorassani et al., 2023)(116)	9	3	4	4	1	21	GOOD
(Shahab et al., 2020)(117)	7	3	3	3	1	17	FAIR
(Shitttu et al., 2022)(118)	9	3	4	3	1	20	GOOD
(Spindle et al., 2017)(119)	8	3	4	4	1	20	GOOD
(Staff et al., 2021)(120)	9	3	4	4	1	21	GOOD
(Stanton et al., 2019)(121)	10	3	4	3	1	21	GOOD
(Stanton et al., 2020)(122)	8	3	4	3	1	19	FAIR
(Stanton et al., 2020)(123)	7	3	4	3	1	18	FAIR
(Stanton et al., 2023)(124)	9	3	4	3	1	20	GOOD
(Stokes et al., 2020)(125)	8	3	4	4	1	20	GOOD
(Sun et al., 2022)(126)	7	3	4	3	1	18	FAIR
(Sun et al., 2023)(127)	10	3	4	4	1	22	GOOD
(Treur et al., 2018)(128)	10	3	4	3	1	21	GOOD
(Unger et al., 2016)(129)	7	3	4	2	0	16	FAIR
(Wan Puteh et al., 2023)(130)	8	3	3	2	0	16	FAIR
(Watkins et al., 2018)(131)	9	3	4	4	1	21	GOOD
(Wei et al., 2020)(132)	8	3	4	4	1	20	GOOD
(Westling et al., 2022)(133)	9	3	4	2	0	18	FAIR
(Wills et al., 2016a)(134)	9	3	4	3	1	20	GOOD
(Wills et al., 2016b)(135)	10	3	4	4	1	22	GOOD
(Wills et al., 2017)(136)	10	3	4	4	1	22	GOOD
(Xu et al., 2022)(137)	8	3	4	4	0	19	FAIR
(Yang et al., 2022)(138)	7	3	4	4	0	18	FAIR
(Young-Wolff et al., 2018)(139)	8	3	4	4	1	20	GOOD

Table 6-2: Overall Distribution of Downs & Black Ratings Among the Included Studies

Study Quality	N	%
Excellent	0	0%
Good	58	59%
Fair	41	41%
Poor	0	0%
**Total**	**99**	**100%**

7 Supplemental Section 7: Strength of Evidence

Performing a systematic, objective, and transparent assessment of the overall SOE allows investigators to grade their confidence in the overall conclusions for each outcome. Several approaches have been used to grade the quality or SOE, and each has strengths and weaknesses. The Agency for Healthcare Research and Quality (AHRQ) Evidence-based Practice Center (EPC) Program developed a guide for assessing SOE in systematic reviews in which the effectiveness of one exposure or intervention is compared with another (140). The EPC approach is based largely on methods developed by the Grading of Recommendations, Assessment, Development and Evaluation (GRADE) Working Group. The EPC approach to grading SOE has been shown to be applicable to observational, non-experimental evidence. Although this review has not been undertaken specifically to inform a guideline or recommendation, the EPC approach "may apply to reviews of other health services research questions such as the effects of exposures (characteristics or risk factors) on health outcomes." With the acknowledgement of inherent limitations to using observational data to assess a potential differential association between an exposure and an outcome, the EPC approach seemed most appropriate for the purpose of expressing confidence in the conclusions based on the high number of observational studies on this topic. Therefore, the SOE evaluation was assessed using five domains: (1) study limitations (previously called risk of bias), (2) directness, (3) consistency, (4) precision, and (5) reporting bias.

The overall strength of the body of evidence was assessed for each individual outcome measure and overall according to "high," "moderate," "low," or "insufficient" grades using the EPC approach. Error! Reference source not found. details the EPC/GRADE grades and definitions.

The SOE evaluation was independently performed by one research associate and checked by a second research associate. The final SOE judgment was necessarily qualitative but reflected a sound, reasoned weighing of domain ratings.

SOE domain scores and overall ratings for adjusted data are included in the main report, as those results minimized the potential for confounding bias and likely represent the "best evidence," which may be more likely to signify the "true" association of e-cigarette use with "smoking" behaviors. Domain scores for these adjusted data are provided in **Table 7-2**: SOE Domain Scores for Adjusted Data; overall SOE and CQ ratings for these data are provided in **Table 7-3**: SOE and Contextual Questions Ratings for Adjusted Data.

The SOE was also assessed for studies that did not control for the key confounders, and allowed all other variables—measured and unmeasured—to vary, potentially obscuring the actual effect of e-cigarette use. Domain scores for these unadjusted data are provided in **Table 7-4**: SOE Domain Scores for Unadjusted Data; overall SOE and CQ ratings for these data are provided in **Table 7-5**: SOE and Contextual Questions Ratings for Unadjusted Data.

Table 7-1: SOE Grades and Definitions (140)

Grade	Interpretation	Description
**High**	We are very confident that the estimate of effect lies close to the true effect for this outcome.	The body of evidence has few or no deficiencies.<br>We believe that the findings are stable, that is, another study would not change the conclusions.
**Moderate**	We are moderately confident that the estimate of effect lies close to the true effect for this outcome.	The body of evidence has some deficiencies.

Grade	Interpretation	Description
		We believe that the findings are likely to be stable, but some doubt remains.
**Low**	We have limited confidence that the estimate of effect lies close to the true effect for this outcome.	The body of evidence has major or numerous deficiencies (or both).<br>We believe that additional evidence is needed before concluding either that the findings are stable or that the estimate of effect is close to the true effect.
**Insufficient**	We have no evidence, we are unable to estimate an effect, or we have no confidence in the estimate of effect for this outcome.	No evidence is available or the body of evidence has unacceptable deficiencies, precluding reaching a conclusion.

Table 7-2: SOE Domain Scores for Adjusted Data

	Study Limitations	Directness	Consistency	Precision	Reporting Bias	SOE
Initiation of Cigarette Smoking						
ADJUSTED	Low	Direct	Consistent	Precise	Unsuspected	High
Age at Initiation of Cigarette Smoking						
ADJUSTED	Medium	Direct	NA	Precise	Unsuspected	Moderate
Initiation and Progression to Regular Cigarette Smoking						
ADJUSTED	Medium	Direct	Inconsistent	Precise	Unsuspected	Moderate

NA=not applicable (only one adjusted study evaluating this outcome); SOE=strength of evidence

Table 7-3: SOE and Contextual Questions Ratings for Adjusted Data

Measure Name	CQ Assessment	Measure SOE
Adjusted Analyses		
Initiation of cigarette smoking	Acceptable	High
Age at initiation of cigarette smoking	Acceptable	Moderate
Progression to regular cigarette smoking	Acceptable	Moderate

CQ=contextual question; NA=not applicable (no data informing this outcome measure was identified in the review); SOE=strength of evidence

Table 7-4: SOE Domain Scores for Unadjusted Data

	Study Limitations	Directness	Consistency	Precision	Reporting Bias	SOE
Initiation of Cigarette Smoking						
UNADJUSTED	Medium	Direct	Consistent	Precise	Unsuspected	Moderate
Age at Initiation of Cigarette Smoking						
UNADJUSTED	Medium	Direct	Inconsistent	Precise	Unsuspected	Moderate
Initiation and Progression to Regular Cigarette Smoking						
UNADJUSTED	Medium	Direct	Inconsistent	Precise	Unsuspected	Moderate

NA=not applicable (no data informing this outcome measure was identified in the review); SOE=strength of evidence

Table 7-5: SOE and Contextual Questions Ratings for Unadjusted Data

Measure Name	CQ Assessment	Measure SOE
Unadjusted Analyses		
Age at initiation of cigarette smoking	Acceptable	Moderate
Initiation of cigarette smoking	Acceptable	Moderate
Progression to regular cigarette smoking	Acceptable	Moderate

CQ=contextual question; NA=not applicable (no data informing this outcome measure was identified in the review); SOE=strength of evidence

8 Supplemental Section 8: Literature Search Output, Studies Reviewed at the Full-Text Level (n=995)

RefID	Reference
41	Perry C.L.,Perez A.,Bluestein M.,Garza N.,Obinwa U.,Jackson C.,Clendennen S.L.,Loukas A.,Harrell M.B. Youth or Young Adults: Which Group Is at Highest Risk for Tobacco Use Onset?. Journal of Adolescent Health; 2018;:<br><br>**Keywords:**<br>adolescent,adult,adulthood,article,case report,*cigar,clinical article,cohort analysis,college,*electronic cigarette,female,follow up,*hookah,human,incidence,*juvenile,male,marketing,Texas,*tobacco use, young adult<br><br>**Abstract:** Purpose: Historically, adolescence has been regarded as the time when most tobacco use initiation occurs. This study examines the initiation of tobacco product use, including cigarettes, e-cigarettes, cigar products, and hookah, among contemporary youth and young adults, to determine whether the developmental timing (youth vs. young adulthood) of initiation has changed. Methods: Three cohort studies were used to examine the onset of ever use and current (past 30 days) use of each tobacco product among never-using youth (11 to <17 years) and young adults (18-24 years) at baseline (2013-2015) to one-year follow-up (2015-2016). These studies include the national Population Assessment of Tobacco and Health Study, and two Texas cohort studies, the Texas Adolescent Tobacco and Marketing Surveillance System (TATAMS), and the Marketing and Promotions Across Colleges in Texas (M-PACT) project. Estimations of onset were computed using generalized linear mixed models for TATAMS and M-PACT. The rates of initiation in Population Assessment of Tobacco and Health Study were compared to standardized incidence rates from TATAMS to M-PACT. Results: Young adults had significantly higher incidence rates than youth to initiate ever and current use of each/all tobacco products for all comparisons. Conclusions: These findings extend prior research on the timing of the onset of tobacco use by using longitudinal analyses from three contemporary cohort studies to include not just cigarettes, but also e-cigarettes, cigar products, and hookah. Among those who were never-users of tobacco products, young adults began to ever and currently use all tobacco products more than youth in these samples, a marked departure from prior decades of research.Copyright © 2018 Society for Adolescent Health and Medicine
58	Chaffee B.W.,Cheng J. Tobacco product initiation is correlated with cross-product changes in tobacco harm perception and susceptibility: Longitudinal analysis of the Population Assessment of Tobacco and Health youth cohort. Preventive Medicine; 2018;114:72.<br><br>**Keywords:**<br>adolescent,article,attitude,child,cigarette,cohort analysis,controlled study,correlational study,electronic cigarette,female,groups by age,hookah,human,longitudinal study,male,outcome assessment, *perception,priority journal,prospective study,risk factor,smokeless tobacco,*tobacco,United States, nicotine<br><br>**Abstract:** Youth initiation with one tobacco product is associated with risk of using additional tobacco types. How use of one product potentially encourages use of others could result from changing tobacco-related perceptions. This study aimed to evaluate how tobacco product initiation correlates with changes in susceptibility (curiosity and willingness) and perceived harm of other tobacco products. For each of cigarettes, e-cigarettes, hookah, and smokeless tobacco, youth (ages 12-16; N = 8005) rated perceived harm, curiosity, and willingness to try in Waves 1 and 2 of the Population Assessment of Tobacco and Health (United States, 2013-2015). For each product, we estimated associations between decreased harm rating, increased curiosity, or increased willingness with initiation (from never- to ever-use) of

	cigarettes, e-cigarettes, hookah, smokeless tobacco, or other combustible products using multivariate (multiple outcomes) regression, adjusting for other tobacco risk factors. Tobacco product initiation was associated with decreased perceived harm for that product and with decreased perceived harm, increased curiosity, and increased willingness in some, but not all, cross-product combinations. Most cross-product combinations of initiation and susceptibility yielded positive associations. For example, trying e-cigarettes was associated with concomitant increases in curiosity about cigarettes (OR: 5.69; 95% CI: 3.68, 8.79) and hookah (OR: 4.19; 95% CI: 2.55, 6.88) and with increased willingness to try cigarettes (OR: 9.61; 95% CI: 5.67, 16.3), hookah (OR: 8.46; 95% CI: 4.76, 15.0), and smokeless tobacco (OR: 3.48; 95% CI: 1.75, 6.94). New use of one tobacco product may catalyze subsequent use of others through cross-product changes in perceptions and susceptibility.Copyright © 2018 Elsevier Inc.
59	Chatterjee K.,Alzghoul B.,Innabi A.,Meena N. Is vaping a gateway to smoking: A review of the longitudinal studies. International Journal of Adolescent Medicine and Health; 2018;30:20160033.<br><br>**Keywords:**<br>adolescent,female,human,incidence,*longitudinal study,male,Medline,*psychology,review,Scopus, smoking cessation,systematic review,tobacco industry,*vaping,Web of Science,young adult<br><br>**Abstract:** Background: The use of e-cigarettes (ECs) is rising globally. There is concern that e-cigarette may actually lead to smoking, especially amongst adolescents. Objective: To perform a comprehensive review of literature reporting the longitudinal effects of e-cigarette use on onset of smoking among adolescents and young adults. Methods: A search was conducted using PubMed, Google Scholar, Scopus, and Web of Science in February 2016 to identify the studies containing data on EC use among adolescents and young adults (age < 30 years). We then narrowed our search to only include longitudinal studies with data on EC and conventional cigarette smoking among this population. Results: Four longitudinal studies were identified that analyzed the use of ECs and smoking at both baseline and follow-ups in the target population. These studies demonstrated that EC use is associated with an increase in combustible cigarette smoking, even amongst the adolescents who were not susceptible to smoking. Conclusion: This review highlights the strong evidence that not only are ECs are not an effective tool for smoking cessation among adolescents, they actually are associated with higher incidence of combustible cigarette smoking. Policy makers need to recognize of the insidious nature of this campaign by the tobacco industry and design policies to regulate it.Copyright © 2018 Walter de Gruyter GmbH, Berlin/Boston.
60	Kozlowski L.T.,Sweanor D.T. Young or adult users of multiple tobacco/nicotine products urgently need to be informed of meaningful differences in product risks. Addictive Behaviors; 2018;76:376.<br><br>**Keywords:**<br>cause of death,chronic obstructive lung disease,electronic cigarette,food and drug administration,health literacy,human,informed consent,longitudinal study,*medical information,Norway,note,observational study,*risk assessment,smokeless tobacco,*smoking,smoking regulation,tertiary prevention,United States,nicotine<br><br>**Abstract:** Previously, it has been argued that health information efforts need to inform the public about meaningful differential risks from tobacco/nicotine products. The fact of multiple product use by the same individual further supports this need. When the majority of youth, for example, who use smokeless tobacco are also current tobacco smokers, it makes little sense to mount a smokeless prevention campaign that fails to include clear messages about the much greater risks from smoking. In April 2016, The Food & Drug Administration (FDA) announced a million campaign for youth that "smokeless doesn't mean harmless." Research shows the public (a) already knows that smokeless tobacco is not harmless, but are (b) also largely unaware that cigarettes are much more harmful than smokeless. Though not harmless, smokeless tobacco has been estimated to be over 90% less harmful than cigarettes. 'Gateway' fears are made moot by current use of multiple tobacco/nicotine products. When multi-tobacco product

	use is commonplace among users, usable information on significant differences in risk is crucial for both adult and younger users. The FDA and like campaigns and health information websites should follow established ethical principles and accepted communication methods to inform the public of less-harmful tobacco/nicotine products as well as the greater harms of smoking, in keeping with the Surgeon-General's advice that reductions in smoking in particular will bring about the greatest public health advances.Copyright © 2017 The Authors
96	Fulton E.,Gokal K.,Griffiths S.,Wild S. More than half of adolescent E-Cigarette users had never smoked a cigarette: findings from a study of school children in the UK. Public Health; 2018;161:33.<br><br>**Keywords:**<br>adolescent,adolescent smoking,article,attitude to health,child,*cigarette smoking,cross-sectional study, electronic cigarette,England,female,*health belief,human,male,questionnaire,sex ratio,student,*student attitude,tobacco use,*vaping,nicotine<br><br>**Abstract:** Objectives: Electronic cigarettes (ECs) are known for their use as a smoking cessation aid; however, experimental use in adolescence is a growing international concern. The proportion of adolescent EC users who have never used tobacco is rising. EC use is associated with later tobacco initiation in young people. Understanding adolescent beliefs about ECs is needed to inform public health campaigns and school education regarding the EC and the associated risks. Study design: A cross-sectional questionnaire-based design was used. Methods: As part of a larger study, questionnaires to assess beliefs about ECs and current use were distributed to 499 school pupils aged 11-16 years in a county in England, UK. Results: More than half of EC users had never used tobacco (52.6%), a substantially greater proportion than previously reported in the literature. Adolescents were aware that ECs were less harmful than tobacco but many were unaware that they contain nicotine and the subsequent risk of addiction could lead to later tobacco use. Conclusions: Given the possible association of EC use and later smoking initiation, education in schools may warrant greater emphasis on ECs, the role of nicotine and the risk of addiction associated with experimentation. Young people who deem ECs as a 'safe' option, and may otherwise have never experimented with tobacco, could be at risk of later tobacco use.Copyright © 2018
97	Hall W.D. Regulating e-cigarettes in Australia: Implications for tobacco use by young people. Medical Journal of Australia; 2018;208:415.<br><br>**Keywords:**<br>*adolescent smoking,Australia,commercial phenomena,*electronic cigarette,human,letter,packaging, prevalence,smoking ban,*smoking regulation,tax,tobacco use,vaping<br><br>**Abstract:**
115	McKeganey N.,Barnard M. Change and continuity in vaping and smoking by young people: A qualitative case study of a friendship group. International Journal of Environmental Research and Public Health; 2018;15:1008.<br><br>**Keywords:**<br>adult,article,case report,clinical article,excitement,female,*friendship,human,male,plasticity,qualitative research,quantitative analysis,Scotland,*vaping<br><br>**Abstract:** This paper reports a qualitative case study of a small friendship group (n = 8) in Glasgow, Scotland. Interviewed twice at six months apart, these 16 to 17 year olds reported a substantial change in their use of and attitudes towards e-cigarettes and tobacco. At time 1, vaping generated much excitement and interest, with six out of eight individuals having their own vape device. At time 2, only two young people still vaped, with the others no longer professing any interest in continued vaping. The

	two regular smokers, who had been smoking before they first vaped, now only vaped privately and to reduce their tobacco intake. This small case study illustrates plasticity in the use of these devices; just as young people can move into their use, so too can they move away from them. This small study underscores the importance of differentiating between long-term, frequent, consistent use and more episodic, experimental and infrequent use by young people and for undertaking a measurement of actual e-cigarette use at multiple time points in both quantitative and qualitative studies. In addition, the case study illustrates the powerful impact which peers can have on teenagers use of e-cigarettes.Copyright © 2018 by the authors. Licensee MDPI, Basel, Switzerland.
129	Pierce J.P.,Sargent J.D.,Portnoy D.B.,White M.,Noble M.,Kealey S.,Borek N.,Carusi C.,Choi K.,Green V.R., Kaufman A.R.,Leas E.,Lewis M.J.,Margolis K.A.,Messer K.,Shi Y.,Silveira M.L.,Snyder K.,Stanton C.A.,Tanski S.E.,Bansal-Travers M.,Trinidad D.,Hyland A. Association between receptivity to tobacco advertising and progression to tobacco use in youth and young adults in the PATH study. JAMA Pediatrics; 2018;172:444.<br><br>**Keywords:**<br>adolescent,adolescent smoking,adult,*advertising,article,*behavior,child,*cigarette smoking,controlled study,electronic cigarette,female,follow up,human,*juvenile,longitudinal study,major clinical study,male, priority journal,smokeless tobacco,tobacco,vaping,young adult,*receptivity<br><br>**Abstract:** IMPORTANCE Cigarette marketing contributes to initiation of cigarette smoking among young people, which has led to restrictions on use of cigarette advertising. However, little is known about other tobacco advertising and progression to tobacco use in youth and young adults. OBJECTIVE To investigate whether receptivity to tobacco advertising among youth and young adults is associated with progression (being a susceptible never user or ever user) to use of the product advertised, as well as conventional cigarette smoking. DESIGN, SETTING, AND PARTICIPANTS The Population Assessment of Tobacco and Health (PATH) Study at wave 1 (2013-2014) and 1-year follow-up at wave 2 (2014-2015) was conducted in a US population-based sample of never tobacco users aged 12 to 24 years from wave 1 of the PATH Study (N = 10 989). Household interviews using audio computer-assisted self-interviews were conducted. EXPOSURES Advertising for conventional cigarettes, electronic cigarettes (e-cigarettes), cigars, and smokeless tobacco products at wave 1. MAIN OUTCOMES AND MEASURES Progression to susceptibility or ever tobacco use at 1-year follow-up in wave 2. RESULTS Of the 10 989 participants (5410 male [weighted percentage, 48.3%]; 5579 female [weighted percentage, 51.7%]), receptivity to any tobacco advertising at wave 1 was high for those aged 12 to 14 years (44.0%; 95%confidence limit [CL], 42.6%-45.4%) but highest for those aged 18 to 21 years (68.7%; 95%CL, 64.9%-72.2%). e-Cigarette advertising had the highest receptivity among all age groups. For those aged 12 to 17 years, susceptibility to use a product at wave 1 was significantly associated with product use at wave 2 for conventional cigarettes, e-cigarettes, cigars, and smokeless tobacco products. Among committed never users aged 12 to 17 years at wave 1, any receptivity was associated with progression toward use of the product at wave 2 (conventional cigarettes: adjusted odds ratio [AOR], 1.43; 95% CL, 1.23-1.65; e-cigarettes: AOR, 1.62; 95%CL, 1.41-1.85; cigars: AOR, 2.01; 95%CL, 1.62-2.49; and smokeless [males only]: AOR, 1.42; 95%CL, 1.07-1.89) and with use of the product (conventional cigarettes: AOR, 1.54; 95%CL, 1.03-2.32; e-cigarettes: AOR, 1.45; 95%CL, 1.19-1.75; cigars: AOR, 2.07; 95%CL, 1.26-3.40). Compared with those not receptive to any product advertising, receptivity to e-cigarette advertising, but not to cigarette advertising, was independently associated with those aged 12 to 21 years having used a cigarette at wave 2 (AOR, 1.60; 95%CL, 1.08-2.38). CONCLUSIONS AND RELEVANCE Receptivity to tobacco advertisingwas significantly associated with progression toward use in adolescents. Receptivity was highest for e-cigarette advertising and was associated with trying a cigarette.Copyright © 2018 American Medical Association. All rights reserved.
137	East K.,Hitchman S.C.,Bakolis I.,Williams S.,Cheeseman H.,Arnott D.,McNeill A. The Association Between Smoking and Electronic Cigarette Use in a Cohort of Young People. Journal of Adolescent Health; 2018;62:539.

	**Keywords:**<br>academic achievement,adolescent,adult,alcohol consumption,article,child,cohort analysis,*electronic cigarette,female,follow up,Great Britain,human,longitudinal study,major clinical study,male,priority journal,*smoking<br><br>**Abstract:** Purpose: Electronic cigarette (e-cigarette) use is associated with smoking initiation among young people; however, it is also possible that smoking is associated with e-cigarette initiation. This study explores these associations among young people in Great Britain. Methods: A longitudinal survey of 1, 152 11- to 18-year-olds was conducted with baseline in April 2016 and follow-up between August and October 2016. Logistic regression models and causal mediation analyses assessed whether (1) ever e-cigarette use and escalation were associated with smoking initiation (ever smoking at follow-up) among baseline never smokers (n = 923), and (2) ever smoking and escalation were associated with e-cigarette initiation (ever e-cigarette use at follow-up) among baseline never e-cigarette users (n = 1,020). Results: At baseline, 19.8% were ever smokers and 11.4% were ever e-cigarette users. Respondents who were ever e-cigarette users (vs. never users, 53% vs. 8%, odds ratio [OR] = 11.89, 95% confidence interval [CI] = 3.56-39.72) and escalated their e-cigarette use (vs. did not, 41% vs. 8%, OR = 7.89, 95% CI = 3.06-20.38) were more likely to initiate smoking. Respondents who were ever smokers (vs. never smokers, 32% vs. 4%, OR = 3.54, 95% CI = 1.68-7.45) and escalated their smoking (vs. did not, 34% vs. 6%, OR = 5.79, 95% CI = 2.55-13.15) were more likely to initiate e-cigarette use. There was a direct effect of ever e-cigarette use on smoking initiation (OR = 1.34, 95% CI = 1.05-1.72), and ever smoking on e-cigarette initiation (OR = 1.08, 95% CI = 1.01-1.17); e-cigarette and smoking escalation, respectively, did not mediate these effects. Conclusions: Among young people in Great Britain, ever e-cigarette use is associated with smoking initiation, and ever smoking is associated with e-cigarette initiation.Copyright © 2017 The Society for Adolescent Health and Medicine
139	Farsalinos K.,Tomaselli V.,Polosa R. Frequency of Use and Smoking Status of U.S. Adolescent E-Cigarette Users in 2015. American Journal of Preventive Medicine; 2018;54:814.<br><br>**Keywords:**<br>adolescent,*adolescent smoking,article,cigarette smoking,*disease course,*electronic cigarette,human, juvenile,smoking<br><br>**Abstract:** Introduction: E-cigarette use by adolescents is an important issue for public health. This study analyzed the frequency of e-cigarette use and its association with smoking status among U.S. adolescents. Methods: The National Youth Tobacco Survey 2015 was analyzed in 2017, focusing on frequency of past-30-day e-cigarette use according to smoking status of participants. Smoking status was classified as never smoker, ever/not a past-30-day smoker, and past-30-day smoker. Infrequent and frequent smoking and e-cigarette use was defined as use for <20 and >=20 of the past 30 days, respectively. Results: Past-30-day e-cigarette use was reported by 54.5% (95% CI=47.8%, 61.0%) of past-30-day smokers, 26.5% (95% CI=23.2%, 30.1%) of ever/not past-30-day smokers, and 4.6% (95% CI=4.0%, 5.2%) of never smokers (p<0.001). No past-30-day e-cigarette use was reported by 94.5% and frequent e-cigarette use by 0.3% of never smokers. Past-30-day e-cigarette use was reported by 50.4% (95% CI=43.6%, 57.3%) of infrequent and 64.7% (95% CI=54.4%, 73.8%) of frequent past-30-day smokers (p<0.001). E-cigarette use frequency correlated with both smoking status and tobacco cigarette consumption (rho=0.42, p<0.001 and rho=0.40, p<0.001, respectively). Past-30-day smokers were more likely to report past-30-day e-cigarette use (OR=11.42, 95% CI=9.06, 14.40) compared with adolescents reporting no past-30-day smoking. Conclusions: E-cigarette use is more prevalent among ever and past-30-day smoking adolescents compared with never smoking adolescents. Frequent e-cigarette use is rare among never smoking adolescents.Copyright © 2018 American Journal of Preventive Medicine
149	Aveyard P.,Arnott D.,Johnson K.C. Should we recommend e-cigarettes to help smokers quit?. BMJ (Online); 2018;361:k1759.

	**Keywords:**<br>advertising,article,cardiovascular risk,health care policy,health hazard,heart disease,human,juvenile, priority journal,*smoking cessation,tobacco industry,United Kingdom,*vaping<br><br>**Abstract:** Smokers want to vape, it can help them quit, and it's less harmful than smoking, say Paul Aveyard and Deborah Arnott. But Kenneth C Johnson argues that smokers who vape are generally less likely to quit and is concerned about youth vaping as a gateway to smoking, dual use, and potential harms from long term use.Copyright © Published by the BMJ Publishing Group Limited.
160	Farsalinos K.E.,Siakas G.,Poulas K.,Voudris V.,Merakou K.,Barbouni A. Electronic cigarette use in Greece: An analysis of a representative population sample in Attica prefecture. Harm Reduction Journal; 2018;15:20.<br><br>**Keywords:**<br>adult,age distribution,article,attitude to health,awareness,cross-sectional study,demography,educational status,*electronic cigarette,Greece,human,miscellaneous named groups,prevalence,sex difference, smoking,telephone interview,current smoker,former smoker,never smoker<br><br>**Abstract:** Background: The purpose was to assess prevalence and correlates of electronic cigarette (e-cigarette) use in Greece in 2017. Methods: A cross-sectional survey of a representative sample of 4058 adults living in Attica prefecture (35% of the Greek adult population) was performed in May 2017 through telephone interviews. Prevalence and frequency of e-cigarette use were assessed according to the smoking status, and logistic regression analysis was performed to identify correlates of use. Results: Current smoking was reported by 32.6% of participants. Ever e-cigarette use was reported by 54.1% (51.4-56.8%) of current smokers, 24.1% (21.7-26.5%) of former smokers and 6.5% (5.3-7.7%) of never smokers. Past experimentation was the most prevalent pattern of e-cigarette use among ever users (P<0.001). Almost 80% of ever and 90% of current e-cigarette users were using nicotine. Extrapolated to the whole Attica population (3.1 million), there were 1 million current smokers, 848,000 ever e-cigarette users and 155,000 current e-cigarette users. The majority of current e-cigarette users (62.2%) were former smokers. Only 0.2% of never smokers were current e-cigarette users. One out of 20 participants considered e-cigarettes a lot less harmful than smoking. Being current or former smoker were the strongest correlates current e-cigarette use (OR 30.82, 95% CI 10. 21-69.33 and OR 69.33, 95% CI 23.12-207.90 respectively). Conclusions: E-cigarette use in Greece is largely confined to current or former smokers, while current use and nicotine use by never smokers is extremely rare. The majority of current e-cigarette users were former smokers. Most participants overestimate the harmfulness of e-cigarettes relative to smoking.Copyright © 2018 The Author(s).
188	Chaffee B.W.,Watkins S.L.,Glantz S.A. Electronic cigarette use and progression from experimentation to established smoking. Pediatrics; 2018;141:e20173594.<br><br>**Keywords:**<br>adolescent,article,child,controlled study,correlation analysis,*electronic cigarette,female,follow up, human,male,outcome assessment,prediction,priority journal,risk factor,school child,*smoking<br><br>**Abstract:** BACKGROUND: It has been shown that never-smoking adolescents who try electronic cigarettes (e-cigarettes) are at increased risk of subsequent conventional cigarette smoking. We evaluated associations between e-cigarette use and progression to established smoking among adolescents who had already tried cigarettes. METHODS: Among participants (age 12.17 years) in the nationally representative Population Assessment of Tobacco and Health survey who had smoked a cigarette (>=1 puff) but not yet smoked 100 cigarettes (N = 1295), we examined 3 outcomes at 1-year follow-up as a function of baseline e-cigarette use: (1) having smoked >=100 cigarettes (established smoking), (2) smoking during the past 30 days, and (3) both having smoked >=100 cigarettes and past-30-day smoking (current established smoking). Survey-weighted multivariable logistic regression models were fitted to

	obtain odds ratios (ORs) and 95% confidence intervals (CIs) adjusted for smoking risk factors. RESULTS: Versus e-cigarette never use, having ever used e-cigarettes was positively associated with progression to established cigarette smoking (19.3% vs 9.7%), past-30-day smoking (38.8% vs 26.6%), and current established smoking (15.6% vs 7.1%). In adjusted models, e-cigarette ever use positively predicted current established smoking (OR: 1.80; 95% CI: 1.04.3.12) but did not reach statistical significance (alpha= .05) for established smoking (OR: 1.57; 95% CI: 0.99.2.49) and past-30-day smoking (OR: 1.32; 95% CI: 0.99.1.76). CONCLUSIONS: Among adolescent cigarette experimenters, using e-cigarettes was positively and independently associated with progression to current established smoking, suggesting that e-cigarettes do not divert from, and may encourage, cigarette smoking in this population.Copyright © 2018 by the American Academy of Pediatrics.
191	Rodriguez E.,Parron T.,Alarcon R. Perceptions and Use of the e-Cigarette Among University Students. Archivos de Bronconeumologia; 2017;53:650.<br><br>**Keywords:**<br>adult,article,awareness,correlational study,cross-sectional study,*electronic cigarette,female,human, male,*perception,physical activity,smoking habit,social status,Spain,undergraduate student,*university student,young adult<br><br>**Abstract:**
198	Treur J.L.,Rozema A.D.,Mathijssen J.J.P.,van Oers H.,Vink J.M. E-cigarette and waterpipe use in two adolescent cohorts: cross-sectional and longitudinal associations with conventional cigarette smoking. European Journal of Epidemiology; 2018;33:323.<br><br>**Keywords:**<br>adolescent,*adolescent smoking,adult,article,Aruba,child,*cigarette smoking,cohort analysis,comparative study,*electronic cigarette,female,groups by age,human,longitudinal study,major clinical study,male, Morocco,Netherlands,Netherlands Antilles,peer pressure,prevalence,school child,sex difference,smoking, smoking regulation,Suriname,tobacco,tobacco use,Turkey (republic),*waterpipe tobacco,young adult, nicotine<br><br>**Abstract:** Alternative tobacco products are increasing in popularity. An important question is whether their use is associated with or even leads to conventional smoking, but large-scale (European) studies are scarce. In two cohorts of Dutch adolescents (Cohort I n = 6819, mean age = 13.8 SD = 1.1, 48.2% female; Cohort II n = 2758, mean age = 17.3 SD = 1.8, 61.3% female), we investigated use of electronic (e)-cigarettes with nicotine, e-cigarettes without nicotine and waterpipe. Generalized estimating equation modelling was conducted with ever conventional smoking as the dependent variable (0 = no, 1 = yes) and ever alternative tobacco use as the independent variable, correcting for clustering within schools, age, sex and education in both cohorts. In a subsample (n = 2100), the association between alternative tobacco use at baseline and conventional smoking 6 months later was tested, taking into account smoking propensity (based on personality, susceptibility to peer pressure and smoking intentions). Ever use prevalence was 13.7% for e-cigarettes with nicotine, 29.4% for e-cigarettes without nicotine and 22.1% for waterpipe in Cohort I and 12.3, 27.6 and 45.3% respectively in Cohort II. Ever smokers had tried alternative tobacco products more often than never smokers. Among never-smoking adolescents at baseline, alternative tobacco use predicted ever smoking 6 months later (e-cigarettes with nicotine OR 11.90 95% CI 3.36-42.11; e-cigarettes without nicotine OR 5.36 95% CI 2.73-10.52; waterpipe OR 5.36 95% CI 2.78-10.31). This association was strongest for adolescents with a low baseline risk of smoking. Experimenting with alternative tobacco products is common among Dutch youth. Alternative tobacco use predicts (future) smoking, especially among adolescents with a low smoking propensity.Copyright © 2017, The Author(s).

204	Lee Y.O.,Pepper J.K.,Macmonegle A.J.,Nonnemaker J.M.,Duke J.C.,Porter L. Examining youth dual and polytobacco use with E-cigarettes. International Journal of Environmental Research and Public Health; 2018;15:699.<br><br>**Keywords:**<br>article,*cigarette smoking,*electronic cigarette,Florida,high school student,human,*juvenile,major clinical study,marketing,perception,public health,risk factor,thinking,*tobacco use<br><br>**Abstract:** E-cigarettes and other non-cigarette tobacco products are increasingly popular among youth. Little is known to inform public health efforts to reduce youth use. We examined psychosocial correlates of single and multiple tobacco product use among youth e-cigarette users. Data were from the 2014 Florida Youth Tobacco Survey (N = 69,923), a representative sample of Florida middle and high school students. Associations between combinations of e-cigarette, cigarette and other tobacco product (OTP) use and psychosocial variables were examined using multinomial logistic regression with an analytic sample of N = 2756. Most e-cigarette-using youth used at least one other product (81%). Perceiving cigarettes as easy to quit was significantly associated with greater likelihood of combined e-cigarette/OTP use (relative risk ratio (RRR) = 2.51, p < 0.001) and combined e-cigarette/cigarette/OTP use (RRR = 3.20, p < 0.0001). Thinking you will be smoking cigarettes in 5 years was associated with product combinations that include cigarettes. Tobacco company marketing receptivity was associated with multiple product user types. Given that specific psychosocial factors put youth at risk for concurrent use of e-cigarettes with tobacco products, public health efforts should address polytobacco use specifically, instead of individual product use. Youth perceptions about the ease of quitting cigarettes, intentions to continue smoking cigarettes and receptivity to tobacco company marketing are promising areas for messaging aimed at reducing e-cigarette polytobacco product use.Copyright © 2018 by the authors. Licensee MDPI, Basel, Switzerland.
241	Primack B.A.,Shensa A.,Sidani J.E.,Hoffman B.L.,Soneji S.,Sargent J.D.,Hoffman R.M.,Fine M.J. Initiation of Traditional Cigarette Smoking after Electronic Cigarette Use Among Tobacco-Naive US Young Adults. American Journal of Medicine; 2018;131:443.<br><br>**Keywords:**<br>adult,article,*cigarette smoking,cohort analysis,*electronic cigarette,environmental factor,female,follow up,health survey,human,longitudinal study,male,outcome assessment,priority journal,prospective study, United States,young adult<br><br>**Abstract:** Background: Although electronic cigarettes (e-cigarettes) may help some smokers quit, some young adult never-smokers are now using e-cigarettes recreationally, potentially increasing their risk for initiation of smoking. We aimed to determine the association between baseline e-cigarette use and subsequent initiation of cigarette smoking among initially never-smoking young adults. Methods: We conducted a prospective cohort study with assessments at baseline (March 2013) and follow-up (October 2014). We used sampling frames representing 97% of the US population to recruit a nationally representative sample of never-smoking young adults aged 18 to 30 years. The independent variable was baseline ever use of e-cigarettes. The main outcome measure was initiation of traditional cigarette smoking between baseline and 18-month follow-up. Results: Baseline surveys were completed by 1506 never-smoking young adults, of whom 915 (60.8%) completed follow-up. There were no demographic differences between responders and nonresponders. After applying survey weights-which accounted for both nonresponse and overcoverage or undercoverage-2.5% of the represented population of never-smokers (801,010 of 32,040,393) used e-cigarettes at baseline. Cigarette smoking was initiated by 47.7% of e-cigarette users and 10.2% of nonusers (P =.001). In fully adjusted multivariable models, e-cigarette use at baseline was independently associated with initiation of smoking at 18 months (adjusted odds ratio, 6.8; 95% confidence interval, 1.7-28.3). Results remained similar in magnitude and statistically significant in all sensitivity analyses. Conclusions: Baseline e-cigarette use was independently associated

	with initiation of traditional cigarette smoking at 18 months. This finding supports policy and educational interventions designed to decrease use of e-cigarettes among nonsmokers.Copyright © 2018
246	Lechner W.V.,Murphy C.M.,Colby S.M.,Janssen T.,Rogers M.L.,Jackson K.M. Cognitive risk factors of electronic and combustible cigarette use in adolescents. Addictive Behaviors; 2018;82:182.<br><br>**Keywords:**<br>*adolescent behavior,article,Caucasian,child,child behavior,*cigarette,*cigarette smoking,*cognition,*electronic cigarette,female,human,longitudinal study,male,middle school student,prediction,*risk factor,school child,smoking cessation,tobacco dependence,United States,*combustible cigarette<br><br>**Abstract:** Introduction: Cognitive susceptibility to cigarette smoking has been demonstrated to predict future cigarette initiation in adolescents. Examining this construct prior to tobacco product initiation may provide useful information on the differential risk of individuals initiating cigarette vs. e-cigarette products. Additionally, examining how susceptibility and tobacco product use relate to perceived harm cognitions will increase understanding of risk predisposition among adolescents. Method: Data were taken from a longitudinal study of middle school students (n = 1023; age = 12.1, 52.2% female, 72.1% white) in the Northeastern U.S. Likelihood of e-cigarette and cigarette ever-use in high school was examined as a function of a validated index of cigarette smoking susceptibility among tobacco naive students in middle school. Prospective associations between cognitive susceptibility to smoking and subsequent perceived harm of e-cigarettes (assessed in high school), and cross-sectional associations between concurrent tobacco product ever-use status and perceived harm of e-cigarettes were examined. Results: Adolescents classified as susceptible to cigarette smoking in middle school were more likely to initiate use of cigarettes (OR = 2.53) and e-cigarettes (OR = 1.95) as compared to adolescents classified as non-susceptible; cigarette smoking susceptibility did not differentially predict use of one product over the other. Adolescents endorsing e-cigarette use, reported significantly less perceived harm associated with e-cigarettes vs. cigarettes, while those who endorsed cigarette only or dual use did not. Conclusion: Our data indicate that cognitive susceptibility to cigarette smoking may index a broad risk factor for using either cigarettes or e-cigarettes in the future, and is prospectively associated with perceived harm of e-cigarette use. Overall, those who used any tobacco product perceived e-cigarettes as less harmful when compared to abstainers. Individual facets of perceived harm (addiction potential and harm vs. cigarettes) differ between cigarette only users and e-cigarette users and may help to explain the choice to use one product vs. the other. Implications: This is the first study to examine prospective associations between cognitive susceptibility to cigarette smoking, predating tobacco use, and subsequent likelihood of cigarette vs. e-cigarette initiation. This study demonstrates that initiation of either product is elevated among youth who are susceptible to smoking; thus susceptibility to smoking may serve as a useful marker of vulnerability to tobacco product use. Furthermore, this study provides novel information on the relationship between tobacco product onset and specific harm perceptions associated with e-cigarettes versus cigarettes among adolescents.Copyright © 2018 Elsevier Ltd
248	Jhanjee S.,Jain R.,Mandal P. E-cigarettes in adolescents: Issues and challenges. Indian Journal of Psychiatry; 2018;60:S43.<br><br>**Keywords:**<br>adolescent,aerosol,burn,cardiovascular system,cigarette smoking,combustion,*electronic cigarette,female,health hazard,human,juvenile,longitudinal study,male,nonhuman,smoking,surgeon,tobacco,cannabis,carbon monoxide,glycerol,nicotine,propylene glycol,conference abstract<br><br>**Abstract:** Electronic cigarettes(EC's) are devices that deliver nicotine to the user Vaporizing a solution that usually contains nicotine, propylene glycol and/or glycerol, plus favours. The number of EC's sold has increased exponentially since their introduction. The foremost reason for their growth is that they are perceived as a reduced harm alternative to traditional cigarettes as purportedly they do not burn tobacco and hence harmful products of combustion, such as tar and carbon monoxide are not generated.

	However many concerns regarding their use persist. Although e-cigarettes are free of combustion, they have different chemicals that could pose harm to health like propylene glycol, glycerine and added favors. E-cigarettes deliver nicotine by creating an aerosol of ultrafne particles which may pose significant health risks in terms of respiratory and cardiovascular problems Additionally in adolescent population of concern is the fact that a substantial proportion of adolescents who are not using cigarettes or other combustible tobacco products are using e-cigarettes. There is also a concern about "Gateway effects" or risk of progression to cigarette smoking amongst adolescents who use e-cigarettes. Recent longitudinal studies have established associations between e-cigarette use and later smoking initiation. A U.S Surgeon general report states that E-cigarettes are now the most commonly used Tobacco products among youth, surpassing conventional cigarettes in 2014. Flavoured EC products are used disproportionately by youth and added favors are a common reason for initiation. Of further concern is that now some adolescents are using e-cigarettes for making Smoke tricks, "dripping" and also use them to administer marijuana. Strong e-cigarette regulation could potentially curb use among youth and possibly limit the future population-level burden of cigarette smoking.
274	Akre C.,Barrense-Dias Y.,Berchtold A.,Suris J.-C. From tobacco-related products to smoking: Results from a longitudinal study. Journal of Adolescent Health; 2018;62:S7.<br><br>**Keywords:**<br>adolescent,*cigarette smoking,controlled study,disease course,electronic cigarette,female,gender, hookah,human,joint,juvenile,lifestyle,*longitudinal study,major clinical study,male,multivariate analysis, nonhuman,questionnaire,*smokeless tobacco,smoking cessation,snus,social status,student,Switzerland, tobacco,alcohol,cannabis,conference abstract<br><br>**Abstract:** Purpose: To assess longitudinal factors related to smoking among youths depending on smoking status at baseline. Methods: Data were drawn from the two first waves (T0 in 2016 and T1 in 2017) of the GenerationFRee longitudinal study conducted in eleven post-mandatory schools in the Canton of Fribourg, Switzerland. Participants (N = 1606, mean age 16.2 at baseline [15-24], 45.8% females) filled out a web-based self-administered anonymous questionnaire which aimed at assessing their lifestyle. Participants were asked at T1 whether they smoked (Group 1, N = 571) or not (Group 2, N = 1035). Groups were compared on the use of tobacco-related products (TRP) (e-cigarettes, hookah, snus, and smokeless tobacco) during the past 12 months, use of other substances (alcohol, canna-bis) and socio-demographic (age, gender, family structure, socioeconomic status, academic track [student/apprentice]) variables at T0 separately by smoking status at T0. Groups were compared at the bivariate level and afterwards at the multivariate level using a logistic regression analysis to assess the variables associated with being a smoker at T1 using non-smokers as the reference category. Data are presented as odds ratios (OR) with 95% confidence interval. Results: Smokers at T0. At the bivariate level, smokers at T1 were significantly more likely to be apprentices and showed a clear trend (p =.052) to be less likely to live in an intact family. At the multi-variate level, both variables remained significant: they were more likely to be apprentices (OR: 2.32 [1.28:4.22]) and to live in a non-intact family (OR: 1.75 [1.01:3.04]). Non-smokers at T0. At the bivariate level, those who became smokers at T1 were significantly more likely to have used TRPs (45.7% vs. 18.2%; p <.001), to have misused alcohol, and to have used cannabis at T0. In the multivariate analysis, having used TRPs (OR: 3.05 [1.92:4.85]) and cannabis (OR: 2.89 [1.41:5.90]) remained significant. Moreover, when the analysis were repeated separately by product, they remained significant (E-cigarettes: 2.46 [1.54:3.91]; Smokeless tobacco: 1.88 [1.12:3.15]; Snus: 2.05 [1.07:3.92]; Hookah: 2.68 [1.73:4.14]). Conclusions: These longitudinal results show an important association between using TRPs and starting to smoke and seem to confirm the gateway effect of products such as hookah, snus, smokeless tobacco, and electronic cigarettes. Therefore the potential harmful impact of these substances should not be underestimated. The association with cannabis is also an important result given that in Switzerland cannabis is mostly consumed in joints including tobacco. As an important percentage of non-smoking youths try different TRPs and they seem to be an entry point to cigarette consumption, TRPs should be part of substance use screening among

	adolescents. Sim-ilarly, tobacco prevention programs should be broadened to include tobacco-related products. Sources of Support: The Programme Intercantonal de Lutte contre la Dependance au Jeu (PILDJ) and the Canton of Fribourg.
283	Lea Watkins S.,Glantz S.A.,Chaffee B.W. Association of noncigarette tobacco product use with future cigarette smoking among youth in the population assessment of tobacco and health (PATH) study, 2013-2015. JAMA Pediatrics; 2018;172:181.<br><br>**Keywords:**<br>adolescent,*adolescent smoking,article,child,*cigarette smoking,cohort analysis,demography,electronic cigarette,environmental factor,female,follow up,high risk behavior,hookah,human,*juvenile,male, outcome assessment,*population,priority journal,prospective study,risk factor,smokeless tobacco, *tobacco use,United States<br><br>**Abstract:** IMPORTANCE Approximately 90% of adult smokers first tried a cigarette by 18 years of age, and even infrequent smoking in adolescence is associated with established adult smoking. Noncigarette tobacco use is increasing and could stimulate subsequent conventional cigarette smoking in youths. OBJECTIVE To estimate the longitudinal association between noncigarette tobacco use and subsequent cigarette smoking initiation among US youth. DESIGN, SETTING, AND PARTICIPANTS In this prospective cohort study of the Population Assessment of Tobacco and Health (PATH) waves 1 (September 12, 2013, to December 14, 2014) and 2 (October 23, 2014, to October 30, 2015), a nationally representative sample of youths who never smoked a conventional cigarette at baseline and completed wave 2 follow-up (N = 10 384) was studied. PATH retention at follow-up was 87.9%. EXPOSURES Ever use and past-30-day use of electronic cigarettes (e-cigarettes), hookah, noncigarette combustible tobacco, or smokeless tobacco at baseline. MAIN OUTCOMES AND MEASURES Ever use and past-30-day use of cigarettes at follow-up. RESULTS The present analysis was based on the 10 384 PATH youth respondents who reported never having smoked a cigarette in wave 1 and whose cigarette ever or past-30-day use was reported in wave 2 (mean [SD] age, 14.3 [1.7] years; age range, 12-17 years; 5087 [49.1%] female; 4829 [52.5%] white). At 1-year follow-up, 469 (4.6%) of all baseline never-smoking youths had tried a cigarette and 219 (2.1%) had smoked a cigarette within the past 30 days. Cigarette ever use at follow-up was higher among youths who had ever used e-cigarettes (78 [19.1%]), hookah (60 [18.3%]), noncigarette combustible tobacco (45 [19.2%]), or smokeless tobacco (29 [18.8%]) at baseline. After adjusting for sociodemographic, environmental, and behavioral smoking risk factors and for baseline ever use of other tobacco products, the odds of past-30-day cigarette use at follow-up were approximately twice as high among baseline ever users of e-cigarettes (odds ratio [OR], 1.87; 95% CI, 1.15-3.05), hookah (OR, 1.92; 95% CI, 1.17-3.17), noncigarette combustible tobacco (OR, 1.78; 95% CI, 1.00-3.19), and smokeless tobacco (OR, 2.07; 95% CI, 1.10-3.87). Youths who had tried more than 1 type of tobacco product at baseline had 3.81 (95% CI, 2.22-6.54) greater adjusted odds of past-30-day cigarette smoking at follow-up than did baseline never tobacco users. CONCLUSIONS AND RELEVANCE Any use of e-cigarettes, hookah, noncigarette combustible tobacco, or smokeless tobacco was independently associated with cigarette smoking 1 year later. Use of more than 1 product increased the odds of progressing to cigarette use.Copyright © 2018 American Medical Association. All rights reserved.
284	Young-Wolff K.C.,Klebaner D.,Folck B.,Tan A.S.L.,Fogelberg R.,Sarovar V.,Prochaska J.J. Documentation of e-cigarette use and associations with smoking from 2012 to 2015 in an integrated healthcare delivery system. Preventive Medicine; 2018;109:113.<br><br>**Keywords:**<br>adolescent,adult,age,aged,article,California,child,cohort analysis,comorbidity,controlled study,correlation analysis,*electronic cigarette,electronic medical record,ethnicity,female,gender,harm reduction,*health care delivery,human,incidence,male,*medical documentation,middle aged,prediction,priority journal, race,relapse,retrospective study,school child,*smoking,smoking cessation,*vaping,young adult

	**Abstract:** It is unclear whether use of electronic nicotine delivery systems (ENDS) precedes cigarette smoking initiation, relapse, and/or quitting. Healthcare systems with electronic health records (EHRs) provide unique data to examine ENDS use and changes in smoking. We examined the incidence of ENDS use (2012-2015) based on clinician documentation and tested whether EHR documented ENDS use is associated with twelve-month changes in patient smoking status using a matched retrospective cohort design. The sample was Kaiser Permanente Northern California (KPNC) patients aged >=12 with documented ENDS use (N = 7926); 57% were current smokers, 35% former smokers, and 8% never-smokers. ENDS documentation incidence peaked in 2014 for current and former smokers and in 2015 for never-smokers. We matched patients with documented ENDS use to KPNC patients without documented ENDS use (N = 7926) on age, sex, race/ethnicity, and smoking status. Documented ENDS use predicted the likelihood of smoking in the following year. Among current smokers, ENDS use was associated with greater odds of quitting smoking (OR = 1.17, 95% CI = 1.05-1.31). Among former smokers, ENDS use was associated with greater odds of smoking relapse (OR = 1.53, 95% CI = 1.22-1.92). Among never-smokers, ENDS use was associated with greater odds of initiating smoking (OR = 7.41, 95% CI = 3.14-17.5). The overall number of current smokers at 12 months was slightly higher among patients with (N = 3931) versus without (N = 3850) documented ENDS use. Results support both potential harm reduction of ENDS use (quitting combustibles among current smokers) and potential for harm (relapse to combustibles among former smokers, initiation for never-smokers).Copyright © 2018 Elsevier Inc.
286	Truman P.,Glover M.,Fraser T. An online survey of new zealand vapers. International Journal of Environmental Research and Public Health; 2018;15:222.<br><br>**Keywords:**<br>access to information,adult,aged,article,attitude to health,body weight gain,controlled study,electronic cigarette,European,exploratory research,female,*health survey,human,male,Maori (people),middle aged,*New Zealand,smoking,smoking cessation,social support,tobacco use,*vaping,nicotine<br><br>**Abstract:** Using electronic cigarettes (vaping) is controversial, but is increasingly widespread. This paper reports the results of an electronic survey of vapers in New Zealand, a country where the sale and supply of e-liquids containing nicotine is illegal, although vapers can legally access e-liquids from overseas. An on-line survey was conducted, using vaper and smoking cessation networks for recruitment, with follow up surveys conducted 1 and 2 months after the initial survey. 218 participants were recruited. Almost all had been smokers, but three quarters no longer smoked, with the remainder having significantly reduced their tobacco use. Three participants were non-smokers before starting to vape, but none had gone on to become smokers. The overriding motivation to begin and continue vaping was to stop or to reduce smoking. The results were consistent with a progression from initially both vaping and smoking using less effective electronic cigarette types, then moving to more powerful devices, experimentation with flavors and nicotine strengths-all resulting in reducing or stopping tobacco use. Lack of access to nicotine and lack of support for their chosen cessation method were the main problems reported. Vaping had resulted in effective smoking cessation for the majority of participants.Copyright © 2018 by the authors. Licensee MDPI, Basel, Switzerland.
291	Penzes M.,Foley K.L.,Nadasan V.,Paulik E.,Abram Z.,Urban R. Bidirectional associations of e-cigarette, conventional cigarette and waterpipe experimentation among adolescents: A cross-lagged model. Addictive Behaviors; 2018;80:59.<br><br>**Keywords:**<br>adolescent,article,cohort analysis,*electronic cigarette,female,*follow up,gender,human,human experiment,male,market,questionnaire,structural equation modeling,*tobacco use<br><br>**Abstract:** Purpose: With an increasingly diverse tobacco product market, it is imperative to understand the trajectories of product experimentation in order to design effective prevention programs. This study aims to explore the bidirectional associations of conventional cigarette, e-cigarette and waterpipe

	experimentation in a large adolescent sample. Methods: Longitudinal assessment of conventional cigarette, e-cigarette and waterpipe use initiation was conducted in a school-based cohort of 1369 9th graders (mean age = 14.88 SD = 0.48 at baseline) during fall 2014 and reassessed 6-months later using online self-reported questionnaires. Autoregressive cross-lagged analysis within structural equation modeling framework was performed to simultaneously estimate the initiation of these products over a six-month period, controlling for age, gender, and participation in an intervention program to reduce conventional cigarette initiation. Results: Tobacco product lifetime use was prevalent at baseline in the sample: conventional cigarettes (48.4%), e-cigarettes (35.8%), and waterpipe (20.8%). At six-month follow-up, trying conventional cigarettes predicted trying e-cigarette (adjusted odds ratio (AOR) = 3.78, CI95%: 2.66-5.37) and trying waterpipe (AOR = 2.82, CI95%: 2.00-3.97). Trying e-cigarette predicted trying conventional cigarette (AOR = 3.57, CI95%: 1.96-6.49) and trying waterpipe (AOR = 1.51, CI95%: 1.07-2.14). Although trying waterpipe predicted trying e-cigarette at follow-up (AOR = 2.10, CI95%: 1.30-3.40), its use did not predict trying conventional cigarette (AOR = 0.55, CI95%: 0.24-1.30). Conclusions: The high rates of poly-tobacco use and the bidirectionality of tobacco product experimentation demands comprehensive tobacco control and prevention programs that address the increasingly diverse tobacco product market targeting adolescents.Copyright © 2018 Elsevier Ltd
303	King A.C.,Smith L.J.,McNamara P.J.,Cao D. Second generation electronic nicotine delivery system vape pen exposure generalizes as a smoking cue. Nicotine and Tobacco Research; 2018;20:246.<br><br>**Keywords:**<br>adult,article,Caucasian,combustion,comparative study,controlled study,female,human,latent period, major clinical study,male,prevalence,priority journal,smoking,smoking cessation,*vaping,young adult, nicotine,vaporizer,*electronic cigarette<br><br>**Abstract:** Introduction: Second generation electronic nicotine delivery systems (ENDS; also known as e-cigarettes, vaporizers or vape pens) are designed for a customized nicotine delivery experience and have less resemblance to regular cigarettes than first generation "cigalikes." The present study examined whether they generalize as a conditioned cue and evoke smoking urges or behavior in persons exposed to their use. Methods: Data were analyzed in N = 108 young adult smokers (>=5 cigarettes per week) randomized to either a traditional combustible cigarette smoking cue or a second generation ENDS vaping cue in a controlled laboratory setting. Cigarette and e-cigarette urge and desire were assessed pre- and post-cue exposure. Smoking behavior was also explored in a subsample undergoing a smoking latency phase after cue exposure (N = 26). Results: The ENDS vape pen cue evoked both urge and desire for a regular cigarette to a similar extent as that produced by the combustible cigarette cue. Both cues produced similar time to initiate smoking during the smoking latency phase. The ENDS vape pen cue elicited smoking urge and desire regardless of ENDS use history, that is, across ENDS naive, lifetime or current users. Inclusion of past ENDS or cigarette use as covariates did not significantly alter the results. Conclusions: These findings demonstrate that observation of vape pen ENDS use generalizes as a conditioned cue to produce smoking urge, desire, and behavior in young adult smokers. As the popularity of these devices may eventually overtake those of first generation ENDS cigalikes, exposure effects will be of increasing importance. Implications: This study shows that passive exposure to a second generation ENDS vape pen cue evoked smoking urge, desire, and behavior across a range of daily and non-daily young adult smokers. Smoking urge and desire increases after vape pen exposure were similar to those produced by exposure to a first generation ENDS cigalike and a combustible cigarette, a known potent cue. Given the increasing popularity of ENDS tank system products, passive exposures to these devices will no doubt increase, and may contribute to tobacco use in young adult smokers.Copyright © The Author(s) 2016. Published by Oxford University Press on behalf of the Society for Research on Nicotine and Tobacco. All rights reserved.
316	Owotomo O.,Maslowsky J.,Loukas A. Perceptions of the Harm and Addictiveness of Conventional Cigarette Smoking Among Adolescent E-Cigarette Users. Journal of Adolescent Health; 2018;62:87.

	**Keywords:** adolescent,*adolescent smoking,advertising,article,attitude,controlled study,female,human,human experiment,male,peer group,*perception,priority journal,risk factor,*tobacco dependence,vulnerable population,*electronic cigarette<br><br>**Abstract:** Purpose Although existing evidence indicates that e-cigarette use is a risk factor for cigarette smoking initiation, mechanisms of this association are not yet known. E-cigarette users perceive e-cigarette use to be less harmful relative to conventional cigarettes, but their absolute perceptions of addictiveness of conventional cigarette smoking are unknown. This study examines how e-cigarette users compare with nonusers (non-e-cigarette users/nonconventional cigarette smokers), conventional cigarette smokers, and dual users on perceptions of harm and the addictiveness of conventional cigarette smoking and on other known predictors of cigarette smoking such as peer smoking, influence of antismoking ads, and risk-taking propensity. Methods National samples of 8th- and 10th-grade students from 2014 and 2015 (N = 14,151) were obtained from the Monitoring the Future Study. Multivariate logistic regression models were used to examine relationships between adolescent smoking status and perceptions of harm and the addictiveness of conventional cigarette smoking while controlling for potential confounders. Results E-cigarette users had lower perceptions of the addictiveness of conventional cigarette smoking compared with nonusers but higher than cigarette smokers and dual users. E-cigarette users reported lower influence by antismoking ads, more conventional cigarette-smoking peers, and greater risk-taking propensity than nonusers. E-cigarette users and cigarette smokers did not differ in their perceived harm of conventional cigarette smoking or in their risk-taking propensity. Conclusions E-cigarette users' attitudes and perceptions regarding conventional cigarette smoking may leave them vulnerable to becoming conventional cigarette smokers. Future studies should explore the prospective relationship between smoking-related perceptions of conventional cigarette smoking among e-cigarette users and the onset of cigarette smoking.Copyright © 2017 The Society for Adolescent Health and Medicine
320	Bold K.W.,Kong G.,Camenga D.R.,Simon P.,Cavallo D.A.,Morean M.E.,Krishnan-Sarin S. Trajectories of E-cigarette and conventional cigarette use among youth. Pediatrics; 2018;141:e20171832.<br><br>**Keywords:** adolescent,age,article,Connecticut,controlled study,correlation analysis,ethnicity,female,gender,high school student,human,longitudinal study,male,prediction,priority journal,*smoking,social status,tobacco use,*vaping<br><br>**Abstract:** Background: Electronic cigarette (e-cigarette) use is common among youth, and there are concerns that e-cigarette use leads to future conventional cigarette use. We examined longitudinal associations between past-month cigarette and e-cigarette use to characterize the stability and directionality of these tobacco use trajectories over time. Methods: High school students (N = 808, 53% female) completed surveys across 3 waves (2013, 2014, and 2015) in 3 public schools in Connecticut. Using autoregressive crosslagged models, we examined bidirectional relationships between past-month cigarette and e-cigarette use over time. Models were adjusted for covariates related to tobacco use (ie, sex, race/ethnicity, socioeconomic status, and use of other tobacco products). Results: Past-month e-cigarette use predicted future cigarette use (wave 1-2: odds ratio [OR] = 7.08, 95% confidence interval [CI] = 2.34-21.42; wave 2-3: OR = 3.87, 95% CI = 1.86-8.06). However, past-month cigarette use did not predict future e-cigarette use (wave 1-2: OR = 2.02, 95% CI = 0.67-6.08; wave 2-3: OR = 1.90, 95% CI = 0.77-4.71). Additionally, frequency of cigarette and e-cigarette use increased over time. By wave 3, 26% of cigarette users and 20.5% of e-cigarette users reported using 21-30 days out of the past month. Conclusions: E-cigarette use was associated with future cigarette use across 3 longitudinal waves, yet cigarette use was not associated with future e-cigarette use. Future research needs to examine mechanisms through which e-cigarette use leads to cigarette use. E-cigarette regulation and prevention

	programs may help prevent future use of cigarettes among youth.Copyright © 2018 by the American Academy of Pediatrics. All rights reserved.
324	Anonymous. Study Links Vaping to Tobacco Use in Teens. Journal of the California Dental Association; 2016;44:539.<br><br>**Keywords:**<br>adolescent,*epidemiology,human,tobacco use,vaping<br><br>**Abstract:**
367	Barrington-Trimis J.L.,Leventhal A.M.,Alonzo T.A.,Cruz T.B.,Urman R.,Liu F.,Pentz M.A.,Unger J.B., McConnell R. Performance of cigarette susceptibility index among e-cigarette and hookah users. Drug and Alcohol Dependence; 2018;183:43.<br><br>**Keywords:**<br>adolescent smoking,adult,article,cross-sectional study,female,human,male,predictive value,priority journal,prospective study,sensitivity and specificity,*smoking,*smoking and smoking related phenomena, smoking habit,social environment,tobacco,United States,*electronic cigarette,*cigarette susceptibility index,*hookah<br><br>**Abstract:** Objective Susceptibility to cigarette smoking has been used since the mid-1990s as a measure to identify youth at risk of cigarette initiation. However, it is unclear how well this measure predicts future smoking among electronic (e-)cigarette or hookah users, or among those in tobacco-friendly social environments. Methods We used prospective data from the Southern California Children's Health Study to evaluate the performance (sensitivity, specificity, predictive value) of a composite index assessing susceptibility to smoking, and to evaluate whether performance of the measure differed by use of e-cigarettes or hookah, or immersion in a tobacco-friendly social environment. Susceptibility to cigarette smoking was measured in 11th/12th grade (2014) among never cigarette-smokers (N = 1266); follow-up data on smoking initiation were obtained approximately 16 months later. Results Overall, 16.4% of youth initiated smoking between baseline and follow-up. The sensitivity of the susceptibility to smoking index was low (46.4%), and specificity was high (79.0%). No difference in sensitivity was observed by baseline e-cigarette use; specificity was higher among never e- cigarette users. Differences in negative predictive value (NPV) and positive predictive value (PPV) were also observed by baseline e-cigarette and hookah use. Specificity was generally lower, and sensitivity was generally higher for those in tobacco-friendly social environments. Conclusions Our findings suggest that use of the susceptibility to smoking index in cross-sectional studies of older adolescents to identify those likely to begin smoking may be inappropriate for those using alternative tobacco products (e.g., e-cigarettes or hookah).Copyright © 2017 Elsevier B.V.
370	Pokhrel P.,Lam T.H.,Pagano I.,Kawamoto C.T.,Herzog T.A. Young adult e-cigarette use outcome expectancies: Validity of a revised scale and a short scale. Addictive Behaviors; 2018;78:193.<br><br>**Keywords:**<br>adult,article,*expectancy,factor analysis,female,health survey,human,male,outcome assessment,passive smoking,qualitative research,rating scale,*social behavior,*tobacco dependence,validity,young adult, nicotine,*electronic cigarette<br><br>**Abstract:** The revised youth e-cigarette outcome expectancies measure adds new items informed by recent qualitative research with young adult e-cigarette users, especially in the domain of positive "smoking" experience. Positive "smoking" experience represents beliefs that use of e-cigarettes provides outcomes associated with a better "smoking" alternative: for example, an alternative that is more socially approved, more suitable for indoor use, and that provides a safer means of enjoying nicotine. In addition,

	we tested a short, 8-item version of the measure which may be more easily incorporated into surveys. We tested the validity of the revised measure, both long and short versions, in terms of factor structure and associations of the expectancy factors with current e-cigarette use, e-cigarette use susceptibility, and e-cigarette use dependence. Participants were young adults (N = 470; 65% women; mean age = 20.9, SD = 2.1). Results replicated the findings of the previous study as well as highlighted the importance of the added domain of positive "smoking" experience and the validity of the short scale. Furthermore, results showed that positive outcome expectancies are strongly associated with e-cigarette use dependence. The long and short versions of the revised youth e-cigarette outcome expectancies scale appear to be valid and useful for application not only among cigarette smokers and e-cigarette users but also among never smokers and never e-cigarette users.Copyright © 2017 Elsevier Ltd
387	Sears C.G.,Walker K.L.,Hart J.L.,Lee A.S.,Siu A.,Smith C.,Bhatnagar A.,Robertson R.M. Perceptions and use of electronic cigarettes among middle and high school students in appalachia. Circulation; 2016;134:<br><br>**Keywords:**<br>advertising,child,*electronic cigarette,female,friend,gender,health hazard,*high school student,human, juvenile,Kentucky,major clinical study,male,New York,North Carolina,*perception,school child,smokeless tobacco,statistics,tobacco consumption<br><br>**Abstract:** Introduction: Across the US, youth use of electronic cigarettes (e-cigarettes) has increased. Recent studies suggest that e-cigarette use is associated with increased intention to smoke traditional cigarettes. In regions, like Appalachia, where youth use of traditional tobacco products is among the most prevalent in the United States, even the slightest increase in acceptance of tobacco products could counteract recent anti-tobacco efforts aimed at reducing cultural acceptance of tobacco in the region. The aim of this study is to compare e-cigarette users' and nonusers' tobacco consumption as well as perceptions of e-cigarette advertising and health effects. Methods: During the 2014-2015 school year, a cross-sectional survey (n=872) about students' (age 11-19 years) perceptions and use of tobacco products was conducted in three Appalachian states: Kentucky, North Carolina, and New York. Descriptive statistics and logistic regression were used to compare tobacco use and perceptions among e-cigarette users and nonusers. Results: About 20% of youth reported trying e-cigarettes. Controlling for age and gender, students who reported traditional cigarette use were 17 times more likely than non-users to also report trying e-cigarettes (95% CI= 10, 32). E-cigarette users were more likely to use smokeless tobacco (p<0.0001), report feeling targeted by e-cigarette ads (p=0.0004), and obtain information about e-cigarettes from friends/family (p<0.0001) and digital media (p=0.004). Students who had not tried e-cigarettes were more likely to consider e-cigarettes tobacco products (p=0.001) and to believe that e-cigarettes cause health problems (p<0.0001). Conclusions: In this study, e-cigarette use was associated with consumption of other tobacco products. Communication channels to obtain information about e-cigarettes varied between users and nonusers, which may shape perceived health risks. Understanding youth perceptions of e-cigarettes is vital to developing successful anti-tobacco and anti-e-cigarette campaigns.
396	Schneider S.,Gorig T.,Schilling L.,Diehl K. E-Cigarettes on Everyone's Lips - Current Representative Data on Use among Adolescents and Adults. Deutsche Medizinische Wochenschrift; 2017;142:e156.<br><br>**Keywords:**<br>adolescent,adult,age,article,controlled study,educational status,gender,human,migrant,*smoking, telephone interview,tobacco use,nicotine,*electronic cigarette<br><br>**Abstract:** Background International data suggest an increase in e-cigarette use. However, there are still significant deficits in the reporting of its current prevalence. The present study reports representative data on e-cigarette use that make it possible to differentiate users both by the nicotine content of the e-cigarette liquids being used and by their smoking status. Methods and material The underlying data are taken from the National Cancer Aid Monitoring (NCAM). In its first wave, 3000 persons aged between 14

	and 45 were interviewed by telephone. Point estimates, confidence intervals and determinants of use will be presented. Results Overall, 20% of respondents had used an e-cigarette before, of which 80% used liquids containing nicotine. Men, younger persons, persons with a migrant background and persons with a lower education level were more likely to have used an e-cigarette before than their respective reference groups. Half of all ever users of e-cigarettes also smoked tobacco cigarettes, while roughly one third had never used tobacco products before. Conclusions For the first time, a representative study was conducted combining questions on the use of e-cigarettes with questions on smoking tobacco cigarettes and the nicotine content of the liquids being used. Target groups for future prevention measures are adolescents, as they had the highest prevalence of e-cigarette use, as well as non-smokers, since one third of e-cigarette users had never used a tobacco product before. Both groups also seem to be relevant target groups for which the introduction to e-cigarettes should be prevented.
398	Afolalu E.F.,Prieto L.,De La Bourdonnaye G.,Sponsiello-Wang Z.,Weitkunat R. Measuring changes in patterns of tobacco product use over time: Transition probability approaches. Value in Health; 2017;20:A778.<br><br>**Keywords:**<br>adult,burn,*electronic cigarette,harm reduction,heat,human,outcome assessment,*probability,smoking, tobacco consumption,nicotine<br><br>**Abstract:** Measuring patterns of tobacco use has typically involved assessing number of units and frequency of use of cigarettes. With the emergence of new types of products (e.g. e-cigarettes, water-pipes, heat-not-burn products), use of more than one tobacco product is increasing in popularity, and therefore quantifying the overall tobacco consumption of individuals is becoming more challenging. Novel products are different in design and consumption to cigarettes and their availability results in multiple and complex combinations that make the measurement of exposure to tobacco and nicotine containing products intricate. Moreover, these complex patterns of use present a challenge when assessing changes over time and evaluating their subsequent impact on health outcomes. Changes in patterns of tobacco product use over time (i.e. moving between different tobacco use status and combinations, the possibilities of single, dual or poly product use, progression from occasional to regular use, initiation, cessation, switching, and re-initiation between products) can be characterized by estimating the probabilities of transition between one tobacco-use state and another. This contribution presents an overview of analytical approaches to assess transitions in complex patterns of tobacco use. It addresses the practical implications of applying these analytical approaches across existing epidemiological surveys and datasets to characterize patterns and determine probabilities of changes in tobacco use status. In addition, it considers the utility of transition probability methods to provide a snapshot into changes in the patterns of tobacco use and how this can be used to inform future tobacco use trajectories, associated health outcomes, and tobacco harm reduction efforts.
406	Collins L.K.,Villanti A.C.,Pearson J.L.,Glasser A.M.,Johnson A.L.,Niaura R.S.,Abrams D.B. Frequency of youth e-cigarette, tobacco, and poly-use in the United States, 2015: Update to Villanti et al., "frequency of youth e-cigarette and tobacco use patterns in the United States: Measurement precision is critical to inform public health". Nicotine and Tobacco Research; 2017;19:1253.<br><br>**Keywords:**<br>human,*juvenile,letter,prevalence,priority journal,*public health,*smoking,United States,*vaping<br><br>**Abstract:**
417	Segura L.E.,Maldonado A.,Santaella J.,Storr C.,Martins S.S. Greater curiosity about smoking cigarettes among 6th and 12th grade students using alternative tobacco-smoking products. Drug and Alcohol Dependence; 2017;171:e186.

	**Keywords:**<br>advertising,Asian,burn,Caucasian,controlled study,drug therapy,electronic cigarette,ethnicity,female, gender,Hispanic,human,juvenile,major clinical study,male,prevalence,prevention,*smokeless tobacco, *smoking habit,*student,television,tobacco,adenosine triphosphate<br><br>**Abstract:** Aims: Weestimated the likelihood of endorsing a higher curiosity about trying cigarette smoking by adolescents, who were past-month ATP users and cigarette-naive. Methods: We used data from the 2014 U.S. National Youth Tobacco Survey. Our analyses were limited to students' aged 9-19 that never used or experimented with cigarettes but that could have used ATP (n = 15,543).Weused weighted ordinal logistic regression to estimate the likelihood of: (1) endorsing great curiosity to smoke cigarettes among past month users of ATP (e-cigarette, hookah, pipe, smokeless tobacco products, and cigars), and (2) endorsing great curiosity to smoke cigarettes among current users of ATP that require or do not require burning tobacco. All analyses were adjusted for gender, ethnicity, and exposure to cigarette advertisement. Results: The prevalence of past-month use of any tobacco product by youth was 10.65%. Among naive cigarette youth, those using e-cigarettes (3%) endorsed greater curiosity about trying cigarettes in the future compared to non-users (97%) (aOR: 2.57, 95% CI: 1.96-3.31). Users of other ATP had similar curiosity levels compared to non-users. Users of nontobacco-burning products (i.e., smokeless tobacco) reported greater curiosity about trying regular cigarettes (aOR: 1.94, 95% CI: 1.54-2.44). Hispanic and Asian students (vs. Whites), those exposed to cigarette advertisement in television and store/supermarkets/gas stations (aOR: 1.36, 95% CI: 1.24-1.49; aOR: 1.43, 95% CI: 1.17-1.73; aOR: 1.08, 95% CI: 1.04-1.12; aOR: 1.10, 95% CI: 1.04-1.16; aOR; respectively) were more likely to show greater curiosity about trying cigarettes. Conclusions: Our findings show that use of ATP products (particularly nontobacco burning products) may increase youth's curiosity about trying cigarettesand could be a gateway to future cigarette smoking. Smoking prevention resources provided to youth already experimenting with ATP could reduce their risk of future smoking habits.
451	Shaddock E. Electronic cigarette and tobacco product use in adolescents in Johannesburg-results from a survey. American Journal of Respiratory and Critical Care Medicine; 2017;195:<br><br>**Keywords:**<br>adolescent,child,controlled study,*electronic cigarette,female,high risk behavior,high school,human, major clinical study,male,questionnaire,sample size,smoking,South Africa,student<br><br>**Abstract:** INTRO: Individuals who start smoking during adolescence are more likely to continue smoking into adulthood. Electronic cigarettes have been shown to encourage progression onto conventional tobacco product smoking. There is currently no data available in South Africa with regards to e-cigarette use amongst school going adolescents METHODS: Questionnaire administered to 14-18 year olds in two different income schools. RESULTS: 229 students were recruited, 79% of which were female. 11 % of total admitted to smoking cigarettes. The median age at which students started smoking was 15 years (range 12-17 years). 23% had tried e-cigarettes, 9% of whom currently used. 25% of students of the lower income school had tried e-cigarettes compared to 20% of the higher income school (p=0.43). 25% of the grade 9 (14-16 year olds) had tried e-cigarettes compared to 20% of grade 11 (16-18 year olds) (p=0.87). 38% of students had tried hookah, 50% from the lower income school versus 17% from the higher income (p=0.0001). LIMITATIONS: The small sample size is an important limitation. This predominant female population occurred due to schools sampled and could introduce bias in view of the more risky behaviour usually favoured by school boys. CONCLUSION: Experimentation with tobacco products is common in secondary school goers, especially in those from the lower income school. Electronic cigarette use is as common as conventional tobacco use.
487	Mayor S. Smoking prevalence falls among adults in England. BMJ (Online); 2017;357:j2953.<br><br>**Keywords:**<br>electronic cigarette,England,health behavior,high school,human,maternal smoking,national health

	service,note,pregnant woman,*prevalence,priority journal,school health service,sex difference,*smoking cessation,student attitude<br><br>**Abstract:**
489	Dai H.,Hao J. Flavored electronic cigarette use and smoking among youth. Pediatrics; 2016;138:e20162513.<br><br>**Keywords:**<br>adolescent,*adolescent smoking,article,attitude to health,controlled study,cross-sectional study, electronic cigarette,female,health hazard,health survey,human,juvenile,male,prevalence,priority journal, risk factor,smoking cessation,smoking habit,*vaping<br><br>**Abstract:** BACKGROUND AND OBJECTIVE: Flavored electronic cigarettes (e-cigarettes) are not prohibited in the United States, and e-cigarette flavors proliferate on the market. This study sought to examine flavored e-cigarette use and its association with smoking among youth. METHODS: Estimates of flavored e-cigarette use from the 2014 National Youth Tobacco Survey were investigated. A logistic regression model was used to assess whether flavored e-cigarette use was associated with (1) intention to initiate cigarette use among neversmoking youth (n = 16 471), (2) intention to quit tobacco use among current-smoking youth (n = 1338), and (3) perception of tobacco's danger among all respondents (n = 21 491). RESULTS: A total of 2017 respondents reported using e-cigarettes in the last 30 days, of whom 1228 (60.9%) reported using flavored e-cigarettes. Among never-smoking youth, 55.6% (288) of current e-cigarette users reported using flavored e-cigarettes. Among current smokers, 68.4% (495) of current e-cigarette users reported using flavored e-cigarettes. Compared with not using e-cigarettes in the past 30 days, using flavored e-cigarettes was associated with higher odds of intention to initiate cigarette use among neversmoking youth (adjusted odds ratio [aOR] = 5.7; P <.0001), lower odds of intention to quit tobacco use among current-smoking youth (aOR = 0.6; P =.006), and a lower prevalence of perception of tobacco's danger among all respondents (aOR = 0.5; P <.0001). CONCLUSIONS: Flavored e-cigarette use is associated with increased risks of smoking among youth. Comprehensive tobacco control and prevention strategies that address flavored e-cigarette products are critically needed to reduce tobacco use among youth.Copyright © 2016 by the American Academy of Pediatrics.
495	Printz C. Researchers call teen vaping "one-way bridge" to smoking. Cancer; 2017;123:2188.<br><br>**Keywords:**<br>adolescent,adolescent behavior,*adolescent smoking,educational status,electronic cigarette,ethnic difference,follow up,health hazard,health survey,high risk behavior,high risk population,human,note, prevalence,priority journal,sex difference,*vaping<br><br>**Abstract:**
500	Wills T.A.,Sargent J.D. Do E-cigarettes reduce smoking?. Preventive Medicine; 2017;100:285.<br><br>**Keywords:**<br>*electronic cigarette,health care policy,human,law,motivation,note,prediction,*preventive medicine, priority journal,propensity score,psychological aspect,public health,*smoking,smoking cessation,social aspect,social environment,tobacco dependence<br><br>**Abstract:**
505	Eastwood B.,East K.,Brose L.S.,Dockrell M.J.,Arnott D.,Cheeseman H.,McNeill A. Electronic cigarette use in young people in Great Britain 2015-2016. Public Health; 2017;149:45.<br><br>**Keywords:**

	adolescent,adult,article,awareness,child,controlled study,*electronic cigarette,Great Britain,health behavior,human,perception,prevalence,*smoking,young adult<br><br>**Abstract:**
511	Bover Manderski M.T.,Giovenco D.P.,Delnevo C.D. The importance of contextual and temporal accuracy when studying novel tobacco products. Pediatrics; 2017;139:e20170455A.<br><br>**Keywords:**<br>*adolescent smoking,high school student,human,letter,middle school student,prevalence,priority journal, time series analysis,tobacco use,United States,*vaping<br><br>**Abstract:**
513	Clancy L.,Babineau K. E-cigarettes: Effective cessation tools or public health threat?. QJM; 2016;109:79.<br><br>**Keywords:**<br>airway resistance,biosafety,clinical effectiveness,clinical pharmacology,conceptual framework,control, *electronic cigarette,epidemiological data,forced expiratory volume,harm reduction,health care policy, human,lung function,nicotine replacement therapy,nonhuman,note,particle size,prevalence,priority journal,*public health,randomized controlled trial (topic),smoking,*smoking cessation,social marketing, *threat,tobacco,tobacco industry,toxicity testing,world health organization,glycerol,nicotine,nicotine patch,propylene glycol<br><br>**Abstract:**
517	Schneider S.,Diehl K. Vaping as a Catalyst for Smoking? An InitialModel on the Initiation of Electronic CigaretteUse and the Transition to Tobacco SmokingAmong Adolescents. Nicotine and Tobacco Research; 2016;18:647.<br><br>**Keywords:**<br>adolescent,*adolescent smoking,cost,*electronic cigarette,fear,human,hypothesis,legal liability,medical research,peer acceptance,peer pressure,personal experience,priority journal,process model,review,risk assessment,risk factor,smoking cessation,taste acuity,tobacco dependence,acceptance hypothesis, accessibility hypothesis,concealment hypothesis,experience hypothesis,flavor hypothesis,liability hypothesis,price hypothesis,renormalization hypothesis,role model hypothesis,*vaping<br><br>**Abstract:** Introduction: The popularity of electronic cigarettes (e-cigarettes) among adolescents is growingworldwide. A more accurate model than the much discussed but inadequate Gateway Hypothesisis needed to explain some adolescents' initial preference for e-cigarettes over tobacco cigarettes,as well as any transition from e-cigarettes to tobacco smoking. Our aim was to summarize the diffusefear that adolescents will be indirectly encouraged to begin smoking tobacco via the use ofe-cigarettes and to systematize the disparate causal hypotheses used thus far in relevant literature.Methods: We summarized the vague and fragmented hypotheses formulated thus far in literatureon both trajectories from abstinence to e-cigarette use and from there to tobacco smoking into aset of empirically testable hypotheses and organized them into a comprehensive model.Results: Our results indicate that the perceived health risks, specific product characteristics (suchas taste, price and inconspicuous use), and higher levels of acceptance among peers and otherspotentially make e-cigarettes initially more attractive to adolescents than tobacco cigarettes. Later,increasing familiarity with nicotine could lead to the reevaluation of both electronic and tobaccocigarettes and subsequently to a potential transition to tobacco smoking. The suggested "catalystmodel" takes variations in the nicotine content of e-cigarettes as well as the dual use of differentsubstances into account.Conclusion: Our model provides causal hypotheses for the initiation of e-cigarette use and forthe potential transition to tobacco smoking

	which, after being tested in empirical studies, couldlead to the formulation of concrete recommendations for healthcare intervention and preventionmeasures.Implications: We developed a model that provides causal hypotheses for the initiation of e-cigaretteuse and for the potential transition to tobacco smoking.Copyright © The Author 2015.
519	Sutfin E.L.,Sparks A.,Pockey J.R.,Suerken C.K.,Reboussin B.A.,Wagoner K.G.,Spangler J.,Wolfson M. First tobacco product tried: Associations with smoking status and demographics among college students. Addictive Behaviors; 2015;51:152.<br><br>**Keywords:**<br>adolescent,adult,article,*college student,controlled study,cross-sectional study,*demography,electronic cigarette,female,groups by age,*health status,health survey,human,major clinical study,male,prevalence, sex difference,smokeless tobacco,*smoking,*tobacco,tobacco use,United States,university student,young adult<br><br>**Abstract:** Introduction: As the tobacco market expands, so too have the opportunities for youth to be introduced to nicotine. The goal of this study was to identify product choice for initial tobacco trial, correlates associated with product choice, and the relationship between first product and current cigarette smoking among college students. Methods: A cross-sectional web survey of 3146 first-year students at 11 universities in North Carolina and Virginia was conducted in fall 2010. Results: Weighted prevalence of ever use of tobacco was 48.6%. Cigarettes were the most common first product (37.9%), followed by cigars (29.3%), hookahs (24.6%), smokeless tobacco (6.1%), and bidis/kreteks (2.2%). Two thirds (65%) of current smokers initiated with cigarettes, but 16.4% started with cigars, 11.1% with hookahs, 5.7% with smokeless, and 1.7% with bidis/kreteks. Females were more likely to report their first product was cigarettes and hookahs, while males were more likely to start with cigars and smokeless tobacco. Compared to those whose first product trial occurred after the age of 18, younger age of initiation (17. years or younger) was associated with cigarettes and smokeless as first products, while older age of initiation (18 or older) was associated with starting with hookahs and cigars. Dual or poly tobacco use was more common among those who initiated with hookahs and smokeless tobacco. Conclusions: While over a third of students used cigarettes first, two thirds started with a non-cigarette product. Just about a third of current cigarette smokers initiated with a non-cigarette product, suggesting that those non-cigarette products may have facilitated escalation to cigarettes.Copyright © 2015 Elsevier Ltd.
526	Toumbis M. Electronic nicotine delivery systems: A review for clinicians. Pneumon; 2016;29:308.<br><br>**Keywords:**<br>adolescent development,adolescent smoking,adulthood,adverse outcome,advertising,aerosol,brain development,cardiovascular system,chemical composition,clinical effectiveness,device safety,*electronic cigarette,fetus development,health hazard,human,immune system,malignant neoplasm,marketing, nonhuman,passive smoking,prevalence,respiratory system,review,risk assessment,smoking cessation, tobacco dependence,*vaping,nicotine<br><br>**Abstract:** The prevalence of the use of electronic nicotine delivery systems (ENDS) has increased rapidly in the past few years. In the absence of clear negative health messages, the decision of adults on whether or not to initiate ENDS use is influenced by various factors, including the pervasive promotion of ENDS as being effective in smoking cessation and a safe alternative to tobacco, which are unsubstantiated claims. The appeal of advertisements promoting ENDS as an enjoyable lifestyle choice and a high-tech product influences the choice of young people. Thus, the most common reasons for ENDS use in adults are its perceived safety and its efficacy as a cigarette cessation aid. Curiosity, flavors, and peer influence have been identified as the top reasons for ENDS use in adolescents and young people. Although ENDS are generally considered as a single product class, the systems constitute a diverse group with differences in the production and delivery of the various agents. The composition of the aerosol generated by ENDS

	depends on several factors, including, the electronic liquid constituents, the ENDS features, and user behavior. Currently available data indicate that ENDS aerosols are not harmless, especially with respect to body systems that are sensitive to various toxic effects. The literature on the effects of ENDS on the various body systems is sparse and marked by a lack of standardization in methods. The long-term effects of ENDS are as yet unknown. Studies of short-term exposure to ENDS aerosol document several biological and functional effects on the respiratory, cardiovascular, immune, and central nervous systems. At least under certain conditions, ENDS have been shown to deliver physiologically active quantities of nicotine, and can thus produce and/or maintain nicotine dependence. The current evidence is sufficient to justify cautioning pregnant women, women of reproductive age, children and adolescents about the hazards of ENDS use, because of the potential for fetal and adolescent nicotine exposure to have long-term consequences for brain development. Passive exposure to ENDS aerosol has not been well studied because ENDS are relatively new products, but it is of concern, because of its potential adverse health effects for people who are involuntarily exposed. The majority of relevant studies concluded that passive exposure to ENDS aerosol may indeed pose a health risk. The evidence for the effectiveness of ENDS as a method for quitting or reducing tobacco smoking is limited and of low quality. Some studies demonstrated a significant relationship between ENDS use and increases in smoking cessation, while others found no association or a negative one. The findings of prospective studies on adolescent and young adult populations suggest that ENDS use is a clear and consistent indicator of the likelihood of subsequent initiation of cigarette and other combustible tobacco product use, at ages spanning from early adolescence through emerging adulthood.Copyright © 2016, Technogramma. All rights reserved.
531	Lobaszewski J.,Didkowska J. Electronic cigarettes: A new challenge for Polish public health. Polskie Archiwum Medycyny Wewnetrznej; 2016;126:905.<br><br>**Keywords:**<br>advertising,cancer risk,cardiovascular disease,chronic obstructive lung disease,commercial phenomena, *electronic cigarette,health care policy,health hazard,human,information dissemination,letter,lung cancer,medical information,medical research,motivation,*Poland,prevalence,*public health service, smoking,smoking cessation program,teratogenicity,nicotine/to [Drug Toxicity]<br><br>**Abstract:**
532	Britton J.,Arnott D.,Mcneill A.,Hopkinson N. Nicotine without smoke-putting electronic cigarettes in context: John britton and colleagues set out why a new royal college of physicians report supports the role of electronic cigarettes as part of a comprehensive tobacco control strategy. BMJ (Online); 2016;353:i1745.<br><br>**Keywords:**<br>article,disease exacerbation,*electronic cigarette,health hazard,health impact assessment,health promotion,human,life expectancy,priority journal,quality of life,risk benefit analysis,risk reduction, *smoking,smoking cessation,smoking regulation,tobacco dependence,tobacco industry,tobacco use, *nicotine<br><br>**Abstract:**
537	Kristjansson A.L.,Mann M.J.,Sigfusson J.,Sarbu E.A.,Grubliauskiene J.,Daily S.M.,Sigfusdottir I.D. Prevalence of e-cigarette use among adolescents in 13 Eastern European towns and cities. Public Health; 2017;147:66.<br><br>**Keywords:**<br>adolescent,*adolescent smoking,article,cross-sectional study,Eastern Europe,electronic cigarette, geographic distribution,human,prevalence,sex difference,Sweden,tobacco dependence,urban population,*vaping

	**Abstract:**
545	Ghazal S.,Akhter S.,Aziz H.W.,Warraich U.A.,Shah W.H.,Javed U.,Deen I.U. Knowledge, attitude and perception regarding e-cigarette among post graduate medical trainees in Pakistan. European Respiratory Journal; 2016;48:<br><br>**Keywords:**<br>analysis of variance,awareness,city,*electronic cigarette,friend,human,human experiment,male, *Pakistan,*perception,*postgraduate student,pulmonology,social media,statistical significance,Student t test,nicotine<br><br>**Abstract:** Despite advertisement by industry about electronic cigarettes (e-cigarettes) as being safer substitute for regular cigarettes scientific data raises serious concerns regarding its effectiveness. AIM: To assess knowledge and perceptions of post graduate medical trainees (PGs) in Pakistan regarding e-cigarettes and their attitude towards its use for themselves and in their patients. Method: uestionnaire was filled by 406 PGs of various specialties from 5 major cities of Pakistan. ata was analyzed using SPSS 22. Correlations were used to assess the relationship between variables. ne-way Anova and Independent Sample t-test were applied. P value < 0.05 was taken as significant. Results: Among respondents 59% know of e-cigarettes, majorly through acquaintances (55%) and social media (28%). Males were more likely to know about e-cigarettes (0.00)) with 1.86 times more chances to use it in future. n evaluating awareness among different cities, arachi scored lower than Islamabad (p 0.03) and Lahore (p 0.056) and greater than Multan (p 0.003). Pulmonology PGs were found to have significantly higher chances of knowing about e-cigarettes compared to other disciplines. Significant correlation was found between current smokers and higher chances of their knowing about e-cigarettes and increased likelihood of its use compared to others. PGs with friends using e-cigarettes had 3 times more chances to indulge in it in future (p 0.00). Age was found to have positive correlation of 0.185 with attitude towards use (0.004). Conclusion: ith growing popularity of e-cigarettes steps to provide medical trainees with updated information about these novel devices of nicotine use need to be taken.
547	Echevarria C.,Sinha I. Heterogeneity in the measurement and reporting of outcomes in studies of electronic cigarette use in adolescents, a systematic analysis of observational studies. European Respiratory Journal; 2016;48:<br><br>**Keywords:**<br>adolescent,consensus,cross-sectional study,*electronic cigarette,human,*observational study, prevalence,Scopus,nicotine<br><br>**Abstract:** Uncertainty about whether electronic cigarettes act as a gateway to tobacco addiction for adolescents has led to global debate on this topic. We examined consistency between cross-sectional studies of conventional and electronic cigarette use among adolescents in terms of the measurement, analysis, and reporting of parameters. A systematic analysis was performed of cross-sectional studies of conventional and electronic cigarette use in adolescents, to identify measured and reported parameters. Studies were identified by searching the SCOPUS database in August 2015. Two reviewers independently selected relevant studies from the search and extracted data. 60 abstracts were identified; 31 papers were eligible for review (23 unique studies). Measured and reported parameters were collated, including the prevalence of cigarette and/ or e-cigarette use, and the definitions of terms. For "ever" or "current" use of electronic or conventional cigarettes, there were 17 unique permutations of measured parameters across the 23 studies, and 33 different data points were presented. Of 16/23 studies in which authors collected information on "dual current use", this prevalence was reported in 11/16. "Dual use" was calculated and reported in six different ways across all studies. Many studies assumed that "e-cigarette" use was synonymous with nicotine use. There are substantial differences in the measurement and

	reporting of parameters in these studies, which are consequently at risk of reporting bias. A core outcome set should be developed in this area, using structured consensus techniques.
551	Keogan S.,Taylor K.,Babineau K.,Clancy L.J. A 2015 national survey of e-cigarette use among Irish youth. European Respiratory Journal; 2016;48:<br><br>**Keywords:**<br>adolescent,controlled study,*electronic cigarette,human,Ireland,*Irish (citizen),juvenile,major clinical study,smoking,student,tobacco dependence,alcohol<br><br>**Abstract:** Background: Tobacco use among young people in Ireland is declining but remains a problem. The use of e-cigarettes is increasing and its implications unsure. In our previous Irish study, 23.8% (n=196) of respondents had used e-cigarettes at least once and 4.2% of never smokers had used e-cigarettes (Babineau, K. et al. (2015) PLoS ONE 10(5)). Design/Methods: A cross-sectional, nationally-representative survey was conducted with 2028 young people aged 15-17 as part of the European School Survey Project on Alcohol and Other Drugs (ESPAD). This included 4 items on e-cigarettes as well as several items on smoking behaviours and attitudes. Results: 24.7% (n=482) reported use of e-cigarettes at least once, including 214 students who reported using e-cigarettes in the last 30 days (11.0%). 130 students reported daily use of e-cigarettes (6.8% of sample). 18.3% of e-cigarette users (n=88) cited quitting tobacco as a reason for using e-cigarettes. The most common reason for using e-cigarettes was "out of curiosity" 61.2%, (n=295). 8.3% of never smokers had used e-cigarettes (n=109). 13.4% (n=272) reported that they had smoked one or more cigarettes in the last 30 days, 6.9% smoked daily (n=139). Two-thirds of students (66.7%, n=1,350) reported that they had never smoked a cigarette. Conclusion: E-cigarettes are popular among youth in Ireland, with around a quarter of respondents reporting they had used e-cigarettes. E-cigarettes are now used as commonly as cigarettes. Even more worrying is the finding that there has been an almost doubling of e-cigarette usage among never smokers in the current study. The implications for future smoking is unknown but nicotine addiction seems set to continue.
561	Kozlowski L.T.,Homish D.L.,Homish G.G. Daily users compared to less frequent users find vape as or more satisfying and less dangerous than cigarettes, and are likelier to use non-cig-alike vaping products. Preventive Medicine Reports; 2017;6:111.<br><br>**Keywords:**<br>adult,army,article,controlled study,*electronic cigarette,female,Fisher exact test,hazard,human,human experiment,logistic regression analysis,longitudinal study,male,multivariate analysis,perception,priority journal,risk assessment,sample size,satisfaction,*smoking,smoking habit,*tobacco use,univariate analysis,*vaping,nicotine<br><br>**Abstract:** We assessed the roles of perceived satisfaction and perceived danger and vaping-product-type as correlates of more frequent use of vaping products. In a baseline assessment of a longitudinal study of US Army Reserve/National Guard Soldiers and their partners (New York State, USA, 2014-2016), participants were asked about current use of vaping products (e-cigarettes) and perceived satisfaction and danger in comparison to cigarettes as well as type of product used. Fisher-exact tests and multiple ordinal logistic regressions were used. In multivariable and univariate models, more perceived satisfaction, less perceived danger, and use of non-cig-alike products were associated with more frequent use of vaping products (ps < 0.05, two-tailed). For self-selected, more frequent adult users, e-cigs can be at least as satisfying as cigarettes and often more satisfying and are perceived as less dangerous than cigarettes. Non-cig-alike products were more likely in daily users. Some concern that e-cigs are a gateway to cigarettes arises from assuming that e-cigs may not be as reinforcing and pleasurable as cigarettes. These results indicate that accurate perception of comparative risk and use of more effective-nicotine delivery product can produce for some users a highly-satisfying alternative to cigarettes.Copyright © 2017 The Authors

569	Farsalinos K.,Le Houezec J. Electronic cigarette experimentation in the Malaysian city of Kuantan: Was there an association with the smoking status?. Journal of Pharmacy and Bioallied Sciences; 2016;8:347.<br><br>**Keywords:**<br>awareness,comparative study,demography,*electronic cigarette,*experimentation,gender,human,letter, lifestyle and related phenomena,logistic regression analysis,Malaysia,odds ratio,perception,prevalence, sex difference,smoking habit,social status,tobacco,vaping<br><br>**Abstract:**
570	Winhusen T. Introduction to Special Issue on Tobacco and Other Substance Use Disorders: Links and Implications. American Journal of Drug and Alcohol Abuse; 2017;43:129.<br><br>**Keywords:**<br>adolescent smoking,age,alcoholism,cholinergic system,disease association,dopaminergic system,*drug dependence,electronic cigarette,human,nonhuman,note,personalized medicine,positron emission tomography,serotoninergic system,sex difference,*tobacco dependence,translational research,treatment outcome,treatment planning,dopamine/ec [Endogenous Compound],neurotransmitter/ec [Endogenous Compound],nicotine,nicotinic receptor/ec [Endogenous Compound],nicotinic receptor blocking agent<br><br>**Abstract:**
601	Braillon A. Electronic nicotine delivery systems, from both sides of the Atlantic. Pediatrics; 2016;137:e20154356.<br><br>**Keywords:**<br>disease control,*drug delivery system,electronic cigarette,epidemic,evidence based medicine,food and drug administration,human,letter,prevalence,priority journal,public health service,risk reduction,smoking cessation,*tobacco dependence/dt [Drug Therapy],*tobacco dependence/ep [Epidemiology],tobacco dependence/dt [Drug Therapy],United Kingdom,*nicotine/dt [Drug Therapy],*nicotine/pr [Pharmaceutics],*electronic nicotine delivery system<br><br>**Abstract:**
610	Ioakeimidis N.,Vlachopoulos C.,Tousoulis D. Efficacy and safety of electronic cigarettes for smoking cessation: A critical approach. Hellenic Journal of Cardiology; 2016;57:1.<br><br>**Keywords:**<br>cardiovascular disease,*electronic cigarette,human,lung disease,medical literature,public health, publication,randomized controlled trial (topic),review,risk factor,safety,*smoking cessation,systematic review,acetaldehyde,formaldehyde,glycerol,heavy metal,nicotine,propylene glycol,tobacco smoke<br><br>**Abstract:**
614	Wise J. Teen vaping is linked to higher odds of heavy cigarette smoking. BMJ (Online); 2016;355:i6025.<br><br>**Keywords:**<br>adolescent,attitude to health,*electronic cigarette,human,note,priority journal,*smoking,smoking cessation<br><br>**Abstract:**
617	Camenga D. E-cigarette use associated with tobacco smoking. Journal of Pediatrics; 2016;178:303.

	**Keywords:**<br>adolescent,*adolescent smoking,adverse outcome,article,demography,*electronic cigarette,human, priority journal,risk factor,self report,smoking cessation<br><br>**Abstract:**
622	Berg C.D. Just say no! Smoking abstinence works. American Journal of Respiratory and Critical Care Medicine; 2016;193:476.<br><br>**Keywords:**<br>African American,cancer mortality,cancer screening,computer assisted tomography,editorial,electronic cigarette,health hazard,human,*lung cancer,mortality risk,patient counseling,prevalence,priority journal, public health service,reimbursement,risk assessment,risk factor,risk reduction,smoking,*smoking cessation,*smoking cessation program,smoking regulation,tax,tobacco consumption<br><br>**Abstract:**
641	Urrutia-Pereira M.,Oliano V.J.,Mallol J.,Sole D. Smoking among adolescents from a countryside city of Brazil and the future impact in respiratory diseases. Allergy: European Journal of Allergy and Clinical Immunology; 2016;71:255.<br><br>**Keywords:**<br>adolescent,*adolescent smoking,*Brazil,child,electronic cigarette,father,female,friend,gender,human, major clinical study,male,prevalence,prospective study,questionnaire,*respiratory tract disease,*risk factor,school child,*smoking cessation<br><br>**Abstract:** Objective: Despite the anti-smoking prevention programs, many teens start smoking at school age. The objectives were to determine the prevalence and risk factors associated with smoking in adolescents living in Uruguaiana, RS, Brazil. Methods: A prospective study conducted in adolescents (12-19 years), enrolled in municipal schools, who answered a selfadministered questionnaire on smoking. Results: 798 adolescents were enrolled in the study, with equal distribution between genders. The tobacco experimentation frequency (ever tried a cigarette, even one or two puffs) was 29.3%, and 14.5% started smoking before 12 years of age and 13.0% of them said they smoked at least one cigarette/day last month. Teens reported having ease in getting cigarettes (OR: 1.77; 95% CI: 1.33-2.35) as well as having been affected by a smoker friend (OR: 1.78; 95% CI: 1.44-2 20). Using electronic cigarettes (OR: 2.10; 95% CI: 1.50-2.94), try hookah (OR: 2.37; 95% CI: 1.91-2.94) and live with smokers (OR: 1.93; 95% CI: 1.57-2.38) were significantly associated with active smoking among teenagers. The non smoking father's or non smoking mother's advices were identified as a protective factor for adolescents not initiate smoking (OR: 0.61; 95% CI: 0.49-0.76). Conclusion: The prevalence of smoking among adolescents in Uruguaiana is high. The implementation of measures to reduce/stop tobacco use and its new forms of consumption, such as electronic cigarettes and hookah in schools are urgent and imperative.
658	Todea D.,Coman A. Factors affecting cigarette smoking and electronic cigarette consumption among teenagers from Cluj-Napoca area, Romania. Chest; 2016;149:A597.<br><br>**Keywords:**<br>*smoking,*electronic cigarette,*human,*Romania,*adolescent,tobacco,parent,friend,risk,money,male, population,demography,questionnaire,adolescence,high school,cross-sectional study,psychological aspect,prevalence,diseases,side effect,Internet,adult,risk factor,protection,tobacco use,tobacco consumption,health service<br><br>**Abstract:** PURPOSE: Tobacco use in teens is highly prevalent and seems to be influenced not only by psychological factors. Also, electronic cigarette (e-cigarette) in teenage population has a great popularity.

	Actually teens interested on cigarette or e-cigarete will become future regular adult smokers. The purpose of this study was to evaluate the prevalence and factors affecting smoking cigarette and e-cigarette consumption among teens in Cluj-Napoca area, Romania. METHODS: Data were obtained from a cross-sectional study in November 2014 by applying to adolescents from 3 high-schools, aged between 15-17 years anonymous questionnaires containing demographic characteristics, use of tobacco, attitudes of family and friends regarding smoking consumption and also question to assessed electronic cigarette use and cigarette use, psychosocial risk and protective variables. RESULTS: From 532 adolescents, mean age was 14.4 (SD=2.23) years, 58% were males, 26% were smokers, 89% have had knowledge about e-cigarette from especially from friends (67%), internet (12%) and parents (4%). From smokers 40% used both electronic cigarette (dual users) and from non smokers only 29% used it; those who were males (OR=1.32), who received money from parents more than 10 euro/day (OR=1.41), whose parents (OR=1.55) or friends smoked (OR=1.98) were more likely to be susceptible to cigarette consumption. Adolescents who received money from parents less than 2 euro/day (OR=0.23),who had access to anti-tobacco media (OR=0.73), and had knowledges about side effects of smoking consumption (OR=0.46) were less susceptible to smoking consumption. Dual users and only cigarette users were highest on risk status (high on risk factors and lower on protective factors) compared with other groups (p=0.000). Electronic cigarette users were higher on risk status than nonusers but lower than dual users. (p<0.0001). CONCLUSIONS: All forms of anti-tobacco media have a protective role against susceptibility to smoking among adolescents. Efforts to prevent adolescents smoking and e-cigarette consumption should be designed and should include all forms of tobacco control. CLINICAL IMPLICATIONS: The succesfull in tobacco control both in cigarette smonking and e-cigarette consumption in adolescents will prevent the future diseases atribute to tobacco consumption and will prevents the costs in Health Services.
673	Dautzenberg B.,Berlin I.,Tanguy M.-L.,Rieu N.,Birkui P. Factors associated with experimentation of electronic cigarettes among Parisian teenagers in 2013. Tobacco Induced Diseases; 2015;13:65.<br><br>**Keywords:**<br>adolescent,*adolescent behavior,adult,article,child,*child behavior,controlled study,cross-sectional study,demography,*electronic cigarette,*experimentation,factorial analysis,family attitude,female, France,friend,health survey,human,male,population research,prevalence,priority journal,questionnaire, *risk factor,school child,school health service,self report,sibling,smoking,*social behavior,urban area<br><br>**Abstract:** Background: Electronic cigarettes (e-cig.) became widely used among adults. Data are insufficient about e-cig. experimentation among youth. Methods: To assess prevalence of e-cig. experimentation and associated factors among the 12 to 19 years old we analyse a cross-sectional school based survey in the city of Paris, France in 2013 on a randomly selected sample of 2 % of schoolchildren (n = 3 279). Self-report questionnaire include demographic, individual and family smoking characteristics and questions about e-cig: "Have you ever used an e-cigarette?"- "Did you use e-cigarette in the last 30 days?"-"Did you try e-cigarette as a first tobacco product?" Results: In 2013, 17.9 % (564) schoolchildren reported having experienced the e-cig (boys: 19.0 %, girls: 16.8 %) compared to 9.8 % in 2012. Experimentation rate increases from 5 % among the 12 to 30 % among the 16-year-old. E-cig. experimentation was significantly associated with 11 parameters including: age >15 years (OR: 0.66 (IC95 % = 0.46-0.94)); smoking 10 cigarettes or more (OR = 5.67 (IC95 % = 3.11-10.34)), best friends and siblings smoker (OR = 1.54 (IC95 % = 1.11-2.14)) and (OR = 1.88 (IC95 % = 1.41-2.52)); experimentation of shisha (OR = 2.60 (IC95 % = 1.75-3.86)), cannabis use (OR = 1.90 (IC95 % = 1.32-2.72)); having two parents who forbid smoking (OR = 2.32 (IC95 % = 1.63-3.30)). Only 5.6 % of the study population (and 32.5 % of e-cig. experimenters (183/564)) have used it in the last 30 days; 1.7 % of the study population and 10.0 % of e-cig. experimenters were non-smokers (56/564). Conclusions: Rate of e-cig. experimentation among schoolchildren increased by 8.1 % in 1 year. Non-smoking youth may use e-cig. Prospective studies are urgently needed to assess the evolution of e-cig. use both among smoking and non-smoking youth.Copyright © 2015 Dautzenberg et al.

683	Stanwick R. E-cigarettes: Are we renormalizing public smoking? Reversing five decades of tobacco control and revitalizing nicotine dependency in children and youth in Canada. Paediatrics and Child Health (Canada); 2015;20:<br><br>**Keywords:**<br>*adolescent smoking,aerosol,article,Canada,human,particulate matter,*smoking,smoking ban,tobacco consumption,*tobacco dependence,*electronic cigarette/to [Drug Toxicity],glycerol,heavy metal, *nicotine,propylene glycol,toxin<br><br>**Abstract:** An electronic cigarette (e-cigarette) is a battery attached to a chamber containing liquid that may (or may not) contain nicotine. The battery heats the liquid and converts it into a vapour, which is inhaled, mimicking tobacco smoking. The e-cigarette does not rely on tobacco as a source of nicotine but, rather, vaporizes a liquid for inhalation. Eliquids are often flavoured and may contain nicotine in various concentrations, although actual amounts are seldom accurately reflected in container labelling. The deleterious effects of nicotine on paediatric health are well established. The use of e-cigarettes in the paediatric age group is on the rise in Canada, as are associated nicotine poisonings. E-devices generate substantial amounts of fine particulate matter, toxins and heavy metals at levels that can exceed those observed for conventional cigarettes. Children and youth are particularly susceptible to these atomized products. Action must be taken before these devices become a more established public health hazard. Policies to denormalize tobacco smoking in society and historic reductions in tobacco consumption may be undermined by this new 'gateway' product to nicotine dependency.Copyright © 2015 Canadian Paediatric Society.
688	Furber A. Electronic cigarettes: Reasons to be cautious. Thorax; 2015;70:307.<br><br>**Keywords:**<br>advertizing,asthma,comparative effectiveness,cost effectiveness analysis,editorial,*government regulation,health hazard,human,licensing,national health service,nicotine replacement therapy, prescription,prevalence,priority journal,risk benefit analysis,risk reduction,*smoking regulation,tobacco dependence/th [Therapy],tobacco industry,United Kingdom,*electronic cigarette<br><br>**Abstract:**
690	Anonymous. E-cigarette users less likely to quit smoking. U.S. Pharmacist; 2015;40:<br><br>**Keywords:**<br>adolescent,*electronic cigarette,human,note,smoking,*smoking cessation,nicotine<br><br>**Abstract:**
692	Ambrose B.K.,Day H.R.,Rostron B.,Conway K.P.,Borek N.,Hyland A.,Villanti A.C. Flavored tobacco product use among US youth aged 12-17 Years, 2013-2014. JAMA - Journal of the American Medical Association; 2015;314:1871.<br><br>**Keywords:**<br>adolescent,adult,child parent relation,cohort analysis,controlled study,electronic cigarette,female,health survey,household,human,informed consent,interview,letter,longitudinal study,male,prevalence,priority journal,smokeless tobacco,smoking,*tobacco use,United States,cigarette smoke,bidis,dissolvable tobacco,hookah,kretek,pipe tobacco<br><br>**Abstract:**
693	Kamerow D. Fuming about e-cigarettes and harm. BMJ (Online); 2015;351:h4634.

	**Keywords:**<br>*electronic cigarette,government regulation,*harm reduction,high school student,human,prevalence, priority journal,public health service,short survey,smoking,smoking cessation,United Kingdom<br><br>**Abstract:**
696	Farsalinos K.E.,Le Houezec J. Regulation in the face of uncertainty: The evidence on electronic nicotine delivery systems (e-cigarettes). Risk Management and Healthcare Policy; 2015;8:157.<br><br>**Keywords:**<br>clinical effectiveness,device safety,*drug delivery device,*electronic cigarette,government regulation, human,*medical device regulation,nicotine replacement therapy,outcome assessment,patient satisfaction,product safety,reimbursement,review,risk reduction,smoking,smoking cessation,tobacco consumption,tobacco dependence/th [Therapy],tobacco industry,*nicotine<br><br>**Abstract:** Tobacco smoking is the largest single preventable cause of many chronic diseases and death. Effective treatments exist; however, few smokers use them and most try to quit by themselves. Most of the tobacco cigarette's toxicity is related to the combustion process. Models of harm reduction applied to tobacco suggest that switching from inhalation of combustible products to a noncombustible nicotine delivery product would likely result in a vast reduction in tobacco-related death and illness. Currently available evidence raises no doubt that electronic cigarettes (e-cigs) are by far less harmful than smoking (although probably not absolutely safe) and have the potential to be the most effective tobacco harm reduction products due to their unique property of resembling smoking and providing satisfaction to the user. A lot of controversy is surrounding e-cigs and their regulation, much of which is based on the precautionary principle. Although monitoring and further research is definitely needed, the arguments used to implement severe restrictions or bans are mostly hypothetical, weakly supported by evidence, and, in some cases, derived from mispresentation or misinterpretation of the study findings. Regulators should keep in mind that the target population is smokers who want to reduce or quit their deadly tobacco consumption. To achieve this goal, smokers should be honestly informed on the relative harmfulness of the different products. E-cigs are not tobacco products and are not used as medications. For this reason, a specific regulatory scheme is needed, separate from tobacco or medicinal products regulation. Regulation should implement specific quality criteria for products, rules for the exclusion of chemicals of reasonable concern, and appropriate testing for possible contaminants. Additionally, manufacturing standards derived from the food industry should be implemented and adjusted for specific conditions related to e-cigs. Finding the appropriate balance between safety and acceptability of use by smokers will be important in achieving the maximum public health benefit. Labeling should be specified, with warnings about exposure to skin or through ingestion and discouragement of use by nonsmokers, related to the presence of nicotine. Finally, advertising and marketing should not be banned, but appropriately regulated in order to encourage use by the intended population while avoiding use by never-smokers. E-cigs should be appealing to smokers (but not to nonsmokers), while availability and pricing should be strong competitive advantages of e-cigs relative to tobacco cigarettes.Copyright © 2015 Farsalinos and Le Houezec.
697	Chaouachi K. Use & misuse of water-filtered tobacco smoking pipes in the world. Consequences for public health, research & research ethics. Open Medicinal Chemistry Journal; 2015;9:1.<br><br>**Keywords:**<br>article,bibliometrics,carbon monoxide intoxication,electronic cigarette,health services research,human, hygiene,prevalence,priority journal,public health,research ethics,*smoking,tobacco dependence,toxicity testing,*tube,arsenic,cadmium,chromium,lead,tobacco smoke,tumor marker/ec [Endogenous Compound],*water filtered tobacco smoking pipe<br><br>**Abstract:** Background: The traditional definition of an "epidemic" has been revisited by antismoking

	researchers. After 400 years, Doctors would have realized that one aspect of an ancient cultural daily practice of Asian and African societies was in fact a "global "epidemic"". This needed further investigation particularly if one keeps in his mind the health aspects surrounding barbecues. Method: Here, up-to-date biomedical results are dialectically confronted with anthropological findings, hence in real life, in order to highlight the extent of the global confusion: from the new definition of an "epidemic" and "prevalence" to the myth of "nicotine "addiction"" and other themes in relation to water filtered tobacco smoking pipes (WFTSPs). Results: We found that over the last decade, many publications, -particularly reviews, "meta-analyses" and "systematic reviews"- on (WFTSPs), have actually contributed to fuelling the greatest mix-up ever witnessed in biomedical research. One main reason for such a situation has been the absolute lack of critical analysis of the available literature and the uncritical use of citations (one seriously flawed review has been cited up to 200 times). Another main reason has been to take as granted a biased smoking robot designed at the US American of Beirut whose measured yields of toxic chemicals may differ dozens of times from others' based on the same "protocol". We also found that, for more than one decade, two other main methodological problems are: 1) the long-lived unwillingness to distinguish between use and misuse; 2) the consistent unethical rejection of biomedical negative results which, interestingly, are quantitatively and qualitatively much more instructive than the positive ones. Conclusion: the great majority of WFTSP toxicity studies have actually measured, voluntarily or not, their misuse aspects, not the use in itself. This is in contradiction with both the harm reduction and public health doctrines. The publication of negative results should be encouraged instead of being stifled.Copyright © Kamal Chaouachi.
698	Roller L.,Gowan J. Tobacco, nicotine and smoking. Australian Journal of Pharmacy; 2015;96:58.<br><br>**Keywords:**<br>apoptosis,article,Australia,electronic cigarette,follow up,human,ischemic heart disease,lung cancer, morbidity,mortality,nicotine replacement therapy,nonhuman,pharmacist,plasma clearance,prevalence, *smoking,smoking cessation,Solanaceae,*tobacco,tobacco dependence/ep [Epidemiology], amfebutamone,*nicotine,varenicline<br><br>**Abstract:**
705	Schraufnagel D.E. Electronic Cigarettes: Vulnerability of Youth. Pediatric, Allergy, Immunology, and Pulmonology; 2015;28:2.<br><br>**Keywords:**<br>health hazard,human,*juvenile,priority journal,review,risk assessment,*smoking,smoking cessation, substance abuse,tobacco,*tobacco dependence,vulnerable population,*electronic cigarette,nicotine<br><br>**Abstract:** Electronic cigarettes have become popular and are heavily promoted as a safer cigarette and an aid to quit smoking. Although they may have value in reducing cigarette use among smokers, they are of limited value in smoking cessation and pose many problems, particularly in children. Nicotine is highly addictive and affects virtually all cells in the body. It is particularly harmful to developing brains and other organs. The electronic nicotine delivery systems are largely uncontrolled and safety risks are manifold. Initiating nicotine use and increasing dependence in the population may be linked with increased tobacco and other addictive substance abuse even if the individual electronic cigarette delivers less harm than a combustible cigarette does.© Copyright 2015, Mary Ann Liebert, Inc.
711	Khan J. E-cigarettes: A gateway to nicotine addiction?. Journal of Postgraduate Medical Institute; 2015;29:213.<br><br>**Keywords:**<br>accidental death,bronchiolitis,commercial phenomena,device approval,device safety,disease association, editorial,*electronic cigarette,elevated blood pressure,government,health education,heart arrhythmia,

	human,intermethod comparison,intoxication,Loeffler pneumonia,mass medium,nicotine replacement therapy,Pakistan,pneumonia,practice guideline,risk assessment,smoking,smoking ban,smoking cessation, tachycardia,*tobacco dependence/dt [Drug Therapy],tobacco dependence/dt [Drug Therapy], amfebutamone/dt [Drug Therapy],flavoring agent,glycerol,nicotine/to [Drug Toxicity],nicotine derivative/dt [Drug Therapy],propylene glycol,varenicline/dt [Drug Therapy]<br><br>**Abstract:**
718	Russi E.W. E-cigarettes: Promise or peril?. Swiss Medical Weekly; 2015;145:w14187.<br><br>**Keywords:**<br>cancer risk,concentration (parameters),editorial,*electronic cigarette,Frenchman,harm reduction,human, long term exposure,prevalence,questionnaire,risk factor,risk reduction,smoking,smoking cessation, tobacco consumption,cigarette smoke,nicotine,cardiopulmonary risk<br><br>**Abstract:**
764	Kaplan A. E cigarettes: Knowledge and attitudes in Canadian primary care physicians. European Respiratory Journal; 2015;46:<br><br>**Keywords:**<br>*Canadian,*general practitioner,*human,*European,*society,*electronic cigarette,physician,smoking, law,Canada,therapy,child,physician attitude,habit,advertizing,safety,patient,male,tobacco,smoking cessation,nicotine,carcinogen<br><br>**Abstract:** Electronic cigarettes(E cig) are being used for smoking cessation(SC), but with controversy about utility and safety. Physicians are discussing and advising E cig, but this has not yet been studied in Canada. An electronic study regarding physician attitude and experience regarding E cig was sent out to Canadian PCPs. 57 respondents, 68% male, 16% current or former smokers responded with 25% considering themselves knowledgeable or very knowledgeable about E cig. ~75% of the physician's patients had used E cig; less than half had discussed this with their physician. 7% said E cig are more effective than traditional SC strategies, 22% said less effective, 33% said non-comprable as they were so mechanistically different; but 38% said that they had insufficient knowledge. 58% agreed that E cig were safer than smoking, 8% less so, and 34% were neutral. 1/4 felt that they should be used to assist SC, 1/4 said they should not and 1/2 were undecided, despite an unidentified subsection of physicians(30%) who had personally tried an E cig which helped with their SC (67%). Specific legislation suggestions to improve the overall safe/appropriate use of E cigarettes included: no selling to minors (76%), no flavouring (53%), no advertising (51%), no nicotine (47%; current law in Canada); only 2% saying that no legislation was needed. Conclusion: E cigarettes can help the habit component of SC, especially if potential harm can be reduced. Concerns about E cig include the presence of carcinogens, being a gateway for children into tobacco, and their use as a social phenomenon with secondary harm in even non-smokers. There is a gap in knowledge about this mode of therapy in primary care physicians that needs to be addressed.
798	Primack B.A.,Soneji S.,Stoolmiller M.,Fine M.J.,Sargent J. Initiation of cigarette smoking after electronic cigarette use: A national study of young adults. Journal of General Internal Medicine; 2015;30:S193.<br><br>**Keywords:**<br>*smoking,*electronic cigarette,*young adult,*society,*internal medicine,human,follow up,risk,sensation seeking,Hispanic,parental smoking,model,African American,smoking cessation,mobile phone,vapor, telephone interview,adolescent,population,income,female,heating,longitudinal study,cross-sectional study,Caucasian,weight,public health,logistic regression analysis,education,household,sensitization, independent variable,dependent variable,tobacco,harm reduction,liquid,nicotine

	**Abstract:** BACKGROUND: An electronic cigarette (e-cigarette) contains a heating element that atomizes nicotine-containing liquid into an inhalable vapor. Compared with regular cigarettes, e-cigarettes seem to be lower in toxicants and associated adverse events, making them potentially valuable tools for smoking cessation. However, e-cigarette use is substantially increasing among young adults who are not users of traditional cigarettes. This large group of individuals may be at risk for sensitization to nicotine and transition to later cigarette smoking, which would have profound negative public health implications. While cross-sectional studies suggest that use of e-cigarettes is associated with intention to use regular cigarettes in the future and being open to future cigarette smoking, these important associations have not been studied longitudinally. Therefore, we conducted a longitudinal study among non-cigarette-smoking young adults to determine associations between baseline e-cigarette use and initiation of regular cigarette smoking 1 year later. METHODS: We studied 607 non-cigarette-smoking individuals ages 16-26 years who had complete longitudinal data from two study waves collected in 2012-2014. These participants had been recruited as part of a larger nationally-representative US study that recruited via a random-digit dial telephone survey using landline and cell phone frames. At baseline for the current study, 874 non-smoking individuals had complete data, and our response rate was 69 % (N=607) for 1-year follow-up data. Our primary dependent variable was initiation of smoking between baseline and follow-up, and our primary independent variable was whether a participant had used e-cigarettes at baseline. We used logistic regression with sample weights to assess bivariable and multivariable associations between baseline e-cigarette use and initiation of regular cigarette smoking while controlling for multiple relevant sociodemographic, personal, and environmental variables. RESULTS: Our sample of 607 individuals was 57 % female, 77 % Caucasian and non- Hispanic, 6 % African-American and non-Hispanic, 8 % Hispanic, and 10 % of mixed or other race. Only 9 (1.5 %) baseline participants was an e-cigarette user. Overall, 77 (12.7 %) baseline participants initiated cigarette smoking at follow-up, including 4 (44.4 %) among the baseline e-cigarette users and 73 (12.2 %) among e-cigarette nonusers. In a multivariable model that included all measured relevant covariates-age, sex, race/ethnicity, maternal education, household income, sensation seeking, parental smoking, and peer smoking-baseline e-cigarette was significantly associated with cigarette smoking initiation (AOR=4.1, P=.028). The other significant predictors were African-American race (AOR=2.6, P=.041), sensation seeking (AOR=1.86, P=.026) and peer smoking (AOR=1.78, P=.001). CONCLUSIONS: Baseline e-cigarette smokers had substantially greater odds (about 4 times as high) of subsequently initiating cigarette smoking. This odds ratio accounted not only for socio-demographic variables but also for important covariates such as sensation seeking tendencies and peer and parental smoking. These findings highlight the concern that the thousands of adolescents and young adults who were previously tobacco-free but are now initiating e-cigarette smoking may be at substantial risk for transition to regular cigarettes. While our study was limited by relatively low power due to the small number of e-cigarette only users at baseline, it is noteworthy that results were still significant and robust in an appropriate multivariable model. In conclusion, it is an important irony that a product which is marketed as a harm reduction tool for established cigarette smokers may in fact also become an important contributor to the development of a new population of cigarette smokers.
809	Akre C.,Suris J.-C. E-cigarettes as a gateway to smoking: What do adolescents themselves think?. Journal of Adolescent Health; 2015;56:S31.<br><br>**Keywords:**<br>*percutaneous transluminal angioplasty balloon,*smoking,*adolescent,*human,*society,*adolescent health,risk,information processing,inhalation,female,taste,adult,qualitative research,health practitioner, public health,parent,gesture,throat,satisfaction,smoke,procedures,addiction,thematic analysis,patient worry,sweetness,consumer,juvenile,policy,*electronic cigarette,nicotine<br><br>**Abstract:** Purpose: To determine whether electronic cigarettes (EC) can act as a gateway to smoking traditional cigarettes (TC) among adolescents. Methods: As part of a larger qualitative study on EC use including 42 adolescents/young adults (16-26y.o., 19 females) in 8 focus groups (FG) (4 FGs with EC users,

	2 with TC users, 1 with non-users, 1 with mixed consumption types), participants were asked if they believed that ECs could act as a gateway to smoking. FGs were audio-recorded and transcribed verbatim. Transcripts were analyzed according to a thematic analysis procedure. Results: Participants expressed significant threat of EC acting as a gateway to TC use among adolescents. Several factors encouraging this passage were reported: (1) EC was a smoothway of starting for those who found TC too brutal at first; (2) EC users got used to the smoking gestures, tastes, nicotine (if present), inhalation, and feel in the throat, all of which acted as an efficient preparation for real smoking; (3) EC did not bring much satisfaction in terms of effect, inducing a great risk to move on to TC; (4) groups of youths always included those who want to take their consumption further and pull their peers along, in this case from EC to TC; and (5) TCs were considered easier to use compared to ECs requiring a lot of preparation. Worries were accentuated by the powerful attractiveness of EC inciting many adolescents to try or use it due to: its fashion and popularity; the perception of EC as harmless, not seeing the potential underlying addiction, while enjoying the same socializing characteristics as smoking; the possibility of using EC in smoke-free areas; the lack of available information on EC and notably on its harmfulness; its ease to hide from parents; its ease to obtain; its sweet taste; and its high-tech/modern aspect. Participants considered an even greater gateway threat regarding very young adolescents given their naive perception of EC as harmless. Some participants reported that EC had acted as a gateway to TC for themselves either by starting their overall cigarette consumption with an EC or by starting EC after having stopped smoking for a while leading them to start smoking again. Only a small minority of participants did not perceive EC as a risk for a gateway to TC for two reasons: (1) EC could have the reverse effect of keeping adolescents away from TC; (2) because of the belief that no one would want to start ECs if not smoking TCs in the first place. Finally, some participants believed that the gateway threat existed only (for some) or specially (for others) if ECs contained nicotine. Conclusions: ECs are considered by adolescents themselves as having a great potential of acting as a gateway to smoking although perceived as a harmless substance. Health professionals should screen for EC use and inform consumers of the potential gateway effect. From a public health perspective, there is an urge for better preventive policies directed at protecting adolescents.
813	West R.,Brown J. Electronic cigarettes: Fact and faction. British Journal of General Practice; 2014;64:442.<br><br>**Keywords:**<br>device safety,editorial,health survey,human,prescription,prevalence,public health,randomized controlled trial (topic),smoking,*smoking cessation,tobacco dependence,world health organization,carcinogen, cigarette smoke,*electronic cigarette,nicotine,propylene glycol,varenicline<br><br>**Abstract:**
815	Anonymous. E-cigarettes linked to smoking among teens. Cancer Discovery; 2014;4:502.<br><br>**Keywords:**<br>adolescent,cross-sectional study,health education,health survey,human,note,public health,*smoking cessation,*electronic cigarette<br><br>**Abstract:** Teenagers who use electronic cigarettes are more likely to smoke conventional cigarettes than those who haven't tried the products, according to a new cross-sectional study published in JAMA Pediatrics. © 2014 American Association for Cancer Research.
824	Goldin M. E-cigarettes should not be promoted in pharmacies. Pharmaceutical Journal; 2014;293:493.<br><br>**Keywords:**<br>health promotion,human,letter,pharmacist,*pharmacy,*smoking,smoking cessation,*electronic cigarette<br><br>**Abstract:**

897	Pratt A.,Su L.,Audera-Lopez C.,Rarick J.,Mercado S. The rise of e-cigarettes: An emerging threat to the tobacco endgame?. Respiratory Medicine; 2013;107:S9.<br><br>**Keywords:**<br>*tobacco,*health care planning,*public health,health,systematic review (topic),devices,smoking, imitation,safety,methodology,tobacco dependence,protection,smoking cessation,government,human, population,marketing,systematic review,policy,world health organization,China,*electronic cigarette<br><br>**Abstract:** Background: Electronic cigarettes (e-cigarettes) first developed in China in 2004are gaining popularity and the number of e-cigarette users is growing rapidly. E-cigarette manufacturers and supporters of the devices argue that e-cigarettes are a safe alternative to tobacco smoking, and that they may aid smoking cessation. However, there is currently insufficient scientific evidence to prove their safety and efficacy. Furthermore, at the 5th session of the WHO FCTC Conference of Parties, concerns were raised that ENDs have been aggressively advertised and marketed as imitation or substitute tobacco products and as cessation devices around the world. Parties called for WHO to gather more evidence on whether e-cigarettes are safe, their efficacy as cessation aids, and that they don' t act as a gateway to nicotine addiction for young people. Objectives: The objective of this paper is to discuss health concerns on ecigarettes and to provide an overview of recent responses and regulations made by governments to ensure protection from potential health threats to individuals and populations. It will also examine the different marketing strategies employed by manufacturers in order to highlight the need for regulation of these products. Methodology: Systematic review of existing online information. Results: This paper consolidates existing information and evidence and outlines priorities for research and policy development.
922	Mathew B.C.,Daniel R.S.,Campbell I.W. E-cigarettes and narghile: Devices to a fatal addiction. Jamahiriya Medical Journal; 2009;9:245.<br><br>**Keywords:**<br>addiction,cardiovascular disease,cell cycle,chronic obstructive lung disease,endothelial dysfunction, epidemic,gene expression regulation,health care policy,health hazard,*health promotion,human, neoplasm,nicotine replacement therapy,oxidative stress,passive smoking,peer review,public health, review,risk assessment,*smoking,smoking cessation,tobacco,*tobacco dependence/th [Therapy], toxicology,carbon monoxide,heavy metal,K ras protein/ec [Endogenous Compound],nicotine,protein p53/ec [Endogenous Compound],tar<br><br>**Abstract:** Although smoking has been a major public health issue for many decades, a fairly new concern is the recent surge in development of new tobacco products and a revival of smoking methods that claim to reduce exposure to harmful tobacco constituents or that have fewer risks than cigarette smoking. Electronic cigarettes and water-pipe smoking are the two such faces of smoking that are gaining popularity worldwide .Electronic cigarettes are being promoted as a healthier alternative to tobacco smoking, and as a legitimate therapy for smokers trying to quit .However, no rigorous peer-reviewed studies have been conducted showing that the electronic cigarette is a safe and effective nicotine replacement therapy. On the contrary, the nicotine delivered by the electronic cigarette may be a "gateway" to other stimulant use. Tobacco smoking using a water pipe or narghile is a re-emerging strain in a global epidemic that has seen a worldwide resurgence in its use. The popular perception of water pipe smoking (WPS) less harmful than cigarettes is a total "farce" with studies reporting that the mainstream narghile smoke contain an abundance of toxic chemicals including carbon monoxide, nicotine," tar" and heavy metals that are thought to be causal factors in the elevated incidence of cancer, cardiovascular disease and addiction. Epidemiological and toxicological research is needed to study the additive properties, and the associated health risks, both for users and "passive smokers" of narghile. Aggressive interventions and a timely public health policy action is needed in order to curb the emerging narghile smoking epidemic.

925	Sundeep Mishra Are e-cigarettes beneficial for public health: Hume's guillotine - The debate continues?.. Indian heart journal; 2017;69:810.<br><br>**Keywords:**<br>*Electronic Nicotine Delivery Systems,Female,Humans,Male,*Public Health,*Smoking Cessation/mt [Methods],*Vaping/is [Instrumentation]<br><br>**Abstract:** Cigarette smoking is the most common preventable cause of death which has led to a search for healthier alternatives to tobacco smoke. Electronic cigarettes are devoid of many harmful constituents found in cigarette smoke and are therefore being positioned as safer alternative to tobacco smoke. The carcinogenic potential may be of lower magnitude compared with cigarette smoke, but it is still there. The use of e-cigarettes, however, is fairly new, so its long-term effects are still unknown. Moreover, they continue to posses the cardiovascular side-effects and are certainly as addicting. Thus on one hand while their substitution could lead to reduction of some side-effects of tobacco smoke, its use may be just shifting the modality of addiction and will be unlikely to lead to total abstinence from tobacco products. On the other hand there is a fear that its use could lead to loosening of the taboo currently associated with use of tobacco products and so the benefits of years of advocacy to ban tobacco products might be lost. Thus currently the scientific community is divided about role of e-cigarettes.Copyright © 2017. Published by Elsevier B.V.
943	Gunnar Saebo, Janne Scheffels Assessing notions of denormalization and renormalization of smoking in light of e-cigarette regulation.. The International journal on drug policy; 2017;49:58.<br><br>**Keywords:**<br>Attitude,*Electronic Nicotine Delivery Systems,Harm Reduction,Health Policy,Humans,Pleasure/de [Drug Effects],Prevalence,Smoking/ep [Epidemiology],Smoking/lj [Legislation & Jurisprudence],*Smoking/px [Psychology],Smoking Prevention,Tobacco,Tobacco Industry,Tobacco Smoking<br><br>**Abstract:** The rationale for 'denormalization' of smoking in tobacco policies has been challenged by the emergence of e-cigarettes and the need to regulate e-cigarette use and promotion. Our aim is to assess the research status on e-cigarettes' contribution to 'renormalization' of smoking and to clarify how renormalization of smoking can be appraised at the conceptual and empirical level. Combining conceptual analysis and narrative review, the paper brings out three dimensions of denormalization/renormalization of smoking ('unacceptability/acceptability'; 'invisibility/visibility'; 'phasing out behaviour/maintaining behaviour') and an inherent duality of the e-cigarette as a smoking-like device and a smoking alternative. These analytical dimensions are applied qualitatively to consider the literature identified by searching the Web of Science database for 'e-cigarettes AND renormalization' (and variants thereof). Theoretically, normative changes in smoking acceptability, increased visibility of e-cigarettes and use, and observations of actual use (prevalence, dual use, gateway) can all be applied to illustrate processes of renormalization. However, only acceptability measures and user measures can be said to be empirical tests of renormalization effects. Visibility measures are only based on logical assumptions of a possible renormalization; they are not in themselves indicative of any "real" renormalization effects and can just as well be understood as possible consequences of normalization of e-cigarettes. Just as a downward trend in smoking prevalence is the litmus test of whether denormalization policy works, stagnating or rising smoking prevalence should be the main empirical indicator of renormalization.Copyright © 2017 Elsevier B.V. All rights reserved.
945	Samir S Soneji, Hai-Yen Sung, Brian A Primack, John P Pierce, James D Sargent Quantifying population-level health benefits and harms of e-cigarette use in the United States.. PloS one; 2018;13:e0193328.<br><br>**Keywords:**<br>Adolescent,Adult,Child,*Electronic Nicotine Delivery Systems,Humans,Life Expectancy,Middle Aged, Models, Theoretical,Monte Carlo Method,*Smoking Cessation/sn [Statistics & Numerical Data],United

	States,Young Adult<br><br>**Abstract:** BACKGROUND: Electronic cigarettes (e-cigarettes) may help cigarette smokers quit smoking, yet they may also facilitate cigarette smoking for never-smokers. We quantify the balance of health benefits and harms associated with e-cigarette use at the population level., METHODS AND FINDINGS: Monte Carlo stochastic simulation model. Model parameters were drawn from census counts, national health and tobacco use surveys, and published literature. We calculate the expected years of life gained or lost from the impact of e-cigarette use on smoking cessation among current smokers and transition to long-term cigarette smoking among never smokers for the 2014 US population cohort., RESULTS: The model estimated that 2,070 additional current cigarette smoking adults aged 25-69 (95% CI: -42,900 to 46,200) would quit smoking in 2015 and remain continually abstinent from smoking for >=7 years through the use of e-cigarettes in 2014. The model also estimated 168,000 additional never-cigarette smoking adolescents aged 12-17 and young adults aged 18-29 (95% CI: 114,000 to 229,000), would initiate cigarette smoking in 2015 and eventually become daily cigarette smokers at age 35-39 through the use of e-cigarettes in 2014. Overall, the model estimated that e-cigarette use in 2014 would lead to 1, 510,000 years of life lost (95% CI: 920,000 to 2,160,000), assuming an optimistic 95% relative harm reduction of e-cigarette use compared to cigarette smoking. As the relative harm reduction decreased, the model estimated a greater number of years of life lost. For example, the model estimated-1,550,000 years of life lost (95% CI: -2,200,000 to -980,000) assuming an approximately 75% relative harm reduction and -1,600,000 years of life lost (95% CI: -2,290,000 to -1,030,000) assuming an approximately 50% relative harm reduction., CONCLUSIONS: Based on the existing scientific evidence related to e-cigarettes and optimistic assumptions about the relative harm of e-cigarette use compared to cigarette smoking, e-cigarette use currently represents more population-level harm than benefit. Effective national, state, and local efforts are needed to reduce e-cigarette use among youth and young adults if e-cigarettes are to confer a net population-level benefit in the future.
946	Christina Akre, Joan-Carles Suris Adolescents and young adults' perceptions of electronic cigarettes as a gateway to smoking: a qualitative study in Switzerland.. Health education research; 2017;32:448.<br><br>**Keywords:**<br>Adolescent,*Electronic Nicotine Delivery Systems/mt [Methods],Female,Humans,Male,*Perception, *Public Health,Qualitative Research,*Smoking/td [Trends],Switzerland,Young Adult<br><br>**Abstract:** Electronic cigarettes (ECs) acting as a gateway to smoking traditional cigarettes (TCs) is a growing public health concern of EC use among youths. To gather the opinions and perceptions of adolescents and young adults (AYAs) on whether and how EC can act as a gateway to smoking TC among youths. A qualitative method included 42 AYAs. Participants identified a significant risk of EC acting as a gateway to TC use, several factors contributing to this phenomenon such as a facilitated transition to smoking or its perception as a harmless toy. Participants considered an even greater gateway threat regarding very young adolescents. A minority did not identify the gateway risk and some believed that it was nicotine-dependent. This potential gateway effect brought forth several recommendations: health professionals should screen adolescents (even very young ones) for EC use and inform consumers of the potential gateway effect; this possible effect should be acknowledged to end the harmless perception many might have; there is an urge for better preventive and regulatory policies directed at protecting adolescents and children who never smoked and support those who have quit.Copyright © The Author 2017. Published by Oxford University Press. All rights reserved. For permissions, please email: journals.permissions@oup.com.
950	Teresa W Wang, Michael A Tynan, Cynthia Hallett, Laura Walpert, Maggie Hopkins, Darryl Konter, Brian A King Smoke-Free and Tobacco-Free Policies in Colleges and Universities - United States and Territories, 2017.. MMWR. Morbidity and mortality weekly report; 2018;67:686.<br><br>**Keywords:**

	District of Columbia,Humans,Micronesia,*Smoke-Free Policy,*Tobacco Smoke Pollution/lj [Legislation & Jurisprudence],Tobacco Smoke Pollution/pc [Prevention & Control],United States,*Universities/lj [Legislation & Jurisprudence],Universities/sn [Statistics & Numerical Data]<br><br>**Abstract:** Each year in the United States, cigarette smoking causes an estimated 480,000 deaths, including approximately 41,000 deaths from secondhand smoke exposure among nonsmoking adults (1). Smoke-free policies protect nonsmokers from secondhand smoke exposure, reduce the social acceptability of smoking, help in preventing youth and young adult smoking initiation, and increase smokers' efforts to quit smoking (1,2). Given that 99% of adult cigarette smokers first start smoking before age 26 years and many smokers transition to regular, daily use during young adulthood (2),* colleges and universities represent an important venue for protecting students, faculty, staff members, and guests from secondhand smoke exposure through tobacco control policies (3). To assess smoke-free and tobacco-free policies in U.S. colleges and universities, CDC and the American Nonsmokers' Rights Foundation (ANRF) determined the number of campuses nationwide that completely prohibit smoking (smoke-free) or both smoking and smokeless tobacco product use (tobacco-free) in all indoor and outdoor areas. As of November 2017, at least 2,082 U.S. college and university campuses had smoke-free policies. Among these campuses, 1,743 (83.7%) were tobacco-free; 1,658 (79.6%) specifically prohibited electronic cigarette (e-cigarette) use; and 854 (41.0%) specifically prohibited hookah smoking. Smoke-free and tobacco-free policies on college and university campuses can help reduce secondhand smoke exposure, tobacco use initiation, and the social acceptability of tobacco use (1-3).
954	Riccardo Polosa, Christopher Russell, Joel Nitzkin, Konstantinos E Farsalinos A critique of the US Surgeon General's conclusions regarding e-cigarette use among youth and young adults in the United States of America.. Harm reduction journal; 2017;14:61.<br><br>**Keywords:**<br>Adolescent,*Electronic Nicotine Delivery Systems,Harm Reduction,Humans,*Public Health/mt [Methods],*Smoking Prevention/mt [Methods],Surgeons,United States,Young Adult<br><br>**Abstract:** BACKGROUND: In December 2016, the Surgeon General published a report that concluded e-cigarette use among youth and young adults is becoming a major public health concern in the United States of America., METHODS: Re-analysis of key data sources on nicotine toxicity and prevalence of youth use of e-cigarettes cited in the Surgeon General report as the basis for its conclusions., RESULTS: Multiple years of nationally representative surveys indicate the majority of e-cigarette use among US youth is either infrequent or experimental, and negligible among never-smoking youth. The majority of the very small proportion of US youth who use e-cigarettes on a regular basis, consume nicotine-free products. The sharpest declines in US youth smoking rates have occurred as e-cigarettes have become increasingly available. Most of the evidence presented in the Surgeon General's discussion of nicotine harm is not applicable to e-cigarette use, because it relies almost exclusively on exposure to nicotine in the cigarette smoke and not to nicotine present in e-cigarette aerosol emissions. Moreover, the referenced literature describes effects in adults, not youth, and in animal models that have little relevance to real-world e-cigarette use by youth. The Surgeon General's report is an excellent reference document for the adverse outcomes due to nicotine in combination with several other toxicants present in tobacco smoke, but fails to address the risks of nicotine decoupled from tobacco smoke constituents. The report exaggerates the toxicity of propylene glycol (PG) and vegetable glycerin (VG) by focusing on experimental conditions that do not reflect use in the real-world and provides little discussion of emerging evidence that e-cigarettes may significantly reduce harm to smokers who have completely switched., CONCLUSIONS: The U.S. Surgeon General's claim that e-cigarette use among U.S. youth and young adults is an emerging public health concern does not appear to be supported by the best available evidence on the health risks of nicotine use and population survey data on prevalence of frequent e-cigarette use. Nonetheless, patterns of e-cigarettes use in youth must be constantly monitored for early detection of significant changes. The next US Surgeon General should consider the possibility that future

	generations of young Americans will be less likely to start smoking tobacco because of, not in spite of, the availability of e-cigarettes.
955	Sunday Azagba, Neill Bruce Baskerville, Kristie Foley Susceptibility to cigarette smoking among middle and high school e-cigarette users in Canada.. Preventive medicine; 2017;103:14.<br><br>**Keywords:**<br>Adolescent,Canada,*Cigarette Smoking,*Electronic Nicotine Delivery Systems/sn [Statistics & Numerical Data],Female,Humans,*Intention,Male,Schools,*Students/sn [Statistics & Numerical Data],Surveys and Questionnaires<br><br>**Abstract:** There is a growing concern that the historic reductions in tobacco consumption witnessed in the past decades may be undermined by the rapid increase in e-cigarette use. This study examined the association between e-cigarette use and future intention to smoke cigarettes among middle and high school students who had never smoked cigarettes. Data were drawn from the 2014-2015 Canadian Student Tobacco, Alcohol and Drugs Survey (n=25,637). A multivariable logistic regression model was used to examine the association between e-cigarette use and susceptibility to cigarette smoking. In addition, an inverse probability of treatment weighted regression adjustment method (doubly robust estimator), which models both the susceptibility to smoking and the probability of e-cigarette use, was conducted. About 10% of the students had ever tried an e-cigarette. There were higher rates of ever e-cigarette use among students in grades 10-12 (12.5%) than those in grades 7-9 (7.3%). Students who had ever tried an e-cigarette had higher odds of susceptibility to cigarette smoking (adjusted odds ratio=2.16, 95% confidence interval=1.80-2.58) compared to those that had never tried an e-cigarette. Current use of an e-cigarette was associated with higher odds of smoking susceptibility (adjusted odds ratio=2.02, 95% confidence interval=1.43-2.84). Similar results were obtained from the doubly robust estimation. Among students who had never smoked cigarettes, e-cigarette use was associated with a higher susceptibility to cigarette smoking.Copyright © 2017 Elsevier Inc. All rights reserved.
967	Alexandra Loukas, C Nathan Marti, Maria Cooper, Keryn E Pasch, Cheryl L Perry Exclusive e-cigarette use predicts cigarette initiation among college students.. Addictive behaviors; 2018;76:343.<br><br>**Keywords:**<br>Adolescent,Adult,*Cigarette Smoking/ep [Epidemiology],*Electronic Nicotine Delivery Systems/sn [Statistics & Numerical Data],Female,Follow-Up Studies,Humans,Male,Risk Factors,*Students/sn [Statistics & Numerical Data],Texas/ep [Epidemiology],Universities,Young Adult<br><br>**Abstract:** INTRODUCTION: An increasing body of research indicates that use of electronic nicotine delivery systems (ENDS) predicts cigarette initiation. However, no studies examine if risk for cigarette initiation varies for exclusive ENDS users versus users of ENDS and other tobacco products. This study examined if: a) cigarette-naive young adults (i.e., never cigarette users) who ever used ENDS had a greater odds of initiating cigarettes than non-ENDS users over a 1.5year period and b) the odds of cigarette initiation was consistent across exclusive ENDS users and users of ENDS and at least one tobacco product., METHODS: Participants were 2558 cigarette-naive 18-25year old (M=19.71; SD=1.61) students from 24 Texas colleges who participated in a four-wave study, with six months between each wave., RESULTS: Overall, 11% of students reported cigarette initiation by wave 4. Of those, 20.1% were wave 1 ENDS users and 8.4% were non-ENDS users. Multivariable, multilevel discrete-time hazard models indicated that wave 1 ENDS use predicted subsequent cigarette initiation, over and above the significant effects of cigarette use susceptibility, family-of-origin tobacco use, friend cigarette use, and other tobacco use. Additional findings indicated that exclusive ENDS users had a greater odds than non-users of subsequent cigarette initiation. Among users of alternative tobacco products, ENDS users did not have a greater odds of initiation than non-ENDS users., CONCLUSION: Findings extend existing research by showing that ENDS use by young adults is a specific risk factor for later cigarette initiation and not an

	extension of a constellation of existing tobacco use behaviors.Copyright © 2017 Elsevier Ltd. All rights reserved.
979	Kristin Brikmanis, Angela Petersen, Neal Doran Do personality traits related to affect regulation predict other tobacco product use among young adult non-daily smokers?.. Addictive behaviors; 2017;75:79.<br><br>**Keywords:**<br>Adolescent,Anhedonia,*Electronic Nicotine Delivery Systems/sn [Statistics & Numerical Data],Female, Humans,Impulsive Behavior,Longitudinal Studies,Male,*Personality,Regression Analysis,Risk Factors, *Self-Control/px [Psychology],*Smokers/px [Psychology],Smokers/sn [Statistics & Numerical Data], Smoking/ep [Epidemiology],*Smoking/px [Psychology],*Tobacco Products/ut [Utilization],Tobacco Smoking,Tobacco Use/ep [Epidemiology],*Tobacco Use/px [Psychology],*Water Pipe Smoking/ep [Epidemiology],Young Adult<br><br>**Abstract:** INTRODUCTION: Understanding factors that influence non-cigarette tobacco use is important given these products' prevalence and health risks. The goal of this study was to test the hypothesis that personality traits related to affect regulation would be associated with greater frequency of other tobacco product (OTP) use in a sample of young adult non-daily smokers., METHODS: Participants (n=518, 51% male) aged 18-24 were non-daily cigarette smokers recruited from the community for a longitudinal study of tobacco use. Personality characteristics (impulsivity, anhedonia, and negative affectivity) were measured at baseline, and participants reported recent tobacco use at baseline and 3, 6, and 9months later. Assessments were conducted online or via mobile phone., RESULTS: Across the 4 assessments, 33-52% of participants reported recent OTP use, with frequency of use decreasing over time. Longitudinal negative binomial regression models indicated that greater sensation seeking and lack of premeditation were associated with more frequent OTP use (ps<0.05). These effects were consistent over time., CONCLUSIONS: Findings suggest that young adult non-daily cigarette smokers with greater propensity for immediately rewarding behaviors may use OTPs more frequently. Young, non-daily cigarette smokers with high levels of sensation seeking and/or lack of premeditation may be at increased risk for harms related to OTP use and may benefit from prevention and cessation strategies that specifically address affect.Copyright © 2017 Elsevier Ltd. All rights reserved.
982	Dennis R Trinidad, John P Pierce, James D Sargent, Martha M White, David R Strong, David B Portnoy, Victoria R Green, Cassandra A Stanton, Kelvin Choi, Maansi Bansal-Travers, Yuyan Shi, Jennifer L Pearson, Annette R Kaufman, Nicolette Borek, Blair N Coleman, Andrew Hyland, Charles Carusi, Sheila Kealey, Eric Leas, Madison L Noble, Karen Messer Susceptibility to tobacco product use among youth in wave 1 of the population Assessment of tobacco and health (PATH) study.. Preventive medicine; 2017;101:8.<br><br>**Keywords:**<br>Adolescent,Adolescent Behavior,Child,Cross-Sectional Studies,*Electronic Nicotine Delivery Systems/sn [Statistics & Numerical Data],Female,Health Surveys,Humans,Male,*Smoking/ep [Epidemiology], Smoking/eh [Ethnology],*Tobacco Products/sn [Statistics & Numerical Data],United States/ep [Epidemiology],Young Adult<br><br>**Abstract:** The purpose of this study was to investigate susceptibility and ever use of tobacco products among adolescents and young adults in the US. Cross-sectional analysis of Wave 1(2013-2014) adolescent (12-17year-olds; n=13,651) and young adult (18-24year-olds; n=9112) data from the nationally-representative Population Assessment of Tobacco and Health (PATH) Study was conducted. At 12years, 5% were ever tobacco users and 36% were susceptible to use. Seventy percent were susceptible at age 17years, and the same proportion were ever users at age 22years. Susceptibility levels were comparable for cigarettes and e-cigarette (28.6% and 27.4%, respectively), followed by hookah (22.0%), pipes (17.5%), cigars (15.2%), and smokeless tobacco (9.7%). Non-Hispanic (NH) Black (Adjusted Odds Ratio [ORadj]=1.36; 95% Confidence Limit [CL], 1.18-1.56) and Hispanic (ORadj=1.34: 95% CL,1.19-1.49) adolescent never- users were more likely to be susceptible to future use of a tobacco product than NH

	Whites. Susceptibility was higher with age (15-17yrs. vs 12-14yrs.: ORadj=1.69; 95% CL, 1.55-1.85) and parental education (college graduates vs less than HS education: ORadj=1.22, 95% CL, 1.08-1.39). Compared to exclusive users of hookah, cigars, or smokeless products, larger proportions of exclusive e-cigarette ever users were also susceptible to cigarette use. Among adolescents, lower levels of ever use of tobacco products are often counterbalanced by higher levels of susceptibility for future use, which may suggest delayed initiation in some groups. Ever users of a given tobacco product were more susceptible to use other tobacco products, putting them at risk for future multiple tobacco product use.Copyright © 2017 Elsevier Inc. All rights reserved.
988	Ahmad Besaratinia, Stella Tommasi An opportune and unique research to evaluate the public health impact of electronic cigarettes.. Cancer causes & control : CCC; 2017;28:1167.<br><br>**Keywords:**<br>Biomedical Research,*Electronic Nicotine Delivery Systems,Humans,National Institute on Drug Abuse (U.S.),Neoplasms,United States<br><br>**Abstract:** In response to the growing public health concern regarding the risks or benefits of electronic cigarettes (e-cig) use relative to smoking, the National Institute on Drug Abuse (NIDA) has recently introduced the first standardized- and well- characterized e-cig device to the research community (see, https://www.drugabuse.gov/funding/supplemental-information-nida-e-cig). E-cig are promoted as safe alternatives to conventional tobacco cigarettes and/or as aides to smoking cessation. E-cig are highly popular among cigarette smokers who are unable/unwilling to quit but are willing to switch to putatively less-harmful tobacco substitutes. E-cig are also becoming increasingly popular among youth who have never experimented with combustible cigarettes. However, chemical analyses of e-cig juices (both in liquid form and after being heated into vapor) have shown that many carcinogens present in cigarette smoke are also found in a range of e-cig products. To date, the cancer-causing potential of e-cig has not been investigated in e-cig users (i.e., vapers). Use of e-cig without a prior history of smoking is currently a rare phenomenon in adults, but is increasingly common among youth. Consequently, investigating the carcinogenic potential of e-cig in nonsmoking youth provides a unique opportunity to verify the health impact of e-cig use, without the confounding effects of cigarette smoking. Within this context, the availability of the NIDA Standard Research e-cig offers a unique research opportunity with tremendous public health implications. Comparing and contrasting the cancer-causing potentials of standard vaping and smoking in youth will help determine the health risks or benefits of e-cig use relative to cigarette smoking. This information will be instrumental in making scientifically based decisions on the development and evaluation of policies and regulations on e-cig manufacture, marketing, and distribution. Ultimately, evidence-based guidelines and legislations on e-cig will help reduce the burden of tobacco-related diseases, particularly on minors and vulnerable populations.
998	Neal Doran, Kristin Brikmanis, Angela Petersen, Kevin Delucchi, Wael K Al-Delaimy, Susan Luczak, Mark Myers, David Strong Does e-cigarette use predict cigarette escalation? A longitudinal study of young adult non-daily smokers.. Preventive medicine; 2017;100:279.<br><br>**Keywords:**<br>Adolescent,California,*Electronic Nicotine Delivery Systems/sn [Statistics & Numerical Data],Female, Humans,Longitudinal Studies,Male,Smokers/px [Psychology],*Smokers/sn [Statistics & Numerical Data], *Tobacco Use/td [Trends],Young Adult<br><br>**Abstract:** Recent studies suggest that e-cigarette use among youth may be associated with increased risk of cigarette initiation. The goal of this study was to test the hypothesis that use of e-cigarettes among young adult non-daily cigarette smokers would be associated with increased cigarette consumption. Participants (n=391; 52% male) were 18-24year-old non-daily cigarette smokers recruited from across California. Cigarette and e-cigarette use were assessed online or via mobile phone every three months for one year between March 2015 and December 2016. Longitudinal negative binomial regression models

	showed that, adjusted for propensity for baseline e-cigarette use, non-daily smokers who reported more frequent use of e-cigarettes upon study entry reported greater quantity and frequency of cigarette smoking at baseline and greater increases in cigarette quantity over 12months than non-daily cigarette only smokers (ps<0.01). During the 12months of assessment, more consistent consumption of e-cigarettes was associated with greater quantity and frequency of cigarette use (ps<0.01); these effects did not vary over time. Findings suggest that among non-daily smokers, young adults who use e-cigarettes tend to smoke more cigarettes and to do so more frequently. Such individuals may be at greater risk for chronic tobacco use and dependence.Copyright © 2017 Elsevier Inc. All rights reserved.
1005	Adam M Lippert Temporal Changes in the Correlates of U.S. Adolescent Electronic Cigarette Use and Utilization in Tobacco Cessation, 2011 to 2013.. Health education & behavior : the official publication of the Society for Public Health Education; 2017;44:254.<br><br>**Keywords:**<br>Adolescent,Adolescent Behavior/px [Psychology],Cross-Sectional Studies,Electronic Nicotine Delivery Systems/is [Instrumentation],*Electronic Nicotine Delivery Systems/mt [Methods],Female,Health Surveys,Humans,Male,Prevalence,Smoking/ep [Epidemiology],*Smoking/td [Trends],*Smoking Cessation/mt [Methods]<br><br>**Abstract:** OBJECTIVE: To examine temporal changes in the correlates of experimental and current e-cigarette use and associations with tobacco quit attempts., METHOD: Repeated cross-sectional analyses of data from the 2011 (n = 17,741), 2012 (n = 23,194), and 2013 (n = 16,858) National Youth Tobacco Surveys-a nationally representative sample of U.S. middle and high school students. Respondents were asked about lifetime and past-month e-cigarette use, conventional cigarette use, and number of quit attempts made in the prior year among current smokers., RESULTS: Multinomial logistic regression models indicate higher odds of experimental or current e-cigarette use versus abstinence in the 2012 and 2013 survey years (vs. 2011). Respondents in the 2013 survey were more likely to use e-cigarettes in 2012, and they were significantly more likely to be current versus experimental users. Temporal increases in past-month e-cigarette use were uneven across demographic subgroups. Among current smokers of conventional cigarettes, fully adjusted models show that across all survey years the probability of past-month e-cigarette use was not significantly different for those who attempted to quit using tobacco in the past year from those who did not., CONCLUSIONS: Adolescent e-cigarette use has become more common between 2011 and 2013 and shows signs of becoming increasingly characterized by current rather than experimental use. Temporal patterns suggest that recent population increases were initially driven by select subgroups (e.g., Whites), with other subgroups contributing to the increase more recently (e.g., Black). Tobacco cessation has remained unrelated to current e-cigarette use over time, suggesting that e-cigarette use may be contributing to increased nicotine use.
1006	Dina M Jones, Ban A Majeed, Scott R Weaver, Kymberle Sterling, Terry F Pechacek, Michael P Eriksen Prevalence and Factors Associated with Smokeless Tobacco Use, 2014-2016.. American journal of health behavior; 2017;41:608.<br><br>**Keywords:**<br>Adolescent,Adult,Aged,Aged, 80 and over,Educational Status,*Electronic Nicotine Delivery Systems/sn [Statistics & Numerical Data],Female,Humans,Male,Middle Aged,Prevalence,*Smoking/ep [Epidemiology],*Tobacco Products/sn [Statistics & Numerical Data],*Tobacco, Smokeless/sn [Statistics & Numerical Data],United States,Young Adult<br><br>**Abstract:** OBJECTIVES: We aimed to determine whether the prevalence of current use of smokeless tobacco products (STPs) changed during 2014-2016 and examine factors associated with use among adults in the United States (US)., METHODS: Data were obtained from Tobacco Products and Risk Perceptions Surveys of probability samples representative of US adults in 2014, 2015, and 2016. Change over time in current (past 30 day) STP use was examined using pairwise comparisons of proportions and

	multivariable logistic regression. Associated factors were examined using Rao-Scott chi2 and multivariable logistic regression., RESULTS: The prevalence of current STP use was higher in 2015 (3.6%) than in 2014 (2.3%, p < .001) and 2016 (2.7%, p = .018) among US adults. In 2016, current STP use was associated with being male, under age 60, currently using hookah or e-cigarettes, and having less than a college degree. Rates of use did not vary by cigarette smoking status, race/ethnicity, income, or metropolitan statistical area (MSA)., CONCLUSIONS: The prevalence of current STP use peaked in 2015. In 2016, current STP use was more prevalent among males and adults with lower education. Continuous monitoring of STP use is needed, particularly non-cigarette tobacco product users.
1011	Andrea C Villanti, Amanda L Johnson, Bridget K Ambrose, K Michael Cummings, Cassandra A Stanton, Shyanika W Rose, Shari P Feirman, Cindy Tworek, Allison M Glasser, Jennifer L Pearson, Amy M Cohn, Kevin P Conway, Raymond S Niaura, Maansi Bansal-Travers, Andrew Hyland Flavored Tobacco Product Use in Youth and Adults: Findings From the First Wave of the PATH Study (2013-2014).. American journal of preventive medicine; 2017;53:139.<br><br>**Keywords:**<br>Adolescent,Adult,Aged,Cross-Sectional Studies,Electronic Nicotine Delivery Systems,Female,*Flavoring Agents,Humans,Male,Middle Aged,Multivariate Analysis,Prevalence,*Smoking Prevention/lj [Legislation & Jurisprudence],Tobacco Products/lj [Legislation & Jurisprudence],*Tobacco Products/ut [Utilization], Tobacco Use Disorder/ep [Epidemiology],*Tobacco Use Disorder/pc [Prevention & Control],United States,Young Adult<br><br>**Abstract:** INTRODUCTION: The 2009 Family Smoking Prevention and Tobacco Control Act banned characterizing flavors other than menthol in cigarettes but did not restrict their use in other forms of tobacco (e.g., smokeless, cigars, hookah, e-cigarettes)., METHODS: A cross-sectional analysis of Wave 1 data from 45,971 U.S. adults and youth, aged >=12 years in the Population Assessment of Tobacco and Health (PATH) Study collected in 2013-2014, was conducted in 2016. This study examined (1) the prevalence and reasons for use of flavored tobacco products; (2) the proportion of ever tobacco users reporting that their first product was flavored; and (3) correlates of current flavored tobacco product use., RESULTS: Current flavored (including menthol) tobacco product use was highest in youth (80%, aged 12-17 years); and young adult tobacco users (73%, aged 18-24 years); and lowest in older adult tobacco users aged >=65 years (29%). Flavor was a primary reason for using a given tobacco product, particularly among youth. Eighty-one percent of youth and 86% of young adult ever tobacco users reported that their first product was flavored versus 54% of adults aged >=25 years. In multivariable models, reporting that one's first tobacco product was flavored was associated with a 13% higher prevalence of current tobacco use among youth ever tobacco users and a 32% higher prevalence of current tobacco use among adult ever users., CONCLUSIONS: These results add to the evidence base that flavored tobacco products may attract young users and serve as starter products to regular tobacco use.Copyright © 2017 American Journal of Preventive Medicine. All rights reserved.
1015	Andrew Hill, Oscar M Camacho A system dynamics modelling approach to assess the impact of launching a new nicotine product on population health outcomes.. Regulatory toxicology and pharmacology : RTP; 2017;86:265.<br><br>**Keywords:**<br>Electronic Nicotine Delivery Systems,*Forecasting,Harm Reduction,Humans,*Models, Theoretical, *Nicotine/ae [Adverse Effects],*Smoking/mo [Mortality],Smoking Cessation,*Tobacco Products/ae [Adverse Effects]<br><br>**Abstract:** In 2012 the US FDA suggested the use of mathematical models to assess the impact of releasing new nicotine or tobacco products on population health outcomes. A model based on system dynamics methodology was developed to project the potential effects of a new nicotine product at a population level. A model representing traditional smoking populations (never, current and former smokers) and

	calibrated using historical data was extended to a two-product model by including electronic cigarettes use statuses. Smoking mechanisms, such as product initiation, switching, transition to dual use, and cessation, were represented as flows between smoking statuses (stocks) and the potential effect of smoking renormalisation through a feedback system. Mortality over a 50-year period (2000-2050) was the health outcome of interest, and was compared between two scenarios, with and without e-cigarettes being introduced. The results suggest that by 2050, smoking prevalence in adults was 12.4% in the core model and 9.7% (including dual users) in the counterfactual. Smoking-related mortality was 8.4% and 8.1%, respectively. The results suggested an overall beneficial effect from launching e-cigarettes and that system dynamics could be a useful approach to assess the potential population health effects of nicotine products when epidemiological data are not available.Copyright © 2017 The Authors. Published by Elsevier Inc. All rights reserved.
1017	Samir Soneji, Brian A Primack, John P Pierce, Hai-Yen Sung, James D Sargent Re: Modeling the Effects of E-cigarettes on Smoking Behavior: Implications for Future Adult Smoking Prevalence.. Epidemiology (Cambridge, Mass.); 2017;28:e1.<br><br>**Keywords:**<br>Adult,*Electronic Nicotine Delivery Systems,Humans,Prevalence,*Smoking/ep [Epidemiology],Smoking Cessation<br><br>**Abstract:**
1032	Sean Esteban McCabe, Brady T West, Phil Veliz, Carol J Boyd E-cigarette Use, Cigarette Smoking, Dual Use, and Problem Behaviors Among U.S. Adolescents: Results From a National Survey.. The Journal of adolescent health : official publication of the Society for Adolescent Medicine; 2017;61:155.<br><br>**Keywords:**<br>Achievement,Adolescent,*Adolescent Behavior,Alcohol Drinking,*Cigarette Smoking/ep [Epidemiology], Cross-Sectional Studies,*Electronic Nicotine Delivery Systems/sn [Statistics & Numerical Data],Female, Humans,Male,Marijuana Smoking,Prescription Drug Misuse,*Problem Behavior,Risk-Taking,Substance-Related Disorders,Surveys and Questionnaires<br><br>**Abstract:** PURPOSE: There is a need to obtain greater clarity regarding adolescents' e-cigarette use and the associations of use with a wider range of risk behaviors. This study examines the associations among past-month e-cigarette use only, traditional cigarette smoking only, dual use (i.e., concurrent e-cigarette use and cigarette smoking), school-related (i.e., truancy and poor academic performance) risk behaviors, and substance-related (i.e., alcohol use, binge drinking, marijuana use, illicit drug use, and nonmedical prescription drug use) risk behaviors., METHODS: Data were collected via self-administered questionnaires from a nationally representative sample of 8,696 high school seniors., RESULTS: An estimated 9.9% of U.S. high school seniors reported past-month e-cigarette use only, 6.0% reported past-month cigarette smoking only, and 7.3% reported past-month dual use. School- and substance-related risk behaviors had strong associations with past-month e-cigarette use. Adolescents who only used e-cigarettes had significantly greater odds of all school- and substance-related risk behaviors relative to nonusers. Dual users had significantly greater odds of frequent/daily e-cigarette use as well as all school- and substance-related risk behaviors relative to those who only used e-cigarettes. Finally, adolescents who engaged in frequent/daily e-cigarette use had significantly greater odds of binge drinking, marijuana use, other illicit drug use and nonmedical prescription drug use, relative to experimental e-cigarette users., CONCLUSIONS: E-cigarette use is common among U.S. adolescents, and there are robust associations between e-cigarette use and school- and substance-related risk behaviors. There is evidence that e-cigarette use clusters with risk behaviors and appears to represent a problem behavior, especially dual use of e-cigarettes and traditional cigarettes.Copyright © 2017 Society for Adolescent Health and Medicine. Published by Elsevier Inc. All rights reserved.

1047	Martin Eichler, Maria Blettner, Susanne Singer The Use of E-Cigarettes.. Deutsches Arzteblatt international; 2016;113:847.<br><br>**Keywords:**<br>Adolescent,Adult,*Electronic Nicotine Delivery Systems,Female,Germany,Humans,Male,Smoking,*Smoking Cessation,Smoking Prevention,Surveys and Questionnaires,Young Adult<br><br>**Abstract:** BACKGROUND: Electronic cigarettes (e-cigarettes) are a consumer product whose benefits and risks are currently debated. Advocates of the "tobacco harm reduction" strategy emphasize their potential as an aid to smoking cessation, while advocates of the precautionary principle emphasize their risks instead. There have been only a few studies to date on the prevalence of e-cigarette use in Germany., METHODS: In May 2016, in collaboration with Forsa, an opinion research firm, we carried out a survey among 4002 randomly chosen persons aged 14 and older, asking them about their consumption of e-cigarettes with and without nicotine, reasons for using e-cigarettes, plans for future use, estimation of danger compared to that of tobacco products, smoking behavior, and sociodemographic features., RESULTS: 1.4% of the respondents used e-cigarettes regularly, and a further 2.2% had used them regularly in the past. 11.8% had at least tried them, including 32.7% of smokers and 2.3% of persons who had never smoked. 24.5% of ex-smokers who had quit smoking after 2010 had used e-cigarettes at least once. 20.7% of the respondents considered electronic cigarettes less dangerous than conventional cigarettes, 46.3% equally dangerous, and 16.1% more dangerous. An extrapolation of these data to the general population suggests that about one million persons in Germany use e-cigarettes regularly and another 1.55 million have done so in the past., CONCLUSION: The consumption of electronic cigarettes in Germany is not very widespread, but it is not negligible either. Nearly 1 in 8 Germans has tried e-cigarettes at least once. Regular consumers of e-cigarettes are almost exclusively smokers and ex-smokers.
1068	Amy L Fairchild, Ju Sung Lee, Ronald Bayer, James Curran E-Cigarettes and the Harm-Reduction Continuum.. The New England journal of medicine; 2018;378:216.<br><br>**Keywords:**<br>Electronic Nicotine Delivery Systems/ec [Economics],Electronic Nicotine Delivery Systems/st [Standards],*Electronic Nicotine Delivery Systems,*Government Regulation,*Harm Reduction,Humans,Smoking Cessation,State Government,Taxes,Tobacco Products/ec [Economics],United States,*United States Food and Drug Administration<br><br>**Abstract:**
1072	Kenneth E Warner Frequency of E-Cigarette Use and Cigarette Smoking by American Students in 2014.. American journal of preventive medicine; 2016;51:179.<br><br>**Keywords:**<br>Adolescent,*Cigarette Smoking/ep [Epidemiology],*Electronic Nicotine Delivery Systems/sn [Statistics & Numerical Data],Female,Humans,Male,*Students/sn [Statistics & Numerical Data],Surveys and Questionnaires,United States/ep [Epidemiology]<br><br>**Abstract:** INTRODUCTION: High school students' electronic cigarette (e-cigarette) use rose rapidly in 2014, to levels higher than cigarette smoking, which declined significantly. This study assesses how frequency of e-cigarette use is associated with students' smoking status., METHODS: Using Monitoring the Future data in 2015, this study evaluated the association between students' smoking and frequency of 30-day e-cigarette use in 2014, focusing on high school seniors. Previous research has considered only whether e-cigarettes were used at all during the past month., RESULTS: Non-smokers were far less likely than smokers to have used an e-cigarette ($p<0.001$). E-cigarette use frequency rose with the amount of ever smoking ($p<0.001$). However, among current smokers, there was no difference in e-cigarette use by

	very light smokers (<1 cigarette/day); light smokers (1-5 cigarettes/day); and heavy smokers (>=1/2 pack/day) (p=0.99). Because most students have never smoked, never smokers constituted 25% of all seniors who used an e-cigarette. Among tenth- and eighth-graders, 43% and 48% of past-month e-cigarette users had never smoked., CONCLUSIONS: Non-smoking high school students are highly unlikely to use e-cigarettes; among those who do, most used them only on 1-2 of the past 30 days. By contrast, current smokers are likely to use e-cigarettes and on many more days. It is unclear whether students' e-cigarette use represents short-term experimentation or future sustained use, and whether it will eventually increase or decrease youth smoking and nicotine addiction. More sophisticated research methods, employing better data, will be essential to unravel the mystery that is the e-cigarette phenomenon.Copyright © 2016 American Journal of Preventive Medicine. Published by Elsevier Inc. All rights reserved.
1073	Karen Arane, Ran D Goldman Electronic cigarettes and adolescents.. Canadian family physician Medecin de famille canadien; 2016;62:897.<br><br>**Keywords:**<br>Adolescent,Canada/ep [Epidemiology],Counseling/mt [Methods],*Electronic Nicotine Delivery Systems/sn [Statistics & Numerical Data],Family Practice/mt [Methods],Female,Humans,Male, Smoking/ae [Adverse Effects],*Smoking/ep [Epidemiology],Smoking Prevention/mt [Methods],United States/ep [Epidemiology],*Vaping/ae [Adverse Effects],Vaping/sn [Statistics & Numerical Data]<br><br>**Abstract:** Question I see in my office an increased number of adolescents who use electronic cigarettes (e-cigarettes). Should I encourage adolescents to choose e-cigarettes over regular cigarettes if they decide to smoke? Are e-cigarettes less harmful and a potential smoking cessation method for adolescents? Answer While e-cigarettes do not have carcinogenic tobacco, most contain nicotine, which not only leads to addiction, but can also impair brain development and cognitive function in youth. Recent studies have also shown that adolescents who use e-cigarettes are more likely to begin smoking tobacco cigarettes. It is therefore essential that physicians explain to adolescents the risks and health concerns e-cigarettes present, and implement measures to prevent or cease e-cigarette use.Copyright© the College of Family Physicians of Canada.
1082	Konstantinos E Farsalinos, Konstantinos Poulas, Vassilis Voudris, Jacques Le Houezec Electronic cigarette use in the European Union: analysis of a representative sample of 27460 Europeans from 28 countries.. Addiction (Abingdon, England); 2016;111:2032.<br><br>**Keywords:**<br>Adolescent,Adult,Age Distribution,Aged,Cross-Sectional Studies,*Electronic Nicotine Delivery Systems/sn [Statistics & Numerical Data],European Union,Female,Humans,Male,Middle Aged,Smoking Cessation/sn [Statistics & Numerical Data],Social Class,Young Adult<br><br>**Abstract:** AIMS: To assess prevalence of electronic cigarette (e-cigarette) use, reported changes in smoking status due to e-cigarette use and correlates of e-cigarette use in the European Union (EU) member states in 2014., DESIGN: Cross-sectional survey of EU citizens representative of the population (Special Eurobarometer 429)., SETTING: All 28 Member States of the EU., PARTICIPANTS: A total of 27460 EU citizens aged >= 15 years (after excluding those who responded 'Do not know' to the questions about smoking status and e-cigarette use)., MEASUREMENTS: Descriptive analysis [%, 95% confidence interval (CI)] of e-cigarette use prevalence (current use, past use and past experimentation) according to smoking status, self-reported changes in smoking status according to patterns of e-cigarette use and logistic regression analysis to examine correlates of e-cigarette use, especially socio-demographic factors and smoking status., FINDINGS: Ever e-cigarette use was reported by 31.1% (95% CI = 30.0-32.2%) of current smokers, 10.8% (95% CI = 10.0-11.7%) of former smokers and 2.3% (95% CI = 2.1-2.6%) of never smokers. Past experimentation [7.2% (95% CI = 6.9-7.5%)] was more common than current [1.8% (95% CI = 1.6-1.9%)] and past use [2.6% (95% CI = 2.4-2.8%)]. Extrapolated to the whole population, approximately 48.5

	million EU citizens were ever e-cigarette users, with 76.8% using nicotine-containing e-cigarettes. An estimated 6.1 and 9.2 million EU citizens had quit and reduced smoking with the help of e-cigarettes, respectively. Initiation with e-cigarettes was reported by 0.8% (95% CI = 0.6-0.9%) of participants who reported ever use of any tobacco-related product. Only 1.3% (95% CI = 1.1-1.5%) of never smokers used nicotine-containing e-cigarettes, with 0.09% (95% CI = 0.04-0.14%) reporting daily nicotine use. Smoking cessation with the help of e-cigarettes was reported by 35.1% (95% CI = 30.7-39.5%) of current e-cigarette users, while a further 32.2% (95% CI = 29.9-36.5%) reported smoking reduction. Being current [odds ratio (OR) = 21.23, 95% CI = 18.32-24.59) or former smokers (OR = 6.49, 95% CI = 5.49-7.67) were the strongest correlates of ever e-cigarette use., CONCLUSIONS: E-cigarette use in the European Union appears to be largely confined to current or former smokers, while current use and nicotine use by people who have never smoked is rare. More than one-third of current e-cigarette users polled reported smoking cessation and reduction.Copyright © 2016 Society for the Study of Addiction.
1101	Jimi Huh, Adam M Leventhal Progression of Poly-tobacco Product Use Patterns in Adolescents.. American journal of preventive medicine; 2016;51:513.<br><br>**Keywords:**<br>Adolescent,Adolescent Behavior,California/ep [Epidemiology],Female,Humans,Longitudinal Studies,Male, Smoking/ep [Epidemiology],*Smoking/px [Psychology]<br><br>**Abstract:** INTRODUCTION: Diverse patterns of adolescent use and poly-use of tobacco products other than conventional cigarettes are emerging. Data characterizing common patterns of youth tobacco product use and typical transitions among patterns may inform tobacco control policy and prevention. This study identified common patterns of use and poly-use of five popular tobacco products (i.e., conventional cigarettes, electronic [e-]cigarettes, hookah, blunts, and cigars) and progression among patterns across time among ninth-graders using latent transition analysis (analyses conducted in 2015)., METHODS: Data were from a longitudinal cohort study of ninth-grade students enrolled in ten public high schools in California (N=3,304; 46.6% male; 48.3% Hispanic; mean age, 14.58 [SD=0.40] years), involving a baseline (2013) and 6-month follow-up (2014). Past 6-month any use of the five tobacco products was assessed., RESULTS: Poly-use (two or more products) constituted 42% and 50% of tobacco-using teens at baseline and follow-up, respectively. Three common patterns were identified, which reflected successfully greater degrees of low, intermediate, and high diversity of tobacco product use: non-users (baseline prevalence, 0.75; follow-up prevalence, 0.64); e-cigarette/hookah users only (prevalence, 0.21, 0.27); and poly-tobacco product users of all five products (prevalence, 0.04, 0.09). Most typical transitions involved progressing to the next more diverse pattern (non-user->e-cigarette/hookah user [probability=0.13] and e-cigarette/hookah user->poly-tobacco product user [probability=0.19]). Transition from one of the user patterns to non-user status was rare (probability<=0.08)., CONCLUSIONS: Adolescent poly-tobacco use is common. E-cigarette and hookah use may reflect an intermediate pattern of tobacco product use progression along a continuum of poly-product use diversity.Copyright © 2016 American Journal of Preventive Medicine. Published by Elsevier Inc. All rights reserved.
1105	Elen de Lacy, Adam Fletcher, Gillian Hewitt, Simon Murphy, Graham Moore Cross-sectional study examining the prevalence, correlates and sequencing of electronic cigarette and tobacco use among 11-16-year olds in schools in Wales.. BMJ open; 2017;7:e012784.<br><br>**Keywords:**<br>Adolescent,*Amphetamine-Related Disorders/ep [Epidemiology],*Binge Drinking/ep [Epidemiology], Child,*Cigarette Smoking/ep [Epidemiology],Cross-Sectional Studies,Female,Humans,Male,*Marijuana Smoking/ep [Epidemiology],Nitrous Oxide,Prevalence,Risk Factors,Schools,Sex Factors,*Students/sn [Statistics & Numerical Data],*Vaping/sn [Statistics & Numerical Data],Wales/ep [Epidemiology]<br><br>**Abstract:** OBJECTIVES: To examine the prevalence and frequency of electronic (e)-cigarette use among young people in Wales, associations with socio-demographic characteristics, smoking and other

	substances and the sequencing of e-cigarette and tobacco use., DESIGN: A cross-sectional survey of school students in Wales undertaken in 2015., SETTING: 87 secondary schools in Wales., PARTICIPANTS: Students aged 11-16 (n=32 479)., RESULTS: Overall, students were nearly twice as likely to report ever using e-cigarettes (18.5%) as smoking tobacco (10.5%). Use of e-cigarettes at least weekly was 2.7% in the whole sample, rising to 5.7% among those aged 15-16. Almost half (41.8%) of daily smokers reported being regular e-cigarette users. Regular e-cigarette use was more prevalent among current cannabis users (relative risk ratio (RRR)=41.82; 95% CI 33.48 to 52.25)), binge drinkers (RRR=47.88; 95% CI 35.77 to 64.11), users of mephedrone (RRR=32.38; 95% CI 23.05 to 45.52) and laughing gas users (RRR=3.71; 95% CI 3.04 to 4.51). Multivariate analysis combining demographics and smoking status showed that only gender (being male) and tobacco use independently predicted regular use of e-cigarettes (p<0.001). Among weekly smokers who had tried tobacco and e-cigarettes (n=877), the vast majority reported that they tried tobacco before using an e-cigarette (n=727; 82.9%)., CONCLUSIONS: Since 2013, youth experimentation with e-cigarettes has grown rapidly in Wales and is now almost twice as common as experimentation with tobacco. Regular use has almost doubled, and is increasing among never and non-smokers. These data suggest that e-cigarette use among youth is an emerging public health issue, even though there remains no evidence that it represents a new pathway into smoking. Mixed methods longitudinal research is needed to explore why young people use e-cigarettes, and to develop interventions to prevent further increases in use.Copyright Published by the BMJ Publishing Group Limited. For permission to use (where not already granted under a licence) please go to http://www.bmj.com/company/products-services/rights-and-licensing/.
1107	Sherazade Kinouani, Philippe Castera, Catherine Laporte, Francois Petregne, Bernard Gay Factors and motivations associated with use of e-cigarette among primary care patients in a prospective cohort study: e-TAC study protocol.. BMJ open; 2016;6:e011488.<br><br>**Keywords:**<br>Adolescent,Adult,Aged,Aged, 80 and over,*Electronic Nicotine Delivery Systems,Female,France/ep [Epidemiology],Humans,Logistic Models,Male,Middle Aged,*Motivation,Multivariate Analysis,Primary Health Care,Proportional Hazards Models,Prospective Studies,Research Design,*Smoking/ep [Epidemiology],Smoking Cessation/mt [Methods],*Socioeconomic Factors,Surveys and Questionnaires, Young Adult<br><br>**Abstract:** INTRODUCTION: While the relationship between electronic cigarette use and smoking has often been studied, the association between electronic cigarette use and socioeconomic factors has received less attention. This is a study protocol aiming to describe the relationship between the consumption of psychoactive products (in particular: smoking) or some socioeconomic factors and the evolution of the use of electronic cigarette in primary healthcare over 1 year., METHODS AND ANALYSIS: Electronic cigarette, Tobacco, Alcohol and Cannabis (e-TAC) is a prospective multisite cohort study, including 473 patients at baseline and carrying out in general practices in the Aquitaine area (France). The volunteer patients participated in the study regardless of their initial reason for consultation. They filled out a self-administered questionnaire at baseline and will also do so after 12 months by phone, email or letter. The study will focus on the factors that explain the experimentation with or the current use of the electronic cigarette, as well as factors associated with their evolutions over time using multivariate logistic regression modelling or Cox regression modelling., ETHICS AND DISSEMINATION: This study received ethical approval from the University of Bordeaux Committee for the protection of persons. It was also approved by the National Commission for Data Processing and Freedoms. Findings will be submitted for publication in peer-reviewed journals and we will disseminate them by presentations at national or international conferences., TRIAL REGISTRATION NUMBER: RCB: 2015-A00778-41; Pre-results.Copyright Published by the BMJ Publishing Group Limited. For permission to use (where not already granted under a licence) please go to http://www.bmj.com/company/products-services/rights-and-licensing/
1108	Titus J Brinker, Julia Holzapfel, Tanja G Baudson, Katharina Sies, Lena Jakob, Hannah Maria Baumert, Marlene Heckl, Ana Cirac, Janina L Suhre, Verena Mathes, Fabian N Fries, Hannah Spielmann, Nancy

	Rigotti, Werner Seeger, Felix Herth, David A Groneberg, Tobias Raupach, Henning Gall, Claudia Bauer, Pat Marek, Anil Batra, Chase H Harrison, Lava Taha, Andreas Owczarek, Felix J Hofmann, Roger Thomas, Ute Mons, Michael Kreuter Photoaging smartphone app promoting poster campaign to reduce smoking prevalence in secondary schools: the Smokerface Randomized Trial: design and baseline characteristics.. BMJ open; 2016;6:e014288.<br><br>**Keywords:**<br>Adolescent,*Aging/de [Drug Effects],Child,Female,Germany/ep [Epidemiology],Humans,Male,*Posters as Topic,Prevalence,Prospective Studies,*School Health Services,*Smartphone,*Smoking/ep [Epidemiology],*Smoking Cessation/mt [Methods],*Software<br><br>**Abstract:** INTRODUCTION: Smoking is the largest cause of preventable death globally. Most smokers smoke their first cigarette in early adolescence. We took advantage of the widespread availability of mobile phones and adolescents' interest in appearance to develop a free photoaging app which is promoted via a poster campaign in secondary schools. This study aims to evaluate its effectiveness regarding smoking prevalence and students' attitudes towards smoking., METHODS AND ANALYSIS: A randomised controlled trial is conducted with 9851 students of both genders with an average age of 12 years in grades 6 and 7 of 126 secondary schools in Germany. At present, cigarette smoking prevalence in our sample is 4.7%, with 4.6% of the students currently using e-cigarettes (1.6% use both). The prospective experimental study design includes measurements at baseline and at 6, 12 and 24 months postintervention via a questionnaire plus a random cotinine saliva sample at 24 months postintervention. The study groups consist of randomised schools receiving the Smokerface poster campaign and control schools with comparable baseline data (no intervention). The primary end point is the difference of change in smoking prevalence in the intervention group versus the difference in the control group at 24 months follow-up. Longitudinal changes in smoking-related attitudes, the number of new smokers and quitters and the change in the number of never-smokers will be compared between the two groups as secondary outcomes., ETHICS AND DISSEMINATION: Ethical approval was obtained from the ethics committee of the University of Giesen and the ministries of cultural affairs, both in Germany. Results will be disseminated at conferences, in peer-reviewed journals, on our websites and throughout the multinational Education Against Tobacco network., TRIAL REGISTRATION NUMBER: NCT02544360, Pre-results.Copyright Published by the BMJ Publishing Group Limited. For permission to use (where not already granted under a licence) please go to http://www.bmj.com/company/products-services/rights-and-licensing/.
1120	Jessica L Barrington-Trimis, Kiros Berhane, Jennifer B Unger, Tess Boley Cruz, Robert Urman, Chih Ping Chou, Steve Howland, Kejia Wang, Mary Ann Pentz, Tamika D Gilreath, Jimi Huh, Adam M Leventhal, Jonathan M Samet, Rob McConnell The E-cigarette Social Environment, E-cigarette Use, and Susceptibility to Cigarette Smoking.. The Journal of adolescent health : official publication of the Society for Adolescent Medicine; 2016;59:75.<br><br>**Keywords:**<br>Adolescent,*Adolescent Behavior/px [Psychology],Cross-Sectional Studies,*Electronic Nicotine Delivery Systems,Female,Humans,Male,*Peer Influence,Risk,*Smoking/td [Trends],*Social Environment, Students/sn [Statistics & Numerical Data],Surveys and Questionnaires,Tobacco Products/ut [Utilization], United States<br><br>**Abstract:** PURPOSE: One concern regarding the recent increase in adolescent e-cigarette use is the possibility that electronic (e-) cigarettes may be used by those who might not otherwise have used cigarettes, and that dual use, or transition to cigarette use alone, may follow., METHODS: Questionnaire data were obtained in 2014 from 11th/12th grade students attending schools in 12 communities included in the Southern California Children's Health Study. We evaluated the cross-sectional association between e-cigarette use, the social environment (family and friends' use and approval of e-cigarettes and

	cigarettes), and susceptibility to future cigarette use among never cigarette smokers (N = 1,694), using previously validated measures based on reported absence of a definitive commitment not to smoke., RESULTS: Among adolescents who had never used cigarettes, 31.8% of past e-cigarette users and 34.6% of current (past-30-day) e-cigarette users indicated susceptibility to cigarette use, compared with 21.0% of never e-cigarette users. The odds of indicating susceptibility to cigarette use were two times higher for current e-cigarette users compared with never users (odds ratio = 1.97; 95% confidence interval: 1.21-3.22). A social environment favorable to e-cigarettes (friends' use of and positive attitudes toward the use of e-cigarettes) was also associated with greater likelihood of susceptibility to cigarette use, independent of an individual's e-cigarette use., CONCLUSIONS: E-cigarette use in adolescence, and a pro-e-cigarette social environment, may put adolescents at risk for future use of cigarettes. E-cigarettes may contribute to subsequent cigarette use via nicotine addiction or social normalization of smoking behaviors.Copyright © 2016 Society for Adolescent Health and Medicine. Published by Elsevier Inc. All rights reserved.
1124	Sean Esteban McCabe, Phil Veliz, Vita V McCabe, Carol J Boyd Smoking behaviors and intentions among current e-cigarette users, cigarette smokers, and dual users: A national survey of U.S. high school seniors.. Preventive medicine; 2017;99:228.<br><br>**Keywords:**<br>Adolescent,*Cigarette Smoking,*Electronic Nicotine Delivery Systems/sn [Statistics & Numerical Data], Female,Humans,*Intention,Male,*Smokers/sn [Statistics & Numerical Data],*Smoking/px [Psychology], Surveys and Questionnaires,United States<br><br>**Abstract:** E-cigarette use among adolescents has increased significantly in recent years, but it remains unclear whether cigarette smoking behaviors and intentions for future cigarette smoking differ among current (i.e., 30-day) non-users, only e-cigarette users, only cigarette smokers, and dual users. A nationally representative sample of 4385 U.S. high school seniors were surveyed during the spring of their senior year via self-administered questionnaires in 2014. An estimated 9.6% of U.S. high school seniors reported current e-cigarette use only, 6.3% reported current cigarette smoking only, and 7.2% reported current dual use of e-cigarettes and cigarette smoking. There were no significant differences between current only cigarette smokers and dual users in the odds of early onset of cigarette smoking, daily cigarette smoking, intentions for future cigarette smoking, friends' cigarette smoking behaviors, attempts to quit cigarette smoking, or the inability to quit cigarette smoking. Adolescents who only used e-cigarettes had higher odds of intentions for future cigarette smoking in the next 5years (AOR=2.57, 95% CI: 1.21-5.24) than current non-users. Dual users and only cigarette smokers had higher odds of cigarette smoking behaviors and intentions for future cigarette smoking than non-users or only e-cigarette users. Adolescents who engage in current dual use have cigarette smoking behaviors and intentions for future cigarette smoking that more closely resemble cigarette smokers than e-cigarette users. Adolescents who only use e-cigarettes have higher intentions to engage in future cigarette smoking relative to their peers who do not engage in e-cigarette use or cigarette smoking.Copyright © 2017 Elsevier Inc. All rights reserved.
1126	David T Levy, Ron Borland, Andrea C Villanti, Raymond Niaura, Zhe Yuan, Yian Zhang, Rafael Meza, Theodore R Holford, Geoffrey T Fong, K Michael Cummings, David B Abrams The Application of a Decision-Theoretic Model to Estimate the Public Health Impact of Vaporized Nicotine Product Initiation in the United States.. Nicotine & tobacco research : official journal of the Society for Research on Nicotine and Tobacco; 2017;19:149.<br><br>**Keywords:**<br>Adolescent,Adult,Aged,Aged, 80 and over,*Decision Support Techniques,Decision Trees,*Electronic Nicotine Delivery Systems/ut [Utilization],Female,Humans,Male,Middle Aged,*Models, Theoretical, Prevalence,*Public Health,Smoking/mo [Mortality],*Smoking Cessation/mt [Methods],*Smoking Prevention,United States/ep [Epidemiology],Young Adult

	**Abstract:** INTRODUCTION: The public health impact of vaporized nicotine products (VNPs) such as e-cigarettes is unknown at this time. VNP uptake may encourage or deflect progression to cigarette smoking in those who would not have otherwise smoked, thereby undermining or accelerating reductions in smoking prevalence seen in recent years., METHODS: The public health impact of VNP use are modeled in terms of how it alters smoking patterns among those who would have otherwise smoked cigarettes and among those who would not have otherwise smoked cigarettes in the absence of VNPs. The model incorporates transitions from trial to established VNP use, transitions to exclusive VNP and dual use, and the effects of cessation at later ages. Public health impact on deaths and life years lost is estimated for a recent birth cohort incorporating evidence-informed parameter estimates., RESULTS: Based on current use patterns and conservative assumptions, we project a reduction of 21% in smoking-attributable deaths and of 20% in life years lost as a result of VNP use by the 1997 US birth cohort compared to a scenario without VNPs. In sensitivity analysis, health gains from VNP use are especially sensitive to VNP risks and VNP use rates among those likely to smoke cigarettes., CONCLUSIONS: Under most plausible scenarios, VNP use generally has a positive public health impact. However, very high VNP use rates could result in net harms. More accurate projections of VNP impacts will require better longitudinal measures of transitions into and out of VNP, cigarette and dual use., IMPLICATIONS: Previous models of VNP use do not incorporate whether youth and young adults initiating VNP would have been likely to have been a smoker in the absence of VNPs. This study provides a decision-theoretic model of VNP use in a young cohort that incorporates tendencies toward smoking and shows that, under most plausible scenarios, VNP use yields public health gains. The model makes explicit the type of surveillance information needed to better estimate the effect of new products and thereby inform public policy.Copyright © The Author 2016. Published by Oxford University Press on behalf of the Society for Research on Nicotine and Tobacco. All rights reserved. For permissions, please e-mail: journals.permissions@oup.com.
1133	Thomas A Wills, James D Sargent, Frederick X Gibbons, Ian Pagano, Rebecca Schweitzer E-cigarette use is differentially related to smoking onset among lower risk adolescents.. Tobacco control; 2016;26:534.<br><br>**Keywords:**<br>Adolescent,*Adolescent Behavior,*Electronic Nicotine Delivery Systems,Female,Humans,Male,Risk, Schools,*Smoking,Students,Surveys and Questionnaires,Tobacco Products<br><br>**Abstract:** OBJECTIVE: E-cigarette use has been linked to onset of cigarette smoking among adolescents, but some commentators have suggested that this simply reflects high-risk adolescents being more likely to use e-cigarettes and to smoke. We tested whether the effect of e-cigarette use for smoking onset differs for youth who are lower versus higher on propensity to smoke., METHODS: School-based survey with a longitudinal sample of 1136 students (9th-11th graders, mean age 14.7 years) in Hawaii, initially surveyed in 2013 (T1) and followed up 1 year later (T2). We assessed e-cigarette use, propensity to smoke based on 3 psychosocial factors known to predict smoking (rebelliousness, parental support and willingness to smoke), and cigarette smoking status. Analyses based on T1 never-smokers tested the relation of T1 e-cigarette use to T2 smoking status for participants lower versus higher on T1 propensity to smoke., RESULTS: The relation between T1 e-cigarette use and T2 smoking onset was stronger among participants with lower levels of rebelliousness and willingness and higher levels of parental support. A multiple logistic regression analysis with T2 smoking as the criterion tested the cross-product of T1 e-cigarette use and T1 smoking propensity score; the interaction (OR=0.88, p=0.01) indicated a significantly larger effect for smoking onset among lower risk youth., CONCLUSIONS: The results indicate e-cigarette use is a risk factor for smoking onset, not just a marker of high risk for smoking. This study provides evidence that e-cigarettes are recruiting lower risk adolescents to smoking, which has public health implications.Copyright Published by the BMJ Publishing Group Limited. For permission to use (where not already granted under a licence) please go to http://www.bmj.com/company/products-services/rights-and-licensing/.

1157	Erika Westling, Julie C Rusby, Ryann Crowley, John M Light Electronic Cigarette Use by Youth: Prevalence, Correlates, and Use Trajectories From Middle to High School.. The Journal of adolescent health : official publication of the Society for Adolescent Medicine; 2017;60:660.<br><br>**Keywords:**<br>Adolescent,*Adolescent Behavior/px [Psychology],*Electronic Nicotine Delivery Systems/sn [Statistics & Numerical Data],Female,Humans,Internet,Male,Oregon,Prevalence,*Schools,*Students/sn [Statistics & Numerical Data],Surveys and Questionnaires<br><br>**Abstract:** PURPOSE: The aim of this study was to examine the use of electronic cigarettes (e-cigarettes) among adolescents over time, including correlates of lifetime use by eighth grade and trajectories of current use across ninth grade., METHODS: Participants (N = 1,091) from seven school districts in Oregon, United States, completed four self-report surveys on substance use, from the spring of eighth grade (M age = 14.4 years old; standard deviation = .50) through the spring of ninth grade., RESULTS: Overall, 27.7% of eighth graders had used e-cigarettes, and 16.8% were current e-cigarette users (used in the past 30 days); use did not significantly differ by gender or ethnicity. Correlates of e-cigarette lifetime use by eighth grade included lifetime and current use of marijuana, alcohol, cigarettes, and chewing tobacco. Five percent of students were "accelerators," on average using e-cigarettes 14 of the last 30 days in eighth grade, increasing to daily use (30/30 days) by the end of ninth grade. Across all substances, those in the accelerator group were more likely to have reported lifetime substance use by eighth grade and current substance use in ninth grade, compared to the "infrequent/no use" group., CONCLUSIONS: A sizeable proportion of young adolescents are using e-cigarettes, and e-cigarette use is highly correlated with use of other substances, including marijuana. Adolescents who progress to daily e-cigarette use in high school are more likely to use other substances compared to low or nonusers. E-cigarettes may be a relatively new addition to a constellation of substances being actively used by a segment of the youth population.Copyright © 2016 Society for Adolescent Health and Medicine. Published by Elsevier Inc. All rights reserved.
1163	Paula Lozano, Inti Barrientos-Gutierrez, Edna Arillo-Santillan, Paola Morello, Raul Mejia, James D Sargent, James F Thrasher A longitudinal study of electronic cigarette use and onset of conventional cigarette smoking and marijuana use among Mexican adolescents.. Drug and alcohol dependence; 2017;180:427.<br><br>**Keywords:**<br>Adolescent,*Cigarette Smoking/ep [Epidemiology],*Electronic Nicotine Delivery Systems/sn [Statistics & Numerical Data],Humans,Longitudinal Studies,*Marijuana Abuse/px [Psychology],Mexican Americans, Mexico,*Students/sn [Statistics & Numerical Data],Surveys and Questionnaires<br><br>**Abstract:** PURPOSE: This study evaluated whether e-cigarette trial among Mexican adolescents increased the likelihood of trial and use of conventional cigarettes or marijuana use at follow-up., METHOD: A school-based longitudinal survey was conducted in 60 public middle schools from the three largest cities in Mexico. Students (12-13years old) were surveyed in 2015 and followed up 20 months later (n=6574). Generalized estimating equations models were used to evaluate the association between e-cigarette trial at baseline and conventional cigarettes smoking and marijuana use at follow-up., RESULT: Adolescents who had tried e-cigarettes (but not cigarettes) at baseline were more likely to have tried conventional cigarettes at followup compared to adolescents who had tried neither e-cigarettes nor cigarettes (43% vs. 24%, respectively; RR 1.41, 95% CI 1.18-1.70). We also found that adolescents who had tried both conventional cigarettes and e-cigarettes at baseline were more likely to have tried marijuana at follow-up compared to adolescents who had tried neither tobacco product (20% vs. 4%, respectively; RR 2.67, 95% CI 1.78-4.02). Trial of only e-cigarettes was not independently associated with marijuana use at followup., CONCLUSIONS: Adolescents who had tried e-cigarettes were more likely to have tried conventional cigarettes and marijuana 20 months later. Although e-cigarettes have been banned in Mexico, it is likely

	that additional policies and public health campaigns are needed to reduce adolescent use of e-cigarettes and its consequences.Copyright © 2017 Elsevier B.V. All rights reserved.
1170	Stephen Miller, James Pike, Alan W Stacy, Bin Xie, Susan L Ames Negative affect in at-risk youth: Outcome expectancies mediate relations with both regular and electronic cigarette use.. Psychology of addictive behaviors : journal of the Society of Psychologists in Addictive Behaviors; 2017;31:457.<br><br>**Keywords:**<br>Adolescent,*Affect/ph [Physiology],*Electronic Nicotine Delivery Systems,Female,Humans,Male,Schools, Smoking/ep [Epidemiology],*Smoking/px [Psychology],Smoking Cessation/mt [Methods],*Students/px [Psychology],United States,Young Adult<br><br>**Abstract:** Despite the general trend of declining use of traditional cigarettes among young adults in the United States, alternative high school students continue to smoke cigarettes and electronic cigarettes at rates much higher than do students attending regular high schools. Challenging life circumstances leading to elevated levels of negative affect may account for increased smoking behavior in this population. Further, a belief in the negative affect-reducing qualities of nicotine may mediate this effect. The current study tested the hypothesis that negative reinforcing outcome expectancies mediate the relationship between negative affect on smoking susceptibility in nonusers, smoking frequency in users, and smoking experimentation in the overall sample. Results support the hypothesis that negative affect in alternative high school students is correlated with smoking experimentation, smoking willingness, and smoking frequency and that the relationship between negative affect and smoking behavior outcomes is mediated by negative reinforcing outcome expectancies (i.e., beliefs in the negative affect-reducing effects of smoking). This finding was supported for both cigarettes and electronic cigarettes and coincides with a rapid increase in the number of high school students nationally who have experimented with electronic cigarettes. Future antismoking initiatives directed at at-risk youth should consider integrating healthier negative affect reduction techniques to counter the use of nicotine products. (PsycINFO Database RecordCopyright (c) 2017 APA, all rights reserved).
1184	Linda Bauld, Anne Marie MacKintosh, Brian Eastwood, Allison Ford, Graham Moore, Martin Dockrell, Deborah Arnott, Hazel Cheeseman, Ann McNeill Young People's Use of E-Cigarettes across the United Kingdom: Findings from Five Surveys 2015-2017.. International journal of environmental research and public health; 2017;14:<br><br>**Keywords:**<br>Adolescent,Adolescent Health,Child,*Electronic Nicotine Delivery Systems/sn [Statistics & Numerical Data],Female,Humans,Male,Prevalence,School Health Services,*Smoking/ep [Epidemiology],Surveys and Questionnaires,United Kingdom/ep [Epidemiology]<br><br>**Abstract:** Concern has been expressed about the use of e-cigarettes among young people. Our study reported e-cigarette and tobacco cigarette ever and regular use among 11-16 year olds across the UK. Data came from five large scale surveys with different designs and sampling strategies conducted between 2015 and 2017: The Youth Tobacco Policy Survey; the Schools Health Research Network Wales survey; two Action on Smoking and Health (ASH) Smokefree Great Britain-Youth Surveys; and the Scottish Schools Adolescent Lifestyle and Substance Use Survey. Cumulatively these surveys collected data from over 60,000 young people. For 2015/16 data for 11-16 year olds: ever smoking ranged from 11% to 20%; regular (at least weekly) smoking between 1% and 4%; ever use of e-cigarettes 7% to 18%; regular (at least weekly) use 1% to 3%; among never smokers, ever e-cigarette use ranged from 4% to 10% with regular use between 0.1% and 0.5%; among regular smokers, ever e-cigarette use ranged from 67% to 92% and regular use 7% to 38%. ASH surveys showed a rise in the prevalence of ever use of e-cigarettes from 7% (2016) to 11% (2017) but prevalence of regular use did not change remaining at 1%. In summary, surveys across the UK show a consistent pattern: most e-cigarette experimentation does not turn into regular use, and levels of regular use in young people who have never smoked remain very low.

1185	Eric K Soule, Sarah F Maloney, Mignonne C Guy, Thomas Eissenberg, Pebbles Fagan User identified positive outcome expectancies of electronic cigarette use: A concept mapping study.. Psychology of addictive behaviors : journal of the Society of Psychologists in Addictive Behaviors; 2017;31:343.<br><br>**Keywords:**<br>Adolescent,Adult,Affect,*Electronic Nicotine Delivery Systems,Female,Humans,Male,Middle Aged, Pleasure,*Vaping/px [Psychology],Young Adult<br><br>**Abstract:** Electronic cigarette (ECIG) use is growing in popularity, but little is known about the perceived positive outcomes of ECIG use. This study used concept mapping (CM) to examine positive ECIG outcome expectancies. Sixty-three past-30-day ECIG users (38.1% female) between the ages of 18 and 64 (M = 37.8, SD = 13.3) completed a CM module. In an online program, participants provided statements that completed a prompt: "A specific positive, enjoyable, or exciting effect (i.e., physical or psychological) that I have experienced WHILE USING or IMMEDIATELY AFTER USING an electronic cigarette/electronic vaping device is. . . ." Participants (n = 35) sorted 123 statements into "piles" of similar content and rated (n = 43) each statement on a 7-point scale (1 = Definitely NOT a positive effect to 7 = Definitely a positive effect). A cluster map was created using data from the sorting task, and analysis indicated a 7 cluster model of positive ECIG use outcome expectancies: Therapeutic/Affect Regulation, High/Euphoria, Sensation Enjoyment, Perceived Health Effects, Benefits of Decreased Cigarette Use, Convenience, and Social Impacts. The Perceived Health Effects cluster was rated highest, although all mean ratings were greater than 4.69. Mean cluster ratings were compared, and females, younger adults, past-30-day cigarette smokers, users of more "advanced" ECIG devices, and nonlifetime (less than 100 lifetime cigarettes) participants rated certain clusters higher than comparison groups (ps < 0.05). ECIG users associate positive outcomes with ECIG use. ECIG outcome expectancies may affect product appeal and tobacco use behaviors and should be examined further to inform regulatory policies. (PsycINFO Database RecordCopyright (c) 2017 APA, all rights reserved).
1186	Kristin Brikmanis, Angela Petersen, Neal Doran E-cigarette use, perceptions, and cigarette smoking intentions in a community sample of young adult nondaily cigarette smokers.. Psychology of addictive behaviors : journal of the Society of Psychologists in Addictive Behaviors; 2017;31:336.<br><br>**Keywords:**<br>Adolescent,*Electronic Nicotine Delivery Systems,Female,Harm Reduction,*Health Knowledge, Attitudes, Practice,Humans,*Intention,Longitudinal Studies,Male,*Smokers/px [Psychology],Smoking Cessation, Smoking Prevention,*Vaping/px [Psychology],Young Adult<br><br>**Abstract:** E-cigarettes have been suggested as a strategy for reducing harm from cigarettes. Although e-cigarettes could be a less-harmful alternative to cigarettes for those trying to quit, there may also be costs that outweigh any benefits of reduction. The purpose of the present study was to prospectively investigate perceptions of e-cigarettes, cigarette smoking intentions, and their associations with e-cigarette use over time. Community participants (N = 348, 57% male) aged 18 to 24 years were recruited for a longitudinal study of tobacco use. Inclusion criteria included nondaily cigarette smoking for >= 6 months with no history of daily smoking. Participants reported e-cigarette use over the past 14 days at baseline, and for the past 9 days at 3, 6, and 9 months. Assessments were completed online or via mobile phone. Across the 4 assessments, 22% to 33% of participants reported recent e-cigarette use. Intent to quit smoking cigarettes and intent to maintain smoking were unrelated to e-cigarette frequency. E-cigarette frequency was positively associated with perceiving e-cigarettes as less harmful than cigarettes and more positive e-cigarette expectancies (ps < .05). E-cigarette use was also more frequent among those who smoked cigarettes frequently and who used e-cigarettes to circumvent cigarette bans more often (ps < .05). The combination of these findings suggests that, at least among nondaily smoking young adults, other factors may influence frequency of e-cigarette use more than harm reduction. Findings

	instead seem consistent with the hypothesis that e-cigarettes are more often used to complement ongoing cigarette smoking. (PsycINFO Database RecordCopyright (c) 2017 APA, all rights reserved).
1187	Sherazade Kinouani, Edwige Pereira, Christophe Tzourio Electronic Cigarette Use in Students and Its Relation with Tobacco-Smoking: A Cross-Sectional Analysis of the i-Share Study.. International journal of environmental research and public health; 2017;14:<br><br>**Keywords:**<br>Adolescent,Adult,Cross-Sectional Studies,*Electronic Nicotine Delivery Systems,Female,Flavoring Agents, France,Humans,Male,*Smokers/px [Psychology],Smokers/sn [Statistics & Numerical Data],Smoking Cessation,*Students/px [Psychology],Students/sn [Statistics & Numerical Data],*Tobacco Smoking,Young Adult<br><br>**Abstract:** While young adults often try e-cigarettes, little is known about its use and the reasons for experimentation, particularly in relation with tobacco-smoking. In 2016, data were collected from 2720 French-speaking students participating in a web-based study on students' health: the internet-based Students Health Research Enterprise (i-Share) project. Univariate analyses and multivariable logistic regressions were performed to study the relationship between e-cigarette use and smoking status. Two out of five students declared having tried e-cigarettes and 3.6% were current users. Former smokers were more likely than current smokers to use e-cigarettes currently. Among those who had never smoked, 13.5% had tried e-cigarettes. Very few (0.3%) were current users, alternating e-liquids with and without nicotine. The three main reasons for trying e-cigarettes were curiosity, offer to try by someone, and attractiveness of e-liquid flavors. Among current smokers, previous attempts to quit smoking and a strong desire to stop tobacco were reported more in e-cigarette current users than in former users. In this large sample of French students, findings were consistent with the possibility that e-cigarettes might be used as smoking cessation or reduction aids by some young adults whereas other young never-smokers could be exposed to nicotine.
1191	Richard A Miech, Patrick M O'Malley, Lloyd D Johnston, Megan E Patrick E-Cigarettes and the Drug Use Patterns of Adolescents.. Nicotine & tobacco research : official journal of the Society for Research on Nicotine and Tobacco; 2016;18:654.<br><br>**Keywords:**<br>Adolescent,Cross-Sectional Studies,*Electronic Nicotine Delivery Systems/sn [Statistics & Numerical Data],Female,Humans,Male,*Substance-Related Disorders/ep [Epidemiology]<br><br>**Abstract:** INTRODUCTION: This study examines the role of e-cigarettes in the drug use patterns of adolescents. Of specific interest is whether adolescent e-cigarette users fall into a group of (1) youth who do not use traditional drugs of abuse or (2) polysubstance users., METHODS: Using latent class analysis, we identify major "classes" of substance users on the basis of recent use of e-cigarettes, alcohol, marijuana, cigarettes, and prescription drugs. Analyses are conducted separately for adolescents in 8th, 10th, and 12th grades. Data come from 16 615 participants in the 2014 Monitoring the Future survey., RESULTS: Youth who do not use traditional drugs of abuse account for about 50% of e-cigarette users in 8th grade, 35% in 10th grade, and 17% in 12th grade. These youth come from a large "low-level users" group found in each grade, characterized by low probability of use for all substances (e-cigarette probability in this group for 8th graders = .046; 10th graders = .071; 12th graders = .027). Other e-cigarette users come from a smaller, "poly-users" group found in each grade, characterized by high-to-moderate probabilities (.83-.21) of using e-cigarettes and other substances. Specific to 12th grade is a third, additional polysubstance group characterized by high likelihood of e-cigarette use (.93)., CONCLUSIONS: The proportion of e-cigarette users who do not use traditional drugs of abuse is larger at younger ages. Longitudinal panel studies starting at 8th and 10th grades may best inform the current debate on whether e-cigarette use is a risk or protective factor for future transition to the use of other substances., IMPLICATIONS: The proportion of e-cigarette users who do not use traditional drugs of

	abuse is larger at younger ages. Longitudinal panel studies starting at 8th and 10th grades may best inform the current debate on whether e-cigarette use is a risk or protective factor for future transition to the use of other substances.Copyright © The Author 2015. Published by Oxford University Press on behalf of the Society for Research on Nicotine and Tobacco. All rights reserved. For permissions, please e-mail: journals.permissions@oup.com.
1195	Shona Hilton, Heide Weishaar, Helen Sweeting, Filippo Trevisan, Srinivasa Vittal Katikireddi E-cigarettes, a safer alternative for teenagers? A UK focus group study of teenagers' views.. BMJ open; 2016;6:e013271.<br><br>**Keywords:**<br>Adolescent,*Electronic Nicotine Delivery Systems,Female,Focus Groups,Humans,Male,Qualitative Research,Safety,*Smoking/th [Therapy],*Smoking Cessation/mt [Methods],Smoking Prevention,United Kingdom<br><br>**Abstract:** OBJECTIVE: Concerns exist that e-cigarettes may be a gateway to traditional cigarettes and/or (re)normalise teenage smoking. This qualitative study explores how teenagers in the UK currently perceive e-cigarettes and how and why they do or do not use them., DESIGN: 16 focus groups were conducted across the UK between November 2014 and February 2015, with 83 teenagers aged 14-17. All discussions were digitally recorded, transcribed verbatim, imported into NVivo 10 and thematically analysed., RESULTS: Teenagers generally agreed that e-cigarettes are useful products for smokers, including teenage smokers, to quit or reduce traditional cigarette use. Concerns were expressed about lack of information on their precise ingredients and any unknown risks for users and bystanders. However, teenagers typically viewed e-cigarettes as substantially less harmful than traditional cigarettes. They perceived e-cigarettes as attractive, with products described as 'fun' and having 'great flavourings'. Seeing websites or social media featuring e-cigarettes, especially YouTube 'vaping tricks', prompted some experimentation and imitation. E-cigarettes were used in a variety of situations, including at parties or when they could not smoke traditional cigarettes. A very few participants suggested covert use was a possibility and that e-cigarettes might help maintain a fledgling nicotine habit., CONCLUSIONS: Teenagers support the use of e-cigarettes as smoking cessation aids for established adult smokers. However, they engage with these products differently from adults, with the novel hypothesis that covert use could potentially reinforce traditional cigarette smoking requiring further investigation. Policy responses should more clearly meet the needs of young people, as well as helping established adult smokers.Copyright Published by the BMJ Publishing Group Limited. For permission to use (where not already granted under a licence) please go to http://www.bmj.com/company/products-services/rights-and-licensing/.
1199	Graham F Moore, Hannah J Littlecott, Laurence Moore, Nilufar Ahmed, Jo Holliday E-cigarette use and intentions to smoke among 10-11-year-old never-smokers in Wales.. Tobacco control; 2016;25:147.<br><br>**Keywords:**<br>Age Factors,Child,*Child Behavior,Cross-Sectional Studies,*Electronic Nicotine Delivery Systems,Female, *Health Behavior,Humans,*Intention,Logistic Models,Male,Multivariate Analysis,Odds Ratio,Parents/px [Psychology],Peer Influence,Prevalence,Risk Factors,Smoking/ep [Epidemiology],*Smoking/px [Psychology],Smoking Prevention,Surveys and Questionnaires,Time Factors,Wales/ep [Epidemiology]<br><br>**Abstract:** BACKGROUND: E-cigarettes are seen by some as offering harm reduction potential, where used effectively as smoking cessation devices. However, there is emerging international evidence of growing use among young people, amid concerns that this may increase tobacco uptake. Few UK studies examine the prevalence of e-cigarette use in non-smoking children or associations with intentions to smoke., METHODS: A cross-sectional survey of year 6 (10-11-year-old) children in Wales. Approximately 1500 children completed questions on e-cigarette use, parental and peer smoking, and intentions to smoke. Logistic regression analyses among never smoking children, adjusted for school-level clustering, examined associations of smoking norms with e-cigarette use, and of e-cigarette use with intentions to smoke tobacco within the next 2 years., RESULTS: Approximately 6% of year 6 children, including 5% of

	never smokers, reported having used an e-cigarette. By comparison to children whose parents neither smoked nor used e-cigarettes, children were most likely to have used an e-cigarette if parents used both tobacco and e-cigarettes (OR=3.40; 95% CI 1.73 to 6.69). Having used an e-cigarette was associated with intentions to smoke (OR=3.21; 95% CI 1.66 to 6.23). While few children reported that they would smoke in 2 years' time, children who had used an e-cigarette were less likely to report that they definitely would not smoke tobacco in 2 years' time and were more likely to say that they might., CONCLUSIONS: E-cigarettes represent a new form of childhood experimentation with nicotine. Findings are consistent with a hypothesis that children use e-cigarettes to imitate parental and peer smoking behaviours, and that e-cigarette use is associated with weaker antismoking intentions.Copyright Published by the BMJ Publishing Group Limited. For permission to use (where not already granted under a licence) please go to http://www.bmj.com/company/products-services/rights-and-licensing/
1203	Kelvin Choi, Rachel Grana, Debra Bernat Electronic Nicotine Delivery Systems and Acceptability of Adult Cigarette Smoking Among Florida Youth: Renormalization of Smoking?.. The Journal of adolescent health : official publication of the Society for Adolescent Medicine; 2017;60:592.<br><br>**Keywords:**<br>Adolescent,Adult,*Advertising as Topic/sn [Statistics & Numerical Data],Child,*Cigarette Smoking/ep [Epidemiology],Cross-Sectional Studies,*Electronic Nicotine Delivery Systems/sn [Statistics & Numerical Data],Female,Florida/ep [Epidemiology],Health Surveys,Humans,Logistic Models,Male,Risk Factors, Students/px [Psychology],*Students/sn [Statistics & Numerical Data]<br><br>**Abstract:** PURPOSE: There is a dearth of research into whether electronic nicotine delivery systems (ENDS) promote acceptance of cigarette smoking. Therefore, we aimed to assess the association between ENDS exposure, acceptance of cigarette smoking, and susceptibility to cigarette smoking., METHODS: Data from the 2014 Florida Youth Tobacco Survey with a state representative sample of middle and high school students (n = 68,928) were analyzed. Own ENDS use, exposure to ENDS advertising, living with ENDS users, acceptance of adult cigarette smoking, demographics, and known predictors of cigarette smoking were assessed. Susceptibility to cigarette smoking was assessed among never smokers. Weighted multiple logistic regression models and mediation analyses were conducted, stratified by middle/high school and never/ever smoking. Analyses were conducted in 2016., RESULTS: Own ENDS use, exposure to ENDS advertising, and living with ENDS users were associated with acceptance of adult cigarette smoking even among never smokers, after accounting for covariates ($p < .05$). In a mediation analysis, own ENDS use, exposure to ENDS advertising, and living with ENDS users were indirectly associated with susceptibility to cigarette smoking among never smokers through acceptance of adult cigarette smoking ($p < .05$)., CONCLUSIONS: Youth ENDS exposure may contribute to normalizing adult cigarette smoking and may in turn heighten susceptibility to cigarette smoking. If confirmed by longitudinal studies, these findings suggest that ENDS policy interventions may help prevent youth cigarette smoking.Copyright Published by Elsevier Inc.
1204	Lauren Porter, Jennifer Duke, Meredith Hennon, David Dekevich, Erik Crankshaw, Ghada Homsi, Matthew Farrelly Electronic Cigarette and Traditional Cigarette Use among Middle and High School Students in Florida, 2011-2014.. PloS one; 2015;10:e0124385.<br><br>**Keywords:**<br>Adolescent,Cross-Sectional Studies,*Electronic Nicotine Delivery Systems/ut [Utilization],Female, Florida/ep [Epidemiology],Humans,Male,*Marketing/es [Ethics],Prevalence,Public Health,Schools, *Smoking/ep [Epidemiology],Smoking/pp [Physiopathology],Smoking/px [Psychology],Students/px [Psychology],Surveys and Questionnaires,*Tobacco Products/ut [Utilization]<br><br>**Abstract:** Recent youth trends in the prevalence of e-cigarette and traditional cigarette use in Florida were examined in a cross-sectional, representative state sample from 2011 to 2014. Traditional cigarette use among youth declined during the study period. Experimentation with and past-30-day use of e-

	cigarettes among Florida youth tripled over 4 years. Past-30-day e-cigarette use exceeded traditional cigarette use in 2014; 10.8% of high school and 4.0% of middle school students reported recent e-cigarette use, compared with 8.7% of high school and 2.9% of middle school students for traditional cigarettes (P<0.001). By 2014, 20.5% of high school and 8.5% of middle school students reported ever use of e-cigarettes. Among ever e-cigarette users in 2014, 30.3% of high school and 42.2% of middle school students had never smoked traditional cigarettes. Given the concern that significant rates of e-cigarette use by U.S. adolescents may have a negative effect on public health, further review of e-cigarette advertising, marketing, sales, and use among U.S. youth is warranted.
1225	Andrea C Villanti, Jennifer L Pearson, Allison M Glasser, Amanda L Johnson, Lauren K Collins, Raymond S Niaura, David B Abrams Frequency of Youth E-Cigarette and Tobacco Use Patterns in the United States: Measurement Precision Is Critical to Inform Public Health.. Nicotine & tobacco research : official journal of the Society for Research on Nicotine and Tobacco; 2017;19:1345.<br><br>**Keywords:**<br>Adolescent,Child,Child Health Services,*Electronic Nicotine Delivery Systems/sn [Statistics & Numerical Data],Female,Humans,Male,Prevalence,Public Health Surveillance,*Smoking/ep [Epidemiology], Students/sn [Statistics & Numerical Data],*Tobacco Products/sn [Statistics & Numerical Data],United States/ep [Epidemiology]<br><br>**Abstract:** Introduction: E-cigarette use occurs with tobacco product use in youth., Methods: Using the 2014 National Youth Tobacco Survey (NYTS), we examined past-30-day frequency of cigarette, cigar, smokeless, and e-cigarette use in the context of past-30-day and ever tobacco product use in US middle and high school students (N = 22 007). Frequency of product-specific use was examined by exclusive versus concurrent use with another product in the past 30 days (poly-use)., Results: In 2014, the majority (83%) of US middle and high school students had not used tobacco or e-cigarettes in the past 30 days. In the 9.3% of youth reporting any past-30-day e-cigarette use, 63% also reported using a tobacco product; among the 3.3% past-30-day exclusive e-cigarette users, about two-thirds (2.1%) had ever used combustible or non-combustible tobacco products and one-third (1.2%) had not. Few never tobacco users had used e-cigarettes on 10 or more days in the past month (absolute percent < 0.1%). Among past-30-day cigarette and smokeless users, the two highest frequency categories were 1-2 days and daily use; among past-30-day e-cigarette and cigar users, prevalence decreased with increasing frequency of use. The majority of past-30-day cigarette, cigar, smokeless, and e-cigarette users reported poly-use., Conclusions: Prevalence estimates for a single product mask the complex patterns of frequency, temporality, and poly-use in youth. Two-thirds of past-30-day exclusive e-cigarette users have ever used tobacco. Poly-use is the dominant pattern of tobacco and e-cigarette use among US middle and high school students., Implications: Our study highlights the complexity of tobacco use patterns in US middle and high school students. Future studies addressing the full public health impact of movement into or out of combustible tobacco use will require longitudinal data with appropriate measures of tobacco and e-cigarette product-specific use (eg, frequency and intensity), as well as adequate sample size and a sufficient number of waves to determine how use of individual products, like e-cigarettes, impact progression into or out of more stable patterns of tobacco and e-cigarette use.Copyright © The Author 2016. Published by Oxford University Press on behalf of the Society for Research on Nicotine and Tobacco. All rights reserved. For permissions, please e-mail: journals.permissions@oup.com.
1244	Sarah T Cherng, Jamie Tam, Paul J Christine, Rafael Meza Modeling the Effects of E-cigarettes on Smoking Behavior: Implications for Future Adult Smoking Prevalence.. Epidemiology (Cambridge, Mass.); 2016;27:819.<br><br>**Keywords:**<br>Adolescent,Adult,Aged,Aged, 80 and over,*Electronic Nicotine Delivery Systems,Female,Humans,Male, Middle Aged,*Models, Psychological,Prevalence,Smoking/ep [Epidemiology],*Smoking/px [Psychology], *Smoking Cessation/px [Psychology],Smoking Cessation/sn [Statistics & Numerical Data],United

States/ep [Epidemiology],Young Adult

Abstract: BACKGROUND: Electronic cigarette (e-cigarette) use has increased rapidly in recent years. Given the unknown effects of e-cigarette use on cigarette smoking behaviors, e-cigarette regulation has become the subject of considerable controversy. In the absence of longitudinal data documenting the long-term effects of e-cigarette use on smoking behavior and population smoking outcomes, computational models can guide future empirical research and provide insights into the possible effects of e-cigarette use on smoking prevalence over time., METHODS: Agent-based model examining hypothetical scenarios of e-cigarette use by smoking status and e-cigarette effects on smoking initiation and smoking cessation., RESULTS: If e-cigarettes increase individual-level smoking cessation probabilities by 20%, the model estimates a 6% reduction in smoking prevalence by 2060 compared with baseline model (no effects) outcomes. In contrast, e-cigarette use prevalence among never smokers would have to rise dramatically from current estimates, with e-cigarettes increasing smoking initiation by more than 200% relative to baseline model estimates to achieve a corresponding 6% increase in smoking prevalence by 2060., CONCLUSIONS: Based on current knowledge of the patterns of e-cigarette use by smoking status and the heavy concentration of e-cigarette use among current smokers, the simulated effects of e-cigarettes on smoking cessation generate substantially larger changes to smoking prevalence compared with their effects on smoking initiation.

1250 Lisa A Peterson, Stephen S Hecht Tobacco, e-cigarettes, and child health.. Current opinion in pediatrics; 2017;29:225.

Keywords:
Adult,Child,*Child Health,Electronic Nicotine Delivery Systems/sn [Statistics & Numerical Data],Female, Fetal Development,Humans,Male,Pregnancy,Pregnancy Outcome,Prenatal Exposure Delayed Effects/ep [Epidemiology],*Prenatal Exposure Delayed Effects/pc [Prevention & Control],*Smoking/ae [Adverse Effects],Smoking/ep [Epidemiology],*Smoking Prevention/og [Organization & Administration],*Tobacco Smoke Pollution/ae [Adverse Effects],Tobacco Smoke Pollution/sn [Statistics & Numerical Data],United States

Abstract: PURPOSE OF THE REVIEW: The availability of the Children's Health Exposure Assessment Resource funded by the National Institute of Environmental Health Sciences provides new opportunities for exploring the role of tobacco smoke exposure in causing harm to children., RECENT FINDINGS: Children of smokers are exposed to nicotine and other harmful tobacco smoke chemicals in utero as well as in their environment. This passive exposure to tobacco smoke has a variety of negative effects on children. In-utero exposure to tobacco smoke causes poor birth outcomes and influences lung, cardiovascular, and brain development, placing children at increased risk of a number of adverse health outcomes later in life, such as obesity, behavioral problems, and cardiovascular disease. Furthermore, most smokers start in their adolescence, an age of increased nicotine addiction risk. Biomarkers of tobacco exposure helps clarify the role tobacco chemicals play in influencing health both in childhood and beyond. Although electronic cigarettes (e-cigarettes) appear to be a nicotine delivery device of reduced harm, it appears to be a gateway to the use of combustible cigarette smoking in adolescents., SUMMARY: Pediatric researchers interested in elucidating the role of tobacco smoke exposure in adverse outcomes in children should incorporate biomarkers of tobacco exposure in their studies.

1251 Tory R Spindle, Marzena M Hiler, Megan E Cooke, Thomas Eissenberg, Kenneth S Kendler, Danielle M Dick Electronic cigarette use and uptake of cigarette smoking: A longitudinal examination of U.S. college students.. Addictive behaviors; 2017;67:66.

Keywords:
Adolescent,*Cigarette Smoking/ep [Epidemiology],*Electronic Nicotine Delivery Systems/sn [Statistics & Numerical Data],Female,Humans,Longitudinal Studies,Male,*Students/sn [Statistics & Numerical Data], Surveys and Questionnaires,United States/ep [Epidemiology],Universities

	**Abstract:** INTRODUCTION: Electronic cigarette (e-cigarette) use prevalence is increasing among U.S. adolescents and adults but recent longitudinal data for college/university students are scarce. Furthermore, the extent that e-cigarette use is associated with the onset of cigarette smoking and the factors that lead to the uptake of e-cigarettes in college students has not been explored., METHODS: 3757 participants from a Mid-Atlantic university (women: 66%; White: 45%; Black: 21%; Asian: 19%; Hispanic/Latino: 6%) were surveyed in 2014 and again in 2015., RESULTS: Among participants reporting never smoking at time 1, those who had ever tried e-cigarettes or were currently using e-cigarettes (at least one use in past 30days) were more likely to have ever tried cigarettes by time 2 relative to individuals who had not used e-cigarettes. Ever use of e-cigarettes (but not current use) also increased participants' likelihood of being current cigarette smokers at time 2. Among initial never users of e-cigarettes or cigarettes, males and ever marijuana users had an increased probability of trying e-cigarettes by time 2. Furthermore, less perseverance (an index of impulsivity) and ever use of other tobacco products increased initial never users' chances of trying both cigarettes and e-cigarettes by time 2., CONCLUSIONS: Given that never-smoking participants who had tried e-cigarettes were more likely to initiate cigarette use later, limiting young adults' access to these products may be beneficial. As the long-term health implications of e-cigarette use become clearer, predictors of e-cigarette use could help identify future populations likely to use and abuse these products.Copyright © 2016 Elsevier Ltd. All rights reserved.
1252	Stephanie T Lanza, Michael A Russell, Jessica L Braymiller Emergence of electronic cigarette use in US adolescents and the link to traditional cigarette use.. Addictive behaviors; 2017;67:38.<br><br>**Keywords:**<br>Adolescent,*Adolescent Behavior,Adult,Age Factors,Child,*Cigarette Smoking/ep [Epidemiology],Cross-Sectional Studies,*Electronic Nicotine Delivery Systems/sn [Statistics & Numerical Data],Female,Humans, Male,Surveys and Questionnaires,United States,Young Adult<br><br>**Abstract:** BACKGROUND: Electronic cigarettes (e-cigarettes) are increasingly used by US adolescents and may be a gateway to traditional cigarette use. We examine rates of both products by age and examine differences in age-varying rates by sex and race/ethnicity., METHODS: Data are from the 2014 National Youth Tobacco Survey, a national sample of US middle and high school students (n=22.007); students ages 11-19 were included. Past-30-day e-cigarette and traditional cigarette use were examined as a function of age; sex and race/ethnicity were included as moderators. The age-varying association between e-cigarette and traditional cigarette use was also examined., RESULTS: Rates of e-cigarette use increase faster than traditional cigarette use from ages 13-16. Compared to females, males had higher rates of e-cigarette use from ages 14-17.5 and traditional cigarette use from ages 15-18. Between ages 12-14, more Hispanic adolescents used e-cigarettes compared to White or Black adolescents; after age 14 Hispanics and Whites reported similar rates, peaking at twice the rate for Blacks. Hispanic adolescents report greater traditional cigarette use versus Whites between ages 12-13, but lower rates between ages 15-18. E-cigarette use was strongly associated with traditional cigarette use, particularly during early adolescence [OR>40 before age 12]., CONCLUSIONS: Young Hispanic adolescents are at elevated risk for use of e-cigarettes and traditional cigarettes during early adolescence. During early adolescence, youth using e-cigarettes are more likely to smoke traditional cigarettes compared to youth not using e-cigarettes. The study of age-varying effects holds promise for advancing understanding of disparities in health risk behaviors.Copyright © 2016 Elsevier Ltd. All rights reserved.
1253	Ji-Yeun Park, Dong-Chul Seo, Hsien-Chang Lin E-Cigarette Use and Intention to Initiate or Quit Smoking Among US Youths.. American journal of public health; 2016;106:672.<br><br>**Keywords:**<br>Adolescent,Adolescent Behavior,*Electronic Nicotine Delivery Systems/ut [Utilization],Female,Humans, *Intention,Male,Propensity Score,*Smoking,Smoking Cessation/mt [Methods],Smoking Cessation/px

	[Psychology],*Smoking Cessation,Surveys and Questionnaires,United States<br><br>**Abstract:** OBJECTIVES: We assessed whether e-cigarette use is associated with (1) intention to smoke cigarettes among never-smoking youths and youth experimenters, and (2) intention to quit smoking among current youth smokers., METHODS: We categorized participants from the 2012 National Youth Tobacco Survey data (n=20193) as never-smoking youth (n=16238), youth experimenters (n=3248), and current youth smokers (n=707). We matched groups on the basis of a propensity score comprising covariates predicting e-cigarette use., RESULTS: E-cigarette users who had never smoked cigarettes (adjusted odds ratio [AOR]=3.62; 95% confidence interval [CI]=2.04, 6.45) and who had experimented with smoking (AOR=1.99; 95% CI=1.50, 2.64) had elevated intention to smoke cigarettes compared with their counterparts who had never used e-cigarettes. We did not find any significant association between e-cigarette use and intention to quit smoking among current youth smokers (P=.33)., CONCLUSIONS: E-cigarette use among US youths is associated with intention to smoke but not with intention to quit smoking.
1261	Sungkyu Lee, Rachel A Grana, Stanton A Glantz Electronic cigarette use among Korean adolescents: a cross-sectional study of market penetration, dual use, and relationship to quit attempts and former smoking.. The Journal of adolescent health : official publication of the Society for Adolescent Medicine; 2014;54:684.<br><br>**Keywords:**<br>Adolescent,*Adolescent Behavior,Cross-Sectional Studies,*Electronic Nicotine Delivery Systems/sn [Statistics & Numerical Data],Female,Health Surveys/mt [Methods],*Health Surveys/sn [Statistics & Numerical Data],Humans,Male,*Marketing/mt [Methods],Marketing/sn [Statistics & Numerical Data], Prevalence,Republic of Korea/ep [Epidemiology],*Smoking/ep [Epidemiology],Smoking Cessation/mt [Methods],*Smoking Cessation/sn [Statistics & Numerical Data],Surveys and Questionnaires<br><br>**Abstract:** PURPOSE: As elsewhere, in South Korea electronic cigarettes (e-cigarettes) are marketed, in part, as a smoking cessation aid. We assessed the prevalence of e-cigarette use among Korean adolescents and the relationship between e-cigarette use and current (past-30-day) smoking, cigarettes/day, attempts to quit conventional cigarettes, and ceasing to use cigarettes., METHODS: Data from the 2011 Korean Youth Risk Behavior Web-based Survey of 75,643 students aged 13-18 years were analyzed with logistic regression., RESULTS: A total of 9.4% (8.0% ever-dual users who were concurrently using e-cigarettes and smoking conventional cigarettes and 1.4% ever-e-cigarette only users) of Korean adolescents have ever used e-cigarettes and 4.7% were current (past-30-day) e-cigarette users (3.6% dual users and 1.1% e-cigarettes only). After adjusting for demographics, current cigarette smokers were much more likely to use e-cigarettes than were nonsmokers. Among current cigarette smokers, those who smoked more frequently were more likely to be current e-cigarette users. The odds of being an e-cigarette user were 1.58 times (95% confidence interval, 1.39-1.79) higher among students who had made an attempt to quit than for those who had not. It was rare for students no longer using cigarettes to be among current e-cigarette users (odds ratio, .10; confidence interval, .09-.12)., CONCLUSIONS: Some Korean adolescents may be responding to advertising claims that e-cigarettes are a cessation aid: those who had made an attempt to quit were more likely to use e-cigarettes but less likely to no longer use cigarettes. E-cigarette use was strongly associated with current and heavier cigarette smoking.Copyright © 2014 Society for Adolescent Health and Medicine. Published by Elsevier Inc. All rights reserved.
1268	Bill Poland, Florian Teischinger Population Modeling of Modified Risk Tobacco Products Accounting for Smoking Reduction and Gradual Transitions of Relative Risk.. Nicotine & tobacco research : official journal of the Society for Research on Nicotine and Tobacco; 2017;19:1277.<br><br>**Keywords:**<br>*Electronic Nicotine Delivery Systems,Harm Reduction,Humans,Models, Statistical,Risk,*Smoking/ep

[Epidemiology],*Smoking Cessation/mt [Methods],*Tobacco Products,United States/ep [Epidemiology], United States Food and Drug Administration

Abstract: Introduction: As suggested by the Food and Drug Administration (FDA) Modified Risk Tobacco Product (MRTP) Applications Draft Guidance, we developed a statistical model based on public data to explore the effect on population mortality of an MRTP resulting in reduced conventional cigarette smoking. Many cigarette smokers who try an MRTP persist as dual users while smoking fewer conventional cigarettes per day (CPD). Lower-CPD smokers have lower mortality risk based on large cohort studies. However, with little data on the effect of smoking reduction on mortality, predictive modeling is needed., Methods: We generalize prior assumptions of gradual, exponential decay of Excess Risk (ER) of death, relative to never-smokers, after quitting or reducing CPD. The same age-dependent slopes are applied to all transitions, including initiation of conventional cigarettes and to a second product (MRTP). A Monte Carlo simulation model generates random individual product use histories, including CPD, to project cumulative deaths through 2060 in a population with versus without the MRTP. Transitions are modeled to and from dual use, which affects CPD and cigarette quit rates, and to MRTP use only., Results: Results in a hypothetical scenario showed high sensitivity of long-run mortality to CPD reduction levels and moderate sensitivity to ER transition rates., Conclusions: Models to project population effects of an MRTP should account for possible mortality effects of reduced smoking among dual users. In addition, studies should follow dual-user CPD histories and quit rates over long time periods to clarify long-term usage patterns and thereby improve health impact projections., Implications: We simulated mortality effects of a hypothetical MRTP accounting for cigarette smoking reduction by smokers who add MRTP use. Data on relative mortality risk versus CPD suggest that this reduction may have a substantial effect on mortality rates, unaccounted for in other models. This effect is weighed with additional hypothetical effects in an example.Copyright © The Author 2017. Published by Oxford University Press on behalf of the Society for Research on Nicotine and Tobacco. All rights reserved. For permissions, please e-mail: journals.permissions@oup.com.

1284 Robert C McMillen, Mark A Gottlieb, Regina M Whitmore Shaefer, Jonathan P Winickoff, Jonathan D Klein Trends in Electronic Cigarette Use Among U.S. Adults: Use is Increasing in Both Smokers and Nonsmokers.. Nicotine & tobacco research : official journal of the Society for Research on Nicotine and Tobacco; 2015;17:1195.

Keywords:
Adolescent,Adult,Cross-Sectional Studies,Electronic Nicotine Delivery Systems/sn [Statistics & Numerical Data],Electronic Nicotine Delivery Systems/td [Trends],*Electronic Nicotine Delivery Systems/ut [Utilization],Female,Humans,Male,Middle Aged,Prevalence,Smoking/ep [Epidemiology],Smoking Cessation/mt [Methods],Smoking Cessation/sn [Statistics & Numerical Data],Smoking Prevention,Surveys and Questionnaires,United States/ep [Epidemiology],Young Adult

Abstract: OBJECTIVES: We assessed trends in use of electronic cigarettes among U.S. adults, demographic predictors of use, and smoking status of current electronic cigarette users., METHODS: Mixed-mode surveys were used to obtain representative, cross-sectional samples of U.S. adults in each of 4 years., RESULTS: Sample sizes for 2010, 2011, 2012, and 2013 were 3,240, 3,097, 3,101, and 3,245, respectively. Ever use of electronic cigarettes increased from 1.8% (2010) to 13.0% (2013), while current use increased from 0.3% to 6.8%, $p < .001$. Prevalence of use increased significantly across all demographic groups. In 2013, current use among young adults 18-24 (14.2%) was higher than adults 25-44 (8.6%), 45-64 (5.5%), and 65+ (1.2%). Daily smokers (30.3%) and nondaily smokers (34.1%) were the most likely to currently use e-cigarettes, compared to former smokers (5.4%) and never-smokers (1.4%), $p < .001$. However, 32.5% of current electronic cigarette users are never- or former smokers., CONCLUSIONS: There has been rapid growth in ever and current electronic cigarette use over the past 4 years. Use is highest among young adults and current cigarette smokers. Although smokers are most likely to use these products, almost a third of current users are nonsmokers, suggesting that e-cigarettes contribute to primary

	nicotine addiction and to renormalization of tobacco use. Regulatory action is needed at the federal, state, and local levels to ensure that these products do not contribute to preventable chronic disease.Copyright © The Author 2014. Published by Oxford University Press on behalf of the Society for Research on Nicotine and Tobacco. All rights reserved. For permissions, please e-mail: journals.permissions@oup.com.
1287	Dinska Van Gucht, Frank Baeyens Health professionals in Flanders perceive the potential health risks of vaping as lower than those of smoking but do not recommend using e-cigarettes to their smoking patients.. Harm reduction journal; 2016;13:22.<br><br>**Keywords:**<br>Adult,*Attitude of Health Personnel,Belgium,Counseling,*Electronic Nicotine Delivery Systems/ae [Adverse Effects],Female,General Practice/sn [Statistics & Numerical Data],Humans,Male,Middle Aged, Patient Education as Topic,Perception,Risk Assessment,Risk-Taking,Smoking Cessation,*Smoking Prevention,Surveys and Questionnaires,*Vaping/ae [Adverse Effects],Young Adult<br><br>**Abstract:** BACKGROUND: Many misperceptions of both risks and opportunities of e-cigarettes (e-cigs) exist among the general population and among physicians, although e-cigs could be a valuable harm reduction tool for current smokers., METHODS: Two groups in Flanders, namely general practitioners (GPs; family doctors) and tobacco counselors filled out an online questionnaire with regard to their attitudes and risk perceptions concerning e-cigs. Statements included were on the safety and the addictive properties of e-cigs in absolute terms, whereas other items compared e-cigs with regular tobacco cigarettes. Statements about possible "gateway" and "renormalization" effects, selling to minors, and use in public places and on the potential of e-cigs as a smoking cessation aid were also included. Respondents were also asked for the rate at which their patients asked information about e-cigs, if they would recommend e-cigs to their smoking patients, and whether they had information brochures on e-cigs., RESULTS: About 70 % believed that e-cigs are harmful to vapers, and about half to two thirds believed that e-cigs are carcinogenic, increase cardiovascular risk, and increase the risk of chronic lung disease. Also, a substantial minority incorrectly believed these risks to be no less than those resulting from regular smoking. Ten to almost 20 % disagreed that e-cigs are healthier and represent less risk for the main serious smoking-related diseases than conventional cigarettes. More than half of the respondents disagreed that e-cigs are an effective smoking cessation aid. None (0 %) offered the strongest level of agreement for recommending e-cigs to their clients/patients, but GPs agreed to a lesser degree a bit more often than tobacco counselors. Almost none had information leaflets for potentially interested patients. Finally, the majority of our sample also believed that e-cigs will cause renormalization of smoking and that e-cigs will lead to an uptake of conventional smoking and disagreed with allowing vaping in enclosed public places., CONCLUSIONS: Health professionals in Flanders perceive the potential health risks of vaping as lower than those of smoking but do not recommend using e-cigs to their smoking patients.
1290	William V Lechner, Tim Janssen, Christopher W Kahler, Janet Audrain-McGovern, Adam M Leventhal Bi-directional associations of electronic and combustible cigarette use onset patterns with depressive symptoms in adolescents.. Preventive medicine; 2017;96:73.<br><br>**Keywords:**<br>Adolescent,Adolescent Behavior,*Cigarette Smoking/ep [Epidemiology],*Depression/eh [Ethnology], *Electronic Nicotine Delivery Systems/sn [Statistics & Numerical Data],Female,Humans,Los Angeles,Male, Risk Factors<br><br>**Abstract:** Whether well-documented patterns of mental health comorbidity with adolescent combustible cigarette use extend to e-cigarette use is unclear. Demonstrating associations between e-cigarette and combustible cigarette use with mental health symptomatology across adolescence may be important for promoting accurate perceptions of populations at risk for and potential consequences of tobacco product

	use. Adolescents (N=2460; mean age at baseline=14.1; 53.4% female; 44.1% Hispanic) who had never previously used combustible or e-cigarettes were assessed at baseline, and 6- and 12-month follow-ups in Los Angeles, CA (2013-2014). Logistic regression was used to examine associations between baseline depressive symptoms and onset of e-cigarette and cigarette single product and dual use at follow-ups. Latent growth modeling was used to examine associations between sustained use of either product (vs. non-use) and changes in depressive symptoms over 12-months. Higher baseline depressive symptoms predicted subsequent onset of cigarette (OR=1.024, 95% C.I.=1.009-1.055), e-cigarette (OR=1.015, C.I.=1.003-1.023), and dual use of both products (OR=1.021, C.I.=1.003-1.043). Sustained use of e-cigarettes over the 12-month observation (vs. non-use) was associated with a greater rate of increase in depressive symptoms over time (b=1.272, SE=0.513, p=0.01). Among those who sustained use of e-cigarettes, higher frequency of use was associated with higher depressive symptoms at the final follow-up (B=1.611, p=0.04). A bi-directional association of depressive symptoms with e-cigarette use onset across mid adolescence was observed. Further research on the causal nature, etiological underpinnings, and intervention implications of mental health and tobacco product use comorbidity is warranted.Copyright © 2016 Elsevier Inc. All rights reserved.
1319	Mark D Litt, Valerie Duffy, Cheryl Oncken Cigarette smoking and electronic cigarette vaping patterns as a function of e-cigarette flavourings.. Tobacco control; 2016;25:ii67.<br><br>**Keywords:**<br>Adolescent,Adult,*Cigarette Smoking/ep [Epidemiology],*Electronic Nicotine Delivery Systems,Female, *Flavoring Agents/ad [Administration & Dosage],Harm Reduction,Humans,Male,Middle Aged,Smokers/px [Psychology],Smokers/sn [Statistics & Numerical Data],Smoking Cessation/mt [Methods],Tobacco Products,*Vaping/sn [Statistics & Numerical Data],Young Adult<br><br>**Abstract:** INTRODUCTION: The present study examined the influence of flavouring on the smoking and vaping behaviour of cigarette smokers asked to adopt e-cigarettes for a period of 6 weeks., METHODS: Participants were 88 current male and female smokers with no intention to stop smoking, but who agreed to substitute e-cigarettes for their current cigarettes. On intake, participants were administered tests of taste and smell for e-cigarettes flavoured with tobacco, menthol, cherry and chocolate, and were given a refillable e-cigarette of their preferred flavour or a control flavour. Participants completed daily logs of cigarette and e-cigarette use and were followed each week., RESULTS: Analyses over days indicated that, during the 6-week e-cigarette period, cigarette smoking rates dropped from an average of about 16 to about 7 cigarettes/day. e-Cigarette flavour had a significant effect such that the largest drop in cigarette smoking occurred among those assigned menthol e-cigarettes, and the smallest drop in smoking occurred among those assigned chocolate and cherry flavours. e-Cigarette vaping rates also differed significantly by flavour assigned, with the highest vaping rates for tobacco- and cherry-flavoured e-cigarettes, and the lowest rates for those assigned to chocolate., CONCLUSIONS: The findings suggest that adoption of e-cigarettes in smokers may influence smoking rates and that e-cigarette flavourings can moderate this effect. e-Cigarette vaping rates are also influenced by flavourings. These findings may have implications for the utility of e-cigarettes as a nicotine replacement device and for the regulation of flavourings in e-cigarettes for harm reduction.Copyright Published by the BMJ Publishing Group Limited. For permission to use (where not already granted under a licence) please go to http://www.bmj.com/company/products-services/rights-and-licensing/.
1321	Allison C Hoffman, Raydel Valdes Salgado, Carolyn Dresler, Rachel Williams Faller, Christopher Bartlett Flavour preferences in youth versus adults: a review.. Tobacco control; 2016;25:ii32.<br><br>**Keywords:**<br>Adolescent,Adult,Age Factors,Aged,Child,Consumer Behavior/sn [Statistics & Numerical Data],Electronic Nicotine Delivery Systems/sn [Statistics & Numerical Data],*Flavoring Agents,Humans,Middle Aged, *Smoking/ep [Epidemiology],Smoking/px [Psychology],*Tobacco Products/sn [Statistics & Numerical Data],Tobacco, Smokeless/sn [Statistics & Numerical Data],Young Adult

	**Abstract:** OBJECTIVE: To understand the available evidence of how children and adults differ in their preferences for flavours that may be used in tobacco products., DATA SOURCES: A total of 474 articles published between 1931 and August 2015 were retrieved through searches conducted in PubMed, EMBASE, Web of Science and PsycINFO., STUDY SELECTION AND EXTRACTION: A 2-phase relevancy review process resulted in the identification of 59 articles and information was extracted by 2 independent reviewers., DATA SYNTHESIS: Findings were grouped by taste and smell preferences, which are important components of overall flavour. For taste, evidence is summarised in the following categories: sweet, salty, sour, bitter, umami and fat; within each of them, findings are organised by age categories. For smell, evidence is summarised as follows: fruit/herbal/spices, tobacco and coffee and other odours. Major findings from this search indicated that sweet preference in children and adolescents was higher than in adults. Examples of preferred food-related tastes and odours for young people included cherry, candy, strawberry, orange, apple and cinnamon. Currently, all these are used to flavour cigars, cartridges for electronic cigarettes, hookah (waterpipe) and smokeless tobacco products., CONCLUSIONS: Infants and children exhibited elevated sweet and salty preference relative to adults. Age-related changes in bitter, sour, umami and fat taste were not clear and more research would be useful. 'Sweet' food odours were highly preferred by children. Tobacco products in flavours preferred by young people may impact tobacco use and initiation, while flavours preferred by adults may impact product switching or dual use.Copyright Published by the BMJ Publishing Group Limited. For permission to use (where not already granted under a licence) please go to http://www.bmj.com/company/products-services/rights-and-licensing/.
1330	Thomas A Wills, Rebecca Knight, James D Sargent, Frederick X Gibbons, Ian Pagano, Rebecca J Williams Longitudinal study of e-cigarette use and onset of cigarette smoking among high school students in Hawaii.. Tobacco control; 2017;26:34.<br><br>**Keywords:**<br>Adolescent,Adolescent Behavior,*Cigarette Smoking/ep [Epidemiology],*Electronic Nicotine Delivery Systems/sn [Statistics & Numerical Data],Female,Follow-Up Studies,Hawaii/ep [Epidemiology],Humans, Longitudinal Studies,Male,Prevalence,Risk Factors,Schools,*Smoking/ep [Epidemiology],*Students/sn [Statistics & Numerical Data],Surveys and Questionnaires,Tobacco Products/sn [Statistics & Numerical Data]<br><br>**Abstract:** OBJECTIVE: Use of electronic cigarettes (e-cigarettes) is prevalent among adolescents, but there is little knowledge about the consequences of their use. We examined, longitudinally, how e-cigarette use among adolescents is related to subsequent smoking behaviour., METHODS: Longitudinal school-based survey with a baseline sample of 2338 students (9th and 10th graders, mean age 14.7 years) in Hawaii surveyed in 2013 (time 1, T1) and followed up 1 year later (time 2, T2). We assessed e-cigarette use, tobacco cigarette use, and psychosocial covariates (demographics, parental support and monitoring, and sensation seeking and rebelliousness). Regression analyses including the covariates tested whether e-cigarette use was related to the onset of smoking among youth who had never smoked cigarettes, and to change in smoking frequency among youth who had previously smoked cigarettes., RESULTS: Among T1 never-smokers, those who had used e-cigarettes at T1 were more likely to have smoked cigarettes at T2; for a complete-case analysis, adjusted OR=2.87, 95% CI 2.03 to 4.05, $p<0.0001$. Among ever-smokers at T1, using e-cigarettes was not related to significant change in their frequency of smoking at T2. Uptake of e-cigarette use among T1 never-users of either product was predicted by age, Caucasian or Native Hawaiian (vs Asian-American) ethnicity, lower parental education and parental support, higher rebelliousness, and perception of e-cigarettes as healthier., CONCLUSIONS: Adolescents who use e-cigarettes are more likely to start smoking cigarettes. This result together with other findings suggests that policies restricting adolescents' access to e-cigarettes may have a rationale from a public health standpoint.Copyright Published by the BMJ Publishing Group Limited. For permission to use (where not

	already granted under a licence) please go to http://www.bmj.com/company/products-services/rights-and-licensing/.
1343	Caroline Franck, Kristian B Filion, Jonathan Kimmelman, Roland Grad, Mark J Eisenberg Ethical considerations of e-cigarette use for tobacco harm reduction.. Respiratory research; 2016;17:53.<br><br>**Keywords:**<br>*Cigarette Smoking/ae [Adverse Effects],Cigarette Smoking/ec [Economics],Commerce/es [Ethics],*Consumer Product Safety,Direct-to-Consumer Advertising/es [Ethics],Electronic Nicotine Delivery Systems/ec [Economics],*Electronic Nicotine Delivery Systems/es [Ethics],*Harm Reduction/es [Ethics],Humans,Lung Diseases/di [Diagnosis],Lung Diseases/ep [Epidemiology],*Lung Diseases/pc [Prevention & Control],Protective Factors,Risk Assessment,Risk Factors,*Risk Reduction Behavior,*Smoke/ae [Adverse Effects],Smoking Cessation/ec [Economics],*Smoking Cessation/mt [Methods],Vaping/ae [Adverse Effects],Vaping/ec [Economics],*Vaping/es [Ethics]<br><br>**Abstract:** Due to their similarity to tobacco cigarettes, electronic cigarettes (e-cigarettes) could play an important role in tobacco harm reduction. However, the public health community remains divided concerning the appropriateness of endorsing a device whose safety and efficacy for smoking cessation remain unclear. We identified the major ethical considerations surrounding the use of e-cigarettes for tobacco harm reduction, including product safety, efficacy for smoking cessation and reduction, use among non-smokers, use among youth, marketing and advertisement, use in public places, renormalization of a smoking culture, and market ownership. Overall, the safety profile of e-cigarettes is unlikely to warrant serious public health concerns, particularly given the known adverse health effects associated with tobacco cigarettes. As a result, it is unlikely that the population-level harms resulting from e-cigarette uptake among non-smokers would overshadow the public health gains obtained from tobacco harm reduction among current smokers. While the existence of a gateway effect for youth remains uncertain, e-cigarette use in this population should be discouraged. Similarly, marketing and advertisement should remain aligned with the degree of known product risk and should be targeted to current smokers. Overall, the available evidence supports the cautionary implementation of harm reduction interventions aimed at promoting e-cigarettes as attractive and competitive alternatives to cigarette smoking, while taking measures to protect vulnerable groups and individuals.
1358	Lauren M Dutra, Stanton A Glantz E-cigarettes and National Adolescent Cigarette Use: 2004-2014.. Pediatrics; 2017;139:<br><br>**Keywords:**<br>Adolescent,Child,Cross-Sectional Studies,*Electronic Nicotine Delivery Systems/ut [Utilization],Female, Health Surveys,Humans,Male,*Smoking/ep [Epidemiology],United States<br><br>**Abstract:** BACKGROUND: E-cigarette use is rapidly increasing among adolescents in the United States, with some suggesting that e-cigarettes are the cause of declining youth cigarette smoking. We hypothesized that the decline in youth smoking changed after e-cigarettes arrived on the US market in 2007., METHODS: Data were collected by using cross-sectional, nationally representative school-based samples of sixth- through 12th-graders from 2004-2014 National Youth Tobacco Surveys (samples ranged from 16614 in 2013 to 25324 in 2004). Analyses were conducted by using interrupted time series of ever (>=1 puff) and current (last 30 days) cigarette smoking. Logistic regression was used to identify psychosocial risk factors associated with cigarette smoking in the 2004-2009 samples; this model was then applied to estimate the probability of cigarette smoking among cigarette smokers and e-cigarette users in the 2011-2014 samples., RESULTS: Youth cigarette smoking decreased linearly between 2004 and 2014 (P = .009 for ever smoking and P = .05 for current smoking), with no significant change in this trend after 2009 (P = .57 and .23). Based on the psychosocial model of smoking, including demographic characteristics, willingness to wear clothing with a tobacco logo, living with a smoker, likelihood of smoking in the next year, likelihood of smoking cigarettes from a friend, and use of tobacco products

	other than cigarettes or e-cigarettes, the model categorized <25% of current e-cigarette-only users (between 11.0% in 2012 and 23.1% in 2013) as current smokers., CONCLUSIONS: The introduction of e-cigarettes was not associated with a change in the linear decline in cigarette smoking among youth. E-cigarette-only users would be unlikely to have initiated tobacco product use with cigarettes.Copyright © 2017 by the American Academy of Pediatrics.
1362	Benjamin W Chaffee, Elizabeth T Couch, Stuart A Gansky Trends in characteristics and multi-product use among adolescents who use electronic cigarettes, United States 2011-2015.. PloS one; 2017;12:e0177073.<br><br>**Keywords:**<br>Adolescent,Cross-Sectional Studies,*Electronic Nicotine Delivery Systems/td [Trends],*Electronic Nicotine Delivery Systems/ut [Utilization],Female,Health Surveys,Humans,Male,Prevalence,Sex Factors, *Smoking/ep [Epidemiology],United States<br><br>**Abstract:** OBJECTIVES: Evaluate trends from 2011-2015 in electronic cigarette (e-cigarette) use among U.S. adolescents, grades 6-12, including prevalence and associations with past month use of cigarettes and other tobacco products, cigarette smoking intensity, quit attempts, and quit contemplation., METHODS: Five consecutive waves from the National Youth Tobacco Survey (N = 101,011) were used to estimate the grade- and race/ethnicity-standardized prevalence of past month use of e-cigarettes and nine non e-cigarette tobacco products. We assessed linear trends by year and compared outcomes (e.g., tobacco use, smoking intensity) by e-cigarette past month use., RESULTS: Past month e-cigarette use rose sharply from 2011-2015. In all years and both sexes, e-cigarette past month use and ever use were positively associated with use of cigarettes and other tobacco products, with past month e-cigarette use reaching 52% in 2015 among individuals who used >=1 non e-cigarette tobacco product in the past month. Meanwhile, from 2011-2015, the population of adolescent past month e-cigarette users increasingly encompassed adolescents who were not past month users of other products (females: 19.0% to 41.7%; males: 11.1% to 36.7%) or had never used other products (females: 7.1% to 13.5%; males: 6.7% to 15.0%). Among male (but not female) past month cigarette users, there was a statistically significant positive association between past month e-cigarette use and daily cigarette smoking but not in all individual years. Past month e-cigarette use among past month cigarette smokers was not associated with cigarette quit attempts or quit contemplation, with no temporal trend., CONCLUSION: Adolescent past month e-cigarette use is associated with past month use of other tobacco but not with cigarette quit attempts or quit contemplation among cigarette users. Over five years, the average characteristics of U.S. adolescents who use e-cigarettes have shifted, increasingly including more adolescents who do not use non e-cigarette tobacco products.
1365	Nicholas I Goldenson, Adam M Leventhal, Matthew D Stone, Rob S McConnell, Jessica L Barrington-Trimis Associations of Electronic Cigarette Nicotine Concentration With Subsequent Cigarette Smoking and Vaping Levels in Adolescents.. JAMA pediatrics; 2017;171:1192.<br><br>**Keywords:**<br>Adolescent,*Adolescent Behavior,*Electronic Nicotine Delivery Systems/mt [Methods],Electronic Nicotine Delivery Systems/sn [Statistics & Numerical Data],Female,Humans,Longitudinal Studies,Los Angeles/ep [Epidemiology],Male,*Nicotine/ad [Administration & Dosage],Risk Factors,Self Report, *Smoking/ep [Epidemiology],Tobacco Products<br><br>**Abstract:** Importance: Research indicates that electronic cigarette (e-cigarette) use (vaping) among adolescents is associated with the initiation and progression of combustible cigarette smoking. The reasons for this association are unknown., Objective: To evaluate whether use of e-cigarettes with higher nicotine concentrations is associated with subsequent increases in the frequency and intensity of combustible cigarette smoking and vaping., Design, Setting, and Participants: In this prospective cohort study involving students from 10 high schools in the Los Angeles, California, metropolitan area, surveys

	were administered during 10th grade in the spring (baseline) and 11th grade in the fall (6-month follow-up) of 2015 to students who reported using e-cigarettes within the past 30 days and the nicotine concentration level they used at baseline., Exposures: Self-report of baseline e-cigarette nicotine concentration of none (0 mg/mL), low (1-5 mg/mL), medium (6-17 mg/mL), or high (>=18 mg/mL) typically used during the past 30 days., Main Outcomes and Measures: Frequency of combustible cigarette smoking and e-cigarette use within the past 30 days (0 days [none], 1-2 days [infrequent], or >=3 days [frequent]) and daily intensity of smoking and vaping (number of cigarettes smoked per day, number of vaping episodes per day, and number of puffs per vaping episode) at the 6-month follow-up., Results: The analytic sample included 181 students (96 boys [53.0%] and 85 girls [47.0%]; mean [SD] age, 16.1 [0.4] years). Each successive increase in nicotine concentration (none to low, low to medium, and medium to high) vaped was associated with a 2.26 (95% CI, 1.28-3.98) increase in the odds of frequent (vs no) smoking and a 1.65 (95% CI, 1.09-2.51) increase in the odds of frequent (vs no) vaping at follow-up after adjustment for baseline frequency of smoking and vaping and other relevant covariates. Use of e-cigarettes with high (vs no) nicotine concentration was associated with a greater number of cigarettes smoked per day at follow-up (adjusted rate ratio [RR], 7.03; 95% CI, 6.11-7.95). An association with a significantly greater number of vaping episodes per day was found with use of low (adjusted RR, 3.32; 95% CI, 2.61-4.03), medium (adjusted RR, 3.32; 95% CI, 2.54-4.10), and high (adjusted RR, 2.44; 95% CI, 1.63-3.24) nicotine concentrations (vs no nicotine) at baseline. Similar results were found for the number of puffs per vaping episode for low (adjusted RR, 2.05; 95% CI, 1.41-2.70), medium (adjusted RR, 3.39; 95% CI, 2.66-4.11), and high (adjusted RR, 2.23; 95% CI, 1.42-3.03) nicotine concentrations., Conclusions and Relevance: The results of this study provide preliminary evidence that use of e-cigarettes with higher nicotine concentrations by youths may increase subsequent frequency and intensity of smoking and vaping.
1368	Sarah D Kowitt, Clare Meernik, Hannah M Baker, Amira Osman, Li-Ling Huang, Adam O Goldstein Perceptions and Experiences with Flavored Non-Menthol Tobacco Products: A Systematic Review of Qualitative Studies.. International journal of environmental research and public health; 2017;14:<br><br>**Keywords:**<br>*Flavoring Agents,Humans,*Menthol,Qualitative Research,Smoking/ep [Epidemiology],*Smoking/px [Psychology],*Surveys and Questionnaires,Taste,*Tobacco Products<br><br>**Abstract:** Although a few countries have banned flavored cigarettes (except menthol), flavors in most tobacco products remain unregulated across the globe. We conducted a systematic review of qualitative studies examining perceptions of and experiences with flavored non-menthol tobacco products. Of 20 studies on flavored tobacco products included in our qualitative systematic review, 10 examined hookah, six examined e-cigarettes, two examined little cigars and cigarillos (LCCs), and three examined other tobacco products, including cigarettes. The majority of studies, regardless of product type, reported positive perceptions of flavored tobacco products, particularly among young adults and adolescents. In six studies that assessed perceptions of harm (including hookah, LCCs, and other flavored tobacco products), participants believed flavored tobacco products to be less harmful than cigarettes. In studies that examined the role of flavors in experimentation and/or initiation (including three studies on e-cigarettes, one hookah study and one LCC study), participants mentioned flavors as specifically leading to their experimentation and/or initiation of flavored tobacco products. Given that many countries have not yet banned flavors in tobacco products, these findings add to existing research on why individuals use flavored tobacco products and how they perceive harm in flavored tobacco products, providing further support for banning non-menthol flavors in most tobacco products.
1378	Rebecca E Bunnell, Israel T Agaku, Rene A Arrazola, Benjamin J Apelberg, Ralph S Caraballo, Catherine G Corey, Blair N Coleman, Shanta R Dube, Brian A King Intentions to smoke cigarettes among never-smoking US middle and high school electronic cigarette users: National Youth Tobacco Survey, 2011-2013.. Nicotine & tobacco research : official journal of the Society for Research on Nicotine and Tobacco; 2015;17:228.

	**Keywords:**<br>Adolescent,Adolescent Health Services,Child,*Electronic Nicotine Delivery Systems/ut [Utilization], Female,Humans,*Intention,Male,School Health Services,*Smoking/px [Psychology],*Smoking Cessation/sn [Statistics & Numerical Data],*Students/sn [Statistics & Numerical Data],Surveys and Questionnaires,United States<br><br>**Abstract:** INTRODUCTION: Electronic cigarette (e-cigarette) use is increasing rapidly, and the impact on youth is unknown. We assessed associations between e-cigarette use and smoking intentions among US youth who had never smoked conventional cigarettes., METHODS: We analyzed data from the nationally representative 2011, 2012, and 2013 National Youth Tobacco Surveys of students in grades 6-12. Youth reporting they would definitely not smoke in the next year or if offered a cigarette by a friend were defined as not having an intention to smoke; all others were classified as having positive intention to smoke conventional cigarettes. Demographics, pro-tobacco advertisement exposure, ever use of e-cigarettes, and ever use of other combustibles (cigars, hookah, bidis, kreteks, and pipes) and noncombustibles (chewing tobacco, snuff, dip, snus, and dissolvables) were included in multivariate analyses that assessed associations with smoking intentions among never-cigarette-smoking youth., RESULTS: Between 2011 and 2013, the number of never-smoking youth who used e-cigarettes increased 3-fold, from 79,000 to more than 263,000. Intention to smoke conventional cigarettes was 43.9% among ever e-cigarette users and 21.5% among never users. Ever e-cigarette users had higher adjusted odds for having smoking intentions than never users (adjusted odds ratio = 1.70, 95% confidence interval = 1.24-2.32). Those who ever used other combustibles, ever used noncombustibles, or reported pro-tobacco advertisement exposure also had increased odds for smoking intentions., CONCLUSION: In 2013, more than a quarter million never-smoking youth used e-cigarettes. E-cigarette use is associated with increased intentions to smoke cigarettes, and enhanced prevention efforts for youth are important for all forms of tobacco, including e-cigarettes.Copyright Published by Oxford University Press on behalf of the Society for Research on Nicotine and Tobacco 2014. This work is written by (a) US Government employee(s) and is in the public domain in the US.
1387	Christina Jeon, Keum Ji Jung, Heejin Kimm, Sungkyu Lee, Jessica L Barrington-Trimis, Rob McConnell, Jonathan M Samet, Sun Ha Jee E-cigarettes, conventional cigarettes, and dual use in Korean adolescents and university students: Prevalence and risk factors.. Drug and alcohol dependence; 2016;168:99.<br><br>**Keywords:**<br>Adolescent,Adult,Cross-Sectional Studies,*Electronic Nicotine Delivery Systems/ut [Utilization],Female, Humans,Male,Prevalence,Republic of Korea/ep [Epidemiology],Risk Factors,*Smoking/ep [Epidemiology], Students,Surveys and Questionnaires,*Tobacco Products/ut [Utilization],Universities,Young Adult<br><br>**Abstract:** OBJECTIVE: To examine risk factors associated with use of e-cigarettes only, conventional cigarettes only, and dual use in Korean adolescents and young adults., METHODS: In a cross-sectional study, anonymous questionnaires were completed between April-May, 2015 among 1) 2744 middle and high school students, aged 13-18, from Seoul, Incheon, Gyeonggi, and Cheongju, Korea and 2) 2167 university students, aged 19-29, from fourteen universities in Korea., RESULTS: The results show that 12.6% of adolescents and 21.2% of university students reported having ever tried e-cigarettes at least once. Among the ever e-cigarette users, 95.1% and 96.3% of adolescents and university students also tried conventional cigarettes, respectively. Dual users were more likely to be male (adolescents: OR 2.63, 95% CI 1.93-3.57; university students: 4.28, 3.21-5.70), have any close friends who smoke (adolescents: 11.56, 7.63-17.53; university students: 11.29, 5.52-23.10), have any siblings who smoke (adolescents: 3.17, 2.25-4.46; university students: 1.78, 1.30-2.43), and have observed teachers smoke cigarettes at school (adolescents: 1.45, 1.05-2.01)., CONCLUSIONS: A majority of e-cigarette users were dual users. Friends' and siblings' smoking status were significantly associated with dual product use in adolescent and young adult populations. Surveillance of e-cigarette use and implementation of evidence-based

	behavioral interventions targeting adolescents and young adults are necessary.Copyright © 2016 Elsevier Ireland Ltd. All rights reserved.
1392	Jennifer B Unger, Daniel W Soto, Adam Leventhal E-cigarette use and subsequent cigarette and marijuana use among Hispanic young adults.. Drug and alcohol dependence; 2016;163:261.<br><br>**Keywords:**<br>Adolescent,Adult,Electronic Nicotine Delivery Systems/td [Trends],*Electronic Nicotine Delivery Systems/ut [Utilization],Female,*Hispanic Americans/px [Psychology],Humans,Longitudinal Studies,Los Angeles/eh [Ethnology],Male,*Marijuana Smoking/eh [Ethnology],Marijuana Smoking/px [Psychology], Marijuana Smoking/td [Trends],*Smoking/eh [Ethnology],*Smoking/px [Psychology],Smoking/td [Trends],Smoking Cessation/eh [Ethnology],Smoking Cessation/px [Psychology],Surveys and Questionnaires,Tobacco Products,Young Adult<br><br>**Abstract:** PURPOSE: Electronic cigarettes (e-cigarettes) could have a multifaceted effect on public health by changing the likelihood that: (a) non-smokers and non-users of marijuana subsequently transition to cigarette and marijuana use, respectively, and/or: (b) cigarette smokers subsequently quit smoking. We analyzed data from a longitudinal study of Hispanic young adults in Los Angeles, California to determine whether e-cigarette use is associated with subsequent cigarette or marijuana use over a one-year period., METHODS: Survey data were collected from 1332 Hispanic young adults (59% female, mean age=22.7years, SD=0.39years) in 2014 and 2015. Logistic regression analyses examined the association between e-cigarette use in 2014 and cigarette/marijuana use in 2015, controlling for age, sex, and other substance use., RESULTS: In 2014, prevalence of past-month use was 9% for e-cigarettes, 21% for cigarettes, and 23% for marijuana. Among past-month cigarette nonsmokers in 2014, those who were past-month e-cigarette users in 2014 were over 3 times more likely to be past-month cigarette smokers in 2015, compared with those who did not report past-month e-cigarette use in 2014 (26% vs. 7%; OR=3.32, 95% CI=1.55, 7.10). Among past-month marijuana non-users in 2014, those who were past-month e-cigarette users in 2014 were nearly 2 times more likely to be past-month marijuana users in 2015 (24% vs. 12%;OR=1.97, 95% CI=1.01, 3.86). Among past-month cigarette and marijuana users in 2014, e-cigarette use in 2014 was not associated with a change cigarette and marijuana use, respectively, in 2015., CONCLUSIONS: Among Hispanic young adults, e-cigarettes could increase the likelihood of transitioning from non-user to user of cigarettes or marijuana and was not associated with smoking cessation.Copyright © 2016 Elsevier Ireland Ltd. All rights reserved.
1404	Dorota Kaleta, Piotr Wojtysiak, Kinga Polanska Use of electronic cigarettes among secondary and high school students from a socially disadvantaged rural area in Poland.. BMC public health; 2016;15:703.<br><br>**Keywords:**<br>Adolescent,*Adolescent Behavior,Adult,Alcohol Drinking,*Electronic Nicotine Delivery Systems/ut [Utilization],Female,Health Knowledge, Attitudes, Practice,Humans,Male,Poland/ep [Epidemiology], Prevalence,Risk-Taking,*Rural Population,Schools,Sex Factors,*Smoking/ep [Epidemiology],Social Environment,Socioeconomic Factors,*Students,Surveys and Questionnaires,Tobacco Products, *Vulnerable Populations,Young Adult<br><br>**Abstract:** BACKGROUND: The use of e-cigarettes has been growing and has become a significant public health concern. Prevention of the youth access to, initiation and continuous use of e-cigarettes with special attention to vulnerable groups is a subject of a health debate. However, still little is known about characteristics of the underage e-cigarette users from different geographic and socio-economic backgrounds as well as other potential factors associated with the use of e-cigarettes, including simultaneous use with tobacco products or alcohol. The aim of the study was to investigate the prevalence and factors associated with ever and continued e-cigarette use among the secondary and high school students from a socially disadvantaged rural area in Poland., METHODS: The study sample consisted of 3552 students aged 13-19 years from Piotrkowski district. The anonymous, self-administered

	questionnaire adapted from the Global Youth Tobacco Survey was implemented to collect relevant information. The uni- and multivariate logistic regression analyses were applied to identify factors associated with ever, current (in the previous 30 days) and continued e-cigarette use., RESULTS: Almost 22 % of the sample reported ever e-cigarettes use and 27 % of the respondents indicated e-cigarettes use in the past month. Boys, in comparison with girls, were more likely to report current e-cigarette use (OR=1.7; p<0.001). Current e-cigarette use was strongly associated with alcohol consumption (OR=4.3; p<0.001), current (OR=32.5; p<0.001) and ever tobacco smoking (OR=7.5; p<0.001) as well as smoking parents (OR=1.4; p<0.05) and friends (OR=4.5; p<0.05). The use of e-cigarettes was also significantly associated with harm perception (p<0.001). A similar pattern was observed among ever e-cigarette users. Male gender (OR=1.4; p<0.05), current tobacco smoking (OR=3.0; p<0.01) and lack of knowledge about a ban on smoking in the school (OR=1.4; p<0.05) were predictors of the continued e-cigarette use. Higher paternal education (OR=0.5; p<0.001) and perception of e-cigarettes as more harmful comparing to tobacco (OR=0.2; p<0.001) protected from the continued e-cigarette use., CONCLUSIONS: E-cigarette use is widespread in the investigated population, especially among boys, those with other risky behaviors and with smoking parents or friends. There is a need for further research and preventive policies to protect the youth from that exposure.
1410	David Hammond, Jessica L Reid, Adam G Cole, Scott T Leatherdale Electronic cigarette use and smoking initiation among youth: a longitudinal cohort study.. CMAJ : Canadian Medical Association journal = journal de l'Association medicale canadienne; 2017;189:E1328.<br><br>**Keywords:**<br>Adolescent,*Adolescent Behavior/ph [Physiology],Alberta/ep [Epidemiology],*Behavior, Addictive/ep [Epidemiology],Behavior, Addictive/px [Psychology],Cohort Studies,Electronic Nicotine Delivery Systems/px [Psychology],*Electronic Nicotine Delivery Systems/sn [Statistics & Numerical Data],Female, Humans,Longitudinal Studies,Male,Ontario/ep [Epidemiology],Peer Group,Risk Factors,Self Report, *Smoking/ep [Epidemiology],Smoking/px [Psychology],Tobacco Use Disorder/pc [Prevention & Control]<br><br>**Abstract:** BACKGROUND: The influence of e-cigarette use on smoking initiation is a highly controversial issue, with limited longitudinal data available for examining temporal associations. We examined e-cigarette use and its association with cigarette-smoking initiation at 1-year follow-up within a large cohort of Canadian secondary school students., METHODS: We analyzed data from students in grades 9-12 who participated in 2 waves of COMPASS, a cohort study of purposefully sampled secondary schools in Ontario and Alberta, Canada, at baseline (2013/14) and 1-year follow-up (2014/15). We assessed cigarette smoking and e-cigarette use at baseline and follow-up using self-completed surveys. We used generalized linear mixed-effects models to examine correlates of past-30-day e-cigarette use at baseline and smoking initiation between waves within the longitudinal sample., RESULTS: Past-30-day e-cigarette use increased from 2013/14 to 2014/15 (7.2% v. 9.7%, p < 0.001), whereas past-30-day cigarette smoking decreased slightly (11.4% v. 10.8%, p = 0.02). Among the 44 163 students evaluated at baseline, past-30-day e-cigarette use was strongly associated with smoking status and smoking susceptibility. In the longitudinal sample (n = 19 130), past-30-day use of e-cigarettes at baseline was associated with initiation of smoking a whole cigarette (adjusted odds ratio [OR] 2.12, 95% confidence interval [CI] 1.68-2.66) and with initiation of daily smoking (adjusted OR 1.79, 95% CI 1.41-2.28) at follow-up., INTERPRETATION: E-cigarette use was strongly associated with cigarette smoking behaviour, including smoking initiation at follow-up. The causal nature of this association remains unclear, because common factors underlying the use of both e-cigarettes and conventional cigarettes may also account for the temporal order of initiation.Copyright © 2017 Joule Inc. or its licensors.
1411	Alexis D Henry, John Gettens, Judith A Savageau, Doris Cullen, Anna Landau Massachusetts Medicaid members that smoked in 2008: Characteristics associated with smoking status in 2014.. PloS one; 2017;12:e0186144.<br><br>**Keywords:**

	Adult,Aged,Female,Health Care Reform,Humans,Male,Massachusetts/ep [Epidemiology],Medicaid/ec [Economics],*Medicaid/og [Organization & Administration],Middle Aged,Patient Protection and Affordable Care Act,Smoking/ec [Economics],*Smoking/ep [Epidemiology],Smoking Prevention,Surveys and Questionnaires,*Tobacco Use Cessation/mt [Methods],United States/ep [Epidemiology],Young Adult<br><br>**Abstract:** The smoking rate among non-elderly Medicaid enrollees is more than double the rate for those privately insured; smoking-related conditions account for 15% of Medicaid expenditures. Under state health reform, Massachusetts Medicaid (MassHealth) made tobacco cessation treatment available beginning in 2006. We used surveys conducted in 2008 and 2014 to examine changes in smoking abstinence rates among MassHealth members identified as smokers and to identify factors associated with being a former smoker. Members previously identified as smokers were surveyed by mail or phone; 2008 and 2014 samples included 3,116 and 2,971 members, respectively. Surveys collected demographic and health information, asked members whether they smoked cigarettes "every day, some days or not at all', and asked questions to assess smoking intensity among current smokers. The 2014 survey included an open ended-question asking members "what helped the most" in quitting or quit attempts. We observed a significant decrease in members reporting smoking "every/some days" of 15.5 percentage points ($p < .0001$) from 2008 to 2014, and a significant decrease in smokers reporting smoking "more than 10 cigarettes on days smoked" of 16.7 percentage points ($p < .0001$). Compared to smokers, former smokers more frequently reported health concerns, the influence of family members, and the use of e-cigarettes as helping the most in quitting. Expanded access to tobacco cessation treatment under the Affordable Care Act may have help to reduce the high smoking rates among Medicaid enrollees. Additionally, smokers' concerns about health and the influence of family and friends provide opportunities for targeted intervention and messaging about quitting.
1428	Thomas A Wills, Frederick X Gibbons, James D Sargent, Rebecca J Schweitzer How is the effect of adolescent e-cigarette use on smoking onset mediated: A longitudinal analysis.. Psychology of addictive behaviors : journal of the Society of Psychologists in Addictive Behaviors; 2016;30:876.<br><br>**Keywords:**<br>Adolescent,Age of Onset,*Electronic Nicotine Delivery Systems/px [Psychology],Female,Humans,Male,*Marijuana Smoking/px [Psychology],Nicotine,*Peer Group,Prospective Studies,*Smoking/px [Psychology],Students/sn [Statistics & Numerical Data],Surveys and Questionnaires<br><br>**Abstract:** E-cigarette use by adolescents has been related to onset of cigarette smoking but there is little knowledge about the process(es) through which this occurs. Accordingly, we tested the role of cognitive and social factors for mediating the relation between e-cigarette use and smoking onset. A school-based survey was conducted with a baseline sample of 2,338 students in Hawaii (9th and 10th graders, mean age 14.7 years) who were surveyed in 2013 (Time 1, T1) and followed up 1 year later (Time 2, T2). We assessed e-cigarette use, cigarette smoking, demographic covariates, and 4 hypothesized mediators: smoking-related expectancies, prototypes, and peer affiliations as well as marijuana use. The primary structural modeling analysis, based on initial never-smokers, used an autoregressive model (entering T2 mediator values adjusted for T1 values) to test for mediational pathways in the relation between e-cigarette use at T1 and cigarette smoking status at T2. Results showed that e-cigarette use was related to all of the mediators. Tests of indirect effects indicated that changes in expectancies, affiliations, and marijuana use were significant pathways in the relation between e-cigarette use and smoking onset. A direct effect from e-cigarette use to smoking onset was nonsignificant. Findings were replicated across autoregressive and prospective models. We conclude that the relation between adolescent e-cigarette use and smoking onset is in part attributable to cognitive and social processes that follow from e-cigarette use. Further research is needed to understand the relative role of nicotine and psychosocial factors in smoking onset. (PsycINFO Database RecordCopyright (c) 2017 APA, all rights reserved).
1431	Jieming Zhong, Shuangshuang Cao, Weiwei Gong, Fangrong Fei, Meng Wang Electronic Cigarettes Use and Intention to Cigarette Smoking among Never-Smoking Adolescents and Young Adults: A Meta-

	Analysis.. International journal of environmental research and public health; 2016;13:<br><br>**Keywords:**<br>Adolescent,Adult,Child,Electronic Nicotine Delivery Systems/px [Psychology],*Electronic Nicotine Delivery Systems/ut [Utilization],Humans,*Intention,Odds Ratio,*Smoking/px [Psychology],Young Adult<br><br>**Abstract:** Electronic cigarettes (e-cigarettes) use is becoming increasingly common, especially among adolescents and young adults, and there is little evidence on the impact of e-cigarettes use on never-smokers. With a meta-analysis method, we explore the association between e-cigarettes use and smoking intention that predicts future cigarette smoking. Studies were identified by searching three databases up to January 2016. The meta-analysis results were presented as pooled odds ratio (OR) with 95% confidence interval (CI) calculated by a fixed-effects model. A total of six studies (91,051 participants, including 1452 with ever e-cigarettes use) were included in this meta-analysis study. We found that never-smoking adolescents and young adults who used e-cigarettes have more than 2 times increased odds of intention to cigarette smoking (OR = 2.21, 95% CI: 1.86-2.61) compared to those who never used, with low evidence of between-study heterogeneity (p = 0.28, I2 = 20.1%). Among never-smoking adolescents and young adults, e-cigarettes use was associated with increased smoking intention.
1446	Samir Soneji, Jessica L Barrington-Trimis, Thomas A Wills, Adam M Leventhal, Jennifer B Unger, Laura A Gibson, JaeWon Yang, Brian A Primack, Judy A Andrews, Richard A Miech, Tory R Spindle, Danielle M Dick, Thomas Eissenberg, Robert C Hornik, Rui Dang, James D Sargent Association Between Initial Use of e-Cigarettes and Subsequent Cigarette Smoking Among Adolescents and Young Adults: A Systematic Review and Meta-analysis.. JAMA pediatrics; 2017;171:788.<br><br>**Keywords:**<br>Adolescent,*Adolescent Behavior,Disease Progression,Electronic Nicotine Delivery Systems/px [Psychology],*Electronic Nicotine Delivery Systems/sn [Statistics & Numerical Data],Female,Humans, Male,Risk Factors,*Smoking/ep [Epidemiology],Smoking/px [Psychology],Smoking Cessation/sn [Statistics & Numerical Data],*Tobacco Use Disorder/ep [Epidemiology],Tobacco Use Disorder/px [Psychology], Young Adult<br><br>**Abstract:** Importance: The public health implications of e-cigarettes depend, in part, on whether e-cigarette use affects the risk of cigarette smoking., Objective: To perform a systematic review and meta-analysis of longitudinal studies that assessed initial use of e-cigarettes and subsequent cigarette smoking., Data Sources: PubMed, EMBASE, Cochrane Library, Web of Science, the 2016 Society for Research on Nicotine and Tobacco 22nd Annual Meeting abstracts, the 2016 Society of Behavioral Medicine 37th Annual Meeting & Scientific Sessions abstracts, and the 2016 National Institutes of Health Tobacco Regulatory Science Program Conference were searched between February 7 and February 17, 2017. The search included indexed terms and text words to capture concepts associated with e-cigarettes and traditional cigarettes in articles published from database inception to the date of the search., Study Selection: Longitudinal studies reporting odds ratios for cigarette smoking initiation associated with ever use of e-cigarettes or past-30-day cigarette smoking associated with past-30-day e-cigarette use. Searches yielded 6959 unique studies, of which 9 met inclusion criteria (comprising 17389 adolescents and young adults)., Data Extraction and Synthesis: Study quality and risk of bias were assessed using the Newcastle-Ottawa Scale and the Risk of Bias in Non-randomized Studies of Interventions tool, respectively. Data and estimates were pooled using random-effects meta-analysis., Main Outcomes and Measures: Among baseline never cigarette smokers, cigarette smoking initiation between baseline and follow-up. Among baseline non-past-30-day cigarette smokers who were past-30-day e-cigarette users, past-30-day cigarette smoking at follow-up., Results: Among 17389 adolescents and young adults, the ages ranged between 14 and 30 years at baseline, and 56.0% were female. The pooled probabilities of cigarette smoking initiation were 30.4% for baseline ever e-cigarette users and 7.9% for baseline never e-cigarette users. The pooled probabilities of past-30-day cigarette smoking at follow-up were 21.5% for

	baseline past-30-day e-cigarette users and 4.6% for baseline non-past-30-day e-cigarette users. Adjusting for known demographic, psychosocial, and behavioral risk factors for cigarette smoking, the pooled odds ratio for subsequent cigarette smoking initiation was 3.62 (95% CI, 2.42-5.41) for ever vs never e-cigarette users, and the pooled odds ratio for past-30-day cigarette smoking at follow-up was 4.28 (95% CI, 2.52-7.27) for past-30-day e-cigarette vs non-past-30-day e-cigarette users at baseline. A moderate level of heterogeneity was observed among studies (I2=60.1%)., Conclusions and Relevance: e-Cigarette use was associated with greater risk for subsequent cigarette smoking initiation and past-30-day cigarette smoking. Strong e-cigarette regulation could potentially curb use among youth and possibly limit the future population-level burden of cigarette smoking.
1447	Hongying Dai Attitudes Toward Tobacco 21 Among US Youth.. Pediatrics; 2017;140:<br><br>**Keywords:**<br>Adolescent,Age Factors,*Attitude to Health,Child,Cross-Sectional Studies,Female,Humans,Male,Self Report,*Smoking/lj [Legislation & Jurisprudence],*Smoking Prevention,Social Control, Formal,United States<br><br>**Abstract:** BACKGROUND: An important movement in reducing youth smoking is to restrict the supply of cigarettes to youth by raising the minimum age of sales to 21, termed Tobacco 21. This study examined attitudes toward Tobacco 21 among youth and their correlations with tobacco use., METHODS: Data from the 2015 National Youth Tobacco Survey (n = 17092, the typical age of 11-18 years) were analyzed to examine the prevalence of support toward Tobacco 21 among youth. This study further assessed whether attitudes toward Tobacco 21 were associated with (1) intention to initiate cigarette smoking among never-smoking youth (n = 16449); and (2) intention to quit tobacco use among current tobacco users (n = 2914)., RESULTS: Approximately 63.9% of respondents reported supporting Tobacco 21. Support for Tobacco 21 was higher among middle school students (versus high school students), girls (versus boys), and noncurrent users of cigarettes or electronic cigarettes (versus current users). Youth support attitudes were significantly associated with perceptions of tobacco's danger and tobacco use by household members. Never smokers who supported Tobacco 21 had lower odds of intention to initiate cigarette use (adjusted odds ratio = 0.2, P < .0001). Current tobacco users who supported Tobacco 21 had higher odds of intention to quit tobacco use in the next 12 months (adjusted odds ratio = 2.6, P < .0001)., CONCLUSIONS: Tobacco 21 is supported by the majority of youth nationwide and youth attitudes were correlated with smoking behaviors. Education programs about the harm of smoking and nicotine addiction at the early stages of life may help increase support of this policy among young tobacco users.Copyright © 2017 by the American Academy of Pediatrics.
1448	Ahmed Jamal, Andrea Gentzke, S Sean Hu, Karen A Cullen, Benjamin J Apelberg, David M Homa, Brian A King Tobacco Use Among Middle and High School Students - United States, 2011-2016.. MMWR. Morbidity and mortality weekly report; 2017;66:597.<br><br>**Keywords:**<br>Adolescent,Child,Cross-Sectional Studies,Female,Health Surveys,Humans,Male,*Smoking/ep [Epidemiology],*Students/sn [Statistics & Numerical Data],United States/ep [Epidemiology]<br><br>**Abstract:** Tobacco use is the leading cause of preventable disease and death in the United States; nearly all tobacco use begins during youth and young adulthood (1,2). Among youths, use of tobacco products in any form is unsafe (1,3). CDC and the Food and Drug Administration (FDA) analyzed data from the 2011-2016 National Youth Tobacco Surveys (NYTS) to determine recent patterns of current (past-30-day) use of seven tobacco product types among U.S. middle (grades 6-8) and high (grades 9-12) school students. In 2016, 20.2% of surveyed high school students and 7.2% of middle school students reported current tobacco product use. In 2016, among current tobacco product users, 47.2% of high school students and 42.4% of middle school students used >=2 tobacco products, and electronic cigarettes (e-cigarettes) were the most commonly used tobacco product among high (11.3%) and middle (4.3%) school students.

	Current use of any tobacco product did not change significantly during 2011-2016 among high or middle school students, although combustible tobacco product use declined. However, during 2015-2016, among high school students, decreases were observed in current use of any tobacco product, any combustible product, >=2 tobacco products, e-cigarettes, and hookahs. Among middle school students, current use of e-cigarettes decreased. Comprehensive and sustained strategies can help prevent and reduce the use of all forms of tobacco products among U.S. youths (1-3).
1458	Jung Ah Lee, Sungkyu Lee, Hong-Jun Cho The Relation between Frequency of E-Cigarette Use and Frequency and Intensity of Cigarette Smoking among South Korean Adolescents.. International journal of environmental research and public health; 2017;14:<br><br>**Keywords:**<br>Adolescent,*Electronic Nicotine Delivery Systems/sn [Statistics & Numerical Data],Female,Humans, Intention,Male,Motivation,Prevalence,Republic of Korea/ep [Epidemiology],*Smoking/ep [Epidemiology], Surveys and Questionnaires,*Tobacco Products/sn [Statistics & Numerical Data],*Vaping/ep [Epidemiology]<br><br>**Abstract:** INTRODUCTION: The prevalence of adolescent electronic cigarette (e-cigarette) use has increased in most countries. This study aims to determine the relation between the frequency of e-cigarette use and the frequency and intensity of cigarette smoking. Additionally, the study evaluates the association between the reasons for e-cigarette use and the frequency of its use., MATERIALS AND METHODS: Using the 2015 Korean Youth Risk Behavior Web-Based Survey, we included 6655 adolescents with an experience of e-cigarette use who were middle and high school students aged 13-18 years. We compared smoking experience, the frequency and intensity of cigarette smoking, and the relation between the reasons for e-cigarette uses and the frequency of e-cigarette use., RESULTS: The prevalence of e-cigarette ever and current (past 30 days) users were 10.1% and 3.9%, respectively. Of the ever users, approximately 60% used e-cigarettes not within 1 month. On the other hand, 8.1% used e-cigarettes daily. The frequent and intensive cigarette smoking was associated with frequent e-cigarette uses. The percentage of frequent e-cigarette users (>=10 days/month) was 3.5% in adolescents who did not smoke within a month, but 28.7% among daily smokers. Additionally, it was 9.1% in smokers who smoked less than 1 cigarette/month, but 55.1% in smokers who smoked >=20 cigarettes/day. The most common reason for e-cigarette use was curiosity (22.9%), followed by the belief that they are less harmful than conventional cigarettes (18.9%), the desire to quit smoking (13.1%), and the capacity for indoor use (10.7%). Curiosity was the most common reason among less frequent e-cigarette users; however, the desire to quit smoking and the capacity for indoor use were the most common reasons among more frequent users., CONCLUSIONS: Results showed a positive relation between frequency or intensity of conventional cigarette smoking and the frequency of e-cigarette use among Korean adolescents, and frequency of e-cigarette use differed according to the reason for the use of e-cigarettes.
1469	Zewditu Demissie, Sherry Everett Jones, Heather B Clayton, Brian A King Adolescent Risk Behaviors and Use of Electronic Vapor Products and Cigarettes.. Pediatrics; 2017;139:<br><br>**Keywords:**<br>Adolescent,Cross-Sectional Studies,*Electronic Nicotine Delivery Systems/ae [Adverse Effects],Electronic Nicotine Delivery Systems/px [Psychology],*Electronic Nicotine Delivery Systems/ut [Utilization],Female, Health Behavior,Health Surveys,Humans,Male,*Risk-Taking,Smoking/ep [Epidemiology],Smoking/px [Psychology],United States,Utilization Review/sn [Statistics & Numerical Data]<br><br>**Abstract:** BACKGROUND: Adolescent use of tobacco in any form is unsafe; yet the use of electronic cigarettes and other electronic vapor products (EVPs) has increased in recent years among this age group. We assessed the prevalence and frequency of cigarette smoking and EVP use among high school students, and associations between health-risk behaviors and both cigarette smoking and EVP use., METHODS: We used 2015 national Youth Risk Behavior Survey data (N = 15624) to classify students into 4

	mutually exclusive categories of smoking and EVP use based on 30-day use: nonuse, cigarette smoking only, EVP use only, and dual use. Prevalence of cigarette smoking and EVP use were assessed overall and by student demographics and frequency of use. Prevalence ratios were calculated to identify associations with health risk-behaviors., RESULTS: In 2015, 73.5% of high school students did not smoke cigarettes or use EVPs, 3.2% smoked cigarettes only, 15.8% used EVPs only, and 7.5% were dual users. Frequency of cigarette smoking and EVP use was greater among dual users than cigarette-only smokers and EVP-only users. Cigarette-only smokers, EVP-only users, and dual users were more likely than nonusers to engage in several injury, violence, and substance use behaviors; have >=4 lifetime sexual partners; be currently sexually active; and drink soda >=3 times/day. Only dual users were more likely than nonusers not to use a condom at last sexual intercourse., CONCLUSIONS: EVP use, alone and concurrent with cigarette smoking, is associated with health-risk behaviors among high school students.Copyright © 2017 by the American Academy of Pediatrics.
1477	Marilyn Urrutia-Pereira, Vinicius J Oliano, Carolina S Aranda, Javier Mallol, Dirceu Sole Prevalence and factors associated with smoking among adolescents.. Jornal de pediatria; 2017;93:230.<br><br>**Keywords:**<br>Adolescent,Adult,Brazil/ep [Epidemiology],Cross-Sectional Studies,Female,Health Knowledge, Attitudes, Practice,Humans,Male,Prevalence,Prospective Studies,Risk Factors,*Smoking/ep [Epidemiology],Smoking Prevention,Surveys and Questionnaires,Young Adult<br><br>**Abstract:** OBJECTIVE: Despite anti-smoking prevention programs, many adolescents start smoking at school age. The main objectives of this study were to determine the prevalence and risk factors associated with smoking in adolescents living in Uruguaiana, RS, Brazil., METHODS: A prospective study was conducted in adolescents (12-19 years), enrolled in municipal schools, who answered a self-administered questionnaire on smoking., RESULTS: 798 adolescents were enrolled in the study, with equal distribution between genders. The tobacco experimentation frequency (ever tried a cigarette, even one or two puffs) was 29.3%; 14.5% started smoking before 12 years of age and 13.0% reported smoking at least one cigarette/day last month. Having a smoking friend (OR: 5.67, 95% CI: 2.06-7.09), having cigarettes offered by friends (OR: 4.21, 95% CI: 2.46-5.76) and having easy access to cigarettes (OR: 3.82, 95% CI: 1.22-5.41) was identified as factors associated with smoking. Having parental guidance on smoking (OR: 0.67, 95% CI: 0.45-0.77), having no contact with cigarettes at home in the last week (OR: 0.51, 95% CI: 0.11-0.79) and knowing about the dangers of electronic cigarettes (OR: 0.88, 95% CI: 0.21-0.92) were identified as protection factors., CONCLUSION: The prevalence of smoking among adolescents in Uruguaiana is high. The implementation of measures to reduce/stop tobacco use and its new forms of consumption, such as electronic cigarettes and hookah, are urgent and imperative in schools.Copyright © 2016 Sociedade Brasileira de Pediatria. Published by Elsevier Editora Ltda. All rights reserved.
1506	George Mammen, Jurgen Rehm, Sergio Rueda Vaporizing cannabis through e-cigarettes: Prevalence and socio-demographic correlates among Ontario high school students.. Canadian journal of public health = Revue canadienne de sante publique; 2016;107:e337.<br><br>**Keywords:**<br>Adolescent,*Cannabis/ch [Chemistry],Cross-Sectional Studies,*Electronic Nicotine Delivery Systems/ut [Utilization],Female,Health Surveys,Humans,Male,*Marijuana Abuse/ep [Epidemiology],Ontario/ep [Epidemiology],Prevalence,Risk Factors,Schools,Socioeconomic Factors,*Students/px [Psychology], Students/sn [Statistics & Numerical Data],Volatilization<br><br>**Abstract:** No abstract available.
1510	Jun Hyun Hwang, Soon-Woo Park Association between Peer Cigarette Smoking and Electronic Cigarette Smoking among Adolescent Nonsmokers: A National Representative Survey.. PloS one; 2016;11:e0162557.

	**Keywords:**<br>Adolescent,Electronic Nicotine Delivery Systems/ut [Utilization],*Electronic Nicotine Delivery Systems, Female,Humans,Male,*Peer Group,Republic of Korea/ep [Epidemiology],*Smoking/ep [Epidemiology], Smoking/px [Psychology],Surveys and Questionnaires<br><br>**Abstract:** We assessed the association between electronic cigarette (e-cigarette) use and peer cigarette smoking, a major risk factor for the initiation of cigarette smoking in adolescents. Data from the 2013 Korea Youth Risk Behavior Web-based Survey of 65,753 nonsmokers aged 13-18 years were analyzed using multiple logistic regression. A total of 3.8% of the Korean adolescents were 'ever e-cigarette' users and 1.2% were current users. Adjusted odds ratios (ORs) for current and ever e-cigarette use compared to those whose closest friends were non-smokers ranged from 2.05 (95% confidence interval [CI], 1.82-2.30) to 5.50 (95% CI, 4.77-6.34), and from 2.23 (95% CI, 1.77-2.81) to 7.82 (95% CI, 5.97-10.25) for those who had 'some' close friends to 'most/all' friends who smoked, respectively. The slopes of the adjusted ORs for e-cigarette use in 'never smokers' were more than twice as steep as those in 'former smokers', showing a significant interaction effect between the proportion of smoking closest friends and cigarette smoking status (never or former smokers) (p<0.001 for interaction). Peer cigarette smoking had a significant association with e-cigarette use in adolescent nonsmokers, and this association was greater on never smokers than former smokers.
1517	John Britton Electronic cigarettes and smoking cessation in England.. BMJ (Clinical research ed.); 2016;354:i4819.<br><br>**Keywords:**<br>*Electronic Nicotine Delivery Systems/sn [Statistics & Numerical Data],England/ep [Epidemiology], Humans,Prevalence,*Smoking/ep [Epidemiology],*Smoking Cessation/sn [Statistics & Numerical Data]<br><br>**Abstract:**
1521	John Hickner What do we really know about e-cigarettes?.. The Journal of family practice; 2016;65:372.<br><br>**Keywords:**<br>Attitude to Health,*Electronic Nicotine Delivery Systems,Health Behavior,*Health Knowledge, Attitudes, Practice,Humans,*Patient Education as Topic/mt [Methods],*Physicians, Primary Care/sn [Statistics & Numerical Data],Product Labeling,Secondary Prevention,*Smoking Prevention,Substance Withdrawal Syndrome/pc [Prevention & Control],*Tobacco Use Disorder/pc [Prevention & Control]<br><br>**Abstract:** Are e-cigarettes a "gateway" to the use of tobacco and other substances of abuse? Or do they represent an effective way to quit smoking?
1526	Matthew B Stanbrook Electronic cigarettes and youth: a gateway that must be shut.. CMAJ : Canadian Medical Association journal = journal de l'Association medicale canadienne; 2016;188:785.<br><br>**Keywords:**<br>Adolescent,Canada/ep [Epidemiology],*Electronic Nicotine Delivery Systems/sn [Statistics & Numerical Data],Humans,*Tobacco Use/ep [Epidemiology],Tobacco Use/pc [Prevention & Control]<br><br>**Abstract:**
1538	Jessica L Barrington-Trimis, Robert Urman, Kiros Berhane, Jennifer B Unger, Tess Boley Cruz, Mary Ann Pentz, Jonathan M Samet, Adam M Leventhal, Rob McConnell E-Cigarettes and Future Cigarette Use.. Pediatrics; 2016;138:<br><br>**Keywords:**

	Adolescent,*Electronic Nicotine Delivery Systems/sn [Statistics & Numerical Data],*Electronic Nicotine Delivery Systems/ut [Utilization],Female,Humans,Male,Prospective Studies,Self Report,*Smoking/ep [Epidemiology],*Tobacco Products/sn [Statistics & Numerical Data],*Tobacco Products/ut [Utilization], Young Adult<br><br>**Abstract:** BACKGROUND: There has been little research examining whether e-cigarette use increases the risk of cigarette initiation among adolescents in the transition to adulthood when the sale of cigarettes becomes legal., METHODS: The Children's Health Study is a prospectively followed cohort in Southern California. Data on e-cigarette use were collected in 11th and 12th grade (mean age = 17.4); follow-up data on tobacco product use were collected an average of 16 months later from never-smoking e-cigarette users at initial evaluation (n = 146) and from a sample of never-smoking, never e-cigarette users (n = 152) frequency matched to e-cigarette users on gender, ethnicity, and grade., RESULTS: Cigarette initiation during follow-up was reported by 40.4% of e-cigarette users (n = 59) and 10.5% of never users (n = 16). E-cigarette users had 6.17 times (95% confidence interval: 3.30-11.6) the odds of initiating cigarettes as never e-cigarette users. Results were robust to adjustment for potential confounders and in analyses restricted to never users of any combustible tobacco product. Associations were stronger in adolescents with no intention of smoking at initial evaluation. E-cigarette users were also more likely to initiate use of any combustible product (odds ratio = 4.98; 95% confidence interval: 2.37-10.4), including hookah, cigars, or pipes., CONCLUSIONS: E-cigarette use in never-smoking youth may increase risk of subsequent initiation of cigarettes and other combustible products during the transition to adulthood when the purchase of tobacco products becomes legal. Stronger associations in participants with no intention of smoking suggests that e-cigarette use was not simply a marker for individuals who would have gone on to smoke regardless of e-cigarette use.Copyright © 2016 by the American Academy of Pediatrics.
1544	Ingrid Torjesen E-cigarettes are a "gateway from smoking," RCP concludes.. BMJ (Clinical research ed.); 2016;353:i2392.<br><br>**Keywords:**<br>Electronic Nicotine Delivery Systems/ae [Adverse Effects],*Electronic Nicotine Delivery Systems/px [Psychology],Humans,*Smoking/px [Psychology],Smoking Cessation/mt [Methods],*Smoking Cessation/px [Psychology],Societies, Medical,*Tobacco Use Disorder/et [Etiology],United Kingdom<br><br>**Abstract:**
1545	John Britton, Deborah Arnott, Ann McNeill, Nicholas Hopkinson,Tobacco Advisory Group of the Royal College of Physicians Nicotine without smoke-putting electronic cigarettes in context.. BMJ (Clinical research ed.); 2016;353:i1745.<br><br>**Keywords:**<br>*Electronic Nicotine Delivery Systems,Health Promotion,Humans,*Nicotine/ad [Administration & Dosage],Nicotine/ae [Adverse Effects],Smoke,Smoking/ae [Adverse Effects],*Smoking Prevention,United Kingdom<br><br>**Abstract:**
1563	Anonymous Teens Using E-Cigarettes May Be More Likely to Start Smoking Tobacco.. Home healthcare now; 2016;34:9.<br><br>**Keywords:**<br>Adolescent,Adolescent Behavior,*Electronic Nicotine Delivery Systems/px [Psychology],Electronic Nicotine Delivery Systems/sn [Statistics & Numerical Data],Female,Humans,Male,Predictive Value of Tests,Prevalence,*Risk-Taking,Smoking/ep [Epidemiology],*Smoking/px [Psychology]

	**Abstract:**
1568	Georgios Chatzopoulos Smoking, Smokeless Tobacco, and Alcohol Consumption as Contributing Factors to Periodontal Disease.. Northwest dentistry; 2016;95:37.<br><br>**Keywords:**<br>*Alcohol Drinking/ae [Adverse Effects],Alcoholism/co [Complications],Electronic Nicotine Delivery Systems/ae [Adverse Effects],Humans,*Periodontal Diseases/et [Etiology],*Smoking/ae [Adverse Effects],*Tobacco Use/ae [Adverse Effects],*Tobacco, Smokeless/ae [Adverse Effects]<br><br>**Abstract:**
1570	Michael F Pesko, Jenna M Hughes, Fatima S Faisal The influence of electronic cigarette age purchasing restrictions on adolescent tobacco and marijuana use.. Preventive medicine; 2016;87:207.<br><br>**Keywords:**<br>Adolescent,Age Factors,*Commerce/lj [Legislation & Jurisprudence],*Electronic Nicotine Delivery Systems/ut [Utilization],Humans,*Marijuana Smoking,Smoking,Surveys and Questionnaires,Tobacco Products,Tobacco, Smokeless,United States<br><br>**Abstract:** OBJECTIVE: In the United States, many states have established minimum legal purchase ages for electronic nicotine delivery systems (ENDS) to ban adolescent purchases, but these policies may also affect other related substance use. We explore whether ENDS are substitutes or complements for cigarettes, cigars, smokeless tobacco, and marijuana among adolescents by using variation in state-level implementation of ENDS age purchasing restrictions., METHODS: We linked data on ENDS age purchasing restrictions to state- and year-specific rates of adolescent tobacco and marijuana use in 2007-2013 from the Youth Risk Behavior Surveillance System. This data provides a nationally representative sample of adolescents who attend public and private schools. We performed a fixed effect regression analysis exploring the influence of ENDS age purchasing restrictions on outcomes of tobacco use and marijuana use, controlling for state and year fixed characteristics, age-race cohorts, cigarette excise taxes, and cigarette indoor use restrictions., RESULTS: For cigarette use, we separate our results into cigarette use frequency. We found causal evidence that ENDS age purchasing restrictions increased adolescent regular cigarette use by 0.8 percentage points. ENDS age purchasing restrictions were not associated with cigar use, smokeless tobacco use, or marijuana use., CONCLUSIONS: We document a concerning trend of cigarette smoking among adolescents increasing when ENDS become more difficult to purchase.Copyright © 2016 Elsevier Inc. All rights reserved.
1576	Anonymous Drugs for tobacco dependence.. The Medical letter on drugs and therapeutics; 2016;58:27.<br><br>**Keywords:**<br>Clinical Trials as Topic/mt [Methods],Electronic Nicotine Delivery Systems/ae [Adverse Effects],Humans,*Nicotine/ad [Administration & Dosage],Nicotine/ae [Adverse Effects],Sleep Initiation and Maintenance Disorders/ci [Chemically Induced],Tobacco Use Cessation Products/ae [Adverse Effects],*Tobacco Use Cessation Products,Tobacco Use Disorder/di [Diagnosis],*Tobacco Use Disorder/dt [Drug Therapy]<br><br>**Abstract:**
1581	Murray Laugesen, Randolph C Grace Excise, electronic cigarettes and nicotine reduction to reduce smoking prevalence in New Zealand by 2025.. The New Zealand medical journal; 2016;129:94.<br><br>**Keywords:**<br>*Exercise,Humans,*Smoking/td [Trends],*Smoking Cessation/sn [Statistics & Numerical Data],*Smoking Prevention,*Tobacco Use Cessation Products/td [Trends],*Tobacco Use Disorder/th [Therapy]

	Abstract:
1588	Laura J Rennie, Cecile Bazillier-Bruneau, Jacques Rouesse Harm Reduction or Harm Introduction? Prevalence and Correlates of E-Cigarette Use Among French Adolescents.. The Journal of adolescent health : official publication of the Society for Adolescent Medicine; 2016;58:440.<br><br>**Keywords:**<br>Adolescent,Adolescent Behavior,*Electronic Nicotine Delivery Systems/sn [Statistics & Numerical Data], Electronic Nicotine Delivery Systems/ut [Utilization],France/ep [Epidemiology],*Harm Reduction,Health Surveys,Humans,Marketing/mt [Methods],Prevalence,*Smoking/ep [Epidemiology],Smoking Cessation/mt [Methods],*Smoking Cessation/sn [Statistics & Numerical Data],Surveys and Questionnaires,*Tobacco Products/sn [Statistics & Numerical Data]<br><br>**Abstract:** PURPOSE: Electronic cigarettes are marketed as a tool to give up or reduce cigarette smoking, and their use has risen sharply in recent years. There is concern that use is increasing particularly among adolescents and that they are not being used as a cessation tool but as a novel experience in their own right., METHODS: The present research assessed prevalence and sociodemographic correlates of e-cigarette use and standard cigarette use and also explored the extent to which e-cigarettes appear to be used as a cessation tool. This was assessed using a questionnaire administered to 1,486 French adolescents aged 16 years., RESULTS: Prevalence of e-cigarette experimentation was high (54%) and comparable to that for standard cigarettes (55%). Furthermore, 20% of those who had experimented with e-cigarettes had never tried standard cigarettes, and among regular smokers of standard cigarettes, intentions to quit were not associated with e-cigarette usage frequency. Experimentation with both e-cigarettes and standard cigarettes was significantly predicted by higher age, higher socioeconomic status, and parental smoking of standard cigarettes (in particular the father). Being male marginally predicted e-cigarette use, whereas being female significantly predicted standard cigarette use., CONCLUSIONS: These findings give cause for concern: e-cigarette usage experimentation is extremely high, and is not associated with attempts to quit smoking standard cigarettes. Rather, it is exposing adolescents to a highly addictive drug (nicotine) and may pave the way for a future cigarette habit.Copyright © 2016 Society for Adolescent Health and Medicine. Published by Elsevier Inc. All rights reserved.
1593	Victor M Cardenas, Victoria L Evans, Appathurai Balamurugan, Mohammed F Faramawi, Robert R Delongchamp, J Gary Wheeler Use of electronic nicotine delivery systems and recent initiation of smoking among US youth.. International journal of public health; 2016;61:237.<br><br>**Keywords:**<br>Adolescent,Cross-Sectional Studies,*Electronic Nicotine Delivery Systems/sn [Statistics & Numerical Data],Female,Humans,Male,Nicotine,Prevalence,*Smoking/ep [Epidemiology],Smoking Cessation,Surveys and Questionnaires,*Tobacco Products/sn [Statistics & Numerical Data],United States/ep [Epidemiology], Young Adult<br><br>**Abstract:** OBJECTIVES: We assessed whether the prevalence of recent (within a year) initiation of cigarette smoking was associated with reports of ever using electronic delivery systems (ENDS) in the National Youth Tobacco Survey (NYTS) and whether the association varied by age., METHODS: Weighted cross-sectional analysis of use of ENDS, cigarette smoking, age at interview and age at initiation of smoking collected systematically through the 2011-2013 NYTS cycles., RESULTS: In multivariate analyses those who ever used ENDS were twice as likely as nonusers of ENDS to have tried cigarette smoking in the last year (multivariate PR: 2.3; 95 % CI 1.9, 2.7). This average hid significant variations by age: a 4.1-fold increase (95 %; 2.6, 6.4) among those 11-13 years of age, compared to a smaller increase among those 16-18 years: 1.4-fold (95 % CI 1.1, 1.8)., CONCLUSIONS: Use of ENDS by adolescents was associated with initiation of cigarette smoking in the last year. This association was stronger in younger adolescents.

1596	Rehab Auf, Mary Jo Trepka, Miguel Angel Cano, Mario De La Rosa, Marah Selim, Elena Bastida Electronic cigarettes: the renormalisation of nicotine use.. BMJ (Clinical research ed.); 2016;352:i425.<br><br>**Keywords:**<br>*Electronic Nicotine Delivery Systems,Humans,*Smoking Cessation<br><br>**Abstract:**
1611	Gerhard Gmel, Stephanie Baggio, Meichun Mohler-Kuo, Jean-Bernard Daeppen, Joseph Studer (2016//) E-cigarette use in young Swiss men: is vaping an effective way of reducing or quitting smoking?.. Swiss medical weekly; 146<br><br>**Keywords:**<br>*Electronic Nicotine Delivery Systems/sn [Statistics & Numerical Data],Follow-Up Studies,Humans, Longitudinal Studies,Male,Odds Ratio,*Smoking/ep [Epidemiology],*Smoking Cessation/sn [Statistics & Numerical Data],Switzerland/ep [Epidemiology],*Tobacco Use Disorder/ep [Epidemiology],Young Adult<br><br>**Abstract:** QUESTION UNDER STUDY: To test longitudinally differences in conventional cigarette use (cigarettes smoked, cessation, quit attempts) between vapers and nonvapers., METHODS: Fifteen months follow-up of a sample of 5128 20-year-old Swiss men. The onset of conventional cigarette (CC) use among nonsmokers, and smoking cessation, quit attempts, changes in the number of CCs smoked among smokers at baseline were compared between vapers and nonvapers at follow-up, adjusted for nicotine dependence., RESULTS: Among baseline nonsmokers, vapers were more likely to start smoking at follow-up than nonvapers (odds ratio [OR] 6.02, 95% confidence interval [CI] 2.81, 12.88 for becoming occasional smokers, and OR = 12.69, 95% CI 4.00, 40.28 for becoming daily smokers). Vapers reported lower smoking cessation rates among occasional smokers at baseline (OR = 0.43 (0.19, 0.96); daily smokers: OR = 0.42 [0.15, 1.18]). Vapers compared with nonvapers were heavier CC users (62.53 vs 18.10 cigarettes per week, p <0.001) and had higher nicotine dependence levels (2.16 vs 0.75, p <0.001) at baseline. The number of CCs smoked increased between baseline and follow-up among occasional smokers (b = 6.06, 95% CI 4.44, 7.68) and decreased among daily smokers (b = -5.03, 95% CI -8.69, -1.38), but there were no differential changes between vapers and nonvapers. Vapers showed more quit attempts at follow-up compared with nonvapers for baseline occasional smokers (incidence rate ratio [IRR] 1.81, 95% CI 1.24, 2.64; daily smokers IRR 1.28, 95% CI 0.95, 1.73)., CONCLUSIONS: We found no beneficial effects of vaping at follow-up for either smoking cessation or smoking reduction.
1635	Jimi Huh, Adam M Leventhal Intraindividual covariation between e-cigarette and combustible cigarette use in Korean American emerging adults.. Psychology of addictive behaviors : journal of the Society of Psychologists in Addictive Behaviors; 2016;30:246.<br><br>**Keywords:**<br>Adolescent,Adult,*Asian Americans,Craving,*Electronic Nicotine Delivery Systems/ut [Utilization],Female, Humans,Linear Models,Male,Republic of Korea/eh [Ethnology],*Smoking,Smoking Cessation,Surveys and Questionnaires,*Tobacco Products/ut [Utilization],*Tobacco Use Disorder,Young Adult<br><br>**Abstract:** Critical gaps exist in understanding the patterns and correlates of dual use of electronic cigarettes (ECs) and combustible cigarettes (CCs), particularly in ethnic minority populations. In this study, we assessed CC and EC use in the naturalistic environment using ecological momentary assessment (EMA). We hypothesized that within-subject variation in EC use (yes/no each day) would be inversely associated with within-subject variation in number of CCs consumed and craving during that same day. We also examined gender and nicotine dependence as moderators of the EC-CC and EC-craving covariations. Korean American emerging adult (KAEA; 18-25 years old) smokers (N = 78) completed 7 days of EMA. Participants completed EMA surveys throughout the day, which assessed CC craving, and end-of-day surveys, which assessed EC use and the number of CCs smoked that day.

	Generalized linear mixed models were used to predict day-level EC use, with number of CCs smoked and craving during that same day, gender, and nicotine dependence as predictors (n = 501). We found that within-subject variation in CC use was not associated with same-day EC use; neither was within-subject variation in craving (ps > .27). Gender moderated the relationship between craving and EC use on a given day (p = .03); only for females, on the days with higher craving, the likelihood of their EC use that day was significantly heightened. This study does not suggest that EC use is linked with lower CC smoking quantity, at least at the day level and among KAEA smokers. CC craving may play a role in dual EC-CC use for KAEA female smokers.Copyright (c) 2016 APA, all rights reserved).
1644	Man Ping Wang, Sai Yin Ho, Lok Tung Leung, Tai Hing Lam Electronic Cigarette Use and Respiratory Symptoms in Chinese Adolescents in Hong Kong.. JAMA pediatrics; 2016;170:89.<br><br>**Keywords:**<br>Adolescent,*Asian Continental Ancestry Group/sn [Statistics & Numerical Data],Child,*Electronic Nicotine Delivery Systems/ae [Adverse Effects],*Electronic Nicotine Delivery Systems/sn [Statistics & Numerical Data],Female,Hong Kong/ep [Epidemiology],Humans,Male,Prevalence,*Respiration Disorders/ep [Epidemiology],*Respiration Disorders/et [Etiology],Surveys and Questionnaires<br><br>**Abstract:**
1647	Jennifer Cantrell, Brittany Emelle, Ollie Ganz, Elizabeth C Hair, Donna Vallone Rapid increase in e-cigarette advertising spending as Altria's MarkTen enters the marketplace.. Tobacco control; 2016;25:e16.<br><br>**Keywords:**<br>Age Factors,Awareness,*Commerce/ec [Economics],Commerce/td [Trends],*Direct-to-Consumer Advertising/ec [Economics],Direct-to-Consumer Advertising/td [Trends],Electronic Nicotine Delivery Systems/ae [Adverse Effects],*Electronic Nicotine Delivery Systems/ec [Economics],Electronic Nicotine Delivery Systems/td [Trends],Health Behavior,Humans,Risk Assessment,Smoking/ae [Adverse Effects], Smoking/ec [Economics],*Smoking Cessation/ec [Economics],*Smoking Cessation/mt [Methods], *Smoking Prevention,Time Factors,Vaping/ae [Adverse Effects],*Vaping/ec [Economics],Vaping/td [Trends]<br><br>**Abstract:**
1650	Maciej L Goniewicz, Noel J Leigh, Michal Gawron, Justyna Nadolska, Lukasz Balwicki, Connor McGuire, Andrzej Sobczak Dual use of electronic and tobacco cigarettes among adolescents: a cross-sectional study in Poland.. International journal of public health; 2016;61:189.<br><br>**Keywords:**<br>Adolescent,Adolescent Behavior,Cross-Sectional Studies,*Electronic Nicotine Delivery Systems/sn [Statistics & Numerical Data],Electronic Nicotine Delivery Systems/ut [Utilization],Female,Humans,Male, Poland/ep [Epidemiology],*Smoking/ep [Epidemiology],Surveys and Questionnaires,*Tobacco Products/sn [Statistics & Numerical Data]<br><br>**Abstract:** OBJECTIVES: Electronic cigarettes (e-cigarettes) are gaining in popularity among youth. While these products may be beneficial in adult smokers, the effect on young users of electronic and tobacco cigarettes (dual users) is unknown. The objective of this study was to assess the frequency of dual use among adolescents and to compare tobacco cigarette consumption among dual and exclusive tobacco cigarette users., METHODS: A cross-sectional survey of a sample of 2213 Polish students aged 16-18 conducted between December 2013 and February 2014., RESULTS: Overall, 21.8 % of students were dual users. Dual users were more likely to smoke tobacco cigarettes on a daily basis [adjusted odds ratio, AOR 3.54 (95 % CI 2.34-5.36) and less likely to smoke fewer cigarettes per day (AOR 0.27 (95 % CI 0.12-0.57)]

	than exclusive tobacco cigarette users., CONCLUSIONS: The frequency of dual use was higher than exclusive use of a single product among Polish adolescents. Young dual users do not smoke a lower number of tobacco cigarettes per day than exclusive tobacco cigarette users.
1661	Sven Schneider, Katharina Diehl Vaping as a Catalyst for Smoking? An Initial Model on the Initiation of Electronic Cigarette Use and the Transition to Tobacco Smoking Among Adolescents.. Nicotine & tobacco research : official journal of the Society for Research on Nicotine and Tobacco; 2016;18:647.<br><br>**Keywords:**<br>Adolescent,*Adolescent Behavior/px [Psychology],Commerce,Electronic Nicotine Delivery Systems/ec [Economics],Electronic Nicotine Delivery Systems/px [Psychology],*Electronic Nicotine Delivery Systems/sn [Statistics & Numerical Data],Humans,Peer Group,Smoking/ec [Economics],*Smoking/ep [Epidemiology],Smoking/px [Psychology],Smoking Prevention<br><br>**Abstract:** INTRODUCTION: The popularity of electronic cigarettes (e-cigarettes) among adolescents is growing worldwide. A more accurate model than the much discussed but inadequate Gateway Hypothesis is needed to explain some adolescents' initial preference for e-cigarettes over tobacco cigarettes, as well as any transition from e-cigarettes to tobacco smoking. Our aim was to summarize the diffuse fear that adolescents will be indirectly encouraged to begin smoking tobacco via the use of e-cigarettes and to systematize the disparate causal hypotheses used thus far in relevant literature., METHODS: We summarized the vague and fragmented hypotheses formulated thus far in literature on both trajectories from abstinence to e-cigarette use and from there to tobacco smoking into a set of empirically testable hypotheses and organized them into a comprehensive model., RESULTS: Our results indicate that the perceived health risks, specific product characteristics (such as taste, price and inconspicuous use), and higher levels of acceptance among peers and others potentially make e-cigarettes initially more attractive to adolescents than tobacco cigarettes. Later, increasing familiarity with nicotine could lead to the reevaluation of both electronic and tobacco cigarettes and subsequently to a potential transition to tobacco smoking. The suggested "catalyst model" takes variations in the nicotine content of e-cigarettes as well as the dual use of different substances into account., CONCLUSION: Our model provides causal hypotheses for the initiation of e-cigarette use and for the potential transition to tobacco smoking which, after being tested in empirical studies, could lead to the formulation of concrete recommendations for healthcare intervention and prevention measures., IMPLICATIONS: We developed a model that provides causal hypotheses for the initiation of e-cigarette use and for the potential transition to tobacco smoking.Copyright © The Author 2015. Published by Oxford University Press on behalf of the Society for Research on Nicotine and Tobacco. All rights reserved. For permissions, please e-mail: journals.permissions@oup.com.
1669	Thomas A Wills, James D Sargent, Rebecca Knight, Ian Pagano, Frederick X Gibbons E-cigarette use and willingness to smoke: a sample of adolescent non-smokers.. Tobacco control; 2016;25:e52.<br><br>**Keywords:**<br>Adolescent,*Adolescent Behavior,Age Factors,Chi-Square Distribution,Cognition,Cross-Sectional Studies, Electronic Nicotine Delivery Systems/ae [Adverse Effects],*Electronic Nicotine Delivery Systems/px [Psychology],Female,Health Behavior,Health Knowledge, Attitudes, Practice,Humans,Linear Models, Logistic Models,Male,Odds Ratio,Peer Influence,Risk Factors,Smoking/ae [Adverse Effects],*Smoking/px [Psychology],Social Behavior,Surveys and Questionnaires,Vaping/ae [Adverse Effects],*Vaping<br><br>**Abstract:** OBJECTIVE: There is little evidence on the consequences of using electronic cigarettes (e-cigarette) in adolescence. With a multiethnic sample of non-smokers, we assessed the relation between e-cigarette use and social-cognitive factors that predict smoking of combustible cigarettes., METHODS: School-based cross-sectional survey of 2309 high school students (mean age 14.7 years). Participants reported on e-cigarette use and cigarette use; on smoking-related cognitions (smoking expectancies, prototypes of smokers) and peer smoker affiliations; and on willingness to smoke cigarettes. Regression

	analyses conducted for non-cigarette smokers tested the association between e-cigarette use and willingness to smoke cigarettes, controlling for demographics, parenting, academic and social competence, and personality variables. Structural equation modelling (SEM) analysis tested whether the relation between e-cigarette use and willingness to smoke was mediated through any of the three smoking-related variables., RESULTS: Non-smokers who had used e-cigarettes (18% of the total sample) showed more willingness to smoke cigarettes compared with those who had never used any tobacco product; the adjusted OR was 2.35 (95% CI 1.73 to 3.19). SEM showed that the relation between e-cigarette use and willingness to smoke was partly mediated through more positive expectancies about smoking, but there was also a direct path from e-cigarette use to willingness., CONCLUSIONS: Among adolescent non-smokers, e-cigarette use is associated with willingness to smoke, a predictor of future cigarette smoking. The results suggest that use of e-cigarettes by adolescents is not without attitudinal risk for cigarette smoking. These findings have implications for formulation of policy about access to e-cigarettes by adolescents.Copyright Published by the BMJ Publishing Group Limited. For permission to use (where not already granted under a licence) please go to http://www.bmj.com/company/products-services/rights-and-licensing/
1687	Jonine Janceya, Colin Binns, James A Smith, Bruce Maycock, Peter Howat The rise of e-cigarettes: implications for health promotion.. Health promotion journal of Australia : official journal of Australian Association of Health Promotion Professionals; 2015;26:79.<br><br>**Keywords:**<br>*Electronic Nicotine Delivery Systems,Government Regulation,Health Promotion/mt [Methods],Humans, Marketing,Smoking Cessation/mt [Methods],World Health Organization<br><br>**Abstract:**
1693	Charlotte A Schoenborn, Renee M Gindi Electronic Cigarette Use Among Adults: United States, 2014.. NCHS data brief; 2015;:1.<br><br>**Keywords:**<br>Adolescent,Adult,Age Distribution,Aged,Continental Population Groups,*Electronic Nicotine Delivery Systems/sn [Statistics & Numerical Data],Female,Humans,Male,Middle Aged,Prevalence,Sex Distribution, Smoking/ep [Epidemiology],Smoking Cessation/sn [Statistics & Numerical Data],United States,Young Adult<br><br>**Abstract:** The National Health Interview Survey (NHIS) first began collecting data about e-cigarette use in 2014. The estimates presented in this report provide a foundation for understanding who is using e-cigarettes and for monitoring changes in e-cigarette use among U.S. adults over time. In 2014, men were more likely than women to have ever tried e-cigarettes but were not more likely to be current users. Younger adults were more likely than older adults to have tried e-cigarettes and to currently use e-cigarettes. Both non-Hispanic AIAN and non-Hispanic white adults were more likely than non-Hispanic black, non-Hispanic Asian, and Hispanic adults to have ever tried e-cigarettes and to be current e-cigarette users. When examined in the context of conventional cigarette smoking, use of e-cigarettes was highest among current and recent former cigarette smokers, and among current smokers who had made a quit attempt in the past year. Although fewer than 4% of adults who had never smoked conventional cigarettes had ever tried an e-cigarette, nearly 1 in 10 never-smokers aged 18-24 had tried an e-cigarette at least once.Copyright All material appearing in this report is in the public domain and may be reproduced or copied without permission; citation as to source, however, is appreciated.
1699	Susan C Walley, Brian P Jenssen,Section on Tobacco Control Electronic Nicotine Delivery Systems.. Pediatrics; 2015;136:1018.<br><br>**Keywords:**

	Adolescent,Child,*Electronic Nicotine Delivery Systems,Humans,*Nicotine/ad [Administration & Dosage], Nicotine/ae [Adverse Effects],*Smoking Prevention<br><br>**Abstract:** Electronic nicotine delivery systems (ENDS) are rapidly growing in popularity among youth. ENDS are handheld devices that produce an aerosolized mixture from a solution typically containing concentrated nicotine, flavoring chemicals, and propylene glycol to be inhaled by the user. ENDS are marketed under a variety of names, most commonly electronic cigarettes and e-cigarettes. In 2014, more youth reported using ENDS than any other tobacco product. ENDS pose health risks to both users and nonusers. Nicotine, the major psychoactive ingredient in ENDS solutions, is both highly addictive and toxic. In addition to nicotine, other toxicants, carcinogens, and metal particles have been detected in solutions and aerosols of ENDS. Nonusers are involuntarily exposed to the emissions of these devices with secondhand and thirdhand aerosol. The concentrated and often flavored nicotine in ENDS solutions poses a poisoning risk for young children. Reports of acute nicotine toxicity from US poison control centers have been increasing, with at least 1 child death reported from unintentional exposure to a nicotine-containing ENDS solution. With flavors, design, and marketing that appeal to youth, ENDS threaten to renormalize and glamorize nicotine and tobacco product use. There is a critical need for ENDS regulation, legislative action, and counter promotion to protect youth. ENDS have the potential to addict a new generation of youth to nicotine and reverse more than 50 years of progress in tobacco control.Copyright © 2015 by the American Academy of Pediatrics.
1703	Timothy C Harrold, Audrey K Maag, Sarah Thackway, Jo Mitchell, Lee K Taylor Prevalence of e-cigarette users in New South Wales.. The Medical journal of Australia; 2015;203:326.<br><br>**Keywords:**<br>Adolescent,Adult,*Electronic Nicotine Delivery Systems/sn [Statistics & Numerical Data],Female,Humans, Male,Middle Aged,New South Wales,Prevalence<br><br>**Abstract:**
1712	Linda J Neff, Rene A Arrazola, Ralph S Caraballo, Catherine G Corey, Shanna Cox, Brian A King, Conrad J Choiniere, Corinne G Husten Frequency of Tobacco Use Among Middle and High School Students--United States, 2014.. MMWR. Morbidity and mortality weekly report; 2015;64:1061.<br><br>**Keywords:**<br>Adolescent,Child,Cross-Sectional Studies,*Electronic Nicotine Delivery Systems/ut [Utilization],Health Surveys,Humans,Schools/sn [Statistics & Numerical Data],*Students/px [Psychology],Students/sn [Statistics & Numerical Data],*Tobacco Products/ut [Utilization],*Tobacco Use/ep [Epidemiology],United States/ep [Epidemiology]<br><br>**Abstract:** The use of tobacco products during adolescence increases the risk for adverse health effects and lifelong nicotine addiction. In 2014, an estimated 4.6 million middle and high school students were current users of any tobacco product, of whom an estimated 2.2 million were current users of two or more types of tobacco products. Symptoms of nicotine dependence are increased for multiple tobacco product users compared with single-product users. CDC and the Food and Drug Administration (FDA) analyzed data from the 2014 National Youth Tobacco Survey (NYTS) to determine how frequently (the number of days in the preceding 30 days) U.S. middle school (grades 6-8) and high school (grades 9-12) students used cigarettes, e-cigarettes, cigars, and smokeless tobacco products. Among current users (>=1 day during the preceding 30 days) in high school, frequent use (>=20 days during the preceding 30 days) was most prevalent among smokeless tobacco users (42.0%), followed by cigarette smokers (31.6%), e-cigarette users (15.5%), and cigar smokers (13.1%); a similar pattern was observed for those who used during all 30 days. Among current users in middle school, frequent use was greatest among smokeless tobacco users (29.2%), followed by cigarette smokers (20.0%), cigar smokers (13.2%) and e-cigarette users (11.8%). Current use of two or more types of tobacco products was common, even among students

	who used tobacco products 1-5 days during the preceding 30 days: 77.3% for cigar smokers, 76.9% for cigarette smokers, 63.4% for smokeless tobacco users, and 54.8% for e-cigarettes users. Preventing youths from initiating the use of any tobacco product is important to tobacco use prevention and control strategies in the United States. Monitoring the frequency and patterns of tobacco use among youths, including the use of two or more tobacco products, is important to inform evidence-based interventions to prevent and reduce all forms of tobacco use among youths.
1722	Judy Li, Rhiannon Newcombe, Rhiannon Newcombe, Darren Walton Susceptibility to e-cigarette use among never-users: findings from a survey of New Zealand adult smokers and ex-smokers.. The New Zealand medical journal; 2015;128:65.<br><br>**Keywords:**<br>Adolescent,Adult,*Electronic Nicotine Delivery Systems/px [Psychology],*Electronic Nicotine Delivery Systems/ut [Utilization],Female,Humans,Male,Middle Aged,New Zealand,*Smoking/px [Psychology], *Smoking Cessation/px [Psychology],Surveys and Questionnaires,Young Adult<br><br>**Abstract:**
1726	Susan Mayor Young adults using e-cigarettes are more likely to progress to smoking, study shows.. BMJ (Clinical research ed.); 2015;351:h4802.<br><br>**Keywords:**<br>*Behavior, Addictive/px [Psychology],Electronic Nicotine Delivery Systems/ae [Adverse Effects], *Electronic Nicotine Delivery Systems/px [Psychology],Electronic Nicotine Delivery Systems/ut [Utilization],Health Knowledge, Attitudes, Practice,Humans,*Smoking/px [Psychology],Tobacco Products, United States/ep [Epidemiology],Young Adult<br><br>**Abstract:**
1728	Brian A Primack, Samir Soneji, Michael Stoolmiller, Michael J Fine, James D Sargent Progression to Traditional Cigarette Smoking After Electronic Cigarette Use Among US Adolescents and Young Adults.. JAMA pediatrics; 2015;169:1018.<br><br>**Keywords:**<br>Adolescent,*Adolescent Behavior,Adult,Cohort Studies,Disease Progression,*Electronic Nicotine Delivery Systems/ae [Adverse Effects],Female,Health Surveys,Humans,Logistic Models,Longitudinal Studies,Male, Risk Assessment,Risk Factors,*Smoking/ep [Epidemiology],Smoking Cessation/sn [Statistics & Numerical Data],United States,Young Adult<br><br>**Abstract:** IMPORTANCE: Electronic cigarettes (e-cigarettes) may help smokers reduce the use of traditional combustible cigarettes. However, adolescents and young adults who have never smoked traditional cigarettes are now using e-cigarettes, and these individuals may be at risk for subsequent progression to traditional cigarette smoking., OBJECTIVE: To determine whether baseline use of e-cigarettes among nonsmoking and nonsusceptible adolescents and young adults is associated with subsequent progression along an established trajectory to traditional cigarette smoking., DESIGN, SETTING, AND PARTICIPANTS: In this longitudinal cohort study, a national US sample of 694 participants aged 16 to 26 years who were never cigarette smokers and were attitudinally nonsusceptible to smoking cigarettes completed baseline surveys from October 1, 2012, to May 1, 2014, regarding smoking in 2012-2013. They were reassessed 1 year later. Analysis was conducted from July 1, 2014, to March 1, 2015. Multinomial logistic regression was used to assess the independent association between baseline e-cigarette use and cigarette smoking, controlling for sex, age, race/ethnicity, maternal educational level, sensation-seeking tendency, parental cigarette smoking, and cigarette smoking among friends. Sensitivity analyses were performed, with varying approaches to missing data and recanting., EXPOSURES: Use of e-

	cigarettes at baseline., MAIN OUTCOMES AND MEASURES: Progression to cigarette smoking, defined using 3 specific states along a trajectory: nonsusceptible nonsmokers, susceptible nonsmokers, and smokers. Individuals who could not rule out smoking in the future were defined as susceptible., RESULTS: Among the 694 respondents, 374 (53.9%) were female and 531 (76.5%) were non-Hispanic white. At baseline, 16 participants (2.3%) used e-cigarettes. Over the 1-year follow-up, 11 of 16 e-cigarette users and 128 of 678 of those who had not used e-cigarettes (18.9%) progressed toward cigarette smoking. In the primary fully adjusted models, baseline e-cigarette use was independently associated with progression to smoking (adjusted odds ratio [AOR], 8.3; 95% CI, 1.2-58.6) and to susceptibility among nonsmokers (AOR, 8.5; 95% CI, 1.3-57.2). Sensitivity analyses showed consistent results in the level of significance and slightly larger magnitude of AORs., CONCLUSIONS AND RELEVANCE: In this national sample of US adolescents and young adults, use of e-cigarettes at baseline was associated with progression to traditional cigarette smoking. These findings support regulations to limit sales and decrease the appeal of e-cigarettes to adolescents and young adults.
1729	Pallav Pokhrel, Thaddeus A Herzog, Nicholas Muranaka, Sakshi Regmi, Pebbles Fagan Contexts of cigarette and e-cigarette use among dual users: a qualitative study.. BMC public health; 2015;15:859.<br><br>**Keywords:**<br>Adult,*Electronic Nicotine Delivery Systems/ut [Utilization],Female,Humans,Male,Qualitative Research,*Smoking Cessation/mt [Methods],Smoking Cessation/px [Psychology],*Tobacco Products/sn [Statistics & Numerical Data],*Tobacco Use Cessation Products,Tobacco Use Disorder,Young Adult<br><br>**Abstract:** BACKGROUND: Not much is currently understood regarding the contexts of cigarette and e-cigarette use among dual users. Proper application of e-cigarettes to smoking cessation or tobacco harm reduction would require an understanding of when and why dual users use cigarettes versus e-cigarettes. This study sought to elucidate the contexts of cigarette versus e-cigarette use among dual users., METHODS: Twelve focus group discussions were conducted with 62 young adult current daily e-cigarette users [63% men; mean age = 25.1 (Standard Deviation = 5.5)]. Almost all participants either concurrently smoked cigarettes or had been recent dual users. Data were analyzed following principles of inductive deduction., RESULTS: Results indicated that dual users' use of cigarettes is influenced by particular activities (e.g., before/after eating), strong craving or need for stimulation (e.g., in response to stress), places/situations (e.g., when cigarette smokers are nearby; outdoors), use of other substances (alcohol, coffee), and unavailability of an e-cigarette when needed. In addition to particular activities and places/situations that are conducive to e-cigarette use, use of e-cigarette when cigarette is not available or where cigarette smoking is not permitted emerged as contexts specific to e-cigarette use., CONCLUSIONS: For habitual cigarette smokers wanting to quit tobacco smoking, switching over completely to e-cigarettes may require skills of cognitive-behavioral management. Future research needs to ascertain the characteristics of dual users who use e-cigarettes as cessation aids versus as cigarette alternative when cigarette is unavailable or smoking is not permitted.
1734	Harold J Farber The Harm of Tobacco Starts Before Birth.. Chest; 2015;148:573.<br><br>**Keywords:**<br>*Asthma/et [Etiology],Child,Child, Preschool,Electronic Nicotine Delivery Systems/ae [Adverse Effects],Female,Humans,Infant,Infant, Newborn,*Nicotine/to [Toxicity],Pregnancy,*Prenatal Exposure Delayed Effects,Risk,*Tobacco Smoke Pollution/ae [Adverse Effects],*Tobacco Use Disorder/co [Complications]<br><br>**Abstract:**
1735	Sara Kalkhoran, Stanton A Glantz Modeling the Health Effects of Expanding e-Cigarette Sales in the United States and United Kingdom: A Monte Carlo Analysis.. JAMA internal medicine; 2015;175:1671.<br><br>**Keywords:**

	Adolescent,Adult,Electronic Nicotine Delivery Systems/ae [Adverse Effects],Electronic Nicotine Delivery Systems/ec [Economics],Electronic Nicotine Delivery Systems/sn [Statistics & Numerical Data],*Electronic Nicotine Delivery Systems,Female,Humans,Male,Marketing/mt [Methods],Monte Carlo Method,Outcome Assessment (Health Care),Population Surveillance,Prevalence,Risk Assessment,Smoking/ae [Adverse Effects],Smoking/ec [Economics],Smoking/ep [Epidemiology],*Smoking,Smoking Cessation/mt [Methods],Smoking Cessation/sn [Statistics & Numerical Data],Smoking Prevention,United Kingdom/ep [Epidemiology],United States/ep [Epidemiology]<br><br>**Abstract:** IMPORTANCE: The prevalence of electronic cigarette (e-cigarette) use is increasing. Population health effects will depend on cigarette smoking behaviors, levels of dual use with conventional cigarettes, and e-cigarette toxicity., OBJECTIVE: To evaluate potential health effects of various scenarios of increasing promotion and use of e-cigarettes., DESIGN, SETTING, AND PARTICIPANTS: A base case model was developed using data on actual cigarette and e-cigarette use patterns that quantifies transitions from an initial state of no cigarette or e-cigarette use to 1 of 5 final states: never use of cigarettes or e-cigarettes, cigarette use, e-cigarette use, dual use of cigarettes and e-cigarettes, or quit. Seven scenarios were created that cover a range of use patterns, depending on how the e-cigarette market might develop, as well as a range of possible long-term health effects of e-cigarette use. Scenarios for changes from the base case were evaluated using Monte Carlo simulations. Separate sets of base case model parameters were evaluated for the US and UK populations., MAIN OUTCOMES AND MEASURES: We assigned unitless health "costs" for each final state on a scale of 0 to 100. Population health "costs" were compared with the base case (status quo) assuming e-cigarette use health "costs" from 1% to 50% as dangerous as conventional cigarette use health costs., RESULTS: Compared with the base case, a harm reduction scenario in which e-cigarette use increases only among smokers who are interested in quitting with more quit attempts and no increased initiation of e-cigarette use among nonsmokers, and another scenario in which e-cigarettes are taken up only by youth who would have smoked conventional cigarettes, had population-level health benefits regardless of e-cigarette health costs in both the United States and United Kingdom. Conversely, scenarios in which e-cigarette promotion leads to renormalization of cigarette smoking or e-cigarettes are used primarily by youth who never would have smoked showed net health harms across all e-cigarette health costs. In other scenarios, the net health effect varied on the basis of the health cost of e-cigarettes., CONCLUSIONS AND RELEVANCE: According to this analysis, widespread promotion of e-cigarettes may have a wide range of population-level health effects, depending on both e-cigarette health risks and patterns of use. Absent the primary effect of e-cigarette promotion being only to divert current or future conventional cigarette smokers to e-cigarette use, the current uncertainty about the health risks of e-cigarettes, increasing e-cigarette use among youth, and the varying health effects at different e-cigarette health costs suggest a potential for harm.
1743	Michael McCarthy Teens who use e-cigarettes are more likely to take up smoking, US study finds.. BMJ (Clinical research ed.); 2015;351:h4471.<br><br>**Keywords:**<br>*Adolescent Behavior,*Electronic Nicotine Delivery Systems,Female,Humans,Male,*Smoking/ep [Epidemiology],*Tobacco Products/ut [Utilization]<br><br>**Abstract:**
1744	Adam M Leventhal, David R Strong, Matthew G Kirkpatrick, Jennifer B Unger, Steve Sussman, Nathaniel R Riggs, Matthew D Stone, Rubin Khoddam, Jonathan M Samet, Janet Audrain-McGovern Association of Electronic Cigarette Use With Initiation of Combustible Tobacco Product Smoking in Early Adolescence.. JAMA; 2015;314:700.<br><br>**Keywords:**<br>Adolescent,*Adolescent Behavior,Data Collection,*Electronic Nicotine Delivery Systems,Female,Follow-Up Studies,Ganglionic Stimulants/ad [Administration & Dosage],Humans,Los Angeles/ep [Epidemiology],

	Male,Nicotine/ad [Administration & Dosage],Odds Ratio,Risk Factors,Self Report,*Smoking/ep [Epidemiology],Students,*Tobacco Products/ut [Utilization]<br><br>**Abstract:** IMPORTANCE: Exposure to nicotine in electronic cigarettes (e-cigarettes) is becoming increasingly common among adolescents who report never having smoked combustible tobacco., OBJECTIVE: To evaluate whether e-cigarette use among 14-year-old adolescents who have never tried combustible tobacco is associated with risk of initiating use of 3 combustible tobacco products (ie, cigarettes, cigars, and hookah)., DESIGN, SETTING, AND PARTICIPANTS: Longitudinal repeated assessment of a school-based cohort at baseline (fall 2013, 9th grade, mean age=14.1 years) and at a 6-month follow-up (spring 2014, 9th grade) and a 12-month follow-up (fall 2014, 10th grade). Ten public high schools in Los Angeles, California, were recruited through convenience sampling. Participants were students who reported never using combustible tobacco at baseline and completed follow-up assessments at 6 or 12 months (N=2530). At each time point, students completed self-report surveys during in-classroom data collections., EXPOSURE: Student self-report of whether he or she ever used e-cigarettes (yes or no) at baseline., MAIN OUTCOMES AND MEASURES: Six- and 12-month follow-up reports on use of any of the following tobacco products within the prior 6 months: (1) any combustible tobacco product (yes or no); (2) combustible cigarettes (yes or no), (3) cigars (yes or no); (4) hookah (yes or no); and (5) number of combustible tobacco products (range: 0-3)., RESULTS: Past 6-month use of any combustible tobacco product was more frequent in baseline e-cigarette ever users (n=222) than never users (n=2308) at the 6-month follow-up (30.7% vs 8.1%, respectively; difference between groups in prevalence rates, 22.7% [95% CI, 16.4%-28.9%]) and at the 12-month follow-up (25.2% vs 9.3%, respectively; difference between groups, 15.9% [95% CI, 10.0%-21.8%]). Baseline e-cigarette use was associated with greater likelihood of use of any combustible tobacco product averaged across the 2 follow-up periods in the unadjusted analyses (odds ratio [OR], 4.27 [95% CI, 3.19-5.71]) and in the analyses adjusted for sociodemographic, environmental, and intrapersonal risk factors for smoking (OR, 2.73 [95% CI, 2.00-3.73]). Product-specific analyses showed that baseline e-cigarette use was positively associated with combustible cigarette (OR, 2.65 [95% CI, 1.73-4.05]), cigar (OR, 4.85 [95% CI, 3.38-6.96]), and hookah (OR, 3.25 [95% CI, 2.29-4.62]) use and with the number of different combustible products used (OR, 4.26 [95% CI, 3.16-5.74]) averaged across the 2 follow-up periods., CONCLUSIONS AND RELEVANCE: Among high school students in Los Angeles, those who had ever used e-cigarettes at baseline compared with nonusers were more likely to report initiation of combustible tobacco use over the next year. Further research is needed to understand whether this association may be causal.
1745	F Alawsi, R Nour, S Prabhu Are e-cigarettes a gateway to smoking or a pathway to quitting?.. British dental journal; 2015;219:111.<br><br>**Keywords:**<br>*Electronic Nicotine Delivery Systems,Humans,*Smoking,*Smoking Cessation/mt [Methods]<br><br>**Abstract:** Over recent years there has been a massive increase in the usage of electronic cigarettes (e-cigs) by the general public. There are mixed views regarding the safety and efficacy of e-cigs, even among healthcare professionals. While some individuals view e-cigs as a public health concern, others recommend them as a safe alternative to conventional cigarettes for smokers who are willing to quit. Since e-cigs are a new phenomena, many clinicians are unaware of their impact on users (known as vapers), who may seek medical advice regarding their use. This clinical review aims to educate healthcare professionals regarding the advantages and disadvantages of e-cigarettes and to discuss whether e-cigarettes help users quit smoking or whether they renormalise smoking. This article will describe the contents of e-cigs and how they are used, the history, advantages, disadvantages and then balance the positive and negative aspects of their use. Due to the lack of long-term follow up of the health effects of e-cigarettes, caution is advised with their use.
1746	Jason M Jerry, Gregory B Collins, David Streem E-cigarettes: Safe to recommend to patients?.. Cleveland Clinic journal of medicine; 2015;82:521.

	**Keywords:**<br>*Electronic Nicotine Delivery Systems,Humans,Safety<br><br>**Abstract:** Electronic cigarettes (e-cigarettes)--devices that generate a nicotine vapor that can be inhaled by the user in a fashion that mimics the experience of smoking--are increasing in popularity, and many people seem to view them as reasonable alternatives to nicotine replacement therapy to help them refrain from smoking. Physicians should not encourage such a view. E-cigarettes are unregulated nicotine delivery systems that have never been subjected to any kind of testing of safety or of efficacy as nicotine replacement therapy. Moreover, for young people who have never smoked, these devices could potentially serve as a gateway drug. Copyright © 2015 Cleveland Clinic.
1755	Manfred Neuberger The electronic cigarette: a wolf in sheep's clothing.. Wiener klinische Wochenschrift; 2015;127:385.<br><br>**Keywords:**<br>Adolescent,Adult,Austria,Child,Deception,*Electronic Nicotine Delivery Systems/ae [Adverse Effects],*Electronic Nicotine Delivery Systems/ut [Utilization],Flavoring Agents/ae [Adverse Effects],Humans,Intention,Nicotine/ad [Administration & Dosage],Nicotine/ae [Adverse Effects],Particulate Matter/ae [Adverse Effects],Smoking/px [Psychology],Smoking Cessation,Social Marketing,Solvents/ae [Adverse Effects],Tobacco Industry,Young Adult<br><br>**Abstract:** The American Heart Association warned from the potential of electronic cigarettes to renormalize smoking in public and the International Respiratory Societies demanded regulation of all nicotine products as medicines or tobacco products. This review summarizes the results of studies on hazards of e-cigarette use, which has increased dramatically and may be the real threat for the achievements in tobacco control of the past 20 years.
1757	Jessica L Barrington-Trimis, Kiros Berhane, Jennifer B Unger, Tess Boley Cruz, Jimi Huh, Adam M Leventhal, Robert Urman, Kejia Wang, Steve Howland, Tamika D Gilreath, Chih-Ping Chou, Mary Ann Pentz, Rob McConnell Psychosocial Factors Associated With Adolescent Electronic Cigarette and Cigarette Use.. Pediatrics; 2015;136:308.<br><br>**Keywords:**<br>Adolescent,Cross-Sectional Studies,*Electronic Nicotine Delivery Systems/px [Psychology],*Electronic Nicotine Delivery Systems/sn [Statistics & Numerical Data],Female,Humans,Male,Risk Assessment,*Smoking/ep [Epidemiology],*Smoking/px [Psychology],Surveys and Questionnaires<br><br>**Abstract:** BACKGROUND: Use of electronic cigarettes (e-cigarettes) among adolescents has increased since their introduction into the US market in 2007. Little is known about the role of e-cigarette psychosocial factors on risk of e-cigarette or cigarette use in adolescence., METHODS: Information on e-cigarette and cigarette psychosocial factors (use and attitudes about use in the home and among friends) was collected from 11th- and 12th-grade participants in the Southern California Children's Health Study during the spring of 2014., RESULTS: Of 2084 participants, 499 (24.0%) had used an e-cigarette, including 200 (9.6%) current users (past 30 days); 390 participants (18.7%) had smoked a combustible cigarette, and 119 (5.7%) were current cigarette smokers. Cigarette and e-cigarette use were correlated. Nevertheless, 40.5% (n = 81) of current e-cigarette users had never smoked a cigarette. Psychosocial factors (home use of each product, friends' use of and positive attitudes toward e-cigarettes and cigarettes) and participant perception of the harm of e-cigarettes were strongly positively associated both with e-cigarette and cigarette use. Most youth who reported e-cigarette use had friends who used e-cigarettes, and almost half of current users reported that they did not believe there were health risks associated with e-cigarette use., CONCLUSIONS: Longitudinal studies of adolescents are needed to determine whether the strong association of e-cigarette psychosocial factors with both e-cigarette and

	cigarette use will lead to increased cigarette use or dual use of cigarettes and e-cigarettes, or whether e-cigarettes will serve as a gateway to cigarette use.Copyright © 2015 by the American Academy of Pediatrics.
1761	Michael Craig Watson, Mark Forshaw Why we shouldn't normalise the use of e-cigarettes.. BMJ (Clinical research ed.); 2015;351:h3770.<br><br>**Keywords:**<br>*Electronic Nicotine Delivery Systems,*Health Policy/lj [Legislation & Jurisprudence],Humans,*Public Opinion,*Social Perception,*Tobacco Industry<br><br>**Abstract:**
1767	Andrew Cooke, Jennifer Fergeson, Adeeb Bulkhi, Thomas B Casale The Electronic Cigarette: The Good, the Bad, and the Ugly.. The journal of allergy and clinical immunology. In practice; 2015;3:498.<br><br>**Keywords:**<br>Electronic Nicotine Delivery Systems/ae [Adverse Effects],Electronic Nicotine Delivery Systems/is [Instrumentation],Electronic Nicotine Delivery Systems/ut [Utilization],*Electronic Nicotine Delivery Systems,Humans,Nicotine/ad [Administration & Dosage],Smoking Cessation<br><br>**Abstract:** Electronic cigarettes (EC) are battery-powered nicotine delivery systems that have increased in popularity since they entered the US market. EC has been reported to contain less carcinogens than traditional cigarettes, cause less acute lung effects in healthy individuals, and may help with smoking cessation. It has also been viewed as a potential safer alternative for asthmatic smokers, but its effects on lung functions are unclear. However, EC do carry some harmful aspects as they contain formaldehyde and formaldehyde-forming hemiacetals as well as potentially toxic particulate matter that deposits on surfaces. EC are an increasingly popular device that could serve as a gateway into traditional cigarette smoking or illicit drugs. The popularity of EC has brought with it money from large tobacco corporations and mass marketing. Lack of regulation has generated product inconsistency and potential health hazards. This review highlights what is known and what still needs to be answered about EC. Copyright © 2015 American Academy of Allergy, Asthma & Immunology. Published by Elsevier Inc. All rights reserved.
1774	Man Ping Wang, Sai Yin Ho, Lok Tung Leung, Tai Hing Lam Electronic cigarette use and its association with smoking in Hong Kong Chinese adolescents.. Addictive behaviors; 2015;50:124.<br><br>**Keywords:**<br>Adolescent,*Adolescent Behavior/px [Psychology],Cross-Sectional Studies,*Electronic Nicotine Delivery Systems/px [Psychology],*Electronic Nicotine Delivery Systems/sn [Statistics & Numerical Data],Female, Hong Kong/ep [Epidemiology],Humans,Intention,Male,Odds Ratio,*Smoking/ep [Epidemiology], *Smoking/px [Psychology],Socioeconomic Factors<br><br>**Abstract:** BACKGROUND: Electronic cigarettes (e-cigarettes) are increasingly used in adolescents with unknown impacts on conventional cigarette smoking. We examined the associations of e-cigarette use with smoking intention, nicotine addiction and smoking cessation in Chinese adolescents., METHODS: A total of 45,128 students (age 14.6 +/- 1.9; boys 51.4%) from 75 randomly selected schools in Hong Kong reported e-cigarette use (in the past 30 days), conventional cigarette use and socio-demographic characteristics in an anonymous questionnaire survey. Adjusted odds ratios (AORs) of intention to smoke, morning smoking urge, intention to quit and quit attempts; and beta-coefficient (beta) of cigarette consumption per day were calculated in relation to e-cigarette use., RESULTS: E-cigarette use was associated with intention to smoke with an AOR (95% CI) of 1.74 (1.30-2.31) in all students, 2.18 (1.12-4.23) in never and 2.79 (2.05-3.79) in ever smokers (non-significant interaction by smoking status). The associations were also significant in experimental and former smokers but not in current smokers. In

	current smokers, e-cigarette use was significantly associated with heavier smoking (beta 2.54, 95% CI 1.28-3.81) and morning smoking urge (AOR 2.54, 95% CI 1.50-3.11), and non-significantly associated with lower quit intention (0.76, 0.52-1.09) and attempts (0.80, 0.56-1.23)., CONCLUSIONS: E-cigarette use was associated with smoking intention in never, experimental and former smokers in Hong Kong Chinese adolescents. In current smokers, e-cigarette use was associated with nicotine addiction but not quit intention and attempts. Prospective studies with detailed measurements on e-cigarette use are warranted for further studies.Copyright © 2015 Elsevier Ltd. All rights reserved.
1776	Stephen T Higgins, Allison N Kurti, Ryan Redner, Thomas J White, Diann E Gaalema, Megan E Roberts, Nathan J Doogan, Jennifer W Tidey, Mollie E Miller, Cassandra A Stanton, Jack E Henningfield, Gary S Atwood A literature review on prevalence of gender differences and intersections with other vulnerabilities to tobacco use in the United States, 2004-2014.. Preventive medicine; 2015;80:89.<br><br>**Keywords:**<br>Adolescent,Adult,Age Factors,Aged,Electronic Nicotine Delivery Systems/px [Psychology],Female,History, 21st Century,Humans,Male,Middle Aged,Prevalence,Sex Factors,Tobacco Use/ep [Epidemiology], *Tobacco Use/td [Trends],United States/ep [Epidemiology],Young Adult<br><br>**Abstract:** This report describes results from a systematic literature review examining gender differences in U.S. prevalence rates of current use of tobacco and nicotine delivery products and how they intersect with other vulnerabilities to tobacco use. We searched PubMed on gender differences in tobacco use across the years 2004-2014. For inclusion, reports had to be in English, in a peer-reviewed journal or federal government report, report prevalence rates for current use of a tobacco product in males and females, and use a U.S. nationally representative sample. Prevalence rates were generally higher in males than in females across all products. This pattern remained stable despite changes over time in overall prevalence rates. Gender differences generally were robust when intersecting with other vulnerabilities, although decreases in the magnitude of gender differences were noted among younger and older users, and among educational levels and race/ethnic groups associated with the highest or lowest prevalence rates. Overall, these results document a pervasive association of gender with vulnerability to tobacco use that acts additively with other vulnerabilities. These vulnerabilities should be considered whenever formulating tobacco control and regulatory policies. Copyright © 2015 Elsevier Inc. All rights reserved.
1780	Jonathan Gornall Why e-cigarettes are dividing the public health community.. BMJ (Clinical research ed.); 2015;350:h3317.<br><br>**Keywords:**<br>Dissent and Disputes,Electronic Nicotine Delivery Systems/ae [Adverse Effects],*Electronic Nicotine Delivery Systems,Government Regulation,Harm Reduction,*Health Policy/lj [Legislation & Jurisprudence], Health Promotion,Humans,*Public Opinion,Smoke-Free Policy/lj [Legislation & Jurisprudence],Social Media,*Social Perception,Tobacco Industry/lj [Legislation & Jurisprudence],*Tobacco Industry,United Kingdom<br><br>**Abstract:**
1784	Melissa D Blank, Thomas Eissenberg Commentary on Brose et al. (2015): Protecting individual and public health by regulating electronic cigarette nicotine delivery.. Addiction (Abingdon, England); 2015;110:1169.<br><br>**Keywords:**<br>*Electronic Nicotine Delivery Systems,Humans,*Nicotine,Public Health,Smoking,Smoking Cessation, Tobacco Products,Tobacco Use Cessation Products<br><br>**Abstract:**

1786	Joan-Carles Suris, Andre Berchtold, Christina Akre Reasons to use e-cigarettes and associations with other substances among adolescents in Switzerland.. Drug and alcohol dependence; 2015;153:140.<br><br>**Keywords:**<br>Adolescent,*Adolescent Behavior/px [Psychology],Alcohol Drinking/ep [Epidemiology],Alcohol Drinking/px [Psychology],*Electronic Nicotine Delivery Systems/px [Psychology],Electronic Nicotine Delivery Systems/sn [Statistics & Numerical Data],Exploratory Behavior,Female,Humans,Longitudinal Studies,Male,Marijuana Smoking/ep [Epidemiology],Marijuana Smoking/px [Psychology],Sex Factors, Smoking/ep [Epidemiology],Smoking/px [Psychology],Students/px [Psychology],Students/sn [Statistics & Numerical Data],Switzerland<br><br>**Abstract:** BACKGROUND: The objectives of this research were to describe the main reason(s) why adolescents use electronic cigarettes, to assess how e-cigarette experimenters and users differ based on personal characteristics, and to determine whether its use is associated with the use of other substances among a representative sample of youths in Switzerland., METHODS: A representative sample of 621 youths (308 females) was divided into never users (n=353), experimenters (Only once, n=120) and users (Several times, n=148) of e-cigarettes. Groups were compared on socio-demographic data and current smoking, alcohol misuse and cannabis use. Reasons for e-cigarette use were compared between experimenters and users. A multinomial regression was performed using never users as the reference category., RESULTS: Forty-three percent had ever tried e-cigarettes, and the main reason was curiosity. Compared to never users, experimenters were more likely to be out of school (Relative Risk Ratio [RRR]: 2.68) and to misuse alcohol (RRR: 2.08), while users were more likely to be male (RRR: 2.75), to be vocational students (RRR: 2.30) or out of school (RRR: 3.48) and to use any of the studied substances (tobacco, RRR: 5.26; alcohol misuse, RRR: 2.71; cannabis use, RRR: 30.2)., CONCLUSIONS: Although often still part of adolescent experimentation, e-cigarettes are becoming increasingly popular among adolescents and they should become part of health providers' standard substance use screening. As health providers (and especially paediatricians) do not seem to have high levels of knowledge and, consequently, little comfort in discussing e-cigarettes, training in this domain should be available to them.Copyright © 2015 Elsevier Ireland Ltd. All rights reserved.
1789	Erin L Sutfin, Beth A Reboussin, Beata Debinski, Kimberly G Wagoner, John Spangler, Mark Wolfson The Impact of Trying Electronic Cigarettes on Cigarette Smoking by College Students: A Prospective Analysis.. American journal of public health; 2015;105:e83.<br><br>**Keywords:**<br>Electronic Nicotine Delivery Systems/px [Psychology],*Electronic Nicotine Delivery Systems/ut [Utilization],Humans,Male,North Carolina/ep [Epidemiology],*Smoking/ep [Epidemiology],Smoking/px [Psychology],Students/px [Psychology],*Students/sn [Statistics & Numerical Data],Surveys and Questionnaires,Universities,Virginia/ep [Epidemiology]<br><br>**Abstract:** OBJECTIVES: We assessed the impact of trying electronic cigarettes (e-cigarettes) on future cigarette smoking in a sample of smokers enrolled in college., METHODS: In this longitudinal study, first-semester college students at 7 colleges in North Carolina and 4 in Virginia completed a baseline survey and 5 follow-up surveys between fall 2010 and fall 2013. Current cigarette smoking at wave 6 was the primary outcome. Participants (n=271) reported current cigarette smoking at baseline and no history of e-cigarette use. We measured trying e-cigarettes at each wave, defined as use in the past 6 months., RESULTS: By wave 5, 43.5% had tried e-cigarettes. Even after controlling for other variables associated with cigarette smoking, trying e-cigarettes was a significant predictor of cigarette smoking at wave 6 (adjusted odds ratio [AOR]=2.48; 95% confidence interval [CI]=1.32, 4.66), as were friends' cigarette smoking (AOR=4.20; 95% CI=2.22, 7.96) and lifetime use of other tobacco products (AOR=1.63; 95% CI=1.22, 2.17)., CONCLUSIONS: Trying e-cigarettes during college did not deter cigarette smoking and may have contributed to continued smoking.

1793	Joshua M Sharfstein Electronic cigarettes: gateway to understanding the FDA?.. The Milbank quarterly; 2015;93:251.<br><br>**Keywords:**<br>Electronic Nicotine Delivery Systems/ae [Adverse Effects],*Electronic Nicotine Delivery Systems/st [Standards],*Government Regulation,Harm Reduction,Humans,Smoking/ae [Adverse Effects],Smoking Cessation/lj [Legislation & Jurisprudence],*Smoking Cessation/mt [Methods],*Tobacco Use Cessation Products/st [Standards],United States,*United States Food and Drug Administration/st [Standards]<br><br>**Abstract:**
1799	Bhargava Krishna Nelluri, Katie Murphy, Farouk Mookadam Electronic cigarettes and cardiovascular risk: hype or up in smoke?.. Future cardiology; 2015;11:271.<br><br>**Keywords:**<br>Cardiovascular Diseases/ep [Epidemiology],Cardiovascular Diseases/et [Etiology],Cardiovascular Diseases/pc [Prevention & Control],*Cardiovascular Diseases,*Electronic Nicotine Delivery Systems, Humans,Prevalence,Risk Factors,*Smoking/ae [Adverse Effects],*Smoking Cessation/mt [Methods], United States/ep [Epidemiology]<br><br>**Abstract:**
1801	Menglu Yuan, Sarah J Cross, Sandra E Loughlin, Frances M Leslie Nicotine and the adolescent brain.. The Journal of physiology; 2015;593:3397.<br><br>**Keywords:**<br>Adolescent,*Adolescent Development/de [Drug Effects],Animals,*Brain/de [Drug Effects],Brain/gd [Growth & Development],Brain/ph [Physiology],Humans,*Nicotine/pd [Pharmacology],*Nicotinic Agonists/pd [Pharmacology]<br><br>**Abstract:** Adolescence encompasses a sensitive developmental period of enhanced clinical vulnerability to nicotine, tobacco, and e-cigarettes. While there are sociocultural influences, data at preclinical and clinical levels indicate that this adolescent sensitivity has strong neurobiological underpinnings. Although definitions of adolescence vary, the hallmark of this period is a profound reorganization of brain regions necessary for mature cognitive and executive function, working memory, reward processing, emotional regulation, and motivated behavior. Regulating critical facets of brain maturation are nicotinic acetylcholine receptors (nAChRs). However, perturbations of cholinergic systems during this time with nicotine, via tobacco or e-cigarettes, have unique consequences on adolescent development. In this review, we highlight recent clinical and preclinical data examining the adolescent brain's distinct neurobiology and unique sensitivity to nicotine. First, we discuss what defines adolescence before reviewing normative structural and neurochemical alterations that persist until early adulthood, with an emphasis on dopaminergic systems. We review how acute exposure to nicotine impacts brain development and how drug responses differ from those seen in adults. Finally, we discuss the persistent alterations in neuronal signaling and cognitive function that result from chronic nicotine exposure, while highlighting a low dose, semi-chronic exposure paradigm that may better model adolescent tobacco use. We argue that nicotine exposure, increasingly occurring as a result of e-cigarette use, may induce epigenetic changes that sensitize the brain to other drugs and prime it for future substance abuse. Copyright © 2015 The Authors. The Journal of Physiology © 2015 The Physiological Society.
1804	Carl V Phillips Gateway Effects: Why the Cited Evidence Does Not Support Their Existence for Low-Risk Tobacco Products (and What Evidence Would).. International journal of environmental research and public health; 2015;12:5439.

	**Keywords:**<br>Bayes Theorem,Causality,Confounding Factors (Epidemiology),Cross-Sectional Studies,*Electronic Nicotine Delivery Systems,Harm Reduction,Humans,Propensity Score,*Smoking/px [Psychology],Smoking Cessation,Tobacco,Tobacco Industry,*Tobacco Use Disorder/et [Etiology],*Tobacco, Smokeless<br><br>**Abstract:** It is often claimed that low-risk drugs still create harm because of "gateway effects", in which they cause the use of a high-risk alternative. Such claims are popular among opponents of tobacco harm reduction, claiming that low-risk tobacco products (e.g., e-cigarettes, smokeless tobacco) cause people to start smoking, sometimes backed by empirical studies that ostensibly support the claim. However, these studies consistently ignore the obvious alternative causal pathways, particularly that observed associations might represent causation in the opposite direction (smoking causes people to seek low-risk alternatives) or confounding (the same individual characteristics increase the chance of using any tobacco product). Due to these complications, any useful analysis must deal with simultaneity and confounding by common cause. In practice, existing analyses seem almost as if they were designed to provide teaching examples about drawing simplistic and unsupported causal conclusions from observed associations. The present analysis examines what evidence and research strategies would be needed to empirically detect such a gateway effect, if there were one, explaining key methodological concepts including causation and confounding, examining the logic of the claim, identifying potentially useful data, and debunking common fallacies on both sides of the argument, as well as presenting an extended example of proper empirical testing. The analysis demonstrates that none of the empirical studies to date that are purported to show a gateway effect from tobacco harm reduction products actually does so. The observations and approaches can be generalized to other cases where observed association of individual characteristics in cross-sectional data could result from any of several causal relationships.
1810	Sarah L White, Jacqui Bell, Quit Victoria We didn't start this fireless vapour: E-cigarette legislation in Australia".. Journal of law and medicine; 2015;22:546.<br><br>**Keywords:**<br>*Electronic Nicotine Delivery Systems,*Government Regulation,Humans<br><br>**Abstract:**
1811	Terry Slevin "We didn't start this fireless vapour: E-cigarette legislation in Australia".. Journal of law and medicine; 2015;22:545.<br><br>**Keywords:**<br>*Electronic Nicotine Delivery Systems,*Government Regulation,Humans<br><br>**Abstract:**
1813	Simon Chapman The future of electronic cigarette growth depends on youth uptake.. The Medical journal of Australia; 2015;202:467.<br><br>**Keywords:**<br>Adolescent,Adolescent Behavior,*Electronic Nicotine Delivery Systems,*Health Knowledge, Attitudes, Practice,Health Policy/lj [Legislation & Jurisprudence],Humans,Smoking/ep [Epidemiology],Smoking Cessation,*Smoking Prevention,Tobacco Industry<br><br>**Abstract:**
1819	Ingrid Torjesen Tobacco industry is investing in e-cigarette types least likely to help smokers quit.. BMJ (Clinical research ed.); 2015;350:h2133.<br><br>**Keywords:**

	Consumer Health Information,Electronic Nicotine Delivery Systems/px [Psychology],Electronic Nicotine Delivery Systems/ut [Utilization],*Electronic Nicotine Delivery Systems,Equipment Design,Health Knowledge, Attitudes, Practice,Humans,*Smoking Cessation,Taste,*Tobacco Industry<br><br>**Abstract:**
1821	Michael McCarthy "Alarming" rise in popularity of e-cigarettes is seen among US teenagers as use triples in a year.. BMJ (Clinical research ed.); 2015;350:h2083.<br><br>**Keywords:**<br>Adolescent,Adolescent Behavior,Electronic Nicotine Delivery Systems/px [Psychology],Electronic Nicotine Delivery Systems/sn [Statistics & Numerical Data],*Electronic Nicotine Delivery Systems,Health Knowledge, Attitudes, Practice,Health Surveys,Humans,*Nicotine/ad [Administration & Dosage], Prevalence,Smoking/ep [Epidemiology],Smoking/px [Psychology],*Smoking,Smoking Cessation,Surveys and Questionnaires,United States/ep [Epidemiology]<br><br>**Abstract:**
1828	Graham Moore, Gillian Hewitt, John Evans, Hannah J Littlecott, Jo Holliday, Nilufar Ahmed, Laurence Moore, Simon Murphy, Adam Fletcher Electronic-cigarette use among young people in Wales: evidence from two cross-sectional surveys.. BMJ open; 2015;5:e007072.<br><br>**Keywords:**<br>Adolescent,Child,Cross-Sectional Studies,*Electronic Nicotine Delivery Systems/ut [Utilization],Female, Humans,Logistic Models,Male,*Marijuana Smoking/ep [Epidemiology],Odds Ratio,Prevalence,Schools, *Smoking/ep [Epidemiology],Surveys and Questionnaires,*Tobacco Products/ut [Utilization],Wales/ep [Epidemiology]<br><br>**Abstract:** OBJECTIVES: To examine the prevalence of electronic(e)-cigarette use, prevalence of e-cigarette and tobacco use by age, and associations of e-cigarette use with sociodemographic characteristics, tobacco and cannabis use among young people in Wales., DESIGN: Data from two nationally-representative cross-sectional surveys undertaken in 2013-2014. Logistic regression analyses, adjusting for school-level clustering, examined sociodemographic characteristics of e-cigarette use, and associations between e-cigarette use and smoking., SETTING: Primary and secondary schools in Wales., PARTICIPANTS: Primary-school children aged 10-11 (n=1601) and secondary-school students aged 11-16 (n=9055)., RESULTS: Primary-school children were more likely to have used e-cigarettes (5.8%) than tobacco (1.6%). Ever use of e-cigarettes remained more prevalent than ever use of tobacco until age 14-15. Overall, 12.3% of secondary-school students (aged 11-16) reported ever using e-cigarettes, with no differences according to gender, ethnicity or family affluence. The percentage of 'never smokers' reporting having used e-cigarettes was 5.3% at age 10-11 to 8.0% at age 15-16. The proportion of children who had ever used an e-cigarette and reported currently smoking increased from 6.9% among 10-11 year olds to 39.2% in 15-16 year olds. Only 1.5% (n=125) of 11-16 year-olds, including 0.3% of never smokers, reported regular e-cigarette use (use at least once a month). Current weekly smokers were 100 times more likely than non-smokers to report regular e-cigarette use (relative risk ratio (RRR=121.15; 95% CI 57.56 to 254.97). Regular e-cigarette use was also more likely among those who had smoked cannabis (RRR 53.03; 95% CI 38.87 to 80.65)., CONCLUSIONS: Many young people (including never-smokers) have tried e-cigarettes. However, regular use is less common, and is associated with tobacco cigarette use. Longitudinal research is needed to understand age-related trajectories of e-cigarette use and to understand the temporal nature of relationships between e-cigarette and tobacco use.Copyright Published by the BMJ Publishing Group Limited. For permission to use (where not already granted under a licence) please go to http://group.bmj.com/group/rights-licensing/permissions.

1830	Bryant Furlow US Government warns against long-term dual use of conventional and e-cigarettes.. The Lancet. Respiratory medicine; 2015;3:345.<br><br>**Keywords:**<br>Centers for Disease Control and Prevention (U.S.),*Electronic Nicotine Delivery Systems/ae [Adverse Effects],Humans,*Smoking/ae [Adverse Effects],United States,*United States Dept. of Health and Human Services<br><br>**Abstract:**
1834	Tracey E Barnett, Eric K Soule, Jamie R Forrest, Lauren Porter, Scott L Tomar Adolescent Electronic Cigarette Use: Associations With Conventional Cigarette and Hookah Smoking.. American journal of preventive medicine; 2015;49:199.<br><br>**Keywords:**<br>Adolescent,Child,*Electronic Nicotine Delivery Systems/ut [Utilization],Female,Florida/ep [Epidemiology], Humans,Logistic Models,Male,Prevalence,Sex Factors,*Smoking/ep [Epidemiology],*Students/sn [Statistics & Numerical Data],Surveys and Questionnaires<br><br>**Abstract:** INTRODUCTION: The emerging trends and rapid growth of electronic cigarettes (e-cigarettes) among adolescents are being monitored closely. The trends are critical as policy to prevent uptake among adolescents is considered. The purpose of this study is to describe the prevalence of e-cigarette use and potential correlates for use. Associations between e-cigarettes, cigarettes, and hookah are assessed., METHODS: This study used data from the 2013 Florida Youth Tobacco Survey. Prevalence estimates were calculated in 2014 and differences were determined based on CIs. Adjusted logistic regression models were used to identify correlates of e-cigarette use among participants based on demographic and other tobacco products used., RESULTS: There were no sex differences in middle school, whereas male high school students reported higher use than their female counterparts. Cigarette smoking and hookah use were significantly associated with ever and current e-cigarette use among both middle and high school students., CONCLUSIONS: Although e-cigarettes are being assessed as a potential replacement product for traditional tobacco, evidence from this study indicates the possibility of multiple product use among adolescents. E-cigarettes are not only associated with traditional cigarettes, but also with hookahs, a similar emerging product that offer tobacco flavors that may appeal to adolescents. Notably, many e-cigarette users also reported no cigarette or hookah use.Copyright © 2015 American Journal of Preventive Medicine. Published by Elsevier Inc. All rights reserved.
1858	Lisa Henriksen The retail environment for tobacco: a barometer of progress towards the endgame.. Tobacco control; 2015;24:e1.<br><br>**Keywords:**<br>*Commerce,Electronic Nicotine Delivery Systems,Humans,*Smoking,*Smoking Cessation,Tobacco, *Tobacco Industry,*Tobacco Products<br><br>**Abstract:**
1860	Tony Kirby Do vaping adverts threaten tobacco control progress in the UK?.. The Lancet. Respiratory medicine; 2015;3:107.<br><br>**Keywords:**<br>Advertising as Topic/lj [Legislation & Jurisprudence],*Advertising as Topic/td [Trends],*Electronic Nicotine Delivery Systems/td [Trends],Humans,Television/lj [Legislation & Jurisprudence],*Tobacco Use Cessation,United Kingdom

	**Abstract:**
1862	M L Saddleson, L T Kozlowski, G A Giovino, L W Hawk, J M Murphy, M G MacLean, M L Goniewicz, G G Homish, B H Wrotniak, M C Mahoney Risky behaviors, e-cigarette use and susceptibility of use among college students.. Drug and alcohol dependence; 2015;149:25.<br><br>**Keywords:**<br>Age Factors,Alcohol Drinking/px [Psychology],Cross-Sectional Studies,Disease Susceptibility,*Electronic Nicotine Delivery Systems,European Continental Ancestry Group,Female,Humans,Male,Marijuana Smoking/px [Psychology],Prevalence,*Risk-Taking,Sex Factors,*Smoking/ep [Epidemiology],*Smoking/px [Psychology],Socioeconomic Factors,Young Adult<br><br>**Abstract:** BACKGROUND: Since 2007, there has been a rise in the use of electronic cigarettes (e-cigarettes). The present study uses cross-sectional data (2013) to examine prevalence, correlates and susceptibility to e-cigarettes among young adults., METHODS: Data were collected using an Internet survey from a convenience sample of 1437, 18-23 year olds attending four colleges/universities in Upstate New York. Results were summarized using descriptive statistics; logistic regression models were analyzed to identify correlates of e-cigarette use and susceptibility to using e-cigarettes., RESULTS: Nearly all respondents (95.5%) reported awareness of e-cigarettes; 29.9% were ever users and 14.9% were current users. Younger students, males, non-Hispanic Whites, respondents reporting average/below average school ability, ever smokers and experimenters of tobacco cigarettes, and those with lower perceptions of harm regarding e-cigarettes demonstrated higher odds of ever use or current use. Risky behaviors (i.e., tobacco, marijuana or alcohol use) were associated with using e-cigarettes. Among never e-cigarette users, individuals involved in risky behaviors or, with lower harm perceptions for e-cigarettes, were more susceptible to future e-cigarette use., CONCLUSIONS: More e-cigarette users report use of another nicotine product besides e-cigarettes as the first nicotine product used; this should be considered when examining whether e-cigarette use is related to cigarette susceptibility. Involvement in risky behaviors is related to e-cigarette use and susceptibility to e-cigarette use. Among college students, e-cigarette use is more likely to occur in those who have also used other tobacco products, marijuana, and/or alcohol.Copyright © 2015 Elsevier Ireland Ltd. All rights reserved.
1868	Harold P Wimmer E-cigarettes: a new challenge for public health and for school nurses.. NASN school nurse (Print); 2015;30:26.<br><br>**Keywords:**<br>Adolescent,Child,*Electronic Nicotine Delivery Systems,*Health Education,Health Promotion,Humans, *Life Style,Public Health,*School Nursing,Students<br><br>**Abstract:**
1879	Susan Galandiuk E-cigarettes: an asset or liability in efforts to lessen tobacco smoking and its consequences.. Mayo Clinic proceedings; 2015;90:1.<br><br>**Keywords:**<br>*Directive Counseling/mt [Methods],*Elective Surgical Procedures/ae [Adverse Effects],*Electronic Nicotine Delivery Systems/mt [Methods],Female,Humans,Male,*Nicotine/pd [Pharmacology], *Postoperative Complications,*Smoking,*Smoking Cessation/mt [Methods],*Tobacco Use Disorder/th [Therapy]<br><br>**Abstract:**
1882	Emily Anne McDonald, Pamela M Ling One of several 'toys' for smoking: young adult experiences with electronic cigarettes in New York City.. Tobacco control; 2015;24:588.

	**Keywords:**<br>Adolescent,Adult,*Electronic Nicotine Delivery Systems/sn [Statistics & Numerical Data],Female,Focus Groups,Humans,Interviews as Topic,Male,*Marketing,New York City/ep [Epidemiology],*Smoking/ep [Epidemiology],*Tobacco Products/sn [Statistics & Numerical Data],Young Adult<br><br>**Abstract:** OBJECTIVE: This qualitative research explores the use of electronic cigarettes and other similar 'vapor' delivery devices among young adults in New York City., METHODS: We employed 17 focus groups followed by 12 semistructured interviews to understand the beliefs, opinions and practices related to the use of electronic cigarettes among young adult smokers (N=87)., RESULTS: Participants were mainly daily (52%) and non-daily (41%) smokers. While experimentation with electronic cigarette devices was frequently reported, participants related an overall lack of information about the devices and what they did know often reflected messages in e-cigarette marketing campaigns. Participants also used their own bodily sensations as a way to gauge potential risks and benefits of the products. Finally, young adults, steeped in a culture of personal technologies, perceived e-cigarettes as one more 'toy' among other technologies integrated into their everyday lives., DISCUSSION: E-cigarettes were also frequently used with other tobacco products, including conventional cigarettes. Our research indicates that public health campaigns may be needed to counter current industry marketing and inform the public that electronic cigarettes are currently unregulated, understudied and contain toxicants and carcinogens.Copyright Published by the BMJ Publishing Group Limited. For permission to use (where not already granted under a licence) please go to http://www.bmj.com/company/products-services/rights-and-licensing/
1890	Ellen M Meier, Alayna P Tackett, Mary B Miller, DeMond M Grant, Theodore L Wagener Which nicotine products are gateways to regular use? First-tried tobacco and current use in college students.. American journal of preventive medicine; 2015;48:S86.<br><br>**Keywords:**<br>Data Collection,Female,Humans,Male,*Students/sn [Statistics & Numerical Data],*Tobacco Products/sn [Statistics & Numerical Data],*Tobacco Use/ep [Epidemiology],*Tobacco Use Disorder/ep [Epidemiology], Universities,Young Adult<br><br>**Abstract:** BACKGROUND: The potential for emerging tobacco products (ETPs) to be gateway products for further tobacco use among youth is of significant concern., PURPOSE: To examine use of various nicotine-containing products on a tobacco-free college campus and whether the first product tried predicts subsequent tobacco use., METHODS: Undergraduate students (N=1,304) at a large university completed an online survey of past/current use of cigarettes; smokeless tobacco (SLT); hookah; ETPs (dissolvables, snus, and electronic cigarettes); and nicotine replacement therapy (NRT). Data were collected from September 2012 to May 2013 and analyses were conducted from June to September 2013. Students were classified as single, dual, or poly tobacco users., RESULTS: The sample consisted of 79.5% non-users, 13.8% single, 4.4% dual, and 1.5% poly users. Overall, 49.4% of participants reported trying a tobacco product. Hookah was the most tried product (38%), but cigarettes were most often the first product ever tried (51%). First product tried did not predict current tobacco use and non-use, but individuals who first tried SLT or cigarettes (rather than hookah or ETPs) were more likely to be poly tobacco users. Current tobacco users who first tried ETPs or hookah were largely non-daily users of hookah; current tobacco users who first tried cigarettes or SLT were largely non-daily or daily users of cigarettes/SLT., CONCLUSIONS: Hookah and ETPs are increasingly becoming the first tobacco product ever tried by youth; however, uptake of ETPs is poor, unlike cigarettes and SLT, and does not appear to lead to significant daily/non-daily use of cigarettes and SLT.Copyright © 2015 American Journal of Preventive Medicine. Published by Elsevier Inc. All rights reserved.
1895	Mary Ann Pentz, HeeSung Shin, Nathaniel Riggs, Jennifer B Unger, Katherine L Collison, Chih-Ping Chou Parent, peer, and executive function relationships to early adolescent e-cigarette use: a substance use pathway?.. Addictive behaviors; 2015;42:73.

	**Keywords:**<br>Adolescent,*Alcohol Drinking/ep [Epidemiology],Alcohol Drinking/px [Psychology],Child,Electronic Nicotine Delivery Systems/px [Psychology],*Electronic Nicotine Delivery Systems/sn [Statistics & Numerical Data],*Executive Function,Female,Humans,*Interpersonal Relations,Male,*Parent-Child Relations,Peer Group,Prevalence,*Smoking/ep [Epidemiology],Smoking/px [Psychology],Substance-Related Disorders/ep [Epidemiology],Substance-Related Disorders/px [Psychology]<br><br>**Abstract:** INTRODUCTION: Little is known about influences on e-cigarette use among early adolescents. This study examined influences that have been previously found to be associated with gateway drug use in adolescents: demographic (age, gender, ethnicity, free lunch), social contextual influences of parents and peers, and executive function deficits (EF)., METHODS: A cross-sectional survey was administered to 410 7th grade students from two diverse school districts in Southern California (M age;=12.4years, 48.3% female, 34.9% on free lunch (low socioeconomic status), 45.1% White, 25.4% Hispanic/Latino, 14.9% Mixed/bi-racial.) Logistic regression analyses examined influences of demographic, parent e-cigarette ownership and peer use, and EF on lifetime e-cigarette, and gateway drug use (cigarette and/or alcohol use)., RESULTS: Lifetime use prevalence was 11.0% for e-cigarettes, 6.8% for cigarettes, and 38.1% for alcohol. Free lunch and age were marginally related to e-cigarette use (p<.10). Parent e-cigarette ownership was associated with use of all substances, while peer use was associated with gateway drug use (p's<.05-.001). EF deficits were associated with use of all substances five times more likely than others to use e-cigarettes and over twice as likely to use gateway drugs., CONCLUSIONS: E-cigarette and gateway drug use may have common underlying risk factors in early adolescence, including parent and peer modeling of substance use, as well as EF deficits. Future research is needed to examine longitudinal relationships of demographics, parent and peer modeling, and EF deficits to e-cigarette use in larger samples, trajectories of e-cigarette use compared to use of other substances, and the potential of EF skills training programs to prevent e-cigarette use.Copyright © 2014. Published by Elsevier Ltd.
1898	Victor M Cardenas, Philip J Breen, Cesar M Compadre, Robert R Delongchamp, Claudia P Barone, Martha M Phillips, Joseph Gary Wheeler The smoking habits of the family influence the uptake of e-cigarettes in US children.. Annals of epidemiology; 2015;25:60.<br><br>**Keywords:**<br>Adolescent,Child,Cross-Sectional Studies,District of Columbia/ep [Epidemiology],*Electronic Nicotine Delivery Systems/sn [Statistics & Numerical Data],Family,Humans,Imitative Behavior,*Nuclear Family, Prevalence,*Smoking/ep [Epidemiology],Smoking/px [Psychology],Students/px [Psychology], *Students/sn [Statistics & Numerical Data],Surveys and Questionnaires,United States/ep [Epidemiology], Young Adult<br><br>**Abstract:**
1904	Blair N Coleman, Benjamin J Apelberg, Bridget K Ambrose, Kerry M Green, Conrad J Choiniere, Rebecca Bunnell, Brian A King Association between electronic cigarette use and openness to cigarette smoking among US young adults.. Nicotine & tobacco research : official journal of the Society for Research on Nicotine and Tobacco; 2015;17:212.<br><br>**Keywords:**<br>Adolescent,Adult,*Behavior, Addictive,Electronic Nicotine Delivery Systems/px [Psychology],*Electronic Nicotine Delivery Systems/ut [Utilization],Female,Humans,Male,*Nicotine/ad [Administration & Dosage], *Smoking/ep [Epidemiology],Smoking/px [Psychology],*Smoking Cessation/sn [Statistics & Numerical Data],Surveys and Questionnaires,United States/ep [Epidemiology],Young Adult<br><br>**Abstract:** INTRODUCTION: Use of electronic nicotine delivery systems (ENDS), including electronic cigarettes (e-cigarettes), is increasing. One concern is the appeal of these products to youth and young

	adults and the potential to influence perceptions and use of conventional cigarettes., METHODS: Using data from the 2012-2013 National Adult Tobacco Survey, characteristics of adults aged 18-29 years who had never established cigarette smoking behavior were examined by ever use of e-cigarettes, demographics, and ever use of other tobacco products (smokeless tobacco, cigars, hookah, and cigarettes). Multivariate logistic regression was used to examine the relationship between e-cigarette use and openness to cigarette smoking among young adults, defined as the lack of a firm intention not to smoke soon or in the next year., RESULTS: Among young adults who had never established cigarette smoking behavior (unweighted n = 4,310), 7.9% reported having ever tried e-cigarettes, and 14.6% of those who reported having ever tried e-cigarettes also reported current use of the product. Ever e-cigarette use was associated with being open to cigarette smoking (adjusted odds ratio = 2.4; 95% confidence interval = 1.7, 3.3), as was being male, aged 18-24 years, less educated, and having ever used hookah or experimented with conventional cigarettes., CONCLUSIONS: Ever use of e-cigarettes and other tobacco products was associated with being open to cigarette smoking. This study does not allow us to assess the directionality of this association, so future longitudinal research is needed to illuminate tobacco use behaviors over time as well as provide additional insight on the relationship between ENDS use and conventional cigarette use among young adult populations.Copyright © The Author 2014. Published by Oxford University Press on behalf of the Society for Research on Nicotine and Tobacco. All rights reserved. For permissions, please e-mail: journals.permissions@oup.com.
1931	Rachel Kornfield, Jidong Huang, Lisa Vera, Sherry L Emery Rapidly increasing promotional expenditures for e-cigarettes.. Tobacco control; 2015;24:110.<br><br>**Keywords:**<br>*Electronic Nicotine Delivery Systems/ec [Economics],*Electronics,Humans,*Marketing/ec [Economics],*Nicotine/ad [Administration & Dosage],*Smoking,*Tobacco Industry/ec [Economics],Tobacco Products<br><br>**Abstract:**
1932	Youn Ok Lee, Annice E Kim 'Vape shops' and 'E-Cigarette lounges' open across the USA to promote ENDS.. Tobacco control; 2015;24:410.<br><br>**Keywords:**<br>*Commerce/lj [Legislation & Jurisprudence],*Commerce/td [Trends],Electronic Nicotine Delivery Systems/ec [Economics],*Electronic Nicotine Delivery Systems/td [Trends],Humans,United States,United States Food and Drug Administration<br><br>**Abstract:**
1933	Ollie Ganz, Jennifer Cantrell, Joyce Moon-Howard, Angela Aidala, Thomas R Kirchner, Donna Vallone Electronic cigarette advertising at the point-of-sale: a gap in tobacco control research.. Tobacco control; 2015;24:e110.<br><br>**Keywords:**<br>*Advertising as Topic,Commerce,*Electronic Nicotine Delivery Systems,Humans,Research,*Smoking, Smoking Cessation,Tobacco,*Tobacco Industry,Tobacco Products<br><br>**Abstract:**
1942	Jacqui Wise Children are three times as likely to try e-cigarettes as tobacco products, study finds.. BMJ (Clinical research ed.); 2014;349:g7508.<br><br>**Keywords:**<br>Child,*Electronic Nicotine Delivery Systems/sn [Statistics & Numerical Data],Humans,*Tobacco Products/sn [Statistics & Numerical Data],Tobacco Smoke Pollution/sn [Statistics & Numerical Data],

	Wales<br><br>**Abstract:**
1946	Melissa Shah Electronic cigarettes: the debate continues.. MD advisor : a journal for New Jersey medical community; 2014;7:34.<br><br>**Keywords:**<br>Carcinogens,*Electronic Nicotine Delivery Systems,Humans,Marketing,Nicotine/ad [Administration & Dosage],United States,*United States Food and Drug Administration/lj [Legislation & Jurisprudence]<br><br>**Abstract:**
1948	Rachelle Springer Electronic cigarettes--patient safety concerns.. Plastic surgical nursing : official journal of the American Society of Plastic and Reconstructive Surgical Nurses; 2014;34:165.<br><br>**Keywords:**<br>Electronic Nicotine Delivery Systems/ae [Adverse Effects],*Electronic Nicotine Delivery Systems/st [Standards],*Health Promotion/mt [Methods],Humans,*Nicotine/tu [Therapeutic Use],*Patient Safety/st [Standards]<br><br>**Abstract:**
1961	Ignatz Wessler Tumor-promoting activities of nicotine not addressed.. Deutsches Arzteblatt international; 2014;111:682.<br><br>**Keywords:**<br>*Electronic Nicotine Delivery Systems/mo [Mortality],*Electronic Nicotine Delivery Systems/ut [Utilization],Humans,*Lung Diseases/mo [Mortality],*Smoking/mo [Mortality],*Smoking Cessation/sn [Statistics & Numerical Data],*Smoking Prevention,*Tobacco Use Disorder/mo [Mortality]<br><br>**Abstract:**
1962	Maciej L Goniewicz, Michal Gawron, Justyna Nadolska, Lukasz Balwicki, Andrzej Sobczak Rise in electronic cigarette use among adolescents in Poland.. The Journal of adolescent health : official publication of the Society for Adolescent Medicine; 2014;55:713.<br><br>**Keywords:**<br>Adolescent,*Adolescent Behavior,*Attitude to Health,Commerce/sn [Statistics & Numerical Data], Electrical Equipment and Supplies/sn [Statistics & Numerical Data],*Electrical Equipment and Supplies/td [Trends],Female,*Ganglionic Stimulants/ad [Administration & Dosage],Humans,Male,Minors/sn [Statistics & Numerical Data],*Nicotine/ad [Administration & Dosage],Poland/ep [Epidemiology], Pregnancy,Prevalence,Risk Factors,Smoking/ep [Epidemiology],*Smoking/td [Trends],Tobacco Products/sn [Statistics & Numerical Data]<br><br>**Abstract:** PURPOSE: Despite the potential negative health effects of electronic cigarettes (e-cigarettes), these devices are increasing in popularity worldwide, especially among youth., METHODS: We compared data from two cross-sectional studies conducted in Poland among students aged 15-19 years in 2010-2011 and 2013-2014. We tested differences between samples in the prevalence of e-cigarette use, tobacco cigarette smoking, and simultaneous use of both tobacco and e-cigarettes ("dual use") using a multilevel linear mixed model regression., RESULTS: We found that the current use of e-cigarettes among adolescents in Poland was significantly higher in the 2013-2014 sample than the 2010-2011 sample (29.9% vs. 5.5%, respectively; $p < .05$). Dual use of tobacco and e-cigarettes was also significantly higher (21.8% vs. 3.6%, respectively; $p < .05$). Interestingly, the prevalence of smoking tobacco cigarettes also

	increased (from 23.9% in 2010-2011 to 38.0% in 2013-2014; p < .05)., CONCLUSIONS: Observed parallel increase in e-cigarette use and smoking prevalence does not support the idea that e-cigarettes are displacing tobacco cigarettes in this population.Copyright © 2014 Society for Adolescent Health and Medicine. Published by Elsevier Inc. All rights reserved.
1975	Nino Kunzli To e-smoke or not to e-smoke: is that a question?.. International journal of public health; 2014;59:679.<br><br>**Keywords:**<br>*Electronic Nicotine Delivery Systems/st [Standards],Female,*Health Promotion,Humans,Male, *Smoking/lj [Legislation & Jurisprudence],*Smoking Cessation/mt [Methods]<br><br>**Abstract:**
1976	Anonymous Confusion over electronic cigarettes continues.. Mayo Clinic health letter (English ed.); 2014;32:4.<br><br>**Keywords:**<br>Humans,*Nicotine/ad [Administration & Dosage],Tobacco Products,*Tobacco Use Cessation Products, United States,United States Food and Drug Administration,Volatilization<br><br>**Abstract:**
1977	Francesco Blasi, Brian Ward Electronic nicotine delivery systems (ENDS): the beginning of the end or the end of the beginning?.. The European respiratory journal; 2014;44:585.<br><br>**Keywords:**<br>*Electronic Nicotine Delivery Systems/td [Trends],*Electronic Nicotine Delivery Systems/ut [Utilization], Europe,Humans,*Nicotine/ad [Administration & Dosage],Pulmonary Medicine/mt [Methods],Pulmonary Medicine/td [Trends],Respiration Disorders/et [Etiology],Respiration Disorders/pc [Prevention & Control], Smoking/ae [Adverse Effects],*Smoking Cessation/mt [Methods],Societies, Medical,*Tobacco Products<br><br>**Abstract:**
1980	Kirsten Bell, Helen Keane All gates lead to smoking: the 'gateway theory', e-cigarettes and the remaking of nicotine.. Social science & medicine (1982); 2014;119:45.<br><br>**Keywords:**<br>*Electronic Nicotine Delivery Systems/px [Psychology],Humans,Mass Media,*Nicotine/ae [Adverse Effects],*Psychological Theory,*Smoking/px [Psychology]<br><br>**Abstract:** The idea that drug use in 'softer' forms leads to 'harder' drug use lies at the heart of the gateway theory, one of the most influential models of drug use of the twentieth century. Although hotly contested, the notion of the 'gateway drug' continues to rear its head in discussions of drug use--most recently in the context of electronic cigarettes. Based on a critical reading of a range of texts, including scholarly literature and media reports, we explore the history and gestation of the gateway theory, highlighting the ways in which intersections between academic, media and popular accounts actively produced the concept. Arguing that the theory has been critical in maintaining the distinction between 'soft' and 'hard' drugs, we turn to its distinctive iteration in the context of debates about e-cigarettes. We show that the notion of the 'gateway' has been transformed from a descriptive to a predictive model, one in which nicotine is constituted as simultaneously 'soft' and 'hard'--as both relatively innocuous and incontrovertibly harmful.Copyright © 2014 Elsevier Ltd. All rights reserved.

2005	Michael McCarthy Youth exposure to e-cigarette advertising on US television soars.. BMJ (Clinical research ed.); 2014;348:g3703.<br><br>**Keywords:**<br>Adolescent,Adult,*Advertising as Topic,Child,Humans,Prevalence,*Smoking/ep [Epidemiology], *Television,Tobacco Use Cessation Products/ut [Utilization],*Tobacco Use Cessation Products,United States/ep [Epidemiology],Young Adult<br><br>**Abstract:**
2021	Kate Hunt, Helen Sweeting You have been QUALIFIED for a smokeless e-cig starter kit.. Journal of epidemiology and community health; 2014;68:786.<br><br>**Keywords:**<br>*Advertising as Topic,*Electronic Nicotine Delivery Systems,Humans,Persuasive Communication, Smoking/px [Psychology]<br><br>**Abstract:**
2038	Zosia Kmietowicz E-cigarettes are "gateway devices" for smoking among young people, say researchers.. BMJ (Clinical research ed.); 2014;348:g2034.<br><br>**Keywords:**<br>Adolescent,*Electrical Equipment and Supplies/ut [Utilization],Humans,*Research Personnel,*Smoking Cessation/mt [Methods],*Smoking Prevention,United States,Young Adult<br><br>**Abstract:**
2055	Jessica K Pepper, Annie-Laurie McRee, Melissa B Gilkey Healthcare providers' beliefs and attitudes about electronic cigarettes and preventive counseling for adolescent patients.. The Journal of adolescent health : official publication of the Society for Adolescent Medicine; 2014;54:678.<br><br>**Keywords:**<br>Adolescent,Adolescent Behavior/px [Psychology],Analysis of Variance,*Attitude of Health Personnel, Child,*Counseling/mt [Methods],Counseling/sn [Statistics & Numerical Data],*Electronic Nicotine Delivery Systems/px [Psychology],Female,Health Knowledge, Attitudes, Practice,*Health Personnel/px [Psychology],Humans,Male,Minnesota,*Tobacco Use/pc [Prevention & Control],Tobacco Use/px [Psychology]<br><br>**Abstract:** PURPOSE: Electronic cigarettes (e-cigarettes) are battery-powered nicotine delivery systems that may serve as a "gateway" to tobacco use by adolescents. Use of e-cigarettes by U.S. adolescents rose from 3% in 2011 to 7% in 2012. We sought to describe healthcare providers' awareness of e-cigarettes and to assess their comfort with and attitudes toward discussing e-cigarettes with adolescent patients and their parents., METHODS: A statewide sample (n = 561) of Minnesota healthcare providers (46% family medicine physicians, 20% pediatricians, and 34% nurse practitioners) who treat adolescents completed an online survey in April 2013., RESULTS: Nearly all providers (92%) were aware of e-cigarettes, and 11% reported having treated an adolescent patient who had used them. The most frequently cited sources of information about e-cigarettes were patients, news stories, and advertisements, rather than professional sources. Providers expressed considerable concern that e-cigarettes could be a gateway to tobacco use but had moderately low levels of knowledge about and comfort discussing e-cigarettes with adolescent patients and their parents. Compared with pediatricians and nurse practitioners, family medicine physicians reported knowing more about e-cigarettes and being more comfortable discussing them with patients (both $p < .05$). Nearly all respondents (92%) wanted to

	learn more about e-cigarettes., CONCLUSIONS: Healthcare providers who treat adolescents may need to incorporate screening and counseling about e-cigarettes into routine preventive services, particularly if the prevalence of use continues to increase in this population. Education about e-cigarettes could help providers deliver comprehensive preventive services to adolescents at risk of tobacco use.Copyright © 2014 Society for Adolescent Health and Medicine. Published by Elsevier Inc. All rights reserved.
2060	Hye-Jin Paek, Sookyong Kim, Thomas Hove, Jung Yoon Huh Reduced harm or another gateway to smoking? source, message, and information characteristics of E-cigarette videos on YouTube.. Journal of health communication; 2014;19:545.<br><br>**Keywords:**<br>*Consumer Health Information/sn [Statistics & Numerical Data],Humans,*Internet,*Marketing/sn [Statistics & Numerical Data],Risk Assessment,Smoking/ep [Epidemiology],*Tobacco Products,United States/ep [Epidemiology],*Videotape Recording/sn [Statistics & Numerical Data]<br><br>**Abstract:** E-cigarettes are widely promoted on the Internet, but little is known about what kinds of information about them are available online. This study examines message, source, and health information characteristics of e-cigarette videos on the popular online video-sharing platform YouTube. A content analysis of 365 e-cigarette videos indicates that 85% of the videos were sponsored by marketers. These videos highlight e-cigarettes' economic and social benefits, featuring a low level of fear appeal and negative message valence and a high level of marketing information about e-cigarette products. They also convey certain health claims that have been proscribed by the U.S. Food and Drug Administration, the prevalence of which warrants ongoing monitoring and regulatory guidelines for online e-cigarette marketing.
2061	Mohammed Al-hamdani A short note on e-cigarette issues: harm reduction, re-normalization, and Big Tobacco.. Journal of public health policy; 2014;35:132.<br><br>**Keywords:**<br>Adolescent,Child,*Electronic Nicotine Delivery Systems/ae [Adverse Effects],Electronic Nicotine Delivery Systems/ut [Utilization],Humans,*Tobacco Industry,Tobacco Use/ae [Adverse Effects],Tobacco Use/pc [Prevention & Control]<br><br>**Abstract:**
2070	Leonie Walker Could e-cigarettes perpetuate addiction?.. Nursing New Zealand (Wellington, N.Z. : 1995); 2013;19:32.<br><br>**Keywords:**<br>Humans,Nicotine/ad [Administration & Dosage],*Nicotine/ae [Adverse Effects],Nicotinic Agonists/ad [Administration & Dosage],*Nicotinic Agonists/ae [Adverse Effects],*Substance-Related Disorders/et [Etiology],*Tobacco Products/ae [Adverse Effects]<br><br>**Abstract:**
2110	Lindsay McCauley, Catherine Markin, Danielle Hosmer An unexpected consequence of electronic cigarette use.. Chest; 2012;141:1110.<br><br>**Keywords:**<br>Adult,Electronics,Female,*Glycerol/ae [Adverse Effects],Humans,*Nicotine/ad [Administration & Dosage],Pneumonia, Lipid/ci [Chemically Induced],*Smoking Cessation/mt [Methods]<br><br>**Abstract:**

2135	Anne Buu, Yi-Han Hu, Megan E Piper, Hsien-Chang Lin The association between e-cigarette use characteristics and combustible cigarette consumption and dependence symptoms: Results from a national longitudinal study.. Addictive Behaviors; 2018;84:69.<br><br>**Keywords:**<br>*Drug Dependency,*Product Design,*Test Construction,*Tobacco Smoking,*Electronic Cigarettes, Stimulus Parameters,Symptoms<br><br>**Abstract:** Background: Existing longitudinal surveys focused on the association between ever use of e-cigarettes and combustible cigarette consumption, making it difficult to infer what characteristics of e-cigarette use could potentially change combustible cigarette use behavior, which may have long-term health consequences. Although e-cigarettes' efficacy of alleviating dependence symptoms was supported by studies conducted in laboratory settings, whether the results can be translated into symptom reduction in the real world and over time is an open question. Methods: This study conducted secondary analysis on the Waves 1-2 data of the Population Assessment of Tobacco and Health (PATH) Study to examine the association between e-cigarette use characteristics (frequency, flavoring, and voltage adjustment) and combustible cigarette use outcomes (frequency, quantity, and symptoms), using the Heckman 2-step selection procedure with the selection bias controlled. The inclusion criteria ensured that we followed an adult cohort of exclusive combustible cigarette users at Wave 1. Results: The result shows that higher frequency of e-cigarette use was associated with lower combustible cigarette consumption and dependence symptoms, controlling for the corresponding baseline cigarette use variable and other confounders. Given the frequency of e-cigarette use, the feature of voltage adjustment was not significantly associated with any of the cigarette use outcomes. Flavoring, on the other hand, was associated with lower quantity of cigarette use. Conclusions: Exclusive smokers who start using e-cigarettes do indeed change the frequency and quantity with which they smoke cigarettes. E-cigarette use may also help reduce dependence symptoms. (PsycINFO Database Record (c) 2018 APA, all rights reserved)
2211	Karen G Duderstadt E-cigarettes: Youth and trends in vaping.. Journal of Pediatric Health Care; 2015;29:555.<br><br>**Keywords:**<br>*Advertising,*Health Care Psychology,*Marketing,*Electronic Cigarettes,Tobacco Smoking<br><br>**Abstract:** This article will address the prevalence and use of e-cigarettes and e-cigarette products among youth, evidence regarding the effect of initiation of e-cigarette use and the use of conventional cigarettes, the current regulatory policies on e-cigarettes, and recommendations for policies to protect the health of children and youth regarding e-cigarette use and initiation of smoking tobacco. Public policy on regulation of e-cigarettes and the labeling of e-cigarette devices and products has lagged behind the rapid increase in use, resulting in adverse effects on children and youth. E-cigarettes remain unregulated, and there are no federal age restrictions on sales to minors. The popularity of youth ''vaping'' continues to rise. Developing strategies to prevent marketing, sales, and use of e-cigarettes among youths is critical. The rapid increase in use of e-cigarettes and youths' susceptibility to social and environmental influences to use tobacco will require multiple pronged restrictions on e-cigarette use, sales, and advertisement to protect vulnerable children and adolescents from early initiation of smoking tobacco and its long-term health effects. (PsycINFO Database Record (c) 2017 APA, all rights reserved)
2232	Angeline Leona Sangalang Using stories to communicate electronic cigarette risk to young adults: Explicating similarity in narrative persuasion.. Dissertation Abstracts International Section A: Humanities and Social Sciences; 2017;77:No.<br><br>**Keywords:**<br>*Communication,*Narratives,*Risk Factors,*Electronic Cigarettes

	**Abstract:** College students are a particularly vulnerable population to electronic cigarette (e-cigarette) initiation and use due to social and environmental pressures. The present investigation tests the efficacy of narrative communication to communicate e-cigarette risk. The study tests the impact of social role similarity (i.e., the degree to which audience members align with the social group or role of the target character) on narrative consistent e-cigarette beliefs, attitudes, and intentions, increased narrative processing (i.e., identification, transportation, and narrative engagement), and reduced message resistance. The study additionally compares relative impact of social role similarity to other forms of actual and perceived similarity (i.e., gender, race, and attitude). The results indicate both actual and social role similarity are the strongest predictors of intentions related to e-cigarette information-seeking and all the processes of narrative involvement. The relationship between forms of similarity and e-cigarette beliefs, attitudes, intentions to use e-cigarettes, and reduced counterarguing and reactance were non-significant. (PsycINFO Database Record (c) 2017 APA, all rights reserved)
2233	Maria Cooper, MeLisa R Creamer, Christina Ly, Brittani Crook, Melissa B Harrell, Cheryl L Perry Social norms, perceptions and dual/poly tobacco use among Texas youth.. American Journal of Health Behavior; 2016;40:761.<br><br>**Keywords:**<br>*Health Behavior,*Social Norms,*Tobacco Smoking,Adolescent Characteristics<br><br>**Abstract:** Objectives: We assessed risk perceptions and social norms about tobacco use across adolescent non-users of tobacco, single-product users, and dual/poly-product users. Methods: Use behaviors specific to e-cigarettes, cigarettes, hookah, cigars, and smokeless tobacco were assessed among 6th, 8th and 10th grade students (sample [n] = 3907 from a population of [N] = 461,069 students). Multivariate regression was used to examine differences in these factors across use categories, adjusted for demographic factors. Results: Results highlight differences between non-users and single- or dual/poly-product users for most tobacco products. Dual/poly-product users differed from single-product users most notably in their higher perceived use of tobacco products by close friends and dating partners, and a higher proportion of single-product users reported most products were not harmful and not addictive compared to non-users. Few differences were seen between dual/poly-product users and single-product users in their perceptions of harm and addictiveness. Conclusions: Findings demonstrate the importance of proximal social influences (ie, close friends and dating partners) for adolescent dual/poly- tobacco product users. Understanding similarities and differences in risk factors between these adolescent groups can guide effective public health prevention and treatment programs. (PsycINFO Database Record (c) 2017 APA, all rights reserved)
2234	Sharon Lipperman-Kreda, Joel W Grube, Karen B Friend, Christina Mair Tobacco outlet density, retailer cigarette sales without ID checks and enforcement of underage tobacco laws: Associations with youths' cigarette smoking and beliefs.. Addiction; 2016;111:525.<br><br>**Keywords:**<br>*Law Enforcement,*Marketing,*Tobacco Smoking,*Electronic Cigarettes<br><br>**Abstract:** Aims: To estimate the relationships of tobacco outlet density, cigarette sales without ID checks and local enforcement of underage tobacco laws with youth's life-time cigarette smoking, perceived availability of tobacco and perceived enforcement of underage tobacco laws and changes over time. Design: The study involved: (a) three annual telephone surveys, (b) two annual purchase surveys in 2000 tobacco outlets and (c) interviews with key informants from local law enforcement agencies. Analyses were multi-level models (city, individual, time). Setting: A sample of 50 mid-sized non-contiguous cities in California, USA. Participants: A total of 1478 youths (aged 13-16 at wave 1, 52.2% male); 1061 participated in all waves. Measurements: Measures at the individual level included life-time cigarette smoking, perceived availability and perceived enforcement. City-level measures included tobacco outlet

	density, cigarette sales without ID checks and compliance checks. Findings: Outlet density was associated positively with life-time smoking [OR = 1.12, P < 0.01]. An interaction between outlet density and wave (OR = 0.96, P < 0.05) suggested that higher density was associated more closely with life-time smoking at the earlier waves when respondents were younger. Greater density was associated positively with perceived availability (beta = 0.02, P < 0.05) and negatively with perceived enforcement (beta = -0.02, P < 0.01). Sales rate without checking IDs was related to greater perceived availability (beta = 0.01, P < 0.01) and less perceived enforcement (beta = -0.01, P < 0.01). Enforcement of underage tobacco laws was related positively to perceived enforcement (beta = 0.06, P < 0.05). Conclusions: Higher tobacco outlet density may contribute to life-time smoking among youths. Density, sales without ID checks and enforcement levels may influence beliefs about access to cigarettes and enforcement of underage tobacco sales laws. (PsycINFO Database Record (c) 2017 APA, all rights reserved)
2235	Mirte A. G Kuipers, Stephanie D Brandhof, Karin Monshouwer, Karien Stronks, Anton E Kunst Impact of laws restricting the sale of tobacco to minors on adolescent smoking and perceived obtainability of cigarettes: An intervention-control pre-post study of 19 European Union Countries.. Addiction; 2017;112:320.<br><br>**Keywords:**<br>*Adolescent Development,*Laws,*Tobacco Smoking,Nicotine,Socioeconomic Status,Electronic Cigarettes<br><br>**Abstract:** Aims: To estimate the impact of introducing sales restriction laws in Europe by measuring changes in adolescent smoking prevalence and perceived obtainability of cigarettes over time in countries that have recently introduced sales restriction laws (intervention countries). These countries were compared with countries where such laws were already in force (control countries). The secondary aim was to test whether these trends differed between adolescents of higher and lower socio-economic position (SEP). Design: Repeated cross-sectional data were used in a quasi-experimental pre-post design. Intervention countries raised tobacco sales restrictions to 18 years in 2007, 2008 or 2009. Control countries have had sales restrictions of 18 years since at least 2004. Setting: Nineteen European Union (EU) countries in 2007 and 2011. Participants: Data of 97 245 15- and 16-year-old adolescents from the 2007 and 2011 European Survey Project on Alcohol and other Drugs (ESPAD) surveys. Measurements: Dependent variables were smoking status (weekly smoking versus non-smoking or less than weekly smoking) and perceived obtainability of tobacco (easy versus difficult). Key independent variables were time (2007 or 2011), intervention exposure (intervention or control country) and their interaction (time x intervention). Covariates included sex, SEP, the tobacco control scale (TCS), gross domestic product (GDP) and adult smoking prevalence. Findings: We did not find a difference in the change in smoking prevalence between intervention and control countries [odds ratio (OR) = 1.00, 95% confidence interval (CI) = 0.93-1.07]. However, sales restrictions were associated with a significantly greater decrease in perceived ease of cigarette obtainability in intervention countries (OR = 0.75, 95% CI = 0.70-0.80). No significant differential effects between high and low SEP adolescents were found (OR = 1.01, 95% CI = 0.81-1.25). Conclusion: Laws prohibiting the sales of tobacco to minors in Europe do not appear to be associated with a reduction in adolescent smoking rates, but lower the perceived obtainability of cigarettes irrespective of their socio-economic position. (PsycINFO Database Record (c) 2017 APA, all rights reserved)
2239	Adam M Leventhal, Matthew D Stone, Nafeesa Andrabi, Jessica Barrington-Trimis, David R Strong, Steve Sussman, Janet Audrain-McGovern Association of e-cigarette vaping and progression to heavier patterns of cigarette smoking.. JAMA: Journal of the American Medical Association; 2016;316:1918.<br><br>**Keywords:**<br>*Smoking Cessation,*Tobacco Smoking,*Electronic Cigarettes<br><br>**Abstract:** E-cigarette vaping is reported by 37% of US 10th-grade adolescents1 and is associated with subsequent initiation of combustible cigarette smoking. Whether individuals who vape and transition to

	combustible cigarettes are experimenting or progress to more frequent and heavy smoking is unknown. In addition, because some adolescents use e-cigarettes as a smoking cessation aid, adolescent smokers who vape could be more likely to reduce their smoking levels over time. Therefore, associations of vaping with subsequent smoking frequency and heaviness pattern among adolescents were examined. (PsycINFO Database Record (c) 2016 APA, all rights reserved)
2240	Donald S Kenkel Healthy innovation: Vaping, smoking, and public policy.. Journal of Policy Analysis and Management; 2016;35:473.<br><br>**Keywords:**<br>*Innovation,*Policy Making,*Public Health,*Smoking Cessation<br><br>**Abstract:** Vaping is a revolutionary product innovation because it does not involve the combustion of tobacco. Instead, vapers inhale a heated nicotine mist. Scientific evidence shows that vaping is much safer than smoking combustible cigarettes and might also aid smoking cessation. At the same time, there are serious public health concerns: for adolescents, vaping might be a gateway to smoking combustible cigarettes; for established smokers, dual use of vaping and smoking might prevent complete cessation. (PsycINFO Database Record (c) 2016 APA, all rights reserved)
2260	Blair N Coleman The association between electronic cigarette use and cigarette smoking behavior among young adults in the United States.. Dissertation Abstracts International: Section B: The Sciences and Engineering; 2016;76:No.<br><br>**Keywords:**<br>*Public Health,*Smoking Cessation,*Tobacco Smoking,*Electronic Cigarettes<br><br>**Abstract:** The prevalence of electronic cigarette (e-cigarette) use is rapidly increasing in adults and youth; however, little is known about the public health impact of their use. A debate over e-cigarettes has emerged in the literature; one side recognizes the potential benefit of e-cigarettes as a harm reduction tool, while others argue e-cigarette use may delay or deter smoking cessation due to dual use or increase the risk of initiation of conventional cigarettes among previous nonsmokers. Drawing on the Theory of Planned Behavior, this dissertation focused on attitudes, beliefs, and perceived social norms of e-cigarettes, as well as openness to conventional cigarette smoking among young adult users of the product. Using a mixed methods approach, this dissertation analyzed secondary data from the 2012-2013 National Adult Tobacco Survey (NATS) as well as focus group data collected in five cities across the U.S. to better understand the relationship between e-cigarette use and cigarette smoking among young adults. In Study 1, quantitative analyses found non-cigarette smoking young adults who have tried e-cigarettes were more likely to report openness to cigarette smoking in the future compared to those who have not tried e-cigarettes (AOR= 2.4; 95% CI= 1.7-3.3). In Study 2, qualitative findings suggest that young adult exclusive e-cigarette users were less interested in conventional cigarette smoking, and overwhelmingly described negative aspects to cigarette smoking that appeared to become more salient as a result of their e-cigarette use. In Study 3, focus group participants expressed many positive attitudes towards e-cigarettes, and simultaneously reported a lack of information and knowledge about the products. The relationship between e-cigarette use and cigarette smoking is complex and multifaceted, and influenced by a myriad of individual and social factors. Although quantitative findings suggest young adults who have used e-cigarettes compared to those who have not used e-cigarettes were more likely to report openness to future cigarette smoking, qualitative findings did not support the notion that young adult e-cigarette users (who may have prior experience with cigarette smoking) are open to future cigarette smoking. These findings provide a basis for further exploration of the association between e-cigarette use and cigarette smoking. (PsycINFO Database Record (c) 2016 APA, all rights reserved)
2286	Nancy A Rigotti E-cigarette use and subsequent tobacco use by adolescents: New evidence about a potential risk of e-cigarettes.. JAMA: Journal of the American Medical Association; 2015;314:673.

	**Keywords:**<br>*Adolescent Development,*Nicotine,*Smokeless Tobacco,*Tobacco Smoking,*Chemical Exposure<br><br>**Abstract:** Comments on an article by Adam M. Leventhal et al. (see record 2015-40648-003). Leventhal et al. used a repeated-measures prospective observational study design to assess combustible tobacco and e-cigarette use among a diverse cohort of 2530 ninth graders attending 10 public high schools in Los Angeles, California, from 2013-2014. Non-smokers with previous exposure to e-cigarettes at initial interview compared with those without exposure toe-cigarettes were more likely to report use of acombustible tobacco product during the next 6 months and 12 months. Significant associations also were found for each individual tobacco product, including cigarettes, cigars, and hookah. The investigators carefully adjusted the analysis for many known sociodemographic, family, environmental, and intrapersonal factors associated with e-cigarette and combustible tobacco use. The study by Leventhal et al. is the first prospective report associating adolescents' use of e-cigarettes with subsequent uptake of combustible tobacco products. Regardless of whether e-cigarettes are a gateway to tobacco product initiation, there is no reason for adolescents to use a product for which the hypothesized public health benefit is harm reduction for adult smokers. (PsycINFO Database Record (c) 2016 APA, all rights reserved)
2289	Ellen M Meier, Alayna P Tackett, Mary B Miller, DeMond M Grant, Theodore L Wagener "Which nicotine products are gateways to regular use? First-tried tobacco and current use in college students": Correction.. American Journal of Preventive Medicine; 2015;49:332.<br><br>**Keywords:**<br>*College Students,*Nicotine,*Smokeless Tobacco,*Tobacco Smoking<br><br>**Abstract:** Reports an error in "Which nicotine products are gateways to regular use? First-tried tobacco and current use in college students" by Ellen M. Meier, Alayna P. Tackett, Mary B. Miller, DeMond M. Grant and Theodore L. Wagener (American Journal of Preventive Medicine, 2015[Jan], Vol 48[1, Suppl 1], S86-S93). In the original article, there are some errors in Table 2. The corrected Table 2 is given in the erratum. (The following abstract of the original article appeared in record 2014-57470-018). Background: The potential for emerging tobacco products (ETPs) to be gateway products for further tobacco use among youth is of significant concern. Purpose: To examine use of various nicotine-containing products on a tobacco-free college campus and whether the first product tried predicts subsequent tobacco use. Methods: Undergraduate students (N = 1,304) at a large university completed an online survey of past/current use of cigarettes; smokeless tobacco (SLT); hookah; ETPs (dissolvables, snus, and electronic cigarettes); and nicotine replacement therapy (NRT). Data were collected from September 2012 to May 2013 and analyses were conducted from June to September 2013. Students were classified as single, dual, or poly tobacco users. Results: The sample consisted of 79.5% non-users, 13.8% single, 4.4% dual, and 1.5% poly users. Overall, 49.4% of participants reported trying a tobacco product. Hookah was the most tried product (38%), but cigarettes were most often the first product ever tried (51%). First product tried did not predict current tobacco use and non-use, but individuals who first tried SLT or cigarettes (rather than hookah or ETPs) were more likely to be poly tobacco users. Current tobacco users who first tried ETPs or hookah were largely non-daily users of hookah; current tobacco users who first tried cigarettes or SLT were largely non-daily or daily users of cigarettes/SLT. Conclusions: Hookah and ETPs are increasingly becoming the first tobacco product ever tried by youth; however, uptake of ETPs is poor, unlike cigarettes and SLT, and does not appear to lead to significant daily/non-daily use of cigarettes and SLT. (PsycINFO Database Record (c) 2016 APA, all rights reserved)
2339	Rachel A Grana Electronic cigarettes: A new nicotine gateway?. Journal of Adolescent Health; 2013;52:135.<br><br>**Keywords:**

	*Adolescent Attitudes,*At Risk Populations,*Awareness,*Tobacco Smoking,Human Males,Electronic Cigarettes<br><br>**Abstract:** Comments on an article by J. K. Pepper et al. (see record 2013-02132-004). Pepper et al. present the first study of a national sample of U.S. male adolescents' awareness, use, and susceptibility to using e-cigarettes. The 228 participants were sons of parents recruited to participate in an online panel. Pepper et al found high awareness of e-cigarettes-67%-but little use; < 1% had tried them. Of even greater importance, 18% of the sample endorsed being susceptible to trying e-cigarettes, and this was much higher (74%) among participants who reported current smoking. To date, only two studies of national data sets have provided a glimpse into awareness and use of e-cigarettes, and both were among adults. The high rate of awareness among youth is concerning, particularly because the literature raises concerns about e-cigarette products with regard to ingredient and device safety. Given the dearth of empirical research on e-cigarette products and who may be using them, the Pepper et al article makes an important contribution toward understanding how youth are responding to their availability. In their multivariate analysis, being a current smoker was strongly associated with willingness to try e-cigarettes. When the analysis was limited to nonsmoking participants, holding negative beliefs about the typical smoker was associated with less willingness to try e-cigarettes. These findings suggest that e-cigarettes may be perceived by adolescents as symbolically and functionally similar to tobacco cigarettes. Although these findings are limited by a male-only sample, known predictors of initiation of cigarette smoking, such as sensation seeking and positive smoking expectancies, may be relevant factors to examine in future research investigating e-cigarette use among adolescents. (PsycINFO Database Record (c) 2017 APA, all rights reserved)
2355	M Conner, S Grogan, R Simms-Ellis, K Flett, B Sykes-Muskett, L Cowap, R Lawton, CJ Armitage, D Meads, C Torgerson, R West, K Siddiqi Do electronic cigarettes increase cigarette smoking in UK adolescents? Evidence from a 12-month prospective study.. Tobacco control; 2017;:<br><br>**Keywords:**<br><br>**Abstract:** BACKGROUND: In cross-sectional surveys, increasing numbers of adolescents report using both electronic cigarettes (e-cigarettes) and cigarettes. This study assessed whether adolescent e-cigarette use was associated prospectively with initiation or escalation of cigarette use. METHODS: Data were from 2836 adolescents (aged 13-14 years at baseline) in 20 schools in England. At baseline, breath carbon monoxide levels, self-reported e-cigarette and cigarette use, sex, age, friends and family smoking, beliefs about cigarette use and percentage receiving free school meals (measure of socioeconomic status) were assessed. At 12-month follow-up, self-reported cigarette use was assessed and validated by breath carbon monoxide levels. RESULTS: At baseline, 34.2% of adolescents reported ever using e-cigarettes (16.0% used only e-cigarettes). Baseline ever use of e-cigarettes was strongly associated with subsequent initiation (n=1726; OR 5.38, 95% CI 4.02 to 7.22; controlling for covariates, OR 4.06, 95% CI 2.94 to 5.60) and escalation (n=318; OR 1.91, 95% CI 1.14 to 3.21; controlling for covariates, this effect became non-significant, OR 1.39, 95% CI 0.97 to 1.82) of cigarette use. CONCLUSIONS: This is the first study to report prospective relationships between ever use of e-cigarettes and initiation and escalation of cigarette use among UK adolescents. Ever use of e-cigarettes was robustly associated with initiation but more modestly related to escalation of cigarette use. Further research with longer follow-up in a broader age range of adolescents is required.
2356	C Best, F Haseen, D Currie, G Ozakinci, AM MacKintosh, M Stead, D Eadie, A MacGregor, J Pearce, A Amos, J Frank, S Haw Relationship between trying an electronic cigarette and subsequent cigarette experimentation in Scottish adolescents: a cohort study.. Tobacco control; 2017;:<br><br>**Keywords:**<br><br>**Abstract:** BACKGROUND: This study examines whether young never smokers in Scotland, UK, who have

	tried an e-cigarette are more likely than those who have not, to try a cigarette during the following year. METHODS: Prospective cohort survey conducted in four high schools in Scotland, UK during February/March 2015 (n=3807) with follow-up 1 year later. All pupils (age 11-18) were surveyed. Response rates were high in both years (87% in 2015) and 2680/3807 (70.4%) of the original cohort completed the follow-up survey. Analysis was restricted to baseline 'never smokers' (n=3001/3807), 2125 of whom were available to follow-up (70.8%). RESULTS: At baseline, 183 of 2125 (8.6%) never smokers had tried an e-cigarette and 1942 had not. Of the young people who had not tried an e-cigarette at baseline, 249 (12.8%) went on to try smoking a cigarette by follow-up. This compares with 74 (40.4%) of those who had tried an e-cigarette at baseline. This effect remained significant in a logistic regression model adjusted for smoking susceptibility, having friends who smoke, family members' smoking status, age, sex, family affluence score, ethnic group and school (adjusted OR 2.42 (95% CI 1.63 to 3.60)). There was a significant interaction between e-cigarette use and smoking susceptibility and between e-cigarette use and smoking within the friendship group. CONCLUSIONS: Young never smokers are more likely to experiment with cigarettes if they have tried an e-cigarette. Causality cannot be inferred, but continued close monitoring of e-cigarette use in young people is warranted.
2357	A Glasser, H Abudayyeh, J Cantrell, R Niaura Patterns of E-Cigarette Use Among Youth and Young Adults: Review of the Impact of E-Cigarettes on Cigarette Smoking.. Nicotine & tobacco research : official journal of the Society for Research on Nicotine and Tobacco; 2018;:<br><br>**Keywords:**<br><br>**Abstract:** Implications: The present article provides a review of the impact of e-cigarette use on subsequent cigarette smoking among youth and YAs. Studies presented here suggest that e-cigarette use among non-smokers is associated with subsequent cigarette smoking, but study designs are subject to numerous limitations. Future research should focus on addressing the characteristics that put youth and YAs at risk of using either product and how appeal and accessibility of these products are related to product use in order to inform future policy-making.
2358	R Miech, ME Patrick, PM O'Malley, LD Johnston E-cigarette use as a predictor of cigarette smoking: results from a 1-year follow-up of a national sample of 12th grade students.. Tobacco control; 2017;26:<br><br>**Keywords:**<br><br>**Abstract:** OBJECTIVE: To prospectively examine vaping as a predictor of future cigarette smoking among youth with and without previous cigarette smoking experience. A secondary aim is to investigate whether vaping may desensitise youth to the dangers of smoking. METHODS: Analysis of prospective longitudinal panel data from the nationally representative Monitoring the Future study. The analysis is based on 347 12th grade students who were part of a randomly selected subsample that completed in-school surveys in 2014 and were resurveyed 1-year later. RESULTS: Among youth who had never smoked a cigarette by 12th grade, baseline, recent vapers were more than 4 times (relative risk (RR)=4.78) more likely to report past-year cigarette smoking at follow-up, even among youth who reported the highest possible level of perceived risk for cigarette smoking at baseline. Among 12th grade students who had smoked in the past but had not recently smoked at baseline, recent vapers were twice (RR=2.15) as likely to report smoking in the past 12 months at the follow-up. Vaping did not predict cessation of smoking among recent smokers at baseline. Among never-smokers at baseline, recent vapers were more than 4 times (RR=4.73) more likely to move away from the perception of cigarettes as posing a 'great risk' of harm, a finding consistent with a desensitisation process. CONCLUSIONS: These results contribute to the growing body of evidence supporting vaping as a one-way bridge to cigarette smoking among youth. Vaping as a risk factor for future smoking is a strong, scientifically-based rationale for restricting youth access to e-cigarettes.

2367	Carey F.R.,Wilkinson A.V.,Harrell M.B.,Cohn E.A.,Perry C.L. Measurement and predictive value of susceptibility to cigarettes, e-cigarettes, cigars, and hookah among Texas adolescents. Addictive Behaviors Reports; 2018;8:95.<br><br>**Keywords:**<br>adolescent,article,*cigarette,confirmatory factor analysis,*electronic cigarette,ethnic group,female, futurology,high school student,*hookah,human,logistic regression analysis,male,measurement,middle school student,predictive value,priority journal,program appropriateness,smoking,Texas,validation study, *cigar<br><br>**Abstract:** Susceptibility to cigarette smoking, defined as the lack of a firm commitment not to smoke in the future, begins in childhood and is a phase in the transition from never to ever use of cigarettes. While a consistent and validated predictor of cigarette use, little research has assessed whether the susceptibility construct applies equally well across other tobacco products. Baseline data were collected in 2014-2015 from a representative sample of (n = 2844) middle and high school students in five counties surrounding the four largest cities in Texas, (49% female and mean age 13.13 years, with subsequent waves at 6, 12, and 18 months. Confirmatory factor analysis examined the appropriateness of a three-item susceptibility measure (product-specific curiosity, intention to use, and peer influence) across product types and ethnic groups (Hispanic versus non-Hispanic). Logistic regression examined whether product specific susceptibility at baseline predicted future product initiation. At baseline, 11.5%, 17.0%, 17.4% and 29.4%, of adolescent never users were susceptible to cigars, cigarettes, hookah and e-cigarettes, respectively; significantly more Hispanic than non-Hispanic adolescents were susceptible to e-cigarettes (32.4% versus 26%, $p < 0.01$) and cigarettes (19.9% versus 13.9%, $p < 0.05$). Product-specific items were significantly and consistently associated with the respective underlying susceptibility product construct and across ethnic groups ($p < 0.001$ for all). Susceptibility to e-cigarettes (AOR = 2.28-6.64) or any combustible product (cigarettes, hookah, cigars; AOR = 3.38-5.20) significantly predicted subsequent ever use. This study confirms the appropriateness of the susceptibility construct across four tobacco product types and ethnic groups, and the utility of susceptibility in predicting future product use among adolescents.Copyright © 2018
2369	Tam J.,Warner K.E. Students' Cigarette Smoking and the Perceived Nicotine Content of Their E-cigarettes. American Journal of Preventive Medicine; 2018;55:376.<br><br>**Keywords:**<br>adolescent,article,awareness,cannabis smoking,*cigarette smoking,controlled study,*electronic cigarette,exposure,flavor,health status,human,prevalence,self report,smoking habit,*student attitude, tobacco use,vaping,*nicotine<br><br>**Abstract:** Introduction: There is concern that youth e-cigarette use may serve as a gateway to cigarette smoking, and that nicotine exposure may harm brain development. It is therefore important to know how much nonsmoking youth perceive being exposed to nicotine through e-cigarettes. Methods: Data on smoking and vaping from the 2016 Monitoring the Future survey of eighth, tenth, and 12th grade students were analyzed in 2017. The weighted percentage distributions of self-reported e-cigarette mist last inhaled were calculated according to ever-smoking status and past-30-day smoking frequency. Chi-square tests of the association between smoking status and use of e-cigarettes perceived to contain nicotine, marijuana or hash oil, or just flavoring were performed. Never smokers and regular smokers were compared regarding the type of e-cigarette mist they believe they last used. Results: A significant relationship exists between smoking behavior and reportedly vaping nicotine ($p<0.01$) or just flavors ($p<0.01$). With increasing smoking intensity, an increasing proportion of students report they are vaping nicotine; a decreasing proportion report just flavors. Among 12th graders, prevalence of vaping nicotine is lowest among never smokers and non-current smokers (14.3% and 18.1%) and highest among current and frequent smokers (61.3% and 60.9%, $p<0.01$). Substantially larger proportions of never smokers and

	30-day nonsmokers report vaping just flavors (76.0% and 69.6%) compared with regular or frequent smokers (31.0% and 29.3%, p<0.01). Conclusions: Most nonsmoking students perceive limited nicotine exposure from vaping. Future research should evaluate the accuracy of self-reported e-cigarette nicotine content and monitor students who are consciously using nicotine-based e-cigarettes.Copyright © 2018 American Journal of Preventive Medicine
2371	Watkins S.L.,Glantz S.A.,Chaffee B.W. In reply. JAMA Pediatrics; 2018;172:784.<br><br>**Keywords:**<br>adolescent smoking,age,ambient air,cigarette smoking,electronic cigarette,environmental policy,high school student,human,letter,mass medium,middle school student,prevalence,priority journal,public health campaign,purchasing,risk factor,smoking cessation,tax,*tobacco<br><br>**Abstract:**
2372	Ip M.,Middlekauff H.R. Noncigarette tobacco products-gateway or diversion?. JAMA Pediatrics; 2018;172:784.<br><br>**Keywords:**<br>adolescent smoking,cigarette smoking,electronic cigarette,high school student,human,letter,market, priority journal,risk,smokeless tobacco,*tobacco<br><br>**Abstract:**
2383	Newton J.N.,Dockrell M.,Marczylo T. Electronic cigarettes: adolescent health and wellbeing - Authors' reply. The Lancet; 2018;392:474.<br><br>**Keywords:**<br>*adolescent health,adolescent smoking,Canada,cigarette smoking,consensus,*electronic cigarette, human,letter,policy,prevalence,priority journal,public health,smoking cessation,United Kingdom,United States,*wellbeing<br><br>**Abstract:**
2388	Anonymous. Annual regional meeting on implementation of the WHO framework convention on tobacco control. Eastern Mediterranean Health Journal; 2018;24:497.<br><br>**Keywords:**<br>advertising,awareness,decision making,electronic cigarette,health care planning,health promotion, human,information dissemination,law,mass medium,non communicable disease,note,prevalence, prevention and control,*smoking ban,*tobacco use,waterpipe tobacco,*world health organization<br><br>**Abstract:**
2414	Michael Cummings K.,Morris P.B.,Benowitz N.L. Another article about e-cigarettes: Why should I care?. Journal of the American Heart Association; 2018;7:e009944.<br><br>**Keywords:**<br>China,cigarette smoking,clinical practice,college student,editorial,*electronic cigarette,evidence based medicine,food and drug administration,health care survey,health hazard,high school student,human, patient safety,practice guideline,prevalence,priority journal,public health,publication,smoking,smoking cessation,United States<br><br>**Abstract:**

2426	Britton J. Denicotinised cigarettes. The Lancet; 2018;392:104.<br><br>**Keywords:**<br>*cigarette,electronic cigarette,food and drug administration,health promotion,human,note,priority journal,public health,risk management,smoking cessation program,smoking regulation,tobacco dependence,nicotine,*denicotinised cigarette<br><br>**Abstract:**
2436	Kamerow D. Start-up e-cigarette brand aims to "improve smokers' lives". BMJ (Online); 2018;362:k2930.<br><br>**Keywords:**<br>algorithm,*cigarette smoking,*electronic cigarette,government regulation,growth rate,high school student,human,juvenile,note,prevalence,priority journal,tobacco dependence,United States,vaping<br><br>**Abstract:**
2438	Helen G.S.,Eaton D.L. Public health consequences of e-cigarette use. JAMA Internal Medicine; 2018;178:984.<br><br>**Keywords:**<br>aerosol,clinical practice,coughing,*electronic cigarette,endothelial dysfunction,food and drug administration,heart rate,human,priority journal,*public health problem,review,smoking,smoking cessation,wheezing,carcinogen,nicotine,toxic substance<br><br>**Abstract:**
2441	Levy D.T.,Borland R.,Fong G.T.,Villanti A.C.,Niaura R.,Meza R.,Holford T.R.,Michael Cummings K.,Abrams D.B. Developing consistent and transparent models of E-cigarette use: Reply to Glantz and Soneji et al. Nicotine and Tobacco Research; 2017;19:268.<br><br>**Keywords:**<br>*biological model,conceptual framework,decision making,groups by age,health hazard,human,letter, prevalence,priority journal,sensitivity analysis,smoking,smoking cessation,tobacco industry,*vaping, biological marker,electronic cigarette<br><br>**Abstract:**
2442	Soneji S.,Sung H.-Y.,Primack B.,Pierce J.P.,Sargent J. Problematic assessment of the impact of vaporized nicotine product initiation in the United States. Nicotine and Tobacco Research; 2017;19:264.<br><br>**Keywords:**<br>cardiovascular function,comparative study,epithelium cell,evidence based practice,harm reduction, *health impact assessment,health survey,heart disease,human,immune response,inflammation,letter, measurement accuracy,nose epithelium,prediction,priority journal,public health,sensitivity analysis, smoking,United States,aldehyde,alpha tocopherol/ec [Endogenous Compound],cigarette smoke, formaldehyde,*electronic cigarette<br><br>**Abstract:**
2453	Black M.M.,Nair P.,Spanier A.J. Dose and timing of prenatal tobacco exposure: Threats to early child development. The Lancet Respiratory Medicine; 2014;2:677.<br><br>**Keywords:**

	attention deficit disorder,brain development,*child development,conduct disorder,correlation analysis, disease association,fetal well being,gestational age,health promotion,human,low birth weight,maternal smoking,medical device,nerve cell differentiation,neurotransmission,nicotine replacement therapy,note, *prenatal exposure,risk assessment,risk reduction,signal transduction,smoking cessation,*tobacco, electronic cigarette,nicotine,nicotinic receptor/ec [Endogenous Compound],nicotine containing vapour device<br><br>**Abstract:**
2455	Nowak D. In reply. Deutsches Arzteblatt International; 2014;111:682.<br><br>**Keywords:**<br>cancer susceptibility,carcinogenesis,*carcinogenic activity,chronic obstructive lung disease,human,letter, lung cancer,nicotine replacement therapy,smoking,tumor growth,carcinogen,cigarette smoke,*electronic cigarette,*nicotine,nicotinic receptor<br><br>**Abstract:**
2459	Cheng T.O. Smoking in China: Can or should China kick the habit?. International Journal of Cardiology; 2014;175:219.<br><br>**Keywords:**<br>adolescent smoking,advertizing,air pollution,China,editorial,health hazard,human,life expectancy,passive smoking,priority journal,profit,*smoking,smoking ban,smoking cessation,smoking regulation,tobacco industry,electronic cigarette<br><br>**Abstract:**
2460	Delnevo C.D.,Manderski M.T.B.,Giovino G.A. To the editor. JAMA Pediatrics; 2014;168:775.<br><br>**Keywords:**<br>*adolescent smoking,high school student,human,letter,prevalence,priority journal,smokeless tobacco, tobacco,*tobacco use,*electronic cigarette<br><br>**Abstract:**
2461	Godlee F.,Malone R.,Timmis A.,Otto C.,Bush A.,Pavord I.,Groves T. Journal policy on research funded by the tobacco industry. Heart; 2014;100:2.<br><br>**Keywords:**<br>advertizing,editorial,funding,health promotion,human,lung cancer,*medical literature,medical research, *policy,priority journal,public health,publication,scientific misconduct,smoking,smoking ban,*tobacco industry,electronic cigarette<br><br>**Abstract:**
2462	Aveyard P.,Lindson-Hawley N.,Hastings G.,De Andrade M. Should smokers be advised to cut down as well as quit?. BMJ (Online); 2014;348:A1593.<br><br>**Keywords:**<br>article,Australia,behavior change,government regulation,health behavior,health care policy,health promotion,human,law,marketing,motivation,nicotine replacement therapy,outcome assessment,priority journal,risk reduction,*smoking,*smoking cessation,smoking cessation program,smoking regulation, tobacco dependence/th [Therapy],tobacco industry,United Kingdom,electronic cigarette,*smoking

	reduction<br><br>**Abstract:**
2463	Prochaska J.J.,Brown-Johnson C.G. Encouraging and supporting smoking cessation in the workforce. Occupational and Environmental Medicine; 2014;71:385.<br><br>**Keywords:**<br>health survey,human,note,passive smoking,prevalence,priority journal,reward,*smoking cessation, tobacco,United States,work environment,*worker,workplace,electronic cigarette,nicotine<br><br>**Abstract:**
2464	Sutherland G. Smoking cessation and mental health. Neuropsychiatry; 2014;4:11.<br><br>**Keywords:**<br>adverse outcome,cognitive therapy,drug efficacy,human,mental disease,*mental health,mood disorder, nicotine replacement therapy,note,prevalence,priority journal,randomized controlled trial (topic), smoking,*smoking cessation,social support,tobacco dependence/dt [Drug Therapy],treatment response, amfebutamone/dt [Drug Therapy],electronic cigarette,insulin,placebo,varenicline/dt [Drug Therapy], warfarin<br><br>**Abstract:**
2467	Turner S. Modifying parental smoking habits - How things have changed since 2010?. Paediatrics and Child Health (United Kingdom); 2014;24:472.<br><br>**Keywords:**<br>advertizing,anamnesis,asthma,child health,disease exacerbation,environmental exposure,government, health care personnel,health education,health personnel attitude,home environment,human,law, motivation,motivational interviewing,note,nursing staff,*parental smoking,passive smoking,prevalence, protection,smoking ban,smoking cessation,*smoking habit,social norm,electronic cigarette<br><br>**Abstract:**
2469	Prochaska J.J. Quitting smoking is associated with long term improvements in mood. BMJ (Online); 2014;348:g1562.<br><br>**Keywords:**<br>anxiety,brain function,disease association,disease predisposition,drug surveillance program,drug use, editorial,exercise,follow up,human,intermethod comparison,long term exposure,major depression, mental disease,*mental health,meta analysis (topic),*mood change,*mood disorder/si [Side Effect],mood disorder/si [Side Effect],neurotransmitter release,nicotine replacement therapy,observational study, posttraumatic stress disorder,priority journal,quality of life,randomized controlled trial (topic),recurrent disease,risk assessment,schizophrenia,*smoking cessation,social support,stress,systematic review (topic), withdrawal syndrome,amfebutamone/ae [Adverse Drug Reaction],amfebutamone/ct [Clinical Trial], electronic cigarette,nicotine,varenicline/ae [Adverse Drug Reaction],varenicline/ct [Clinical Trial]<br><br>**Abstract:**
2470	Zwar N.A.,Mendelsohn C.P.,Richmond R.L. Supporting smoking cessation. BMJ (Online); 2014;348:f7535.<br><br>**Keywords:**<br>adolescent smoking,article,cognitive therapy,counseling,drug metabolism,drug safety,drug tolerability,

	drug withdrawal,early childhood intervention,evidence based medicine,health hazard,human,mental disease,mindfulness,nausea/si [Side Effect],nicotine replacement therapy,patient assessment,physical activity,physician attitude,pregnant woman,prevalence,priority journal,relapse,*smoking cessation, smoking habit,tobacco dependence/dt [Drug Therapy],treatment planning,withdrawal syndrome/dt [Drug Therapy],amfebutamone/dt [Drug Therapy],cytisine/dt [Drug Therapy],electronic cigarette,nicotine patch/dt [Drug Therapy],nortriptyline/dt [Drug Therapy],placebo,varenicline/ae [Adverse Drug Reaction], varenicline/dt [Drug Therapy]<br><br>**Abstract:**
2472	O'Moore E.,Davies K.,Mulholland I. A partnership approach to implementing smoke-free prisons. BMJ (Online); 2014;349:g5443.<br><br>**Keywords:**<br>electronic cigarette,harm reduction,health care access,*health care policy,health promotion,human, letter,nicotine replacement therapy,priority journal,*prison,smoking cessation,*smoking cessation program<br><br>**Abstract:**
2479	Nan Jiang, Sai Yin Ho, Tai Hing Lam Electronic cigarette marketing tactics in mainland China.. Tobacco control; 2017;26:230.<br><br>**Keywords:**<br>China,*Electronic Nicotine Delivery Systems/ec [Economics],Humans,*Marketing/mt [Methods], Marketing/og [Organization & Administration]<br><br>**Abstract:**
2480	Ruth E Malone Conflicts and controversies in contemporary tobacco control.. Tobacco control; 2017;26:e1.<br><br>**Keywords:**<br>*Dissent and Disputes,Electronic Nicotine Delivery Systems,Humans,Nicotine,*Public Policy,Smoke-Free Policy,*Smoking Prevention,Tobacco,*Tobacco Industry/lj [Legislation & Jurisprudence],Tobacco Products,*Tobacco Smoking/lj [Legislation & Jurisprudence],*Vaping/lj [Legislation & Jurisprudence]<br><br>**Abstract:**
2499	Richard Miech, Megan E Patrick, Patrick M O'Malley, Lloyd D Johnston E-cigarette use as a predictor of cigarette smoking: results from a 1-year follow-up of a national sample of 12th grade students.. Tobacco control; 2017;26:e106.<br><br>**Keywords:**<br>Adolescent,*Cigarette Smoking/ep [Epidemiology],Electronic Nicotine Delivery Systems/sn [Statistics & Numerical Data],Female,Follow-Up Studies,Humans,Longitudinal Studies,Male,Prospective Studies,Risk Factors,*Smoking Cessation/sn [Statistics & Numerical Data],Students/sn [Statistics & Numerical Data], Surveys and Questionnaires,*Vaping/sn [Statistics & Numerical Data]<br><br>**Abstract:** OBJECTIVE: To prospectively examine vaping as a predictor of future cigarette smoking among youth with and without previous cigarette smoking experience. A secondary aim is to investigate whether vaping may desensitise youth to the dangers of smoking., METHODS: Analysis of prospective longitudinal panel data from the nationally representative Monitoring the Future study. The analysis is based on 347 12th grade students who were part of a randomly selected subsample that completed in-school surveys

	in 2014 and were resurveyed 1-year later., RESULTS: Among youth who had never smoked a cigarette by 12th grade, baseline, recent vapers were more than 4 times (relative risk (RR)=4.78) more likely to report past-year cigarette smoking at follow-up, even among youth who reported the highest possible level of perceived risk for cigarette smoking at baseline. Among 12th grade students who had smoked in the past but had not recently smoked at baseline, recent vapers were twice (RR=2.15) as likely to report smoking in the past 12 months at the follow-up. Vaping did not predict cessation of smoking among recent smokers at baseline. Among never-smokers at baseline, recent vapers were more than 4 times (RR=4.73) more likely to move away from the perception of cigarettes as posing a 'great risk' of harm, a finding consistent with a desensitisation process., CONCLUSIONS: These results contribute to the growing body of evidence supporting vaping as a one-way bridge to cigarette smoking among youth. Vaping as a risk factor for future smoking is a strong, scientifically-based rationale for restricting youth access to e-cigarettes.Copyright Published by the BMJ Publishing Group Limited. For permission to use (where not already granted under a licence) please go to http://www.bmj.com/company/products-services/rights-and-licensing/.
2531	(2018///) 2018 ASAM Annual Poster Presenter Abstracts. Journal of Addiction Medicine; 12<br><br>**Keywords:**<br>abstinence,adolescent,Canada,cannabis addiction,clinician,Colorado,controlled study,drug overdose,drug therapy,electronic cigarette,emergency ward,endocarditis,epidemic,health care system,hospitalization, human,infection,injection,mental health,Pennsylva<br><br>**Abstract:** The proceedings contain 60 papers. The topics discussed include: addressing the opioid crisis: assessment of clinicians in a large healthcare system; an opportunity to intervene? the opioid epidemic and the Pennsylvania warm hand-off; assessing risk of cannabis dependence among adolescent dual marijuana and e-cigarette users; are long hospital stays needed for opioid use disorder and injection-related infections?; barriers that limit emergency department staff from distributing take-home naloxone kits; association between marijuana, mental health, and tobacco in Colorado; baseline characteristics predictive of transition onto extended-release naltrexone; caring for patients with opioid use disorder and endocarditis: a qualitative study; ceasing to require stimulant abstinence increases engagement in buprenorphine treatment; changes in polysubstance use among patients on methadone for opioid use disorder; changes in substance use disorder prevalence from 2008-2016: what about stimulants?; and characterizing fentanyl use and related overdose and their challenges for overdose response: findings from a rapid ethnographic study in Vancouver, Canada.
2532	(2018///) 14th Annual Conference of the ISPTID. Tobacco Induced Diseases; 16<br><br>**Keywords:**<br>adolescent,breast cancer,cancer growth,cross-sectional study,electronic cigarette,female,human,India, Iran,male,marketing,perception,prevalence,Qatari,saliva,undergraduate student,water pipe smoking, amino acid,testosterone,Conference Review<br><br>**Abstract:** The proceedings contain 121 papers. The topics discussed include: direct effects of waterpipe tobacco smoking on cardiovascular health; effect of smoking on salivary free amino acid levels; rise of popularity of electronic cigarettes and correlates with marketing and regulations trajectory; role of water-pipe smoking in breast cancer progression; impact of water-pipe smoking on testosterone levels in Qatari males; hookah use among adolescent school students from urban slums of Mumbai, India; the role of socio-demographic factors associated with water pipe smoking among male adolescents, in western Iran: a cross-sectional study; and prevalence, harm perception, correlates of favorable harm perception and predictors of waterpipe smoking among university of ibadan undergraduate students.
2533	(2018///) 17th WCTOH Abstract. Tobacco Induced Diseases; 16

	**Keywords:**<br>awareness,electronic cigarette,human,India,librarian,library,Nigeria,perception,prevalence,rural population,smokeless tobacco,Sri Lanka,tobacco industry,world health organization,Conference Review<br><br>**Abstract:** The proceedings contain 969 papers. The topics discussed include: are strong tobacco control policies associated with bans on the retail sales of electronic nicotine delivery systems?; awareness and implementation of cigarette and other tobacco products act in selected communities in northern India; global policy progress in article 20 of world health organization's framework convention on tobacco control (WHO FCTC) on smokeless tobacco (SLT); the United Nations and its agencies, and their relationship with tobacco industry - an analysis of positions, policy and practice; empowering librarians on effective tobacco demand reduction through public libraries in Sri Lanka; upgrade needed: South African tobacco control policy and the who-framework convention on tobacco control; and prevalence, perceptions and predictors of smokeless tobacco use in a rural community in South Eastern Nigeria.
2535	(2019///) Smokers who use e-cigarettes no more likely to quit in the long term, study finds. Clinical Pharmacist; 11<br><br>**Keywords:**<br>human,short survey,*smoking cessation,*electronic cigarette<br><br>**Abstract:**
2536	J Ab Rahman, M H N Mohamed, M F M Yusoff, N S Ab Rahman, S Draman, M Kartiwi, B Mahadir, T Aris (2018///) Prevalence and characteristics of e-cigarette use among adults in Malaysia. Tobacco Induced Diseases; 16<br><br>**Keywords:**<br>adult,cigarette smoking,controlled study,education,*electronic cigarette,ethnicity,female,household, human,*Malaysia,Malaysian,male,manager,married person,Muslim,*prevalence,private sector, residential area,sampling,nicotine,conference abstract<br><br>**Abstract:** Background: E-cigarette (e-cig) use has become a worldwide phenomenon since 2010. This study aims to determine the prevalence of e-cig use, distribution and characteristics among Malaysian adults. Malaysia is a typical Asian country. The results of this study will provide evidence for policy makers to formulate appropriate measures towards regulation of e-cig in Malaysia and can become a reference for other similar countries. Method(s): Complex sampling design was used to represent 19 million of Malaysian adult household. Samples were stratified by states and urbanity. Sampling units were districts, enumeration blocks and living quarters. All adults from the selected houses were invited to participate in this survey. Analysis was done using sampling weight and complex sampling analysis. Characteristics of current e-cig users were described unweighted. Result(s): A total of 4,288 individual responded in this survey. Majority of the respondents were at 25-44 years of age group (44%), Malay ethnicity (73%), completed at least secondary level of education (69%), working in private sectors (26%), Muslim (79%) and married (68%). Male and female were equally represented. Based on weighted population, 72% of the respondents were from the urban residential area. The prevalence of current e-cig users in Malaysia was 3.2% (95%CI 2.5-4.1), involving an estimated population of about 600,000 household residence. The prevalence in urban and rural were 3.3% (95%CI 2.4-4.4) and 2.8% (95%CI 2.1-3.8) respectively. E-cig users were common among young, male, Malay, highly educated, Muslim, single and those in urban area. Most of current e-cig users started smoking e-cig when they were 19 years and above. 48% used e-cig just to try, while 17% used it to quit cigarette smoking. 62% preferred nicotine in their e-cig liquid. Conclusion(s): This study able to represent adults Malaysians and results can be used to guide healthcare managers to manage e-cigarette use countries with similar characteristics.

2537	J Ab Rahman, M F Mohd Yusoff, M H Nik Mohamed, B Mahadir Naidu, L K Hock, T G Hiong, M S Mohamad, M Kartiwi, S Draman, N S Ab Rahman, T Aris (2019///) The Prevalence of E-Cigarette Use Among Adults in Malaysia: Findings From the 2016 National E-Cigarette Survey. Asia-Pacific journal of public health;<br><br>**Keywords:**<br>adult,article,controlled study,education,*electronic cigarette,human,major clinical study,*Malaysia, Malaysian,male,*prevalence,questionnaire,rural area,*sampling,smoking<br><br>**Abstract:** Electronic cigarettes (e-cigarettes) are a new method for the consumption of nicotine. A nationwide survey among 4288 Malaysian adults was conducted in 2016 to measure the prevalence and to describe the population characteristics of e-cigarette users. A complex sampling design was used, and data were collected using a face-to-face questionnaire. The overall prevalence of current, ever, former, and dual users of e-cigarettes in Malaysia were 3.2% (95% confidence interval [CI] = 2.5-4.1), 11.9% (95% CI = 10.5-13.5), 8.6% (95% CI = 7.5-9.8), and 2.3% (95% CI = 1.8-3.1), respectively. The prevalence of all type of e-cigarette use was higher in urban than in rural areas. Current e-cigarette users were likely to be younger, males, and with higher education level. Among current e-cigarette users, 74% (95% CI = 64-82) also smoked conventional cigarettes (dual user). E-cigarette use is prevalent in Malaysia. It is common among younger adults, males, and cigarette smokers.
2538	H S Abdel Magid, P T Bradshaw, P M Ling, B Halpern-Felsher (2019///) Association of Alternative Tobacco Product Initiation With Ownership of Tobacco Promotional Materials Among Adolescents and Young Adults. JAMA network open; 2<br><br>**Keywords:**<br>adolescent,adult,article,California,cigarette smoking,clinical assessment,cohort analysis,controlled study, female,follow up,high school,Hispanic,human,major clinical study,male,marketing,outcome variable, Pacific Islander,*tobacco use,young adult<br><br>**Abstract:** Importance: Use of alternative tobacco products (ATPs) such as electronic cigarettes, chewing tobacco, pipes, cigars, cigarillos, little cigars, and hookah is rapidly increasing. Although marketing restrictions exist for cigarettes, marketing of ATPs is not yet fully regulated, and studies have not assessed the association between ownership of ATP promotional materials and subsequent ATP or cigarette initiation among adolescents and young adults. Objective(s): To estimate the association between marketing receptivity measured at baseline and ATP and any tobacco initiation 1 year later, including cigarettes, among adolescents and young adults. Design, Setting, and Participant(s): Longitudinal cohort study of adolescents and young adults aged 13 to 19 years recruited at high schools in California from July 2014 to October 2015, with follow-up 1 year later. Data were analyzed from January to March 2018. Exposures: Ownership of ATP-specific promotional material and ownership of any tobacco promotional materials (eg, samples, coupons, branded caps, t-shirts, or posters) assessed in wave 1. Main Outcomes and Measures: Outcomes were (1) ATP initiation and (2) either ATP or cigarette initiation in wave 2. Result(s): Of 757 participants (mean [SD] age at wave 1, 16.1 [1.1] years; 481 [63.5%] female; 166 [21.9%] Asian or Pacific Islander, 202 [26.7%] white, and 276 [36.4%] Latino), 129 (17.0%) initiated ATP use and 141 (18.6%) initiated ATP or cigarette use 1 year later. In unadjusted models, wave 2 ATP initiation was found to be significantly associated with wave 1 ownership of ATP promotional materials (odds ratio, 2.23; 95% CI, 1.26-3.97). After adjustment for wave 1 demographic covariates, the association between ownership of ATP promotional material and ATP initiation 1 year later yielded similar results (odds ratio, 2.13; 95% CI, 1.16-3.91). Results of models assessing a combined outcome variable of either ATP or cigarette ever use were not statistically significant. Conclusions and Relevance: Ownership of ATP promotional materials was associated with subsequent initiation of ATPs. The results of this study are consistent with the suggestion that current marketing restrictions for cigarettes,

	including restrictions of the distribution of samples, coupons, and other promotional material, should extend to ATPs.
2544	W P Adelman (2019///) Dual Use and Dual Truths: Categorizing the Adolescent Electronic Cigarette User. Journal of Adolescent Health; 65<br><br>**Keywords:**<br>adolescent,adolescent behavior,*adolescent smoking,cannabis use,*cigarette smoking,editorial, exposure,high school student,human,nicotine replacement therapy,nonhuman,priority journal,problem behavior,risk reduction,smoking cessation,tobacco dependence,v<br><br>**Abstract:**
2553	C Akre, A Berchtold, Y Barrense-Dias, J C Suris (2018///) From tobacco-related products to smoking: Results from a longitudinal study. Swiss Medical Weekly; 147<br><br>**Keywords:**<br>adolescent,cell cycle G1 phase,*cigarette smoking,controlled study,disease course,electronic cigarette, female,gender,hookah,human,joint,juvenile,*longitudinal study,major clinical study,male,nonsmoker, *smokeless tobacco,snus,student,Switzerland,tobacco,<br><br>**Abstract:** Introduction: To assess longitudinal factors related to smoking among youths depending on smoking status at baseline. Method(s): Data were drawn from the 2 first waves (T0:2016-T1:2017) of the GenFRee longitudinal study conducted in 11 post-mandatory schools in the Canton of Fribourg. Participants (N = 1606, mean age 16.2 at T0 [15-24], 45.8% females) were asked at T1 whether they smoked(G1, N = 571) or not(G2, N = 1035). Groups were compared on the use of tobacco-related products(TRP) (e-cigarettes, hookah, snus, and smokeless tobacco) in the past 12 months, use of other substances (alcohol, cannabis) and socio-demographic (age, gender, family structure, SES, academic track) variables at T0 separately by smoking status at T0. Groups were compared at the bivariate level and then at the multivariate level using a logistic regression analysis to assess the variables associated with being a smoker at T1 using non-smokers as reference category. Data are presented as odds ratios(OR) with 95% CI. Result(s): Smokers at T0: At the bivariate level, smokers at T1 were significantly more likely to be apprentices and showed a clear trend (p = .052) to be less likely to live in an intact family. At the multivariate level, both variables remained significant: they were more likely to be apprentices(OR: 2.32 [1.28:4.22]) and to live in a non-intact family(1.75 [1.01:3.04]). Non-smokers at T0: At the bivariate level, those who became smokers at T1 were significantly more likely to have used TRPs(45.7% vs. 18.2%; p <.001), to have misused alcohol, and to have used cannabis at T0. In the multivariate analysis, having used TRPs(3.05 [1.92:4.85]) and cannabis(2.89 [1.41:5.90]) remained significant. Moreover, when analyses were repeated separately by product, they remained significant(E-cig: 2.46 [1.54:3.91]; Smokeless tobacco: 1.88 [1.12:3.15]; Snus: 2.05 [1.07:3.92]; Hookah: 2.68 [1.73:4.14]). Conclusion(s): These longitudinal results show a key association between using TRPs and starting to smoke and seem to confirm the gateway effect of products such as hookah, snus, smokeless tobacco, and e-cigarettes. Thus, the potential harmful impact of these should not be underestimated. The association with cannabis is also important given that in Switzerland cannabis is mostly consumed in joints including tobacco. As an important percentage of non-smoking youths try different TRPs and act as an entry point to cigarette use, TRPs should be part of substance use screening among adolescents. Tobacco prevention should be broadened to include TRPs.
2554	Oakly Alana, Martin Greg (2019///) Dual use of electronic cigarettes and tobacco in New Zealand from a nationally representative sample. Australian and New Zealand journal of public health; 43<br><br>**Keywords:**<br>Adolescent,Adult,Age Factors,*Electronic Nicotine Delivery Systems,Female,Health Surveys,Humans,

	Male,Middle Aged,New Zealand/ep [Epidemiology],Nicotine/ad [Administration & Dosage],Prevalence, Socioeconomic Factors,*Tobacco Smoking/eh [Ethnology],*Vaping<br><br>**Abstract:** OBJECTIVE: There is strong interest in the use of electronic cigarettes (e-cigarettes) globally. Not much is known about the dual use of e-cigarettes and combustible tobacco cigarettes, or if there are demographic differences among dual users and e-cigarette only users. This paper reports on the demographics of dual users and e-cigarette only users in New Zealand in a nationally representative sample., METHODS: The Health and Lifestyles Survey (HLS) is a biennial face-to-face in-house survey of New Zealand adults aged 15 years or over. The HLS was completed by 3,854 participants in 2016., RESULTS: There is clear evidence of significant dual use in the current sample: most current e-cigarette users (63.9%) were dual users. Respondents 45 years and older were twice as likely to be dual users as those aged 15 to 34 years., CONCLUSION: The current study found evidence for substantial dual use of e-cigarettes and combustible tobacco cigarettes among adult e-cigarette users, particularly among users aged 45 years and over. Implications for public health: Public health initiatives should provide clear advice that e-cigarettes should be used as a smoking cessation tool and not as a way to allow the consumption of combustible tobacco to continue. Copyright © 2019 Health Promotion Agency.
2556	A Lopez Alexa, Redner Ryan, N Kurti Allison, R Keith Diana, C Villanti Andrea, A Stanton Cassandra, E Gaalema Diann, Y Bunn Janice, J Doogan Nathan, Cepeda-Benito Antonio, E Roberts Megan, T Higgins Stephen (2018///) Tobacco and nicotine delivery product use in a U.S. national sample of women of reproductive age. Preventive Medicine; 117<br><br>**Keywords:**<br>Adolescent,Adult,*Cigarette Smoking/ep [Epidemiology],*Electronic Nicotine Delivery Systems/sn [Statistics & Numerical Data],Female,Humans,Longitudinal Studies,Pregnancy,Prevalence,*Smokers/sn [Statistics & Numerical Data],Surveys and Questionnaires,*To<br><br>**Abstract:** This study examined prevalence and correlates of using cigarettes, e-cigarettes, and other tobacco/nicotine delivery products in a U.S. national sample of women of reproductive age. Weighted data were obtained from women aged 15-44years who were not currently pregnant in the first wave of the Population Assessment of Health and Tobacco (PATH, 2013-2014) study (N=12,848). 20.1% of women were current cigarette smokers, 5.9% current e-cigarette users, 4.9% current cigar smokers, and 6.5% current hookah users. Prevalence of current use of other tobacco products was <1.0%. Current cigarette smoking was the strongest correlate of current e-cigarette use (OR=65.7, 95% CI=44.8-96.5), cigar smoking (OR=19.2, 95% CI=14.1-26.1), and hookah use (OR=6.6, 95% CI=5.1-8.5). Among former cigarette smokers, 3.8%, 6.9%, and 3.2% were also currently using e-cigarettes, hookah, and cigars, respectively. Use of other tobacco and nicotine delivery products was low among those who never smoked tobacco cigarettes: 2.5% used hookah and <1.0% used other products. Cigarette smoking prevalence remains relatively high among women of reproductive age and strongly correlated with use of other tobacco products. Monitoring tobacco and nicotine use in this population is important due to the additional risk of adverse health impacts should they become pregnant. Clinicians working with cigarette smokers should assess for use of other tobacco products. Among women of reproductive age, use of emerging tobacco and nicotine products appears to be largely, although not exclusively, restricted to current cigarette smokers. Copyright © 2018 Elsevier Inc. All rights reserved.
2563	L Kristjansson Alfgeir, J Mann Michael, L Smith Megan, Sigfusdottir Inga Dora (2018///) Social Profile of Middle School-Aged Adolescents Who Use Electronic Cigarettes: Implications for Primary Prevention. Prevention science : the official journal of the Society for Prevention Research; 19<br><br>**Keywords:**<br>Adolescent,Adolescent Behavior,Cross-Sectional Studies,*Electronic Nicotine Delivery Systems,Humans, Logistic Models,*Primary Prevention,Schools,*Smoking Prevention,*Social Behavior,Surveys and Questionnaires,West Virginia

	**Abstract:** Electronic cigarette (EC) use continues to increase among adolescents. From a primary prevention point of view, an important gap in our knowledge includes determining whether young smokers that use EC maintain a distinct social profile from kids who use combustible cigarettes (CC). Survey data from middle school students in West Virginia, USA were collected between September and November of 2015 (N = 6547, response rate 84.7%) as part of a state wide school-based mental health intervention program. Data was analyzed using multinomial logistic regression for categorical data. The results show that compared to never smokers, EC-only users possessed a weaker social support and parental monitoring profile and performed worse in school. Additionally, EC-only users were more likely to feel alienated from school, to associate with delinquent peers, to spend time outside late at night, and to engage in unsupervised gatherings with their friends. In 11 of 13 statistical models no difference was observed between EC-only users compared with CC-only users. However, dual users (that had used both EC and CC in their lifetime) demonstrated a significantly greater risk profile compared with EC-only users. We conclude that middle school-aged kids that use EC share a similar risk profile as kids of the same age that use CC. Similar to traditional cigarette smoking, EC use in young adolescents is likely to be a social marker of a pathway to further delinquency and should therefore be subject to primary prevention approaches.
2586	S Gentzke Andrea, Creamer MeLisa, A Cullen Karen, K Ambrose Bridget, Willis Gordon, Jamal Ahmed, A King Brian (2019///) Vital Signs: Tobacco Product Use Among Middle and High School Students - United States, 2011-2018. MMWR. Morbidity and mortality weekly report; 68<br><br>**Keywords:**<br>Adolescent,Child,Cross-Sectional Studies,Ethnic Groups/sn [Statistics & Numerical Data],Female,Health Surveys,Humans,Male,Schools/sn [Statistics & Numerical Data],*Smoking/ep [Epidemiology],Smoking/eh [Ethnology],*Students/px [Psychology],Students/sn [S<br><br>**Abstract:** INTRODUCTION: Tobacco use is the leading cause of preventable disease and death in the United States; nearly all tobacco product use begins during youth and young adulthood., METHODS: CDC, the Food and Drug Administration, and the National Cancer Institute analyzed data from the 2011-2018 National Youth Tobacco Surveys to estimate tobacco product use among U.S. middle and high school students. Prevalence estimates of current (past 30-day) use of seven tobacco products were assessed; differences over time were analyzed using multivariable regression (2011-2018) or t-test (2017-2018)., RESULTS: In 2018, current use of any tobacco product was reported by 27.1% of high school students (4.04 million) and 7.2% of middle school students (840,000); electronic cigarettes (e-cigarettes) were the most commonly used product among high school (20.8%; 3.05 million) and middle school (4.9%; 570,000) students. Use of any tobacco product overall did not change significantly during 2011-2018 among either school level. During 2017-2018, current use of any tobacco product increased 38.3% (from 19.6% to 27.1%) among high school students and 28.6% (from 5.6% to 7.2%) among middle school students; e-cigarette use increased 77.8% (from 11.7% to 20.8%) among high school students and 48.5% (from 3.3% to 4.9%) among middle school students., CONCLUSIONS AND IMPLICATIONS FOR PUBLIC HEALTH PRACTICE: A considerable increase in e-cigarette use among U.S. youths, coupled with no change in use of other tobacco products during 2017-2018, has erased recent progress in reducing overall tobacco product use among youths. The sustained implementation of comprehensive tobacco control strategies, in coordination with Food and Drug Administration regulation of tobacco products, can prevent and reduce the use of all forms of tobacco products among U.S. youths.
2596	A Laverty Anthony, T Filippidis Filippos, I Vardavas Constantine (2018///) Patterns, trends and determinants of e-cigarette use in 28 European Union Member States 2014-2017. Preventive Medicine; 116<br><br>**Keywords:**<br>Adolescent,Adult,*Electronic Nicotine Delivery Systems/sn [Statistics & Numerical Data],European

	Union/sn [Statistics & Numerical Data],Female,Humans,Male,Middle Aged,*Smoking/ep [Epidemiology], Surveys and Questionnaires,*Vaping/td [Trends],Young Adult<br><br>**Abstract:** There is a lack of nationally representative data on regular e-cigarette use, as well as on the transition from experimentation to regular use. This study examines changes in these in Europe between 2014 and 2017. Data come from the 2014 (n=27,801) and 2017 (n=27,901) adult Special Eurobarometer for Tobacco Survey, providing nationally representative surveys of 28 EU member states. We defined regular use of e-cigarettes as daily or weekly use from a question on frequency of e-cigarette use. Among ever users of e-cigarettes we assessed socio-demographic correlates of becoming a regular user. 1.5% of the EU population were regular e-cigarette users in 2014, which had risen to 1.8% in 2017. In 2017 63 million Europeans aged 15 or older had ever used e-cigarettes (95% CI, 59.9 million-66.2 million), and 7.6 million (95% CI, 6.5million-8.9 million) were regular e-cigarette users. Among those who had ever used e-cigarettes, participants aged 15-24years were less likely to be regular user than those aged >=55years (16.9% vs. 38.1%), as were never smokers compared with current and former smokers (12.8% vs. 27.0% vs. 41.3%). The proportion of adults who were regular e-cigarette users in 2017 ranged from 4.7% in the UK to 0.2% in Bulgaria. There have been slight rises in the proportion of people regularly using e-cigarettes in the EU, and this varies considerably between member states, indicating the role of the regional environment in supporting or deterring e-cigarette use. Copyright © 2018 Elsevier Inc. All rights reserved.
2598	T M J Antin, G Hunt, E Kaner, S Lipperman-Kreda (2019///) Youth perspectives on concurrent smoking and vaping: Implications for tobacco control. International Journal of Drug Policy; 66<br><br>**Keywords:**<br>adolescent,adult,article,California,*cigarette smoking,harm reduction,human,interview,prevalence, priority journal,public health,smoking cessation,smoking regulation,tobacco dependence,United States, *vaping,young adult<br><br>**Abstract:** Most people who smoke cigarettes begin young. Consequently, public health efforts directed at youth are a priority. The increasing popularity of electronic nicotine delivery systems (ENDS) among youth in the United States has raised concerns in the public health community about the potential of ENDS to renormalize cigarette smoking and perpetuate nicotine addiction, creating dual users who both vape and smoke. Despite limited and inconclusive evidence about dual use for young people, restrictive approaches towards ENDS have shaped tobacco control agendas in the United States. Based on analysis of 26 interviews with young dual users in California, we explored the meanings young people ascribe to their dual using practices and how those meanings relate to the broader tobacco control environment which structures their lives. Results suggest that dual users of ENDS and cigarettes overwhelmingly perceive a utilitarian meaning of dual use and view vaping as a tool for reducing smoking-related harm in the near term and facilitating quitting smoking in the long term. Also, participants' narratives related to Tobacco 21 laws, which prohibit sales of both ENDS and cigarettes to individuals under 21 years of age, revealed concerns about restrictive policies that limit access to less harmful tobacco products. Results of this study raise important questions about whether we are working towards further reductions in the prevalence of smoking and tobacco-related diseases.Copyright © 2019
2599	G O Antwi, D K Lohrmann, W Jayawardene, A Chow, C S Obeng, A M Sayegh (2019///) Associations between e-cigarette and combustible cigarette use among U.S. cancer survivors: implications for research and practice. Journal of cancer survivorship : research and practice; 13<br><br>**Keywords:**<br>adult,alcohol consumption,article,behavioral risk factor surveillance system,*cancer survivor,*cigarette smoking,controlled study,*electronic cigarette,female,human,human tissue,major clinical study,male, nonsmoker,prevalence,probability sample,substitut

	**Abstract:** PURPOSE: Prior studies established significant associations between e-cigarette use and combustible cigarette smoking in the general population; however, little is known about such associations among cancer survivors. Thus, the current study examined possible associations between e-cigarette use and combustible cigarette smoking among U.S. cancer survivors. METHOD(S): Cross-sectional data were drawn from the 2016 Behavioral Risk Factor Surveillance System Survey. Binary logistic regression was used to analyze the associations between e-cigarette user status and combustible cigarette-smoking status in a sample of 4680 cancer survivors, controlling for alcohol use and sociodemographic factors. Analyses were weighted for unequal probability of sample selection to reflect national cancer survivor population estimates. RESULT(S): Prevalence for current e-cigarette use and combustible cigarette smoking for cancer survivors was 2.57% and 16.16%, respectively. In the adjusted analyses, cancer survivors who reported current e-cigarette use, compared to never-users, had greater odds of being current combustible cigarette smokers (odds ratio [OR]=11.81, 95% confidence interval [CI]=5.38-25.93). Likewise, former e-cigarette users, compared to never-users, had greater odds of being current combustible cigarette smokers (OR=15.90, 95% CI=10.68-23.36). CONCLUSION(S): Among cancer survivors in the USA, e-cigarette use had a positive and highly significant association with combustible cigarette smoking. IMPLICATIONS FOR CANCER SURVIVORS: In order to prevent multiple and substitute use of nicotine-delivery products, prevention interventions and cessation programs designed for cancer survivors should specifically target both current combustible cigarette smokers and non-smokers who report former and current e-cigarette use.
2608	L Merianos Ashley, F Mancuso Tierney, S Gordon Judith, J Wood Kelsi, A Cimperman Katherine, E Melinda Mahabee-Gittens (2018///) Dual- and Polytobacco/Nicotine Product Use Trends in a National Sample of High School Students. American journal of health promotion : AJHP; 32<br><br>**Keywords:**<br>Adolescent,*Adolescent Behavior/px [Psychology],*Electronic Nicotine Delivery Systems/sn [Statistics & Numerical Data],Female,Humans,Male,Risk Factors,*Students/sn [Statistics & Numerical Data],Surveys and Questionnaires,*Tobacco Products/sn [Statistics<br><br>**Abstract:** PURPOSE: The study purpose was to examine changes in patterns of ever and current dual- and polyproduct use over time and to examine demographic and modifiable risk factors including tobacco smoke exposure (TSE)., DESIGN: A secondary analysis of the 2013 to 2015 National Youth Tobacco Survey data., SETTING: Nationwide high schools were selected., SUBJECTS: A total of 31 022 high school students., MEASURES: Ever and current (past 30 days) tobacco/nicotine product use, home tobacco/nicotine product use, TSE and e-cigarette vapor exposure, and demographic characteristics were measured., ANALYSIS: Multivariable logistic regression and multinomial logistic regression models., RESULTS: Of the students, 9.4% were ever dual users and 18.6% were ever poly users. Rates of ever/current use of e-cigarettes and hookah increased from 2013 to 2015 (all Ps < .001). In 2015, participants were 4.8 times (95% confidence interval [CI], 4.5-5.2) and 4.0 times (95% CI, 3.5-4.4) more likely to report ever/current e-cigarette use and 1.61 times (95% CI, 1.5-1.7) and 1.48 times (95% CI, 1.3-1.7) more likely to report ever/current hookah use. Participants reporting TSE were 15.4 times (95% CI, 11.5-21.0) more likely to report current poly use, and those with e-cigarette exposure were 10.4 times (95% CI, 7.8-13.8) more likely to report current poly use., CONCLUSION: From 2013 to 2015, rates of ever and current use of e-cigarettes and hookah increased. Tobacco smoke and e-cigarette exposure were associated with higher rates of dual and poly use. Prevention efforts targeting these products are needed.
2613	R L Astor, R Urman, J L Barrington-Trimis, K Berhane, J Steinberg, M Cousineau, A M Leventhal, J B Unger, T Cruz, M A Pentz, J M Samet, R McConnell (2019///) Tobacco retail licensing and youth product use. Pediatrics; 143<br><br>**Keywords:**<br>adolescent,age,article,cigarette smoking,cohort analysis,controlled study,female,follow up,human,

	*licensing,male,*policy,prevalence,priority journal,*tobacco,cigar,cigarette,electronic cigarette,hookah, *tobacco retail licensing<br><br>**Abstract:** BACKGROUND: Restricting youth access to tobacco is a central feature of US tobacco regulatory policy, but impact of local tobacco retail licensing (TRL) regulation on cigarette smoking rates remains uncertain. Effects of TRL on other tobacco product use and use as adolescents reach the age to legally purchase tobacco products has not been investigated. METHOD(S): Prevalences of ever and past 30-day cigarette, electronic cigarette (e-cigarette), cigar, and hookah use were assessed in a survey of a cohort of 1553 11th- and 12th-grade adolescents (mean age: 17.3 years); rates of initiation were evaluated 1.5 years later. An American Lung Association (2014) youth access grade was assigned to each of 14 political jurisdictions in which participants lived on the basis of the strength of the local TRL ordinance. RESULT(S): At baseline, participants living in 4 jurisdictions with "A" grades (ie, with most restrictive ordinances) had lower odds of ever cigarette use (odds ratio [OR] 0.61; 95% confidence interval [CI] 0.41-0.90) and of past 30-day use (OR 0.51; 95% CI 0.29-0.89) than participants in 10 D- to F-grade jurisdictions. At follow-up at legal age of purchase, lower odds of cigarette use initiation (OR 0.67; 95% CI 0.45-0.99) occurred in jurisdictions with stronger TRL policy. Lower odds of e-cigarette initiation at follow-up (OR 0.74; 95% CI 0.55-0.99) and of initiation with past 30-day use (OR 0.45; 95% CI 0.23-0.90) were also associated with better regulation. CONCLUSION(S): Strong local TRL ordinance may lower rates of cigarette and e-cigarette use among youth and young adults.Copyright © 2019 by the American Academy of Pediatrics.
2615	A H Nur Atikah, Wee Lei Hum, M S Nur Zakiah, Chan Caryn Mei Hsien, N M Mohamed Haniki, J S Swinderjit, Siau Ching Sin (2019///) Factors associated with different smoking statuses among Malaysian adolescent smokers: a cross-sectional study. BMC public health; 19<br><br>**Keywords:**<br>Adolescent,Cross-Sectional Studies,Electronic Nicotine Delivery Systems/sn [Statistics & Numerical Data], Ethnic Groups/sn [Statistics & Numerical Data],Female,Humans,Logistic Models,Malaysia/ep [Epidemiology],Male,*Smokers/sn [Statistics & Numerical Dat<br><br>**Abstract:** BACKGROUND: This study focused on the associations between socioeconomic status (SES) and adolescent smoking among secondary school students (13 to 17 years) in the Federal Territory of Kuala Lumpur, Malaysia. Our objective was to evaluate the relationships between adolescent demographics, socioeconomic status and smoking status., METHODS: The survey data were based on baseline findings from a cross-sectional study (N = 422 adolescents). Chi-square test was used to assess the relationship between demographic characteristics, socioeconomic status (household monthly income and daily allowance) and adolescent smoking status. Exhaled carbon monoxide (CO) reading and the Hooked on Nicotine Checklist (HONC) were used to evaluate adolescent smoking status. A Multivariate Multinomial Logistic Regression (MMLR) was employed to test selected demographic and socioeconomic predictors of smoking status., RESULTS: Of the 422 adolescents (M age = 15.58, SD = 1.24), more than half of the participants initiated smoking between 13 to 17 years old (59.0%). A total of 308 (73.0%) were electronic cigarette users, with more than 50% comprising of single users. The mean CO reading was 2.14 ppm with 78.0% of adolescents scoring more than 0 on the Hooked on Nicotine Checklist (HONC). Males and participants aged 15 and 16 years were at increased risks of sole CC smoking. Meanwhile, males, those who are not hooked on smoking and with a non-smoker CO reading were at increased risks of sole EC smoking. Finally, Bumiputeras were at less risk of EC smoking., CONCLUSIONS: Demographic variables such as age, gender and ethnicity predicted smoking status predicted smoking risk, but not socioeconomic factors. The findings allow policy makers to target specific high-risk demographic groups when designing smoking cessation programs for adolescents.
2618	Mayet Aurelie, Lavagna Chrystel (2018///) Electronic cigarettes: harm reduction tool or new substance use behavior?. Addiction (Abingdon, England); 113<br><br>**Keywords:**

	*Electronic Nicotine Delivery Systems,Harm Reduction,Humans,*Smoking Cessation,*Tobacco Use Disorder<br><br>**Abstract:**
2621	S Azagba, K Kah, K Latham (2019///) Frequency of E-cigarette use and cigarette smoking among Canadian students. Preventive Medicine; 126<br><br>**Keywords:**<br>adolescent,*adolescent smoking,article,Canadian,controlled study,cross-sectional study,descriptive research,female,health survey,household income,human,male,outcome variable,population research, priority journal,rural area,urban area,*electronic cigarett<br><br>**Abstract:** The emergence of electronic nicotine delivery systems (commonly referred to as e-cigarettes) has created an ongoing public health debate and concerns, especially in regards to adolescents. The present study examined associations between the frequency of e-cigarette use and cigarette smoking among school students. Data on students (grades 7-12) was obtained from the 2016-2017 Canadian Student Tobacco, Alcohol and Drugs Survey (n = 51,661). Multivariable logistic regression analyses were conducted to examine associations between the frequency of e-cigarette use and smoking. Subgroup analyses were performed for male and female students. Among those who reported any cigarette smoking in the past 30 days, 55% reported e-cigarette use at least once in the last 30 days. Additionally, 17% reported e-cigarette use for 21-30 days among those who smoked cigarettes for at least 21 days. Multivariable logistic regression analysis showed e-cigarette use was associated with higher odds of cigarette smoking, especially among more frequent e-cigarette users. Those who used an e-cigarette 21-30 days had higher odds of cigarette smoking (smoked cigarette at least once AOR = 4.83, CI = 3.33-7.01; at least 11 days AOR = 3.73, CI = 2.40-5.80; at least 21 days AOR = 3.39, CI = 2.16-5.34). Findings suggest that those who smoked cigarettes at least once in the past 30 days had a higher frequency of e-cigarette use.Copyright © 2019 Elsevier Inc.
2622	S Azagba, K Latham, L Shan (2019///) Cigarette smoking, e-cigarette use, and sexual identity among high school students in the USA. European Journal of Pediatrics; 178<br><br>**Keywords:**<br>adolescent,article,bisexual female,bisexuality,*cigarette smoking,cohort analysis,controlled study, drinking behavior,female,*gender identity,heterosexual female,heterosexuality,high school student, homosexual female,human,LGBT people,male,priority journa<br><br>**Abstract:** Although tobacco use has decreased in the general population in recent years, smoking remains high among subpopulations. This study examined whether sexual identity is associated with cigarette smoking and e-cigarette use among high school students. Data were drawn from the US Youth Risk Behavior Surveillance System (n = 28811). We grouped participants according to sexual identity status. Propensity score matching technique was used to address covariate imbalance among sexual identity groups. Subgroup analyses were performed for male and female students. Of the four sexual identity groups analyzed in this study, bisexual adolescents had significantly higher odds of cigarette smoking (cigarette smoking, OR, 1.61; 95% CI, 1.24-2.08; cigarette smoking for >= 10 days, OR, 1.79; 95% CI, 1.28-2.51; cigarette smoking for >= 20 days, OR, 2.04; 95% CI, 1.38-3.03). Further, in a sex-based subgroup analysis, results showed significant differences, with bisexual females more likely to smoke cigarettes and use e-cigarettes for >= 20 days compared with heterosexual female adolescents. Conclusion(s): Sexual identity status is strongly associated with cigarette smoking and e-cigarette use. Female bisexual adolescents have an increased risk of cigarette smoking and e-cigarette use for >= 20 days. While tobacco control policies have been effective in reducing cigarette use in the general population, targeting policy toward sexual minorities is an important consideration.What is Known:* Sexual minority groups face a disproportionate amount of stress and have a heightened risk of substance use.* Methodological

	concerns exist in the extant literature, including limited data at the national level.What is New:* Propensity score matching was used to account for imbalances in sexual identity subgroups, and findings show significant heterogeneity in cigarette and e-cigarette use among sexual minority high school students.* Sexual identity status is strongly associated with cigarette smoking and e-cigarette use, especially among bisexual identity female subgroup.Copyright © 2019, Springer-Verlag GmbH Germany, part of Springer Nature.
2624	S Azagba, L Shan, K Latham (2019///) Adolescent Dual Use Classification and Its Association With Nicotine Dependence and Quit Intentions. Journal of Adolescent Health; 65<br><br>**Keywords:**<br>adolescent,*adolescent smoking,article,Caucasian,*cigarette smoking,female,*health behavior,health belief,Hispanic,human,major clinical study,male,priority journal,race difference,retrospective study,self concept,*smoking cessation,*tobacco dependence,*<br><br>**Abstract:** Purpose: Electronic cigarettes (e-cigarette) use is rapidly increasing in the U.S., especially among adolescents. A significant number of adolescents use both cigarettes and e-cigarettes, often referred to as dual use. We used a new classification of dual use, taking into account the frequency of use of both products. In addition, we examined the association between dual use with time to first cigarette after waking (a nicotine dependence measure) and quit intention. Method(s): Data were drawn from the 2015-2018 National Youth Tobacco Survey. We grouped participants by dual use frequency. Multivariable logistic regression was used to examine the association of dual use frequency with nicotine dependence and quit intention. Result(s): Different categories of dual users varied in demographic characteristics and beliefs on the harms of cigarette smoking and e-cigarette use. Compared with cigarette-only smokers, significantly higher odds of nicotine dependence were found for high-frequency e-cigarette dual users (within 5 minutes of awakening, odds ratio [OR]: 1.67, 95% confidence interval [CI], 1.07-2.62; within 30 minutes, OR: 1.61, 95% CI, 1.15-2.27), high-frequency cigarette dual users (5 minutes, OR: 2.85, 95% CI, 1.76-4.63; within 30 minutes, OR: 4.14, 95% CI, 2.96-5.80), and high-frequency dual users (5 minutes, OR: 4.46, 95% CI, 2.88-6.91; 30 minutes, OR: 3.94, 95% CI, 2.43-6.42). In addition, high-frequency e-cigarette dual users had significantly lower quit intention compared with both cigarette-only smokers and low-frequency dual users. Conclusion(s): Findings highlight the need for a standard, granulated classification of dual user, as important characteristics may vary between different categories. Future studies on dual use should consider categorizing dual use into the four classifications described in this study.Copyright © 2019 Society for Adolescent Health and Medicine
2631	R Barrett (2019///) Adverse-event management and reporting for electronic cigarettes (e-cigarettes). European Journal of Hospital Pharmacy; 26<br><br>**Keywords:**<br>acute diarrhea,alcohol abuse,clinical practice,coughing,drug use,editorial,health care personnel,human, Human immunodeficiency virus infection,leprosy,nicotine replacement therapy,pregnancy,public health campaign,respiratory tract disease/co [Complicatio<br><br>**Abstract:**
2634	J L Barrington-Trimis, M S Bello, F Liu, A M Leventhal, G Kong, M Mayer, T B Cruz, S Krishnan-Sarin, R McConnell (2019///) Ethnic Differences in Patterns of Cigarette and E-Cigarette Use Over Time Among Adolescents. Journal of Adolescent Health; 65<br><br>**Keywords:**<br>adolescent,article,cigarette smoking,cohort analysis,controlled study,demography,*ethnic difference, female,follow up,health care policy,human,major clinical study,male,prevalence,priority journal, prospective study,*tobacco use,*cigarette,*electronic cig

	**Abstract:** Purpose: Little is known about whether adolescent cigarette and e-cigarette use patterns over time differ by ethnicity. Method(s): Data were pooled from three prospective cohort studies of adolescents in California and Connecticut (baseline: 2013-2014; 12-month follow-up: 2014-2015; N = 6, 258). Adjusted polytomous regression models evaluated the association of baseline exclusive ever e-cigarette use, exclusive ever cigarette use, ever use of both e-cigarettes and cigarettes (dual use) with past 30-day use at follow-up (exclusively e-cigarettes, exclusively cigarettes, dual use; no use at baseline/follow-up were the referent groups). Interaction analyses evaluated differences by race/ethnicity (Hispanic white [HW], non-Hispanic white [NHW], Other). Result(s): A significant global interaction was observed for the association of baseline with follow-up tobacco use by ethnicity (p = .009). Among NHW participants, ever e-cigarette or cigarette users at baseline (vs. never users) had significantly higher odds of every past 30-day use tobacco use pattern at follow-up. Among HW participants, compared with never users, exclusive e-cigarette users at baseline had increased odds of continued e-cigarette use (ORexclusive e-cigarettes = 5.22; 95% confidence interval [CI]: 3.50, 7.79; ORdual use = 3.64; 95% CI: 1.62, 8.18) but not of transition to exclusive cigarette use at follow-up (ORexclusive cigarettes = 1.27; 95% CI: .47, 3.46), and HW exclusive cigarette users at baseline had greater odds of continued cigarette use (ORexclusive e-cigarettes = 12.3; 95% CI: 5.87, 25.8; ORdual use = 3.82; 95% CI: 1.06, 13.7) but not of transition to exclusive e-cigarette use at follow-up (ORexclusive cigarettes = 1.61; 95% CI: .62, 4.18). Conclusion(s): Findings that NHW youth report more transitional use patterns and HW youth report more stable use patterns suggest a potential for differential impacts of e-cigarettes, by ethnicity, in increasing subsequent transition to or cessation from cigarette smoking.Copyright © 2019 Society for Adolescent Health and Medicine
2635	J L Barrington-Trimis, F Liu, J B Unger, T Alonzo, T B Cruz, R Urman, M A Pentz, K Berhane, R McConnell (2019///) Evaluating the predictive value of measures of susceptibility to tobacco and alternative tobacco products. Addictive Behaviors; 96<br><br>**Keywords:**<br>adolescent,adult,article,disease association,*disease predisposition,female,groups by age,human,male, *predictive value,prospective study,questionnaire,risk factor,*tobacco dependence,tobacco use,young adult,cigarette smoke,*tobacco smoke,electronic ciga<br><br>**Abstract:** Background: The "cigarette susceptibility index" has been adapted for other products, yet, the validity of these adapted measures-particularly among youth who have used other tobacco products-has not been evaluated. Method(s): We used prospective data from the Southern California Children's Health Study to evaluate the association of questionnaire measures assessing susceptibility to e-cigarette, cigarette, hookah and cigar/cigarillo/little cigar use at wave 1 (W1; 11th/12th grade)with subsequent initiation between W1 and W2 (16 months later, N = 1453). We additionally examined whether each effect estimate differed by use of other tobacco products at W1. Result(s): Odds ratios, attributable risk%, and risk differences for product initiation among susceptible vs. non-susceptible youth were consistently higher among never users of any tobacco product than among youth with any tobacco use history. For example, susceptible (vs. non-susceptible)youth with no prior tobacco use had 3.64 times the odds of subsequent initiation of e-cigarettes (95%CI:2.61,5.09), while among users of another product, susceptible (vs. non-susceptible)youth had 1.95 times the odds of e-cigarette initiation (95%CI:0.98,3.89; p-interaction = 0.016). 60.4% of e-cigarette initiation among never users of any product could be attributed to susceptibility, compared to 19.8% among users of another product. The e-cigarette absolute risk difference between susceptible and non-susceptible youth was 21.9%(15.2,28.6)for never users, vs. 15.4%(0.2,30.7)for users of another product. Conclusion(s): Tobacco product-specific susceptibility associations with initiation of use at W2 were markedly attenuated among prior users of other products, demonstrating reduced utility for these measures among subjects using other products.Copyright © 2019 Elsevier Ltd

2637	R S Barua, N A Rigotti, N L Benowitz, K M Cummings, M A Jazayeri, P B Morris, E V Ratchford, L Sarna, E C Stecker, B S Wiggins (2018///) 2018 ACC Expert Consensus Decision Pathway on Tobacco Cessation Treatment: A Report of the American College of Cardiology Task Force on Clinical Expert Consensus Documents. Journal of the American College of Cardiology; 72<br><br>**Keywords:**<br>agitation,article,behavior change,body weight gain,cardiology service,cardiovascular disease,cigarette smoking,cognitive behavioral therapy,coughing/si [Side Effect],craving,depression/si [Side Effect],drug efficacy,drug safety,e-counseling,education pr<br><br>**Abstract:**
2649	K Bentivegna, N C Atuegwu, C Oncken, E L Mead, M F Perez, E M Mortensen (2019///) E-cigarette Use Is Associated with Non-prescribed Medication Use in Adults: Results from the PATH Survey. Journal of General Internal Medicine;<br><br>**Keywords:**<br>adult,*electronic cigarette,human,note<br><br>**Abstract:**
2650	A W Bergen, E K Do, L S Chen, S P David (2018///) Tobacco genomics: Complexity and translational challenges. Nicotine and Tobacco Research; 21<br><br>**Keywords:**<br>African American,aging,alcohol consumption,alcoholism,allele,Caucasian,chromosome 11,chromosome 15,chromosome 15q,chromosome 19,chromosome 20,chromosome 8,chronic obstructive lung disease, comorbidity,correlational study,disease association,drinking beha<br><br>**Abstract:**
2652	I Berlin, B Nalpas, R Targhetta, P Perney (2019///) Comparison of e-cigarette use characteristics between exclusive e-cigarette users and dual e-cigarette and conventional cigarette users: an online survey in France. Addiction (Abingdon, England);<br><br>**Keywords:**<br>adult,article,controlled study,drug therapy,*electronic cigarette,female,*France,human,major clinical study,male,outcome assessment,*nicotine<br><br>**Abstract:** BACKGROUND AND AIMS: Completely substituting e-cigarettes (EC) for combustible tobacco cigarettes reduces exposure to toxicants and carcinogens. However, a large proportion of EC users (dual users) continue to smoke conventional cigarettes. This study aimed to compare estimated nicotine intake and e-cigarette use characteristics between exclusive EC users and dual users. DESIGN: Web-based anonymous cross-sectional survey.France. PARTICIPANTS: 3189 adults, current users of electronic cigarettes (EC). Data collection between 4 October 2014 and 11 November 2014. MEASUREMENTS: Primary outcome: estimated nicotine intake per day (mg) from participants' reports. SECONDARY OUTCOMES: duration, frequency of EC use, and nicotine content of e-liquids used/day. Dual use was defined as using at least one cigarette per day while also using EC. FINDINGS: 2836 respondents reported exclusive EC use and 353 reported being dual users. Backward stepwise logistic regression showed that dual users had higher estimated combined daily nicotine intake from e-liquids and cigarettes [estimate: 2.14 SE: 0.26, adjusted odds ratio (aOR): 8.48, 95% confidence interval (CI)= 5.11 to 14.09, P<0.001], but lower daily nicotine intake from EC [estimate: -2.14, SE: 026, aOR: 0.12, CI=0.07 to 0.196, P<0.001] and reported fewer months of EC use [estimate -0.31, SE: 0.14, aOR: 0.73, CI=0.56 to 0.95, P=0.022]

	compared with exclusive EC users. CONCLUSION(S): Dual e-cigarette users in France may have higher nicotine intake overall than exclusive e-cigarette users but they may take in less nicotine from their e-cigarettes.Copyright This article is protected by copyright. All rights reserved.
2655	K M Berry, J L Fetterman, E J Benjamin, A Bhatnagar, J L Barrington-Trimis, A M Leventhal, A Stokes (2019///) Association of Electronic Cigarette Use With Subsequent Initiation of Tobacco Cigarettes in US Youths. JAMA network open; 2<br><br>**Keywords:**<br>adolescent,article,child,*cigarette smoking,cohort analysis,controlled study,data analysis,*electronic cigarette,female,human,*juvenile,major clinical study,male,prospective study,*tobacco use<br><br>**Abstract:** Importance: The use of electronic cigarettes (e-cigarettes) and other noncigarette tobacco products may increase the odds of cigarette initiation, even among low-risk youths. Objective(s): To evaluate the associations of prior e-cigarette use and other tobacco product use with subsequent cigarette initiation within 2 years of follow-up. Design, Setting, and Participant(s): In this prospective cohort study, data from waves 1 through 3 of the Population Assessment of Tobacco and Health Study (2013-2016) were used to assess youths aged 12 to 15 years who had never used cigarettes, e-cigarettes, or other tobacco products at wave 1. This was a nationally representative study of the US population. Data analysis was conducted in 2018. Exposures: First noncigarette tobacco product used (none, e-cigarette, or other tobacco product) between wave 1 and wave 3. Main Outcomes and Measures: Ever cigarette use and current cigarette use at wave 3. Result(s): In the sample (N=6123), respondents were 49.5% female; 54.1% non-Hispanic, white; and the mean (SD) age was 13.4 (1.2) years. Of these, 8.6% reported e-cigarettes as their first tobacco product, while 5.0% reported using another noncigarette product first; 3.3% reported using cigarettes first. Cigarette use at wave 3 was higher among prior e-cigarette users (20.5%) compared with youths with no prior tobacco use (3.8%). Prior e-cigarette use was associated with more than 4 times the odds of ever cigarette use (odds ratio, 4.09; 95% CI, 2.97-5.63) and nearly 3 times the odds of current cigarette use (odds ratio, 2.75; 95% CI, 1.60-4.73) compared with no prior tobacco use. Prior use of other tobacco products was similarly associated with subsequent ever cigarette use (OR, 3.84; 95% CI, 2.63-5.63) and current cigarette use (OR, 3.43; 95% CI, 1.88-6.26) compared with no prior tobacco use. The association of prior e-cigarette use with cigarette initiation was stronger among low-risk youths (OR, 8.57; 95% CI, 3.87-18.97), a pattern not seen for prior other product use. Over the 2 years between 2013 and 2014 and 2015 and 2016, 21.8% of new cigarette ever use (178850 youths) and 15.3% of current cigarette use (43446 youths) among US youths aged 12 to 15 years may be attributable to prior e-cigarette use. Conclusions and Relevance: This study's findings support the notion that e-cigarette use is associated with increased risk for cigarette initiation and use, particularly among low-risk youths. At the population level, the use of e-cigarettes may be a contributor to the initiation of cigarette smoking among youths.
2656	N Bertoni, A Szklo, R De Boni, C Coutinho, M Vasconcellos, P Nascimento Silva, L M e Almeida, F I Bastos (2019///) Electronic cigarettes and narghile users in Brazil: Do they differ from cigarettes smokers?. Addictive Behaviors; 98<br><br>**Keywords:**<br>adolescent,adult,age,aged,article,Brazil,Brazilian,child,*cigarette smoking,controlled study,family income, female,geography,high risk population,human,male,prevalence,sex difference,sexual orientation,social status,*vaping,*water pipe smoking,electronic<br><br>**Abstract:** Background: Cigarette smoking prevalence is declining, however, other tobacco products have emerged recently, such as electronic cigarettes (e-cigarettes) and narghile (hookah/shisha/waterpipe). Narghile sales are not prohibited in Brazil, but e-cigarettes are. Accurate estimates of such products are key for proper monitoring and control. Objective(s): To describe the prevalence of e-cigarettes and narghile use and to investigate whether sociodemographic characteristics of individuals who are using

	these products differ from those who are using manufactured cigarettes. Method(s): Using a nationally representative sample survey of Brazilians aged 12-65 years in 2015, we estimated the prevalence rates of each tobacco products within the last 12-months, stratified by macro-region, municipality size, sex-at-birth, sexual orientation, color/race, age-group, and monthly income. Multivariable logistic models were fitted to understand determinants of each tobacco product use, considering the complex sample design. Result(s): E-cigarette, narghile, and cigarette prevalence rates were estimated at 0.43%, 1.65% and 15.35%, respectively, corresponding to around 0.6million e-cigarette users, 2.5 million narghile users and 23.5 million cigarette users. Non-heterosexual individuals were a most-at-risk group for both e-cigarette/narghile and cigarette use. Despite similarities, e-cigarette/narghile users were younger and had higher socioeconomic status than cigarette users. Additional analyses showed that recent cigarette use seems to be more associated with e-cigarette/narghile use among youth than among adults. Conclusion(s): Our findings may provide valuable information about e-cigarette/narghile use in Brazil. Prevention strategies targeted to youth to both narghile and e-cigarettes use should be implemented together, which might be one strategy to prevent the emergency of a new generation of smokers in Brazil.Copyright © 2019 Elsevier Ltd
2659	A Bhatnagar, T J Payne, R M Robertson (2019///) Is There A Role for Electronic Cigarettes in Tobacco Cessation?. Journal of the American Heart Association; 8<br><br>**Keywords:**<br>follow up,harm reduction,health hazard,human,note,population risk,prevalence,priority journal,risk factor,smoking,*smoking cessation,tobacco dependence,*electronic cigarette<br><br>**Abstract:**
2663	Kilibarda Biljana, Krstev Srmena, Milovanovic Minja, K Foley (2019///) E-cigarette use in Serbia: Prevalence, reasons for trying and perceptions. Addictive Behaviors; 91<br><br>**Keywords:**<br>*Epidemiology,*Motivation,*Electronic Cigarettes,Test Construction<br><br>**Abstract:** This study examines the prevalence, reasons for trying, and perceptions of E-cigarette use in Serbia. The aim of this study was to assess rates of electronic cigarette use among the adult population of Serbia in 2017, three years after the first data on the prevalence of e-cigarette use were obtained, to identify the motivation for its use, smoking patterns of those who used electronic cigarettes and to explore population attitudes toward electronic cigarette use. The highest prevalence of ever use of e-cigarettes is in the age group 25-34. Percentage of those who only tried electronic cigarettes is the highest (13.7%) in the youngest age groups and difference is statistically significant compared to age groups 35-44, 55-64 and 65+. Majority of Serbian adults agree or totally agree that e-cigarette use is harmful to health with no statistically significant differences in any category. Finding that most e-cigarette users perceive e-cigarettes to have no influence on their smoking behavior, together with support to its regulation as tobacco product should be communicated urgently with all national stakeholders. Perception of the harmfulness of both e-cigarettes and second-hand vapor should additionally foster changing of current legislation which would better regulate this product. (PsycINFO Database Record (c) 2019 APA, all rights reserved)
2667	Coleman Blair, Rostron Brian, E Johnson Sarah, Persoskie Alexander, Pearson Jennifer, Stanton Cassandra, Choi Kelvin, Anic Gabriella, Goniewicz Maciej Lukasz, Cummings Kenneth Michael, A Kasza Karin, L Silveira Marushka, Delnevo Cristine, Niaura Raymond, B Abrams David, L Kimmel Heather, Borek Nicolette, M Compton Wilson, Hyland Andrew (2019///) Transitions in electronic cigarette use among adults in the Population Assessment of Tobacco and Health (PATH) Study, Waves 1 and 2 (2013-2015). Tobacco control; 28

	**Keywords:**<br>Adolescent,Adult,*Cigarette Smoking/ep [Epidemiology],Cohort Studies,*Electronic Nicotine Delivery Systems,Humans,Longitudinal Studies,Prevalence,Probability,*Smoking Cessation/sn [Statistics & Numerical Data],Time Factors,United States/ep [Epidemiology<br><br>**Abstract:** INTRODUCTION: This study assessed patterns of e-cigarette and cigarette use from Wave 1 to Wave 2 among adult e-cigarette users at Wave 1 of the Population Assessment of Tobacco and Health (PATH) Study., METHODS: We examined changes in e-cigarette use frequency at Wave 2 among adult e-cigarette users at Wave 1 (unweighted n=2835). Adjusted prevalence ratios (aPR) were calculated using a predicted marginal probability approach to assess correlates of e-cigarette discontinuance and smoking abstinence at Wave 2., RESULTS: Half (48.8%) of adult e-cigarette users at Wave 1 discontinued their use of e-cigarettes at Wave 2. Among dual users of e-cigarettes and cigarettes at Wave 1, 44.3% maintained dual use, 43.5% discontinued e-cigarette use and maintained cigarette smoking and 12.1% discontinued cigarette use at Wave 2, either by abstaining from cigarette smoking only (5.1%) or discontinuing both products (7.0%). Among dual users at Wave 1, daily e-cigarette users were more likely than non-daily users to report smoking abstinence at Wave 2 (aPR=1.40, 95% CI 1.02 to 1.91). Using a customisable device (rather than a non-customisable one) was not significantly related to smoking abstinence at Wave 2 (aPR=1.14, 95% CI 0.81 to 1.60)., CONCLUSIONS: This study suggests that e-cigarette use patterns are highly variable over a 1-year period. This analysis provides the first nationally representative estimates of transitions among US adult e-cigarette users. Future research, including additional waves of the PATH Study, can provide further insight into long-term patterns of e-cigarette use critical to understanding the net population health impact of e-cigarettes in USA. Copyright © Article author(s) (or their employer(s) unless otherwise stated in the text of the article) 2019. All rights reserved. No commercial use is permitted unless otherwise expressly granted.
2670	S A Boddu, C M Bojanowski, M T Lam, I N Advani, E L Scholten, X Sun, P Montgrain, A Malhotra, S Jain, L E Crotty Alexander (2019///) Use of E-cigarettes with Conventional Tobacco is Associated with Decreased Sleep Quality in Women. American journal of respiratory and critical care medicine;<br><br>**Keywords:**<br>adult,article,*electronic cigarette,female,*gender,human,*Pittsburgh Sleep Quality Index,*sleep quality, *tobacco<br><br>**Abstract:**
2676	R Borland, K Murray, S Gravely, G T Fong, M E Thompson, A McNeill, R J O'Connor, M L Goniewicz, H H Yong, D T Levy, B W Heckman, K M Cummings (2019///) A new Classification System for describing concurrent use of Nicotine Vaping Products alongside Cigarettes (so-called "Dual Use"): Findings from the ITC-4 Country Smoking and Vaping Wave 1 Survey t. Addiction (Abingdon, England);<br><br>**Keywords:**<br>adult,article,Australia,Canada,controlled study,England,female,human,major clinical study,male,smoke, tobacco dependence,United States,*vaping,*nicotine<br><br>**Abstract:** AIMS: To determine whether a simple combination of level of smoking and level of vaping results in a useful typology for characterising smoking and vaping behaviours. METHOD(S): Cross-sectional data from adults (>=18 years) in the 2016 Wave 1 ITC Four Country Smoking and Vaping Survey in the United States (n=2291), England (n=3591), Australia (n=1376), and Canada (n=2784) were used. Participants who either smoked, vaped or concurrently used both at least monthly were included and divided into 8 groups based on use frequency of each product (daily, non-daily, no current use). This resulted in 4 concurrent use groups (predominant smokers, dual daily users, predominant vapers and concurrent non-daily users). These groups were compared with each other and with the 4 exclusive use groups, on socio-demographics, nicotine dependence, beliefs and attitudes about both products, and

	quit-related measures using data weighted to reference population surveys in each country. RESULT(S): 10.8% of the sample were concurrent users, with daily smokers vaping non-daily (predominant smokers) constituting 51.6% of this group. All 8 categories differed from other categories on at least some measures. Concurrent daily nicotine users reported higher levels of indicators of nicotine dependence, and generally more positive attitudes toward both smoking and vaping than concurrent non-daily users. Among daily nicotine users, compared with exclusive daily smokers, reports of interest in quitting were higher in all concurrent use groups. Dual daily users had the most positive attitudes about smoking overall, and saw it as the least denormalised, and at the same time were equally interested in quitting as other concurrent users and were most likely to report intending to continue vaping. CONCLUSION(S): In Australia, Canada, England and the United States in 2016, daily nicotine users differed considerably from non-daily nicotine users. Among daily nicotine users, dual daily users (those who smoke and vape concurrently) should be treated as a distinct grouping when studying relationships between smoking and vaping. The 8 level typology characterising concurrent and exclusive use of smoking and vaping should be considered when studying both products.Copyright This article is protected by copyright. All rights reserved.
2683	J Bozier, S Rutting, D Xenaki, M Peters, I Adcock, B G Oliver (2019///) Heightened response to e-cigarettes in COPD. ERJ Open Research; 5<br><br>**Keywords:**<br>airway smooth muscle cell,*chronic obstructive lung disease,cytotoxicity,enzyme linked immunosorbent assay,exposure,flavor,forced expiratory volume,forced vital capacity,human,inflammation,letter, physician,pneumonia,pollution,public health,smoking,smoki<br><br>**Abstract:**
2686	A Braillon (2018///) E-cigarettes and the Youngest, Not a Problem in Europe: No Data Yet. American Journal of Preventive Medicine; 54<br><br>**Keywords:**<br>age,epidemic,*Europe,European,human,letter,pediatrics,prevalence,public health,smoking,*smoking cessation,tobacco consumption,*vaping,menthol,nicotine,*tobacco control<br><br>**Abstract:**
2688	T Brandon, U Martinez, V Simmons, L Meltzer, S Sutton, D Drobes, M Unrod, P Harrell (2018///) Dual use of combustible and electronic cigarettes: Patterns and associations between products. Tobacco Induced Diseases; 16<br><br>**Keywords:**<br>adult,controlled study,female,human,inferential statistics,Internet,longitudinal study,male,questionnaire, randomization,randomized controlled trial,smoking cessation,*vaping,nicotine,conference abstract<br><br>**Abstract:** Background: The use of electronic cigarettes (e-cigarettes) has increased dramatically over the past decade. Although the majority of users report initiating e-cigarette use (vaping) to quit or reduce use of combustible cigarettes (smoking), a substantial proportion become "dual users" of both. Little is known about the patterns of dual use of, and dependence on, these products. This study describes those patterns as well as associations between smoking with vaping variables. Method(s): During 2016-17, 2894 dual users were recruited into a 2-year longitudinal study testing nicotine cessation interventions. Inclusion criteria required at least weekly use of both products. This report uses baseline questionnaire data collected via mail or Internet before randomization. Descriptive and inferential statistics were calculated. Result(s): Participants' mean age was 29.9 (SD 11.3) years, and 63% were male. They reported smoking a median of 16-20 cigarettes per day (CPD) for a mean of 13 years before vaping, which was

	initiated a median of 13-24 months earlier. Smoking declined to 6-10 CPD currently, with 79% reporting decreased and 3% reporting increased smoking. 69% reported daily vaping, with 57% reporting vaping "continuously" or at least 30 times/ day. CPD was inversely related to both weekly (days) and daily (uses) vaping frequency (ps < .001), but positively associated with nicotine dosage of the e-cigarette solution (p < .001). Duration of vaping was not associated with cigarette use (CPD, time to first cigarette) or dependence, but was positively associated with a measure of e-cigarette dependence (p < .001). Additional results will be reported. Conclusion(s): The current cross-sectional findings suggest that vaping may reduce and partially replace cigarette use. However, e-cigarette dependence may increase over time without further reduction in smoking among those who maintain dual use. Future results from this longitudinal study should clarify use patterns, with implications for both tobacco intervention and policy.
2700	G Brozek, M Jankowski, A Shpakou, M Poznanski, L Klimackaya, J Loginovic, J Gereova, M Rachel, I Naumau, K Kornicki, Z Kristufkova, K Bielewicz, J Zejda (2018///) The prevalence of E-cigarette and cigarette smoking among students in central and eastern Europe-preliminary results of YUPESS study. European Respiratory Journal; 52<br><br>**Keywords:**<br>adult,Belarus,*cigarette smoking,controlled study,cross-sectional study,*electronic cigarette,female, habit,human,*Lithuania,major clinical study,male,monitoring,*non-medical student,Poland,*preliminary data,*prevalence,questionnaire,Slovakia,smoking,you<br><br>**Abstract:** Introduction: The prevalence of e-cigarette and cigarette smoking among the young adults pose a very significant epidemiological issue. Aim(s): The aim of the study was to assess the prevalence of e-cigarette and cigarette smoking usage among students in Europe. Method(s): The study was carried out as a part of international multi-centre cross-sectional study - YoUng People E-Smoking Study (YUPESS). A population based survey was performed among students from five countries: Belarus (B), Lithuania (L), Poland (P), Russia (R) and Slovakia (S). We used previously validated, created for the purpose of the study, questionnaire focused on e-cigarette and cigarette smoking habits. Result(s): The study population were 11974 students (6797 medical, 5177 non-medical) aged 20.7+/-2.4 y-rs: the response rate was 73%. Ever e-cigarette use declared 43.1% of the respondents (B:42.7%; L:56.7%; P:44.1%; R:33.4%; S:34.4%; p<0.001); smoking ever traditional tobacco by 65.5% (B:55%; L:73%; P:72%; R:59%; S:76.5%; p<0.001); tobacco smoking regularly by 12.4% (B:10.3%; L:14.9%; P:13.5%; R:12.3%; S:13.1%; p<0.001), e-cigarettes only by 1.04% (B:0.72%; L:1.42%; P:1.13%; R:1.4%; S:0.98%; p<0.001) and 1.86% (B:1.98%; L:2.13%; P:1.56%; R:2.64%; S:1.54%; p<0.001) were dual users. The overall frequency of e-smokers was 2.9%, (B:2.7%; L:3.55%; P:2.69%; R:4.04%; S:2.52% (p<0.001), with no difference (p=0.2) among medical and non-medical students. Conclusion(s): Among students cigarettes are more popular than e-cigarettes. The highest prevalence of e-cigarette usage was observed in Russia and Lithuania. The frequency of using e-cigarettes requires constant monitoring.
2701	G M Brozek, M Jankowski, J A Lawson, A Shpakou, M Poznanski, T M Zielonka, L Klimatckaia, Y Loginovich, M Rachel, J Gereova, J Golonko, I Naumau, K Kornicki, P Peplowska, V Kovalevskiy, A Raskiliene, K Bielewicz, Z Kristufkova, R Mroz, P Majek, J Lubanski, D Kaleta, J Pinkas, J E Zejda (2019///) The prevalence of cigarette and e-cigarette smoking among students in central and eastern europe-results of the YUPESS study. International Journal of Environmental Research and Public Health; 16<br><br>**Keywords:**<br>adult,article,Belarus,controlled study,disease course,female,habit,human,*Lithuania,major clinical study, male,nonsmoker,perception,Poland,*prevalence,questionnaire,Slovakia,*university student,*vaping, young adult<br><br>**Abstract:** Electronic cigarettes (e-cigarettes) are an alternative to traditional tobacco cigarette smoking. The aim of this study was to assess the prevalence of cigarette smoking and e-cigarette use among university students from Central and Eastern Europe and to investigate personal characteristics

	associated with cigarette and e-cigarette smoking. A questionnaire-based cross-sectional survey was performed between 2017-2018 among university students in five European countries: Belarus, Lithuania, Poland, Russia, and Slovakia. The questionnaire included 46 questions related to the frequency and habits of traditional cigarettes and e-cigarettes use. Completed questionnaires were obtained from 14,352 students (8800 medical; aged 20.9 +/- 2.4 years) with an overall response rate of 72.2%. Two-thirds of the respondents had smoked a traditional tobacco cigarette and 43.7% had used an e-cigarette. Overall current smoking status included 12.3% traditional cigarette smokers, 1.1% e-cigarette users, and 1.8% were dual users with the remainder being non-smokers. Smoking status differed between the research centres (p < 0.001). Females were less likely to try either cigarettes (OR = 0.83) or e-cigarettes (OR = 0.62) and were less likely to be current cigarette (OR = 0.64), e-cigarette (OR = 0.34), or dual users (OR = 0.33) than males. Perception of e-cigarettes significantly differed between smokers and non-smokers (p < 0.001). Among university students, cigarettes are more popular than e-cigarettes.Copyright © 2019 by the authors. Licensee MDPI, Basel, Switzerland.
2709	A Buu, Y H Hu, S W Wong, H C Lin (2019///) Comparing American college and noncollege young adults on e-cigarette use patterns including polysubstance use and reasons for using e-cigarettes. Journal of American college health : J of ACH;<br><br>**Keywords:**<br>adult,alcohol consumption,article,cannabis use,chi square test,*college student,controlled study, *electronic cigarette,female,human,major clinical study,male,prevalence,social environment,tobacco, workplace,young adult,*alcohol,*cannabis<br><br>**Abstract:** OBJECTIVE: Existing literature on young adults' e-cigarette and polysubstance use focused on college students. This study examined the differences between college and noncollege groups on prevalence and patterns of e-cigarette and other substance use using data from a national survey. PARTICIPANTS: Adults aged 18-24 from the 2013-2014 Population Assessment of Tobacco and Health Study (n=6,608). METHOD(S): Independent sample t-tests and Chi-square tests were conducted to examine group differences. RESULT(S): Noncollege young adults had higher prevalence of cigarette, e-cigarette, and marijuana use; college students had higher prevalence of alcohol use. Among current e-cigarette users, college students had higher prevalence of polysubstance use of alcohol and marijuana. College students used e-cigarettes for socializing purposes more. CONCLUSION(S): Differences in prevalence and patterns of e-cigarette and other substance use between college and noncollege groups exist. Future interventions should target the social context of college life and reach out to noncollege young adults in workplaces.
2711	B Campbell, D Yip, T Le, N Gubner, J Guydish (2019///) Relationship between Tobacco Use and Health-Related Quality of Life (HRQoL) among Clients in Substance Use Disorders Treatment. Journal of Psychoactive Drugs; 51<br><br>**Keywords:**<br>adult,article,cost,depression,*drug dependence,education,ethnicity,female,gender,health survey,human, major clinical study,male,marriage,mental disease,prevalence,*quality of life,questionnaire,smoking, *smoking cessation,tobacco dependence,electronic cig<br><br>**Abstract:** We examined relationships of smoking status and tobacco-related variables with health-related quality of life (HRQoL), a metric of disease burden, among clients in substance use disorders (SUDs) treatment. Participants (N = 2,068; 46.6% female) completed surveys reporting demographics, smoking status, and past-month days they experienced physical and/or mental health distress. Smokers (n = 1, 596; 77.2% of sample) answered questions on tobacco-related variables. Multinomial regression models assessed relationships between tobacco-related variables (smoking status, nicotine dependence, menthol smoking, electronic-cigarette use, health concerns, and cost as reasons affecting reducing/quitting smoking, past and future quit attempts) with HRQol in four categories (good health, physical health

	distress, mental health distress, or both physical and mental health distress). Current smokers were more likely than former smokers to report frequent physical and mental health distress than good health (OR = 1.97, 95% CI = 1.16, 3.34), as were smokers with higher nicotine dependence (OR = 1.18, 95% CI = 1.03, 1.35). Smokers reporting both frequent physical and mental health distress were more sensitive to cigarettes' cost (OR = 1.56, 95% CI = 1.06, 2.29), and less likely to use e-cigarettes (OR = 0.59, 95% CI = 0.38, 0.94). Findings of poor HRQoL among nicotine-dependent smokers with additional SUDs strengthen the imperative to provide smoking cessation interventions in addictions treatment.Copyright © 2018, © 2018 Taylor & Francis Group, LLC.
2726	J L Carwile, A F Fleisch, K Young, K A Ahrens (2019///) Electronic Cigarette Use in US Households with Children: The "new" Secondhand Smoke. JAMA Pediatrics; 173<br><br>**Keywords:**<br>aerosol,asthmatic state/et [Etiology],behavioral risk factor surveillance system,District of Columbia, human,letter,*passive smoking,prediction,prevalence,priority journal,telephone interview,*electronic cigarette<br><br>**Abstract:**
2729	A C Catalano, N E Cranswick, J Grindlay, M Creati, M H Danchin, J Robinson, N Williams, A Gwee (2019///) Unintended consequences of a cautious approach to e-cigarette laws. Medical Journal of Australia; 211<br><br>**Keywords:**<br>accident,Australia,brain hypoxia,child death,concentration (parameter),heart arrest,human,intoxication, *law,lethal dose,letter,morbidity,mortality,vomiting,nicotine/to [Drug Toxicity],nicotine/pv [Special Situation for Pharmacovigilance],*electronic cig<br><br>**Abstract:**
2742	G Chan, J Leung, C Gartner, H H Yong, R Borland, W Hall (2019///) Correlates of electronic cigarette use in the general population and among smokers in Australia - Findings from a nationally representative survey. Addictive Behaviors; 95<br><br>**Keywords:**<br>adult,age distribution,alcohol consumption,article,attitude to health,*Australia,controlled study,distress syndrome,female,*health survey,high risk behavior,human,male,sex difference,*smoking,smoking cessation,tobacco use,vaping,*electronic cigarette<br><br>**Abstract:** Aims: In Australia, sales of nicotine containing electronic cigarettes (also known as e-cigarette) are banned unless approved as a therapeutic good. The aims of this study were to estimate the prevalence of e-cigarette use and its correlates in Australia using a nationally representative survey. Method(s): We analysed data from the largest drug use survey in Australia (the National Drug Strategy Household Survey [NDSHS]; N = 22,354). Result(s): We estimated that 227,000 Australians (1.2% of the population) were current e-cigarettes users, and 97,000 (0.5%) used them daily. Individuals who were male, younger, had higher level of psychological distress, and smoked were more likely to use electronic cigarettes. Among smokers, an intention to quit and reduction in smoking was associated with experimentation and daily use of e-cigarette, but not with occasional use. Recent quitting was associated with daily use. Conclusion(s): Overall, the prevalence of e-cigarette use was low in 2016 in Australia. Smoking status was the strongest correlates of e-cigarette use. Patterns of vaping were differentialy associated with an intention to quit smoking, smoking reduction and recent quitting. Some smokers may attempt to use e-cigarettes to cut-down their tobacco use, and those who vaped daily were mostly likely to be recent quitters.Copyright © 2019

2743	C Y Chang, C S Ho (2019///) E-cigarette exposure among high schools students in Taiwan. Journal of Substance Use; 24<br><br>**Keywords:**<br>adolescent,article,female,*high school student,human,knowledge,male,passive smoking,personal experience,prevalence,priority journal,Taiwan,*electronic cigarette<br><br>**Abstract:** Objective: This study assessed the prevalence and determinants of e-cigarette experience among junior and senior high school students in Taiwan. Method(s): Data from the General Tobacco Hazard Proficiency Test were analyzed for n = 7,599 respondents during 2017. The knowledge and determinants of e-cigarettes exposure experience were assessed. The chi square, ANOVA test, and logistic regression were performed for inferential statistical analysis. Result(s): A total of 30.5% of students had seen someone trying e-cigarettes; 70.6% of the students agree or strongly agree having clear knowledge about the shape and hazards of e-cigarettes. Among the students who had e-cigarettes exposure experience, adolescent boys were more likely than female students (aOR 1.315, 95% CI 1.186-1.457), vocational high school students have the highest rate (34.3%) of the schools (aOR 1.391, 95% CI 1.253-1.543), and students who had smoking family members were higher than those without smoking family members (aOR 2.220, 95% CI 1.978-2.491). Moreover, experiencing e-cigarettes was more likely among the students who had been exposed to second-hand smoke daily (aOR 2.188, 95% CI 1.864-2.568). Conclusion(s): These findings underscore the need to evaluate the potential risk of e-cigarette use in adolescents, and develop effective intervention programs for e-cigarette regulation.Copyright © 2018, © 2018 Taylor & Francis Group, LLC.
2745	L Chang, P Chen, C Hsu, Y Lee (2018///) Electronic Cigarettes and Intention To Smoke Cigarettes Among Adolescents in Taiwan. Value in Health; 21<br><br>**Keywords:**<br>adolescent,*cigarette smoking,controlled study,cross-sectional study,*electronic cigarette,female,friend, human,juvenile,major clinical study,male,maternal smoking,monitoring,random sample,risk factor, smokeless tobacco,*Taiwan,*tobacco,conference abstrac<br><br>**Abstract:** Objectives: Electronic cigarettes (e-cigarettes) use among youth has continued to increase worldwide. One concern regarding relationship between e-cigarette use and future intention to cigarette smoking also has raised. We investigated the association between electronic cigarettes (e-cigarettes) use and future intention to cigarette smoking among adolescents who had never smoked cigarettes. Method(s): We conducted a pooled cross-sectional study based on data drawn from a nationally representative random sample in the 2014-2016 Taiwan Global Youth Tobacco Survey (GYTS). After excluding adolescents who had missing information on current e-cigarette use or ever smoke cigarette, this study included 4,033,242 adolescents. Multivariable logistic regression models were used to examine the association between e-cigarette use and susceptibility to cigarette smoking. Result(s): A total of 1.11% of Taiwan non-smoking adolescents were current (past 30-day) e-cigarettes users. Adolescents who were current e-cigarette users had higher odds of susceptibility to cigarette smoking (adjusted OR= 1.78) compared to those that were not current e-cigarette users. Besides that, we also identified year 2016 (adjusted OR=1.08), male (adjusted OR=1.45), monthly allowance more than NTD 3500 (adjusted OR=1.21), smoking mother (adjusted OR=1.34), some or most friends smoking (adjusted OR=2.43 and 5.48), use of other tobacco products (adjusted OR=1.63), use of smokeless tobacco (adjusted OR=2.31), free cigarette (adjusted OR=4.88) as risk factors for future intention to cigarette smoking. Conclusion(s): E-cigarette use was strongly associated with future intention to cigarette smoking among adolescents. Continuous preventing and monitoring use of e-cigarette among adolescents are needed.Copyright © 2018
2747	S Chapman, D Bareham, W Maziak (2019///) The gateway effect of e-cigarettes: Reflections on main criticisms. Nicotine and Tobacco Research; 21

	**Keywords:**<br>adolescent smoking,*cigarette smoking,health behavior,health hazard,health promotion,human,note, peer acceptance,priority journal,public health,social marketing,tobacco dependence,vaping,*electronic cigarette<br><br>**Abstract:**
2756	J Chen, S Y Ho, L T Leung, M P Wang, T H Lam (2019///) School-level electronic cigarette use prevalence and student-level tobacco use intention and behaviours. Scientific reports; 9<br><br>**Keywords:**<br>adolescent,article,child,*cigarette smoking,controlled study,*electronic cigarette,female,high school, Hong Kong,human,major clinical study,male,*prevalence,school child,smoking,*student<br><br>**Abstract:** Prevalent electronic cigarette (e-cigarette) use in schools may undermine tobacco denormalisation, and thus increase tobacco use in students. We investigated the associations of school-level e-cigarette use prevalence with student-level intention and behaviours related to e-cigarettes, cigarettes, and other tobacco products. In a 2014-15 school-based cross-sectional survey of 41035 secondary school students (grade 7-12; age 11-18 years) in Hong Kong, information was collected on the use of e-cigarettes, cigarettes, and non-cigarette tobacco products (NCTPs), susceptibility to e-cigarette and cigarette use, intention to quit cigarette smoking, and sociodemographic characteristics. The adjusted odds ratio (AOR) of e-cigarette use susceptibility in relation to high (vs low) school-level e-cigarette use prevalence was 1.40 (95% CI 1.05-1.87) in never e-cigarette users. The AORs of cigarette smoking susceptibility in relation to medium and high (vs low) school-level e-cigarette use prevalence were 1.24 (1.01-1.52) and 1.34 (1.02-1.75), respectively, in never cigarette smokers. School-level e-cigarette use prevalence was associated with ever and past 30-day cigarette smoking, but not with intention to quit (in past 30-day cigarette smokers) or past 30-day NCTP use. The findings highlight the importance of strictly banning e-cigarettes in schools, and add to the evidence that prevalent e-cigarette use in adolescents may increase cigarette smoking prevalence.
2760	P C Chen, L C Chang, C H Ms, Y C Lee (2019///) Dual use of e-cigarettes and traditional cigarettes among adolescents in Taiwan, 2014-2016. Nicotine and Tobacco Research; 21<br><br>**Keywords:**<br>adolescent,adult,advertising,article,child,*cigarette smoking,educational status,female,government, health survey,human,income,major clinical study,male,parent,peer group,priority journal,risk factor, school child,sex difference,smoking cessation program,<br><br>**Abstract:** Introduction: We investigated the use of electronic cigarettes (e-cigarettes) with traditional cigarettes among adolescents during 2014 to 2016 to identify risk factors for using e-cigarettes only, traditional cigarettes only, or both products. Method(s): We used cross-sectional data from the Taiwan Global Youth Tobacco Survey, (conducted over a 3-year period by the Health Promotion Administration, Ministry of Health and Welfare, Taiwan), which is representative of tobacco use among adolescents aged 12-18 years.The outcome variable was smoking behavior. Dependent variables included gender, grade, monthly income/ allowance, parents' educational level, parents' smoking status, close friends' smoking status, use of other tobacco products, contact with cigarette/e-cigarette advertisements, and access to free cigarettes/e-cigarettes. Multinomial regression identify factors influencing the smoking behaviors of adolescents, as manifested in the use of traditional cigarettes only, e-cigarettes only, e-cigarettes with traditional cigarettes, and nonsmoking. Result(s): When weighted to the population, the sample included 1 723 150 adolescents in 2014, 1 691 568 adolescents in 2015, and 1 627 216 adolescents in 2016. The rates averaged over three years were as follows: nonsmoking (91.6%), traditional cigarettes only (5.4%), e-cigarettes only (1.5%), and dual usage (1.6%). Among adolescents in Taiwan, the following were risk

	factors for dual use: male, older, high monthly allowance, smoking parents, smoking friends, use of other tobacco products, contact with cigarette advertisements, and access to free cigarettes. Conclusion(s): Our results revealed an increase in the number of adolescents using e-cigarettes with traditional cigarettes. We recommend that the government continue smoking cessation programs while maintaining control over advertisements and promotions for tobacco products. Implications:This is the first study to examine the dual use of e-cigarettes and traditional cigarettes among adolescents in Taiwan. This study identified the risk factors of using traditional cigarettes only, using e-cigarettes only, and the dual use of e-cigarettes and traditional cigarettes, with nonsmokers used as a reference group. This study examined the relationship between exposure to cigarette promotions and the use of various tobacco products including the dual use of e-cigarettes and traditional cigarettes, both of which have been disregarded in previous studies.Copyright © The Author(s) 2018. Published by Oxford University Press on behalf of the Society for Research on Nicotine and Tobacco. All rights reserved.
2763	J C Chen-Sankey, G Kong, K Choi (2019///) Perceived ease of flavored e-cigarette use and e-cigarette use progression among youth never tobacco users. PLoS ONE; 14<br><br>**Keywords:**<br>adolescent,article,clinical outcome,cohort analysis,comparative study,controlled study,female,*flavor, follow up,health survey,human,longitudinal study,male,outcome assessment,prospective study,sample size,*smoking reduction,social aspect,social marketin<br><br>**Abstract:** Background There is an increased need to understand how e-cigarette flavors may contribute to e-cigarette uptake and use among youth. We examined the relationship between perceived ease of flavored e-cigarette use and e-cigarette use susceptibility and progression among a nationally representative sample of U.S. youth never tobacco users. Methods The wave 1 (2013-2014) and wave 2 (2014-2015) surveys of PATH Study were used. Youth never tobacco users (ages 12-17) who reported whether flavored e-cigarettes were easier to use than unflavored e-cigarettes at wave 1 (n = 6,983) were included in the study. Multivariable logistic regressions were used to examine the associations between perceived ease of using flavored e-cigarettes (wave 1) and e-cigarette use outcomes including e-cigarette use susceptibility (wave 1) and e-cigarette initiation and past-30-day use (wave 2). Results Overall, 21.2% of the sample perceived flavored e-cigarettes easier to use than unflavored e-cigarettes; and 28.9% of the sample were susceptible to using e-cigarettes at wave 1, and 7.5% and 2.0% initiated e-cigarettes and used e-cigarettes in the past 30 days at wave 2, respectively. Among those who perceived flavored e-cigarettes easier to use, 41.0% were susceptible to using e-cigarettes at wave 1, and 10.6% and 3.4% initiated and used e-cigarettes in the past 30 days at wave 2, respectively. Perceiving flavored e-cigarettes as easier to use than unflavored e-cigarettes at wave 1 was positively associated with e-cigarette use susceptibility at wave 1 (AOR = 1.43, CI = 1.21, 1.69), and e-cigarette initiation (AOR = 1.32, CI = 1.12, 1.67) and past-30-day use (AOR = 1.25, CI = 1.10, 2.47) at wave 2. Conclusions Perceiving flavored e-cigarettes as easier to use than unflavored e-cigarettes may lead to e-cigarette use progression among youth never tobacco users. Determining the factors (including e-cigarette marketing and specific e-cigarette flavors) that lead to perceived ease of using flavored e-cigarettes would inform efforts to prevent and curb youth e-cigarette use.Copyright © 2019 Public Library of Science. All Rights Reserved.
2767	A G Cole, A Chaurasia, R D Kennedy, S T Leatherdale (2019///) Identifying behavioural characteristics of tobacco product and e-cigarette use clusters: A repeat cross-sectional analysis. Addictive Behaviors; 90<br><br>**Keywords:**<br>article,binge drinking,Canadian,cannabis use,cohort analysis,cross-sectional study,environmental factor, female,human,male,prevalence,smoking cessation,substance use,*tobacco,cannabis,*electronic cigarette<br><br>**Abstract:** Background: Youth may use a variety of tobacco products and e-cigarettes. However, there is a lack of evidence for the combinations of tobacco products and e-cigarettes commonly used by youth in

	Canada and whether youth from different usage classes share similar characteristics. Method(s): A cohort of 9th grade students from Ontario, Canada was identified at baseline (2013-14) of the COMPASS study (n = 4651). Classes of youth that currently use similar combinations of tobacco products and e-cigarettes were identified at baseline, one (FY1) and two years later (FY2) using latent class analysis. Multinomial logistic regression models identified demographic and behavioural characteristics (e.g., environmental influences, substance use behaviours, etc.) of youth in current tobacco and e-cigarette use classes relative to youth in non-current use classes. Result(s): At baseline, a three-class model was identified as best, while a four-class model was identified at FY1 and FY2. A non-current use group and an all-product use group were identified every year. Students that reported having friends that smoked cigarettes, binge drinking, and using marijuana were more likely to be classified into a current use class. Conclusion(s): Tobacco cigarettes were more likely to be used with other products than on their own. An all-product use group was identified across all three survey waves and the prevalence of this group increased over time. Given that many youth in this study used more than one tobacco product or e-cigarette and commonly reported binge drinking and using marijuana, prevention and cessation activities should address the use of multiple products.Copyright © 2018 Elsevier Ltd
2768	B Y Cho (2019///) Parent's Depression as a Moderator of the Association Between Offspring's Depressive Symptoms and Use of Combustible Cigarettes and Electronic Vapor Products. The journal of behavioral health services & research;<br><br>**Keywords:**<br>article,*cigarette,*depression,human,*progeny,*vapor<br><br>**Abstract:**
2771	H J Cho, L M Dutra, S A Glantz (2018///) Differences in Adolescent E-cigarette and Cigarette Prevalence in Two Policy Environments: South Korea and the United States. Nicotine and Tobacco Research; 20<br><br>**Keywords:**<br>adolescent,adult,article,child,controlled study,*health care policy,health hazard,health survey,high school student,human,Korean (people),*prevalence,priority journal,questionnaire,school child,*South Korea, *United States,*cigarette,*electronic cigarett<br><br>**Abstract:** Introduction: In the context of different regulatory environments, different patterns of e-cigarette use have emerged among adolescents worldwide. The United States and South Korea are two examples, the latter of which has maintained much more extensive regulation of e-cigarettes. Method(s): This analysis compares the prevalence of e-cigarette and conventional cigarette use between 2011 and 2015 from the Korean Youth Risk Behavior Web-based Survey and the U.S. NationalYouthTobacco Survey, both nationally representative samples of middle and high school students that use similar questions. Result(s): E-cigarette prevalence (past 30 days) among South Korean adolescents decreased from 4.7% in 2011 (95% confidence interval [CI]: 4.4-5.0) to 4.0% (3.7-4.3) in 2015 but increased dramatically among US adolescents from 0.9% (0.7-1.2) to 11.2% (9.9-12.7). Cigarette prevalence (past 30 days) decreased in South Korea from 12.1% (11.6-12.7) to 7.8% (CI: 7.3-8.3) and in the United States from 11.1% (9.5-12.6) to 6.1% (5.1-7.3). Combined prevalence of cigarette and e-cigarette use (adjusting for dual users) decreased in South Korea from 13.2% (12.7-13.8) to 8.5% (8.0-9.1) but increased in the United States from 11.3% (9.7-12.9) to 14.0% (12.4-15.7). Conclusion(s): In South Korea, where e-cigarettes are extensively regulated, adolescent e-cigarette use remained stable at a low level, whereas in the United States, where e-cigarette regulation has been limited, e-cigarette use increased. Combined e-cigarette plus cigarette use declined in South Korea whereas it increased in the United States.The restrictive policies in South Korea likely contributed to lower overall product use. Implications:This paper compared the strength of regulation of South Korea and the United States with the prevalence of adolescents' e-cigarette and cigarette use and suggests that more restrictive policies likely to contribute to lower e-cigarette use and overall use of e-cigarette and cigarette.Copyright © The Author(s) 2017. Published by

	Oxford University Press on behalf of the Society for Research on Nicotine and Tobacco. All rights reserved.
2772	J Cho, N I Goldenson, M D Stone, R McConnell, J L Barrington-Trimis, C P Chou, S Y Sussman, N R Riggs, A M Leventhal (2018///) Characterizing polytobacco use trajectories and their associations with substance use and mental health across mid-adolescence. Nicotine and Tobacco Research; 20<br><br>**Keywords:**<br>*adolescence,adolescent,alcohol consumption,anxiety disorder,article,attention deficit disorder, California,cannabis use,cigarette smoking,controlled study,depression,female,follow up,health survey, high school student,human,longitudinal study,male,*menta<br><br>**Abstract:** Background Polytobacco product use is suspected to be common, dynamic across time, and increase risk for adverse behavioral outcomes. We statistically modeled characteristic types of polytobacco use trajectories during mid-adolescence and tested their prospective association with substance use and mental health problems. Methods Adolescents (N = 3393) in Los Angeles, CA, were surveyed semiannually from 9th to 11th grade. Past 6-month combustible cigarette, e-cigarette, or hookah use (yes/no) over four assessments were analyzed using parallel growth mixture modeling to identify a parsimonious set of polytobacco use trajectories. A tobacco product use trajectory group was used to predict substance use and mental health at the fifth assessment. Results Three profiles were identified: (1) tobacco nonusers (N = 2291, 67.5%) with the lowest use prevalence (<3%) of all products across all timepoints; (2) polyproduct users (N = 920, 27.1%) with moderate use prevalence of each product (8-35%) that escalated for combustible cigarettes but decreased for e-cigarettes and hookah across time; and (3) chronic polyproduct users (N = 182, 5.4%) with high prevalence of each product use (38-86%) that escalated for combustible cigarettes and e-cigarettes. Nonusers, polyproduct users, and chronic polyproduct users reported successively higher alcohol, marijuana, and illicit drug use and ADHD at the final follow-up, respectively. Both tobacco using groups (vs. nonusers) reported greater odds of depression and anxiety at the final follow-up but did not differ from each other. Conclusions Adolescent polytobacco use may involve a common moderate risk trajectory and a less common high-risk chronic trajectory. Both trajectories predict substance use and mental health symptomology. Implications Variation in use and co-use of combustible cigarette, e-cigarette, and hookah use in mid-adolescence can be parsimoniously characterized by a small set common trajectory profiles in which polyproduct use are predominant patterns of tobacco product use, which predict adverse behavioral outcomes. Prevention and policy addressing polytobacco use (relative to single product use) may be optimal tobacco control strategies for youth, which may in turn prevent other forms of substance use and mental health problems.Copyright © The Author(s) 2018. Published by Oxford University Press on behalf of the Society for Research on Nicotine and Tobacco.
2796	M Conner, S Grogan, R Simms-Ellis, K Scholtens, B Sykes-Muskett, L Cowap, R Lawton, C J Armitage, D Meads, L Schmitt, C Torgerson, R West, K Siddiqi (2019///) Patterns and predictors of e-cigarette, cigarette and dual use uptake in UK adolescents: Evidence from a 24-month Prospective Study. Addiction (Abingdon, England);<br><br>**Keywords:**<br>adolescent,article,*cigarette smoking,controlled study,*electronic cigarette,England,ethnicity,female, follow up,friend,gender,human,impulsiveness,major clinical study,male,prevalence,*prospective study, smoke,social status<br><br>**Abstract:** BACKGROUND AND AIMS: To assess prevalence and predictors of e-cigarettes/cigarettes patterns of use in adolescents in England. DESIGN: Prospective study with 24-month follow-up of e-cigarette/cigarette ever/regular use with data from an intervention evaluation. SETTING: Forty-five schools in England (Staffordshire and Yorkshire). PARTICIPANTS: 3,210 adolescents who, at baseline, were aged 13-14 years and had never used e-cigarettes/cigarettes. MEASUREMENTS: Based on e-

	cigarette/cigarette ever use at follow-up, six groups were created: (a) never user, (b) e-cigarette only, (c) cigarette only, (d) dual use - order of use unclear, (e) dual use - e-cigarettes used first, (f) dual use - cigarettes used first. Baseline measures were: gender, ethnicity, socioeconomic status, impulsivity, family plus friend smoking, and smoking-related beliefs (attitude and perceived behavioural control). FINDINGS: In groups (a) through (f), there were 71.5%, 13.3%, 3.3%, 5.7%, 2.9%, and 3.4% adolescents, respectively. Among groups using cigarettes, regular smoking was more prevalent in group (f) (dual use - cigarettes used first) (17.6%, 95%CI 10.4, 24.8) than in groups (c), (d) and (e) combined (7.3%, 95%CI 4.7, 9.9). Among groups using e-cigarettes, regular use was less prevalent in group (b) (e-cigarette only) (1.9%, 95%CI 0.6, 3.2) than in groups (d), (e) and (f) combined (12.2%, 95%CI 8.9, 15.5). Higher impulsivity plus friends and family smoking were predictive of being in groups (b) to (f) compared with group (a) (never users). Males were more likely to be in group (b) compared to group (a); females were more likely to be in groups (c) to (f) compared to group (a). CONCLUSION(S): Regular use of e-cigarettes/cigarettes varies across groups defined by ever use of e-cigarettes/cigarettes. Interventions targeted at tackling impulsivity or adolescents whose friends and family members smoke may represent fruitful avenues for future research.Copyright This article is protected by copyright. All rights reserved.
2799	Gartner Coral (2018///) How can we protect youth from putative vaping gateway effects without denying smokers a less harmful option?. Addiction (Abingdon, England); 113<br><br>**Keywords:**<br>Adolescent,*Cigarette Smoking,*Electronic Nicotine Delivery Systems,Humans,Smokers,*Smoking Cessation,*Vaping<br><br>**Abstract:**
2810	M Creamer, K Case, A Loukas, M Cooper, C L Perry (2019///) Patterns of sustained e-cigarette use in a sample of young adults. Addictive Behaviors; 92<br><br>**Keywords:**<br>adult,article,controlled study,demography,female,Hispanic,human,longitudinal study,male,regression analysis,symptom,Texas,tobacco dependence,tobacco use,young adult,disposable equipment,*electronic cigarette<br><br>**Abstract:** Introduction: E-cigarette use and devices are rapidly changing, yet there is not much scientific evidence examining these changes over time. The purpose of this study is to describe patterns of e-cigarette use in a sample of sustained (i.e., reporting past 30-day e-cigarette use at every wave) e-cigarette users over a two-year period. Method(s): Data are drawn from five waves of the Project M-PACT cohort. Analyses are limited to those reporting past 30-day e-cigarette use at each wave (n = 75). Mixed effects regressions were conducted for the following dependent variables: device type, number of days used, combustible tobacco product use, and symptoms of nicotine dependence. Each model used survey wave as the time variable, and controlled for sociodemographic variables. Result(s): Among sustained users, the majority reported using a rechargeable device. The average number of days used was about 2 for disposable devices and 14 for rechargeable devices (p <.0001). The odds of combustible tobacco product use decreased over time (AOR = 0.71; 95% CI 0.57-0.89), while symptoms of e-cigarette nicotine dependence increased over time (beta = 0.07 SE = 0.03, p-value =.02). For both e-cigarette device types, there were no changes in device type or number of days used over time. Conclusion(s): This is one of the first studies to look at changes in e-cigarette use, including symptoms of dependence and number of days used over a two-year period. This brief report extends the current literature by examining more than the prevalence and frequency of e-cigarette use.Copyright © 2018 Elsevier Ltd
2813	Martinez Cristina, Baena Antoni, Castellano Yolanda, Fu Marcela, Margalef Merce, Tigova Olena, Feliu Ariadna, Laroussy Kenza, Galimany Jordi, Puig Montse, Bueno Albert, Lopez Antonio, Fernandez Esteve (2019///) Prevalence and determinants of tobacco, e-cigarettes, and cannabis use among nursing

	students: A multicenter cross-sectional study. Nurse education today; 74<br><br>**Keywords:**<br>Adolescent,Adult,Cross-Sectional Studies,*Electronic Nicotine Delivery Systems/sn [Statistics & Numerical Data],Female,Humans,Male,*Marijuana Smoking/ep [Epidemiology],Prevalence,Risk Factors, *Smoking/ep [Epidemiology],Spain/ep [Epidemiology],*Students,<br><br>**Abstract:** BACKGROUND: Nurses are important agents in public health, which includes being active in tobacco control. Studies show that nurses who smoke are less inclined to offer smoking cessation aid. Nursing students, as the future labor force of nursing, are one of the key groups to monitor., OBJECTIVES: To identify the prevalence and determinants of use of several tobacco products, e-cigarettes, and cannabis among nursing students in Catalonia., DESIGN: Cross-sectional multicenter study., SETTINGS: 15 university nursing schools in Catalonia (Spain) in 2015-2016., PARTICIPANTS: Nursing students attending class at the day of the survey., METHODS: An anonymous, self-administered questionnaire based on the Global Health Professional Survey was designed. The questions included information on consumption of several tobacco products (manufactured cigarettes, roll your own cigarettes, etc.), e-cigarettes, and cannabis. We estimated the prevalence of use (%) and computed multilevel logistic regressions models, at two levels, to calculate the odds ratios (OR) and their corresponding 95% confidence intervals (CI), adjusting for several individual sociodemographic variables and the nursing school as a grouping variable., RESULTS: 4381 students participated in the study (57.2% of Nursing students in Catalonia at the time of the survey). 29.7% (95%CI: 27.2-32.2) were smokers (18.4% daily and 11.3% occasionally). 66.4% smoked manufactured cigarettes, 47.0% roll your own cigarettes, 10.0% waterpipe, and 0.4% e-cigarettes. The main predictors of smoking were: being >=25years (OR=2.57, 95%CI: 2.03-3.26) and belonging to other Spanish regions (OR=1.82, 95%CI: 1.30-2.54). 71.5% had low nicotine dependence as defined by the Heavy Smoking Index. Among 11.5% (95%CI: 10.6-12.4) of students used cannabis (daily or occasionally), and men presented higher odds of use (OR=2.81, 95%CI: 2.11-3.73) than women., CONCLUSIONS: Tobacco and cannabis use is high among nursing students. It is necessary to carry out early tobacco and cannabis cessation programs among young nursing students. Copyright © 2018 Elsevier Ltd. All rights reserved.
2815	K Crosby (2019///) How the Food and Drug Administration Convinced Teens to Rethink Their Relationship With Cigarettes. American Journal of Preventive Medicine; 56<br><br>**Keywords:**<br>*adolescence,*behavior change,coping behavior,craving,direct-to-consumer advertizing,editorial,*food and drug administration,high risk population,human,*public health campaign,tobacco dependence, tobacco use,underage drinking,*vaping,nicotine<br><br>**Abstract:**
2820	H Dai, J Hao (2019///) Flavored Tobacco Use Among U.S. Adults by Age Group: 2013-2014. Substance use & misuse; 54<br><br>**Keywords:**<br>adult,aged,article,cigar,*cigarette smoking,controlled study,electronic cigarette,female,groups by age, hookah,human,major clinical study,male,prevalence,*smoking,tobacco,young adult,menthol<br><br>**Abstract:** OBJECTIVE: This study sought to assess patterns of flavored tobacco product (FTP) use and their associations with (1) susceptibility to smoke cigarettes among young adults aged 18-29 who have never smoked; (2) quit attempts in the past 12months among current cigarette users by age group (18-29, 30-49, and 50+). METHOD(S): We used the 2013-2014 National Adult Tobacco Survey (n=75,233) to estimate the prevalence of single-, dual-, and poly-use of FTP (menthol cigarettes, e-cigarettes, cigars, regular pipes/hookahs, and smokeless). Logistic regression models were performed to examine the associations

	between FTP use and smoking behaviors. RESULT(S): Among current tobacco users, young adults aged 18-29 (72.7%) had a higher prevalence of FTP use than other age groups (30-49: 51.7% and 50+: 37.9%). For FTP use within each age group, dual- and poly-FTP use was most popular among young adults aged 18-29 and menthol cigarette use was most popular among older adults aged 50+. Compared with not using any tobacco products, FTP use was associated with higher odds of susceptibility to cigarette smoking among young adult never smokers (single FTP use: aOR =5.5, CI: 4.2-7.3; dual/poly FTP use: aOR =10.7, CI: 6.2-18.4). FTP use was not associated with quit attempts among current cigarettes users aged under 50, while dual/poly-FTP use was associated with higher odds of quit attempts among current cigarette users aged 50+(aOR =1.7, CI: 1.2-2.4). CONCLUSION(S): This study shows the heterogeneity of FTP use among adults by age group and demonstrates its associations with susceptibility to cigarette smoking among young adult never smokers.
2822	S Mantey Dale, Omega-Njemnobi Onyinye, Montgomery LaTrice (2019///) Flavored tobacco use is associated with dual and poly tobacco use among adolescents. Addictive Behaviors; 93<br><br>**Keywords:**<br>*Nicotine,*Tobacco Smoking,*Electronic Cigarettes,Prevention<br><br>**Abstract:** Objectives: This study examines the relationships between flavored tobacco use and single, dual, and poly tobacco product use, among adolescents. Given the increased use of flavored tobacco products, it is vital to understand if flavored products are associated with dual and poly tobacco product use. Methods: Data were obtained from the 2017 National Youth Tobacco Survey. Participants were 2042 adolescent past 30-day tobacco users. Multivariable multinomial logistic regression models were used to assess the relationship between flavored tobacco use and past 30-day single, dual, and poly (three or more) tobacco product use. Two analyses were conducted using different categories as referent groups to allow for a comprehensive examination of the relationship between all groups. Covariates included sex, grade level, race/ethnicity and exposure to tobacco marketing. Results: Approximately half of all adolescent tobacco users (45.7%) reported use of more than one product. The majority of adolescent tobacco users reported using flavors (69.4%). Flavored tobacco use was significantly correlated with a greater risk of dual (RRR: 2.09) and poly (RRR: 5.54) tobacco use, relative to single product use, controlling for covariates. Similarly, flavored tobacco use was significantly correlated with a greater risk of poly (RRR: 2.66) tobacco use, relative to dual tobacco use, controlling for covariates. Conclusions: A positive relationship was observed for flavored tobacco use and multiple tobacco product use. Findings suggest the need to consider stronger regulations of flavored tobacco products. Similarly, as most adolescent tobacco users reported using flavors, findings suggest the need to emphasize flavored tobacco use in prevention and education programs. (PsycINFO Database Record (c) 2019 APA, all rights reserved)
2826	S McGrath Daniel, S Kim Hyoun, A Boothby Celina, K Romanow Nicole, C Hodgins David (2018///) Patterns and motives for electronic cigarette use in a sample of community-recruited gamblers. International Journal of Mental Health and Addiction; 16<br><br>**Keywords:**<br>*Comorbidity,*Tobacco Smoking,*Electronic Cigarettes,*Gambling Disorder<br><br>**Abstract:** Many smokers are replacing tobacco with electronic cigarettes (e-cigarettes) or are engaging in dual-use. Evidence indicates that smoking rates are higher amongst gamblers; however, the extent to which gamblers use e-cigarettes is unknown. The current study examined rates of e-cigarette use in gamblers, identified associations between e-cigarette use and gambling, and assessed motives for e-cigarette use during gambling. A community-recruited sample of gamblers (N = 564) completed questionnaires on e-cigarettes, smoking, and gambling. 'Ever use' of e-cigarettes was 38.7% with 17.6% reporting 'past 30-day use'. Furthermore, 11.9% used e-cigarettes while gambling in the past 12 months. Regression analyses for 'past 30-day use' revealed that occasional smoking, gambling severity, and

	number of gambling activities were associated with the highest odds of use. Reasons for use while gambling included: relaxation/stress, nicotine dependence and legal in casinos. These findings suggest e-cigarette use is common in gamblers and may be used to circumvent casino smoking bans. (PsycINFO Database Record (c) 2019 APA, all rights reserved)
2831	D Dave, B Feng, M F Pesko (2019///) The effects of e-cigarette minimum legal sale age laws on youth substance use. Health Economics (United Kingdom); 28<br><br>**Keywords:**<br>*age,alcohol consumption,article,behavior,cannabis use,human,juvenile,priority journal,*smoking regulation,statistics,vaping,*electronic cigarette,*minimum legal sale age<br><br>**Abstract:** We use difference-in-differences models and individual-level data from the national and state Youth Risk Behavior Surveillance System from 2005 to 2015 to examine the effects of e-cigarette minimum legal sale age (MLSA) laws on youth cigarette smoking, alcohol consumption, and marijuana use. Our results suggest that these laws increased youth smoking participation by about one percentage point and approximately half of the increased smoking participation could be attributed to smoking initiation. We find little evidence of higher cigarette smoking persisting beyond the point at which youth age out of the laws. Our results also show little effect of the laws on youth drinking, binge drinking, and marijuana use. Taking these together, our findings suggest a possible unintended effect of e-cigarette MLSA laws-rising cigarette use in the short term while youth are restricted from purchasing e-cigarettes.Copyright © 2019 John Wiley & Sons, Ltd.
2833	Bareham David, Ahmadi Keivan, Elie Mathieu, W Jones Arwel, McKee Martin (2018///) E-cigarettes: further flavours of controversy within the controversy. The Lancet. Respiratory medicine; 6<br><br>**Keywords:**<br>*Electronic Nicotine Delivery Systems,*Flavoring Agents/ae [Adverse Effects],Health Promotion/mt [Methods],Humans,Smoking Cessation/mt [Methods],United Kingdom<br><br>**Abstract:**
2836	Hammond David, L Reid Jessica, L Rynard Vicki, T Fong Geoffrey, K Michael Cummings, McNeill Ann, Hitchman Sara, F Thrasher James, L Goniewicz Maciej, Bansal-Travers Maansi, O'Connor Richard, Levy David, Borland Ron, M White Christine (2019///) Prevalence of vaping and smoking among adolescents in Canada, England, and the United States: repeat national cross sectional surveys. BMJ (Clinical research ed.); 365<br><br>**Keywords:**<br>Adolescent,Canada/ep [Epidemiology],Cross-Sectional Studies,England/ep [Epidemiology],Female, Humans,Male,Prevalence,*Smoking/ep [Epidemiology],United States/ep [Epidemiology],*Vaping/ep [Epidemiology],Young Adult<br><br>**Abstract:** OBJECTIVE: To examine differences in vaping and smoking prevalence among adolescents in Canada, England, and the United States., DESIGN: Repeat cross sectional surveys., SETTING: Online surveys in Canada, England, and the US., PARTICIPANTS: National samples of 16 to 19 year olds in 2017 and 2018, recruited from commercial panels in Canada (n=7891), England (n=7897), and the US (n=8140)., MAIN OUTCOME MEASURES: Prevalence of vaping and smoking was assessed for use ever, in the past 30 days, in the past week, and on 15 days or more in the past month. Use of JUUL (a nicotine salt based electronic cigarette with high nicotine concentration) and usual vaping brands were also assessed. Logistic regression models examined differences in vaping and smoking between countries and over time., RESULTS: The prevalence of vaping in the past 30 days, in the past week, and on 15 days or more in the past month increased in Canada and the US between 2017 and 2018 (P<0.001 for all), including

	among non-smokers and experimental smokers, with no changes in England. Smoking prevalence increased in Canada (P<0.001 for all measures), with modest increases in England, and no changes in the US. The percentage of ever vapers who reported more frequent vaping increased in Canada and the US (P<0.01 for all), but not in England. The use of JUUL increased in all countries, particularly the US and Canada-for example, the proportion of current vapers in the US citing JUUL as their usual brand increased threefold between 2017 and 2018., CONCLUSIONS: Between 2017 and 2018, among 16 to 19 year olds the prevalence of vaping increased in Canada and the US, as did smoking in Canada, with little change in England. The rapidly evolving vaping market and emergence of nicotine salt based products warrant close monitoring. Copyright Published by the BMJ Publishing Group Limited. For permission to use (where not already granted under a licence) please go to http://group.bmj.com/group/rights-licensing/permissions.
2851	J Delahanty, O Ganz, L Hoffman, J Guillory, E Crankshaw, M Farrelly (2019///) Tobacco use among lesbian, gay, bisexual and transgender young adults varies by sexual and gender identity. Drug and Alcohol Dependence; 201<br><br>**Keywords:**<br>adult,article,bisexual female,bisexual male,cigar smoking,cigarette smoking,controlled study,female, *gender identity,homosexual male,human,*LGBT people,male,multivariate logistic regression analysis, prevalence,priority journal,sexual and gender minority<br><br>**Abstract:** Background: Research shows that tobacco products are disproportionately used by sexual and gender minorities, known collectively as those who are lesbian, gay, bisexual, and transgender (LGBT). While usage varies within this population by identity, differences in tobacco use patterns are not well understood. The present study uses evaluation data for This Free Life, a tobacco public education campaign from the FDA, to examine differences in tobacco use among LGBT young adults by subgroups based on sex at birth, gender and sexual identity. Method(s): Data are from 4,057 18-24 LGBT young adults who completed the baseline This Free Life evaluation survey in 2016. Multivariable logistic regression models examined differences in tobacco use (cigarettes, cigars, hookah and electronic nicotine products) and poly use (2+ of any product, 2+ combustibles, at least one combustible and one noncombustible) between LGBT subgroups (cisgender gay males, cisgender bisexual males, cisgender lesbian/gay females, cisgender bisexual females and gender minorities). Result(s): Sexual minority females were more likely than gay males to use any tobacco product, electronic nicotine products and hookah. Cisgender bisexuals were more likely than gay males and gender minorities to use electronic nicotine products. Cisgender bisexual males were less likely than all other groups to use cigarettes. Cisgender sexual minority females were more likely than gender minorities to engage in poly use. Conclusion(s): Findings suggest that sexual minority females are more likely to use tobacco than other subgroups. More research can help campaign developers better understand reasons for intragroup differences in tobacco use among LGBT subgroups.Copyright © 2019
2852	J Delk, F R Carey, K R Case, M R Creamer, A V Wilkinson, C L Perry, M B Harrell (2019///) Adolescent Tobacco Uptake and Other Substance Use: A Latent Class Analysis. American journal of health behavior; 43<br><br>**Keywords:**<br>adolescence,adolescent,*alcohol consumption,article,binge drinking,controlled study,electronic cigarette,female,groups by age,human,*latent class analysis,major clinical study,male,marketing,Texas, *tobacco,alcohol,cannabis<br><br>**Abstract:** Objective: The purpose of this study was to identify patterns of tobacco uptake and other substance use, from early to late adolescence. Method(s): We used weighted latent class analysis, conducted separately for 7th, 9th, and 11th graders, to assess patterns of susceptibility, ever and current use of combustible tobacco and e-cigarettes, and other substance use (ie, current alcohol, binge drinking, and marijuana). Data were from Wave 3 of the Texas Adolescent Tobacco and Marketing Surveillance

	System (n = 2733; N = 461,069), collected in fall 2015. Multinomial regression was used to examine differences in class membership by demographic factors. Result(s): Two latent classes were identified in 7th grade, 3 classes in 9th grade, and 4 classes in 11th grade models. In each grade, classes included both a "no risk" and a "tobacco susceptible" class. For 9th grade, there was an additional "tobacco ever use" class, and 11th grade had the same additional class as well as an "all products use" class. Conclusion(s): Distinct patterns of polysubstance use emerged as grade level increased, supporting a stage-sequential model of onset and progression across developmental age groups. Future research can examine other factors affecting transitions across these stages.
2853	C Denis-Vatant, C Merieux, L Leclerc, H Duc, C Berton, R Jarrige, M Nekaa, J M Vergnon, J Pourchez (2019///) Relationship between vaping and smoking among 15-year-olds in high school. Results of a descriptive cross-sectional and monocentric observational study conducted in the metropolitan area of Saint-Etienne. Revue des Maladies Respiratoires;<br><br>**Keywords:**<br>adolescent,art,article,child,controlled study,female,France,girl,*high school,human,major clinical study, male,nonsmoker,*observational study,prevalence,*smoking habit,spring,student,tobacco dependence, *vaping,vocation<br><br>**Abstract:** Introduction: The initiation of smoking among adolescents by vaping is a subject of controversy. This study focuses on the prevalence of electronic cigarette use among teenagers and its connection with the consumption of tobacco. Method(s): A cross-sectional and monocentric study was conducted in the spring of 2018 and included 1435 students (15-16 years old) from the metropolitan area of Saint-Etienne in France. Result(s): Nearly half of the adolescents experimented with e-cigarettes (50.30%) or tobacco (50.40%). Nearly a quarter are vapers (23.60%) or smokers (28.20%), with low daily use (3.65% for vaping and 9.40% for smoking). In regard to the link between smoking and vaping, 64.85% of adolescents are "non-smokers and non-vapers", 17.60% "smokers and vapers", 11.25% "smokers and non-vapers", and 6.30% "non-smokers and vapers". Discussion(s): The portrait-types of the vaper and the smoker are similar: boy rather than girl, educated in private school rather than public, and enrolled in a vocational rather than a general educational course. On one hand, the use of electronic cigarettes in non-smoking adolescents does not appear to be a major mode of entry into smoking or nicotine addiction. On the other hand, the use of electronic cigarettes among adolescent's smokers seems to have a beneficial effect on their smoking habit (stopping or reducing the consumption of tobacco).Copyright © 2019 SPLF
2867	P Du, T Fan, J Yingst, S Veldheer, S Hrabovsky, C Chen, J Foulds (2019///) Changes in E-Cigarette Use Behaviors and Dependence in Long-term E-Cigarette Users. American Journal of Preventive Medicine; 57<br><br>**Keywords:**<br>adult,article,behavior assessment,cigarette smoking,clinical article,controlled study,correlation coefficient,data analysis,*drug dependence/di [Diagnosis],female,follow up,*health behavior,human,long term care,longitudinal study,male,medical research,o<br><br>**Abstract:** Introduction: Little is known about the effects of long-term e-cigarette use, particularly the risks of relapse to cigarette smoking or increased dependence. Method(s): In a 2012-2014 baseline online e-cigarette survey, 1,863 respondents consented to participate in future research. A follow-up online survey was conducted in 2017-2018 to assess changes in e-cigarette use behaviors and e-cigarette-related dependence. For both surveys, exclusive e-cigarette use was defined as only using e-cigarettes in the past 7 days, and poly use was defined as using both e-cigarettes and other tobacco or nicotine products in the past 7 days. The Penn State Electronic Cigarette Dependence Index (PSECDI) score was calculated for each study subject and was used to evaluate e-cigarette dependence. Paired t-tests or Pearson correlation coefficients were used to examine changes in e-cigarette use behaviors or PSECDI scores between baseline and follow-up. Baseline and follow-up survey data were analyzed in January 2019. Result(s): A total of 494 subjects provided complete data on both surveys. At baseline, 402 subjects

	(81.4%) were exclusive e-cigarette users, and 71 subjects (14.4%) were poly users. Among baseline exclusive e-cigarette users, the majority (88.3%) continued using e-cigarettes exclusively, but 37 users (9.2%) became poly users and 1 returned to cigarette smoking at follow-up. Among baseline poly users, 60.6% became exclusive e-cigarette users at follow-up. The mean PSECDI score remained similar over time (8.4 at baseline vs 8.3 at follow-up). Conclusion(s): Findings suggest that the risk of relapse to cigarette smoking is low, and e-cigarette-related dependence remains stable in long-term e-cigarette users.Copyright © 2019 American Journal of Preventive Medicine
2868	Y Du, M Shih, M D Shah, M D Weber, A S Lightstone (2019///) Prevalence and sociodemographic disparities in ever E-cigarette use among adults in Los Angeles County. Preventive Medicine Reports; 15<br><br>**Keywords:**<br>adult,age,article,Asian,binge drinking,Black person,California,cannabis use,Caucasian,cigarette smoking, *demography,descriptive research,disability,ex smoker,female,graduate,*health disparity,health survey, high school graduate,Hispanic,human,major clini<br><br>**Abstract:** E-cigarette use has increased rapidly among U.S. adults. Few studies have examined the prevalence and risk factors of e-cigarette use among adults in the United States. We conducted descriptive analyses to identify characteristics of ever e-cigarette users among adults (18 years and older)living in Los Angeles (LA)County, the most populous county in the U.S., using data from the 2015 LA County Health Survey. We used logistic regression to examine independent factors associated with ever e-cigarette use. Age-adjusted prevalence of ever e-cigarette use was 8.4%. A significant interaction between conventional cigarette smoking status and gender was found ($p < 0.05$), and several factors were identified as associated with ever e-cigarette use in models stratified by gender. Various social and demographic factors are associated with ever e-cigarette use and should be incorporated into evidence-based interventions.Copyright © 2019
2869	M S Dunbar, J P Davis, A Rodriguez, J S Tucker, R Seelam, E J D'Amico (2018///) Disentangling Within- and Between-Person Effects of Shared Risk Factors on E-cigarette and Cigarette Use Trajectories From Late Adolescence to Young Adulthood. Nicotine & tobacco research : official journal of the Society for Research on Nicotine and Tobacco;<br><br>**Keywords:**<br>*adolescence,adolescent,adult,*adulthood,alcohol consumption,article,cannabis use,*cigarette smoking, cohort analysis,*electronic cigarette,female,human,juvenile,longitudinal study,major clinical study,male, mental disease,risk assessment,*risk factor,alc<br><br>**Abstract:** Background: Longitudinal studies report associations between use of electronic cigarettes (ECs) and cigarettes over time among young people, but do not distinguish within- from between-person effects, which complicates interpretation of findings. Further, the role of shared risk factors, such as substance use and mental health, in explaining longitudinal associations between EC and cigarette use remains unclear. This study used within- and between-person analyses to assess longitudinal associations between youths' EC and cigarette use and shared risk factors. Method(s): Between 2015 and 2017, 2039 youths completed three Web-based surveys, allowing us to model EC and cigarette use from ages 16 to 20. Auto-regressive latent growth models with structured residuals (ALT-SR) examined both between-person and within-person associations between past-month frequency of EC use, cigarette use, and third variables (alcohol and marijuana use, mental health symptoms) over time. Result(s): Models revealed robust reciprocal associations between EC and cigarette use, such that more frequent EC use at one time predicted more frequent cigarette use at the subsequent time, and vice versa. Between-person analyses showed associations between shared risk factors and both EC and cigarette use. However, shared risk factors did not predict frequency of subsequent EC and cigarette use in within-person analyses. Conclusion(s): Findings add to a growing body of research suggesting that EC use among youth is prospectively associated with progression toward greater cigarette use. Shared risk factors may help

	explain differences in EC and cigarette use patterns between young people, but do not appear to influence longitudinal trajectories of EC and cigarette use within individuals. Implications: This study examined within- and between-person associations between e-cigarette use, cigarette use, and shared risk factors (alcohol use, marijuana use, mental health symptoms) in a longitudinal cohort of youths. Within- and between-person analyses revealed reciprocal prospective associations between e-cigarette and cigarette use, suggesting a progression toward more frequent use of both products over time. The shared risk factors examined here did not affect escalations in e-cigarette or cigarette use over time within individuals, but likely influence which youths use these products. Findings add to a growing body of evidence suggesting that e-cigarette use increases subsequent cigarette use in young people.
2873	G M M e Oliveira, M Mendes, O P Dutra, A Achutti, M Fernandes, V Azevedo, M B S E C S Ferreira, A S Coelho, M B P C Soares, M A B L Evora, M G Mariotto, J A Morais (2019///) 2019: Recommendations for reducing tobacco consumption in Portuguese-speaking countries-positioning of the federation of Portuguese language cardiology societies. Arquivos Brasileiros de Cardiologia; 112<br><br>**Keywords:**<br>article,Buerger disease,cardiovascular risk,chronic obstructive lung disease,clinical practice,cognitive behavioral therapy,coronary artery disease,counseling,follow up,health care personnel,heart failure,heart infarction,human,hypertension,law,morbidit<br><br>**Abstract:**
2878	F El-Awa, M A Tageldin, V Prasad, A Al-Mulla, G Heydari, R Alebshehy (2019///) Lung health in the Eastern Mediterranean Region: The need to end designated smoking areas in public places. Eastern Mediterranean Health Journal; 25<br><br>**Keywords:**<br>asthma,cancer risk,cardiovascular risk,cigarette smoking,disease severity,editorial,environmental decision making,human,lung cancer,morbidity,mortality,passive smoking,prevalence,*public health,risk factor,*smoking,tobacco dependence,tobacco use,waterpi<br><br>**Abstract:**
2882	K Elin Smith (2019///) Prevalence and Correlates of Electronic Cigarette Use Among a Clinical Sample of Polysubstance Users in Kentucky: Long Live the Cigarette?. Substance use & misuse; 54<br><br>**Keywords:**<br>adult,article,*cigarette smoking,Clostridium ljungdahlii,*electronic cigarette,female,human,human tissue,*Kentucky,major clinical study,male,nonhuman,population,*prevalence,*tobacco use,illicit drug,nicotine<br><br>**Abstract:** BACKGROUND: E-cigarette use has increased in the US, yet, in some regions rates of cigarette use remain high. PURPOSE: To describe the prevalence and features of lifetime and past-year e-cigarette use among a clinical sample of polysubstance users in Kentucky, and to determine significant associations of past-year e-cigarette use. RESULT(S): Of the final sample (N = 497), 83.5% reported having ever used e-cigarettes and 97.2% reported having ever used cigarettes. These rates surpass those found among Kentucky's general population. Compared to those who did not report e-cigarette use, e-cigarette users were more likely to be younger (formula presented = 33.4 vs. 43.6, p<.001) and White (88.2.1% vs. 62.5%, p = .001). E-cigarette users showed higher rates for lifetime incarceration (91.1% vs. 72.8%, p = .001) and past-year arrest (75.0% vs. 47.5%, p = .001). This group also presented with more severe substance use history and lower mean age for illicit drug use initiation (formula presented = 13.8 vs. 16.4, p = .001). Approximately 65% of the sample reported past-year e-cigarette use and 96.6% reported past-year cigarette use. Logistic regression indicated that being younger (AOR = .973, p = .030), White (AOR = 1.92, .046), having a past-year arrest (AOR = 1.73, p = .047) and having used cigarettes (AOR =

	8.93, p = .001) or kratom (AOR = 3.04, p = .025) within the past year were significantly associated with past-year e-cigarette use. CONCLUSION(S): E-cigarette use was related to more severe drug-using patterns. Rates of dual tobacco use among this sample are high, particularly among younger individuals. In ecological contexts where cigarette use remains normative, it is likely that dual use will persist for nicotine-dependent, polysubstance-using individuals.
2884	Turner Elise, A Fedele David, Thompson Lindsay, G Salloum Ramzi (2018///) Patterns of electronic cigarette use in youth with asthma: Results from a nationally representative sample. Annals of allergy, asthma & immunology : official publication of the American College of Allergy, Asthma, & Immunology; 120<br><br>**Keywords:**<br>Adolescent,*Asthma/ep [Epidemiology],Cigarette Smoking/ae [Adverse Effects],Female,Humans,Male, Patient Education as Topic,Perception,*Population Groups,Prevalence,Risk Factors,Smoking Cessation, Surveys and Questionnaires,United States/ep [Epidemiology],<br><br>**Abstract:**
2885	F El-Khoury, C Bolze, R Gomajee, V White, M Melchior (2019///) Lower smoking rates and increased perceived harm of cigarettes among French adults one year after comprehensive tobacco control measures. Drug and Alcohol Dependence; 201<br><br>**Keywords:**<br>adult,age distribution,article,*cigarette smoking,controlled study,cross-sectional study,female, Frenchman,health hazard,human,male,middle aged,*perception,prevalence,priority journal,program effectiveness,public health message,*smoking regulation,teleph<br><br>**Abstract:** Context: France has high smoking rates, and recently intensified tobacco control policies spearheaded by the introduction of plain tobacco packaging (PP), and an increase in graphic health warnings (GHW). We examine smoking and e-cigarettes use rates, as well as smoking-related perceptions before (2016) and one year after (2017) comprehensive tobacco control measures. Method(s): DePICT is a two waves cross-sectional national telephone survey of French adults aged 18-64 years (2016: 4456 - 2017: 4114). Data were weighted to be representative of the French adult population. Adjusted prevalence ratios (PR, 95% CI) estimating changes between the two study waves were calculated using multivariate Log-Binomial regression models. Main Findings: In 2017, as compared with 2016, smoking rates (PR = 0.93 (0.88-0.99) and current e-cigarette use (PR = 0.76 (0.61-0.96)) decreased in France. Further, French adults were more likely to report fear of the consequences of smoking (PR = 1.10 (1.06-1.14)) and that smoking is dangerous (PR = 1.08 (1.06-1.11)). Smokers were also more likely to report that health messages on tobacco products are efficient (PR = 1.18 (1.05-1.32)). Conclusion(s): Our study provides early and encouraging results on potential effects of the comprehensive tobacco control strategies in France introduced in 2017, including PP and larger GHW. Our findings also suggest that e-cigarettes did not replace traditional smoking.Copyright © 2019
2887	O El-Shahawy, S H Park, D T Duncan, L Lee, K Tamura, J A Shearston, M Weitzman, S E Sherman (2019///) Evaluating State-Level Differences in E-cigarette and Cigarette Use among Adults in the United States between 2012 and 2014: Findings from the national adult tobacco survey. Nicotine and Tobacco Research; 21<br><br>**Keywords:**<br>adult,article,*cigarette smoking,Delaware,demography,female,health care delivery,*health survey, human,male,Oklahoma,prevalence,priority journal,scoring system,social control,social status,United States,*vaping,Vermont,West Virginia

	**Abstract:** Objective: To examine the association between state-level tobacco control measures and current use estimates of both e-cigarettes and cigarettes, while accounting for socio-demographic correlates. Method(s): Using the 2012-2013 and 2013-2014 National AdultTobacco Survey (NATS), we assessed prevalence estimates of US adults' e-cigarette and cigarette current use. Four state groups were created based on the combined state-specific prevalence of both products: low cigarette/e-cigarette (n = 15), high cigarette/e-cigarette (n = 16), high cigarette/low e-cigarette (n = 11), and low cigarette/ high e-cigarette) (n = 9). To evaluate the implementation of state-level tobacco control measures, Tobacco Control Index (TCI) was calculated using the State of Tobacco Control annual reports for 2012 and 2013. Multinomial logistic regression models were used to examine differences among the four groups on socio-demographic factors and TCI. Low cigarette/e-cigarette group was used as the referent group. Result(s): Current use estimates of each product varied substantially by state; current e-cigarette use was highest in Oklahoma (10.3%) and lowest in Delaware (2.7%), and current cigarette use was highest in West Virginia (26.1%), and lowest in Vermont (12.6%). Compared to low cigarette/e-cigarette, all other US-state categories had significantly lower TCI scores (high cigarette/e-cigarette: adjusted Relative Risk Ratio [aRRR] = 0.61; 95% confidence interval [CI]: 0.60-0.61, high cigarette/low e-cigarette: aRRR = 0.74; 95% CI: 0.73-0.74, and low cigarette/high e-cigarette: aRRR = 0.72; 95% CI: 0.71-073). Conclusion(s): Enforcing existing tobacco control measures likely interacts with e-cigarette use despite being cigarette-focused. Continuing to monitor e-cigarette use is critical to establish baseline use and evaluate future e-cigarette specific federal and state-level tobacco regulatory actions while accounting for the existing tobacco control environment. Implications:This study investigates state-level current use estimates of e-cigarettes and cigarettes among US adults; and their association with four existing tobacco control measures.The overall score of these measures was negatively associated with state-level current use estimates such that states with low current e-cigarette and cigarette use had the highest mean overall score.This study assesses the potential relationship between existing state-level tobacco control measures and e-cigarette use and calls for improving the enforcement of the known-to-work tobacco control measures across all US states, while developing evidence-based regulations and interventions specific to e-cigarettes within the existing US tobacco use environment.Copyright © The Author(s) 2018. Published by Oxford University Press on behalf of the Society for Research on Nicotine and Tobacco. All rights reserved.
2896	Balogh Erika, Faubl Nora, Riemenschneider Henna, Balazs Peter, Bergmann Antje, Cseh Karoly, Horvath Ferenc, Schelling Jorg, Terebessy Andras, Wagner Zoltan, Voigt Karen, Fuzesi Zsuzsanna, Kiss Istvan (2018///) Cigarette, waterpipe and e-cigarette use among an international sample of medical students. Cross-sectional multicenter study in Germany and Hungary. BMC public health; 18<br><br>**Keywords:**<br>Adult,*Cigarette Smoking/ep [Epidemiology],Cross-Sectional Studies,Female,Germany/ep [Epidemiology], Humans,Hungary/ep [Epidemiology],Internationality,Male,Prevalence,*Students, Medical/px [Psychology],Students, Medical/sn [Statistics & Numerical Data],*<br><br>**Abstract:** BACKGROUND: Tobacco use is the leading preventable cause of death worldwide. Besides cigarette smoking, waterpipe and e-cigarettes are gaining popularity among young adults. Medical students' smoking behavior is of particular interest because of their impending role in health promotion as future physicians. Aim of our study is to examine the prevalence and predictors of cigarette, waterpipe and e-cigarette use and the association of tobacco use with self-reported health status in an international sample of medical students., METHODS: In a multicenter cross-sectional study data on different aspects of health behavior were collected from medical students of 65 nationalities using a self-administered questionnaire in Germany (Dresden, Munich) and Hungary (Budapest, Pecs). The survey was conducted among 1st, 3rd and 5th year students. To explore associations between smoking behavior and socio-cultural factors Pearson's chi2-tests and multivariate binary logistic regression analyses were performed., RESULTS: The largest subpopulations were formed by German (n = 1289), Hungarian (n = 1055) and Norwegian (n = 147) students. Mean age was 22.5 +/- 3.3 years. Females represented 61.6% of the

	sample. In the whole sample prevalence of cigarette smoking was 18.0% (95% CI 16.6-19.4%), prevalence of waterpipe use was 4.8% (95% CI 4.0-5.7%), that of e-cigarette 0.9% (95% CI 0.5-1.2%). More males (22.0%) than females (15.5%) reported cigarette smoking. The lowest prevalence of cigarette smoking was found among Norwegian students (6.2%). Cigarette smokers were older, waterpipe users were younger than non-users. E-cigarette use was not associated with age of the students. Religious involvement was protective only against cigarette smoking. Financial situation showed no association with any kind of tobacco consumption. Cigarette smokers and e-cigarette users were less likely to report very good or excellent health status., CONCLUSIONS: Cigarette smoking is still the most popular way of consuming tobacco, although alternative tobacco use is also prevalent among medical students. To further health consciousness, medical schools should pay more attention to students' health behavior, especially their smoking habits. Tobacco prevention and cessation programs for medical students should consider not only the health risks of cigarette smoking but the need to discourage other forms of tobacco use, such as waterpipe.
2903	J F Etter (2018///) Electronic cigarette: A longitudinal study of regular vapers. Nicotine and Tobacco Research; 20<br><br>**Keywords:**<br>adult,article,cohort analysis,drug craving,female,follow up,human,longitudinal study,male,priority journal,relapse,smoking cessation,smoking habit,tobacco,trend study,*vaping,*electronic cigarette<br><br>**Abstract:** Introduction: It is unclear how vaping behavior changes over time in regular vapers, and what occurs when vapers relapse to smoking or when they stop vaping. We assessed change in vaping and smoking behaviors over 12 months in regular vapers. Method(s): A longitudinal study of 3868 regular vapers enrolled on the Internet in 2012-2015 ("baseline"), followed after one (n = 1631, 42%), three (n = 1337, 35%), six (n = 1148, 30%), and 12 months (n = 893, 23%). Result(s): Participants had been vaping for a median of five months at baseline. Most (77%) were former smokers, who had not smoked for a median of three months at baseline. Over 12 months, enjoyment gradually became the most frequently cited reason to vape (93%), and vaping to deal with craving for tobacco gradually decreased (from 87% to 56%). In exclusive vapers (ex-smokers), nicotine concentration in e-liquids decreased over time (from 12 to 9 mg/mL), but puffs/day remained stable (200 puffs/day). After 12 months, 9% of 687 former smokers relapsed to smoking and 28% of 64 daily smokers (dual users) stopped smoking. After 12 months, when participants stopped vaping, they tended to relapse to smoking (+18% daily smokers among those who stopped vaping versus -2% in permanent vapers, p < .001). When ex-smokers relapsed to smoking, they tended to stop vaping. Conclusion(s): After 12 months, enjoyment and relapse prevention were the most important reasons to vape. Rates of relapse to smoking were low in former smokers and quit rates were high in current smokers. Stopping vaping was associated with relapsing to smoking. Implications: Even in established vapers, vaping behavior and reasons to vape change over time. This should be taken into account by clinicians, manufacturers and regulators. Results from this non-representative sample can help generate hypotheses that can later be tested in representative samples of vapers.Copyright © The Author(s) 2017. Published by Oxford University Press on behalf of the Society for Research on Nicotine and Tobacco. All rights reserved.
2914	G Feng, Y Nan, Y Jiang (2018///) Prevalence of e-cigarette in China: Preliminary findings from two surveys. Tobacco Induced Diseases; 16<br><br>**Keywords:**<br>adult,awareness,case report,*China,clinical article,cohort analysis,*electronic cigarette,human,male, monitoring,nonsmoker,*prevalence,smoking,conference abstract<br><br>**Abstract:** Background: China is the world's largest tobacco producer and consumer, in recent years has become the world's largest producer of e-cigarette. According to media reports, China-made e-cigarette is mainly exported to foreign countries, and e-cigarette are not popular in China, but such conclusions

	need support from surveillance data. Method(s): Data of 2015 China Adult Tobacco Survey (CATS) and International Tobacco Control Policy Evaluation Project China Survey (ITC China Survey) were analyzed to describe rate of awareness and prevalence on e-cigarette. 2015 CATS is a nationally representative cross-sectional survey of adults aged 15 and over. ITC China survey is a cohort study designed to measure the impact of tobacco control policies on tobacco users over time. Between 2006 and 2015, five survey waves were conducted among a cohort of adult smokers and non-smokers in China. Result(s): The CATS found that 40.5% of adults had heard of e-cigarette and 3.1% of adults had ever tried e-cigarette, these two rates are higher among male, urban, young and educated groups. Only 0.5% of adults reported using e-cigarette currently, but most of them are occasional users. ITC China survey revealed that the percentage of smokers who had ever heard of e-cigarette went up from 29% (wave 3, 2009) to 60% (wave5, 2014). Similarly, percentage of smokers who had ever tried e-cigarette went up from 2% (wave3, 2009) to 11% (wave5, 2014). Among smokers who had tried e-cigarette at Wave 5, only 8% reported they were using currently. Conclusion(s): Although rate of current e-cigarette use is relatively low, attention should be paid to the fact that rate of awareness and ever tried use were going up among smokers. E-cigarette prevalence indicators should be included in tobacco use monitoring system, and more researches need be conducted to understand the characteristics of the population using e-cigarette.
2915	F Filippidis, I Agaku, C Vardavas (2018///) Transition from experimentation with tobacco and nicotine products to regular smoking in Europe. Tobacco Induced Diseases; 16<br><br>**Keywords:**<br>adult,*chewing tobacco,cigar,controlled study,death,electronic cigarette,*Europe,European Union, female,*hookah,human,major clinical study,male,mortality,nose,public policy,*nicotine,conference abstract<br><br>**Abstract:** Background: Most of the tobacco-attributable deaths are from combustible tobacco products. We explored transitions from experimentation of different tobacco/nicotine products to regular use of combustible products in a European population. Method(s): We analysed a representative sample of persons aged >=15 years from 28 European Union member states (Eurobarometer wave 87.1; March 2017; n=27,901). Respondents who indicated ever trying tobacco/nicotine products, including combustible products (boxed and hand-rolled cigarettes; cigars/cigarillos; pipe; waterpipe); e-cigarettes; and smokeless products (oral/nasal/chewing tobacco), were asked which product they first tried. Questions on history of regular tobacco use (>=1-time weekly) were further asked to determine whether ever tobacco users became established users at a point in their lifetime, regardless of their current use status. We used multilevel logistic regression to measure the association between first product tried and ever becoming an established smoker of combustible tobacco (ESCT), adjusting for age, sex, area of residence and financial difficulties We restricted analysis to those aged < 40 years (n=8,125) to reduce differential smoking-attributable mortality from different age cohorts. Result(s): Overall, 58.7% of persons aged < 40 years had ever tried a tobacco/nicotine product. Among these 79.7% first tried boxed cigarettes, 7.0% hand-rolled cigarettes, 6.2% waterpipe and 1.9% e-cigarettes. Compared to those who first tried e-cigarettes, the odds of ever becoming an ESCT were higher among those who first tried boxed (aOR=3.50; 95%CI: 2.26-5.44) or hand-rolled cigarettes (aOR=4.70; 95%CI: 2.74-8.04), but lower among those who first tried waterpipe (aOR=0.30; 95%CI: 0.18-0.51). Adjusted odds did not differ significantly among those who first tried pipe or smokeless tobacco compared to first trial with e-cigarettes. Conclusion(s): About 4 in 5 EU smokers first tried cigarettes, and these had increased likelihood of becoming established combustible tobacco smokers in their lifetime. Exploring reasons for experimenting with emerging products (e.g., e-cigarettes, hookahs) can inform public policy.
2917	M E Flacco, M Ferrante, M Fiore, C Marzuillo, C La Vecchia, M R Gualano, G Liguori, G Fragassi, T Carradori, F Bravi, R Siliquini, W Ricciardi, P Villari, L Manzoli (2019///) Cohort study of electronic cigarette use: Safety and effectiveness after 4 years of follow-up. European Review for Medical and Pharmacological Sciences; 23

	**Keywords:**<br>adult,aged,article,carbon monoxide blood level,*clinical effectiveness,cohort analysis,female,follow up, hospital discharge,human,longitudinal study,male,*patient safety,self report,smoking,smoking cessation, validation process,*vaping,carbon monoxide/ec<br><br>**Abstract:** OBJECTIVE: More than a decade after e-cigarette (e-cig) market launch, limited information are available on their safety after 24 months of use. In 2013, we started the first observational study assessing e-cig long-term effectiveness and safety, directly comparing tobacco smokers and e-cig users. Here we report the results after four years of follow-up. PATIENTS AND METHODS: Adults (30-75 years) were included if: smokers of =1 tobacco cigarette/day (tobacco smokers); users of any type of e-cig inhaling =50 puffs weekly (e-cig users); users of both tobacco and e-cig (dual users). Data were collected by phone and/or internet, and carbon monoxide levels tested in 50% of those declaring tobacco abstinence. Main outcomes were: possibly smoking-related diseases (PSRD; validated through hospital discharge data or visit in 62.6% of the sample); 4-year tobacco abstinence; number of tobacco cigarettes/day. RESULT(S): Data were available for 228 e-cig users (all ex-smokers), 471 tobacco smokers, 216 dual users. A PSRD was observed in 73 subjects (8.0%). No differences emerged across groups in PSRD rates, with negligible variations in self-reported health. Of e-cig users, 63.6% remained tobacco abstinent; dual users and tobacco smokers showed non-significantly different rates of tobacco (33.8% vs. 26.8%) and all-product (20.2% vs. 19.4%) cessation, and a similar decrease in cigarettes/day. Almost 40% of the sample switched at least once (tobacco smokers: 17.2%; dual users: 81.9%). CONCLUSION(S): After four years, a scarce, non-significant harm reduction was observed among e-cig or dual users. Given the long-lasting health effects of tobacco smoking, the benefits of e-cig use may start being detectable at the next follow-up (six years). The complete switch to e-cig may help tobacco quitters remain abstinent, but e-cig use in addition to tobacco did not increase the likelihood of smoking cessation or reduction.Copyright © 2019 Verduci Editore s.r.l. All rights reserved.
2922	A S Friedman, J Buckell, J L Sindelar (2019///) Patterns of Youth Cigarette Experimentation and Onset of Habitual Smoking. American Journal of Preventive Medicine; 56<br><br>**Keywords:**<br>adult,article,*cigarette smoking,controlled study,cross-sectional study,female,human,male,*minor (person),online system,outcome assessment,*predictor variable,*smoking habit,vaping,young adult<br><br>**Abstract:** Introduction: Although research suggests that youth e-cigarette experimentation is associated with later combustible cigarette experimentation, it is unclear how this relates to habitual smoking. This study assesses how minors' patterns of combustible cigarette and e-cigarette experimentation relate to habitual smoking at ages 18-21 years. Method(s): Between November 2016 and May 2017, a cross-sectional, online survey of current and retrospective cigarette use was fielded among individuals aged 18-21 years who had tried combustible cigarettes or e-cigarettes (n=1,424). Logistic regressions tested how experimentation patterns prior to age 18 years related to two indicators of current habitual smoking: daily smoking and current established smoking (past 30-day use among those who had smoked >=100 cigarettes). Result(s): Respondents who first tried combustible cigarettes or e-cigarettes as minors (n=1, 096)were more likely to be current established users (64%)than those who first experimented at ages 18-21 years (41%). Experimentation patterns in individuals aged <18 years beginning with combustible cigarettes were the most predictive of later smoking. Relative to those who first experimented at ages >17 years (n=328), trying only combustible cigarettes as a minor yielded a 175% increase in one's odds of being an established smoker (AOR=2.75, 95% CI=1.99, 3.79)and a 161% increase for daily smoking (AOR=2.61, 95% CI=1.75, 3.90). Trying combustibles and then e-cigarettes yielded sizable increases in both habitual smoking measures, whereas trying e-cigarettes before combustibles yielded smaller effects. Trying only e-cigarettes as a minor yielded a 78% decrease in both outcomes, relative to those who did not try either product as minors. Conclusion(s): Minors who tried combustible cigarettes were

	more likely to be habitual smokers at ages 18-21 years than those who tried e-cigarettes alone.Copyright © 2019 American Journal of Preventive Medicine
2927	M Anic Gabriella, D Sawdey Michael, Jamal Ahmed, F Trivers Katrina (2018///) Frequency of Use Among Middle and High School Student Tobacco Product Users - United States, 2015-2017. MMWR. Morbidity and mortality weekly report; 67<br><br>**Keywords:**<br>Adolescent,Child,Cross-Sectional Studies,Electronic Nicotine Delivery Systems/sn [Statistics & Numerical Data],Health Surveys,Humans,Schools/sn [Statistics & Numerical Data],*Students/px [Psychology], Students/sn [Statistics & Numerical Data],*Tobacco Pr<br><br>**Abstract:** Tobacco product use during adolescence increases the risk for lifelong nicotine addiction and immediate adverse health effects (1,2). During 2011-2017, current use of cigarettes, cigars, smokeless tobacco, and pipe tobacco decreased significantly among middle and high school students, but current use of e-cigarettes increased significantly from 1.5% to 11.7% (3). In 2017, an estimated 19.6% of high school students (2.95 million) and 5.6% of middle school students (0.67 million) were current users of any tobacco product; e-cigarettes were the most commonly used tobacco product for both middle (3.3%) and high (11.7%) school students (3). The Food and Drug Administration (FDA) and CDC analyzed combined data from the 2015-2017 National Youth Tobacco Surveys (NYTS) to determine past 30-day (current) frequency of use of cigarettes, e-cigarettes, cigars, smokeless tobacco, and hookahs among U.S. high school and middle school students. During 2015-2017, the proportion of students currently using tobacco products who used a product for >=20 of the past 30 days ranged from 14.0% of cigar smokers to 38.7% of smokeless tobacco users among high school students and from 13.1% of e-cigarette users to 24.5% of hookah smokers among middle school students. Among current users, use of two or more tobacco products ranged from 76.7% (e-cigarettes) to 90.9% (hookahs) among those using the product >=20 of the preceding 30 days, from 68.0% (e-cigarettes) to 84.2% (hookahs) among those using the product for 6 to 19 of the preceding 30 days, and from 48.8% (e-cigarettes) to 77.2% (cigarettes) among those using the product for 1 to 5 of the preceding 30 days. Sustained implementation of proven tobacco control strategies focusing on all types of tobacco products, in coordination with the regulation of tobacco products by FDA, are needed to reduce tobacco product initiation and use among U.S. youths.
2930	M Gambaryan, A M Kalinina, M V Popovich, M L Starovoytov, O M Drapkina, S A Boytsov (2019///) Electronic cigarettes in Russia: Time for an action. Results from Russian Tobacco Control policy evaluation survey. European Journal of Preventive Cardiology; 26<br><br>**Keywords:**<br>achievement,addiction,adult,advertising,controlled study,ex smoker,female,groups by age,household, human,Internet,law,major clinical study,male,marketing,randomized controlled trial,restaurant,*Russian (citizen),*smoking cessation,social network,stratifi<br><br>**Abstract:** Electronic nicotine delivery systems (ENDS) or e-cigarettes (e-cigs) become more and more popular in Russia, due to aggressive marketing, and yet are not regulated under the Russian Tobacco Control Law. Still very little is known about exposure of people to these products in Russia. Aim(s): To assess the exposure of population to ENDS as well as the exposure of population to advertisement, promotion of ENDS in Russia, and its attitudes and beliefs regarding these products. Method(s): Data from Russian Tobacco Control Law Evaluation Survey (June-August 2017) are analysed 11699 randomly selected respondents from 4320 households in 5 Russian Subjects were interviewed and a stratified sample (n=5289 aged>=18 years) of smokers (n=3228), former smokers (n=1012) and never smokers (n=1049) participated in the survey. Result(s): 34% of the respondents were familiar with ENDS; 11.9% had tried e-cigs, 25.8% of those were regular users. 69.2% of cigarette smokers said they hadn't reduce the amount of smoked tobacco while using e-cigarettes. 44.4% had made a quit attempt by means of e-cigarettes: of those 65.2% failed to quit smoking, and 75.3% could not quit neither smoking nor e-

	cigarettes in a long term. Only 18.7% ENDS users agreed that e-cigarettes cause addiction, 35.8% were not sure, and 45.5% even denied the fact. Those were mostly the youngest respondents aged 18-24 years (52.3%), among all age groups (p<.05). Vaping was not allowed in the household of 70% respondents. Mostly vaping was allowed at home of the current tobacco smokers (21.8%), compared to quitters (p<.05) and in the youngest age group 18-24 (28%) (p<.05). 90.3% said that vaping was not allowed at their workplace. However 64% had seen people vaping e-cigs in public places where smoking is banned, 27.1% had witnessed vaping in restaurants and bars, 59.8% had been exposed to vaping in public places and 45.9% of those had found it annoying. In the past 6 month 19.6% of the respondents were exposed to promotion of ENDS via internet, 18.4% - at the points of sale, 18.1% - via social networks. The other sources of advertisement had lesser contribution in marketing and promotion of ENDS. Exposure of the youngest age group (18-24) to e-cigs promotion was 2-3 times higher compared to the other age groups-via all sources (p<.0001). 61% of respondents believed that the second hand vape of e-cigarettes is harmful for the bystanders. Majority agreed that is necessary to regulate allowed amounts of the nicotine in eliquids (65.4%), ban the aromatic supplements (56,5%), ban vaping in public places where tobacco smoking is banned (70.6%), ban the sales of these products to minors (82%), and the use of e-cigarettes by those(83,7%). 77,6% support the need for ban of all forms of advertisement, marketing and promotion of ENDS. Conclusion(s): These findings are used to support strong legislation to regulate ENDS in Russia, which is crucial for the achievements of tobacco control policy.
2938	T A Ghebreyesus (2019///) Progress in beating the tobacco epidemic. The Lancet; 394<br><br>**Keywords:**<br>adolescent smoking,brain development,cardiovascular risk,epidemic,evidence based practice,health hazard,human,note,passive smoking,prefrontal cortex,priority journal,*smoking ban,*smoking cessation, tax,text messaging,tobacco dependence,*tobacco use,nico<br><br>**Abstract:**
2939	T S Ghosh, R Tolliver, A Reidmohr, M Lynch (2019///) Youth vaping and associated risk behaviors - A snapshot of Colorado. New England Journal of Medicine; 380<br><br>**Keywords:**<br>addiction,*adolescent smoking,Asian,Black person,brain development,Caucasian,cognitive defect, Colorado,ethnic difference,heterosexuality,*high risk behavior,human,impulsiveness,letter,LGBT people, mood disorder,prevalence,priority journal,sexual behavior<br><br>**Abstract:**
2940	Helen Gideon St, L Eaton David (2018///) Public Health Consequences of e-Cigarette Use. JAMA Internal Medicine; 178<br><br>**Keywords:**<br>*Electronic Nicotine Delivery Systems,Humans,Public Health,*Vaping/ae [Adverse Effects]<br><br>**Abstract:**
2951	D Golinelli, D Siconolfi, W G Shadel, R Seelam, J S Tucker (2019///) Patterns of alternative tobacco product use among youth experiencing homelessness. Addictive Behaviors; 99<br><br>**Keywords:**<br>adolescent,adult,article,California,chewing tobacco,controlled study,demography,descriptive research, female,*homelessness,human,male,population research,probability sample,*smoking,*tobacco use, cigar,cigarette,electronic cigarette,hookah,little cigar,na

	**Abstract:** Introduction: Identifying distinct patterns of tobacco product use can inform efforts to reduce poly-tobacco use among young people, but little is known regarding patterns of use among youth experiencing homelessness. This study identified patterns of using tobacco/nicotine products among youth experiencing homelessness, and assessed whether certain subgroups of youth were more likely than others to engage in specific patterns of use. Method(s): Data were collected from a probability sample of 469 homeless youth who used tobacco in the past month, recruited from 25 service and street sites in Los Angeles County. Participants reported on lifetime and past month use of natural cigarettes, cigars, little cigars/cigarillos, electronic nicotine delivery systems, hookah, and chewing tobacco. Latent class analysis was used to identify patterns of tobacco product use. Result(s): We identified four main classes of use: traditional cigarettes smokers (34.7% of the sample), poly-tobacco experimenters (24.9%), current users of combustible products (natural cigarettes, cigars, little cigars/cigarillos; 27.1%), and current poly-tobacco users (13.1%). Youth who were male, slept outdoors, and screened positive for substance abuse disorder were more than twice as likely as their counterparts to be current poly-tobacco users relative to traditional cigarettes smokers. Conclusion(s): Rates of poly-tobacco experimentation and current use among youth experiencing homelessness are high. This suggests that efforts to reduce the use of tobacco products in this population should focus on the combined use of these products, and further, that users with the greatest poly-use may have competing unmet needs such as substance use disorders and more severe homelessness.Copyright © 2019 Elsevier Ltd
2958	S Gravely, S C Hitchman, A McNeil, K M Cummings, R Borland, H H Yong, D Hammond, G T Fong (2018///) Use of electronic cigarettes across 13 ITC countries with different regulatory environments. Tobacco Induced Diseases; 16<br><br>**Keywords:** adult,Australia,awareness,Bangladesh,Brazil,Canada,China,country economic status,cross-sectional study, *electronic cigarette,England,ex smoker,female,human,Malaysia,male,Mexico,Netherlands,prevalence, South Korea,tobacco,Uruguay,Zambia,conference abstrac<br><br>**Abstract:** Background: Electronic cigarettes have become an international phenomenon, although few studies have compared e-cigarette use across countries. This paper presents prevalence estimates of self-reported e-cigarette awareness, trial (ever-tried), current use (daily/weekly/monthly), and daily use from 13 countries that vary on the strictness of e-cigarette policies/regulations. Method(s): Cross-sectional analyses of representative samples of adult (>=18-years) current and former smokers participating in International Tobacco Control Project (ITC) surveys in 13 countries from the most recent ITC survey wave (2013-2017). Countries were categorized into those with no e-cigarette policies (NP), less restrictive policies (LRP), or more restrictive policies (MRP). Result(s): Weighted prevalence estimates of four key measures were computed: [1] Awareness: for NP countries: Zambia (2014): 3%, Bangladesh (2014/15): 7%, China (2013/15): 59%; LRP countries: Netherlands (2017): 92%, Republic of Korea (2016): 94%, United States (US) and England (2016): 99%; MRP countries: Uruguay (2014): 52%, Mexico (2014/15): 61%, Brazil (2016): 73%, Malaysia (2013/2014): 86%, Australia and Canada (2016): 99%. [2] Trial: for NP countries: Zambia (0%), Bangladesh (1%), China (11%); LRP countries: Netherlands (39%), Korea (44%), England (52%). US (58%); MRP countries: Uruguay (7%), Mexico (10%), Malaysia (38%), Australia (45%), Canada (49%). [3] Daily/weekly/monthly use: for NP countries: Zambia and Bangladesh (< 1%); China (1%); LRP countries: Korea (6%), Netherlands, US, England (7%); MRP countries: Uruguay (0%), Brazil (1%), Mexico (2%), Australia and Canada (6%), Malaysia (12%). [4] Daily use: for NP countries: Zambia, Bangladesh, China (all 0%); LRP countries: Korea (3%), Netherlands (3%), US (3%), England (4%); MRP countries: Uruguay and Brazil (0%), Mexico (1%), Canada (2%), Australia (3%), Malaysia (4%). (Figure presented) Conclusion(s): With minor exceptions (e.g. Australia), awareness, trial, and use of e-cigarettes across the 13 countries generally reflected the de facto environment rather than the statutory environment implied by the law(s). Country income classification and survey year also appear to be strongly associated with use.

2961	V R Green, K P Conway, M L Silveira, K A Kasza, A Cohn, K M Cummings, C A Stanton, P Callahan-Lyon, W Slavit, J D Sargent, N Hilmi, R S Niaura, C J Reissig, E Lambert, I Zandberg, M F Brunette, S E Tanski, N Borek, A J Hyland, W M Compton (2018///) Mental Health Problems and Onset of Tobacco Use Among 12- to 24-Year-Olds in the PATH Study. Journal of the American Academy of Child and Adolescent Psychiatry; 57<br><br>**Keywords:**<br>adolescent,adult,African American,alcohol consumption,article,Asian,cannabis use,Caucasian,child,cohort analysis,ethnicity,female,follow up,health behavior,Hispanic,human,longitudinal study,major clinical study,male,*mental disease,mental health,prescri<br><br>**Abstract:** Objective: To examine whether mental health problems predict incident use of 12 different tobacco products in a nationally representative sample of youth and young adults. Method(s): This study analyzed Wave (W) 1 and W2 data from 10,533 12- to 24-year-old W1 never tobacco users in the Population Assessment of Tobacco and Health (PATH) Study. Self-reported lifetime internalizing and externalizing symptoms were assessed at W1. Past 12-month use of cigarettes, electronic nicotine delivery systems (ENDS), traditional cigars, cigarillos, filtered cigars, pipe, hookah, snus pouches, other smokeless tobacco, bidis and kreteks (youth only), and dissolvable tobacco was assessed at W2. Result(s): In multivariable regression analyses, high-severity W1 internalizing (adjusted odds ratio [AOR] = 1.5, 95% CI = 1.3-1.8) and externalizing (AOR = 1.3, 95% CI = 1.1-1.5) problems predicted W2 onset of any tobacco use compared to no/low/moderate severity. High-severity W1 internalizing problems predicted W2 use onset across most tobacco products. High-severity W1 externalizing problems predicted onset of any tobacco (AOR = 1.6, 95% CI = 1.3-1.8), cigarettes (AOR = 1.4, 95% CI = 1.0-2.0), ENDS (AOR = 1.8, 95% CI = 1.5-2.1), and cigarillos (AOR = 1.5, 95% CI = 1.0-2.1) among youth only. Conclusion(s): Internalizing and externalizing problems predicted onset of any tobacco use. However, findings differed for internalizing and externalizing problems across tobacco products, and by age, gender, and race/ethnicity. In addition to screening for tobacco product use, health care providers should screen for a range of mental health problems as a predictor of tobacco use. Interventions addressing mental health problems may prevent youth from initiating tobacco use.Copyright © 2018 American Academy of Child and Adolescent Psychiatry
2971	E C Hair, A R Romberg, R Niaura, D B Abrams, M A Bennett, H Xiao, J M Rath, L Pitzer, D Vallone (2019///) Longitudinal tobacco use transitions among adolescents and young adults: 2014-2016. Nicotine and Tobacco Research; 21<br><br>**Keywords:**<br>adolescent,adult,age,article,controlled study,female,health care policy,human,longitudinal study,male, nonsmoker,prediction,priority journal,probability,public health,tobacco,*tobacco use,vaping,young adult<br><br>**Abstract:** Introduction: Among youth, the frequency and prevalence of using more than one tobacco (small cigar, cigarette, and hookah) or nicotine-containing product (e-cigarettes-ENDS) are changing. These shifts pose challenges for regulation, intervention, and prevention campaigns because of scant longitudinal data on the stability of use patterns in this changing product landscape. Method(s): A nationally representative longitudinal survey of 15- to 21-year olds (n = 15,275) was used to describe transitions between never use, noncurrent use, and past 30-day use of combustible tobacco, e-cigarettes (ENDS), and dual use of both kinds of products. A multistate model was fit to observations collected every 6 months across 2.5 years to estimate the probability of transitions between states (TPs), the average time in state (sojourn time), and the effect of age on transitions. Result(s): Current state strongly predicted future state over time intervals of 1 year or less, but only weakly predicted future state at longer intervals: TP to noncurrent use was higher for ENDS-only than combustible-only users over a 6-month interval but was similar for both groups over a 2-year interval. Sojourn time was significantly longer for combustible-only (0.52 years) and dual use (0.55 years) than ENDS-only use (0.27 years); older

	youth were more likely than younger youth to stay combustible tobacco users or noncurrent users. Conclusion(s): The dynamics of transitions between combustible tobacco products and ENDS in a population of youth and young adults suggest that policy and prevention efforts must consider the frequent changes and instability over a 1-year or less time period in use patterns among young people. Implications: The study addresses an urgent need in public health for timely information on how youth and young adults use tobacco and nicotine products. We found that youth, particularly adolescents, moved frequently between using ENDS and combustible tobacco products either alone or together. Importantly, the utility of current-use states for predicting future use states declined for time horizons longer than 1 year. Our results demonstrate a need for caution in interpreting product transitions. Longitudinal data with frequent observations and coverage of a wide range of possible product types is required to fully characterize usage patterns in youth.Copyright © The Author(s) 2018. Published by Oxford University Press on behalf of the Society for Research on Nicotine and Tobacco. All rights reserved.
2974	B Hallingberg, O M Maynard, L Bauld, R Brown, L Gray, E Lowthian, A M MacKintosh, L Moore, M R Munafo, G Moore (2019///) Have e-cigarettes renormalised or displaced youth smoking? Results of a segmented regression analysis of repeated cross sectional survey data in England, Scotland and Wales. Tobacco control;<br><br>**Keywords:**<br>alcohol consumption,article,cannabis use,controlled study,*electronic cigarette,*England,female,*harm reduction,human,*juvenile,major clinical study,male,prevalence,*public policy,*regression analysis, *Scotland,sensitivity analysis,*smoking,time series<br><br>**Abstract:** OBJECTIVES: To examine whether during a period of limited e-cigarette regulation and rapid growth in their use, smoking began to become renormalised among young people. DESIGN: Interrupted time-series analysis of repeated cross-sectional time-series data. SETTING: Great Britain PARTICIPANTS: 248324 young people aged approximately 13 and 15 years, from three national surveys during the years 1998-2015. INTERVENTION: Unregulated growth of e-cigarette use (following the year 2010, until 2015). OUTCOME MEASURES: Primary outcomes were prevalence of self-reported ever smoking and regular smoking. Secondary outcomes were attitudes towards smoking. Tertiary outcomes were ever use of cannabis and alcohol. RESULT(S): In final models, no significant change was detected in the pre-existing trend for ever smoking (OR 1.01, CI 0.99 to 1.03). There was a marginally significant slowing in the rate of decline for regular smoking (OR 1.04, CI 1.00 to 1.08), accompanied by a larger slowing in the rate of decline of cannabis use (OR 1.21, CI 1.18 to 1.25) and alcohol use (OR 1.17, CI 1.14 to 1.19). In all models and subgroup analyses for smoking attitudes, an increased rate of decline was observed after 2010 (OR 0.88, CI 0.86to 0.90). Models were robust to sensitivity analyses. CONCLUSION(S): There was a marginal slowing in the decline in regular smoking during the period following 2010, when e-cigarettes were emerging but relatively unregulated. However, these patterns were not unique to tobacco use and the decline in the acceptability of smoking behaviour among youth accelerated during this time. These analyses provide little evidence that renormalisation of youth smoking was occurring during a period of rapid growth and limited regulation of e-cigarettes from 2011 to 2015. TRIAL REGISTRATION NUMBER: Research registry number: researchregistry4336.Copyright © Author(s) (or their employer(s)) 2019. Re-use permitted under CC BY. Published by BMJ.
2980	M O Harhay, A B Troxel, C Brophy, K Saulsgiver, K G Volpp, S D Halpern (2019///) Financial incentives promote smoking cessation directly, not by increasing use of cessation AIDS. Annals of the American Thoracic Society; 16<br><br>**Keywords:**<br>adult,controlled study,evidence based practice,female,human,intermethod comparison,letter,major clinical study,male,program effectiveness,randomized controlled trial,*reward,smoking,*smoking cessation,*electronic cigarette

	**Abstract:**
2982	M Harrell, C Jackson, J Delk, S Opara, C Perry (2018///) Youth who initiate tobacco use with e-cigarettes only differ from those who start with cigarettes only or both products concomitantly. Tobacco Induced Diseases; 16<br><br>**Keywords:**<br>controlled study,*electronic cigarette,expectation,human,human experiment,*juvenile,major clinical study,marketing,peer pressure,risk factor,sensation seeking,student,Texas,*tobacco use,urban area, alcohol,cannabis,conference abstract<br><br>**Abstract:** Background: Few studies have compared the psychosocial risk profiles for youth who begin using tobacco with e-cigarettes only, cigarettes only, or both of these products. Method(s): More than 40 known psychosocial risk factors for youth tobacco use, including intra-personal (e.g., sensation seeking), inter-personal (e.g., peer influence), and environmental (e.g., tobacco marketing) factors were compared between those who started with e-cigarettes only (n=156, N=14550), cigarettes only (n=59, N=7325), or both of these products (n=35, N=3510). Psychosocial risk factors were measured at baseline (2014-15) and tobacco use behaviors at 6, 12, and 18 month follow-ups (2015-16) in a population-based sample (n) of 3,116 students in 4 large urban areas of Texas that generalizes back to 353,552 students (N) who were in the 6th, 8th, and 10th grades and had never used tobacco at baseline. Result(s): Youth who started with cigarettes only or both, compared to youth who started with e-cigarettes only, were more likely to have a family member that used cigarettes or e-cigarettes (p< 0.01) and be past 30-day marijuana users (p=0.01) at baseline. Youth who started with e-cigarettes only, compared with the others, reported a higher positive affect (p=0.01) and lower negative affect at baseline (p=0.01) and were more likely to have positive outcome expectations about tobacco use (e.g., it helps me concentrate; p=0.05) and be past 30-day alcohol users (p=0.05) at baseline. Youth who started with both products, compared to one exclusively, were more likely to have engaged with tobaccorelated content on digital media (e.g., posted pictures, blogged) (p< 0.05) and less likely to report seeing displays for cigarettes at retail point-of-sale (p< 0.01) at baseline. No other differences in known risk factors (e.g., sensation seeking) were observed. Conclusion(s): Psychosocial risk profiles do vary significantly by starter tobacco products. Implications for intervention will be discussed.
2988	L Hedman, H Backman, C Stridsman, M Andersson, A Lindberg, E Ronmark (2018///) E-cigarette use among Swedish teenagers. European Respiratory Journal; 52<br><br>**Keywords:**<br>adolescent,asthma,child,chronic obstructive lung disease,cohort analysis,controlled study,eating, *electronic cigarette,ex smoker,female,girl,healthy diet,human,major clinical study,male,nonsmoker, parental smoking,physical activity,prevalence,questionnai<br><br>**Abstract:** Electronic (e) cigarettes are portrayed as a substitute for conventional cigarettes and as a means for smoking cessation. In contradiction, much of the marketing is designed to appeal to teenagers and nonsmokers. Aim(s): to estimate the prevalence of e-cigarette use in relation to smoking habits and demographic factors in teenagers. Within the Obstructive Lung Disease in Northern Sweden (OLIN) studies a cohort study of schoolchildren (n=2430) was initiated at age 7-8y, with follow-ups at age 11-12y, 14-15y and 19y by completion of an extended ISAAC questionnaire. Questions on smoking and snus use were included from age 14-15y and use of e-cigarettes at 19y. At age 19y, 22.0% (95%CI 20.3-23.7) had ever tried e-cigarettes, with higher proportion in boys than girls, 28.3 vs 15.2% p<.001. The prevalence of current use was 4.4% (95%CI 3.6-5.2), also higher among boys, 5.4 vs 3.4% p=.01. Current smoking (10.3%) was slightly higher in girls than boys, 11.1 vs 9.0% p=.20. Of current e-cigarette users, 39% were smokers, 22% ex-smokers and 39% nonsmokers. 'Ever tried' e-cigarettes was related to tobacco use at age 14-15y: 60.9% in smokers and 50.8% in snus users, however 19.6% of nonsmokers had tried e-

	cigarettes. In adjusted logistic regression analysis, current e-cigarette use was related to male sex (OR 2.1 95%CI 1.7-2.7), former smoking (OR 3.6 95%CI 2.5-5.3), current smoking (OR 5.3 95%CI 3.9-7.4), use of snus (OR 1.6 95%CI 1.3-2.1) and inversely related to eating healthy diet (OR 0.9 95%CI 0.8-0.9). There were no significant associations with parental smoking, socioeconomic status, physical activity or having asthma. In summary, e-cigarette use was common among smokers but alarmingly almost 40% of e-cigarette users at age 19y were nonsmokers.
2989	K R Hefner, A Sollazzo, S Mullaney, K L Coker, M Sofuoglu (2019///) E-cigarettes, alcohol use, and mental health: Use and perceptions of e-cigarettes among college students, by alcohol use and mental health status. Addictive Behaviors; 91<br><br>**Keywords:**<br>adult,*alcohol consumption,alcoholic beverage,alcoholism,anxiety disorder,article,binge drinking,bipolar disorder,college student,controlled study,depression,female,human,major clinical study,male,*mental health,personality disorder,posttraumatic stress<br><br>**Abstract:** Introduction: Electronic cigarettes (e-cigarettes) are popular among college students, who display risky alcohol use patterns. However, little is known about patterns of co-use of e-cigarettes and alcohol. Further, relationships between e-cigarette use and mental illness among college students are unclear. Method(s): College student participants (N = 631) at a northeastern U.S. university were invited via email to participate in a survey about e-cigarettes and alcohol use. Mental health was self-reported diagnosis of psychiatric (depression, bipolar disorder, schizophrenia, PTSD, anxiety disorder, personality disorder), and substance (alcohol and other drug) use disorders. Current use of e-cigarette, combustible cigarette, and other tobacco products were assessed via self-reported past 30-day use frequency. Alcohol consumption was assessed via number of self-reported standard alcoholic beverages consumed during a typical drinking episode. Participants also reported regarding co-use of alcohol, e-cigarettes and/or combustible cigarettes. Participants were categorized as non-drinkers, moderate drinkers or binge drinkers, and associations between e-cigarette use, drinking patterns and mental health diagnoses were examined. Result(s): E-cigarette use was associated with drinking alcohol chi2 = 18.62, p <.001, and binge drinking (vs. moderate drinking) chi2 = 12.20, p <.001. Students who had tried e-cigarettes reported drinking more alcohol per episode (chi2 = 15.94, p <.001). E-cigarette use was more prevalent among those with psychiatric and substance use disorders chi2 = 11.65, p <.001. Conclusion(s): Drinking college students (especially binge drinkers) and those with mental illness may have heightened risks for e-cigarette use. More research is needed to elucidate relationships between risky alcohol and/or nicotine use and mental illness, and to guide appropriate prevention and intervention efforts for vulnerable college students.Copyright © 2018 Elsevier Ltd
2990	C Heiss (2019///) Electronic cigarettes: Replacing one evil with another?. European Journal of Preventive Cardiology; 26<br><br>**Keywords:**<br>arterial stiffness,atrial fibrillation,blood pressure,cardiovascular disease,cardiovascular risk,editorial, endothelial dysfunction,heart rate,human,hypertension,knowledge,nicotine replacement therapy, prevalence,priority journal,risk factor,*smoking,*smo<br><br>**Abstract:**
2998	A Hinton, H N Nagaraja, S Cooper, M E Wewers (2018///) Tobacco product transition patterns in rural and urban cohorts: Where do dual users go?. Preventive Medicine Reports; 12<br><br>**Keywords:**<br>adult,article,cohort analysis,female,human,longitudinal study,male,multicenter study,normal human, Ohio,priority journal,rural area,smokeless tobacco,*tobacco use,urban area,electronic cigarette

	**Abstract:** Introduction: Understanding diverse tobacco product consumption represents a crucial area for tobacco regulatory science. With the increase in dual/poly use of tobacco products, transition patterns among exclusive and dual users are of considerable interest. We describe transition patterns of dual users over 18 months. Method(s): A cohort of 145 adults in urban and rural Ohio who reported dual tobacco product use at least some days/week was enrolled during 2014-17. Participants completed follow-up interviews every six months where they were classified into one of five categories: 1) exclusive combustible, 2) exclusive smokeless, 3) exclusive e-cigarette, 4) dual (at least 2 of the previous 3 categories), and 5) less than some days/week. Participants categorized as exclusive and dual (1-4) used their products at least some days per week. Separately within the rural and urban cohorts, 6, 12, and 18 month transition probabilities between the categories were estimated. Result(s): The probability of remaining a dual user after 6 months is 43% in the rural and 37% in the urban cohort. The decline continues through 18 months with 24% of rural and 22% of urban dual users remaining in the category. The probability of a dual user consuming combustibles and e-cigarettes transitioning to exclusive combustible use in 6 months is over 50% in both the rural and urban cohorts. Conclusion(s): Dual use is an unstable state with users being more likely to transition to exclusive combustible use than to remain in the dual use category. Transitions are similar in the rural and urban cohorts.Copyright © 2018 The Authors
3003	Dai Hongying (2018///) Single, Dual, and Poly Use of Flavored Tobacco Products Among Youths. Preventing chronic disease; 15<br><br>**Keywords:**<br>Adolescent,Cross-Sectional Studies,Data Collection,*Electronic Nicotine Delivery Systems,Female, Humans,Logistic Models,Male,Students,Surveys and Questionnaires,*Tobacco Products/sn [Statistics & Numerical Data]<br><br>**Abstract:** INTRODUCTION: Flavoring has become the leading reason for current tobacco use among adolescents. This study sought to evaluate patterns of flavored tobacco product use and associated risk factors among youths., METHODS: Weighted estimates of single, dual, and poly use of flavored tobacco products were calculated from the 2014 National Youth Tobacco Survey (n = 21,926). Multinomial logistic regression was performed to assess factors associated with flavored product use., RESULTS: Among current tobacco users (n = 3,805), 70.0% of students were current users of flavored tobacco products: 42.6% used a single flavored product, 16.8% used 2 flavored products (dual users), and 10.6% used more than 2 flavored products (poly users). Flavored product use, especially dual and poly use, was higher among high school students compared with middle school students. Compared with single flavored tobacco product users (36%), dual (57%) and poly users (79%) of flavored tobacco products had higher prevalences of using flavored e-cigarettes (P < .001). Non-Hispanic blacks and those of other races had lower prevalences than non-Hispanic whites of using flavored products but not nonflavored products. Tobacco use by household members, no perception of harm from tobacco products, and more frequent exposure to tobacco advertisement in newspapers/magazines and stores were associated with increased odds of flavored product use., CONCLUSION: The concurrent use of flavored tobacco products is prevalent among youths. E-cigarettes were the leading flavored product and often concurrently used with other flavored tobacco products. Comprehensive control and prevention strategies to reduce flavored tobacco use among youths are needed.
3007	S Sean Hu, M Homa David, Wang Teresa, Gomez Yessica, Walton Kimp, Lu Hua, Neff Linda (2019///) State-Specific Patterns of Cigarette Smoking, Smokeless Tobacco Use, and E-Cigarette Use Among Adults - United States, 2016. Preventing chronic disease; 16<br><br>**Keywords:**<br>Adult,Behavioral Risk Factor Surveillance System,*Cigarette Smoking/ep [Epidemiology],Cross-Sectional Studies,*Electronic Nicotine Delivery Systems/sn [Statistics & Numerical Data],Female,Humans,Male,

	Population Surveillance,Prevalence,*Tobacco, Smokeles<br><br>**Abstract:** INTRODUCTION: State-level monitoring of changes in tobacco product use can help inform tobacco control policy and practice. This study examined state-specific prevalence of cigarette, smokeless tobacco, and e-cigarette use among US adults., METHODS: Data came from the 2016 Behavioral Risk Factor Surveillance System (BRFSS), a state-based telephone survey of US adults aged 18 years or older (N = 477,665). Prevalence estimates for current (every day or some days) cigarette smoking, smokeless tobacco use, and e-cigarette use were calculated for all 50 states and the District of Columbia (DC) and stratified by sex and race/ethnicity. Because the 2016 BRFSS measured e-cigarette use for the first time, estimates of ever e-cigarette use and concurrent use of cigarettes and e-cigarettes were also calculated. We assessed subgroup differences with chi2 tests., RESULTS: In 2016, prevalence of current cigarette smoking among US adults ranged from 8.8% (Utah) to 24.8% (West Virginia), while prevalence of current smokeless tobacco use ranged from 1.3% (DC) to 9.8% (Wyoming). For e-cigarettes, ever use ranged from 16.2% (DC) to 28.4% (Arkansas), and current use ranged from 2.4% (DC) to 6.7% (Oklahoma). Across all states, current e-cigarette use was significantly higher among current cigarette smokers than among former or never cigarette smokers. States with the highest prevalence of cigarette smoking generally had a high prevalence of current e-cigarette use., CONCLUSION: Prevalence of adult cigarette smoking, smokeless tobacco use, and e-cigarette use varies across states. These findings underscore the importance of comprehensive statewide tobacco control and use prevention efforts that address the diverse tobacco products used among adults.
3009	J Huang, P Redmon, Z Duan, Y Duan, Y Jiang, M Eriksen (2018///) Use of electronic nicotine delivery systems (ENDS) among Chinese adults: Evidence from citywide representative surveys from 12 cities in China (2014 to 2016). Tobacco Induced Diseases; 16<br><br>**Keywords:** abstinence,adolescent,adult,aging,awareness,*China,*city,controlled study,death,education,*electronic cigarette,human,major clinical study,male,postmarketing surveillance,public health,sampling,smoking, young adult,nicotine,conference abstract<br><br>**Abstract:** Background: Tobacco is the leading cause of preventable death in China. Any comprehensive strategies to reduce tobacco use in China need to increase smoking cessation among current smokers. While the evidence is limited, ENDS may reduce the health risks for current smokers if they switch completely to ENDS. ENDS have the potential of reducing harms of tobacco smoking if Chinese smokers who do not intent to quit can switch completely to ENDS. To systematically assess the awareness and use of ENDS among Chinese adults, Tobacco Questions for Surveys (TQS) was conducted in twelve Chinese cities from 2014 to 2016. Method(s): Citywide representative surveys were conducted in 12 Chinese cities between 2014 and 2016. The target population was noninstitutionalized urban adults age 15 and above. Multistage cluster sampling was applied to select 2,500 individuals from each city. SAS and R were used to obtain point estimates and adjusted associations accounting for the complex sample design features. Result(s): Awareness of ENDS was above 40% in all 12 cities, the percent of respondents who have heard about ENDS ranging from 41.3% (Qingdao) to 63.2% (Xi'an). The overall rate of ever use ENDS was 2.9%, and the overall rate of current use was 0.9%. In addition, across most cities, awareness of ENDS were statistically significantly associated with current smoking, being male, aging 25-34, and having higher education. Conclusion(s): Significant variations existed in awareness and use of ENDS among urban Chinese adults. Tailored interventions, as part of a comprehensive tobacco control and cessation strategy, which may include ENDS in assisting smokers in achieving complete cessation, may have a positive public health impact in China. In addition, nation-wide surveillance is needed to monitor the use of ENDS among both adult smokers and among adolescents and young adults, with an emphasis on the complete abstinence from any nicotine product among adolescents.
3019	M Ickes, J W Hester, A T Wiggins, M K Rayens, E J Hahn, R Kavuluru (2019///) Prevalence and reasons for Juul use among college students. Journal of American college health : J of ACH;

	**Keywords:**<br>adult,article,cigarette smoking,*college student,convenience sample,cross-sectional study,*electronic cigarette,friend,human,juvenile,male,odor,*prevalence,risk factor,*tobacco dependence,young adult<br><br>**Abstract:** OBJECTIVE: Examine Juul use patterns, sociodemographic and personal factors associated with Juul use, and reasons for Juul initiation and current use, among college students. PARTICIPANTS: Convenience sample of 371 undergraduates at a large university in the southeast; recruited April 2018. METHOD(S): Cross-sectional design using an online survey. Logistic regression identified the personal risk factors for current use. RESULT(S): Over 80% of participants recognized Juul; 36% reported ever use and 21% past 30-day use. Significant risk factors for current Juul use were: male, White/non-Hispanic, lower undergraduate, and current cigarette smoker. Current Juul users chose ease of use and lack of a bad smell as reasons for use. Ever Juul users most commonly endorsed curiosity and use by friends as reasons for trying Juul. CONCLUSION(S): Given the propensity for nicotine addiction among youth and young adults, rates of Juul use are alarming and warrant immediate intervention.
3021	I Ilic, S Grujicic Sipetic, D Radovanovic, M Ilic (2019///) Cigarette Smoking and E-Cigarette Use by Pharmacy Students in Serbia. Behavioral Medicine;<br><br>**Keywords:**<br>alcohol consumption,article,*cigarette smoking,controlled study,cross-sectional study,*electronic cigarette,financial management,human,*pharmacy student,prevalence,*Serbia,smoking<br><br>**Abstract:** The purpose of this study was to assess the use of e-cigarettes among pharmacy students. In 2016, we conducted a cross-sectional study among pharmacy students enrolled at the University of Kragujevac (Serbia). Overall, 47.0% of pharmacy students reported that they ever smoked cigarettes; 29.7% were former and 17.3% were current smokers. The overall prevalence of e-cigarette use was 9.9%. The final year pharmacy students significantly more frequently smoked tobacco cigarettes (p =.001) and used e-cigarettes (p =.009) compared to younger students. Also, alcohol use was positively related to ever smoking tobacco cigarettes (adjusted OR = 4.57, 95% CI = 2.80-7.43; p <.001) and to ever use of e-cigarettes (adjusted OR = 5.58, 95% CI = 1.58-19.71; p =.008). The pharmacy students who ever used e-cigarettes more frequently reported a history of self-funded study financing (adjusted OR = 14.68, 95% CI = 2.42-89.17; p =.004) and use of psychoactive substances (adjusted OR = 13.63, 95% CI = 2.52-73.69; p =.002). In pharmacy students, a higher overall grade was related to a significantly less frequent ever use of tobacco cigarettes (adjusted OR = 0.43, 95% CI = 0.22-0.84, p =.015). This paper highlights the need for a more effective tobacco control among pharmacy students in Serbia in order to reduce smoking prevalence.Copyright © 2019, © 2019 Taylor & Francis Group, LLC.
3024	N Iqbal, J A Khan (2018///) Electronic cigarettes use and perception amongst medical students, a cross sectional survey from Sindh, Pakistan. Tobacco Induced Diseases; 16<br><br>**Keywords:**<br>adult,controlled study,*electronic cigarette,female,hookah,human,major clinical study,male,*medical student,*Pakistan,*perception,pregnancy,questionnaire,respiratory tract disease,smokeless tobacco, young adult,conference abstract<br><br>**Abstract:** Background: The manufacturers of e-cigarettes are actively marketing their product by using electronic and social media. However there is scarcity of data regarding it use and perception among medical students of a developing country like Pakistan. Method(s): A Cross sectional survey was conducted from 1st July till 30th September 2016 in five medical universities of Sindh, Pakistan. A self-administered questionnaire was used, a total of 500 medical students consented to participate in the study Results: The mean age was 21.5+/-1.7 years and 58% were females. Over 65% had knowledge about e-cigarettes and majority were tobacco user (87.6%, p< 0.001).The frequency of e-cigarette use

	was 6.2% while daily use was 1.2% and was found higher among tobacco user 13.9% and males 74% (p< 0.001).E-Cigarettes were mostly perceived as less harmful than tobacco cigarettes(39%), helpful in quitting smoking (27%),associated with respiratory diseases (36%),addictive (36%) and not safe for pregnancy(44%).E-cigarette users were found to be more indulged in other tobacco products including conventional cigarettes (80.6%), shisha(83.9%) and smokeless tobacco (38.7%). Conclusion(s): Although the use of e-cigarettes among medical students is low but most of the students who took on e-cigarettes ended up as a dual user rather than quitter.
3037	A Rohde Jacob, M Noar Seth, Horvitz Casey, J Lazard Allison, Ross Jennifer Cornacchione, L Sutfin Erin (2018///) The Role of Knowledge and Risk Beliefs in Adolescent E-Cigarette Use: A Pilot Study. International Journal of Environmental Research and Public Health; 15<br><br>**Keywords:**<br>Adolescent,Adolescent Behavior,Cross-Sectional Studies,*Electronic Nicotine Delivery Systems/sn [Statistics & Numerical Data],Family Relations,Female,*Health Knowledge, Attitudes, Practice,Humans, Male,Pilot Projects,Risk,Socioeconomic Factors,Tobacco Us<br><br>**Abstract:** The use of e-cigarettes and other vaping devices among adolescents is an urgent public health problem due to the concern about adolescent exposure to nicotine. This study examined: (1) adolescents’ knowledge and beliefs about e-cigarette risks; and (2) whether knowledge and risk beliefs were associated with e-cigarette use. N = 69 adolescents completed a cross-sectional survey about e-cigarette knowledge, attitudes (i.e., risk beliefs), and behavior (KAB). Nearly half (47%) of the sample reported ever using e-cigarettes. The majority of adolescents knew about many of the risks of e-cigarettes, with no differences between never- and ever-users. However, risk beliefs, such as worrying about health risks of using e-cigarettes, varied across groups. Compared to never-users, e-cigarette ever-users were significantly less likely to worry about e-cigarette health risks, less likely to think that e-cigarettes would cause them negative health consequences, and less likely to believe that e-cigarette use would lead to addiction. In a multivariable logistic regression, prior combustible cigarette use, mother’s education, and addiction risk beliefs about e-cigarettes emerged as significant predictors of adolescents’ e-cigarette use. This study reveals that while knowledge is not associated with adolescent e-cigarette use, risk beliefs do predict use.
3040	M Eberth Jan, C McLain Alexander, Hong Yuan, Sercy Erica, Diedhiou Abdoulaye, J Kilpatrick Daniel (2018///) Estimating county-level tobacco use and exposure in South Carolina: a spatial model-based small area estimation approach. Annals of epidemiology; 28<br><br>**Keywords:**<br>Adult,Electronic Nicotine Delivery Systems,Female,Health Behavior,Humans,Male,Middle Aged, Population Surveillance,Prevalence,*Smoking/ep [Epidemiology],South Carolina/ep [Epidemiology], Tobacco Smoke Pollution,*Tobacco Use<br><br>**Abstract:** PURPOSE: Local health statistics are increasingly requested for policy-making and programmatic purposes; however, population-based surveys are often inadequate to support direct estimation for small areas. Model-based estimation techniques can be used to create local estimates for public health outcomes. Using the 2014-2015 South Carolina (SC) Adult Tobacco Survey, we examined tobacco-related outcomes at the county level using a spatial multilevel, poststratification approach., METHODS: To create county-level tobacco estimates, we used a two-level model with a spatially intrinsic conditional autoregressive random intercept. Stratum-specific (race, age, and sex) estimates for each county were then created and averaged based on population data obtained from the U.S. Census., RESULTS: The estimated prevalence of current smoking in SC counties among adults ranged from 7.4% to 35.1%, and the percentage reporting ever trying an e-cigarette ranged from 4.2% to 30.2%. Model validation showed considerable agreement between direct and indirect estimates (Pearson and Spearman correlations all >0.75) that varied by the sample size of the outcome, as hypothesized.,

	CONCLUSIONS: Data from the SC Adult Tobacco Survey were used to develop county-level estimates of multiple tobacco-related outcomes using a spatial multilevel, poststratification approach. The results showed heterogeneity in smoking behaviors across the state along with marked spatial correlation. Copyright © 2018 Elsevier Inc. All rights reserved.
3041	Audrain-McGovern Janet, D Stone Matthew, Barrington-Trimis Jessica, B Unger Jennifer, M Leventhal Adam (2018///) Adolescent E-Cigarette, Hookah, and Conventional Cigarette Use and Subsequent Marijuana Use. Pediatrics; 142<br><br>**Keywords:**<br>Adolescent,Adolescent Behavior/px [Psychology],*Adolescent Behavior,Cigarette Smoking/ep [Epidemiology],Cigarette Smoking/px [Psychology],*Cigarette Smoking/td [Trends],*Electronic Nicotine Delivery Systems,Female,*Health Surveys/td [Trends],Humans,Long<br><br>**Abstract:** OBJECTIVES Noncigarette tobacco products may confer a risk of marijuana use similar to combustible cigarettes. We examined whether adolescent electronic cigarette (e-cigarette), hookah, or combustible cigarette use is associated with initiating and currently using marijuana as well as using both tobacco and marijuana concurrently., METHODS: Adolescents from 10 public schools in Los Angeles, California, completed in-classroom surveys at baseline (fall 2013, ninth grade) and at a 24-month follow-up (fall 2015, 11th grade). Among adolescents who never used marijuana at baseline (N = 2668), associations of baseline e-cigarette, hookah, or combustible cigarette use with ever marijuana use (initiation), current marijuana use (past 30 days), and current dual use of marijuana and these tobacco products at the 24-month follow-up were examined., RESULTS: Baseline ever versus never e-cigarette use was associated with initiation (odds ratio [OR] 3.63; 95% confidence interval [CI] 2.69-4.90) and current (OR 3.67; 95% CI 2.51-5.36) marijuana use 24 months later. Ever versus never hookah use was associated with initiation (OR 3.55; 95% CI 2.49-5.08) and current (OR 4.10; 95% CI 2.69-6.25) marijuana use 24 months later. Similar associations were observed for combustible cigarette smoking and initiation (OR 4.30; 95% CI 2.79-6.63) and current use of marijuana (OR 1.97; 95% CI 1.05-3.68). Current use of any of these tobacco products at baseline was associated with current use of both tobacco and marijuana (OR 2.28; 95% CI 1.47-3.55) 24 months later., CONCLUSIONS: The association between tobacco use and subsequent marijuana use across adolescence extends to multiple tobacco products. Copyright © 2018 by the American Academy of Pediatrics.
3043	M Jankowski, M Krzystanek, J E Zejda, P Majek, J Lubanski, J A Lawson, G Brozek (2019///) E-cigarettes are more addictive than traditional cigarettes-A study in highly educated young people. International Journal of Environmental Research and Public Health; 16<br><br>**Keywords:**<br>adult,article,attitude,cigarette smoking,cross-sectional study,female,health behavior,health survey, human,male,questionnaire,smoking habit,*tobacco dependence,vaping,*cigarette,*electronic cigarette<br><br>**Abstract:** E-cigarettes are often considered less addictive than traditional cigarettes. This study aimed to assess patterns of e-cigarette use and to compare nicotine dependence among cigarette and e-cigarette users in a group of highly educated young adults. From 3002 healthy adults, a representative group of 30 cigarette smokers, 30 exclusive e-cigarette users, and 30 dual users were recruited. A 25-item questionnaire was used to collect information related to the patterns and attitudes towards the use of cigarettes and e-cigarettes. The Fagerstrom test for nicotine dependence (FTND) and its adapted version for e-cigarettes were used to analyze nicotine dependence in each of the groups. The nicotine dependence levels measured with FTND were over two times higher among e-cigarette users (mean 3.5) compared to traditional tobacco smokers (mean 1.6; p<0.001). Similarly, among dual users, nicotine dependence levels were higher when using an e-cigarette (mean 4.7) compared to using traditional cigarettes (mean 3.2; p=0.03). Habits and behaviors associated with the use of e-cigarettes did not differ significantly (p>0.05) between exclusive ecigarette users and dual users. The findings suggest that e-

	cigarettes may have a higher addictive potential than smoked cigarettes among young adults.Copyright © 2019 by the authors. Licensee MDPI, Basel, Switzerland.
3056	A Kulak Jessica, L Saddleson Megan, J Travers Mark, Bansal-Travers Maansi, G Homish Gregory, C Mahoney Martin, A Giovino Gary (2018///) Examining Hookah as an Introduction to Nicotine Products among College Students. Substance use & misuse; 53<br><br>**Keywords:**<br>Adolescent,Electronic Nicotine Delivery Systems/sn [Statistics & Numerical Data],Female,Humans,Male, New York/ep [Epidemiology],Prevalence,Risk Factors,*Smoking/ep [Epidemiology],*Smoking Water Pipes/sn [Statistics & Numerical Data],*Students/sn [Statist<br><br>**Abstract:** BACKGROUND: Limited data exist on what young adults report as their first-ever nicotine product; some evidence suggests that they report hookah as their first product smoked., OBJECTIVES: This study reports on the first nicotine product used among undergraduates who had ever tried tobacco, and explores correlates of hookah as that first product., METHODS: Participants included a convenience sample of undergraduate students (n = 1538) at four universities in upstate New York during fall 2013. Descriptive statistics assessed first nicotine product used and prevalence of current use. Logistic regression was used to examine correlates of hookah as the first nicotine product used., RESULTS: Among the 832 students who reported ever use of any nicotine product, 25.4% reported hookah as their first product smoked; only combustible cigarettes (39.5%) were reported more frequently. Among students who ever smoked cigarettes, most reported cigarettes as their introductory product. Among students who never smoked cigarettes, nearly half reported hookah as their introductory product. Among ever nicotine users, current hookah smoking was common (34.9%), and greater than current e-cigarette (25.9%) and current combustible cigarette (26.4%) use. Never users of cigarettes, females, and non-Hispanic African Americans, had higher adjusted odds of reporting hookah as their introductory product., CONCLUSIONS: The results of this study have implications for the identification of risk factors for tobacco initiation, the assessment of tobacco use patterns and behaviors, and the tailoring of tobacco prevention initiatives among youth. Our findings suggest that broadening prevention efforts beyond a focus on combustible cigarettes may be warranted.
3059	L Barrington-Trimis Jessica, Kong Grace, M Leventhal Adam, Liu Feifei, Mayer Margaret, Cruz Tess Boley, Krishnan-Sarin Suchitra, McConnell Rob (2018///) E-cigarette Use and Subsequent Smoking Frequency Among Adolescents. Pediatrics; 142<br><br>**Keywords:**<br>Adolescent,*Adolescent Behavior/px [Psychology],*Cigarette Smoking/ep [Epidemiology],*Cigarette Smoking/px [Psychology],Cigarette Smoking/td [Trends],Cohort Studies,*Electronic Nicotine Delivery Systems,Female,Humans,Male,Prospective Studies,*Vaping/ep<br><br>**Abstract:** BACKGROUND AND OBJECTIVES Electronic cigarette (e-cigarette) use is associated with cigarette initiation among adolescents. However, it is unclear whether e-cigarette use is associated with more frequent cigarette use after initiation. Also, the extent to which cigarette or dual cigarette and e-cigarette users transition to exclusive e-cigarette use or to the nonuse of either product is not yet known., METHODS: Data were pooled from 3 prospective cohort studies in California and Connecticut (baseline: 2013-2014; follow-up: 2014-2016; N = 6258). Polytomous regression models were used to evaluate the association of baseline e-cigarette use (never or ever) with cigarette use frequency at follow-up (experimental: initiation but no past-30-day use; infrequent: 1-2 of the past 30 days; frequent: 3-5 or more of the past 30 days). Polytomous regression models were also used to evaluate transitions between baseline ever or past-30-day single or dual product use and past-30-day single or dual product use at follow-up., RESULTS: Among baseline never smokers, e-cigarette users had greater odds of subsequent experimental (odds ratio [OR] = 4.58; 95% confidence interval [CI]: 3.56-5.88), infrequent (OR = 4.27; 95% CI: 2.75-6.62) or frequent (OR = 3.51; 95% CI: 1.97-6.24) cigarette use; the 3 OR estimates

	were not significantly different. Baseline past-30-day exclusive cigarette use was associated with higher odds at follow-up of exclusive cigarette or dual product use than of exclusive e-cigarette use., CONCLUSIONS: Tobacco control policy to reduce adolescent use of both e-cigarettes and cigarettes is needed to prevent progression to more frequent tobacco use patterns and reduce combustible cigarette use (with or without concurrent e-cigarette use) to lessen the adverse public health impact of e-cigarettes. Copyright © 2018 by the American Academy of Pediatrics.
3065	Li Jingjing, Haardorfer Regine, Vu Milkie, Windle Michael, J Berg Carla (2018///) Sex and sexual orientation in relation to tobacco use among young adult college students in the US: a cross-sectional study. BMC public health; 18<br><br>**Keywords:**<br>Adolescent,Adult,Cross-Sectional Studies,Electronic Nicotine Delivery Systems/sn [Statistics & Numerical Data],Female,Georgia/ep [Epidemiology],Humans,Longitudinal Studies,Male,Risk Factors,Sex Distribution,*Sexual Behavior/sn [Statistics & Numerical Da<br><br>**Abstract:** BACKGROUND: Sexual minority young adults represent a high-risk population for tobacco use. This study examined cigarette and alternative tobacco product (ATP) use prevalence across sexual orientation (heterosexual, gay/lesbian, and bisexual) among college-attending young adult men and women, respectively., METHODS: Baseline data from a two-year longitudinal study of 3386 young adult college students aged 18-25 in Georgia were analyzed. Correlates examined included sociodemographics (age, sex, sexual orientation, race/ethnicity, college type, and parental education). Outcomes included past 30-day use of tobacco (cigarette, little cigars/cigarillos [LCCs], e-cigarettes, hookah, any tobacco product used, and number of tobacco products used, respectively). Two-group, multivariate multiple regression models were used to examine predictors of tobacco use among men and women, respectively., RESULTS: Among men (N = 1207), 34.7% used any tobacco product; 18.6% cigarettes; 12.3% LCCs; 16.8% e-cigarettes; and 14.7% hookah. Controlling for sociodemographics, gay sexual orientation (OR = 1.62, p = 0.012) was associated with higher odds of cigarette use; no other significant associations were found between sexual orientation and tobacco use. Among women (N = 2179), 25.3% used any tobacco product; 10.4% cigarettes; 10.6% LCCs; 7.6% e-cigarettes; and 10.8% hookah. Being bisexual was associated with cigarette (p < 0.001), LCC (p < 0.001), and e-cigarette use (p = 0.006). Lesbian sexual orientation was associated with cigarette (p = 0.032) and LCC use (p < 0.001). Being bisexual predicted any tobacco product used (p = 0.002), as well as number of tobacco products used (p = 0.004). Group comparisons showed that the effect of sexual minority status on LCC use was significantly different for men versus women., CONCLUSION: Sexual minority women, especially bisexual women, are at higher risk for using specific tobacco products compared to heterosexual women; homosexual men are at increased risk of cigarette use compared to heterosexual men. These nuances in tobacco use should inform interventions targeting sexual minorities.
3069	B Correa John, O Brandon Karen, R Meltzer Lauren, J Hoehn Hannah, Pineiro Barbara, H Brandon Thomas, N Simmons Vani (2018///) Electronic cigarette use among patients with cancer: Reasons for use, beliefs, and patient-provider communication. Psycho-oncology; 27<br><br>**Keywords:**<br>Adult,Aged,Cross-Sectional Studies,Electronic Nicotine Delivery Systems/sn [Statistics & Numerical Data],*Electronic Nicotine Delivery Systems,Female,Florida/ep [Epidemiology],*Health Communication,Humans,Male,Middle Aged,Neoplasms/ep [Epidemiology],*Ne<br><br>**Abstract:** OBJECTIVE: Smoking tobacco cigarettes after a cancer diagnosis increases risk for several serious adverse outcomes. Thus, patients can significantly benefit from quitting smoking. Electronic cigarettes are an increasingly popular cessation method. Providers routinely ask about combustible cigarette use, yet little is known about use and communication surrounding e-cigarettes among patients with cancer. This study aims to describe patterns, beliefs, and communication with oncology providers

	about e-cigarette use of patients with cancer., METHODS: Patients with cancer (N = 121) who currently used e-cigarettes were surveyed in a cross-sectional study about their patterns and reasons for use, beliefs, and perceptions of risk for e-cigarettes, combustible cigarettes, and nicotine replacement therapies. Patient perspectives on provider communication regarding e-cigarettes were also assessed., RESULTS: Most participants identified smoking cessation as the reason for initiating (81%) and continuing (60%) e-cigarette use. However, 51% of patients reported current dual use of combustible cigarettes and e-cigarettes, and most patients reported never having discussed their use of e-cigarettes with their oncology provider (72%). Patients characterized e-cigarettes as less addictive, less expensive, less stigmatizing, and less likely to impact cancer treatment than combustible cigarettes (Ps < .05), and more satisfying, more useful for quitting smoking, and more effective at reducing cancer-related stress than nicotine replacement therapies (Ps < .05)., CONCLUSIONS: Patients with cancer who use e-cigarettes have positive attitudes toward these devices and use them to aid in smoking cessation. This study also highlights the need for improved patient-provider communication on the safety and efficacy of e-cigarettes for smoking cessation. Copyright © 2018 John Wiley & Sons, Ltd.
3071	A L Johnson, L K Collins, A C Villanti, J L Pearson, R S Niaura (2018///) Patterns of nicotine and tobacco product use in youth and young adults in the United States, 2011-2015. Nicotine and Tobacco Research; 20<br><br>**Keywords:**<br>adult,African American,age distribution,article,attitude to health,Black person,Caucasian,child,female, *health education,human,male,prevalence,priority journal,race difference,smokeless tobacco,snus, *tobacco use,trend study,United States,waterpipe tobac<br><br>**Abstract:** Introduction As cigarette smoking has decreased among youth and young adults (YAs) in the United States, the prevalence of other tobacco and nicotine product use has increased. Methods This study identified common past 30-day patterns of tobacco and nicotine product use in youth (grades 6-12) and YAs (aged 18-24). Using data from the 2011-2015 National Youth Tobacco Survey (NYTS) and corresponding years of the Truth Initiative Young Adult Cohort Study (TIYAC), past 30-day use of the following products was assessed: Cigarettes, e-cigarettes, any type of cigar, smokeless tobacco, hookah, and other tobacco products (pipe, bidis, kreteks, dissolvable tobacco, and snus). A user-generated program in R was used to assess all possible combinations of product-specific and polytobacco use. Results The top five patterns of past 30-day use in youth were exclusive cigarette use (12.0%), exclusive cigar use (10.3%), exclusive e-cigarette use (10.0%), dual use of cigarettes and cigars (6.1%), and exclusive hookah use (5.2%). In YAs, the top five patterns were exclusive cigarette use (46.5%), exclusive cigar use (10.0%), dual use of cigarettes and cigars (6.4%), exclusive hookah use (5.9%), and dual use of cigarettes and e-cigarettes (3.9%). Conclusions As noncigarette tobacco and nicotine products become increasingly popular among tobacco users, further research is needed to identify predictors and correlates of specific tobacco use patterns in youth and YAs. This analysis can inform tobacco prevention efforts focusing on emerging tobacco products such as e-cigarettes and hookah. Educational and other intervention efforts should focus on the diversity of products and use patterns in these age groups. Implications This study uses population-based data to provide new information on the most prevalent patterns of past 30-day nicotine and tobacco use over a 5-year period among youth and young adults. Study findings demonstrate that youth and young adults report using tobacco and nicotine products in different combinations, with varying popularity over time. Additionally, by examining young adults as a separate group, this study highlights the unique patterns of use not previously discussed in the adult literature.Copyright © The Author(s) 2018. Published by Oxford University Press on behalf of the Society for Research on Nicotine and Tobacco.
3074	S E Johnson, E K O'Brien, B Coleman, G K Tessman, L Hoffman, J Delahanty (2019///) Sexual and Gender Minority U.S. Youth Tobacco Use: Population Assessment of Tobacco and Health (PATH) Study Wave 3, 2015-2016. American Journal of Preventive Medicine; 57

	**Keywords:**<br>adolescent,*adolescent smoking,article,cigar smoking,cigarette smoking,female,human,male,*sexual and gender minority,smokeless tobacco,*tobacco use,transgender,United States,vaping,water pipe smoking<br><br>**Abstract:** Introduction: Tobacco use disparities by sexual identity are well characterized among adults, but relatively less is known about tobacco use disparities in sexual minority youth. Moreover, no national study has reported tobacco use behaviors among gender minority (e.g., transgender) youth. Method(s): Data from Wave 3 (2015-2016) of the Population Assessment of Health Study from youth aged 14-17 years (n=7,772), analyzed in 2018, were stratified by transgender identity, sex, and sexual identity to describe tobacco product use. Result(s): Transgender youth (n=79) were more likely to report ever use of tobacco products compared with nontransgender youth (electronic nicotine products, 40.2% vs 23.0%; cigarettes, 33.9% vs 14.1%; and smokeless tobacco, 21.5% vs 6.0%, respectively) and more likely to report past 30-day use of these products. Similarly, female sexual minority youth, compared with straight female youth, were more likely to report ever use of electronic nicotine products (37.9% vs 20.1%); cigarettes (28.9% vs 12.0%); cigars (11.3% vs 5.2%); hookah (12.7% vs 7.6%); and past 30-day use of electronic nicotine products, cigarettes, and cigars. Among male youth, sexual minority youth were more likely to report ever use of cigarettes compared with straight youth (25.1% vs 13.5%), but they did not differ in ever or past 30-day use of any other tobacco products. Conclusion(s): Sexual minority youth and transgender youth are more likely to use tobacco products compared with their nonminority peers. Similar to patterns among adults, this is especially true for female sexual minority youth. This information is useful for developing communication and education efforts to prevent youth tobacco initiation in these high-risk groups.Copyright © 2019
3078	Klein Jonathan (2018///) Adolescent electric cigarette use is associated with subsequent marijuana use. The Journal of pediatrics; 200<br><br>**Keywords:**<br>Adolescent,*Electronic Nicotine Delivery Systems,Humans,Longitudinal Studies,Marijuana Smoking, *Marijuana Use,Smoking<br><br>**Abstract:**
3081	M I Jongenelis, E Brennan, T Slevin, C Kameron, D Rudaizky, S Pettigrew (2019///) Differences in use of electronic nicotine delivery systems by smoking status and demographic characteristics among Australian young adults. Health promotion journal of Australia : official journal of Australian Association of Health Promotion Professionals; 30<br><br>**Keywords:**<br>adult,article,*Australian,cigar,controlled study,*disease course,*electronic cigarette,female,*hookah, human,male,nonsmoker,perception,smoking cessation,young adult<br><br>**Abstract:** ISSUE ADDRESSED: Electronic nicotine delivery systems (ENDS) continue to grow in popularity, particularly among young adults. Understanding by whom various ENDS are being used and why within this population segment can assist the development of appropriate interventions targeting those most vulnerable to use and inform policy decisions in this area. Accordingly, this study assessed the demographic characteristics associated with ENDS use among Australian young adults, and reasons for current e-cigarette use. METHOD(S): An online survey was administered to 1116 Australians aged 18-25 years (59% female). RESULT(S): Smokers were more likely than non-smokers to report (a) ever use of ENDS (67% vs 28%), (b) ever use of an e-cigarette (64% vs 24%), e-cigar (17% vs 8%) or e-hookah (15% vs 8%) and (c) current use of e-cigarettes (19% vs 6%). Male smokers were more likely than female smokers to be current e-cigarette users. The most common reason for current e-cigarette use was enjoyment. CONCLUSION(S): Young male adults appear to be particularly vulnerable to becoming regular e-cigarette users. This demographic group may need to be a primary focus of prevention and intervention efforts.

	Contrary to popular belief, smoking cessation was not a primary reason for e-cigarette use. SO WHAT?: Greater efforts are needed to educate young adults on the harms associated with ENDS use, especially if use is being driven by the perception that they are a harmless means of amusement. Maintaining existing regulations limiting the accessibility of these products is crucial to ensuring widespread use is minimised.Copyright © 2018 Australian Health Promotion Association.
3082	M I Jongenelis, E Jardine, C Kameron, D Rudaizky, S Pettigrew (2019///) E-cigarette use is associated with susceptibility to tobacco use among Australian young adults. International Journal of Drug Policy;<br><br>**Keywords:**<br>adult,article,Australia,*Australian,*cigarette smoking,controlled study,*electronic cigarette,female, human,major clinical study,male,regression analysis,smoking,social aspect,tobacco,young adult<br><br>**Abstract:** Background: Calls have been made to relax current Australian regulations related to e-cigarettes to increase the accessibility of the devices for smoking cessation purposes. However, e-cigarettes have been found to increase risk of initiation of conventional cigarette smoking, especially among young adults. To assist in guiding the development of policy in Australia, the present study examined whether e-cigarette use among Australian young adults who have never smoked a tobacco cigarette is associated with susceptibility to future tobacco cigarette use. Method(s): An online web panel provider recruited 519 never smokers aged 18-25 years (55% female; average age = 21.21 years, SD = 2.32). Respondents completed an online survey that assessed their curiosity about tobacco smoking, willingness and intentions to smoke, and a number of individual and social factors. Cross-sectional regression analyses were conducted to assess the association between e-cigarette use and susceptibility to tobacco cigarette use while controlling for multiple covariates. Result(s): Curiosity about tobacco smoking, willingness to smoke, and intentions to smoke were significantly higher among users of e-cigarettes than never users. The relationship between e-cigarette use and susceptibility to future tobacco cigarette use remained significant after controlling for numerous covariates. Conclusion(s): E-cigarette use, even just one or two puffs, has the potential to increase susceptibility to tobacco cigarette use among Australian young adults. Findings suggest that increasing the availability of e-cigarettes by relaxing current strict regulations surrounding their sale may have unintended consequences.Copyright © 2019 Elsevier B.V.
3086	M Braciszewski Jordan, Vose-O'Neal Adam, E Gamarel Kristi, M Colby Suzanne (2019///) Combustible cigarette smoking and alternative tobacco use in a sample of youth transitioning from foster care. Children and Youth Services Review; 96<br><br>**Keywords:**<br>*Foster Care,*Smoking Cessation,*Tobacco Smoking,*Electronic Cigarettes<br><br>**Abstract:** Among the struggles faced by youth currently in or recently exiting foster care, tobacco use remains a low priority for practitioners and researchers, alike. Indeed, despite the dramatically altered landscape of tobacco products on the market, there have been no studies evaluating the use of alternative tobacco products among this vulnerable population. The current study aimed to determine the prevalence of lifetime and current combustible and non-combustible tobacco use among youth exiting foster care, and report on the prevalence of nicotine dependence, motivation to quit, and preferred methods of tobacco cessation. Youth aged 18-24 (M = 20.13, SD = 1.16) who were transitioning from foster care (N = 154) completed a survey of tobacco product use adapted from the Population Assessment of Tobacco and Health Baseline Survey. Most participants (76%) reported lifetime use of combustible cigarettes, while almost half (42%) were current combustible cigarette smokers. Current use of electronic cigarettes was comparable to general population rates. Many participants (76%) reported interest in quitting and willingness to try through patches/gum (56%) and technology-based (61%) approaches. Youth exiting foster care are at high risk for smoking and other tobacco product use, as well as dependence, yet are rarely screened for use or advised to quit. As tobacco use remains among the

	most preventable causes of mortality and morbidity, future work should involve implementation of screening within child welfare and tailoring interventions to the unique needs of this population. The current results underscore a missed opportunity to promote public health in a vulnerable population. (PsycINFO Database Record (c) 2019 APA, all rights reserved)
3087	L Treur Jorien, D Rozema Andrea, J P Mathijssen Jolanda, Oers Hans van, M Vink Jacqueline (2018///) E-cigarette and waterpipe use in two adolescent cohorts: cross-sectional and longitudinal associations with conventional cigarette smoking. European journal of epidemiology; 33<br><br>**Keywords:**<br>Adolescent,*Adolescent Behavior,Cohort Studies,*Electronic Nicotine Delivery Systems/sn [Statistics & Numerical Data],Female,Humans,Longitudinal Studies,Male,Netherlands/ep [Epidemiology],Prevalence, Risk Factors,Schools,*Smoking/ep [Epidemiology],*Stude<br><br>**Abstract:** Alternative tobacco products are increasing in popularity. An important question is whether their use is associated with or even leads to conventional smoking, but large-scale (European) studies are scarce. In two cohorts of Dutch adolescents (Cohort I n = 6819, mean age = 13.8 SD = 1.1, 48.2% female; Cohort II n = 2758, mean age = 17.3 SD = 1.8, 61.3% female), we investigated use of electronic (e)-cigarettes with nicotine, e-cigarettes without nicotine and waterpipe. Generalized estimating equation modelling was conducted with ever conventional smoking as the dependent variable (0 = no, 1 = yes) and ever alternative tobacco use as the independent variable, correcting for clustering within schools, age, sex and education in both cohorts. In a subsample (n = 2100), the association between alternative tobacco use at baseline and conventional smoking 6 months later was tested, taking into account smoking propensity (based on personality, susceptibility to peer pressure and smoking intentions). Ever use prevalence was 13.7% for e-cigarettes with nicotine, 29.4% for e-cigarettes without nicotine and 22.1% for waterpipe in Cohort I and 12.3, 27.6 and 45.3% respectively in Cohort II. Ever smokers had tried alternative tobacco products more often than never smokers. Among never-smoking adolescents at baseline, alternative tobacco use predicted ever smoking 6 months later (e-cigarettes with nicotine OR 11.90 95% CI 3.36-42.11; e-cigarettes without nicotine OR 5.36 95% CI 2.73-10.52; waterpipe OR 5.36 95% CI 2.78-10.31). This association was strongest for adolescents with a low baseline risk of smoking. Experimenting with alternative tobacco products is common among Dutch youth. Alternative tobacco use predicts (future) smoking, especially among adolescents with a low smoking propensity.
3089	Chin Joseph, B Lustik Michael, Pflipsen Matthew (2018///) Prevalence of Use and Perceptions of Electronic Smoking Devices in a US Army Infantry Division. Military medicine; 183<br><br>**Keywords:**<br>Adult,Chi-Square Distribution,Cross-Sectional Studies,Female,Hawaii,Humans,Longitudinal Studies,Male, *Military Personnel/sn [Statistics & Numerical Data],*Perception,*Prevalence,Surveys and Questionnaires,*Vaping/px [Psychology]<br><br>**Abstract:** Introduction: The increasing interest in electronic smoking devices (ESDs) among consumers shows promise for ESDs as a less harmful alternative for smoking and possibly for smoking cessation. Limited data on prevalence of ESD use in the military are available and data on perceptions of ESD use have not yet been published within an active duty, military-specific population. The primary outcome of this study was to define the prevalence of ESD use in a US Army division., Methods: We surveyed 1,288 military service members (response rate 95%, n = 1,225) from the 25th Infantry Division, Schofield Barracks, HI, in late 2015, on smoking habits, ESD usage, and perceptions surrounding ESDs. Chi-squared and Fisher's exact tests were used to assess categorical associations between smoking status, ESD usage, socio-demographic factors, cessation strategies, and perceptions regarding ESDs., Results: The prevalence of current ESD use was 20%, whereas the prevalence of having ever used an ESD was 61%. The daily smoking rate of participants was 23%. Those with lower education, current smokers, and ex-smokers were the most likely to be using an ESD. Current smokers and ESD users had a more favorable

	perception of ESDs with regard to their utility and health effectiveness., Conclusions: There is a high prevalence of current and ever use ESD among active duty service members assigned to a US Army Infantry Division. Data from similar studies suggest that this rate is growing. The rates of ESD use in military populations should continue to be monitored as the clinical utility of these devices continues to be investigated. Copyright Published by Oxford University Press on behalf of the Association of Military Surgeons of the United States 2017. This work is written by (a) US Government employee(s) and is in the public domain in the US.
3091	T Hinds Josephine, Loukas Alexandra, L Perry Cheryl (2018///) Sexual and Gender Minority College Students and Tobacco Use in Texas. Nicotine & tobacco research : official journal of the Society for Research on Nicotine and Tobacco; 20<br><br>**Keywords:**<br>Adolescent,Adult,Electronic Nicotine Delivery Systems,Female,Humans,Male,Prospective Studies,Self Report,Sexual Behavior/px [Psychology],*Sexual and Gender Minorities/px [Psychology],*Students/px [Psychology],Surveys and Questionnaires,Texas/ep [Epidemi<br><br>**Abstract:** Introduction: Most research regarding sexual and gender minority (SGM) populations is limited to examination of cigarette or general tobacco use or does not examine heterogeneity across SGM groups other than lesbian or gay and bisexual individuals. This study examined differences in the odds of current use and age of initiation of five tobacco/nicotine products among three groups of SGM young adults who self-identified as (1) gay or lesbian, (2) bisexual, and (3) queer, transgender, or "other," compared to their heterosexual peers., Methods: Participants were 4252 college students aged 18-29 years from 24 colleges in Texas who completed an online tobacco use survey. Multilevel logistic regression models were used to examine the odds of SGM participants currently using each tobacco product. Multilevel linear regression models were used to examine the association of current product users' SGM status with self-reported age of each product's initiation. All models were adjusted for sociodemographic factors and accounted for students clustered within each college., Results: At least one SGM group had significantly greater odds of currently using every tobacco product type compared to heterosexual participants, except hookah. There were few differences across groups in age of initiation. However, queer, transgender, and "other-" identified participants initiated e-cigarettes 1.34 years younger than heterosexual participants, and bisexual participants initiated smokeless tobacco 3.66 years younger than heterosexual participants., Conclusions: Findings highlight some significant tobacco use disparities among SGM young adults compared to their heterosexual peers. Longitudinal studies with larger group sizes will identify prospective predictors of sustained SGM-related tobacco use disparities., Implications: This study extends the current literature by including the sexual and gender minority identity options of queer, transgender, and "other," highlighting disparities in tobacco use between young adults in these subgroups compared to their heterosexual peers, particularly in noncigarette tobacco product use. Findings underscore the need for the Food and Drug Association and other health agencies to tailor health communication efforts specific to sexual and gender minority populations pertaining to the risks and harms surrounding tobacco product use. Copyright © The Author 2017. Published by Oxford University Press on behalf of the Society for Research on Nicotine and Tobacco. All rights reserved. For permissions, please e-mail: journals.permissions@oup.com.
3094	B Wang Julie, E Olgin Jeffrey, Nah Gregory, Vittinghoff Eric, K Cataldo Janine, J Pletcher Mark, M Marcus Gregory (2018///) Cigarette and e-cigarette dual use and risk of cardiopulmonary symptoms in the Health eHeart Study. PLoS ONE; 13<br><br>**Keywords:**<br>Adult,Asthma/ci [Chemically Induced],*Asthma/ep [Epidemiology],Asthma/pp [Physiopathology], Electronic Nicotine Delivery Systems,Female,Heart/de [Drug Effects],Heart/pp [Physiopathology], Humans,Male,Middle Aged,Pulmonary Heart Disease/ci [Chemically Indu

	**Abstract:** E-cigarettes are promoted as healthier alternatives to conventional cigarettes. Many cigarette smokers use both products. It is unknown whether the additional use of e-cigarettes among cigarette smokers (dual users) is associated with reduced exposure to tobacco-related health risks. Cross-sectional analysis was performed using baseline data from the Health eHeart Study, among English-speaking adults, mostly from the United States. Cigarette use (# cigarettes/day) and/or e-cigarette use (# days, # cartridges, and # puffs) were compared between cigarette only users vs. dual users. Additionally, we examined cardiopulmonary symptoms/ conditions across product use: no product (neither), e-cigarettes only, cigarettes only, and dual use. Among 39,747 participants, 573 (1.4%) reported e-cigarette only use, 1,693 (4.3%) reported cigarette only use, and 514 (1.3%) dual use. Dual users, compared to cigarette only users, reported a greater median (IQR) number of cigarettes per day, 10.0 (4.0-20.0) vs. 9.0 (3.0-15.0) (p < .0001), a lower (worse) median (IQR) SF-12 general health score, 3.3 (2.8-3.8) vs. 3.5 (2.8-3.9) (p = .0014), and a higher (worse) median (IQR) breathing difficulty score in the past month, 2.0 (1.0-2.0) vs. 1.0 (1.0-2.0) (p = .001). Of the 19 cardiopulmonary symptoms/ conditions, having a history of arrhythmia was significantly different between cigarette only users (14.2%) and dual users (17.8%) (p = .02). In this sample, dual use was not associated with reduced exposure to either (i) cigarettes, compared to cigarette only users or (ii) e-cigarettes, compared to e-cigarette only users. E-cigarette only use, compared to no product use, was associated with lower general health scores, higher breathing difficulty scores (typically and past month), and greater proportions of those who responded 'yes' to having chest pain, palpitations, coronary heart disease, arrhythmia, COPD, and asthma. These data suggest the added use of e-cigarettes alone may have contributed to cardiopulmonary health risks particularly respiratory health risks.
3095	Pattinson Julie, Lewis Sarah, Bains Manpreet, Britton John, Langley Tessa (2018///) Vape shops: who uses them and what do they do?. BMC public health; 18<br><br>**Keywords:**<br>Adolescent,Adult,*Commerce/sn [Statistics & Numerical Data],Cross-Sectional Studies,Electronic Nicotine Delivery Systems/ec [Economics],Electronic Nicotine Delivery Systems/sn [Statistics & Numerical Data], Female,Humans,Male,Middle Aged,Smoking Cessatio<br><br>**Abstract:** BACKGROUND: 'Vape shops' are a popular source for buying electronic cigarettes (e-cigarettes) and related products. The products that vape shops sell, their marketing techniques and the extent to which they provide information or encouragement to smokers to quit tobacco use, as well as the patterns of tobacco and e-cigarette use of their customers are not well understood., METHODS: We conducted cross-sectional surveys in vape shops in the East Midlands region of the United Kingdom, one with shop staff (n = 41), and one with customers (n = 197)., RESULTS: The majority of customers (84%) currently used e-cigarettes. Among current vapers, 19% were dual users and 78% had quit smoking. Over half of vapers reported using a lower level of nicotine in their current e-liquid than when they started using e-cigarettes. There was a wide variety in products and price ranges between the shops. Many staff reported that customers ask for information about quitting smoking (90%). Less than half reported providing smoking cessation advice, although 76% of staff reported feeling confident about delivering cessation advice to customers who ask for it. Just under half of customers and shop staff said they thought it was appropriate to deliver formal in-store smoking cessation support., CONCLUSIONS: The majority of vape shop customers are vapers who have quit smoking. Shop staff play a central role in providing customers with product information, and many provide smoking cessation advice. Further research is needed to investigate the potential for smoking cessation interventions in vape shops, including the extent to which these would appeal to non-vapers.
3101	Bunch Kailey, Fu Marcela, Ballbe Montse, Matilla-Santader Nuria, Lidon-Moyano Cristina, Martin-Sanchez Juan Carlos, Fernandez Esteve, M Martinez-Sanchez Jose (2018///) Motivation and main flavour of use, use with nicotine and dual use of electronic cigarettes in Barcelona, Spain: a cross-sectional study. BMJ Open; 8

	**Keywords:** Adolescent,Adult,Aged,*Cigarette Smoking/ep [Epidemiology],Cross-Sectional Studies,Electronic Nicotine Delivery Systems,Female,Humans,Logistic Models,Male,Middle Aged,*Motivation,Multivariate Analysis, *Smokers/px [Psychology],Spain/ep [Epidemiology],Sur<br><br>**Abstract:** OBJECTIVE: This study seeks to describe current e-cigarette users' patterns of use including primary motivation for use, dual use, use with nicotine and principal flavour used, according to individuals' sociodemographic characteristics and conventional tobacco consumption in Barcelona, Spain., DESIGN, SETTING AND PARTICIPANTS: This is a cross-sectional study of adult (>=18) current e-cigarette users of Barcelona (n=600), recruited in 2015. Researchers calculated percentages and ORs with their 95% CIs of users' e-cigarette use with nicotine, primary motivation for use and preferred flavour. The analyses were stratified by sociodemographic variables including sex, age and level of education and by patterns of e-cigarette use and past and current conventional tobacco use., RESULTS: The most prevalent motivation for using e-cigarettes was to reduce tobacco smoking (48%, n=288), followed by quitting smoking (39.2%, n=235), and to use e-cigarettes in places where tobacco smoking was prohibited (10.2%, n=61). The most prevalent primary flavour used was tobacco (49%, n=294), followed by fruit (19.5%, n=117). People younger than 25 years old (OR=4.36, 95% CI 1.99 to 9.57) were more likely than older people to use them where smoking cigarettes was prohibited. A greater proportion of those in the youngest age group preferred fruit flavour compared with older individuals (29.3%, n=41)., CONCLUSIONS: Younger users of e-cigarettes and non-smokers are more likely to use flavours other than tobacco and to use e-cigarettes mainly for recreational purposes. Copyright © Article author(s) (or their employer(s) unless otherwise stated in the text of the article) 2018. All rights reserved. No commercial use is permitted unless otherwise expressly granted.
3102	M Berry Kaitlyn, M Reynolds Lindsay, M Collins Jason, B Siegel Michael, L Fetterman Jessica, M Hamburg Naomi, Bhatnagar Aruni, J Benjamin Emelia, Stokes Andrew (2019///) E-cigarette initiation and associated changes in smoking cessation and reduction: the Population Assessment of Tobacco and Health Study, 2013-2015. Tobacco control; 28<br><br>**Keywords:** Adult,Aged,*Cigarette Smoking/ep [Epidemiology],*Electronic Nicotine Delivery Systems,Female, Humans,Logistic Models,Male,Middle Aged,*Smoking Cessation/sn [Statistics & Numerical Data],Surveys and Questionnaires,United States/ep [Epidemiology],*Vaping/e<br><br>**Abstract:** BACKGROUND: The role of electronic cigarettes (e-cigarettes) in product transitions has been debated., METHODS: We used nationally representative data from the Population Assessment of Tobacco and Health Study waves 1 (2013-2014) and 2 (2014-2015) to investigate the associations between e-cigarette initiation and cigarette cessation/reduction in the USA. We limited the sample to current cigarette smokers aged 25+ years who were not current e-cigarette users at wave 1. We modelled 30-day cigarette cessation and substantial reduction in cigarette consumption as a function of e-cigarette initiation between surveys using multivariable logistic regression., RESULTS: Between waves 1 and 2, 6.9% of cigarette smokers who were not current e-cigarette users transitioned to former smokers. After adjusting for covariates, cigarette smokers who initiated e-cigarette use between waves and reported they used e-cigarettes daily at wave 2 had 7.88 (95% CI 4.45 to 13.95) times the odds of 30-day cigarette cessation compared with non-users of e-cigarettes at wave 2. Cigarette smokers who began using e-cigarettes every day and did not achieve cessation had 5.70 (95% CI 3.47 to 9.35) times the odds of reducing their average daily cigarette use by at least 50% between waves 1 and 2 compared with e-cigarette non-users., CONCLUSIONS: Daily e-cigarette initiators were more likely to have quit smoking cigarettes or reduced use compared with non-users. However, less frequent e-cigarette use was not associated with cigarette cessation/reduction. These results suggest incorporating frequency of e-cigarette use is important for developing a more thorough understanding of the association between e-cigarette use and cigarette cessation. Copyright © Article author(s) (or their employer(s) unless otherwise

	stated in the text of the article) 2019. All rights reserved. No commercial use is permitted unless otherwise expressly granted.
3105	E Karakas, A Zumbul, T Balataci, R Durusoy, G Yararbas, O Basoglu, C Gurgun (2018///) Smoking status of medical students at Ege University: A cross-sectional survey of 1040 students in 2018. Tobacco Induced Diseases; 16<br><br>**Keywords:**<br>adolescent,controlled study,*disease course,education program,electronic cigarette,female,friend, *hookah,human,major clinical study,male,medical school,*medical student,prevalence,questionnaire,risk factor,smoke,smoking cessation,stress,Turkey (republic<br><br>**Abstract:** Introduction We aimed to determine the smoking prevalence and related factors among 1,2,3 and 6th grade students at Ege University Faculty of Medicine. Methods This cross-sectional survey was a part of a multi-centre study throughout Turkey and the data were collected in May-June 2018. The target group of this study was 1537 medical students studying in the first, second, third and sixth grades. Questionnaires were applied to 1040 students (67.7%) who agreed to participate in the study. The coverage was 94.5%, 78.6%, 57.4%, 36.7% for the first, second, third and sixth grades, respectively. Results The overall prevalence of smoking was 18.4%, with 18.1%, 18.6%, 17.0% and 22.1% in the first, second, third and sixth grades, respectively. The mean age at starting to smoke was 16.5+/-2.3 yrs (min.8, max.23). Current smoking was more prevalent in males compared to females (24.3% vs. 11.7%, p<0.001). Among current smokers, 27.4% had started smoking during medical school. The most common reasons for starting to smoke were the effects of close friends (37.1%) and stress (19.2%). Current smokers spend 201+/-139 Turkish Liras (min.0, max.800) monthly for cigarettes. Among smokers, 60.4% have tried to quit smoking at least once, and 78% of them thought of smoking cessation. Among e-cigarette users, 93.6% were ever-smokers. According to univariate analyses; male students, students whose parents, partners or best friends were eversmokers, students living alone and the ones who have smoked hookah at least once have significantly higher prevalence of being ever-smokers compared to their counter-parts. In multivariate analyses; having the hookah use (OR:12.4), living alone at home (OR:3.4), partner (OR:2.3) or best friend (OR:1.9) smoking, and were found to be independent risk factors related to being an eversmoker. Conclusions The present study shows that the prevalence of smoking in especially male medical students was high (24.3%) in Turkey, and nearly one third of the students started smoking during medical faculty. Therefore, there is a need to include intensive education programs regarding smoking-related health problems and smoking cessation at an early stage in the medical curriculum.
3108	A Kasza Karin, Coleman Blair, Sharma Eva, P Conway Kevin, K Michael Cummings, L Goniewicz Maciej, S Niaura Raymond, Y Lambert Elizabeth, M Schneller Liane, P Feirman Shari, A Donaldson Elisabeth, Cheng Yu-Ching, Murphy Iilun, L Pearson Jennifer, R Trinidad Dennis, Bansal-Travers Maansi, Elton-Marshall Tara, A Gundersen Daniel, A Stanton Cassandra, B Abrams David, T Fong Geoffrey, Borek Nicolette, M Compton Wilson, J Hyland Andrew (2018///) Correlates of Transitions in Tobacco Product Use by U.S. Adult Tobacco Users between 2013-2014 and 2014-2015: Findings from the PATH Study Wave 1 and Wave 2. International Journal of Environmental Research and Public Health; 15<br><br>**Keywords:**<br>Adolescent,Adult,Age Factors,Electronic Nicotine Delivery Systems,Female,Humans,Male,Middle Aged, Research Design,Sex Factors,Sexuality,Socioeconomic Factors,Tobacco,*Tobacco Products/cl [Classification],*Tobacco Use/ep [Epidemiology],*Tobacco Use Disord<br><br>**Abstract:** More than half of adult tobacco users in the United States (U.S.) transitioned in tobacco product use between 2013-2014 and 2014-2015. We examine how characteristics of adult tobacco users in the U.S. relate to transitions in tobacco product use. Population Assessment of Tobacco and Health (PATH) Study data were analyzed from 12,862 adult current tobacco users who participated in Wave 1 (W1, 2013-2014) and Wave 2 (W2, 2014-2015). Three types of transitions were examined-(1) adding

	tobacco product(s); (2) switching to non-cigarette tobacco product(s); and (3) discontinuing all tobacco use-among those currently using: (1) any tobacco product; (2) cigarettes only (i.e., exclusive cigarette); and (3) cigarettes plus another tobacco product(s) (i.e., poly-cigarette). Multinomial logistic regression analyses determined relative risk of type of transition versus no transition as a function of demographic and tobacco use characteristics. Transitions in tobacco product use among adult tobacco users were common overall, but varied among different demographic groups, including by age, sex, sexual orientation, race/ethnicity, educational attainment, and poverty level. Further, cigarette smokers with higher dependence scores were more likely to add product(s) and less likely to discontinue tobacco use compared to those with low dependence scores. That high nicotine dependence is a barrier to discontinuing tobacco use adds evidence to support policy to lower nicotine content of cigarettes and to evaluate new products for their potential to reduce cigarette use.
3109	A Kasza Karin, Borek Nicolette, P Conway Kevin, L Goniewicz Maciej, A Stanton Cassandra, Sharma Eva, T Fong Geoffrey, B Abrams David, Coleman Blair, M Schneller Liane, Y Lambert Elizabeth, L Pearson Jennifer, Bansal-Travers Maansi, Murphy Iilun, Cheng Yu-Ching, A Donaldson Elisabeth, P Feirman Shari, Gravely Shannon, Elton-Marshall Tara, R Trinidad Dennis, A Gundersen Daniel, S Niaura Raymond, K Michael Cummings, M Compton Wilson, J Hyland Andrew (2018///) Transitions in Tobacco Product Use by U.S. Adults between 2013-2014 and 2014-2015: Findings from the PATH Study Wave 1 and Wave 2. International Journal of Environmental Research and Public Health; 15<br><br>**Keywords:**<br>Adult,Aged,Electronic Nicotine Delivery Systems/sn [Statistics & Numerical Data],Humans,Middle Aged, Prevalence,*Tobacco Products/sn [Statistics & Numerical Data],*Tobacco Use/ep [Epidemiology], Tobacco, Smokeless/sn [Statistics & Numerical Data],United S<br><br>**Abstract:** In 2013-2014, nearly 28% of adults in the United States (U.S.) were current tobacco users with cigarettes the most common product used and with nearly 40% of tobacco users using two or more tobacco products. We describe overall change in prevalence of tobacco product use and within-person transitions in tobacco product use in the U.S. between 2013-2014 and 2014-2015 for young adults (18-24 years) and older adults (25+ years). Data from Wave 1 (W1, 2013-2014) and Wave 2 (W2, 2014-2015) of the Population Assessment of Tobacco and Health (PATH) Study were analyzed (N = 34,235). Tobacco product types were categorized into: (1) combustible (cigarettes, cigars, pipe tobacco, hookah), (2) noncombustible (smokeless tobacco, snus pouches, dissolvable tobacco), and (3) electronic nicotine delivery systems (ENDS). Transitions for individual combustible-product types, and for single- and multiple-product use, were also considered. Overall prevalence of current tobacco use decreased from 27.6% to 26.3%. Among W1 non-tobacco users, 88.7% of young adults and 95.8% of older adults were non-tobacco users at W2. Among W1 tobacco users, 71.7% of young adults transitioned, with 20.7% discontinuing use completely, and 45.9% of older adults transitioned, with 12.5% discontinuing use completely. Continuing with/transitioning toward combustible product(s), particularly cigarettes, was more common than continuing with/transitioning toward ENDS. Tobacco use behaviors were less stable among young adults than older adults, likely reflecting greater product experimentation among young adults. Relative stability of cigarette use compared to other tobacco products (except older adult noncombustible use) demonstrates high abuse liability for cigarettes.
3119	H Kathuria, R G Seibert, V Cobb, N Herbst, Z M Weinstein, M Gowarty, R Jhunjhunwala, E D Helm, R S Wiener (2019///) Perceived barriers to quitting cigarettes among hospitalized smokers with substance use disorders: A mixed methods study. Addictive Behaviors; 95<br><br>**Keywords:**<br>adult,alcohol consumption,article,controlled study,disease association,disease predisposition,disease severity,*drug dependence,educational status,female,heroin dependence,high risk patient, homelessness,hospital admission,*hospital patient,human,income,

	**Abstract:** Aims: Smoking cessation may promote long-term recovery in patients with substance use disorders (SUD). Yet smoking rates remain alarmingly high in this population. Using a sequential explanatory mixed methods approach, we examined smoking rates among hospitalized patients with SUD at a large safety-net hospital, and then characterized factors associated with smoking behaviors both quantitatively and qualitatively. Method(s): We abstracted data from all hospital admissions (7/2016-6/2017) and determined demographics, substance use type, and other characteristics associated with cigarette use among those with SUD. We then conducted semi-structured qualitative interviews with 20 hospitalized SUD smokers. We analyzed transcripts to characterize factors that affect patients' smoking habits, focusing on the constructs of the Health Belief Model. Result(s): The prevalence of cigarette smoking among hospitalized smokers with SUD was three times higher than those without SUD. Qualitative analyses showed that patients perceived that smoking cigarettes was a less serious concern than other substances. Some patients feared that quitting cigarettes could negatively impact their recovery and perceived that clinicians do not prioritize treating tobacco dependence. Almost all patients with heroin use disorder described how cigarette use potentiated their heroin high. Many SUD patients are turning to vaping and e-cigarettes to quit smoking. Conclusion(s): Hospitalized patients with SUD have disproportionately high smoking rates and perceive multiple barriers to quitting cigarettes. When designing and implementing smoking cessation interventions for hospitalized patients with SUD, policymakers should understand and take into account how patients with SUD perceive smoking-related health risks and how that influences their decision to quit smoking.Copyright © 2019 Elsevier Ltd
3121	M G Katz, K W Russell (2019///) Injury from e-cigarette explosion. New England Journal of Medicine; 380<br><br>**Keywords:**<br>adolescent,*blast injury/su [Surgery],case report,clinical article,comminuted fracture/su [Surgery], computer assisted tomography,debridement,explosion,human,jaw pain,laceration,male,mandible fracture/su [Surgery],mandibular central incisor,mandibular la<br><br>**Abstract:**
3128	Choi Kelvin, Bestrashniy Jessica, Forster Jean (2018///) Trends in Awareness, Use of, and Beliefs About Electronic Cigarette and Snus Among a Longitudinal Cohort of US Midwest Young Adults. Nicotine & tobacco research : official journal of the Society for Research on Nicotine and Tobacco; 20<br><br>**Keywords:**<br>Adult,*Behavior, Addictive,*Electronic Nicotine Delivery Systems/sn [Statistics & Numerical Data], Female,*Health Knowledge, Attitudes, Practice,Humans,Longitudinal Studies,Male,Minnesota/ep [Epidemiology],Prevalence,Smoking/ep [Epidemiology],*Tobacco Us<br><br>**Abstract:** Introduction: Few longitudinal studies have examined how awareness, use of, and beliefs about electronic cigarettes (e-cigarettes) and snus change over time. We assessed these trends in a cohort of young adults from the US Midwest., Methods: Data were from the Minnesota Adolescent Community Cohort (MACC) Study, collected annually during 2010-2013 when participants were 21-29 years old (n = 2622). Participants were asked if they had heard of and ever used e-cigarettes and snus, and the number of days they used these products in the past 30 days. Beliefs about whether these products are less harmful than cigarettes, less addictive than cigarettes, and could help people quit smoking were assessed. Repeated measures multiple linear and logistic regression models, adjusting for demographics, peer smoking and smoking status, were used to assess trends., Results: Compared to 2010-2011, participants in 2012-2013 were five times more likely to be aware of e-cigarettes, report ever used them, and report using them in the past 30-days. Increases in e-cigarette use were observed in all smoking status. Participants were also increasingly likely to believe that e-cigarettes are less harmful than combustible cigarettes and could help people quit smoking. There was only a modest increase in awareness of and ever using snus, as well as believing snus is less harmful than combustible cigarettes. These trends did not differ by smoking status., Conclusions: The increasingly favorable beliefs about e-

	cigarettes may explain the increasing prevalence of their use particularly among young adults, both among smokers and nonsmokers., Implications: Awareness and use of e-cigarettes have increased substantially over the past few years, and positive beliefs of e-cigarettes have also become more prevalent among young adults. Meanwhile, little changes in awareness, use of, and beliefs about snus among young adults. Given the potential of these products to have both positive and negative impact on public health depending on who use them and how they are used, strategically communicating the risks associated with e-cigarettes and snus use may help minimize the burden of tobacco use at the population level. Copyright Published by Oxford University Press on behalf of the Society for Research on Nicotine and Tobacco 2017. This work is written by (a) US Government employee(s) and is in the public domain in the US.
3135	J Kim, S Lee (2018///) Smoking prevalence and smoking behavior change among the soldiers in the Korean military. Tobacco Induced Diseases; 16<br><br>**Keywords:**<br>adult,air force,*behavior change,*cigarette smoking,controlled study,electronic cigarette,health promotion,human,major clinical study,male,*military service,navy,*prevalence,smoking,*soldier,stress, conference abstract<br><br>**Abstract:** Background: Republic of Korea has an obligatory military service. All Korean men in their 20s are obliged to serve the military for 21 months. The purpose of this study was to identify smoking prevalence among the Korean military and to understand how the soldiers change their smoking behavior during their obligated military service. Method(s): A tobacco use survey for the military was carried out by the Korean Association of Health Promotion on behalf of the Ministry of Health in July, 2016. The participants were asked to answer the questions regarding current and past experience of tobacco use, their smoking behavior change during the military service. Result(s): The total number of participants in the survey were 3,800 soldiers. Among them, 44.9% (1,707 soldiers) of the participants were current smokers. Marine Corps had the highest smoking prevalence with 51.7%, following Army with 48.5%, Navy with 39.1% and Air force with 32.7%. The branches of the military were associated with soldiers' smoking prevalence (p< .001). There were 726 participants (63.5%) who answered that their smoking frequency has increased in the military service. The reasons being; tough training or stress of the military life (72.6%, 527 soldiers). In addition, the participants answered that in order to have a break, they smoked more often. There was a large group of dual users (34.9%) between cigarettes and electronic cigarettes. The rank was associated with dual use of cigarettes and electronic cigarettes (p=.007). Conclusion(s): The smoking prevalence among soldiers were higher compared to that of the same age group in the population. The military life has affected the young soldiers' smoking behavior. Effective tobacco control intervention should be employed in the military and this will contribute to the decrease in the smoking prevalence among Korean males.
3139	B A King, M R Creamer, M Harrell, S Kelder, L Norman, C L Perry (2018///) Surgeon General's Reports Ontobacco: A continued legacy of unbiased and rigorous synthesis of the scientific evidence. Nicotine and Tobacco Research; 20<br><br>**Keywords:**<br>*evidence based medicine,exposure,*health care personnel,health care policy,health hazard,health program,human,medical information,note,outcome assessment,priority journal,smoking,smoking cessation,smoking cessation program,*tobacco,tobacco industry,Uni<br><br>**Abstract:**
3141	J M Kinnunen, H Ollila, J Minkkinen, P L Lindfors, D S Timberlake, A H Rimpela (2019///) Nicotine matters in predicting subsequent smoking after e-cigarette experimentation: A longitudinal study among Finnish adolescents. Drug and Alcohol Dependence; 201

	**Keywords:**<br>adolescent,article,cohort analysis,controlled study,female,Finn (citizen),follow up,high school student, human,longitudinal study,male,outcome assessment,population research,*prediction,priority journal, sample size,*smoking,substance use,tobacco dependen<br><br>**Abstract:** Background: Several studies indicate an association between e-cigarette use and subsequent smoking among youth. However, most previous studies lack measures of the nicotine content of e-liquid and have not usually measured regular smoking. Method(s): We tested the association between e-cigarette use, with and without nicotine, and subsequent daily use of conventional cigarettes and nicotine e-cigarettes among study population of 3474 students. A survey was conducted in lower secondary schools of the Helsinki metropolitan area, Finland, with 15 - 16-year-olds in 2014 (baseline) and in upper secondary schools in 2016 when the cohort was 17 - 18-year-olds (follow-up). Firth logistic regression and generalized linear mixed models (GLMM) were used. Result(s): Of students, 25% had experimented with nicotine e-cigarettes at baseline and 40% at follow-up. Among baseline never-smokers, experimentation with or use of nicotine e-cigarettes predicted the uptake of daily smoking at follow-up (AOR 2.92; 95% CI 1.09-7.85), but baseline experimentation with non-nicotine e-cigarettes did not when compared with the non-e-cigarette experimenters. Nicotine e-cigarette experimentation at baseline predicted daily nicotine e-cigarette use at follow-up (AOR 2.96; 95% CI 1.22-7.22). Non-nicotine e-cigarette experimentation at baseline did not predict statistically significantly daily nicotine e-cigarette use at follow-up (AOR 3.13; 95% CI 0.98-10.02). The small number of cases may have diminished the statistical significance. Conclusion(s): The findings suggest that experimentation with nicotine e-cigarettes serves as a gateway to subsequent use of conventional cigarettes as well as nicotine e-cigarettes. Our results support the actions to limit youths' access to e-cigarettes in order to prevent nicotine addiction.Copyright © 2019 The Authors
3152	G Kong, M E Mayer, J L Barrington-Trimis, R McConnell, A M Leventhal, S Krishnan-Sarin (2019///) Longitudinal associations between use and co-use of cigars and cigarettes: A pooled analysis of three adolescent cohorts. Drug and Alcohol Dependence; 201<br><br>**Keywords:**<br>adolescent,*adolescent smoking,article,*cigar smoking,*cigarette smoking,cohort analysis,controlled study,early intervention,female,follow up,geographic distribution,human,longitudinal study,male,priority journal,prospective study,public policy,risk ass<br><br>**Abstract:** Introduction: Patterns of adolescent tobacco product use are evolving rapidly and need examination. We assessed whether ever use of cigars (i.e., lifetime use) was related to an increased risk of subsequent cigarette initiation and dual use of cigars and cigarettes. Method(s): Leveraging data from three prospective cohort studies of adolescents (n = 6258), we assessed the odds of initiating cigarettes at one-year follow-up among ever cigar users at baseline, relative to never cigar users, after adjusting for demographics and e-cigarette use. We also assessed patterns of transition between exclusive use of cigars, exclusive use of cigarettes, and dual use of both cigars and cigarettes between baseline and follow-up and whether these associations differed by e-cigarette use. Result(s): Among never cigarette smokers (n = 4876; 79.3% of the total sample), 3.4% reported ever cigar use by baseline. Ever cigar use by baseline was associated with higher likelihood of initiating cigarettes by follow-up (31.3%) relative to never cigar use at baseline (8.4%; adjusted odds ratio = 2.26, 95% confidence interval: 1.52, 3.35). Effect estimates were stronger if e-cigarette was used by baseline. Furthermore, exclusive ever cigar use by baseline was associated with a 2-4-fold increase in the odds of transition to exclusive cigar, exclusive cigarette, and dual use at follow-up relative to non-users of either product by baseline. Conclusion(s): Comprehensive tobacco regulations and early prevention efforts focused on reducing youth appeal of cigars may be warranted, as cigar use may place youth at risk for subsequent cigarette use as well as dual use of cigars and cigarettes.Copyright © 2019 Elsevier B.V.

3154	E Farsalinos Konstantinos, Siakas Georgios, Poulas Konstantinos, Voudris Vassilis, Merakou Kyriakoula, Barbouni Anastasia (2018///) Electronic cigarette use in Greece: an analysis of a representative population sample in Attica prefecture. Harm Reduction Journal; 15<br><br>**Keywords:**<br>Adolescent,Adult,Cross-Sectional Studies,*Electronic Nicotine Delivery Systems/sn [Statistics & Numerical Data],Female,Greece/ep [Epidemiology],Harm Reduction,Humans,Male,Middle Aged,Surveys and Questionnaires/sn [Statistics & Numerical Data],*Vaping/ep<br><br>**Abstract:** BACKGROUND: The purpose was to assess prevalence and correlates of electronic cigarette (e-cigarette) use in Greece in 2017., METHODS: A cross-sectional survey of a representative sample of 4058 adults living in Attica prefecture (35% of the Greek adult population) was performed in May 2017 through telephone interviews. Prevalence and frequency of e-cigarette use were assessed according to the smoking status, and logistic regression analysis was performed to identify correlates of use., RESULTS: Current smoking was reported by 32.6% of participants. Ever e-cigarette use was reported by 54.1% (51.4-56.8%) of current smokers, 24.1% (21.7-26.5%) of former smokers and 6.5% (5.3-7.7%) of never smokers. Past experimentation was the most prevalent pattern of e-cigarette use among ever users (P < 0.001). Almost 80% of ever and 90% of current e-cigarette users were using nicotine. Extrapolated to the whole Attica population (3.1 million), there were 1 million current smokers, 848,000 ever e-cigarette users and 155,000 current e-cigarette users. The majority of current e-cigarette users (62.2%) were former smokers. Only 0.2% of never smokers were current e-cigarette users. One out of 20 participants considered e-cigarettes a lot less harmful than smoking. Being current or former smoker were the strongest correlates current e-cigarette use (OR 30.82, 95%CI 10. 21-69.33 and OR 69.33, 95%CI 23.12-207.90 respectively)., CONCLUSIONS: E-cigarette use in Greece is largely confined to current or former smokers, while current use and nicotine use by never smokers is extremely rare. The majority of current e-cigarette users were former smokers. Most participants overestimate the harmfulness of e-cigarettes relative to smoking.
3155	Farsalinos Konstantinos (2018///) Measuring aldehyde emissions in e-cigarettes and the contribution of flavors: A response to Khlystov and Samburova. Food and chemical toxicology : an international journal published for the British Industrial Biological Research Association; 120<br><br>**Keywords:**<br>*Aldehydes/an [Analysis],*Electronic Nicotine Delivery Systems/is [Instrumentation],Humans,Taste<br><br>**Abstract:**
3158	S D Kowitt, A O Goldstein, E L Sutfin, A Osman, C Meernik, C Heck, L M Ranney (2019///) Adolescents' first tobacco products: Associations with current multiple tobacco product use. PLoS ONE; 14<br><br>**Keywords:**<br>adolescent,article,controlled study,cross-sectional study,demography,ethnicity,female,health hazard,high school student,human,human cell,male,prevalence,smokeless tobacco,social psychology,*tobacco use, waterpipe tobacco,cigar,cigarette,electronic cigare<br><br>**Abstract:** Understanding which tobacco products adolescents use first can lead to insights for tobacco prevention interventions and policies. We used cross-sectional data from high school students who reported ever using a tobacco product from the 2017 North Carolina Youth Tobacco Survey (n = 1,053). In multivariable regressions, we examined how demographic and psychosocial factors were associated with adolescents' first product tried and how first product tried was associated with current tobacco use (i.e., no use, use of a single product, use of multiple products) and frequency of tobacco use. Cigarettes (34.8%) and e-cigarettes (33.7%) were the most frequently reported first products tried, followed by cigars (15.6%), smokeless tobacco (10.7%), waterpipe (4.0%), and other tobacco products (i.e., pipe

	tobacco or some other tobacco product) (1.2%). Demographic differences in adolescents' first product tried existed, with Black adolescents having higher odds of initiating tobacco use via cigars (aOR: 6.17, 95% CI: 3.75, 10.14). Adolescents who initiated tobacco use via cigars (aOR: 2.33, 95% CI: 1.31, 4.13) or smokeless tobacco (aOR: 2.45, 95% CI: 1.18, 5.04) had higher odds of being a multiple current tobacco product user, whereas adolescents who initiated tobacco use via e-cigarettes (aOR: 0.57, 95% CI: 0.34, 0.93) had lower odds of being a multiple current tobacco product user. Additionally, adolescents who initiated tobacco use via smokeless tobacco had higher odds of currently using at least one tobacco product frequently (aOR: 1.90, 95% CI: 1.04, 3.48), while adolescents who initiated tobacco use via e-cigarettes had lower odds of currently using at least one tobacco product frequently (aOR: 0.40, 95% CI: 0.23, 0.70). These findings suggest that most adolescents reported initiating tobacco use via cigarettes or e-cigarettes and that trying certain products first (e.g., cigars, smokeless tobacco) was associated with higher odds of multiple current tobacco product use.Copyright © 2019 Kowitt et al. This is an open access article distributed under the terms of the Creative Commons Attribution License, which permits unrestricted use, distribution, and reproduction in any medium, provided the original author and source are credited.
3164	A L Kristjansson, J P Allegrante, J Sigfusson, I D Sigfusdottir (2019///) Do population trends in adolescent electronic cigarette use coincide with changes in prevalence of cigarette smoking?. Preventive Medicine Reports; 15<br><br>**Keywords:**<br>adolescent,*adolescent smoking,article,*cigarette smoking,cross sectional study,female,health care survey,human,Iceland,major clinical study,male,population research,priority journal,*vaping,*electronic cigarette<br><br>**Abstract:** Adolescents who initiate electronic cigarette (EC) use without having ever used tobacco are more likely than those that have not initiated EC use to try cigarette smoking over time. However, whether rates of EC use coincide with cigarette smoking rates at the population level remains unknown. This study aimed to compare trends in ever, current, and daily use of EC and cigarette smoking among adolescents in Iceland from 2015 to 2018. We analyzed four waves of pooled cross-sectional population-based school survey data with students enrolled in the 8th, 9th and 10th grades in the national Icelandic school system (n = 42,440, boys = 50.1%). Response rates ranged between 83.3% and 86.0%. Findings for 8th, 9th, and 10th grade students, and the combined sample, revealed a consistent pattern: prevalence of cigarette smoking either remained unchanged or decreased minimally over the study period. In the combined sample rates of ever smoking remained between 9 and 10% during the entire study period, whereas the prevalence of daily cigarette smoking was around 1%. The use of EC increased 2- to 3-fold in all age groups. Ever use increased from 12% to roughly 30% in the combined group and daily use increased from about 2% to 6% during the same period. We conclude that the prevalence of adolescent cigarette smoking in Iceland remained mostly unchanged between the years 2015 and 2018, whereas EC use increased exponentially during the same period. The prevalence of EC use now far outweighs cigarette smoking in 8th-10th grade youth in Iceland.Copyright © 2019 The Authors
3173	A N Kurti, J Y Bunn, A C Villanti, C A Stanton, R Redner, A A Lopez, D E Gaalema, N J Doogan, A Cepeda-Benito, M E Roberts, J K Phillips, A J Quisenberry, D R Keith, S T Higgins (2018///) Patterns of single and multiple tobacco product use among us women of reproductive age. Nicotine and Tobacco Research; 20<br><br>**Keywords:**<br>adult,article,attitude to health,cohort analysis,controlled study,female,gestational age,human, longitudinal study,pregnant woman,prevalence,priority journal,*reproductive health,retrospective study, self report,*tobacco use,United States,*Women's Health,<br><br>**Abstract:** Introduction Understanding patterns of single and multiple tobacco product use among reproductive-aged women is critical given the potential for adverse health effects on mother and infant

	should a woman become pregnant. Methods Patterns of tobacco use over a 2-year period were examined among all women (18-44 years) who completed wave 1 (W1) and wave 2 (W2) of the US Population Assessment of Tobacco and Health (PATH, 2013-2014, 2014-2015) Study. We examined the most common patterns of single and multiple tobacco product use in W1, and longitudinal trajectories of women engaged in each of these patterns of use from W1 to W2, among women not pregnant in either wave (n = 7480), not pregnant in W1 and pregnant in W2 (n = 332), and pregnant in W1 and not pregnant in W2 (n = 325). Results The most prevalent patterns of tobacco use in W1 among all three subgroups were using cigarettes alone followed by using cigarettes plus e-cigarettes. In all three subgroups, women using multiple products in W1 were more likely to adopt new use patterns in W2 relative to single-product users, with the new patterns generally involving dropping rather than adding products. The majority of multiple product use included cigarettes, and transitions to single product use typically involved dropping the noncigarette product. The most common trajectory among tobacco users transitioning to or from pregnancy was to use cigarettes alone in W2. Discussion This study contributes new knowledge characterizing tobacco use patterns across time and reproductive events among reproductive-aged women.Copyright © The Author(s) 2018. Published by Oxford University Press on behalf of the Society for Research on Nicotine and Tobacco.
3176	G Kypriotakis, J D Robinson, C E Green, P M Cinciripini (2018///) Patterns of tobacco product use and correlates among adults in the Population Assessment of Tobacco and Health (PATH) Study: A latent class analysis. Nicotine and Tobacco Research; 20<br><br>**Keywords:**<br>adult,age distribution,aged,article,combustion,disease severity,female,food and drug administration, household income,human,major clinical study,male,middle aged,policy,poverty,priority journal, smokeless tobacco,smoking cessation,social status,tobacco de<br><br>**Abstract:** Introduction As the tobacco industry and market evolves, there is a growing need to understand the patterns of use of tobacco products and how they relate to demographics, dependency, withdrawal, and quit behavior. Methods We analyzed data from wave 1 of the PATH Study consisting of 14856 individuals. Current users were defined as consuming at least 1 of 10 tobacco products. We performed a latent class analysis (LCA) to identify patterns of tobacco use. We used multinomial regression analysis to explore the association between these patterns with covariates representing socioeconomic status dependence/addiction, past quit attempts, and withdrawal severity. Results We identified four groups of current tobacco users with distinct profiles: (1) 61% of the sample were identified as cigarette-only users; (2) 9% were smokeless tobacco users; (3) 17% of the sample were characterized by being current users of all types of combustible tobacco including cigars, cigarillos, filtered cigars, and smoking a pipe (4) finally, 13% were e-cig and hookah users. All classes also shared a varying frequency of cigarette use. Exclusive cigarette users were more likely to be older and female, and experienced higher dependency and withdrawal. Users of e-cigs and hookah were the younger, most educated of all four subgroups, and presented the lowest dependency and withdrawal among the four groups. Conclusions FDA policy makers may want to discourage the use of tobacco products associated with higher tobacco dependency, and products that may contribute to experiencing higher levels of withdrawal symptoms by the user when trying to quit. Implications We identified four patterns of tobacco product use that are significantly related to demographic characteristics, dependency, and withdrawal. Policies should target users more likely to use tobacco products that increase dependency and withdrawal, making quitting more difficult.Copyright © The Author(s) 2018. Published by Oxford University Press on behalf of the Society for Research on Nicotine and Tobacco.
3191	K R Laurene, G Kodukula, R Fischbein, D R Kenne (2019///) What is the draw? Assessing a risk model and conventional exploration model of e-cigarette use. Journal of Substance Use; 24<br><br>**Keywords:**<br>addiction,adult,article,attitude,controlled study,female,heart disease,human,major clinical study,male,

	online system,perception,policy,priority journal,*risk assessment,sensation seeking,United States, university,cigarette,*electronic cigarette<br><br>**Abstract:** Background/Objective: The use of electronic cigarettes (e-cigarettes) among young adults has been increasing, leading to concern due to unknown health effects. The purpose of the current study is to replicate and expand research on two contrasting models of e-cigarette use: risk model and conventional exploration model. Method(s): An anonymous online survey was distributed at a midwestern university in fall 2015. There were 3,626 respondents, aged between 18 and 25, who answered the study's measures: sensation seeking, risk perception, public use attitudes, and policy views. Result(s): Respondents were categorized into mutually exclusive groups: nonuser (58.8%), e-cigarette only user (24.2%), cigarette only user (2.7%), and dual user (13.2%). Lending support to the risk model, higher disinhibition and endorsing e-cigarette use in public was associated with lower odds of being nonusers compared to e-cigarette only users. Support for the conventional model was found with higher perceived likelihood of getting heart disease, a risk of harm, and a risk of addiction due to e-cigarette use being associated with higher odds of being nonusers compared to e-cigarette only users. Conclusion(s): Results provide some support for both models. Findings can be used to inform prevention and intervention programs and services.Copyright © 2018, © 2018 Taylor & Francis Group, LLC.
3194	Hoffman Leah, Delahanty Janine, E Johnson Sarah, Zhao Xiaoquan (2018///) Sexual and gender minority cigarette smoking disparities: An analysis of 2016 Behavioral Risk Factor Surveillance System data. Preventive Medicine; 113<br><br>**Keywords:**<br>Adult,Behavioral Risk Factor Surveillance System,*Cigarette Smoking/ep [Epidemiology],Female,Humans, Male,Middle Aged,Prevalence,Public Health,*Sexual and Gender Minorities/sn [Statistics & Numerical Data],United States/ep [Epidemiology],*Vaping/ep [Epid<br><br>**Abstract:** We examined the association between lesbian, gay, bisexual, and transgender (LGBT) identity, cigarette and e-cigarette use, and potential risk factors in the United States. Using data from 198,057 adults in 26 states in the 2016 Behavioral Risk Factor Surveillance System (BRFSS), we estimated the prevalence of cigarette use, e-cigarette use, and potential risk factors by gender identity and sexual identity. Overall and sex-stratified bivariate and multivariate logistic regressions examined whether the relationship between sexual and gender identity and cigarette and e-cigarette use persisted after adjusting for demographics, socio-economic status, and other unhealthy behaviors. After adjusting for covariates, gender minority identity was no longer associated with increased likelihood of currently smoking cigarettes and ever use of e-cigarettes. Sexual minority identity continued to be significant after adjusting for covariates, indicating that sexual identity disparities in cigarette and e-cigarette use are not fully explained by these factors. Findings varied by identity. Compared to their straight peers, likelihood of tobacco product use among LGB individuals varied between sexes, by product, and by sexual identity (gay/lesbian versus bisexual). More research is needed to understand the mechanisms that influence diverse patterns of cigarette and e-cigarette use among sexual and gender minority adults. Copyright © 2018 The Authors. Published by Elsevier Inc. All rights reserved.
3196	E L S Leavens, E M Stevens, E I Brett, E T Hebert, A C Villanti, J L Pearson, T L Wagener (2019///) JUUL electronic cigarette use patterns, other tobacco product use, and reasons for use among ever users: Results from a convenience sample. Addictive Behaviors; 95<br><br>**Keywords:**<br>adult,article,cigarette smoking,correlational study,exposure,female,human,male,risk factor,smoking habit,*tobacco use,United States,vaping,*electronic cigarette<br><br>**Abstract:** Introduction: JUUL, an e-cigarette from PAX Labs, has captured 70% of the e-cigarette market. The current study examines JUUL use patterns and reasons for initiation in a large convenience sample of

	U.S. adults. Method(s): Respondents were 979 U.S. adults registered on Amazon Mechanical Turk (MTurk) who reported ever using JUUL. Items included frequency/quantity of JUUL use, reasons for trying JUUL, flavor preferences, and use of other tobacco products. Result(s): The majority of participants reported only trying JUUL once or twice (59.5%), 29.2% reported regular nondaily use and 10.3% reported daily use. The average quantity of JUUL pod use was low in the overall sample (4 pods per month). Daily users reported using ~10 pods per month and engaging in 4-9 separate vaping sessions per day. The most frequently reported reasons for JUUL use were because friends were using it (26.5%), curiosity (20.5%), and similarity to a cigarette (7.7%). Approximately 26% of current JUUL users reported current exclusive JUUL use, while 56% reported using JUUL and another e-cigarette. Of the entire sample, 37.1% were former smokers. Of those, 14.9% were daily JUUL users, 21.4% were nondaily JUUL users, and 63.8% were JUUL triers. Conclusion(s): This is the first study to examine patterns and reasons for use of the most popular e-cigarette on the market. In this convenience sample, nearly 40% of those who ever tried JUUL reported current daily or daily use. JUUL use may be associated with limited puffing patterns compared to earlier generation e-cigarettes. Research is needed to investigate if JUUL puffing patterns result in decreased exposure to potentially harmful non-nicotine e-liquid constituents compared to other e-cigarettes.Copyright © 2019
3199	A N Lee, Y S Yang, J E Lee, J H Kong, P H Sun, M Y Kim, A H Park, J H Kim (2018///) Factors associated with ever-use of e-cigarette among college students in the Republic of Korea. Tobacco Induced Diseases; 16<br><br>**Keywords:**<br>adult,binge drinking,cigarette smoking,*college student,controlled study,disease course,*electronic cigarette,father,female,gender,human,major clinical study,male,questionnaire,smoking prevention, *South Korea,young adult,conference abstract<br><br>**Abstract:** Background: Since e-cigarette was introduced into the global market, its use has become popular especially among young adults. But little is known about the prevalence and associated factors of e-cigarette use among young adults in Korea. The purpose of this study is to identify the factors associated with ever-use of e-cigarette among Korean college students. Method(s): A survey to collect the data for this study was distributed to 550 students in 5 universities in S city and K city from October to December 2016. Data from the survey which consists of self-reporting questionnaires were analyzed using binary logistic regression analysis. Ever-use of e-cigarette was assessed by asking "Have you ever used an e-cigarette?" (yes/no). The final sample for this study (n=399) included those who had ever heard of e-cigarette. Result(s): Among 399 students who had ever heard of e-cigarette, 68(17%) were e-cigarette ever-users. E-cigarette ever-use was higher among male students(OR=6.82, 95% CI=2.02, 23.02, p< 0.05), students living in dormitory(OR=10.19, 95% CI=1.16, 89.49, p< 0.05), whose father is a smoker(OR=58.41, 95% CI=8.70, 392.39, p< .001), who have the experience of past 2-week binge drinking(OR=43.78, 95% CI=5.61, 341.93, p< .001) and past 30-day cigarette smoking(OR=1.02, 95% CI=1.01, 1.03, p< .001). Conclusion(s): This study showed significant association between e-cigarette ever-use and gender, residence type, father's smoking status, past 2-week binge drinking and past 30-day cigarette smoking. Associated factors of e-cigarette use should be considered for smoking prevention program for college students. To better understand the patterns and determinants of e-cigarette use among young adults in Korea, further studies are needed.
3202	J Lee, M Oh (2019///) The moderating effect of gender on the association between E-cigarette use and smoking status: A cross-sectional study. Addictive Behaviors; 93<br><br>**Keywords:**<br>adult,article,behavioral risk factor surveillance system,controlled study,*cross sectional study,*disease course,female,human,major clinical study,male,monitoring,*sex difference,*smoking cessation,Texas, *vaping<br><br>**Abstract:** Introduction: The novel features of e-cigarettes in the recent vaping boom are appealing to

	females. Given increasing concerns about using e-cigarettes among females, understanding the patterns of using e-cigarettes in females compared to males is critical. We investigate the moderating effect of gender on the association between ever vaping and smoking status and gender differences in reason for ever vaping and in having ever stopped smoking. Method(s): We used population data (n = 38,661) from the 2015, 2016, and 2017 Texas Behavioral Risk Factor Surveillance System (BRFSS) to examine a gender difference in ever vaping associated with smoking status. Additionally, we restricted the sample to current smokers who have ever vaped (n = 4733) to examine the association of gender with quitting smoking as reason for ever vaping and with having ever stopped smoking. Result(s): We found a significant association between ever vaping and gender (OR = 0.77, 95%CI = 0.65, 0.91). When smoking status was considered, female never-smokers had a significantly lower likelihood of ever vaping than male never-smokers (OR = 0.43, 95%CI = 0.27, 0.76). No significant gender difference was found in ever vaping to quit smoking (OR = 1.12, 95%CI = 0.73, 1.74) and having ever stopped smoking (OR = 1.41, 95%CI = 0.95, 2.10). Conclusion(s): The findings of this study provide evidence of gender differences in vaping patterns. Female never-smokers are less likely than male never-smokers to experiment with e-cigarettes. However, there is no evidence of a gender difference in both ever vaping to quit smoking and having ever stopped smoking. Consistent monitoring of gender differences in using e-cigarettes and stratified policy approaches tailored for genders are necessary.Copyright © 2019 Elsevier Ltd
3204	K S Lee, H Gu (2018///) The association of electronic vapor product use and conventional cigarette smoking with Korean adolescents' oral health. Tobacco Induced Diseases; 16<br><br>**Keywords:**<br>adolescent,adolescent smoking,awareness,chi square test,*cigarette smoking,controlled study,drinking, eating,education program,electronic cigarette,female,gender,*health behavior,human,juvenile,major clinical study,male,mouth hygiene,questionnaire,school<br><br>**Abstract:** Background: Smoking is more harmful to the physical health of adolescents than it is for adults. The demand for studies on electronic cigarettes as a substitute for tobacco has increased, but there is a lack of research on its harmful effects on the oral health. Therefore, we investigated the characteristics of conventional cigarette smoking, electronic cigarette use, and the dual use among adolescents and examined the relationship between oral health factors according to smoking patterns. Method(s): The subjects of this study were 65,528 adolescents who completed a questionnaire of the 12th online survey of the youth health behaviors(2016). The types of smoking were analyzed by conventional cigarette smoking, electronic cigarette use, and nonsmoking. A chi-square test and logistic regression analysis were performed. Result(s): There were significant differences in general characteristics such as gender, grade, school type, scholastic performance, location, perceived socioeconomic status, and drinking status according to type of smoking. There were significant difference in the number of brushing days, oral hygiene auxiliary products and treatment, and subjective oral health awareness. There was a significant difference also in oral disease symptoms. The relationship between type of smoking and oral health was as follows: "Chipped or broken tooth", "Tooth pain when eating", or "Aching or throbbing in tooth", it was significantly higher in dual users and conventional cigarette only smoker, and it was higher in dual users in conventional cigarette only smoker. Conclusion(s): It was found that there was a difference in oral health effects according to the type of smoking. Tailored education programs and policies should be developed to promote oral health and prevent smoking in adolescents.
3206	P N Lee, K J Coombs, E F Afolalu (2018///) Considerations related to vaping as a possible gateway into cigarette smoking: an analytical review. F1000Research; 7<br><br>**Keywords:**<br>adolescent,article,cohort analysis,confounding variable,female,human,juvenile,male,population health, prevalence,prospective study,*vaping<br><br>**Abstract:** Background: Toxicant levels are much lower in e-cigarettes than cigarettes. Therefore,

	introducing e-cigarettes into the market seems likely to reduce smoking-related diseases (SRD). However, vaping might provide a gateway into cigarette smoking for those who otherwise would never have smoked, a concern fueled by cohort studies showing vaping predicts subsequent smoking initiation in young people. Method(s): In this discussion paper, we consider various aspects of the gateway issue in youths. We provide a descriptive critical review of results from prospective studies relating to the gateway effect and the extent to which the studies considered other potential confounding variables associated with smoking initiation. We then estimate the effects of omitting a confounding variable, or misclassifying it, on the association between vaping and subsequent smoking initiation, and determine how the prevalence of smoking might be affected by any true gateway-in effects of vaping. Finally, we examine trends in e-cigarette and smoking prevalence in youths based on national surveys. Result(s): First, we demonstrate that although studies report that vaping significantly predicts smoking initiation following adjustment for various other predictors, the sets of predictors considered are quite incomplete. Furthermore, no study considered residual confounding arising from inaccurate measurement of predictors. More precise adjustment may substantially reduce the association. Second, we show any true gateway effect would likely affect smoking prevalence only modestly. Third, we show smoking prevalence in U.S. and U.K. youths in 2014-2016 declined somewhat faster than predicted by the preceding trend; a substantial gateway effect suggests the opposite. Finally, we argue that even if some gateway effect exists, introducing e-cigarettes still likely reduces SRDs. Conclusion(s): We have shown that the existence of any true gateway-in effect in youth is not yet clearly demonstrated and that the population health impact of introducing e-cigarettes is still likely to be beneficial.
3236	N E Lisha, J Thrul, P M Ling (2019///) Latent Class Analysis to Examine Patterns of Smoking and Other Tobacco Products in Young Adult Bar Patrons. Journal of Adolescent Health; 64<br><br>**Keywords:**<br>adolescent,article,cigarette smoking,comparative study,comprehension,female,human,*latent class analysis,major clinical study,male,priority journal,risk factor,smokeless tobacco,*smoking,social behavior,*tobacco,cigar,cigarette,electronic cigarette,hook<br><br>**Abstract:** Purpose: Use of multiple tobacco products is increasing, particularly among young adults. Latent class analysis of substance-use patterns provides a framework for understanding the heterogeneity of use. We sought to identify different patterns of cigarette, e-cigarette, hookah, cigarillo, and smokeless tobacco use among young adult bar patrons. Method(s): We conducted repeated cross-sectional surveys of randomized time location samples of young adult California bar patrons in 2013 and 2014. Latent class analysis was used to examine patterns of use among current (past 30-day) tobacco users. Classes were compared on demographic characteristics and tobacco use correlates. Result(s): Overall 84.4% of the current tobacco users were cigarette smokers, 38.7% used electronic cigarettes, 35.9% used hookah, 30.1% smoked cigars/cigarillos, and 15.4% used smokeless tobacco in the past 30 days. We extracted six latent classes: "Cigarette only" (n = 1690), "Hookah mostly" (n = 479), "High overall use" (n = 528), "Smokeless mostly" (n = 95), "E-cigarette mostly" (n = 439), "Cigars mostly" (n = 435). These classes differed in their risk profiles on both current use compared to no use, and number of days they used each tobacco product. Differences between classes emerged on demographics (age, sex, race/ethnicity) and tobacco correlates including perceived peer smoking, antitobacco industry attitudes, prioritizing social activities, and advertising receptivity. Conclusion(s): Understanding different patterns of multiple tobacco product use may inform both prevention and cessation programming for young adults. It may be efficient to tailor messages to different latent classes and address the distinct demographic and attitudinal profiles of groups of multiple tobacco product users.Copyright © 2018 Society for Adolescent Health and Medicine
3237	B Liu, G Xu, S Rong, D A Santillan, M K Santillan, L G Snetselaar, W Bao (2019///) National Estimates of e-Cigarette Use among Pregnant and Nonpregnant Women of Reproductive Age in the United States, 2014-2017. JAMA Pediatrics; 173

	**Keywords:**<br>adult,*age,age distribution,cigarette smoking,controlled study,ex smoker,female,human,letter,major clinical study,*pregnant woman,prevalence,priority journal,*reproduction,United States,*vaping<br><br>**Abstract:**
3239	K Llanes, J A Cabriales, N Hernandez, T V Cooper (2019///) Electronic cigarette use after the adoption of a tobacco-free campus policy. Addictive Behaviors; 90<br><br>**Keywords:**<br>adolescent,adult,aged,alcohol consumption,article,college drinking,community college,cross-sectional study,e-mail,female,groups by age,human,male,online system,prevalence,risk factor,*smoking,*smoking ban,student attitude,substance use,tobacco use,Unite<br><br>**Abstract:** Electronic (e)-cigarette use has increased markedly across groups in the past few years. For this reason, risk factors associated with e-cigarette use warrant further research. This study presents secondary data analyses on e-cigarette use from a large cross-sectional database assessing attitudes toward a tobacco-free campus (TFC) policy prior to policy implementation (n = 1188), one-month post policy (n = 1442), and one-year post policy(n = 1125). Students from a U.S. university located on the border with Mexico (Mage = 25.02 years, SD = 7.99; 59.2% female) were recruited via email to complete an online assessment of their tobacco use, attitudes toward TFC policies, perceived problematic tobacco use, and knowledge of tobacco use risk. The prevalence of any past-30-day e-cigarette use significantly increased from 4.4% to 26.6% between baseline and one-month post-policy, and reduced to 17.3% between one-month post-policy and one-year post policy. Weekly alcohol use was associated with e-cigarette use at each assessment point. There was some evidence of higher e-cigarette use among younger students and current smokers. Additional studies are needed to understand the influence of comprehensive TFC policies on both e-cigarette use and polysubstance use (i.e., alcohol and combustible cigarettes).Copyright © 2018 Elsevier Ltd
3246	J J Lorza Blasco (2019///) Emerging Trends in Nicotine Consumption. Archivos de Bronconeumologia; 55<br><br>**Keywords:**<br>allergic pneumonitis,article,asbestosis,asthma,bronchiolitis,cancer risk,cerebrovascular accident,chemical composition,chronic obstructive lung disease,*health hazard,human,infectious pneumonia,interstitial lung disease,interstitial pneumonia,Langerhans<br><br>**Abstract:**
3247	A Loukas, C N Marti, M R Creamer, C L Perry (2018///) Does use of electronic nicotine delivery systems predict current cigarette use among young adults?. Tobacco Induced Diseases; 16<br><br>**Keywords:**<br>adolescent,adult,cigar,*cigarette smoking,college,*electronic cigarette,ethnicity,female,friend,growth curve,Hispanic,hookah,human,major clinical study,male,race,student,Texas,tobacco use,young adult, conference abstract<br><br>**Abstract:** Background: Studies indicate that use of electronic nicotine delivery systems (ENDS) predicts subsequent cigarette use, but these are limited by a focus primarily on adolescents or examination of cigarette initiation/onset. Little is known about the impact of ENDS use in current (past 30-day) cigarette use or in changes in current use across young adulthood. This study examined if 1) ENDS use predicted trajectories of young adult current cigarette use over a two-year period. Method(s): Data are from a five-wave 24-college study in Texas, USA. Wave 1 data were collected in Nov 2014-Feb 2015 and every six months thereafter. To examine the role of ENDS in current cigarette use, only students who reported

	never using cigarettes at Wave 1 were included, N=2577. At Wave 1, students were 18-25 year olds (m=19.7; sd=1.60); 67.7% female; 31.9%; non-Hispanic white, 27.4% Hispanic, and 40.7% another race/ethnicity. Growth curve modeling was used to examine predictors of the current cigarette use trajectory across the five waves. Result(s): Wave 1 ever ENDS use predicted a faster increase in the current cigarette use trajectory (OR= 2.87 [1.32, 6.25]) in the unconditional model (model with no covariates). However, this effect became non-significant when Wave 1 time-invariant covariates, including socio-demographics, family tobacco use, friend cigarette use, cigarette use susceptibility, and use of other tobacco (i.e., hookah, cigars, smokeless) products were added. Examination of the full model indicated that peer use (OR=1.44 [1.01, 2.06]), susceptibility to cigarette use (OR=4.49 [2.84, 7.10]), and other tobacco use (OR=2.40 [1.62, 3.56]) predicted an increasing cigarette use trajectory. Conclusion(s): Findings are in contrast to research indicating the ENDS use predicts cigarette initiation, but also extend it by showing that young adults who are susceptible to cigarette use and who use other tobacco products (besides ENDS) are at elevated risk for current cigarette use.
3248	A Loukas, C N Marti, C L Perry (2019///) Trajectories of Tobacco and Nicotine Use Across Young Adulthood, Texas, 2014-2017. American journal of public health; 109<br><br>**Keywords:**<br>adult,*adulthood,article,cigar,*cigarette smoking,college,controlled study,electronic cigarette,female, growth curve,hookah,human,major clinical study,male,*smokeless tobacco,snus,spring,student,*Texas, young adult,*nicotine<br><br>**Abstract:** OBJECTIVES: To examine intraindividual change in polytobacco and individual tobacco and nicotine product use across young adulthood. METHOD(S): Participants were 2711 students from 24 Texas colleges participating in a 6-wave online study, with 6 months between each wave. Participants were aged 18 to 25 years at baseline in fall 2014 or spring 2015 and aged 20 to 28 years at wave 6. We used growth curve modeling for an accelerated longitudinal design to examine change from ages 18 to 28 years in polytobacco use (use of 2 or more products) and in use of 5 individual products (cigarettes, smokeless tobacco or snus, large cigars, cigarillos, or little cigars, hookah, and electronic nicotine delivery systems [ENDS]). RESULT(S): There was a statistically significant decline in polytobacco use from ages 18 to 28 years. There were also statistically significant declines in ENDS, hookah, and cigar use but not in smokeless tobacco use, for which use was negligible, or in cigarette use. Importantly, cigarettes were the most used product at virtually all ages. CONCLUSION(S): Young adults may mature out of polytobacco use with increasing age, but they may continue to use some products, most notably cigarettes, potentially the most toxic and addictive tobacco and nicotine product.
3249	K Y Low, S J Jag Singh, H S Mohd Hashim, C Lim, N I Junazli, S L Choo, M Munisamy (2018///) Prevalence and characteristics of smoking among school-going adolescents in kuala lumpur, Malaysia. Journal of Global Oncology; 4<br><br>**Keywords:**<br>adolescent,*adolescent smoking,adult,air quality,analysis of variance,cardiovascular disease,controlled study,cross-sectional study,death,disease course,*Federal Territory of Kuala Lumpur,female,gender,high school,hookah,human,independent variable,lung<br><br>**Abstract:** Background: According to the World Health Organization (WHO) (2014), noncommunicable diseases (NCDs) such as cancer, cardiovascular disease and respiratory disease account for a majority ofpremature deaths in the Malaysian population. Tobacco smokingremainsa leadingrisk factor for these diseases. Although there have been previous population-level studies which have highlighted adolescent smokers in Malaysia, little has been done to specifically ascertain smoking prevalence within a school-going population using objective measurement tools. Aim(s): This study aimed to identify the prevalence of school-going adolescent smokers in Kuala Lumpur via an objective measurement-expired carbon monoxide (CO) in parts per million (ppm). Method(s): This study was a cross-sectional study that involved

	2,679 high school-going adolescents aged between 13 to 18 years old in Kuala Lumpur, Malaysia. The level of exhaled CO (Smokerlyzer test) of all the participants was measured. Participants were also administered with a survey conducted right after the Smokerlyzer test to determine i) whether they smoked or not; ii) type of tobacco products used if smoked; iii) vaping or shisha usage; and iv) exposure to secondhand smoke. The categorization of smoking status was based on CO-ppm reading: # 3 ppm (nonsmoker); 3, ppm # 6 (light smoker or nonsmoker breathing in poor air quality or passive smoke inhalation); $ 7 ppm (regular smoker with higher levels of CO in blood). Descriptive analysis was conducted to determine the demographics of the study population. Inferential analysis was carried out utilizing x2 tests or Student t-tests to test associations between smoking status and other independent variables. One-way ANOVA test was conducted to compare the differences in CO-ppm readings. Result(s): The mean age of the adolescents (n52,679) was 14.5 6 1.2 years old (median: 14) and 55.1% were male. Interestingly, vaping was the most commonly used method of tobacco use among the studied adolescents, regardless of gender, followed by cigarettes and shisha. The mean CO-ppm reading for male adolescents was significantly higher than female adolescents. Passive smoke inhalation (exposure to secondhand smoke) was significantly associated with higher readings of CO-ppm. A significant association was determined between status of exposure to secondhand smoke and a history of usage of any form of tobacco products. Conclusion(s): The prevalence of smoking adolescents, regardless of gender, in Kuala Lumpur remains at a worrying state. Objective measurements such as Smokerlyzer test can be coupled with self-reported questionnaire in targeting adolescent smokers within a structured setting such as a school and can help form part of an effective school-based smoking cessation intervention program targeting adolescents in Malaysia. This is in line with efforts to make Malaysia a smoke-free nation by 2045.
3252	Popova Lucy, Owusu Daniel, R Weaver Scott, B Kemp Catherine, C K Mertz, F Pechacek Terry, Slovic Paul (2018///) Affect, risk perception, and the use of cigarettes and e-cigarettes: a population study of U.S. adults. BMC public health; 18<br><br>**Keywords:**<br>Adult,*Affect,*Electronic Nicotine Delivery Systems/sn [Statistics & Numerical Data],Female,Humans, Male,Middle Aged,Risk Assessment,Smoking/ep [Epidemiology],*Smoking/px [Psychology],*Tobacco Products/sn [Statistics & Numerical Data],United States/ep [E<br><br>**Abstract:** BACKGROUND: Tobacco companies argue that the decision to smoke is made by well-informed rational adults who have considered all the risks and benefits of smoking. Yet in promoting their products, the tobacco industry frequently relies on affect, portraying their products as part of a desirable lifestyle. Research examining the roles of affect and perceived risks in smoking has been scant and non-existent for novel tobacco products, such as electronic cigarettes (e-cigarettes)., METHODS: We examined the relationship between affect, perceived risk, and current use for cigarettes and e-cigarettes in 2015 in a nationally representative sample of 5398 U.S. adults who were aware of e-cigarettes., RESULTS: Participants held various affective associations with tobacco products, and affect towards cigarettes was more negative than affect towards e-cigarettes. Using structural equation modeling (SEM), affect towards cigarettes and e-cigarettes was associated with cigarette smoking and e-cigarette use respectively, and these associations were both direct and partially mediated by risk perceptions towards smoking and e-cigarette use. More positive affect towards cigarettes or e-cigarettes was associated with lower perceived risks, which in turn was associated with higher odds of being a current cigarette or e-cigarette user., CONCLUSIONS: In developing models explaining tobacco use behavior, or in creating public communication campaigns aimed at curbing tobacco use, it is useful to focus not only on the reason based predictors, such as perceptions of risks and benefits, but also on affective predictors. Educational efforts aimed at further smoking reductions should highlight and reinforce negative images and associations with cigarettes.
3254	Zavala-Arciniega Luis, Reynales-Shigematsu Luz Myriam, Lozano Paula, Rodriguez-Andrade Miguel Angel, Arillo-Santillan Edna, F Thrasher James (2018///) Patterns of awareness and use of electronic cigarettes in

	Mexico, a middle-income country that bans them: Results from a 2016 national survey. Preventive Medicine; 116<br><br>**Keywords:**<br>Adolescent,Adult,Age Factors,*Awareness,Cross-Sectional Studies,*Developing Countries,*Electronic Nicotine Delivery Systems/sn [Statistics & Numerical Data],Female,Humans,Male,Mexico/ep [Epidemiology],Middle Aged,Prevalence,Sex Factors,Smokers/sn [Stati<br><br>**Abstract:** Among high-income countries, awareness and use of e-cigarettes is lower in countries with more restrictive e-cigarette regulations. Little is known about e-cigarettes in middle-income countries, many of which like Mexico, ban e-cigarette sales and marketing. The current study determined the national-level prevalence and correlates of e-cigarette awareness and consumption in Mexico. Data were analyzed from a 2016 nationally representative survey. Prevalence of e-cigarette awareness, trial and current use was estimated separately for adolescents (n=12,436), adult nonsmokers (n=36,966), and adult smokers (n=7347). For each group, crude and adjusted logistic models regressed e-cigarette outcomes on sociodemographic and smoking-related variables, adjusting for the sampling design and weights. Prevalence varied across subgroups for e-cigarette awareness (adolescents=45.3%; adult nonsmokers=33.9%; adult smokers=54.3%), e-cigarette trial (adolescents=6.5%; adult nonsmokers=2.6%; adult smokers=18.2%) and current use of electronic cigarettes (adolescents=1.1%; adult nonsmokers=0.3%; adult smokers=4.5%). Among adolescents and adults, current smoking and higher smoking frequency were significantly associated with e-cigarette awareness, trial and use. Among adolescents and nonsmokers, awareness, trial and use of e-cigarette were significantly lower among females than males. Among adult smokers, however, females were more likely to use e-cigarettes than males (AOR=1.80; 95% CI=1.08-3.02). Higher education and greater wealth increased the likelihood of trial of e-cigarettes among adult nonsmokers and smokers. Despite the ban on the distribution and sales of e-cigarettes, a substantial number of Mexicans, have access to e-cigarettes. Decision makers and advocates should consider e-cigarette regulations as recommended by the WHO, at the same time Mexico must invest in the infrastructure needed to enforce these regulations. Copyright © 2018 Elsevier Inc. All rights reserved.
3263	H Mamudu, L Wang, D Owusu, C Robertson, C Collins, M Littleton (2019///) Prospective study of dual use of e-cigarettes and other tobacco products among school-going youth in rural Appalachian Tennessee. Annals of Thoracic Medicine; 14<br><br>**Keywords:**<br>adolescent,article,child,child protection,*cigarette smoking,cohort analysis,education,female,health disparity,health promotion,human,major clinical study,male,marketing,preschool child,prevalence, prospective study,questionnaire,rural area,school child,<br><br>**Abstract:** INTRODUCTION: E-cigarettes have emerged as the most commonly used tobacco or nicotine products among youth in the United States (US), and usage with other products (dual use) is not well understood. This study assessed dual use and identified associated factors of usage in school-going youth in the high tobacco burdened region of rural Appalachian Tennessee. METHOD(S): Two waves of data for the same cohort of students were collected in 2014 (Wave 1) and 2016 (Wave 2). Dual use of e-cigarettes with any other tobacco product was the dependent variable. The independent variables consisted of exposure to tobacco use at home, home smoking rules, smoking inside the vehicle, attitudes toward smoking, exposure to tobacco industry marketing/promotion, and peer/family pressure. Descriptive statistics and multivariate logistic regression analyses were conducted to determine the prevalence of dual use and delineate factors associated with usage. RESULT(S): Dual use increased from 13.3% in Wave 1 to 18.6% in Wave 2. Results of logistic regression show that exposure to tobacco industry marketing/promotion was significantly associated with dual use in the two waves, odds ratio (OR) = 4.18 (confidence interval [CI] = 1.69-10.38) and OR = 4.43 (CI = 2.03-9.67), respectively. While allowing

	smoking inside the vehicles, sometimes, significantly increased dual use in Wave 1 (OR = 3.18 [CI = 1.19-8.48]), exposure to tobacco use at home (OR = 2.94 [1.24-6.97]), and peer/family pressure (OR = 2.92 [1.87-7.19]) increased usage in Wave 2. CONCLUSION(S): The increasing trend in dual use of e-cigarettes and other tobacco products among youth in Appalachia Tennessee suggests a critical need for comprehensive tobacco control programs to avert exacerbating disparities in tobacco use and tobacco-related diseases in the US.Copyright © 2019 Annals of Thoracic Medicine Published by Wolters Kluwer - Medknow.
3265	Yu Mansoo, Sacco Paul, Choi Hye Jeong, Wintemberg Jenna (2018///) Identifying patterns of tobacco use among US middle and high school students: A latent class analysis. Addictive Behaviors; 79<br><br>**Keywords:**<br>Adolescent,African Americans/sn [Statistics & Numerical Data],Asian Americans/sn [Statistics & Numerical Data],Child,*Cigar Smoking/ep [Epidemiology],*Cigarette Smoking/ep [Epidemiology],*Ethnic Groups/sn [Statistics & Numerical Data],European Continent<br><br>**Abstract:** OBJECTIVES: This study sought to (a) understand patterns of tobacco use among US middle and high school students based on their lifetime or current use of tobacco products: cigarettes, cigars, chewing tobacco, pipes, e-cigarettes, and hookah tobacco, and (b) examine differences in the underlying patterns by sociodemographic and tobacco-related characteristics (e.g., exposure to tobacco products, tobacco advertising, parental disapproval of tobacco use and feedback from healthcare providers)., METHODS: We analyzed self-report data from the 2013 National Youth Tobacco Survey (n=18,046). Latent Class Analysis was conducted to identify patterns of tobacco use and tested how sociodemographic and tobacco-related characteristics are associated with such patterns., RESULTS: Four subtypes of tobacco use were identified: "Very Low Risk (VLR)," "Lifetime Smoking (LS)," "Lifetime Multiple Tobacco Use (LMT)," and "Past Month Multiple Tobacco Use (PMT)." Latino and Asian American youth were more likely to be in PMT. Compared with students in LS, students in VLR were less likely to receive advice not to use tobacco from their healthcare providers, but more likely to have parental disapproval of smoking. Students in LMT (vs. LS) were more likely to use tobacco coupons and promotional materials, report greater access to tobacco, and receive advice from a healthcare professional. Students in PMT (vs. LS) were more likely to use coupons, take part in tobacco promotions, and be exposed to smoking in a vehicle., CONCLUSIONS: Our study suggests that it may be important to consider the underlying patterns and correlates of tobacco use in designing tobacco control programs for youth. Copyright © 2017 Elsevier Ltd. All rights reserved.
3270	C Fahey Margaret, Bursac Zoran, O Ebbert Jon, C Klesges Robert, A Little Melissa (2019///) Prevalence and correlates of dual tobacco use in cancer survivors. Cancer causes & control : CCC; 30<br><br>**Keywords:**<br>Adult,Aged,*Cancer Survivors/sn [Statistics & Numerical Data],Cross-Sectional Studies,*Electronic Nicotine Delivery Systems/sn [Statistics & Numerical Data],Female,Humans,Male,Middle Aged,Physicians, Prevalence,Smoking/ep [Epidemiology],*Smoking Cessatio<br><br>**Abstract:** PURPOSE: Tobacco use in cancer survivors remains a significant problem, however, the use of non-cigarette tobacco products (NCTPs) in this population is less understood. This study examined prevalence and correlates of tobacco use among cancer survivors who were never, current, and former cigarette users. Tobacco-related behaviors and quitting attitudes were compared between survivors dually using cigarettes and electronic cigarettes (ECs) and cigarette-only users., METHODS: In this cross-sectional study, patients at Mid-South cancer centers (n = 629; 50.4% never, 17.8% current, and 31.8% former cigarette users) responded to an anonymous questionnaire about tobacco-related behaviors and quitting attitudes., RESULTS: Among current cigarette users, 27.7% reported using two or more tobacco products. Most commonly, 15.2% of cigarettes users were using ECs. Compared to cigarette only use, dual use of tobacco products was associated with male gender (p < 0.0001), being single (p = 0.009), and

	a lung cancer diagnosis (p < 0.0001). Dual users of cigarettes and ECs were more likely to report a readiness to quit cigarettes within 6 months (p = 0.0317) and that a physician recommended ECs as a quit resource (p = 0.0361) compared to cigarette-only users., CONCLUSIONS: Results suggest that using more than one tobacco product is common among cancer survivor cigarette users. Dual use of cigarettes and ECs was associated with an increased readiness for cigarette cessation and a physician recommendation of ECs. Targeting potential dual use of tobacco products, particularly cigarettes and ECs, might be beneficial for cigarette cessation among cancer survivors.
3281	U Martinez, V Martinez-Loredo, V N Simmons, L R Meltzer, D J Drobes, K O Brandon, A M Palmer, T Eissenberg, C R Bullen, P T Harrell, T H Brandon (2019///) How Does Smoking and Nicotine Dependence Change after Onset of Vaping? A Retrospective Analysis of Dual Users. Nicotine & tobacco research : official journal of the Society for Research on Nicotine and Tobacco;<br><br>**Keywords:**<br>adult,article,controlled study,education,female,harm reduction,human,human tissue,male,recall, regression analysis,*retrospective study,self report,*smoking cessation,*tobacco dependence,*vaping, nicotine<br><br>**Abstract:** INTRODUCTION: Although some smokers switch to exclusive use of electronic cigarettes (e-cigarettes), others become dual users of combustible cigarettes and e-cigarettes. Little is known about how the onset of vaping affects the use of and dependence on combustible cigarettes or total nicotine use and dependence, which may influence health-related and cessation outcomes. Using self-report data of current combustible and e-cigarette use and retrospective recall of pre-vaping smoking in a sample of dual users, the aims of this study were: 1) to compare pre- and post-vaping number of cigarettes per day and combustible cigarette dependence; 2) to compare pre- and post-vaping total nicotine use frequency (number of vaping sessions and cigarettes smoked per day), and total nicotine dependence; and 3) to examine predictors of nicotine dependence. METHOD(S): We used baseline data from a smoking cessation trial with 2896 dual users. Nicotine use frequency and the Heaviness of Smoking Index (HSI) were used as measures of nicotine use and dependence, respectively. RESULT(S): Participants decreased cigarettes/day from pre- (M=19.24, SD=9.01) to post-vaping (M=11.15, SD=8.02, p<.0001) and combustible cigarette dependence declined from pre- (M=3.55, SD=1.51) to post-vaping (M=2.11, SD=1.60, p<.0001). Total daily nicotine use frequency increased after initiating vaping (M=19.25, SD=9.01 vs. M=29.46, SD=8.61; p<.0001), as did total nicotine dependence (M=3.55, SD=1.51 vs. M=4.68, SD=1.38; p<.0001). Hierarchical regression analyses indicated that variables associated with greater overall nicotine dependence included: younger age, lower education, more years smoking, higher pre-vaping nicotine dependence, using e-cigarettes more days/month, more puffs/vaping session, higher e-liquid nicotine concentration, and longer vaping history. CONCLUSION(S): Dual use leads to a reduction in the number of combustible cigarettes, but total nicotine use and dependence increases. IMPLICATIONS: In dual users, a reduction in smoking following onset of vaping may offer some harm reduction via reduction in cigarette intake. However, the increase in total nicotine use and dependence could affect the ability to quit either or both products.Copyright © The Author(s) 2019. Published by Oxford University Press on behalf of the Society for Research on Nicotine and Tobacco. All rights reserved. For permissions, please e-mail: journals.permissions@oup.com.
3283	P Martinasek Mary, Bowersock Amy, W Wheldon Christopher (2018///) Patterns, Perception and Behavior of Electronic Nicotine Delivery Systems Use and Multiple Product Use Among Young Adults. Respiratory Care; 63<br><br>**Keywords:**<br>Cross-Sectional Studies,*Electronic Nicotine Delivery Systems/sn [Statistics & Numerical Data],Female, Florida,Humans,Male,Perception,*Smoking/px [Psychology],*Students/px [Psychology],Students/sn [Statistics & Numerical Data],Surveys and Questionnaires,

	**Abstract:** BACKGROUND: Electronic nicotine delivery systems (ENDS) are battery-operated devices used to inhale vaporized or aerosolized nicotine. There is increasing research uncovering negative health effects of these devices. Less is known about the social and behavioral aspects among college students., METHODS: This cross-sectional study was conducted at a mid-sized private university in Florida. The survey was sent via e-mail to the student body of undergraduates. A final sample size of 989 students was analyzed to understand demographic differences between users and nonusers, initiation factors, and influencers, as well as multiple product behaviors., RESULTS: Approximately 51.4% (n = 508) of participants reported ever using an ENDS and other tobacco consumption. Males were significantly more likely to be users of ENDS. Polytobacco use, or the use of multiple tobacco products, was also more common among participants who have tried ENDS (P < .001). Perceptions of harm of both the primary and secondary vapor were considered to be less than that of conventional cigarettes. Peers were the primary influencer for initial use. A 4-class latent variable model differentiated between usage patterns characterized as abstainers (70%), hookah users only (14%), ENDS only (11%), and polytobacco users (4%)., CONCLUSIONS: ENDS are not commonly used as a quit tool among college students, but rather as a secondary source of nicotine, most commonly in current smokers. Copyright © 2018 by Daedalus Enterprises.
3286	A N Mat Ruzlin, M Omar, H H Yong, B Tangiisuran, N A Abdul Rani, A S Mohd Samin, A C Quah, N Baharom, P Driezen (2018///) Self-reported of awareness, trial and current use of electronic cigarettes (ECS) among of adult smokers in Malaysia. Journal of Global Oncology; 4<br><br>**Keywords:**<br>adult,*awareness,controlled study,*electronic cigarette,female,human,human tissue,interview,major clinical study,*Malaysia,male,rural area,sampling,*smoking cessation,young adult,conference abstract<br><br>**Abstract:** Background: In many parts of the world, E-cigarette (EC) devices are becoming popular and an increasing trend in its usage especially among young people and adult smokers. Aim(s): To examine the self-reported awareness, trial and current use of ECs among current smokers andto determine the predictors associated with the outcomes. Method(s): A total of 40,000 current smokers aged 18 years and above were recruited through intercept face-to-face interview in wave 1 (May-September 2016), wave 2 (December 2016-April 2017) and wave 3 (May-August 2017). The samples were drawn from 5 streets randomly (two in hotspot and three in nonhotspot) within stratified urban and rural areas in 14 states of Malaysia. Respondents were recruited using systematic sampling. Every fifth person passing an interview station in each street was approached. Descriptive analysis and multivariate logistic regression were applied by using PASW 18. Result(s): Overall, 93% were aware of ECs, 19.9% were ECs trials and 14.6% were current users. Multivariate logistic regression shows that those states with no ban of ECs sales were more likely and significantly associated with these outcomes compared with those in states that this device has already been banned. Those who believe that ECs are "less harmful"(AOR56.28; 95% CI55.79-6.81, P, 0.001; AOR54.84; 95% CI54.12-5.69, P, 0.001) and "equally harmful" (AOR52.06; 95% CI51.91-2.22, P, 0.001; AOR52.25; 95% CI51.93-2.62, P, 0.001) were significantly associated with EC trials and current use of ECs respectively. In addition, intention to quit smoking (AOR52.91; 95% CI52.72-3.13) was also directly associated with EC trials. Conclusion(s): Awareness, trials and current use of ECs are likely due to the banning regulation implemented in specific states and strong belief that ECs is less harmful to health. Hence, this should be considered in the formulation of ECs policy in Malaysia.
3287	A N Mat Ruzlin, M Omar, H H Yong, B Tangiisuran, N A Abdul Rani, A S Mohd Samin, A C Quah, N Baharom, P Driezen (2018///) Motivations for current e-cigarette use among adult smokers in Malaysia. Journal of Global Oncology; 4<br><br>**Keywords:**<br>adult,advertising,controlled study,*electronic cigarette,human,interview,*Malaysia,Malaysian,market, *motivation,rural area,*smoking cessation,tax,conference abstract

	**Abstract:** Background: Recommendations from WHO regarding the comprehensive ban on tobacco advertisements, promotions and scholarships (TAPS) have recently caused tobacco companies in shifting their market strategies to focus on promoting unregulated devices such aselectronic cigarettes (ECs) and vapes. Aim(s): This study aims to explore the motivations of EC use among dual users and its associations with sociodemographic factors and smoking related characteristics under different regulatory environments in Malaysia. Method(s): Atotalof5823 dual users was collected usinga multistage sampling study design. Data collection was conducted through intercept face-to-face interviews within 3 waves; wave1 (May-September 2016), wave2 (December 2016-April 2017) and wave3(May-August 2017). Thesampleswere drawn from5streetsrandomly(twoin hotspot and three in nonhotspot locations) within stratified urban and rural areas in 14 states of Malaysia. Every fifth person passing an interview station in each street was approached. All statistical tests were conducted using PASW 18. Result(s): The top four self-reported motivations of using EC by dual users included "tasted better than conventional cigarettes" (85.1%), "to reduce the number of cigarettes smoked" (72.9%), "to quit smoking" (63.5%) and "increase price of cigarette taxes" (62.9%). Based on a multivariate analysis, respondents from states that have no current restrictions toward the sale and usage of ECs and who use ECs less than weekly (Adj.OR52.54; 95% CI51.93-3.34), weekly (Adj.OR52.22; 95% CI51.72-2.85) and daily users (Adj.OR5 1.77; 95% CI51.36-2.31) were more likely to use ECs due to its better taste as compared with those who use ECs less than once a month. In states that have banned the sales and usage of ECs, there was a significant association between daily users of ECs and the four primary motivations. It was found that an increasing amount of cigarettes smoked per day (Adj. OR52.46; 95% CI51.59-3.81) had significantly influenced smokers in these states to more likely use ECs due to the increase price of cigarette tax. Conclusion(s): This study was conducted to show associations between smoking characteristics, EC use and self-reported motivations under different regulatory environments in Malaysia. Frequency of EC use was significantly associated with these self-reported motivations. Further research should be conducted to monitor EC use by Malaysians as well as to contribute to the formulation of EC policy in Malaysia.
3290	T Hall Matthew, P Austin Ryan, A Do Tai, McGlynn Andrea (2018///) Vape and Aviate: Electronic-Cigarette Use and Misuse in Naval Aviation. Military medicine; 183<br><br>**Keywords:**<br>Adult,*Aerospace Medicine/td [Trends],Aviation/td [Trends],Cross-Sectional Studies,Electronic Nicotine Delivery Systems/is [Instrumentation],*Electronic Nicotine Delivery Systems/sn [Statistics & Numerical Data],Female,Humans,Male,Middle Aged,Military P<br><br>**Abstract:** Introduction: Electronic cigarettes (EC) are an emerging form of nicotine replacement that has had a discernible increase in prevalence in the general population. Little is known regarding EC use among different military demographic groups or the extent of influence that social determinants of health may have on the behavior. The purpose of this study was to assess the prevalence and correlates of EC use in a select population of active duty U.S. Naval personnel. This study is unique in that it allows for EC behavior comparison between a traditionally healthy demographic (aviators, a subset of aircrew) and a representative general military population sample., Materials and methods: Cross-sectional survey data were collected anonymously and analyzed in 2015-2016. Active duty Naval personnel (n = 977) were asked about ever trying ECs, frequency of use, and when was the last time an EC was used. Participants were assessed similarly regarding cigarette use. Descriptive and inferential statistics as well as multinomial logistic regression analyses were conducted using categorical and ordinal variables assigned to usage and demographic factors. This protocol was approved by the Institutional Review Board at Naval Hospital Portsmouth located in Portsmouth, Virginia., Results: Within the study population, 31.4% have tried ECs, 9.3% were current users, and only 3.8% were dual (EC and cigarettes) users. EC use was significantly associated with paygrade (enlisted), primary job duty (non-aircrew), and education (less than a bachelor degree, p < 0.001, p < 0.01, and p < 0.001, respectively). ECs were used at some point as a smoking cessation tool for 43.6% of current EC users (p < 0.001), 21.6% of current smokers, and only 5.6% of former smokers. Continued EC use after ever trying ECs (30.0%) was only slightly more prevalent as

	continued cigarette use after ever trying cigarettes (27.3%). Smokers were nearly five times more likely to currently use ECs compared with non-smokers. Trying cigarettes was associated with nearly 12 times the risk for trying ECs compared with those who never tried cigarettes. No significant misuse of ECs was noted; however, several cases of drinking, tasting, and touching EC fluid were reported., Conclusion: This study is unique in that it examines the use of electronic cigarettes among selected healthy and baseline groups within the active duty U.S. Navy population. The current prevalence of EC use among active duty personnel is much higher than previously thought. Continued use of ECs after ever trying them appears to exceed that of cigarettes even among healthy populations such as aircrew. The findings of increased risk of EC use among those with lower income and less than a bachelor's degree suggest social determinants of health implications. This study sheds new light on EC use, characteristics of use, addiction implications, and highlights concerns for a growing health risk behavior.
3291	Morgenstern Matthis, Nies Alina, Goecke Michaela, Hanewinkel Reiner (2018///) E-Cigarettes and the Use of Conventional Cigarettes. Deutsches Arzteblatt international; 115<br><br>**Keywords:**<br>Adolescent,*Adolescent Behavior/px [Psychology],*Behavior, Addictive/ep [Epidemiology],Cigarette Smoking/ep [Epidemiology],*Electronic Nicotine Delivery Systems/sn [Statistics & Numerical Data], Female,Germany,Humans,Male,Mass Screening,*Smoking/ep [Epid<br><br>**Abstract:** BACKGROUND: In 2015, 12.1% of 12- to 17-year-olds in Germany had reportedly already tried e-cigarette smoking at least once. We carried out a study of the "gateway" hypothesis, according to which the use of e-cigarettes can motivate adolescents to start smoking conventional cigarettes., METHODS: During the 2015/2016 school year, 2186 tenth-graders in the German states of Lower Saxony and Schleswig-Holstein who had never smoked conventional cigarettes before took part in a survey over a 6-month period (mean age 15.5 years, standard deviation 0.65; 53.6% female)., RESULTS: 14.3% of the survey population (313 adolescents) said at the start of the survey period that they had already tried e-cigarettes at least once. By the end of the survey period, 12.3% (268) of those who had never smoked before had begun to experiment with conventional cigarettes. The risk of beginning such experimentation was 2.2 times higher among e-cigarette users. This association remained (relative risk = 2.18 [1.65; 2.83]) after statistical control for age, sex, state, immigrant background, type of school, socioeconomic status, various personality traits (sensation-seeking, impulsivity, anxiety, hopelessness, extraversion, agreeableness, conscientiousness, neuroticism, openness), and the use of alcohol, cannabis, and other illicit drugs. Further analysis revealed that the association between the use of e-cigarettes and the onset of conventional cigarette smoking was stronger among adolescents with low sensation-seeking scores and without any experience of alcohol intoxication., CONCLUSION: Among adolescents who have never smoked, experimentation with conventional cigarettes is more common in those who have used e-cigarettes. This effect seems to be stronger among adolescents who, in general, have a lower risk of starting to smoke. The 6-month observation period of this study is too short to allow any inference regarding a connection between e-cigarette use and the development of tobacco dependence.
3296	S E McCabe, P Veliz, V V McCabe, C J Boyd (2019///) Initiation Sequence of E-Cigarette and Cigarette Smoking among US Adolescents: A National Study. The American journal on addictions; 28<br><br>**Keywords:**<br>adolescent,article,*cigarette smoking,controlled study,drug dependence,*electronic cigarette,female, high school,human,juvenile,major clinical study,male,prospective study,questionnaire,student,world health organization<br><br>**Abstract:** BACKGROUND AND OBJECTIVES: The primary objectives were to: (1) examine the initiation sequence of e-cigarette use and cigarette smoking; (2) assess other substance use as a function of the initiation sequence of e-cigarettes and cigarettes; and (3) investigate the role of early e-cigarette initiation among US secondary school students. METHOD(S): Data were collected via self-administered

	questionnaires from independent 2015 and 2016 nationally representative cross-sectional samples of 8th grade, 10th grade, and 12th grade students (N=36410). RESULT(S): The lifetime initiation sequence included: (1) e-cigarette use before cigarette smoking (1.7%); (2) e-cigarette use and cigarette smoking in same grade (4.2%); (3) cigarette smoking before e-cigarette use (6.1%); (4) e-cigarette use only (12.6%); (5) cigarette smoking only (3.6%); and (6) no e-cigarette use or cigarette smoking (71.8%). The risk of substance use was highest among dual users (regardless of initiation sequence), followed by cigarette smokers, e-cigarette users, and no e-cigarette use or cigarette smoking. The most prevalent initiation sequence of e-cigarette use involved initiating other substances before e-cigarettes, especially among older adolescents. Early initiation of e-cigarette use was associated with increased odds of substance use behaviors for all three age groups. DISCUSSION AND CONCLUSION(S): Adolescents who report a history of both e-cigarette use and cigarette smoking should be considered at high risk for substance-related problems. Early initiation of e-cigarette use is a signal for other substance use. SCIENTIFIC SIGNIFICANCE: Youth substance use prevention programs and prospective studies must take into account polysubstance use when addressing the relationships between e-cigarette use and cigarette smoking. (Am J Addict 2019;28:285-294).Copyright © 2019 American Academy of Addiction Psychiatry.
3297	S E McCabe, B T West, V V McCabe (2018///) Associations between early onset of e-cigarette use and cigarette smoking and other substance use among us adolescents: A national study. Nicotine and Tobacco Research; 20<br><br>**Keywords:**<br>adolescent,*adolescent smoking,alcohol consumption,article,cannabis use,*cigarette smoking,female, human,major clinical study,male,prescription drug misuse,priority journal,risk factor,substance abuse, *substance use,United States,illicit drug,*electronic<br><br>**Abstract:** Introduction: This study examines the associations between early onset of e-cigarette use and cigarette smoking and other substance use behaviors among US adolescents. Method(s): Data were collected via self-administered questionnaires from a nationally representative sample of 2299 US high school seniors attending public and private high schools during the spring of their senior year in 2015 as part of the Monitoring the Future study. Result(s): A higher percentage of adolescents who began using e-cigarettes in ninth grade or earlier (early onset) were found to report current and lifetime cigarette smoking and other substance use relative to those individuals who never used e-cigarettes or those who began using e-cigarettes later in the 12th grade. Multivariate logistic regression analyses indicated that the adjusted odds of alcohol use, cigarette smoking, marijuana use, nonmedical prescription drug use, and other illicit drug use among early onset e-cigarette users were significantly greater than those for individuals never having used e-cigarettes (adjusted odds ratios [AORs] ranged 9.5-70.6, $p < .001$). While these associations were significant for both experimental and frequent e-cigarette users, the effects of early onset were stronger among frequent e-cigarette users. Similarly, the odds of these substance use behaviors (except alcohol) among early onset e-cigarette users were also significantly greater than the odds for later onset e-cigarette users (AORs ranged 2.8-4.1, $p < .05$). Conclusion(s): Early onset of e-cigarette use was significantly associated with increased odds of cigarette smoking and other substance use behaviors. E-cigarette use is often preceded by alcohol use, cigarette smoking, and marijuana use, suggesting that more long-term prospective studies are warranted. Implications: To date, no studies have examined the probability of cigarette smoking and other substance use behaviors as a function of age at onset of e-cigarette use. In the present study, early onset of e-cigarette use was significantly associated with increased odds of cigarette smoking and other substance use behaviors. The findings reinforce the importance of addressing a wide range of substances including alcohol, traditional cigarettes, and marijuana when developing early primary prevention efforts to reduce e-cigarette use among youth.Copyright © The Author(s) 2017. Published by Oxford University Press on behalf of the Society for Research on Nicotine and Tobacco. All rights reserved.
3315	A Little Melissa, C Klesges Robert, Bursac Zoran, P Halbert Jennifer, Ebbert Jon, W Talcott Gerald, Weksler Benny (2018///) Correlates of smoking status in cancer survivors. Journal of cancer survivorship : research

	and practice; 12<br><br>**Keywords:**<br>Adult,Aged,*Cancer Survivors/sn [Statistics & Numerical Data],Cross-Sectional Studies,Electronic Nicotine Delivery Systems/sn [Statistics & Numerical Data],Female,Humans,Male,Middle Aged,Prevalence, *Smoking/ep [Epidemiology],*Smoking Cessation/sn [Stati<br><br>**Abstract:** PURPOSE: To determine the characteristics associated with cancer survivors which indicate continued cigarette smoking at or around the time of cancer diagnosis., METHODS: A total of 631 survivors were recruited in four cancer centers in Memphis, TN, between March 2015 and June 2016. To increase the probability of accurate reporting, surveys were conducted anonymously. A total of 112 respondents reported they were current smokers and 202 reported they were former smokers (n = 314), who comprised the sample., RESULTS: We found that the rate of daily e-cigarette use among cancer survivors who smoked was 15.2% versus 3.9% in cancer survivors who no longer smoked. The national rate of adult e-cigarette use is 3.5%. Multivariate models correlated the characteristics of current versus former smokers and revealed that increasing age (aOR = 0.97, p < .0001), decreasing education (aOR = 2.39, p < .02), and current use of e-cigarettes (aOR = 3.74, p < .00045) were frequently associated with current cigarette smoking., CONCLUSIONS: While age and gender were associated with continued smoking, current use of e-cigarettes was associated with almost four times higher odds of being a current smoker. Further research is needed to determine if use of e-cigarettes deters or promotes the smoking cessation process, at least in cancer survivors., IMPLICATIONS FOR CANCER SURVIVORS: Among cancer survivors who continue to smoke after a cancer diagnosis, use of e-cigarettes is highly prevalent; research is needed to determine whether use of e-cigarettes promotes, has no effect, or hinders smoking cessation efforts among this vulnerable population.
3316	R Dvorsky Melissa, M Langberg Joshua (2019///) Cigarette and e-cigarette use and social perceptions over the transition to college: The role of ADHD symptoms. Psychology of addictive behaviors : journal of the Society of Psychologists in Addictive Behaviors; 33<br><br>**Keywords:**<br>Adolescent,*Attention Deficit Disorder with Hyperactivity/px [Psychology],*Cigarette Smoking/ep [Epidemiology],Cigarette Smoking/px [Psychology],Female,Humans,Longitudinal Studies,Male, Prevalence,Prospective Studies,Risk Factors,*Social Perception,*Univ<br><br>**Abstract:** Cigarette and electronic cigarette (e-cigarette) use prevalence increases during adolescence and peaks in young adulthood, with substantial increases during the transition from high school to college especially more recently for e-cigarette use. It is important to identify the underlying factors that serve as risk factors for tobacco use and social perceptions about cigarette and e-cigarette use. It is unknown whether attention-deficit/hyperactivity disorder (ADHD) symptoms are associated with social perceptions about tobacco or increased tobacco use during the high school to college transition. This three timepoint prospective longitudinal study evaluates the reciprocal relationship between ADHD symptoms and social perceptions about tobacco as well as the frequency of cigarette and e-cigarette use in a sample of 150 high school seniors (Mage = 18.25, 66.0% female, 65.3% White) across the transition to college. ADHD symptoms in high school predicted increases in e-cigarette use during the first semester of college, and this association maintained through the end of the first year. ADHD symptoms predicted changes in social perceptions about cigarette and e-cigarette use after the transition to college. ADHD symptoms were predicted by social perceptions about e-cigarettes at the beginning of college. Understanding the psychosocial mechanisms underlying the pathways from ADHD symptoms to e-cigarette use may advance tobacco use etiology and prevention efforts, which is important considering the rapid growth in e-cigarette use among emerging adults. (PsycINFO Database Record (c) 2019 APA, all rights reserved).

3318	A S Melka, C L Chojenta, E G Holliday, D J Loxton (2019///) Predictors of E-cigarette Use Among Young Australian Women. American Journal of Preventive Medicine; 56<br><br>**Keywords:**<br>adult,age,alcohol consumption,article,Australian,cigarette smoking,cohort analysis,cross-sectional study, drinking behavior,female,financial management,human,longitudinal study,medicare,partner violence, prevalence,risk factor,rural area,smoking habit,tob<br><br>**Abstract:** Introduction: E-cigarette use is controversial worldwide. The majority of previous studies on e-cigarette use were not gender specific. This study aimed to identify the predictors of e-cigarette use among young Australian women. Method(s): This study used cross-sectional data from the 1989-1995 cohort of the Australian Longitudinal Study on Women's Health. In 2015, study participants (N=8,915) aged 19-26 years completed an online survey. Multivariable logistic regression was used to identify predictors of e-cigarette use. Data were analyzed in 2018. Result(s): The prevalence of ever and past-year e-cigarette use among young Australian women was 11.1% and 6.4%, respectively. More than a quarter of past-year and ever e-cigarette users were never cigarette smokers. Use of e-cigarettes in the past year was associated with younger age (AOR per year increase=0.87, 95% CI=0.82, 0.93); financial difficulty (AOR=0.68, 95% CI=0.54, 0.87); being an ex-smoker (AOR=5.05, 95% CI=3.64, 7.01) or current cigarette smoker (AOR=10.01, 95% CI=7.77, 12.89); drinking at a level of lifetime risk of harm from alcohol-related disease or injury (AOR=1.23, 95% CI=1.01, 1.53). Ever e-cigarette use showed similar associations and was also associated with rural residence (AOR=0.74, 95% CI=0.60, 0.91) and intimate partner violence (AOR=1.44, 95% CI=1.17, 1.76). Conclusion(s): The high prevalence of e-cigarette use among never cigarette smokers has significant public health implications. Interventions to curb the use of e-cigarettes among young Australian women should focus on risk factors, such as early age, cigarette smoking, alcohol use, and intimate partner violence.Copyright © 2018 Elsevier Ltd
3319	Noland Melody, Rayens Mary Kay, T Wiggins Amanda, Huntington-Moskos Luz, A Rayens Emily, Howard Tiffany, J Hahn Ellen (2018///) Current Use of E-Cigarettes and Conventional Cigarettes Among US High School Students in Urban and Rural Locations: 2014 National Youth Tobacco Survey. American journal of health promotion : AJHP; 32<br><br>**Keywords:**<br>Adolescent,*Adolescent Behavior/px [Psychology],*Cigarette Smoking/px [Psychology],Cross-Sectional Studies,*Electronic Nicotine Delivery Systems/sn [Statistics & Numerical Data],Female,Humans,Male, Population Surveillance,*Rural Population/sn [Statistics<br><br>**Abstract:** PURPOSE: Adolescent tobacco use is higher in rural than in urban areas. While e-cigarette use is increasing rapidly among this age group, differences in prevalence between rural versus urban populations for this relatively novel product have not been explored. The purpose is to investigate whether location of school (rural-urban) is associated with e-cigarette use and dual use (defined as the use of both e-cigarettes and conventional cigarettes) among high school students., DESIGN: Cross-sectional survey obtained using a stratified, 3-stage cluster sample design., SETTING: United States., PARTICIPANTS: A nationally representative sample of US high school students (N = 11 053) who completed the 2014 National Youth Tobacco Survey (NYTS); slightly more than half were urban (54%)., MEASURES: The NYTS measures tobacco-related knowledge, attitudes, and use behavior and demographics of students in the United States., ANALYSIS: Weighted logistic regression assessed the relationships of urban-rural location with current e-cigarette use and dual use, adjusting for demographic factors, perceived risk, and social norms., RESULTS: There were clear differences in patterns of adolescent e-cigarette and cigarette use in rural versus urban areas. Social norms and perceptions may play a role in understanding these differences., CONCLUSION: Urban youth current cigarette smokers were nearly twice as likely as rural cigarette smokers to also use e-cigarettes. Reasons for urban-rural differences need to be taken into account when designing prevention programs and policy changes.

3320	C P Mendelsohn, A D Wodak (2019///) Unintended consequences of a cautious approach to e-cigarette laws. Medical Journal of Australia; 211<br><br>**Keywords:**<br>Australia,bioavailability,exposure,first pass effect,gastrointestinal symptom,human,ingestion,intoxication, *law,letter,nicotine replacement therapy,risk reduction,vaping,vomiting,nicotine/to [Drug Toxicity], *electronic cigarette<br><br>**Abstract:**
3322	A L Merianos, R A Jandarov, J D Klein, E M Mahabee-Gittens (2019///) Characteristics of Daily E-Cigarette Use and Acquisition Means Among a National Sample of Adolescents. American journal of health promotion : AJHP;<br><br>**Keywords:**<br>adolescent,article,controlled study,*electronic cigarette,flavor,*health care policy,human,Internet,logic, shopping,*tobacco dependence,cannabis,menthol,nicotine<br><br>**Abstract:** PURPOSE: To examine the relationship between several e-cigarette-related characteristics and daily e-cigarette use among adolescent current users. DESIGN: Secondary analysis of 2016 National Youth Tobacco Survey data. SETTING: United States middle schools and high schools. SAMPLE: One thousand five hundred seventy-nine current e-cigarette users. MEASURES: Daily e-cigarette use and e-cigarette flavors, brands, device type, and acquisition were measured. ANALYSIS: Logistic regression and Poisson regression models were built. RESULT(S): A total of 13.6% of current users reported daily use. Results indicated that daily users were at increased odds of using all flavor types (all P < .001), with the exception of menthol/mint, and using a higher number of flavors than nondaily users (P < .001). Daily users were more likely to use Blu, eGo, Logic, Halo, NJOY, and another unlisted brand but less likely to report they did not know the brand used (all P <= .01). Daily users also reported using a higher number of brands than nondaily users (P < .001). Daily users were at increased odds of using marijuana/tetrahydrocannabinol (THC) oil wax as device ingredients (P < .001) and less likely to use only nicotine (P < .001) or unknown ingredients (P = .004). Daily users were more likely to acquire e-cigarettes from a vape shop, gas station/convenience store, Internet, mall/shopping center kiosk, drug store, grocery store, or other place (all P <= .01). CONCLUSION(S): Comprehensive efforts are needed to reduce e-cigarette use and nicotine addiction among adolescents.
3328	R Miech, L Johnston, P M O'Malley, J G Bachman, M E Patrick (2019///) Adolescent vaping and nicotine use in 2017-2018 - U.S. National estimates. New England Journal of Medicine; 380<br><br>**Keywords:**<br>*adolescent smoking,human,letter,prevalence,priority journal,tobacco use,United States,*vaping, *nicotine,electronic cigarette<br><br>**Abstract:**
3329	R Miech, L D Johnston, P M O'Malley, Y M Terry-McElrath (2019///) The national prevalence of adolescent nicotine use in 2017: Estimates taking into account student reports of substances vaped. Addictive Behaviors Reports; 9<br><br>**Keywords:**<br>adolescent,article,cannabis use,cigar smoking,cigarette smoking,human,priority journal,*vaping,cigarette smoke,*nicotine<br><br>**Abstract:** Introduction: This study presents the first nationally-representative estimates of adolescent

	nicotine prevalence that take into account adolescent reports of substances vaped. These reports allow nicotine estimates that consider the impact of the newly-emerged group of adolescents who report vaping only non-nicotine substances such as flavoring and/or marijuana and do not use nicotine in any form - a group typically treated as nicotine users. Method(s): Data come from Monitoring the Future and are a randomly-selected subsample of 2231 U.S. 12th grade students who answered surveys with detailed questions on tobacco use and vaping in 2017. Result(s): Among 12th grade students 24.7% used nicotine in the last 30 days. This estimate does not include the 3.8% of students who vaped only non-nicotine substances and did not use nicotine in any other form. These students more closely resemble their peers who do not use nicotine than those who do, in terms of perceived risk and disapproval of cigarettes, as well as percentage of friends who use cigarettes. Conclusion(s): A decline in nicotine prevalence was statistically significant, but not strikingly large, after taking into account students who vape non-nicotine substances and do not use nicotine in any form. These students are largely similar to their peers who do not use nicotine, which underscores the importance of efforts to alert youth that they may be vaping nicotine unknowingly, and prevent them from doing so.Copyright © 2019 The Authors
3337	M Mirbolouk, P Charkhchi, O A Orimoloye, S M I Uddin, S Kianoush, R Jaber, A Bhatnagar, E J Benjamin, M E Hall, A P DeFilippis, W Maziak, K Nasir, M J Blaha (2019///) E-cigarette use without a history of combustible cigarette smoking among U.S. Adults: Behavioral risk factor surveillance system, 2016. Annals of Internal Medicine; 170<br><br>**Keywords:**<br>adult,age distribution,aged,*behavioral risk factor surveillance system,binge drinking,*cigarette smoking, controlled study,female,human,letter,major clinical study,male,medical history,middle aged,prevalence, priority journal,tobacco use,United States,yo<br><br>**Abstract:**
3341	Mirbolouk Mohammadhassan, Charkhchi Paniz, Kianoush Sina, S M Iftekhar Uddin, A Orimoloye Olusola, Jaber Rana, Bhatnagar Aruni, J Benjamin Emelia, E Hall Michael, P DeFilippis Andrew, Maziak Wasim, Nasir Khurram, J Blaha Michael (2018///) Prevalence and Distribution of E-Cigarette Use Among U.S. Adults: Behavioral Risk Factor Surveillance System, 2016. Annals of Internal Medicine; 169<br><br>**Keywords:**<br>Adolescent,Adult,Age Factors,Aged,Aged, 80 and over,Behavioral Risk Factor Surveillance System,Cross-Sectional Studies,Electronic Nicotine Delivery Systems/sn [Statistics & Numerical Data],Female,Humans, Male,Middle Aged,Prevalence,Risk Factors,Sex Facto<br><br>**Abstract:** Background: Contemporary data on the prevalence of e-cigarette use in the United States are limited., Objective: To report the prevalence and distribution of current e-cigarette use among U.S. adults in 2016., Design: Cross-sectional., Setting: Behavioral Risk Factor Surveillance System, 2016., Participants: Adults aged 18 years and older., Measurements: Prevalence of current e-cigarette use by sociodemographic groups, comorbid medical conditions, and states of residence., Results: Of participants with information on e-cigarette use (n = 466 842), 15 240 were current e-cigarette users, representing a prevalence of 4.5%, which corresponds to 10.8 million adult e-cigarette users in the United States. Of the e-cigarette users, 15% were never-cigarette smokers. The prevalence of current e-cigarette use was highest among persons aged 18 to 24 years (9.2% [95% CI, 8.6% to 9.8%]), translating to approximately 2.8 million users in this age range. More than half the current e-cigarette users (51.2%) were younger than 35 years. In addition, the age-standardized prevalence of e-cigarette use was high among men; lesbian, gay, bisexual, and transgender (LGBT) persons; current combustible cigarette smokers; and those with chronic health conditions. The prevalence of e-cigarette use varied widely among states, with estimates ranging from 3.1% (CI, 2.3% to 4.1%) in South Dakota to 7.0% (CI, 6.0% to 8.2%) in Oklahoma., Limitation: Data were self-reported, and no biochemical confirmation of tobacco use was available., Conclusion: E-cigarette use is common, especially in younger adults, LGBT persons, current cigarette

	smokers, and persons with comorbid conditions. The prevalence of use differs across states. These contemporary estimates may inform researchers, health care policymakers, and tobacco regulators about demographic and geographic distributions of e-cigarette use., Primary Funding Source: American Heart Association Tobacco Regulation and Addiction Center, which is funded by the U.S. Food and Drug Administration and National Heart, Lung, and Blood Institute.
3346	M Moran, T Alexander, R Kennedy, C Weiger (2018///) Differences in rates of tobacco product use among U.S. Hispanic youth. Tobacco Induced Diseases; 16<br><br>**Keywords:**<br>adult,child,controlled study,education,electronic cigarette,friend,*hookah,human,inheritance,*juvenile, *Mexican American,school child,smoking,United States,conference abstract<br><br>**Abstract:** Background: Hispanics are the fastest growing ethnic group in the United States, and projected to grow by 86% by 2050. Hispanics are also a diverse ethnic group, consisting of individuals with heritage from many areas, including Mexico, South America, Cuba and Puerto Rico. Little is known about variations in tobacco use and susceptibility among such Hispanic sub-groups. Method(s): We analyzed Wave 2 youth data from the Population Assessment of Tobacco and Health (PATH) study. PATH is a nationally representative survey of 12,172 youth aged 12-17 and adults, living in the United States. This dataset provides information on whether a participant is Mexican, Cuban, Puerto Rican, or of another Hispanic heritage. We examined differences in susceptibility to use and ever use of four tobacco products (cigarettes, e-cigarettes, cigarillos and hookah) among Hispanic youth. We also examined correlates of susceptibility and use. Result(s): Mexican, Cuban, Puerto Rican and other Hispanic youth were more likely than non-Hispanic youth to be susceptible to hookah use (ORs=1.7, 2.5, 1.5 and 1.6, respectively), while Mexican American and other Hispanic youth were more likely than non-Hispanic youth to be susceptible to e-cigarette use (ORs=1.3 and 1.2, respectively) and cigarillo use (ORs=1.4 and 1.4, respectively). Mexican, Cuban, and other Hispanic youth were more likely to have tried smoking hookah (ORs=1.5, 4.1 and 1.65, respectively). Parental education, friends' tobacco use, and beliefs that tobacco would calm them down were consistent predictors of tobacco use. Specific differences in correlates of susceptibility and use of each product, and by Hispanic subgroup, will be discussed. Conclusion(s): It is critical to take into account the diversity among the U.S. Hispanic population when examining rates of tobacco use. Failing to do so can mask disparities in tobacco use among specific sub-groups. Tobacco prevention campaigns and policies should similarly consider the diversity of this population.
3350	M B Moran, A C Villanti, A Johnson, J Rath (2019///) Patterns of Alcohol, Tobacco, and Substance Use Among Young Adult Peer Crowds. American Journal of Preventive Medicine; 56<br><br>**Keywords:**<br>adult,*alcohol consumption,article,cohort analysis,controlled study,cross-sectional study,female,human, male,religion,*self concept,smokeless tobacco,social status,*substance use,*tobacco use,*alcohol, cannabis,cigar,cigarette,electronic cigarette,hookah,<br><br>**Abstract:** Introduction: The association between peer crowd identification and substance use is well documented among adolescents, but less is known about substance use among young adult peer crowds. Method(s): This study leverages data from the Truth Initiative Young Adult Cohort Study (Wave 8, June-July 2015), a nationally representative cohort sample of young adults aged 18-34 years. The current cross-sectional analyses (conducted in 2018)focused on 1,341 individuals aged 18-24 years in this sample. Participants reported their peer crowd identification and current use of alcohol, marijuana, other drugs, and tobacco (cigarettes, little cigars/cigarillos, e-cigarettes, hookah, smokeless tobacco). Adjusted logistic regression models assessed associations between peer crowd identification and substance use. Result(s): In general, young adults who identified as homebody, young professional, or religious had lower odds of substance use than their counterparts. Young adults who identified as social/partier were more likely to be current users of alcohol, marijuana, any tobacco, cigarettes, and e-cigarettes than those who did not

	identify as social/partier. Those who identified as alternative were more likely to be current users of marijuana and other drugs than those not identified as alternative. Those who identified as country were more likely than those not identified as country to be current users of cigarettes and smokeless tobacco. Those who identified as hip hop were more likely to be current users of marijuana and e-cigarettes. Conclusion(s): Peer crowd identification is associated with substance use among young adults. These findings can help identify target populations for prevention and cessation interventions and inform intervention design and delivery.Copyright © 2019 American Journal of Preventive Medicine
3359	I Munteanu, F Mihaltan, A Trofor, D Todea, M Marc, C Marginean, M Trenchea, O Arghir (2018///) Prevalence and knowledge on e-cigarette among teenagers in Romania 2016. Tobacco Induced Diseases; 16<br><br>**Keywords:**<br>adolescent,child,*electronic cigarette,female,girl,high school,human,major clinical study,male,money, nonsmoker,*prevalence,questionnaire,*Romania,smoke,conference abstract<br><br>**Abstract:** Background: The prevalence of smoking for adolescents in Romania is growing, especially due to girls. According to the study GATS 2011 most of daily smokers started smoking at age 17-19 years (43.1%); 17% started smoking even before 15 years, mostly in rural areas Identifying factors that favor its use will allow better management of the phenomenon. Worrying is the fact that they use the device for inhalation of other psychoactive substances . The number of users among young people the electronic cigarette tends to be a in an ascendant trend among teenagers and especially girls. Method(s): The main objectives were to establish the prevalence of using, condition of using and knowledge about e-cigarette among them A number of 2000 original questionnaires was directly distribute to teenagers for high schools from 6 important towns from Romania. Result(s): A number of 776 (457 girls and 319 boys) adolescents from different parts of the country where involved. The main objectives were to establish the prevalence of using, condition of using and knowledge about e-cigarette among them. 324 (42%) had used the device 177 girls and 147 boys. Majority of users were smokers but a number of 61 (21%) were nonsmokers and they used it. Although the majority said they use it occasionally they are able to present the advantages and disadvantages of the device . The one who never used it related no advantages and the users mentioned : less toxicity, money save, you can smoke it everywhere . Regarding disadvantages the option were more variety like: battery power, price, utility and so on. Conclusion(s): In conclusion, in the absence of specific legislative measures for Romania we can expect an increasing number of users among young people the electronic cigarette tends to be a in an ascendant trend among teenagers and especially girls.
3360	T Munzel, O Hahad, A Daiber (2019///) Double hazard of smoking and alcohol on vascular function in adolescents. European Heart Journal; 40<br><br>**Keywords:**<br>*alcohol consumption,alcohol intoxication,arterial stiffness,atherosclerosis,*blood vessel function, cardiovascular risk,cholesterol blood level,disease burden,Europe,exposure,Germany,health care policy, human,life expectancy,oxidative stress,pathophysiol<br><br>**Abstract:**
3361	E Nabi-Burza, S Regan, B H Walters, J E Drehmer, N A Rigotti, D J Ossip, J A Gorzkowski, D E Levy, J P Winickoff (2019///) Parental Dual Use of e-Cigarettes and Traditional Cigarettes. Academic Pediatrics; 19<br><br>**Keywords:**<br>aerosol,article,child,controlled study,*electronic cigarette,female,harm reduction,human,infant,major clinical study,male,nicotine replacement therapy,*passive smoking,pediatrics,self report,*smoking cessation

	**Abstract:** Background: E-cigarettes are growing in popularity. Dual use of e-cigarettes and cigarettes is an increasingly common practice, but little is known about patterns of dual use in parents. We sought to describe smoking-related behaviors among dual-users. Method(s): Parent exit surveys were conducted following their child's visit in 5 control pediatric practices in 5 states participating in the Clinical Effort Against Secondhand Smoke Exposure trial. We examined factors associated with dual use of e-cigarettes and cigarettes versus cigarette-only smokers, assessed by self-report. Result(s): Of 1382 smokers or recent quitters screened after their child's visit between April and October 2017, 943 (68%) completed the survey. Of these, 727 parents reported current use of cigarettes; of those, 81 (11.1%) also reported e-cigarette use, meeting the definition of dual use. Compared with cigarette-only smokers, dual users were more likely to have a child younger than 1 year old, planned to quit in the next 6 months, and had tried to quit in the past (had a quit attempt in the past 3 months, called the quitline, or used medicine to quit in the past 2 years; P < .05 for each). Conclusion(s): Parents who use both e-cigarettes and cigarettes may have greater rates of contemplating smoking cessation than parents who only smoke cigarettes. These parents may be using e-cigarettes for harm reduction or as a step toward cessation. Identification of these parents may provide an opportunity to deliver effective treatment, including nicotine-replacement therapies that do not expose infants and children to e-cigarette aerosol.Copyright © 2019 Academic Pediatric Association
3363	Bhandari Naleen Raj, D Day Kanesha, Payakachat Nalin, M Franks Amy, R McCain Keith, Ragland Denise (2018///) Use and Risk Perception of Electronic Nicotine Delivery Systems and Tobacco in Pregnancy. Women's health issues : official publication of the Jacobs Institute of Women's Health; 28<br><br>**Keywords:**<br>Adolescent,Adult,Attitude to Health,Cross-Sectional Studies,*Electronic Nicotine Delivery Systems/sn [Statistics & Numerical Data],Female,*Health Knowledge, Attitudes, Practice,Humans,Middle Aged, Nicotine/ae [Adverse Effects],Pregnancy,Pregnant Women,Ri<br><br>**Abstract:** INTRODUCTION: Given the rapid rise in availability and use, understanding the perception of electronic nicotine delivery systems (ENDS) products in pregnant women is vital. As more women of reproductive age use these products, it is likely that their use during pregnancy is also increasing. This study investigated the use of ENDS and tobacco cigarettes, along with knowledge and perceptions of associated health risks in pregnant women., METHODS: A cross-sectional survey was conducted at a university-based obstetrical clinic. A 32-item self-administered survey was used to collect participants' knowledge, use, and risk perceptions of ENDS and tobacco smoking. Bivariate associations of demographics and ENDS user status were explored using Chi-square or Fisher's exact tests. Average differences in agreement with perception statements across ENDS user status were tested using ANOVA with Tukey's tests for multiple comparisons., RESULTS: Of 382 participants, 57.9% were 21-29 years old and 60.1% had some college or higher education. 30.3% reported using both ENDS and tobacco cigarettes and 11.9% were current ENDS users. The majority of participants had adequate knowledge about the facts and safety of ENDS and there was no difference across three ENDS user status groups. ENDS users perceived significantly lower risk of ENDS and higher benefit of using ENDS to aid quitting tobacco smoking, compared to non-ENDS users. The majority of participants reported that their healthcare providers less frequently assessed ENDS use during their prenatal visits, compared to tobacco cigarette use., CONCLUSIONS: There is critical need for healthcare providers to increase the screening for ENDS use during pregnancy and promote awareness of risks and benefits of ENDS in pregnant women. Copyright © 2018 Jacobs Institute of Women's Health. Published by Elsevier Inc. All rights reserved.
3373	P M Ndunda, M Vindhyal, T Muutu, C Munguti (2019///) Two-year trends and determinants of electronic cigarette use Among patients with cardiovascular disease in the United States. Circulation; 139<br><br>**Keywords:**<br>adult,*angina pectoris,behavioral risk factor surveillance system,Black person,Caucasian,cerebrovascular

	accident,chronic disease,controlled study,*electronic cigarette,female,heart infarction,human,major clinical study,male,mental disease,middle aged,m<br><br>**Abstract:** Background: The use of electronic cigarettes (e-cigarettes) is on the rise in the US. It increased in adults aged 25-44 years from 2.4% in 2012 to 4.2% in 2016 and from 2% to 2.8% between ages 55 to 64 over the same period. Among the youth, the increase was 900% between 2011 and 2015. Much remains to be known about the health effects of e-cigarette use, but epidemiologic studies suggest an independent association between e-cigarette use and cardiovascular disease. There is a need for data on the social and demographic factors that are predictive of use in adults with cardiovascular disease (CVD), to identify the appropriate focus of intervention. Method(s): This is an analysis of the 2016 and 2017 Behavioral Risk Factor Surveillance System (BRFSS) data, an annual chronic disease and behavioral risk factor survey conducted by the CDC. The sample included the 110,028 respondents that reported a history of heart attack, angina/coronary heart disease or stroke. The rates of e-cigarette use were calculated, and a multiple logistic regression analysis was used to assess associated factors. Result(s): Among respondents with CVD, there was no change in e-cigarette use between 2016 and 2017 [3.1% to 2.8% adjusted OR (aOR) 1.08 (95% CI 0.95-1.24)]. The median age of e-cigarette users was 58 years and that of non-users was 69 years (p<0.0001). Women had a higher rate of ecigarette use compared with men [3.2% vs 2.7% aOR 1.17(1.04-1.31)]. Other factors associated with e-cigarette use were: race [blacks vs whites 1.9% vs 3% aOR 0.37(0.28-0.49], sexual orientation [LGBTQ vs non-LGBTQ 7.1% vs 2.9% aOR 1.56 (1.24-1.96)], smoking [current smokers vs non-smokers 11.1% vs 1.2% aOR 6.0 (5.3-6.8), smoking on some days vs never smoked 12.7% vs 0.24 aOR 51(35.4-73.5)], mental illness [6.4% vs 1.8% aOR 1.57 (1.37-1.810)] and state of residence (p<0.0001). Conclusion(s): In conclusion, there was no significant change in e-cigarette use between 2016-2017 among respondents with CVD. The highest rates of e-cigarette use were among subjects who were: smokers on some days, current smokers, LGBTQ and had a mental illness.
3377	A B Nguyen (2019///) Disaggregating Asian American and Native Hawaiian and Other Pacific Islander (AANHOPI) Adult Tobacco Use: Findings from Wave 1 of the Population Assessment of Tobacco and Health (PATH) Study, 2013-2014. Journal of racial and ethnic health disparities; 6<br><br>**Keywords:**<br>adult,article,*Asian American,cigar,*cigarette smoking,cohort analysis,control strategy,controlled study, electronic cigarette,female,hookah,human,Indian,juvenile,major clinical study,male,*Native Hawaiian, *Pacific Islander,*population health,prevalence,<br><br>**Abstract:** INTRODUCTION: Tobacco studies often combine data for Asian American and Native Hawaiian and Other Pacific Islander (AANHOPI) subgroups, masking subgroup differences. This study describes tobacco use (ever use and past 30-day use) among some disaggregated AANHOPI subgroups. METHOD(S): Data are from Wave 1 of the 2013-2014 Population Assessment of Tobacco and Health (PATH) Study, a nationally representative, longitudinal cohort study of civilian non-institutionalized adults and youth in the USA. The dataset contains a sample of 32,320 adults, of which 1623 identified as being of AANHOPI origin. Asian Americans further identified as being Asian Indian, Chinese, Filipino, Japanese, Korean, Vietnamese, or other Asian. Those who identified as Native Hawaiian, Guamanian or Chamarro, Samoan, and Other Pacific Islander were combined into an NHOPI group. Tobacco measures included ever and past 30-day use of cigarettes, e-cigarettes, cigars (traditional cigar, cigarillos, filtered cigar), hookah, and smokeless tobacco including snus pouches, and pipe tobacco. Unadjusted and adjusted estimates for tobacco use are reported by AANHOPI membership and sex. RESULT(S): In general, Asian Indians and Chinese had the lowest and NHOPI had the highest tobacco use prevalence compared to other AANHOPI subgroups. Males generally had higher prevalence compared to females. Prevalence varied by AANHOPI membership and tobacco product. Adjusted prevalence estimates were higher compared to unadjusted estimates for many subgroups, attenuating some unadjusted differences found between AANHOPI subgroups. DISCUSSION: Tobacco use varies by AANHOPI subgroup and product type.

	Unadjusted and adjusted analyses can be conducted as tobacco use differences in AANHOPI subgroups may be attributed to socio-economic status differences. Treating these distinct subgroups as a monolithic group may contribute to reliance on tobacco prevention and control strategies that may have limited impact on specific subgroups.
3379	N Nguyen, J L Barrington-Trimis, R Urman, J Cho, R McConnell, A M Leventhal, B Halpern-Felsher (2019///) Past 30-day co-use of tobacco and marijuana products among adolescents and young adults in California. Addictive Behaviors; 98<br><br>**Keywords:**<br>adolescent,*adolescent smoking,adult,article,*cannabis use,controlled study,female,frequency,human, male,*tobacco use,United States,young adult,cigar,electronic cigarette,hookah<br><br>**Abstract:** Background: Limited research among adolescents and young adults (AYA) has assessed tobacco and marijuana co-use in light of specific products. We examined the patterns of past 30-day co-use of tobacco and marijuana products, and the product-specific associations among past 30-day use of these substances. Method(s): Data from three school-based convenience samples of California AYA (aged 15-22) (Sample 1 = 3008; Sample 2 = 1419; Sample 3 = 466) were collected during 2016-2017. Proportions of past 30-day co-use of tobacco (e-cigarettes, cigarettes, hookah, cigars) and marijuana (combustible, vaporized, edible, blunt) were estimated. Multivariable logistic regression analyses examined associations between use of each tobacco and marijuana product for individual samples, then the pooled analysis calculated combined ORs. Result(s): In the three samples, 7.3-11.3% of participants reported past 30-day co-use. Combinations of e-cigarettes or cigarettes and combustible marijuana were the most common co-use patterns. Past 30-day use of e-cigarettes or cigarettes (vs. non-use) increased the odds of past 30-day use of all marijuana products [e-cigarettes: ORs (95%CI) ranging from 2.5 (1.7, 3.2) for edible marijuana to 4.0 (2.8, 5.2) for combustible marijuana; cigarettes: from 3.2 (2.1, 4.2) for vaporized marijuana to 5.5 (3.8, 7.3) for combustible marijuana]. Past 30-day use of hookah or cigars was positively associated with past 30-day use of three of four marijuana products, except for hookah and vaporized marijuana, and for cigars and combustible marijuana. Conclusion(s): Given various co-use patterns and significant associations among tobacco and marijuana products, interventions targeting AYA should address co-use across the full spectrum of specific products for both substances.Copyright © 2019 Elsevier Ltd
3381	N E Nicksic, A J Barnes (2019///) Is susceptibility to E-cigarettes among youth associated with tobacco and other substance use behaviors one year later? Results from the PATH study. Preventive Medicine; 121<br><br>**Keywords:**<br>adolescent,alcohol consumption,article,attitude to health,child,cigarette smoking,controlled study, female,high risk behavior,human,male,*population exposure,priority journal,risk factor,*substance use, *tobacco use,alcohol,cannabis,*electronic cigarette<br><br>**Abstract:** E-cigarettes are the most commonly used tobacco product among youth. In addition to harm potential, e-cigarette use is associated with initiating cigarette smoking. Limited research exists whether susceptibility to e-cigarette use is a risk factor for future tobacco and other substance use initiation. This study examined associations between baseline e-cigarette susceptibility and initiation and past 30-day use of e-cigarettes and cigarettes as well as initiation of marijuana and alcohol one year later, after adjusting for other risk factors and sociodemographic confounders. The study sample consisted of 5156 nationally representative youth (12-17 years) who completed both waves 1 (2013-2014) and 2 (2014-2015) of the Population Assessment of Tobacco and Health (PATH) study and were never users of tobacco, marijuana, and alcohol in Wave 1. Youth who were susceptible to e-cigarettes had increased odds of initiating e-cigarettes (adjusted OR: 2.22, 95% CI: 1.55-3.18), marijuana (aOR: 1.66, 95% CI: 1.12-2.46), and alcohol (aOR: 1.61, 95% CI: 1.26-2.06) between waves, as well as past reporting 30-day e-cigarette use (aOR: 3.64, 95% CI: 1.93-6.89) in Wave 2. Additionally, cigarette susceptibility, but not e-

	cigarette susceptibility, was associated with cigarette initiation (aOR: 3.36, 95% CI: 1.95-5.82) and past 30-day use (aOR: 2.83, 95% CI: 1.34-5.97). Prevention policies, as well as future research, could target youth susceptible to e-cigarettes to reduce the current trends on the use of these alternative tobacco products. Such efforts may also reduce the use of cigarettes and other substances.Copyright © 2019 Elsevier Inc.
3390	Iqbal Nousheen, A Khan Zain, Anwar Syed Muhammad Hamza, Irfan Omar, Irfan Babar, Mushtaq Ammar, Bibi Maria, Siddiqui Faraz, A Khan Javaid (2018///) Electronic cigarettes use and perception amongst medical students: a cross sectional survey from Sindh, Pakistan. BMC research notes; 11<br><br>**Keywords:**<br>Awareness,Cross-Sectional Studies,*Electronic Nicotine Delivery Systems,Female,*Health Knowledge, Attitudes, Practice,Humans,Logistic Models,Male,Multivariate Analysis,Pakistan,Perception,Students, Medical/px [Psychology],*Students, Medical/sn [Statisti<br><br>**Abstract:** OBJECTIVE: The manufacturers of electronic cigarettes (e-cigarettes) are actively marketing their product through electronic and social media. Undergraduate medical students are expected to have better knowledge and awareness as they directly interact with patients in their training, The purpose of this study is therefore, to determine knowledge, use and perception regarding e-cigarettes among medical students from Sindh, Pakistan., RESULTS: A cross-sectional study was conducted between 1st July and 30th September 2016 at five different medical colleges situated in the second largest province of Sindh, Pakistan. The data was collected through a structured, self-administered questionnaire. Of the 500 students, the mean age was 21.5 +/- 1.7 years and 58% were females. Over (65.6%) students were aware of e-cigarettes, 31 (6.2%) reported having used e-cigarettes, of whom 6 (1.2%) self-reported daily use. Users of conventional tobacco products were significantly more likely to have heard of e-cigarettes (87.6% vs 51.6%, p < 0.001) and having used them (13.9% vs 1.3%, p < 0.001). On multivariable logistic regression analysis we found a strong association of e-cigarette use with consumption of conventional cigarettes [OR: 10.6, 95% CI 3.6-30.8, p < 0.001], use of smokeless tobacco products [OR: 7.9, 95% CI 2.7-23.4, p < 0.001] however a weak association was observed for Shisha use [OR: 3.05, 95% CI 0.9-9.6, p = 0.05].
3391	A Oakly, R Edwards, G Martin (2019///) Prevalence of e-cigarette use from a nationally representative sample in New Zealand. Addictive Behaviors; 98<br><br>**Keywords:**<br>adolescent,adult,article,controlled study,demography,female,human,informed consent,lifestyle,male, Maori (people),middle aged,New Zealand,population research,*prevalence,public health,risk factor, sample size,*smoking,smoking cessation,young adult,*electr<br><br>**Abstract:** Objective: To explore the prevalence of e-cigarette use in New Zealand in a nationally representative sample aged 15 years and over. Method(s): The Health and Lifestyles Survey (HLS) is a biennial face-to-face in-house survey of New Zealand adults aged 15 years or over. The HLS was completed by 3854 participants in 2016. Result(s): 17% of adults have tried e-cigarettes, while only 2% reported current use. The prevalence of ever use of e-cigarettes was greater in young and middle-aged adults, compared with older adults, Maori compared with NZ European, and current and former smokers, compared to never smokers. Current smokers and recent quitters displayed the highest levels of e-cigarette use, with never smokers reporting low ever use (2.8%) and no regular use. Following adjusted analyses only current smokers and recent quitters were more likely to report current or daily e-cigarette use. Conclusion(s): Although 17% of adults report having tried e-cigarettes, very few report current use. It is possible that difficulty of use, compounded by lack of support, is preventing conversion to regular use or successful use as a quitting aid. Public health initiatives should inform individuals how to correctly use e-cigarettes optimally for smoking cessation, ideally targeting this messaging toward younger and middle-aged adults, Maori, current smokers, and recent quitters.Copyright © 2019 Elsevier Ltd

3393	S Odani, K Marynak, B Armour, I Agaku (2018///) Electronic cigarette use and conventional cigarette smoking initiation among youth, United States, 2015-2016. Tobacco Induced Diseases; 16<br><br>**Keywords:**<br>cigar,*cigarette smoking,controlled study,*electronic cigarette,high school student,hookah,human, *juvenile,major clinical study,outcome variable,smokeless tobacco,*United States,conference abstract<br><br>**Abstract:** Background: Electronic cigarettes (e-cigarettes) are the most commonly used tobacco product among U.S youth. We used cross-sectional, nationally representative data of U.S. middle and high school students to examine the association between e-cigarette use and cigarette smoking initiation. Method(s): Data were from the 2015 and 2016 National Youth Tobacco Surveys, a school-based survey of U.S. 6th-12th graders (pooled N=38,386). Questions on current age and age at initiation of different tobacco products were used to assess temporality. The study included 35,775 students who had never smoked conventional cigarettes five years before the survey (i.e., baseline), including never-smokers and those who first smoked < 5 years ago. Baseline never smokers were classified by e-cigarette use status into: (1) those who ever used e-cigarettes on/before or without ever smoking cigarettes; or (2) those who had never used e-cigarettes, or started only after initiating cigarette smoking. The outcome variables were cigarette smoking at pre-determined periods: any time within the past 5 years; past 1 year; past 6 months; past 30 days; and past 7 days. Adjusted odds ratios (AOR) were calculated using multivariable logistic regression. The models controlled for socio-demographic characteristics and use of smokeless tobacco, cigars, and hookah on/before cigarette smoking initiation. Result(s): Among baseline never cigarette smokers, 17.4% used e-cigarettes, and 16.7% initiated cigarette smoking within the past 5 years. Those who used e-cigarettes on/before ever smoking cigarettes had higher odds of smoking cigarettes than those who did not at all periods assessed: any time within the past 5 years (AOR=2.61); past 1 year (AOR=3.18), past 6 months (AOR=2.59), past 30 days (AOR=1.75), and past 7 days (AOR=1.38) (all p< 0.05). Conclusion(s): These cross-sectional findings reveal that e-cigarette use was associated with conventional cigarette smoking initiation among U.S. youth. Comprehensive efforts are important to prevent and reduce all forms of youth tobacco product use.
3394	M Olfson, M M Wall, S M Liu, R S Sultan, C Blanco (2019///) E-cigarette Use Among Young Adults in the U.S. American Journal of Preventive Medicine; 56<br><br>**Keywords:**<br>adult,African American,age distribution,article,cigarette smoking,controlled study,correlation coefficient, DSM-5,female,health behavior,Hispanic,human,income,logistic regression analysis,major clinical study, male,prevalence,smoking cessation,test retest<br><br>**Abstract:** Introduction: Use of e-cigarettes is increasing among young adults in the U.S. Whether e-cigarette use serves as an aid to smoking reduction or cessation among young adults remains a matter of contention. This analysis examines patterns of e-cigarette use in relation to cigarette smoking in a nationally representative sample of U.S. young adults. Method(s): Data were analyzed from nationally representative U.S. adults, aged 18 to 35years (N=12,415), in the 2012-2013 National Epidemiologic Survey on Alcohol and Related Conditions-III. Logistic regression assessed associations between e-cigarette use and smoking intensity, continuity, and reduction while controlling for several potential confounding factors. Data were analyzed in 2018. Result(s): Among cigarette smokers, e-cigarette use was associated with higher odds of tobacco use disorder (AOR=2.58, 95% CI=1.73, 3.83) and daily cigarette smoking (AOR=1.67, 95% CI=1.73, 3.83). Among adults aged 26-35years, e-cigarette use was also associated with heavy cigarette smoking (AOR=2.01, 95% CI=1.09, 3.74). Among lifetime smokers, e-cigarette use was associated with lower odds of stopping smoking (AOR=0.14, 95% CI=0.08, 0.23) and lower odds of a 50% reduction in cigarettes smoked per day (AOR=0.63, 95% CI=0.43, 0.93). Only 13.1% of young adults who ever used e-cigarettes reported using them to help stop or quit smoking. Conclusion(s): Use of e-cigarettes by U.S. young adults, most of which is not intended to help reduce

	smoking, is related to more rather than less frequent and intensive cigarette smoking.Copyright © 2019 American Journal of Preventive Medicine
3397	A Wackowski Olivia, M Jane Lewis (2018///) E-cigarette brand mocks tobacco control warning labels. Tobacco control; 27<br><br>**Keywords:**<br>*Advertising as Topic,*Electronic Nicotine Delivery Systems,Humans,*Product Labeling,Product Packaging<br><br>**Abstract:**
3399	Ganz Ollie, L Johnson Amanda, M Cohn Amy, Rath Jessica, Horn Kimberly, Vallone Donna, C Villanti Andrea (2018///) Tobacco harm perceptions and use among sexual and gender minorities: findings from a national sample of young adults in the United States. Addictive Behaviors; 81<br><br>**Keywords:**<br>Adolescent,Adult,Case-Control Studies,Cigarette Smoking/ep [Epidemiology],*Cigarette Smoking/px [Psychology],Female,*Health Knowledge, Attitudes, Practice,Humans,Male,*Sexual and Gender Minorities/px [Psychology],Sexual and Gender Minorities/sn [Statist<br><br>**Abstract:** INTRODUCTION: In the United States, the prevalence of tobacco use among sexual and gender minorities (SGM) is higher compared to their non-SGM counterparts. Tobacco harm perceptions have gone largely unexamined as a potential mechanism supporting disproportionate tobacco use among this population. The purpose of this study was to examine differences between SGM and non-SGM young adults in harm perceptions of various tobacco products and tobacco use behavior and whether low tobacco-related harm perceptions moderate the relationship between identifying as a SGM and tobacco use behavior., METHODS: This study used data from Wave 10 of the Truth Initiative Young Adult Cohort Study, a sample of U.S. young adults (ages 18-34). Data were collected from September to October 2016 and the study sample included 3089 individuals. Demographics, past 30-day use of cigarettes, little cigars/cigarillos/bidis and electronic cigarettes were assessed. Absolute and relative harm perceptions of these products were also examined., RESULTS: Identifying as a SGM and low tobacco harm perceptions were found to be positively associated with past 30-day tobacco use. There was no interaction between SGM status and harm perceptions on past 30-day tobacco use CONCLUSIONS: Findings confirm that SGM young adults continue to disproportionately use tobacco products, compared to non-SGM young adults. More research is needed to understand moderators of the relationship between SGM status and tobacco use in young adults. Copyright © 2018 Elsevier Ltd. All rights reserved.
3400	Owotomo Olusegun, Maslowsky Julie, Loukas Alexandra (2018///) Perceptions of the Harm and Addictiveness of Conventional Cigarette Smoking Among Adolescent E-Cigarette Users. The Journal of adolescent health : official publication of the Society for Adolescent Medicine; 62<br><br>**Keywords:**<br>Adolescent,Behavior, Addictive/px [Psychology],*Cigarette Smoking/ae [Adverse Effects],Cross-Sectional Studies,*Electronic Nicotine Delivery Systems/mt [Methods],Female,Health Knowledge, Attitudes, Practice,Humans,Male,Peer Group,*Perception/de [Drug Ef<br><br>**Abstract:** PURPOSE: Although existing evidence indicates that e-cigarette use is a risk factor for cigarette smoking initiation, mechanisms of this association are not yet known. E-cigarette users perceive e-cigarette use to be less harmful relative to conventional cigarettes, but their absolute perceptions of addictiveness of conventional cigarette smoking are unknown. This study examines how e-cigarette users compare with nonusers (non-e-cigarette users/nonconventional cigarette smokers), conventional cigarette smokers, and dual users on perceptions of harm and the addictiveness of conventional cigarette

	smoking and on other known predictors of cigarette smoking such as peer smoking, influence of antismoking ads, and risk-taking propensity., METHODS: National samples of 8th- and 10th-grade students from 2014 and 2015 (N = 14,151) were obtained from the Monitoring the Future Study. Multivariate logistic regression models were used to examine relationships between adolescent smoking status and perceptions of harm and the addictiveness of conventional cigarette smoking while controlling for potential confounders., RESULTS: E-cigarette users had lower perceptions of the addictiveness of conventional cigarette smoking compared with nonusers but higher than cigarette smokers and dual users. E-cigarette users reported lower influence by antismoking ads, more conventional cigarette-smoking peers, and greater risk-taking propensity than nonusers. E-cigarette users and cigarette smokers did not differ in their perceived harm of conventional cigarette smoking or in their risk-taking propensity., CONCLUSIONS: E-cigarette users' attitudes and perceptions regarding conventional cigarette smoking may leave them vulnerable to becoming conventional cigarette smokers. Future studies should explore the prospective relationship between smoking-related perceptions of conventional cigarette smoking among e-cigarette users and the onset of cigarette smoking. Copyright © 2017 The Society for Adolescent Health and Medicine. Published by Elsevier Inc. All rights reserved.
3404	A Osman, S D Kowitt, L M Ranney, C Heck, A O Goldstein (2018///) Trends and racial disparities in mono, dual, and poly use of tobacco products among youth. Nicotine and Tobacco Research; 20<br><br>**Keywords:**<br>adolescent,American Indian,article,Asian,Black person,Caucasian,female,high school student,Hispanic, human,male,Native Hawaiian,Pacific Islander,prevalence,priority journal,*race difference,smoking, *tobacco use,trend study<br><br>**Abstract:** Introduction We examined racial disparities in mono, dual, and poly use of tobacco products including whether racial disparities changed over time. Methods We analyzed data on high school students from the North Carolina Youth Tobacco Survey collected in 2011, 2013, and 2015. Dual and poly use included use of two and three or more tobacco products, respectively, in the past month. Multinomial regression models assessed racial differences and changes over time in mono, dual, and poly use. Data include product combinations most commonly used by youth from different racial groups. Results In total, 24% (in 2011) and 26% (in 2013 and 2015) of students used tobacco products. No significant changes over time were observed in mono (12%) or dual use (6%). Poly use was 6%, 8%, and 7% in 2011, 2013, and 2015, respectively. Relative to nonuse of tobacco, White students had a higher relative risk than Blacks for mono use. Whites and Hispanics had a higher relative risk than Blacks for dual and poly use. Observed racial differences in tobacco use did not change over time. Types of tobacco products used varied by year and race. In 2015, e-cigarette was the most commonly used product among mono users from all racial groups. Conclusions Substantial racial variation persists over time in mono and multiple tobacco product use among North Carolina youth, including racial variation in the types of tobacco products used. Research and policy efforts should examine and eliminate factors that drive multiple tobacco use and racial disparities in use among youth. Implications This study reports on racial disparities in mono and multiple tobacco product use among youth. White and Hispanic youth have higher relative risk for dual and poly use of tobacco products than Black youth. In addition, significant racial variation exists in the types of tobacco products used among youth mono, dual, and poly users, with cigarettes, cigars, smokeless tobacco, and e-cigarettes being the most commonly used products. Patterns of multiple tobacco product use vary by race and may warrant tailored prevention efforts. Strengthening tobacco control regulations for other tobacco products than cigarettes is critical to reduce multiple tobacco product use among youth.Copyright © The Author(s) 2018. Published by Oxford University Press on behalf of the Society for Research on Nicotine and Tobacco.
3405	A Osman, S D Kowitt, L M Ranney, C Heck, A O Goldstein (2019///) Risk factors for multiple tobacco product use among high school youth. Addictive Behaviors; 99<br><br>**Keywords:**

	advertising,article,controlled study,electronic cigarette,friend,*high school student,household,human, *juvenile,major clinical study,marketing,North Carolina,*passive smoking,perception,risk assessment, *risk factor,vapor<br><br>**Abstract:** Purpose: To examine how youth multiple tobacco product (MTP) users differ relative to non-users and to single-product users on risk factors at multiple levels of influence. Method(s): We analyzed data on high school students from the North Carolina Youth Tobacco Survey, 2015 (n = 2922). Single- and MTP use were defined as use of one, or two or more tobacco products in the past month, respectively. Multinomial regressions estimated the association between risk factors and MTP use compared to single-product use and non-use of tobacco. Risk factors included intra-personal (e.g., harm perceptions), and interpersonal (e.g., household, peer tobacco use, secondhand smoke or vapor, and advertising) factors. Result(s): Of students, 12% and 13% were single product and MTP users, respectively. Many differences emerged between MTP users and non-tobacco users, with MTP users showing lower harm perceptions, higher perceived social benefits of smoking, significantly higher relative risk of having friends who use tobacco (RRR = 4.79, 95% CI 3.42, 6.70), of exposure to secondhand e-cigarette vapor (RRR = 1.35, 95% CI 1.23, 1.48), and of being receptive to tobacco marketing (RRR = 4.01, 95% CI 2.87, 5.61). Fewer differences emerged between MTP and single product users with MTP users having significantly higher relative risk of having friends who use tobacco (RRR = 2.31 95% CI 1.73, 3.07), of exposure to secondhand vapor (RRR = 1.10, 95% CI 1.02, 1.18), and of being receptive to tobacco marketing (RRR = 1.71, 95% CI 1.17, 2.50). Conclusion(s): Efforts that target multiple tobacco product use should increase youth tobacco-related harm perceptions, and protect youth from social, peer, and industry influences.Copyright © 2019 Elsevier Ltd
3407	V L Owens, T Ha, J N Soulakova (2019///) Widespread use of flavored e-cigarettes and hookah tobacco in the United States. Preventive Medicine Reports; 14<br><br>**Keywords:**<br>adult,article,*cigarette smoking,clinical evaluation,controlled study,ethnic group,female,health survey, human,male,manufacturing industry,population,practice guideline,prevalence,priority journal,resident, smoking,tobacco,tobacco use,United States,*water<br><br>**Abstract:** In 2009, the U.S. Food and Drug Administration banned the sale of flavored cigarettes (excluding menthol) in the U.S. However, the sale of flavored e-cigarettes (FE) and flavored hookah tobacco (FHT) is still legal. In this study (conducted in the US in the period from October 2018 to February 2019), we estimated the prevalence of use of FE and FHT across users' sociodemographic characteristics (e.g., age, sex, race/ethnicity) and identified the key characteristics of tobacco users associated with use of FE and FHT. We analyzed the 2014-2015 Tobacco Use Supplement to the Current Population Survey data for current e-cigarette (n = 3691) and hookah tobacco (n = 658) users. Among e-cigarette users, 65.5% used FE; and among hookah tobacco users, 87.3% used FHT. Among e-cigarette users, the prevalence of use of FE was significantly higher for 18-24 year-old than 45+ year-old adults; women than men; Southern than Northeastern residents; and never smokers of regular cigarettes than current smokers of regular cigarettes (all adjusted p's < 0.05). Among hookah tobacco users, the rates of FHT use were significantly higher for women than men, and never smokers of regular cigarettes than current smokers (all adjusted p's < 0.05). Because availability and accessibility of flavored tobacco products may promote tobacco use, revising regulatory guidelines concerning manufacturing and distribution of FE and FHT may help reduce the popularity of emerging tobacco products. Implications: Among users of emerging tobacco products such as e-cigarettes and hookah tobacco, use of flavored products is very common. Among e-cigarette users, 66% consumed flavored e-cigarettes; and among users of hookah tobacco, 87% consumed flavored hookah tobacco. Use of flavored products was especially common among users who were young adults, women, or never and former smokers of regular cigarettes. Availability of emerging tobacco products in many different flavors could be a reason for tobacco use

	initiation and continued use of tobacco after cessation of regular-cigarette smoking.Copyright © 2019 The Authors
3408	O Owotomo, J Maslowsky (2018///) Adolescent Smoking Susceptibility in the Current Tobacco Context: 2014-2016. American journal of health behavior; 42<br><br>**Keywords:**<br>adolescent,*adolescent smoking,advertising,article,cigar,cigarette smoking,controlled study,electronic cigarette,female,human,landscape,major clinical study,male,monitoring,perception,risk factor,*smoking<br><br>**Abstract:** OBJECTIVES: We examined perceptions and behaviors associated with smoking susceptibility among adolescents in the current tobacco landscape. METHOD(S): Participants were 8th and 10th grade never-smokers of conventional cigarettes (N = 19,853) from Monitoring the Future surveys (2014-2016). Using weighted multivariable logistic regression, we examined risk factors for smoking susceptibility: alternative tobacco product use (e-cigarettes, large cigars, little cigars/cigarillos, and flavored little cigars/cigarillos), ownership of tobacco promotional items (TPIs), access to cigarettes, perceived influence of antismoking advertisements, and perceived addictiveness of conventional cigarette smoking. RESULT(S): Among never-smokers of conventional cigarettes, 16.7% were susceptible to smoking, 6.2% were past 30-day alternative tobacco product users, and 3.5% owned TPIs. Alternative tobacco product use, ownership of TPIs, and easy access to cigarettes were associated with increased likelihood of smoking susceptibility. Perceived great influence by antismoking ads and higher perceived addictiveness of conventional cigarette smoking were associated with lower odds of smoking susceptibility. CONCLUSION(S): Alternative tobacco product use, ownership of TPIs, easy access to cigarettes, low influence by antismoking ads, and low perceptions of the addictiveness of conventional cigarettes are significant and actionable risk factors for smoking susceptibility among today's adolescents.
3410	D Owusu, H M Mamudu, C Collins, C Robertson, L Wang, B Rafie, M A Littleton (2019///) The Usage and Associated Factors of Alternative Tobacco Products among School-going Youth in Central Appalachia. Journal of health care for the poor and underserved; 30<br><br>**Keywords:**<br>adult,article,cigar,electronic cigarette,female,friend,human,*juvenile,major clinical study,male,middle school,multivariate logistic regression analysis,*smokeless tobacco,smoking,Tennessee<br><br>**Abstract:** OBJECTIVE: To examine alternative tobacco product (ATP) use and associated factors among middle school students in Appalachian Tennessee. METHOD(S): Data (2015-2016; N=573) from school-based tobacco surveys in 11 middle schools in Appalachian Tennessee were analyzed. Multiple logistic regression model described factors associated with ATP use. RESULT(S): More than one-tenth (13.2%) of participants have ever used ATPs. Approximately 9%, 7%, 6%, and 2% of participants have ever used e-cigarettes, cigarettes, smokeless tobacco, and little cigars or cigarillos, respectively. The following were significantly associated with ever use of ATPs: believing tobacco users have more friends, owning tobacco-branded item(s), living with a tobacco user, having ever smoked cigarettes, and living in homes where smoking is allowed. CONCLUSION(S): More than one in 10 of participants have ever used at least one ATP, and association with desirable objects and situations may promote ATP use. Interventions should seek to reduce these positive images and make all tobacco products unappealing.
3415	K M Palipudi, C Jones, K A Lee, E Pun, R Arrazola, I Ahluwalia (2018///) Awareness and use of electronic cigarettes among youth: Evidence from the Global Youth Tobacco Survey 2012 - 2015. Tobacco Induced Diseases; 16<br><br>**Keywords:**<br>adolescent,Albania,*awareness,Belize,child,controlled study,*electronic cigarette,female,gender,Georgia (republic),*girl,human,Kazakhstan,Macao,male,Palestine,Paraguay,Peru,prevalence,refugee,Russian

	Federation,sample size,Seychelles,student,Sudan,*toba<br><br>**Abstract:** Background: Globally, evidence on the awareness and use of electronic cigarettes (e-cigarettes) is limited. We examined the awareness and current use of e-cigarettes among male and female students aged 13-15 years old across 16 countries or regions that conducted the Global Youth Tobacco Survey (GYTS). Method(s): GYTS is a cross-sectional, nationally representative, school-based survey of students aged 13-15 years. Data from the16 countries or regions were used to examine student awareness (ever heard of) and current use (in the past 30 days) of e-cigarettes. Sample sizes across the 16 countries or regions ranged from 962 in Georgia to 5,153 in Paraguay and response rates ranged from 75.4% in Georgia to 99.2% in Sudan. Weighted point estimates and 95% confidence intervals (CI) were calculated and statistically significant differences (p< 0.05) by gender were assessed using a two-sample t-test. Result(s): Awareness of e-cigarettes ranged from 19.6% (95% CI: 16.1-23.6) in Peru to 93.0% (95% CI: 91.4-94.3) in the Moscow region of Russia. Across all sites, only Seychelles and United Nations Relief and Works Agency for Palestine Refugees in the Near East (UNRWA) had significantly higher awareness of e-cigarette among boys than girls. Current use of e-cigarettes ranged from 1.6% (95% CI: 1.1-2.5) in Kazakhstan to 15.4% (95% CI: 12.6-18.5) in UNRWA. In Albania, Belize, Macao, Seychelles, and UNRWA, boys had a significantly higher prevalence of current e-cigarette use than girls. Conclusion(s): The findings show that awareness and use of e-cigarettes varies considerably across 16 countries/regions. GYTS is a viable platform to measure tobacco use among youth, including emerging products such as e-cigarettes.
3431	M R Peltier, A F Waters, M R Roys, S A Stewart, K M Waldo, A L Copeland (2019///) Dual users of e-cigarettes and cigarettes have greater positive smoking expectancies than regular smokers: a study of smoking expectancies among college students. Journal of American college health : J of ACH;<br><br>**Keywords:**<br>adult,appetite,article,body weight control,*college student,controlled study,disease course,*electronic cigarette,*expectancy,female,human,major clinical study,male,questionnaire,reinforcement,*smoking<br><br>**Abstract:** OBJECTIVE: In the present study, we sought to determine the differences among college students using e-cigarettes, cigarettes, or both products (dual users) on smoking outcome expectancies and the role of smoking expectancies and e-cigarette use in cessation attempts. PARTICIPANTS: We surveyed 1,370 undergraduate college students from November 2014 to November 2016. METHOD(S): Participants completed questionnaires regarding demographics, smoking status/history, and expectancies. RESULT(S): Dual users reported significantly longer smoking histories and more past quit attempts than other smoking groups. Those reporting dual use reported higher expectancies on positive/negative reinforcement and appetite/weight control subscales of the SCQ. E-cigarette use was a negative predictor of cessation attempts. CONCLUSION(S): College students appear to be less motivated to use e-cigarettes for cessation and dual users endorsed higher levels of smoking expectancies previously shown to negatively impact treatment outcomes. Students, especially dual users, need targeted interventions to address the dangers of using tobacco products.
3436	A Perez, P Morello, S Braun, J Thrasher, R Mejia (2018///) Family and school socioeconomic status as predictors of tobacco and e-cigarette use in adolescents: A study from a perspective of material, human, and social capital. Tobacco Induced Diseases; 16<br><br>**Keywords:**<br>adolescent,city,controlled study,*electronic cigarette,female,human,major clinical study,male,medical research,middle school,nuclear family,*smoking,*social capital,*social status,sociological theory,student, conference abstract<br><br>**Abstract:** Background: The contradictory findings about the role of the socioeconomic status (SES) in accounting for substance use in adolescence can be explained by the lack of consensus on how best to conceptualize and measure SES. Based on Coleman's social theory, this study examines the association of

	family and school SES with tobacco and e-cigarette use. Method(s): Data came from a cross-sectional, school-based survey of middle-school early adolescents attending 33 schools in three Argentinian cities (n=3,110). Family SES was evaluated using: 1) family affluence scale (FAS) for material capital, 2) parent education for human capital, and 3) nuclear family and parentadolescent communication for social capital. A school SES index was computed aggregating FAS and parent education from students. Multilevel logistic regression models regressed current tobacco use (in the past 30 days) and ever e-cigarette use on family and school-level SES, controlling for sex and age. Result(s): 17.7% students were current tobacco smokers and 7.7% have ever used e-cigarette. At family level, material capital was unassociated with tobacco smoking, but positively associated with e-cigarette use; human capital was unassociated with both tobacco smoking and e-cigarette use; and social capital was inversely associated with both tobacco smoking and e-cigarette use. After adjusting for family SES, students from schools with higher SES had lower likelihood of tobacco smoking (OR=0.25, 95%CI 0.09- 0.74) and higher likelihood of e-cigarette use (OR=4.36, 95%CI 1.25-15.23) than those from schools with lower SES. Conclusion(s): Family capital measures are differentially related to student substance use, pointing out that SES indicators are not interchangeable. School SES has an independent effect on substance use above and beyond that of family SES. These findings emphasize the need for SES conceptualization and consistent selection of indicators in order to study the effect of SES on health research and its role in identifying students at-risk of substance use.
3438	K Perialathan, A B Rahman, K H Lim, Y Adon, A Ahmad, N Juatan, N Jaafar (2018///) Prevalence and associated factors of ever use of electronic cigarettes: Findings from a hospitals and health clinics study based in Malaysia. Tobacco Induced Diseases; 16<br><br>**Keywords:**<br>adult,age,article,attitude to health,cross-sectional study,demography,educational status,female,health care facility,health education,human,Malaysia,male,middle aged,prevalence,priority journal,product safety,public hospital,questionnaire,*risk factor,s<br><br>**Abstract:** INTRODUCTION Electronic cigarettes (e-cigarettes) are new smoking devices that have gained popularity recently. However, there is limited evidence on e-cigarette consumption in Malaysia. This study aims to determine the prevalence, risk factors and perception associated with e-cigarette use among those attending government hospitals and health clinics in Malaysia. METHODS A cross-sectional hospital-based study was conducted in seven public hospitals and health clinics in Malaysia, which were selected through a two-stage cluster sampling. A validated questionnaire was used to obtain data from the selected participants. Multivariable logistic regression was employed to determine the association between sociodemographics and perceptions of e-cigarette use. RESULTS Almost three-quarters (73.6%; n=923/1254) of participants were aware of e-cigarettes and 13.2% (n=122/923) reported having ever used e-cigarettes. The prevalence was significantly higher among males (18.1%), smokers (21.4%), and younger age group 18-34 years (30.2%). Ever users showed favourable perceptions towards e-cigarettes compared to non-users (23.3% vs 30.14%, p<0.001). Multivariable logistic regression revealed that current smokers, younger age group and those possessing a positive perception towards e-cigarettes were likely to be ever users of e-cigarettes. CONCLUSIONS The study showed that the awareness level of e-cigarettes was high amongst the population but the prevalence of ever e-cigarette user was moderate. Most of the ever e-cigarette users were male, current smokers, young adults and those with favourable perceptions towards e-cigarettes. Therefore, effective health educational activities regarding safe usage of e-cigarettes targeting those group identified in this study are warranted to reduce the negative outcomes from the use of this product.Copyright © 2018 Perialathan K.
3443	A Persoskie, E K O'Brien, K Poonai (2019///) Perceived Relative Harm of Using E-Cigarettes Predicts Future Product Switching among U.S. Adult Cigarette and E-Cigarette Dual Users. Addiction (Abingdon, England);<br><br>**Keywords:**<br>adult,article,*cigarette smoking,controlled study,*electronic cigarette,female,human,major clinical study,

	male,perception,prospective study,smoking,United States<br><br>**Abstract:** BACKGROUND AND AIMS: People's perceptions of the harmfulness of e-cigarettes, compared with cigarettes, may influence their product use decisions. We tested if perceiving e-cigarettes as less harmful than cigarettes predicted whether cigarette and e-cigarette dual users switched their product use status one year later, becoming exclusive e-cigarette users, exclusive cigarette smokers, or non-users of both product types. DESIGN: Longitudinal analyses of Waves 2 (2014-15) and 3 (2015-16) of the prospective, national Population Assessment of Tobacco and Health (PATH) Study. SETTING: United States. PARTICIPANTS: Adults who reported using both cigarettes and e-cigarettes within the past 30-days at Wave 2, reported their perceptions of e-cigarette harm at Wave 2, and reported whether they used cigarettes and e-cigarettes within the past 30-days at Wave 3 (n=2211). MEASUREMENTS: The key predictor was Wave 2 perceptions of e-cigarette harm compared with cigarettes ("Less harmful," "About the same," "More harmful," or "Don't know"). The key outcome was Wave 3 past 30-day use of e-cigarettes and cigarettes, classified into four categories: exclusive e-cigarette use (i.e., use of e-cigarettes but not cigarettes), exclusive cigarette smoking (i.e., use of cigarettes but not e-cigarettes), dual use of both product types, and non-use of both product types. FINDINGS: At Wave 2, 59.4% of dual users perceived e-cigarettes as less harmful than cigarettes (95%CI: 56.9,61.9). Compared with those with other perceptions of e-cigarette harm, dual users who perceived e-cigarettes as less harmful than cigarettes were more likely to become exclusive e-cigarette users one year later (7.5% vs. 2.7%; AOR=2.9, 95%CI: 1.7-4.8), more likely to remain dual users (39.6% vs. 29.9%; AOR=1.5, 95%CI: 1.2-1.8), less likely to become exclusive cigarette smokers (44.8% vs. 59.4%; AOR=0.6, 95%CI: 0.5-0.7), and similarly likely to become non-users of both product types (8.2% vs. 8.0%; AOR=1.1, 95%CI: 0.7-1.7). CONCLUSION(S): U.S. adult dual users of e-cigarettes and cigarettes who perceive e-cigarettes as less harmful than cigarettes appear to be more likely to switch to exclusive e-cigarette use, more likely to remain dual users, and less likely to switch to exclusive cigarette use one year later than dual users with other perceptions of e-cigarette harm.Copyright This article is protected by copyright. All rights reserved.
3444	M F Pesko, J M Currie (2019///) E-cigarette minimum legal sale age laws and traditional cigarette use among rural pregnant teenagers. Journal of Health Economics; 66<br><br>**Keywords:**<br>adolescent,*adolescent pregnancy,*adolescent smoking,adult,*age,article,attitude to health,cigarette smoking,cross sectional study,female,health care policy,health education,health impact assessment,high risk pregnancy,human,law,longitudinal study,mater<br><br>**Abstract:** Teenagers under 18 could legally purchase e-cigarettes until states passed minimum legal sale age laws. These laws may have curtailed teenagers' use of e-cigarettes for smoking cessation. We investigate the effect of e-cigarette minimum legal sale age laws on prenatal cigarette smoking and birth outcomes for underage rural teenagers using data on all births from 2010 to 2016 from 32 states. We find that the laws increased prenatal smoking in by 0.6 percentage points (pp) overall. These effects were concentrated in prepregnancy smokers, with no effect on prepregnancy non-smokers. These results suggest that the laws reduced cigarette smoking cessation during pregnancy rather than causing new cigarette smoking initiation. Our results may indicate an unmet need for assistance with smoking cessation among pregnant teenagers.Copyright © 2019 Elsevier B.V.
3455	B Philipenko, E Penz, N Shojaati, L K Kreuger, N Osgood, K Whittemore (2018///) Perceptions and patterns of e-cigarette use in saskatchewan-a pilot study using mobile technology. Canadian Journal of Respiratory Critical Care and Sleep Medicine; 2<br><br>**Keywords:**<br>adult,clinical article,controlled study,craving,female,habit,human,male,mobile phone,*perception,*pilot study,prospective study,questionnaire,*Saskatchewan,smoking cessation,social environment,*vaping, conference abstract

	**Abstract:** Introduction: Electronic cigarettes have achieved rapid market penetration despite uncertainty about their safety, efficacy as a smoking cessation aide, and relationship to tobacco-related behaviours. This study utilizes mobile technology to examine users' perceptions of e-cigarettes, and to compare the patterns of use between e-cigarettes and tobacco cigarettes. Method(s): This is a prospective observational pilot study that recruited participants through a snowball technique. Participants were e-cigarette users 18 years of age or older living in Saskatoon, Saskatchewan. An entrance survey was administered to document individual perceptions of e-cigarette use. A novel Ethica Health application for mobile phones was utilized to administer questionnaires regarding tobacco or e-cigarette use over a three-month period. Questionnaires collected information on smoking or vaping events, the social context of use, location, inciting events, and other factors. Result(s): There were nine participants in the study. In the entrance survey, 7/9 (78%) self-identified as a concurrent tobacco user. The most common reason for e-cigarette use was to help quit smoking tobacco in 3/9 (33%) of participants. There were 1550 reports of smoking or vaping; 1431 (92%) of these were vaping events. Smoking and vaping most often occurred alone, accounting for 84/119 (71%) and 1002/1378 (73%) of responses respectively. The most common location for both smoking and vaping was at home; however, a larger proportion of vaping occurred at home or in the vehicle compared to smoking. The most common factor leading to smoking tobacco was craving 33/ 133 (25%), while e-cigarette use was prompted by craving 951/1902 (50%) of the time. Smoking events appeared to be briefer than vaping. Conclusion(s): Inciting events and social contexts of tobacco and e-cigarette use were similar. This is likely due to preexisting habits as most participants were concurrent tobacco users. The finding that e-cigarette use is often prompted by craving may relate to its utilization as a smoking cessation aide. There were also significant differences in patterns of use, including the location and length of time when smoking or vaping. The Ethica application was successful in capturing circumstantial trends in tobacco and e-cigarette smoking events, and could be further utilized in larger studies.
3472	S Qanash, S Alemam, E Mahdi, J Softah, A Touman, A Alsulami (2019///) Electronic cigarette among health science students in Saudi Arabia. Annals of Thoracic Medicine; 14<br><br>**Keywords:**<br>adult,article,*college student,comparative effectiveness,controlled study,cross-sectional study,female, flavor,fruit,*health science,human,major clinical study,male,*medical student,prevalence,questionnaire, regression analysis,*Saudi Arabia,smoke,smoking<br><br>**Abstract:** BACKGROUND: Electronic cigarette (E-cigarette) is an electronic nicotine delivery device that has been advocated as a safe alternative for cigarette smokers. Since the introduction of E-cigarette internationally and in the Kingdom of Saudi Arabia (KSA), it gained popularity, particularly among the youth and young adults. Moreover, many nonsmoker (nicotine-naive) youth started to use E-cigarette as a new social habit. Recent researches have casted shadows on the E-cigarette safety profile. OBJECTIVE(S): The aim of this study is to assess the prevalence of E-cigarette use among students of health science colleges in Jeddah-KSA. It also assesses E-cigarette effectiveness as a smoking cessation method and its possible addictiveness. METHOD(S): The study is an epidemiological, cross-sectional study, which was conducted between November 2017 and December 2017 in three different universities in Jeddah. A self-administered online questionnaire related to E-cigarette and the conventional cigarette was used. RESULT(S): A total of 1007 completed an electronic survey which was distributed to 3000 health sciences colleges' students. Of the participants 14.1% were cigarette smoker, 46% of them smoke regularly. Students who smoke half a pack per day and above were 22% of the smokers. More college students use the E-cigarette (27.7%). Moreover, one-fifth of the E-cigarettes users were using it on the regular daily basis. The study found that 42.7% of E-cigarettes users have used it as a tool to quit smoking. Interestingly, more than half (56.7%) of the students who used it to stop smoking has succeeded. However, only 46% of E-cigarettes users who tried to quit vaping have succeeded. Young aged, students believed that smoking is more addictive than vaping or recommended E-cigarette for

	smoking cessation found to have a higher chance of quitting smoking in the univariate regression analysis. While, in the multivariate analysis, students who believed that conventional smoking is more addictive than E-cigarettes; students started vaping to quit smoking, or used E-cigarettes with fruit flavor found to have significantly higher chance of quitting. CONCLUSION(S): The E-cigarettes vaping is more prevalent than conventional cigarette smoking among health sciences students in Jeddah-KSA. E-cigarettes are used as a tool to help smoking cessation in less than half of the user. E-cigarettes help some smokers to quit smoking. However, it seems as addictive to the users as conventional cigarette smoking.Copyright © 2019 Annals of Thoracic Medicine.
3474	H Quintana, F Bajura, R Roa (2018///) Comparison of tobacco use prevalence between Panama nationwide and the Guna Yala Indigenous Territory. Tobacco Induced Diseases; 16<br><br>**Keywords:**<br>adolescent,child,controlled study,electronic cigarette,female,funding,girl,high school,human,major clinical study,male,*Panama,*prevalence,secondary prevention,sex difference,smokeless tobacco, *smoking,statistical significance,conference abstract<br><br>**Abstract:** Aims According the Global Adult Tobacco Survey (GATS), Panama has the lowest prevalence of tobacco products consumption in the Americas (6.4%). The top subnational prevalence of tobacco products use, according to GATS, corresponds to the Guna Yala Indigenous territories (GYIT) (16.2%). In 2017, the Global Youth Tobacco Survey (GYTS) took place in Panama with national and with GYIT representativeness. We aim comparing the national tobacco products consumption with the consumption at GYIT. Methods GYTS was applied to teens aged 13-15 years attending public and private high schools. GYTS follows a standard protocol. The nationwide representative sample included 51 schools, and 2,084 respondents with 83.0% response rate. The GYIT sample included 27 schools, and 1, 083 pupils with 80.3% response rate. Results National current tobacco smoking prevalence was 5.9% (p value for sex: > 0.05), and the GYIT figure was 10.7% (boys: 14.2%; girls: 7.3%, p value for sex: < 0.05). The national current smokeless tobacco consumption prevalence was 2.3% (p value for sex differences: > 0.05), and the GYIT figure was 5.7% (boys: 7.1%; girls: 4.1%, p value for sex differences: < 0.05). National prevalence of current e-cigarettes consumption was 6.4% (p value for sex differences: > 0.05), the figure in GYIT was 5.1% (p value for sex differences: > 0.05). Conclusions GYIT tended to have a higher prevalence of tobacco products consumption compared to the national consumption. The MPOWER elements assessed in the GYTS will guide tobacco control policies to decrease the consumption of tobacco products in the youth. Funding This study is financed by public funds. Some of these public funds come from the Selective Tax on the Consumption of Cigarettes and other Tobacco Products. This tax discourages the use of tobacco products. However, the funds coming from this tax are used in activities to improve the primary and secondary prevention of tobaccoassociated diseases, such as this study.
3483	Ramji Rathi, B Arnetz Bengt, Nilsson Maria, Wiklund Ywonne, Jamil Hikmet, Maziak Wasim, Arnetz Judy (2018///) Waterpipe use in adolescents in Northern Sweden: Association with mental well-being and risk and health behaviours. Scandinavian journal of public health; 46<br><br>**Keywords:**<br>Adolescent,Female,Health Behavior,Humans,Male,Mental Disorders/ep [Epidemiology],Risk-Taking, Surveys and Questionnaires,Sweden/ep [Epidemiology],*Water Pipe Smoking/ep [Epidemiology],Young Adult<br><br>**Abstract:** AIMS: There is a lack of studies examining the association between waterpipe smoking and mental well-being among adolescents. This study sought to determine whether waterpipe smoking is associated with mental well-being and other risk and health behaviours in adolescents., METHODS: A questionnaire was distributed to 1006 adolescents in grades 9-12 (with a response rate of >95%), containing questions on measures of stress, mental energy and sleep. In addition, the questionnaire assessed risk and health behaviours, including use of a waterpipe, cigarettes, e-cigarettes, snus, alcohol,

	narcotics, gambling and exercise. Logistic regression was used to assess factors associated with waterpipe use., RESULTS: Thirty-seven per cent (n=371) of the participants had used a waterpipe at some point. Waterpipe use was associated with lower mental energy (odds ratio [OR] = 0.90, 95% confidence interval [CI] 0.81-0.99), higher stress (OR = 1.10, 95% CI 1.02-1.20) and use of cigarettes (OR = 3.82, 95% CI 2.33-6.03), e-cigarettes (OR = 3.26, 95% CI 2.12-4.99), snus (OR = 2.29, 95% CI 2.12-4.99), alcohol (OR = 1.92, 95% CI 1.07-3.44) and narcotics (OR = 3.64, 95% CI 1.75-7.58). Waterpipe use was not significantly associated with gambling, exercise or sleep quality., CONCLUSIONS: Waterpipe use in adolescents is associated with worse mental well-being, as well as use of other nicotine products, alcohol and narcotics. Prospective studies are needed to delineate causal and temporal relationships further between waterpipe use and mental well-being and its relationship to other risky behaviours in order to design effective prevention programs.
3485	S B Ravara, V Afreixo, M Condinho, M Santos Rua, C Albuquerque, P Aguiar, J Bonito (2018///) Tobacco and E-cigarette use, and exposure to SHS among health sciences students: A national cross-sectional study. European Respiratory Journal; 52<br><br>**Keywords:**<br>adult,cigar,college,controlled study,*cross-sectional study,disease course,*electronic cigarette,female, *health science,hookah,human,major clinical study,male,motivation,*nursing student,Portugal, prevalence,questionnaire,regression analysis,*smoking,con<br><br>**Abstract:** Aim: To assess tobacco and e-cigarette (EC) use, and exposure to SHS among pharmaceutical (PHs), nursing (Ns), medical (MDs), and medical dental (Ds) finalists students. Method(s): In 2016, a national cross-sectional questionnaire-based study involved health science schools in Portugal (46.6% online). A descriptive/inferential and regression analysis was performed. Result(s): Participants: 2095 students, 79.5% females, mode age 23 years, 34.5% collaboration rate. Of the finalists, more than half had experimented tobacco (in descending order: box cigarettes, RYO, shisha, cigarillos, cigars, p<0.001). Experimentation of EC ranged from 9.3% (MD) to 13.4% (Ns). EC occasional consumption was more frequent than daily. Prevalence of tobacco use was: Ns-22.1% (23.5% in males; 21.8% in females, p=0.6); Ds-19.7% (29.7% in males; 15.9% in females, p=0,018); PHs19.4% (32.3% in males, 15.1% in females; p=0, 003); MDs12,2%, (17.1% in males; 10.2% in females, p=0,049), p<0.001. Among all courses, regular smoking overlaps with college admission; most smokers reported low dependence and desire to quit, while 20% reported readiness to quit; motivation to quit was not triggered by being a role model; Nursing students reported significantly higher dual consumption and experimentation of multiple tobacco products, p<0.001. Being female or an MD predicted not using tobacco. Exposure to SHS was rather common in leisure spaces and did not significantly varied among courses. Conclusion(s): Tobacco use, experimentation of e-cigarette, and exposure to SHS are common among health science students, highlighting the need to implement comprehensive tobacco control policies in health science schools.
3487	Auf Rehab, Trepka Mary Jo, Selim Mazen, Taleb Ziyad Ben, Rosa Mario De La, Bastida Elena, Cano Miguel Angel (2019///) E-cigarette use is associated with other tobacco use among US adolescents. International journal of public health; 64<br><br>**Keywords:**<br>Adolescent,*Electronic Nicotine Delivery Systems/sn [Statistics & Numerical Data],Female,Humans, Longitudinal Studies,Male,Odds Ratio,Retrospective Studies,Socioeconomic Factors,*Tobacco Smoking/ep [Epidemiology],*Tobacco, Smokeless/sn [Statistics & Nume<br><br>**Abstract:** OBJECTIVES: To examine whether tobacco initiation via e-cigarettes increases the likelihood of subsequent tobacco use among a large representative sample of US adolescents., METHODS: This study is a retrospective longitudinal analysis from a representative sample of US middle and high school students (n = 39,718) who completed the 2014 and 2015 National Youth Tobacco Survey. The adjusted odds ratios of lifetime and current use of tobacco use were estimated by logistic regression analysis while controlling

	for important socio-ecological factors associated with tobacco use., RESULTS: E-cigarette initiators were more likely to report current use of cigarettes (AOR 2.7; 1.9-4.0, p < 0.001), cigars (AOR 1.7; 1.2-2.4, p < 0.01), or smokeless tobacco (AOR 3.1; 2.2-5.4, p < 0.001), and lifetime use of the same products as well. Also, lifetime and current use of e-cigarettes significantly increased the likelihood of cigarettes, cigars, and smokeless tobacco use., CONCLUSIONS: Initiation of tobacco via e-cigarette, lifetime, and current use of e-cigarettes are associated with higher odds of lifetime and current use of cigarettes, cigars, and smokeless tobacco. Collectively this suggests e-cigarettes may lead to an increased use of tobacco among adolescents.
3488	Auf Rehab, Trepka Mary Jo, Selim Moaz, Taleb Ziyad Ben, Rosa Mario De La, Cano Miguel Angel (2018///) E-cigarette marketing exposure and combustible tobacco use among adolescents in the United States. Addictive Behaviors; 78<br><br>**Keywords:**<br>Adolescent,Advertising as Topic,Cigar Smoking/ep [Epidemiology],*Electronic Nicotine Delivery Systems/sn [Statistics & Numerical Data],Female,Humans,Male,*Marketing/sn [Statistics & Numerical Data],Tobacco Smoking/ep [Epidemiology],*Tobacco Smoking/px [<br><br>**Abstract:** E-cigarette advertising has been shown to be associated with use of e-cigarettes, but its association with tobacco use has not been studied. Therefore, we examined the association between e-cigarettes advertisement and tobacco use. Data from nationally representative 22,007 middle and high school students (grades 6-12) were used to conduct the analysis. Logistic regression models estimated the adjusted odds ratios (AOR) of ever and current use of cigarette, hookah, cigar, and polytobacco use. Odds ratios were weighted and adjusted for study design, non-response rates, school level, gender, race/ethnicity, e-cigarette use, and smoking at home. E-cigarette marketing exposure was significantly associated with ever use of cigarettes (AOR: 1.3, 95% CI: 1.1-1.5), hookah (AOR: 1.4, 95% CI: 1.2-1.7), cigars (AOR: 1.5, 95% CI: 1.4-1.6), and polytobacco (AOR: 1.7, 95% CI: 1.5-1.8). Likewise, E-cigarette marketing exposure was significantly associated with current use of cigarettes (AOR: 1.3, 95% CI: 1.1-1.6), hookah (AOR: 1.3, 95% CI: 1.03-1.7), cigars (AOR: 1.3, 95% CI: 1.1-1.6), and polytobacco use (AOR: 1.8, 95% CI: 1.5-2.1). The results suggest that e-cigarette advertisement is associated with use of cigarettes, hookah, cigars, and polytobacco products. These results add to the evidence about the risks of e-cigarette marketing and highlight the need for stricter regulation of e-cigarette advertisements. Copyright © 2017 Elsevier Ltd. All rights reserved.
3490	D A Rhoades, A L Comiford, J D Dvorak, K Ding, L M Driskill, A M Hopkins, P Spicer, T L Wagener, M P Doescher (2019///) Dual Versus Never Use of E-Cigarettes Among American Indians Who Smoke. American Journal of Preventive Medicine; 57<br><br>**Keywords:**<br>adult,American Indian,article,cohort analysis,controlled study,cross sectional study,demography, depression,enzyme linked immunosorbent assay,female,health care facility,health service,household income,human,male,rural area,self report,*smoking,smoking c<br><br>**Abstract:** Introduction: Many American Indian communities have a high prevalence of smoking and e-cigarette use, but factors associated with their dual use are rarely studied. Method(s): In 2016, a total of 375 American Indian adults who smoke completed paper surveys regarding cigarette and e-cigarette use and provided saliva for cotinine levels. In 2018, cross-sectional analyses were performed, comparing dual users (12%), defined as using e-cigarettes on some or every day for the past 30 days, with never users of e-cigarettes (37%). Result(s): Compared with never users, dual users were younger, more often reported history of depression (56% and 29%, respectively; p<0.01) and family history of smoking-related disease (77% and 59%, respectively; p<0.05), had lower harm perceptions of e-cigarettes (27% and 47%, respectively; p<0.01) or vapor (14% and 35%, respectively; p<0.01), and more often perceived e-cigarettes as cessation aids (75% and 16%, respectively; p<0.01) and as less harmful than cigarettes (70%

	and 17%, respectively; p<0.01). Dual users were less often uncertain/unknowing about e-cigarette benefits or harms (p<0.01) and more often reported likelihood to quit smoking (49% and 24%, respectively; p<0.01) and prior attempt to quit smoking, ever (89% and 67%, respectively; p<0.01) or in the past year (55% and 32%, respectively; p=0.01). Cigarette consumption and cotinine levels did not differ between groups. Dual users more often tried other nicotine products (p<0.02) and more often lived with a vaping partner/spouse (45% and 6%, respectively; p<0.01). Conclusion(s): Dual users perceived e-cigarettes as less harmful than cigarettes and more as cessation aids than cigarette-only users did, but cigarette consumption did not differ between groups. Whether e-cigarettes will reduce smoking-related disparities among American Indian people remains undetermined.Copyright © 2019 American Journal of Preventive Medicine
3491	F Riahi, S Rajkumar, D Yach (2019///) Tobacco smoking and nicotine delivery alternatives: Patterns of product use and perceptions in 13 countries. F1000Research; 8<br><br>**Keywords:**<br>adult,age,aged,article,cigarette smoking,female,gender,human,income group,Lebanon,male,middle aged, motivation,New Zealand,nicotine replacement therapy,*perception,population risk,prevalence,risk factor,*smoking,smoking cessation,substance use,young adul<br><br>**Abstract:** Background: Smoking tobacco products remains a significant public health problem. The Foundation for a Smoke-Free World commissioned a 13-country survey to gain a clearer understanding of the current landscape of smoking behavior and preferences across the world. Method(s): Over 17,000 participants in 13 countries, representing different regions and income groups, answered questions on their smoking patterns and product use, their social context, their motivation to smoke, quit, or switch, and their perception of risks of products and substances. Rim weighting was done for each country to align responses with population demographics, and an additional 200 smokers for each country were surveyed to achieve sufficient sample size for sub-analyses of smoker data. Result(s): The observed prevalence of smoking ranged from an age-adjusted high of 57.5% in Lebanon to 8.4% in New Zealand among men, with lower rates for women. The majority of smokers were between 25-54 years old, had daily routines and social patterns associated with smoking, used boxed cigarettes, and rated their health more poorly compared to never smokers. Among a range of products and substances, smokers tended to give both cigarettes and nicotine the highest harm ratings. Smokers in high income countries were largely familiar with electronic nicotine delivery systems; the most commonly given reasons for using them were to cut down or quit smoking. A majority of smokers had tried to quit at least once, and while many tried without assistance, motivations, intentions, and methods for smoking cessation, including professional help, nicotine replacement therapies or medications, or electronic cigarettes, varied among countries. Conclusion(s): Smoking is deeply integrated in smokers' lives worldwide. Although a majority of smokers have tried to quit, and are concerned for their health, they do not seek help. Smokers lack understanding of the harmful components of smoking tobacco products and the risk profile of alternatives.Copyright © 2019 Riahi F et al.
3493	K E Riehm, A S Young, K A Feder, N Krawczyk, K N Tormohlen, L R Pacek, R Mojtabai, R M Crum (2019///) Mental health problems and initiation of e-cigarette and combustible cigarette use. Pediatrics; 144<br><br>**Keywords:**<br>adolescent,article,child,*cigarette smoking,cohort analysis,correlational study,follow up,human, longitudinal study,*mental health,population risk,priority journal,prospective study,risk factor,trend study,United States,*electronic cigarette<br><br>**Abstract:** BACKGROUND AND OBJECTIVES: During adolescence, mental health problems may increase the risk of initiating combustible cigarette use. However, it is unknown if this association extends to electronic cigarettes (e-cigarettes). We examined whether internalizing and externalizing problems were associated with initiation of e-cigarette, combustible cigarette, and dual-product use among adolescents.

	METHOD(S): Participants were drawn from the Population Assessment of Tobacco and Health Study, a nationally representative longitudinal study of US adolescents followed from 2013 to 2015. The study sample included 7702 adolescents aged 12 to 17 years who at baseline reported no lifetime use of tobacco products. We examined the respective associations between baseline internalizing and externalizing problems and initiating use of e-cigarettes, combustible cigarettes, or both at 1-year follow-up. RESULT(S): Compared with adolescents with low externalizing problems, adolescents with high externalizing problems were significantly more likely to initiate use of e-cigarettes (adjusted relative risk ratio [aRRR] = 2.78; 95% confidence interval [CI]: 1.76-4.40), combustible cigarettes (aRRR = 5.59; 95% CI: 2.63-11.90), and both products (aRRR = 2.23; 95% CI: 1.15-4.31). Adolescents with high internalizing problems were at increased risk of initiating use of e-cigarettes (aRRR = 1.61; 95% CI: 1.12-2.33) but not combustible cigarettes or both products. CONCLUSION(S): Mental health problems are associated with increased risk for initiating e-cigarette, combustible cigarette, and dual-product use in adolescence. This association is more consistent for externalizing problems than internalizing problems. Addressing mental health problems could be a promising target for preventing initiation of nicotine- and/or tobacco-product use by adolescents.Copyright © 2019 by the American Academy of Pediatrics.
3494	N A Rigotti (2018///) Monitoring the rapidly changing landscape of e-cigarettes. Annals of Internal Medicine; 169<br><br>**Keywords:**<br>aerosol,behavioral risk factor surveillance system,*biological monitoring,*cigarette smoking,editorial, health care policy,health hazard,health survey,human,LGBT people,medical information,prevalence, priority journal,public health,sample size,smoking ces<br><br>**Abstract:**
3495	D C Rigsby, S A Keim, A Adesman (2019///) Electronic Vapor Product Usage and Substance Use Risk Behaviors Among U.S. High School Students. Journal of child and adolescent psychopharmacology;<br><br>**Keywords:**<br>adolescent,article,controlled study,dependent variable,drug dependence,female,*high school student, human,juvenile,major clinical study,male,*prevalence,*risk factor,*vaping,*vapor<br><br>**Abstract:** Objectives: The initiation of substance use at a young age increases one's likelihood of developing substance use disorders (SUDs). Understanding trends in youth substance-related risk behaviors is important in identification and prevention of SUDs. The objective of this study was to assess, in a nationally representative sample of U.S. high school students, how current electronic vapor product (EVP), cigarette, and dual-product usage as well as EVP usage frequency are related to other substance use behaviors. Method(s): Respondents (N=12,667) of the 2017 National Youth Risk Behavior Survey were categorized by previous 30-day EVP and cigarette usage into one of four groups: nonuse, cigarette only, EVP only, or dual use. Separately, respondents were categorized by 30-day EVP usage frequency: 0, 1-9, 10-29, or 30 days. Thirteen substance use behaviors were selected as dependent variables. Adjusted prevalence ratios (aPRs) were calculated using multivariable modified Poisson regression to determine associations between outcome behaviors and both current EVP/cigarette usage and EVP usage frequency. Linear contrasts were conducted to compare aPRs across categories. Result(s): Cigarette, EVP, and dual users were more likely to engage in 12 of 13 substance use behaviors compared to nonusers (p<0.001). Dual-users were more likely than EVP-only users to engage in 12 of 13 behaviors. Past 30-day EVP users at any frequency were also more likely to engage in nearly all risk behaviors examined. Occasional EVP users were similarly likely as frequent and daily users to engage in 11 of 13 behaviors. Conclusion(s): Adolescent EVP use, with or without concurrent cigarette smoking, is associated with a higher likelihood of engagement in several substance use behaviors. Prevalence of risk behaviors is generally greater for dual users than EVP-only users, but prevalence proportions do not significantly differ among occasional, frequent, or daily EVP users for most behaviors.

3503	L Robertson, J Hoek, M L Blank, R Richards (2018///) "It Didn't Fill the Void": Reasons for e-cigarette and tobacco dual use. Tobacco Induced Diseases; 16<br><br>**Keywords:**<br>adult,alcohol consumption,clinical article,craving,female,human,interview,male,motivation,mouth, reward,ritual,*smoking cessation,social acceptance,stigma,stress,thematic analysis,*vaping,nicotine, conference abstract<br><br>**Abstract:** Background: Although moving from smoking to electronic nicotine delivery systems (ENDS) could reduce the harms smokers face, many ENDS users continue to smoke tobacco. We explored the contributing factors to tobacco use among ENDS users. Method(s): We conducted in-depth interviews with n=20 past 30- day ENDS and tobacco users (16 used ENDS daily; the median duration of ENDS use was 4.5 months). Interviews explored motivations for using ENDS, experiences of using ENDS and tobacco, and dual use patterns. We used qualitative thematic analysis to analyse interview data. Result(s): Most, but not all, participants used ENDS to try and quit smoking entirely. Among those who reported trying to quit, social exposure to tobacco use and alcohol consumption cued cravings and undermined a full transition to vaping. Some participants found ENDS did not sufficiently alleviate stress or replicate the nicotine hit, mouth-feel or rituals they experienced with smoking. The convenience and perceived social acceptability of using ENDS indoors also facilitated dual use. Several participants cited financial reasons as the primary motivation for using ENDS; they reported alternating between ENDS and smoking as rationing their 'real' cigarettes helped manage the cost of tobacco. Younger, social tobacco smokers reported experiencing stigma when using ENDS, which inhibited their ENDS use in some social settings where tobacco use was normative. While many participants saw dual use as a transition towards becoming smokefree, others viewed their reduced smoking levels alongside ENDS use as a successful outcome. Some perceived ENDS as healthful, not merely less harmful than tobacco; this view appeared to offset dissonance about using tobacco and positioned cigarette smoking as a reward for being "good". Conclusion(s): Understanding factors that facilitate and support dual use of ENDS and smoked tobacco may help inform interventions that support ENDS users to make a full transition from tobacco smoking to vaping.
3505	B Rodu, N Plurphanswat (2018///) E-cigarette use among US adults: Population assessment of tobacco and health (PATH) study. Nicotine and Tobacco Research; 20<br><br>**Keywords:**<br>adult,article,*cigarette smoking,controlled study,health survey,human,malignant neoplasm,outcome assessment,perception,population research,prevalence,priority journal,smoking,structured interview, tobacco use,United States,nicotine,*electronic cigarette<br><br>**Abstract:** Introduction: In 2011 the US Food and Drug Administration launched the Population Assessment of Tobacco and Health (PATH) Study to gather information for regulatory activities authorized by the Family Smoking Prevention and Tobacco Control Act (TCA). Method(s): Data were drawn from the first wave of the PATH survey, involving interviews of 32 320 civilian and non-institutionalized adults. In addition to conventional classifications for current, former and never smoking and e-cigarette use, we used PATH questions to classify former and current triers of these products. Descriptive statistics were used to describe the prevalence, patterns of, and reasons for e-cigarette use, and the perceptions of nicotine and e-cigarettes among user groups, according to smoking status. Result(s): The prevalence of current smoking was 18%; an additional 3.1% of participants were current triers. The prevalence of current e-cigarette use was 2.4% (1.0% every day, 1.4% some days), with another 3.2% as current triers. The majority of nonsmokers who were current e-cigarette users were already current (56%) or former (34%) cigarette triers. Reasons for e-cigarette use were similar across subgroups, but patterns of use were different. Majorities of participants believed that nicotine is the main substance driving tobacco use, that nicotine causes most cancers, and that e-cigarettes were less

	harmful than cigarettes. Conclusion(s): E-cigarettes were used primarily by current smokers and recent former smokers. The main reasons for use center around perceptions that e-cigarettes are less harmful than cigarettes to users and others. Implications: This study reports detailed information about the prevalence, patterns of, and reasons for e-cigarette use in the first (baseline) wave of the PATH Study in 2014. In addition to conventional categories for current, former and never smoking and e-cigarette use, the PATH questionnaire facilitated classification of new usage groups consisting of current and former triers of these products, which may impact prevalence estimates.Copyright © The Author(s) 2017.
3507	K Romijnders, M Beijaert, L Van Osch, H De Vries, R Talhout (2018///) Tobacco cigarette use versus electronic cigarette use: Determinants of smoking and vaping behavior. Tobacco Induced Diseases; 16<br><br>**Keywords:**<br>adult,controlled study,female,human,major clinical study,male,Netherlands,perception,questionnaire, risk assessment,social environment,social status,*vaping,conference abstract<br><br>**Abstract:** Background: It is important to know why individuals use electronic cigarettes (e-cigarettes) compared to tobacco cigarettes. This comparison provides policy makers with opportunities to target different types of users. This study examined behavioral determinants associated with both tobacco and e-cigarette use. Differences between non-users (neither e-cigarette users nor smokers), smokers, e-cigarette users, and dual users were assessed for tobacco use versus e-cigarette use among adults (>18 years). Method(s): A cross-sectional survey (n=726) among 223 nonusers, 287 smokers, and 216 e-cigarette users was conducted in the Netherlands. The questionnaire assessed demographics, tobacco and e-cigarette behavior, knowledge, attitudes, social ties, risk perception, intention, and deliberation. Result(s): Health benefits was the most frequently reported reason for e-cigarette use. Overall, 30.8% of the respondents were nonusers, 39.5% smokers, 29.7% e-cigarette users, and 15.4% dual users. Ever use of e-cigarettes was 29.6 %. Of the ever users, 45.6 % used e-cigarettes daily, 20.9% weekly, and 10.7 % on a monthly basis. A low socio-economic status was associated with ever use of e-cigarettes, and older participants were less likely to have used e-cigarettes. Participants knew more about tobacco cigarettes than e-cigarettes. Furthermore, non-users perceived the social environment to be more negative towards e-cigarette and tobacco use. Additionally, non-users and smokers perceived e-cigarettes as more harmful than e-cigarette users. Smokers were significantly more likely to have an intention to start vaping compared to nonusers. Finally, e-cigarette users made a deliberate choice to vape. Conclusion(s): Determinants of smoking and vaping behavior differ between non-users, smokers, e-cigarette users, and dual users. Using the determinants of behavior assessed, we are able to predict e-cigarette use among non-users and smokers. Policy makers could use these determinants, such as e-cigarette attractiveness, to specifically target dual users, smokers, e-cigarette users or non-users.
3517	R Sharapova Saida, Singh Tushar, T Agaku Israel, M Kennedy Sara, A King Brian (2018///) Patterns of e-cigarette use frequency-National Adult Tobacco Survey, 2012-2014. American Journal of Preventive Medicine; 54<br><br>**Keywords:**<br>*Health Promotion,*Smoking Cessation,*Electronic Cigarettes,Public Health<br><br>**Abstract:** Introduction: Accounting for occasional or intermittent e-cigarette use has yielded higher estimates of current use among U.S. adults. However, frequency of e-cigarette use by population subgroups is not well described. This study assessed e-cigarette use frequency by sociodemographic characteristics and other tobacco product use among U.S. adults. Methods: This study analyzed combined data from the 2012-2013 (n = 60,192) and 2013-2014 (n = 75,233) National Adult Tobacco Survey, a telephone-based survey of U.S. adults aged >= 18 years, to assess frequency of e-cigarette use (daily, some days, rarely) by sex, age, race/ethnicity, education, annual household income, U.S. region, marital status, sexual orientation, and cigarette smoking and other tobacco product use (cigars, cigarillos, little filtered cigars; traditional pipes; water pipes; and chewing tobacco, snuff, dip, snus, and dissolvable

	tobacco products). Analyses took place in 2016. Results: Among current e-cigarette users, 19.3% used daily, 29.3% used some days, and 51.4% used rarely. Daily use was lowest among younger adults, Hispanics, and those who were single, never married, or not living with a partner; and greatest among non-Hispanic Asians (p < 0.0001). Among current e-cigarette users, 25.3% were cigarette-only smokers, 52.8% smoked cigarettes and used other tobacco products, 5.5% used other tobacco products only, 6.5% were former cigarette-only smokers, 6.7% were former users of cigarettes and other tobacco products, 1.3% were former other tobacco product users only, and 1.8% never used cigarettes or other tobacco products. Conclusions: E-cigarette use frequency varies by sociodemographic characteristics and other tobacco use. Further surveillance, particularly related to frequency of e-cigarette use and its impact on cigarette cessation, could inform public health policy, planning, and practice. (PsycINFO Database Record (c) 2018 APA, all rights reserved)
3524	N N Sanford, D J Sher, X Xu, A A Aizer, B A Mahal (2019///) Trends in Smoking and e-Cigarette Use among US Patients with Cancer, 2014-2017. JAMA Oncology; 5<br><br>**Keywords:**<br>adult,age distribution,aged,*cancer patient,cancer risk,clinical practice,cross sectional study,health care policy,health care survey,human,letter,major clinical study,population research,practice guideline, prevalence,sex difference,*smoking,smoking hab<br><br>**Abstract:**
3528	Aleyan Sarah, Cole Adam, Qian Wei, T Leatherdale Scott (2018///) Risky business: a longitudinal study examining cigarette smoking initiation among susceptible and non-susceptible e-cigarette users in Canada. BMJ Open; 8<br><br>**Keywords:**<br>Adolescent,Adolescent Behavior,Alberta/ep [Epidemiology],*Cigarette Smoking/ep [Epidemiology], *Electronic Nicotine Delivery Systems/sn [Statistics & Numerical Data],Female,Humans,Logistic Models, Longitudinal Studies,Male,Ontario/ep [Epidemiology],Risk F<br><br>**Abstract:** OBJECTIVES: Given that many adolescent e-cigarette users are never-smokers, the possibility that e-cigarettes may act as a gateway to future cigarette smoking has been discussed in various studies. Longitudinal data are needed to explore the pathway between e-cigarette and cigarette use, particularly among different risk groups including susceptible and non-susceptible never-smokers. The objective of this study was to examine whether baseline use of e-cigarettes among a sample of never-smoking youth predicted cigarette smoking initiation over a 2-year period., DESIGN: Longitudinal cohort study., SETTING: 89 high schools across Ontario and Alberta, Canada., PARTICIPANTS: A sample of grade 9-11 never-smoking students at baseline (n=9501) who participated in the COMPASS study over 2 years., PRIMARY AND SECONDARY OUTCOME MEASURES: Participants completed in-class questionnaires that assessed smoking susceptibility and smoking initiation., RESULTS: Among the baseline sample of non-susceptible never-smokers, 45.2% of current e-cigarette users reported trying a cigarette after 2 years compared with 13.5% of non-current e-cigarette users. Among the baseline sample of susceptible never-smokers, 62.4% of current e-cigarette users reported trying a cigarette after 2 years compared with 36.1% of non-current e-cigarette users. Overall, current e-cigarette users were more likely to try a cigarette 2 years later. This association was stronger among the sample of non-susceptible never-smokers (AOR=5.28, 95% CI 2.81 to 9.94; p<0.0001) compared with susceptible never-smokers (AOR=2.78, 95% CI 1.84 to 4.20; p<0.0001)., CONCLUSIONS: Findings from this large, longitudinal study support public health concerns that e-cigarette use may contribute to the development of a new population of cigarette smokers. They also support the notion that e-cigarettes are expanding the tobacco market by attracting low-risk youth who would otherwise be unlikely to initiate using cigarettes. Careful consideration will be needed in developing an appropriate regulatory framework that prevents e-cigarette use among youth. Copyright

	© Article author(s) (or their employer(s) unless otherwise stated in the text of the article) 2018. All rights reserved. No commercial use is permitted unless otherwise expressly granted.
3533	Merry Sarah, R Bullen Christopher (2018///) E-cigarette use in New Zealand-a systematic review and narrative synthesis. The New Zealand medical journal; 131<br><br>**Keywords:**<br>Adolescent,Adult,Child,*Electronic Nicotine Delivery Systems/sn [Statistics & Numerical Data],Female, Health Knowledge, Attitudes, Practice,Humans,Male,New Zealand/ep [Epidemiology],Prevalence, *Smoking/ep [Epidemiology],Young Adult<br><br>**Abstract:** AIM: This study aimed to systematically review the literature on e-cigarette use in New Zealand, focusing on prevalence, rationale for use, perceptions and exposure to the devices., METHODS: Six databases were systematically searched for articles regarding e-cigarette use in New Zealand, supplemented with a grey literature search. Seven hundred and eighteen abstracts were identified and full text of 100 articles reviewed. Studies addressing prevalence of and rationale for use, perceptions of and exposure to e-cigarettes were included. Relevant data were synthesised in a narrative summary., RESULTS: Fourteen studies addressed aspects of e-cigarette use in New Zealand, published between 2010 and 2017. Ever-use of e-cigarettes among adults and adolescents has increased, although current use remains low. Smoking strongly predicts use, and ever-use may decrease with age. Investigation of other predictors has been hindered by low prevalence and small samples. While curiosity is commonly cited for sampling e-cigarettes, many smokers are drawn by harm reduction or cessation. More complex motivators are becoming evident. Although exposure to e-cigarettes is common, many remain uncertain about their harm relative to tobacco., CONCLUSION: While the available evidence provides an overview of current use, exposure and acceptance of e-cigarettes in New Zealand, it highlights knowledge deficits and informs future monitoring.
3536	Odani Satomi, S Armour Brian, T Agaku Israel (2018///) Racial/Ethnic Disparities in Tobacco Product Use Among Middle and High School Students - United States, 2014-2017. MMWR. Morbidity and mortality weekly report; 67<br><br>**Keywords:**<br>Adolescent,Child,*Continental Population Groups/sn [Statistics & Numerical Data],*Ethnic Groups/sn [Statistics & Numerical Data],Female,*Health Status Disparities,Health Surveys,Humans,Male, *Students/sn [Statistics & Numerical Data],*Tobacco Use/eh [Eth<br><br>**Abstract:** During the past few decades, wide disparities in tobacco product use have been documented among the largest racial/ethnic groups in the United States (1,2); however, little is known about tobacco product use among youths from racial/ethnic groups other than whites, blacks, and Hispanics. Surveillance reports typically aggregate these racial/ethnic minorities into a single category because of small sample sizes (3). To assess tobacco product use among U.S. middle and high school students from seven racial/ethnic groups (non-Hispanic whites [whites], non-Hispanic blacks [blacks], Hispanics, non-Hispanic Asians [Asians], non-Hispanic American Indian/Alaska natives [AI/ANs], non-Hispanic Native Hawaiians/Other Pacific Islanders [NHOPIs], and non-Hispanic multiracial persons [multiracial]), CDC analyzed pooled data from the 2014-2017 National Youth Tobacco Surveys (NYTS). Prevalence of ever (>=1 time in lifetime) and current (>=1 time in past 30 days) use of seven tobacco products (cigarettes, cigars, smokeless tobacco, electronic cigarettes [e-cigarettes], hookahs, pipes, and bidis) was assessed; any tobacco product use was defined as use of one or more tobacco products, including hand-rolled cigarettes. During 2014-2017, ever-use of any tobacco product among U.S. middle and high school students was as follows: NHOPIs (45.1%), AI/ANs (43.8%), multiracial persons (38.2%), Hispanics (35.1%), blacks (32.3%), whites (32.0%), and Asians (16.3%). Current use of any tobacco product was as follows: NHOPIs (23.4%), AI/ANs (20.6%), multiracial persons (16.5%), whites (15.3%), Hispanics (14.6%), blacks (11.5%), and Asians (5.0%). Among black middle and high school students, cigars were the most common

	product currently used, whereas e-cigarettes were the most commonly used product for all other racial/ethnic groups. Comprehensive and sustained implementation of evidence-based, population-level tobacco control interventions could reduce prevalence and disparities in tobacco product use among U.S. youths.
3539	M D Sawdey, H R Day, B Coleman, L D Gardner, S E Johnson, J Limpert, H T Hammad, M L Goniewicz, D B Abrams, C A Stanton, J L Pearson, A R Kaufman, H L Kimmel, C D Delnevo, W M Compton, M Bansal-Travers, R S Niaura, A Hyland, B K Ambrose (2019///) Associations of risk factors of e-cigarette and cigarette use and susceptibility to use among baseline PATH study youth participants (2013-2014). Addictive Behaviors; 91<br><br>**Keywords:**<br>adolescent,age,anxiety disorder,article,cannabis use,child,*cigarette smoking,depression,exposure, female,high risk population,household,human,major clinical study,male,*risk factor,sensation seeking, tobacco use,*cigarette,*electronic cigarette<br><br>**Abstract:** Introduction: Improved understanding of the distribution of traditional risk factors of cigarette smoking among youth who have ever used or are susceptible to e-cigarettes and cigarettes will inform future longitudinal studies examining transitions in use. Method(s): Multiple logistic regression analysis was conducted using data from youth (ages 12-17 years) who had ever heard of e-cigarettes at baseline of the PATH Study (n = 12,460) to compare the distribution of risk factors for cigarette smoking among seven mutually exclusive groups based on ever cigarette/e-cigarette use and susceptibility status. Result(s): Compared to committed never users, youth susceptible to e-cigarettes, cigarettes, or both had increasing odds of risk factors for cigarette smoking, with those susceptible to both products at highest risk, followed by cigarettes and e-cigarettes. Compared to e-cigarette only users, dual users had higher odds of nearly all risk factors (aOR range = 1.6-6.8) and cigarette only smokers had higher odds of other (non-e-cigarette) tobacco use (aOR range = 1.5-2.3), marijuana use (aOR = 1.9, 95%CI = 1.4-2.5), a high GAIN substance use score (aOR = 1.9, 95%CI = 1.1-3.4), low academic achievement (aOR range = 1.6-3.4), and exposure to smoking (aOR range = 1.8-2.1). No differences were observed for externalizing factors (depression, anxiety, etc.), sensation seeking, or household use of non-cigarette tobacco. Conclusion(s): Among ever cigarette and e-cigarette users, dual users had higher odds of reporting traditional risk factors for smoking, followed by single product cigarette smokers and e-cigarette users. Understanding how e-cigarette and cigarette users differ may inform youth tobacco use prevention efforts and advise future studies assessing probability of progression of cigarette and e-cigarette use.Copyright © 2018
3542	K Schaller, S Kahnert, U Mons (2018///) E-cigarette use in Germany from 2015 to 2017. Tobacco Induced Diseases; 16<br><br>**Keywords:**<br>adolescent,adult,controlled study,*electronic cigarette,ex smoker,female,*Germany,human,juvenile, major clinical study,male,smoking cessation,tobacco dependence,young adult,conference abstract<br><br>**Abstract:** Background: Worldwide sales of e-cigarettes are increasing markedly as well as their use by adults and youths. The utility of e-cigarettes for smoking cessation is a matter of scientific debate. Increasing use among youths however - and especially among nonsmoking adolescents - is a matter of concern, as e-cigarette use is suspected to contribute to nicotine addiction and smoking initiation. Currently, for Germany, data on e-cigarette use are sparse. Method(s): The German Cancer Research Center conducted annual representative cross-sectional surveys from 2015 to 2017, each comprising a sample of n=2000 persons aged 16 years and older. All respondents were asked whether they used e-cigarettes, smokers and former smokers were also asked about cessation methods they used in their last quit attempt. For February 2018, another survey is planned. Result(s): Ever use of e-cigarettes increased from 2015 to 2017; in 2017 8.6% of all respondents had ever used e-cigarettes. Ever use of e-cigarettes was highest in ages 16 - 19. Regular use was generally lower, but also markedly increased from 2015 to

	2017; in 2017 2.5% of all respondents used e-cigarettes at least weekly. Regular use was highest in the age groups 20 - 29 and 50+. More smokers (4.3%) than former (2.8%) or never (1.0%) smokers used e-cigarettes regularly. The percentage of smokers who used e-cigarettes in an attempt to quit smoking decreased from 3.1% in 2015 to 2.5% in 2017, but the percentage of former smokers who said that they successfully quit by using e-cigarettes increased from 0.5% in 2015 to 1.8 % in 2017. Conclusion(s): In Germany, regular e-cigarette use is low, but increasing. E-cigarette experimentation is highest among youths, and regular use is highest among young adults. The percentage of former smokers who successfully stopped smoking by using e-cigarettes as cessation method is still low, but increasing.
3553	A S Selya, J S Rose, L Dierker, D Hedeker, R J Mermelstein (2018///) Evaluating the mutual pathways among electronic cigarette use, conventional smoking and nicotine dependence. Addiction (Abingdon, England); 113<br><br>**Keywords:**<br>adult,article,*cigarette smoking,cohort analysis,controlled study,driver,*electronic cigarette,female, human,Illinois,major clinical study,male,nonsmoker,path analysis,prospective study,smoking reduction, *structural equation modeling,syndrome,*tobacco de<br><br>**Abstract:** BACKGROUND AND AIMS: The implications of the rapid rise in electronic cigarette (e-cigarette) use remain unknown. We examined mutual associations between e-cigarette use, conventional cigarette use and nicotine dependence over time to (1) test the association between e-cigarette use and later conventional smoking (both direct and via nicotine dependence), (2) test the converse associations and (3) determine the strongest pathways predicting each product's use. DESIGN: Data from four annual waves of a prospective cohort study were analyzed. Path analysis modeled the bidirectional, longitudinal relationships between past-month smoking frequency, past-month e-cigarette frequency and nicotine dependence. SETTING: Chicago area, Illinois, USA. PARTICIPANTS: A total of 1007 young adult smokers and non-smokers (ages 19-23 years). MEASUREMENTS: Frequency of (1) cigarettes and (2) e-cigarettes was the number of days in the past 30 on which the product was used. The Nicotine Dependence Syndrome Scale measured nicotine dependence to cigarettes. FINDINGS: E-cigarette use was not associated significantly with later conventional smoking, either directly (beta = 0.021, P = 0.081) or through nicotine dependence (beta = 0.005, P = 0.693). Conventional smoking was associated positively with later e-cigarette use, both directly (beta = 0.118, P < 0.001) and through nicotine dependence (beta = 0.139, P < 0.001). The strongest predictors of each product's use was prior use of the same product; this pathway was strong for conventional cigarettes (beta = 0.604, P < 0.001) but weak for e-cigarettes (beta = 0.120, P < 0.001). Nicotine dependence moderately strongly predicted later conventional smoking (beta = 0.169, P < 0.001), but was a weak predictor of later e-cigarette use (beta = 0.069, P = 0.039). CONCLUSION(S): Nicotine dependence is not a significant mechanism for e-cigarettes' purported effect on heavier future conventional smoking among young adults. Nicotine dependence may be a mechanism for increases in e-cigarette use among heavier conventional smokers, consistent with e-cigarettes as a smoking reduction tool. Overall, conventional smoking and, to a lesser extent, its resulting nicotine dependence, are the strongest drivers or signals of later cigarette and e-cigarette use.Copyright © 2017 Society for the Study of Addiction.
3554	D R Sendoya, E Bianco, M Yan, A Quah, G Fong (2018///) Prevalence of flavored cigarettes and e-cigarettes in Uruguay: Findings from the Wave 5 of the ITC Uruguay survey. Tobacco Induced Diseases; 16<br><br>**Keywords:**<br>adult,awareness,child,city,controlled study,education,*electronic cigarette,human,major clinical study, monitoring,onset age,preschool child,*prevalence,sample size,smoking,*Uruguay,young adult,menthol, conference abstract<br><br>**Abstract:** Background: Uruguay has implemented strong tobacco control policies over the years which has resulted in decreased tobacco consumption. However, there is little information about flavored

	cigarettes and on electronic cigarettes (ECs). EC sales are banned in Uruguay. The purpose of this study is to evaluate consumption of these products. Method(s): Data is from the International Tobacco Control (ITC) Uruguay Wave 5 smokers aged 18 and older in five cities. The sample size was 1,428 smokers. Result(s): The minimum age of onset of smoking was 5 years old and 96% of smokers started smoking before the age of 25. Two out of three (68.6%) smokers chose their cigarette brand because of the taste. 72.5% have tried flavored (menthol) cigarettes, and1.3% currently smoking menthol brands. Younger age (94% aged 18-24) and smokers with higher education (84% high, 74% medium, 61% low) have tried menthol cigarettes associated with. Awareness of ECs was 56% among the smokers, but it is higher among smokers aged 18-24 (62%) and smokers with higher education (81%). Among the smokers who had heard of e-cigarettes. 12.8% had tried once (higher under age 24 (24%) and higher among smokers with high education level (20%)). Among the smokers, 1.3% using EC daily and 1.9% using EC weekly. Conclusion(s): Taste is a very important factor for Uruguayan smokers. Almost all young smokers have tried menthol cigarettes. ECs use is also on the rise, mainly among young peopleand people with higher education. There is a need for monitoring flavored cigarettes and EC consumptionto defining new regulations for them.
3571	F Siddiqui, M Mishu, A M Marshall, K Siddiqi (2019///) E-cigarette use and subsequent smoking in adolescents and young adults: a perspective. Expert Review of Respiratory Medicine; 13<br><br>**Keywords:**<br>adolescent,*adolescent smoking,adult,editorial,health hazard,human,longitudinal study,prevalence, smoking cessation,young adult,cigarette smoke,*electronic cigarette<br><br>**Abstract:**
3579	D M Smith, M Gawron, L Balwicki, A Sobczak, M Matynia, M L Goniewicz (2019///) Exclusive versus dual use of tobacco and electronic cigarettes among adolescents in Poland, 2010-2016. Addictive Behaviors; 90<br><br>**Keywords:**<br>adolescent,*adolescent smoking,adult,article,cigarette smoking,clinical assessment,comparative study, cross-sectional study,female,frequency,health survey,human,male,Poland,rural area,social problem, tobacco dependence,*tobacco use,urban area,*electronic<br><br>**Abstract:** Globally, electronic cigarette (e-cigarette) use among adolescents has increased, along with concerns about potential health implications from exclusive and concurrent tobacco and e-cigarette use (i.e., "dual use"). This study used data from a cross-sectional survey administered to 5708 students aged 15-19 in secondary and technical schools throughout two regions of Poland. Data from 2010-2011 (n = 1760); 2013-2014 (n = 1970) and 2015-2016 (n = 1978) were analyzed to assess changes in use of cigarettes and e-cigarettes from 2010-2016, and correlates of exclusive and dual use, daily use, and intensity of product use from 2013-2016. In this sample, e-cigarette trial and past 30-day exclusive e-cigarette use significantly increased over time (2010-2011: 2%; 2013-2014: 8%; 2015-2016: 11% (p <.05)). Dual use increased from 2010-2011 (4%) to 2013-2014 only (23%, p <.05); and remained stable from 2013-2014 to 2015-2016 (24%, p =.60). Most dual users reported using cigarettes before trying e-cigarettes (82% in 2013-2014, 76% in 2015-2016). Analyses comparing dual users to exclusive users showed significant differences in frequency and intensity of product use, similar findings were observed within dual users. Following the introduction of e-cigarettes, both total tobacco use and total cigarette use increased. Among youth in Poland, e-cigarettes do not seem to replace conventional cigarettes, but instead contribute to a more diverse array of available products from which youth can experiment.Copyright © 2018
3584	S S Sohal, M S Eapen, V G M Naidu, P Sharma (2019///) IQOS exposure impairs human airway cell homeostasis: Direct comparison with traditional cigarette and e-cigarette. ERJ Open Research; 5

	**Keywords:**<br>*airway cell,airway remodeling,airway smooth muscle cell,*cigarette smoking,comparative study, cytotoxicity,epithelial mesenchymal transition,extracellular matrix,global health,glycolysis,HBEC cell line (bronchial epithelium),*homeostasis,human,letter,mi<br><br>**Abstract:**
3592	I Allen Sophia, Foulds Jonathan, Wasserman Emily, Veldheer Susan, Hrabovsky Shari, Yingst Jessica, Liu Guodong (2018///) Tobacco Use Among Middle and High School Students in Pennsylvania. Preventing chronic disease; 15<br><br>**Keywords:**<br>Adolescent,Adolescent Behavior,*Cigarette Smoking/ep [Epidemiology],*Electronic Nicotine Delivery Systems/sn [Statistics & Numerical Data],Female,Health Surveys,Humans,Male,Pennsylvania/ep [Epidemiology],Sex Distribution,Students/sn [Statistics & Numeri<br><br>**Abstract:** We analyzed data from the 2014-2015 Pennsylvania Youth Tobacco Survey to determine prevalence of tobacco use among middle and high school students. For the first time, questions on current (past 30-day) use of electronic cigarettes (e-cigarettes) were included in the survey. For current use, e-cigarettes were the most commonly used tobacco product among middle school students (2.3%; 95% confidence interval [CI], 1.4%-3.2%), and cigarettes were the most commonly used tobacco product among high school students (11.0%; 95% CI, 8.1%-13.8%). Given the changing landscape of tobacco products, collection of comprehensive data on tobacco use, including frequency, is important for monitoring behaviors among adolescents.
3593	E K Soule, A D Plunk, P T Harrell, R B Hayes, K C Edwards (2019///) Longitudinal analysis of associations between reasons for electronic cigarette use and change in smoking status among adults in the Population Assessment of Tobacco and Health Study. Nicotine & tobacco research : official journal of the Society for Research on Nicotine and Tobacco;<br><br>**Keywords:**<br>adult,article,controlled study,*electronic cigarette,*ex smoker,female,flavor,human,juvenile,major clinical study,male,odor,prevalence,prevention,public figure,*relapse,scientist,*smoking cessation, smoking reduction<br><br>**Abstract:** Introduction: Electronic cigarette (ECIG) use and changes in cigarette smoking status may be influenced by self-reported reasons for using ECIGs. Method(s): We analyzed adult current and former cigarette smokers who were also current or former ECIG users at Wave 1 (n=3044) using Wave 1 and Wave 2 Population Assessment of Tobacco and Health Study data (2013-2015). Prevalence of reporting 13 reasons for ECIG use at Wave 1 was examined and weighted logistic regressions were conducted predicting smoking status changes from Wave 1 to Wave 2. Result(s): Reasons for ECIG use ranged from 18.1% (people in the media/public figures use them) to 82.5% (they might be less harmful to people around me than cigarettes). From Wave 1 to Wave 2, 27.2% of former smokers (n=249) became current smokers and 11.6% of current smokers (n=246) became former smokers. Among Wave 1 former smokers, using ECIGs because of the availability of flavors (AOR=0.57, 95% CI=0.39-0.85) or because they don't smell (AOR=0.64, 95% CI=0.42-0.97) was associated with lower odds of relapse to smoking, but using ECIGs because using them helps people quit smoking (AOR=1.55, 95% CI=1.01-2.38) was associated with greater odds of relapse. Among Wave 1 current smokers, using ECIGs because they can be used where smoking is not allowed (AOR=0.56, 95% CI=0.38-0.85) was associated with reduced odds of quitting cigarettes. Conclusion(s): Some reasons for ECIG use are associated with changes in self-reported smoking status. Researchers should examine ECIG user characteristics when assessing associations between ECIG use and smoking status transitions. Implications: Given that certain reasons for ECIG use,

	such as using ECIGs in locations are where smoking is not allowed, may inhibit smoking reduction, policies may be developed to prevent ECIG use in locations where smoking is banned. Additionally, because certain reasons for ECIG use may aid in relapse prevention, such as availability of desired flavors, efforts should be made to identify ECIG device characteristics that are appealing to smokers but not youth or non-tobacco users. These results provide support for future research on reasons for ECIG use to inform regulatory policies.
3594	C A Spears, D M Jones, S R Weaver, J Huang, B Yang, T F Pechacek, M P Eriksen (2019///) Sociodemographic Correlates of Electronic Nicotine Delivery Systems (ENDS) Use in the United States, 2016-2017. American journal of public health; 109<br><br>**Keywords:**<br>adult,article,Black person,education,*electronic cigarette,employment,ethnicity,female,health insurance, human,human experiment,major clinical study,male,poverty,public health,sexual and gender minority, sexual orientation,smoking,*United States,young adu<br><br>**Abstract:** Objectives. To investigate use of electronic nicotine delivery systems (ENDS) among priority populations.Methods. Using 2016 through 2017 US nationally representative surveys (n=11688), we examined ENDS use by sociodemographic variables (age, education, poverty status, insurance, employment, race/ethnicity, sexual orientation) and combustible tobacco use.Results. Among individuals who currently use noncigarette combustible tobacco, those from certain backgrounds (young adults, those living below the poverty level, those less educated, sexual minorities, Blacks, Hispanics, and those without health insurance) were more likely to use ENDS. Among current cigarette smokers, those who were younger, living at or above poverty (ever use), with higher education (current use), sexual minority, and non-Black were more likely to use ENDS.Conclusions. Associations between sociodemographic variables and ENDS use varied depending on combustible tobacco use status, highlighting the need to consider multiple types of tobacco products to understand ENDS use among priority populations. The impact on tobacco disparities will ultimately depend on whether ENDS are used to transition completely away from combustible tobacco products and how this may differ across priority populations who use diverse tobacco products.
3595	C A Spears, D M Jones, S R Weaver, B Yang, T F Pechacek, M P Eriksen (2019///) Electronic nicotine delivery system (ENDS) use in relation to mental health conditions, past-month serious psychological distress and cigarette smoking status, 2017. Addiction (Abingdon, England); 114<br><br>**Keywords:**<br>adult,article,*cigarette smoking,controlled study,diagnosis,disease course,education,*electronic cigarette,ethnicity,*expectation,female,gender,Hispanic,household income,human,leisure,major clinical study,male,*mental health,*mental stress,race,United S<br><br>**Abstract:** BACKGROUND AND AIMS: Adults with mental health conditions (MHC) exhibit disproportionately high smoking prevalence and experience profound tobacco-related disparities. US nationally representative surveys from 2012 to 2015 found relatively high usage of electronic nicotine delivery systems (ENDS; e.g. e-cigarettes) among adults with MHC. However, research has not examined these associations specifically among never smokers. Aims were to examine associations among MHC diagnosis, serious psychological distress (SPD) and ENDS use and to test whether associations varied by cigarette smoking status. DESIGN: Cross-sectional US nationally representative survey. SETTING: United States, 2017. PARTICIPANTS: A total of 5762 adults (52.0% female; 64.8% non-Hispanic white, 11.4% non-Hispanic black, 15.9% Hispanic, 7.9% non-Hispanic other). MEASUREMENTS: Outcomes were lifetime, current and current daily ENDS use. Predictors were lifetime MHC, past-month SPD and cigarette smoking status, and covariates were gender, age, race/ethnicity, education and annual household income. FINDINGS: lifetime MHC and past-month SPD were each associated with higher likelihood of having ever used ENDS (P <= 0.001), currently using ENDS (P <= 0.001) and currently using ENDS daily (P < 0.05).

	There were interactions between MHC and smoking status in predicting ENDS use, such that MHC status predicted higher lifetime and current ENDS use specifically among never and current smokers. Never smokers with MHC had 2.62 higher odds [95% confidence interval, (CI) = 1.54, 4.45] of current ENDS use than those without MHC. Among never smokers, those with MHC indicated higher expectations that ENDS would improve relaxation and concentration (P < 0.05). CONCLUSION(S): In 2017, US adults with versus without mental health conditions (MHC) were more likely to use electronic nicotine delivery systems (ENDS). In particular, both never and current smokers with MHC reported disproportionately high rates of current ENDS use.Copyright © 2018 Society for the Study of Addiction.
3599	S Steigerwald, P O Wong, B E Cohen, J H Ishida, M Vali, E Madden, S Keyhani (2018///) Smoking, vaping, and use of edibles and other forms of marijuana among U.S. adults. Annals of Internal Medicine; 169<br><br>**Keywords:**<br>adult,aged,ancestry group,*cannabis addiction/ep [Epidemiology],*cannabis smoking,*cannabis use, controlled study,drug formulation,drug legislation,female,health survey,human,letter,major clinical study,male,middle aged,prevalence,priority journal,tobacc<br><br>**Abstract:**
3600	J S Tucker, W G Shadel, D Golinelli, R Seelam, D Siconolfi (2019///) Correlates of cigarette and alternative tobacco product use among young tobacco users experiencing homelessness. Addictive Behaviors; 95<br><br>**Keywords:**<br>adolescent,adult,article,California,chewing tobacco,*cigarette smoking,correlational study,cost effectiveness analysis,depression,disease severity,female,health hazard,*homelessness,human,human experiment,male,normal human,probability sample,snus,*tobac<br><br>**Abstract:** Introduction: Most young people experiencing homelessness smoke cigarettes, but little is known about use of alternative tobacco products (ATPs) such as e-cigarettes or other electronic nicotine delivery systems (ENDS) and little cigars/cigarillos (LCCs). This study examines past month use and correlates of cigarettes and ATP among young tobacco users experiencing homelessness. Method(s): We surveyed a probability sample of N = 469 unaccompanied homeless 13-25 year olds (mean age = 22; 71% male), who reported past month use of any type of tobacco product, from 25 service and street sites in Los Angeles County. Result(s): Nearly all (90%) participants reported smoking regular cigarettes, and 78% reported using at least one tobacco product other than regular cigarettes. The most commonly used of these other products was natural cigarettes (55%), followed by LCCs (43%), ENDS (34%), cigars (31%), hookah (14%), chewing tobacco (7%), and snus (5%). Multivariable models indicated that correlates of past month use differed by product, but included sociodemographic characteristics, homelessness severity, depression, exposure to other people who used the product, and product perceptions (e.g., relative access, cost, and harm compared to cigarettes). Conclusion(s): Use of cigarettes and ATPs are both widespread among young homeless tobacco users, suggesting that efforts to reduce tobacco use in this population should have a broad focus that includes a variety of products. The effectiveness of these efforts may be enhanced by addressing their considerable exposure to other tobacco users, as well as their perceptions of certain products as being less harmful or more cost-effective options than regular cigarettes.Copyright © 2019
3603	R Baldassarri Stephen, A Fiellin David, Savage Mary Ellen, M Madden Lynn, Beitel Mark, K Dhingra Lara, Fucito Lisa, Camenga Deepa, Bollampally Pooja, T Barry Declan (2019///) Electronic cigarette and tobacco use in individuals entering methadone or buprenorphine treatment. Drug and Alcohol Dependence; 197<br><br>**Keywords:**<br>Adult,Buprenorphine/tu [Therapeutic Use],*Electronic Nicotine Delivery Systems/sn [Statistics & Numerical Data],European Continental Ancestry Group,Female,Humans,Male,Methadone/tu

	[Therapeutic Use],Middle Aged,Odds Ratio,Opiate Substitution Treatment/px<br><br>**Abstract:** BACKGROUND: Although smoking is prevalent among populations with opioid use disorder (OUD), few studies have examined electronic cigarette (EC) use in individuals seeking opioid agonist therapy (OAT). The aim of this study was to evaluate the prevalence and correlates of EC use among individuals seeking OAT., METHODS: 782 patients seeking OAT for OUD completed surveys assessing current and past EC use, reasons for use, current and past cigarette smoking, nicotine dependence, psychiatric distress, trauma, and pain. Bivariate and multivariate models evaluated correlates of daily EC use, past-30-day EC use, and current cigarette smoking., RESULTS: 6% of patients reported daily EC use, 18% reported past-30-day use, 62% reported EC use history, and 85% reported current cigarette smoking. 46% reported using ECs to quit or cut down smoking. In multivariate analyses, daily EC use was associated with higher odds of being a former smoker (OR 21; CI 1.7-273) and lower odds of ever smoking more than 100 cigarettes (OR 0.07; CI 0.01-0.32), while EC use in the past 30 days was associated with lower odds of being Caucasian (OR 0.55; CI 0.34-0.89), ever smoking more than 100 cigarettes (OR 0.13; CI 0.02-0.67), and history of chronic pain (OR 0.59; CI 0.38-0.90), and higher odds of reporting psychiatric distress (OR 1.5; CI 1.1-2.2)., CONCLUSIONS: EC use is common among people with OUD who smoke cigarettes. Those with daily use had higher odds of being former smokers than current smokers. Interventions using ECs may be effective to help reduce harms and mortality in OUD. Copyright © 2019. Published by Elsevier B.V.
3613	W Flint Stuart, W Jones Arwel (2018///) The irresponsible promotion of e-cigarettes and Swaptober. The Lancet. Respiratory medicine; 6<br><br>**Keywords:**<br>*Electronic Nicotine Delivery Systems,England,*Health Promotion/es [Ethics],Health Promotion/mt [Methods],Humans,*Smoking/px [Psychology],*Smoking Cessation/px [Psychology],*Social Responsibility,State Medicine<br><br>**Abstract:**
3614	Krishnan-Sarin Suchitra, Jackson Asti, Morean Meghan, Kong Grace, W Bold Krysten, R Camenga Deepa, A Cavallo Dana, Simon Patricia, Wu Ran (2019///) E-cigarette devices used by high-school youth. Drug and Alcohol Dependence; 194<br><br>**Keywords:**<br>Adolescent,*Electronic Nicotine Delivery Systems,Female,Humans,Male,Schools,Smoking Water Pipes, *Students,Surveys and Questionnaires,*Vaping<br><br>**Abstract:** Background E-cigarette use rates are high among youth, but there is limited information on the types of e-cigarette devices that are used by youth. Methods During Spring 2017, students from 4 high schools completed surveys on use of e-cigarette devices (cig-a-like, vape/hookah pen, modified devices or mods, and JUUL). Among youth who endorsed ever (lifetime) use of an e-cigarette and of at least one device (n = 875), we assessed 1) prevalence rates of ever and current (past-month) use of each device, 2) use of nicotine in each device, and 3) predictors [age, sex, race, socioeconomic status (SES), other tobacco use] of ever use of each device and of use of single versus multiple devices. Results Cig-a-likes were used least frequently (Ever use: cig-a-likes: 25.4%; vape/hookah pens: 60.6%; JUUL: 64.2%; mods: 71.2%; Current use: cig-a-likes: 7.3%; vape/hookah pens; 18.7%; mods: 33.1%; JUUL: 47.1%;). Nicotine use was highest for JUUL (JUUL: 80.3%; mods: 56.3%; cig-a-likes: 51.4%; vape/hookah pens: 46.8%). Among ever users of single devices, use of JUUL was highest (JUUL: 43%; mods: 32%; vape/hookah pens: 21%; cig-a-likes: 4%). Ever use of all devices, except JUUL, was associated with other tobacco product use. Ever use of JUUL was associated with higher SES. Ever use of multiple devices (two: 34.7%; three: 25.8%; four: 11.7%) compared with a single device (27.8%) was associated with other tobacco product use. Conclusions Targeted regulatory and prevention efforts that consider the use of multiple e-cigarette

	devices are needed to lower youth e-cigarette use rates. Copyright © 2018 Elsevier B.V. All rights reserved.
3619	Azagba Sunday (2018///) E-cigarette use, dual use of e-cigarettes and tobacco cigarettes, and frequency of cannabis use among high school students. Addictive Behaviors; 79<br><br>**Keywords:**<br>Adolescent,Canada/ep [Epidemiology],*Cigarette Smoking/ep [Epidemiology],Comorbidity,Female, Humans,Logistic Models,Male,*Marijuana Use/ep [Epidemiology],Odds Ratio,Students/sn [Statistics & Numerical Data],*Vaping/ep [Epidemiology]<br><br>**Abstract:** BACKGROUND: The proliferation of electronic vaping products raises many concerns, including whether these products will lead to risky behaviors among adolescents. Evidence suggests that e-cigarettes may be used to vaporize cannabis (marijuana). The current study examined associations between e-cigarette use, dual use of e-cigarettes and tobacco cigarettes, and frequency of cannabis use., METHODS: Data on high school students (grades 9-12) were from the 2014-2015 Canadian Student Tobacco, Alcohol and Drugs Survey (n=23,429 respondents). Multinomial logistic regression analyses were used to examine frequency of cannabis use among those who reported using only e-cigarettes, only tobacco cigarettes, both products, and nonusers., RESULTS: Approximately 15% of respondents reported using cannabis at least once in the past month. Likelihood of using cannabis was significantly higher for e-cigarette only and cigarette- only users. Users of both products had increased odds for a higher frequency of cannabis use. These results indicated a sequential risk gradient in the use of cannabis for dual use, cigarette only, e-cigarette only, and non-use groups, respectively., CONCLUSIONS: Youth who reported use of e-cigarettes, tobacco cigarettes, and both products showed a heightened risk of using cannabis more frequently. Copyright © 2017 Elsevier Ltd. All rights reserved.
3620	Azagba Sunday, Wolfson Mark (2018///) E-cigarette use and quantity of cigarette smoking among adolescent cigarette smokers: A finite mixture model analysis. Drug and Alcohol Dependence; 185<br><br>**Keywords:**<br>Adolescent,Canada,*Cigarette Smoking,Cross-Sectional Studies,*Electronic Nicotine Delivery Systems/sn [Statistics & Numerical Data],Female,Humans,Male,Models, Theoretical,*Smokers<br><br>**Abstract:** BACKGROUND: E-cigarette use is popular among adolescents and youth, but its long-term public health implications remain largely unknown. Much of the literature has focused on understanding the relationship between e-cigarette use and youth cigarette initiation. However, very little is known about e-cigarette use and cigarette quantity among those who continue to smoke cigarettes. The objective of the present study was to examine the association between current e-cigarette use and quantity of cigarette smoking., METHODS: Cross-sectional data on current smokers were drawn from the 2014-2015 Canadian Student Tobacco, Alcohol and Drugs Survey among high school students (n=1411). A finite mixture model (FMM) was employed to account for unobserved heterogeneity due to clusters of finite sub-populations., RESULTS: Current e-cigarette users reported smoking more conventional cigarettes in the past week compared to non-e-cigarette users (t [1409]=4.7998; p<0.001 in unadjusted analysis). Results from a finite mixture regression showed that current e-cigarette use was significantly associated with the number of cigarettes smoked in the past week, but only among light smokers (IRR=1.40; CI=1.05-1.85). However, additional analyses found that the association between e-cigarette use and quantity of cigarette smoked varied by individual smoking pattern. An FMM with a group or class modelling using individual smoking pattern showed a weaker association between e-cigarette use and quantity of cigarette smoking., CONCLUSION: Findings of this study suggest that the significant association between e-cigarette use and quantity of cigarette smoking may be driven by patterns of use among experimental or beginner smokers. Copyright © 2018 Elsevier B.V. All rights reserved.

3621	H Y Sung, Y Wang, T Yao, J Lightwood, W Max (2018///) Polytobacco use and nicotine dependence symptoms among US adults, 2012-2014. Nicotine and Tobacco Research; 20<br><br>**Keywords:**<br>adult,age distribution,aged,article,Caucasian,cross-sectional study,demography,educational status, female,household income,human,major clinical study,male,middle aged,prevalence,priority journal,sex ratio,smokeless tobacco,*tobacco dependence,*tobacco us<br><br>**Abstract:** Introduction The tobacco product landscape has changed substantially. Little is known about the recent pattern of polytobacco use (at least two tobacco products) among US adults and its relationship to nicotine dependence. Methods Using the 2012-2013 and 2013-2014 National Adult Tobacco Survey (NATS) data (N = 135 425 adults), we analyzed the prevalence and correlates of polytobacco use among each of the six categories of current tobacco user (cigarettes, cigars, pipes, hookah, e-cigarettes, and smokeless tobacco). Based on five nicotine dependence symptom measures from the NATS, difference in the prevalence of dependence symptoms between polytobacco and sole-product users for each category of tobacco user was assessed using multivariable regression analyses. Results During 2012-2014, 25.1% of adults were current users of any tobacco product. Among them, 32.5% were poly users with the largest poly use category being dual use of cigarettes and e-cigarettes (30.2%). Poly use prevalence was the lowest among current cigarette smokers (38.7%), followed by current users of smokeless tobacco (52.4%), hookah (59.2%), cigars (69.3%), e-cigarettes (80.9%), and pipes (86.2%). Among each category of current tobacco user, the prevalence of dependence symptom was consistently greater in polytobacco users than sole users for every symptom measure. After controlling for frequency of use and demographic covariates, the difference in nicotine dependence between poly users and sole users was statistically significant and consistent across all symptom measures for each category of tobacco user. Conclusions Between 52% and 86% of noncigarette tobacco users and nearly 40% of cigarette smokers engaged in polytobacco use. Poly users showed greater nicotine dependence than sole-product tobacco users. Implications This study examines recent patterns of polytobacco use separately for US adult current cigarette smokers, cigar smokers, pipe smokers, hookah users, e-cigarette users, and smokeless tobacco users. By including more tobacco products, particularly e-cigarettes and hookah, this study provides more comprehensive insight into polytobacco use. This study is also unique in comparing nicotine dependence between polytobacco and sole-product users among each category of tobacco users. Our results indicate that polytobacco use is very common and is associated with greater likelihood of reporting nicotine dependence symptoms. Tobacco cessation policies and programs should be tailored to address polytobacco use.Copyright © The Author(s) 2018. Published by Oxford University Press on behalf of the Society for Research on Nicotine and Tobacco.
3626	Yoong Sze Lin, Stockings Emily, Chai Li Kheng, Tzelepis Flora, Wiggers John, Oldmeadow Christopher, Paul Christine, Peruga Armando, Kingsland Melanie, Attia John, Wolfenden Luke (2018///) Prevalence of electronic nicotine delivery systems (ENDS) use among youth globally: a systematic review and meta-analysis of country level data. Australian and New Zealand journal of public health; 42<br><br>**Keywords:**<br>Adolescent,Child,*Electronic Nicotine Delivery Systems/sn [Statistics & Numerical Data],*Global Health/sn [Statistics & Numerical Data],Humans,Prevalence,*Smoking/ep [Epidemiology],Young Adult<br><br>**Abstract:** OBJECTIVE: To describe the prevalence and change in prevalence of electronic nicotine delivery systems (ENDS) use in youth by country and combustible smoking status., METHODS: Databases and the grey literature were systematically searched to December 2015. Studies describing the prevalence of ENDS use in the general population aged <=20 years in a defined geographical region were included. Where multiple estimates were available within countries, prevalence estimates of ENDS use were pooled for each country separately., RESULTS: Data from 27 publications (36 surveys) from 13 countries were included. The prevalence of ENDS ever use in 2013-2015 among youth were highest in Poland

	(62.1%; 95%CI: 59.9-64.2%), and lowest in Italy (5.9%; 95%CI: 3.3-9.2%). Among non-smoking youth, the prevalence of ENDS ever use in 2013-2015 varied, ranging from 4.2% (95%CI: 3.8-4.6%) in the US to 14.0% in New Zealand (95%CI: 12.7-15.4%). The prevalence of ENDS ever use among current tobacco smoking youth was the highest in Canada (71.9%, 95%CI: 70.9-72.8%) and lowest in Italy (29.9%, 95%CI: 18.5-42.5%). Between 2008 and 2015, ENDS ever use among youth increased in Poland, Korea, New Zealand and the US; decreased in Italy and Canada; and remained stable in the UK., CONCLUSIONS: There is considerable heterogeneity in ENDS use among youth globally across countries and also between current smokers and non-smokers. Implications for public health: Population-level survey data on ENDS use is needed to inform public health policy and messaging globally. Copyright © 2018 The Authors.
3627	A Szklo, C Perez, T Cavalcante, L Almeida, L Craig, S Kaai, A Quah, G Fong (2018///) Increase of electronic cigarette use and awareness in Brazil: Findings from a country that has strict regulatory requirements for electronic cigarette sales, import, and advertising. Tobacco Induced Diseases; 16<br><br>**Keywords:**<br>adult,*advertising,*awareness,*Brazil,Brazilian,controlled study,data correlation,disease course, *electronic cigarette,female,human,major clinical study,male,nonsmoker,prevalence,sampling,smoking, uncertainty,nicotine,conference abstract<br><br>**Abstract:** Background: Because of the uncertainty about the health impact of Electronic Cigarettes (EC), Brazil established strict regulatory requirements in 2009 that prohibit the sale, import,and advertising of them until a manufacturer can demonstrate that the devices are safe and/or effective as cessation aid.This paper examined self-reported:(1) EC awareness and ever-use;(2) correlates of awareness and ever-use. Method(s): Data were analyzed from the 2012/2013 (n=1,830) and 2016/2017 (n=1,828) waves of the International Tobacco Control Survey in Brazil.We calculated the differences over time in the proportion of individuals who have ever heard about ECs, and have ever tried an EC, stratified by smoking status.All estimates were adjusted for sampling weights and time in sample, taking also into consideration intraindividual correlation of data from participants observed at both waves. Moreover, a log-Binomial regression model was used to estimate the association between sociodemographic covariates and EC awareness (and ever-use). Result(s): The proportion of EC awareness increased over time for both smokers (37.5% to 71.9%) and non-smokers (28.8% to 61.6%).The proportion of EC ever-use also increased over time for both smokers (7.8% to 18.3%) and non-smokers (1.1% to 2.2%). However, among EC users interviewed in 2016/2017 (n=218), the proportion of recent/regular EC use was very low (daily use, 2.5%; weekly use, 0.6%, monthly use, 2.6%; less than monthly, 20.7%; not all, 73.6%).Irrespective of the smoking status,EC awareness and ever-use were associated with younger ages, having higher income, and being bettereducated. Conclusion(s): Despite strict EC regulatory requirements in Brazil (i.e.,EC are de facto banned),this was not sufficient to prevent EC awareness and consumption among Brazilians.Our findings may help in the discussion about what can happen to Brazil's strong reduction in the prevalence of ordinary cigarette smokers achieved in the last few years if the current EC regulatory regime is changed to allow smokers/non-smokers to have legal access to non-combusted sources of nicotine.
3628	Williams Tahlia, White Victoria (2018///) What Factors are Associated with Electronic Cigarette, Shisha-Tobacco and Conventional Cigarette Use? Findings from a Cross-Sectional Survey of Australian Adolescents?. Substance use & misuse; 53<br><br>**Keywords:**<br>Adolescent,Australia,Child,*Cigarette Smoking/px [Psychology],Cross-Sectional Studies,Electronic Nicotine Delivery Systems,Female,Humans,Male,*Smoking,*Smoking Water Pipes,Students,*Vaping/px [Psychology]<br><br>**Abstract:** BACKGROUND: Adolescents' use of electronic cigarettes and shisha-tobacco increased in several countries during the 2000s, including in the United States, United Kingdom and Canada. Few

	studies have examined the factors associated with exclusive e-cigarette or shisha-tobacco use and whether adolescents using these substances exclusively differ from those using traditional cigarettes., OBJECTIVES: To examine the socio-demographic and behavioural correlates of exclusive e-cigarette and exclusive shisha-tobacco use to those found for users of tobacco cigarettes and multiple nicotine products in Australian adolescents., METHODS: Cross-sectional survey of smoking behaviours of a random sample of 4,576 students in the Australian state of Victoria aged 12-17 conducted between June 2014 and December 2014., RESULTS: Overall, 14% of students had used an e-cigarette with 3% using e-cigarettes exclusively. 13% had used shisha-tobacco, with 2% using shisha-tobacco exclusively. Most students (65%) using e-cigarette and shisha-tobacco (67%) had also used tobacco cigarettes. After adjusting for demographic factors, students using e-cigarettes only were more likely to have never used cannabis or drink alcohol in the past year compared to tobacco cigarette users. Compared to tobacco cigarette users, students using only shisha-tobacco were younger, less likely to use cannabis or alcohol or have friends or parents who smoke., CONCLUSIONS: Most Australian adolescents who use alternative nicotine and tobacco-related products do so in conjunction with tobacco cigarettes. Students using e-cigarettes or shisha-tobacco exclusively were less likely to use other substances. Australian longitudinal studies are needed to determine whether this group of students are on a pathway to traditional tobacco use.
3631	E Jenson Tara (2018///) Psychosocial and Behavioral Risk Profiles of Cigarette Smokers and E-Cigarette Users Among Adolescents in Minnesota: The 2016 Minnesota Student Survey. Preventing chronic disease; 15<br><br>**Keywords:**<br>Adolescent,Adolescent Behavior,Alcohol Drinking/ep [Epidemiology],*Cigarette Smoking/ep [Epidemiology],Cigarette Smoking/px [Psychology],Cross-Sectional Studies,*Electronic Nicotine Delivery Systems/sn [Statistics & Numerical Data],Female,Health Surveys<br><br>**Abstract:** INTRODUCTION: Understanding differences in predictors of adolescent cigarette smoking and e-cigarette use can inform public health strategies for preventing and reducing tobacco use among this population. The objective of this study was to examine the association of socioeconomic, psychosocial, and behavioral factors with cigarette smoking and e-cigarette use among adolescents in Minnesota., METHODS: Records (n = 126,868) were used from the 2016 Minnesota Student Survey for prevalence of and factors associated with cigarette smoking and e-cigarette use among students in grades 8, 9, and 11. Logistic regression models were used to estimate risk for smoking cigarettes, using e-cigarettes, or concurrent use of both for key independent variables., RESULTS: American Indian students were 3.6 times as likely to report smoking cigarettes (OR = 3.57; 95% CI, 3.04-4.19), and 1.7 times as likely to report using e-cigarettes (OR = 1.72; 95% CI, 1.47-2.01) as non-Hispanic white students. Bisexual students were 4 times as likely (adjusted odds ratio [AOR] = 4.40; 95% confidence interval [CI], 4.01-4.82) as heterosexual students to smoke cigarettes and twice as likely (AOR = 2.24; 95% CI, 2.06-2.43) to use e-cigarettes. Students receiving free/reduced lunch were nearly twice as likely (AOR = 1.92; 95% CI, 1.80-2.05) to smoke cigarettes and 1.3 times as likely (AOR = 1.33; 95% CI, 1.27-1.39) to use e-cigarettes. Increasing alcohol use and decreasing academic performance were associated with increasing likelihood of cigarette smoking and e-cigarette use, more so with cigarette smoking., CONCLUSION: Results expand on existing research that show differences in psychosocial and behavioral risk factors between adolescent cigarette smokers and adolescent e-cigarette users.
3632	W Wang Teresa, Asman Kat, S Gentzke Andrea, A Cullen Karen, Holder-Hayes Enver, Reyes-Guzman Carolyn, Jamal Ahmed, Neff Linda, A King Brian (2018///) Tobacco Product Use Among Adults - United States, 2017. MMWR. Morbidity and mortality weekly report; 67<br><br>**Keywords:**<br>Adolescent,Adult,Aged,Female,Health Surveys,Humans,Male,Middle Aged,Socioeconomic Factors, *Tobacco Products/sn [Statistics & Numerical Data],*Tobacco Use Disorder/ep [Epidemiology],Tobacco

	Use Disorder/eh [Ethnology],United States/ep [Epidemiology],Youn<br><br>**Abstract:** Cigarette smoking harms nearly every organ of the body and causes adverse health consequences, including heart disease, stroke, and multiple types of cancer (1). Although cigarette smoking among U.S. adults has declined considerably, tobacco products have evolved in recent years to include various combustible, noncombustible, and electronic products (1,2). To assess recent national estimates of tobacco product use among U.S. adults aged >=18 years, CDC, the Food and Drug Administration (FDA), and the National Institutes of Health's National Cancer Institute analyzed data from the 2017 National Health Interview Survey (NHIS). In 2017, an estimated 47.4 million U.S. adults (19.3%) currently used any tobacco product, including cigarettes (14.0%; 34.3 million); cigars, cigarillos, or filtered little cigars (3.8%; 9.3 million); electronic cigarettes (e-cigarettes) (2.8%; 6.9 million); smokeless tobacco (2.1%; 5.1 million); and pipes, water pipes, or hookahs (1.0%; 2.6 million). Among current tobacco product users, 86.7% (41.1 million) smoked combustible tobacco products, and 19.0% (9.0 million) used >=2 tobacco products. By univariate analyses, the prevalence of current use of any tobacco product was higher among males than among females; adults aged <65 years than among those aged >=65 years; non-Hispanic American Indian/Alaska Natives, whites, blacks, or multiracial adults than among Hispanics or non-Hispanic Asians; adults who lived in the South or Midwest than among those in the West or Northeast; adults who had a general educational development certificate (GED) than among those with other levels of education; adults who earned an annual household income of <,000 than among those with those with higher income; lesbian, gay, or bisexual adults than among heterosexual/straight adults; and adults who were divorced/separated/widowed or single/never married/not living with a partner than among those who were married/living with a partner. Prevalence was also higher among those who were uninsured, insured by Medicaid, or had some other public insurance than among those with private insurance or Medicare only; those who had a disability/limitation than among those who did not; and those who had serious psychological distress than among those who did not. Full implementation of evidence-based tobacco control interventions that address the diversity of tobacco products used by U.S. adults, in coordination with regulation of tobacco product manufacturing, marketing, and sales, can reduce tobacco-related disease and death in the United States (1-3).
3635	Oncology The Lancet (2018///) E-cigarettes-new product, old tricks. The Lancet Oncology; 19<br><br>**Keywords:**<br>editorial,health care policy,health promotion,human,priority journal,public health problem,*smoking, smoking cessation,social marketing,tobacco dependence,tobacco industry,nicotine,*electronic cigarette<br><br>**Abstract:**
3637	L Caputi Theodore (2018///) Sex and orientation identity matter in the substance use behaviors of sexual minority adolescents in the United States. Drug and Alcohol Dependence; 187<br><br>**Keywords:**<br>Adolescent,*Adolescent Behavior/px [Psychology],*Bisexuality/px [Psychology],Female, Heterosexuality/px [Psychology],Homosexuality, Female/px [Psychology],Humans,Male,Risk-Taking, *Sexual Behavior/px [Psychology],Sexual and Gender Minorities/px [Psycholog<br><br>**Abstract:** BACKGROUND: Health sciences researchers are beginning to understand the differing experiences and health risks among sexual minority subgroups (i.e., those who describe themselves as homosexual/gay/lesbian, bisexual, or unsure/questioning). Such research can promote the allocation of resources to high-risk groups and the development of interventions tailored to their needs. The present study extends this line of research to substance use among adolescents., METHODS: The lifetime and/or past 30-day alcohol, tobacco, cigarette, e-cigarette, marijuana, prescription drug, and illicit drug use of sexual minority and heterosexual adolescents was analyzed using data from the 2015 National Youth Risk Behavior Survey. Controlling for confounders, separate logistic regression models were fit for each

	substance use outcome. A simulation-based strategy was employed to report adjusted risk ratios for each substance use outcome for each sexual minority subgroup., RESULTS: Sexual minority females, particularly bisexual females, were at an elevated risk for substance use. For example, compared to heterosexual females, sexual minority females were 1.35 (95%CI 1.16-1.56) times more likely to have used a substance in the past 30days, and bisexual females had an even further elevated risk ratio (RR: 1.48, 95%CI 1.28-1.69)., CONCLUSIONS: Studying the variance among sexual minority subgroups will help practitioners, advocates, and policymakers identify high risk subgroups. In the case of substance use, this study suggests sexual minority females, particularly bisexual females, should become a target population for prevention and other interventions. The study conducts post-hoc analyses on secondary data, and so these results should be verified in more targeted studies. Copyright © 2018. Published by Elsevier B.V.
3652	K F Trivers, E Phillips, A S Gentzke, M A Tynan, L J Neff (2018///) Prevalence of Cannabis Use in Electronic Cigarettes among US Youth. JAMA Pediatrics; 172<br><br>**Keywords:**<br>*cannabis use,health hazard,high school student,human,letter,middle school student,*prevalence, priority journal,race,smokeless tobacco,social acceptance,tobacco,United States,*cannabis,cigar, *electronic cigarette,hookah,tube<br><br>**Abstract:**
3654	L K Tully, J B Correa, N Doran (2019///) The relationship between family history of tobacco use and progression to tobacco use among young adult e-cigarette users. Preventive Medicine Reports; 15<br><br>**Keywords:**<br>age,article,Caucasian,cigarette smoking,*demography,ethnicity,gender,human,priority journal,race, student<br><br>**Abstract:** Family history of tobacco use and use of e-cigarettes have both been associated with increased tobacco use among young adults. However, to our knowledge, these variables have not been considered simultaneously. This study was designed to evaluate the hypothesis that family history of tobacco use would predict tobacco product use over time among young adult e-cigarette users with limited combustible tobacco use experience. A sample of 124 participants were recruited online from across the United States, completed five surveys over 12 months, and reported use of nicotine and tobacco products over the previous two weeks at each survey. Family history of nicotine and tobacco use was reported at the 6-month assessment, and data were collected in 2017 and 2018. Regression models indicated that family history was associated with likelihood of cigarette use (odds ratio = 1.02 [95% confidence interval = [1.00, 1.04]). Significant interactions between family history and linear (OR = 1.05 [1.01, 1.10]) and quadratic (OR = 0.99 (0.98, 1.00)] time terms indicated that the association between family history and likelihood of combustible product use shifted over time. In both cases, more extensive family histories predicted greater use frequency, and follow-up analyses showed that more extensive family history was associated with greater combustible tobacco product use 3, 6, and 9 months following baseline. Family history of nicotine/tobacco use may promote initiation of tobacco use among e-cigarette users. These results suggest family history of tobacco use may comprise a risk factor for combustible tobacco use within the evolving tobacco product landscape.Copyright © 2019
3659	R Urman, R McConnell, J B Unger, T B Cruz, J M Samet, K Berhane, J L Barrington-Trimis (2018///) Electronic Cigarette and Cigarette Social Environments and Ever Use of Each Product: A Prospective Study of Young Adults in Southern California. Nicotine & tobacco research : official journal of the Society for Research on Nicotine and Tobacco;<br><br>**Keywords:**<br>adolescent,adult,article,*California,child,child health,*cigarette smoking,controlled study,*electronic

	cigarette,female,follow up,friend,human,juvenile,major clinical study,male,*prospective study,social acceptance,*social environment,young adult<br><br>**Abstract:** Background: A supportive youth cigarette social environment, e.g. friends' approval of use, leads to cigarette use initiation, and cigarette users develop a more supportive social environment. Whether there is a bidirectional relationship of e-cigarette social environment with e-cigarette use has not been studied. Method(s): Prospective data were collected from 1441 Children's Health Study participants in 2014 (median age=17.3 years) and at follow-up 1.5 years later. Associations were examined of (1) supportive e-cigarette social environment with subsequent e-cigarette use initiation and (2) of baseline e-cigarette use with supportive e-cigarette social environment at follow-up (among those with a non-supportive baseline social environment). Result(s): Participants with 3-4 friends using e-cigarettes at baseline (vs. no friends) had an odds ratio (OR) of 4.08 of subsequent initiation (95% confidence interval (CI): 1.96,8.49); those with best friends who would have a very friendly (vs. unfriendly) reaction to e-cigarette use had OR 2.54 of initiation (95%CI: 1.57,4.10); and those with someone in the home using e-cigarettes had OR 1.94 of initiation (95%CI: 1.19,3.15). Participants who had ever used e-cigarettes at baseline developed a supportive social environment at follow-up (OR 2.06 of having any friends who used e-cigarettes (95%CI: 1.29, 3.30) and OR 2.33 of having friends who were friendly toward use (95%CI: 1.32, 4.11). Similar bidirectional associations were observed between ever cigarette use and a supportive cigarette social environment. Conclusion(s): The bidirectional relationship between a supportive e-cigarette social environment and ever use of e-cigarettes was similar to that previously observed between cigarette social environment and cigarette use. Implications: Disrupting the social acceptability of youth e-cigarette use merits consideration as a strategy for preventing initiation of e-cigarette use, just as the social de-normalization of cigarette use has proven to be effective in preventing cigarette initiation.
3660	M Urrutia-Pereira, C Sanchez Aranda, P Polles, H Badellino, V Acosta, M Medina Avalos, C Fernandez, J C Sisul-Alvariza, R Castro-Almarales, M Gonzales Leon, P Nunes Rinelli, D Sole (2018///) Smoking in adolescents in Latin America-A sad reality. Allergy: European Journal of Allergy and Clinical Immunology; 73<br><br>**Keywords:**<br>adolescent,*adolescent smoking,adult,*Brazil,California,child,city,controlled study,Cuba,electronic cigarette,female,friend,Hispanic,hookah,human,major clinical study,male,Mexico,Paraguay,prevalence, prospective study,questionnaire,smoking cessation,toba<br><br>**Abstract:** Background: Smoking in Latino America (LA) is very common in adults. Despite the anti-smoking prevention programs, many teens start smoking at school age. We evaluated the prevalence of smoking in adolescents living in LA. Method(s): A prospective study conducted in adolescents (N = 5847; 12-19 years; mean = 14.5 years), enrolled in municipal schools in 8 cities from 5 countries (Argentine, Brazil, Cuba, Mexico, Paraguay) in LA, who answered a self-administered questionnaire on smoking (California Tobacco Survey*) Results: Although 75.3% of adolescents in Latin America are told by their parents not to smoke, 39.5% have had tobacco experimentation (ever tried a cigarette, even one or two puffs), and 22.7% smoked at least once in the last month. 36.3% of those have had tobacco experimentation started smoking before 12 years of age. Teens reported having ease in getting cigarettes (66.7%), although 92.6% believe that smoking at least one cigarette/day is harmful to their health, 43.8% have a smoker friend and 27.3% would smoke a cigarette if it were given to them, 26.1% believe they are more respected because they smoke, and 30.7% believe that smokers have more friends, 49.4% have tried to quit smoking, 32.2% stated that they would smoke if there was a less toxic cigarette, 63.5% knew the electronic cigarette, 57.1% know about hookah. 45.0% are prohibited from smoking at home and 25.3% have no restriction on smoking at home. Conclusion(s): The prevalence of smoking among adolescents in LA is high. The implementation of measures to reduce/stop tobacco use and its new forms of consumption, such as electronic cigarettes and hookah in schools are urgent and imperative.

3661	M Urrutia-Pereira, D Sole, H J Chong Neto, H Badellino, V Acosta, R L Castro-Almarales, M G Leon, M M Avalos, C C Fernandez, J C Sisul-Alvariza, V J Oliano, P N Rinelli (2019///) Youth tobacco use in Latin America: What is the real extent of the problem?. Allergologia et Immunopathologia; 47<br><br>**Keywords:**<br>adolescent,article,child,cigarette smoking,female,health hazard,health survey,human,male,prospective study,risk factor,smoking cessation,*South and Central America,tobacco consumption,*tobacco use, water pipe smoking,electronic cigarette<br><br>**Abstract:** Introduction: Cigarette consumption among teenagers is one of the most critical health-related risk behaviors. Method(s): Prospective study carried out in seven sites of five Latin American countries (Argentina [Cordoba, N = 958, Corrientes, N = 1013], Brazil [Curitiba, N = 650; Uruguaiana, N = 997], Cuba [Havana, N = 1004], Mexico [Veracruz, N = 991] and Paraguay [Ciudad del Este, N = 868]) with public-school adolescents (aged 12-19 years). Respondents were asked to answer the California Student Tobacco Survey. Result(s): 6550 adolescents took part in the survey (average age: 14 years). 38.5% (N = 2517) "tried smoking" and 37.5% started smoking before the age of 12. Sixty-one percent of adolescents think that cigarettes are easily accessible; 41.7% considered that smokers have more friends; 88% indicated knowledge of the harms of smoking one to five cigarettes per day; 58.9% would smoke new cigarette types with less harmful substances; 27.8% have already used e-cigarettes; 28% have smoked hookah. Fifty-seven point five percent have been, in the past seven days, in the same room with someone who was smoking a cigarette; and 30.5% indicated that there were not any no-smoking rules inside their homes. Identifiable risk factors were (logistic regression analysis): smoking cigarettes offered by friends, smoking cigarettes with less harmful substances, knowing what a hookah is, being in the same room with a smoker in the past week. Identifiable protective factors against tobacco use were: knowing the health risks caused by smoking hookah and to have their own room. Conclusion(s): Youth tobacco use in Latin America is a major public health concern, and tobacco control measures are highly needed.Copyright © 2018
3664	S Veldheer, J Yingst, V Midya, B Hummer, C Lester, N Krebs, S Hrabovsky, A Wilhelm, J Liao, M S Yen, C Cobb, T Eissenberg, J Foulds (2019///) Pulmonary and other health effects of electronic cigarette use among adult smokers participating in a randomized controlled smoking reduction trial. Addictive Behaviors; 91<br><br>**Keywords:**<br>adult,aged,article,blood pressure,controlled study,diastolic blood pressure,expired air,female,health status,human,*lung function,male,outcome assessment,population exposure,pulse rate,randomized controlled trial,*smoking,*smoking reduction,carbon monox<br><br>**Abstract:** Background: There is limited evidence about the effects of dual electronic cigarette (e-cig) and combustible cigarette use on lung health or other health outcomes. Studies that have evaluated these outcomes have not included estimates of e-cig or cigarette exposure in the analyses. Material(s) and Method(s): Data analyzed were from 263 smokers participating in a randomized controlled trial designed to encourage participants to reduce their combustible cigarette use by substituting with an e-cig or a non-electronic cigarette substitute (cig-sub). t-tests were used to evaluate changes from baseline at 1 month and 3 months in lung function, blood pressure, pulse, exhaled carbon monoxide, and weight. Linear mixed effects models were used to test associations between health outcomes and study product group, including exposure to the study products (e-cig and cig-sub times used and days used in the past 7 days) and cigarettes per day (CPD). Result(s): There were few significant differences between the groups for lung function indices at any time point in the unadjusted analyses. There were significant reductions in diastolic blood pressure and pulse at 1 month in the unadjusted analyses for those in the e-cig group compared to the cig-sub group. CPD decreased significantly more for the e-cig group than for the cig-sub group at both time points. There were no significant associations between any measured health

	outcomes and group in the linear mixed effects models. Conclusion(s): E-cig use did not contribute to significant changes in health outcome markers as compared with use of a non-electronic cig-sub.Copyright © 2018 Elsevier Ltd
3665	P Veliz, A Eisman, S E McCabe, R Evans-Polce, V V McCabe, C J Boyd (2019///) E-Cigarette Use, Polytobacco Use, and Longitudinal Changes in Tobacco and Substance Use Disorder Symptoms Among U.S. Adolescents. The Journal of adolescent health : official publication of the Society for Adolescent Medicine;<br><br>**Keywords:**<br>adolescent,article,child,*cigarette smoking,controlled study,drug combination,*electronic cigarette, female,follow up,human,human tissue,juvenile,major clinical study,male,school child,*tobacco dependence,nicotine<br><br>**Abstract:** PURPOSE: The objective of this study was to examine the combinations of e-cigarette use, cigarette use, and other tobacco use over time and the relationship these longitudinal use patterns have with symptoms of tobacco use disorder (TUD) and substance use disorders (SUDs) among a sample of adolescents. METHOD(S): Data from U.S. adolescents (aged 12-17 years) who were surveyed for the Population Assessment of Tobacco and Health Study at baseline, first follow-up, and second follow-up (2013-2014, 2014-2015, and 2015-2016; n = 7,595) was used to analyze symptoms of TUD and SUDs based on longitudinal combinations of tobacco/nicotine use. RESULT(S): The most common combination of tobacco/nicotine use across the three waves was "no use of any tobacco/nicotine products" at baseline and first follow-up to "e-cigarette use only" at the second follow-up. Multivariable analyses found that past 30-day cigarette use and other tobacco use at the most recent follow-up was associated with an increase in both current TUD and SUD symptoms, whereas past 30-day e-cigarette use at the most recent follow-up was modestly associated with an increase in current SUD symptoms. CONCLUSION(S): Individuals who transitioned to e-cigarette use were at relatively low risk for increased TUD and SUD symptoms. However, individuals who transitioned or continually used cigarettes were typically at greater risk for indicating more TUD and SUD symptoms. Given the low risk of e-cigarette only users to indicate TUD and SUD symptoms, prevention efforts need to be made to target these youth before they transition to cigarettes and other types of tobacco use.Copyright © 2019 Society for Adolescent Health and Medicine. Published by Elsevier Inc. All rights reserved.
3666	P T Veliz, R Evans-Polce, S E McCabe, V V McCabe, C J Boyd (2019///) Longitudinal changes in US adolescents' e-cigarette use and tobacco use disorder symptoms. Journal of Addiction Medicine; 13<br><br>**Keywords:**<br>adolescent,child,cigar,cigarette smoking,controlled study,drug combination,*electronic cigarette,female, follow up,health behavior,hookah,human,human tissue,juvenile,major clinical study,male,smokeless tobacco,*tobacco dependence,nicotine,conference abst<br><br>**Abstract:** Introduction: There is public concern that e-cigarettes may de-stigmatize or ''re-normalize'' cigarettes. In addition, there are worries that ecigarettes/ products (eg, JUUL, vaporizers) will increase the health risks associated with combustible cigarettes among non-nicotine users. Nicotine is a highly addictive drug, and when youth engage in dual use (ie, ecigarette and cigarette use) they get even higher levels of nicotine than with simply using only 1 tobacco product (eg, only cigarettes). Unlike our knowledge of cigarette use among youth and its relationship to other social and health behaviors, more needs to be known about if and how e-cigarettes are used in combination with other tobacco products and the short- and long-term consequences of youth electronic cigarette/product use (ecigarette use). Accordingly, the purpose of this study is to examine the combinations of e-cigarette use, cigarette use, and other tobacco use (eg, cigar, pipe, hookah, smokeless tobacco) behaviors over-time and the relationship these longitudinal tobacco use behaviors have with symptoms of tobacco use disorders (TUD) and substance use disorders (SUD) among a sample of adolescents. Method(s): Data from US

	adolescents (ages 12-17) who were surveyed for the Population Assessment of Tobacco and Health study (PATH) at baseline and first follow-up (2013-2014/2014-2015; n=13,651) were used for the analysis. Changes in past-year symptoms of TUD and SUD were assessed as a function of different longitudinal combinations of tobacco use behaviors (ie, e-cigarette, cigarette, and 'other tobacco' use) between baseline and first follow-up, 2 years later. Result(s): The most common combination of tobacco use behaviors across the 2-year period was no use of any tobacco/nicotine products at baseline (wave 1) to other tobacco use at first follow-up (wave 2). Adolescents who used all 3 types of productse-cigarette, cigarette, and other tobacco useat both waves had the highest mean symptom count at wave 2 for TUD (M=3.36, SE=0.233) and SUD (M=1.56, SE=0.158). Adolescents who transitioned from no nicotine/tobacco product use at baseline to using all 3 types of products had the largest increase in symptoms for both TUD (M=2.32, SE=0.228) and SUD (M=1.22, SE=0.225) across the 2 waves of the study. Conclusion(s): The study provides needed epidemiological information on the longitudinal combinations of e-cigarette use and other nicotine/tobacco use (eg, cigarette smoking and other tobacco use), and their risk for the development of symptoms of TUD and SUD. Individuals who transitioned to ecigarette use only were at relatively low risk for increased TUD and SUD symptoms. However, the co-occurrence of using e-cigarettes with other nicotine/tobacco products was associated with more symptoms of TUD and SUD. The findings have clinical implications for screening and timing interventions, especially among youth.
3668	M V Versella, A M Borges, C Lin, T M Leyro (2019///) Co-use of Electronic Nicotine Delivery Systems and Combustible Cigarettes, and Their Association with Internalizing Pathology and Vulnerabilities. Cognitive Therapy and Research; 43<br><br>**Keywords:**<br>adult,anxiety,anxiety assessment,Anxiety Sensitivity Index,article,cigarette smoking,depression, Depression Anxiety Stress Scale,distress syndrome,*emotionality,female,human,major clinical study, male,*mental disease,*smoking,vaping,*nicotine,*electronic<br><br>**Abstract:** Nicotine use and psychological distress exert negative bidirectional effects on one another, and are impacted by shared vulnerabilities. Little work has examined the extent to which these relations differ between adult electronic nicotine delivery system (ENDs) users with varied combustible cigarette use histories. The current study examined differences in internalizing symptoms and vulnerabilities between adult dual and single ENDs users with and without a history of combustible cigarette use. Single ENDs users without combustible use histories reported significantly greater stress and anxiety symptoms than single ENDs users with combustible use histories. Single ENDs users without combustible use histories reported greater anxiety and difficulty regulating their emotions than dual-users. Dual- and single users with prior combustible use histories did not differ in internalizing pathology or vulnerability presentations. This suggests that pathology and vulnerability presentation among nicotine users are influenced by both current and past nicotine use history.Copyright © 2018, Springer Science+Business Media, LLC, part of Springer Nature.
3679	E A Vogel, J J Prochaska, D E Ramo, J Andres, M L Rubinstein (2019///) Adolescents' E-Cigarette Use: Increases in Frequency, Dependence, and Nicotine Exposure Over 12 Months. Journal of Adolescent Health; 64<br><br>**Keywords:**<br>adolescent,*adolescent health,article,*cigarette smoking,exposure,female,follow up,human,longitudinal study,male,priority journal,saliva analysis,*tobacco dependence,cotinine,*nicotine,*electronic cigarette<br><br>**Abstract:** Purpose: This study examined changes in e-cigarette and dual-use frequency, levels of nicotine exposure and e-cigarette dependence, and device and e-liquid preferences over 12 months. Method(s): Adolescents (N = 173, aged 13-18 years) who reported past-month e-cigarette use and at least 10 lifetime uses were recruited from the San Francisco Bay Area. The sample was 75.1% male, 54.9% non-Hispanic

	White, mean age 16.6 years (standard deviation = 1.2); 26.6% reported past-month cigarette smoking at baseline (i.e., dual use). At baseline, 6-month, and 12-month follow-up, participants provided saliva samples for cotinine testing and self-reported e-cigarette use frequency, dependence, past-month smoking, product preference, and flavor preference. Result(s): Most (80.3%) were still using e-cigarettes at 12 months, and daily use increased from 14.5% to 29.8%. Model testing indicated an overall increase from baseline to 12 months in frequency of e-cigarette use (F(2, 166) = 5.69, p = .004), dependence (F(2, 164) = 5.49, p = .005), and cotinine levels (F(2, 103) = 4.40, p = .038). Among those reporting only e-cigarette use at baseline, 28.8% reported combustible cigarette use during follow-up. Among those reporting dual use at baseline, 57.1% were still dual using at 12 months, 31.4% reported e-cigarette use only, and none abstained from both products. Higher nicotine delivering e-cigarette devices (i.e., Juul, mods) became more popular over time, whereas flavor preferences (i.e., fruit, mint/menthol, and candy) remained stable. Conclusion(s): Adolescents' e-cigarette use persisted over a 12-month period with significant increases in frequency of use, nicotine exposure, and e-cigarette dependence. Transitions from single to dual and dual to single nicotine product use were observed in approximately one in three users over the study period.Copyright © 2019 Society for Adolescent Health and Medicine
3691	S E Wan Puteh, R A Manap, T M Hassan, I S Ahmad, I B Idris, F M Sham, A B Yu Lin, C I Soo, R M Pakri Mohamed, A I Mokhtar, H Zakaria, J Lee, A S Amer Nordin, S Ariaratnam, M Z Mohd Yusoff (2018///) The use of e-cigarettes among university students in Malaysia. Tobacco Induced Diseases; 16<br><br>**Keywords:**<br>adult,article,cigarette smoking,clinical feature,controlled study,coughing/co [Complication],cross-sectional study,demography,dizziness/co [Complication],female,headache/co [Complication],human, Malaysia,male,priority journal,self concept,semi structured<br><br>**Abstract:** INTRODUCTION E-cigarette use is an emerging phenomenon with increasing recognition and acceptance globally. This study aims to create a profile of e-cigarette users among university students in Malaysia. METHODS The study was conducted using a cross-sectional research involving six universities in Malaysia. A semi-structured questionnaire was distributed to 1302 randomly selected students, who either smoked cigarettes and/or e-cigarettes. The 2011 version of Global Adult Tobacco Surveys (GATS) tool was used to record the respondents' sociodemographic data. RESULTS The study revealed that 74.9% of the respondents smoked e-cigarettes; 40.3% used both cigarettes and e-cigarettes (dual users), and 34.5% were exclusive e-cigarette users. The exclusive use of e-cigarettes was related to gender (OR=0.18, 95% CI: 0.09-0.39). Also, male respondents were the majority users (95%). Of the respondents, 75.2 % were Malays, 98.0% single and most believed they have no health problems (92.1%). Further findings revealed the occurrence of adverse effects, dizziness 14.4%, cough 14.1%, and headaches 12.4%. Overall, 57.8% of the respondents used e-cigarettes as a smoking cessation tool, while others consider e-cigarettes a self-image enhancing tool or as part of social activities. CONCLUSIONS Further research on the use of e-cigarettes should be conducted on a large number of respondents in other settings to augment the findings of this study, and also guide policy making on and prevention practice of e-cigarette use, among the general student population in Malaysia.Copyright © 2018 EMH Swiss Medical Publishers Ltd. All rights reserved.
3695	X Wang, X Zhang, X Xu, Y Gao (2019///) Perceptions and use of electronic cigarettes among young adults in China. Tobacco Induced Diseases; 17<br><br>**Keywords:**<br>adult,article,*attitude to health,China,correlational study,economic status,female,gift giving,health care policy,health economics,health status,human,human experiment,male,mobile application,motivation, normal human,online system,personal experience,pri<br><br>**Abstract:** INTRODUCTION Little is known about the perception and use of e-cigarettes by the Chinese, particularly the young people. This study reveals the awareness, attitudes, and use of e-cigarettes among

	young adults in China, examines the relationship between smoking behavior and e-cigarette perception and use, and demonstrates the phenomenon of e-cigarette gifting. METHODS We used results from a mobile app-based survey conducted in November 2015 that included 10477 young Chinese adults aged between 19 and 29 years. Bivariate tests were conducted to analyze perception and use of e-cigarettes by respondents of different smoking status. Multivariate logistic regressions were applied to examine the correlates of e-cigarette use and perception and e-cigarette gifting behavior, particularly the factors of tobacco smoking status and quitting behavior. RESULTS Among the surveyed young adults, 88.40% were aware of e-cigarettes, and nearly a quarter of all respondents had used e-cigarettes by the time of our survey. Multivariate regression results demonstrated that current smokers with quitting experience were more likely to be aware of and to use e-cigarettes than current smokers with no quitting experience. Smokers with quitting experience also were more inclined to promote e-cigarettes to others by either recommending them or giving them as gifts. CONCLUSIONS E-cigarettes have gained popularity among young adults in China and smokers, especially those who had tried quitting, were more likely to have known and used e-cigarettes. More empirical research on the relationship between e-cigarette use and smoking cessation is warranted to better inform a potential regulatory framework in China.Copyright © 2019 Wang X.
3712	O Wynne, B Bonevski (2018///) Developments in the research base on reducing exposure to second-hand smoke. International Journal of Environmental Research and Public Health; 15<br><br>**Keywords:**<br>asthma,bacterial infection,cannabis use,cardiovascular disease,editorial,human,ischemic heart disease, law,lung cancer,malignant neoplasm,middle ear disease,*passive smoking,population research,*practice guideline,pregnant woman,premature labor,prevalenc<br><br>**Abstract:**
3715	Li Xiaoyin, Loukas Alexandra, L Perry Cheryl (2018///) Very light smoking and alternative tobacco use among college students. Addictive Behaviors; 81<br><br>**Keywords:**<br>Adolescent,Adult,*Cigar Smoking/ep [Epidemiology],*Cigarette Smoking/ep [Epidemiology],Female, Humans,Logistic Models,Male,Multilevel Analysis,Prevalence,*Students/sn [Statistics & Numerical Data], Tobacco Products,Universities,*Vaping/ep [Epidemiology],*<br><br>**Abstract:** INTRODUCTION: Concurrent use of cigarettes with alternative tobacco products (ATPs), even among very light smokers, may be harmful. This study examined current use of e-cigarettes, cigars, and hookah, and susceptibility to future use of these products in a sample of college student cigarette smokers., METHODS: Participants were 1161 18-29year old (M age=21.15; SD=2.72; 52.7% female; 41.2% non-Hispanic white) current, or past 30-day cigarette smokers, drawn from a larger study. Current smokers were categorized as very light smokers [<=5 cigarettes per day (cpd)] and heavier smokers (>5cpd)., RESULTS: 88.6% of all participating college student smokers were very light smokers and 67.7% used at least one ATP concurrently. The prevalence of current use in this sample was 42.9% for e-cigarettes, 36.4% for hookah, and 25.9% for cigars. Compared to heavier smokers, very light smokers were more likely to be younger, racial/ethnic minorities, and four-year versus two-year college students. Multilevel logistic regression models showed that after controlling for socio-demographic characteristics and substance use, being a very light smoker, compared with a heavier smoker, was negatively associated with concurrent e-cigarette use, but positively associated with concurrent cigar use, and not associated with concurrent hookah use. Moreover, compared to heavier smokers, very light smokers reported being more susceptible to future cigar and hookah use, but not e-cigarette use., CONCLUSIONS: Concurrent use of cigarettes with ATPs is popular among all college student smokers, but very light smokers are more likely than heavier smokers to use combustible ATPs. Smoking intervention programs and campus policies

	should caution smokers, especially very light smokers, against ATP use. Copyright © 2018 Elsevier Ltd. All rights reserved.
3716	Chen Xinguang, Yu Bin, Chen Ding-Geng (2018///) Probabilistic Discrete Event Systems Modeling of Nonlinear Transitions between Electronic and Combustible Cigarette Smoking with the 2014 National Youth Tobacco Survey Data. Nonlinear dynamics, psychology, and life sciences; 22<br><br>**Keywords:**<br>Adolescent,*Adolescent Behavior,*Cigarette Smoking,Cross-Sectional Studies,*Electronic Nicotine Delivery Systems,Female,Humans,Male,Risk-Taking,Smoking,Students/sn [Statistics & Numerical Data], Surveys and Questionnaires,Tobacco Products,United States<br><br>**Abstract:** A growing number of young people smoke electronic (e-) cigarettes, presenting an emerging challenge to tobacco control. Analyses are needed to better understand the dynamics of mutual transitions between e-cigarette and conventional combustible (c-) cigarette smoking to advance evidence-based tobacco control. In this study we modeled the nonlinear dynamics of c- and e-cigarette smoking behavior transitions using the 2014 National Youth Tobacco Survey (NYTS) data. A model with five Markov-Chain transition stages linked by 21 transitional paths/probabilities was constructed, guided by the probabilistic discrete event systems (PDES) theory. The Moore-Penrose generalized inverse matrix was used to solve the proposed model that was under-determined. The estimated transitional probabilities indicate that each year, 7-8% of middle school students and 11% of high school students initiated smoking. E-cigarettes were more attractive then c-cigarettes but the behavior of smoking c-cigarettes was more stable than that of smoking e-cigarettes. Female students were more likely to remain as quitters or ex-smokers while male students were more likely to relapse and smoke again. Findings of this nonlinear dynamics modelling indicate potential increases in both e- and c-cigarette smoking among U.S. middle and high school students if no further measures are taken. The PDES-based approach is effective to investigate the nonlinear dynamics of mutual transitions of two risk behaviors in adolescents with one wave cross-sectional data.
3721	Lee Yeji, Lee Kang-Sook (2019///) Factors Related to Smoking Status Among Young Adults: An Analysis of Younger and Older Young Adults in Korea. Journal of preventive medicine and public health = Yebang Uihakhoe chi; 52<br><br>**Keywords:**<br>Adolescent,Adult,Age Factors,Female,Humans,Male,Nutrition Surveys,Prevalence,Republic of Korea/ep [Epidemiology],Risk Factors,*Smoking/ep [Epidemiology],Socioeconomic Factors,Young Adult<br><br>**Abstract:** OBJECTIVES: Young adulthood represents a critical developmental period during which the use of tobacco may begin or cease. Furthermore, differences in smoking behaviors between younger (aged 18-24 years) and older (aged 25-34 years) young adults may exist. This study aimed to characterize patterns related to current smoking in younger and older young adults., METHODS: This study used data acquired from the Sixth Korea National Health and Nutrition Examination Survey conducted from 2013 to 2014. A total of 2069 subjects were categorized as younger (712 subjects) and older (1357 subjects) young adults. The chi-square test was used to assess the relationships between smoking status and socio-demographic, health-related, and smoking-related factors. Multivariable logistic regression models were constructed to assess the factors affecting current smoking in these age groups., RESULTS: The current smoking prevalence was 18.3% among the younger young adults and 26.0% among the older young adults. Sex, education level, occupation, perceived health status, alcohol consumption, and electronic cigarette use were related to current smoking in both age groups. Secondhand smoke exposure at home and stress levels showed significant relationships with smoking in younger and older young adults, respectively., CONCLUSIONS: Strong correlations were found between the observed variables and smoking behaviors among young adults. Determining the factors affecting smoking and designing interventions based on these factors are essential for smoking cessation in young adults.

3724	E O Yildirim, S Duru, T Sevim, F Topcu, B Gemicioglu, I Ozmen, B Ocakli, E Tuncay, H Altinoz, Y K Apak, Y Varol, G Polat, S Ozkan, N Dursunoglu, D Sakarya, P Y Gulhan, O E Diken, P D Cetinkaya, A Yorgancioglu, N T Selcuk, F Yildiz, T Atacag, Y Niksarlioglu, E Uzaslan, N Kokturk (2018///) Electronic smoking in parents in pregnancy; A multicenter cross-sectional study. European Respiratory Journal; 52<br><br>**Keywords:**<br>adult,child health,controlled study,*cross-sectional study,female,gynecology,habit,housewife,human, infant,interview,lung,major clinical study,morbidity,mortality,multicenter study,obstetrician,outpatient, *pregnancy,pregnant woman,primary school,question<br><br>**Abstract:** Background and Aim: Smoking in pregnancy is a preventable risk factor affecting morbidity and mortality.A multicentre cross-sectional study was planned by "Turkish Thoracic Society Women and Lung Health As a task force group ". The aim of the study is to determine the attitudes of pregnant women and their surroundings to electronic cigarettes, which have become increasingly prevalent in recent years. Method(s): The study was conducted in 11 provinces and 15 centers in Turkey, between September 2017 and December 2017. A Questionnaires were conducted with face-toface interviews to gynecology outpatients who agreed to participate in the study. There were a total of 60 questions regarding education, socioeconomic status and parents' habits of smoking cigarettes and electronic cigarettes. Result(s): A total of 1123 pregnant women with a mean age of 27 +/- 5.6 were enrolled. 48% of them were were primary school graduates, 7% were not illiterate, and 56% were housewives. 10% of the pregnant women were active smokers. The most important factor in smoking cessation was the thought it could be harmful to the baby. Nevertheless, 30% of them thought electronic cigarette was not harmful to the baby. The rate of smoking electronic cigarettes among pregnant women was 3%.25% of pregnant women thought that cigarette smoking was more harmful, and the 45% were not aware of the harms and addictive effects of electronic cigarettes. Conclusion(s): regnant women seem to need more information about electronic cigarettes lately, as if they were harmless. Educational sessions, planned together with the obstetricians and chest physcians would make a significant contribution to maternal and child health.
3725	Chen Yi-Lung, Wu Shang-Chi, Chen Yen-Tyng, Hsiao Po-Chang, Yu Ya-Hui, Ting Te-Tien, Chen Chuan-Yu, Tu Yu-Kang, Huang Jiun-Hau, Yang Hao-Jan, Li Chung-Yi, Strong Carol, Yen Cheng-Fang, Yen Chia-Feng, J Chen Wei (2019///) E-Cigarette Use in a Country With Prevalent Tobacco Smoking: A Population-Based Study in Taiwan. Journal of epidemiology; 29<br><br>**Keywords:**<br>Adolescent,Adult,Age Distribution,Child,Cross-Sectional Studies,*Electronic Nicotine Delivery Systems/sn [Statistics & Numerical Data],Female,Health Surveys,Humans,Male,Middle Aged,Prevalence,Taiwan/ep [Epidemiology],*Tobacco Smoking/ep [Epidemiology],Y<br><br>**Abstract:** BACKGROUND: The different profiles of e-cigarette users in different age groups have seldom been investigated, particularly in populations facing a high prevalence of cigarette smoking. This study aims to examine the prevalence and correlates of e-cigarette use separately for adolescents and adults in nationally representative samples in Taiwan., METHODS: Among 17,837 participants in the 2014 National Survey of Substance Use in Taiwan, 4445 were aged 12 to 17 years and 13,392 were aged 18 to 64 years. Individuals' lifetime tobacco use was divided into four groups: non-use, exclusive e-cigarette use, exclusive cigarette use, and dual use. Questions on sociodemographic features, use and problematic use of tobacco, alcohol, and other drugs, and psychosocial distress, among others, were administered using a computer-assisted self-interview on tablet computers., RESULTS: Among lifetime users of e-cigarette (2.2% for adults and 0.8% for adolescents), 4.5% for adults and 36.6% for adolescents were exclusive e-cigarette users. From use of exclusive e-cigarettes to use of exclusive cigarettes to dual use, those usage groups were related to an increasing trend of adjusted odds ratios for use of other psychoactive substances, particularly problematic use of alcohol or drugs, and with more depressive symptoms. Two correlates were specific to e-cigarette use: alcohol use had stronger relationships with e-cigarette use

	among adolescents, and younger adults (18-34) were more likely to try e-cigarettes compared to older adults., CONCLUSIONS: These results provide essential information regarding e-cigarette use in the general population, and future prevention strategies should account for its specific correlates in young people.
3733	Lee Youn Ok, K Pepper Jessica, J MacMonegle Anna, M Nonnemaker James, C Duke Jennifer, Porter Lauren (2018///) Examining Youth Dual and Polytobacco Use with E-Cigarettes. International Journal of Environmental Research and Public Health; 15<br><br>**Keywords:**<br>Adolescent,Child,*Electronic Nicotine Delivery Systems,Female,Florida,Humans,Intention,Logistic Models,Male,Marketing,Odds Ratio,Perception,*Smoking/px [Psychology],*Students/px [Psychology], Surveys and Questionnaires,*Tobacco Products<br><br>**Abstract:** E-cigarettes and other non-cigarette tobacco products are increasingly popular among youth. Little is known to inform public health efforts to reduce youth use. We examined psychosocial correlates of single and multiple tobacco product use among youth e-cigarette users. Data were from the 2014 Florida Youth Tobacco Survey (N = 69,923), a representative sample of Florida middle and high school students. Associations between combinations of e-cigarette, cigarette and other tobacco product (OTP) use and psychosocial variables were examined using multinomial logistic regression with an analytic sample of N = 2756. Most e-cigarette-using youth used at least one other product (81%). Perceiving cigarettes as easy to quit was significantly associated with greater likelihood of combined e-cigarette/OTP use (relative risk ratio (RRR) = 2.51, p < 0.001) and combined e-cigarette/cigarette/OTP use (RRR = 3.20, p < 0.0001). Thinking you will be smoking cigarettes in 5 years was associated with product combinations that include cigarettes. Tobacco company marketing receptivity was associated with multiple product user types. Given that specific psychosocial factors put youth at risk for concurrent use of e-cigarettes with tobacco products, public health efforts should address polytobacco use specifically, instead of individual product use. Youth perceptions about the ease of quitting cigarettes, intentions to continue smoking cigarettes and receptivity to tobacco company marketing are promising areas for messaging aimed at reducing e-cigarette polytobacco product use.
3736	Chien Yu-Ning, Gao Wayne, Sanna Mattia, Chen Ping-Ling, Chen Yi-Hua, Glantz Stanton, Chiou Hung-Yi (2019///) Electronic Cigarette Use and Smoking Initiation in Taiwan: Evidence from the First Prospective Study in Asia. International Journal of Environmental Research and Public Health; 16<br><br>**Keywords:**<br>Adolescent,*Adolescent Behavior,Adult,*Electronic Nicotine Delivery Systems/sn [Statistics & Numerical Data],Female,Humans,Male,Prospective Studies,Schools,*Smoking/ep [Epidemiology],Students/sn [Statistics & Numerical Data],Surveys and Questionnaires,T<br><br>**Abstract:** A growing literature indicates that electronic cigarette use increases the risk of subsequent initiation of conventional smoking among cigarette-naive adolescents in several Western countries. This research assesses the same relationship in an Asian country, Taiwan. The Taiwan Adolescent to Adult Longitudinal Study is a school-based survey that was carried out in two waves in 2014 (baseline) and in 2016 (follow-up). It employs probability sampling to create nationally representative samples of students in junior high school (mean age 13, 7th grade at baseline) and in senior high school (mean age 16, 10th grade at baseline). Data from this survey were analyzed via logistic regression to estimate the association between ever use of e-cigarettes at baseline and smoking initiation at follow-up, accounting for susceptibility to smoking, socio-demographic profile, depression status, and peer support. Among the 12, 954 cigarette-naive students surveyed, those with e-cigarette experience at baseline exhibited higher odds of smoking initiation at follow-up (Odds Ratio = 2.14, 95% CI (1.66, 2.75), p < 0.001). For the first time, we confirmed, through a longitudinal survey, a prospective association between ever use of e-

	cigarettes and smoking initiation in an Asian adolescent population. The restrictive policy on e-cigarettes currently in force in Taiwan is justified to prevent both e-cigarette and cigarette use among adolescents.
3738	Cahn Zachary, J Berg Carla (2018///) Commentary on Seyla et al. (2018): Advantages in the consideration of causal mechanisms for studies of gateway e-cigarette use. Addiction (Abingdon, England); 113<br><br>**Keywords:**<br>*Electronic Nicotine Delivery Systems,Humans,Smoking,Tobacco Smoking,*Tobacco Use Disorder, *Vaping<br><br>**Abstract:**
3744	L Zhao, L Mbulo, K Palipudi, J Wang, B King (2019///) Awareness and use of e-cigarettes among urban residents in China. Tobacco Induced Diseases; 17<br><br>**Keywords:**<br>adolescent,adult,age,*awareness,*China,education,*electronic cigarette,high school,household,human, major clinical study,male,monitoring,prevalence,public health,questionnaire,review,sample size,smoking, statistics,tablet,*urban population<br><br>**Abstract:** INTRODUCTION The long-term health effects of e-cigarettes are uncertain, and data on e-cigarette use among Chinese adults are limited. This study examined the prevalence and correlates of e-cigarette awareness and use among urban residents in China. Data came from the China City Adult Tobacco Survey (CCATS), a city-representative household survey conducted using electronic tablets during 2013-2014 in 14 major Chinese cities. METHODS CCATS used multistage geographically clustered samples with standardized survey protocols and questionnaire to ensure data comparability. Overall, 31151 adults completed the survey, with sample size varying from 1977 to 3838 across cities, and survey response rates ranging from 79.8% to 97.5%. Respondents were considered current e-cigarette users if they self-reported using e-cigarettes 'daily' or 'less than daily' at the time of the survey. Descriptive statistics and multivariate logistic regression were conducted. Assessed correlates included: age, education, quit attempts in past 12 months, cigarettes smoked per day, and monthly expenditures on cigarettes. RESULTS Overall, 46.7% of respondents were aware of e-cigarettes, 2.9% ever used, and 0.8% currently used. Most current e-cigarette users (93.0%) also currently smoked tobacco. Among male current tobacco smokers, adjusted odds ratio (AOR) of current e-cigarette use was higher among those aged 15-29 (AOR=2.5; 95% CI: 1.5-4.3) or 30-49 (AOR=1.9; 95% CI: 1.0-3.4) than those >=50 years; those who attempted to quit in the past 12 months than those who did not (AOR=4.7; 95% CI: 2.2-10.1); those with a college degree (AOR=3.4; 95% CI: 1.9-6.2) or just finished high school (AOR=2.2; 95% CI: 1.2-4.2) than those who did not finish high school; and those who smoked >=15 cigarettes per day (AOR=2.8; 95% CI: 1.4-5.6) than those who smoked fewer. CONCLUSIONS These findings reveal that during 2013-2014, many urban Chinese adults were aware of e-cigarettes, while use was relatively low and most current users also smoked tobacco. Continued monitoring of e-cigarettes could help inform public health policy, planning, and practice.Copyright © 2019 Zhao L.
3745	L Zhao, J Wang (2018///) E-cigarettes use among urban male tobacco smokers age 15 years or older in China. Tobacco Induced Diseases; 16<br><br>**Keywords:**<br>adolescent,adult,*China,controlled study,education,*electronic cigarette,human,major clinical study, male,monitoring,prevalence,public health,*smoking cessation,urban population,conference abstract<br><br>**Abstract:** Background: There is paucity of data regarding e-cigarette use in China. Monitoring e-cigarette use is important given the rapid increase of e-cigarette use in some countries. We investigated the prevalence and determinants of e-cigarette use among Chinese male urban tobacco smokers, as most

	Chinese urban residents who use e-cigarettes are male smokers (~90%). Method(s): We analyzed data from China City Adult Tobacco Surveys, which were conducted in 14 major Chinese cities individually in 2013-14 with 15,008 males aged >=15 years. Current e-cigarette users were defined as persons who reported using e-cigarette daily or less than daily at the time of the survey. We calculated the prevalence of e-cigarette use among male current tobacco smokers. Multivariate logistic regression was used to assess determinants of e-cigarette use, including age, education, quit attempts in the past 12 months, cigarettes smoked per day, and monthly expenditure on cigarettes. Result(s): Among urban male current smokers in China, 3.6% were current e-cigarette users. The likelihood of being current e-cigarette users was significantly higher among: smokers aged 15- 29 (OR=2.5) or 30-49 (OR=2.1) years than 50+ years; those who attempted to quit than those who made no attempt (OR=4.5); those with >=college education than those with <=high school education (OR=2.6); and those who smoked >=15 cigarettes per day (OR=2.7) than those who smoked 1-14 cigarettes per day (all p< 0.05). Conclusion(s): Among urban male tobacco smokers in China, current e-cigarette use was higher among those who were younger, had more education, attempted to quit smoking in the past year, and smoked more cigarettes per day. Given that e-cigarettes are not currently regulated and often marketed as healthier alternatives to cigarettes in China, continued monitoring of e-cigarette use is important to inform public health policy, planning, and practice.
3748	A M E Zuckermann, G Williams, K Battista, M e Groh, Y Jiang, S T Leatherdale (2019///) Trends of poly-substance use among Canadian youth. Addictive Behaviors Reports; 10<br><br>**Keywords:**<br>acceleration,adolescent,article,Canadian,cannabis use,child,cigarette smoking,cohort analysis,drinking behavior,female,high school student,human,indigenous people,*juvenile,major clinical study,male, prevalence,priority journal,prospective study,sex diff<br><br>**Abstract:** Introduction: Poly-substance use, increasingly understood as a behaviour with uniquely adverse consequences, is on the rise among Canadian youth. High levels of e-cigarette vaping and the recent legalization of recreational cannabis use may result in an acceleration of this trend. The aim of this work was to characterise changes in youth poly-substance use over time, generate baseline data for future investigations, and highlight areas of interest for policy action. Method(s): Descriptive statistics and regression models explored patterns and trends in concurrent use of multiple substances (alcohol, cigarettes, cannabis, and e-cigarettes) among Canadian high school students taking part in the COMPASS prospective cohort study during Y2 (2013/2014; n = 45,298), Y3 (2014/2015, n = 42,355), Y4 (2015/2016; n = 40,436), Y5 (2016/2017; n = 37,060), and Y6 (2017/2018; n = 34,879). Result(s): Poly-substance use increased significantly over time, with over 50% of students who used substance reporting past-year use of multiple substances by 2017/2018. Male and Indigenous students were significantly more likely to report poly-substance use than female and white students respectively. E-cigarette vaping doubled from Y5 to Y6 and was included in all increasingly prevalent substance use combinations. Conclusion(s): Youth poly-substance use, rising since 2012/2013, saw a particularly steep increase after 2016/2017. Differential effects were observed for distinct demographic subpopulations, indicating tailored interventions may be required. E-cigarette vaping surged in parallel with the observed increase, suggesting a key role for this behaviour in shaping youth poly-substance use.Copyright © 2019
3749	M Conner, S Grogan, R Simms-Ellis, K Flett, B Sykes-Muskett, L Cowap, R Lawton, C Armitage, D Meads, L Schmitt, C Torgerson, R West, K Siddiqi (2019) Evidence that an intervention weakens the relationship between adolescent electronic cigarette use and tobacco smoking: a 24-month prospective study.. Tobacco control;<br><br>**Keywords:**<br><br>**Abstract:** BACKGROUND: The electronic cigarette (e-cigarette) use to subsequent smoking relationship in

	adolescents has received much attention. Whether an intervention to reduce smoking initiation attenuated this relationship was assessed. METHOD: Data were from 3994 adolescent never smokers (aged 13-14 years at baseline) as part of a cluster randomised controlled trial. Self-report measures of smoking, e-cigarette use and covariates were assessed and used to predict ever smoked cigarettes, any recent tobacco smoking and regularly smoked cigarettes at 24-month follow-up. RESULTS: Baseline ever use of e-cigarettes was associated with ever smoked cigarettes (OR=4.03, 95% CI 3.33 to 4.88; controlling for covariates, OR=2.78, 95% CI 2.20 to 3.51), any recent tobacco smoking (OR=3.38, 95% CI 2.72 to 4.21; controlling for covariates, OR=2.17, 95% CI 1.76 to 2.69) and regularly smoked cigarettes (OR=3.60, 95% CI 2.35 to 5.51; controlling for covariates, OR=1.27, 95% CI 1.17 to 1.39) at follow-up. For ever smoked cigarettes only, the impact of e-cigarette use was attenuated in the intervention (OR=1.83) compared with control (OR=4.53) condition. For ever smoked cigarettes and any recent tobacco smoking, the impact of e-cigarette use was attenuated among those with friends who smoked (OR=2.05 (ever smoked); 1·53 (any tobacco use)) compared with those without friends who smoked (OR=3.32 (ever smoked); 2·17 (any tobacco use)). CONCLUSIONS: This is one of the first studies to show that e-cigarette use was robustly associated with measures of smoking over 24 months and the first to show an intervention to attenuate the relationship. Further research with a broader age range of adolescents is required.
3750	JC Chen, B Das, EL Mead, DLG Borzekowski (2017) Flavored E-cigarette Use and Cigarette Smoking Susceptibility among Youth.. Tobacco regulatory science; 3<br><br>**Keywords:**<br><br><br>**Abstract:** Objectives: Research indicates youth e-cigarette use may lead to the use of conventional cigarettes, but the specific effects of flavored e-cigarettes-which greatly appeal to youth-are unknown. Therefore, this study examines how flavored e-cigarette use predicts cigarette smoking susceptibility among youth non-smokers. Methods: We used 2014 National Youth Tobacco Survey to explore the relationship between current e-cigarette use (non-use, flavored and plain e-cigarette use) and smoking susceptibility among 18,392 youth non-smokers (ages 11-18 years). Results: Overall, 2.2% and 2.1% of non-smoking youth currently used plain and flavored e-cigarettes. Compared to 30.0% of non-users, 61.1% and 74.1% of plain and flavored e-cigarette users reported smoking susceptibility. Flavored e-cigarette users were more likely to be susceptible than plain e-cigarette users (AOR=1.7, p< .001) and non-users (AOR=3.8, < .0001). The magnitude of the relationship between flavored e-cigarette use and smoking susceptibility was significantly higher for females (AOR=6.5, p< .01) than males (AOR=2.5, p< .01). Conclusions: Flavored more so than plain e-cigarette use is strongly associated with smoking susceptibility among non-smoking youth. Flavored e-cigarettes are recruiting females and those with low smoking-risk profile to experiment conventional cigarettes. Legislative efforts to ban e-cigarettes with child-friendly flavors should be enhanced.
3772	Aljandaleh, H., Bolze, C., El-Khoury Lesueur, F., Melchior, M., Mary-Krause, M. (2020) Factors Associated with Electronic Cigarette Use among Young Adults: The French "Trajectoires EpideMiologiques en POpulation" (TEMPO) Cohort Study. Substance use & misuse; 55<br><br>**Keywords:**<br>adult article asthma cannabis use cigarette smoking *cohort analysis controlled study *electronic cigarette ex-smoker female *France human major clinical study male mental capacity obesity occupation perception questionnaire young adult alcohol<br><br>**Abstract:** Background: Electronic cigarettes (e-cigarettes) are sold in France since 2010 and have rapidly become popular. However, factors associated with e-cigarette use among young adults are not well known. Method(s): We used data from the 2015 French TEMPO community based cohort study, restricted to current and former smokers with data on e-cigarette use (n=368 adults, 23-41years).

	Participants completed a self-administered questionnaire including information on family status, educational attainment, occupation and type of work contract, health problems, alcohol and cannabis use, electronic cigarette use, as well as perceptions of e-cigarettes. Use of traditional tobacco was assessed in 2011 and 2015. Data were analyzed using logistic regression models. Result(s): Among current and former smokers, 26.9% reported lifetime e-cigarettes use and 15.2% current use. Factors associated with lifetime use were: low socioeconomic position (OR=2.2; 95% CI=1.2-4.2), traditional cigarette use (OR associated with smoking in 2011 and 2015=13.1; 95% CI=5.2-32.6) and positive perceptions of e-cigarettes (OR = 4.4; 95% CI=2.4-8.1) as well as asthma (OR=2.1; 95% CI=0.9-4.9) and overweight/obesity (OR=2.5, 95% CI=0.9-6.9). Factors associated with current use were traditional cigarette smoking (OR associated with smoking in 2011 and 2015=3.9; 95% CI= 1.3-12.2) and positive perceptions of e-cigarettes (OR =4.4; 95% CI=2.3-8.4). Conclusion(s): Young adults who use e-cigarettes tend to persist in smoking traditional cigarettes. The conditions under which e-cigarette use can help individuals quit traditional tobacco products remain to be elucidated.
3773	Allen, B., Stuart, G. L. (2019) Decreased Cigarette Smoking but No Change in Use of Electronic Cigarettes Following a University-Wide Smoking Ban. Substance Abuse: Research and Treatment; 13<br><br>**Keywords:**<br>adult article *cigarette smoking college student comparative study female human male prevalence self report *smoking ban *smoking cessation smoking regulation *electronic cigarette<br><br>**Abstract:** The prevalence of electronic cigarette use is increasing, particularly among youth. This recent trend is troubling given that electronic cigarette use is associated with future cigarette smoking. Here, we assessed the prevalence of cigarette smoking and use of electronic cigarettes among college students before and after implementation of a university-wide smoking ban on campus. We found that after the smoking ban was implemented, the prevalence of self-reported combustible cigarette smoking decreased (12% versus 7%; unadjusted: OR = 0.55, 95% CI = 0.34, 0.89, p = 0.015), but we did not observe a difference in the prevalence of electronic cigarette use (26.3% versus 27.5%; unadjusted: OR = 1.06, 95% CI = 0.78, 1.43, p = 0.699). Future studies should identify factors that increase the impact of university smoking policies on electronic cigarette use.Copyright © The Author(s) 2019.
3782	Anand, V. (2019) 50.3 E-CIGARETTE AWARENESS, USE, AND PERCEPTION, AND EFFECTS OF A SCHOOL-BASED INTERVENTION. Journal of the American Academy of Child and Adolescent Psychiatry; 58<br><br>**Keywords:**<br>adolescent African American *awareness controlled study convenience sample *electronic cigarette female Food and Drug Administration high school student Hispanic human major clinical study male North Carolina *perception questionnaire tobacco use cannab<br><br>**Abstract:** Objectives: Electronic cigarettes (e-cigarettes) have become popular and currently are the most commonly used form of tobacco. Their easy access, flavors, advertisements with untested claims, and widespread belief about their role in tobacco cessation have fueled their use. An increasing number of adolescents have reported using other substances via e-cigarettes. The studies examining these tobacco products among adolescents have indicated that these products act as a gateway to combustible tobacco. Recently, the US FDA regulations have presumably restricted access to electronic cigarettes to protect the American youth. We examined a subset of the high school population with regard to their access, knowledge, attitudes, and effect of an e-cigarette-related educational intervention. Method(s): A 20-item questionnaire was modeled after our previous study and the Monitoring the Future study. The questionnaire included information about demographics, tobacco use, and quit attempts. It was designed, piloted, and administered to a convenience sample group of public high school students (N = 119) before and after a 30-minute educational intervention in May 2018, in an urban county in eastern North Carolina. Result(s): Completers (108/119) were aged 15.2 +/- 0.93 years; 56.5 percent were males, and 36 percent were females; 48 percent were African American, 30.5 percent were white, and 5.5

	percent were Hispanic. A large majority (75%) were in grade 9, whereas 17.6 percent were in grade 10, 5.5 percent were in grade 11, and 1.8 percent were in grade 12. Most (90.6%) knew about e-cigarettes, and 33 percent reported they had tried an e-cigarette before. Of the sample group, 21 percent used e-cigarettes for nicotine, whereas 13.2 percent reported using hash oil or marijuana wax. A considerable number of students (40.6%) perceived e-cigarettes as less harmful than traditional cigarettes, and 38.7 percent thought of e-cigarettes as a quitting aid. A large majority (72.3%) were reported to have a better understanding of the dangers posed by e-cigarettes after a 20-minute educational intervention. Conclusion(s): E-cigarette use is ever increasing and evident among high school students in eastern North Carolina. A brief, school-based, educational intervention indicated positive effects on harm perception and future use. More school-based education and awareness are needed to prevent further escalation of e-cigarette use. ADOL, SUD, SIICopyright © 2019
3785	Anoruo, N., Houston, T. K., Flahive, J., Chen, J., Sadasivam, R. S. (2020) Effect of E-cigarette use on smoking cessation in low motivation smokers. American Journal of Respiratory and Critical Care Medicine; 201<br><br>**Keywords:**<br>adult *cigarette smoking cohort analysis conference abstract controlled study current smoker demography *electronic cigarette female follow up high school human major clinical study male *motivation prospective study *smoking cessation<br><br>**Abstract:** RATIONALE: The use of e-cigarettes as a harm reduction strategy is controversial. Many smokers believe e-cigarettes are an effective smoking cessation aid. However, prior studies are mixed regarding the impact of e-cigarette use on combustible cigarette use. Among a cohort of low motivation smokers (active smokers who express low or no intention of quitting smoking), we sought to investigate the association of e-cigarette use with smoking cessation outcomes. METHOD(S): In a prospective cohort study of low motivation smokers, we assessed baseline demographic characteristics and smoking behavior. The following baseline question "have you ever tried an 'e-cigarette', even just one time?" was used to characterize subjects as traditional smokers (reported use of combustible cigarettes alone) or dual use smokers (reported use of combustible cigarettes + ever use of e-cigarettes). All smokers were then encouraged to consider a period of brief abstinence from combustible cigarettes during the three weeks after baseline. We compared follow-up cessation outcomes of traditional smokers versus dual smokers using ttests for continuous variables and chi-square tests for dichotomous variables. RESULT(S): Out of 405 smokers, 49% were female, 82% identified as white, 8.8% as black, and 29.9% had a high school/GED-equivalent education or less. Dual smokers were on average younger than traditional smokers, with 27% of dual smokers less than 40 years old versus 16% of traditional smokers (p=0.02). At baseline, dual smokers smoked an average 16 cigarettes per day, compared with 14 cigarettes per day in traditional smokers (p=0.03). At three weeks, dual smokers were able to abstain from smoking approximately half as long as traditional smokers, reporting an average 0.93 day abstinence interval compared to 1.8 days for traditional smokers (p=0.01). 6.3% of dual smokers were successfully able to quit smoking completely at three weeks, versus 13% of traditional smokers (p=0.02). At six months, dual smokers smoked more cigarettes daily than traditional smokers, with an average daily number of 12 versus 9.4 for traditional smokers (p=0.04). Dual smokers also had a smaller percent reduction in baseline cigarette use at six months: 21% percent reduction compared to 33% reduction in traditional smokers (p=0.04). CONCLUSION(S): In low motivation smokers, e-cigarette use was not associated with lower combustible cigarette use. When compared to traditional smokers, dual smokers smoked more cigarettes daily on average, had shorter smoking abstinence intervals, and had less success at quitting smoking completely. More research is needed on how best to help low motivation smokers quit smoking.
3787	Aqeeli, A. A., Makeen, A. M., Al Bahhawi, T., Ryani, M. A., Bahri, A. A., Alqassim, A. Y., El-Setouhy, M. (2020) Awareness, knowledge and perception of electronic cigarettes among undergraduate students in Jazan Region, Saudi Arabia. Health & social care in the community;

	**Keywords:**<br>adult article *awareness controlled study cross-sectional study *electronic cigarette female health hazard human human tissue major clinical study male *perception prevalence questionnaire *Saudi Arabia *smoking cessation *undergraduate student universi<br><br>**Abstract:** The use of electronic cigarettes (e-cigarettes) has steadily grown over the past few years, rising in popularity amongst young adults, especially. Owing to the perception that e-cigarettes are less harmful than conventional cigarettes, some people have resorted to using them as a means of quitting cigarette smoking. This study aimed to assess the awareness, knowledge, perception and use of e-cigarettes among Jazan University students in Saudi Arabia. An observational cross-sectional study was conducted with 775 students. Using stratified random sampling to recruit study participants, the dataset tabling the use of e-cigarettes, as well as knowledge, perception and awareness was collected using a translated and culturally adapted structured Arabic questionnaire. Our results showed that 21.0% of the participants used e-cigarettes and 35.1% of them used them to quit smoking. E-cigarette use was mostly reported by students from the College of Medical Applied Sciences (31.7%). The overall knowledge score was 3.9, with a standard deviation of 1.3 for the score range from zero to seven. About half of the participants correctly identified e-cigarettes as not less addictive' than cigarettes. However, 70.2% of students failed to identify e-cigarettes as a source of second-hand exposure to nicotine. About two-thirds of the sample believed that e-cigarettes could adversely affect health. The perception of using e-cigarettes for smoking cessation was not significantly associated with e-cigarette use. In conclusion, the prevalence of e-cigarette use was similar to that reported in the literature. However, Jazan University students failed to identify the health risks associated with e-cigarette use. Therefore, a strategy to enhance students' awareness of the benefits and harms of e-cigarettes is needed.Copyright © 2020 John Wiley & Sons Ltd.
3791	Asher, Tim, Belden, Jenna Leah, Kelsberg, Gary, Safranek, Sarah (2019) Does using e-cigarettes increase cigarette smoking in adolescents?. The Journal of family practice; 68<br><br>**Keywords:**<br>Adolescent *Adolescent Behavior/px [Psychology] *Cigarette Smoking/px [Psychology] Cohort Studies Electronic Nicotine Delivery Systems Female Humans Male Prospective Studies Risk Factors *Vaping/ae [Adverse Effects] *Vaping/px [Psychology]<br><br>**Abstract:** Probably. Electronic cigarette (e-cigarette) use by adolescents is associated with a 2- to 4-fold increase in cigarette smoking over the next year (strength of recommendation: A, meta-analysis and subsequent prospective cohort studies).
3795	Auderset, D., Barrense-Dias, Y., Akre, C., Suris, J. C. (2020) 66. To Quit or to Start? The Effect of E-Cigarettes Use On Smoking Traditional Cigarettes Among Young People in Switzerland. Journal of Adolescent Health; 66<br><br>**Keywords:**<br>adolescent adult controlled study counseling *electronic cigarette female human juvenile lifestyle longitudinal study major clinical study male marketing non-smoker smoke smoking cessation *smoking habit *Switzerland conference abstract<br><br>**Abstract:** Purpose: The aim of this research was to study trajectories of use of electronic cigarettes (EC) over time among youths distinguishing between EC only and dual users (EC + traditional cigarettes) and to assess reasons to use EC. Particularly, we aimed to observe whether ECs were a gateway to traditional cigarettes (TC) and if they effectively helped in quitting smoking. Method(s): Data came from the GenerationFRee survey, a longitudinal study on youths' lifestyles (15-24 years at baseline [T1]) in the canton of Fribourg, Switzerland. Participants (N=1437, mean age 16.7; 51.8% females) were divided at T1 in 4 groups according to their current smoking status: TC-only smokers (N=378, 26.3%; 51.5% females), EC-only (N=42, 2.9%; 27.3% females), dual users (N=97, 6.7%; 39.6% females) and non-smokers (N=920,

	64%; 53.4% females). Additionally, participants in the EC-only and dual groups at T1 reported the reasons for EC use. Finally, we determined the trajectories between T1 and T2 (2016 and 2017) by observing in which of the 4 categories EC-only and dual users at T1 were at T2. Result(s): Males outnumbered females in the use of ECs, alone or dually. Among current EC (alone or dually) users at T1, 79.9% responded having used it to "try", 13.8% to "smoke where TCs were not allowed", 12.8% to "reduce tobacco use" and 8.4% to "stop smoking". Regarding group trajectories, among EC-only users at T1, 23.7% became TC-only smokers, 20.1% remained EC-only users, 4.6% became dual users and 51.5% non-smokers at T2. Among dual users at T1, 68.7% became TC-only smokers, 1.1% EC users only, 17.9% dual users and 12.2% non-smokers at T2. Moreover, the percentage of quitters among dual users was lower than among TC-only users at T2 (12.2% vs. 16.4%). Among dual users indicating wanting to quit as a reason to use ECs, 15.3% effectively quitted smoking, while among those indicating to smoke when TCs were not allowed, none stopped smoking a year later. Conclusion(s): The vast majority of youths used ECs "to try", suggesting that the aggressive marketing campaigns that target them work. Furthermore, ECs seem to be a gateway to smoking and have little impact on helping to quit. Among EC-only users at baseline, about half maintained their smoking habits a year later implying that ECs are attractive for youth and a gateway to TCs for almost a third of them. As dual users at baseline reported the lowest quitting rate at T2, it suggests that using both products may make it more difficult to quit rather than making it easier, for example allowing to smoke where TCs are prohibited. There is a need to protect youths from the marketing of products that are presented as an alternative to traditional tobacco use with no evidence to support it among young people. It is important to include ECs in prevention counseling, as they may be a gateway to smoking traditional cigarettes. Sources of Support: The survey was financed by the Programme Intercantonal de Lutte contre la Dependance au Jeu (PILDJ) and the Canton of Fribourg.Copyright © 2019
3798	Azagba, S., Qeadan, F., Shan, L., Latham, K., Wolfson, M. (2020) E-Cigarette Use and Transition in Adult Smoking Frequency: A Longitudinal Study. American Journal of Preventive Medicine; 59<br><br>**Keywords:**<br>adult aged article Black person body mass Caucasian *cigarette smoking cohort analysis demography female Hispanic human longitudinal study male population research risk assessment smoking cessation *smoking habit United States *electronic cigarette<br><br>**Abstract:** Introduction: The relationship between e-cigarette use and cigarette smoking is the subject of ongoing debate. There is limited research on e-cigarette use and changes in the frequency of cigarette smoking. This study examines whether the frequency of e-cigarette use is associated with changes in cigarette smoking behavior among U.S. adults. Method(s): The study used data (n=20,558) from Waves 1 (2013-2014) and 2 (2014-2015) of the Population Assessment of Tobacco and Health, analyzed in 2019. Multivariable multinomial logistic regression assessed the association between e-cigarette use at Wave 1 and change in cigarette smoking frequency between Waves 1 and 2. Result(s): Every day cigarette smokers who used e-cigarettes some days (OR=1.95, 95% CI=1.27, 2.98) and every day (OR=3.37, 95% CI=1.78, 6.36) in Wave 1 had significantly higher odds of switching to some days cigarette smoking in Wave 2. Every day smokers who used e-cigarettes every day in Wave 1 had higher odds of becoming former cigarette smokers in Wave 2. Likewise, e-cigarette use at baseline among former cigarette smokers was associated with higher odds of switching to some days cigarette smoking (experimental e-cigarette use: OR=5.43, 95% CI=2.13, 10.72; some days e-cigarette use: OR=4.78, 95% CI=2.13, 10.72). In addition, experimental smokers who were also some days e-cigarettes users in Wave 1 had significantly lower odds of switching to experimental former smokers. Conclusion(s): Although e-cigarette use may reduce cigarette smoking frequency among continuing smokers, findings suggest that e-cigarette use may be associated with cigarette smoking relapse among former smokers.Copyright © 2020 American Journal of Preventive Medicine
3801	Baig, S. A., Giovenco, D. P. (2020) Behavioral heterogeneity among cigarette and e-cigarette dual-users and associations with future tobacco use: Findings from the Population Assessment of Tobacco and

	Health Study. Addictive Behaviors; 104<br><br>**Keywords:**<br>adult article controlled study demography education female *harm reduction *health disparity human major clinical study male prevalence public health *vaping<br><br>**Abstract:** Dual-users of cigarettes and e-cigarettes are commonly treated as a single group. Our study applied a more nuanced classification of this complex behavior to examine its associations with future tobacco use behaviors using data from Waves 1 and 3 of the Population Assessment of Tobacco and Health. Dual-users at Wave 1 (n = 1,665) were categorized into 4 groups based on the frequency with which they used each product (i.e., some days, daily). Analyses identified sociodemographic correlates of group membership and the prevalence of (1) completely switching to e-cigarettes and (2) quitting both products by Wave 3. Dual-users who smoked cigarettes every day and used e-cigarettes some days (69.6%) were the majority and more likely to have lower education ($p < .001$). Although some day smoking and daily e-cigarette use was the least common category (5.9%), these individuals were most likely to have completely switched to e-cigarettes by Wave 3 (aOR = 6.19, 95% CI = 3.91, 9.79). Dual-users who smoked and used e-cigarettes some days were most likely to have completely quit tobacco by Wave 3 (aOR = 3.98, 95% CI = 2.93, 5.40). In general, dual-users who had higher education or income were more likely to have completely switched to e-cigarettes or quit tobacco use by Wave 3. Adults who concurrently use cigarettes and e-cigarettes exhibit considerable heterogeneity in their use of these tobacco products. Dual-users that are higher on the socioeconomic gradient are more likely to engage in plausibly less harmful dual-use behaviors, which are more strongly associated with harm reduction and cessation behaviors. Future research should consider this variation to more accurately characterize the public health impact of dual-use.Copyright © 2020 Elsevier Ltd
3805	Bao, Wei, Liu, Buyun, Du, Yang, Snetselaar, Linda G., Wallace, Robert B. (2020) Electronic Cigarette Use Among Young, Middle-aged, and Older Adults in the United States in 2017 and 2018. JAMA internal medicine; 180<br><br>**Keywords:**<br>Adolescent Adult Age Distribution Aged Female Humans Male Middle Aged Prevalence United States/ep [Epidemiology] *Vaping/ep [Epidemiology] Vaping/td [Trends] Young Adult<br><br>**Abstract:**
3809	Barrington-Trimis, Jessica L., Yang, Zhi, Schiff, Sara, Unger, Jennifer, Cruz, Tess Boley, Urman, Robert, Cho, Junhan, Samet, Jonathan M., Leventhal, Adam M., Berhane, Kiros, McConnell, Rob (2020) E-cigarette Product Characteristics and Subsequent Frequency of Cigarette Smoking. Pediatrics; 145<br><br>**Keywords:**<br>Adolescent *Cigarette Smoking/ep [Epidemiology] Cigarette Smoking/px [Psychology] *Cigarette Smoking/td [Trends] *Electronic Nicotine Delivery Systems Female Follow-Up Studies Humans Male *Vaping/ep [Epidemiology] Vaping/px [Psychology] *Vaping/td [Tren<br><br>**Abstract:** BACKGROUND: There is a dearth of evidence regarding the association of use of electronic cigarettes (e-cigarettes) with certain product characteristics and adolescent and young adult risk of unhealthy tobacco use patterns (eg, frequency of combustible cigarette smoking), which is needed to inform the regulation of e-cigarettes., METHODS: Data were collected via an online survey of participants in the Southern California Children's Health Study from 2015 to 2016 (baseline) and 2016 to 2017 (follow-up) (N = 1312). We evaluated the association of binary categories of 3 nonmutually exclusive characteristics of the e-cigarette used most frequently with the number of cigarettes smoked in the past 30 days at 1-year follow-up. Product characteristics included device (vape pen and/or modifiable electronic cigarette [mod]), use of nicotine in electronic liquid (e-liquid; yes or no), and use for dripping

	(directly dripping e-liquid onto the device; yes or no)., RESULTS: Relative to never e-cigarette users, past-30-day e-cigarette use was associated with greater frequency of past-30-day cigarette smoking at follow-up. Among baseline past-30-day e-cigarette users, participants who used mods (versus vape pens) smoked >6 times as many cigarettes at follow-up (mean: 20.8 vs 1.3 cigarettes; rate ratio = 6.33; 95% confidence interval: 1.64-24.5) after adjustment for sociodemographic characteristics, baseline frequency of cigarette smoking, and number of days of e-cigarette use. After adjustment for device, neither nicotine e-liquid nor dripping were associated with frequency of cigarette smoking., CONCLUSIONS: Baseline mod users (versus vape pen users) smoked more cigarettes in the past 30 days at follow-up. Regulation of e-cigarette device type warrants consideration as a strategy to reduce cigarette smoking among adolescents and young adults who vape. Copyright © 2020 by the American Academy of Pediatrics.
3812	Beard, E., West, R., Michie, S., Brown, J. (2020) Association of prevalence of electronic cigarette use with smoking cessation and cigarette consumption in England: a time-series analysis between 2006 and 2017. Addiction (Abingdon, England); 115<br><br>**Keywords:**<br>adolescent article *cigarette smoking controlled study current smoker *electronic cigarette *England human major clinical study *prevalence *smoking cessation *time series analysis<br><br>**Abstract:** AIMS: To provide up-to-date estimates of how changes in the prevalence of electronic cigarette (e-cigarette) use in England have been associated with changes in smoking cessation activities and daily cigarette consumption among smokers in England. DESIGN: Time-series analysis of population trends.England. PARTICIPANTS: Participants came from the Smoking Toolkit Study, which involves repeated, cross-sectional household surveys of individuals aged 16 years and older in England. Data were aggregated on approximately 1200 past-year smokers each quarter (total n = 50498) between 2007 and 2017. MEASUREMENTS: Prevalence of e-cigarette use in current smokers was used to predict (a) prevalence of quit attempts among last-year smokers, (b) overall quit rates among last-year smokers and (c) mean cigarette consumption per day among current smokers. Prevalence of e-cigarette use during a quit attempt among last-year smokers was used to predict (a) quit success rate among last-year smokers and (b) overall quit rates among last-year smokers. FINDINGS: Overall quit rates increased by 0.054% [95% confidence interval (CI) = 0.032-0.076, P < 0.001] and 0.050% (95% CI = 0.031-0.069, P < 0.001) respectively for every 1% increase in the prevalence of e-cigarette use by smokers and e-cigarette use during a quit attempt. Quit success rates increased by 0.060% (95% CI = 0.043-0.078, P < 0.001) for every 1% increase in the prevalence of e-cigarette use during a quit attempt. No clear evidence was found for an association between e-cigarette use and either prevalence of quit attempt (BAdj = 0.011, 95% CI = -0.046 to 0.069, P = 0.698) or cigarette consumption (BAdj = 0.019, 95% CI = -0.043 to 0.082, P = 0.542). CONCLUSION(S): Changes in prevalence of e-cigarette use in England have been positively associated with the overall quit rates and quit success rates but not clearly associated with the prevalence of quit attempts and mean cigarette consumption.Copyright © 2019 The Authors. Addiction published by John Wiley & Sons Ltd on behalf of Society for the Study of Addiction.
3814	Begh, Rachna, Coleman, Tim, Yardley, Lucy, Barnes, Rebecca, Naughton, Felix, Gilbert, Hazel, Ferrey, Anne, Madigan, Claire, Williams, Nicola, Hamilton, Louisa, Warren, Yolanda, Grabey, Jenna, Clark, Miranda, Dickinson, Anne, Aveyard, Paul (2019) Examining the effectiveness of general practitioner and nurse promotion of electronic cigarettes versus standard care for smoking reduction and abstinence in hardcore smokers with smoking-related chronic disease: protocol for a randomised controlled trial. Trials; 20<br><br>**Keywords:**<br>Chronic Disease *Electronic Nicotine Delivery Systems *General Practitioners Humans *Nurses Outcome Assessment, Health Care Quality Assurance, Health Care *Randomized Controlled Trials as Topic Smoking/ae [Adverse Effects] *Smoking Prevention/mt [Method

	**Abstract:** BACKGROUND: Despite the clear harm associated with smoking tobacco, many people with smoking-related chronic diseases or serious mental illnesses (SMI) are unwilling or unable to stop smoking. In many cases, these smokers have tried and exhausted all methods to stop smoking and yet clinicians are repeatedly mandated to offer them during routine consultations. Providing nicotine through electronic cigarettes (e-cigarettes) may reduce the adverse health consequences associated with tobacco smoking, but these are not currently offered. The aim of this study is to examine the feasibility, acceptability and effectiveness of general practitioners (GPs) and nurses delivering a brief advice intervention on e-cigarettes and offering an e-cigarette starter pack and patient support resources compared with standard care in smokers with smoking-related chronic diseases or SMI who are unwilling to stop smoking., METHODS/DESIGN: This is an individually randomised, blinded, two-arm trial. Smokers with a smoking-related chronic condition or SMI with no intention of stopping smoking will be recruited through primary care registers. Eligible participants will be randomised to one of two groups if they decline standard care for stopping smoking: a control group who will receive no additional support beyond standard care; or an intervention group who will receive GP or nurse-led brief advice about e-cigarettes, an e-cigarette starter pack with accompanying practical support booklet, and telephone support from experienced vapers and online video tutorials. The primary outcome measures will be smoking reduction, measured through changes in cigarettes per day and 7-day point-prevalence abstinence at 2 months. Secondary outcomes include smoking reduction, 7-day point-prevalence abstinence and prolonged abstinence at 8 months. Other outcomes include patient recruitment and follow-up, patient uptake and use of e-cigarettes, nicotine intake, contamination of randomisation and practitioner adherence to the delivery of the intervention. Qualitative interviews will be conducted in a subsample of practitioners, patients and the vape team to garner their reactions to the programme., DISCUSSION: This is the first randomised controlled trial to investigate whether e-cigarette provision alongside a brief intervention delivered by practitioners leads to reduced smoking and abstinence among smokers with smoking-related chronic diseases or SMI., TRIAL REGISTRATION: ISRCTN registry, ISRCTN59404712. Registered 28/11/17.
3822	Bhatta, D. N., Glantz, S. A. (2020) Association of E-Cigarette Use With Respiratory Disease Among Adults: A Longitudinal Analysis. American Journal of Preventive Medicine; 58<br><br>**Keywords:**<br>adult aged article asthma causality chronic bronchitis chronic obstructive lung disease clinical evaluation combustion *disease association disease control emphysema female health status human incidence longitudinal study major clinical study male middl<br><br>**Abstract:** Introduction: E-cigarettes deliver an aerosol of nicotine by heating a liquid and are promoted as an alternative to combustible tobacco. This study determines the longitudinal associations between e-cigarette use and respiratory disease controlling for combustible tobacco use. Method(s): This was a longitudinal analysis of the adult Population Assessment of Tobacco and Health Waves 1, 2, and 3. Multivariable logistic regression was performed to determine the associations between e-cigarette use and respiratory disease, controlling for combustible tobacco smoking, demographic, and clinical variables. Data were collected in 2013-2016 and analyzed in 2018-2019. Result(s): Among people who did not report respiratory disease (chronic obstructive pulmonary disease, chronic bronchitis, emphysema, or asthma) at Wave 1, the longitudinal analysis revealed statistically significant associations between former e-cigarette use (AOR=1.31, 95% CI=1.07, 1.60) and current e-cigarette use (AOR=1.29, 95% CI=1.03, 1.61) at Wave 1 and having incident respiratory disease at Waves 2 or 3, controlling for combustible tobacco smoking, demographic, and clinical variables. Current combustible tobacco smoking (AOR=2.56, 95% CI=1.92, 3.41) was also significantly associated with having respiratory disease at Waves 2 or 3. Odds of developing respiratory disease for a current dual user (e-cigarette and all combustible tobacco) were 3.30 compared with a never smoker who never used e-cigarettes. Analysis controlling for cigarette smoking alone yielded similar results. Conclusion(s): Use of e-cigarettes is an independent risk factor for

	respiratory disease in addition to combustible tobacco smoking. Dual use, the most common use pattern, is riskier than using either product alone.Copyright © 2019 American Journal of Preventive Medicine
3823	Bigwanto, M., Nurmansyah, M. I., Orlan, E., Farradika, Y., Purnama, T. B. (2019) Determinants of e-cigarette use among a sample of high school students in Jakarta, Indonesia. International Journal of Adolescent Medicine and Health;<br><br>**Keywords:**<br>adolescent article controlled study cross-sectional study *electronic cigarette female health hazard *high school student human human tissue *Indonesia juvenile major clinical study male malignant neoplasm money perception *smoking teacher<br><br>**Abstract:** The aim of this study was to determine the predisposing, enabling and reinforcing factors associated with electronic cigarette (e-cigarette) use among high school students in Jakarta, Indonesia. This cross-sectional study took place in eight high schools in Jakarta, Indonesia. A total of 767 students were recruited by multistage cluster random sampling. Bivariate and multivariate statistical analyses were employed to determine the associations between socio-demographic, predisposing, reinforcing and enabling factors and e-cigarette use. Respondents were 54.1% male and the mean age was 16 years old [standard deviation (SD): 1.02]. In this sample, 32.2% of students (n = 247) had ever used e-cigarettes and 11.8% of students were e-cigarette users (n = 90). Several measured factors were positively associated with e-cigarette use, including: current smoking of conventional cigarettes [odds ratio (OR): 2.06]; perception that e-cigarettes are less addictive than conventional cigarettes (OR: 1.98); perception that e-cigarettes do not cause cancer (OR: 2.38); parental acceptance of e-cigarette use (OR: 3.80); and having enough money to buy e-cigarettes (OR: 3.24). The only variable found that was negatively associated with e-cigarette use was teacher's use of e-cigarettes (OR: 0.34). This study found that student social influences, perceptions about and accessibility to e-cigarettes were significantly positively associated with e-cigarette use among high school students in Jakarta, Indonesia. This study highlights the importance of educating students, their parents and teachers regarding safety and potential health hazards of using e-cigarettes. Efforts to implement and enforce youth access restrictions on e-cigarettes in Indonesia are crucial to preventing further uptake of these products.Copyright © 2019 Walter de Gruyter GmbH, Berlin/Boston.
3830	Blank, Mei-Ling, Hoek, Janet, George, Mark, Gendall, Philip, Conner, Tamlin S., Thrul, Johannes, Ling, Pamela M., Langlotz, Tobias (2019) An Exploration of Smoking-to-Vaping Transition Attempts Using a "Smart" Electronic Nicotine Delivery System. Nicotine & tobacco research : official journal of the Society for Research on Nicotine and Tobacco; 21<br><br>**Keywords:**<br>*Electronic Nicotine Delivery Systems Feasibility Studies Humans Self Report Smokers/sn [Statistics & Numerical Data] *Smoking Cessation/mt [Methods] *Tobacco Smoking/th [Therapy] *Vaping/th [Therapy]<br><br>**Abstract:** INTRODUCTION: Electronic nicotine delivery systems (ENDS) are used to aid smoking cessation attempts; however, many smokers continue to smoke while using an ENDS (dual use). Although uncertainty remains regarding whether specific ENDS patterns hinder or support successful smoking cessation, recent advances in "smart" technology allow passive and active recording of behaviors in real time, enabling more detailed insights into how smoking and vaping patterns may coevolve. We describe patterns of ENDS initiation, and subsequent use, including any changes in cigarette consumption, among daily smokers using a "smart" ENDS (S-ENDS) to quit smoking., METHOD: An 8-week long mixed-methods feasibility study used Bluetooth-enabled S-ENDS that passively recorded real-time device use by participants (n = 11). Daily surveys administered via smartphones collected data on self-reported cigarette consumption., RESULTS: All 11 participants were dual users, at least initially, during their quit attempt. We observed three provisional vaping and smoking patterns: immediate and intensive ENDS

	initiation coupled with immediate, dramatic, and sustained smoking reduction, leading to smoking abstinence; gradual ENDS uptake with gradual smoking reductions, leading to daily dual use throughout the study period; and ENDS experimentation with return to exclusive smoking. For six participants, the patterns observed in week 1 were similar to the vaping and smoking patterns observed throughout the rest of the study period., CONCLUSION: Technological advances now allow fine-grained description of ENDS use and smoking patterns. Larger and longer studies describing smoking-to-vaping patterns, and estimating associations with smoking outcomes, could inform ENDS-specific cessation advice promoting full transition from smoking to exclusive ENDS use., IMPLICATIONS: The use of an S-ENDS that recorded real-time device use among daily smokers engaged in a quit attempt provides insight into patterns and trajectories of dual use (continuing to smoke while using ENDS), and the possible associations between ENDS initiation, subsequent use, and smoking cessation outcomes. Such work could support more targeted cessation counseling and technical advice for smokers using ENDS to quit smoking, reduce the risk of users developing long-term dual use patterns, and enhance the contributions ENDS may make to reducing smoking prevalence. Copyright © The Author(s) 2018. Published by Oxford University Press on behalf of the Society for Research on Nicotine and Tobacco. All rights reserved. For permissions, please e-mail: journals.permissions@oup.com.
3848	Bricker, J. B., Watson, N. L., Mull, K. E., Sullivan, B. M., Heffner, J. L. (2020) Efficacy of Smartphone Applications for Smoking Cessation: A Randomized Clinical Trial. JAMA internal medicine;<br><br>**Keywords:**<br>adult article avoidance behavior cigarette smoking controlled study double blind procedure effect size electronic cigarette ethnic group female follow up human major clinical study national health organization outcome assessment practice guideline preva<br><br>**Abstract:** Importance: Smoking is a leading cause of premature death globally. Smartphone applications for smoking cessation are ubiquitous and address barriers to accessing traditional treatments, yet there is limited evidence for their efficacy. Objective(s): To determine the efficacy of a smartphone application for smoking cessation based on acceptance and commitment therapy (ACT) vs a National Cancer Institute smoking cessation application based on US clinical practice guidelines (USCPG). Design, Setting, and Participant(s): A 2-group, stratified, double-blind, individually randomized clinical trial was conducted from May 27, 2017, to September 28, 2018, among 2415 adult cigarette smokers (n=1214 for the ACT-based smoking cessation application group and n=1201 for the USCPG-based smoking cessation application group) with 3-, 6-, and 12-month postrandomization follow-up. The study was prespecified in the trial protocol. Follow-up data collection started on August 26, 2017, and ended at the last randomized participant's 12-month follow-up survey on December 23, 2019. Data were analyzed from February 25 to April 3, 2020. The primary analysis was performed on a complete-case basis, with intent-to-treat missing as smoking and multiple imputation sensitivity analyses. Intervention(s): iCanQuit, an ACT-based smoking cessation application, which taught acceptance of smoking triggers, and the National Cancer Institute QuitGuide, a USCPG-based smoking cessation application, which taught avoidance of smoking triggers. Main Outcomes and Measures: The primary outcome was self-reported 30-day point prevalence abstinence (PPA) at 12 months after randomization. Secondary outcomes were 7-day PPA at 12 months after randomization, prolonged abstinence, 30-day and 7-day PPA at 3 and 6 months after randomization, missing data imputed with multiple imputation or coded as smoking, and cessation of all tobacco products (including e-cigarettes) at 12 months after randomization. Result(s): Participants were 2415 adult cigarette smokers (1700 women [70.4%]; 1666 White individuals [69.0%] and 868 racial/ethnic minorities [35.9%]; mean [SD] age at enrollment, 38.2 [10.9] years) from all 50 US states. The 3-month follow-up data retention rate was 86.7% (2093), the 6-month retention rate was 88.4% (2136), and the 12-month retention rate was 87.2% (2107). For the primary outcome of 30-day PPA at the 12-month follow-up, iCanQuit participants had 1.49 times higher odds of quitting smoking compared with QuitGuide participants (28.2% [293 of 1040] vs 21.1% [225 of 1067]; odds ratio [OR], 1.49; 95% CI, 1.22-1.83; P<.001). Effect sizes were very similar and statistically significant for 7-day PPA at the 12-month

	follow-up (OR, 1.35; 95% CI, 1.12-1.63; P=.002), prolonged abstinence at the 12-month follow-up (OR, 2.00; 95% CI, 1.45-2.76; P<.001), abstinence from all tobacco products (including e-cigarettes) at the 12-month follow-up (OR, 1.60; 95% CI, 1.28-1.99; P<.001), 30-day PPA at 3-month follow-up (OR, 2.20; 95% CI, 1.68-2.89; P<.001), 30-day PPA at 6-month follow-up (OR, 2.03; 95% CI, 1.63-2.54; P<.001), 7-day PPA at 3-month follow-up (OR, 2.04; 95% CI, 1.64-2.54; P<.001), and 7-day PPA at 6-month follow-up (OR, 1.73; 95% CI, 1.42-2.10; P<.001). Conclusions and Relevance: This trial provides evidence that, compared with a USCPG-based smartphone application, an ACT-based smartphone application was more efficacious for quitting cigarette smoking and thus can be an impactful treatment option. Trial Registration: ClinicalTrials.gov Identifier: NCT02724462.
3852	Brossier, M., Querre, M., Regnier Denois, V. (2020) Are French adolescents ready to adopt the electronic cigarette? A qualitative study of their knowledge and representations. Journal of Public Health (Germany);<br><br>**Keywords:**<br>adolescent anthropology article *electronic cigarette female France *Frenchman human integration interview major clinical study male non-smoker peer group public health *qualitative research smoking tobacco vapor<br><br>**Abstract:** Aim: A qualitative study was carried out with the objective of documenting the experiences, knowledge, and representations of young people regarding electronic cigarettes. Subjects and methods: The growth of electronic nicotine delivery systems (ENDS), or electronic cigarettes, has prompted regulation concerns among policy makers, although little is known about the short- and long-term public health consequences of e-cigarette use. We conducted 52 individual interviews with adolescents aged 15-17 from February to July 2018. The sample included teenagers from two different regions of France, of various habitation sizes, females and males, and tobacco smokers/non-smokers. The interviews were carried out by two anthropologists. The data were collected and analyzed according to the methodology of the anchored theory. Result(s): The study demonstrated that 79% of 15-17-year-olds have admitted to already trying electronic nicotine delivery systems due to the influence of their peers, to feel integrated in the group, to enjoy the vapour and play with it. Most of the teens stopped usage after this experience. The majority of participants showed little or no knowledge of the composition of the electronic nicotine delivery systems. Many of them expressed doubts about its safety, unsure that the health effects were less harmful than those of traditional tobacco cigarettes. The adolescents also indicated that they would be more inclined to smoke tobacco compared to e-cigarettes. Conclusion(s): Young people remain convinced of the role of the traditional cigarette as a factor of social integration in their peer group.Copyright © 2020, Springer-Verlag GmbH Germany, part of Springer Nature.
3855	Brozek, G., Mateusz, J., Zejda, J., Shpakou, A., Poznanski, M., Zielonka, T., Klimatckaia, L., Loginovic, Y., Rachel, M., Gereova, J., Minarowski, L., Majek, P., Lawson, J. (2019) Attitude towards cigarette or E-cigarette use and the smoking initiation year among young European adults. European Respiratory Journal; 54<br><br>**Keywords:**<br>adolescent adult *attitude Belarus child *cigarette smoking conference abstract controlled study cross-sectional study *electronic cigarette *European female human Lithuania major clinical study male Poland preschool child prevalence questionnaire schoo<br><br>**Abstract:** The attitudes towards smoking are changing. More and more European are reaching for first cigarette under 15 years old. The aim of the study was to assess the prevalence of ever cigarette and e-cigarette usae and smoking initiation among young European adults. A cross-sectional study was carried out among students from five European countries: Belarus (BY), Lithuania (LT), Poland (PL), Russia (RU) and Slovakia (SK). A self-prepared, previously validated questionnaire focused on attitudes toward cigarettes and e-cigarette use was used. This study is a part of YoUng People E-Smoking Study (YUPESS).

	Completed questionnaires were obtained from 14,352 young adults (70.4% female), aged 20.9+/-2.4 y-old: BY:3895; LT:1128; PL: 7324; RU:1290; SK:715, the overall response rate: 72.2%. Ever cigarette use was declared by 66.1% of respondents, mean age cigarette start: 16.0 +/- 2.5 y-old. Among respondents, 43.7% had ever used e-cigarette, mean age e-smoking start: 18.2 +/- 2.2. The proportion of Europeans, who had ever used a cigarettes or e-cigarette, significantly differed (p<0.001) between the research centres. Females were less (p<0.001) likely to try either cigarette (64.7%) or e-cigarette (40.5%) compared to male (69.5% and 51.3% respectively). The average age of smoking and e-smoking initiation significantly differed (p=0.001) across Eastern and Central Europe. The earliest nicotine initiation was observed in non EU members; for cigarette in Russia: 15.3 +/- 2.6 y-old, and for e-cigarette in Belarus: 17.3 +/- 2.0. Europeans reach for cigarettes at an earlier age than e-cigarettes. Attitudes towards cigarette and e-cigarette use differ between EU and non-EU countries.
3879	Cavazos-Rehg, P., Li, X., Kasson, E., Kaiser, N., Borodovsky, J. T., Grucza, R., Chen, L. S., Bierut, L. J. (2020) Exploring how social media exposure and interactions are associated with ENDS and tobacco use in adolescents from the PATH study. Nicotine & tobacco research : official journal of the Society for Research on Nicotine and Tobacco;<br><br>**Keywords:**<br>adolescent article controlled study electronic cigarette female follow up human major clinical study male motivation public health *social media *tobacco use<br><br>**Abstract:** INTRODUCTION: Tobacco use is a current public health epidemic that puts individuals at risk for many health conditions and diseases, and adolescents are at high risk for the initiation and persistence of tobacco use behaviors partly due to engagement with social media content. The objective of this study is to examine the association between engaging in social media behaviors and patterns of electronic nicotine delivery systems (ENDS) and tobacco use at a one year follow up among 11,279 adolescents from the PATH study. METHOD(S): Five social media variables were questioned at Wave 2 and then compared to ENDS and tobacco status transitions (i.e., initiation, persistence, and escalation) at a 1-year follow up, respectively. Survey-weighted multivariable logistic regression models were used to calculate adjusted odds ratios (AOR) and 95% confidence interval (CI). RESULT(S): Passive behaviors on social media were related to higher likelihoods of starting to use ENDS and other tobacco products. Additionally, active behaviors on social media were related to higher likelihoods for the initiation and persistence of tobacco use. In particular, sending tobacco content to other users was further associated with a higher likelihood of escalation of tobacco product use. DISCUSSION: Both exposure to and interactions with social media tobacco content had a significant impact on the patterns of ENDS and tobacco use in adolescents. Due to the amount of time adolescents spend engaging with online content, social media may be a critical place in which to intervene, possibly with the use of anti-tobacco or tobacco prevention messages. IMPLICATIONS: The results of this study have implications for public health and the policies surrounding adolescents and their exposure to social media. Reducing the ENDS and tobacco content to which adolescents are exposed has the potential to decrease the instances of initiation and persistence of ENDS and tobacco use. Intervening on social media may prevent or slow the progression of ENDS and tobacco use, and increase motivation and actions toward the cessation of tobacco use in adolescents.Copyright © The Author(s) 2020. Published by Oxford University Press on behalf of the Society for Research on Nicotine and Tobacco. All rights reserved. For permissions, please e-mail: journals.permissions@oup.com.
3890	Chan, G. C. K., Stjepanovic, D., Lim, C., Sun, T., Shanmuga Anandan, A., Connor, J. P., Gartner, C., Hall, W. D., Leung, J. (2020) Gateway or common liability? A systematic review and meta-analysis of studies of adolescent e-cigarette use and future smoking initiation. Addiction (Abingdon, England);<br><br>**Keywords:**<br>adolescent female follow up human longitudinal study male meta analysis *non-smoker publication bias review sample size social evolution systematic review *vaping

	**Abstract:** BACKGROUND AND AIMS: Studies have consistently found a longitudinal association between e-cigarette use (vaping) and cigarette smoking. Many have interpreted such association as causal. This systematic review and meta-analysis evaluated the plausibility of a causal interpretation by (1) estimating the effect of adolescent vaping on smoking initiation, adjusted for study quality characteristics, (2) evaluating the sufficiency of adjustment for confounding based on the Social Development Model (SDM) and the Social Ecological Model (SEM) and E-value analyses, and (3) investigating sample attrition and publication bias. METHOD(S): Systematic review and meta-analysis of longitudinal studies that examined the association between e-cigarette use at baseline and smoking at follow-up. Participants were non-smokers aged <= 18 at baseline. RESULT(S): Meta-analysis of 11 studies showed a significant longitudinal association between vaping and smoking (adjusted OR=2.93, 95% CI [2.22,3.87]). Studies with sample sizes<1000 had a significantly higher odds ratio (OR = 6.68, 95% CI [3.63, 12.31]) than studies with sample sizes>1000 (OR = 2.49, 95% CI [1.97,3.15]). Overall, the attrition rate was very high (Median: 30%). All but one study reported results from complete sample analysis, despite those dropping out having higher risk profiles. Only two studies comprehensively adjusted for confounding. The median E-value was 2.90, indicating that the estimates were not robust against unmeasured confounding. CONCLUSION(S): There is a longitudinal association between adolescent vaping and smoking initiation; however, the evidence is limited by publication bias, high sample attrition and inadequate adjustment for potential confounders.Copyright This article is protected by copyright. All rights reserved.
3895	Chateau, G., Boykan, R., Eliscu, A., Messina, C. R. (2019) Adolescent ends and tobacco use: A preliminary analysis of an outpatient clinical cohort. Pediatrics; 144<br><br>**Keywords:**<br>adolescent adult child *cohort analysis controlled study female friend gender high risk behavior high school human major clinical study male middle school student *outpatient prevalence questionnaire self report speech tobacco use transgender *vaping ca<br><br>**Abstract:** Purpose As the prevalence of cigarette smoking among adolescents has steadily decreased in the past two decades, adolescents' use of electronic nicotine delivery systems (ENDS: electronic cigarettes, hookah, other non-combustible sources of nicotine) has increased significantly. This study describes usage patterns among adolescents in three suburban pediatric outpatient clinics, for ENDS, cigarettes and marijuana. Methods English or Spanish speaking adolescents ages 12-21 seen at three Stony Brook Children's Clinics (Long Island, NY) between 4/2017-1/2018, who provided assent/consent, completed a 60 item anonymous self-report questionnaire (adapted from the Population Assessment of Tobacco and Health Study). Demographic information and information regarding use of and exposure to ENDS, tobacco products, and marijuana were obtained. Participants received a .00 gift card incentive. Results Data obtained from 332 surveys was included in this preliminary analysis: 34.9% (n=116) of participants were male, 64.2% (n=213) female, and 0.9% identified as transgender or no gender identified (n=3). The mean age was 16.2 (SD=2.3) years with 69.5% (n=230) in grades 6-12 and 30.5% (n=101) in college, trade school or other. 12.4% (n=40) of adolescents reported smoking cigarettes (one time or more) with 4.5% reporting current use (past 30-days). 35.7% (n=112) reported using an electronic vape product at least once: of those, 70.5% vaped only once or a few times compared with 26.8% who vaped sometimes or often. Current (past 30-day) ENDS users (n=52) made up 15.6% of the sample. E-cigarette products were most commonly used (70.2%, n=40); JUUL, Bo and Smok were most frequently named. The most common reasons for trying vaping products were curiosity, use among friends or flavoring. 67.6% (n=75) of those who used or tried vaping products had friends who vaped, while only 22.6% (n = 45) of non-vaping adolescents had friends who vaped (p<0.001). Of those who used vaping products, 21.9% (n=23) reported living with individuals who also used vaping products compared to 11.3% (n=22) of those who did not use vape products (p=0.02). Co-use of other products differed among vaping (n=112) vs. non-vaping adolescents (n=200): 6.3% vs. 0.5% tried dip (p<0.002); 32.4% vs. 2% tried cigarettes (p<0.001); 65.8% vs. 11.7% tried marijuana (p<0.001), respectively. Conclusions Adolescents in

	our cohort were more likely to use ENDS than cigarettes, which is consistent with national trends. Additionally, overall prevalence of ENDS past 30-day usage was comparable to national data: per 2016 NYTS, 11.3% of high school and 4.3% of middle school students were current e-cigarette users. Several studies have shown that ENDS use leads to subsequent cigarette use. Although we cannot determine causality, ENDS users in this cohort were over 15 times more likely to try cigarettes, and over 5 times more likely to use marijuana than non-ENDS users. This high-risk behavior warrants further investigation.
3901	Cheng, H. G., Largo, E. G., Gogova, M. (2020) E-cigarette use and onset of first cigarette smoking among adolescents: An empirical test of the 'common liability' theory. F1000Research; 8<br><br>**Keywords:**<br>adolescent adolescent behavior *adolescent smoking article ethnic difference exploratory research female human male self report smoking habit *statistical analysis structural equation modeling United States *electronic cigarette *common liability theory<br><br>**Abstract:** Background: E-cigarettes have become the most commonly used tobacco products among youth in the United States (US) recently. It is not clear whether there is a causal relationship between e-cigarette use and the onset of cigarette smoking. The 'common liability'; theory postulates that the association between e-cigarette use and cigarette smoking can be attributed to a common risk construct of using tobacco products. This study aims to investigate the relationship between ever e-cigarette use and cigarette smoking onset in the US using a structural equation modeling approach guided by the 'common liability'; theory. Method(s): The study population is non-institutionalized civilian adolescents living in the US, sampled in the longitudinal Population Assessment of Tobacco and Health study. Information about tobacco product use was obtained via confidential self-report. A structural equation modeling approach was used to estimate the relationship between e-cigarette use at wave 1 and the onset of cigarette smoking at wave 2 after controlling for a latent construct representing a 'common liability to use tobacco products.'; Results: After controlling for a latent construct representing a 'common liability to use tobacco products';, ever e-cigarette use does not predict the onset of cigarette smoking (beta=0.13, 95% CI= -0.07, 0.32, p=0.204). The latent 'common liability to use tobacco products'; is a robust predictor for the onset of cigarette smoking (beta=0.38; 95% CI=0.07, 0.69; p=0.015). Conclusion(s): Findings from this study provide supportive evidence for the 'common liability' underlying observed associations between e-cigarette use and smoking onset.Copyright © 2020 Cheng HG et al.
3910	Chun, J., Yu, M., Kim, J., Kim, A. (2020) E-Cigarette, Cigarette, and Dual Use in Korean Adolescents: A Test of Problem Behavior Theory. Journal of Psychoactive Drugs; 52<br><br>**Keywords:**<br>adolescent adult alcohol consumption article child *cigarette smoking dependent variable female gender high school hopelessness human Korean (people) male mathematical variable obesity personality physical activity prevalence prevention and control *Pro<br><br>**Abstract:** E-cigarettes are becoming increasingly popular among adolescent cigarette users. However, little is known about the various factors related to adolescents' use of e-cigarettes, cigarettes, or both in South Korea. Using nationally representative data from the 2017 Korea Youth Risk Behavior Web-based Survey, this study examined the possible problem behavior theory factors that contribute to (1) current e-cigarette use among those who have ever used cigarettes (Model 1), (2) current cigarette use among those who have ever used e-cigarettes (Model 2), and (3) current dual use among current cigarette or e-cigarette users (Model 3). In all three models, our analysis showed that tobacco accessibility and sexual intercourse were significant factors. For Model 2, reasons for using e-cigarettes and alcohol use were correlated with current cigarette use. Except for gender, the significant factors, which included secondhand smoke exposure at home and school type, were the same in Models 1 and 3. Based on these findings, we encourage the South Korean government to pay close attention to potential increase in dual

	use among adolescents and to take the necessary steps in addressing adolescent e-cigarette use in tobacco prevention and control programs.Copyright © 2020, © 2020 Taylor & Francis Group, LLC.
3913	Chyderiotis, S., Benmarhnia, T., Beck, F., Spilka, S., Legleye, S. (2020) Does e-cigarette experimentation increase the transition to daily smoking among young ever-smokers in France?. Drug and Alcohol Dependence; 208<br><br>**Keywords:**<br>adolescent article controlled study female *France Frenchman human major clinical study male probability *propensity score retrospective study smoking habit *vaping<br><br>**Abstract:** Introduction: According to multiple studies, e-cigarette use among adolescents is associated with subsequent smoking initiation. However, little is known about its effect on the transition from smoking initiation to daily smoking. Method(s): Using retrospective data from a French national representative survey collected in 2017 (n = 39,115), we analyzed the role of ever using e-cigarettes on daily cigarette smoking status at 17 among ever smokers (n = 21,401). Risk-ratios (RR) were computed through modified Poisson regressions with an inverse probability of treatment weighting (IPTW) approach. Result(s): Among French adolescents aged 17, 16.8 % declared current e-cigarette use (1.9 % reported daily use) and 34.1 % cigarette smoking (25.1 % reported daily smoking). Among ever-smokers, adolescents who declared having ever used e-cigarettes were less likely than those who did not to transition to daily smoking at 17: RR = 0.62 95 %CI [0.60 - 0.64]. We found similar results for those who experimented with e-cigarettes before initiating smoking, RR = 0.76 95 %CI [0.66 - 0.89]. Conclusion(s): Our results found no evidence of an increased risk of transitioning to daily smoking at 17 among ever-smokers who also experimented with e-cigarettes. Further studies should investigate the longer-term role of vaping on future smoking habits with the use of causal inference methods.Copyright © 2020
3917	Conner, M., Grogan, S., Simms-Ellis, R., Flett, K., Sykes-Muskett, B., Cowap, L., Lawton, R., Armitage, C., Meads, D., Schmitt, L., Torgerson, C., West, R., Siddiqi, K. (2020) Evidence that an intervention weakens the relationship between adolescent electronic cigarette use and tobacco smoking: a 24-month prospective study. Tobacco control; 29<br><br>**Keywords:**<br>adolescent article attention controlled study *electronic cigarette female follow up friend *harm reduction human major clinical study male *prospective study randomized controlled trial self report *smoking<br><br>**Abstract:** BACKGROUND: The electronic cigarette (e-cigarette) use to subsequent smoking relationship in adolescents has received much attention. Whether an intervention to reduce smoking initiation attenuated this relationship was assessed. METHOD(S): Data were from 3994 adolescent never smokers (aged 13-14 years at baseline) as part of a cluster randomised controlled trial. Self-report measures of smoking, e-cigarette use and covariates were assessed and used to predict ever smoked cigarettes, any recent tobacco smoking and regularly smoked cigarettes at 24-month follow-up. RESULT(S): Baseline ever use of e-cigarettes was associated with ever smoked cigarettes (OR=4.03, 95% CI 3.33 to 4.88; controlling for covariates, OR=2.78, 95% CI 2.20 to 3.51), any recent tobacco smoking (OR=3.38, 95% CI 2.72 to 4.21; controlling for covariates, OR=2.17, 95% CI 1.76 to 2.69) and regularly smoked cigarettes (OR=3.60, 95% CI 2.35 to 5.51; controlling for covariates, OR=1.27, 95% CI 1.17 to 1.39) at follow-up. For ever smoked cigarettes only, the impact of e-cigarette use was attenuated in the intervention (OR=1.83) compared with control (OR=4.53) condition. For ever smoked cigarettes and any recent tobacco smoking, the impact of e-cigarette use was attenuated among those with friends who smoked (OR=2.05 (ever smoked); 1.53 (any tobacco use)) compared with those without friends who smoked (OR=3.32 (ever smoked); 2.17 (any tobacco use)). CONCLUSION(S): This is one of the first studies to show that e-cigarette use was robustly associated with measures of smoking over 24 months and the first to show an intervention to attenuate the relationship. Further research with a broader age range of adolescents is

	required.Copyright © Author(s) (or their employer(s)) 2020. Re-use permitted under CC BY. Published by BMJ.
3935	Dai, Hongying (2019) Changes in Flavored Tobacco Product Use Among Current Youth Tobacco Users in the United States, 2014-2017. JAMA pediatrics; 173<br><br>**Keywords:**<br>Adolescent Cross-Sectional Studies *Electronic Nicotine Delivery Systems/sn [Statistics & Numerical Data] Female *Flavoring Agents/pd [Pharmacology] Follow-Up Studies Humans Incidence Male Prevalence Retrospective Studies Schools *Self Report Students *<br><br>**Abstract:**
3953	Dobbs, P. D., Hodges, E. J., Dunlap, C. M., Cheney, M. K. (2020) Addiction vs. dependence: A mixed methods analysis of young adult JUUL users. Addictive Behaviors; 107<br><br>**Keywords:**<br>adult alcoholism article cannabis addiction college student controlled study cross-sectional study *drug dependence female human information processing interview logistic regression analysis major clinical study male online system qualitative analysis s<br><br>**Abstract:** Objective: JUUL-brand electronic cigarettes (e-cigarettes) contain higher levels of nicotine than other e-cigarettes. The current study examined the relationship between young adults' self-reported addiction and measured JUUL dependence. Method(s): This study used a sequential, explanatory mixed methods design. Data were collected from a large, southwestern college in the US. College students who were weekly JUUL users and owned a JUUL device (n = 595) completed a cross-sectional online survey, then a subsample (n = 51) participated in in-person interviews. All data were collected between March-April 2019. T-tests were used to examine differences between JUUL dependence and self-reported addiction, and logistic regressions were employed to examine factors that may influence self-reported addiction to JUUL, nicotine, and other e-cigarettes. Interviews were independently coded by 2 coders (kappa ranged from 0.46 to 0.99) then analyzed for corresponding themes. Result(s): Participants who had a higher JUUL dependence were more likely to report addiction to several substances/products, including nicotine, JUULs, cigarettes, other e-cigarettes, alcohol, and marijuana. Interview participants minimized JUUL addiction by comparing JUUL to other socially acceptable addictions and describing JUUL addiction as a habit. Interview participants suggested addiction to nicotine was more stigmatizing than JUUL. The number of days participants used JUUL per week, use of traditional tobacco products, and measured JUUL dependence were each associated with self-reported JUUL addiction. Conclusion(s): Social construction of self-reported addictions may be changing young audiences' views of addiction to JUUL. Research is needed to see if using the phrase 'nicotine dependence' rather than 'JUUL addiction' influences young adults' JUUL use.Copyright © 2020 Elsevier Ltd
3973	Elton-Marshall, T., Driezen, P., Fong, G. T., Cummings, K. M., Persoskie, A., Wackowski, O., Choi, K., Kaufman, A., Strong, D., Gravely, S., Taylor, K., Kwan, J., Bansal-Travers, M., Travers, M., Hyland, A. (2020) Adult perceptions of the relative harm of tobacco products and subsequent tobacco product use: Longitudinal findings from waves 1 and 2 of the population assessment of tobacco and health (PATH) study. Addictive Behaviors; 106<br><br>**Keywords:**<br>adult age article cigar smoking cohort analysis controlled study demography female *health hazard human longitudinal study male *perception prediction *tobacco *tobacco use vaping water pipe smoking<br><br>**Abstract:** Objectives: To examine: (1) How perceptions of harm for seven non-cigarette tobacco products predict subsequent use; (2) How change in use is associated with changes in perceptions of product

	harm; (3) Whether sociodemographic variables moderate the association between perceptions and use. Method(s): Data are from the adult sample (18+) of the Population Assessment of Tobacco and Health (PATH) Study, a nationally representative longitudinal cohort survey conducted September 2013-December 2014 (Wave 1 (W1) n = 32,320) and October 2014-October 2015 (Wave 2 (W2) n = 28,362). Result(s): Wave 1 users and non-users of e-cigarettes, filtered cigars, cigarillos, and pipes, who perceived these products as less harmful had greater odds of using the product at W2. For the other products, there was an interaction between W1 perceived harm and W1 use status in predicting W2 product use. At W2, a smaller percentage of U.S. adults rated e-cigarettes as less harmful than cigarettes compared to W1 (41.2% W1, 29.0% W2). Believing non-cigarette products to be less harmful than cigarettes was more strongly associated with subsequent product use in the oldest age group (55+ years) while weaker effects were observed in the youngest age group (18-24 years). This moderating effect of age was significant for e-cigarettes, hookah, traditional cigars, and cigarillos. Conclusion(s): Strategies to prevent initiation and promote cessation of these products may benefit from understanding and addressing perceptions of these products.Copyright © 2020 Elsevier Ltd
3983	Evans-Polce, R. J., Veliz, P. T., Boyd, C. J., McCabe, S. E. (2020) E-Cigarette and Cigarette Use Among U.S. Adolescents: Longitudinal Associations With Marijuana Use and Perceptions. American Journal of Preventive Medicine; 58<br><br>**Keywords:**<br>adolescent *adolescent smoking article binge drinking *cannabis use *cigarette smoking cohort analysis controlled study cross-sectional study female health hazard human longitudinal study male *perception prospective study risk assessment United States<br><br>**Abstract:** Introduction: Researchers have shown a connection between e-cigarette use and subsequent cigarette use. This study prospectively examines the connection between e-cigarette use and marijuana use. Method(s): This study used data from the Monitoring the Future panel study of 12th graders in 2014 who were followed up 1 year later (n=305). Past-30-day marijuana use and perceived riskiness of marijuana use for 12th graders who used neither e-cigarettes nor cigarettes (78.6%), only e-cigarettes (10.3%), and cigarettes (with or without e-cigarettes; 11.1%) were compared. Data was analyzed in 2019. Result(s): At baseline, 40.1% of e-cigarette only users, 48.8% of cigarette users, and 13.2% of nonusers reported past-30-day marijuana use at baseline. E-cigarette only users were less likely to perceive any marijuana as risky at follow-up than nonusers (AOR=0.15, 95% CI=0.04, 0.65). Similarly, e-cigarette only users were more likely to report past-30-day marijuana use at follow-up than nonusers (AOR=3.82, 95% CI=1.45, 10.04), as were those who used cigarettes (AOR=7.63, 95% CI=2.65, 21.97). Conclusion(s): E-cigarette use, even when not in conjunction with cigarette use, may be a marker of marijuana use risk during the transition from adolescence to young adulthood. The e-cigarette and marijuana use link may strengthen in the future with the increasing trend of adolescents vaping marijuana.Copyright © 2020 American Journal of Preventive Medicine
3999	Firth, C., LaBrie, J. W., D'Amico, E. J., Klein, D. J., Griffin, B. A., Pedersen, E. R. (2020) Changes in Cigarette, E-Cigarette, and Cannabis Use among U.S. College Students Studying Abroad. Substance use & misuse; 55<br><br>**Keywords:**<br>adult article *cannabis use *cigarette smoking *college student controlled study demography *electronic cigarette human indigenous people major clinical study male multiple drug abuse prevalence *smoking young adult *cannabis *nicotine<br><br>**Abstract:** Background: Study abroad experiences among U.S. college students are risky environments for alcohol use; however little work has examined how studying abroad may affect other substance use. Purpose(s): This study assesses changes in frequency of cigarette, e-cigarette, and cannabis use among students studying abroad. Method(s): We recruited study abroad students from 63 institutions who studied in the 12 most popular destinations; 2,086 students completed surveys before leaving the U.S.

	and during their last month abroad. Descriptive and multivariate mixed-effects regression analysis compared substance use by location between students and country natives using publicly available data, identified behavioral and demographic predictors of frequency of substance use abroad, and estimated average adjusted prevalence of substance use by location. Result(s): Students' cigarette smoking increased three-fold while abroad; patterns of use varied significantly by location and mirrored smoking behaviors of native young adults. Rates of e-cigarette use declined while abroad and rates of cannabis use were similar abroad to pre-departure rates. However, cannabis use varied substantially by location and were higher than native peer rates. For all substances, most frequent substance use abroad was reported among males and those with prior histories of single drug and polydrug use. Conclusion(s): Findings highlight the importance of continued research on drug use abroad among U.S. college students and pave the way for initial prevention and intervention efforts to target drug use abroad, particularly for cigarettes due to the negative health consequences of use and for cannabis given the potential legal ramification of use in non-legalized countries.
4001	Ford, E. W., Chan, K. S., Parikh, M., Lowe, K. B., Huerta, T. R. (2020) E-cigarette and hookah adoption patterns: Is the harm reduction theory just so much smoke?. Addictive Behaviors Reports; 11<br><br>**Keywords:**<br>adult aged article cohort analysis consumer attitude female Food and Drug Administration groups by age *harm reduction health survey human male middle aged priority journal smoking cessation smoking habit snus theory tobacco use United States *waterpipe<br><br>**Abstract:** Aims: This paper explores inter-generational changes in consumers smoking product uptake and use patterns after the introduction of e-cigarettes and hookahs. Design(s): Item Response Theory (IRT) is used to analyze the Health Information National Trends Survey sponsored by the Food and Drug Administration (HINTS-FDA). The survey was fielded in 2015. IRT allows the pattern of product use to be described and help assess whether the new tobacco products (i.e., e-cigarettes, hookahs) serve as gateway to other products or act in harm reduction modality. Finding(s): The results indicate that the new product alternatives have changed the how tobacco products are adopted in the U.S. In particular, younger respondents were more likely to have engaged in cigar, e-cigarette and water-pipe use than the older cohort. Conclusion(s): The introduction of nicotine products previously unavailable in the U.S is creating new modes for smoking initiation in the age groups most likely to begin a new habit. There is little evidence that smokers in the older HINTS cohorts are using the e-cigarette as a smoking cessation tool. The rise of cigar use in the younger cohort may indicate that legal products are being mixed with illicit substances (i.e., 'blunting').Copyright © 2020
4002	Foxon, F., Selya, A. S. (2020) Electronic Cigarettes, Nicotine Use Trends, and Use Initiation Ages among US Adolescents from 1999-2018. Addiction (Abingdon, England);<br><br>**Keywords:**<br>adolescent article child cigarette smoking controlled study *electronic cigarette female human juvenile male observational study prevalence school child smoking *nicotine<br><br>**Abstract:** BACKGROUND AND AIMS: Recent nicotine use trends raise concerns that electronic cigarettes (ECs) may act as a gateway to cigarettes among adolescents. The aims of this study were to examine prevalence trends of exclusive EC use, exclusive cigarette use, and dual use, and to determine the corresponding ages of initiation, and investigate hypothetical trends in total nicotine use and cigarette use in the absence of ECs among US adolescents. DESIGN: Observational study using data from the National Youth Tobacco Survey (NYTS) to statistically model trends in the prevalences of each user group and their initiation ages. Projections from counterfactual models based on data from 1999-2009 (before EC introduction) were compared with actual trends based on data from 1999-2018. Rigorous error analyses were applied, including Theil proportions.USA. PARTICIPANTS AND MEASUREMENTS: Adolescents aged 12-17 years who were established exclusive cigarette users (>=100 cigarettes smoked

	AND <=100 days vaped), established exclusive EC users (<100 cigarettes smoked AND >100 days vaped), and established dual users (>=100 cigarettes smoked AND >100 days vaped), based on cumulative lifetime exposure (N=12,500-31,000 per wave). FINDINGS: Exclusive cigarette use prevalence declined from 1999-2018, while exclusive EC use and dual use prevalences increased since their introduction in 2009. The age of cigarette initiation began a slight increase after 2014, whereas the age for EC use remained approximately constant and was higher than that of cigarettes. The counterfactual comparison results were consistent with ECs not increasing the number of US adolescent nicotine users, and in fact diverting adolescents from cigarettes. CONCLUSION(S): Electronic cigarettes may have offset conventional smoking among US adolescents between 2010-2018 by maintaining the total nicotine use prevalence and diverting them from more harmful conventional smoking. Additionally, electronic cigarette users appear to initiate at older ages relative to conventional smokers, which is associated with lower risk.Copyright This article is protected by copyright. All rights reserved.
4004	Friedman, A. S., Xu, S. (2020) Associations of Flavored e-Cigarette Uptake with Subsequent Smoking Initiation and Cessation. JAMA Network Open; 3<br><br>**Keywords:**<br>adolescent adult article child cohort analysis controlled study data analysis female flavor human human tissue juvenile major clinical study male *people by smoking status smoking cessation tobacco *vaping young adult<br><br>**Abstract:** Importance: Several states have banned sales of flavored e-cigarettes, but evidence on the association between vaping flavors and subsequent smoking initiation and cessation is limited. Objective(s): To evaluate whether new uptake of flavored e-cigarettes is more strongly associated with subsequent smoking initiation and cessation than uptake of unflavored e-cigarettes, separately for youths (12-17 years), emerging adults (18-24 years), and prime-age adults (25-54 years). Design, Setting, and Participant(s): This cohort study conducted secondary data analyses of longitudinal survey data from waves 1 to 4 of the Population Assessment of Tobacco and Health Study (collected from 2013 to 2018). The analytic sample was limited to 17929 respondents aged 12 to 54 years at wave 1 who completed at least 3 consecutive waves of the survey and did not use e-cigarettes at baseline. Data were collected from 2013 to 2018 and analyzed in February 2020. Exposures: Flavored vs unflavored e-cigarette use reported in wave 2 of the Population Assessment of Tobacco and Health Study. Main Outcomes and Measures: Binary indicators captured wave 3 smoking among 7311 youths and 4634 emerging adults who did not smoke at baseline (ie, initiation) and not smoking at wave 3 among 1503 emerging adults and 4481 prime-age adults who smoked at baseline (ie, cessation). Smoking status was based on having smoked in the past 30 days for youths and established smoking (ie, current smoking among those who smoked at least 100 cigarettes in their lifetime) for emerging and prime-age adults. Result(s): The youths who did not smoke at baseline, emerging adults who smoked at baseline, and prime-age adults who smoked at baseline consisted of 51.4% to 58.0% male participants and 66.9% to 77.0% white individuals. Vaping uptake was positively associated with smoking initiation in youth (adjusted odds ratio [AOR], 6.75; 95% CI, 3.93-11.57; P <.001) and in emerging adults (AOR, 3.20; 95% CI, 1.70-6.02; P <.001). Vaping uptake was associated with cessation in adults (AOR, 1.34; 95% CI, 1.02-1.75; P =.03). Vaping nontobacco flavors was no more associated with youth smoking initiation than vaping tobacco-flavors (AOR in youth, 0.66; 95% CI, 0.16-2.76; P =.56) but was associated with increased adult smoking cessation (AOR in adults, 2.28; 95% CI, 1.04-5.01; P =.04). Conclusions and Relevance: In this study, adults who began vaping nontobacco-flavored e-cigarettes were more likely to quit smoking than those who vaped tobacco flavors. More research is needed to establish the relationship between e-cigarette flavors and smoking and to guide related policy..Copyright © 2020 JAMA Network Open. All rights reserved.
4014	Gao, W., Sanna, M., Chuluunbaatar, E., Tsai, M. K., Levy, D. T., Wen, C. P. (2020) Are e-cigarettes reviving the popularity of conventional smoking among Taiwanese male adolescents? A time-trend population-based analysis for 2004-2017. Tobacco control;

	**Keywords:**<br>adolescent adult article *cigarette smoking controlled study *electronic cigarette *global health high school student human juvenile male prevalence *Taiwanese time series analysis *nicotine<br><br>**Abstract:** INTRODUCTION: In Taiwan, national tobacco use surveys show that e-cigarette use has increased since 2014 among youth, while, at the same time, conventional cigarette smoking has continuously decreased. The purpose of this study is to examine whether the increased popularity of e-cigarettes has undermined this favourable declining trend for cigarette smoking. METHOD(S): We examined conventional cigarette and e-cigarette prevalence among male high school students (aged 16-18 years) and adults from 2004 to 2017, using data from cross-sectional nationally representative surveys. Applying interrupted time series analysis, we assessed whether there was a change in trend in 2014, when e-cigarette use started to gain popularity from long-term trends in prior years (2004-2013). RESULT(S): E-cigarette use prevalence increased from 2.5% in 2014 to 6.4% in 2017 among male high school students but was negligible among male adults, declining from 1.4% in 2015 to 0.8% in 2017. The annual relative decline in the cigarette smoking rate after e-cigarettes started to gain popularity was greater (-10%) than the long-term trend (-2%) among high school students. Among adults, the change in trend over time after e-cigarettes started to gain popularity was not significant (ie, not significantly different from 0). CONCLUSION(S): The increased popularity of e-cigarettes since 2014 is associated with a greater decline in youth smoking, compared with previous years. On the contrary, e-cigarette use has remained very low among Taiwanese male adults and no additional impact on the conventional smoking trend is found.Copyright © Author(s) (or their employer(s)) 2020. No commercial re-use. See rights and permissions. Published by BMJ.
4027	Gilbert, P. A., Kava, C. M., Afifi, R. (2020) High school students rarely use e-cigarettes alone: A socio-demographic analysis of poly-substance use among adolescents in the USA. Nicotine & tobacco research : official journal of the Society for Research on Nicotine and Tobacco;<br><br>**Keywords:**<br>adolescent article behavioral risk factor surveillance system bisexuality controlled study *demography drug combination ethnic group female *high school student human *juvenile major clinical study male population health prevalence risk assessment *vapi<br><br>**Abstract:** INTRODUCTION: Most adolescents reporting e-cigarette use have also used combustible tobacco; however, the extent to which they use other substances is less clear. This study assessed e-cigarette use with tobacco, alcohol, or cannabis and quantified the risk of poly-substance use among adolescents overall and by socio-demographic characteristics. METHOD(S): Using 2017 Youth Behavioral Risk Factor Surveillance System data from adolescents (grades 9-12) with complete substance use information (n=11,244), we examined e-cigarette poly-use status (none [referent]; e-cigarettes only; or e-cigarettes + other substances). We estimated prevalence of substance use and modeled odds of e-cigarette use, alone or with other substances, by several socio-demographic characteristics. Analyses were completed in Stata version 15.1 using survey procedures to account for the complex survey design. RESULT(S): Approximately 12% of adolescents reported past 30-day e-cigarette use. Almost all (93%) e-cigarette users also reported other substance use; alcohol appeared most frequently in combinations. Odds of e-cigarette single use and e-cigarette poly-use (vs. no use) were higher for males and adolescents with lower grades (ORs 1.44-2.31). Racial/ethnic minorities had lower odds of e-cigarette poly-use than White peers (ORs 0.18-0.61), and bisexual (vs. straight) adolescents were more likely to be e-cigarette poly-users (OR=1.62). E-cigarette use increased from 9th grade (7%) to 12th grade (16%). CONCLUSION(S): Poly-substance use is highly prevalent among adolescents who use e-cigarettes. Therefore, e-cigarette screening should include assessment of other substances, especially alcohol. Early and comprehensive prevention efforts to reduce e-cigarette and other substance use could have a substantial beneficial impact on population health over time.Copyright © The Author(s) 2020. Published

	by Oxford University Press on behalf of the Society for Research on Nicotine and Tobacco. All rights reserved. For permissions, please e-mail: journals.permissions@oup.com.
4041	Gravely, S., Cummings, K. M., Hammond, D., Lindblom, E., Smith, D. M., Martin, N., Loewen, R., Borland, R., Hyland, A., Thompson, M. E., Boudreau, C., Kasza, K., Ouimet, J., Quah, A. C. K., O'Connor, R., Fong, G. T. (2020) The association of e-cigarette flavors with satisfaction, enjoyment, and trying to quit or stay abstinent from smoking among regular adult vapers from Canada and the United States: Findings from the 2018 ITC Four Country Smoking and Vaping Survey. Nicotine & tobacco research : official journal of the Society for Research on Nicotine and Tobacco;<br><br>**Keywords:**<br>adult article *Canada candy coffee controlled study cross-sectional study *current smoker drug therapy *ex-smoker female *flavor fruit human major clinical study male *satisfaction smoking cessation *United States *vaping menthol<br><br>**Abstract:** INTRODUCTION: This study examined whether non-tobacco flavors are more commonly used by vapers (e-cigarette users) compared to tobacco flavor, described which flavors are most popular, and tested whether flavors are associated with: vaping satisfaction relative to smoking, level of enjoyment with vaping, reasons for using e-cigarettes, and making an attempt to quit smoking by smokers. METHOD(S): This cross sectional study included 1,603 adults from Canada and the US who vaped at least weekly, and were either current smokers (concurrent users) or former smokers (exclusive vapers). Respondents were categorized into one of seven flavors they used most in the last month: tobacco, tobacco-menthol, unflavored, or one of the non-tobacco flavors: menthol/mint, fruit, candy, or 'other' (e.g. coffee). RESULT(S): Vapers use a wide range of flavors, with 63.1% using a non-tobacco flavor. The most common flavor categories were fruit (29.4%) and tobacco (28.7%), followed by mint/menthol (14.4%) and candy (13.5%). Vapers using candy (41.0%, p<0.0001) or fruit flavors (26.0%, p=0.01) found vaping more satisfying (compared to smoking) than vapers using tobacco flavor (15.5%) and rated vaping as very/extremely enjoyable (fruit: 50.9%; candy: 60.9%) than those using tobacco flavor (39.4%). Among concurrent users, those using fruit (74.6%, p=0.04) or candy flavors (81.1%, p=0.003) were more likely than tobacco flavor users (63.5%) to vape in order to quit smoking. Flavor category was not associated with the likelihood of a quit attempt (p=0.46). Among exclusive vapers, tobacco and non-tobacco flavors were popular; however, those using tobacco (99.0%) were more likely than those using candy (72.8%, p=0.002) or unflavored (42.5%, p=0.005) to vape in order to stay quit. CONCLUSION(S): A majority of regular vapers in Canada and the US use non-tobacco flavors. Greater satisfaction and enjoyment with vaping is higher among fruit and candy flavor users. While it does not appear that certain flavors are associated with a greater propensity to attempt to quit smoking among concurrent users, non-tobacco flavors are popular among former smokers who are exclusively vaping. Future research should determine the likely impact of flavor bans on those who are vaping to quit smoking or to stay quit.Copyright © The Author(s) 2020. Published by Oxford University Press on behalf of the Society for Research on Nicotine and Tobacco.
4043	Gueorguieva, R., Buta, E., Simon, P., Krishnan-Sarin, S., O'Malley, S. S. (2020) Data visualization tools of tobacco product use patterns, transitions and sex differences in the PATH youth data. Nicotine & tobacco research : official journal of the Society for Research on Nicotine and Tobacco;<br><br>**Keywords:**<br>adolescent article child controlled study *data visualization *electronic cigarette female *heat *hookah human major clinical study male *sex difference *tobacco use<br><br>**Abstract:** INTRODUCTION: Evaluations of multiple tobacco product use and temporal changes in patterns of use are complicated by the large number of combinations and transitions. Visualization tools could easily identify most common patterns and transitions. METHOD(S): Set intersection bar plots describe ever use of five tobacco products among 12-17 years old youth in Wave 1 of the Population Assessment

	of Tobacco and Health (PATH) study (N=11497). Heat maps visualize unweighted frequencies of transitions from ever use at Wave 1 (2013-2014) to past 12-month use at Wave 2 (2014-2015). Weighted calibrated heat maps assess differences in relative frequencies of transitions by pattern at Wave 1 and identify differences in transitions by sex. RESULT(S): The most common tobacco product ever use patterns in Wave 1 were of cigarettes only, e-cigarettes only or hookah only, followed by ever use of both cigarettes and e-cigarettes. Initiation of use between Waves was uncommon. The most frequent transition among those who reported use at Wave 2 but not at Wave 1 (N=971) was to e-cigarette use (N=301). However, among e-cigarette-only ever users at Wave 1 (N=260), about half did not report any product use at Wave 2. Use of three or more products remained stable. Adolescent girls compared to boys appeared more likely to report hookah use at both Waves. CONCLUSION(S): Set intersection bar plots and heat maps are useful for visualizing tobacco product use patterns and transitions, especially for multiple products. Both techniques could identify common problematic tobacco use patterns across and within populations.Copyright © The Author(s) 2020. Published by Oxford University Press on behalf of the Society for Research on Nicotine and Tobacco. All rights reserved. For permissions, please e-mail: journals.permissions@oup.com.
4051	Hair, E. C., Barton, A. A., Perks, S. N., Kreslake, J., Xiao, H., Pitzer, L., Leventhal, A. M., Vallone, D. M. (2021) Association between e-cigarette use and future combustible cigarette use: Evidence from a prospective cohort of youth and young adults, 2017-2019. Addictive Behaviors; 112<br><br>**Keywords:**<br>adult age distribution article *cigarette smoking cohort analysis controlled study female human male prospective study sensation seeking sex difference smoking cessation social psychology tobacco use young adult *electronic cigarette<br><br>**Abstract:** Introduction: A surge in popularity of e-cigarettes prompts concern given the association between e-cigarettes and future cigarette use. However, much of the evidence for this association comes from early, less efficient, and lower nicotine e-cigarettes than are available and widely used now. The goal of this study was to examine the relationship between e-cigarette use in 2018 and subsequent smoking initiation and continued e-cigarette use. Method(s): Participants included members of a national longitudinal panel of youth and young adults aged 15-27 who, in 2017, reported never having used a nicotine containing product (n = 3360). Logistic regression analyses assessed associations between participants' self-reported ever e-cigarette use in 2018 and ever cigarette use, current cigarette use, and current e-cigarette use in 2019, after controlling for demographic and psychosocial variables. Result(s): Compared with those who still had never used an e-cigarette, those who reported ever e-cigarette use in 2018 had significantly higher odds of ever cigarette use (aOR = 7.29, 95% CI [4.10, 12.97]), current cigarette use (aOR = 8.26, 95% CI [3.17, 21.53]), and current e-cigarette use (aOR = 9.70, 95% CI [6.41, 14.69]) one year later in 2019. Conclusion(s): These findings show that the pod mod style, high nicotine containing e-cigarettes subject young users to the same risks of transitioning to combustible cigarettes as their earlier, less efficient predecessors. Strong regulation of all nicotine products, including e-cigarettes, is needed to prevent the trajectory of e-cigarette to cigarette use among youth and young adults.Copyright © 2020 Elsevier Ltd
4059	Hansen, J., Hanewinkel, R., Morgenstern, M. (2020) Electronic cigarette advertising and teen smoking initiation. Addictive Behaviors; 103<br><br>**Keywords:**<br>adolescent *adolescent smoking *advertising article child controlled study environmental exposure environmental factor female follow up human logistic regression analysis longitudinal study major clinical study male migration *onset age peer pressure pr<br><br>**Abstract:** Introduction: The objective of this study was to investigate the associations between recall of exposure to e-cigarette advertisements and initial use of e-cigarettes, conventional cigarettes and

	hookahs one year later among German adolescents. Method(s): Longitudinal school-based survey with a sample of 4,529 German adolescents (mean age = 12.5 years, SD = 1.55). Baseline assessment took place in the fall/winter 2016/2017, and a follow-up assessment 12 months later. Exposure to e-cigarette advertisements was measured at baseline with self-rated contact frequency to three advertising images. Multilevel mixed-effect logistic regression models were used to assess associations between exposure to e-cigarette advertisements at baseline and adolescents' initiation of e-cigarette, smoking and hookah use one year later. Result(s): About 14% (N = 472) baseline never-users initiated e-cigarette use within one year, about 11% (N = 384) initiated cigarette use, and 12% (N = 406) used a hookah for the first time within the observation period. After statistical control for age, gender, school type, subjective socioeconomic status, sensation seeking, lifetime smoking behavior and peer substance use, adolescents with higher contact to e-cigarette advertisements had higher proportion of subsequent e-cigarette (aOR = 1.37 (CI = 1.04-1.81) p =.024), cigarette (aOR = 1.44 (CI = 1.09-1.91) p =.010), and hookah use (aOR = 1.82 (CI = 1.37-2.42) p <.001). Conclusion(s): This longitudinal study demonstrates that exposure to e-cigarette advertisements may increase the likelihood of initial use of e-cigarettes, cigarettes, and hookahs. Findings raise concerns about e-cigarette marketing regulations in Germany, and about the broader impact of e-cigarette advertising on traditional smoking.Copyright © 2019 Elsevier Ltd
4066	Hawkins, Summer Sherburne, Ghiani, Marco, Baum, Christopher F. (2019) Associations Between State Tobacco Control Policies and Adolescent ENDS Use. Journal of public health management and practice : JPHMP; 25<br><br>**Keywords:**<br>Adolescent Female *Health Policy Humans Male Smoking/ep [Epidemiology] Smoking/lj [Legislation & Jurisprudence] Smoking Prevention/lj [Legislation & Jurisprudence] *State Government United States Vaping/ep [Epidemiology] Vaping/lj [Legislation & Jurispr<br><br>**Abstract:** BACKGROUND: Despite an increase in adolescent use of electronic nicotine delivery systems (ENDS), little is known about the role of tobacco control policies on ENDS use., OBJECTIVE: For aim 1, we examined how trends in adolescent use of cigarettes were affected by the introduction of ENDS; for aim 2, we examined the associations between ENDS age restrictions, cigarette taxes, and smoke-free legislation and adolescent use of ENDS and cigarettes., DESIGN AND SETTING: Repeated cross-sections of the 1999-2015 Youth Risk Behavior Surveys linked to state-level tobacco control policies., PARTICIPANTS: 938 486 adolescents aged 14 to 18 years from 45 states., MAIN OUTCOME MEASURES: For aim 1, we examined adolescent use of cigarettes. For aim 2, we examined adolescent use of ENDS, only ENDS, and cigarettes., RESULTS: We found there was an overall decreasing trend in adolescent use of cigarettes, but the actual decline was greater than the predicted decline for 17- and 18-year-olds. While we found no associations between ENDS use and ENDS age restrictions or cigarette taxes, ENDS use was 3.8 percentage points higher in those states with smoke-free legislation for combustible tobacco products., CONCLUSIONS: Our findings highlight that ENDS age restrictions may not be adequate to curb ENDS use and additional local- and state-level policies governing ENDS are needed.
4069	Hedman, L., Backman, H., Stridsman, C., Lindberg, A., Lundback, B., Ronmark, E. (2019) E-cigarette use is most common in persistent smokers in a Swedish prospective population study. European Respiratory Journal; 54<br><br>**Keywords:**<br>adult age chronic obstructive lung disease cohort analysis conference abstract controlled study *electronic cigarette ex-smoker female follow up human major clinical study male occupation physical disease by body function *population research *prospecti<br><br>**Abstract:** Background: Prospective studies are needed to study e-cigarette use in relation to smoking habits in the general population. Aim(s): to study predictors of e-cigarette use among adults in a population-based cohort study. Method(s): The starting point of a prospective cohort study within the

	Obstructive Lung Disease in Northern Sweden (OLIN) studies was a postal questionnaire (PQ1) survey of a random sample of the population (age 20-69y, 51% women) in 2006, n=10414 (76% of invited). The cohort was followed-up in 2016 and n=8424 participated (PQ2). Both PQs included questions on respiratory symptoms, age, sex, occupation and smoking, and the 2016 PQ also included e-cigarette use. Result(s): At follow-up, 0.9% (n=68) used e-cigarettes, with identical rates in men and women (n=34 0.9%). Based on smoking habits at PQ1, e-cigarette use was more common in smokers (4.1%) than former smokers (0.3%) and nonsmokers (0.1%), p<0.001. In a regression analysis adjusted for sex and socioeconomic status, e-cigarette use was related to smoking in PQ1 (OR 32.5 95% CI 13.7-76.9), and age-group 20-29y (OR 3.3 95% CI 1.1-10.0). Among ecigarette users 82% were persistent smokers (both PQ1 and PQ2). Of the n=225 that quit smoking between PQ1 and PQ2, only one individual (0.4%) used e-cigarettes, while 6.4% used e-cigarettes among persistent smokers. The majority of dual users at PQ2 (68.9%) smoked the same number of cig/day as at PQ1, while 20.0% decreased and 11.1% increased the number of cig/day. Conclusion(s): E-cigarette use among Swedish adults was associated with young age and persistent smoking, but not smoking cessation.
4094	Hwang, J. E., Cho, S. I. (2020) The association between new graphic health warning labels on tobacco products and attitudes toward smoking among south Korean adolescents: a national cross-sectional study. BMC public health; 20<br><br>**Keywords:**<br>adolescence adolescent adult advertising article *attitude awareness child childhood controlled study *cross-sectional study *education electronic cigarette female government high school student human major clinical study male *passive smoking perceptio<br><br>**Abstract:** BACKGROUND: Graphic health warning labels (GHWLs) on tobacco products are more effective than text warnings for communicating the risk of smoking. The implementation of GHWLs can prevent adolescents from initiating smoking. Therefore, this study examined the association between GHWLs newly implemented on December 23, 2016, in South Korea and attitudes toward smoking among adolescents. METHOD(S): This post-implementation cross-sectional analysis examined the responses of 62,276 students (31,624 boys and 30,652 girls) who participated in the 2017 Web-based Korean Youth Risk Behavior Survey, which was completed anonymously as a self-administered questionnaire by middle and high school students. Multinomial logistic regression was applied to explore the attitudes toward smoking among the youth (13-18years old) who have been exposed to GHWLs in order to identify relationship of exposure to the GHWLs with smoking initiation and awareness of the danger of smoking. RESULT(S): Six months after implementation, 69.4% of adolescents reported having been exposed to GHWLs in the previous 30days. Among those exposed to GHWLs both boys and girls in grade 7 were significantly more likely than grade 12 high school students to decide not to start smoking (boys: AOR=3.96, 95% CI 3.31-4.75, p<0.001; girls: AOR=2.76, 95% CI 2.32-3.30, p<0.001) and to think that smoking was dangerous to their health (boys: AOR=3.01, 95% CI 2.52-3.58, p<0.001; girls: AOR=2.42, 95% CI 2.03-3.88, p<0.001) after seeing GHWLs. These associations were greater for adolescents who had experienced smoking-prevention education or had been exposed to anti-tobacco advertisements. However, those who smoked, used e-cigarettes, or experienced secondhand smoking were significantly less likely to decide not to smoke and to view smoking as dangerous. CONCLUSION(S): To maintain the perception of the harm of tobacco from childhood through adolescence, the government should implement both comprehensive tobacco controls, including smoking-prevention education in schools, and measures to encourage a smoke-free environment in homes.
4100	Ingels, J. B., Thapa, K., Shrestha, S., Rajbhandari-Thapa, J. (2020) Cigarette and electronic vapor product use among high school students in Georgia, 2015-2018. Preventive Medicine Reports; 19<br><br>**Keywords:**<br>adolescent article *cigarette smoking controlled study health survey *high school student human major clinical study tobacco *vapor

	**Abstract:** Adolescent use of electronic vapor products (EVP) is increasing; however, changes in EVP use in the context of cigarette smoking is less certain. We analyzed trends in EVP and cigarette use among high school students in the state of Georgia. We used self-reported EVP and cigarette use from the annual Georgia Student Health Survey 2.0 for 2015 to 2018 (N = 1,405,108). Users were categorized as exclusive EVP users, exclusive cigarette users, or dual users. We assessed current (>=1 day in past 30 days) use of EVPs, cigarettes, and dual users of both products, as well as number of days the products were used among current users. We compared current users, as well as number of days used, across adjacent years using tests for proportion and Wilcoxon t-tests, respectively. The proportion of current exclusive EVP users and dual users increased during 2017-2018 (4.2% to 6.9% and 1.6% to 3.7%, p < 0.001, respectively) after declining during 2015-2017, while the proportion of exclusive cigarette users declined during 2015-2018 (2.0% to 1.0%, p < 0.001). Similarly, the mean number of days of EVP use increased among exclusive EVP and dual users, and mean number of days of cigarette use increased among dual users during 2017-2018 (p < 0.001). These findings reinforce the importance of continued efforts to reduce all forms of tobacco products use among Georgia high school students.Copyright © 2020
4109	Jackson, D. B., Boccio, C. M., Leal, W. E. (2020) Do youth who vape exhibit risky health lifestyles? Monitoring the future, 2017. Preventive Medicine; 136<br><br>**Keywords:**<br>adolescent article cannabis use cigarette smoking controlled study exercise female health behavior *health hazard human *lifestyle modification male nutritional status priority journal sleep quality *vaping *cannabis *nicotine<br><br>**Abstract:** Previous research links cigarette smoking with an array of unhealthy behaviors including poor diet, poor sleep quality, and reduced levels of physical exercise. To date, however, limited research has explored whether vaping nicotine (or marijuana) is associated with these same health risk behaviors. This paper addresses this gap in the literature by examining associations between vaping (nicotine or marijuana) and poor diet, poor sleep quality, and low levels of exercise in a sample of 7765 young adolescents (grades 8 and 10) enrolled in American schools in 2017. The data were analyzed in 2019. The results suggest that youths who vaped nicotine (or marijuana) did not exhibit significant elevations in risky health behavior outcomes relative to abstaining youths. Even so, cigarette smoking and recent use of marijuana through traditional means are significantly associated with risky health outcomes.Copyright © 2020 Elsevier Inc.
4120	Jankowski, M., Minarowski, L., Mroz, R. M., Guziejko, K., Mojsak, D., Poznanski, M., Zielonka, T. M., Rachel, M., Kornicki, K., Peplowska, P., Bielewicz, K., Piotrowski, W., Ziobron, A., Pekala-Wojciechowska, A., Zborowski, J., Shpakou, A., Skoczynski, S., Majek, P., Lawson, J., Zejda, J. E., Brozek, G. M. (2020) E-cigarette use among young adults in Poland: Prevalence and characteristics of e-cigarette users. Advances in medical sciences; 65<br><br>**Keywords:**<br>adolescent adult article attitude child *cigarette smoking controlled study demography education *electronic cigarette female human major clinical study male multicenter study overall response rate *Poland preschool child *prevalence questionnaire smoke<br><br>**Abstract:** PURPOSE: Electronic cigarette (e-cigarette) use is one of the most popular alternatives to conventional cigarette smoking. This study aimed to investigate the prevalence of cigarette and e-cigarette use among university students from Poland, with particular emphasis on ever and current cigarette and e-cigarette use as well as smoking initiation age. PATIENTS AND METHODS: A cross-sectional survey was conducted between 2017 and 2018 in a group of university students in 5 academic centers in Poland. The questionnaire addressed 46 questions about personal attitudes toward cigarette smoking and e-cigarette use. RESULT(S): Data were collected from 7324 participants (67.3% females,

	aged 21.9 +/- 2.1 years), with an overall response rate of 70.1%. Among participants, 71.2% had ever smoked a cigarette, and almost half of the respondents (45%) declared ever use of an e-cigarette. The mean age of first use of a cigarette was significantly lower (16.5 +/- 2.5 y-old) than of an e-cigarette (18.6 +/- 2.2 y-old; p < 0.001). Exclusive cigarette smoking was declared by 12.9%, 1.3% were e-cigarette users and 1.5% were dual users. Those in the medical field were less likely to try e-cigarettes (odds ratio, OR = 0.73) or to currently smoke conventional cigarettes (OR = 0.82). Older participants were more likely to have ever smoked conventional cigarettes (OR = 1.06), but less likely to have ever used e-cigarettes (OR = 0.88). CONCLUSION(S): In this study, we found a high proportion of young adults who have tried e-cigarettes with both regional and demographic differences. The education profile influences cigarette smoking and e-cigarette use behaviors.Copyright © 2020. Published by Elsevier B.V.
4150	Kasza, K. A., Edwards, K. C., Tang, Z., Stanton, C. A., Sharma, E., Halenar, M. J., Taylor, K. A., Donaldson, E., Hull, L. C., Day, H., Bansal-Travers, M., Limpert, J., Zandberg, I., Gardner, L. D., Hammad, H. T., Borek, N., Kimmel, H. L., Compton, W. M., Hyland, A. (2020) Correlates of tobacco product initiation among youth and adults in the USA: findings from the PATH Study Waves 1-3 (2013-2016). Tobacco control; 29<br><br>**Keywords:**<br>adolescent adult aged article child cigarette cohort analysis demography female follow up heterosexuality human human experiment *juvenile major clinical study male school child *tobacco use young adult *nicotine<br><br>**Abstract:** OBJECTIVE: To report on demographic and tobacco product use correlates of tobacco product initiation (cigarettes, electronic nicotine delivery systems (ENDS), cigars, hookah and smokeless tobacco) among the US population. DESIGN: Data were from the first three waves (2013-2016) of the Population Assessment of Tobacco and Health Study, a nationally representative, longitudinal cohort study of US youth (aged 12-17 years) and adults (aged 18+ years). Never users of at least one type of tobacco product at Wave 1 (W1, 2013/14) or Wave 2 (W2, 2014/15) were included (n=12987 youth; n=25116 adults). Generalised estimating equations were used to evaluate the association between demographic and tobacco product use characteristics at baseline, and tobacco product initiation at follow-up (ever, past 30day (P30D), frequent (use on 20 or more of thepast 30 days)) over two 1-year periods (W1-W2 and W2-Wave 3). RESULT(S): Youth aged 15-17 years were more likely than youth aged 12-14 years and adults aged 18-24 years were more likely than older adults to initiate P30D tobacco use across products; non-heterosexuals were more likely than heterosexuals to initiate P30D cigarette and ENDS use. Older adults were more likely than young adults, and males were more likely than females, to be frequent users of ENDS on initiation. Ever use of another tobacco product predicted P30D initiation of each tobacco product. DISCUSSION: Other tobacco product use and age predict P30D tobacco initiation across products whereas associations with other demographic characteristics vary by product. Continued contemporary evaluation of initiation rates within the changing tobacco product marketplace is important.Copyright © Author(s) (or their employer(s)) 2020. No commercial re-use. See rights and permissions. Published by BMJ.
4180	King, Brian A. (2020) Flavors Are a Major Driver of the Youth E-Cigarette Epidemic. American journal of public health; 110<br><br>**Keywords:**<br>Adolescent *Consumer Behavior/sn [Statistics & Numerical Data] *Electronic Nicotine Delivery Systems/sn [Statistics & Numerical Data] Female *Flavoring Agents Humans Incidence Male Prevalence *Students/px [Psychology] Students/sn [Statistics & Numerical<br><br>**Abstract:**
4202	Kuwabara, Y., Kinjo, A., Fujii, M., Imamoto, A., Osaki, Y., Jike, M., Otsuka, Y., Itani, O., Kaneita, Y., Minobe, R., Maezato, H., Higuchi, S., Yoshimoto, H., Kanda, H. (2020) Heat-not-burn tobacco, electronic cigarettes,

	and combustible cigarette use among Japanese adolescents: a nationwide population survey 2017. BMC public health; 20<br><br>**Keywords:**<br>adolescent article *burn child *cigarette smoking controlled study directory *electronic cigarette *heat high school human Japan *Japanese (people) lifestyle major clinical study male *prevalence questionnaire *smoking<br><br>**Abstract:** BACKGROUND: From among the global public health concerns, smoking remains one of the most crucial challenges. Especially for adolescents, the increase in the use of electronic cigarettes is controversial, as its use may lead to established smoking. In Japan, where a unique tobacco regulation system exists, the heat-not-burn tobacco market has been growing. However, the prevalence and association of combustible cigarettes and new tobacco-related products have not yet been closely investigated among Japanese adolescents. This study aimed to clarify the prevalence of smoking among adolescents, including new types of tobacco-related products, and to compare the characteristics of their users. METHOD(S): The 2017 Lifestyle Survey of Adolescents is a nationally-representative survey collected in Japan. From the national school directory, 98 junior high schools and 86 high schools were randomly sampled throughout Japan. The students completed an anonymous questionnaire at school. We calculated the prevalence of use for each type of tobacco product. Then, the use of a combination of products and the characteristics of different types of products were examined. RESULT(S): In total, 64,152 students from 48 junior high schools and 55 high schools were included the analysis (school response rate=56%, Mage=15.7years, 53.9% boys). The age-adjusted rate of ever (current) use of electronic cigarettes was 2.1% (0.7%) in junior high school and 3.5% (1.0%) in high school; that of combustible cigarettes was 2.6% (0.6%) in junior high school and 5.1% (1.5%) in high school. The rate of heat-not-burn tobacco use was lower relative to other products: 1.1% (0.5%) in junior high school and 2.2% (0.9%) in high school. An examination of the combined use of the three products identified a high number of dual users. Comparisons between different types of users indicated different backgrounds for combustible cigarette users and new product users. CONCLUSION(S): The prevalence of new tobacco-alternative products is growing in popularity among Japanese adolescents. Dual use is common, and many adolescents use new products only. Moreover, e-cigarettes might attract a broader range of groups to smoking. Continuous monitoring and research are needed to investigate their influence as a possible gateway to tobacco smoking.
4223	Leventhal, A. M., Miech, R., Barrington-Trimis, J., Johnston, L. D., O'Malley P, M., Patrick, M. E. (2019) Flavors of e-Cigarettes Used by Youths in the United States. JAMA - Journal of the American Medical Association; 322<br><br>**Keywords:**<br>*adolescent smoking *flavor human letter prevalence priority journal public policy smoking regulation United States vaping *electronic cigarette<br><br>**Abstract:**
4226	Levy, David T., Warner, Kenneth E., Cummings, K. Michael, Hammond, David, Kuo, Charlene, Fong, Geoffrey T., Thrasher, James F., Goniewicz, Maciej Lukasz, Borland, Ron (2019) Examining the relationship of vaping to smoking initiation among US youth and young adults: a reality check. Tobacco control; 28<br><br>**Keywords:**<br>Adolescent Adolescent Behavior Behavior, Addictive Cigarette Smoking/ep [Epidemiology] Cigarette Smoking/pc [Prevention & Control] Cigarette Smoking/px [Psychology] *Cigarette Smoking Electronic Nicotine Delivery Systems/sn [Statistics & Numerical Data]<br><br>**Abstract:** BACKGROUND: The 2018 National Academies of Sciences, Engineering, and Medicine Report

	found substantial evidence that electronic cigarette use (vaping) by youth is strongly associated with an increased risk of ever using cigarettes (smoking) and moderately associated with progressing to more established smoking. However, the Report also noted that recent increases in vaping have been associated with declining rates of youth smoking. This paper examines the temporal relationship between vaping and youth smoking using multiple data sets to explore the question of whether vaping promotes smoking initiation in the USA., METHODS: Using publicly available, nationally representative data on smoking and vaping among youth and young adults, we conducted a trend line analysis of deviations from long-term trends in smoking starting from when vaping became more prevalent., RESULTS: There was a substantial increase in youth vaping prevalence beginning in about 2014. Time trend analyses showed that the decline in past 30-day smoking prevalence accelerated by two to four times after 2014. Indicators of more established smoking rates, including the proportion of daily smokers among past 30-day smokers, also decreased more rapidly as vaping became more prevalent., CONCLUSIONS: The inverse relationship between vaping and smoking was robust across different data sets for both youth and young adults and for current and more established smoking. While trying electronic cigarettes may causally increase smoking among some youth, the aggregate effect at the population level appears to be negligible given the reduction in smoking initiation during the period of vaping's ascendance. Copyright © Author(s) (or their employer(s)) 2019. Re-use permitted under CC BY-NC. No commercial re-use. See rights and permissions. Published by BMJ.
4230	Li, D., Shi, H., Xie, Z., Rahman, I., McIntosh, S., Bansal-Travers, M., Winickoff, J. P., Drehmer, J. E., Ossip, D. J. (2020) Home smoking and vaping policies among US adults: results from the Population Assessment of Tobacco and Health (PATH) study, wave 3. Preventive Medicine; 139<br><br>**Keywords:**<br>adult aerosol article controlled study *current smoker *ex-smoker female harm reduction health care personnel human human tissue major clinical study male prevalence tobacco *vaping<br><br>**Abstract:** We examined the prevalence of home smoking and vaping restrictions among US adults, and compared home policy differences for smoking and vaping among vapers, smokers, and dual users. Secondary data from the Population Assessment of Tobacco and Health (PATH) Study Wave 3 (2015-2016) with 28,148 adults were analyzed using weighted multivariable logistic regression models that account for complex sampling design to compare differences in home policies among non-users, vapers only, smokers only, and dual users. Compared to never-users, current vapers who were ex-smokers and dual users were more likely to allow home vaping (aOR = 11.06, 95% CI: 8.04-15.21; aOR = 6.44, 95% CI: 5.01-8.28) and smoking (aOR = 1.62, 95% CI: 1.19-2.22; aOR = 3.58, 95% CI: 2.88-4.45). Current smokers were more likely to allow vaping (aOR = 3.53, 95% CI: 3.06-4.06) and smoking (aOR = 4.27, 95% CI: 3.73-4.89) inside the home than never-users. Current vapers who never smoked were more likely to allow vaping inside the home than never-users (aOR = 2.45, 95% CI: 1.53-3.93). Vapers reported much lower rates of vape-free home policies relative to both their smoke-free home policies and to vape-free home policies among smokers. Vapers may be using e-cigarettes in hopes of harm reduction, but interpreting "harm reduction" as safe, thus exposing non-users in their homes to second- and thirdhand aerosols. This underscores the need to healthcare providers to extend intervention with vapers to include implementing vape-free home policies.Copyright © 2020 Elsevier Inc.
4239	Liu, X., Lugo, A., Davoli, E., Gorini, G., Pacifici, R., Fernandez, E., Gallus, S. (2020) Electronic cigarettes in Italy: a tool for harm reduction or a gateway to smoking tobacco?. Tobacco control; 29<br><br>**Keywords:**<br>adolescent age article education *electronic cigarette ex-smoker *harm reduction human *Italy major clinical study male *non-smoker prevalence public health smoking cessation *nicotine<br><br>**Abstract:** INTRODUCTION: More than a decade after electronic cigarettes (e-cigarette) hit the European market, we are still debating whether they may help or hinder tobacco control. It is therefore useful to

	explore the potential net effect of e-cigarette use in the general population. METHOD(S): We annually conduct a face-to-face survey on smoking in Italy on a representative sample of the general population aged 15 years or over (52.4million). A total of 15406 subjects were interviewed in 2014-2018. We investigated the consequences of using e-cigarettes on tobacco smoking behaviour among ever and regular e-cigarette users. RESULT(S): In all, 5.7% of our sample reported ever e-cigarette use. Multivariate analyses showed more use by men, ex-smokers and current smokers. E-cigarette use decreased with age and increased with education and calendar year. Only 1.1% of subjects were regular e-cigarette users. This prevalence rose from 0.4% in 2014-2015 to 1.8% in 2016-2017 and was 1.3% in 2018. Among 522 ever users, 13.2% stopped smoking after trying e-cigarettes and 22.2% started smoking or relapsed after using e-cigarettes. The corresponding estimates among regular users were 24.7% and 28.0%, respectively. CONCLUSION(S): Among Italian e-cigarette users, those (re)starting smoking after using e-cigarettes outnumber those who stop smoking after using e-cigarettes. From a public health point of view, e-cigarettes may have an unfavourable net effect. Consequently, if we are not able to prevent sales of e-cigarettes to non-smokers, this product will more likely stimulate smoking tobacco than reduce harm.Copyright © Author(s) (or their employer(s)) 2020. No commercial re-use. See rights and permissions. Published by BMJ.
4255	Mamudu, H. M., Nwabueze, C., Weierbach, F. M., Yang, J., Jones, A., McNabb, M., Adeniran, E., Liu, Y., Wang, L., Blair, C. J., Awujoola, A., Wood, D. L. (2020) Exploring associations between susceptibility to the use of electronic nicotine delivery systems and e-cigarette use among school-going adolescents in rural appalachia. International Journal of Environmental Research and Public Health; 17<br><br>**Keywords:**<br>adolescent *adolescent behavior article demography female high risk population human male rural area social determinants of health social status Tennessee tobacco use vulnerable population *electronic cigarette<br><br>**Abstract:** Electronic nicotine delivery systems (ENDS) use, including e-cigarettes, has surpassed the use of conventional tobacco products. Emerging research suggests that susceptibility to e-cigarette use is associated with actual use among adolescents. However, few studies exist involving adolescents in high-risk, rural, socioeconomically distressed environments. This study examines susceptibility to and subsequent usage in school-going adolescents in a rural distressed county in Appalachian Tennessee using data from an online survey (N = 399). Relying on bivariate analyses and logistic regression, this study finds that while 30.6% of adolescents are ever e-cigarette users, 15.5% are current users. Approximately one in three adolescents are susceptible to e-cigarettes use, and susceptibility is associated with lower odds of being a current e-cigarette user (OR = 0.03; CI: 0.01-0.12; p < 0.00). The age of tobacco use initiation was significantly associated with decreased current use of e-cigarettes (OR = 0.89; CI: 0.83-0.0.97; p < 0.01). Overall, the results of this exploratory study suggest the need for larger studies to identify unique and generalizable factors that predispose adolescents in this high-risk rural, socioeconomically disadvantaged region to ENDS use. Nevertheless, this study offers insight into e-cigarette usage among U.S adolescents in rural, socioeconomically disadvantaged environments and provides a foundation for a closer examination of this vulnerable population.Copyright © 2020 by the authors. Licensee MDPI, Basel, Switzerland.
4263	Mayer, M. E., Kong, G., Barrington-Trimis, J. L., McConnell, R., Leventhal, A. M., Krishnan-Sarin, S. (2020) Blunt and Non-Blunt Cannabis Use and Risk of Subsequent Combustible Tobacco Product Use Among Adolescents. Nicotine & tobacco research : official journal of the Society for Research on Nicotine and Tobacco; 22<br><br>**Keywords:**<br>adolescent article *cannabis use controlled study electronic cigarette female follow up hookah human major clinical study male prospective study *tobacco use cannabis nicotine

	**Abstract:** INTRODUCTION: Cannabis-including blunts (cannabis rolled in tobacco-containing cigar casing) - is commonly the first substance used among adolescents and may increase the likelihood of subsequent initiation of combustible tobacco products. AIMS AND METHODS: Data were pooled from two prospective studies of adolescents in California and Connecticut (total N = 4594). Logistic regression models assessed the association of baseline ever blunt use and ever non-blunt cannabis use (vs. never cannabis use) with subsequent initiation of any combustible tobacco-only product (ie, cigarettes, cigars, or cigarillos) by 1-year follow-up after adjustment for demographic characteristics and other tobacco product use at baseline. We also assessed whether estimates differed by prior e-cigarette or hookah use at baseline. RESULT(S): Among never combustible tobacco-only product users (N = 2973), 221 (7.4%) had ever used a blunt and 114 (3.8%) had ever used only non-blunt cannabis at baseline. Blunt use (adjusted odds ratio [AOR] = 1.98, 95% confidence interval [CI]: 1.30 to 3.01) and non-blunt cannabis use (AOR = 2.38, 95% CI: 1.41 to 4.00) were independently associated with greater odds of combustible tobacco-only product initiation by follow-up. Among those who had not tried e-cigarettes or who had not tried hookah, blunt use and non-blunt cannabis use were associated with significantly increased odds of combustible tobacco product initiation; among those who had tried e-cigarettes or hookah, the association was not significant. CONCLUSION(S): We found blunt and non-blunt cannabis use to be associated with subsequent combustible tobacco-only product initiation, particularly among adolescents who had not also tried other products containing nicotine. IMPLICATIONS: Adolescent-focused tobacco prevention efforts should consider incorporating cannabis products, including blunts. More research is needed to understand how blunt use and cannabis use more broadly are associated with initiation of tobacco products.Copyright © The Author(s) 2019. Published by Oxford University Press on behalf of the Society for Research on Nicotine and Tobacco. All rights reserved. For permissions, please e-mail: journals.permissions@oup.com.
4265	McBrayer, K., Katz, A., Hensel, D., Hunt, A., Ott, M. (2020) 270. Vapor Use and Associated Substance Use Among Rural Middle School Students. Journal of Adolescent Health; 66<br><br>**Keywords:**<br>adolescent Adverse Childhood Experiences Questionnaire African American *alcohol consumption American Indian awards and prizes cannabis use child childhood adversity controlled study cultural factor female gender Hispanic human juvenile language major c<br><br>**Abstract:** Purpose: National data suggests that middle school vapor product use is increasing. Most of our understanding comes from urban populations. Less is known about the risk and protective factors for vapor use among rural middle school youth, nor associations of vaping with other drug use. We examined risk and protective factors of vaping and other drug use in a rural public middle school. Method(s): 6th to 8th grade students completed a survey in health classes in a rural Midwestern low-to-middle income school district. The outcome variable was ever vaped (single, six-category item: never to 40+ times; dichotomized for analysis to no/yes). Risk and protective factors included grade, ever used tobacco, alcohol, or marijuana (no/yes), adverse childhood experience (ACE) (additive index; 8-items; e.g. parent or guardian that served time in jail or prison, no/yes), language acculturation (2-items, range 2-10), parent connectedness (5-items, range 0-20, alpha=0.925) and school connectedness (5-items, range 5-10, alpha=0.803). Data were analyzed using Chi-square and t-tests. Significant bivariate factors were entered into a multivariable logistic regression to estimate the odds of ever vaping; all models were stratified by gender (male/female) because of interactions. The study was IRB approved. Result(s): 1946 surveys were completed. Participants were 51% male, 49% female; 36% White, 47% Hispanic/Latinx, 2.4% Native American and 1.1% African American/Black. Average age was 12.4 years, and a similar proportion of youth were represented across grade level (6th 36%, 7th 33%, 8th 30% 8th). There was high overlap between vaping and other drug use. Compared to adolescents that did not vape, vapor users were more likely to use tobacco (2.3% vs 44.1%, p<.001), alcohol (21.2% vs 80.4%, p<.001), and marijuana (1.6% vs 35.4%, p<.001). In multivariate analysis for females, higher odds of vaping was associated with 8th graders compared to 6th graders (OR=2.05, 95% CI 1.06-3.97), tobacco use (OR=15.07, 95% CI 6.67-

	34.06), alcohol use (OR=7.47, 95% CI 4.29-12.98), marijuana use (OR=7.04, 95% CI 2.7-18.37), and higher scores on language acculturation (OR=1.24, 95% CI 1.08-1.43). Lower odds of vaping were associated with parent connectedness (OR=0.95, 95% CI 0.90-0.999). Race, ACE score, and school connectedness were not significant in multivariate analysis. Among males, higher odds of vaping were associated with tobacco use (OR=5.63, 95% CI 2.62-12.07), alcohol use (OR=6.76, 95% CI 4.16-10.97), marijuana use (OR=9.75, 95% CI 4.31-22.05), higher ACEs (OR=1.13, 95%CI 0.98-1.30) and language acculturation scores (OR=1.12, 95% CI 0.98-1.27). Race, grade, parent and school connectedness were not significant. Conclusion(s): Vapor use is common in this sample of rural school students. In contrast to common assumptions about vaping replacing tobacco use, we observed high levels of co-occurrence of vaping with tobacco, marijuana, and alcohol. Parent connectedness was an important protective factor for females. These data can inform rural prevention programs. Sources of Support: This publication was supported by Award No. #TP1AH000115 from the Office of Population Affairs (OPA). Its contents are solely the responsibility of the authors and do not necessarily represent the official views of OPA or HHS.Copyright © 2019
4268	McElvaney, O. J., Hobbs, B. D., Murphy, M., McElvaney, O. F., Gunaratnam, C., Reeves, E. P., McElvaney, N. G. (2020) Attitudes of irish second-level students towards vaping. American Journal of Respiratory and Critical Care Medicine; 201<br><br>**Keywords:**<br>adolescent advertising anxiety *attitude child chocolate conference abstract controlled study craving female flavor gender human Irish (citizen) major clinical study male nonhuman perception public health smoking cessation strawberry urban area *vaping<br><br>**Abstract:** RATIONALE: E-cigarettes are nicotine delivery devices for humans. e-cigarette use, or vaping, among young people has increased dramatically within the past decade. Attitudes towards vaping and trends in e-cigarette use in this cohort have been documented in the United States. However, similar studies examining the issue in Europe are lacking. METHOD(S): We conducted a survey-based study of Irish second-level students aged 16-18. The study aimed to examine the extent of vaping in schools, factors influencing use and perceptions regarding risk to health. RESULT(S): Of 4,084 students surveyed (96% response rate, M/F=1,659/1,425), 81% had vaped. Of these, 92% had never smoked previously. The average age at first use was 15.7 years. Almost two-thirds were current vapers, with 22% reporting regular use. Students were more likely to vape if they lived in an urban area, if a parent smoked or vaped, and if their parents were separated. No gender-specific patterns were identified. Flavour was considered the most attractive attribute, with the top 3 flavours being strawberry, bubblegum and chocolate, respectively. Only 22% believed vaping was potentially harmful, with less than 2% aware of the current US public health warnings in effect. 94% thought vaping was less likely to be addictive than smoking, and 80% believed a standard-strength vaping cartridge contained less nicotine than in a single cigarette. Despite this, almost one-fifth of regular vapers reported feelings of significant anxiety or craving when not vaping, and 48% reported an inability to control use. In this study, 16% of respondents had tried cannabis or marijuana. Of this subset, 84% first did so via a vaping device, with 88% stating that vaping devices made it easier to take drugs, 98% saying such devices made it easier to avoid being caught, and 91% replying that they would not have used cannabis or marijuana if a vaping device had not been available. Advertisers had clear access to children - 98% of students had previously seen advertisements for vaping products in public places. In urban areas, 82% of students used public transport, with 61% taking the bus to school. Of these, 84% have seen advertisements inside or beside bus shelters. CONCLUSION(S): Use of e-cigarettes among Irish students is widespread. Despite being positioned as smoking cessation aids, most teenagers using e-cigarettes never smoked in the first place. Vaping may represent an entry point not only into the world of smoking, but also to that of illicit drug use. (Figure Presented).
4270	McMillen, Robert, Klein, Jonathan D., Wilson, Karen, Winickoff, Jonathan P., Tanski, Susanne (2019) E-Cigarette Use and Future Cigarette Initiation Among Never Smokers and Relapse Among Former Smokers in the PATH Study. Public health reports (Washington, D.C. : 1974); 134

	**Keywords:**<br>Adolescent Adult Electronic Nicotine Delivery Systems/sn [Statistics & Numerical Data] *Electronic Nicotine Delivery Systems Female Humans Longitudinal Studies Male Population Surveillance Recurrence *Smoking *Smoking Cessation United States Young Adult<br><br>**Abstract:** OBJECTIVES: Any potential harm-reduction benefit of electronic cigarettes (e-cigarettes) could be offset by nonsmokers who initiate e-cigarette use and then smoke combustible cigarettes. We examined correlates of e-cigarette use at baseline with combustible cigarette smoking at 1-year follow-up among adult distant former combustible cigarette smokers (ie, quit smoking >=5 years ago) and never smokers., METHODS: The Population Assessment of Tobacco and Health Study, a nationally representative, longitudinal study, surveyed 26 446 US adults during 2 waves: 2013-2014 (baseline) and 2014-2015 (1-year follow-up). Participants completed an audio computer-assisted interview in English or Spanish. We compared combustible cigarette smoking at 1-year follow-up by e-cigarette use at baseline among distant former combustible cigarette smokers and never smokers., RESULTS: Distant former combustible cigarette smokers who reported e-cigarette past 30-day use (9.3%) and ever use (6.7%) were significantly more likely than those who had never used e-cigarettes (1.3%) to have relapsed to current combustible cigarette smoking at follow-up (P < .001). Never smokers who reported e-cigarette past 30-day use (25.6%) and ever use (13.9%) were significantly more likely than those who had never used e-cigarettes (2.1%) to have initiated combustible cigarette smoking (P < .001). Adults who reported past 30-day e-cigarette use (7.0%) and ever e-cigarette use (1.7%) were more likely than those who had never used e-cigarettes (0.3%) to have transitioned from never smokers to current combustible cigarette smokers (P < .001). E-cigarette use predicted combustible cigarette smoking in multivariable analyses controlling for covariates., CONCLUSIONS: Policies and counseling should consider the increased risk for nonsmokers of future combustible cigarette smoking use as a result of using e-cigarettes and any potential harm-reduction benefits e-cigarettes might bring to current combustible cigarette smokers.
4274	Melka, A., Chojenta, C., Holliday, E., Loxton, D. (2020) E-cigarette use and cigarette smoking initiation among Australian women who have never smoked. Drug and alcohol review;<br><br>**Keywords:**<br>adult article *Australia *Australian binge drinking childhood adversity *cigarette smoking cohort analysis controlled study *electronic cigarette female follow up human longitudinal study major clinical study never smoker prevention risk factor smoking<br><br>**Abstract:** INTRODUCTION AND AIM: Evidence regarding the association between e-cigarette use and subsequent initiation of smoking mostly relates to the US population. In Australia, no studies are available investigating the association between the uses of e-cigarettes and smoking initiation among young adults who have never smoked. This study aimed to determine the association between lifetime e-cigarette use and subsequent initiation of cigarette smoking among tobacco-naive Australian women aged 20-27. DESIGN AND METHODS: The current study used data (n = 5398) from the third (2015) and fourth (2016) surveys collected from a cohort of Australian women born in 1989-1995 who participated in the Australian Longitudinal Study on Women's Health. Multivariable logistic regression was used to identify the association between lifetime e-cigarette use at the baseline survey and initiation of cigarette smoking (smoked 100 cigarettes or more in the last year) at the follow up adjusting for possible confounders. Effects were expressed as odds ratios with 95% confidence interval. RESULT(S): The mean (+/- SD) age of the study participants at baseline (third survey) was 22.5 (+/-1.7). Ever e-cigarette use at baseline was positively associated with smoking initiation at follow up (adjusted odds ratio 3.71; 95% confidence interval 2.33, 5.93). History of depression, binge drinking and higher childhood adversity score were also risk factors for subsequent smoking initiation in the follow up. DISCUSSION AND CONCLUSION(S): This study identified a strong association between e-cigarette use and subsequent initiation of smoking. Enforcing the existing restriction of sale and supply of e-liquid containing nicotine is

	essential to prevent never smokers from nicotine addiction via e-cigarettes.Copyright © 2020 Australasian Professional Society on Alcohol and other Drugs.
4288	Moore, Graham F., Angel, Lianna, Gray, Linsay, Copeland, Lauren, Van Godwin, Jordan, Segrott, Jeremy, Hallingberg, Britt (2020) Associations of Socioeconomic Status, Parental Smoking and Parental E-Cigarette Use with 10-11-Year-Old Children's Perceptions of Tobacco Cigarettes and E-Cigarettes: Cross Sectional Analysis of the CHETS Wales 3 Survey. International journal of environmental research and public health; 17<br><br>**Keywords:**<br>*Attitude to Health Child Cross-Sectional Studies *Electronic Nicotine Delivery Systems Female Humans Male *Parent-Child Relations Smoking Social Class Surveys and Questionnaires Tobacco *Tobacco Products *Vaping<br><br>**Abstract:** BACKGROUND: This study examines primary schoolchildren's perceptions of e-cigarettes and tobacco cigarettes, and associations with parental smoking, vaping and socioeconomic status., METHODS: Survey of 2218 10-11-year-old children in 73 schools in Wales., RESULTS: Overall, 36% reported that a parent figure smoked compared to 21% for vaping, with parental smoking lower in affluent families (OR = 0.72; 95% CI = 0.68 to 0.76). Overall, 1% had tried a cigarette, while 5% had tried an e-cigarette. Most said they would not smoke or vape in 2 years' time; susceptibility to vaping (20%) was higher than smoking (12%). Exposure to and perceptions of tobacco cigarettes were more positive for children of smokers. Having a parent who vaped was associated with exposure to and positive perceptions of e-cigarettes, but not smoking. Most children perceived e-cigarettes as used by adults to stop smoking (64%). Susceptibility to smoking (OR = 0.57; 95% CI = 0.41 to 0.79) and vaping (OR = 0.78; 95% CI = 0.62 to 0.99) were lower among children who perceived e-cigarettes as cessation aids., CONCLUSIONS: Parental smoking continues to be concentrated in poorer families. This study provides no evidence that parental vaping in the absence of smoking is associated with more positive perceptions of tobacco cigarettes. Communicating to children the role of e-cigarettes as cessation devices for smokers may help to limit their appeal to young people.
4315	Odani, S., Armour, B. S., King, B. A., Agaku, I. T. (2020) E-Cigarette Use and Subsequent Cigarette Initiation and Sustained Use Among Youth, U.S., 2015-2017. Journal of Adolescent Health; 66<br><br>**Keywords:**<br>article cigar *cigarette smoking controlled study *electronic cigarette human *juvenile *smokeless tobacco<br><br>**Abstract:** Purpose: Using cross-sectional data, we measured the association between electronic cigarette (e-cigarette) use and subsequent initiation and sustained use of cigarettes among U.S. youth. Method(s): Data were pooled from the 2015-2017 National Youth Tobacco Survey, a school-based survey of U.S. students in grades 6-12. Questions on current age and age of first use of different tobacco products (cigarettes, e-cigarettes, cigars, and smokeless tobacco) were used to ascertain the temporal sequence of tobacco product use. The pooled study population was 52,579 youth who 5 years before the survey had never smoked cigarettes. E-cigarette users were defined as those who used e-cigarettes before or without ever smoking cigarettes. Cigarette smoking was assessed with the following measures: ever smoking a cigarette at any time within the past 5 years and sustained smoking (smoked >=1 year ago and within past 30 days). Adjusted odds ratios (AORs) were calculated, controlling for other tobacco product use and sociodemographics. Result(s): Among never cigarette smokers as of 5 years before the survey, 17.4% used e-cigarettes, and 15.6% first smoked within the past 5 years. Compared with those who did not use e-cigarettes, those who used e-cigarettes had higher odds of ever smoking cigarettes within the past 5 years (AOR = 2.73) and had higher odds of sustained smoking (AOR = 1.55; all p < .05). Conclusion(s): E-cigarette use is associated with subsequent initiation and sustained use of cigarettes

	among youth. Efforts are warranted to reduce youth use of all tobacco products, including e-cigarettes.Copyright © 2019 Society for Adolescent Health and Medicine
4322	Opazo Breton, M., Britton, J., Bogdanovica, I. (2020) Effect of UK plain tobacco packaging and minimum pack size legislation on tobacco and nicotine product switching behaviour. Addiction (Abingdon, England); 115<br><br>**Keywords:**<br>adult article cigar consumer controlled study electronic cigarette female Great Britain household human *law longitudinal study male nicotine replacement therapy purchasing *smoking *nicotine<br><br>**Abstract:** BACKGROUND AND AIMS: The introduction of plain tobacco packaging and minimum pack size legislation in the United Kingdom between May 2016 and May 2017 was accompanied by substantial changes in tobacco product diversity and pricing. This study investigates the extent to which these changes were associated with consumer switching between tobacco product types and price segments, and from tobacco to non-tobacco nicotine products. DESIGN: Longitudinal study (changing trends in product and price choices) and survey (reasons for consumer choices). SETTING: Great Britain PARTICIPANTS: We used 11695 British households from Kantar Worldpanel who purchased tobacco and non-tobacco nicotine products from March 2011 to December 2017. MEASUREMENTS: Product choice was defined using household's monthly purchases of tobacco (cigarettes, roll-your-own (RYO) tobacco, cigar/cigarillo and pipe tobacco) and non-tobacco nicotine products (e-cigarettes and nicotine replacement therapy), while price was defined using price paid by pack size and by price quartiles. Our switching analysis considered three switching alternatives: switching to a different tobacco product or to a combination of tobacco products, switching to any non-nicotine tobacco product, and switching out of our dataset. We explored changes in price quartile purchasing behaviour using binary variables for whether a household purchased or not from each price quartile monthly. Finally, self-declared consumer's choice was assessed through survey responses. FINDINGS: The introduction of plain packaging and minimum pack sizes coincided with major switching to purchasing of pack sizes of 20 cigarettes or 30 g or larger RYO tobacco (>99% of purchases in December 2017) and a migration towards lower-price cigarettes, as for cigarettes, around 80% of purchases were in the lowest price quartiles at the end of the study period compared to about 50% at the start of the study period. During the first 6 months of implementation there was also a marked increase in the likelihood that cigarette smokers would switch to non-tobacco nicotine (OR 1.74, 95% CI: 1.18 to 2.57), predominantly e-cigarettes, compared with the period before May 2016. Survey results suggest that price was the main driver of changes in purchasing behaviour. CONCLUSION(S): Implementation of plain packaging and minimum pack size legislation in the UK appears to have been associated with tobacco users switching to lower price tobacco products and to e-cigarettes.Copyright © 2020 The Authors. Addiction published by John Wiley & Sons Ltd on behalf of Society for the Study of Addiction.
4326	Osibogun, O., Bursac, Z., Maziak, W. (2020) E-Cigarette Use and Regular Cigarette Smoking Among Youth: Population Assessment of Tobacco and Health Study (2013-2016). American Journal of Preventive Medicine; 58<br><br>**Keywords:**<br>adolescent adult article child *cigarette smoking cohort analysis controlled study female follow up human *juvenile longitudinal study male non-smoker United States *electronic cigarette<br><br>**Abstract:** Introduction: This study examines the association between current e-cigarette use at baseline and regular cigarette smoking at follow-up among U.S. youth. Method(s): A longitudinal analysis of youth (aged 12-17 years) data from Waves 1-3 of the Population Assessment of Tobacco and Health Study (2013-2016) was conducted between January 2019 and December 2019. Youth who reported past-30-day current e-cigarette use at baseline were identified and followed for regular cigarette smoking (>=20 days) at follow-up. Result(s): Compared with noncurrent e-cigarette users at baseline, current e-cigarette users

	(cigarette nonsmokers) had 5.0 (95% CI=1.9, 12.8) times higher odds of becoming regular cigarette smokers 1 year later. Additionally, there was a direct linear relationship between the number of days of e-cigarette use at baseline and the number of days of cigarette smoking 1 year later. Conclusion(s): Current e-cigarette use among U.S. youth is associated with higher odds of transitioning to regular cigarette smoking, likely reflecting robust transitions rather than experimentation. These results suggest that promoting e-cigarettes as the current practice for tobacco harm reduction will likely have the unintended consequence of initiating youth cigarette smokers.Copyright © 2020 American Journal of Preventive Medicine
4375	Ratneswaran, C., Steier, J., Reed, K., Khong, T. K. (2019) Electronic Cigarette Advertising Impacts Adversely on Smoking Behaviour Within a London Student Cohort: A Cross-Sectional Structured Survey. Lung; 197<br><br>**Keywords:**<br>Consumer Product Safety Cross-Sectional Studies *Direct-to-Consumer Advertising *Electronic Nicotine Delivery Systems Female *Health Behavior *Health Knowledge, Attitudes, Practice Humans London Male *Non-Smokers/px [Psychology] Risk Assessment *Smokers<br><br>**Abstract:** INTRODUCTION: In contrast to tobacco smoking, electronic cigarette ("vaping") advertisement had been approved in the United Kingdom (UK) in January 2013. Currently, there are an estimated 3.2 million UK e-cigarette users. The impact of e-cigarette advertisement on tobacco use has not been studied in detail. We hypothesised that e-cigarette advertisement impacts on conventional smoking behaviour., METHODS: A cross-sectional structured survey assessed the impact of e-cigarette advertising on the perceived social acceptability of cigarette and e-cigarette smoking and on using either cigarettes or e-cigarettes (on a scale of 1 to 5/'not at all' to 'a lot'). The survey was administered between January to March 2015 to London university students, before and after viewing 5 UK adverts including a TV commercial., RESULTS: Data were collected from 106 participants (22 +/- 2 years, 66% male), comprising cigarette smokers (32%), non-smokers (54%) and ex-smokers (14%). This included vapers (16%), non-vapers (77%) and ex-vapers (7%). After viewing the adverts, smokers (2.6 +/- 1.0 vs. 3.8 +/- 1.1, p = 0.001) and non-smokers (3.2 +/- 0.7 vs. 3.7 +/- 0.8, p = 0.007) felt smoking was more socially acceptable, compared to before viewing them. Participants were more likely to try both e-cigarettes (1.90 +/- 1.03 to 3.09 +/- 1.11, p < 0.001) and conventional cigarettes (1.73 +/- 0.83 to 2.27 +/- 1.13, p < 0.001) after viewing the adverts compared to before. Vapers were less likely to smoke both an e-cigarette, and a conventional cigarette after viewing the adverts., CONCLUSION: E-cigarette advertising encourages both e-cigarette and conventional cigarette use in young smokers and non-smokers. The adverts increase the social acceptability of smoking without regarding the importance of public health campaigns that champion smoking cessation.
4391	Roh, E. J., Chen-Sankey, J. C., Wang, M. Q. (2020) Electronic nicotine delivery system (ENDS) use patterns and its associations with cigarette smoking and nicotine addiction among Asian Americans: Findings from the national adult tobacco survey (NATS) 2013-2014. Journal of Ethnicity in Substance Abuse;<br><br>**Keywords:**<br>adult article *Asian American *cigarette smoking controlled study *electronic cigarette female human major clinical study male *smoking tobacco *tobacco dependence<br><br>**Abstract:** This study examined the ENDS use and their associations with conventional cigarette smoking and nicotine addiction among Asian Americans (AAs). The study sample was restricted to Non-Hispanic AAs (n = 1,956) using the National Adult Tobacco Survey (NATS) 2013-2014. Chi-square tests and logistic regressions were employed. AAs who ever used ENDS reported they were more likely to smoke conventional cigarettes, smoke <=100 cigarettes in a lifetime, and felt more difficulty concentrating without using tobacco than those who never used END. Study findings indicate that ENDS use may be

	associated with conventional cigarette smoking and may lead to nicotine addiction among AAs.Copyright © 2020, © 2020 Taylor & Francis Group, LLC.
4429	Shahab, L., Beard, E., Brown, J. (2020) Association of initial e-cigarette and other tobacco product use with subsequent cigarette smoking in adolescents: a cross-sectional, matched control study. Tobacco control;<br><br>**Keywords:**<br>adolescent article *cigarette smoking controlled study *electronic cigarette female *harm reduction human juvenile major clinical study male propensity score *smoking *nicotine<br><br>**Abstract:** INTRODUCTION: This study assessed whether initiating e-cigarette use increases the uptake of cigarette smoking in US adolescents compared with behavioural and synthetic controls. METHOD(S): Data come from 78265 adolescents in the National Youth Tobacco Survey (2014-2017) of whom 38630 provided information about the first tobacco product they had used in 2014/15. Ever, past 30day and established (30day use and 100+ lifetime cigarettes) cigarette smoking was compared in adolescents who first used an e-cigarette (exposure group), a non-cigarette combustible (CT) or other non-combustible tobacco (NT) product (behavioural controls), and propensity score matched adolescents without initial e-cigarette use (synthetic controls). RESULT(S): Relative to behavioural controls, adolescents who tried e-cigarettes first were less likely to have ever smoked cigarettes (26% vs CT (42.4%; OR 0.48, 95%CI 0.40 to 0.57), or NT initiators (52.7%; OR 0.32, 95%CI 0.26 to 0.39)), to be past 30day (6% vs CT (11.9%; OR 0.48, 95%CI 0.36 to 0.62), or NT initiators (20.0%; OR 0.26, 95%CI 0.19 to 0.35)) or be established cigarette smokers (0.7% vs CT (3.9%; OR 0.17, 95%CI 0.10 to 0.30), or NT initiators (8.4%; OR 0.08, 95%CI 0.04 to 0.13)). E-cigarette initiators were also less likely than synthetic controls (without initial e-cigarette use) to have ever smoked cigarettes (OR 0.76, 95%CI 0.62 to 0.93), be past 30day (OR 0.71, 95%CI 0.55 to 0.91) or be established cigarette smokers (OR 0.26, 95%CI 0.13 to 0.51). CONCLUSION(S): Less than 1% of US adolescents who use e-cigarettes first were established cigarette smokers. They were less likely to be smokers than adolescents who tried other combustible or non-combustible tobacco products first and propensity score matched adolescents without initial e-cigarette use.Copyright © Author(s) (or their employer(s)) 2020. Re-use permitted under CC BY. Published by BMJ.
4430	Sharapova, S., Reyes-Guzman, C., Singh, T., Phillips, E., Marynak, K. L., Agaku, I. (2020) Age of tobacco use initiation and association with current use and nicotine dependence among US middle and high school students, 2014-2016. Tobacco control; 29<br><br>**Keywords:**<br>adolescent article child cigar controlled study craving electronic cigarette ethnicity female *high school student hookah human juvenile major clinical study male race school child *smokeless tobacco *tobacco dependence wakefulness<br><br>**Abstract:** INTRODUCTION: Tobacco use mostly begins in adolescence and young adulthood. Earlier age of initiation of cigarette smoking is associated with greater nicotine dependence and sustained tobacco use. However, data are limited on the age of initiation of non-cigarette tobacco products, and the association between using these products and nicotine dependence and progression to established use. METHOD(S): Combined 2014-2016 National Youth Tobacco Survey data, a nationally representative cross-sectional survey of US students in grades 6-12 yielded 19580 respondents who reported ever using any of five tobacco products: electronic cigarettes, cigarettes, cigars, smokeless tobacco and hookah. Analyses assessed age of reported first use of each product among ever-users, overall and by sex and race/ethnicity. Current daily use, past 30-day use, feelings of craving tobacco and time to first tobacco use after waking were assessed by age of first use. RESULT(S): Among ever-users, weighted median age for first use was 12.6 years for cigarettes, 13.8 years for cigars, 13.4 years for smokeless tobacco, 14.1 years for hookah and 14.1 years for e-cigarettes. First trying these tobacco products at age <=13 years was associated with greater current use of the respective product and nicotine dependence compared

	with initiating use at age >13 years. CONCLUSION(S): First tobacco use at age <=13 years is associated with current daily and past 30-day use of non-cigarette tobacco products, and with the development of nicotine dependence among youth ever-users. Proven tobacco prevention interventions that reach early adolescents are important to reduce overall youth tobacco use.Copyright © Author(s) (or their employer(s)) 2020. No commercial re-use. See rights and permissions. Published by BMJ.
4466	Sumbe, A., Clendennen, S. L., Opara, S. C., Jackson, C. D., Chen, B., Wilkinson, A. V., Harrell, M. B. (2020) Ends Device Type and Initiation of Combustible Tobacco Products among Adolescents. Nicotine & tobacco research : official journal of the Society for Research on Nicotine and Tobacco;<br><br>**Keywords:**<br>adolescent article cigar cohort analysis demography *disposable equipment *electronic cigarette female hookah human major clinical study male marketing Texas *tobacco use<br><br>**Abstract:** INTRODUCTION: The aim of this study was to determine whether ENDS device type (disposable devices, replaceable cartridges, and refillables) at initial or first ENDS use predicts subsequent initiation of combustible tobacco products (cigarettes, hookah, cigars) among adolescents and/or differentiates between those who initiate use of both ENDS and combustible tobacco products at the same time. METHOD(S): The study examined data from the Texas Adolescent Tobacco and Marketing Surveillance System (TATAMS), a longitudinal population-based cohort of students in major metropolitan areas of Texas (n=3907; N=461069). Data were collected every 6 months, from 2014-2018; 33.9% (n=1324; N=151784) of the sample initiated ENDS use across this period. Unadjusted and adjusted logistic regression models were used to assess the odds of initiating combustible tobacco products at a subsequent or similar wave as ENDS initiation, given initial ENDS device type. RESULT(S): After adjusting for sociodemographic factors, the odds of initiating combustible tobacco use subsequent to ENDS initiation were significantly lower among those who reported using Cartridges as their initial device type compared to those who reported Refillables as their initial device type [AOR=0.42(0.18-0.98), p=0.05]. In addition, after controlling for sociodemographic variables, the odds of initiating combustible tobacco use in the same wave as ENDS initiation were significantly higher among those who reported using Cartridges as their initial device type, compared to those who reported Refillables as their initial device type [AOR=2.31(1.05-5.10), p=0.04]. No significant differences were found in adjusted models when Disposables were compared to Refillables and Cartridges to Disposables. CONCLUSION(S): ENDS device type differentiates between adolescents who start using combustible tobacco products at the same time as initial ENDS use, or subsequently.Copyright © The Author(s) 2020. Published by Oxford University Press on behalf of the Society for Research on Nicotine and Tobacco. All rights reserved. For permissions, please e-mail: journals.permissions@oup.com.
4477	Terry-McElrath, Y. M., O'Malley, P. M., Johnston, L. D. (2020) Changes in the Order of Cigarette and Marijuana Initiation and Associations with Cigarette Use, Nicotine Vaping, and Marijuana Use: U.S. 12th Grade Students, 2000-2019. Prevention science : the official journal of the Society for Prevention Research; 21<br><br>**Keywords:**<br>adolescent article *cannabis use clinical article controlled study female human male prevalence *vaping *cannabis *nicotine<br><br>**Abstract:** This study (a) examined changes in marijuana and cigarette initiation sequencing and (b) considered implications of such changes for prevention efforts by examining associations between initiation sequencing and current adolescent substance use. Analyses used 2000-2019 cross-sectional data from the national Monitoring the Future (MTF) study (78,252 U.S. 12th grade students). Models examined trends in six distinct patterns of initiation order, and multivariable associations between order of initiation and 30-day cigarette and marijuana use prevalence, cigarette and marijuana use frequency among users, and nicotine and marijuana vaping prevalence. While the percentage of students initiating

	neither cigarettes nor marijuana increased, increases also were observed in marijuana-only initiation (the fastest-growing pattern) and initiation of marijuana before cigarettes; these increases were accompanied by a significant decrease in cigarette-only initiation. Cigarette use prevalence and frequency were highest among students initiating cigarettes before marijuana; marijuana use prevalence and frequency were highest among students initiating marijuana before cigarettes. Cigarette and marijuana prevalence, as well as marijuana frequency, were lowest among students initiating only a single substance. Nicotine vaping was less prevalent among students initiating a single substance versus both substances, but no significant differences were observed in nicotine vaping prevalence between those initiating only cigarettes versus only marijuana. Implications of these findings for prevention efforts are discussed in the frameworks of both the common liability model and route of administration model.
4493	Vallone, D. M., Cuccia, A. F., Briggs, J., Xiao, H., Schillo, B. A., Hair, E. C. (2020) Electronic Cigarette and JUUL Use among Adolescents and Young Adults. JAMA Pediatrics; 174<br><br>**Keywords:**<br>adolescent *adolescent smoking adult article cohort analysis disease association female follow up health survey human longitudinal study major clinical study male outcome assessment patient monitoring population prevalence priority journal risk factor s<br><br>**Abstract:** Importance: The increasing use rates of electronic cigarettes (e-cigarettes) among young people in the United States have been largely associated with the emergence of high-nicotine-delivery device JUUL. Relevant data are needed to monitor e-cigarette, specifically JUUL, use to help inform intervention efforts. Objective(s): To estimate the prevalence, patterns, and factors associated over time with e-cigarette use among adolescents and younger adults in the United States. Design, Setting, and Participant(s): Two nationally representative longitudinal samples of adolescents and younger adults aged 15 to 34 years were drawn from the Truth Longitudinal Cohort, a national, probability-based cohort. Participants in this cohort were recruited through address-based sampling, and subsamples were recruited from a probability-based online panel. The present cohort study used data from follow-up online surveys, specifically, wave 7 (N = 14379; collected from February 15, 2018, to May 25, 2018) and wave 8 (N = 12114; collected from February 10, 2019, to May 17, 2019). Respondents reported their use of e-cigarettes, JUUL, and combustible tobacco products as well as their harm perceptions, household smoking status, sensation-seeking, friends' e-cigarette use, and demographic information. Main Outcomes and Measures: The main outcomes were ever and current (past 30 days) JUUL use. chi2 Analyses assessed differences in JUUL use by psychosocial and demographic characteristics. Logistic regression models identified the significant factors associated with wave 8 ever and current JUUL use among wave 7 e-cigarette-naive participants. Result(s): A total of 14379 participants (mean [SD] age, 24.3 [0.09] years; 8142 female [51.0%]) were included in wave 7 and 12114 (mean [SD] age, 24.5 [0.10] years; 6835 female [50.1%]) in wave 8. JUUL use statistically significantly increased from wave 7 to wave 8 among ever users (6.0% [n = 1105] to 13.5% [2111]; P <.001) and current users (3.3% [680] to 6.1% [993]; P <.001). JUUL use increased among every age group and was highest among those aged 18 to 20 years (23.9% [491] ever users and 12.8% [340] current users) and 21 to 24 years (18.1% [360] ever users and 8.2% [207] current users). Users reported a higher prevalence of frequent use in wave 8 compared with wave 7 (37.6% vs 26.1%; P <.01). Significant factors associated with future JUUL use among e-cigarette-naive participants included younger age, combustible tobacco use, lower harm perceptions, sensation seeking, and friends' e-cigarette use. Conclusions and Relevance: This study found that the e-cigarette device JUUL appears to be associated with the youth e-cigarette epidemic, attracting new users and facilitating frequent use with their highly addictive nicotine content and appealing flavors. Findings of this study underscore the critical need for increased e-cigarette product regulation at the federal, state, and local levels..Copyright © 2020 American Medical Association. All rights reserved.
4495	Vargas, M., Ebrahimi Kalan, M., Ward-Peterson, M., Osibogun, O., Li, W., Brown, D., Eissenberg, T., Maziak, W. (2020) Effect of flavour manipulation on ENDS (JUUL) users' experiences, puffing behaviour and nicotine exposure among US college students. Tobacco control;

	**Keywords:**<br>adult article *chromosome puff clinical article *college student controlled study electronic cigarette female *flavor *harm reduction human human experiment human tissue male motivation perception pilot study pleasure pod questionnaire satisfaction *smo<br><br>**Abstract:** SIGNIFICANCE: Electronic nicotine delivery system (ENDS) use has continued to increase exponentially among young people in the USA, with unique flavours being one of the most cited reasons for use. Yet, controlled studies examining the effects of restricting flavour are lacking. This study evaluates the impact of ENDS flavour manipulation on user's puffing behaviour, subjective experience, harm perception and nicotine exposure among college-aged ENDS users. METHOD(S): JUUL users (n=30, age 18 to 24 years) attended two 60min ad libitum ENDS use sessions (JUUL preferred flavour vs JUUL classic tobacco flavour) in a cross-over design. Puff topography and plasma nicotine concentration were measured, and participants completed subjective experience questionnaires. RESULT(S): Increases were observed on measures of satisfaction, taste, enjoyment, urges to vape/smoke, pleasure, product appeal and increased concentration following using the preferred flavour pod (p values <0.05). Compared with preferred flavour, participants in the tobacco flavour were less motivated to use it in the future (70.9 vs 19.1 scores, p<0.001), even if it was the only product on the market (75.8 vs 30.7 scores, p<0.001). While nicotine levels significantly increased in both conditions from pre to post session (p values <0.001), no significant differences were observed in nicotine boost levels or on puff topography parameters when comparing both flavour conditions. CONCLUSION(S): This pilot study provides evidence that ENDS flavours have a substantial effect in enhancing young current ENDS users' experiences, product appeal and motivation to use the product in the future. It highlights that limiting flavours could play a potential role when designing strategic policies to reduce the appeal of ENDS use among young people.Copyright © Author(s) (or their employer(s)) 2020. No commercial re-use. See rights and permissions. Published by BMJ.
4496	Veliz, P. T., McCabe, S. E., Evans-Polce, R. J., Boyd, C. J. (2020) Assessing how the history of e-cigarette and cigarette use are associated with the developmental course of marijuana use in a sample of United States adolescents. Drug and Alcohol Dependence; 216<br><br>**Keywords:**<br>adolescent adult article *cannabis use *cigarette smoking controlled study *electronic cigarette female human major clinical study male tobacco *United States *cannabis<br><br>**Abstract:** Background: The purpose of this study was to determine the developmental course of marijuana use among adolescents based on their history of cigarette and e-cigarette use among a national U.S. sample of adolescents who were followed over a four year time-period. Method(s): The data for this study used four waves of the Population Assessment of Tobacco and Health (PATH) Study provided by a panel of 12 to 17-year-olds at Wave 1 (n = 11,059) who completed each of the four annual waves of the adolescent/adult survey. We examined recent use (i.e., past 30-day) of e-cigarettes, cigarettes, and marijuana use at each of the four waves. Result(s): Respondents who had a history of non-concurrent dual use (AOR = 1.67, 95 % CI = 1.24, 2.24) and a history of concurrent dual use (AOR = 1.67, 95 % CI = 1.40, 1.99) had greater odds of past 30-day marijuana use when compared to respondents who had a history of past 30-day e-cigarette use only. Interaction effect models found that e-cigarette only users were at lower risk for past 30-day marijuana use at Wave 1, however, the risk of past 30-day marijuana use increased at a faster rate across the four waves for e-cigarette only users when compared to their peers who used cigarettes or a combination of cigarettes and e-cigarettes. Conclusion(s): While concurrent and non-concurrent dual use was strongly associated with marijuana use over the study period, marijuana use increased at a faster rate across the four-year span of the study among e-cigarette only users.Copyright © 2020 Elsevier B.V.

4498	Verplaetse, T. L., Peltier, M. R., Roberts, W., Moore, K. E., Pittman, B. P., McKee, S. A. (2020) Associations Between Nicotine Metabolite Ratio and Gender With Transitions in Cigarette Smoking Status and E-Cigarette Use: Findings Across Waves 1 and 2 of the Population Assessment of Tobacco and Health (PATH) Study. Nicotine & tobacco research : official journal of the Society for Research on Nicotine and Tobacco; 22<br><br>**Keywords:**<br>adult article case report *cigarette smoking clinical article clinical trial cohort analysis drug therapy early intervention *electronic cigarette female *gender human interview male prediction *smoking biological marker *nicotine<br><br>**Abstract:** INTRODUCTION: Nicotine metabolite ratio (NMR), the ratio of trans 3'-hydroxycotinine to cotinine, is a biomarker of nicotine metabolism. Discrepant findings among clinical trials and population-based studies warrant replication on whether higher NMR, or faster nicotine metabolism, is associated with quitting cigarette smoking. Associations of NMR and e-cigarette use are largely unknown, as well as the relationship between NMR and gender on quitting cigarette smoking or e-cigarette use. METHOD(S): The Population Assessment of Tobacco and Health (PATH) Study is a nationally representative, longitudinal cohort study assessing tobacco use in the US population. In the current study, the PATH (waves 1 and 2; adult interviews) was used to evaluate longitudinal predictions in relationships among NMR and gender and their association with transitions (quit vs. current stable) in cigarette smoking status and e-cigarette use status across waves 1 and 2 of the PATH study. RESULT(S): NMR and gender were not significantly associated with quit behavior for combustible cigarettes. Regarding e-cigarettes, a significant two-way interaction demonstrated that women with higher NMR were less likely to quit e-cigarette use compared to women with lower NMR (odds ratio [OR] = 0.10, 95% confidence interval [CI] = 0.02-0.57; p = .01). CONCLUSION(S): Findings identify that women with faster nicotine metabolism were 10 times less likely to quit e-cigarettes compared to women with slower nicotine metabolism across waves 1 and 2 of the PATH study. Results suggest that NMR may be used as a biomarker for transitions in e-cigarette quit behavior for women. IMPLICATIONS: Findings identify that women with faster nicotine metabolism were 10 times less likely to quit e-cigarettes compared to women with slower nicotine metabolism. Results suggest that NMR may be used as a biomarker for transitions in e-cigarette quit behavior for women. Establishing parameters for NMR collection and for the use of NMR as a biomarker for cigarette smoking behavior and e-cigarette use is an important next step, and may have implications for early intervention and treatment for cessation.Copyright © The Author(s) 2020. Published by Oxford University Press on behalf of the Society for Research on Nicotine and Tobacco. All rights reserved. For permissions, please e-mail: journals.permissions@oup.com.
4499	Villanti, Andrea C., Johnson, Amanda L., Glasser, Allison M., Rose, Shyanika W., Ambrose, Bridget K., Conway, Kevin P., Cummings, K. Michael, Stanton, Cassandra A., Edwards, Kathryn C., Delnevo, Cristine D., Wackowski, Olivia A., Feirman, Shari P., Bansal-Travers, Maansi, Bernat, Jennifer K., Holder-Hayes, Enver, Green, Victoria R., Silveira, Marushka L., Hyland, Andrew (2019) Association of Flavored Tobacco Use With Tobacco Initiation and Subsequent Use Among US Youth and Adults, 2013-2015. JAMA network open; 2<br><br>**Keywords:**<br>Adolescent Adult Child *Electronic Nicotine Delivery Systems Female Flavoring Agents Humans Longitudinal Studies Male *Tobacco Smoking/ep [Epidemiology] *Tobacco Smoking/px [Psychology] United States/ep [Epidemiology] *Vaping/ep [Epidemiology] *Vaping/p<br><br>**Abstract:** Importance: Flavors in tobacco products may appeal to young and inexperienced users., Objective: To examine among youth (aged 12-17 years), young adults (aged 18-24 years), and adults (aged >=25 years) the prevalence of first use of flavored tobacco products among new tobacco users and the association between first flavored use of a given tobacco product and tobacco use 1 year later,

	including progression of tobacco use., Design, Setting, and Participants: This cohort study represents a longitudinal analysis of data from the Population Assessment of Tobacco and Health (PATH) Study, a nationally representative study with data collected in 2013 to 2014 (wave 1) and 2014 to 2015 (wave 2). Participants were noninstitutionalized individuals, including 11996 youth and 26447 adults, in selected households who participated in both waves of the PATH Study. Data analysis was conducted from July 2016 to June 2019., Main Outcomes and Measures: Prevalence of tobacco product use at wave 2., Results: The mean (SE) age of the participants was 14.5 (0.0) years for youth, 21.1 (0.0) years for young adults, and 50.3 (0.0) for adults. Most youth (71.9%; 95% CI, 69.7%-74.0%) and young adults (57.6%; 95% CI, 54.9%-60.3%) who were new users of tobacco products over the 10- to 13-month follow-up period used flavored products. First use of a menthol or mint or other flavored cigarette documented at wave 1 was positively associated with past 12-month and past 30-day cigarette use in all age groups at wave 2 compared with first use of a nonflavored cigarette (youth, flavored cigarette, past 12-month use adjusted prevalence ratio [aPR], 1.14 [95% CI, 1.05-1.25] and past 30-day use aPR, 1.15 [95% CI, 1.00-1.31]; youth, menthol or mint cigarette, past 12-month use aPR, 1.18 [95% CI, 1.08-1.29] and past 30-day use aPR, 1.19 [95% CI, 1.04-1.37]; young adult, flavored cigarette, past 12-month use aPR, 1.09 [95% CI, 1.04-1.15] and past 30-day use aPR, 1.13 [95% CI, 1.06-1.21]; young adult menthol or mint cigarette, past 12-month use aPR, 1.10 [95% CI, 1.05-1.16] and past 30-day use aPR, 1.15 [95% CI, 1.07-1.23]; adult flavored cigarette, past 12-month use aPR, 1.10 [95% CI, 1.05-1.15] and past 30-day use aPR, 1.09 [95% CI, 1.04-1.14]; adult menthol or mint cigarette, past 12-month use aPR, 1.13 [95% CI, 1.08-1.18] and past 30-day use aPR, 1.12 [95% CI, 1.07-1.17]). Among young adults, first use of flavored e-cigarettes (aPR, 2.05; 95% CI, 1.61-2.61), any cigars (aPR, 1.60; 95% CI, 1.26-2.02), cigarillos (aPR, 1.49; 95% CI, 1.08-2.05), filtered cigars (aPR, 3.69; 95% CI, 2.08-6.57), hookah (aPR, 1.91; 95% CI, 1.23-2.98), and any smokeless tobacco (aPR, 1.54; 95% CI, 1.08-2.20) was prospectively associated with current regular use of those products at wave 2 compared with first nonflavored use. Among adults aged 25 years and older, first use of flavored e-cigarettes (aPR, 1.60; 95% CI, 1.41-1.82), any cigars (aPR, 1.56; 95% CI, 1.29-1.87), cigarillos (aPR, 1.29; 95% CI, 1.01-1.64), filtered cigars (aPR, 1.79; 95% CI, 1.25-2.54), hookah (aPR, 5.66; 95% CI, 2.04-15.71), and any smokeless tobacco (aPR, 1.55; 95% CI, 1.32-1.82) was prospectively associated with current regular use of those products at wave 2 compared with first nonflavored use., Conclusions and Relevance: In this longitudinal cohort study, flavors in tobacco products were associated with youth and young adult tobacco experimentation. First use of a flavored tobacco product may place youth, young adults, and adults at risk of subsequent tobacco use.
4500	Villarroel, M. A., Cha, A. E., Vahratian, A. (2020) Electronic Cigarette Use Among U.S. Adults, 2018. NCHS data brief;<br><br>**Keywords:**<br>adult article cigarette smoking demography *electronic cigarette health hazard human lung injury public health tobacco nicotine<br><br>**Abstract:** In 2018, an estimated 8.1 million U.S. adults were current electronic cigarette (e-cigarette) users (1). E-cigarette use is a public health concern (2), and it has been linked to a recent outbreak of lung injury and deaths among adults (3). Although the potential long-term health risks of e-cigarettes are not yet as well-known as they are with cigarettes, e-cigarettes usually contain nicotine, and nicotine is highly addictive (2). Moreover, the most common tobacco product combination among adults is e-cigarettes and cigarettes (4). This report examines e-cigarette use among U.S. adults aged 18 and over by selected sociodemographic characteristics and in relation to cigarette smoking status.Copyright All material appearing in this report is in the public domain and may be reproduced or copied without permission; citation as to source, however, is appreciated.
4516	Wang, Meng, Hu, Ru-Ying, Pan, Jin, Wang, Hao, Yu, Min, Xie, Kai-Xu, Gong, Wei-Wei (2019) Awareness, current use of electronic cigarettes and associated smoking factors in Zhejiang Chinese adolescents. PloS one; 14

	**Keywords:**<br>Adolescent China/ep [Epidemiology] Cross-Sectional Studies *Electronic Nicotine Delivery Systems/sn [Statistics & Numerical Data] Female *Health Knowledge, Attitudes, Practice Humans Male Prevalence Risk-Taking *Students/px [Psychology] Surveys and Ques<br><br>**Abstract:** OBJECTIVES: The present study aims at examining the prevalence of awareness and current use of electronic cigarettes (e-cigarettes) among middle and high school students from Zhejiang, China. Smoking-related factors associated with e-cigarettes use will also be explored., METHODS: This cross-sectional study was based on 2017 Zhejiang Youth Risk Behavior Survey. A total of 24,157 adolescents were recruited and relevant data of e-cigarettes and smoking-related factors were collected via a self-reported questionnaire. Logistic regression models were used to examine the association between e-cigarettes current use and the smoking-related factors. Odds ratios (ORs) and their 95% confidence intervals (CIs) were reported., RESULTS: Overall, 70.61% of middle and high school students reported hearing of e-cigarettes, while only 2.15% reported using e-cigarettes in the past month. Among smoking-related factors, cigarette smoking (ever and current), use of other tobacco products, second hand smoke exposure and previous attempts to quit smoking were significantly associated with higher current e-cigarettes use in adolescents., CONCLUSIONS: These results presented high awareness of e-cigarettes while relatively low use in Chinese adolescents. Smoking-related factors were significantly associated with increased e-cigarettes use.
4528	Weinberger, A. H., Zhu, J., Lee, J., Xu, S., Goodwin, R. D. (2020) Cannabis use and the onset of cigarette and e-cigarette use: A prospective, longitudinal study among youth in the United States. Nicotine & tobacco research : official journal of the Society for Research on Nicotine and Tobacco;<br><br>**Keywords:**<br>adolescent article *cannabis use controlled study demography education female human incidence *juvenile *longitudinal study major clinical study male perception prevalence *prospective study public health *United States *vaping *cannabis<br><br>**Abstract:** INTRODUCTION: Cigarette use is declining among youth in the United States (US), whereas cannabis use and e-cigarette use are increasing. Cannabis use has been linked with increased uptake and persistence of cigarette smoking among adults. The goal of this study was to examine whether cannabis use was associated with the prevalence and incidence of cigarette, e-cigarette, and dual product use among US youth. METHOD(S): Data included US youth ages 12 to 17 from two waves of the Population Assessment of Tobacco and Health (PATH) Study (Wave 1 youth n=13,651; Wave 1 tobacco-naive youth n=10,081). Weighted logistic regression models were used to examine the association between Wave 1 cannabis use and (1) Wave 1 prevalence of cigarette/e-cigarette use among Wave 1 youth, and (2) Wave 2 incidence of cigarette/e-cigarette use among Wave 1 tobacco-naive youth. Analyses were run unadjusted and adjusted for demographics and internalizing/externalizing problem symptoms. RESULT(S): Wave 1 cigarette and e-cigarette use were significantly more common among youth who used versus did not use cannabis. Among Wave 1 tobacco-naive youth, Wave 1 cannabis use was associated with significantly increased incidence of cigarette and e-cigarette use by Wave 2. CONCLUSION(S): Youth who use cannabis are more likely to report cigarette and e-cigarette use and cannabis use is associated with increased risk of initiation of cigarette and e-cigarette use over one year. Continued success in tobacco control-specifically toward reducing smoking among adolescents-may require focusing on vaping, cannabis, and cigarette use in public health education, outreach, and intervention efforts. IMPLICATIONS: These data extend our knowledge of cigarette and e-cigarette use among youth by showing that cannabis use is associated with increased prevalence and incidence of cigarette and e-cigarette use among youth, relative to youth who do not use cannabis. The increasing popularity of cannabis use among youth and diminished perceptions of risk, coupled with the strong link between cannabis use and tobacco use, may have unintended consequences for cigarette control efforts among youth. Continued success in tobacco control among youth may require focusing on cannabis, e-cigarette,

	and cigarette use in public health, outreach, and intervention efforts.Copyright © The Author(s) 2020. Published by Oxford University Press on behalf of the Society for Research on Nicotine and Tobacco. All rights reserved. For permissions, please e-mail: journals.permissions@oup.com.
4559	Her, W. (2020) How Is Use of Electronic Cigarettes Related to Conventional Cigarette Use? A Qualitative Study among Korean American Young Adults. Social work in public health; 35<br><br>**Keywords:**<br>adult *American article *cigarette smoking clinical article data analysis *electronic cigarette exploratory research female human interview male *qualitative research *smoking cessation *social work thematic analysis young adult<br><br>**Abstract:** Given the growing popularity of e-cigarettes in the United States, there is a need to understand the relationship between conventional and electronic cigarette use among Korean American young adults. Thus, this study aimed to explore Korean American young adults' experience of electronic cigarettes and its association with use of conventional cigarettes. This study employed an exploratory qualitative design. Individual interviews were conducted with 15 Korean American young adults aged between 18 and 25 years who reported having smoked at least one conventional cigarette per day for the past 6 months. Interview data were subjected to thematic analysis in order to gain an in-depth understanding of the interconnection between e-cigarette and conventional cigarette use. Data analysis revealed three themes involving reasons for buying, using, and stopping use of e-cigarettes, each of which had several sub-themes. Study results suggest that experience of e-cigarettes was related to conventional cigarette use. Thus, the experience of e-cigarettes as well as conventional cigarettes should be considered in designing smoking cessation interventions for Korean American young adults.
4571	Yang, Y., Lindblom, E. N., Salloum, R. G., Ward, K. D. (2020) The impact of a comprehensive tobacco product flavor ban in San Francisco among young adults. Addictive Behaviors Reports; 11<br><br>**Keywords:**<br>adult article *California *cigar smoking controlled study female *flavor human human tissue major clinical study male prevalence resident *vaping young adult<br><br>**Abstract:** Objective: Flavors play an important role in the initiation and use of tobacco products. The FDA, states, and cities have been implementing or considering banning flavored e-cigarettes or any flavored tobacco products. This study empirically assessed the impact of one of the first comprehensive bans of all flavored tobacco products other than tobacco-flavored e-cigarettes among young adults in San Francisco, California. Method(s): Using Amazon Mechanical Turk, a sample of San Francisco residents aged 18-34 who previously used tobacco products (N = 247) were surveyed about their tobacco use both before and after the ban. Descriptive statistics and regression models were applied. Result(s): The prevalence of overall flavored tobacco use decreased from 81% and 85% to 69% and 76% for 18-24 years and 25-34 years old, respectively. The prevalence of flavored e-cigarettes decreased from 57% and 56% to 45% and 48% for 18-24 years and 25-34 years old, respectively. The prevalence of cigars uses reduced as well. However, cigarette smoking increased, although not statistically significant among 25-34 years old. 66% of participants did not support the ban and 65% believed the ban had not been enforced completely. Most users reported being able to obtain flavored tobacco products in multiple ways despite the ban. Conclusion(s): These findings suggest that comprehensive local flavor bans, by themselves, cannot sharply reduce the availability or use of flavored tobacco products among residents. Nevertheless, local bans can still significantly reduce overall e-cigarette use and cigar smoking but may increase cigarette smoking.Copyright © 2020 The Authors
4574	Villanti, Andrea C., West, Julia C., Mays, Darren, Donny, Eric C., Cappella, Joseph N., Strasser, Andrew A. (2019) Impact of Brief Nicotine Messaging on Nicotine-Related Beliefs in a U.S. Sample. American journal of preventive medicine; 57

	**Keywords:**<br>Adolescent Adult Aged *Cigarette Smoking/px [Psychology] Cigarette Smoking/th [Therapy] Female *Health Education/mt [Methods] *Health Knowledge, Attitudes, Practice Humans Linear Models Male Middle Aged Sampling Studies Smoking Cessation/mt [Methods] *S<br><br>**Abstract:** INTRODUCTION: The current study pilot tested the effect of a single, brief exposure to nicotine education messages on beliefs about nicotine, nicotine-replacement therapy (NRT), E-cigarettes, and cigarettes with reduced nicotine content (RNC)., METHODS: Five hundred and twenty-one U.S. adults (aged >=18 years) completed a 15-minute survey on Amazon Mechanical Turk in 2018. After completing items on sociodemographics, literacy, and cancer risk behaviors, participants were randomized in a 2:1:1 ratio to 1 of 3 conditions: nicotine education (n=263), sun safety education (attention control, n=128), or no message control (n=130). All participants completed items regarding nicotine, NRT, E-cigarette, and RNC cigarette beliefs, as well as norms about nicotine use, behavioral control regarding cigarette/tobacco use, and intention to use cigarettes, NRT, E-cigarettes, and RNC cigarettes in the next 12 months. Analyses were conducted in 2019., RESULTS: Following exposure, nicotine education participants reported fewer false beliefs about nicotine (p<0.001), NRT (p<0.001), E-cigarettes (p<0.05), and RNC cigarettes (p<0.05) compared with the control conditions. Nicotine messaging doubled the probability of a correct response (false, 78.3% vs 36.8%) to nicotine is a cause of cancer and dramatically reduced the probability of responding don't know to this item (5.3% vs 26.0%). There was no impact of the intervention on beliefs about other substances within cigarette, norms, or behavioral intentions., CONCLUSIONS: Findings from the current study support the hypothesis that a brief nicotine messaging intervention-similar to the messages likely to be seen on warning labels or in media campaigns-is likely to correct misperceptions of nicotine, NRT, E-cigarettes, and RNC cigarettes. Copyright © 2019 American Journal of Preventive Medicine. Published by Elsevier Inc. All rights reserved.
4575	Wei, L., Muhammad-Kah, R. S., Hannel, T., Pithawalla, Y. B., Gogova, M., Chow, S., Black, R. A. (2020) The impact of cigarette and e-cigarette use history on transition patterns: a longitudinal analysis of the population assessment of tobacco and health (PATH) study, 2013-2015. Harm reduction journal; 17<br><br>**Keywords:**<br>adult article *cigarette smoking controlled study *electronic cigarette human prediction probability *smoking<br><br>**Abstract:** BACKGROUND: Population models have been developed to evaluate the impact of new tobacco products on the overall population. Reliable input parameters such as longitudinal tobacco use transitions are needed to quantify the net population health impact including the number of premature deaths prevented, additional life years, and changes in cigarette smoking prevalence. METHOD(S): This secondary analysis assessed transition patterns from PATH wave 1 (2013-14) to wave 2 (2014-15) among adult exclusive cigarette smokers, exclusive e-cigarette users, and dual users. Transition probabilities were calculated by taking into account factors including cigarette smoking and e-cigarette use histories and experimental or established use behaviors. Multinomial logistic regression models were constructed to further evaluate factors associated with transition patterns. RESULT(S): Differential transition probabilities emerged among study subgroups when taking into account cigarette smoking and e-cigarette use histories and experimental or established use behaviors. For example, overall 45% of exclusive e-cigarette users in wave 1 continued using e-cigarettes exclusively in wave 2. However, we observed approximately 11 to 14% of wave 1 exclusive experimental e-cigarette users continued to use e-cigarette exclusively in wave 2, compared to about 62% of exclusive established e-cigarette users. The history of cigarette smoking and e-cigarette use is another important factor associated with transition patterns. Among experimental e-cigarette users, 7.5% of individuals without a history of cigarette smoking transitioned to exclusive cigarette smoking, compared to 30% of individuals with a history of cigarette smoking. Additionally, 1.3% of exclusive cigarette smokers in wave 1 transitioned to exclusive e-

	cigarette use, with the highest transition probability (3.7%) observed in the established cigarette smoker with a history of e-cigarette use subgroup. CONCLUSION(S): Product use histories and current use behaviors are important factors influencing transitions between product use states. Given that experimental users' transition behaviors may be more variable and more influenced by tobacco use history, long-term predictions made by population models could be improved by the use of transition probabilities from established users. As transition patterns might be changing over time, long-term transition patterns can be examined through analysis of future waves of PATH data.
4576	Cantrell, J., Huang, J., Greenberg, M. S., Xiao, H., Hair, E. C., Vallone, D. (2020) Impact of e-cigarette and cigarette prices on youth and young adult e-cigarette and cigarette behaviour: evidence from a national longitudinal cohort. Tobacco control; 29<br><br>**Keywords:**<br>adolescent adult article *cigarette smoking *cohort analysis controlled study *electronic cigarette female human human tissue *juvenile major clinical study male market outcome assessment probability *public policy *tobacco young adult *nicotine<br><br>**Abstract:** INTRODUCTION: Understanding the impact of prices for tobacco and nicotine products is critical for creating policies to prevent use among young people. This study examines the impact of electronic cigarette (e-cigarette) and cigarette prices on current e-cigarette and cigarette use among youth and young adults. METHOD(S): Data were from a national probability-based sample aged 15-21 collected in 2014 and followed every 6 months for 2.5 years through 2016. We conducted separate conditional likelihood logistic regression models with past 30-day e-cigarette use and past 30-day cigarette use outcomes on the sample of individuals who participated in at least two survey waves (n=11 578) with linked Nielsen market-level price data for rechargeable e-cigarettes and cigarettes. Models controlled for time-varying variables at the individual and state policy levels, and fixed effects at the individual, wave and market levels. RESULT(S): Higher cigarette prices were associated with increased past 30-day e-cigarette use, indicating e-cigarettes may serve as a substitute for cigarettes. We did not find a statistically significant relationship between rechargeable e-cigarette prices and past 30-day e-cigarette use; neither did we find a significant relationship between rechargeable e-cigarette prices and past 30-day cigarette smoking. CONCLUSION(S): This is the first study to examine e-cigarette and cigarette prices on e-cigarette and cigarette behaviour longitudinally among young people. Findings suggest the need for better measuring the costs associated with e-cigarette use among this population, as well as a careful assessment of price and tax policies that takes into account cross-product impact to sufficiently discourage e-cigarette and cigarette use among young people.Copyright © Author(s) (or their employer(s)) 2020. No commercial re-use. See rights and permissions. Published by BMJ.
4579	Comiford, A. L., Rhoades, D. A., Spicer, P., Dvorak, J. D., Ding, K., Wagener, T. L., Doescher, M. P. (2020) Impact of e-cigarette use among a cohort of American Indian cigarette smokers: associations with cigarette smoking cessation and cigarette consumption. Tobacco control;<br><br>**Keywords:**<br>adult *American Indian article *cigarette smoking cohort analysis controlled study *electronic cigarette female follow up human major clinical study male rank sum test smoke smoking cessation biological marker carbon monoxide cotinine *nicotine<br><br>**Abstract:** INTRODUCTION: Despite American Indian/Alaska Native (AI/AN) people having the highest prevalence of cigarette smoking nationwide, few studies have evaluated e-cigarette use among AI/AN adults who smoke. The primary objective of this observational pilot cohort study was to determine if e-cigarette use is associated with cigarette smoking cessation or reduction among adult AI individuals who smoke. METHOD(S): In 2016, we collected baseline survey and biomarker data among AI adults who smoke. The survey included questions about cigarette consumption and use of e-cigarettes and biomarkers, such as salivary cotinine markers and exhaled carbon monoxide. After 18 months, we

	repeated data collection, and asked about changes in cigarette smoking status and cigarettes per day (CPD). Comparisons between groups were performed using the chi2 test, Fisher's exact test or Wilcoxon rank-sum test. RESULT(S): Of 375 baseline participants, 214 (57.07%) returned for follow-up and were included in analyses. Of these, 20 (9.3%) reported having stopped cigarette smoking and had biochemical verification of cigarette smoking abstinence. Among those who quit smoking, 15% were baseline e-cigarette users; while among those who continued to smoke at follow-up, about 11% were baseline e-cigarette users. This difference was not statistically significant (p=0.48). Among all those who continued to smoke at follow-up, there was no overall decrease in CPD, nor a significant difference in change in CPD between baseline e-cigarette users and non-users (p=0.98). CONCLUSION(S): E-cigarette use at baseline was not associated with smoking cessation or a change in CPD in this cohort of AI adults who smoke after an 18-month follow-up period.Copyright © Author(s) (or their employer(s)) 2020. No commercial re-use. See rights and permissions. Published by BMJ.
4582	Kurti, A. N., Bunn, J. Y., Tang, K., Nighbor, T., Gaalema, D. E., Coleman-Cowger, V., Coleman, S. R. M., Higgins, S. T. (2020) Impact of electronic nicotine delivery systems and other respondent characteristics on tobacco use transitions among a U.S. national sample of women of reproductive age. Drug and Alcohol Dependence; 207<br><br>**Keywords:**<br>adult age distribution article Black person combustion controlled study demography female harm reduction health education Hispanic human longitudinal study mental disease poverty pregnancy priority journal smoking cessation social psychology *tobacco us<br><br>**Abstract:** Background: Identifying predictors of tobacco use patterns that differ in harm among reproductive-aged women may inform efforts to protect women and children against adverse health impacts of tobacco use. Method(s): Changes in tobacco use patterns were examined among women (18-49 years) who completed Wave 1 (W1) and Wave 2 (W2), or W2 and Wave 3 (W3) of the U.S. Population Assessment of Tobacco and Health (PATH, 2013-2016) study, and were using cigarettes, filtered cigars and/or cigarillos in the first wave over which data were included for that respondent (Time 1; T1). We examined the proportion of respondents whose tobacco use transitions from T1 to Time 2 (T2) were harm-maintaining (continued using combusted tobacco), harm-reducing (transitioned to electronic nicotine delivery systems (ENDS), or harm-eliminating (quit tobacco). Multinomial logistic regressions (with harm-maintaining as the baseline category) were conducted to examine associations between ENDS use, demographic, and psychosocial characteristics with each transition. Result(s): A majority of women (83 %) exhibited harm-maintaining transitions, followed by harm-eliminating (14.7 %) and harm-reducing (2.3 %) transitions. Use of ENDS at T1 was associated with increased odds of harm reduction and decreased odds of harm elimination. Younger women were more likely to make both harm-reducing and harm-eliminating transitions. Increased educational attainment, identifying as Black or Hispanic, increased psychiatric symptoms, and pregnancy were associated with harm elimination, whereas living at or above poverty was associated with harm reduction. Conclusion(s): Study results contribute new information on the impact of ENDS, sociodemographic characteristics, psychiatric symptoms, and pregnancy on tobacco use transitions among reproductive-aged women.Copyright © 2019 Elsevier B.V.
4592	Heckman, Bryan W., Fong, Geoffrey T., Borland, Ron, Hitchman, Sara, O'Connor, Richard J., Bickel, Warren K., Stein, Jeffrey S., Yong, Hua-Hie, Nahhas, Georges J., Pope, Derek A., Shang, Ce, Cheng, Kai-Wen, Levy, David T., Cummings, K. Michael (2019) The impact of vaping and regulatory environment on cigarette demand: Behavioral economic perspective across four countries. Addiction; 114<br><br>**Keywords:**<br>*Government Policy Making *Nicotine *Tobacco Smoking *Behavioral Economics *Electronic Cigarettes Costs and Cost Analysis Countries Supply and Demand<br><br>**Abstract:** Background and Aims: Government regulations of nicotine vaping products (NVP) have evolved

	rapidly during the past decade. The impact of NVP regulatory environment and vaping on cigarette demand is unknown. The current study aims to investigate whether or not respondents' reported cigarette demand, as measured by a hypothetical cigarette purchase task, varies with (1) smoking status, (2) vaping status or (3) NVP regulatory environment (country used as proxy). Design: Cross-sectional survey data from wave 1 of the International Tobacco Control (ITC) Four Country Smoking and Vaping (4CV) Survey (2016). Setting: Australia, Canada, England and the United States. Participants: A total of 10 316 adult smokers. Measurements: A hypothetical purchase task asked smokers to estimate how many cigarettes they would purchase for consumption in a single day across multiple cigarette prices. Responses were used to derive measures of cigarette demand. Overall sensitivity of cigarette consumption to price increases was quantified to index cigarette demand elasticity, whereas estimated consumption when cigarettes are free was used to index cigarette demand intensity. Findings: A majority of the non-daily smokers had previously smoked daily (72.3%); daily vapers were more likely to be former daily smokers (89.9%) compared to non-daily vapers (70.1%) and non-vapers (69.2%) (P < 0.001). The smoking status x vaping status interaction was significant for cigarette demand intensity (F = 4.93; P = 0.007) and elasticity (F = 7.30; P = 0.001): among non-daily smokers, vapers reported greater intensity but lower elasticity (i.e. greater demand) relative to non-vapers (Ps < 0.05). Among daily smokers, daily vapers reported greater intensity relative to non-vapers (P = 0.005), but vaping status did not impact elasticity (Ps > 0.38). Intensity was higher in Australia compared with all other countries (Ps < 0.001), but elasticity did not vary by country (F = 2.15; P = 0.09). Conclusions: In a hypothetical purchase task, non-daily smokers showed lower price elasticity if they used e-cigarettes than if they did not, while there was no clear difference in elasticity between e-cigarette users and non-users among daily smokers or according to regulatory environment of their country with regard to e-cigarettes. (PsycInfo Database Record (c) 2020 APA, all rights reserved)
4597	Wagner, L. A., Walley, S. C., Molina, A., Smola, C., Hofto, M. E., Nassetta, L., Samuy, N., Orr, M., Schmit, E., Grizzle, K. (2019) Improving screening for tobacco use in adolescent inpatients at a children's hospital. Pediatrics; 144<br><br>**Keywords:**<br>adolescent child controlled study electronic cigarette female *hospital patient human leadership major clinical study male nursing staff process control psychiatric department questionnaire *smoking cessation software tertiary health care total quality<br><br>**Abstract:** Introduction: Tobacco use is the leading preventable cause of death and disease in the United States. The majority of tobacco users begin before 19 years of age and approximately 25% of high school students report using tobacco products with 16% of those using electronic cigarettes. Despite high rates of tobacco use in adolescents and known associations with poor health outcomes, screening and treatment in the inpatient setting often is lacking. The specific aims of this quality improvement project were 1) to increase screening for tobacco use to 95% of hospitalized patients 13 years and older; 2) to increase reporting of tobacco use by 20%; and 3) to increase reporting of electronic cigarette use by 20% in 13 months. Methods Our study used the Institute for Healthcare Improvement Plan-Do-Study-Act (PDSA) cycles and included all medical-surgical units of a tertiary care, freestanding, urban children's hospital with the exception of the inpatient psychiatric unit. Four iterative PDSA cycles included workgroups with nursing leadership, focus groups with nursing staff, adding electronic cigarette use to the mandatory nursing admission intake questionnaire, and dissemination of an educational video to nursing staff on tobacco screening. Run charts were created of monthly data on the percentage of patients admitted to the institution >= 13 years of age screened for tobacco use, percentage of those screened who reported tobacco use, and types of tobacco products used. Results were analyzed using statistical process control analysis using QI Macros Excel software. Results During the study period (January 2017-January 2018), 3940 patients >= 13 years were admitted to a medical-surgical unit. Overall screening for tobacco use in children >=13 years for the one year prior and throughout the study period was 92.5% and remained unchanged throughout the project. Prior to interventions, reported tobacco use

	in patients who were screened was 2%. After the 4 PDSA cycles there was an increase to 3% reported tobacco use. Reported electronic cigarette use increased from 0 to 3 patients per month. Further review of data noted that two hospital units accounted for 50% of the deficits in tobacco screening. Conclusions Screening for tobacco use in admitted patients >=13 years at a children's hospital was high and did not increase after interventions. Reported tobacco use was much lower than expected based on national data. Implementation of interventions resulted in a 50% increase in the reporting of tobacco use and electronic cigarette use was the major reason for the increased reporting of tobacco use. Next steps include targeting of specific hospital units with real-time feedback to improve tobacco use screening. Future goals include tobacco use cessation interventions to inpatient adolescents who report tobacco use.
4604	Veliz, Philip T., Boyd, Carol J., Evans-Polce, Rebecca J., McCabe, Sean Esteban (2020) Increases in the prevalence of frequent E-cigarette use among adolescents. American Journal of Preventive Medicine; 59<br><br>**Keywords:**<br>*Tobacco Smoking *Trends *Electronic Cigarettes *Adolescent Health Private School Education<br><br>**Abstract:** Presents a study which aims to assess the trends in the frequency of e-cigarette use among adolescents in 2 national studies between 2014 and 2018. The analysis used 2 national surveys that assessed past 30-day frequency. All analyses were conducted in 2019. The National Youth Tobacco Survey (NYTS) annually surveys a cross-sectional, nationally representative sample of U.S. secondary students in public and private schools. The Monitoring the Future (MTF) study annually surveys a cross-sectional, nationally representative sample of 8th, 10th, and 12th grade students attending U.S. public and private secondary schools. Between 2014 and 2018, NYTS data demonstrated that frequent e-cigarette use increased across all grades, especially among 12th graders who increased from 2.6% in 2014 to 9.3% in 2018, primarily between 2017 and 2018. Moreover, the MTF data corroborated NYTS findings showing that frequent e-cigarette use among 12th graders increased from 3.1% in 2017 to 8.9% in 2018. among 12th graders was 24.3% and 25.6%, respectively). Additional analyses assessing trends in the NYTS among only past 30-day e-cigarette users who were in the 12th grade found that in 2018 daily use was the most frequent type of use among current e-cigarette users and experimental use substantially declined between 2014 and 2018. This study provides evidence that frequent use of e-cigarettes has increased substantially among older adolescents during a relatively short period. (PsycInfo Database Record (c) 2020 APA, all rights reserved)
4605	Balogh, E., Wagner, Z., Faubl, N., Riemenschneider, H., Voigt, K., Terebessy, A., Horvath, F., Fuzesi, Z., Kiss, I. (2020) Increasing Prevalence of Electronic Cigarette Use among Medical Students. Repeated Cross-Sectional Multicenter Surveys in Germany and Hungary, 2016-2018. Substance use & misuse; 55<br><br>**Keywords:**<br>adult article *cigarette smoking controlled study *cross-sectional study demography *electronic cigarette female gender German (citizen) *Germany human Hungarian (citizen) *Hungary male medical school *medical student multicenter study non-smoker *preva<br><br>**Abstract:** BACKGROUND: Use of electronic cigarettes (e-cigarettes) is gaining popularity among young adults. Medical students' nicotine use behavior is of particular interest because of their impending role in health promotion. Objective(s): Aim of our study is to assess changes that occurred between 2016 and 2018 in the prevalence of e-cigarette use among medical students and to explore associations between e-cigarette use, demographic characteristics, and cigarette smoking. Self-administered questionnaire surveys were used to obtain cross-sectional data of medical students in Budapest and Pecs, Hungary, and Dresden, Germany. Result(s): Sample sizes for 2016 and 2018 were 2297 and 1514, respectively. In the whole sample, past-30-day use of e-cigarettes increased from 4.5% to 8.0% (p<0.001). The increase in e-cigarette use was significant in both genders (from 3.6% to 5.6% among females, p=0.028, and from 5.9 to 11.4% among males, p<0.001). Prevalence of e-cigarette use was higher among Hungarian students

	than among German students (2.2% versus 5.7% in 2016, and 4.1% versus 10.5% in 2018, p<0.05 for both years). There was no significant difference in e-cigarette use among different academic years. The ratio of e-cigarette users increased significantly among current cigarette smokers but not among nonsmokers. We could not detect a decrease in cigarette smoking. Conclusion(s): Prevalence of e-cigarette use increased significantly among medical students without a reduction in cigarette smoking. Medical schools should add the topic of e-cigarettes to their curricula and need to develop cessation programs to help their students quit both cigarettes and e-cigarettes.
4610	Zvolensky, M. J., Shepherd, J. M., Garey, L., Case, K., Gallagher, M. W. (2020) The influence of neuroticism in terms of E-cigarette dependence and beliefs about use and quitting among dual users of combustible and electronic cigarettes. Addictive Behaviors; 107<br><br>**Keywords:**<br>adult age article correlational study cross-sectional study educational status female human major clinical study male *neurosis risk factor *smoking cessation smoking habit *electronic cigarette<br><br>**Abstract:** Objective: Dual use of combustible and electronic cigarettes (e-cigarettes) is increasing in prevalence and may be related to an increased risk for health problems. Accordingly, dual users represent a particularly vulnerable group. Neuroticism is an individual difference factor that demonstrates robust relations to combustible cigarette use. Yet, no work has examined neuroticism in relation to e-cigarette dependence or cognitions among dual users. Method(s): To address this limitation, the present study examined neuroticism in relation to e-cigarette dependence, expectancies about the negative effects of e-cigarettes, and perceived barriers for quitting e-cigarettes. The present study analyzed data collected from a cross-sectional survey of 437 dual combustible cigarette and e-cigarette users (49.9% female, Mage = 35.12 years, SD = 10.04). Result(s): Results indicated that neuroticism was significantly, positively related to e-cigarette dependence (DELTAR2 = 0.02, p < .001), expectancies about the negative effects of e-cigarettes (DELTAR2 = 0.03, p < .001), and perceived barriers for quitting e-cigarettes (DELTAR2 = 0.10, p < .001). Conclusion(s): These novel findings suggest there may be merit in further exploring the role of neuroticism in the maintenance of concurrent combustible cigarette and e-cigarette use.Copyright © 2020 Elsevier Ltd
4615	Stanton, C. A., Sharma, E., Seaman, E. L., Kasza, K. A., Edwards, K. C., Halenar, M. J., Taylor, K. A., Day, H., Anic, G., Hull, L. C., Bansal-Travers, M., Limpert, J., Gardner, L. D., Hammad, H. T., Borek, N., Kimmel, H. L., Compton, W. M., Hyland, A. (2020) Initiation of any tobacco and five tobacco products across 3 years among youth, young adults and adults in the USA: findings from the PATH Study Waves 1-3 (2013-2016). Tobacco control; 29<br><br>**Keywords:**<br>adult article cigar cohort analysis controlled study cross-sectional study electronic cigarette female hookah human human experiment *juvenile major clinical study male prevalence *smokeless tobacco tobacco use young adult<br><br>**Abstract:** OBJECTIVE: This study reports weighted cross-sectional prevalence of never use of tobacco, and longitudinal past 12-month (P12M), past 30-day (P30D) and frequent P30D any tobacco or specific tobacco product initiation across three 1-year waves. Longitudinal three-wave pathways are examined to outline pathways of exclusive and polytobacco initiation, as well as pathways of new initiators of electronic nicotine delivery systems (ENDS) or cigarettes. DESIGN: Data were drawn from the first three waves (2013-2016) of the Population Assessment of Tobacco and Health Study, a nationally representative, longitudinal cohort study of US youth and adults. Respondents with data at all three waves (youth, N = 11 046; young adults, N = 6478; adults 25+, N = 17 188) were included in longitudinal analyses. RESULT(S): Across the three age groups, weighted cross-sectional analyses revealed never any tobacco use decreased each year from 2013 to 2016, reflecting overall increases in tobacco initiation in the population during this time. Compared with cigarettes, cigars, hookah and smokeless tobacco, ENDS

	had the highest proportion of P12M initiation from Wave 1 to Wave 3 (W3) for each age group. Among youth Wave 2 P30D initiators of exclusive ENDS or cigarettes, the most common W3 outcome was not using any tobacco (ENDS: 59.0% (95% CI 48.4 to 68.8); cigarettes: 40.3% (95% CI 28.7 to 53.1)). CONCLUSION(S): Initiation rates of ENDS among youth and young adults have increased the number of ever tobacco users in the US prevention strategies across the spectrum of tobacco products which can address youth initiation of tobacco products.Copyright © Author(s) (or their employer(s)) 2020. No commercial re-use. See rights and permissions. Published by BMJ.
4616	Evans-Polce, R. J., Veliz, P., Boyd, C. J., McCabe, S. E. (2019) Initiation Patterns and Trends of E-Cigarette and Cigarette Use Among U.S. Adolescents. Journal of Adolescent Health;<br><br>**Keywords:**<br>adolescent article *cigarette smoking controlled study *electronic cigarette female human juvenile major clinical study male perception prevalence questionnaire *tobacco *nicotine<br><br>**Abstract:** Purpose: The primary objectives were to (1) examine the initiation patterns of e-cigarette and cigarette smoking, (2) compare recent trends in initiation patterns for 2015, 2016, and 2017, (3) examine sociodemographic differences in initiation patterns over time and, (4) examine how initiation patterns are associated with cigarette-related perceptions, behaviors, and intentions. Method(s): Data were collected via self-administered questionnaires from 2015 to 2017 nationally representative samples of eighth-grade (modal ages 13-14 years) and 10th-grade (modal ages 15-16 years) students (N = 36,506) attending U.S. secondary public and private schools. Result(s): Among lifetime e-cigarette or cigarette users (n = 9,858), initiating e-cigarettes only was the most common (47.45%). This was followed by cigarette before e-cigarette initiation (18.50%), which decreased in prevalence from 2015 to 2017. E-cigarette before cigarette initiation was the smallest group (6.89%) but increased from 2015 to 2017. E-cigarette before cigarette users were more likely to perceive cigarette use as risky (adjusted odds ratios [aOR]: 1.40; 95% CI: 1.11-1.77) but also more likely to currently smoke cigarettes (aOR:1.30; 95% CI: 1.03-1.63) compared with those who initiated cigarettes before e-cigarettes. Both cigarette only initiators (aOR:.52; 95% CI:.33-.80) and e-cigarette only initiators (aOR:.22; 95% CI:.07-.16) were less likely to report future intentions to smoke compared with those who initiated cigarettes before e-cigarettes. Conclusion(s): Initiation patterns related to e-cigarette and cigarette use among U.S. youth appears to be changing rapidly. Furthermore, patterns of initiation should be considered in future research as cigarette-related risk among different e-cigarette and cigarette use initiation patterns is heterogeneous.Copyright © 2019 Society for Adolescent Health and Medicine
4622	Chu, K. H., Shensa, A., Colditz, J. B., Sidani, J. E., Hoffman, B. L., Sinclair, D., Krauland, M. G., Primack, B. A. (2020) Integrating Social Dynamics Into Modeling Cigarette and E-Cigarette Use. Health education & behavior : the official publication of the Society for Public Health Education; 47<br><br>**Keywords:**<br>article *cigarette smoking controlled study demography *electronic cigarette human juvenile outcome assessment Pennsylvania prevalence public health campaign sensitivity analysis *nicotine<br><br>**Abstract:** Background. The use of electronic cigarettes (e-cigarette) offers potential to facilitate cigarette smoking cessation, yet potentially increases risk of cigarette smoking initiation. This relationship has been primarily modeled in mathematical ways that often do not represent real-world complexities, which could inform decisions regarding local prevention programs or policies. Aims. To develop a model of cigarette and e-cigarette use that combines current research on tobacco use and incorporates real-world geographic and demographic data. Method. We used a platform for developing agent-based models with demographic information representative of the population in Pennsylvania. We developed three models of cigarette and e-cigarette use. The primary outcome for each was the total number of users for cigarette, e-cigarette, and total nicotine. The first model applied current cigarette and e-cigarette data, the second tested the effect of implementing a program of e-cigarette education and policies, and the

	third considered a social contagion factor, where local schools functioned as a transmission vector. Results. The baseline and social contagion models found an overall decline in cigarette use but an increase in e-cigarette and total nicotine use. The education/policies model had declines in all categories. Sensitivity analysis suggested the importance of nuanced e-cigarette/cigarette interactions when modeling tobacco use. Discussion. Public health campaigns that focus on reducing youth e-cigarette usage can have a large effect. Social contagion should be strongly considered when studying e-cigarette spread. Conclusion. Targeted public health campaigns focused on reducing school prevalence of e-cigarette use may be particularly valuable.
4623	Pearson, J. L., Zhou, Y., Smiley, S. L., Rubin, L. F., Harvey, E., Koch, B., Niaura, R., Abrams, D. B. (2020) Intensive longitudinal study of the relationship between cigalike e-cigarette use and cigarette smoking among adult cigarette smokers without immediate plans to quit smoking. Nicotine & tobacco research : official journal of the Society for Research on Nicotine and Tobacco;<br><br>**Keywords:**<br>adult African American article *cigarette smoking controlled study *electronic cigarette female follow up human *longitudinal study major clinical study satisfaction smoke *smoking cessation menthol<br><br>**Abstract:** INTRODUCTION: This study examined the association between the introduction of an e-cigarette and subsequent change in cigarette smoking among smokers who were not immediately interested in quitting. METHOD(S): The Moment Study was a 21-day intensive longitudinal study with an online follow-up survey at 30 days. After observing baseline cigarette smoking for one week, participants received 10 cigalike e-cigarettes on Study Days 6 and 13. Participants reported cigarettes per day (CPD), e-cigarette puffs per day, and e-cigarette satisfaction using text-message based surveys. RESULT(S): The sample of 96 daily smokers was majority female (53.1%), African American (67.7%), and non-Hispanic (95.8%). When e-cigarettes were provided (Day 6), average CPD dropped by 1.82 cigarettes (p<0.0001). The within-person e-cigarette puff effect on daily cigarette smoking was significantly negative (beta = -0.023; p = 0.005); a participant who consumed 100 more e-cigarette puffs in a day than usual for that person was expected to smoke 2.3 fewer cigarettes that day, but this was only true for non-menthol smokers (p = 0.006). Smokers over the age of 45 and those who started smoking at a younger age rated e-cigarettes as less satisfying (ps <0.05). Participants with greater than the median reported satisfaction were 6.5 times more likely to use an e-cigarette at follow-up. CONCLUSION(S): Giving e-cigarettes to smokers who did not intend to quit reduced their cigarette smoking on days when they used e-cigarette more frequently, but this relationship did not hold for menthol smokers. Satisfaction with e-cigarette use was predictive of continued use 30 days later.Copyright © The Author(s) 2020. Published by Oxford University Press on behalf of the Society for Research on Nicotine and Tobacco. All rights reserved. For permissions, please e-mail: journals.permissions@oup.com.
4630	Verplaetse, Terril L., Moore, Kelly E., Pittman, Brian P., Roberts, Walter, Oberleitner, Lindsay M., Peltier, Mac Kenzie R., Hacker, Robyn, Cosgrove, Kelly P., McKee, Sherry A. (2019) Intersection of E-Cigarette Use and Gender on Transitions in Cigarette Smoking Status: Findings Across Waves 1 and 2 of the Population Assessment of Tobacco and Health Study. Nicotine & tobacco research : official journal of the Society for Research on Nicotine and Tobacco; 21<br><br>**Keywords:**<br>*Cigarette Smoking/ep [Epidemiology] Female Humans Male Sex Factors *Smoking Cessation/sn [Statistics & Numerical Data] *Vaping/ep [Epidemiology]<br><br>**Abstract:** INTRODUCTION: Cigarette smokers report using electronic cigarettes (e-cigarettes) to reduce or quit smoking, but findings are mixed regarding the benefit and risk of e-cigarettes in this population, and effects of gender are unknown., METHODS: The Population Assessment of Tobacco and Health (PATH; waves 1 and 2; adult interviews) was used to evaluate relationships among wave 1 e-cigarette use (daily, nondaily, never) and gender and their association with transitions (quit vs. current; relapse vs. former) in

	cigarette smoking status across waves 1 and 2 of the PATH study., RESULTS: Daily e-cigarette users had higher odds of quitting smoking (odds ratio [OR] = 1.56, 95% confidence interval [CI] = 1.12 to 2.18) compared with never e-cigarette users. Conversely, daily and nondaily e-cigarette users were at greater risk of smoking relapse (OR = 1.84, 95% CI = 1.15 to 2.94 and OR = 1.85, 95% CI = 0.99 to 3.46, respectively) compared with never e-cigarette users. Women were less likely to quit smoking compared with men independent of e-cigarette use (OR = 0.76, 95% CI = 0.59 to 0.99). In stratified analyses, daily or nondaily e-cigarette use did not increase the likelihood of quitting or relapse in women. In men, daily and nondaily e-cigarette users were at greater risk of smoking relapse (OR = 2.96, 95% CI = 1.49 to 5.86 and OR = 3.05, 95% CI = 1.29 to 7.17, respectively) compared with men who were never e-cigarette users., CONCLUSIONS: Findings identify e-cigarettes as a potential aid for smoking cessation but also as a potential risk for smoking relapse in men only. Overall, women were less likely to quit smoking, and e-cigarette use did not impact their ability to quit or to stay quit., IMPLICATIONS: Cigarette smokers report using e-cigarettes to reduce or quit smoking, but findings are mixed regarding the benefit and risk of e-cigarettes in this population. Using data from the newly available PATH (waves 1 and 2; adult interviews), our findings identify e-cigarettes as a potential aid for smoking cessation but also identify e-cigarettes as a potential risk for smoking relapse in men only. These findings may have implications for the regulation of e-cigarettes by the Food and Drug Administration and the benefit-cost ratio of e-cigarette use in smokers. Copyright © The Author(s) 2018. Published by Oxford University Press on behalf of the Society for Research on Nicotine and Tobacco. All rights reserved. For permissions, please e-mail: journals.permissions@oup.com.
4632	Lee, P., Fry, J. (2019) Investigating gateway effects using the PATH study. F1000Research; 8<br><br>**Keywords:**<br>adolescent article child *cigarette smoking controlled study female human male never smoker prediction propensity score *psychological aspect smoking tobacco *vaping electronic cigarette *gateway effect smoking initiation<br><br>**Abstract:** Background: A recent meta-analysis of nine cohort studies in youths reported that baseline ever e-cigarette use strongly predicted cigarette smoking initiation in the next 6-18 months, with an adjusted odds ratio of 3.62 (95% confidence interval 2.42-5.41). A recent review of e-cigarettes agreed there was substantial evidence for this "gateway effect". However, the number of confounders considered in the studies was limited, so we investigated whether the effect might have resulted from inadequate adjustment, using Waves 1 and 2 of the Population Assessment of Tobacco and Health study. Method(s): Our main analyses considered Wave 1 never cigarette smokers who, at Wave 2, had information available on smoking initiation. We constructed a propensity score for ever e-cigarette use from Wave 1 variables, using this to predict ever cigarette smoking. Sensitivity analyses accounted for use of other tobacco products, linked current e-cigarette use to subsequent current smoking, or used propensity scores for ever smoking or ever tobacco product use as predictors. We also considered predictors using data from both waves to attempt to control for residual confounding from misclassified responses. Result(s): Adjustment for propensity dramatically reduced the unadjusted odds ratio (OR) of 5.70 (4.33-7.50) to 2.48 (1.85-3.31), 2.47 (1.79-3.42) or 1.85 (1.35-2.53), whether adjustment was made as quintiles, as a continuous variable or for the individual variables. Additional adjustment for other tobacco products reduced this last OR to 1.59 (1.14-2.20). Sensitivity analyses confirmed adjustment removed most of the gateway effect. Control for residual confounding also reduced the association. Conclusion(s): We found that confounding is a major factor, explaining most of the observed gateway effect. However, our analyses are limited by small numbers of new smokers considered and the possibility of over-adjustment if taking up e-cigarettes affects some predictor variables. Further analyses are intended using Wave 3 data which should avoid these problems.Copyright © 2019 Lee P and Fry J.
4633	Khouja, Jasmine N., Munafo, Marcus R., Relton, Caroline L., Taylor, Amy E., Gage, Suzanne H., Richmond, Rebecca C. (2020) Investigating the added value of biomarkers compared with self-reported smoking in predicting future e-cigarette use: Evidence from a longitudinal UK cohort study. PloS one; 15

	**Keywords:**<br>Adolescent Adult Biomarkers/me [Metabolism] Cohort Studies *Electronic Nicotine Delivery Systems/sn [Statistics & Numerical Data] Female Humans Longitudinal Studies Male *Self Report *Smoking/ep [Epidemiology] *Smoking/me [Metabolism] United Kingdom/ep<br><br>**Abstract:** Biomarkers can be used to assess smoking behaviour more accurately and objectively than self-report. This study assessed the association between cotinine (a biomarker of smoke exposure) and later e-cigarette use among a population who were unexposed to e-cigarettes in youth. Young people in the Avon Longitudinal Study of Parents and Children took part in the study. We observed associations between cotinine at 15 years (measured between 2006 and 2008 before the wide availability of e-cigarettes) and self-reported ever use of e-cigarettes at 22 (measured between 2014 and 2015 when e-cigarettes were widely available) using logistic regression. A range of potential confounders were adjusted for (age, sex, body mass index, alcohol use and passive smoke exposure). Additionally, we adjusted for the young people's self-reported smoking status/history to explore potential misreporting and measurement error. In a sample of N = 1,194 young people, cotinine levels consistent with active smoking at 15 years were associated with increased odds of e-cigarette ever use at 22 years (Odds Ratio [OR] = 7.24, 95% CI 3.29 to 15.93) even when self-reported active smoking status at age 16 (OR = 3.14, 95% CI 1.32 to 7.48) and latent classes of smoking behaviour from 14 to 16 (OR = 2.70, 95% CI 0.98 to 7.44) were included in the model. Cotinine levels consistent with smoking in adolescence were strongly associated with increased odds of later e-cigarette use, even after adjusting for reported smoking behaviour at age 16 and smoking transitions from 14 to 16.
4639	Jackson, S. E., Farrow, E., Brown, J., Shahab, L. (2020) Is dual use of nicotine products and cigarettes associated with smoking reduction and cessation behaviours? A prospective study in England. BMJ Open; 10<br><br>**Keywords:**<br>adolescent adult article cohort analysis current smoker demography England evidence based practice female follow up human major clinical study male middle aged *nicotine replacement therapy outcome assessment prospective study *smoking cessation *smokin<br><br>**Abstract:** Objectives To investigate associations of dual use of e-cigarettes and cigarettes with subsequent quitting activity (smoking reduction, quit attempts and use of evidence-based cessation aids). To overcome potential confounding by factors associated with use of pharmacological support, we selected dual use of over-the-counter nicotine replacement therapy (OTC NRT) and cigarettes as a behavioural control. Design Prospective cohort study with 6-month follow-up. Setting England, 2014-2016. Participants 413 current smokers participating in the Smoking Toolkit Study, a representative survey of adults in England, who reported current use of e-cigarettes or OTC NRT and provided data at 6-month follow-up. Main outcome measures The exposure was dual use of e-cigarettes or OTC NRT at baseline. Outcomes were change in cigarette consumption, quit attempts and use of evidence-based cessation aids during quit attempts over 6-month follow-up. Relevant sociodemographic and smoking characteristics were included as covariates. Results After adjustment for covariates, dual e-cigarette users smoked two fewer cigarettes per day at follow-up than at baseline compared with dual OTC NRT users (B=2.01, 95% CI -3.62; -0.39, p=0.015). While dual e-cigarette users had 18% lower odds than dual OTC NRT users to make a quit attempt at follow-up (risk ratio (RR) 0.82, 95% CI 0.67 to 1.00, p=0.049), the groups did not differ in use of cessation aids (RR 1.06, 95% CI 0.93 to 1.21, p=0.388). Conclusions Dual use of e-cigarettes is associated with a greater reduction in cigarette consumption than dual use of OTC NRT. It may discourage a small proportion of users from making a quit attempt compared with dual OTC NRT use but it does not appear to undermine use of evidence-based cessation aids.Copyright © Author(s) (or their employer(s)) 2020. Re-use permitted under CC BY. Published by BMJ.

4640	Wong, S. W., Lohrmann, D. K., Middlestadt, S. E., Lin, H. C. (2020) Is E-cigarette use a gateway to marijuana use? Longitudinal examinations of initiation, reinitiation, and persistence of e-cigarette and marijuana use. Drug and Alcohol Dependence; 208<br><br>**Keywords:**<br>adult aged alcohol consumption article *cannabis use cigarette smoking cohort analysis controlled study female follow up high risk behavior human logistic regression analysis longitudinal study male prevalence priority journal tool use *electronic cigar<br><br>**Abstract:** Background: Concerns have been raised regarding e-cigarette use as a potential stepping-stone to marijuana use. Based on Kandel's gateway hypothesis, this study investigated if e-cigarette use could lead to marijuana use by testing two hypotheses with a longitudinal national U.S. adult sample, including (1) primary hypothesis: e-cigarette use is a gateway to marijuana use; and (2) falsification hypothesis: marijuana use is not a gateway to e-cigarette use. Method(s): Adults were extracted from the Population Assessment of Tobacco and Health Study Waves 1-3 (2013-2016). For primary hypotheses, based on baseline e-cigarette use statuses, three study groups were defined, and three logistic regressions were conducted to examine associations between baseline e-cigarette use and follow-up marijuana use initiation, reinitiation, and persistence, respectively. Similarly, for falsification hypotheses, three additional study groups were defined, and three logistic regressions were conducted to examine associations between baseline marijuana use and follow-up e-cigarette use behaviors. Result(s): Baseline e-cigarette use was associated with marijuana use initiation and reinitiation (ORs = 2.08, 1.37, respectively, both ps < .05) but not persistence at follow-up. Additionally, baseline marijuana use was associated with only e-cigarette use initiation (OR = 2.23, p < .01) but not reinitiation or persistence at follow-up. Conclusion(s): Findings partially support the gateway hypothesis that e-cigarette use could be a steppingstone to marijuana use reinitiation among the U.S. adults. The mechanisms and behavioral characteristics, such as etiological and psychosocial factors, that may pertain to the progression from e-cigarette use to reinitiation of other substance use should be further investigated to inform effective behavioral, educational, and policy interventions.Copyright © 2020 Elsevier B.V.
4642	Khouja, J. N., Suddell, S. F., Peters, S. E., Taylor, A. E., Munafo, M. R. (2020) Is e-cigarette use in non-smoking young adults associated with later smoking? A systematic review and meta-analysis. Tobacco control;<br><br>**Keywords:**<br>*addiction adult behavioral medicine *cigarette smoking Cochrane Library controlled study *electronic cigarette Embase extraction female human male Medline meta analysis non-smoker outcome assessment review self report smoking systematic review Web of S<br><br>**Abstract:** OBJECTIVE: The aim of this review was to investigate whether e-cigarette use compared with non-use in young non-smokers is associated with subsequent cigarette smoking. DATA SOURCES: PubMed, Embase, Web of Science, Wiley Cochrane Library databases, and the 2018 Society for Research on Nicotine and Tobacco and Society for Behavioural Medicine conference abstracts. STUDY SELECTION: All studies of young people (up to age 30 years) with a measure of e-cigarette use prior to smoking and an outcome measure of smoking where an OR could be calculated were included (excluding reviews and animal studies). DATA EXTRACTION: Independent extraction was completed by multiple authors using a preprepared extraction form. DATA SYNTHESIS: Of 9199 results, 17 studies were included in the meta-analysis. There was strong evidence for an association between e-cigarette use among non-smokers and later smoking (OR: 4.59, 95% CI: 3.60 to 5.85) when the results were meta-analysed in a random-effects model. However, there was high heterogeneity (I2 =88%). CONCLUSION(S): Although the association between e-cigarette use among non-smokers and subsequent smoking appears strong, the available evidence is limited by the reliance on self-report measures of smoking history without biochemical verification. None of the studies included negative controls which would provide stronger evidence for

	whether the association may be causal. Much of the evidence also failed to consider the nicotine content of e-liquids used by non-smokers meaning it is difficult to make conclusions about whether nicotine is the mechanism driving this association.Copyright © Author(s) (or their employer(s)) 2020. Re-use permitted under CC BY. Published by BMJ.
4657	Roberts, M. E., Keller-Hamilton, B., Ferketich, A. K., Berman, M. L. (2020) Juul and the upsurge of e-cigarette use among college undergraduates. Journal of American college health : J of ACH;<br><br>**Keywords:**<br>adult article cohort analysis *college student *electronic cigarette female gender human male public health social status *tobacco dependence young adult<br><br>**Abstract:** Objective: Examine trends in e-cigarette use, and Juul use specifically, among U.S. college students. Participant(s): In 2016, we established a cohort of 529 incoming first-year students to a large Midwestern University. In 2018, these students (now third-years) were re-contacted, and a new sample of 611 incoming first-year students was enrolled. Method(s): First-year students in 2016 completed a survey assessing their e-cigarette use; in 2018, first- and second-year students reported on e-cigarette use, and use of Juul specifically. Result(s): From 2016 to 2018, past 30-day e-cigarette use rose from 5.9% to 27.7%. In 2018, for Juul alone, ever use was above 35% and past 30-day use was above 20% for both cohorts. Juul use did not differ by gender, but was associated with higher socioeconomic status (SES) and being White. Conclusion(s): Findings present disturbing possibilities for long-term nicotine addiction among the next generation, and underscore the need for a rapid public health response.
4662	Patel, Minal, Cuccia, Alison, Willett, Jeffrey, Zhou, Yitong, Kierstead, Elexis C., Czaplicki, Lauren, Schillo, Barbara, Hair, Elizabeth C., Vallone, Donna (2019) JUUL use and reasons for initiation among adult tobacco users. Tobacco control; 28<br><br>**Keywords:**<br>Adolescent Adult *Electronic Nicotine Delivery Systems/sn [Statistics & Numerical Data] Female Humans Male Middle Aged Smokers/sn [Statistics & Numerical Data] Smoking Cessation/sn [Statistics & Numerical Data] Surveys and Questionnaires *Tobacco Produc<br><br>**Abstract:** BACKGROUND: The electronic nicotine delivery system (ENDS) JUUL has quickly captured the ENDS market, representing 74.6% of the total dollar share for this category as of November 2018. Although JUUL is marketed as an alternative to cigarettes intended for current adult smokers, evidence suggests that a majority of ENDS users are concurrently current cigarette smokers. Little is known about the dual use of JUUL and cigarettes, as well as the reasons for trying JUUL among adult tobacco users., METHODS: A survey fielded via web and phone of 1332 current cigarette, cigar, little cigar or cigarillo (CLCC), and ENDS users aged 18-54 years was conducted from March to May 2018. Weighted descriptive and bivariate analyses examined JUUL use and reasons for trying JUUL by demographics, combustible tobacco use, ENDS use and intention to quit., RESULTS: Approximately 81% of our sample reported current use of two or more tobacco products, including cigarettes, CLCCs or ENDS. Among current tobacco users, 15% ever used JUUL and 12% used JUUL in the past 30 days. Most individuals (74%) reporting ever JUUL use indicated using it for 5 days or fewer in the past 30 days. The most common reason for trying JUUL was trying to quit smoking cigarettes (37%), followed by family, friends or colleagues using the product (32%)., CONCLUSION: Although some tobacco users may be initiating JUUL to decrease combustible use, most were using the product infrequently and concurrently with other products. Findings have significant implications for cessation intervention efforts and policy development to help smokers quit. Copyright © Author(s) (or their employer(s)) 2019. No commercial re-use. See rights and permissions. Published by BMJ.
4663	Fucito, L. M., Robledo, D. S., LaVigne, J. P., Ash, G. I., DeMartini, K. S., Pittman, B., Redeker, N. S., O'Malley, S. S. (2020) Juul use associated with greater alcohol-related harmin heavy-drinking

	youngadults. Alcoholism: Clinical and Experimental Research; 44<br><br>**Keywords:**<br>adult alcoholism cannabis use clinical trial conference abstract controlled study DSM-5 electronic cigarette female *heavy drinking human major clinical study male Positive and Negative Affect Schedule prevention psychiatric diagnosis questionnaire rewa<br><br>**Abstract:** Young adults are using e-cigarettes, particularly JUUL, at increasing rates. JUUL quickly delivers nicotine at higher levels than older generation e-cigarette devices. Despite the rapid rise in JUUL use, research on young adult JUUL users is limited. Importantly, nicotinemay increase alcohol reward and counteract the sedating/impairing effects of alcohol.Moreover, recent NESARC findings showed an association between e-cigarette use (older generation devices) and harmful alcohol use. The goal of this study was to examine alcohol-related risks among young adult JUUL users and nonusers. Baseline data were drawn from an ongoing trial of a sleep alcohol prevention program for young adults who reported recent heavy drinking (N = 72). Intake measures included: (1) the Timeline Followback Interview to assess alcohol, tobacco, and marijuana use within the past 30 days; (2) the Structured Diagnostic Interview for DSM-V to assess lifetime psychiatric diagnoses; (3) selfreport questionnaires of alcohol-related consequences (BYAACQ), positive and negative mood, (PANAS), and sleep disturbance (PROMIS-SD). More than a third of the sample (26/72; 36.1%) reported JUUL use at baseline. Most JUUL users reported non-daily JUUL use (17/26; 65.4%). Compared to non-users, JUUL users reported a significantly higher maximum number of drinks consumed on 1 occasion (M = 12.87, SD = 4.23 vs. M = 10.17, SD = 4.32; t(70) = 2.57, p = 0.01), significantly greater alcohol-related consequences (M = 9.96, SD = 3.88 vs. M = 6.89, SD = 4.28; t (70) = 3.02, p = 0.003), and a higher total number of drinks (M = 102.58, SD = 46.67 vs. M = 80.35, SD = 48.31; t(70) = 1.90, p = 0.06). Among non-daily JUUL users, themean percentage of JUUL occasions that also involved alcohol use was 83.76 (SD = 21.19). Any JUUL use was associated with a greater likelihood ofmarijuana use (X2(1) = 6.93, p = 0.01) and having a mental health disorder (X2(1) = 4.19, p = 0.04). Compared to non-users, JUUL users also reported significantly worse negativemood (M = 22.23, SD = 6.56 vs. M = 17.91, SD = 5.78; t(70) = 2.90, p = 0.01) and sleep (M = 55.32, SD = 7.62 vs.M = 51.71, SD = 6.35; t(68) = 2.13, p = 0.04). Conclusions JUUL usemay elevate alcohol-related risks among young adults who engage in heavy drinking. This increased riskmay be due to the direct effects of high nicotine levels and/or secondary risks associated with JUUL use (e.g., poor sleep, negativemood). Additional research is needed to understand the mechanisms by which co-use of JUUL and alcohol increases alcohol-related harm.
4668	Abdel-Qader, D. H., Al Meslamani, A. Z. (2020) Knowledge and Beliefs of Jordanian Community Toward E-cigarettes: A National Survey. Journal of community health;<br><br>**Keywords:**<br>adult article attitude child controlled study female flavor friend human human tissue *Jordan major clinical study middle aged pregnant woman prevalence *public health questionnaire random sample reliability satisfaction *smoking cessation sociology val<br><br>**Abstract:** Electronic nicotine delivery systems were promoted publically as a healthier replacement for conventional cigarettes. Knowledge and beliefs of the public can drive their behaviours to adapt or reject the new habit. No previous research was conducted in Jordan to assess electronic cigarettes (e-cigarettes) prevalence, and limited data are available on public opinions and orientation toward this new habit. To assess prevalence of, knowledge, attitude and beliefs about e-cigarettes, and examine factors associated with plans of quitting or initiating e-cigarettes among adults in Jordan. A large cross-sectional face-to-face survey on a random sample of adult population aged>=18 years was conducted over two months to include 1820 adults in Jordan. A representative sample was collected using proportionate random sampling technique, which enabled us to geographically categorise the study population. Reliability and validity measures were taken to ensure a comprehensive and appropriate study tool. The

	Statistical Package for Social Science (SPSS) version 24 was used to conduct descriptive analysis, logistic regression, and Rao-Scott chi-square. Findings were considered statistically significant at p value<0.05 (with a confidence limit at 95%). Of 2164 adults approached, 1820 completed the questionnaire (84.1% response rate). The prevalence of current e-cigarette smokers and dual smokers were 11.7% and 4.0%, respectively. Quitting conventional smoking (38.8%) and enjoying the flavour (32.5%) were the most common reasons to start vaping. Friends were the major source of information about e-cigarettes. There was evidence that adults aged 45-59 years were less likely to be dual smokers (OR 0.51; 95%CI 0.29-0.66; p=0.03). Poor knowledge about the content (23.7%) and types of e-cigarettes (14.5%) was reported. Plans to reduce or quit e-cigarettes were significantly associated with three factors: smokers' knowledge about its content, social impression, smokers' satisfaction with e-cigarettes. Social impression was also associated with plans of initiating e-cigarettes. Most participants thought vaping cannot be harmful to children and pregnant women (73.1%) and cannot be addictive (58.2%). Electronic cigarettes were considered helpful in smoking cessation by 69.1% of participants. The proportions of adults who were currently electronic cigarette and dual smokers were 11.7% and 4.0%, respectively. Our research may provide insight to product and individual factors that were associated with plans to sustain, quit or initiate electronic smoking.
4670	Canzan, Federica, Finocchio, Eliana, Moretti, Francesca, Vincenzi, Silvia, Tchepnou-Kouaya, Alex, Marognolli, Oliva, Poli, Albino, Verlato, Giuseppe (2019) Knowledge and use of e-cigarettes among nursing students: results from a cross-sectional survey in north-eastern Italy. BMC public health; 19<br><br>**Keywords:**<br>Adult Cross-Sectional Studies Female *Health Knowledge, Attitudes, Practice Humans Italy/ep [Epidemiology] Male Smokers/sn [Statistics & Numerical Data] Smoking Cessation/sn [Statistics & Numerical Data] *Students, Nursing/px [Psychology] Students, Nurs<br><br>**Abstract:** BACKGROUND: Data on electronic cigarette (e-cigarette) use among health professional students, who can play a central role in promoting healthy habits and smoking cessation, are sparse. Moreover, the association between e-cigarettes and smoking habits is still debated. The present study aimed to investigate the diffusion of e-cigarette use among nursing students in north-eastern Italy and explore its association with tobacco smoking., METHODS: In 2015, a questionnaire focused on e-cigarette use and tobacco smoking habits was anonymously administered to 2020 students attending nursing courses held by Verona University in 5 different centres. Of these students, 1463 (72.4%) answered the questionnaire. The influence of e-cigarette ever use on both tobacco smoking initiation in all subjects and smoking cessation among ever smokers was investigated by multivariable logistic models., RESULTS: Most responders were female (77.1%), and the mean (SD) age was 23.2 (4.2) years. Nearly all students (94.7%) had heard about e-cigarettes. Approximately one-third (30.3, 95% CI 27.9-32.7%) had ever used e-cigarettes, but only 2.1% (1.5-3.0%) had used e-cigarettes in the last month. Very few (2.1%) of those responders who had never used e-cigarettes were willing to try them. Prevalence values were much higher for tobacco smoking: 40.9% of responders reported being current tobacco smokers, and 10.1% reported being past smokers. Ever use and current use of e-cigarettes were reported by 57.2 and 4.4% of current tobacco smokers and by 12.0 and 0.6% of never or past smokers, respectively (p < 0.001). In multivariable analysis, students who ever used e-cigarettes had 13 times greater odds of being an ever tobacco smoker than never users, whereas they had three times lower odds of being a former smoker. Only 26 students were currently using both electronic and tobacco cigarettes, and most declared that they used e-cigarettes to stop or reduce tobacco smoking. Of note, only three students reported that they had completely stopped smoking thanks to e-cigarette use., CONCLUSION: Use of e-cigarettes seemed to be rather rare among Italian nursing students and was mainly restricted to current smokers. E-cigarette use was not associated with smoking cessation in nursing students.
4673	Boyd, C. J., Veliz, P., Evans-Polce, R., Eisman, A. B., Esteban McCabe, S. (2020) Latent class trajectories: U.S. adolescents' nicotine use and its association with nicotine dependence. Addictive Behaviors Reports; 12

	**Keywords:**<br>adolescent article child *cigarette smoking controlled study education *electronic cigarette entropy female household human interview juvenile latent class analysis major clinical study male probability sample race smoking social class *tobacco dependen<br><br>**Abstract:** Introduction: Twenty-seven percent of adolescents used a nicotine/tobacco product in 2018. Our study analyzed three waves from the Population Assessment of Tobacco and Health (PATH) Study and examined adolescent nicotine/tobacco use trajectories over time to determine which latent classes were associated with symptoms of nicotine dependence. Method(s): The PATH Study used a four-stage, stratified area probability sample and annual household interviews with adolescents (12-17 years). Adolescents who indicated past 30-day nicotine/tobacco use at least once were included (n = 1101). We used latent class analysis (LCA) to identify nicotine/tobacco trajectories across three waves of PATH data and their association with six symptoms consistent with nicotine dependence from the Wisconsin Inventory of Smoking Dependence Motives (WISDM-68). Result(s): All types of past 30-day nicotine/tobacco use increased across the three waves. An LCA model fit was assessed using both the CIV and entropy measures in conjunction with the Vuong-Lo-Mendell-Rubin LRT. A five latent class solution had the lowest BIC value (BIC = 9784.272), and resulted in: (1) "Stable/consistent multiproduct use trajectory", (2) "Increasing cigarette use trajectory", (3) "Increasing e-cigarette use trajectory", (4) "Experimental (poly-nicotine/tobacco) use trajectory", and (5) "Increasing other nicotine/tobacco use trajectory". The most prevalent was the "Experimental (poly-nicotine/tobacco) use trajectory" (33.8%) although sex, race, and social class were associated with different trajectories. Those represented by the "Increasing cigarette use trajectory" (19.4%) reported significantly more past-year nicotine dependence symptoms ($b = 1.73$, $p < 0.001$) compared to the "Increasing e-cigarette use trajectory". Findings varied by sex and race. Conclusion(s): Results indicate that the relationship between e-cigarette use and other forms of nicotine/tobacco and substance use is complex and that adolescent nicotine/tobacco users are a heterogenous group with different risks for nicotine dependence. Findings can inform tailored prevention education and messaging for different groups of youth.Copyright © 2020 The Authors
4674	Villanti, Andrea C., Naud, Shelly, West, Julia C., Pearson, Jennifer L., Wackowski, Olivia A., Hair, Elizabeth, Rath, Jessica M., Niaura, Raymond S. (2019) Latent Classes of Nicotine Beliefs Correlate with Perceived Susceptibility and Severity of Nicotine and Tobacco Products in US young adults. Nicotine & tobacco research : official journal of the Society for Research on Nicotine and Tobacco; 21<br><br>**Keywords:**<br>Adolescent Adult Cohort Studies Health Knowledge, Attitudes, Practice Humans *Nicotine Smokers/px [Psychology] Smokers/sn [Statistics & Numerical Data] *Smokers *Smoking Cessation/px [Psychology] Young Adult<br><br>**Abstract:** BACKGROUND: Pervasive misperceptions about nicotine may influence uptake of quit smoking aids and the impact of policies addressing nicotine as a tobacco product constituent., METHODS: Latent class analyses were conducted using four items on nicotine beliefs asked of 4037 adults aged 18-40 in wave 9 (February-March 2016) of the Truth Initiative Young Adult Cohort Study. Confirmatory factor analyses identified three factors from 12 items: nicotine susceptibility (NSUS), nicotine severity (NSEV), and tobacco severity (TSEV). Analyses assessed correlations between latent classes, sociodemographics, and nicotine/tobacco factor scores., RESULTS: A four-class model of nicotine beliefs was the best fit, with the largest class believing that nicotine plays a major part in smoking risks (class 1, n = 2070; 52%). Class 2 shared that belief but also responded "Don't know" to addiction questions (class 2, n = 382; 11%). Fewer belonged in class 3, who reported that nicotine plays a small part in health risks (n = 1277; 30%), and class 4, who perceived nicotine as not cancer causing (n = 308; 7%). Latent class membership was correlated with sociodemographics, peer smoking, and past 30-day tobacco use. Classes 1 and 2 had similar NSUS scores and classes 3 and 4 had similar NSEV and TSEV scores., DISCUSSION: Differences in

	the perceptions of nicotine and tobacco-related harms can be partially explained by clustering of underlying nicotine beliefs. These classes of beliefs are correlated with sociodemographic predictors of smoking. These findings may help to identify specific beliefs or groups to be targeted by public education efforts on nicotine., IMPLICATIONS: The current study supports that underlying nicotine beliefs are associated with perceived harms of specific nicotine and tobacco products (relative to cigarettes), with greater false beliefs about nicotine correlated with greater perceived susceptibility to nicotine addiction. Two important inferences emerge from this study: first, that education to address nicotine beliefs may also reframe perceptions of the harms of nicotine and tobacco products; and second, that this type of education may differentially impact perceptions of the harms of nicotine products (e.g., nicotine replacement therapy and e-cigarettes) and tobacco products (e.g., cigars, smokeless, and hookah). Copyright © The Author(s) 2019. Published by Oxford University Press on behalf of the Society for Research on Nicotine and Tobacco.
4686	Park, Eunhee, Kwon, Misol, Gaughan, Mary Rose, Livingston, Jennifer A., Chang, Yu-Ping (2019) Listening to Adolescents: Their Perceptions and Information Sources About E-cigarettes. Journal of pediatric nursing; 48<br><br>**Keywords:**<br>Adolescent *Adolescent Behavior/px [Psychology] *Electronic Nicotine Delivery Systems Female Health Behavior Humans Male New York Smoking/ae [Adverse Effects] *Smoking/px [Psychology] *Social Perception *Students/px [Psychology]<br><br>**Abstract:** PURPOSE: There has been a sharp increase in adolescent electronic cigarette (e-cigarette) use, and e-cigarettes are now the most commonly used tobacco product among U.S. middle and high school students. Frequent use among high school students was reported to be 27.7% in 2018, an increase of almost 8% from 2017. As yet we have only a limited understanding of adolescent perceptions of e-cigarettes and where adolescents receive information about them., DESIGN AND METHODS: Thirty-four adolescents from Western New York participated in semi-structured interviews. Both adolescents who had used e-cigarettes and never used e-cigarettes were included to capture a broad perspective. The interview data were analyzed using content analysis., RESULTS: Both adolescents who had used e-cigarettes and never used e-cigarettes acknowledged the popularity and acceptance of e-cigarettes among their peers. E-cigarettes were viewed as a healthy alternative to regular cigarettes that mimicked the appearance but were less harmful and more enjoyable. Reasons for not using e-cigarettes included harms to health, risk of addiction, and the gateway effects for other risky behaviors. Major information sources about e-cigarettes included advertisements, family, peers, social media, and the internet., CONCLUSIONS: Adolescent perceptions of e-cigarettes, which contribute to reasons for e-cigarette use or not use, and the sources of information that may influence those perceptions were identified., PRACTICAL IMPLICATIONS: Our findings provide valuable information to guide prevention initiatives and develop interventions, particularly planning for preventive messages and effective communication methods to deliver for adolescents. Copyright © 2019 Elsevier Inc. All rights reserved.
4688	Cooper, M., Yaqub, M., Hinds, J. T., Perry, C. L. (2019) A longitudinal analysis of tobacco use in younger and older U.S. veterans. Preventive Medicine Reports; 16<br><br>**Keywords:**<br>adult air force alcohol consumption army article cigar smoking cigarette smoking cohort analysis comorbidity controlled study female groups by age human longitudinal study male mental health middle aged military service navy observational study pipe smo<br><br>**Abstract:** United States (U.S.) veterans are prone to higher rates of smoking and smoking-related disease. We describe the prevalence of cigarette and non-cigarette product use and determine longitudinal predictors of tobacco use transitions in this vulnerable population. Data are from Waves 1 (2013-2014) and 2 (2014-2015) of the adult cohort in the Population Assessment of Tobacco and Health. Wave 1

	prevalence was calculated for past 30-day use of all queried tobacco products, and compared by veteran status. Weighted multinomial logistic regression was used to determine predictors-demographics, substance use, and physical and psychological comorbidities-of tobacco use transitions (continued use, initiation, and cessation) among veterans. Compared to non-veterans, use of nearly all tobacco products was significantly higher among veterans and was highest among younger veterans. Compared to continued nonusers, continued users were more likely to: be of younger age (OR = 0.95, 95% CI: 0.95-0.96), have poorer physical health (OR = 1.58, 95% CI: 1.22-2.06) and mental health (OR = 1.48, 95% CI: 1.18-1.85), report substance use (OR = 1.79, 95% CI: 1.21-2.64), and report problematic alcohol use (OR = 4.23, 95% CI: 2.38-7.52) and were less likely to be female (OR = 0.57, 95% CI: 0.35-0.93). Compared to continued nonusers, initiators were more likely to report problematic alcohol use (OR = 8.63, 95% CI: 3.79-19.63), and those in the cessation category were more likely to be of younger age (OR = 0.97, 95% CI: 0.95-0.99). Cigarette and non-cigarette use is especially prevalent among young veterans, so prevention should begin during military service. Tobacco cessation programs should be tailored for this population, incorporating aspects related to concomitant health conditions.Copyright © 2019 The Author(s)
4691	Stanton, Cassandra A., Bansal-Travers, Maansi, Johnson, Amanda L., Sharma, Eva, Katz, Lauren, Ambrose, Bridget K., Silveira, Marushka L., Day, Hannah, Sargent, James, Borek, Nicolette, Compton, Wilson M., Johnson, Sarah E., Kimmel, Heather L., Kaufman, Annette R., Limpert, Jean, Abrams, David, Cummings, K. Michael, Goniewicz, Maciej L., Tanski, Susanne, Travers, Mark J., Hyland, Andrew J., Pearson, Jennifer L. (2019) Longitudinal e-Cigarette and Cigarette Use Among US Youth in the PATH Study (2013-2015). Journal of the National Cancer Institute; 111<br><br>**Keywords:**<br>Adolescent Child *Electronic Nicotine Delivery Systems Female History, 21st Century Humans Male Prevalence Propensity Score Public Health Surveillance *Smoking/ep [Epidemiology] *Tobacco Products United States/ep [Epidemiology] Young Adult<br><br>**Abstract:** BACKGROUND: Evidence is accumulating that youth who try Electronic Nicotine Delivery Systems (ENDS, e-cigarettes) may go on to try cigarettes. This analysis examines the bidirectional patterns of ENDS and cigarette use among US youth over one year and uses propensity score matching (PSM) to examine frequency of ENDS use on changes in cigarette smoking., METHODS: Our analysis included 11 996 participants who had two waves of available data (Wave 1 [W1] 2013-2014; Wave 2 [W2] 2014-2015) drawn from the longitudinal Population Assessment of Tobacco and Health Study. Cross-sectional weighted prevalence estimates are reported for cigarettes and ENDS. We used PSM to estimate the likelihood of ENDS use at W1 and to draw matched analytic samples, then used regression (logistic or linear) models to examine the effect of W1 ENDS use on W2 cigarette smoking. All statistical tests were two-sided., RESULTS: In weighted analyses, 69.3% of W1 past-30-day cigarette smokers exhibited past-30-day smoking at W2; 42.2% of W1 past-30-day ENDS users were using ENDS at W2. W1 ever use of either product was similarly associated with W2 new use of the other product. Unweighted PSM models indicated W1 cigarette-naive ENDS use was associated with W2 ever-cigarette smoking (n = 676; adjusted odds ratio = 3.21, 95% confidence interval [CI] = 1.95 to 5.45, P < .001); W1 ever-ENDS use did not affect change in cigarette frequency at W2 (n = 1020, beta = 0.31, 95% CI = -0.76 to 1.39, P = .57); 1-5 days ENDS use compared with ever, no past-30-day ENDS use was associated with a statistically significant decrease of W2 smoking days (n = 256, beta = -2.64, 95% CI = -4.96 to -0.32; P = .03); and W1 6+ day ENDS users did not show a decrease in frequency of cigarette smoking., CONCLUSIONS: Ever-ENDS use predicts future cigarette smoking, and frequency of ENDS use has a differential impact on subsequent cigarette smoking uptake or reduction. These results suggest that both cigarettes and ENDS should be targeted in early tobacco prevention efforts with youth. Copyright © The Author, 2019. Published by Oxford University Press. All rights reserved. For permissions, please email: journals.permissions@oup.com.

4692	Ortega, A., Sutton, M., McConville, A., Cushing, C. C., Fite, P. J. (2020) Longitudinal investigation of the bidirectional associations between initiation of e-cigarettes and other substances in adolescents. Journal of Substance Use;<br><br>**Keywords:**<br>adolescent alcohol consumption article controlled study *electronic cigarette high school human juvenile risk assessment *tobacco alcohol cannabis<br><br>**Abstract:** Background: Although research on adolescent e-cigarette use is growing, several questions remain unanswered, including the timing of initiation and the co-occurrence with initiation of other substances. The current study longitudinally evaluated the risk for initiation of e-cigarettes and examined associations with this risk and initiation of other substances (alcohol, traditional tobacco products, and marijuana). Method(s): 141 youth from a single high school were in 9th and 10th grade at the initial data collection. Data were collected annually for the following two years. Rates of initiation were calculated. Cross-lagged panel models estimated how prior initiation of e-cigarette use affected the risk for initiation of other substances and vice-versa. Result(s): Risk for initiation of e-cigarettes was comparable across the two years. Youth were most at risk for having used alcohol at baseline. As youth aged through high school, they were most at risk for initiation of e-cigarettes relative to other substances. There is preliminary evidence that endorsement of alcohol use at baseline increased the risk for initiation of e-cigarettes one year later. Conclusion(s): Rates of e-cigarette initiations increased throughout the two-year period. These preliminary findings support the notion that efforts to prevent initiation of e-cigarettes should target adolescents who have used alcohol.Copyright © 2020, © 2020 Taylor & Francis Group, LLC.
4693	Taylor, K. A., Sharma, E., Edwards, K. C., Halenar, M. J., Kissin, W., Kasza, K. A., Day, H., Anic, G., Gardner, L. D., Hammad, H. T., Hull, L. C., Bansal-Travers, M., Limpert, J., Borek, N., Kimmel, H. L., Compton, W. M., Hyland, A., Stanton, C. (2020) Longitudinal pathways of exclusive and polytobacco cigarette use among youth, young adults and adults in the USA: findings from the PATH Study Waves 1-3 (2013-2016). Tobacco control; 29<br><br>**Keywords:**<br>adult article *cigarette smoking cohort analysis controlled study electronic cigarette female human *juvenile major clinical study male prevalence *smoking tobacco young adult<br><br>**Abstract:** OBJECTIVE: Cigarettes are the most harmful and most prevalent tobacco product in the USA. This study examines cross-sectional prevalence and longitudinal pathways of cigarette use among US youth (12-17 years), young adults (18-24 years) and adults 25+ (25 years and older). DESIGN: Data were drawn from the first three waves (2013-2016) of the Population Assessment of Tobacco and Health Study, a nationally representative, longitudinal cohort study of US adults and youth. Respondents with data at all three waves (youth, N=11 046; young adults, N=6478; adults 25+, N=17 188) were included in longitudinal analyses. RESULT(S): Among Wave 1 (W1) any past 30-day (P30D) cigarette users, more than 60%, persistently used cigarettes across three waves in all age groups. Exclusive cigarette use was more common among adult 25+ W1 P30D cigarette users (62.6%), while cigarette polytobacco use was more common among youth (57.1%) and young adults (65.2%). Persistent exclusive cigarette use was the most common pathway among adults 25+ and young adults; transitioning from exclusive cigarette use to cigarette polytobacco use was most common among youth W1 exclusive cigarette users. For W1 youth and young adult cigarette polytobacco users, the most common pattern of use was persistent cigarette polytobacco use. CONCLUSION(S): Cigarette use remains persistent across time, regardless of age, with most W1 P30D smokers continuing to smoke at all three waves. Policy efforts need to continue focusing on cigarettes, in addition to products such as electronic nicotine delivery systems that are becoming more prevalent.Copyright © Author(s) (or their employer(s)) 2020. No commercial re-use. See rights and permissions. Published by BMJ.

4694	Clendennen, Stephanie L., Loukas, Alexandra, Creamer, MeLisa R., Pasch, Keryn E., Perry, Cheryl L. (2019) Longitudinal Patterns of Multiple Tobacco and Nicotine Product Use Among Texas College Students: a Latent Transition Analysis. Prevention science : the official journal of the Society for Prevention Research; 20<br><br>**Keywords:**<br>Adolescent Adult Electronic Nicotine Delivery Systems Female Humans Latent Class Analysis Male *Students Surveys and Questionnaires Texas/ep [Epidemiology] *Tobacco Use/ep [Epidemiology] Universities Young Adult<br><br>**Abstract:** Diverse tobacco and nicotine products have altered the terrain of tobacco use behaviors. Limited research has examined contemporary patterns of use among young adults. This study identified tobacco and nicotine product use groups and examined changes in young adults' use patterns, across a 1.5-year period. Participants were 5,482 18-29-year-old students (M age = 20.5, SD = 2.36; 63% female) from 24 Texas colleges who completed a four-wave bi-annual online survey. Latent transition analysis was used to identify groups from 10 items (ever and current use of cigarettes, cigars, smokeless tobacco, e-cigarettes, and hookah) and to examine probabilities of transitioning between groups over four waves. Five groups were identified: Non-users (30%), Poly-experimenters (26%), Hookah experimenters (18%), E-cigarette & hookah experimenters (15%), and Poly-cigarette users (11%). Few students transitioned between groups over time. Poly-cigarette users had the highest average probability of remaining stable over time (1.00), followed by E-cigarette & hookah experimenters (.97), Non-users (.94), Poly-experimenters (.93), and Hookah experimenters (.92). All groups became more stable over time except Hookah experimenters whose members were most likely to transition to Poly-cigarette users or other experimenter groups. The greatest transition was from Poly-experimenters to Poly-cigarette users with probabilities of .10, .08, and .03 for transitioning between waves one and two, two and three, and three and four, respectively. There was substantial poly-use and experimentation, which may explain little movement between groups over the 1.5-year time period and underscores the need for prevention programs targeting multiple product use among college students.
4695	Stanton, C. A., Sharma, E., Edwards, K. C., Halenar, M. J., Taylor, K. A., Kasza, K. A., Day, H., Anic, G., Gardner, L. D., Hammad, H. T., Bansal-Travers, M., Limpert, J., Borek, N., Kimmel, H. L., Compton, W. M., Hyland, A. (2020) Longitudinal transitions of exclusive and polytobacco electronic nicotine delivery systems (ENDS) use among youth, young adults and adults in the USA: findings from the PATH Study Waves 1-3 (2013-2016). Tobacco control; 29<br><br>**Keywords:**<br>adult article cohort analysis controlled study *electronic cigarette female health education human human experiment *juvenile major clinical study male prevalence *tobacco use young adult *nicotine<br><br>**Abstract:** OBJECTIVE: Electronic nicotine delivery systems (ENDS; including e-cigarettes) are rapidly evolving in the US marketplace. This study reports cross-sectional prevalence and longitudinal pathways of ENDS use across 3 years, among US youth (12-17 years), young adults (18-24 years) and adults 25+ (25 years and older). DESIGN: Data were from the first three waves (2013-2016) of the Population Assessment of Tobacco and Health Study, a nationally representative, longitudinal cohort study of US youth and adults. Respondents with data at all three waves (youth, n=11 046; young adults, n=6478; adults 25+, n=17 188) were included in longitudinal analyses. RESULT(S): Weighted cross-sectional ever use of ENDS increased at each wave. Across all three waves, young adults had the highest percentages of past 12-month, past 30-day (P30D) and daily P30D ENDS use compared with youth and adults 25+. Only about a quarter of users had persistent P30D ENDS use at each wave. Most ENDS users were polytobacco users. Exclusive Wave 1 ENDS users had a higher proportion of subsequent discontinued any tobacco use compared with polytobacco ENDS users who also used cigarettes. CONCLUSION(S): ENDS use is most common among young adults compared with youth and adults 25+. However, continued use of ENDS

	over 2 years is not common for any age group. Health education efforts to reduce the appeal and availability of ENDS products might focus on reducing ENDS experimentation, and on reaching the smaller subgroups of daily ENDS users to better understand their reasons for use.Copyright © Author(s) (or their employer(s)) 2020. No commercial re-use. See rights and permissions. Published by BMJ.
4745	Rogers, S. M., Loukas, A., Harrell, M. B., Chen, B., Springer, A., Perry, C. L. (2020) Multidirectional Pathways of Tobacco and Marijuana Use, Including Comorbid Use, Among Young Adults (Aged 18-25 Years) in Texas: A Six-Wave Cross-Lagged Model. Journal of Adolescent Health;<br><br>**Keywords:**<br>adult adulthood article *cannabis use college student controlled study *electronic cigarette ethnicity female human major clinical study male race *Texas *tobacco use young adult *cannabis<br><br>**Abstract:** Purpose: Prevalence of past 30-day tobacco and marijuana use are highest among young adults in the U.S., and comorbid use of these products is common. However, it is unclear whether the use of one product precedes the other-an important consideration, given the new climate surrounding marijuana legalization and the popularity of e-cigarette products. Method(s): Six-panel cross-lagged regression models, with 6 months between each panel/wave, were used to model bidirectional paths between past 30-day use and comorbid use of marijuana and tobacco products from 2014 to 2017 among young adults (N = 5,221 college students, aged 18-25 years). Result(s): Combustible tobacco use and e-cigarette use were prospectively associated with greater risk of future marijuana use while controlling for the stability of use over time, age, race/ethnicity, and sex. Marijuana use was prospectively associated with greater risk of future combustible tobacco and e-cigarette use. Comorbid use was common, but while the odds of comorbid use decreased with increasing age, odds of tobacco use increased. Conclusion(s): More young adults may be consolidating their use than are successfully ceasing their tobacco or marijuana use as they mature through young adulthood. Although we observed decreases in comorbid use of combustible tobacco with marijuana and e-cigarettes with marijuana, we simultaneously observed strong stability in single use of these products over time. Taken together, these results should help structure interventions specific to young adults that target use and comorbid use in early young adulthood to discourage initiation, encourage cessation, and curb subsequent escalation in later young adulthood.Copyright © 2020 Society for Adolescent Health and Medicine
4764	Jankowski, M., Brozek, G., Krzystanek, M., Lawson, J., Zejda, J. (2019) Nicotine dependence levels among e-cigarettes users. European Respiratory Journal; 54<br><br>**Keywords:**<br>adult cigarette smoking conference abstract controlled study *electronic cigarette female habit human major clinical study male people by smoking status questionnaire satisfaction smoking cessation *tobacco dependence young adult nicotine<br><br>**Abstract:** Electronic cigarettes (e-cigarettes) are advertised as a safer alternative to conventional cigarettes. The potential role of e-cigarettes in smoking cessation is still unknown. The aim of this study was to assess nicotine dependence levels among exclusive e-cigarette users and dual users and to compare them with cigarette smokers. A group of 90 young adults (age: 22.4 +/- 2.2 years) was divided into 3 sub-groups (30 participants/each) depending on self-reported smoking status: cigarette smokers (C), exclusive e-cigarette users (E) and dual users (D). A questionnaire was used to collect information related to the habits of cigarette or e-cigarette use. Nicotine dependence was measured using the Fagerstrom test for nicotine dependence (FTND) and its adapted versions for e-cigarettes. Nicotine content in the e-cigarette, the number of e-cigarettes used, and the e-liquid consumption did not differ (p>0.05) between exclusive e-cigarette users and dual users. Among dual users, the higher satisfaction level was declared when smoking cigarettes (46.2%) compared to using an e-cigarette (23%). The nicotine dependence level (FTND score) was almost two times higher in exclusive e-cigarette users (mean 3.54) compared to conventional cigarette smokers (mean 1.64, p<0.001). In a group of dual users, the nicotine

	dependence level was also higher when using an e-cigarette (mean 4.68) than conventional cigarette smoking (3.2, p=0.03). A stronger nicotine dependence when using e-cigarettes compared to conventional cigarettes suggests that ecigarettes should not be recommended as a potential smoking cessation tool.
4808	Parks, M. J., Davis, L., Wilhelm, A. K., McMorris, B. J., Borowsky, I. W., Shlafer, R. J. (2020) Parental incarceration and youth tobacco product use: Implications for prevention and the e-cigarette epidemic. Addictive Behaviors; 107<br><br>**Keywords:**<br>adolescent *adolescent smoking adult article child child protection childhood adversity demography descriptive research ethnic difference female health care survey health promotion high risk population human male Minnesota *parent *preventive medicine *<br><br>**Abstract:** Purpose: Youth smokers have high rates of adverse childhood experiences, and particularly parental incarceration (PI). In Minnesota, 16% of youth have experienced PI, but 55% of daily smokers report PI. However, no research has examined how PI relates to a range of tobacco products, which is critical considering the current e-cigarette epidemic. There is also limited research on protective factors for tobacco use among youth with PI. Method(s): Data came from 2016 Minnesota Student Survey (N = 111,091); 85% of Minnesota schools participated. We assessed 30-day use of cigarettes, non-cigarette combustible products, smokeless products, e-cigarettes, and dual/poly use. Using descriptive statistics and logistic regressions, we compared use across current, previous, and no PI experience. We also tested how protective factors related to tobacco use. Result(s): Youth with current PI experience used all products with higher frequency compared to youth with previous and no PI experience. Use prevalence among youth with current PI were 26.0% (95% CI = 24.1, 27.8) for e-cigarettes, 20.8% (95% CI = 19.1, 22.5) for dual/poly use, 17.8% (95% CI = 16.2, 19.5) for cigarettes, 17.4% (95% CI = 15.8, 19.0) for combustible non-cigarettes, and 9.9% (95% CI = 8.6, 11.2) for smokeless products. Nearly all protective factors were significantly and negatively related to use of all products, regardless of PI experience. Conclusion(s): Youth with PI experience are at high risk for using multiple tobacco products. These disparities were most pronounced for e-cigarettes, demonstrating the e-cigarette epidemic is disproportionately occurring among youth with current and previous PI experience. All examined protective factors buffer risks for this population of youth.Copyright © 2020 Elsevier Ltd
4813	Antin, T. M. J., Hess, C., Kaner, E., Lipperman-Kreda, S., Annechino, R., Hunt, G. (2020) Pathways of Nicotine Product Use: A Qualitative Study of Youth and Young Adults in California. Nicotine & tobacco research : official journal of the Society for Research on Nicotine and Tobacco; 22<br><br>**Keywords:**<br>adolescent adult article attention *California clinical article controlled study female harm reduction human interview *juvenile male narrative public health *qualitative research risk factor smoking cessation vaping young adult *nicotine<br><br>**Abstract:** INTRODUCTION: Existing research on youth's adoption of alternative nicotine delivery systems (ANDS) has focused on identifying pathways of nicotine product use, specifically examining whether vaping encourages progression to smoking. Few studies have considered other pathways of initiation. Qualitative studies suggest that meanings of vaping vary significantly, suggestive of the need for a more nuanced understanding of the role of vaping for youth with different pathways into vaping and smoking. METHOD(S): We conducted in-depth qualitative interviews with 49 Californian youth between 15 and 25 years old who reported ever vaping nicotine to gain a deeper understanding of their initiation pathways of vaping and smoking, paying special attention to youth's experiences and reasons for ANDS initiation and use. Categorizing participants into initiation pathways by self-reported use and age of initiation of ANDS and cigarettes, we then compared the meaning and role of vaping across three distinct pathways of use: (1) smoking to vaping, (2) vaping to smoking, and (3) vaping only. RESULT(S): The most common

	pathway reported was smoking to vaping (74%), eight participants began vaping before smoking, and five participants reported only vaping but never smoking. Analysis of participants' narratives emphasized that youth in our study, regardless of initiation pathway, were generally aware of the health consequences of smoking and negotiated their use of nicotine products considering relative risks. CONCLUSION(S): Findings from this study suggest that ANDS serve as a transitional tool for youth who are keenly aware of the health consequences of smoking, thus challenging conventional discourses about ANDS as a threat to youth's health. IMPLICATIONS: This qualitative study queries concerns about the potential of alternative nicotine delivery systems (ANDS) to serve as a gateway into cigarette smoking for youth and young adults. Findings suggest that most of the youth participants discussed and considered relative risks in their pathways of initiation, highlighting the need to acknowledge harm reduction in constructing public health messaging and policies for smoking cessation.Copyright © The Author(s) 2019. Published by Oxford University Press on behalf of the Society for Research on Nicotine and Tobacco. All rights reserved. For permissions, please e-mail: journals.permissions@oup.com.
4821	Sharan, R. N., Chanu, T. M., Chakrabarty, T. K., Farsalinos, K. (2020) Patterns of tobacco and e-cigarette use status in India: A cross-sectional survey of 3000 vapers in eight Indian cities. Harm Reduction Journal; 17<br><br>**Keywords:**<br>adult article controlled study cross-sectional study female health belief health hazard human India interview male patient-reported outcome public health smokeless tobacco smoking smoking cessation *tobacco use *vaping nicotine *electronic cigarette<br><br>**Abstract:** Background: Tobacco smoking is one of the biggest and avoidable public health threats in the world, especially in low- and middle-income countries. India represents a highly complex public health environment due to the large number of smokers and complexities arising from tobacco use in different forms, including a variety of smokeless tobacco (SLT) products. Electronic cigarettes, an alternative nicotine delivery system with significantly less harmful emissions than smoke, could be an option for those who are unable to achieve smoking abstinence using other available means. This study, which we believe is the first of this kind in India, was conducted to obtain data on the characteristics and tobacco use profile of e-cigarette users (vapers) in India. Method(s): An interview-based survey was performed in the 8 largest metropolitan cities in India using a convenience sampling approach involving a total of 3000 subjects. Inclusion criteria were being a current e-cigarette user and aged 18 years or more. Interviewers were asked to continue recruitment until a total sample of 375 was reached in each target city. Result(s): A total of 3000 vapers (81.4% males and 18.6% females, median age 29 years) participated to the study. The majority (80%) were first exposed to nicotine via tobacco smoking, SLT use, or both. Most of the subjects (79%) believed that e-cigarettes were less harmful than smoking. The vast majority of smokers (71.3%) reported smoking cessation (30.0%) or reduction in consumption (41.3%) with the help of e-cigarettes. Similar changes were observed in SLT users. Participants reported minimal side effects and some health benefits after e-cigarette use initiation. Conclusion(s): Indian vapers who participated to this study were predominantly smokers and SLT users before e-cigarette use initiation, with the majority subsequently quitting or reducing tobacco use. Minimal side effects of e-cigarette were experienced, while some health benefits were also reported.Copyright © 2020 The Author(s).
4833	Xiao, Lin, Parascandola, Mark, Wang, Congxiao, Jiang, Yuan (#year#) Perception and Current Use of E-cigarettes Among Youth in China. Nicotine & tobacco research : official journal of the Society for Research on Nicotine and Tobacco; 21<br><br>**Keywords:**<br>Adolescent China/ep [Epidemiology] Cross-Sectional Studies Female Health Knowledge, Attitudes, Practice Humans Male Smokers/px [Psychology] Smokers/sn [Statistics & Numerical Data] *Smokers Surveys and Questionnaires Vaping/ep [Epidemiology] Vaping/px [PY - 2019

	**Abstract:** INTRODUCTION: This study provides nationally representative estimates of electronic cigarette (e-cigarette) use among youth in China and explores the factors associated with awareness and use of e-cigarettes and the relationship between e-cigarette and conventional tobacco use., METHODS: This study examined data from the Global Youth Tobacco Survey, which was completed by 155 117 middle school students (51.8% boys and 48.2% girls) in China, and employed a multistage stratified cluster sampling design. For data analysis, SAS 9.3 complex survey procedures were used, and logistic regression was used to explore factors associated with e-cigarette use and the relationship between e-cigarette and conventional tobacco use., RESULTS: About 45.0% of middle school students had heard of e-cigarettes, but only 1.2% reported using e-cigarettes in the last 30 days. Among never-smokers, e-cigarette users were more likely to intend to use a tobacco product in the next 12 months than nonusers (adjusted odds ratio [OR] = 6.970, 95% confidence interval [CI] = 4.474% to 10.857%), and more likely to say that they would enjoy smoking a cigarette (adjusted OR = 14.633, 95% CI = 11.328% to 18.902%). E-cigarette use was associated with previous experimentation with cigarette smoking (OR = 3.2), having noticed tobacco advertising in the past 30 days (OR = 2.7), having close friends who smoke (OR = 1.4), and thinking tobacco helps people feel more comfortable in social situations (OR = 3.3) and makes young people look more attractive (OR = 1.3)., CONCLUSIONS: E-cigarette use among youth in China remains low but awareness is high. E-cigarette use was associated with increased intentions to use tobacco. Enhanced prevention efforts are needed targeting e-cigarette use among youth., IMPLICATIONS: This study is the first nationally representative survey of e-cigarette use among youth in China. It found that among middle school students, prevalence of e-cigarette use is 1.2% and prevalence of e-cigarette awareness is 45.0%. Chinese youths use e-cigarettes as a tobacco product rather than an aid to quitting. Among never-smokers, e-cigarette users were more likely to have intentions to use a tobacco product in the next 12 months, more likely to use a tobacco product offered by their best friends and enjoy smoking a cigarette than nonusers. Copyright © The Author(s) 2018. Published by Oxford University Press on behalf of the Society for Research on Nicotine and Tobacco. All rights reserved. For permissions, please e-mail: journals.permissions@oup.com.
4876	Dobbs, P. D., Hodges, E. J., Dunlap, C. M., Cheney, M. K. (2020) Potential risk factors for cigarette use among a sample of college JUUL users. Journal of American college health : J of ACH;<br><br>**Keywords:**<br>adult article *cigarette smoking *college student controlled study *electronic cigarette human human tissue longitudinal study major clinical study *risk factor<br><br>**Abstract:** OBJECTIVE: To examine the relationship between demographics, other tobacco use, and JUUL dependency on combustible cigarette use among college JUUL users. Participant(s): Undergraduates (n=595) at a large southwestern university who used JUUL weekly completed a cross-sectional online survey in March 2019. Method(s): Logistic regressions examined associations between covariates and ever use/past 30-day use of cigarettes. Result(s): As age increased, odds of ever trying a cigarette increased (AOR = 1.87; 95% CI = 1.45-2.41); however, as age of JUUL initiation increased, odds of ever (AOR = 0.80; 95% CI = 0.65-0.99) or past 30-day (AOR = 0.78; 95% CI = 0.63-0.97) cigarette use decreased. Those moderately (AOR = 2.03; 95% CI = 1.07-3.82) or highly (AOR = 8.01; 95% CI = 3.08-20.83) dependent on JUUL were more likely to have tried cigarettes than those not dependent. However, dependence was not associated with past 30-day use. Conclusion(s): JUUL users may not transition to combustible cigarettes during college. Longitudinal studies are needed to examine transitions in JUUL users' tobacco product use after college.
4884	Patino-Maso, J., Font-Mayolas, S., Salamo, A., Arboix, M., Sullman, M. J. M., Gras, M. E. (2019) Predictors of intentions to use cigarettes and electronic-cigarettes among high school students. Journal of Multidisciplinary Healthcare; 12<br><br>**Keywords:**<br>adolescent article Catalonia child *cigarette smoking cross-sectional study family female friend health

	program health survey *high school student home environment human male *predictor variable prevalence risk assessment school child smoking prevention<br><br>**Abstract:** Purpose: Tobacco is the second most commonly used drug by Spanish high school students and e-cigarette use has increased over the last three years among this population. Students who reported smoking tobacco and/or e-cigarette use often have friends and family that also consume these substances. The aims of the present study were to: determine the prevalence of tobacco and electronic cigarette consumption among young adolescents, the age of initiation and the intention to consume both of these substances in the future; study the relationships between their current tobacco and e-cigarette consumption status, intentions to consume these substances in the future and the consumption of these substances by family members living in their home and friend's consumption status; investigate the relationship between their current tobacco and e-cigarette consumption status, friends' consumption status and their intention to consume each substance in the future; analyse the relationship between current consumption status, intentions to consume in the future and perceived risk; identify the main predictors of intentions to use these substances in the future. Method(s): A cross-sectional survey was conducted in 2017. The sample consisted of 468 school students in the first cycle of secondary education in Catalonia, Spain. The participants who were aged between 11 and 15 years old, with 49.4% being female. Result(s): A stepwise binary logistic regression showed that the best predictors of the intention to smoke tobacco in the future were: having tried tobacco, being a current consumer, having a family member smoking at home and having a low perceived risk of smoking. The best predictors of the intention to use electronic cigarettes in the future were: having tried tobacco, being a current consumer and having friends who smoke or use electronic cigarettes. Conclusion(s): Smoking prevention programs must consider the social network surrounding teenagers, particularly close friends and family. Intervention strategies for delaying the onset of tobacco or e-cigarette consumption are greatly needed.Copyright © 2019 Patino-Maso et al.
4885	Case, K. R., Obinwa, U. C., Clendennen, S. L., Perry, C. L., Harrell, M. B. (2020) Predictors of JUUL, other electronic nicotine delivery systems, and combustible tobacco initiation among Texas youth. Preventive Medicine; 138<br><br>**Keywords:**<br>adolescent *adolescent smoking adult alcohol consumption article cannabis use female health belief human male marketing peer pressure priority journal sensation seeking Texas tobacco use *vaping *electronic cigarette<br><br>**Abstract:** The objectives of this study were to 1) examine longitudinal predictors of JUUL and other tobacco product initiation, 2) compare these predictors across product type, and 3) describe cross-sectional characteristics of JUUL initiators, among a cohort of Texas adolescents. Analyses were also stratified to examine whether predictors of initiation differed by susceptibility to tobacco use at baseline. This study utilized data from Waves 7 and 8 (Fall 2017 and Spring 2018) of the Texas Adolescent Tobacco and Marketing Surveillance System (n = 2272). Chi-square tests and multinomial logistic regressions were conducted to examine differences in predictors of initiation. Among those who initiated at Wave 8 (n=107), 40.2% initiated JUUL, 43.9% initiated other ENDS, and 15.9% initiated other combustible tobacco. For the full sample, ever marijuana use predicted the initiation of all tobacco products (Relative Risk Ratios "RRRs" from 2.31-4.13) as compared to non-users. For non-susceptible youth, ever marijuana use significantly predicted the initiation of JUUL (RRR = 10.08, 95% CI = 2.11-48.17) and other ENDS use (RRR = 12.07, 95% CI = 2.97-49.04). Peer tobacco use predicted the initiation of JUUL (RRR = 3.06, 95% CI = 1.38-6.81) and other ENDS use (RRR = 5.36, 95% CI = 2.11-13.64) for the full sample, as well as those who were susceptible to tobacco use. For non-susceptible youth, peer tobacco use predicted the initiation of combustible tobacco use (RRR = 16.56, 95% CI = 1.56-175.84). Prominent reasons for JUUL use included curiosity, friend use, and less harmful that cigarettes. Results highlight the role of marijuana in the initiation of all tobacco products, even among low-risk youth; other predictors varied between

	product type. Interventions should address specific predictors to prevent youth from transitioning to tobacco use.Copyright © 2020 Elsevier Inc.
4913	Soteriades, Soteris, Barbouni, Anastasia, Rachiotis, George, Grevenitou, Panoraia, Mouchtouri, Varvara, Pinaka, Ourania, Dadouli, Katerina, Hadjichristodoulou, Christos (2020) Prevalence of Electronic Cigarette Use and its Determinants among 13-to-15-Year-Old Students in Greece: Results from the 2013 Global Youth Tobacco Survey (GYTS). International journal of environmental research and public health; 17<br><br>**Keywords:**<br>Adolescent Cross-Sectional Studies *Electronic Nicotine Delivery Systems/sn [Statistics & Numerical Data] Female Greece Humans Male Prevalence Students Surveys and Questionnaires Tobacco *Tobacco Products *Vaping/ep [Epidemiology]<br><br>**Abstract:** Electronic cigarette use has increased over the past decade. Its potential role in smoking cessation, in addiction and as a 'gateway' to tobacco smoking is subject to intense research. This cross-sectional study, carried out in 2013, aims to present the habits of students aged 13-15 in Greece with regard to e-cigarettes and investigate potential risk factors and the relationship between e-cigarettes, tobacco smoking and other nicotine products. It is the first such study to be carried out in Greece. The survey was based on the standardized methodology of the Global Youth Tobacco Survey. All potential associations were investigated using multiple logistic regression. In total 2.8% of the 4096 participating students were current e-cigarette users and 12.3% of them were ever users. E-cigarette use was associated with male gender, being older, current use of combustible tobacco products and e-cigarette use in the family. Ever e-cigarette use, being older, female gender and higher pocket money were associated with an increased susceptibility to tobacco smoking. E-cigarette prevalence in Greece in 2013 was similar to that of other, developed countries. The smoking and vaping habits of adolescents must be monitored in order to assess trends over time and whether any policy alterations are necessary.
4933	Evans-Polce, R. J., Patrick, M. E., McCabe, S. E., Miech, R. A. (2020) Prospective associations of e-cigarette use with cigarette, alcohol, marijuana, and nonmedical prescription drug use among US adolescents. Drug and Alcohol Dependence; 216<br><br>**Keywords:**<br>adolescent adult adulthood *alcohol consumption article *cannabis use controlled study cross-sectional study female follow up human incidence major clinical study male secondary prevention *vaping *alcohol *cannabis *prescription drug<br><br>**Abstract:** Background: As e-cigarette use continues to increase in the U.S., research is needed to understand its prospective risk for cigarette smoking and other substance use in young adulthood, including alcohol, marijuana, and nonmedical prescription drugs (NMPDs). Method(s): This study used data from the Monitoring the Future (MTF) study a nationally representative annual survey of 12th graders (modal age 18) in the US. The analytic sample included 2014-2016 MTF cohorts that were selected and completed follow up one year later (modal age 19; n = 717). Using logistic regression, we examined cross-sectional and prospective associations of past 30-day e-cigarette use with past 30-day cigarette, alcohol, marijuana, and NMPD use. We examined prospective associations among the full sample and associations with incidence of each of these substances among those who reported no history of use in 12th grade. Result(s): In cross-sectional analysis, those who reported past 30-day e-cigarette use at age 18 were more likely to report past 30-day cigarette use, alcohol use, marijuana use, and NMPD use at age 19. In multivariable longitudinal analysis, past 30-day e-cigarette users at age 18 were more likely to report past 30-day cigarette, marijuana, and NMPD use at age 19, including e-cigarette users who had no history of using these substances at age 18. Conclusion(s): This study suggests that e-cigarette use may be an indicator of future substance use risk in young adulthood. Adolescent e-cigarette users may benefit from secondary prevention efforts to mitigate this risk.Copyright © 2020 Elsevier B.V.

4956	Brown, R., Bauld, L., e Lacy, E., Hallingberg, B., Maynard, O., McKell, J., Moore, L., Moore, G. (2020) A qualitative study of e-cigarette emergence and the potential for renormalisation of smoking in UK youth. International Journal of Drug Policy; 75<br><br>**Keywords:**<br>adult article awareness controlled study England flavor human interview *juvenile marketing perception prevalence *qualitative research Scotland social behavior *vaping Wales nicotine<br><br>**Abstract:** Background: Growth of e-cigarette use among smokers has raised concerns over uptake by non-smokers, particularly young people. Legislative changes aimed in part at reducing youth exposure to e-cigarettes include the EU Tobacco Products Directive (TPD). A core justification for such measures is the belief that e-cigarettes can lead to tobacco smoking through mechanisms of renormalisation including: mimicking and normalizing the act of smoking; increasing product acceptability via marketing; nicotine exposure. These mechanisms are here explored in relation to findings from qualitative research. Method(s): This paper reports results from twenty-one group interviews with 14-15 year olds in Wales, England and Scotland, conducted as part of an ongoing evaluation of the impact of the TPD on youth smoking and e-cigarette use. Interviews were conducted around the end of the transitional period for TPD implementation, and explored perceptions of e-cigarettes and tobacco, as well as similarities and differences between them. Result(s): Young people differentiated between tobacco and e-cigarettes, rejecting the term e-cigarette in favour of alternatives such as 'vapes'. Experimental or occasional use was common and generally approved of where occurring within social activity with peers. However, regular use outside of this context was widely disapproved of, unless for the purpose of stopping smoking. Increased prevalence of e-cigarettes did not challenge strongly negative views of smoking or reduce perceived harms caused by it, with disapproval of smoking remaining high. Nicotine use was variable, with flavour a stronger driver for choice of e-liquid, and interest more generally. Conclusion(s): The extent to which participants differentiated between vaping and smoking, including styles and reasons for use in adults and young people; absence of marketing awareness; and continued strong disapproval of smoking provides limited support for some of the potential mechanisms through which e-cigarettes may renormalise smoking. However caution over nicotine exposure is still necessary.Copyright © 2019 The Authors
4963	Stokes, A., Wilson, A. E., Lundberg, D. J., Xie, W., Berry, K. M., Fetterman, J. L., Harlow, A. F., Cozier, Y. C., Barrington-Trimis, J. L., Sterling, K. L., Benjamin, E. J., Blaha, M. J., Hamburg, N. M., Bhatnagar, A., Robertson, R. M. (2020) Racial/Ethnic Differences in Associations of Noncigarette Tobacco Product Use with Subsequent Initiation of Cigarettes in US Youths. Nicotine & tobacco research : official journal of the Society for Research on Nicotine and Tobacco;<br><br>**Keywords:**<br>adolescent article child cigar *cigarette smoking controlled study electronic cigarette *ethnic difference follow up Hispanic human *juvenile school child smoking<br><br>**Abstract:** INTRODUCTION: Understanding which noncigarette tobacco products precede smoking in youth across different racial/ethnic groups can inform policies that consider tobacco related health disparities. METHOD(S): We used nationally representative, longitudinal data from the Population Assessment of Tobacco and Health Study waves 1-4. The sample was a dynamic cohort of cigarette-naive youth aged 12-17 years. Mixed effects models were used to assess noncigarette product [e-cigarette, cigar product, or other product] use with cigarette use over 1-year intervals. RESULT(S): Of the 28,788 observations pooled across waves 1-4, respondents were 48.7% non-Hispanic white, 13.9% non-Hispanic black, and 23.1% Hispanic. Odds of cigarette initiation over 1-year follow up were higher among youth with prior use of e-cigarettes (OR, 2.76; 95% CI, 2.21-3.45), cigars (OR, 2.00; 95% CI, 1.42-2.80), or other products (OR, 1.66; 95% CI, 1.28-2.14) compared to never users. At the population level, 20.6% of cigarette initiation was attributable to e-cigarette use among white youth and 21.6% among Hispanic

	youth, while only 3.5% of cigarette initiation was attributable to e-cigarette use among black youth. In contrast, 9.1% of cigarette initiation for black youth was attributable to cigar use compared to only 3.9% for both white and Hispanic youth. CONCLUSION(S): Prior use of e-cigarettes, cigars, and other noncigarette products were all associated with subsequent cigarette initiation. However, white and Hispanic youth were more likely to initiate cigarettes through e-cigarette use (vs. cigar or other product use), while black youth were more likely to initiate cigarettes through cigar use (vs. e-cigarette or other product use). IMPLICATIONS: Our findings suggest that previous studies on effects of noncigarette tobacco products may overlook the critical role of cigar products as a pathway into cigarette smoking among US youth, particularly black youth. While our data support the importance of e-cigarette use as a pathway into smoking, regulatory actions aimed at addressing youth e-cigarette use alone may contribute to disparities in black vs. white tobacco use and further exacerbate inequities in tobacco-related disease. Thus, contemporary policy development and discourse about the effects of noncigarette tobacco products on cigarette initiation should consider cigar and other noncigarette products as well as e-cigarettes.Copyright © The Author(s) 2020. Published by Oxford University Press on behalf of the Society for Research on Nicotine and Tobacco.
4977	Hiler, M., Spindle, T. R., Dick, D., Eissenberg, T., Breland, A., Soule, E. (2019) Reasons for Transition From Electronic Cigarette Use to Cigarette Smoking Among Young Adult College Students. Journal of Adolescent Health;<br><br>**Keywords:**<br>adult anxiety article *college student concept mapping controlled study female human longitudinal study major clinical study male multidimensional scaling social behavior stigma stress *vaping young adult<br><br>**Abstract:** Purpose: Longitudinal studies indicate that e-cigarette use among youth and young adults is associated with cigarette smoking initiation. The purpose of this study was to identify reasons why nonsmoking young adults transition from e-cigarette use to cigarette smoking. Method(s): The study used concept mapping (CM), a mixed-method participatory approach. Fifty-five college students who endorsed initiation of e-cigarettes before cigarettes (lifetime e-cigarette uses >= 100 and >= 100 cigarettes in lifetime) completed at least one part of the study. In an online program, participants brainstormed (n = 54) statements describing reasons for transition from e-cigarette use to cigarette smoking, sorted statements (n = 46) into conceptually similar categories, and rated (n = 47) how true each statement was for them. Result(s): Participants generated 60 unique statements, and multidimensional scaling analysis generated eight thematic clusters characterizing reasons for transition which included the following: "Sharing with Others," "Psychological Coping," "Cigarette Appeal," "Reinforcing Effects of Cigarettes," "Accessibility," "Social Influence," "Vaping Stigma," and "Vaping Deficiencies." Participants rated "Sharing with Others" and "Psychological Coping" highest (most true) and "Vaping Deficiencies" lowest (least true). For college students, the ability to share cigarettes with peers and access cigarettes from peers and smoking for stress/anxiety management were among the top reasons for transition. Conclusion(s): Results suggest that tailored prevention efforts aimed at reducing cigarette smoking uptake among college students who use tobacco as a means for psychological coping or social facilitation may be warranted. Furthermore, regulatory decisions aimed at limiting cigarette appeal, reinforcing effects, and accessibility may be relevant to reducing transition.Copyright © 2019 Society for Adolescent Health and Medicine
5012	Kintz, N., Liu, M., Chou, C. P., Urman, R., Berhane, K., Unger, J. B., Boley Cruz, T., McConnell, R., Barrington-Trimis, J. L. (2020) Risk factors associated with subsequent initiation of cigarettes and e-cigarettes in adolescence: A structural equation modeling approach. Drug and Alcohol Dependence; 207<br><br>**Keywords:**<br>*adolescence article California child child health *cigarette smoking controlled study *electronic cigarette female follow up hookah human major clinical study male marketing *risk factor social environment *structural equation modeling

	**Abstract:** Background: Previous youth tobacco research has identified multiple correlated risk factors for initiation of cigarette and e-cigarette use; whether these factors are independently associated with initiation is not known, due to challenges with disentangling the independent effects of these correlated risk factors. Method(s): Students in 11th/12th grade enrolled in the Southern California Children's Health Study were surveyed in 2014 (baseline) and again in 2015 (N = 1553). Structural equation models (SEM) were developed to investigate associations of susceptibility, marketing, and the social environment (as latent factors), and other tobacco use at baseline with cigarette or e-cigarette initiation between baseline and follow-up. Analyses were restricted to baseline never cigarette users (N = 1293) for models evaluating cigarette initiation, and to never e-cigarette users (N = 1197) for models evaluating e-cigarette initiation. Result(s): In fully-adjusted prospective SEM models, latent factors for cigarette susceptibility, marketing, and the social environment, along with ever e-cigarette use and ever hookah use at baseline were independently associated with cigarette initiation between baseline and follow-up (P < 0.05). Similarly, latent factors for e-cigarette susceptibility, marketing, and the social environment, along with ever hookah use at baseline were associated with e-cigarette initiation between baseline and follow-up (P < 0.05); however, cigarette use at baseline was not associated with e-cigarette initiation in SEM models (P = 0.16). Conclusion(s): We identified independent effects of multiple risk factors in SEM models on initiation of cigarettes and e-cigarettes. E-cigarette use was associated with cigarette initiation, but cigarette use was not associated with e-cigarette initiation in fully adjusted models. Research to identify underlying causal mechanisms is warranted.Copyright © 2019 Elsevier B.V.
5085	Lim, K. H., Ghazali, S. M., Lim, H. L., Cheong, K. C., Teh, C. H., Lim, K. K., Heng, P. P., Cheah, Y. K., Lim, J. H. (2019) Smoking susceptibility among non-smoking school-going adolescents in Malaysia: Findings from a national school-based survey. BMJ Open; 9<br><br>**Keywords:**<br>adolescent *adolescent smoking adult advertising child cross-sectional study female groups by age high school human major clinical study Malaysia male *non-smoker passive smoking prevalence primary school review risk factor school health education sex d<br><br>**Abstract:** Objective The identification of susceptible non-smoking adolescents is an essential step in reducing smoking initiation among adolescents. The aim of this study was to examine the prevalence and factors associated with smoking susceptibility among non-smoking school-going adolescents in Malaysia. Design Cross-sectional study. Setting Primary and secondary schools in Malaysia. Participants 11 246 non-smoking school-going adolescents. Outcome measures The prevalence and factors associated with smoking susceptibility among non-smoking school-going adolescents in Malaysia. Results Approximately 14% of non-smokers were susceptible to smoking, and the prevalence of susceptibility was significantly higher among males, ever-smokers and e-cigarette users. The odds of susceptibility to smoking were higher among males, e-cigarette users, those aged 12 years and under and those who had ever smoked or tried cigarettes. Students from schools with educational programmes on the health effects of second-hand smoke (SHS) and who perceived smoking to be harmful were less likely to be susceptible to smoking. Conclusion Smoking susceptibility is prevalent among school-going adolescents. A comprehensive approach that enhances or reinforces health education programmes on the adverse health effects of smoking and SHS among school children, that considers multiple factors and that involves all stakeholders is urgently needed to reduce the prevalence of smoking susceptibility among vulnerable subgroups, as identified from the present findings.Copyright © Author(s) (or their employer(s)) 2019. Re-use permitted under CC BY-NC. No commercial re-use. See rights and permissions. Published by BMJ.
5092	Chao, Dingding, Hashimoto, Hideki, Kondo, Naoki (2019) Social influence of e-cigarette smoking prevalence on smoking behaviours among high-school teenagers: Microsimulation experiments. PloS one; 14

	**Keywords:**<br>Adolescent *Cigarette Smoking/ep [Epidemiology] *Electronic Nicotine Delivery Systems Female Humans Male Models, Theoretical Prevalence Schools *Social Change *Students<br><br>**Abstract:** The prevalence of electronic cigarette (e-cigarette) use has rapidly increased among young people, while conventional cigarette use has decreased in this age group. However, some evidence suggests that e-cigarette use is likely to induce conventional cigarette smoking. The present study explored the social influence of the prevalence of e-cigarette use in the peer network and in the general population as a potential mechanism by which e-cigarette use affects adolescents' overall smoking behaviours. For this purpose, we developed an agent-based model in which young agents repeatedly choose to smoke conventional cigarettes and/or e-cigarettes, or to remain non-smokers. The choice is based on the agent's evaluation of the utility derived from smoking and attitude towards smoking ('openness'), which is influenced by smoking prevalence in the agent's peer network and in the broader society. We also assumed a 'crossover' effect between the different types of smoking. The model was calibrated with United States National Youth Tobacco Survey data to reflect real-world numbers. We further simulated the prevalence of different types of smoking under counterfactual scenarios with different levels of openness and crossover effects. The models developed successfully reproduced actual prevalence trends in different types of smoking from 2011 to 2014. Openness to smoking is associated with a dramatic increase in e-cigarette smoking and especially in dual smoking, which cancels out the decline in sole conventional smoking. Larger crossover effects are associated with a higher prevalence of conventional smoking. The simulation results indicate that the social influence of the prevalence of e-cigarette use may influence young people to initiate or continue conventional cigarette smoking. Assessing the impact of e-cigarettes in the general population as a 'healthier' alternative to conventional smoking may require carefully monitoring trends in young people's smoking behaviours.
5197	Simon, P., Buta, E., Gueorguieva, R., Kong, G., Morean, M. E., Camenga, D. R., Bold, K. W., Krishnan-Sarin, S. (2020) Transitions across tobacco use profiles among adolescents: results from the Population Assessment of Tobacco and Health (PATH) study waves 1 and 2. Addiction (Abingdon, England); 115<br><br>**Keywords:**<br>adolescent article child cigar *cigarette smoking controlled study electronic cigarette high school hookah human *juvenile male prevalence probability prospective study school child *smokeless tobacco nicotine<br><br>**Abstract:** BACKGROUND AND AIMS: To estimate progression to polytobacco use (PTU) over 1 year among a sample of US youth. DESIGN: Prospective survey with two waves 1 year apart: wave 1 (2013-14) and wave 2 (2014-15). We conducted latent transition analysis (LTA) to identify latent class transitions and examine socio-demographic predictors of transition types. SETTING: United States. PARTICIPANTS: A total of 11, 996 people who were aged 12-17 years at wave 1. MEASUREMENTS: Publicly available data were used from the Population Assessment of Tobacco and Health (PATH) study, a nationally representative sample of US civilian, non-institutionalized population aged 12 years and older. Tobacco use status was assessed and classified in terms of: never use, non-current (not in the past 30 days) and current (past 30-day) use of cigarettes, cigars, e-cigarettes, hookah and smokeless tobacco. Other nicotine products were excluded because rates of use were either too low to model (e.g. pipe) or the product was not assessed in the PATH youth sample (e.g. nicotine replacement products). FINDINGS: We identified three distinct patterns: class 1, non-use (wave 1 prevalence = 86%; wave 2 prevalence = 78%); class 2, ever use of cigarettes and e-cigarettes (wave 1 prevalence = 11%; wave 2 prevalence = 14%); and class 3, current PTU (wave 1 prevalence = 4%; wave 2 prevalence = 7%). Probability of progression from non-use to ever use of cigarettes and e-cigarettes was 0.06 and ever use of cigarettes and e-cigarettes to current PTU was 0.32. Non-users were more likely to transition to ever use of cigarettes and e-cigarettes if they were older (versus younger), white (versus non-white) or if their parental education level was high school or less (versus more than high school); and ever users of cigarettes and e-cigarettes to current PTU if they were older, male or white. CONCLUSION(S): US youth who had previously tried e-cigarettes and cigarettes at

	wave 1 (2013-14) had a 32% chance of transitioning to current use of two or more tobacco products within 1 year.Copyright © 2019 Society for the Study of Addiction.
5207	Evans-Polce, Rebecca, Veliz, Phil, Boyd, Carol J., McCabe, Vita V., McCabe, Sean Esteban (2020) Trends in E-Cigarette, Cigarette, Cigar, and Smokeless Tobacco Use Among US Adolescent Cohorts, 2014-2018. American journal of public health; 110<br><br>**Keywords:**<br>Adolescent Cross-Sectional Studies Health Surveys Humans Prospective Studies *Tobacco Smoking/td [Trends] *Tobacco, Smokeless/sn [Statistics & Numerical Data] United States *Vaping/td [Trends]<br><br>**Abstract:** Objectives. To examine changes in age of initiation of e-cigarette, cigarette, cigar, and smokeless tobacco use among adolescents in the United States. Methods. We used data from 5 cohorts of the National Youth Tobacco Survey (2014-2018; n = 26 662). Results. In 2014, 8.8% of lifetime e-cigarette users initiated use at 14 years or younger, as compared with 28.6% of lifetime e-cigarette users in 2018. There was no such change in initiation ages for cigarettes, cigars, and smokeless tobacco among lifetime users of each of these products. Conclusions. US adolescents are initiating e-cigarette use at younger ages in recent years. This is concerning given the association of e-cigarette use with subsequent cigarette use. Continued surveillance of these trends and additional prospective research are needed. Tobacco prevention programs, policies, and regulations that make it more difficult for youths to obtain e-cigarettes are warranted.
5244	Chyderiotis, S., Spilka, S., Beck, F. (2019) Use of electronic cigarette in France among adolescents aged 17: Results from the ESCAPAD 2017 survey. Bulletin du Cancer; 106<br><br>**Keywords:**<br>adolescent article cannabis use cross-sectional study demography drinking behavior drug use France human smoking vaping water pipe smoking illicit drug *electronic cigarette<br><br>**Abstract:** Introduction: The use of electronic cigarettes has become relatively popular in France since 2010, including among adolescents. However, its use in relation to smoking and other factors is not well understood today. Method(s): The data come from the ESCAPAD 2017 survey, a nationally representative cross-sectional survey taking place at a 1-day session of civic and military information compulsory for all French nationals around 17 (39,115 respondents). Descriptive analyses and multivariate regressions were undertaken to describe the recent use of e-cigarette at 17 and its associated factors. Result(s): e-cigarettes were experimented by 52.4 % of 17 year-olds, and used by 16.8 % in the preceding month, 1.9 % daily. Most recent users were also daily smokers (62.5 %), and only 7.6 % had never experimented cigarettes before. Among those who experimented with both products, only 13.3 % tried e-cigarettes before cigarettes. The associated uses of other products were the most striking factors: daily smoking (relative risk [RR] = 2.73), ever use of hookah (RR = 2.31), cannabis use in the last year (RR = 1.60), regular alcohol drinking (RR = 1.20) and ever use of another illicit drug (RR = 1.11). Recent vapers that were also daily smokers had a more pronounced sociodemographic profile and a higher level of other drugs consumptions than recent vapers only. Discussion(s): Although a majority of French adolescents experiment with vaping, they are fewer to use it regularly and its current use is frequently associated with daily smoking. Future trends and the relationship between smoking and vaping among adolescents will have to be further investigated, including the motivations of its use.Copyright © 2019 Societe Francaise du Cancer
5327	Weaver, S. R., Heath, J. W., Ashley, D. L., Huang, J., Pechacek, T. F., Eriksen, M. P. (2020) What are the reasons that smokers reject ENDS? A national probability survey of U.S. Adult smokers, 2017-2018. Drug and Alcohol Dependence; 211<br><br>**Keywords:**

	adult age article controlled study cost craving cross-sectional study current smoker female harm reduction human male perception priority journal probability questionnaire safety *smoking smoking cessation tobacco dependence United States *electronic ci<br><br>**Abstract:** Background: The objective was to examine the reasons smokers have discontinued or chosen not to use electronic nicotine delivery systems (ENDS). Method(s): Data were obtained from a national probability sample of 1843 US adult current smokers who were not current ENDS users pooled from the 2017 and 2018 annual, cross-sectional Tobacco Products and Risk Perceptions Surveys. Participants reported their ENDS use, reasons for discontinuing or not initiating ENDS use, quit smoking intentions, perceptions, and use intentions. Weighted proportions and logistic regression models were estimated. Result(s): Twenty-three percent of smokers were former ENDS users who reported prior "regular use", and 7.5% were former ENDS users who reported regular use. Three most cited reasons for discontinuing ENDS were: ENDS "didn't feel like smoking" (23%), "only ever tried them to see what they were like" (20%), and "didn't help me deal with cravings for smoking" (14%). Reasons for discontinuing ENDS were associated with the regularity of former ENDS use and ENDS type. Nearly 40% of current smokers had not tried ENDS with the most commonly cited reasons being not wanting to substitute one addiction for another (60%), concerns about their safety (53%), skepticism that ENDS could help them quit smoking (52%), and cost (43%). Reasons were associated with smoking quit intentions, harm perceptions, and age. Conclusion(s): Whereas smokers who had formerly used ENDS cited inadequate craving reduction or incomparability to smoking for their discontinuation, the larger segment of smokers who have never used ENDS cited "safety," "effectiveness," and "costs" as reasons for non-use.Copyright © 2020 The Authors
5361	Niaura, Raymond, Rich, Ilan, Johnson, Amanda L., Villanti, Andrea C., Romberg, Alexa R., Hair, Elizabeth C., Vallone, Donna M., Abrams, David B. (2020) Young Adult Tobacco and E-cigarette Use Transitions: Examining Stability Using Multistate Modeling. Nicotine & tobacco research : official journal of the Society for Research on Nicotine and Tobacco; 22<br><br>**Keywords:**<br>Adolescent Adult *Electronic Nicotine Delivery Systems/sn [Statistics & Numerical Data] Female Humans Male Surveys and Questionnaires *Tobacco Products/sn [Statistics & Numerical Data] *Tobacco Use/px [Psychology] *Vaping/ep [Epidemiology] *Vaping/px [P<br><br>**Abstract:** INTRODUCTION: The objective of this study was to describe tobacco and nicotine product use state transition probabilities among youth and young adults over time., METHODS: A national sample of young adult tobacco product users and nonusers between the ages of 18 and 34 years at baseline was surveyed at 6-month intervals for 3 years. Use and nonuse states were defined as mutually exclusive categories based on self-reported, past 30-day use of the various products. Never use, noncurrent use, and current use of combustible, noncombustible tobacco, and electronic cigarette (e-cigarette) products was assessed at each interval. A multistate model was fit to estimate transition probabilities between states and length of stay within each state., RESULTS: After 6 months, same-state transition probabilities were high for all use states (0.76-0.96), except for dual product use (0.48). After 3 years, transition probabilities were smaller and tended to converge toward combustible product use for baseline e-cigarette (0.42), combustible (0.51), and dual product users (0.52). Age was inversely associated with transition risk from never or noncurrent use to use of combustible or e-cigarette products., CONCLUSIONS: Never and noncurrent users, followed by combustible product users, were most likely to remain in those states throughout the 3-year observation interval. Users of any tobacco or e-cigarette product at baseline were most likely to transition to combustible product use or noncurrent use by the final follow-up., IMPLICATIONS: This study describes the probability of transitioning between various states of tobacco product use, including never and no current use, over a span of 3 years in a sample of young adults. This type of longitudinal description, which includes all tobacco product use states, is lacking in most studies that tend to focus on one or only a few products. The results suggest that it is important to assess outcomes over a sufficiently long period to capture true variability in patterns of

	product use. Copyright © The Author(s) 2019. Published by Oxford University Press on behalf of the Society for Research on Nicotine and Tobacco. All rights reserved. For permissions, please e-mail: journals.permissions@oup.com.
5365	Green, M. J., Gray, L., Sweeting, H. (2020) Youth vaping and smoking and parental vaping: a panel survey. BMC public health; 20<br><br>**Keywords:**<br>article controlled study human *juvenile *parental smoking school child *vaping<br><br>**Abstract:** BACKGROUND: Concerns remain about potential negative impacts of e-cigarettes including possibilities that: youth e-cigarette use (vaping) increases risk of youth smoking; and vaping by parents may have impacts on their children's vaping and smoking behaviour. METHOD(S): With panel data from 3291 youth aged 10-15years from the 7th wave of the UK Understanding Society Survey (2015-2017), we estimated effects of youth vaping on youth smoking (ever, current and past year initiation), and of parental vaping on youth smoking and vaping, and examined whether the latter differed by parental smoking status. Propensity weighting was used to adjust for measured confounders and estimate average effects of vaping for all youth, and among youth who vaped. E-values were calculated to assess the strength of unmeasured confounding influences needed to negate our estimates. RESULT(S): Associations between youth vaping and youth smoking were attenuated considerably by adjustment for measured confounders. Estimated average effects of youth vaping on youth smoking were stronger for all youth (e.g. OR for smoking initiation: 32.5; 95% CI: 9.8-107.1) than among youth who vaped (OR: 4.4; 0.6-30.9). Relatively strong unmeasured confounding would be needed to explain these effects. Associations between parental vaping and youth vaping were explained by measured confounders. Estimates indicated effects of parental vaping on youth smoking, especially for youth with ex-smoking parents (e.g. OR for smoking initiation: 11.3; 2.7-46.4) rather than youth with currently smoking parents (OR: 1.0; 0.2-6.4), but these could be explained by relatively weak unmeasured confounding. CONCLUSION(S): While measured confounding accounted for much of the associations between youth vaping and youth smoking, indicating support for underlying propensities, our estimates suggested residual effects that could only be explained away by considerable unmeasured confounding or by smoking leading to vaping. Estimated effects of youth vaping on youth smoking were stronger among the general youth population than among the small group of youth who actually vaped. Associations of parental vaping with youth smoking and vaping were either explained by measured confounding or could be relatively easily explained by unmeasured confounding.
5400	Ahmed, Luai A., Verlinden, Marina, Alobeidli, Mariam Ali, Alahbabi, Reem Hamad, AlKatheeri, Radeya, Saddik, Basema, Oulhaj, Abderrahim, Al-Rifai, Rami H. (2021) Patterns of Tobacco Smoking and Nicotine Vaping among University Students in the United Arab Emirates: A Cross-Sectional Study. International journal of environmental research and public health; 18<br><br>**Keywords:**<br>United Arab Emirates e-cigarettes electronic nicotine delivery system midwakh smoking Index Medicus Young Adult United Arab Emirates -- epidemiology Cross-Sectional Studies Humans Adult Tobacco Smoking Universities Students Adolescent Male Female Vaping Electronic Nicotine Delivery Systems<br><br>**Abstract:** Various forms of tobacco smoking and nicotine vaping tools are available on the market. This study quantified the prevalence of and identified factors associated with patterns of smoking and nicotine vaping among university students in the United Arab Emirates (UAE). A cross-sectional sample of students enrolled in three public universities was surveyed. Self-reported current smoking and nicotine vaping were recorded. Of 1123 students, 81.7% completed the online survey (mean age, 20.7 ± 3.4 (SD) years; 70.7% females). The prevalence of current smoking was 15.1% while the prevalence of current nicotine vaping was nearly 4.0%. Among current smokers, 54.7% reported conventional smoking only, 15.1% reported nicotine vaping only, and 28.8% were poly-users. Conventional midwakh (47.5%),

	followed by conventional shisha/waterpipe (36.7%), conventional cigarettes (36.7%), electronic shisha/waterpipe (25.2%), and electronic cigarettes (24.5%), were most commonly reported by students. Students aged 20-25 years (adjusted odds ratios (aOR): 2.08, 95% confidence interval (CI): 1.18-3.67) or >25 years (aOR: 4.24, 95% CI: 1.41-12.80) had higher odds of being current smokers compared to those aged 17-19 years. The male gender was also independently associated with higher odds of being a current smoker (aOR: 5.45, 95% CI: 3.31-8.97) as well as higher odds of smoking cigarettes, shisha, and midwakh, or nicotine vaping compared to being female. Of nicotine vaping users, 36.1% reported using nicotine vaping because they enjoyed the flavor and vaporizing experience and 34.4% used it to help them to quit smoking. A relatively high prevalence of self-reported smoking was reported among university students in the UAE. The findings also suggest that nicotine vaping use is relatively widespread, but still less common than traditional smoking. Vigilant and tailored university-based smoking control and preventive measures are warranted.
5411	Al Rifai, Mahmoud, Kianoush, Sina, Saeed, Anum, Krittanawong, Chayakrit, Merchant, Anwar T., Virani, Salim S. (2020) Temporal Trends in the Prevalence of Current E-Cigarette and Cigarette Use by Annual Household Income from 2016 to 2018 (from the Behavioral Risk Factor Surveillance System BRFSS Survey). American Journal of Cardiology; 137<br><br>**Keywords:**<br>Embase MEDLINE 17:Public Health, Social Medicine and Epidemiology United States controlled study cigarette smoking household income vaping smoking cessation prevalence male trend study adult priority journal human population research questionnaire behavioral risk factor surveillance system female self report Letter Hispanic electronic cigarette<br><br>**Abstract:**
5429	Aleyan, Sarah, Ferro, Mark A., Hitchman, Sara C., Leatherdale, Scott T. (2021) Does having one or more smoking friends mediate the transition from e-cigarette use to cigarette smoking: a longitudinal study of Canadian youth. Cancer causes & control : CCC; 32<br><br>**Keywords:**<br>Longitudinal study Mediation Smoking Youth e-cigarettes Index Medicus Cigarette Smoking Vaping Adolescent Behavior Canada Risk Factors Humans Tobacco Longitudinal Studies Friends Adolescent Male Female Electronic Nicotine Delivery Systems<br><br>**Abstract:** PURPOSEStudies have shown consistent associations between youth e-cigarette use and subsequent smoking uptake. However, it remains unclear why, as limited evidence exists regarding the mechanisms underlying these associations. Our study investigated whether having one or more smoking friends mediated the association between e-cigarette use and cigarette smoking onset among a longitudinal sample of Canadian youth who were never smokers at baseline.METHODSA longitudinal sample of youth that participated in three waves of the COMPASS study (2015-2016 to 2017-2018) was identified (N = 5,535). The product of coefficients method was used to assess whether having one or more smoking friends mediated the association between: (1) past 30-day e-cigarette use and cigarette smoking onset and (2) past 30-day e-cigarette use and subsequent dual use of e-cigarettes and cigarettes.RESULTSHaving one or more smoking friends did not mediate the association between (1) past 30-day e-cigarette use and cigarette smoking onset (β = 0.38, 95% CI - 0.12, 0.89) or (2) past 30-day e-cigarette use and subsequent dual use (β = 0.46, 95% CI - 0.16, 1.07). Post hoc tests indicated that smoking friends significantly predicted past 30-day e-cigarette use and cigarette smoking at wave 3 (aOR 1.68 and 2.29, respectively).CONCLUSIONHaving smoking friends did not explain the association between e-cigarette use and smoking uptake despite being a common risk factor for both e-cigarette use and cigarette smoking. Prevention efforts should consider how best to incorporate effective programming to address these social influences.

5436	Ali, Fatma Romeh M., Dave, Dhaval M., Colman, Gregory J., Wang, Xu, Saffer, Henry, Marynak, Kristy L., Dench, Daniel, Grossman, Michael (2021) Association of e-cigarette advertising with e-cigarette and cigarette use among US adults. Addiction; 116<br><br>**Keywords:**<br>E- cigarette advertising e- cigarette use cigarette use Empirical Study Quantitative Study Human Male Female Adulthood (18 yrs & older) Young Adulthood (18-29 yrs) Thirties (30-39 yrs) Middle Age (40-64 yrs) Electronic Cigarettes Tobacco Smoking Awareness Advertising Television Advertising Magazines US article 3383:Drug & Alcohol Rehabilitation<br><br>**Abstract:** Aims: To estimate the association of e-cigarette advertisement exposure with e-cigarette and cigarette use behavior among US adults. Design: Data from the 2013–14 National Adult Tobacco Survey (NATS) were linked to Kantar Media and National Consumer Study data to construct measures of e-cigarette advertisements on TV and in magazines. The relationship between advertisement measures and outcomes was estimated using logistic and Poisson regressions, controlling for socio-demographics, state cigarette taxes and state and year fixed-effects. Setting: United States. Participants/cases: A total of 98 746 adults aged ≥ 18 years who responded to the 2013–14 NATS. Measurements: The independent variables of interest were the number of e-cigarette advertisements in magazines to which an adult was exposed in the past 6 months and the number of e-cigarette advertisements on TV to which an adult was exposed in the past 6 months. Outcomes were awareness of e-cigarettes, ever e-cigarette use, current e-cigarette use, current cigarette use and number of cigarettes smoked per month. Findings: Exposure to one additional e-cigarette advertisement on TV was associated with a 0.18, 0.13 and 0.03 percentage point increase, respectively, in awareness, ever use and current use of e-cigarettes among all adults ($P < 0.05$). This exposure also was associated with a 0.11 percentage point increase in current cigarette use among all adults and an increase in cigarette consumption of 2.24 cigarettes per month among adults aged ≥ 45 ($P < 0.05$). Conclusions: Exposure to e-cigarette advertising appears to be positively associated with the use of e-cigarettes and cigarettes among adults of all ages, and with increased cigarette consumption among older adults. (PsycInfo Database Record (c) 2021 APA, all rights reserved) (Source: journal abstract)
5484	Ashford, Kristin, McCubbin, Andrea, Barnett, Janine, Blair, Lisa M., Lei, Feitong, Bush, Heather, Breland, Alison (2021) Longitudinal Examination of Prenatal Tobacco Switching Behaviors and Birth Outcomes, Including Electronic Nicotine Delivery System (ENDS) and Dual Use. Maternal and child health journal; 25<br><br>**Keywords:**<br>Birth outcomes Electronic cigarettes ENDS Pregnancy Switching behaviors MEDLINE nicotine prematurity -- epidemiology longitudinal study tobacco adult newborn human electronic cigarette<br><br>**Abstract:** INTRODUCTION: In the US, approximately 8% of pregnant women smoke, and 5-11.9% currently use ENDS products. The health effects of ENDS use are debated; however, most contain nicotine which is known to cause adverse perinatal outcomes. Studies have shown adult ENDS users significantly alter use behaviors over time (switch to conventional cigarettes-only or dual use) thus complicating efforts to examine health effects of ENDS use. The purpose of this study was to describe switching behaviors and associated birth outcomes among infants of women using conventional cigarettes only, ENDS-only, or both. METHODS: This was a multisite, longitudinal study of biologically confirmed perinatal tobacco users, with nicotine product use assessed each trimester. For the purpose of analysis, participants were defined as switchers, no-switchers, or quitters. Birth outcomes were abstracted from electronic medical records. Analysis included descriptive statistics, linear and multivariate logistic regression adjusted for age, preterm birth, smoking behavior in the first trimester, and an interaction between smoking switching behavior and smoking behavior in the first trimester. Analysis was conducted using SAS v9.4 with significance determined as $p < 0.05$. RESULTS: At enrollment, 48.6% of participants used only conventional cigarettes, 41.7% were dual users, and 10% used ENDS-only.

	While almost two-thirds of participants used the same tobacco product throughout pregnancy, 26% reported switching behaviors that were complex and not easily clustered. No differences were found in birth outcomes between switchers and no-switchers; however, a difference emerged in birth weight between no-switchers and quitters. DISCUSSION: Given the limited data on health effects of ENDS use, and the known harmful consequences of perinatal nicotine use, capturing and classifying product switching behaviors is imperative to inform public health, and remains a challenge requiring further research.
5491	Auderset, Diane, Barrense-Dias, Yara, Akre, Christina, Suris, Joan-Carles (2020) 66. To Quit or to Start? The Effect of E-Cigarettes Use On Smoking Traditional Cigarettes Among Young People in Switzerland. Journal of Adolescent Health; 66<br><br>**Keywords:**<br>Embase controlled study smoking habit marketing smoking cessation male adult Switzerland smoke conference abstract human non-smoker adolescent female longitudinal study lifestyle major clinical study juvenile counseling electronic cigarette<br><br>**Abstract:** Purpose: The aim of this research was to study trajectories of use of electronic cigarettes (EC) over time among youths distinguishing between EC only and dual users (EC + traditional cigarettes) and to assess reasons to use EC. Particularly, we aimed to observe whether ECs were a gateway to traditional cigarettes (TC) and if they effectively helped in quitting smoking. Methods: Data came from the GenerationFRee survey, a longitudinal study on youths' lifestyles (15-24 years at baseline [T1]) in the canton of Fribourg, Switzerland. Participants (N=1437, mean age 16.7; 51.8% females) were divided at T1 in 4 groups according to their current smoking status: TC-only smokers (N=378, 26.3%; 51.5% females), EC-only (N=42, 2.9%; 27.3% females), dual users (N=97, 6.7%; 39.6% females) and non-smokers (N=920, 64%; 53.4% females). Additionally, participants in the EC-only and dual groups at T1 reported the reasons for EC use. Finally, we determined the trajectories between T1 and T2 (2016 and 2017) by observing in which of the 4 categories EC-only and dual users at T1 were at T2. Results: Males outnumbered females in the use of ECs, alone or dually. Among current EC (alone or dually) users at T1, 79.9% responded having used it to "try", 13.8% to "smoke where TCs were not allowed", 12.8% to "reduce tobacco use" and 8.4% to "stop smoking". Regarding group trajectories, among EC-only users at T1, 23.7% became TC-only smokers, 20.1% remained EC-only users, 4.6% became dual users and 51.5% non-smokers at T2. Among dual users at T1, 68.7% became TC-only smokers, 1.1% EC users only, 17.9% dual users and 12.2% non-smokers at T2. Moreover, the percentage of quitters among dual users was lower than among TC-only users at T2 (12.2% vs. 16.4%). Among dual users indicating wanting to quit as a reason to use ECs, 15.3% effectively quitted smoking, while among those indicating to smoke when TCs were not allowed, none stopped smoking a year later. Conclusions: The vast majority of youths used ECs "to try", suggesting that the aggressive marketing campaigns that target them work. Furthermore, ECs seem to be a gateway to smoking and have little impact on helping to quit. Among EC-only users at baseline, about half maintained their smoking habits a year later implying that ECs are attractive for youth and a gateway to TCs for almost a third of them. As dual users at baseline reported the lowest quitting rate at T2, it suggests that using both products may make it more difficult to quit rather than making it easier, for example allowing to smoke where TCs are prohibited. There is a need to protect youths from the marketing of products that are presented as an alternative to traditional tobacco use with no evidence to support it among young people. It is important to include ECs in prevention counseling, as they may be a gateway to smoking traditional cigarettes. Sources of Support: The survey was financed by the Programme Intercantonal de Lutte contre la Dépendance au Jeu (PILDJ) and the Canton of Fribourg.
5492	Audrain-McGovern, Janet, Rodriguez, Daniel, Pianin, Stephen, Testa, Shannon (2021) Conjoint Developmental Trajectories of Adolescent E-cigarette and Combustible Cigarette Use. Pediatrics; 148<br><br>**Keywords:**<br>Abridged Index Medicus Index Medicus Schools Sex Factors Epidemiologic Methods Humans Philadelphia

	Surveys and Questionnaires Adolescent Male Female Cigarette Smoking -- psychology Cigarette Smoking -- epidemiology Electronic Nicotine Delivery Systems -- statistics & numerical data<br><br>**Abstract:** OBJECTIVESIn this study, we sought to identify which adolescents progress to regular electronic cigarette (e-cigarette) use (without cigarette smoking), which adolescents become dual users of both types of cigarettes, and how dual use develops across time.METHODSAdolescents (N = 1808) from public high schools outside Philadelphia, Pennsylvania, completed in-classroom surveys at wave 1 (fall 2016, beginning of ninth grade) and at 6-month intervals for the following 36 months (fall 2019, beginning of 12th grade).RESULTSA sequential processes growth mixture model identified 4 conjoint latent classes: later, rapid e-cigarette uptake (class 1: n = 230); no use of e-cigarettes or combustible cigarettes (class 2: n = 1141); earlier, steady e-cigarette uptake (class 3: n = 265); and dual use of e-cigarettes and combustible cigarettes (class 4: n = 204). Using a rich set of potential risk factors, multinomial logistic regression assessed the likelihood of belonging to each conjoint class compared with the comparison class (dual use). Adolescents in the dual use class were characterized by a greater number and severity of e-cigarette and combustible cigarette risk factors. Adolescents in the 2 e-cigarettes-only classes were characterized by either e-cigarette-specific risk factors (earlier onset) or no risk factors (later onset). The no use class had an absence of risk factors for e-cigarette and cigarette use.CONCLUSIONSThis study provides new prospective evidence for distinct patterns and profiles of adolescents who progress to current e-cigarette use, including adolescents who were initially cigarette smokers. The findings have implications for prevention intervention timing, tobacco product focus, content, and the adolescent subgroups to target.
5493	Audrain-McGovern, Janet, Rodriguez, Daniel, Testa, Shannon, Alexander, Emily, Pianin, Stephen (2021) Adolescent e-cigarette onset and escalation: Associations with internalizing and externalizing symptoms. Journal of Adolescent Health; 68<br><br>**Keywords:**<br>Adolescents E-cigarettes Depression Anxiety Substance use Empirical Study Quantitative Study Human Male Female Adolescence (13-17 yrs) Externalizing Symptoms Electronic Cigarettes High Schools Risk Factors Onset (Disorders) Major Depression Drug Usage US article 3233:Substance Abuse & Addiction<br><br>**Abstract:** Purpose: We sought to evaluate if internalizing symptoms (i.e., anxiety and depression) and/or externalizing symptoms (i.e., impulsivity, sensation seeking, and substance use) were risk factors for the onset of 30-day e-cigarette use and escalation in the number of days used across the following 30 months among adolescents. Methods: Adolescents (N = 1,808) from public high schools outside Philadelphia, PA, completed in-classroom surveys at wave 1 (fall 2016, beginning of ninth grade) and at 6-month intervals for the following 30 months (spring 2019, end of 11th grade). Results: A two-part latent growth curve model of e-cigarette use revealed significant positive associations between externalizing factors, past 30-day e-cigarette use, and the number of days of e-cigarette use only at wave 1 (p values <.05). Cigarette smoking was associated with a slowing in the rate of onset of 30-day e-cigarette use across the 30-month follow-up ($\beta = -.24$, $z = -2.41$, $p = .02$). Depression was associated with an increased rate of escalation in the number of days of e-cigarette use across the following 30 months ($\beta = .01$, $z = 2.52$, $p = .01$), whereas anxiety was associated with a decreased rate ($\beta = -.72$, $z = -2.36$, $p = .02$). Conclusions: The findings highlight two groups of adolescents at risk for e-cigarette use: adolescents whose e-cigarette use reflects a higher-risk profile with early e-cigarette onset, and adolescents who have a lower-risk profile, at least initially, with later e-cigarette use onset. The timing and content of prevention efforts should be tailored to each group. (PsycInfo Database Record (c) 2021 APA, all rights reserved) (Source: journal abstract)
5519	Ball, Jude, Fleming, Theresa, Drayton, Bradley, Sutcliffe, Kylie, Lewycka, Sonia, Clark, Terryann C. (2021) New Zealand Youth19 survey: vaping has wider appeal than smoking in secondary school students, and most use nicotine-containing e-cigarettes. Australian and New Zealand journal of public health;<br><br>**Keywords:**

	adolescent e-cigarettes smoking tobacco vaping MEDLINE controlled study nicotine high school prevalence male health survey human New Zealander article non-smoker female drug therapy major clinical study juvenile New Zealand public health pod<br><br>**Abstract:** OBJECTIVE: To investigate smoking and vaping in secondary school students (aged 13-18 years) in New Zealand (NZ) following the introduction of 'pod' e-cigarettes, which have been associated with the rapid escalation of youth vaping elsewhere. METHODS: Data on smoking and vaping were collected in 2019 as part of a comprehensive youth health survey (N=7,721). RESULTS: Vaping was 2-3 times more prevalent than smoking, with 10% of students vaping regularly (monthly or more often), and 6% weekly or more often, compared with 4% and 2%, respectively, for tobacco smoking. Nicotine-containing e-cigarettes were sometimes or always used by 80% of regular and 90% of weekly vapers. Regular and weekly smoking was rare in low deprivation (affluent) areas, whereas regular and weekly vaping prevalence was similar across the socioeconomic spectrum. More than 80% of ever-vapers (N=2732) reported they were non-smokers when they first vaped, and 49% of regular vapers (N=718) had never smoked. CONCLUSIONS: A significant proportion of New Zealand adolescents, many of whom have never smoked, use nicotine-containing e-cigarettes regularly. Implications for public health: Vaping is less harmful than smoking, but it is not harmless. Public health action is needed to support young non-smokers to remain smokefree and vape-free.
5523	Bandi, Priti, Cahn, Zachary, Goding Sauer, Ann, Douglas, Clifford E., Drope, Jeffrey, Jemal, Ahmedin, Fedewa, Stacey A. (2021) Trends in E-Cigarette Use by Age Group and Combustible Cigarette Smoking Histories, U.S. Adults, 2014-2018. American journal of preventive medicine; 60<br><br>**Keywords:**<br>Index Medicus Young Adult Cross-Sectional Studies Humans Adult Middle Aged Adolescent Prevalence Cigarette Smoking -- epidemiology Vaping Electronic Nicotine Delivery Systems Smoking Cessation<br><br>**Abstract:** INTRODUCTIONThe trends in e-cigarette prevalence and population count of users according to cigarette smoking histories are unknown. These data are needed to inform public health actions against a rapidly changing U.S. e-cigarette market.METHODSData collected between 2014 and 2018 (analyzed in 2020) from cross-sectional, nationally representative National Health Interview Surveys were used to estimate current e-cigarette prevalence, adjusted prevalence differences (percentage points), and population counts of users. Analyses were stratified by age group (younger, 18-29 years, n=25,445; middle age, 30-49 years, n=47,745; older, ≥50 years, n=79,517) and cigarette smoking histories (current smokers, recent quitters [quit <1 year ago], near-term quitters [quit 1-8 years ago], long-term quitters [quit >8 years ago], never smokers).RESULTSAmong younger adults, e-cigarette use increased in all groups of smokers, with notable increases between 2014 and 2018 among never smokers (1.3%-3.3%, adjusted prevalence difference=2%, p<0.001) and near-term quitters (9.1%-19.2%, adjusted prevalence difference=8.8%, p=0.024). Conversely, the only substantial increase in e-cigarette use between 2014 and 2018 among middle-aged (5.8%-14.4%, adjusted prevalence difference=8.2%, p<0.001) and older (6.3%-9.5%, adjusted prevalence difference=3.3%, p=0.045) adults was among near-term quitters. The largest absolute population increase in e-cigarette users between 2014 and 2018 was among younger-adult never smokers (0.49-1.35 million), followed by near-term quitters among middle-aged (0.36-0.95 million), younger (0.23-0.57 million), and older (0.35-0.50 million) adults.CONCLUSIONSThe continuous increase among younger-adult never smokers suggests a rise in primary nicotine initiation with e-cigarettes. The concomitant increase among near-term quitters of all age groups suggests continuing e-cigarette use among smokers who may have switched from cigarettes previously.
5544	Beck, Dana C., Boyd, Carol J., Evans-Polce, Rebecca, McCabe, Sean Esteban, Veliz, Phil T. (2021) An examination of how e-cigarette/cigarette use during adolescence is associated with future use during the third trimester of pregnancy. Substance abuse;<br><br>**Keywords:**

	adolescent cigarettes E-cigarettes pregnancy MEDLINE controlled study nicotine cigarette smoking smoking cessation adult adolescence human human tissue third trimester pregnancy young adult article female major clinical study electronic cigarette<br><br>**Abstract:** Background: Adolescent e-cigarette use has increased, as has e-cigarette use during pregnancy, yet little is known about how these types of tobacco/nicotine (cigarettes/e-cigarettes) use during adolescence are associated with tobacco/nicotine use during the third trimester of pregnancy among young adults. Methods: National longitudinal data (2013-2018) from the Population Assessment of Tobacco and Health (PATH) study were used. Young adults ages 18-20 who indicated past-year pregnancy made up the analytic sample (N = 246). Logistic regression was used to evaluate the association between history of past 30-day use of cigarettes/e-cigarettes during adolescence (i.e., 14-17) and later use during the third trimester of pregnancy among young women (i.e., 18-20). Results: Within the sample of young women who indicated a pregnancy during the past year, 18.9% indicated smoking cigarettes and 4.2% indicated using e-cigarettes during their last trimester. Cigarette smoking in adolescence (wave 1 or 2) was associated with cigarette use during the last trimester (aOR = 4.76, 95% CI = 1.36, 16.6); however, e-cigarette use during adolescence was not associated with either cigarette or e-cigarette use during the third trimester of pregnancy. Conclusions: Tobacco/nicotine prevention in early adolescence has implications for preventing tobacco/nicotine-related harms during pregnancy among young adults. Intervention programs and clinicians informed about various types of tobacco/nicotine are needed to address tobacco/nicotine cessation among adolescents to prevent consequences of tobacco/nicotine use during pregnancy.
5558	Bentivegna, Kathryn, Atuegwu, Nkiruka C., Oncken, Cheryl, DiFranza, Joseph R., Mortensen, Eric M. (2021) Electronic cigarettes associated with incident and polysubstance use among youth. Journal of Adolescent Health; 68<br><br>**Keywords:**<br>Electronic cigarettes Electronic nicotine delivery systems Vaping Tobacco Substance use Marijuana use Youth Adolescent Polysubstance use Empirical Study Longitudinal Study Quantitative Study Human Male Female Childhood (birth-12 yrs) School Age (6-12 yrs) Adolescence (13-17 yrs) Polydrug Abuse Sedatives Health Risk Behavior Nicotine Logistic Regression Marijuana Usage Adolescent Development Marijuana Drug Abuse US article 3233:Substance Abuse & Addiction<br><br>**Abstract:** Purpose: Electronic cigarette (e-cigarette) use has increased exponentially among the youth in the United States and may increase the incidence of substance use. Methods: Youth participants (12–17 years) were surveyed through the Population Assessment of Tobacco and Health study over a three-year time period. Youth with any baseline substance use or diagnosis of an attention deficit disorder were excluded from the analysis. Multivariable logistic regressions were used to assess the association between e-cigarette use at Wave 1 and incident substance use (marijuana, painkillers, sedatives, or tranquilizers and Ritalin/Adderall) and polysubstance use at Wave 2 or 3, and marijuana use in the electronic nicotine device at Wave 3. Results: Baseline ever e-cigarette users who had no history of marijuana, nonprescribed drugs and illicit substance use in Wave 1 had increased odds of reporting incident use of marijuana (odds ratio 2.59, 95% confidence interval: 1.90–3.52), nonprescribed Ritalin/Adderall use (1.89, 1.09–3.28), or polysubstance use (2.09, 1.43–3.05) in Wave 2 or 3 compared to never e-cigarette users. They were also more likely to report use of marijuana in the electronic nicotine product (2.26, 1.56–3.27) in Wave 3 compared to never e-cigarette users. There was no statistically significant association between baseline e-cigarette use and incident use of painkillers, sedatives, or tranquilizers in Wave 2 or 3 (1.21, .79–1.87). Conclusions: E-cigarette use is associated with incident use of marijuana, marijuana in electronic nicotine devices, Ritalin/Adderall, and polysubstance use but not painkillers, sedatives, or tranquilizers. Results indicate that e-cigarettes are associated with subsequent additional risky health behaviors in youth. (PsycInfo Database Record (c) 2021 APA, all rights reserved) (Source: journal abstract)

5578	Bhutia, Tshering D., Mehendale, Abhiram, Lad, Narayan, Vaishnav, Priti (2021) E-cigarettes: Gateway to tobacco addiction among adolescents. Tobacco Induced Diseases; 19<br><br>**Keywords:**<br>E-cigarettes ENDS Embase controlled study hookah randomized controlled trial data analysis software conference abstract cross-sectional study human India organization quantitative analysis adolescent female interview smoking tobacco dependence major clinical study government juvenile electronic cigarette<br><br>**Abstract:** Introduction: Electronic nicotine delivery systems (ENDS) are often a controversial product in tobacco control debates. Previous studies suggest that use of ENDS by youth in numerous countries is rampant, however very limited information on use and access is known for the Indian market. Objectives: Salaam Bombay Foundation (SBF) conducted cross-sectional study among Mumbai youth to assess the prevalence, knowledge and perceptions of youth about e-cigarettes. Methods: This study was conducted using both quantitative and qualitative survey methods. Close ended self-administered paper and pencil survey was administered to 306 randomly selected youth followed by personal interviews of around 20 youth using interview schedule. The quantitative data was analyzed using SPSS and qualitative data was coded and analyzed using Microsoft Excel. Results: 'Pen Hookah' was found to be common term for ENDS devices being sold in Mumbai. Out of 306 respondents 73.2% (n=224) admitted to be exposed to it. 33.5% (n=75) of exposed reported its ever use. Ever use (n=75) was higher among 20-25 years (52.7%) compared to 16-19 years (46.7%). Out of ever users, 46.3% (n=63) were males and 13.6% (n=12) were females. Only 20% (n=15) ever users of ENDS were current tobacco users. Participants in this survey reported that youth starts using ENDS out of curiosity and gradually turn towards traditional smoking tobacco products. Continued efforts of SBF and like-minded organizations using this data as evidence resulted in Government of India releasing an ordinance in September 2019 banning sale of e-cigarettes. Conclusion(s): Worldwide, there is strong evidence that shows that e-cigarettes are gaining popularity among youth and acting as gateway to tobacco use among youth. Realizing its potential of being gateway to tobacco use and health harms, 25 countries have banned or restricted its use. Worldwide ban on e-cigarettes is the need of the hour to protect youth from falling prey to tobacco products.
5597	Boccio, Cashen M., Jackson, Dylan B. (2021) Examining potential risk factors for early age of nicotine vaping initiation in a sample of Florida youth. Addictive Behaviors; 120<br><br>**Keywords:**<br>Vaping Nicotine Adolescence Risk factors Empirical Study Quantitative Study Human Male Female Adolescence (13-17 yrs) Adulthood (18 yrs & older) Young Adulthood (18-29 yrs) Electronic Cigarettes Life Span Test Construction Age Differences Drug Abuse US article 3233:Substance Abuse & Addiction<br><br>**Abstract:** Previous research has examined risk factors for nicotine vaping in young adults and older adolescents. Even so, significantly less research has examined risk factors for initiating nicotine vaping in early adolescence. This paper addresses this gap in the literature by examining risk factors for initiating nicotine vaping in a sample of middle- and high-school students. In order to do so, we analyze data from the 2019 cohort of the Florida Youth Substance Abuse Survey (FYSAS). We employ multinomial logistic regression and logistic regression to examine potential risk factors for early age of nicotine vaping initiation. Our findings reveal that early age of nicotine vaping initiation is associated with cigarette use, alcohol use, having substance using peers, lifetime incidence of arrest, and lifetime incidence of school suspension. In conclusion, our findings reveal that early age of nicotine vaping initiation is associated with other forms of risky behavior in adolescence (i.e., substance use, misbehavior leading to arrest and/or school suspension). We discuss the implications of these findings for future research. (PsycInfo Database Record (c) 2021 APA, all rights reserved) (Source: journal abstract)
5609	Bowe, Andrea K., Doyle, Frank, Stanistreet, Debbi, O'Connell, Emer, Durcan, Michéal, Major, Emmet, O'Donovan, Diarmuid, Kavanagh, Paul (2021) E-Cigarette-Only and Dual Use among Adolescents in

	Ireland: Emerging Behaviours with Different Risk Profiles. International journal of environmental research and public health; 18<br><br>**Keywords:**<br>adolescent dual-use electronic nicotine delivery systems risk factors tobacco products Index Medicus Cross-Sectional Studies Electronic Nicotine Delivery Systems Humans Male Ireland -- epidemiology Female Cigarette Smoking -- epidemiology Vaping -- epidemiology<br><br>**Abstract:** E-cigarette-only use and dual-use are emerging behaviours among adolescent nicotine product users which have not yet been sufficiently explored. This study examines the prevalence of, and the factors associated with, nicotine product use in adolescence. The study is a cross-sectional analysis of the 2018 Planet Youth survey completed by 15-16 year olds in the West of Ireland in 2018. The outcome of interest was current nicotine product use, defined as use at least once in the past 30 days. A main effects multinomial logistic regression model was used to examine the association between potential risk and protective factors and nicotine product use. Among 4422 adolescents 22.1% were current nicotine product users, consisting of 5.1% e-cigarette only users, 7.7% conventional cigarette only users, and 9.3% dual-users. For risk factors, the odds of association were weaker for e-cigarette only use compared to conventional cigarette and dual use. Participating in team sport four times/week or more significantly reduced the odds of conventional cigarette and dual use but had no association with e-cigarette only use (Cig: adjusted odds ratio (AOR) 0.63, 95% confidence interval (CI) 0.44-0.90; Dual-use: AOR 0.63, 95% CI 0.43-0.93). Similarly, having higher value for conventional social norms reduced the odds of conventional cigarette and dual use but not e-cigarette only use. This is the first study to show, among a generalisable sample, that dual-use is the most prevalent behaviour among adolescent nicotine product users in Ireland. Risk factor profiles differ across categories of use and prevention initiatives must be cognisant of this.
5613	Boyd, Carol J., Veliz, Philip T. (2020) Latent Classes of Youths' Nicotine Use and Association with Nicotine Dependence. Journal of Addiction Medicine; 14<br><br>**Keywords:**<br>nicotine 54-11-5 Embase social status controlled study demography adulthood secondary prevention vaping male probability sample adult African American adolescence conference abstract human latent class analysis child secondary analysis adolescent female tobacco dependence major clinical study Wisconsin juvenile<br><br>**Abstract:** Introduction: Increasing numbers of adolescents living in the U.S. are vaping nicotine products; their numbers doubling between 2017 to 2019. While the scientific community acknowledges this increase in adolescents' vaping, it is unknown which adolescents who vape nicotine are developing tobacco use disorder symptoms. Using latent class analysis (LCA), we sought to examine the heterogeneity of adolescents' nicotine/tobacco use trajectories, including vaping and other nicotine products and their possible combinations, to determine the association between each trajectory class and risk of nicotine dependence and possible differences among demographic subgroups. Methods: To meet the aforementioned aim, we used three waves of the Population Assessment of Tobacco and Health (PATH) Study data from adolescents (12-17 years old at Wave 1) and conducted a secondary analysis of those adolescents who stated they used a nicotine/tobacco product in the past 30 days. The PATH Study implemented a four-stage, stratified area probability sample design. Three waves of PATH Study data produced a sample of 1,101 youth (12-17 years old) who were nicotine/tobacco users at any wave. Nicotine/tobacco use was determined by asking adolescents about past 30-day use and frequency. A five latent class solution resulted: (1)''Stable/ consistent multiproduct use trajectory'' (n=80), (2)''Increasing cigarette use trajectory'' (n=115), (3)'' Increasing e-cigarette use trajectory'' (n=214), (4)''Experimental (poly-tobacco/nicotine) use trajectory'' (n=373), and (5)''Increasing other nicotine/tobacco use trajectory'' (n=319). Past-year nicotine/tobacco dependence was assessed with six

	items from the Wisconsin Inventory of Smoking Dependence Motives (WISDM-68). Results: All types of past 30-day nicotine/tobacco use increased across the threewaves. Themost prevalentwas the ''Experimental (poly-nicotine/tobacco) use trajectory'' (33.3%). Those represented by the ''Increasing cigarette use trajectory'' reported significantly more past-year nicotine dependence symptoms compared to the ''Increasing e-cigarette use trajectory''. Logistic regression analyses were conducted with sociodemographic characteristics and trajectories. Females had higher odds of being in either the ''Increasing cigarette use trajectory'' or the ''Experimental (poly-nicotine/ tobacco) use trajectory'' compared to males; however, females had lower odds of being in the ''Stable/consistent multiproduct use trajectory'' and ''Increasing other nicotine/tobacco use trajectory''. African Americans had lower odds of being in the ''Increasing e-cigarette use trajectory'' when compared to White respondents, while they had higher odds of being in the ''Increasing other nicotine/tobacco use trajectory''. There were some socioeconomic status and regional differences as well as differences in other substance use. Conclusions: Our study differs from other research because we examined the heterogeneity of adolescents' nicotine/tobacco use to determine latent classes associated with symptoms of nicotine dependence. With LCA, we accounted for all current nicotine/tobacco users in our analyses and thus, we were able to demonstrate that several nicotine/tobacco user trajectories had a greater risk of nicotine dependence compared to only e-cigarette users. We found that adolescent nicotine/tobacco users are a heterogenous group with different risks for nicotine dependence. Since nearly all nicotine/tobacco use begins in adolescence and early adulthood, understanding the different use trajectories and their relationship to nicotine dependence will help clinicians tailor their educational messages and more precisely determine appropriate secondary prevention for at-risk youth.
5629	Braymiller, Jessica (2019) Epidemiology of nicotine use among adolescents and young adults in the United States: Findings from the PATH study. #journal#; AAI28123312<br><br>**Keywords:**<br>epidemiology nicotine use adolescents young adults electronic nicotine delivery systems Empirical Study Quantitative Study Human Male Female Adolescence (13-17 yrs) Adulthood (18 yrs & older) Young Adulthood (18-29 yrs) Empirical Methods Electronic Cigarettes Nicotine Tobacco Smoking Marijuana Usage Marijuana Trends US 2100:General Psychology<br><br>**Abstract:** In 2014, electronic nicotine delivery systems (ENDS, also referred to as vaping or e-cigarette use) surpassed combustible cigarettes as the most commonly used tobacco product among youth. This is a critical public health concern as early onset of ENDS use has been linked to subsequent initiation of cigarettes and other tobacco products. Among adolescents and young adults who have used tobacco, the use of two or more products is also quite common. However, detailed information regarding specific patterns and trends in use within the context of increasingly popular ENDS is lacking. The current dissertation aimed to address this critical gap in three ways. First, chapter one reviews the distinguishing features of a variety of vaping devices, as well as the correlates and consequences associated with their use among adolescents and young adults. Next, chapters two and three empirically examine national patterns and age-trends in the use of ENDS and other nicotine delivery systems. Finally, sociodemographic differences in these patterns and trends, as well as the association between nicotine and marijuana use, are examined.The first empirical study examined nuanced age-trends in recent combustible cigarette, ENDS, and hookah use continuously from ages 14 to 24. Age-varying associations between marijuana use and each tobacco product were also examined. Data were from Wave 3 of the Population Assessment of Tobacco and Health (PATH) Study. The prevalence of past 30-day cigarette, ENDS, hookah, and marijuana use followed distinct age-trends. Cigarette and ENDS use were also significantly related to recent marijuana use across most ages. The association between ENDS and marijuana use was strongest at ages 14 and 15, whereas the association between cigarette and marijuana use was strongest at age 17. Age-varying prevalences, as well as the age-varying association between ENDS and marijuana, differed by sex and race/ethnicity.The second empirical study examined patterns and transitions in lifetime nicotine use among young adults ages 18 to 24 using latent class and

	latent transition analysis (LCA, LTA). Data were from Wave 1 and Wave 2 of the PATH Study. LCA demonstrated that young adults were best represented by five distinct patterns of use: ENDS Only (2%), Hookah Only (10%), Cigarettes Only (19%), Combustibles Only (23%), and Poly, No Smokeless (45%). Differences in latent class membership emerged based on sex and race/ethnicity; the proportion of males and NH white young adults were highest in the ENDS only, Combustibles Only, and Poly, No Smokeless classes. Young adults who had ever used marijuana were also more prevalent within the Combustibles Only and Poly, No Smokeless classes. Young adults in the Combustibles Only class at Wave 1 had a 31% chance of starting to use ENDS by Wave 2. Young adults in the Poly, No Hookah class at Wave 1 had an 11% chance of starting to use hookah by Wave 2.Overall, this dissertation highlights the importance of research examining ENDS, polytobacco, and marijuana use among young people. Innovative statistical techniques provided added insight into who is at risk for using specific nicotine delivery systems at what age, as well as the factors contributing to polytobacco use and co-use with other drugs. Detailed information of this kind is necessary in order to prevent nicotine addiction and the associated health consequences, and to reduce overall burden of nicotine-related illnesses on the United States. (PsycInfo Database Record (c) 2020 APA, all rights reserved)
5642	Brouwer, Andrew F., Jeon, Jihyoun, Hirschtick, Jana L., Jimenez-Mendoza, Evelyn, Mistry, Ritesh, Bondarenko, Irina V., Land, Stephanie R., Holford, Theodore R., Levy, David T., Taylor, Jeremy M. G., Fleischer, Nancy L., Meza, Rafael (2020) Transitions between cigarette, ENDS and dual use in adults in the PATH study (waves 1-4): multistate transition modelling accounting for complex survey design. Tobacco control;<br><br>**Keywords:**<br>disparities electronic nicotine delivery devices non-cigarette tobacco products surveillance and monitoring MEDLINE controlled study demography nicotine cigarette smoking male adult education race human tobacco use article female major clinical study ethnicity electronic cigarette<br><br>**Abstract:** INTRODUCTION: Even prior to 2018, electronic nicotine delivery systems (ENDS) began to dramatically change the landscape of tobacco products and product use patterns in the USA. METHODS: Using a Markov multistate transition model accounting for complex survey design, transition rates between never, non-current, cigarette, ENDS and dual use states were estimated for 23 253 adult participants in waves 1-4 (approximately 2013-2017) of the Population Assessment of Tobacco and Health study. We made short-term transition projections and estimated HRs for age, sex, race/ethnicity, education and income. RESULTS: Cigarette use was persistent among adults, with 89.7% (95% CI 89.1% to 90.3%) of exclusive cigarette users and 86.1% (95% CI 84.4% to 87.9%) of dual users remaining cigarette users (either exclusive or dual) after one wave. In contrast, ENDS use was less persistent, with 72.1% (95% CI 69.6% to 74.6%) of exclusive ENDS users and 50.5% (95% CI 47.8% to 53.3%) of dual users remaining ENDS users (with or without cigarettes) after one wave. Exclusive ENDS users were more likely to start cigarette use after one wave than either never users (HR 25.2; 95% CI 20.9 to 30.5) or non-current users (HR 5.0; 95% CI 4.3 to 5.8). Dual users of ENDS and cigarettes were more likely to stop using cigarettes than exclusive cigarette users (HR 1.9; 95% CI 1.6 to 2.3). Transition rates varied among sociodemographic groups. CONCLUSIONS: Multistate transition models are an effective tool for uncovering and characterising longitudinal patterns and determinants of tobacco use from complex survey data. ENDS use among US adults was less persistent than cigarette use prior to 2018.
5651	Buckner, Julia D., Abarno, Cristina N., Zvolensky, Michael J., Morris, Paige E., Walukevich-Dienst, Katherine, Garey, Lorra, Mayorga, Nubia A. (2021) E-cigarettes use prior to smoking combustible cigarettes among dual users: The roles of social anxiety and E-cigarette outcome expectancies. Addictive Behaviors; 117<br><br>**Keywords:**<br>Cigarettes E-cigarettes Expectancies Smoking Social anxiety Vaping Embase MEDLINE controlled study body weight control article expectation anxiety expectancy adult reinforcement human

	**Abstract:** E-cigarette use is prevalent, and rates of use continue to increase. Although e-cigarettes are often used to help combustible users quit or reduce smoking, some use e-cigarettes in the absence of combustible cigarettes, increasing risk for smoking combustible cigarettes. Yet, little research has examined individual vulnerability factors implicated in transitioning from exclusive e-cigarettes use to dual use of combustible cigarettes. Social anxiety may be one such factor given it is related to a variety of negative smoking-related outcomes. Thus, the current study tested whether social anxiety was related to using e-cigarettes before smoking combustibles among 226 current undergraduate dual users (use both e-cigarettes and combustible cigarettes). Most dual users reported initiating with e-cigarettes (67%). Those who initiated with e-cigarettes reported statistically significantly greater negative reinforcement (d = 0.59) and weight control expectancies (d = 0.37) and greater social anxiety (d = 0.37) than those who initiated with combustibles. Social anxiety was indirectly related to e-cigarette initiation via negative and weight control expectancies. Findings add to a growing literature that dual users initiated with e-cigarettes and extend understanding of this phenomenon by identifying that socially anxious persons may be especially vulnerable to doing so, at least partially due to expectations regarding e-cigarette's ability to manage negative affect and/or weight.
5663	Buu, Anne, Cai, Zhanrui, Li, Runze, Wong, Su-Wei, Lin, Hsien-Chang, Su, Wei-Chung, Jorenby, Douglas E., Piper, Megan E. (2021) Validating E-Cigarette Dependence Scales Based on Dynamic Patterns of Vaping Behaviors. Nicotine & tobacco research : official journal of the Society for Research on Nicotine and Tobacco; 23<br><br>**Keywords:**<br>Index Medicus Humans Adult Tobacco Smoking Retrospective Studies Vaping Electronic Nicotine Delivery Systems Tobacco Products<br><br>**Abstract:** INTRODUCTIONExisting e-cigarette dependence scales are mainly validated based on retrospective overall consumption or perception. Further, given that the majority of adult e-cigarette users also use combustible cigarettes, it is important to determine whether e-cigarette dependence scales capture the product-specific dependence. This study fills in the current knowledge gaps by validating e-cigarette dependence scales using novel indices of dynamic patterns of e-cigarette use behaviors and examining the association between dynamic patterns of smoking and e-cigarette dependence among dual users.METHODSSecondary analysis was conducted on the 2-week ecological momentary assessment data from 116 dual users. The Smoothly Clipped Absolute Deviation penalty (SCAD) was adopted to select important indices for dynamic patterns of consumption or craving and estimate their associations with e-cigarette dependence scales.RESULTSThe fitted linear regression models support the hypothesis that higher e-cigarette dependence is associated with higher levels of e-cigarette consumption and craving as well as lower instability of e-cigarette consumption. Controlling for dynamic patterns of vaping, dual users with lower e-cigarette dependence tend to report higher day-to-day dramatic changes in combustible cigarette consumption but not higher average levels of smoking.CONCLUSIONSWe found that more stable use patterns are related to higher levels of dependence, which has been demonstrated in combustible cigarettes and we have now illustrated in e-cigarettes. Furthermore, the e-cigarette dependence scales may capture the product-specific average consumption but not product-specific instability of consumption.IMPLICATIONSThis study provides empirical support for three e-cigarette dependence measures: PS-ECDI, e-FTCD, and e-WISDM, based on dynamic patterns of e-cigarette consumption and craving revealed by EMA data that have great ecological validity. This is the first study that introduces novel indices of dynamic patterns and demonstrates their potential applications in vaping research.
5664	Buu, Anne, Cai, Zhanrui, Li, Runze, Wong, Su-Wei, Lin, Hsien-Chang, Su, Wei-Chung, Jorenby, Douglas E., Piper, Megan E. (2021) The association between short-term emotion dynamics and cigarette dependence: A comprehensive examination of dynamic measures. Drug and Alcohol Dependence; 218

	**Keywords:**<br>EMA Measurement Negative affect Nicotine dependence Positive affect Embase MEDLINE 40:Drug Dependence, Alcohol Abuse and Alcoholism cigarette smoking follow up ecological momentary assessment male withdrawal syndrome adult drug craving priority journal Article emotion human secondary analysis female tobacco dependence risk factor major clinical study<br><br>**Abstract:** Background: The association between short-term emotion dynamics and long-term psychopathology has been well established in the psychology literature. Yet, dynamic measures for inertia and instability of negative and positive affect have not been studied in terms of their association with cigarette dependence. This study builds an important bridge between the psychology and substance use literatures by introducing these novel measures and conducting a comprehensive examination of such association with intervention implications. Methods: This study conducted secondary analysis on the data from a community sample of 136 dual users (e-cigarette + cigarette) and 101 exclusive smokers who completed both the two-week ecological momentary assessment (EMA) and cigarette dependence assessments in a recent study. Results: Among dual users, a higher average level of negative affect, lower inertia of negative affect (i.e., less sustained negative affect), and higher instability of positive affect (i.e., greater magnitude of changes in positive affect) were associated with higher cigarette dependence. The patterns of associations among exclusive smokers were, however, different. Higher inertia of negative affect, lower instability of positive affect, and higher variability of negative affect were associated with higher dependence. Conclusions: The results illustrate the importance of examining not only negative affect but also positive affect in order to fully understand the association between emotion dynamics and cigarette dependence. The different patterns of association between emotion dynamics and cigarette dependence across the two groups of cigarette users also call for future research that is designed to compare cigarettes and e-cigarettes in terms of their effects on emotion regulation.
5665	Buu, Anne, Hu, Yi-Han, Wong, Su-Wei, Lin, Hsien-Chang (2020) Comparing American college and noncollege young adults on e-cigarette use patterns including polysubstance use and reasons for using e-cigarettes. Journal of American college health : J of ACH; 68<br><br>**Keywords:**<br>alcohol cigarette college e-cigarette marijuana noncollege MEDLINE United States cannabis university educational status vaping prevalence male human young adult student adolescent female smoking cannabis smoking psychology cannabis use attitude to health drug dependence -- epidemiology electronic cigarette<br><br>**Abstract:** Objective: Existing literature on young adults' e-cigarette and polysubstance use focused on college students. This study examined the differences between college and noncollege groups on prevalence and patterns of e-cigarette and other substance use using data from a national survey. Participants: Adults aged 18-24 from the 2013-2014 Population Assessment of Tobacco and Health Study (n = 6,608). Methods: Independent sample t-tests and Chi-square tests were conducted to examine group differences. Results: Noncollege young adults had higher prevalence of cigarette, e-cigarette, and marijuana use; college students had higher prevalence of alcohol use. Among current e-cigarette users, college students had higher prevalence of polysubstance use of alcohol and marijuana. College students used e-cigarettes for socializing purposes more. Conclusions: Differences in prevalence and patterns of e-cigarette and other substance use between college and noncollege groups exist. Future interventions should target the social context of college life and reach out to noncollege young adults in workplaces.
5675	Camenga, Deepa R., Haeny, Angela M., Krishnan-Sarin, Suchitra, O’malley, Stephanie S., Bold, Krysten W. (2021) Pilot ecological momentary assessment study of subjective and contextual factors surrounding e-cigarette and combustible tobacco product use among young adults. International Journal of Environmental Research and Public Health; 18<br><br>**Keywords:**

	E-cigarette Tobacco Young adult Embase controlled study drinking ecological momentary assessment male travel adult human human tissue article female smoking eating smartphone major clinical study craving electronic cigarette<br><br>**Abstract:** Background: Dual use of e-cigarettes and combustible tobacco products is common in young adults. We aimed to explore how ratings of subjective and contextual factors differed between discrete episodes of e-cigarette use vs. combustible tobacco product smoking among a sample of young adults. Methods: Young adults (N = 29, ages 18–30) who used e-cigarettes and ≥1 combustible tobacco product at least once weekly completed a 1-week smartphone-based ecological momentary assessment (EMA). Twice daily random prompts assessed past-15-min use of tobacco products, ratings of subjective factors (e.g., negative affect, craving), and contextual factors related to activity, location, and companionship. A multivariable GEE model assessed whether subjective or contextual factors were associated with e-cigarette vs. combustible tobacco product episodes. Results: 184 tobacco use episodes were reported (39.7% e-cigarette, 60.3% combustible tobacco product). High baseline cigarette dependence, as measured by the Fagerström Test for Cigarette Dependence, was associated with lower odds of e-cigarette vs. combustible tobacco product episodes (aOR 0.01, 95% CI (0.002–0.08); p < 0.001). Neither between-or within-subjects negative affect or craving scores were associated with e-cigarette use. Activities of eating/drinking (aOR 0.20, 95% CI (0.08–0.49); p = 0.001) and being in the companionship of a person who smoked cigarettes (aOR 0.13, 95% CI (0.04–0.43); p = 0.001) were associated with lower odds of e-cigarette vs. combustible tobacco product use episodes. However, traveling (aOR 12.02, 95% CI (3.77–38.26); p ≤ 0.001) and being in a public space (aOR 2.76, 95% CI (1.10–6.96); p = 0.03) were associated with higher odds of e-cigarette than combustible tobacco product use episodes. Conclusions: This pilot data suggests that unique contextual factors may be associated with e-cigarette use, compared to combustible tobacco smoking in a sample of young adults who use both e-cigarettes and combustible tobacco products. Future research with larger samples is needed to better characterize varying contexts and cues for tobacco use among young adults who are dual users.
5687	Cardona, Stephanie, Calixte, Rose, Rivera, Argelis, Islam, Jessica Yasmine, Vidot, Denise Christina, Camacho-Rivera, Marlene (2021) Perceptions and Patterns of Cigarette and E-Cigarette Use among Hispanics: A Heterogeneity Analysis of the 2017-2019 Health Information National Trends Survey. International journal of environmental research and public health; 18<br><br>**Keywords:**<br>Hispanic cigarettes e-cigarettes health information smoking Index Medicus Hispanic Americans Perception Humans Adult Surveys and Questionnaires Puerto Rico -- epidemiology Male Female Vaping Electronic Nicotine Delivery Systems Tobacco Products<br><br>**Abstract:** There are documented disparities in smoking behaviors among Hispanic adults in the U.S., but little is known about patterns of e-cigarette use. Using data from the HINTS 5 cycle 1-3, we examined cigarette and e-cigarette history and current use, as well as perceptions of the dangers of e-cigarette use relative to cigarette use. Primary predictors were Hispanic ethnic group, gender, age, education, income, and English language proficiency. Binary outcomes were modeled using the logit link, and multinomial outcome variables were modeled using generalized logit model. Fifty-three percent of participants were Mexican, 8% Puerto Rican, 4% were Cuban, and 35% identified as other Hispanics. Of the 1618 respondents, 23% were former cigarette smokers and 10% were current cigarette smokers. Twenty percent reported history of electronic cigarettes and 4% reported current use. In multivariable models, Hispanic women were significantly less likely to report ever being smokers compared to Hispanic men (aOR = 0.61, 95% CI = 0.42, 0.88). Puerto Ricans were 2.4 times as likely to report being current smokers (95% CI = 1.11, 5.11) compared to Mexicans. Among Hispanics, significant differences in e-cigarette and cigarette use behaviors emerged by gender, age, ethnicity, and cancer history, with implications for tailoring smoking prevention and cessation messages.

5698	Cavazos-Rehg, Patricia, Li, Xiao, Kasson, Erin, Kaiser, Nina, Borodovsky, Jacob, Grucza, Richard A. (2021) Investigating the role of familial and peer-related factors on electronic nicotine delivery systems (ENDS) use among U.S. adolescents. Journal of adolescence; 87<br><br>**Keywords:**<br>Adolescents ENDS Parent tobacco use Peer tobacco use Substance use Tobacco Index Medicus Humans Tobacco Use Interpersonal Relations Surveys and Questionnaires Adolescent Electronic Nicotine Delivery Systems Tobacco Products<br><br>**Abstract:** INTRODUCTIONTobacco use is a public health concern, and adolescents are at an increased risk of starting and continuing to use tobacco relative to other age groups. Parent involvement and other social environmental factors may influence the development and persistence of tobacco use behaviors in adolescence. This study used the nationally representative Population Assessment of Tobacco and Health (PATH) study data set, and responses from 7025 adolescent participants were analyzed to illustrate the longitudinal relationship between social environment influences and use of adolescent electronic nicotine delivery systems (i.e., ENDS).METHODSSocial environmental factors were assessed at Wave 3 and then compared to electronic nicotine delivery systems (ENDS) status transitions (i.e., initiation, expansion, persistence) at follow-up. Survey-weighted multivariable logistic regression models were used to calculate adjusted odds ratios (AOR) and 95% confidence intervals (CI).RESULTSInitiation and persistence of ENDS use were associated with tobacco availability in the house, perception of having no adverse parent reaction to discovering ENDS use and having a best friend who uses ENDS. Initiation of ENDS use was further associated with having biological relatives who have ever been diagnosed with a substance use disorder (SUD) and persistence of ENDS use was also positively associated with lack of in-house rules. Parents talking about ENDS use with adolescents had no association across all three outcomes.CONCLUSIONSFindings suggest that parental and environmental factors alter the risk of initiation and persistence of ENDS and tobacco use in adolescents, and these should be considered when working with this at-risk population.
5699	Cavazos-Rehg, Patricia, Li, Xiao, Kasson, Erin, Kaiser, Nina, Borodovsky, Jacob T., Grucza, Richard, Chen, Li-Shiun, Bierut, Laura J. (2021) Exploring How Social Media Exposure and Interactions Are Associated with ENDS and Tobacco Use in Adolescents from the PATH Study. Nicotine and Tobacco Research; 23<br><br>**Keywords:**<br>Embase MEDLINE 40:Drug Dependence, Alcohol Abuse and Alcoholism 17:Public Health, Social Medicine and Epidemiology 7:Pediatrics and Pediatric Surgery 32:Psychiatry United States controlled study exposure follow up social media male motivation social interaction Article health survey human multivariate logistic regression analysis tobacco use adolescent female online system prevention health care policy public health electronic cigarette<br><br>**Abstract:** Introduction: Tobacco use is a current public health epidemic that puts individuals at risk for many health conditions and diseases, and adolescents are at high risk for the initiation and persistence of tobacco use behaviors partly due to engagement with social media content. The objective of this study is to examine the association between engaging in social media behaviors and patterns of electronic nicotine delivery systems (ENDS) and tobacco use at a 1-year follow-up among 11 279 adolescents from the PATH study. Methods: Five social media variables were questioned at Wave 2 and then compared to ENDS and tobacco status transitions (i.e., initiation, persistence, and escalation) at a 1-year follow-up, respectively. Survey-weighted multivariable logistic regression models were used to calculate adjusted odds ratios and 95% confidence interval. Results: Passive behaviors on social media were related to higher likelihoods of starting to use ENDS and other tobacco products. Additionally, active behaviors on social media were related to higher likelihoods for the initiation and persistence of tobacco use. In particular, sending tobacco content to other users was further associated with a higher likelihood of escalation of tobacco product use. Discussion: Both exposure to and interactions with social media

	tobacco content had a significant impact on the patterns of ENDS and tobacco use in adolescents. Due to the amount of time adolescents spend engaging with online content, social media may be a critical place in which to intervene, possibly with the use of antitobacco or tobacco prevention messages. Implications: The results of this study have implications for public health and the policies surrounding adolescents and their exposure to social media. Reducing the ENDS and tobacco content to which adolescents are exposed has the potential to decrease the instances of initiation and persistence of ENDS and tobacco use. Intervening on social media may prevent or slow the progression of ENDS and tobacco use, and increase motivation and actions toward the cessation of tobacco use in adolescents.
5703	Chadi, Nicholas, Belanger, Richard E. (2020) Teen vaping: There is no vapour without fire. Paediatrics and Child Health (Canada); 25<br><br>**Keywords:**<br>Adolescent E-cigarettes Nicotine Policy Smoking Vaping 54-11-5 Embase 17:Public Health, Social Medicine and Epidemiology 7:Pediatrics and Pediatric Surgery social marketing Canadian smoking cessation trend study Review human electronic cigarette vapor tobacco dependence health care policy social aspect electronic cigarette<br><br>**Abstract:** E-cigarettes have become the most important source of nicotine exposure among adolescents. While e-cigarettes may have the potential to help some adults quit smoking, there is a lack of reliable evidence that this would apply to adolescents. On the contrary, e-cigarette use is associated with subsequent use of cigarettes and other tobacco products in teens and is also associated with increased use of alcohol, marijuana, and other drugs. Research on the health effects of e-cigarettes is rapidly emerging suggesting that they carry several acute and long-term risks, particularly for adolescents' still-developing bodies and brains. While several strategies to help youth quit smoking exist, much less is known about effective clinical interventions for adolescents presenting with an addiction to nicotine consumed through e-cigarettes. We discuss the latest research on e-cigarettes with a focus on health effects in youth and propose potential avenues for concerted action among paediatric providers and decision makers.
5726	Chang, Yu-Ping, Seo, Young Sik (2021) E-cigarette use and concurrent risk behaviors among adolescents. Nursing outlook; 69<br><br>**Keywords:**<br>Adolescents Electronic cigarette Risk behaviors Substance use Vaping Abridged Index Medicus Index Medicus Nursing Journals United States Age Factors Vaping -- epidemiology Sex Factors Risk-Taking Humans Surveys and Questionnaires Adolescent Male Female Substance-Related Disorders -- epidemiology Students -- psychology Electronic Nicotine Delivery Systems Adolescent Behavior -- psychology Vaping -- psychology Substance-Related Disorders -- psychology<br><br>**Abstract:** PURPOSEAlthough electronic cigarette (e-cigarette) use is on the rise among adolescents, its relationship with risk behaviors is unclear. This study aimed to examine whether e-cigarette use was related to other risk behaviors and whether age and sex play a role in those associations.METHODSData from the 2017 Youth Risk Behavior Surveillance System was used. Weighted logistic regression analysis was conducted to examine the relationships among variables of interest.FUNDINGSThe results showed that e-cigarette users, compared to nonusers, had higher odds of using other types of substances, as well as being involved in other risk behaviors including driving-related behaviors, sexual activity-related behaviors, and violence-related behaviors. Furthermore, some of these relationships are moderated by age and sex.DISCUSSIONOur findings suggest that e-cigarette use is linked to other risk behaviors among adolescents. Policy makers and educators should address multiple risk behaviors as part of comprehensive tobacco-free policies and youth substance use prevention programs.

5734	Chavez, JeanFelix, Smit, Tanya, Olofsson, Hannah, Mayorga, Nubia A., Garey, Lorra, Zvolensky, Michael J. (2021) Substance Use among Exclusive Electronic Cigarette Users and Dual Combustible Cigarette Users: Extending Work to Adult Users. Substance use & misuse; 56<br><br>**Keywords:**<br>Dual combustible cigarette user alcohol use cannabis nicotine dependence nonprescription opioids Hallucinogens Index Medicus Smokers Humans Adult Male Female Electronic Nicotine Delivery Systems Substance-Related Disorders Tobacco Products<br><br>**Abstract:** BACKGROUNDElectronic cigarettes (e-cigarettes) have become increasingly popular in recent years. Existing evidence indicates e-cigarettes used in isolation are less harmful than combustible cigarettes, yet emerging work has demonstrated that adults use e-cigarettes largely in combination with combustible cigarettes (i.e. dual use). Despite this data, little is understood about how exclusive and dual e-cigarette users may differ across behavioral outcomes, such as substance use dependence and behaviors among adults.OBJECTIVESThus, the current project examined differences in e-cigarette dependence, problematic alcohol use, cannabis use, and nonmedical prescription opioid use (e.g. methadone and oxycodone) across both exclusive and dual e-cigarette adult users.RESULTSParticipants included 531 (53.6% female; Mage = 35.29 years, SD = 10.44) adults with past-month e-cigarette use. Dual users reported greater e-cigarette dependence, alcohol use, current cannabis use, and endorsement of nonprescription opioid use. Conclusions/Importance: Importantly, this work suggests that adults who use both combustible and e-cigarettes may constitute a group more vulnerable to experiencing problematic substance use and more severe e-cigarette dependence than exclusive e-cigarette users.
5757	Cho, Beomyoung, Hirschtick, Jana L., Usidame, Bukola, Meza, Rafael, Mistry, Ritesh, Land, Stephanie R., Levy, David T., Holford, Theodore, Fleischer, Nancy L. (2021) Sociodemographic patterns of exclusive, dual, and polytobacco use among U.S. high school students: A comparison of three nationally representative surveys. Journal of Adolescent Health; 68<br><br>**Keywords:**<br>Polytobacco use Sociodemographic differences High school students ENDS Empirical Study Longitudinal Study Quantitative Study Human Male Female Adolescence (13-17 yrs) Adulthood (18 yrs & older) Young Adulthood (18-29 yrs) Electronic Cigarettes Sociocultural Factors Adolescent Health Risk Taking Tobacco Smoking Demographic Characteristics Smokeless Tobacco Parent Educational Background US article 2990:Drug & Alcohol Usage (Legal)<br><br>**Abstract:** Purpose: This study examines sociodemographic patterns of exclusive/dual/polytobacco use among U.S. high school students using multiple national surveys. Methods: Using three national youth surveys (Population Assessment of Tobacco and Health [PATH] Wave 4 [2016–2017], 2017 Youth Risk Behavior Survey, and 2017 National Youth Tobacco Survey), we classified tobacco products into four groups: (1) electronic nicotine delivery systems (ENDS), (2) conventional cigarettes (CCs), (3) other combustible tobacco products, and (4) smokeless tobacco products. We created 16 categories of non/exclusive/dual/polytobacco use within the past 30 days using the four product groups and calculated weighted population prevalence by sex and race/ethnicity (all surveys) and parental education and income (PATH), based on variable availability. Results: The results from 9,331, 12,407, and 9,699 high school students in PATH, Youth Risk Behavior Survey, and National Youth Tobacco Survey, respectively, largely agreed and pointed to similar conclusions. ENDS was the most prevalent exclusive use product (3.8%–5.2% across surveys), with CCs falling to second or third (1.2%–2.0% across surveys). By sex, exclusive, dual, and poly smokeless tobacco product use were more common for males, whereas exclusive CC use was more common for females. By race/ethnicity, non-Hispanic Whites had a higher prevalence of exclusive ENDS use and ENDS/CC dual use than non-Hispanic Blacks. As income and parental education levels increased from low to high, the prevalence of exclusive CC use decreased, whereas the prevalence of exclusive ENDS use increased. Conclusion: Understanding sociodemographic

	patterns of tobacco use can help identify groups who may be at greater risk for tobacco-related health outcomes. (PsycInfo Database Record (c) 2021 APA, all rights reserved) (Source: journal abstract)
5759	Cho, J., Ketema, E., Barrington-Trimis, J. L., Leventhal, A. M. (2021) Racial/ethnic and gender differences in joint substance use initiation patterns among high school students. Alcoholism: Clinical and Experimental Research; 45<br><br>**Keywords:**<br>alcohol 64-17-5 cannabis 8001-45-4 8063-14-7 opiate 53663-61-9 8002-76-4 8008-60-4 Embase high school student controlled study GRADE approach male human adolescence conference abstract California child female adolescent cohort analysis alcohol consumption tobacco sex difference probability major clinical study Hispanic ethnicity electronic cigarette<br><br>**Abstract:** Purpose: Identifying variations in the timing of alcohol in relation to other substance use initiation can inform prevention efforts, including possible tailoring for subpopulations. The current study was designed to identify prototypical developmental patterns of initiation of alcohol, combustible cigarettes, e-cigarettes, cannabis, and opioids during adolescence and investigate differences by ethnicity and gender in these patterns. Data: Students (46.9% male; Baseline median age = 14.1; 42.7% Hispanic) recruited from ten high schools in the Los Angeles metropolitan area from 2013-2017 who had never used any of these substances at baseline 9th grade assessment (N = 2081). Methods: Using a longitudinal cohort with eight semiannual assessments during high school, Multiple Event Process Survival Mixture modeling identified latent initiation classes with distinct patterns in timing of initiation across the five substances. Classes were then compared with respect to ethnicity and gender. Results: Four distinct classes were identified. (1) High-Risk Alcohol, Cannabis, Opioid, and Poly-Tobacco Initiators (12.3%) displayed a large initial risk of initiating all substance use, which increased over time, with a cumulative probability of initiation approaching over 80% for all substances other than opioids by the end of high school. (2) Early Alcohol, Cannabis, and Poly-Tobacco Initiators (8.1%) displayed the highest initiation risk in 9th grade, decreasing thereafter. (3) Late Alcohol, Cannabis, and E-cigarette Initiators (22.1%) showed low risk through 10th grade, with an escalation in risk of alcohol, cannabis, and e-cigarette use initiation later in high school. (4) Abstainers (57.5%) remained at low risk across substances throughout high school. Hispanic students were overrepresented in the Early Alcohol, Cannabis, and Poly-Tobacco Initiators class (60.8%) compared to the other three (39.0%?46.4%). A higher proportion of girls were classified as Late Alcohol, Cannabis, and E-cigarette Initiators (58.0%) compared to High-Risk Alcohol, Cannabis, Opioid, and Poly-Tobacco Initiators (49.4%). Conclusions: Early alcohol use initiation was associated with two distinct patterns of cross substance use initiation: consistently high risk across substances, including opioids (1), and early initial but decreasing risk thereafter for cannabis, e-cigarettes, and combustibles (2). Findings suggest that early alcohol use among Hispanic youth is likely to reflect the early initial pattern and that girls are more likely to follow a cross-substance late onset initiation pattern.
5786	Cole, Adam G., Aleyan, Sarah, Battista, Kate, Leatherdale, Scott T. (2021) Trends in youth e-cigarette and cigarette use between 2013 and 2019: insights from repeat cross-sectional data from the COMPASS study. Canadian journal of public health = Revue canadienne de sante publique; 112<br><br>**Keywords:**<br>Adolescent Cigarette E-cigarette Smoking Trends Youth MEDLINE cigarette smoking Canada female vaping male epidemiology human cross-sectional study<br><br>**Abstract:** OBJECTIVES: E-cigarettes are an increasingly popular product among youth in Canada. However, there is a lack of long-term data presenting trends in use. As such, the objective of this study was to examine trends in e-cigarette and cigarette use across various demographic characteristics between 2013 and 2019 among a large sample of secondary school youth in Canada. METHODS: Using repeat cross-sectional data from a non-probability sample of students in grades 9 to 12, this study explored trends in the prevalence of ever and current e-cigarette use and cigarette smoking between

	2013-2014 and 2018-2019 in British Columbia, Alberta, Ontario, and Quebec. Trends in ever and current e-cigarette use and cigarette smoking were studied across demographic variables among students in Ontario. RESULTS: The prevalence of e-cigarette ever and current use was variable across province and increased over time, particularly between 2016-2017 and 2018-2019. In contrast, the prevalence of current cigarette smoking was relatively stable over the study period, decreasing significantly in Alberta and Ontario between 2017-2018 and 2018-2019. In Ontario, the prevalence of ever and current e-cigarette use increased among all grades, both genders, and all ethnicities. CONCLUSION: Consistent with data from the United States, the prevalence of e-cigarette use among our large sample of Canadian youth has increased substantially in a short period of time. Surveillance systems should continue to monitor the prevalence of tobacco use among youth. Additional interventions may be necessary to curb e-cigarette use among Canadian youth.
5788	Coleman, Michael, Donaldson, Candice D., Crano, William D., Pike, James R., Stacy, Alan W. (2021) Associations Between Family and Peer E-Cigarette Use With Adolescent Tobacco and Marijuana Usage: A Longitudinal Path Analytic Approach. Nicotine & tobacco research : official journal of the Society for Research on Nicotine and Tobacco; 23<br><br>**Keywords:**<br>Nicotine 6M3C89ZY6R Index Medicus Marijuana Use Schools Humans Tobacco Use Tobacco Substance-Related Disorders Cannabis Longitudinal Studies Adolescent Tobacco Products Male Female Cigarette Smoking Vaping Electronic Nicotine Delivery Systems Adolescent Behavior Peer Group Parents Marijuana Smoking<br><br>**Abstract:** INTRODUCTIONResearch indicates a link between adolescent e-cigarette use and combustible tobacco cigarette (CTC) initiation, and recent studies suggest their connection with marijuana uptake. Our 3-year longitudinal cohort study investigated the implications of adolescent, peer, and family e-cigarette use with adolescents' expectations and willingness to initiate CTC use, and subsequent CTC and marijuana use.AIMS AND METHODSRelationships were examined in a secondary analysis of a 3-year longitudinal cohort subsample involving adolescents enrolled in alternative California high schools (N = 1025). Analyses examined responses over three yearly observations. Family, peer, and respondents' e-cigarette use, respondents' positive cigarette expectancies and willingness to use CTCs were assessed in the study's first year (T1). CTC use in the survey's second year (T2) and marijuana use in the third year (T3) were assessed via path analysis.RESULTSRespondents reporting at least one family member or peer using e-cigarettes were more likely to use e-cigarettes at T1 than those whose peers/family members did not. They reported more positive expectancies about CTCs and greater willingness to initiate use. These variables predicted CTC use at T2, which directly anticipated marijuana use in the survey's third year (T3), as did adolescents' use of e-cigarettes at T1. All model relations were statistically significant.CONCLUSIONSAnalysis demonstrated the strong association of family members' and peers' behaviors with adolescent e-cigarette use, and the temporal precedence of e-cigarette use with subsequent CTC and marijuana uptake. The predictive implications of e-cigarettes for other dangerous substance use should be examined in future prevention campaigns.IMPLICATIONSThe presented study expands upon existing literature connecting adolescent e-cigarette use and later CTC and marijuana use. The findings indicate the significant implications of exposure to e-cigarette use by parents and peers and demonstrate in a longitudinal 4-year panel survey the direct and indirect predictive implications of e-cigarette use for CTC and marijuana uptake. The research illustrates the utility of programs and campaigns that target peer and family groups to maximize impacts on adolescent willingness to try CTCs, positive expectancies, and possible onset of CTC and marijuana use.
5794	Conner, Mark, Grogan, Sarah, Simms-Ellis, Ruth, Cowap, Lisa, Armitage, Christopher J., West, Robert, Marshall, Anna-Marie, Siddiqi, Kamran (2021) Association between age at first reported e-cigarette use and subsequent regular e-cigarette, ever cigarette and regular cigarette use. Addiction (Abingdon, England); 116

	**Keywords:**<br>Adolescents E-cigarettes electronic nicotine delivery systems harm reduction intervention smoking Index Medicus Prospective Studies Humans Adolescent Smoking -- epidemiology Vaping Tobacco Products<br><br>**Abstract:** BACKGROUND AND AIMSAssociation of electronic cigarette use and subsequent smoking has received considerable attention, although age of first use has not. This study tested differences in regular (e-cigarettes, cigarettes) and ever (cigarettes) use between e-cigarette user groups: early versus never users, late versus never users, early versus late users and effects of controlling for covariates.DESIGNProspective study with 12- and 24-month follow-up of e-cigarette/cigarette ever/regular use with data from an intervention.SETTINGForty-five schools in England (Staffordshire and Yorkshire).PARTICIPANTSNever smokers (3289 13-14-year-olds) who were part of a cluster randomized controlled trial.MEASUREMENTSThe sample was divided into groups of e-cigarette users: early users (at 13-14 years), late users (at 14-15 years) and never users (at 13-14 and 14-15 years). Dependent variables were self-reported regular e-cigarette and cigarette use and ever cigarette use at 15-16 years. Covariates were assessed.FINDINGSEarly and late users compared with never users were significantly more likely to be regular e-cigarette users [early: odds ratio (OR) = 9.42, 95% confidence interval (CI) = 5.38, 16.49, P < 0.001; late: OR = 6.89, 95% CI = 4.11, 11.54, P < 0.001], ever cigarette users (early: OR = 7.96, 95% CI = 6.02, 10.53, P < 0.001; late: OR = 5.13, 95% CI = 3.85, 6.84, P < 0.001) and regular cigarette users (early: OR = 7.80, 95% CI = 3.99, 15.27, P < 0.001; late: OR = 4.34, 95% CI = 1.93, 9.77, P < 0.001) at age 15-16 years. Late users compared with early users had significantly lower rates of ever use of cigarettes at 15-16 years (OR = 0.48, 95% CI = 0.35, 0.66, P < 0.001), although this difference was non-significant at 12 months after first use of e-cigarettes (OR = 0.89, 95% CI = 0.64, 1.25, P = 0.498). Controlling for covariates did not change the findings.CONCLUSIONSAdolescents in England who report using e-cigarettes at age 13-14 years have higher rates of subsequently initiating cigarette use than adolescents who report using e-cigarettes at age 14-15 years, a difference that may be attributable to a longer period of time to initiate cigarette use in former group.
5795	Conner, Tamlin S., Zeng, Jiaxu, Blank, Mei-Ling, He, Vicky, Hoek, Janet (2021) A Descriptive Analysis of Transitions from Smoking to Electronic Nicotine Delivery System (ENDS) Use: A Daily Diary Investigation. International journal of environmental research and public health; 18<br><br>**Keywords:**<br>daily diary dual use e-cigarettes electronic nicotine delivery systems Index Medicus Smoking Humans Tobacco Smoking Adolescent New Zealand -- epidemiology Vaping Smoking Cessation<br><br>**Abstract:** OBJECTIVESWe aimed to examine patterns in smoking and electronic nicotine delivery system (ENDS) use over an extended period of time (up to 20 weeks) in people who smoked and who had never previously made a successful quit attempt using an ENDS.DESIGN AND SETTINGWe conducted a longitudinal mixed-methods study in Dunedin, New Zealand, during 2018 and 2019.PARTICIPANTSPurposively selected participants (N = 45; age (≥18 years), gender, ethnicities, cigarettes/day) who wished to quit smoking.INTERVENTIONSParticipants were provided with a second-generation ENDS device (vape pen or starter "tank" device) at the start of their quit attempt, and asked to complete smartphone-based daily diary surveys assessing smoking and ENDS use.OUTCOME MEASURESSunburst plots and a sequence plot were used to describe weekly and daily patterns of smoking and ENDS use (smoking only, ENDS use only, dual use, abstinent).RESULTSThe most frequently reported movements among participants, classified according to their study week behaviour, occurred between dual use and exclusive ENDS use (and vice versa). A smaller group reported moving from dual use to exclusive smoking (and often back to dual use), and a small number reported moving between abstinence and different ENDS and smoked tobacco usage behaviours. Data visualisations focussing on those participants who had provided data during each of weeks 9-12 indicate that only a minority reported sustained dual use; instead, most participants indicated varied smoked tobacco and ENDS use, which included periods of dual use.CONCLUSIONSThe considerable variety observed within and between

	study participants suggests that high variability is typical rather than exceptional. Transitions from smoking to ENDS use may involve considerable periods of dual use, which is likely to be dynamic and potentially sustained over several months.
5800	Coreas, Saida I., Rodriquez, Erik J., Rahman, Sana G., El-Toukhy, Sherine, Compton, Wilson M., Blanco, Carlos, Kimmel, Heather L., Pérez-Stable, Eliseo J. (2021) Smoking susceptibility and tobacco media engagement among youth never smokers. Pediatrics; 147<br><br>**Keywords:**<br>Embase MEDLINE controlled study cigarette smoking tobacco industry marketing current smoker social media male never smoker human child article adolescent female smoking major clinical study juvenile Hispanic friend electronic cigarette<br><br>**Abstract:** BACKGROUND: Susceptibility to future smoking among youth never smokers has not changed in the past 20 years, although experimental cigarette smoking has decreased. We assessed how smoking susceptibility and tobacco industry-related marketing influenced smoking initiation. METHODS: Four waves (2013-2018) of the Population Assessment of Tobacco and Health Study data were analyzed among youth aged 12 to 17 years at wave 1 who completed wave 4. Susceptibility was assessed by 4 items (openness to curiosity, try soon, try in the next year, and if your best friend offered) and categorized into 4 levels (0 = definitely no to all; 1 = yes to 1; 2 = yes to 2; and 3 1 4 = yes to 3 or 4 susceptibility items). Multivariable logistic regression evaluated how susceptibility levels, electronic cigarette use, and tobacco-related media activity predicted future experimental (puff), current (past 30 days), or established (0 cigarettes) smoking. RESULTS: Among 8899 never smokers at wave 1, 16.4% became experimental smokers, 7.6% current smokers, and 1.8% established smokers at wave 4. Black and Latino/a youth were less likely to experiment. Youth who endorsed 3 or 4 susceptibility items at wave 1 were more likely to be experimental (adjusted odds ratio [aOR] = 6.0; confidence interval [CI] = 4.8-7.4), current (aOR = 4.2; CI = 3.2-5.4), or established (aOR = 4.4; CI = 2.4-7.9) smokers at wave 4. Exposure to tobacco marketing, using tobacco-related apps, seeing social media content posted about tobacco, and ever use of electronic cigarettes also predicted experimental smoking. CONCLUSIONS: Smoking susceptibility and exposure to tobacco industry-related marketing were predictive of cigarette smoking. Clinicians should consider screening adolescents for smoking susceptibility and tobacco-related media exposure.
5810	Creamer, MeLisa R., Dutra, Lauren M., Sharapova, Saida R., Gentzke, Andrea S., Delucchi, Kevin L., Smith, Ruben A., Glantz, Stanton A. (2021) Effects of e-cigarette use on cigarette smoking among U.S. youth, 2004–2018. Preventive Medicine; 142<br><br>**Keywords:**<br>Adolescent Cigarette smoking E-cigarettes Tobacco use Embase MEDLINE 17:Public Health, Social Medicine and Epidemiology United States controlled study demography high school student Black person time series analysis prevalence male prediction simulation priority journal Article cross-sectional study human sample size female risk factor Hispanic electronic cigarette<br><br>**Abstract:** Objective: To determine if the declining trend in U.S. youth cigarette smoking changed after e-cigarettes were introduced, and if youth e-cigarette users would have been likely to smoke cigarettes based on psychosocial and demographic predictors of smoking. Methods: An interrupted time series analysis was used for cross-sectional data from the 2004 to 2018 National Youth Tobacco Surveys (NYTS) to assess changes in cigarette and e-cigarette use over time. A multivariable logistic regression model used 2004–2009 NYTS data on psychosocial risk factors to predict individual-level cigarette smoking risk from 2011 to 2018. Model-predicted and actual cigarette smoking behavior were compared. Results: The decline in current cigarette smoking slowed in 2014 (−0.75 [95% CI: −0.81, −0.68] to −0.26 [95% CI: −0.40, −0.12] percentage points per year). The decline in ever cigarette smoking accelerated after 2012 (−1.45 [95% CI: −1.59, −1.31] to −1.71 [95% CI: −1.75, −1.66]). Ever and current combined cigarette and/or e-

	cigarette use declined during 2011–2013 and increased during 2013–2014 with no significant change during 2014–2018 for either variable. The psychosocial model estimated that 69.0% of current cigarette smokers and 9.3% of current e-cigarette users (who did not smoke cigarettes) would smoke cigarettes in 2018. Conclusions: The introduction of e-cigarettes was followed by a slowing decline in current cigarette smoking, a stall in combined cigarette and e-cigarette use, and an accelerated decline in ever cigarette smoking. Traditional psychosocial risk factors for cigarette smoking suggest that e-cigarette users do not fit the traditional risk profile of cigarette smokers.
5829	Dai, Hongying (2021) Youth Observation of E-Cigarette Use in or Around School, 2019. American Journal of Preventive Medicine; 60<br><br>**Keywords:**<br>Embase MEDLINE 17:Public Health, Social Medicine and Epidemiology 7:Pediatrics and Pediatric Surgery United States exposure controlled study cigarette high risk population race difference vaping school child correlation analysis child Caucasian student adolescent self report bath gender risk factor juvenile health education high school student observation household marketing prevalence male never smoker Article human multivariate logistic regression analysis data analysis school female middle school student policy locker room electronic cigarette<br><br>**Abstract:** Introduction: E-cigarette use increased dramatically among U.S. students during 2017–2019, and school plays an important role in preventing and reducing youth substance use. This study examines the prevalence and correlates of self-reported youth observation of e-cigarette use on school grounds. Methods: Data from the 2019 National Youth Tobacco Survey (N=19,018) were analyzed to examine the prevalence and factors associated with youth observation of e-cigarette use in or around the school. Multivariable logistic regressions were performed to assess the associations between youth observation of e-cigarette use and the susceptibility to initiate cigarettes or e-cigarettes. Analyses were conducted in 2020. Results: In 2019, about 63.9% of students (16.8 million) reported noticing youth use of e-cigarettes in or around the school, with bathroom or locker room as the most common location (33.2%). Female (versus male) students; high-school (versus middle-school) students; non-Hispanic Whites (versus other groups); former and past 30–day e-cigarette users (versus never users); students with exposure to tobacco marketing (versus none); and students living with a household member using e-cigarettes (versus not) had higher odds of reporting the observation of vaping in schools. Among never tobacco users (n=11,518), observation of vaping in schools was associated with higher odds of being susceptible to smoking cigarettes (AOR=1.2, 95% CI=1.0, 1.3) and using e-cigarettes (AOR=1.7, 95% CI=1.6, 1.9), especially among middle-school students. Conclusions: E-cigarette use is common on school grounds, and youth observation of vaping in schools may increase the risk of initiating tobacco use in the future. School vaping policy and education programs are needed to curb youth e-cigarette use.
5861	Dennison, Elizabeth, Kashiwabara, Mina, Moadsiri, Ada (2021) Vaping among Western Pacific youth: A threat to public health. Tobacco Induced Diseases; 19<br><br>**Keywords:**<br>ENDS Vaping Western Pacific Youth Embase controlled study Samoa prevalence Cook Islands awareness conference abstract human Kiribati Cambodia conflict of interest Papua New Guinea adolescent Vanuatu Guam Fiji Macao gauge Niue Marshall Islands juvenile public health<br><br>**Abstract:** Introduction: The use of electronic nicotine delivery systems (ENDS), or "electronic cigarettes" has skyrocketed among youth. There is an emerging scientific consensus on the harmful effects relating to ENDS use and evidence that ENDS use is a risk factor for future cigarette experimentation. In the Western Pacific Region, only 6 countries have banned ENDS; while, 8 countries have regulated ENDS (as of October 2019). Objectives: To gauge the awareness of ENDS existence, determine the prevalence of ENDS use, and to explore the relationship between tobacco and ENDS use among Western Pacific youth. Methods: Global Youth Tobacco Survey (GYTS), a nationally representative survey of students aged 13-

	15, was administered between 2015-2019 in 12 countries/areas in the Western Pacific Region using a globally standardized protocol (Cambodia, Cook Islands, Fiji, Guam, Kiribati, Lao PDR, Macao, Marshall Islands, Niue, Papua New Guinea, Samoa, and Vanuatu). Results: Guam had the highest percentage of students who had heard of ENDS (81.2%), followed by Niue (74.5%), and Macao (68.7%); Cambodia had the lowest (7.3%). Niue had the highest percentage of students who used ENDS (37.5%), followed by Guam (34.6%), and Marshall Islands (16.7%); Cambodia had the lowest (2.3%). Tobacco use/smoking was associated with increased ENDS use, compared to non-tobacco use/smoking in the majority of the countries/areas. Conclusion(s): Students' use of ENDS varies widely throughout the Western Pacific Region. Interventions targeting Regional Action Plan for Tobacco Control in the Western Pacific (2020-2030). A limitation was the respondents' ability to understand the definition of ENDS. Only 12 out of 37 countries/areas reported data; therefore, results are not representative of the entire region. Further investigation is needed. The authors have no known conflicts of interest.
5875	Doggett, Amanda, Godin, Katelyn M., Schell, Olena, Wong, Suzy L., Jiang, Ying, Leatherdale, Scott T. (2021) Assessing the impact of sports and recreation facility density within school neighbourhoods on Canadian adolescents' substance use behaviours: Quasi-experimental evidence from the COMPASS study, 2015-2018. BMJ Open; 11<br><br>**Keywords:**<br>epidemiology public health substance misuse Embase MEDLINE 17:Public Health, Social Medicine and Epidemiology 7:Pediatrics and Pediatric Surgery demography cigarette smoking vaping built environment social interaction urbanization binge drinking recreational park adolescent cannabis use risk factor physical activity high school student protection high school Canadian substance use male prevalence neighborhood health behavior Article human female sport facility major clinical study<br><br>**Abstract:** Objectives There has been relatively little exploration to date of potential protective effects within school neighbourhoods, such as those conferred by facilities that seek to promote health with respect to substance use and related harms. This study examined how the density of sports and recreation facilities in the school neighbourhood is associated with the likelihood of binge drinking, e-cigarette use, cigarette smoking and cannabis use among Canadian secondary school students. Design Longitudinal data from the COMPASS study on Canadian youth health behaviours from 2015/2016 to 2017/2018 was linked with school neighbourhood data capturing the number of sports and recreation facilities within a 1500 m radius of schools. Setting Secondary schools and school neighbourhoods in Alberta, British Columbia, Ontario and Quebec who participated in the COMPASS study. Participants 16 471 youth who participated in the COMPASS study over three school years (2015/2016-2017/2018). Primary and secondary outcome measures Binge drinking, e-cigarette use, cigarette use, cannabis use. Results Logistic regression models using generalised estimating equations identified that greater density of sports and recreation facilities within the school neighbourhood was significantly associated with lower likelihood of binge drinking and e-cigarette use but was not associated with cigarette smoking or cannabis use. Conclusions This research can help to support evidence-informed school community-based efforts to prevent substance-related harms among youth.
5878	Donaldson, Elisabeth A., Robinson, Joelle N., Nguyen Zarndt, Anh (2020) Association between free tobacco product sample receipt and tobacco use in youth and adults in the PATH Study, 2014-2016. Preventive medicine; 131<br><br>**Keywords:**<br>Marketing Samples Tobacco Youth Index Medicus Cross-Sectional Studies Humans Adult Vulnerable Populations -- psychology Aged Middle Aged Tobacco Industry Adolescent United States -- epidemiology Male Female Prevalence Marketing -- trends Smoking -- trends Tobacco Use -- epidemiology Tobacco Products -- supply & distribution Gift Giving Electronic Nicotine Delivery Systems -- statistics & numerical data Tobacco Use -- trends Electronic Nicotine Delivery Systems -- economics Tobacco Products -- economics Smoking -- epidemiology

	**Abstract:** Tobacco companies have distributed free samples of tobacco products in the past. While prior studies have found a relationship between various marketing strategies and tobacco use, no study has assessed the prevalence of free sample receipt or the relationship between receipt and subsequent tobacco use. We analyze three waves of Population Assessment of Tobacco and Health (PATH) Study data to provide the first nationally representative prevalence estimates of free tobacco product receipt among US youth and adults in 2014 to 2016. This analysis also examines the relationship between free tobacco product receipt and ever, past 30-day, and new tobacco use one year later. The prevalence of free sample receipt in the US population in 2014-2016 was 0.8% for any tobacco product but 1.6% for ENDS/e-liquid in adults, and 0.5% for any tobacco product but 1.05% for ENDS/e-liquid in youth. Free sample receipt was higher among vulnerable subgroups. Receipt of free tobacco product samples was associated with tobacco use status using cross-sectional data in youth and adults, as well as one year later in youth. Receipt of a free ENDS or e-liquid sample was associated with any tobacco and ENDS use status using cross-sectional data in youth and adults. After these data were collected, the US Food and Drug Administration broadened the ban on distributing free samples to include all tobacco products except smokeless tobacco in adult-only facilities. Our findings support limiting free samples to further prevent youth access to tobacco products.
5896	Duan, Zongshuan, Wang, Yu, Huang, Jidong (2021) Sex Difference in the Association between Electronic Cigarette Use and Subsequent Cigarette Smoking among U.S. Adolescents: Findings from the PATH Study Waves 1-4. International journal of environmental research and public health; 18<br><br>**Keywords:**<br>adolescents cigarettes electronic cigarettes sex difference youth tobacco prevention Index Medicus Prospective Studies Sex Characteristics Humans Tobacco Aged Adolescent Male Female Cigarette Smoking Vaping Electronic Nicotine Delivery Systems Tobacco Products<br><br>**Abstract:** E-cigarettes are the most-used tobacco products among U.S. adolescents. Emerging evidence suggests that adolescents using e-cigarettes are at elevated risk for initiating cigarette smoking. However, whether this risk may differ by sex remains unknown. This study analyzed data from Wave 1 to 4 of the Population Assessment of Tobacco and Health (PATH) Study, a nationally representative longitudinal survey. Generalized estimation equations (GEE) were performed to estimate the associations between baseline e-cigarette use and subsequent cigarette smoking, controlling for sociodemographic characteristics, mental health conditions, and other tobacco use. Effect modifications by sex were examined. Multivariate analyses showed that, among baseline never cigarette smokers, past-30-day e-cigarette use at baseline waves was significantly associated with past-30-day cigarette smoking at follow-up waves (aOR = 3.90, 95% CI: 2.51-6.08). This association was significantly stronger for boys (aOR = 6.17, 95% CI: 2.43-15.68) than for girls (aOR = 1.10, 95% CI: 0.14-8.33). Additionally, using other tobacco products, older age, and having severe externalizing mental health problems at baseline were significantly associated with an increased likelihood of cigarette smoking at follow-up. The prospective association between e-cigarette use and cigarette smoking differs by sex among U.S. adolescents. Sex-specific tobacco control interventions may be warranted to curb the youth tobacco use epidemic.
5909	East, Katherine A., Reid, Jessica L., Rynard, Vicki L., Hammond, David (2021) Trends and patterns of tobacco and nicotine product use among youth in Canada, England, and the United States from 2017 to 2019. Journal of Adolescent Health; 69<br><br>**Keywords:**<br>Adolescent Electronic nicotine delivery systems Nicotine Prevalence Smoking Surveys and questionnaires Tobacco products Empirical Study Quantitative Study Human Male Female Adolescence (13-17 yrs) Adulthood (18 yrs & older) Young Adulthood (18-29 yrs) Electronic Cigarettes Vaping Public Health Tobacco Smoking Trends US England article Canada 3360:Health Psychology & Medicine

	**Abstract:** Purpose: The tobacco and nicotine market is diversifying. Implications for public health will depend on trends in use, including overall use, and who is using these products. This study examined differences over time (2017–2019), across countries (Canada, England, the United States (US)), and by smoking and vaping, in use of other tobacco/nicotine products and overall use. Methods: The study includes online repeat cross-sectional surveys of youth aged 16–19 years in Canada (N = 11,714), England (N = 11,170), US (N = 11,838) in 2017, 2018, and 2019. Past 30-day use of tobacco/nicotine products (any, cigarette, e-cigarette, other combusted [cigars/cigarillos/waterpipe], other non-combusted [smokeless tobacco/nicotine replacement therapies) were examined by country, year, and, for other combusted and non-combusted products, past 30-day cigarette smoking and vaping. Use of emerging products (IQOS, nicotine pouches) was explored in 2018–2019. Results: From 2017 to 2019, use of any product increased in Canada (17.1%–23.2%, AOR = 1.07 [95% CI = 1.04–1.09]) and the US (18.0%–24.0%, AOR = 1.06 [1.04–1.09]) but not England (20.8%–21.7%, AOR = 1.01 [.99–1.03]). Use of other combusted products (cigars/cigarillos/waterpipe) showed little change (Canada: 8.1%–7.8%; England: 6.3%–7.3%; US: 8.6%–8.5%; p ≥ .151). Use of other non—combusted products (smokeless/nicotine replacement therapies) increased in all countries (Canada: 1.5%–3.2%, AOR = 1.02 [1.01–1.02]; England: 1.4%–2.6%, AOR = 1.02 [1.01–1.03]; US: 3.3%–4.9%, AOR = 1.02 [1.01–1.02]). Vaping increased in all countries (Canada: 8.4%–17.8%; England: 8.7%–12.6%; United States: 11.1%–18.5%; all p < .001). Smokers and vapers reported greater use of other combusted and non-combusted products than those who neither smoked/vaped (p < .001). Emerging product use was rare (≤1.5%). Conclusions: Youth past 30-day tobacco and nicotine product use increased from 2017 to 2019 in Canada and the United States, largely due to increases in vaping and other non-combusted products. “Other” tobacco/nicotine products were used predominantly by youth who smoked cigarettes and/or vaped. (PsycInfo Database Record (c) 2021 APA, all rights reserved) (Source: journal abstract)
5934	Epstein, Marina, Bailey, Jennifer A., Kosterman, Rick, Rhew, Isaac C., Furlong, Madeline, Oesterle, Sabrina, McCabe, Sean Esteban (2021) E-cigarette use is associated with subsequent cigarette use among young adult non-smokers, over and above a range of antecedent risk factors: A propensity score analysis. Addiction; 116<br><br>**Keywords:**<br>Addiction combustible cigarettes electronic cigarettes propensity score analysis risk factors for smoking young adulthood Empirical Study Quantitative Study Human Male Female Childhood (birth-12 yrs) School Age (6-12 yrs) Adolescence (13-17 yrs) Measurement Childhood Development Risk Factors Emerging Adulthood Tobacco Smoking Confidence Limits (Statistics) Adolescent Development US article 2990:Drug & Alcohol Usage (Legal)<br><br>**Abstract:** Background and Aims: There is a public health concern that the use of e-cigarettes among non-smoking young adults could be associated with transition to combustible cigarette use. The current study is a quasi-experimental test of the relationship between e-cigarette use and subsequent combustible cigarette use among young adult non-smokers, accounting for a wide range of common risk factors. Design: Logistic regression was used to predict combustible cigarette use on three or more occasions at age 23 years based on age 21 e-cigarette use. Inverse probability weighting (IPW) was used to account for confounding variables. Setting: Data were drawn from the Community Youth Development Study (CYDS), a cohort study of youth recruited in 2003 in 24 rural communities in seven US. states Participants: Youth in the CYDS study (n = 4407) were surveyed annually from ages 11 to 16, and at ages 18, 19, 21 and 23 years (in 2016). The sample was gender balanced (50% female) and ethnically diverse (20% Hispanic, 64% white, 3% black and 12% other race or ethnicity). The current study was limited to participants who had never used combustible cigarettes by age 21 (n = 1825). Measurements: Age 21 use of e-cigarettes and age 23 use of combustible cigarettes (three or more occasions) were included in the regression analysis. Age 11–19 measures of 22 common predictors of both e-cigarette and combustible cigarette use (e.g. pro-cigarette attitudes, peer smoking, family monitoring) were used to create IPWs. Findings: After applying IPW, e-cigarette use at age 21 was associated with a twofold increase in odds of combustible

	cigarette use on three or more occasions 2 years later (odds ratio = 2.16, confidence interval 1.23, 3.79). Conclusions: Among previously never-smoking US young adults, e-cigarette use appears to be strongly associated with subsequent combustible cigarette smoking, over and above measured preexisting risk factors. (PsycInfo Database Record (c) 2021 APA, all rights reserved) (Source: journal abstract)
5997	Fox, Andrew T., Jarmolowicz, David P., Reed, Derek D., Naude, Gideon P., Martin, Laura E. (2020) Combustible cigarette use and other risky behavior by adult e-cigarette users in a 2019 survey. Drug and Alcohol Dependence; 216<br><br>**Keywords:**<br>Smoking E-cigarettes Vaping Risky behavior Empirical Study Quantitative Study Human Male Female Adulthood (18 yrs & older) Electronic Cigarettes Nicotine Risk Factors Tobacco Smoking article 2990:Drug & Alcohol Usage (Legal)<br><br>**Abstract:** This study examine the relationship between risky behavior and vaping in 4000+ adults using an online survey. The data indicated that vaping compared to not vaping is associated with engagement in risky behaviors and that over 25 % of vapers are never-smokers, who risk transitioning to combustible cigarettes. Support for vaping as a gateway to smoking traditional cigarettes is mixed. Some vapers are being exposed to nicotine prior to traditional tobacco initiation—they remain relatively few in number and while their pattern of use is dabbling, this does not preclude vaping as a gateway. Additionally, other risk factors, which are present regardless of tobacco-naïve status, limit the extent to which vaping can be said to cause later cigarette use; vapers who do not smoke cigarettes are clearly already in a high-risk category. (PsycInfo Database Record (c) 2020 APA, all rights reserved)
6019	Gagné, Thierry, Lee, Joseph G. L., O'Loughlin, Jennifer (2021) Cigarette smoking and E-cigarette use among young adults in the United States: Findings from the 2016–18 behavioral risk factor surveillance system. Addictive Behaviors; 113<br><br>**Keywords:**<br>Cigarette Smoking E-cigarettes United States Young Adults Embase MEDLINE 17:Public Health, Social Medicine and Epidemiology controlled study age distribution ex-smoker smoking habit current smoker vaping smoking cessation prevalence male never smoker adult Article human behavioral risk factor surveillance system female<br><br>**Abstract:** Because there are few proven smoking cessation approaches for young adults, it is critical to consider the potential of e-cigarettes as an option. Evidence from 2012 to 2013 in the United States (U.S.) suggested that current e-cigarette use was associated with a higher probability of daily smoking and a lower probability of quitting among young adults. This study examines the associations between e-cigarette use frequency and cigarette smoking in more recent data. Data on e-cigarette use were available for 32,656 current smokers and 9,565 past-year quitters ages 18–34 in the 2016–2018 U.S. Behavioral Risk Factor Surveillance System. Participants were categorized as “daily”, “non-daily”, “former”, or “never” e-cigarette users. Adjusted prevalence ratios were estimated to test the associations between e-cigarette use and each of daily smoking and past-year cessation. With e-cigarette never-users as the reference: 1) daily e-cigarette users were 30% (95%CI 0.61–0.79) less likely to be daily smokers; non-daily and former e-cigarette users were 8% (95%CI 1.02–1.15) and 16% (95%CI 1.11–1.21) more likely to be daily smokers, respectively; 2) daily e-cigarette users were 54% (95%CI 1.38–1.73) more likely to have quit in the past year; non-daily and former e-cigarette users were 50% (95%CI 0.42–0.59) and 21% (95%CI 0.73–0.86) less likely to have quit in the past year, respectively. The findings suggest that non-daily e-cigarette use was associated with greater frequency of smoking and less quitting whereas daily e-cigarette use was associated with a lower frequency of smoking and more quitting among young adults in 2016–18.

6032	Gallus, Silvano, Borroni, Elisa, Odone, Anna, van den Brandt, Piet A., Gorini, Giuseppe, Spizzichino, Lorenzo, Pacifici, Roberta, Lugo, Alessandra (2021) The Role of Novel (Tobacco) Products on Tobacco Control in Italy. International journal of environmental research and public health; 18<br><br>**Keywords:**<br>Italy e-cigarette harm reduction heat-not-burn tobacco products heated tobacco products smoking prevalence Index Medicus Cross-Sectional Studies Humans Tobacco Aged Italy -- epidemiology Electronic Nicotine Delivery Systems Tobacco Products<br><br>**Abstract:** In Italy, electronic cigarettes have spread since 2010 and heated tobacco products (HTP) since 2016. We investigated their public health consequences on conventional cigarette smoking, taking advantage of a series of cross-sectional studies annually conducted between 2001 and 2019 in Italy. Every year, the sample, including around 3000 individuals, was representative of the general Italian population aged ≥15 years. In Italy, smoking prevalence steadily declined from 29.1% in 2001 to 20.6% in 2013, then increased to 22.0% in 2019. In 2017-2019, current electronic cigarette users were 2.1% and in 2019 current HTP users were 1.1%. Among 498 ever electronic cigarette users, 23.2% started or re-started smoking and 15.7% quit smoking after electronic cigarette use; of 49 ever HTP users, 19.1% started or re-started smoking combusted cigarettes and 14.6% quit smoking after HTP use. The availability of novel products in Italy resulted in a halt of the decreasing trend in smoking prevalence. For the first time, we observed an increase of Italians inhaling nicotine, concurrently with the spread of novel (tobacco) products. More importantly, the use of novel products appears to increase-rather than decrease-the likelihood of smoking conventional cigarettes. Considering this evidence, we see no argument to justify the huge fiscal and regulatory benefits these products continue to have, at least in Italy.
6035	Gambaryan, Marine, Kalinina, Anna, Popovich, Marina, Startovoytov, Michail, Drapkina, Oxana (2020) Exposure to E-cigarettes advertisement and product use in Russian population: results from Russian Tobacco Control Policy evaluation survey. European Respiratory Journal; 56<br><br>**Keywords:**<br>Embase controlled study demography ex-smoker prevalence male advertising sport never smoker adult conference abstract human social network female shopping Russian (citizen) major clinical study Internet electronic cigarette<br><br>**Abstract:** Prevention: of e-cigarettes (EC) use from early age is essential for respiratory and general health. Exposure to marketing of these products may be crucial for early EC use. We aimed to investigate prevalence of EC use related to exposure to EC advertisement and promotion (A&P) in Russian population. Methods: Cross-sectional data of adult representative sample (n=11625) from Russian Tobacco Control Policy Evaluation Survey are analysed, based on multistage sampling in 10 Russian regions in 2017-2018. Ever use of EC, heated tobacco products(HTP) and Current EC use are analysed in relation with exposure to A&P of EC. Multiple logistic regression is employed; odds ratios(OR) adjusted by socio-demographic factors and smoking status. Results: Ever EC use was prevalent in 9.3%, HTP use-in 3.3%; and current EC use-in 2.5% of population. Higher likelihood of EC/HTP ever and current use was strongly associated with (1)age: in age group 18-24 OR 7.4, 2.2, 4.4; (2)smoking status-highest in smokers: OR 7.2, 7.3, 5.4 vs.former smokers OR 4.9, 5.8, 3.4 and never smokers(p<.001). Exposure to EC A&P was highest in the youngest age group. Chances to be EC ever/current user were significantly higher in case of exposure to EC A&P (1)on Internet:OR 2.2 and 2.8; (2)social networks:OR 1.6 and 1.9; (3)TV: OR 1.8 and 3.7 respectively, (4)in shops: OR 1.8. Chances to be HTP ever users were significantly higher when exposed to EC A&P (1)on TV:OR 3.7, (2) in shops: OR 4.7, (3) at sport events: OR 3.0, (4) in bars and pubs: OR 2.2 and (5) at temporary shopping facilities:OR 2.1. Conclusion: Use of EC is strongly related to A&P of these products. Legal ban on EC A&P is required.

6069	Glasser, Allison M., Johnson, Amanda L., Niaura, Raymond S., Abrams, David B., Pearson, Jennifer L. (2021) Youth Vaping and Tobacco Use in Context in the United States: Results from the 2018 National Youth Tobacco Survey. Nicotine and Tobacco Research; 23<br><br>**Keywords:**<br>Embase MEDLINE United States high school vaping prevalence adult human experiment human child tobacco use article adolescent vapor major clinical study juvenile public health<br><br>**Abstract:** Introduction: According to the National Youth Tobacco Survey (NYTS), youth e-cigarette use (vaping) rose between 2017 and 2018. Frequency of vaping and concurrent past 30-day (p30d) use of e-cigarettes and tobacco products have not been reported. Methods: We analyzed the 2018 NYTS (N = 20 189) for vaping among all students (middle and high school; 6-12th grades; 9-19 years old) by frequency of vaping, exclusive vaping, p30d poly-product use (vaping and use of one or more tobacco product), and any past tobacco product use. Results: In 2018, 81.4% of students had not used any tobacco or vapor product in the p30d, and 86.2% had not vaped in the p30d. Among all students, of the 13.8% vaped in the p30d, just over half vaped on ≤5 days (7.0%), and roughly a quarter each vaped on 6-19 days (3.2%) and on 20+ days (3.6%). Almost three quarters of p30d vapers (9.9%) reported past or concurrent tobacco use and the remainder (3.9%) were tobacco naïve. 2.8% of students were tobacco naïve and vaped on ≤5 days; 0.7% were tobacco-naïve and vaped on 6-19 days, and 0.4% were tobacco-naïve and vaped on 20+ days. Conclusions: Vaping increased among US youth in 2018 over 2017. The increases are characterized by patterns of low p30d vaping frequency and high poly-product use, and a low prevalence of vaping among more frequent but tobacco naïve vapers. Implications: Results underscore the importance of including the full context of use patterns. The majority of vapers (60.0%-88.9% by use frequency) were concurrent p30d or ever tobacco users. About 4% of students were tobacco naïve and vaped in the p30d, but few (0.4%) vaped regularly on 20 or more days. Reporting youth vaping data with frequency and tobacco product co-use will give public health decision-makers the best possible information to protect public health.
6084	Gomez, Yessica, Creamer, MeLisa, Trivers, Katrina F., Anic, Gabriella, Morse, Aura Lee, Reissig, Chad, Agaku, Israel (2020) Patterns of tobacco use and nicotine dependence among youth, United States, 2017–2018. Preventive Medicine; 141<br><br>**Keywords:**<br>Dependence Multiple product use Nicotine Poly-tobacco use Tobacco Youth 54-11-5 Embase MEDLINE 40:Drug Dependence, Alcohol Abuse and Alcoholism 17:Public Health, Social Medicine and Epidemiology 32:Psychiatry United States cigarette hookah smokeless tobacco American male race priority journal Article human child cigar tobacco use school female tobacco dependence major clinical study juvenile ethnicity sex factor electronic cigarette<br><br>**Abstract:** This study examined patterns of tobacco product use and their association with nicotine dependence among U.S. youth. Combined data from the 2017–2018 National Youth Tobacco Surveys were analyzed for students that reported current (past-30-day) use of e-cigarettes, cigarettes, cigars, smokeless tobacco, or hookah (n = 6106). Analyses assessed multiple product use (≥2 tobacco products) overall and by sex, school level, race/ethnicity, current use, and frequent use (use of a product for ≥20 of the preceding 30 days). Multivariable logistic regression was used to identify correlates of nicotine dependence. During 2017–2018, 40.8% of tobacco product users reported use of multiple products. Multiple product use ranged from 47.0% among e-cigarette users to 80.7% among cigarette smokers. Among frequent users of each respective product, 80.0% of cigarette smokers, 74.9% of cigar smokers, 73.6% of smokeless tobacco users, 70.7% of hookah smokers, and 40.3% of e-cigarette users reported use of multiple products. Most youth who reported nicotine dependence (64.0%) were multiple product users. E-cigarettes were the most common single product used (36.3%) and the product most commonly used in combination with other products; e-cigarettes plus cigarettes (6.7%) was the most common

	product combination. Combustible product use, smokeless tobacco use, multiple product use and frequent use were associated with greater odds of nicotine dependence. Nicotine dependence among youth is especially influenced by cigarette use, smokeless tobacco use, frequent use of any tobacco product, and multiple product use. Proven tobacco control interventions in coordination with regulatory efforts can reduce youth tobacco product use.
6108	Gravely, Shannon, Meng, Gang, Cummings, K. Michael, Hyland, Andrew, Borland, Ron, Hammond, David, O'connor, Richard J., Goniewicz, Maciej L., Kasza, Karin A., McNeill, Ann, Thompson, Mary E., Hitchman, Sara C., Levy, David T., Thrasher, James F., Quah, Anne C. K., Martin, Nadia, Ouimet, Janine, Boudreau, Christian, Fong, Geoffrey T. (2020) Changes in smoking and vaping over 18 months among smokers and recent ex-smokers: Longitudinal findings from the 2016 and 2018 ITC four country smoking and vaping surveys. International Journal of Environmental Research and Public Health; 17<br><br>**Keywords:**<br>Adults E-cigarettes Nicotine vaping products Smoking Tobacco Transitions Embase MEDLINE 17:Public Health, Social Medicine and Epidemiology United States controlled study nicotine vaping male England middle aged adult Article health survey human prospective study young adult Canada longitudinal study female behavior change cohort analysis Australia<br><br>**Abstract:** This descriptive study of smokers (smoked at least monthly) and recent ex-smokers (quit for ≤2 years) examined transitions over an 18 month period in their smoking and vaping behaviors. Data are from Waves 1 (W1: 2016) and 2 (W2: 2018) of the ITC Four Country Smoking and Vaping Survey, a cohort study of adult (≥18+) smokers, concurrent users (smoke and vape), and recent ex-smokers from Australia, Canada, England, and the United States (US). Respondents (N = 5016) were classified according to their smoking and vaping status, which resulted in eight subgroups: (1) exclusive daily smokers (2) exclusive non-daily smokers; (3–6) concurrent users (subdivided into four groups by each combination of daily/non-daily smoking and daily/non-daily vaping); (7) ex-smokers who vape; (8) ex-smokers not vaping. The analyses focused first on describing changes between groups from W1 to W2. Second, transition outcomes were assessed based on changes in smoking and vaping between W1 and W2. Transitions focused on smoking were: no change in smoking (continued smoking at the same frequency); decreased smoking; increased smoking; discontinued smoking; relapsed (ex-smokers at W1 who were smoking at W2). Transitions focused on vaping were: initiated vaping; switched from smoking to vaping. Overall, this study found that the vast majority of smokers were smoking 18 months later. Non-daily smokers were more likely than daily smokers to have discontinued smoking ($p < 0.0001$) and to have switched to exclusive vaping ($p = 0.034$). Exclusive non-daily smokers were more likely than exclusive daily smokers to have initiated vaping ($p = 0.04$). Among all W1 daily smokers, there were no differences in discontinued smoking between daily smokers who vaped (concurrent users) and exclusive daily smokers; however, concurrent users were more likely than exclusive daily smokers to have decreased to non-daily smoking ($p < 0.001$) or to have switched to vaping by W2 ($p < 0.001$). Among all W1 non-daily smokers, there were no significant differences in increased smoking or discontinued smoking between concurrent users or exclusive smokers. Most ex-smokers remained abstinent from smoking, and there was no difference in relapse back to smoking between those who vaped and those who did not.
6113	Grobman, Ben, Wu, Ran, Jackson, Asti, Bold, Krysten W., Morean, Meghan E., Camenga, Deepa R., Davis, Danielle R., Simon, Patricia, Krishnan-Sarin, Suchitra, Kong, Grace (2021) First tobacco product tried among adolescents based on race/ethnicity and socioeconomic status. Addictive Behaviors; 113<br><br>**Keywords:**<br>Socioeconomic status Race Ethnicity E-cigarettes Blunts Tobacco Empirical Study Quantitative Study Human Male Female Adolescence (13-17 yrs) Whites Electronic Cigarettes Prevention Reference Groups Logistic Regression Risk Factors Tobacco Smoking Surveys Students US article 3233:Substance Abuse & Addiction

	**Abstract:** In recent years, the tobacco use landscape among youth has changed greatly, underscoring a need to understand current tobacco product initiation patterns. This study aimed to examine if adolescents' first tobacco product tried differs by race/ethnicity and socioeconomic status (SES). Cross-sectional survey data were collected from 6 Connecticut high schools in Spring 2019 (N = 4875). The analytic sample comprised ever users of tobacco products who reported on the first tobacco product they tried (N = 2530; 53.6% female). Of ever tobacco product users, 64.6% reported that their first tobacco product tried was an e-cigarette, 24.4% reported blunts, 6.5% reported another tobacco product, and 4.5% reported cigarettes. A multinomial logistic regression model examined race/ethnicity and SES as predictors of the first tobacco product tried with cigarettes as the reference group. Compared to Non-Hispanic (NH) White students, NH Black students (OR = 0.38, 95%CI = 0.17–0.82), Hispanic students (OR = 0.44, 95%CI = 0.27–0.71), and students of other NH races (OR = 0.30, 95%CI = 0.18–0.52) were less likely to report initiating tobacco product use with e-cigarettes. Additionally, compared to NH White students, NH Black students were more likely to report using blunts as their first tobacco product (OR = 3.01, 95%CI = 1.38–6.56). Compared to low SES, middle SES (OR = 1.62, 95%CI = 1.04–2.52) was associated with greater odds of reporting e-cigarettes as the first tobacco product. These findings suggest that youth of different backgrounds initiate tobacco use with different tobacco products and tailored prevention strategies are needed. (PsycInfo Database Record (c) 2021 APA, all rights reserved) (Source: journal abstract)
6117	Gueorguieva, Ralitza, Buta, Eugenia, Simon, Patricia, Krishnan-Sarin, Suchitra, O'Malley, Stephanie S. (2021) Data visualization tools of tobacco product use patterns, transitions and sex differences in the path youth data. Nicotine and Tobacco Research; 22<br><br>**Keywords:**<br>Embase MEDLINE 17:Public Health, Social Medicine and Epidemiology 7:Pediatrics and Pediatric Surgery controlled study cigarette smoking data visualization water pipe smoking vaping male confidence interval calibration population research human Article calculation child questionnaire tobacco use longitudinal study adolescent female cohort analysis sex difference adolescent smoking<br><br>**Abstract:** Introduction: Evaluations of multiple tobacco product use and temporal changes in patterns of use are complicated by a large number of combinations and transitions. Visualization tools could easily identify most common patterns and transitions. Methods: Set intersection bar plots describe ever use of five tobacco products among 12-17 years old youth in wave 1 of the Population Assessment of Tobacco and Health (PATH) study (N = 11 497). Heat maps visualize unweighted frequencies of transitions from ever use at wave 1 (2013-2014) to past 12-month use at wave 2 (2014-2015). Weighted calibrated heat maps assess differences in relative frequencies of transitions by pattern at wave 1 and identify differences in transitions by sex. Results: The most common tobacco product ever use patterns in wave 1 were of cigarettes only, e-cigarettes only or hookah only, followed by ever use of both cigarettes and e-cigarettes. Initiation of use between waves was uncommon. The most frequent transition among those who reported use at wave 2 but not at wave 1 (N = 971) was to e-cigarette use (N = 301). However, among e-cigarette-only ever users at wave 1 (N = 260), about half did not report any product use at wave 2. Use of three or more products remained stable. Adolescent girls compared to boys appeared more likely to report hookah use at both waves. Conclusion: Set intersection bar plots and heat maps are useful for visualizing tobacco product use patterns and transitions, especially for multiple products. Both techniques could identify common problematic tobacco use patterns across and within populations.
6139	Hair, Elizabeth C., Barton, Alexis A., Perks, Siobhan N., Kreslake, Jennifer, Xiao, Haijun, Pitzer, Lindsay, Leventhal, Adam M., Vallone, Donna M. (2021) Association between e-cigarette use and future combustible cigarette use: Evidence from a prospective cohort of youth and young adults, 2017–2019. Addictive Behaviors; 112<br><br>**Keywords:**<br>Electronic cigarette Pod Mod Transition to Combustible Cigarette Youth and young adults Embase

	MEDLINE 17:Public Health, Social Medicine and Epidemiology controlled study nicotine age distribution cigarette smoking smoking cessation male adult sensation seeking Article human prospective study tobacco use young adult female cohort analysis social psychology sex difference<br><br>**Abstract:** Introduction: A surge in popularity of e-cigarettes prompts concern given the association between e-cigarettes and future cigarette use. However, much of the evidence for this association comes from early, less efficient, and lower nicotine e-cigarettes than are available and widely used now. The goal of this study was to examine the relationship between e-cigarette use in 2018 and subsequent smoking initiation and continued e-cigarette use. Methods: Participants included members of a national longitudinal panel of youth and young adults aged 15–27 who, in 2017, reported never having used a nicotine containing product (n = 3360). Logistic regression analyses assessed associations between participants' self-reported ever e-cigarette use in 2018 and ever cigarette use, current cigarette use, and current e-cigarette use in 2019, after controlling for demographic and psychosocial variables. Results: Compared with those who still had never used an e-cigarette, those who reported ever e-cigarette use in 2018 had significantly higher odds of ever cigarette use (aOR = 7.29, 95% CI [4.10, 12.97]), current cigarette use (aOR = 8.26, 95% CI [3.17, 21.53]), and current e-cigarette use (aOR = 9.70, 95% CI [6.41, 14.69]) one year later in 2019. Conclusions: These findings show that the pod mod style, high nicotine containing e-cigarettes subject young users to the same risks of transitioning to combustible cigarettes as their earlier, less efficient predecessors. Strong regulation of all nicotine products, including e-cigarettes, is needed to prevent the trajectory of e-cigarette to cigarette use among youth and young adults.
6140	Hair, Elizabeth C., Kreslake, Jennifer M., Mowery, Paul, Pitzer, Lindsay, Schillo, Barbara, Vallone, Donna M. (2021) A longitudinal analysis of e-cigarette use and cigar, little cigar or cigarillo initiation among youth and youth adults: 2017–2019. Drug and Alcohol Dependence; 226<br><br>**Keywords:**<br>Adolescents Cigars E-cigarettes Longitudinal data Tobacco use Young adults Embase MEDLINE cannabis controlled study current smoker vaping male adult epidemic human cigar young adult article adolescent female cohort analysis probability major clinical study juvenile<br><br>**Abstract:** Purpose: To examine whether youth and young adult e-cigarette use is associated with initiation of cigars, little cigars, or cigarillos (CLCCs) and current use of flavored CLCCs. Basic procedures: The sample is drawn from the Truth Longitudinal Cohort, a probability-based longitudinal cohort of youth and young adults recruited at ages 15–21 and surveyed every six months. The sample for this study was CLCC-naïve defined as those who had never used CLCCs as of 2017 (N = 5586). The outcomes were the odds of (1) initiating any CLCC use and (2) reporting current (past 30-day) use of flavored CLCCs from 2018 to late 2019. The main predictor was use of e-cigarettes by 2018. Results: The odds of initiating a CLCC was greater for those who had used ever used JUUL (OR: 3.30, p < 0.001) or were current users of another type of e-cigarette by 2018 (OR: 3.57, p < 0.001). Odds of CLCC initiation was also greater for those who had ever used combustible cigarettes (OR: 1.62, p < 0.05), were current smokers (OR: 3.12, p < 0.001) or had used marijuana (OR: 1.92, p < 0.001) by 2018. E-cigarette use that occurred by 2018 was associated with greater odds of current use of flavored CLCCs compared to non-flavored CLCCs (ever users of JUUL: OR: 2.57, p < 0.01; current users of some other e-cigarette: OR: 3.06, p < 0.05). Conclusion and relevance: This study raises new concerns about the effects of e-cigarette use on subsequent combustible tobacco use. Restrictions on CLCCs should be considered in conjunction with current policies designed to reduce the youth vaping epidemic.
6147	Hallingberg, Britt, Maynard, Olivia M., Bauld, Linda, Brown, Rachel, Gray, Linsay, Lowthian, Emily, MacKintosh, Anne-Marie, Moore, Laurence, Munafo, Marcus R., Moore, Graham (2020) Have e-cigarettes renormalised or displaced youth smoking? Results of a segmented regression analysis of repeated cross sectional survey data in England, Scotland and Wales. Tobacco control; 29<br><br>**Keywords:**

	denormalization electronic nicotine delivery devices harm reduction public policy Index Medicus Regression Analysis Cross-Sectional Studies Interrupted Time Series Analysis England -- epidemiology Humans Surveys and Questionnaires Wales -- epidemiology Alcohol Drinking -- epidemiology Adolescent Marijuana Use -- epidemiology Male Female Scotland -- epidemiology Prevalence Tobacco Smoking -- epidemiology Vaping -- epidemiology Electronic Nicotine Delivery Systems -- statistics & numerical data<br><br>**Abstract:** OBJECTIVESTo examine whether during a period of limited e-cigarette regulation and rapid growth in their use, smoking began to become renormalised among young people.DESIGNInterrupted time-series analysis of repeated cross-sectional time-series data.SETTINGGreat Britain PARTICIPANTS: 248 324 young people aged approximately 13 and 15 years, from three national surveys during the years 1998-2015.INTERVENTIONUnregulated growth of e-cigarette use (following the year 2010, until 2015).OUTCOME MEASURESPrimary outcomes were prevalence of self-reported ever smoking and regular smoking. Secondary outcomes were attitudes towards smoking. Tertiary outcomes were ever use of cannabis and alcohol.RESULTSIn final models, no significant change was detected in the pre-existing trend for ever smoking (OR 1.01, CI 0.99 to 1.03). There was a marginally significant slowing in the rate of decline for regular smoking (OR 1.04, CI 1.00 to 1.08), accompanied by a larger slowing in the rate of decline of cannabis use (OR 1.21, CI 1.18 to 1.25) and alcohol use (OR 1.17, CI 1.14 to 1.19). In all models and subgroup analyses for smoking attitudes, an increased rate of decline was observed after 2010 (OR 0.88, CI 0.86 to 0.90). Models were robust to sensitivity analyses.CONCLUSIONSThere was a marginal slowing in the decline in regular smoking during the period following 2010, when e-cigarettes were emerging but relatively unregulated. However, these patterns were not unique to tobacco use and the decline in the acceptability of smoking behaviour among youth accelerated during this time. These analyses provide little evidence that renormalisation of youth smoking was occurring during a period of rapid growth and limited regulation of e-cigarettes from 2011 to 2015.TRIAL REGISTRATION NUMBERResearch registry number: researchregistry4336.
6162	Hanafin, Joan, Sunday, Salome, Clancy, Luke (2021) E-cigarettes and smoking in Irish teens: a logistic regression analysis of current (past 30-day) use of e-cigarettes. Journal of Public Health (Germany);<br><br>**Keywords:**<br>E-cigarettes Gender Smoking Social determinates Teenagers Tobacco control Embase random sample cannabis controlled study prevalence male Ireland sport human child article adolescent female tertiary education alcohol tobacco dependence social determinants of health cannabis use prevention major clinical study friend electronic cigarette<br><br>**Abstract:** Aim: Electronic cigarette (e-cigarette) use among Irish teenagers has risen significantly. In 2019, prevalence of current use (last 30 days) among 15–17-year-olds was 17.3%. We examine social determinants of adolescent e-cigarette current use. Subject and methods: A stratified random sample of 50 schools in Ireland was surveyed in 2019, part of the European School Survey Project on Alcohol and other Drugs (ESPAD), with 3495 students aged 15, 16, and 17. Bivariate and multivariable logistic regression [providing adjusted odds ratios (AORs)] analyses were performed using Stata version 16. Results: Current e-cigarette users were more likely to be male (AOR = 0.55, 95% CI:0.32–0.96, p <.01), younger (AOR = 0.34, 95% CI: 0.17–0.67, p = < .05), to participate in sport (AOR = 2.21, 95% CI: 1.05–4.65, p <.05), to have higher-educated parents (maternal higher education: AOR = 27.54, 95% CI: 1.50–505.77, p = < .05, paternal higher education: AOR = 2.44, 95% CI: 1.00–5.91, p <.05), and less likely to consider their families better off (AOR = 0.29, 95% CI: 0.13–0.65, p <.01), or to report familial support (AOR = 0.78, 95% CI: 0.64–0.95, p <.05). They were more likely to be cigarette smokers (AOR = 7.22, 95% CI: 3.97–13.12, p <.001), to report problem cannabis use (AOR = 3.12, 95% CI: 1.40–6.93, p <.01), to be 'binge' drinkers (AOR = 1.81, 95% CI : 1.00–3.32, p =.054), and to have friends who get drunk (AOR = 5.30, 95% CI: 1.34–20.86, p <.05). Conclusion: Boys, smokers, binge drinkers, problem cannabis users, and sport-playing teenagers from higher-educated families, are at particular risk. As the number of young people using e-cigarettes continues to rise, including teenagers who have never smoked, improved regulation of

	e-cigarettes, similar to other tobacco-related products, is needed urgently to prevent this worrying new trend of initiation into nicotine addiction.
6167	Harlow, Alyssa F., Fetterman, Jessica L., Ross, Craig S., Robertson, Rose Marie, Bhatnagar, Aruni, Benjamin, Emelia J., Stokes, Andrew C. (2021) Association of device type, flavours and vaping behaviour with tobacco product transitions among adult electronic cigarette users in the USA. Tobacco control;<br><br>**Keywords:**<br>cessation electronic nicotine delivery devices non-cigarette tobacco products MEDLINE controlled study demography disposable equipment nicotine ex-smoker current smoker vaping male flavor adult human article female major clinical study<br><br>**Abstract:** BACKGROUND: Few studies assess whether electronic cigarette (e-cigarette) device characteristics or flavours impact longitudinal patterns of cigarette and e-cigarette use. DESIGN: We examined data from waves 2-4 of the Population Assessment of Tobacco and Health Study (2014-2018). Among adult (≥18 years) current e-cigarette users at wave 2 who were current smokers (dual users; n=1759) and former smokers (exclusive e-cigarette users; n=470), we classified participants into four use patterns at wave 3 (~12 months later) and wave 4 (~24 months later): (1) dual use of e-cigarettes and cigarettes; (2) exclusive cigarette smoking; (3) exclusive e-cigarette use; (4) non-use of both products. We used multinomial logistic regression to assess correlates of changing use patterns at 24 months, relative to no change, adjusting for sociodemographic factors. RESULTS: At 24 months, 26.5% of baseline exclusive e-cigarette users, and 9% of baseline dual users, abstained from both vaping and smoking. Participants who vaped non-tobacco flavours (vs tobacco flavours), and used refillable tank or modifiable devices (vs disposable, cartridges and other devices) were less likely to transition to non-use of both products and to exclusive cigarette smoking. Baseline daily vaping (vs non-daily) was positively associated with exclusive e-cigarette use at 24 months for baseline daily cigarette smokers, but negatively associated with exclusive e-cigarette use and non-use of both products at 24 months for baseline non-daily smokers. CONCLUSIONS: Non-tobacco flavours, daily vaping and modifiable e-cigarette devices may help some smokers abstain from cigarette smoking via transitioning to exclusive e-cigarette use, but are also associated with ongoing exclusive e-cigarette use.
6169	Harrell, Melissa B., Chen, Baojiang, Clendennen, Stephanie L., Sumbe, Aslesha, Case, Kathleen R., Wilkinson, Anna V., Loukas, Alexandra, Perry, Cheryl L. (2021) Longitudinal trajectories of E-cigarette use among adolescents: A 5-year, multiple cohort study of vaping with and without marijuana. Preventive Medicine; 150<br><br>**Keywords:**<br>Adolescents E-cigarette use Marijuana use Tobacco use Embase MEDLINE cannabis controlled study demography middle school vaping male Texas adult growth curve adolescence human young adult article longitudinal study adolescent female cohort analysis primary prevention cannabis use major clinical study onset age<br><br>**Abstract:** E-cigarette use harms adolescent health, yet it continues to escalate rapidly among teens nationwide. This longitudinal study sought to identify and differentiate between developmental trajectories of past 30-day e-cigarette use with and without marijuana (i.e., liquid THC) across adolescence (11–19 years old). Three population-based cohorts of adolescents (n = 3907; N = 461,069) living in major metropolitan areas of Texas (Houston, Dallas-Ft. Worth, San Antonio, Austin) completed up to 9 Waves of an e-cigarette use survey, from 2014 to 2019. Growth curve models (GCMs) were used to identify average trajectories of past 30-day e-cigarette use, by cohort. Growth mixture models (GMMs) were used to investigate developmental patterns in these trajectories, by cohort. Sociodemographic differences in trajectories were also investigated. Stable trajectories of e-cigarette use with and without marijuana were identified, from 11 through 19 years of age. Trajectories varied by age of onset; frequency and escalation in use; and substance used. With one exception, all trajectories of e-cigarette

	use escalated with age. Moreover, age of onset and progression in use were positively related. The most problematic trajectories, corresponding to more frequent use, were observed among the younger cohorts compared to the oldest. Primary prevention is critical. Interventions to prevent the onset and progression in e-cigarette use among teens must begin early (e.g., in middle school) and be sustained throughout adolescence.
6221	Hillyer, Grace C., Nazareth, Meaghan, Lima, Sarah, Schmitt, Karen M., Reyes, Andria, Fleck, Elaine, Schwartz, Gary K., Terry, Mary Beth (2021) E-cigarette Use Among Young Adult Patients: The Opportunity to Intervene on Risky Lifestyle Behaviors to Reduce Cancer Risk. Journal of community health;<br><br>**Keywords:**<br>Binge alcohol consumption E-cigarettes Electronic nicotine delivery systems Tobacco smoking Young adults MEDLINE controlled study male people by smoking status adult binge drinking human risk reduction passive smoking young adult article female alcohol consumption lifestyle cancer risk major clinical study electronic cigarette<br><br>**Abstract:** Use of e-cigarettes and other electronic nicotine delivery systems (ENDS) is on the rise. We administered a health needs survey via email to 804 adult primary care and oncology patients at a large urban academic medical center in 2019. We examined differences in e-cigarette use by smoking status, personal history of cancer, alcohol use, and second-hand tobacco smoke exposure. Of the 804 participants, 90 (11.2%) reported ever using e-cigarettes. E-cigarette use was more prevalent in young adults (risk ratio [RR] for 18-24 years: 4.58, 95% confidence interval [95% CI] 2.05, 10.26), current smoking (RR 4.64, 95% CI 1.94, 11.07), very often/often binge drinking (RR 3.04, 96% CI 1.38, 6.73), and ≥ 1 smokers in the home (RR 3.90, 95% CI 2.10, 7.23). Binge alcohol consumption and tobacco smoking are associated with increased risk cancer. Inquiries about e-cigarette use among adults 25-40 years present providers the opportunity to also counsel young adult about reducing cancer risk.
6224	Hirschtick, Jana L., Mattingly, Delvon T., Cho, Beomyoung, Arciniega, Luis Zavala, Levy, David T., Sanchez-Romero, Luz Maria, Jeon, Jihyoun, Land, Stephanie R., Mistry, Ritesh, Meza, Rafael, Fleischer, Nancy L. (2021) Exclusive, Dual, and Polytobacco Use Among US Adults by Sociodemographic Factors: Results From 3 Nationally Representative Surveys. American journal of health promotion : AJHP; 35<br><br>**Keywords:**<br>dual use health disparities inequities multiple tobacco products patterns of tobacco use polytobacco Health Technology Assessment Tobacco Use -- epidemiology Humans Adult Surveys and Questionnaires United States -- epidemiology Male Electronic Nicotine Delivery Systems Tobacco Products Tobacco, Smokeless<br><br>**Abstract:** PURPOSETo provide tobacco product use patterns for US adults by sociodemographic group.DESIGNA secondary analysis of Tobacco Use Supplement to the Current Population Survey (2014-15), National Health Interview Survey (2015), and Population Assessment of Tobacco and Health (2015-16).SETTINGUnited States.SAMPLEThree nationally representative samples of adults (N = 28,070-155, 067).MEASURESAll possible combinations of cigarette, Electronic Nicotine Delivery Systems (ENDS), other combustible product, and smokeless tobacco use, defined as current use every day or some days.ANALYSISWeighted population prevalence and proportion among tobacco users of exclusive, dual, and polyuse patterns by sex, race/ethnicity, education, income, and age.RESULTSExclusive cigarette use was the most prevalent pattern (10.9-12.8% of US population). Dual and polyuse were less prevalent at the population level (2.6-5.2% and 0.3-1.3%, respectively) but represented 16.7-25.5% of product use among tobacco users. Cigarette plus ENDS use was similar by sex, but men were more likely to be dual users of cigarettes plus other combustibles or smokeless tobacco. Among race/ethnic subgroups, non-Hispanic (NH) Whites were most likely to use cigarettes plus ENDS, while NH Blacks were most likely to use cigarettes plus other combustibles. Dual and polyuse were generally less common among adults with higher education, income, and age.CONCLUSIONDifferences in product use patterns by

	sociodemographic group likely represent different risk profiles with important implications for resulting health disparities.
6248	Hughes, Jason, Sykes, Grace, Hughes, Kahryn, O'Reilly, Michelle, Goodwin, John, Sutton, Charlie, Karim, Khalid (2021) From gateways to multilinear connections: A qualitative longitudinal investigation of the relationships between vaping and smoking among adolescent users. International Journal of Drug Policy; 97<br><br>**Keywords:**<br>Adolescents E-cigarettes Gateway theory Logics of connection Smoking Trajectories Embase MEDLINE risk behavior article interview adolescent vaping career genetic transcription logic theoretical study human<br><br>**Abstract:** Background: The international growth of e-cigarette use has been accompanied by a corresponding concern that e-cigarettes will act as a 'gateway' to smoking and the use of other drugs. Taking these concerns as our point of departure, we explore the relationships between vaping and smoking among a cohort of young people. Methods: Qualitative longitudinal methods with a diverse sample of 36 14–18-year olds from the UK city of Leicester. A total of 66 depth interviews conducted across two phases separated by 6–12 months. The interviews were recorded, transcribed verbatim and thematically analysed. Findings: We highlight a complex 'tangle' of connections between substances/risk behaviours recounted to us by our adolescent study participants, including multiple and multilinear relationships between vaping and smoking. These findings problematise some of the core axioms of the notion of gateways as an explanatory model of causality and sequential connection between smoking and vaping. They also throw into question gateway logics more fundamentally. While many of our study participants themselves consciously invoked ideas of 'gateway effects', the accounts they produced repeatedly disrupted the logics of connection (between e-cigarettes and smoking; one set of behaviours and another) presupposed in gateway theory and our own early lines of questioning. Accordingly, we explore how cultural understandings of gateway effects are invoked by users in accounting for their vaping and smoking behaviours, noting the potential influence of these ideas upon the very processes they are understood to apprehend. Conclusion: Our findings suggest there is a case to be made to reinforce the distinctiveness of tobacco and e-cigarettes in the life-worlds of young people to avoid naturalising a 'gateway' logic of connection that might ultimately inform the associative logic of young users themselves, and potentially the development of their usage careers.
6271	Ilic, Irena, Grujicic Sipetic, Sandra, Radovanovic, Djordje, Ilic, Milena (2020) Cigarette Smoking and E-Cigarette Use by Pharmacy Students in Serbia. Behavioral Medicine; 46<br><br>**Keywords:**<br>Cross-sectional study e-cigarettes pharmacy students Embase MEDLINE 17:Public Health, Social Medicine and Epidemiology Serbia pharmacy student cigarette smoking ex-smoker funding current smoker vaping male Article human non-smoker female age alcohol consumption student attitude psychotropic agent<br><br>**Abstract:** The purpose of this study was to assess the use of e-cigarettes among pharmacy students. In 2016, we conducted a cross-sectional study among pharmacy students enrolled at the University of Kragujevac (Serbia). Overall, 47.0% of pharmacy students reported that they ever smoked cigarettes; 29.7% were former and 17.3% were current smokers. The overall prevalence of e-cigarette use was 9.9%. The final year pharmacy students significantly more frequently smoked tobacco cigarettes (p =.001) and used e-cigarettes (p =.009) compared to younger students. Also, alcohol use was positively related to ever smoking tobacco cigarettes (adjusted OR = 4.57, 95% CI = 2.80–7.43; p <.001) and to ever use of e-cigarettes (adjusted OR = 5.58, 95% CI = 1.58–19.71; p =.008). The pharmacy students who ever used e-cigarettes more frequently reported a history of self-funded study financing (adjusted OR = 14.68, 95% CI = 2.42–89.17; p =.004) and use of psychoactive substances (adjusted OR = 13.63, 95% CI = 2.52–73.69; p =.002). In pharmacy students, a higher overall grade was related to a significantly less frequent ever use of tobacco cigarettes (adjusted OR = 0.43, 95% CI = 0.22–0.84, p =.015). This paper highlights the need for

	a more effective tobacco control among pharmacy students in Serbia in order to reduce smoking prevalence.
6352	Kaleta, Dorota, Polanska, Kinga (2021) Trends of e-cigarettes and tobacco use among secondary and high school students from Poland over three years observation. Tobacco Induced Diseases; 19<br><br>**Keywords:**<br>E-cigarettes Tobacco use Trends Youth Embase controlled study high school student cigarette smoking funding prevalence male writing trend study conference abstract cross-sectional study human child questionnaire female smoking Poland government major clinical study electronic cigarette<br><br>**Abstract:** Introduction: Since electronic cigarettes (e-cigarettes) became available on the market, they have gained unexpectedly a large popularity, especially among young people. Objectives: The aim of current study was to evaluate the trends of e-cigarettes and tobacco use over the 3 years period among youth from Poland. Methods: A cross-sectional study was conducted between 2014/2015 (N=3552) and 2017/2018 (N=2509) among secondary and high school students from Piotrkowski district. The anonymous, self-administered questionnaire adapted from the Global Youth Tobacco Survey was implemented to collect data. Results: Despite the amendment of Tobacco Control Act regulating e-cigarette use among youth, the percentages of the secondary school girls who currently use that products has been increased over 3 years period (from 20.7% to 31.7%; p<0.001). Among high school students that percentages have not been changed significantly (p>0.05). What is more, the prevalence of current smoking of traditional cigarettes was stable among secondary school students and increased among youth from high schools (from 25.1% to 27.9%; p <0.05). Among the younger students the decrement in dual use (smoking of traditional cigarettes and e-cigarettes use) has been noted (from 40.6% to 16.8%; p<0.001) whereas among the older boys the increasing pattern of dual use has been identified (45.7% vs. 56.8% p<0.01%). Conclusion(s): E-cigarette and tobacco use is widespread in the investigated population. There is a need for further research and preventive measures to protect the youth from that exposure. This study was supported by the Local Government of the Piotrkowski District (grant number: AZ-I.033.2.2014), Medical University of Lodz (grant no. 503/6-024-01/503-66-001. The funding bodies had no role in the design of the study and collection, analysis, and interpretation of the data, and in writing the manuscript.
6362	Kang, Heewon, Cho, Sung-Il (2020) Longitudinal transitions of cigarettes and electronic nicotine delivery systems among adolescents: Construction of a retrospective cohort using recall data from a cross-sectional sample. Tobacco Induced Diseases; 18<br><br>**Keywords:**<br>Adolescents Gateway effect Longitudinal transitions Non-cigarette tobacco/nicotine products nicotine 54-11-5 Embase 17:Public Health, Social Medicine and Epidemiology 7:Pediatrics and Pediatric Surgery cigarette controlled study cigarette smoking current smoker smoking cessation prevalence male never smoker retrospective study Korean (people) priority journal human Article cross-sectional study health survey longitudinal study adolescent female cohort analysis Markov chain electronic cigarette<br><br>**Abstract:** Introduction: A major concern regarding non-cigarette tobacco or nicotine products (NCTNPs) is whether they facilitate or mitigate overall tobacco or nicotine use. We examined longitudinal transitions of cigarettes and electronic nicotine delivery systems (ENDS) by constructing a retrospective cohort based on the recall data of a cross-sectional sample. Methods: Using the Korea Youth Risk Behavior Survey, we constructed crosssectional data of 59576 adolescents into retrospective cohort data. Participants were categorized into 4 mutually exclusive tobacco or nicotine use states. We used a multistate Markov model to identify transitions between the states to calculate transition intensity ratios (TIRs), and examined the current use of tobacco or nicotine products to assess both gateway effects to cigarettes, and whether ENDS use helps adolescents quit cigarette smoking. Results: Compared with never use, use of ENDS was associated with an increased risk of initiation of cigarette use (TIR=6.8; 95%

	CI: 4.5-10.2). The risk of transitioning from cigarette ever use to ENDS, compared with never use to ENDS, was even more pronounced (TIR=44.1; 95% CI: 34.1-56.9). The prevalence of current cigarette smoking was higher among those who started ENDS then cigarettes, compared to those who began cigarette use without experimenting with ENDS (43.1% vs 35.8%). Moreover, 27.8% (95% CI: 23.6-32.0%) of adolescents who experimented first with cigarettes then moved to ENDS were current users of cigarettes, and 46.4% (95% CI: 42.1-51.1%) of these adolescents were current users of both cigarettes and ENDS. Conclusions: Based on the recall data of a cross-sectional sample, we demonstrate that ENDS experimentation increases the likelihood of cigarette smoking initiation. A significant proportion of these adolescents continue to use cigarettes. Moreover, those who experimented with cigarettes then ENDS also continue smoking cigarettes or both cigarettes and ENDS. We suggest comprehensive tobacco control policies for all tobacco/nicotine products and monitoring the timing of NCTNP initiation in cross-sectional surveys.
6402	Keller-Hamilton, Brittney, Lu, Bo, Roberts, Megan E., Berman, Micah L., Root, Elisabeth D., Ferketich, Amy K. (2021) Electronic cigarette use and risk of cigarette and smokeless tobacco initiation among adolescent boys: A propensity score matched analysis. Addictive Behaviors; 114<br><br>**Keywords:**<br>Adolescents Cigarettes Electronic cigarettes Smokeless tobacco Embase MEDLINE 17:Public Health, Social Medicine and Epidemiology controlled study cigarette smoking follow up tobacco consumption current smoker urban area male observational study propensity score Article human child data analysis adolescent cohort analysis risk assessment adolescent smoking Ohio electronic cigarette<br><br>**Abstract:** Introduction: Electronic cigarette (e-cigarette) use among adolescents is associated with increased risk of subsequent cigarette smoking initiation in observational research. However, the existing research was not designed to answer causal questions about whether adolescent e-cigarette users would have initiated cigarette smoking if they had never used e-cigarettes. The current study used a causal inference framework to identify whether male adolescent e-cigarette users were at increased risk of initiating cigarette smoking and smokeless tobacco (SLT) use, compared to similar boys who had never used e-cigarettes. Methods: Boys from urban and Appalachian Ohio (N = 1220; ages 11–16 years at enrollment) reported use of e-cigarettes, cigarettes, and SLT at baseline and every six months for two years. A propensity score matching design was implemented, matching one e-cigarette user to two similar e-cigarette non-users. This analysis was completed in 25 multiple imputed datasets to account for missing data. Risk ratios (RRs) comparing risk of initiating cigarettes and SLT for e-cigarette users and nonusers were estimated. Results: Compared to non-users, e-cigarette users were more than twice as likely to later initiate both cigarette smoking (RR = 2.71; 95% CI: 1.89, 3.87) and SLT (RR = 2.42; 95% CI: 1.73, 3.38). They were also more likely to become current (i.e., past 30-day) cigarette smokers (RR = 2.20; 95% CI: 1.33, 3.64) and SLT users (RR = 1.64; 95% CI: 1.01, 2.64). Conclusions: Adolescent boys who used e-cigarettes had increased risk of later initiating traditional tobacco products when compared to similar boys who had never used e-cigarettes.
6421	Khouja, Jasmine N., Wootton, Robyn E., Taylor, Amy E., Smith, George Davey, Munafò, Marcus R. (2021) Association of genetic liability to smoking initiation with e-cigarette use in young adults: A cohort study. PLoS Medicine; 18<br><br>**Keywords:**<br>Embase MEDLINE controlled study adulthood genome-wide association study genetic association high risk behavior child young adult sexuality Berkson's bias nicotine male never smoker phenotype adult gambling human sample size genetic risk score prospective study article female longitudinal study cohort analysis alcohol alcohol consumption childhood disease conduct disorder major clinical study electronic cigarette<br><br>**Abstract:** Background Tobacco smoking and e-cigarette use are strongly associated, but it is currently

	unclear whether this association is causal, or due to shared factors that influence both behaviours such as a shared genetic liability. The aim of this study was to investigate whether polygenic risk scores (PRS) for smoking initiation are associated with ever use of e-cigarettes. Methods and findings Smoking initiation PRS were calculated for young adults (N = 7,859, mean age = 24 years, 51% male) of European ancestry in the Avon Longitudinal Study of Parents and Children, a prospective birth cohort study initiated in 1991. PRS were calculated using the GWAS & Sequencing Consortium of Alcohol and Nicotine use (GSCAN) summary statistics. Five thresholds ranging from 5 × 10−8 to 0.5 were used to calculate 5 PRS for each individual. Using logistic regression, we investigated the association between smoking initiation PRS and the main outcome, self-reported e-cigarette use (n = 2,894, measured between 2016 and 2017), as well as self-reported smoking initiation and 8 negative control outcomes (socioeconomic position at birth, externalising disorders in childhood, and risk-taking in young adulthood). A total of 878 young adults (30%) had ever used e-cigarettes at 24 years, and 150 (5%) were regular e-cigarette users at 24 years. We observed positive associations of similar magnitude between smoking initiation PRS (created using the p < 5 × 10−8 threshold) and both smoking initiation (odds ratio (OR) = 1.29, 95% CI 1.19 to 1.39, p < 0.001) and ever e-cigarette use (OR = 1.24, 95% CI 1.14 to 1.34, p < 0.001) by the age of 24 years, indicating that a genetic predisposition to smoking initiation is associated with an increased risk of using e-cigarettes. At lower p-value thresholds, we observed an association between smoking initiation PRS and ever e-cigarette use among never smokers. We also found evidence of associations between smoking initiation PRS and some negative control outcomes, particularly when less stringent p-value thresholds were used to create the PRS, but also at the strictest threshold (e.g., gambling, number of sexual partners, conduct disorder at 7 years, and parental socioeconomic position at birth). However, this study is limited by the relatively small sample size and potential for collider bias. Conclusions Our results indicate that there may be a shared genetic aetiology between smoking and e-cigarette use, and also with socioeconomic position, externalising disorders in childhood, and risky behaviour more generally. This indicates that there may be a common genetic vulnerability to both smoking and e-cigarette use, which may reflect a broad risk-taking phenotype.
6423	Kianersi, Sina, Zhang, Yijia, Rosenberg, Molly, Macy, Jonathan T. (2021) Prevalence of e-cigarette use (2016 to 2018) and cigarette smoking (2012 to 2019) among U.S. adults by state: An interactive data visualization dashboard. Drug and Alcohol Dependence; 218<br><br>**Keywords:**<br>Embase MEDLINE 17:Public Health, Social Medicine and Epidemiology United States cigarette smoking vaping self report prevalence trend study standardization Letter priority journal population research human electronic cigarette<br><br>**Abstract:**
6435	Kim, Sooyong, Selya, Arielle S. (2020) The relationship between electronic cigarette use and conventional cigarette smoking is largely attributable to shared risk factors. Nicotine & Tobacco Research; 22<br><br>**Keywords:**<br>electronic cigarette use conventional smoking risk factors life span Empirical Study Quantitative Study Human Male Female Adolescence (13-17 yrs) Adulthood (18 yrs & older) Electronic Cigarettes Vaping Adolescent Health Tobacco Smoking US article 2840:Psychosocial & Personality Development Cross-Sectional Studies Cigarette Smoking Electronic Nicotine Delivery Systems Smokers Humans Adolescent<br><br>**Abstract:** Introduction: The growing popularity of electronic cigarettes (e-cigarettes) among youth raises concerns about possible causal effects on conventional cigarette smoking. However, past research remains inconclusive due to heavy confounding between cigarette and e-cigarette use. This study uses propensity score methods to robustly adjust for shared risk in estimating the relationship between e-cigarette use and conventional smoking. Methods: Cross-sectional data from 8th and 10th graders were drawn from the 2015–2016 waves of Monitoring the Future (n = 12 421). The effects of (1) lifetime and

	(2) current e-cigarette use on (A) lifetime and (B) current conventional cigarette smoking were examined using logistic regression analyses with inverse propensity weighting based on 14 associated risk factors. Results: After accounting for the propensity for using e-cigarettes based on 14 risk factors, both lifetime and current e-cigarette use significantly increased the risk of ever smoking a conventional cigarette (OR = 2.49, 95% CI = 1.77 to 3.51; OR = 2.32, 95% CI = 1.66 to 3.25, respectively). However, lifetime (OR = 2.17, 95% CI = 0.62 to 7.63) and current e-cigarette use (OR = 0.95, 95% CI = 0.55 to 1.63) did not significantly increase the risk of current conventional cigarette smoking. Conclusions: E-cigarette use does not appear to be associated with current, continued smoking. Instead, the apparent relationship between e-cigarette use and current conventional smoking is fully explained by shared risk factors, thus failing to support claims that e-cigarettes have a causal effect on concurrent conventional smoking among youth. E-cigarette use has a remaining association with lifetime cigarette smoking after propensity score adjustment; however, future research is needed to determine whether this is a causal relationship or merely reflects unmeasured confounding. Implications: This study examines the relationship between e-cigarette use and conventional smoking using inverse propensity score weighting, an innovative statistical method that produces less-biased results in the presence of heavy confounding. Our findings show that the apparent relationship between e-cigarette use and current cigarette smoking is entirely attributable to shared risk factors for tobacco use. However, e-cigarette use is associated with lifetime cigarette smoking, though further research is needed to determine whether this is a causal relationship or merely reflects unaccounted-for confounding. Propensity score weighting produced significantly weaker effect estimations compared to conventional regression control. (PsycInfo Database Record (c) 2021 APA, all rights reserved) (Source: journal abstract)
6453	Kinnunen, Jaana M., Rimpelä, Arja H., Lindfors, Pirjo L., Clancy, Luke, Alves, Joana, Hoffmann, Laura, Richter, Matthias, Kunst, Anton E., Lorant, Vincent (2021) Electronic cigarette use among 14- to 17-year-olds in Europe. European journal of public health; 31<br><br>**Keywords:**<br>MEDLINE female adolescent smoking vaping male Europe epidemiology human electronic cigarette cross-sectional study<br><br>**Abstract:** BACKGROUND: Studies comparing adolescent e-cigarette use in different countries are scarce. We study students' e-cigarette and conventional cigarette ever-use, their social correlates and e-liquid use in seven EU countries. METHODS: SILNE-R data (N=12 167, response rate 79.4%) of 14-17-year-olds from Amersfoort (NL), Coimbra (PT), Dublin (IR), Hanover (GE), Latina (IT), Namur (BE) and Tampere (FI) were used. E-cigarette and conventional cigarette ever-use, dual-use, type of e-liquid and social correlates were measured with a school survey and analyzed with cross-tabulations and multinomial logistic regression. RESULTS: About 34% had tried e-cigarettes, but the variation was large between the cities (Latina 50%; Hanover 23%). Of e-cigarette ever-users, 37% had used nicotine e-liquid, 43% exclusively non-nicotine liquid and 20% did not know the content. Nicotine e-liquid was more prevalent among monthly e-cigarette users and weekly smoking e-cigarette users. The social correlates were mainly the same for exclusive e-cigarette ever-use, exclusive conventional cigarette ever-use and dual-use. Boys had greater odds for exclusive e-cigarette and dual-use compared to girls. Of social correlates, low academic achievement and parental smoking were positively associated with all categories of use, but parental education and immigrant background were not. The strongest association was found between peer smoking (most/all best friends smoke) and dual-use (OR 34.29). CONCLUSIONS: Students' e-cigarette ever-use varies greatly between EU countries. E-cigarettes seem not to be a substitute for conventional cigarettes but more a complementary product. Tobacco control policies might also prevent e-cigarette use but specific regulations on e-cigarettes are needed to prevent nicotine addiction originating from them.
6491	Krishnan, Nandita, Abroms, Lorien C., Berg, Carla J. (2021) Electronic nicotine product cessation and cigarette smoking: analysis of waves 3 and 4 from the PATH study. Nicotine & tobacco research : official journal of the Society for Research on Nicotine and Tobacco;

	**Keywords:**<br>MEDLINE demography nicotine cigarette smoking household smoking cessation male adult clinical article human article female non-smoker case report<br><br>**Abstract:** INTRODUCTION: Identifying predictors of electronic nicotine product (ENP) cessation can inform ENP cessation interventions. High rates of co-occurring ENP and cigarette (dual) use and transitions between these products underscore the importance of considering cigarette smoking status when assessing and addressing ENP cessation. METHODS: We analyzed waves 3 (W3) and 4 (W4) of the Population Assessment of Tobacco and Health (PATH) study to identify (i) W3 socio-demographics, tobacco and ENP use characteristics, and psychosocial correlates of W3 cigarette smoking status (non-smoker, former and current) among W3 adult ENP users, and (ii) W3 predictors of W4 combined ENP and cigarette smoking abstinence relative to use of one or both products. RESULTS: At W3, 65.6% of ENP users concurrently smoked cigarettes. Adjusted multinomial regression results indicated that different W3 socio-demographics, tobacco and ENP use characteristics and psychosocial correlates were significantly associated with distinct W3 cigarette use profiles. At W4, 9.9% of individuals were abstinent from both products. These individuals were less likely to: (i) be current smokers (vs non-smokers) or be advised to quit using tobacco, compared to cigarette only or dual users, and (ii) use ENPs daily or live in a household allowing ENP use, compared to ENP only or dual users (p's <0.05). CONCLUSIONS: ENP cessation approaches need to be tailored to the distinct cigarette use profiles of ENP users. Dual users and daily ENP users may require more intensive interventions to achieve cessation of both products. Supportive physical environments, such as home vape-free policies, may facilitate ENP cessation. IMPLICATIONS: This analysis contributes to advancing the nascent literature on predictors of electronic nicotine product (ENP) cessation, which can guide the development of ENP cessation interventions by indicating which populations, psychosocial and environmental constructs and co-occurring behaviors interventions should target. This research also highlights the importance of considering cigarette smoking status when designing ENP cessation interventions and defining intervention outcomes.
6512	Kwon, Elizabeth, Adams, Zachary, Seo, Dong-Chul (2021) Trajectories and determinants of adolescents' nicotine product use risk among U.S. adolescents in a nationally representative sample of longitudinal cohort. Addictive Behaviors; 116<br><br>**Keywords:**<br>Adolescence Nicotine product use Trajectory nicotine 54-11-5 Embase MEDLINE 40:Drug Dependence, Alcohol Abuse and Alcoholism 32:Psychiatry United States controlled study demography illness trajectory cigarette smoking vaping environmental factor trend study health status observational study logistic regression analysis child adolescent tobacco dependence cannabis use risk factor adolescent smoking prevalence male population research Article human social network cigar clinical study female longitudinal study cohort analysis mental health alcohol consumption major clinical study electronic cigarette<br><br>**Abstract:** Introduction: To understand the heterogeneous longitudinal trends in adolescent nicotine product use risk, we investigated the trajectories and determinants of nicotine product use among adolescents. Methods: Using Waves 1–4 (Years 2013–2018) data of the Population Assessment of Tobacco and Health Study, we conducted group-based trajectory modeling to identify groups of individuals who followed homogeneous trajectories of nicotine product use risk over time and weighted multinomial logistic regression to examine relative risk ratios (RRRs) of different levels of predictors of group membership in each identified trajectory. Results: For adolescents, who were 12–17 years old at baseline (N = 10,086), 5 trajectory groups emerged: (1) nonusers (73.7%); (2) stable low to moderate risk of developing poly use (5.0%); (3) gradual increase in risk of poly use (12.8%); (4) rapid linear increase in risk of poly use until Wave 3 and then plateauing for cigarettes or slightly diminishing for e-cigarettes and other tobacco products at Wave 4 (4.6%); and (5) stable moderate to high risk of poly use (3.9%). Adolescents who used social network services daily at Wave 1 were more likely to belong to each of the

	four trajectory groups compared to nonusers (RRRs ranging from 1.43 to 2.04). Conclusions: The current study findings imply that the majority of adolescents who initiated any nicotine product use have an elevated risk of transitioning to poly users. Risk and protective factors for different risk patterns are discussed.
6573	Legleye, Stéphane, Aubin, Henri-Jean, Falissard, Bruno, Beck, François, Spilka, Stanislas (2021) Experimenting first with e-cigarettes versus first with cigarettes and transition to daily cigarette use among adolescents: the crucial effect of age at first experiment. Addiction (Abingdon, England); 116<br><br>**Keywords:**<br>Adolescents age at initiation E-cigarette propensity score tobacco smoking transition MEDLINE adolescent smoking tobacco epidemiology illicit drug human electronic cigarette cross-sectional study<br><br>**Abstract:** BACKGROUND AND AIMS: Most studies in English-speaking countries have found a positive association between e-cigarette experimentation and subsequent daily tobacco smoking among adolescents. However, this result may not be valid in other cultural contexts; in addition, few studies have assessed whether this association varies with the subject' age at the time of e-cigarette experimentation. This study aimed to estimate the association between experimenting first with e-cigarette (rather than tobacco) and subsequent daily smoking according to age at the time of experimentation. DESIGN: Secondary analysis; risk ratios (RRs) computed using modified Poisson regressions with inverse probability weighting. SETTING: A cross-sectional nation-wide representative survey performed in 2017 in France. PARTICIPANTS: French adolescents (n = 24 111), aged 17 to 18.5 years, who had previously experimented with either e-cigarettes or tobacco. MEASURES: Exposure was defined as the experimentation with e-cigarettes first (whether or not followed by experimentation with tobacco); the outcome as daily tobacco smoking at the time of data collection. Gender, age, literacy, socio-economic status, pre-exposure repeat school years and experimentation with drunkeness, 3 licit and 8 illicit drugs were adjusted for. Uncertainties about the sequence of events defining exposure were handled by the definition of three patterns of exposure, to avoid a misclassification bias. FINDINGS: Exposure reduced the risk of transition to daily smoking: RR = 0.58, 95% confidence interval (CI) = 0.54, 0.62. This effect increased in a linear manner with age at exposure (RR = 0.87, 95% CI = 0.78; 0.98 for 1 year, P < 0.001): from RR = 1.30, 95% CI = 1.09; 1.54 at age 9 to RR = 0.38, 95% CI = 0.32; 0.45 at age 17. CONCLUSIONS: Experimenting with e-cigarettes first (as opposed to tobacco first) appears to be associated with a reduction in the risk of daily tobacco smoking among French adolescents aged 17-18.5, but this risk varies negatively with age at experimentation, and early e-cigarette experimenters are at higher risk.
6591	Levy, David T., Tam, Jamie, Sanchez-Romero, Luz María, Li, Yameng, Yuan, Zhe, Jeon, Jihyoun, Meza, Rafael (2021) Public health implications of vaping in the USA: the smoking and vaping simulation model. Population Health Metrics; 19<br><br>**Keywords:**<br>NVPs Public health Simulation model Smoking Vaping Embase MEDLINE 17:Public Health, Social Medicine and Epidemiology 32:Psychiatry United States nicotine smoking cessation male adult middle aged mortality rate simulation priority journal aged Article health survey human female sensitivity analysis cohort analysis validation process risk factor health care policy<br><br>**Abstract:** Background: Nicotine vaping products (NVPs) are increasingly popular worldwide. They may provide public health benefits if used as a substitute for smoking, but may create public health harms if used as a gateway to smoking or to discourage smoking cessation. This paper presents the Smoking and Vaping Model (SAVM), a user-friendly model which estimates the public health implications of NVPs in the USA. Methods: SAVM adopts a cohort approach. We derive public health implications by comparing smoking- and NVP-attributable deaths and life-years lost under a No-NVP and an NVP Scenario. The No-NVP Scenario projects current, former, and never smoking rates via smoking initiation and cessation

	rates, with their respective mortality rates. The NVP Scenario allows for smoking- and NVP-specific mortality rates, switching from cigarette to NVP use, separate NVP and smoking initiation rates, and separate NVP and smoking cessation rates. After validating the model against recent US survey data, we present the base model with extensive sensitivity analyses. Results: The SAVM projects that under current patterns of US NVP use and substitution, NVP use will translate into 1.8 million premature smoking- and vaping-attributable deaths avoided and 38.9 million life-years gained between 2013 and 2060. When the NVP relative risk is set to 5%, the results are sensitive to the level of switching and smoking cessation rates and to a lesser extent smoking initiation rates. When the NVP relative risk is raised to 40%, the public health gains in terms of averted deaths and LYL are reduced by 42% in the base case, and the results become much more sensitive to variations in the base case parameters. Discussion: Policymakers, researchers, and other public health stakeholders can apply the SAVM to estimate the potential public health impact of NVPs in their country or region using their own data sources. In developing new simulation models involving NVPs, it will be important to conduct extensive sensitivity analysis and continually update and validate with new data. Conclusion: The SAVM indicates the potential benefits of NVP use. However, given the uncertainty surrounding model parameters, extensive sensitivity analysis becomes particularly important.
6645	Lotrean, Lucia Maria, Man, Milena, Gavrilescu, Cristina, Florea, Mira (2021) Electronic Cigarette Use and Its Relationship with Smoking and Alcohol and Illicit Drug Consumption among Romanian University Students. Medicina (Kaunas, Lithuania); 57<br><br>**Keywords:**<br>Romania e-cigarettes health risk behaviors youth Illicit Drugs Index Medicus Cross-Sectional Studies Humans Surveys and Questionnaires Romania -- epidemiology Universities Students Adolescent Smoking -- epidemiology Vaping Electronic Nicotine Delivery Systems<br><br>**Abstract:** Background and objectives: This study assesses electronic cigarette (e-cigarette) use and its relationship with smoking and alcohol and illicit drug consumption among Romanian university students. Materials and methods: A cross sectional study using anonymous questionnaire was performed in 2017 among 400 university students from Cluj-Napoca, Romania. Results: 95.5% of the participants had heard about e-cigarettes and 43.7% of these had tried e-cigarettes during their lifetime, while 8.9% declared using cigarettes in the previous month (one out of five students who had tried them during their lifetime). Half of the students had smoked during their lifetime and one third had smoked in the previous month. Eighty-five percent of participants had experimented alcohol intoxication during their lifetime and 45% had done so in the previous month, while illicit drug use during their lifetime and the previous month was 34% and 9.5%, respectively. The results of the linear regression analyses show a positive correlation between e-cigarette use, smoking, experimentation with alcohol intoxication, and the use of illicit drugs. Conclusions: Future studies as well as educational activities should address the complex relationship between e-cigarette and other substance use among Romanian youth.
6711	Martinelli, Thomas, Candel, Math J. J. M., de Vries, Hein, Talhout, Reinskje, Knapen, Vera, van Schayck, Constant P., Nagelhout, Gera E. (2021) Exploring the gateway hypothesis of e-cigarettes and tobacco: a prospective replication study among adolescents in the Netherlands and Flanders. Tobacco control;<br><br>**Keywords:**<br>electronic nicotine delivery devices harm reduction public policy MEDLINE controlled study nicotine cigarette smoking replication study follow up high school male never smoker Flanders human prospective study article adolescent female smoking risk factor major clinical study Netherlands convenience sample electronic cigarette<br><br>**Abstract:** BACKGROUND: Studies demonstrated that adolescent e-cigarette use is associated with subsequent tobacco smoking, commonly referred to as the gateway effect. However, most studies only investigated gateways from e-cigarettes to tobacco smoking. This study replicates a cornerstone study

	revealing a positive association between both adolescent e-cigarette use and subsequent tobacco use; and tobacco and subsequent e-cigarette use in the Netherlands and Flanders. DESIGN: The longitudinal design included baseline (n=2839) and 6-month (n=1276) and 12-month (n=1025) follow-up surveys among a school-based cohort (mean age: 13.62). Ten high schools were recruited as a convenience sample. The analyses involved (1) associations of baseline e-cigarette use and subsequent tobacco smoking among never smokers; (2) associations of e-cigarette use frequency at baseline and tobacco smoking frequency at follow-up; and (3) the association of baseline tobacco smoking and subsequent e-cigarette use among non-users of e-cigarettes. FINDINGS: Consistent with prior findings, baseline e-cigarette use was associated with higher odds of tobacco smoking at 6-month (OR=1.89; 95% CI 1.05 to 3.37) and 12-month (OR=5.63; 95% CI 3.04 to 10.42) follow-ups. More frequent use of e-cigarettes at baseline was associated with more frequent smoking at follow-ups. Baseline tobacco smoking was associated with subsequent e-cigarette use (OR=3.10; 95% CI 1.58 to 6.06 at both follow-ups). CONCLUSION: Our study replicated the positive relation between e-cigarette use and tobacco smoking in both directions for adolescents. This may mean that the gateway works in two directions, that e-cigarette and tobacco use share common risk factors, or that both mechanisms apply.
6740	McCausland, Kahlia, Jancey, Jonine, Leaver, Tama, Freeman, Becky, Wolf, Katharina, Maycock, Bruce (2021) A qualitative exploration of Western Australian vapers process of e-cigarette initiation, continuance and cessation. Tobacco Induced Diseases; 19<br><br>**Keywords:**<br>E-cigarette Health promotion Qualitative Vaping Embase controlled study social media Western Australia smoking cessation genetic transcription adult conference abstract human data analysis software young adult interview relapse telephone Internet<br><br>**Abstract:** Introduction: E-cigarettes have become increasingly popular over the last 10 years. These devices represent a new paradigm for tobacco control offering smokers an opportunity to inhale nicotine without inhaling tobacco smoke. To date there are no definitive conclusions regarding the safety and long-term health effects of these devices however the increased uptake and popularity of them suggests that they may be perceived as a healthier alternative to traditional cigarettes. Objectives: To describe the factors influencing e-cigarette initiation, maintenance and cessation, and determine opportunities for health promotion intervention. Methods: Participants were recruited through online e-cigarette forums, closed social media groups, and snowball sampling to participate in face-to-face and telephone (only when required) interviews. To be eligible to participate interviewees were required to be current or past (within the last 12 months) e-cigarette users, aged 18 years or older, and reside within the Perth Metropolitan or Peel Region of Western Australia. Interviews were audio recorded and transcribed verbatim before being imported into NVivo software for analysis. Results: influence of other vapers, and the internet/social media. Factors supporting maintenance: improved health, quit smoking, decreased cost, vaping community (online, other vapers and vape stores), enjoyment, hobby, and starting a vaping related business. Factors influencing cessation: relapse to cigarettes, and if new research determined vaping was bad for health and/or worse than tobacco. Conclusion(s): The only reason participants said they would cease using e-cigarettes is if research determined vaping was detrimental to their health and/or worse than tobacco. As data analysis continues this study aims to determine where then are our opportunities for health promotion intervention?
6775	Melka, Alemu, Chojenta, Catherine, Holliday, Elizabeth, Loxton, Deborah (2021) E-cigarette use and cigarette smoking initiation among Australian women who have never smoked. Drug and alcohol review; 40<br><br>**Keywords:**<br>Australia cigarette smoking e-cigarette initiation young adult women Index Medicus Young Adult Australia -- epidemiology Humans Adult Tobacco Longitudinal Studies Vaping Electronic Nicotine Delivery Systems

	**Abstract:** INTRODUCTION AND AIMEvidence regarding the association between e-cigarette use and subsequent initiation of smoking mostly relates to the US population. In Australia, no studies are available investigating the association between the uses of e-cigarettes and smoking initiation among young adults who have never smoked. This study aimed to determine the association between lifetime e-cigarette use and subsequent initiation of cigarette smoking among tobacco-naïve Australian women aged 20-27.DESIGN AND METHODSThe current study used data (n = 5398) from the third (2015) and fourth (2016) surveys collected from a cohort of Australian women born in 1989-1995 who participated in the Australian Longitudinal Study on Women's Health. Multivariable logistic regression was used to identify the association between lifetime e-cigarette use at the baseline survey and initiation of cigarette smoking (smoked 100 cigarettes or more in the last year) at the follow up adjusting for possible confounders. Effects were expressed as odds ratios with 95% confidence interval.RESULTSThe mean (± SD) age of the study participants at baseline (third survey) was 22.5 (±1.7). Ever e-cigarette use at baseline was positively associated with smoking initiation at follow up (adjusted odds ratio 3.71; 95% confidence interval 2.33, 5.93). History of depression, binge drinking and higher childhood adversity score were also risk factors for subsequent smoking initiation in the follow up.DISCUSSION AND CONCLUSIONSThis study identified a strong association between e-cigarette use and subsequent initiation of smoking. Enforcing the existing restriction of sale and supply of e-liquid containing nicotine is essential to prevent never smokers from nicotine addiction via e-cigarettes.
6882	Nguyen, Nhung, Kapiteni, Kwinoja, Straus, Elana R., Guydish, Joseph (2021) Factors associated with dual and polytobacco use among people in residential substance use disorder treatment. American Journal on Addictions; 30<br><br>**Keywords:**<br>Embase MEDLINE demography controlled study California independent variable article female tobacco dependence prevalence male adult human electronic cigarette<br><br>**Abstract:** Background and Objectives: Little is known about whether people in substance use disorder (SUD) treatment are at risk for multiple-tobacco use. We examined the factors associated with dual- and polytobacco use among clients in SUD treatment. Methods: A cross-sectional survey was conducted in 2019 among 562 clients (Mage = 39, 74% male) in 20 residential SUD treatment programs in California, USA. The outcomes included single-, dual- (use of two products), and polytobacco use (use of three of more products). Independent variables were nicotine dependence, quitting-related factors, blunt/spliff use, and health-related factors. A multinomial model examined associations between the independent variables and the outcome, controlling for demographics, and time in treatment. Results: Overall, 32.6%, 18.9%, and 14.0% of the sample were single-, dual-, and polytobacco users, respectively. Factors associated with increased odds of polytobacco use included greater nicotine dependence (adjusted odds ratio [AOR] = 1.60; 95% CI = 1.19, 2.16), ever using e-cigarettes for quitting (AOR = 4.56; 95% CI = 2.23, 9.34), and past 30-day use of blunt/spliff (AOR = 2.96; 95% CI = 1.48, 5.89). Factors associated with increased odds of dual use were ever using e-cigarettes for quitting (AOR = 3.19; 95% CI = 1.79, 5.66) and reporting more mentally unhealthy days (AOR = 1.05; 95% CI = 1.02, 1.07). Conclusion and Scientific Significance: This study extends the literature on tobacco use among people in SUD treatment by revealing the high prevalence of dual- and polytobacco use and the unique characteristics of users. The findings have implications for interventions reducing all types of tobacco use in this understudied population.
6885	Nguyen Zarndt, Anh, Donaldson, Elisabeth A., Bernat, Jennifer K., Henrie, James A., Portnoy, David B. (2020) Adult use of and transitions from nicotine and non-nicotine-containing e-cigarettes: Data from the Population Assessment of Tobacco and Health (PATH) study, 2013–2016. Nicotine & Tobacco Research; 22<br><br>**Keywords:**

	nicotine e-cigarettes self-report demographic characteristics Empirical Study Longitudinal Study Quantitative Study Human Male Female Adulthood (18 yrs & older) Young Adulthood (18-29 yrs) Thirties (30-39 yrs) Middle Age (40-64 yrs) Aged (65 yrs & older) Electronic Cigarettes US article 2990:Drug & Alcohol Usage (Legal) United States Young Adult Vaping Electronic Nicotine Delivery Systems Tobacco Use Disorder Humans Aged Longitudinal Studies Tobacco Products Research Design Self Report Adult Middle Aged Adolescent Prevalence<br><br>**Abstract:** Introduction: Studies largely focus on nicotine-containing e-cigarettes (NiCE) though non-nicotine-containing e-cigarettes (NoCE) exist; NoCE prevalence and patterns of use are largely unknown. This study examines self-reported prevalence and patterns of NiCE/NoCE use. Methods: We analyzed adult (18 + years) data from the Population Assessment of Tobacco and Health (PATH) study from Wave 1 (2013–2014, n = 32,320), Wave 2 (2014–2015, n = 28,632), and Wave 3 (2015–2016, n = 28,148). We test associations between Wave 1 self-reported current NoCE/NiCE use status and demographic characteristics and assess the proportion of self-reported current NoCE/NiCE users at Wave 1 or Wave 2 who continue to use NoCE or NiCE, switch to NiCE or NoCE, discontinue e-cigarette use, switch to use other nicotine products (ONP), or add ONP use 1 year later (i.e., at Wave 2 or 3). Results: Maintaining the same self-reported NiCE/NoCE and ONP use status 1 year later was the most common use pattern between waves. However, 15.65% of exclusive NoCE users in Wave 2 transitioned to NoCE plus nicotine product use in Wave 3. Also, some exclusive NoCE users transitioned to exclusive NiCE use (17.77% Waves 1–2; 11.55% Waves 2–3). Discussion: Some exclusive NoCE users transitioned to NiCE or added nicotine product use, suggesting there may be other factors (e.g., familiarity with using an aerosolizing device) in addition to the presence of nicotine in influencing initiation or sustained use of nicotine products. Implications: Studies largely focus on nicotine-containing e-cigarettes (NiCE) though non-nicotine-containing e-cigarettes (NoCE) exist; this study adds to the literature by describing demographic characteristics and tobacco use of adult self-reported NoCE users. In addition, the study examines transitions in self-reported NoCE/NiCE use, revealing that some exclusive NoCE users transition to other nicotine product use 1 year later. (PsycInfo Database Record (c) 2021 APA, all rights reserved) (Source: journal abstract)
6929	Ortega, Adrian, Sutton, Moneika, McConville, Andrew, Cushing, Christopher C., Fite, Paula J. (2021) Longitudinal investigation of the bidirectional associations between initiation of e-cigarettes and other substances in adolescents. Journal of Substance Use; 26<br><br>**Keywords:**<br>adolescent E-cigarettes gateway hypothesis substance initiation tobacco alcohol 64-17-5 cannabis 8001-45-4 8063-14-7 Embase 17:Public Health, Social Medicine and Epidemiology 7:Pediatrics and Pediatric Surgery high school student cigarette smoking vaporizer substance use vaping male tobacco snuff underage drinking priority journal human Article longitudinal study female cannabis smoking alcohol consumption risk assessment adolescent smoking chewing tobacco juvenile electronic cigarette<br><br>**Abstract:** Background: Although research on adolescent e-cigarette use is growing, several questions remain unanswered, including the timing of initiation and the co-occurrence with initiation of other substances. The current study longitudinally evaluated the risk for initiation of e-cigarettes and examined associations with this risk and initiation of other substances (alcohol, traditional tobacco products, and marijuana). Methods: 141 youth from a single high school were in 9th and 10th grade at the initial data collection. Data were collected annually for the following two years. Rates of initiation were calculated. Cross-lagged panel models estimated how prior initiation of e-cigarette use affected the risk for initiation of other substances and vice-versa. Results: Risk for initiation of e-cigarettes was comparable across the two years. Youth were most at risk for having used alcohol at baseline. As youth aged through high school, they were most at risk for initiation of e-cigarettes relative to other substances. There is preliminary evidence that endorsement of alcohol use at baseline increased the risk for initiation of e-cigarettes one year later. Conclusions: Rates of e-cigarette initiations increased throughout the two-year

	period. These preliminary findings support the notion that efforts to prevent initiation of e-cigarettes should target adolescents who have used alcohol.
6935	Owotomo, Olusegun, Maslowsky, Julie (2021) Adolescent E-cigarette Users at Highest Risk of Cigarette Smoking Intention. American journal of health behavior; 45<br><br>**Keywords:**<br>MEDLINE cigarette smoking male clinical article smoke human latent class analysis article adolescent female case report peer pressure adolescent smoking friend electronic cigarette<br><br>**Abstract:** Objectives: Adolescent e-cigarette users are at risk of developing smoking intention, an established predictor of conventional cigarette smoking. In this study, we identify subgroups of adolescent e-cigarette users who are most likely to intend to smoke conventional cigarettes. Methods: Cross-sectional data on 1357 8th and 10th grade e-cigarette users who had never smoked conventional cigarettes were obtained from 2014-2017 Monitoring the Future Surveys. We conducted latent class analysis to identify subgroups of adolescent e-cigarette users; through latent class regression analysis, we examined the association between subgroup membership and smoking intention. Results: We identified 3 subgroups of adolescent e-cigarette users: socially-protected (56.6%), peer-driven (29.8%), and market-vulnerable (13.6%). The peer-driven class reported the highest number of peers who smoke and the lowest proportion of friends who strongly disapproved of daily cigarette smoking. They were significantly more likely than the socially-protected and market-vulnerable classes to have smoking intention (AOR=2.46; 95% CI 1.84-3.28, and AOR=2.29; 95% CI 1.48-3.53, respectively). Conclusion: Our findings provide insights on the constellation of risk and protective factors that contribute to smoking intention among adolescent e-cigarette users. It highlights peer influence as an important area of emphasis for adolescent smoking prevention programs.
6936	Owotomo, Olusegun, Stritzel, Haley, McCabe, Sean Esteban, Boyd, Carol J., Maslowsky, Julie (2020) Smoking intention and progression from e-cigarette use to cigarette smoking. Pediatrics; 146<br><br>**Keywords:**<br>smoking intention e-cigarette use cigarette smoking Empirical Study Longitudinal Study Prospective Study Quantitative Study Human Male Female Childhood (birth-12 yrs) School Age (6-12 yrs) Adolescence (13-17 yrs) Electronic Cigarettes Tobacco Smoking Intention US article 2990:Drug & Alcohol Usage (Legal)<br><br>**Abstract:** Objectives: To investigate whether the prospective association between electronic cigarette (e-cigarette) use and cigarette smoking is dependent on smoking intention status. Methods: Waves 2 and 3 data of the Population Assessment of Tobacco and Health (PATH) Study, a US nationally representative prospective cohort study of tobacco use. Data were collected in 2014-2015 (wave 2) and 2015-2016 (wave 3) and analyzed in 2019. Results: At wave 2, 12.8% of adolescent never-smokers of conventional cigarettes had intention to smoke and 8.5% had ever used an e-cigarette. At wave 3, 3.2% had ever smoked a cigarette. Both smoking intention and ever using e-cigarettes at wave 2 were positively associated with cigarette smoking at wave 3 (adjusted odds ratio [aOR] = 3.03; 95% confidence interval [CI] = 1.97-4.68, P < .001; aOR = 4.62, 95% CI = 2.87-7.42, P < .001, respectively). The interaction between smoking intention and ever using e-cigarettes was significant (aOR = 0.34, 95% CI = 0.18-0.64, P < .01). Among adolescents who had expressed intention to smoke conventional cigarettes at wave 2, the odds of cigarette smoking at wave 3 did not significantly differ for e-cigarette users and never e-cigarette users (aOR = 1.57; 95% CI 0.94-2.63; P = .08). Among adolescents who had no intention to smoke at wave 2, e-cigarette users, compared with never e-cigarette users, had > 4 times the odds of cigarette smoking (aOR = 4.62; 95% CI 2.87-7.42; P < .0001). Conclusions: E-cigarette use is associated with increased odds of cigarette smoking among adolescents who had no previous smoking intention. E-cigarette use may create intention to smoke and/or nicotine use disorder that lead to onset of cigarette smoking. (PsycInfo Database Record (c) 2021 APA, all rights reserved) (Source: journal abstract)

6964	Patel, Akash, Hirschtick, Jana L., Cook, Steven, Usidame, Bukola, Mistry, Ritesh, Levy, David T., Meza, Rafael, Fleischer, Nancy L. (2021) Sociodemographic patterns of exclusive and dual use of ENDS and menthol/non-menthol cigarettes among US youth (Ages 15–17) using two nationally representative surveys (2013–2017). International Journal of Environmental Research and Public Health; 18<br><br>**Keywords:**<br>Adolescents Cigarettes Electronic nicotine delivery systems (ENDS) Epidemiology Population Tobacco Youth menthol 1490-04-6 89-78-1 15356-70-4 2216-51-5 98167-53-4 Embase MEDLINE 17:Public Health, Social Medicine and Epidemiology United States social status cigarette smoke demography cigarette smoking household income smoking cessation prevalence male groups by age sex ratio human Article cross-sectional study Caucasian longitudinal study adolescent female cohort analysis parenting education ethnicity home environment electronic cigarette<br><br>**Abstract:** The use of electronic nicotine delivery systems (ENDS) among youth in the United States has increased rapidly in the past decade. Simultaneously, while youth cigarette smoking has declined considerably, youth are still more likely to use menthol cigarettes than any other age group. We used nationally representative data on 15–17-year-olds from the Population Assessment of Tobacco and Health (PATH) Study and the National Youth Tobacco Survey (NYTS) (2013–2017) to better understand current cigarette (by menthol flavoring) and ENDS use in the US. We calculated weighted population prevalence estimates across years for multiple patterns of current cigarette and ENDS use (i.e., exclusive menthol cigarette, exclusive non-menthol cigarette, exclusive ENDS, dual ENDS and menthol cigarette, and dual ENDS and non-menthol cigarette) by sex, race/ethnicity, parental education level, household income, and homeownership. Overall, both exclusive menthol and non-menthol cigarette use declined from 2013–2017. Exclusive ENDS use increased, particularly among youth who were non-Hispanic White or had a higher socioeconomic status (measured by parental education, household income, and homeownership). Dual use of ENDS with either menthol or non-menthol cigarettes did not change significantly. Monitoring changes in these sociodemographic patterns will help inform future youth tobacco prevention strategies.
6982	Pearson, Jennifer L., Sharma, Eva, Rui, Ning, Halenar, Michael J., Johnson, Amanda L., Cummings, K. Michael, Hammad, Hoda T., Kaufman, Annette R., Tworek, Cindy, Goniewicz, Maciej L., Kimmel, Heather L., Tanski, Susanne, Compton, Wilson M., Day, Hannah, Ambrose, Bridget K., Bansal-Travers, Maansi, Silveira, Marushka L., Abrams, David, Limpert, Jeannie, Travers, Mark J., Borek, Nicolette, Hyland, Andrew J., Stanton, Cassandra A. (2020) Association of Electronic Nicotine Delivery System Use With Cigarette Smoking Progression or Reduction Among Young Adults. JAMA network open; 3<br><br>**Keywords:**<br>Index Medicus Socioeconomic Factors Young Adult Electronic Nicotine Delivery Systems Risk Factors Humans Cohort Studies Adult Longitudinal Studies United States -- epidemiology Male Female Prevalence Cigarette Smoking -- epidemiology Smoking Cessation -- psychology Vaping -- epidemiology Vaping -- psychology Smoking Cessation -- statistics & numerical data Cigarette Smoking -- psychology<br><br>**Abstract:** ImportanceThe prevalence of electronic nicotine delivery systems (ENDS) use, including e-cigarettes, among US young adults (YAs) has raised questions about how these products may affect future tobacco and nicotine use among YAs. Given this prevalence and that young adulthood is a critical period for the establishment of tobacco and nicotine use, it is important to consider the association between ENDS use and cigarette smoking specifically in this age group.ObjectiveTo examine whether ENDS use frequency or intensity is associated with changes in cigarette smoking among US YA ever smokers during 1 year.Design, Setting, and ParticipantsThis cohort study used 3 waves of data (2013-2014, 2014-2015, and 2015-2016) from the Population Assessment of Tobacco and Health (PATH) Study, an ongoing longitudinal cohort study of adults and youth. Unweighted 1:6 propensity score matching was used to match participants on wave 1 risk factors for ENDS use at wave 2. The changes in smoking

	between wave 2 and wave 3 were assessed using the matched sample. In total, 1096 ENDS-naive, ever cigarette-smoking YAs (18-24 years of age) at wave 1 who participated in wave 2 and wave 3 and who had complete data in the PATH Study were included in the analyses, which were conducted from August 2018 to October 2019.ExposuresNever ENDS use (n = 987), any previous 30-day ENDS use (n = 109), 1 to 5 days of ENDS use in the previous 30 days (n = 75), and 6 or more days ENDS use in the previous 30 days at wave 2 (n = 34).Main Outcomes and MeasuresThe analytic sample was selected using multiple variables based on peer-reviewed literature supporting associations with ENDS use. The main outcomes-changes in cigarette smoking behavior between wave 2 and wave 3-were defined using 2 measures: (1) change in smoking frequency, defined as the number of smoking days in the previous 30 days at wave 3 vs wave 2, and (2) change in smoking intensity, defined as the number smoking days in the previous 30 days multiplied by the mean number of cigarettes consumed on smoking days at wave 3 vs wave 2.ResultsThe present cohort analyses included 1096 YA ever smokers who were ENDS naive at wave 1. The majority of the sample were women (609 [55.6%]) and White individuals (698 [63.7%]), and the mean (SD) age was 21.4 (1.9) years. In wave 1, 161 YAs (14.7%) were daily smokers in the previous 30 days. After propensity score matching, no statistically significant associations were observed between any definition of wave 2 ENDS use and changes in either the frequency or intensity of smoking at wave 3.Conclusions and RelevanceIn this cohort study of US YA ever smokers, ENDS use was not associated with either decreased or increased cigarette smoking during a 1-year period. However, it is possible that the rapidly evolving marketplace of vaping products may lead to different trajectories of YA cigarette and ENDS use in the future.
6992	Pérez, Adriana, Kuk, Arnold E., Bluestein, Meagan A., Penedo, Elena, N'Hpang, Roi San, Chen, Baojiang, Perry, Cheryl L., Sterling, Kymberly L., Harrell, Melissa B. (2021) Prospective estimation of the age of initiation of cigarettes among young adults (18–24 years old): Findings from the Population Assessment of Tobacco and Health (PATH) waves 1–4 (2013–2017). PLoS ONE; 16<br><br>**Keywords:**<br>cigarettes tobacco-related health outcomes ethnicity Empirical Study Quantitative Study Human Male Female Adulthood (18 yrs & older) Young Adulthood (18-29 yrs) Latinos/Latinas Electronic Cigarettes Prevention Emerging Adulthood Tobacco Smoking Intervention Estimation Ethnic Identity Age Differences US article 3360:Health Psychology & Medicine<br><br>**Abstract:** Objectives: To prospectively estimate the age of cigarette initiation among young adults (18-24 years old) who were never cigarette users at their first wave of adult study participation overall, by sex, and by race/ethnicity given recent increases in cigarette initiation occurring in young adulthood. Methods: Secondary analyses were conducted using the PATH restricted-use adult datasets among young adult never users of cigarettes in waves 1-3 (2013-2016) with outcomes followed-up in waves 2-4 (2014-2017). Interval censoring survival methods were used to estimate the age of initiation of (i) ever, (ii) past 30-day, and (iii) fairly regular cigarette use. Among never cigarette users when they first entered the adult study, interval censoring Cox proportional hazard models were used to explore differences in the estimated age of initiation of the three cigarette use outcomes by sex and by race/ethnicity, controlling for the effect of previous e-cigarette use and the total number of other tobacco products ever used (0-5 products) before cigarette initiation outcomes. Results: Among the young adults who were never cigarette users at their first wave of adult participation, the highest increase in cigarette initiation occurred between 18 and 19 years old. By age 21, 10.6% (95% CI: 9.5-11.7) initiated ever cigarette use, 7.7% (95% CI: 6.1-8.1) initiated past 30-day of cigarette use, and 1.9% (95% CI: 1.4-2.5) initiated fairly regular cigarette use. After controlling for other tobacco products: (a) males were 83% more likely to initiate past 30-day cigarette use at earlier ages than females; (b) Hispanic and Non-Hispanic Black young adults had increased risk to initiate past 30-day cigarette use at earlier ages than Non-Hispanic White young adults (62% and 34%, respectively). Conclusions: The substantial amount of cigarette initiation among young adults reinforces the need for prevention strategies among this population. Although, interventions are needed for all young adult populations, strategies should target 18-21-year-olds, with

	potentially differential prevention targets by sex and by race/ethnicity. (PsycInfo Database Record (c) 2021 APA, all rights reserved) (Source: journal abstract)
6993	Pérez, Adriana, Penedo, Elena, Bluestein, Meagan A., Chen, Baojiang, Perry, Cheryl L., Harrell, Melissa B. (2020) The Recalled Age of Initiation of Multiple Tobacco Products among 26-34 Year Olds: Findings from the Population Assessment of Tobacco and Health (PATH) Study Wave 1 (2013-2014). International journal of environmental research and public health; 17<br><br>**Keywords:**<br>cigarettes cigarillos e-cigarettes filtered cigars hazard ratio histogram hookah nationally representative smokeless tobacco traditional cigars Index Medicus Electronic Nicotine Delivery Systems Age of Onset Humans Adult United States -- epidemiology Male Female Tobacco, Smokeless Tobacco Use -- epidemiology Tobacco Products -- statistics & numerical data<br><br>**Abstract:** This study examined the recalled age of initiation of seven different tobacco products (TPs) and explored potential influences of sex, race/ethnicity, and cigarette-smoking status on tobacco use initiation among adults 26-34 years old using the PATH study.METHODSSecondary analyses were conducted in the adult restricted PATH wave 1 (2013-2014) dataset. Weighted statistics are reported using the balanced repeated replication method and Fay's correction to account for PATH's complex study design. Distributions and histograms of the recalled age of initiation of seven different TPs (cigarettes, cigarillos, traditional cigars, filtered cigars, hookah, smokeless tobacco, and e-cigarettes) are reported, as well as the impact of sex and race/ethnicity using Cox proportional hazard models. The impact of cigarette-smoking status on the recalled age of initiation of each tobacco product other than cigarettes was explored.RESULTSThe highest modes of the recalled age of initiation of cigarette use were at 14-15 and 15-16 years old. The distributions of the recalled age of initiation of cigarillos, traditional cigars, filtered cigars, hookah, and smokeless tobacco occurred later, with the highest modes at 15-16 and 17-18 years old. The distribution of the recalled age of initiation of e-cigarettes had a different shape than the other TPs, with the highest mode reported at 27-28 years old.CONCLUSIONDue to the ever-changing tobacco marketplace, understanding when contemporary adults aged 26-34 years recall initiating TP use is important and will inform prevention researchers.
7015	Pierce, John P., Chen, Ruifeng, Leas, Eric C., White, Martha M., Kealey, Sheila, Stone, Matthew D., Benmarhnia, Tarik, Trinidad, Dennis R., Strong, David R., Messer, Karen (2021) Use of E-cigarettes and Other Tobacco Products and Progression to Daily Cigarette Smoking. Pediatrics; 147<br><br>**Keywords:**<br>Abridged Index Medicus Index Medicus Young Adult Smoking -- trends Humans Child Longitudinal Studies Adolescent United States -- epidemiology Smoking -- epidemiology Male Female Population Surveillance Cigarette Smoking -- epidemiology Cigarette Smoking -- trends Electronic Nicotine Delivery Systems Vaping -- epidemiology Vaping -- trends Tobacco Products<br><br>**Abstract:** OBJECTIVESTo identify predictors of becoming a daily cigarette smoker over the course of 4 years.METHODSWe identified 12- to 24-year-olds at wave 1 of the US Population Assessment of Tobacco and Health Study and determined ever use, age at first use, and daily use through wave 4 for 12 tobacco products.RESULTSSixty-two percent of 12- to 24-year-olds (95% confidence interval [CI]: 60.1% to 63.2%) tried tobacco, and 30.2% (95% CI: 28.7% to 31.6%) tried ≥5 tobacco products by wave 4. At wave 4, 12% were daily tobacco users, of whom 70% were daily cigarette smokers (95% CI: 67.4% to 73.0%); daily cigarette smoking was 20.8% in 25- to 28-year-olds (95% CI: 18.9% to 22.9%), whereas daily electronic cigarette (e-cigarette) vaping was 3.3% (95% CI: 2.4% to 4.4%). Compared with single product triers, the risk of progressing to daily cigarette smoking was 15 percentage points higher (adjusted risk difference [aRD] 15%; 95% CI: 12% to 18%) among those who tried ≥5 products. In particular, e-cigarette use increased the risk of later daily cigarette smoking by threefold (3% vs 10%; aRD 7%; 95% CI: 6% to 9%). Daily smoking was 6 percentage points lower (aRD -6%; 95% CI: -8% to -4%) for those who experimented

	after age 18 years.CONCLUSIONSTrying e-cigarettes and multiple other tobacco products before age 18 years is strongly associated with later daily cigarette smoking. The recent large increase in e-cigarette use will likely reverse the decline in cigarette smoking among US young adults.
7037	Popova, Lucy, Henderson, Katherine, Kute, Nikita, Singh-Looney, Manu, Ashley, David L., Reynolds, Reed M., Nayak, Pratibha, Spears, Claire Adams (2021) "I'm bored and I'm stressed": A qualitative study of exclusive smokers, ENDS users, and transitioning smokers or ENDS users in the time of COVID-19. Nicotine & tobacco research : official journal of the Society for Research on Nicotine and Tobacco;<br><br>**Keywords:**<br>MEDLINE controlled study nicotine physiological stress vaping male boredom qualitative research pandemic adult combustion qualitative analysis human mindfulness article female coronavirus disease 2019 major clinical study<br><br>**Abstract:** BACKGROUND: To better understand the various influences of COVID-19 on tobacco use, we examined three different tobacco user groups using qualitative methods. METHODS: Ten online focus groups with 61 adults from the Atlanta, GA area were held in October-November 2020: four with exclusive smokers (n=16), three with Electronic Nicotine Delivery System (ENDS) users (dual and exclusive, n=22), and three with transitioning (recently quit or currently quitting) smokers and/or ENDS users (n=23). RESULTS: Exclusive smokers reported smoking more frequently, driven by COVID-19-related stress, time at home, and boredom. They were not motivated to quit during the pandemic, and some considered smoking to be protective against COVID-19. ENDS users reported vaping less, with dual users often increasing their smoking; many were concerned about health effects of smoking and ENDS use during the pandemic. Transitioning smokers/ENDS users worried about their health and wanted to quit, but many found the stress of COVID-19 unbearable without tobacco use. CONCLUSIONS: There were some similarities among the groups, but also pronounced differences. Educational campaigns should capitalize on the teachable moment of COVID-19 to increase perceived risk of smoking. Smokers need access to more adaptive ways to deal with stress (such as mindfulness training) in lieu of smoking and systems-level approaches should address structural determinants of health that cause high levels of stress. The proposed policy to lower nicotine in combusted tobacco products might help smokers choose other means of coping instead of cigarettes by reducing the stress-relieving properties of smoking particularly salient during the pandemic. IMPLICATIONS: Smokers believe that cigarettes help them deal with the stresses and challenges of the COVID-19 pandemic. This needs to be counteracted by educational campaigns to increase perceived harm of smoking, alternative stress-relief strategies, and mandated changes to the combusted tobacco products to make them less appealing.
7042	Prakash, Shivaani, Hatcher, Cameron, Shiffman, Saul (2021) Prevalence of ENDS and JUUL Use, by Smoking Status, in National Samples of Young Adults and Older Adults in the U.S. American journal of health behavior; 45<br><br>**Keywords:**<br>Index Medicus Young Adult Cross-Sectional Studies Humans Adult Adolescent Prevalence Electronic Nicotine Delivery Systems Vaping -- epidemiology Smoking -- epidemiology<br><br>**Abstract:** Objectives: In this paper, we estimate the prevalence of electronic nicotine delivery systems (ENDS) and JUUL brand ENDS use among adults in the US, overall and by smoking history. Methods: We obtained 2019 cross-sectional online surveys assessing smoking, use of any ENDS, and JUUL specifically, in a national probability sample of 11,833 US adults. Data were analyzed for young adults (YA; aged 18-24) and older adults (OA; aged 25+). Results: Past 30-day ENDS prevalence was 8.0% in YA and 4.7% in OA; equivalent JUUL figures were 3.1% and 1.2%. ENDS/JUUL use was more prevalent among current and former smokers than never smokers, where prevalence was ≤ 2% (YA: 2.0%/0.9%; OA: 0.9%/0.1%). JUUL use was higher among recent (< 1 year) quitters than among long-term (≥ 1 year) quitters. Among those who had ever used both ENDS and other tobacco, strong majorities reported using other tobacco first.

	Among JUUL users who also had used other tobacco ≥ 95% had used other tobacco first. Conclusions: Past-30-day ENDS use (including JUUL) was ≤ 8% among young adults and ≤ 5% in older adults. Most (> 98%) ENDS and JUUL users were current or former smokers, which is relevant to assessment of the population impact of these products.
7043	Prakash, Shivaani, Xu, Yingying, Goldenson, Nicholas I., Wissmann, Rasmus, Gougelet, Robyn, Shiffman, Saul (2021) Transitions in Smoking among Adults Newly Purchasing the JUUL System. American journal of health behavior; 45<br><br>**Keywords:**<br>Index Medicus United States Smokers Humans Adult Longitudinal Studies Electronic Nicotine Delivery Systems Vaping -- epidemiology Smoking Cessation Smoking -- epidemiology<br><br>**Abstract:** Objectives: In this study, we prospectively assessed changes in smoking a year after US adults' first-time purchase of a JUUL Starter Kit (JSK). Methods: Descriptive analyses assessed transitions in smoking status at 12 months among adult (age ≥21) JSK purchasers providing baseline and 12-month follow-up data (N = 27,164 [49.0% of baseline]), stratified by baseline smoking status. Baseline regular use of other ENDS was also considered. Results: Purchasers included baseline past 30-day smokers (65.2%), former smokers (12.0%), and never smokers (9.7%); over 90% were ever-smokers. One year later, the majority (58%) of those smoking at baseline reported no longer smoking. Former smokers or never smokers who were smoking at 12 months represented less than 2% of respondents. Former and current smokers who were regularly using other ENDS at baseline were less likely to report smoking at 12 months. Conclusions: Past 30-day smoking prevalence in a large longitudinal study of first-time JSK purchasers fell by more than half over 12 months. Analyzing the sample composition at purchase and transitions within subgroups defined by smoking status allows for a detailed understanding to help inform assessments of the population health impact of ENDS.
7071	Rahman, Azizur, Mohamed, Mohamad Haniki Nik, Mahmood, Syed (2021) Evaluating nicotine abstinence, smoking cessation, reduction and its relapsed among electronic cigarettes single and dual malaysian users: A one year observational study. Journal of Pharmacy and Pharmaceutical Sciences; 24<br><br>**Keywords:**<br>carbon monoxide 630-08-0 Embase MEDLINE 17:Public Health, Social Medicine and Epidemiology coughing breathing disorder smoking cessation male Malaysian withdrawal syndrome adult observational study Article human prospective study young adult relapse female cohort analysis self report craving electronic cigarette<br><br>**Abstract:** -Purpose: Evidence for the complete nicotine cessation is inadequate among electronic cigarettes (ECs) single users (SUs, use only ECs), and dual users (DUs, use both ECs and conventional cigarettes (CCs). The primary aim of this study was to evaluate the nicotine cessation among SUs and DUs who used ECs over one year. Methods: We observed 70 SUs and 148 DUs for 52 weeks and tested their exhaled carbon monoxide and saliva cotinine to confirm their complete nicotine cessation status through cotinine in saliva. Safety issues were to be identified through self-report. Smoking cessation, CCs reduction of ≥ 50%, and relapsed to CCs smoking and safety issues were also documented. Results: The nicotine cessation rate was higher in SUs then DUs (15.9% vs. 6.8%; P = 0.048; 95% CI (2.328-0.902). A similar result for smoking cessation (34.8% SUs vs. 17.1% DUs; P = 0.005; 95% CI: 2.031-0.787), whereas CCs ≥ 50% reduction was 23.3% DUs vs 21.7% SUs (P = 0.863; 95% CI:1.020-0.964). Relapse to CC smoking was 47.3% in DUs versus 30.4% in SUs (P = 0.026; 95% CI: 1.555-0.757). The adverse effects reported were coughing and breathing problems, whereas craving smoking was documented as a major withdrawal symptom. Smoking-related diseases were also identified, five in DUs and two in SUs, during the one-year study period. Conclusions: Study showed SUs achieved higher complete nicotine and smoking cessation rates as compared to DUs. However, the rates of reduced CC use were not different between both the groups. No serious adverse effects related to the sole use of ECs were detected.

	However, the safety of the sole use of ECs in absolute terms needs to be further validated in different populations.
7111	Reynolds, Lindsay M., Zamora, Cristian, Lee, Un Jung, Stokes, Andrew C., Benjamin, Emelia J., Bhatnagar, Aruni, Payne, Thomas J., Rodriguez, Carlos J. (2021) Tobacco use prevalence and transitions from 2013 to 2018 among adults with a history of cardiovascular disease. Journal of the American Heart Association; 10<br><br>**Keywords:**<br>Cardiovascular disease Race and ethnicity Smoking Embase MEDLINE United States heart infarction controlled study cigarette smoking educational status smoking cessation prevalence male heart failure adult education race human cerebrovascular accident cigar human tissue article female self report major clinical study ethnicity cardiovascular effect electronic cigarette<br><br>**Abstract:** BACKGROUND: Although tobacco product use and transitions have been characterized in the general population, few studies have focused on individuals with established cardiovascular disease (CVD) in a population-based sample. METHODS AND RESULTS: We examined tobacco use prevalence and longitudinal patterns of tobacco product transitions in adults (≥18 years) of the nationally representative PATH (Population Assessment of Tobacco and Health) study, from 2013 to 2014 (Wave 1) through 2016 to 2018 (Wave 4). Prevalent CVD was classified through self-report of having had a heart attack, heart failure, stroke, or other heart condition. Factors associated with tobacco product use and transitions were investigated using survey logistic regression. We examined 2615 participants with self-reported CVD at Wave 1. Overall, 28.9% reported current tobacco use, equating to ≈6.2 million adults in the United States with prevalent CVD and current tobacco use. Among adults with CVD who are current tobacco users, the most commonly used product was cigarettes (82.8%), followed by any type of cigar (23.7%), and e-cigarette use (23.3%). E-cigarette use without concurrent cigarette use among participants with prevalent CVD was uncommon (1.1%). Factors associated with tobacco use were younger age, male sex, had lower education level, and lack of knowledge about the association between smoking and CVD. Men with prevalent CVD were less likely to use e-cigarettes compared with women (odds ratio [OR], 0.7; 95% CI, 0.5–0.9). Among cigarette users with CVD, transition rates between Waves 1 and 4 demonstrated <5% decrease in cigarette, with a 0.5% increase in e-cigarette use. Only ≈10% were in formal tobacco cessation programs. CONCLUSIONS: Despite known harmful cardiovascular effects, over one fourth of adults with prevalent CVD use tobacco products and few quit smoking over the 4 waves of the PATH data set.
7225	Selya, Arielle S., Foxon, Floe (2021) Trends in electronic cigarette use and conventional smoking: Quantifying a possible 'diversion' effect among US adolescents. Addiction; 116<br><br>**Keywords:**<br>Adolescents cigarettes diversion electronic cigarettes nicotine use simulation modeling Empirical Study Quantitative Study Human Childhood (birth-12 yrs) School Age (6-12 yrs) Adolescence (13-17 yrs) Measurement Computer Simulation Nicotine Tobacco Smoking Surveys Simulation Trends US article 2840:Psychosocial & Personality Development<br><br>**Abstract:** Background and aims: The impact of electronic cigarettes (ECs) on nicotine use is hotly debated: some fear that ECs are a 'catalyst' to conventional smoking, while others argue that they divert adolescents from the more harmful product. This study used simulation modeling to evaluate the plausibility of catalyst and diversion hypotheses against real-world data. Design: A simulation model represented life-time exclusive EC use, exclusive conventional smoking and dual use as separate subpopulations. The 'catalyst' effect was modeled as EC use increasing dual use initiation (i.e. EC users also start smoking). The 'diversion' effect was modeled as EC use decreasing exclusive cigarette initiation. The model was calibrated using data from the US National Youth Tobacco Survey (NYTS). The plausibility of each scenario was evaluated by comparing simulated trends with NYTS data. This is the first study, to

	our knowledge, to estimate the magnitude of a diversion effect through simulation. Setting: United States. Participants and measurements: Adolescents aged 12–17 years in NYTS, a cross-sectional study from 2000 to 2019 (n = 12 500 to 31 000 per wave). Exclusive cigarette use, exclusive EC use and dual use of both products were defined using cumulative life-time criteria (100+ cigarettes smoked and/or > 100 days vaped). Findings: A null model (no catalyst or diversion) over-predicts NYTS smoking by up to 87%. Under the conservative assumption that the catalyst effect accounts for all dual use, an exponential decay constant of 19.6% EC users/year initiating smoking is required; however, this further over-predicts actual smoking by up to 109%. A diversion effect with an exponential decay constant of 55.4%/year or 65.4%/year, with the maximum possible opposing catalyst effect also active, is required optimally to match NYTS smoking trends (root mean square error = 286 632 versus 391 396 in the null model). Conclusions: A simulation model shows that a substantial diversion effect is needed to explain observed nicotine use trends among US adolescents, and it must be larger than any possible opposing catalyst effect, if present. (PsycInfo Database Record (c) 2021 APA, all rights reserved) (Source: journal abstract)
7237	Shahab, Lion, Beard, Emma, Brown, Jamie (2021) Association of initial e-cigarette and other tobacco product use with subsequent cigarette smoking in adolescents: a cross-sectional, matched control study. Tobacco control; 30<br><br>**Keywords:**<br>electronic nicotine delivery devices harm reduction non-cigarette tobacco products priority/special populations MEDLINE cigarette smoking nicotine adolescent tobacco human electronic cigarette cross-sectional study<br><br>**Abstract:** INTRODUCTION: This study assessed whether initiating e-cigarette use increases the uptake of cigarette smoking in US adolescents compared with behavioural and synthetic controls. METHODS: Data come from 78 265 adolescents in the National Youth Tobacco Survey (2014-2017) of whom 38 630 provided information about the first tobacco product they had used in 2014/15. Ever, past 30 day and established (30 day use and 100+ lifetime cigarettes) cigarette smoking was compared in adolescents who first used an e-cigarette (exposure group), a non-cigarette combustible (CT) or other non-combustible tobacco (NT) product (behavioural controls), and propensity score matched adolescents without initial e-cigarette use (synthetic controls). RESULTS: Relative to behavioural controls, adolescents who tried e-cigarettes first were less likely to have ever smoked cigarettes (26% vs CT (42.4%; OR 0.48, 95% CI 0.40 to 0.57), or NT initiators (52.7%; OR 0.32, 95% CI 0.26 to 0.39)), to be past 30 day (6% vs CT (11.9%; OR 0.48, 95% CI 0.36 to 0.62), or NT initiators (20.0%; OR 0.26, 95% CI 0.19 to 0.35)) or be established cigarette smokers (0.7% vs CT (3.9%; OR 0.17, 95% CI 0.10 to 0.30), or NT initiators (8.4%; OR 0.08, 95% CI 0.04 to 0.13)). E-cigarette initiators were also less likely than synthetic controls (without initial e-cigarette use) to have ever smoked cigarettes (OR 0.76, 95% CI 0.62 to 0.93), be past 30 day (OR 0.71, 95% CI 0.55 to 0.91) or be established cigarette smokers (OR 0.26, 95% CI 0.13 to 0.51). CONCLUSION: Less than 1% of US adolescents who use e-cigarettes first were established cigarette smokers. They were less likely to be smokers than adolescents who tried other combustible or non-combustible tobacco products first and propensity score matched adolescents without initial e-cigarette use.
7239	Shan, Lingpeng, Azagba, Sunday (2021) Longitudinal associations of tobacco-related social media involvement with cigarette and e-cigarette initiation among US adolescents. European Journal of Pediatrics;<br><br>**Keywords:**<br>Adolescents Cigarette E-cigarette Initiation Social media Embase MEDLINE controlled study cigarette smoking marketing male race human article adolescent female tobacco academic achievement major clinical study ethnicity electronic cigarette<br><br>**Abstract:** Tobacco is the leading cause of preventable morbidity and mortality in the USA. Evidence

	suggests adolescents are particularly vulnerable to online tobacco marketing. This study examined longitudinal associations of following or liking of tobacco brands with subsequent cigarette and e-cigarette initiation among US adolescents. We used Wave 1–Wave 4 Population Assessment of Tobacco and Health study data (n = 6997) and discrete-time survival regression models to examine associations of past-year tobacco-related social media interactions with the initiation of cigarettes and e-cigarettes among US adolescents. About 4.8% (n = 280) of adolescent never cigarette users and 4.9% (n = 288) of never e-cigarette users followed or liked tobacco brands on social media between Wave 1 and Wave 2. By Wave 4, 8.8% of all cigarettes never users had initiated cigarette use, and 18.7% of never e-cigarette users initiated e-cigarette use. The following or liking tobacco brands on social media was significantly associated with increased odds of cigarette initiation (adjusted odds ratio (aOR) 2.12, 95% CI 1.56–2.88) and e-cigarette initiation (aOR 2.11, 95% CI 1.66–2.69). Also, the initiation of cigarettes and e-cigarettes differed significantly among race/ethnicity, school performance, and other tobacco and substance use. Conclusion: Adolescents who followed or liked tobacco products on social media were more likely to initiate cigarette or e-cigarette use subsequently. Increasing anti-tobacco efforts on social media sites could be beneficial.What is Known:• Evidence suggests adolescents are particularly vulnerable to online tobacco marketing.What is New:• The following or liking tobacco brands on social media was significantly associated with the odds of cigarette and e-cigarette initiation.
7264	Shiffman, Saul, Holt, Nathan M. (2021) Smoking Trajectories of Adult Never Smokers 12 Months after First Purchase of a JUUL Starter Kit. American journal of health behavior; 45<br><br>**Keywords:**<br>Index Medicus Smokers Non-Smokers Humans Adult Electronic Nicotine Delivery Systems Vaping -- epidemiology Smoking -- epidemiology<br><br>**Abstract:** Objectives: We assessed adult never smokers' trajectories of smoking over 12 months after a first-time JUUL Starter Kit (JSK) purchase. Methods: Adult (≥ 21) never smokers (N = 3853) who purchased a JSK were recruited into an observational naturalistic study. Analyses distinguished those who had previously used ENDS (NS+E, N = 2848) from those who had not (NS-NE, N = 1005). Participants were invited to complete follow-up assessments at 1, 2, 3, 6, 9, and 12 months. "Smoking" was defined as any past-30-day smoking ("even a puff"). Results: Past-30-day smoking was reported by 5.01% of NS+E at month one, and 7.56% at month 12; for NS-NE, these were 10.23% and 12.35%. In both groups, < 5% reported smoking at both 9 and 12 months. Across follow-ups, 25%-49% of those reporting having smoked then said they were now smoking "not at all"; the remainder reported low frequency (10-12 days-per-month) and quantity (2-4 cigarettes-per-day) of smoking. Past-30-day use of JUUL remained at ≥ 80% across follow-ups. Each additional day-per-month of JUUL use decreased the odds of smoking by 1%. Conclusions: Some adult never smokers who purchased a JSK reported smoking during the suceeding year; smoking was light and intermittent. Participants who used JUUL more frequently were less likely to smoke.
7285	Singh, Tavleen, Cobb, Nathan, Cohen, Trevor, Myneni, Sahiti (2021) Transitions Between Electronic and Combustible Cigarettes: A Mixed Methods Analysis of Peer Interactions in an Online Community for Tobacco Cessation. Studies in health technology and informatics; 281<br><br>**Keywords:**<br>behavior change e-cigarettes health beliefs social media Health Technology Assessment Humans Peer Group Electronics Electronic Nicotine Delivery Systems Tobacco Use Cessation Tobacco Products<br><br>**Abstract:** The growing popularity of e-cigarettes is a public health concern. There is an emerging need to understand the pathways between electronic and combustible modes due to the specialized nature of risks associated with each transition. Online social media has become the most dominant knowledge space for these evolving behaviors, and as such, can provide unique opportunities for modeling switching patterns. In this paper, we describe the utility of online peer interactions using qualitative inquiry and

	network visualizations using 500 messages to characterize (a) transition pathways and (b) psychosocial attributes as individuals contemplate and act on such transitions. Our results indicate that the E2A pathway is the most prevalent in e-cigarette-related transitions, where most of the individuals are in the "active e-cig use" stage. Perceived benefits and barriers are the most commonly held health beliefs, while counterconditioning and stimulus control behavior change processes are frequently manifested. Such insights can help in the design of personalized pathway-specific behavior change interventions.
7318	Sokol, Natasha A., Feldman, Justin M. (2021) High School Seniors Who Used E-Cigarettes May Have Otherwise Been Cigarette Smokers: Evidence From Monitoring the Future (United States, 2009-2018). Nicotine & tobacco research : official journal of the Society for Research on Nicotine and Tobacco; 23<br><br>**Keywords:**<br>Index Medicus Cross-Sectional Studies Schools Smokers Humans Adolescent United States -- epidemiology Vaping Electronic Nicotine Delivery Systems Tobacco Products<br><br>**Abstract:** INTRODUCTIONStudies have indicated that youth who use e-cigarettes are more likely to progress to cigarette smoking; however, the likelihood that these youth would have used tobacco products in the pre-vaping era is unclear.AIMS AND METHODSThis study sought to determine whether youth who used e-cigarettes in 2014-2018 would have likely been smokers in the period preceding e-cigarette availability. Analyzing Monitoring the Future 12th grade data (United States, 2009-2018), we forecasted the prevalence of current smoking with logistic regression-derived propensity scores. Models predicted smoking for all subsequent years, incorporating sociodemographic, family, alcohol, and school-related variables, and a linear time trend. We compared forecasted to observed smoking prevalence annually, and prevalence of current e-cigarette use among nonsmokers across smoking propensity tertiles.RESULTSUntil 2014, observed smoking prevalence mirrored forecasted prevalence. Afterward, forecasted rates consistently overestimated prevalence. Among nonsmoking youth, e-cigarette use was lowest among those with lowest predicted probability of cigarette smoking (3.8%; 95% confidence interval [CI]: 3.3, 4.4) and highest among those with highest probability (23.5%; 95% CI: 22.2, 24.9).CONCLUSIONSYouth e-cigarette use has increased rapidly, with high prevalence among nonsmoking youth. However, the decline in current smoking among 12th graders has accelerated since e-cigarettes have become available. E-cigarette use is largely concentrated among youth who share characteristics with smokers of the pre-vaping era, suggesting e-cigarettes may have replaced cigarette smoking.IMPLICATIONSAmong nonsmoking youth, vaping is largely concentrated among those who would have likely smoked prior to the introduction of e-cigarettes, and the introduction of e-cigarettes has coincided with an acceleration in the decline in youth smoking rates. E-cigarettes may be an important tool for population-level harm reduction, even considering their impact on youth.
7341	Soule, Eric K., Plunk, Andrew D., Harrell, Paul T., Hayes, Rashelle B., Edwards, Kathryn C. (2020) Longitudinal analysis of associations between reasons for electronic cigarette use and change in smoking status among adults in the Population Assessment of Tobacco and Health Study. Nicotine & Tobacco Research; 22<br><br>**Keywords:**<br>electronic cigarette use smoking Empirical Study Longitudinal Study Interview Quantitative Study Human Male Female Adulthood (18 yrs & older) Young Adulthood (18-29 yrs) Thirties (30-39 yrs) Middle Age (40-64 yrs) Aged (65 yrs & older) Electronic Cigarettes Vaping Tobacco Smoking US article 2990:Drug & Alcohol Usage (Legal) United States Flavoring Agents Young Adult Electronic Nicotine Delivery Systems Humans Aged Longitudinal Studies Self Report Cigarette Smoking Smokers Smoking Cessation Adult Middle Aged Adolescent Prevalence<br><br>**Abstract:** Introduction: Electronic cigarette (ECIG) use and changes in cigarette smoking status may be influenced by self-reported reasons for using ECIGs. Methods: We analyzed adult current and former cigarette smokers who were also current or former ECIG users at wave 1 (n = 3044) using wave 1 and

	wave 2 Population Assessment of Tobacco and Health Study data (2013–2015). Prevalence of reporting 13 reasons for ECIG use at wave 1 was examined and weighted logistic regressions were conducted predicting smoking status changes from wave 1 to wave 2. Results: Reasons for ECIG use ranged from 18.1% (people in the media or public figures use them) to 82.5% (they might be less harmful to people around me than cigarettes). From wave 1 to wave 2, 27.2% of former smokers (n = 249) became current smokers and 11.6% of current smokers (n = 246) became former smokers. Among wave 1 former smokers, using ECIGs because of the availability of flavors (AOR = 0.57, 95% CI = 0.39–0.85) or because they don't smell (AOR = 0.64, 95% CI = 0.42–0.97) was associated with lower odds of relapse to smoking, but using ECIGs because using them helps people quit smoking (AOR = 1.55, 95% CI = 1.01–2.38) was associated with greater odds of relapse. Among wave 1 current smokers, using ECIGs because they can be used where smoking is not allowed (AOR = 0.56, 95% CI = 0.38–0.85) was associated with reduced odds of quitting cigarettes. Conclusions: Some reasons for ECIG use are associated with changes in self-reported smoking status. Researchers should examine ECIG user characteristics when assessing associations between ECIG use and smoking status transitions. Implications: Given that certain reasons for ECIG use, such as using ECIGs in locations are where smoking is not allowed, may inhibit smoking reduction, policies may be developed to prevent ECIG use in locations where smoking is banned. In addition, because certain reasons for ECIG use may aid in relapse prevention, such as availability of desired flavors, efforts should be made to identify ECIG device characteristics that are appealing to smokers but not youth or nontobacco users. These results provide support for future research on reasons for ECIG use to inform regulatory policies. (PsycInfo Database Record (c) 2021 APA, all rights reserved) (Source: journal abstract)
7352	Staff, Jeremy, Kelly, Brian C., Maggs, Jennifer L., Vuolo, Mike (2021) Adolescent Electronic Cigarette Use and Tobacco Smoking in the Millennium Cohort Study. Addiction (Abingdon, England);<br><br>**Keywords:**<br>catalyst Coarsened Exact Matching common liability E-cigarette use Millennium Cohort Study tobacco use youth smoking MEDLINE demography controlled study cigarette smoking infant child British citizen adolescent smoking risk factor juvenile confounding variable externalization (behavior) human infancy prospective study human tissue article female cohort analysis alcohol consumption pregnancy tobacco maternal smoking risk assessment major clinical study electronic cigarette<br><br>**Abstract:** AIMS: To evaluate the catalyst, diversion, and common liability hypotheses by examining associations between e-cigarette use and tobacco cigarette smoking at modal ages 14 and 17, controlling for adolescent and infancy risk factors. DESIGN: Intergenerational, prospective cohort data from the Millennium Cohort Study (MCS). Nationally representative sample of infants born September, 2000 through January, 2002. SETTING: United Kingdom. PARTICIPANTS: Parent and child data from 10,625 youth assessed in infancy and modal ages 11, 14, and 17 years. MEASUREMENTS: Age 14 and 17 e-cigarette and combustible cigarette use (recency, frequency). Potential confounders were age 11 risk factors (e.g., alcohol use, externalizing behaviors, parental tobacco use, permissiveness), infancy risk factors (e.g., maternal smoking during pregnancy, smoke exposure in infancy) and demographic characteristics. FINDINGS: Among youth who had not smoked tobacco by age 14 (n=9,046), logistic regressions estimated that teenagers who used e-cigarettes by age 14, compared with non e-cigarette users, had over 5 times higher odds of initiating tobacco smoking by age 17 (odds ratio (OR)=5.25, 95% confidence interval (CI):[3.28-8.38]), and nearly triple the odds of being a frequent tobacco smoker at age 17 (OR=2.91, 95% CI:[1.56-5.41]), net of risk factors and demographics. Among youth who had not used e-cigarettes by age 14 (n=9,078), teenagers who had smoked tobacco cigarettes by age 14 had 3 times higher odds of initiating e-cigarettes by age 17 (OR=2.98, 95% CI:[1.74-5.09]), compared with non-tobacco smokers, and nearly 3 times higher odds of frequently using e-cigarettes at age 17 (OR=2.90, 95% CI:[1.21-6.95]), net of confounders. Similar links between e-cigarette and tobacco cigarette use were observed in regressions following Coarsened Exact Matching (CEM). CONCLUSIONS: E-cigarette use by age 14 is associated with increased odds of tobacco cigarette initiation and frequent smoking at age 17

	among British youth. Similarly, tobacco smoking at age 14 is associated with increased odds of both e-cigarette initiation and frequent use at age 17.
7361	Stokes, Andrew C., Wilson, Anna E., Lundberg, Dielle J., Xie, Wubin, Berry, Kaitlyn M., Fetterman, Jessica L., Harlow, Alyssa F., Cozier, Yvette C., Barrington-Trimis, Jessica L., Sterling, Kymberle L., Benjamin, Emelia J., Blaha, Michael J., Hamburg, Naomi M., Bhatnagar, Aruni, Robertson, Rose Marie (2021) Racial/Ethnic Differences in Associations of Non-cigarette Tobacco Product Use with Subsequent Initiation of Cigarettes in US Youths. Nicotine and Tobacco Research; 23<br><br>**Keywords:**<br>Embase MEDLINE 17:Public Health, Social Medicine and Epidemiology United States controlled study exposure cigarette smoking follow up hookah smokeless tobacco race difference male school child population research human Article racial disparity child cigar ethnic difference tobacco use longitudinal study adolescent female cohort analysis electronic cigarette<br><br>**Abstract:** Introduction: Understanding which non-cigarette tobacco products precede smoking in youth across different racial/ethnic groups can inform policies that consider tobacco-related health disparities. Methods: We used nationally representative, longitudinal data from the Population Assessment of Tobacco and Health Study waves 1-4. The sample was a dynamic cohort of cigarette-naïve youth aged 12-17 years. Mixed-effects models were used to assess non-cigarette product (e-cigarette, cigar product, or other product) use with cigarette use over 1-year intervals. Results: Of the 28 788 observations pooled across waves 1-4, respondents were 48.7% non-Hispanic white, 13.9% non-Hispanic black, and 23.1% Hispanic. Odds of cigarette initiation over 1-year follow-up were higher among youth with prior use of e-cigarettes (odds ratio [OR], 2.76; 95% confidence interval [CI], 2.21-3.45), cigars (OR, 2.00; 95% CI, 1.42-2.80), or other products (OR, 1.66; 95% CI, 1.28-2.14) compared to never users. At the population level, 20.6% of cigarette initiation was attributable to e-cigarette use among white youth and 21.6% among Hispanic youth, while only 3.5% of cigarette initiation was attributable to e-cigarette use among black youth. In contrast, 9.1% of cigarette initiation for black youth was attributable to cigar use compared to only 3.9% for both white and Hispanic youth. Conclusions: Prior use of e-cigarettes, cigars, and other non-cigarette products were all associated with subsequent cigarette initiation. However, white and Hispanic youth were more likely to initiate cigarettes through e-cigarette use (vs. cigar or other product use), while black youth were more likely to initiate cigarettes through cigar use (vs. e-cigarette or other product use). Implications: Our findings suggest that previous studies on effects of non-cigarette tobacco products may overlook the critical role of cigar products as a pathway into cigarette smoking among US youth, particularly black youth. While our data support the importance of e-cigarette use as a pathway into smoking, regulatory actions aimed at addressing youth e-cigarette use alone may contribute to disparities in black versus white tobacco use and further exacerbate inequities in tobacco-related disease. Thus, contemporary policy development and discourse about the effects of non-cigarette tobacco products on cigarette initiation should consider cigar and other non-cigarette products as well as e-cigarettes.
7379	Sumbe, Aslesha, Clendennen, Stephanie L., Opara, Samuel C., Jackson, Christian D., Chen, Baojiang, Wilkinson, Anna V., Harrell, Melissa B. (2021) ENDS Device Type and Initiation of Combustible Tobacco Products among Adolescents. Nicotine and Tobacco Research; 23<br><br>**Keywords:**<br>Embase MEDLINE 17:Public Health, Social Medicine and Epidemiology social status cigarette controlled study demography hookah race difference male Texas population research human Article prospective study cigar ethnic difference longitudinal study female adolescent combustible tobacco product cohort analysis tobacco adolescent smoking electronic cigarette<br><br>**Abstract:** Introduction: The aim of this study was to determine whether Electronic Nicotine Delivery Systems (ENDS) device type (disposable devices, replaceable cartridges, and refillables) at initial or first ENDS use predicts subsequent initiation of combustible tobacco products (cigarettes, hookah, cigars)

	among adolescents and/or differentiates between those who initiate use of both ENDS and combustible tobacco products at the same time. Methods: The study examined data from the Texas Adolescent Tobacco and Marketing Surveillance System (TATAMS), a longitudinal population-based cohort of students in major metropolitan areas of Texas (n = 3907; N = 461 069). Data were collected every 6 months, from 2014 to 2018; 33.9% (n = 1324; N = 151 784) of the sample initiated ENDS use across this period. Unadjusted and adjusted logistic regression models were used to assess the odds of initiating combustible tobacco products at a subsequent or similar wave as ENDS initiation, given initial ENDS device type. Results: After adjusting for sociodemographic factors, the odds of initiating combustible tobacco use subsequent to ENDS initiation were significantly lower among those who reported using Cartridges as their initial device type compared to those who reported Refillables as their initial device type (adjusted odds ratio = 0.42 [0.18-0.98], p =. 05). In addition, after controlling for sociodemographic variables, the odds of initiating combustible tobacco use in the same wave as ENDS initiation were significantly higher among those who reported using Cartridges as their initial device type, compared with those who reported Refillables as their initial device type (adjusted odds ratio = 2.31 [1.05-5.10], p =. 04). No significant differences were found in adjusted models when Disposables were compared to Refillables and Cartridges to Disposables. Conclusion: ENDS device type differentiates between adolescents who start using combustible tobacco products at the same time as initial ENDS use, or subsequently. Implications: Previous research has shown ENDS use predicts subsequent combustible use among adolescents, but there is lack of research on the role of specific ENDS device types and the timing of initiation. Findings from this longitudinal study show that initiation of combustible tobacco product use varies by initial ENDS device type among adolescents. These findings can become a focal point for developing interventions for adolescents and could have regulatory implications for ENDS products.
7385	Sun, Tianze, Lim, Carmen C. W., Stjepanović, Daniel, Leung, Janni, Connor, Jason P., Gartner, Coral, Hall, Wayne D., Chan, Gary C. K. (2021) Has increased youth e-cigarette use in the USA, between 2014 and 2020, changed conventional smoking behaviors, future intentions to smoke and perceived smoking harms?. Addictive Behaviors; 123<br><br>**Keywords:**<br>Adolescents Cigarettes E-cigarettes Renormalization Tobacco Vaping Embase MEDLINE controlled study article prevalence perception probability major clinical study juvenile human<br><br>**Abstract:** Introduction: An increase in electronic cigarette (EC) use among US youth has raised concerns that their use may increase conventional cigarette smoking. Methods: Repeated cross-sectional data from two large nationally representative samples of US youth were drawn from the National Youth Tobacco Survey (NYTS) and Monitoring the Future (MTF). Students from grade 6–12 of the NYTS (N = 98, 454–132,003) and students from grade 12 of the MTF (N = 17,870–30,981) were included in the analyses. Trends in smoking behavior, intention to smoke in the future and perceived smoking harms among youth who do and do not use ECs were estimated from 2014 to 2020 using weighted multinomial logistic regression and predicted marginal probabilities. Results: The prevalence for regular smoking decreased among youth who regularly use ECs. For example, among regular EC users, smoking prevalence decreased from 27.8% to 6.7% (−21%; 99.67% CI:[−32.3%, −9.9%]) in the NYTS between 2014 and 2020 and from 31.8% to 10.6% (−21.2%, 99.67% CI:[−35.0%, −7.3%]) in the MTF between 2014 and 2018. Intent to smoke in the future and perceiving smoking as harmless decreased or remained unchanged during the same period. For example, among regular EC users, there was no significant change in intention to smoke next year in the NYTS between 2014 and 2018; intention to smoke in the next 5 years decreased significantly from 30.7% to 11.2% (−19.5%, 99.67% CI:[−37.7%, −1.3%]) in the MTF. Conclusions: In two national samples of US youth, smoking prevalence declined by a sizeable relative percentage. Intent to smoke in the future and harm perceptions of smoking declined or remained unchanged while EC use increased. Results provide little evidence that EC use has increased conventional cigarette smoking among youth.

7409	Tam, Jamie (2021) E-cigarette, combustible, and smokeless tobacco product use combinations among youth in the united states, 2014–2019. Addictive Behaviors; 112<br><br>**Keywords:**<br>Adolescents Cross-sectional survey E-cigarettes Electronic cigarettes Smoking Embase MEDLINE 17:Public Health, Social Medicine and Epidemiology United States high school student smokeless tobacco water pipe smoking vaping prevalence pipe smoking tobacco snuff bidi smoking Article human snus tobacco use middle school student chewing tobacco cigar smoking<br><br>**Abstract:** Introduction: Youth e-cigarette use has been rising, however U.S. prevalence data are generally reported without disaggregating by individuals' use of other tobacco products. It is not clear how the proportion of youth e-cigarette users naïve to all combustible tobacco is changing. Methods: Annual prevalence estimates of ever and current (defined as past 30-day use) tobacco use prevalence by school type are reported using the 2014–2019 National Youth Tobacco Surveys (NYTS) with mutually exclusive categories of e-cigarette, smokeless tobacco, and/or combustible tobacco product use. T-tests were used to compare annual estimates with the preceding year. The annual percent change (APC) for each category from 2014 to 2018 were analyzed using JoinPoint regression. Data for 2019 were reported separately due to the change in survey format from paper to electronic. Results: Current use of only e-cigarettes among HS students who never used combustible tobacco increased significantly from 2014 to 2018 (APC = +42.4%, 95% CI: 0.7, 101.3); by 2019, prevalence peaked at 9.2% (95% CI: 8.2, 10.2) among never combustible users and 8.3% (95% CI: 7.3, 9.3) among former combustible users. This coincided with significant declines in use of only combustible tobacco (APC = −14.5%, 95% CI: −18.3, −10.5). Conclusions: Use of only e-cigarettes among US youth with no history of combustible tobacco use has increased substantially over time, even as combustible tobacco use continues to decline. Of the 17.5% (95% CI: 15.7, 19.0) of HS students who currently used only e-cigarettes (but not other tobacco) in 2019, more than half have no history of combustible tobacco use.
7410	Tam, Jamie, Brouwer, Andrew F. (2021) Comparison of e-cigarette use prevalence and frequency by smoking status among youth in the United States, 2014-19. Addiction (Abingdon, England); 116<br><br>**Keywords:**<br>adolescent smoking Cigarette cross-sectional survey e-cigarettes electronic cigarettes smoking United States youth MEDLINE child questionnaire young adult adolescent vaping prevalence tobacco epidemiology adult human electronic cigarette cross-sectional study<br><br>**Abstract:** BACKGROUND AND AIMS: Reports of youth e-cigarette use often do not disaggregate by underlying smoking status. This study compared annual 2014-19 youth estimates of past 30-day e-cigarette use prevalence and frequency by smoking status in the United States. DESIGN: Nationally representative, cross-sectional, school-based survey [National Youth Tobacco Surveys (NYTS)]. General linear models accounting for complex survey design compared e-cigarette use prevalence by smoking status by year, overall and stratified by frequency, separately for high school (HS) and middle school (MS) students. The 2019 survey was analyzed separately because of its change in survey methodology. SETTING: MSs and HSs in the United States. PARTICIPANTS: A total of 116 704 students from 1268 schools, ages 9-19. MEASUREMENTS: Students self-reported (paper 2014-18, electronic 2019) ever and past 30-day (current) use of e-cigarettes and cigarettes, as well as frequent use (20-30 days of month). FINDINGS: From 2014 to 2018, current e-cigarette use prevalence increased among never, current and former smokers in HS, but only among never and current smokers in MS (each P-value < 0.001). E-cigarette use increases for current HS smokers were primarily among frequent e-cigarette users. In 2018, the absolute number of HS frequent users who were never or former smokers (420 000 combined) surpassed current smokers (370 000). In 2019, current e-cigarette use prevalence for never, former and current smokers was 17.5% [95% confidence interval (CI) = 16.0-19.0], 53.6% (95% CI = 45.2-61.9) and 85.8% (95% CI = 81.6-89.9) for HS students, respectively, and 6.8% (95% CI = 5.9-7.7), 40.8% (95%

	CI = 34.7-47.0) and 78.0% (95% CI = 71.9-84.2) for MS students. That year, the number of HS never (420 000) and former smokers (570 000) using e-cigarettes frequently eclipsed that of current smokers (390 000). CONCLUSIONS: E-cigarette use prevalence and frequency among youth vary by smoking status, with highest levels of use among current smokers. However frequent e-cigarette use among never smokers and former smokers has increased.
7483	Veliz, Philip, Eisman, Andria, McCabe, Sean Esteban, Evans-Polce, Rebecca, McCabe, Vita V., Boyd, Carol J. (2020) E-Cigarette Use, Polytobacco Use, and Longitudinal Changes in Tobacco and Substance Use Disorder Symptoms Among U.S. Adolescents. Journal of Adolescent Health; 66<br><br>**Keywords:**<br>E-cigarettes Substance use disorders Tobacco use Embase MEDLINE 40:Drug Dependence, Alcohol Abuse and Alcoholism 17:Public Health, Social Medicine and Epidemiology 7:Pediatrics and Pediatric Surgery United States nicotine follow up household income drug misuse vaping male priority journal human Article child adolescent female tobacco dependence alcohol consumption cannabis use major clinical study adolescent smoking ethnicity electronic cigarette<br><br>**Abstract:** Purpose: The objective of this study was to examine the combinations of e-cigarette use, cigarette use, and other tobacco use over time and the relationship these longitudinal use patterns have with symptoms of tobacco use disorder (TUD) and substance use disorders (SUDs) among a sample of adolescents. Methods: Data from U.S. adolescents (aged 12–17 years) who were surveyed for the Population Assessment of Tobacco and Health Study at baseline, first follow-up, and second follow-up (2013–2014, 2014–2015, and 2015–2016; n = 7,595) was used to analyze symptoms of TUD and SUDs based on longitudinal combinations of tobacco/nicotine use. Results: The most common combination of tobacco/nicotine use across the three waves was “no use of any tobacco/nicotine products” at baseline and first follow-up to “e-cigarette use only” at the second follow-up. Multivariable analyses found that past 30-day cigarette use and other tobacco use at the most recent follow-up was associated with an increase in both current TUD and SUD symptoms, whereas past 30-day e-cigarette use at the most recent follow-up was modestly associated with an increase in current SUD symptoms. Conclusions: Individuals who transitioned to e-cigarette use were at relatively low risk for increased TUD and SUD symptoms. However, individuals who transitioned or continually used cigarettes were typically at greater risk for indicating more TUD and SUD symptoms. Given the low risk of e-cigarette only users to indicate TUD and SUD symptoms, prevention efforts need to be made to target these youth before they transition to cigarettes and other types of tobacco use.
7534	Wamamili, Ben, Coope, Pat, Grace, Randolph C. (2021) Cigarette smoking and e-cigarette use among university students in New Zealand before and after nicotine-containing e-cigarettes became widely available: results from repeat cross-sectional surveys. The New Zealand medical journal; 134<br><br>**Keywords:**<br>Index Medicus Young Adult Humans Students Cross-Sectional Studies Logistic Models Adult Surveys and Questionnaires Tobacco Adolescent Universities New Zealand -- epidemiology Female Male Cigarette Smoking -- epidemiology Electronic Nicotine Delivery Systems Vaping -- epidemiology Ethnic Groups -- statistics & numerical data<br><br>**Abstract:** AIMTo estimate prevalence and patterns of smoking and vaping, and associations between smoking and vaping, among university students in New Zealand when access to nicotine-containing e-cigarettes was restricted (ie, time point 1 or T1) and 12-months after restrictions were relaxed (ie, time point 2 or T2).METHODCross-sectional surveys of university students across all eight universities: T1, March 2018 (n=1,932), and T2, March 2019 (n=2,004). Chi-squared tests compared responses between T1 and T2 and logistic regression examined associations between smoking and vaping with student characteristics.RESULTSThe patterns of smoking (T1 vs T2): current (10.6% vs 12.1%, p=0.716), daily (5.0% vs 4.6%, p=0.121), and cigarettes/day, time to first cigarette, and avoidance of smoking in smoke-free

	spaces were not significantly different. In contrast, vaping: current (6.8% vs 13.5%, p<0.001), daily (2.7% vs 5.4%, p<0.001), and possibly vaping in smoke-free spaces, were significantly higher at T2. At both periods, males had higher odds of smoking, vaping and dual use; students aged 25-34 and long-term residents had higher odds of vaping. Asian and Other students had lower odds of smoking at T1, and Other students had higher odds of vaping at T2.CONCLUSIONVaping was significantly more prevalent at T2 than T1, without their being a corresponding decrease in smoking. Age, sex, student type and ethnicity predicted smoking and vaping.
7539	Wang, Gang, Wu, Liyun (2020) Healthy People 2020: Social Determinants of Cigarette Smoking and Electronic Cigarette Smoking among Youth in the United States 2010-2018. International journal of environmental research and public health; 17<br><br>**Keywords:**<br>Healthy People 2020 National Health Interview Survey (NHIS) cigarette smoking electronic cigarette smoking social determinant of health youth Index Medicus Young Adult Humans Adult Tobacco Adolescent United States -- epidemiology Smoking -- epidemiology Male Female Prevalence Vaping Electronic Nicotine Delivery Systems Healthy People Programs Social Determinants of Health<br><br>**Abstract:** The purpose of this study was to determine social determinants of cigarette smoking and ever using electronic cigarettes (e-cigarettes) among young adults aged 18 to 25 years in the United States between 2010 and 2018. Using secondary data from National Health Interview Surveys (NHIS) across the 2010, 2014, and 2018 survey years, this study analyzed the prevalence rates of cigarette smoking and ever using e-cigarettes between 2010 and 2018, demographic and socioeconomic disparities in smoking, and the relationship between previous e-cigarette use and current smoking. First, the past decade witnessed a notable decline in conventional cigarette smoking and a sharp increase in e-cigarette use among youth. These trends were consistent regardless of socioeconomic status. Second, demographic and socioeconomic disparities persisted in cigarette smoking. Non-Hispanic white male youth were more likely to become smokers as they grew older. Young people with lower educational attainment, living below the U.S. federal poverty level, and having a poor physical health status had a higher smoking prevalence. Third, previous e-cigarette use was more likely to relate to subsequent cigarette use among young people. To achieve the Healthy People 2020 objectives, tobacco control programs and interventions need to be more specific in higher prevalence groups and service providers should not assume that there is a one-size-fits-all model for youth.
7677	Abadi, Melissa H., Shamblen, Stephen R., Thompson, Kirsten, Lipperman-Kreda, Sharon, Grube, Joel, Richard, Bonnie O., Aramburu, Camila (2022) Socio-temporal contextual and community factors associated with daily exclusive ENDS use and dual use with tobacco cigarettes among adolescent vapers: an ecological momentary assessment study. BMC public health; 22<br><br>**Keywords:**<br>Adolescent Humans *Electronic Nicotine Delivery Systems Ecological Momentary Assessment Tobacco<br><br>**Abstract:** BACKGROUND: Adolescents who dual use ENDS with tobacco cigarettes are more likely to have an increased risk of developing dependence. Yet, little is understood about the factors driving dual use among adolescents. The current study sought to reveal the day-to-day socio-temporal contextual and community factors associated with adolescents' use of electronic nicotine delivery systems (ENDS), and how these factors predict dual use with tobacco cigarettes., METHODS: We collected ecological momentary assessments (EMA) from a sample of 50 adolescent past two-week vapers (ages 14-17 years old) over 14 days. Daily EMA data were collected on ENDS and tobacco cigarette use, as well as a range of contextual (i.e., motivations to vape, location of vaping, who with when vaping) and community factors (i.e., exposure to peers vaping, to adults vaping, to ENDS advertising, to ENDS warning messages). Our primary analyses were multilevel regressions, accounting for daily observations nested within individuals (N = 700 observations)., RESULTS: Participants used ENDS exclusively on 44% of days and dual used ENDS

	and tobacco cigarettes on 8% of the days. Dual use days (versus exclusive ENDS use days) were associated with "vaping because tobacco use was prohibited" (OR = 34.65, $p < .05$). Also, dual use days (versus no use days) were associated with greater exposure to adults vaping (OR = 5.59, $p < .05$), peers vaping (OR = 7.48, $p < .05$), and (c) ENDS advertisements or promotions (OR = 2.12, $p < .01$), whereas exclusive use days (versus no use days) were only associated with greater exposure to peers vaping (OR = 2.58, $p < .01$)., CONCLUSIONS: Results showed that exposure to peers and adults vaping and exposure to ENDS marketing were associated with same day dual use behaviors. And, that adolescents who dual used were motivated to use ENDS because they were easy to conceal. Findings support stricter regulation of ENDS marketing and for smoke-free air laws that include ENDS. In addition, these findings support prioritizing family- and school-based prevention programming that effectively communicates risk associated with ENDS use, including heightened risk of dual use and dependence. Such efforts can reduce the number of adolescents who use ENDS as well as the number who transition to tobacco cigarette use. Copyright © 2022. The Author(s).
7678	Abbasi, Yasir, Hout, Marie-Claire Van, Faragalla, Mohamed, Itani, Lynn (2022) Knowledge and Use of Electronic Cigarettes in Young Adults in the United Arab Emirates, Particularly during the COVID-19 Pandemic. International journal of environmental research and public health; 19<br><br>**Keywords:**<br>Adolescent COVID-19/ep [Epidemiology] *COVID-19 Cross-Sectional Studies *Electronic Nicotine Delivery Systems Humans Nicotine Pandemics United Arab Emirates/ep [Epidemiology] Vaping/ep [Epidemiology] *Vaping Young Adult<br><br>**Abstract:** (1) Background: The popularity of electronic cigarettes (e-cigarettes) has recently increased. Although they are less harmful than regular cigarettes, they still cause health consequences and their use for smoking cessation is inconclusive. The objective of this study was to evaluate patterns of use, knowledge about, and attitude towards e-cigarettes among youth in the United Arab Emirates (UAE) while also researching the impact of the COVID-19 pandemic on smoking behavior. (2) Methods: An online cross-sectional survey was distributed across three major universities in the UAE (n = 240) between March and November 2021. Descriptive analysis, comparison across gender and nationality groups, and correlates between 30-day e-cigarette use and self-reported increases in nicotine consumption during the pandemic were studied. (3) Results: About 37% of students had used an e-cigarette in their lifetime, and 23% had smoked e-cigarettes in the past month. During the pandemic, 52% of university students self-reported no change in nicotine consumption, while only 17.5% had reported an increase. The current smoking of regular cigarettes, waterpipe, and medwakh increased the odds of having an increase in smoking during the pandemic by 5.3 times. (4) Conclusions: The findings inform about youth behavior and knowledge about vaping in the UAE and could also support the development of awareness interventions.
7681	Abi Nehme, Anna Maria, Lou, XiangYang, Yan, Xinyu, Lee, Ji-Hyun, Salloum, Ramzi G. (2022) Transition to smoking cessation among dual cigarette and e-cigarette users in the population assessment of tobacco and health study, Waves 3 and 4 (2015-2017). Addictive behaviors; 129<br><br>**Keywords:**<br>Adolescent Adult *Electronic Nicotine Delivery Systems Humans Middle Aged Smokers *Smoking Cessation Tobacco *Tobacco Products Young Adult<br><br>**Abstract:** INTRODUCTION: This study evaluated the patterns of smoking cessation at Wave 4 of the Population Assessment of Tobacco and Health (PATH) Study among established cigarette smokers who were also current e-cigarettes users at Wave 3., METHODS: We assessed changes in smoking frequency at Wave 4 among established cigarette and e-cigarette users at Wave 3. The demographics of participants were analyzed as well as e-cigarettes device types, number of cigarettes smoked and time from waking up until the first cigarette. Unadjusted and adjusted odds ratios as well as percentage of

	discontinuing cigarettes use at Wave 4 were calculated for the different categories of dual users., RESULTS: Among dual users at Wave 3, 13.1% quit cigarette smoking at Wave 4, whereas half discontinued e-cigarette use and maintained exclusive cigarette smoking. Significant differences in the percentage of smoking cessation were observed across demographic groups - dual users with a bachelor's degree were more likely to quit smoking than dual users who did not finish high school (aOR = 3.06, 95% CI: 1.17-8.00) and smokers aged 25-64 years were less likely to quit smoking than those aged 18-24 years (aOR = 0.49, 95% CI: 0.28-0.86 for smokers aged 25-44 years, and aOR = 0.42, 95% CI: 0.20-0.87 for those aged 45-64). Dual users who smoked more than 15 cigarettes per day were significantly less likely to stop smoking (aOR = 0.38, 95% CI: 0.17-0.88)., CONCLUSION: E-cigarettes have the potential to aid in smoking cessation. Questions remain as to whether dual use of e-cigarettes and cigarettes should be encouraged as a transitional phase on the path to smoking cessation. Copyright © 2022 Elsevier Ltd. All rights reserved.
7692	Adeosun, Sherri (2023) Accessibility to vaping products, vaping, and use of cigarettes among adolescents. Dissertation Abstracts International: Section B: The Sciences and Engineering; 84<br><br>**Keywords:**<br>*Prevention *Tobacco Smoking *Electronic Cigarettes *Adolescent Behavior *Vaping<br><br>**Abstract:** There has been a continuous increase in the use of e-cigarettes among adolescents. The growth threatens to nullify the years of successfully reducing cigarette smoking among this age demographic. Additionally, the increase has been a concern for public healthcare workers due to the limited research on the effects of long-term use of e-cigarettes on adolescents. I investigated the possible side effects of exposure to e-cigarette advertisements and the use of e-cigarettes. Additionally, the study reviewed the relationship between the age e-cigarette use was initiated and the subsequent use of cigarettes. The conceptual framework was based on the multi-theory model and Bandura's cognitive theory. Data were taken from the 2020 National Youth Tobacco Survey. There were 24,544 students who participated in the survey across 254 schools from around the country. The secondary analysis of the Centers for Disease Control and Prevention National Youth Tobacco Survey dataset was done using binomial and ordinal regression. Ordinal regression analysis revealed an association between the age of e-cigarette initiation and the use of cigarettes, while the analysis revealed no association between the extent of access to vaping products and the frequency of use of e-cigarettes. Additionally, the application of binary logistic regression to the predictor variable (extent of exposure to e-cigarettes ads) and the outcome variable (use of e-cigarettes) showed a significant association between these variables. The findings contribute to the understanding of the accessibility of adolescents to e-cigarettes and how it influences the use of other cigarettes. (PsycInfo Database Record (c) 2023 APA, all rights reserved)
7698	Adzrago, David, Shi, Yue, Fujimoto, Kayo (2022) Association between perceived health risks of e-cigarettes and actual e-cigarette use, based on cigarette smoking status and sexual and gender minority status among U.S. adults. Zeitschrift fur Gesundheitswissenschaften = Journal of public health;<br><br>**Keywords:**<br><br>**Abstract:** BACKGROUND: There is a lack of consensus in the literature about the association between the perceived health risks of e-cigarettes and their actual use, an association that may be based on cigarette smoking status or sexual and gender minority status. This study examined the moderating effect of these variables as well as differences in e-cigarette use between and within perceived harmfulness of e-cigarettes and cigarette smoking among U.S. adults., METHODS: The data were drawn from the 2020 and 2019 Health Information National Trends Survey (HINTS 5), Cycles 4 and 3, on adults (N = 9303). We estimated weighted multivariable logistic regression models and conducted marginal analyses based on perceived harmfulness of e-cigarettes., RESULTS: Of the study population, 30.75% currently used e-cigarettes daily or some days. No significant difference in e-cigarette use was found between sexual

	minorities and heterosexuals, but there was a significant interaction between sexual identity and perceived harmfulness of e-cigarettes. Compared to non-smokers, current daily or some days cigarette smokers were more likely to use e-cigarettes (AOR = 3.48, 95% CI = 1.25, 9.72). Those who perceived e-cigarettes to be just as harmful as actual cigarettes (AOR = 0.13, 95% CI = 0.06, 0.27) or more harmful (AOR = 0.03, 95% CI = 0.01, 0.10), or were uncertain (AOR = 0.18, 95% CI = 0.08, 0.45), were less likely to use e-cigarettes compared to those who perceived e-cigarettes to be less harmful. Former and current cigarette smokers who perceived e-cigarettes as less harmful had a higher probability of using e-cigarettes daily or some days than did non-cigarette smokers. For the current and former cigarette smoking groups, those who perceived e-cigarettes as less harmful had the highest probability of current e-cigarette use than did those who were uncertain or perceived e-cigarettes as just as harmful, whereas the probability was not significant for those who perceived e-cigarettes as more harmful., CONCLUSION: Current daily or some days use of e-cigarettes is associated with current daily or some days cigarette smoking and lowered perceived harmfulness of e-cigarettes among the U.S. adult population. Tailored health promotion and intervention efforts may reduce the potential perceived health and behavioral/lifestyle risks related to the use of tobacco products, especially the use of e-cigarettes daily among former and current cigarette smokers as well as those who perceive e-cigarettes as less harmful. Copyright © This is a U.S. government work and not under copyright protection in the U.S.; foreign copyright protection may apply 2021.
7704	Ahmad, Safa, Wang, Tianru, Schwartz, Robert, Bondy, Susan J. (2022) Predictors of pod-type e-cigarette device use among Canadian youth and young adults. Predicteurs de l'utilisation de la cigarette electronique a capsule chez les jeunes et les jeunes adultes canadiens.; 42<br><br>**Keywords:**<br>Adolescent Canada/ep [Epidemiology] Child *Electronic Nicotine Delivery Systems Humans Male Surveys and Questionnaires *Tobacco Products *Vaping Young Adult<br><br>**Abstract:** INTRODUCTION: Changes to federal legislation allowed nicotine-based e-cigarettes legal entry into the Canadian market in 2018. This included pod-type e-cigarettes (pods), such as JUUL, that were later found to be associated with steeply increasing prevalence and greater frequency of e-cigarette use among US and Canadian youth. Multiple studies of risk factors of JUUL use and use initiation have been conducted among various population groups in the US, but little evidence exists pointing to similar risk factors of pod use among Canadian youth and young adults. Understanding these risk factors can inform use prevention and intervention strategies in Canadian and other jurisdictions., METHODS: A total of 668 Canadian youth and young adults recruited by the 2018-19 Youth and Young Adult Panel Study were provided a baseline survey 3 months before and a follow-up survey 9 months after the relaxation of federal nicotine e-cigarette regulations. We used multivariable logistic regression to understand and rank importance of baseline predictors of future pod use among respondents., RESULTS: Past-month cannabis use (OR [odds ratio] = 2.66, 95% CI: 1.66-4.21, p < 0.001), established cigarette use (OR = 3.42, 1.53-7.65, p < 0.01), past cigarette experimentation (OR = 2.40, 1.34-4.31, p < 0.01), having many friends who vaped (OR = 2.15, 1.37-3.34, p < 0.001), age below 18 compared to age over 22 (OR = 5.26, 2.63-10.00, p < 0.001) and male sex (OR = 1.69, 1.16-2.50, p < 0.01) were significant and the most influential predictors of future pod use., CONCLUSION: Similar factors drove pod use among Canadian and US youth and young adults. Appropriate preventive strategies can benefit from considering polysubstance use among high school-aged youth.
7709	Ait Ouakrim, Driss, Wilson, Tim, Waa, Andrew, Maddox, Raglan, Andrabi, Hassan, Mishra, Shiva Raj, Summers, Jennifer A., Gartner, Coral E., Lovett, Raymond, Edwards, Richard, Wilson, Nick, Blakely, Tony (2023) Tobacco endgame intervention impacts on health gains and Maori:non-Maori health inequity: a simulation study of the Aotearoa/New Zealand Tobacco Action Plan. Tobacco control;<br><br>**Keywords:**

	**Abstract:** BACKGROUND: The Aotearoa/New Zealand Government is aiming to end the tobacco epidemic and markedly reduce Maori:non-Maori health inequalities by legislating: (1) denicotinisation of retail tobacco, (2) 95% reduction in retail outlets and (c) a tobacco free-generation whereby people born after 2005 are unable to legally purchase tobacco. This paper estimates future smoking prevalence, mortality inequality and health-adjusted life year (HALY) impacts of these strategies., METHODS: We used a Markov model to estimate future yearly smoking and vaping prevalence, linked to a proportional multistate life table model to estimate future mortality and HALYs., RESULTS: The combined package of strategies (plus media promotion) reduced adult smoking prevalence from 31.8% in 2022 to 7.3% in 2025 for Maori, and 11.8% to 2.7% for non-Maori. The 5% smoking prevalence target was forecast to be achieved in 2026 and 2027 for Maori males and females, respectively. The HALY gains for the combined package over the population's remaining lifespan were estimated to be 594 000 (95% uncertainty interval (UI): 443 000 to 738 000; 3% discount rate). Denicotinisation alone achieved 97% of these HALYs, the retail strategy 19% and tobacco-free generation 12%.By 2040, the combined package was forcat to reduce the gap in Maori:non-Maori all-cause mortality rates for people 45+ years old by 22.9% (95% UI: 19.9% to 26.2%) for females and 9.6% (8.4% to 11.0%) for males., CONCLUSION: A tobacco endgame strategy, especially denicotinisation, could deliver large health benefits and dramatically reduce health inequities between Maori and non-Maori in Aotearoa/New Zealand. Copyright © Author(s) (or their employer(s)) 2023. Re-use permitted under CC BY-NC. No commercial re-use. See rights and permissions. Published by BMJ.
7747	Almutairi, Rami M., Al-Dubai, Sami A. R., Almutairi, Mai M., Alharbi, Hani H., Alshlahi, Hatim M., Albadrani, Muayad S. (2023) The degree of nicotine dependence among users of different tobacco and nicotine products in Madinah City, Saudi Arabia. Journal of family & community medicine; 30<br><br>**Keywords:**<br><br>**Abstract:** BACKGROUND: Nicotine addiction has many consequences. The World Health Organization has classified nicotine dependence as a disorder of substance use. This study aimed to assess the dependence in users of different tobacco and/or nicotine-containing products (TNPs)., MATERIALS AND METHODS: This analytical, cross-sectional study involved 211 TNP users in Madinah, Saudi Arabia. The data was collected using a self-administered questionnaire that consisted of two main sections. The first section included sociodemographic domain, TNP status domain, and the Stages of Change model domain. The second section of the instrument included the ABOUT dependence construct comprising 12 items. Independent t-test, analysis of variance, and correlation analysis were used to assess the relationship between the study variables., RESULTS: Most users of TNPs exclusively used tobacco cigarettes (53.1%). Total dependence score was significantly associated with gender, marital status, age group, monthly income, nicotine concentration in electronic cigarettes (e-cigarettes) liquid, and the number of cigarettes smoked per day ($P < 0.05$). Total dependence score was correlated with the duration of TNP usage ($r = 0.24$, $P < 0.001$), Switching attempts to another TNP ($r = 0.16$, $P = 0.020$), attempts of quitting TNPs ($r = 0.25$, $P < 0.001$), and willingness to quit ($r = -0.37$, $P < 0.001$)., CONCLUSION: Dependence was associated with gender, marital status, age group, monthly income, nicotine concentration in e-cigarette liquid, and the number of cigarettes smoked per day. It was also associated with duration of TNP usage, Switching attempts to another TNP, attempts of quitting TNPs and the willingness to quit. Copyright: © 2022 Journal of Family and Community Medicine.
7757	Alsanea, Sary, Alrabiah, Ziyad, Samreen, Sana, Syed, Wajid, Bin Khunayn, Rawan M., Al-Arifi, Nasser M., Alenazi, Miteb, Alghadeer, Sultan, Alhossan, Abdulaziz, Alwhaibi, Abdulrahman, Al-Arifi, Mohamed N. (2022) Prevalence, knowledge and attitude toward electronic cigarette use among male health colleges students in Saudi Arabia-A cross-sectional study. Frontiers in public health; 10<br><br>**Keywords:**

	Humans Male Vaping/ep [Epidemiology] *Vaping Cross-Sectional Studies *Electronic Nicotine Delivery Systems Prevalence Saudi Arabia/ep [Epidemiology] *Students, Nursing<br><br>**Abstract:** Background: Health care professionals have an important role in increasing awareness about smoking harms and serving as role models. This study aims to assess knowledge, attitude and perception toward electronic cigarettes (ECs) as well as prevalence of ECs use among male health colleges students., Method: This is a cross-sectional survey-based study conducted among students in the male campus of five different health colleges over a 4-month period from February 2020 to May 2020. Descriptive analysis was used to assess the knowledge, perception and attitude, and inferential testing was used to evaluate the association of different participant's variables and knowledge toward ECs usage using SPSS., Results: A total of 333 students were included in the analysis. Most of students (n = 205; 61.6%) had never used ECs, while 22.8 and 15.6% used them for recreational and smoking cessation purposes, respectively. Focusing on ECs users from each college individually, medical students had the highest prevalence followed by dental, pharmacy and nursing students (47.4, 40.7, 34.5, and 32%, respectively). Many students had misconceptions and a low level of knowledge about ECs, such as recognizing them as smoking-cessation tools and not knowing whether toxic and carcinogenic components levels in ECs are similar to conventional cigarettes, respectively. Medical students had significantly higher knowledge compared to dental students [3 (2) vs. 2 (1); p = 0.033]. Moreover, smokers were less knowledgeable than non-smokers [2.5 (1) vs. 2.1 (1), p = 0.027]. At least 62.8% of students perceived using ECs as a fashionable alternative smoking method and 59.2% believed that they may become a gateway for smoking addiction. Only 120 (36.0%) health colleges students were confidently able to advise smokers regarding ECs., Conclusion: Our study highlights an increased trend of ECs use accompanied with insufficient knowledge and several misconceptions about ECs among health colleges students. This was associated with a negative influence on their attitude toward ECs use, which would potentially lead to negative consequences on public health. Copyright © 2022 Alsanea, Alrabiah, Samreen, Syed, Bin Khunayn, Al-Arifi, Alenazi, Alghadeer, Alhossan, Alwhaibi and Al-Arifi.
7761	Althobaiti, Nawaf K., Mahfouz, Mohammad Eid M. (2022) Prevalence of Electronic Cigarette Use in Saudi Arabia. Cureus; 14<br><br>**Keywords:**<br><br>**Abstract:** BACKGROUND: The electronic cigarette (e-cigarette) was aggressively promoted as a healthier alternative to tobacco smoking. Despite the fact that e-cigarettes gained popularity among youth and young adults, there are no national data assessing the prevalence of e-cigarette use in Saudi Arabia., OBJECTIVE: The present study aims at establishing the prevalence and correlates of electronic cigarette use among adults in Saudi Arabia., DESIGN AND SETTING: A cross-sectional study was carried out to evaluate the prevalence of using electronic cigarettes in Saudi Arabia through a self-administered questionnaire. The data collected from the questionnaire were focusing on socio-demographic data as well as information on the type of smoking that adults are using. Data were represented in terms of frequencies and valid percentages for categorical variables. Statistical analysis was carried out using SPSS version 26., RESULTS: A total of 3374 participants responded to this questionnaire. Around 26% of this cohort have tried electronic cigarettes at least once in their lives. Additionally, there was a statistically significant difference (p value <0.001) in using smokeless tobacco, where e-cigarette smokers had higher incidence of smokeless tobacco smoking. Also, e-cigarette smokers had significantly higher (p value=0.002) frequency of smoking tobacco. Moreover, e-cigarette smoking was significantly higher among males (p value <0.001), with higher educational levels (p value <0.001) and age group between 18 and 24 years old., CONCLUSION: Electronic cigarette use is more common among young adults and those who have previously tried tobacco smoking. Other randomized studies are urgent to explore the extent of harmful effects of electronic cigarettes smoking (e-smoking) in the Kingdom. Copyright © 2022, Althobaiti et al.

7766	Alzahrani, Z., Zaidi, S. F., Alsolami, H., Bashrahil, B., Alghamdi, N., Nooh, M., Khan, M. A., Alshanberi, A. M., Qanash, S. (2022) Electronic cigarettes consumption and associated factors among general population in Western Saudi Arabia. Journal of Public Health Research; 11<br><br>**Keywords:**<br>anxiety article controlled study cross-sectional study depression education female household income human major clinical study male observational study occupation physiological stress prevalence questionnaire risk factor sample size Saudi Arabia *smoking smoking cessation tobacco dependence vaping menthol *electronic cigarette<br><br>**Abstract:** Background: In recent years, consumption of e-cigarettes has increased dramatically. Several studies have focused on the prevalence of e-cigarettes among specific groups of people, using it as a substitute to traditional cigarettes, or the participant knowledge regarding risks. This research was aimed on E-cigarettes' prevalence and its association to several factors in the general population of Western Saudi Arabia. Design and methods: Using an observational cross-sectional study, data were collected from (n=465) above 18 years old smokers during the survey at public attractions. A validated self-administered questionnaire acquired from previous studies was employed to insure the suitability for the general population of Jeddah Saudi Arabia. Sample size was calculated via Raosoft and adults of either gender were included in the study. Descriptive or inferential statistical analysis was performed using SPSS. Result(s): The preponderance of e-cigarette smokers used entertainment as the reason for smoking, with an average of (33.9%). Although one reason for e-cigarette consumption was to cease traditional smoking, results showed an average of (49.4%), which is the majority of those who attempted to cease traditional smoking via e-cigarette, did not succeed in quitting traditional smoking. This study also demonstrated that participant that believed that e-cigarette is beneficial had a higher chance to cease conventional smoking than who did not (32.1% versus 14.6%) which is significant (p<0.001). Conclusion(s): In conclusion, this study estimated the prevalence of e-cigarette consumption among the population of Jeddah, Saudi Arabia along with assessment of elements which help increase the overall e-cigarette consumption in Jeddah.Copyright © The Author(s), 2021.
7775	Andijani, O. (2022) Exploring The Effectiveness of E-Cigarettes usage upon Saudi Society as A Smoking cessation Tool. Australasian Medical Journal; 15<br><br>**Keywords:**<br>adult cross-sectional study current smoker demography female human Internet major clinical study male prevalence questionnaire Saudi Arabia short survey smoking *smoking cessation smoking habit tobacco use *vaping *electronic cigarette<br><br>**Abstract:** Background The electronic cigarette, often known as an "E-cigarette" or "vaporizer," is a nicotine delivery device that has been hailed as a healthier option for smokers. Since electronic cigarettes were first introduced, both internationally and in the Kingdom of Saudi Arabia (KSA), they have seen rapid growth in popularity, especially among the country's youth and young adults. In addition, many formerly non-smoking teens have taken up E-cigarette use. The safety profile of electronic cigarettes has been called into question by recent studies. Objective The purpose of this research is to quantify the extent to which youngsters and adults in Saudi Arabia use electronic cigarettes in their daily lives. The search also evaluates the potential addictiveness of electronic cigarettes and their efficacy as a smoking cessation tool. Design and methods Self-administered questionnaires were used in a cross-sectional study to determine how widespread the use of electronic cigarettes is in Saudi Arabia. The demographic and smoking habits of adult respondents were the primary focus of the survey's inquiry. Frequencies and reliable percentages were used to depict the data for categorical variables Results A total of 6,348 people participated in the internet questionnaire. Only 40.5 per cent of the individuals smoked regular cigarettes, but 59.5 per cent used electronic cigarettes. Results showed that over half (53.3 per cent) of e-cigarette users successfully used them to break the habit. A majority of current smokers (66.7 per cent)

	are considering switching to electronic cigarettes. About a quarter of the population (27.9 per cent) held the opinion that electronic cigarette use is more addictive than tobacco use. Additionally, 63.5 per cent of people said they think electronic cigarettes are safer than traditional cigarettes. Intriguingly, 41.5 per cent of those who tried quitting e-cigarettes were successful. Additionally, 63.7 per cent of respondents believed that using e-cigarettes helped them finally break the habit. Conclusion In conclusion, this study assessed the factors that contribute to the rising tide of e-cigarette use in Saudi Arabia and evaluated the prevalence of e-cigarette use among the population.Copyright © 2022, Australasian Medical Journal Pty Ltd. All rights reserved.
7791	Aonso-Diego, Gema, Secades-Villa, Roberto, Garcia-Perez, Angel, Weidberg, Sara, Fernandez-Hermida, Jose Ramon (2023) Association between e-cigarette and conventional cigarette use among Spanish adolescents. Asociacion entre el uso de cigarrillos electronicos y cigarrillos convencionales en adolescentes espanoles.; 0<br><br>**Keywords:**<br><br>**Abstract:** In recent years, studies have highlighted the upward trend in electronic cigarette use among adolescents, as well as the potential of e-cigarette use to lead to subsequent conventional cigarette use. The study's aims were two-fold: 1) to examine the progression from e-cigarette use to conventional cigarette use; and 2) to analyze the differences in the severity of smoking pattern among dual users (i.e., e-cigarette and conventional cigarette use), cigarette-only smokers, and e-cigarette-only users in a Spanish adolescent population. Data were obtained from the ESTUDES, a representative survey of addictive behaviors of Spanish adolescents aged 14-18, which was comprised of 38,010 adolescents (Mage = 15.69; SD = 1.19; 51.35% females). Results indicate that lifetime e-cigarette use increased the prevalence of subsequent conventional cigarette use by 1.86 times (95% CI 1.74, 1.99), and the prevalence of conventional cigarette use in the last month by 2.38 times (95% CI 2.19, 2.58), independently of whether the e-cigarette contains nicotine or not. Dual users showed a higher percentage of daily smokers, and a greater number of cigarettes per day, a higher use of e-cigarettes with nicotine, and an earlier age of smoking onset. Regarding risk perception, e-cigarette-only users perceived both conventional tobacco and e-cigarettes as less harmful (all p-values < .001). These findings document the strength of association between e-cigarette and conventional cigarettes, and underscore the importance of developing legal restrictions and prevention strategies aimed at reducing e-cigarette use, which in turn would reduce tobacco use.
7794	April-Sanders, A. K., Daviglus, M. L., Lee, U. J., Perreira, K., Pirzada, A., Bandiera, F., Giachello, A. L., Vu, T. T., Rodriguez, C. J. (2021) Prevalence of electronic cigarette use and its determinants in US persons of hispanic heritage: The hispanic community health study / study of Latinos (HCHS/SOL). Circulation; 144<br><br>**Keywords:**<br>adult aged alcohol consumption conference abstract controlled study coronary artery disease cultural factor demography *electronic cigarette English (language) female foreigner *Hispanic human *inheritance lifestyle major clinical study male middle aged non insulin dependent diabetes mellitus non-smoker obesity physical activity *prevalence *public health tertiary education young adult<br><br>**Abstract:** Introduction: Popularity and use of electronic nicotine delivery systems (ENDS or e-cigarettes), have increased tremendously in the United States (US). Yet, data in a representative sample of US Hispanics/Latinos is limited. We determined the prevalence and correlates of ENDS use from the national Hispanic Community Health Study/Study of Latinos (HCHS/SOL). Method(s): Cross-sectional data collected between the years 2015-2017 were analyzed to assess ENDS use (ever (current: use <= past 30 days; former: use > past 30 days) and never) among 11,623 adults aged 23+ years. Weighted prevalence estimates were reported and age-adjusted logistic regression models were used to examine associations between sociodemographic, lifestyle/behavioral, and clinical exposures with ENDS use (vs. nonsmokers

	and cigarette-only smokers, separately). Result(s): Mean age 47 years +/- 0.3 and 52% women. The weighted prevalence of current and former ENDS use was 2.0% and 10.4%, respectively. Over 67% of current ENDS users reported dualcigarette use. Current ENDS use prevalence estimates ranged from 1.1% in Hispanics/Latinos of South American background to 2.1% in Hispanics/Latinos of Mexican and Puerto Rican background. In logistic regression models, compared with nonsmokers, increased odds of current ENDS use was associated with males (OR=4.90, p<0.001), higher education (OR=2.03, p<0.05), English language preference (OR=7.59, p<0.001), Puerto Rican background (OR=2.09, p<0.05), a high level of physical activity (OR=4.02, p<0.001), and history of alcohol use (OR=15.93, p<0.001). Older age (>=45 years) (OR=0.30, p<0.001), foreign-born participants (OR=0.12, p<0.001), and those with obesity (OR=0.60, p<0.05) and type 2 diabetes (OR=0.46, p<0.001) had lower odds of current ENDS use. These trends were generally similar when cigarette-only smokers were the reference group. Having ever used ENDS was associated with prevalent coronary artery disease. Conclusion(s): Our study suggest that Hispanic/Latino individuals who are young adults, male, USborn, or have high acculturation are more likely to report current ENDS use.
7795	April-Sanders, A. K., Daviglus, M. L., Lee, U. J., Perreira, K. M., Kaplan, R. C., Blaha, M. J., Pirzada, A., Giachello, A. L., Bhatnagar, A., Robertson, R. M., Vu, T. H. T., Rodriguez, C. J. (2023) Prevalence of electronic cigarette use and its determinants in us persons of Hispanic/Latino background: The Hispanic community health study / study of Latinos (HCHS/SOL). American Journal of Medicine Open; 9<br><br>**Keywords:**<br>adult aged article cigarette smoking cohort analysis controlled study coronary artery disease cross-sectional study cultural factor English (language) female Food and Drug Administration Hispanic human major clinical study male middle aged non-smoker population research *prevalence public health *social determinants of health sociodemographics socioeconomics tertiary education *electronic cigarette<br><br>**Abstract:** Objective: To determine the prevalence and determinants of electronic nicotine delivery systems (ENDS) use among Hispanic/Latino adults from the Hispanic Community Health Study/Study of Latinos (HCHS/SOL). Method(s): Cross-sectional data collected between the years 2015-2017 were analyzed to assess ENDS use (ever (current: use <= past 30 days; former: use > past 30 days) and never) among 11,623 adults (mean age 47 years+/-0.3 years; 52% women). Weighted prevalence estimates were reported, and age-adjusted logistic regression models were used to examine associations between sociodemographic and clinical exposures with ENDS use. Result(s): The prevalence of current and former ENDS use was 2.0% and 10.4%, respectively. Having ever used ENDS was associated with prevalent coronary artery disease. Current ENDS use was higher in males and associated with higher education, English language preference, and Puerto Rican background compared with nonsmokers and cigarette-only smokers (all p<0.05). Conclusion(s): Hispanic/Latino individuals who are young adults, male, US-born, and have high acculturation were more likely to report current ENDS use. These findings could inform preventive and regulatory interventions targeted to Hispanics/Latinos.Copyright © 2022 The Author(s)
7809	Asharani, P. V., Seet, Vanessa, Devi, Fiona, Wang, Peizhi, Roystonn, Kumarasan, Subramaniam, Mythily (2022) Electronic nicotine delivery systems: prevalence and perception of risk/harm in individuals with mental illness. Singapore medical journal;<br><br>**Keywords:**<br><br>**Abstract:**
7815	Atem, Folefac D., Bluestein, Meagan A., Harrell, Melissa B., Chen, Baojiang, Messiah, Sarah E., Kuk, Arnold E., Sterling, Kymberle L., Spells, Charles E., Perez, Adriana (2022) Precise Estimation for the Age of Initiation of Tobacco Use Among U.S. Youth: Finding from the Population Assessment of Tobacco and

	Health (PATH) Study, 2013-2017. Biostatistics and biometrics open access journal; 11<br><br>**Keywords:**<br>**Abstract:** Context: Youth tobacco use remains a prominent United States public health issue with a high economic and health burden., Method: We pooled never and ever users at youth's first wave of PATH participation (waves 1-3) to estimate age of initiation for hookah, e-cigarettes, cigarettes, traditional cigars, cigarillos, and smokeless tobacco prospectively (waves 2-4). Age of initiation of each tobacco product was estimated using weighted interval-censored survival analyses. Weighted interval censoring Cox-proportional hazards regression models were used to assess the association of ever use of the TP at the first wave of PATH participation, sex, and race/ethnicity on the age of initiation of ever use of each tobacco product. Sensitivity analyses were performed to understand the impact of the recalled age of initiation for the left-censored participants by replacing the recalled age of initiation with a uniform "6" years lower bound., Results: The proportion of those who ever used each tobacco product at the first wave of PATH participation ranged from 1.8% for traditional cigars to 10.4% for cigarettes. There was a significant increase in ever use of each tobacco product after the age of 14, with e-cigarettes and cigarettes showing the highest cumulative incidence of initiation by age 21, while smokeless and cigarillos recorded the lowest cumulative incidence by age 21. The adjusted Cox models showed boys initiated at earlier ages for all of these tobacco products except for hookah, which showed no difference. Similarly, apart from ever use of hookah, non-Hispanic White youth were more likely to initiate each tobacco product at earlier ages compared to Hispanic, non-Hispanic Black, and non-Hispanic Other youth., Conclusion: The increased sample size and the inclusion of ever users yielded greater precision for age of initiation of each tobacco product than analyses limited to never users at the first wave of PATH participation. These analyses can help elucidate population selection criteria for estimating the age of initiation of tobacco products.
7819	Austin-Datta, Rebecca J., Chaudhari, Piyush Vilas, Cheng, Ting-Yuan David, Klarenberg, Geraldine, Striley, Catherine Woodstock, Cottler, Linda B. (2023) Electronic Nicotine Delivery Systems (ENDS) use Among Members of a Community Engagement Program. Journal of community health; 48<br><br>**Keywords:**<br>Humans Male Female Adolescent Young Adult Adult *Electronic Nicotine Delivery Systems Tobacco Use Florida/ep [Epidemiology] Data Collection Longitudinal Studies *Tobacco Products<br><br>**Abstract:** Electronic nicotine delivery systems (ENDS) are relatively new and ENDS use data from community engagement programs may help us understand usage patterns and facilitate targeted longitudinal studies. Community members in Florida, USA, were asked about ENDS use, tobacco use, and health history/concerns by Community Health Workers. Among 7253 members recruited during 2014 to 2021 into our HealthStreet program, 1177 had ever used ENDS; the proportion increased from 12 to 27% from 2014 to 2021 (adjusted odds ratio (aOR) 2.5; 95% CI 1.7-3.5; Ever versus never used ENDS). Ever tobacco use was strongly associated with ENDS use; 69% of ever users were current tobacco users. Demographic determinants (sex, age, race) and food insecurity were strongest predictors of ENDS use. Most who had ever used ENDS were aged 18-25 (aOR 5.9; 95% CI 4.6-7.6; vs. aged 60 + years), White (aOR 3.7; 95% CI 3.2-4.3; vs. Black/African American), male (aOR 1.5; 95% CI 1.3-1.7; vs. female), and recently food insecure (aOR 1.8; 95% CI 1.5-2.0; vs. not recently food insecure). Those with respiratory issues were more likely to have used ENDS compared to those without (aOR 2.0; 95% CI 1.6-2.6; aOR 1.3; 95% CI 1.1-1.5). Members concerned about hypertension were less likely to have used ENDS (aOR 0.7; 95% CI 0.5-0.9). In this relatively rural, micropolitan sample, tobacco use, socio-economic determinants, and certain health history/concerns were strongly associated with ENDS use. Community outreach approaches are needed to further understand these factors and implement interventions. Copyright © 2022. The Author(s), under exclusive licence to Springer Science+Business Media, LLC, part of Springer Nature.

7843	Baishya, Mona L., Martinasek, Mary, Bell, Lisa, Wheldon, Christopher W. (2023) Adverse Childhood Experiences and Adult Tobacco Use Among Sexual and Gender Minority Populations: Examining the Role of Mental, Social, and Community Health. LGBT health; 10<br><br>**Keywords:**<br>Humans Adult Male Female Gender Identity *Adverse Childhood Experiences Public Health Sexual Behavior/px [Psychology] *Sexual and Gender Minorities Tobacco Use<br><br>**Abstract:** Purpose: We sought to evaluate the relationship between adverse childhood experiences (ACEs) and tobacco use, to assess the intersection of sexual orientation or gender identity (SOGI), and to explore risk and protective aspects of mental, social, and community health. Methods: This was a secondary analysis of the sexual and gender minorities (SGM) subsample (n = 1102) from a 2019 Community Health Needs Assessment. Nested binary logistic regression models were created to evaluate associations between ACEs and type of tobacco use: (1) cigarette use versus no cigarette or electronic nicotine delivery systems (ENDS) use, (2) ENDS use versus no cigarette or ENDS use, and (3) dual use versus use of either cigarettes or ENDS. Results: The sample population that reported four or more ACEs and ENDS use had statistically significant adjusted odds ratios (aORs) when adjusting the models for sociodemographic variables (aOR = 2.55; 95% confidence intervals [CIs]: 1.54-4.22), SOGI (aOR = 2.44; 95% CI: 1.46-4.10), current mental health (aOR = 2.23; 95% CI: 1.32-3.75), and social and community health (aOR = 2.19; 95% CI: 1.30-3.71). The sample population that reported poor mental health had statistically significant aORs when adjusting the models for sociodemographic variables, SOGI, and mental health (aOR = 2.26; 95% CI: 1.32-3.89) and social and community health (aOR = 2.06; 95% CI: 1.17-3.54). There was no statistically significant finding related to the other tobacco use variables (tobacco use and dual use). Conclusion: We found that high-risk ACEs were positively associated with cigarette use and ENDS use. SGM populations have differing experiences when considering community and social support.
7869	Beard, Emma, Brown, Jamie, Shahab, Lion (2022) Association of quarterly prevalence of e-cigarette use with ever regular smoking among young adults in England: a time-series analysis between 2007 and 2018. Addiction (Abingdon, England); 117<br><br>**Keywords:**<br>Adolescent Bayes Theorem *Electronic Nicotine Delivery Systems Humans Prevalence Smoking/ep [Epidemiology] Vaping/ep [Epidemiology] *Vaping Young Adult<br><br>**Abstract:** AIMS: To assess how changes in the prevalence of e-cigarette use among young adults have been associated with changes in the uptake of smoking in England between 2007 and 2018., DESIGN: Time-series analysis of population trends with autoregressive integrated moving average with exogeneous input (ARIMAX models)., SETTING: England., PARTICIPANTS: Data were aggregated quarterly on young adults aged 16-24 years (n = 37 105) taking part in the Smoking Toolkit Study., MEASURES: In the primary analysis, prevalence of e-cigarette use was used to predict prevalence of ever regular smoking among those aged 16-24. Sensitivity analyses stratified the sample into those aged 16-17 and 18-24. Bayes' factors and robustness regions were calculated for non-significant findings [effect size beta coefficient (B) = 3.1]., FINDINGS: There was evidence for no association between the prevalence of e-cigarette use and ever regular smoking among those aged 16-24 [B = -0.015, 95% confidence interval (CI) = -0.046 to 0.016; P = 0.341; Bayes factor (BF) = 0.002]. Evidence for no association was also found in the stratified analysis among those aged 16-17 (B = 0.070, 95% CI -0.014 to 0.155, P = 0.102; BF = 0.015) and 18-24 (B = -0.021, 95% CI -0.053 to 0.011; P = 0.205; BF = 0.003). These findings were able to rule out percentage point increases or decreases in ever regular smoking prevalence greater than 0.31% or less than -0.03% for 16-17-year-olds and 0.01 or -0.08% for 18-24-year-olds for every 1%-point increase in e-cigarette prevalence., CONCLUSION: Prevalence of e-cigarette use among the youth population in England does not appear to be associated with substantial increases or decreases in the prevalence of

	smoking uptake. Small associations cannot be ruled out. Copyright © 2022 The Authors. Addiction published by John Wiley & Sons Ltd on behalf of Society for the Study of Addiction.
7880	Benjakul, Sarunya, Nakju, Saroj, Termsirikulchai, Lakkhana (2022) Use of e-cigarettes among public health students in Thailand: Embedded mixed-methods design. Tobacco induced diseases; 20<br><br>**Keywords:**<br><br><br>**Abstract:** INTRODUCTION: E-cigarettes are new tobacco products widely used among adolescents. Public health students are not only susceptible to e-cigarette use, but they should also serve as non-smoking role models. The study aimed to investigate the current situation of e-cigarette use and the factors associated with its use among public health students., METHODS: In this embedded mixed-methods design, the primary approach was a cross-sectional online survey. The samples were 2302 third-year public health students from 37 public health education institutes across Thailand. Stratified two-stage cluster random sampling was employed to select the subjects. Data were collected using self-administered questionnaires from January to March 2021. A checklist form was employed to collect qualitative data about teaching and learning activities related to tobacco control in June 2021. Descriptive statistics were used for data analysis, including inferential statistics regarding logistic regression., RESULTS: Overall, 3.9% (95% CI: 3.1-4.6) of the students currently used e-cigarettes in the past 30 days. The significant factors that could explain 43.4% of e-cigarette use were predisposing factors: being male (adjusted odds ratio, AOR=1.8; 95% CI: 1.0-3.3), having a neutral attitude toward e-cigarette use (AOR=2.2; 95% CI: 1.1-4.5), and not believing that public health professionals should serve as non-smoking role models for clients and the general public (AOR=2.3; 95% CI: 1.2-4.0). The enabling factor was having tried tobacco products (AOR=40.7; 95% CI: 19.1-87.1), and the reinforcing factor was having three or more close friends who smoke cigarettes (AOR=3.2; 95% CI: 1.8-5.8)., CONCLUSIONS: Students' behaviors should be modified through curriculum-based teaching and learning activities to develop negative attitudes toward e-cigarette smoking, increase students' awareness as non-smoking role models, and establish smoke-free environments. Copyright © 2022 Benjakul S. et al.
7881	Bennett, Breesa, Romm, Katelyn F., Berg, Carla J. (2022) Changes in cigarette and e-cigarette use among US young adults from before to during the COVID-19 pandemic: News exposure and risk perceptions as potential predictors. Tobacco prevention & cessation; 8<br><br>**Keywords:**<br><br><br>**Abstract:** INTRODUCTION: COVID-19 impacted cigarette and e-cigarette use behaviors among some individuals. This study examined COVID-19 factors and prior substance use as predictors of cigarette and e-cigarette cessation and initiation among US young adults from before to during the COVID-19 pandemic., METHODS: We analyzed data from Wave 3 (Sept-Dec 2019) and Wave 5 (Sept-Dec 2020) of a 2-year, 5-wave longitudinal study of young adults across six US metropolitan areas. We examined COVID-19 news exposure, perceived smoking and e-cigarette use risk, and prior substance use, as predictors of cigarette and e-cigarette cessation and initiation, respectively., RESULTS: Of W3 cigarette users (n=516), 37.8% (n=195) quit cigarettes at W5; predictors of cessation included younger age, fewer days of W3 past-month cigarette use, and no W3 e-cigarette use. Of W3 e-cigarette users (n=687), 38.7% (n=266) quit e-cigarettes at W5; predictors included greater COVID-19 news exposure, fewer days of W3 past-month e-cigarette use, and no W3 cigarette use. Of W3 cigarette non-users (n=1693), 5.0% (n=85) initiated cigarettes at W5; predictors of initiation included younger age, lower perceived smoking risk, lifetime cigarette and e-cigarette use, and W3 e-cigarette use. Of W3 e-cigarette non-users (n=1522), 6.3% (n=96) initiated e-cigarettes at W5; predictors included younger age, less news exposure, lifetime cigarette and e-cigarette use, and W3 cigarette use., CONCLUSIONS: These findings underscore the need

	to address cigarette and e-cigarette co-use and related risk perceptions in prevention and cessation interventions. Copyright © 2022 Bennett B. et al.
7890	Berlowitz, Jonathan B., Xie, Wubin, Harlow, Alyssa F., Blaha, Michael J., Bhatnagar, Aruni, Benjamin, Emelia J., Stokes, Andrew C. (2023) Cigarette-E-cigarette Transitions and Respiratory Symptom Development. American journal of preventive medicine; 64<br><br>**Keywords:**<br>Adult Humans *Electronic Nicotine Delivery Systems Cough/ep [Epidemiology] Cough/et [Etiology] *Tobacco Products Smoking/ae [Adverse Effects] Smoking/ep [Epidemiology] Vaping/ae [Adverse Effects] Vaping/ep [Epidemiology] *Vaping<br><br>**Abstract:** INTRODUCTION: E-cigarette use is associated with pulmonary inflammation, functional respiratory changes, and chronic lung disease. Most population-level E-cigarette research has utilized point-in-time measures of E-cigarette exposures, which may not generalize to adults who transition between cigarettes and E-cigarettes., METHODS: Data obtained from the Population Assessment of Tobacco and Health study were collected from 2013 to 2019 and analyzed in 2022. Three observations were created per respondent, with exposure intervals assessed over Waves 1-2, 2-3, and 3-4. Each wave of the exposure interval was classified as nonuse, exclusive E-cigarette use, exclusive smoking, or dual use, producing 16 possible cigarette-E-cigarette transitions. The association between transitions and both dry nighttime cough and wheeze symptom development during follow-up were assessed using mixed-effects Poisson models., RESULTS: Among 33,231 observations from 13,528 unique participants, transitioning from nonuse to exclusive E-cigarette use was associated with 1.62 times higher incidence rate of wheeze (incident rate ratio=1.62; 95% CI=1.12, 2.34) than persistent nonuse. There was no change in reported dry nighttime cough (incident rate ratio=0.84; 95% CI=0.52, 1.35) or wheeze (incident rate ratio=0.87; 95% CI=0.52, 1.46) in individuals who switched from cigarettes to E-cigarettes, whereas transitioning from dual use to E-cigarette use was associated with large reductions in both symptoms (incident rate ratio=0.58; 95% CI=0.39, 0.87 and incident rate ratio=0.36; 95% CI=0.20, 0.63, respectively)., CONCLUSIONS: E-cigarette initiation among nonusers is associated with increased respiratory morbidity. Further research should assess the risks and benefits of E-cigarette-assisted cigarette cessation given the reduction in symptom development rates among dual use to E-cigarette switchers. Copyright © 2022 American Journal of Preventive Medicine. Published by Elsevier Inc. All rights reserved.
7920	Blank, Melissa D., Romm, Katelyn F., Childers, Margaret G., Douglas, Ashley E., Dino, Geri, Bray, Bethany C. (2023) Longitudinal transitions in adolescent polytobacco use across waves 1-4 of the Population Assessment of Tobacco and Health study. Addiction (Abingdon, England); 118<br><br>**Keywords:**<br>Humans Male Adolescent United States/ep [Epidemiology] Female Tobacco *Electronic Nicotine Delivery Systems Tobacco Use/ep [Epidemiology] *Tobacco Products *Tobacco, Smokeless<br><br>**Abstract:** AIMS: This study aims to identify adolescent patterns of polytobacco use and measure transitions between patterns over time., DESIGN: Longitudinal analysis using data derived from waves 1-4 (2013-18) of the Population Assessment of Tobacco and Health (PATH) study. Transitions in tobacco use patterns were examined via latent transition analysis, and then, socio-demographic characteristics were used to predict transitions via logistic regression., SETTING: United States., PARTICIPANTS: Participants included 975 adolescents who used at least one tobacco product at any wave (W1 mean age = 13.29, standard deviation = 0.86; W4 54.2% male; 54.5% white, 25.9% Hispanic)., MEASUREMENTS: Measurements included past 30-day use of cigarettes, electronic cigarettes (e-cigs), traditional cigars, cigarillos, filtered cigars, snus, smokeless tobacco (SLT) or hookah., FINDINGS: Six latent classes were identified. Cigarette users (43.5-58.8%) and SLT users (50.8-79.6%) tended to persist in their use over time. E-cig users began to probably transition to non-users (80.0%), but became more likely to persist in

	this use over time (31.1%). Non-users at a given wave were most likely to transition to e-cig users (8.5-43.7%) or cigarette users (6.7-28.6%). Cigarillo/poly-users and hookah/poly-users displayed more variable transition patterns. Adolescents were more likely to transition to non-use (versus become/remain e-cig users) if they were older (cigarette users, SLT users), younger (e-cig users), other race (SLT users), male (SLT users) or had less-educated parents (SLT users) compared with their counterparts. Hispanic (versus white) cigarette users were more likely to transition to non-users than to persist in this use., CONCLUSIONS: Among adolescents in the United States, patterns of tobacco use characterized by the use, mainly, of single, specific products appear to be stable, particularly by late adolescence. In contrast, patterns characterized by polytobacco use appear to be more variable and may represent experimentation without specialization. Copyright © 2022 Society for the Study of Addiction.
7926	Bluestein, Meagan A., Kuk, Arnold E., Harrell, Melissa B., Chen, Baojiang, Hebert, Emily T., Perez, Adriana (2023) Longitudinal Transition Patterns of Tobacco Use Among Youth and Young Adults Never Tobacco Product Users: Findings From the Population Assessment of Tobacco and Health Study, 2014-2019. Tobacco use insights; 16<br><br>**Keywords:**<br><br>**Abstract:** Aims: To identify, visualize, and describe the prevalence of within-product patterns of tobacco use behaviors for e-cigarettes, cigarettes, and hookah (TP) by 3 age groups (ie, 12-14-year-old, 15-17-year-old, and 18-20-year-old) with U.S. nationally representative data., Methods: In 2014-2015, never users of each (TP) and age group were followed-up longitudinally between 2015-2019 using five transition states: non-susceptible to (TP) use, susceptible to (TP) use, ever (TP) use, past 30-day (TP) use, and discontinued past 30-day (TP) use. Sankey diagrams were used to graphically visualize patterns in tobacco use behaviors across time., Results: Among 12-14-year-old who were never users and susceptible to each TP from 2014-2017, 7% initiated ever e-cigarette use and 9.4% first reported past 30-day use by 2018-2019; 5.8% initiated ever cigarette use and 3% first reported past 30-day cigarette use by 2018-2019; and, 4.5% initiated ever hookah use and 1.0% first reported past 30-day hookah use by 2018-2019. Among 15-17-year-old who were never users and susceptible to each TP from 2014-2017, 4.2% initiated ever e-cigarette use and 9.0% first reported past 30-day use by 2018-2019; 4.5% initiated ever cigarette use and 3% first reported past 30-day cigarette use by 2018-2019; and, 4.5% initiated ever hookah use and 2.4% first reported past 30-day hookah use by 2018-2019. Among 18-20-year-old who were never users and susceptible to each TP from 2014-2017, 3.2% initiated ever e-cigarette use and 3.6% first reported past 30-day e-cigarette use by 2018-2019; 3.0% initiated ever cigarette use and 2.3% first reported past 30-day cigarette use; and, 2.8% initiated ever hookah use and 1.0% first reported past 30-day hookah use by 2018-2019., Conclusions: From 2014 to 2019, onset and progression of e-cigarette, cigarette, and hookah use occurred more frequently in 12-14 and 15-17-year-old than in young adults 18-20-year-old. Copyright © The Author(s) 2023.
7955	Braillon, Alain (2022) Could Smoking Initiation Among Youth by E-cigarettes Warrant a Shorter Definition for Epidemiology?. Epidemiology (Cambridge, Mass.); 33<br><br>**Keywords:**<br>Adolescent *Electronic Nicotine Delivery Systems Humans Smoking/ep [Epidemiology] *Tobacco Products<br><br>**Abstract:**
7956	Braillon, Alain, Lang, Adam Edward (2023) Electronic nicotine delivery systems: pragmatism or expectancy?. Lancet (London, England); 401<br><br>**Keywords:**

	Humans *Electronic Nicotine Delivery Systems Nicotine/ae [Adverse Effects] *Tobacco Products<br><br>**Abstract:**
7958	Braymiller, Jessica (2021) Epidemiology of nicotine use among adolescents and young adults in the United States: Findings from the PATH study. Dissertation Abstracts International: Section B: The Sciences and Engineering; 82<br><br>**Keywords:**<br>*Nicotine *Tobacco Smoking *Trends *Electronic Cigarettes *Cannabis Use Empirical Methods Epidemiology Marijuana<br><br>**Abstract:** In 2014, electronic nicotine delivery systems (ENDS, also referred to as vaping or e-cigarette use) surpassed combustible cigarettes as the most commonly used tobacco product among youth. This is a critical public health concern as early onset of ENDS use has been linked to subsequent initiation of cigarettes and other tobacco products. Among adolescents and young adults who have used tobacco, the use of two or more products is also quite common. However, detailed information regarding specific patterns and trends in use within the context of increasingly popular ENDS is lacking. The current dissertation aimed to address this critical gap in three ways. First, chapter one reviews the distinguishing features of a variety of vaping devices, as well as the correlates and consequences associated with their use among adolescents and young adults. Next, chapters two and three empirically examine national patterns and age-trends in the use of ENDS and other nicotine delivery systems. Finally, sociodemographic differences in these patterns and trends, as well as the association between nicotine and marijuana use, are examined.The first empirical study examined nuanced age-trends in recent combustible cigarette, ENDS, and hookah use continuously from ages 14 to 24. Age-varying associations between marijuana use and each tobacco product were also examined. Data were from Wave 3 of the Population Assessment of Tobacco and Health (PATH) Study. The prevalence of past 30-day cigarette, ENDS, hookah, and marijuana use followed distinct age-trends. Cigarette and ENDS use were also significantly related to recent marijuana use across most ages. The association between ENDS and marijuana use was strongest at ages 14 and 15, whereas the association between cigarette and marijuana use was strongest at age 17. Age-varying prevalences, as well as the age-varying association between ENDS and marijuana, differed by sex and race/ethnicity.The second empirical study examined patterns and transitions in lifetime nicotine use among young adults ages 18 to 24 using latent class and latent transition analysis (LCA, LTA). Data were from Wave 1 and Wave 2 of the PATH Study. LCA demonstrated that young adults were best represented by five distinct patterns of use: ENDS Only (2%), Hookah Only (10%), Cigarettes Only (19%), Combustibles Only (23%), and Poly, No Smokeless (45%). Differences in latent class membership emerged based on sex and race/ethnicity; the proportion of males and NH white young adults were highest in the ENDS only, Combustibles Only, and Poly, No Smokeless classes. Young adults who had ever used marijuana were also more prevalent within the Combustibles Only and Poly, No Smokeless classes. Young adults in the Combustibles Only class at Wave 1 had a 31% chance of starting to use ENDS by Wave 2. Young adults in the Poly, No Hookah class at Wave 1 had an 11% chance of starting to use hookah by Wave 2.Overall, this dissertation highlights the importance of research examining ENDS, polytobacco, and marijuana use among young people. Innovative statistical techniques provided added insight into who is at risk for using specific nicotine delivery systems at what age, as well as the factors contributing to polytobacco use and co-use with other drugs. Detailed information of this kind is necessary in order to prevent nicotine addiction and the associated health consequences, and to reduce overall burden of nicotine-related illnesses on the United States. (PsycInfo Database Record (c) 2022 APA, all rights reserved)
7969	Brouwer, A. F., Jeon, J., Cook, S. F., Usidame, B., Hirschtick, J. L., Jimenez-Mendoza, E., Mistry, R., Fleischer, N. L., Holford, T. R., Mendez, D., Levy, D. T., Meza, R. (2022) The Impact of Menthol Cigarette Flavor in the U.S.: Cigarette and ENDS Transitions by Sociodemographic Group. American Journal of Preventive Medicine; 62

	**Keywords:**<br>adult article cigarette smoking educational status female *flavor human income male race difference smoking ban smoking cessation United States vaping young adult *menthol *cigarette *electronic cigarette<br><br>**Abstract:** Introduction: A better understanding of how menthol cigarette flavoring and ENDS impact smoking initiation, cessation, and transitions between tobacco products could help elucidate the potential impact of a U.S. menthol ban on combustible tobacco products. Method(s): A multistate transition model was applied to data on 23,232 adults from Waves 1-4 (2013-2017) of the Population Assessment of Tobacco and Health Study (analysis was conducted in 2020-2021). Transition rates among never, noncurrent, nonmenthol versus menthol cigarette, ENDS, and dual everyday/someday use were estimated, as were transition-specific hazard ratios for age, sex, race/ethnicity, education, and income. Result(s): Non-Hispanic Blacks who smoked menthol discontinued smoking at a much lower rate than those who smoked nonmenthol (hazard ratio=0.43, 95% CI=0.29, 0.64), but there was no statistically significant difference in the discontinuation rates among non-Hispanic Whites (hazard ratio=0.97, 95% CI=0.80, 1.16) or Hispanics (hazard ratio=0.81, 95% CI=0.56, 1.16). Non-Hispanic Whites who smoked menthol were more likely to become dual users than those who smoked nonmenthol (hazard ratio=1.43, 95% CI=1.14, 1.80). Young adults initiated menthol smoking at a higher rate than older adults (age 18-24 years versus >=55 years: hazard ratio=2.45, 95% CI=1.44, 4.15) but not nonmenthol smoking (hazard ratio=1.02, 95% CI=0.62, 1.69). There were differences by sex in the impact of menthol flavor on smoking initiation and discontinuation but little difference by education or income. Conclusion(s): Sociodemographic differences in product transitions should be accounted for when estimating the potential impact of a menthol ban.Copyright © 2021 American Journal of Preventive Medicine
7970	Brouwer, Andrew F., Jeon, Jihyoun, Jimenez-Mendoza, Evelyn, Land, Stephanie R., Holford, Theodore R., Friedman, Abigail S., Tam, Jamie, Mistry, Ritesh, Levy, David T., Meza, Rafael (2023) Changing patterns of cigarette and ENDS transitions in the USA: a multistate transition analysis of youth and adults in the PATH Study in 2015-2017 vs 2017-2019. Tobacco control;<br><br>**Keywords:**<br><br>**Abstract:** INTRODUCTION: It is unknown how recent changes in the tobacco product marketplace have impacted transitions in cigarette and electronic nicotine delivery system (ENDS) use., METHODS: A multistate transition model was applied to 24 242 adults and 12 067 youth in waves 2-4 (2015-2017) and 28 061 adults and 12 538 youth in waves 4 and 5 (2017-2019) of the Population Assessment of Tobacco and Health Study. Transition rates for initiation, cessation and product transitions were estimated in multivariable models, accounting for gender, age group, race/ethnicity and daily versus non-daily product use., RESULTS: Changes in ENDS initiation/relapse rates depended on age, including among adults. Among youth who had never established tobacco use, the 1-year probability of ENDS initiation increased after 2017 from 1.6% (95% CI 1.4% to 1.8%) to 3.8% (95% CI 3.4% to 4.2%). Persistence of ENDS-only use (ie, 1-year probability of continuing to use ENDS only) increased for youth from 40.7% (95% CI 34.4% to 46.9%) to 65.7% (95% CI 60.5% to 71.1%) and for adults from 57.8% (95% CI 54.4% to 61.3%) to 78.2% (95% CI 76.0% to 80.4%). Persistence of dual use similarly increased for youth from 48.3% (95% CI 37.4% to 59.2%) to 60.9% (95% CI 43.0% to 78.8%) and for adults from 40.1% (95% CI 37.0% to 43.2%) to 63.8% (95% CI 59.6% to 67.6%). Youth and young adults who used both products became more likely to transition to ENDS-only use, but middle-aged and older adults did not., CONCLUSIONS: ENDS-only and dual use became more persistent. Middle-aged and older adults who used both products became less likely to transition to cigarette-only use but not more likely to discontinue cigarettes. Youth and young adults became more likely to transition to ENDS-only use. Copyright © Author(s) (or their employer(s))

	2023. Re-use permitted under CC BY-NC. No commercial re-use. See rights and permissions. Published by BMJ.
7971	Brouwer, Andrew F., Levy, David T., Jeon, Jihyoun, Jimenez-Mendoza, Evelyn, Sanchez-Romero, Luz Maria, Mistry, Ritesh, Meza, Rafael (2022) The Impact of Current Tobacco Product Use Definitions on Estimates of Transitions Between Cigarette and ENDS Use. Nicotine & tobacco research : official journal of the Society for Research on Nicotine and Tobacco; 24<br><br>**Keywords:**<br>Adult Humans United States/ep [Epidemiology] *Electronic Nicotine Delivery Systems Nicotine *Tobacco Products Tobacco Use/ep [Epidemiology] *Tobacco Use Disorder Tobacco<br><br>**Abstract:** INTRODUCTION: Definitions of current tobacco and nicotine delivery product use vary and depend on frequency of use, established-use criteria, and the product type. Previous research has not considered how transition rates between current use of different products depend on the current use definition., AIMS AND METHODS: We applied a multistate transition model to data on U.S. adults from waves 1-4 (2013-2017) of the Population Assessment of Tobacco and Health (PATH) study. We estimated transition rates between never, non-current, cigarette, electronic nicotine delivery systems (ENDS), and dual use states with and without established-use criteria (has smoked 100+ cigarettes in their lifetime; ever fairly regularly used ENDS) and different frequency thresholds (1+, 10+, 20+, and 30 days of the past 30 days). We considered use below a frequency threshold as either non-current use or a distinct, infrequent use category., RESULTS: When treating use below a frequency threshold as non-current use, transition probability estimates were largely robust to the choice of use frequency threshold, although sole ENDS users were more likely to transition to non-current use or dual use as the current use threshold increased. Removing the established-use criterion for ENDS reduced the estimates of sole ENDS and dual users staying in their use state. When treating infrequent use as a separate category, transition probability estimates were dependent on the use frequency threshold, particularly transitions among the dual use states., CONCLUSIONS: Product use definitions have important implications for assessing product use transitions and thus the public health implications of cigarette and ENDS control strategies., IMPLICATIONS: How we define "current use" of tobacco and nicotine delivery products changes our estimates of how individuals transition to, between, and from different patterns of use. We show that the robustness of transition estimates to whether or not non-established users are included as current users and to different frequency-of-use threshold depends in part on whether low-frequency users are categorized as non-current users or as a distinct category. Our results emphasize the importance of intentional definitions of product use that reflect the larger goals of public health and tobacco control. Copyright © The Author(s) 2022. Published by Oxford University Press on behalf of the Society for Research on Nicotine and Tobacco.
7990	Buu, Anne, Hu, Yi-Han, Wong, Su-Wei, Lin, Hsien-Chang (2021) Internalizing and externalizing problems as risk factors for initiation and progression of e-cigarette and combustible cigarette use in the US youth population. International Journal of Mental Health and Addiction; 19<br><br>**Keywords:**<br>*Externalization *Internalization *Risk Factors *Tobacco Smoking *Electronic Cigarettes Intervention Mental Health Prevention Public Health Adolescent Behavior Child Behavior<br><br>**Abstract:** This study examined whether internalizing and externalizing problems were associated with initiation and progression of e-cigarette/cigarette use. The youth from the Population Assessment of Tobacco and Health Study Waves 1-2 were included in this study (n = 9258). Multinomial logistic and linear regression were performed to investigate whether Wave 1 internalizing and externalizing problems were associated with Wave 2 initiation and progression of cigarette/e-cigarette use. Higher internalizing and externalizing problems were both associated with higher risk for initiating cigarette smoking and exclusive e-cigarette use. Neither internalizing nor externalizing problems were associated with (1)

	differential likelihood of initiating exclusive e-cigarette use when compared with initiating cigarette smoking and (2) more frequent use of cigarettes or e-cigarettes. Schools and community public health practitioners should provide prevention and intervention efforts among the target population. Improving the youth's mental and behavioral health could have a secondary effect on preventing cigarette and e-cigarette use initiation. (PsycInfo Database Record (c) 2022 APA, all rights reserved)
7992	Buu, Anne, Tong, Zhaoxue, Cai, Zhanrui, Li, Runze, Yang, James J., Jorenby, Douglas E., Piper, Megan E. (2023) Subtypes of Dual Users of Combustible and Electronic Cigarettes: Longitudinal Changes in Product Use and Dependence Symptomatology. Nicotine & tobacco research : official journal of the Society for Research on Nicotine and Tobacco; 25<br><br>**Keywords:**<br>Humans *Electronic Nicotine Delivery Systems *Smoking Cessation Cross-Sectional Studies Smoking/ep [Epidemiology] *Tobacco Products Vaping/ep [Epidemiology] *Vaping<br><br>**Abstract:** INTRODUCTION: Cross-sectional surveys found behavioral heterogeneity among dual users of combustible and electronic cigarettes. Yet, prior classification did not reflect dynamic interactions between cigarette and e-cigarette consumption, which may reveal changes in product-specific dependence. The contexts of dual use that could inform intervention were also understudied., METHODS: This study conducted secondary analysis on 13 waves of data from 227 dual users who participated in a 2-year observational study. The k-means method for joint trajectories of cigarette and e-cigarette consumption was adopted to identify the subtypes of dual users. The time-varying effect model was used to characterize the subtype-specific trajectories of cigarette and e-cigarette dependence. The subtypes were also compared in terms of use contexts., RESULTS: The four clusters were identified: light dual users, predominant vapers, heavy dual users, and predominant smokers. Although heavy dual users and predominant smokers both smoked heavily at baseline, by maintaining vaping at the weekly to daily level the heavy dual users were able to considerably reduce cigarette use. Yet, the heavy dual users' drop in cigarette dependence was not as dramatic as their drop in cigarette consumption. Predominant vapers appeared to engage in substitution, as they decreased their smoking and increased their e-cigarette dependence. They were also more likely to live in environments with smoking restrictions and report that their use of e-cigarettes reduced cigarette craving and smoking frequency., CONCLUSIONS: Environmental constraints can drive substitution behavior and the substitution behavior is able to be sustained if people find the substitute to be effective., IMPLICATIONS: This study characterizes subtypes of dual users based on the dynamic interactions between cigarette use and e-cigarette use as well as product-specific trajectories of dependence. The subtypes differ in not only sociodemographic characteristics but also contexts of cigarette and e-cigarette use. Higher motivation to use e-cigarettes to quit smoking and less permissive environment for smoking may promote substitution of cigarettes by e-cigarettes. Copyright © The Author(s) 2022. Published by Oxford University Press on behalf of the Society for Research on Nicotine and Tobacco. All rights reserved. For permissions, please e-mail: journals.permissions@oup.com.
8017	Carreras, Giulia, Minardi, Valentina, Lugo, Alessandra, Gallus, Silvano, Masocco, Maria, Spizzichino, Lorenzo, Gorini, Giuseppe (2022) Italians are still loyal to conventional cigarettes, despite novel tobacco products. Annali dell'Istituto superiore di sanita; 58<br><br>**Keywords:**<br>Adult Humans *Electronic Nicotine Delivery Systems Tobacco Cross-Sectional Studies *Tobacco Products *Vaping Surveys and Questionnaires<br><br>**Abstract:** INTRODUCTION: Over the last few decades in Italy, we observed a substantial reduction in conventional tobacco cigarette consumption, the introduction of electronic cigarettes (e-cigarette) in 2010, and the launch of heated tobacco products (HTP) in 2015., METHODS: We investigated novel products, i.e. e-cigarettes and HTP, use in Italy in 2018-2021 using data from the cross-sectional annual

	PASSI (Progressi delle Aziende Sanitarie per la Salute in Italia) survey conducted in representative samples of adults aged 18-69 (overall n = 101,458). We compared characteristics of conventional cigarette smokers with those of novel product users., RESULTS: A stall in e-cigarette use at around 2.4% and a three-fold increase in HTP use from 0.5% in 2018 to 2.5% in 2021 were recorded, with around 60% of e-cigarette users and 70% of HTP users who kept on smoking conventional cigarettes. Around 86% of smokers did not use novel products at all. Novel products use among former smokers was more likely in younger e-cigarette with no nicotine users, whereas older users of both novel products were less able to completely shift to an exclusive use., CONCLUSIONS: After 10 years from the introduction of e-cigarettes and 5 years from that of HTP, the majority of smokers in Italy were still loyal to conventional tobacco cigarettes, and more than half of novel product users kept on smoking conventional cigarettes.
8024	Casseus, Myriam, Cooney, James M., Wackowski, Olivia A. (2022) Tobacco Use, Dependence, and Age of Initiation among Youths with Cognitive Disability. The Journal of pediatrics; 247<br><br>**Keywords:**<br>Adolescent Cognition Cross-Sectional Studies *Electronic Nicotine Delivery Systems Humans *Tobacco Products Tobacco Use/ep [Epidemiology] Tobacco Use Disorder/ep [Epidemiology] *Tobacco Use Disorder United States<br><br>**Abstract:** OBJECTIVES: To examine the prevalence of tobacco use by product type among youths with cognitive disability; the prevalence of tobacco dependence among youths with cognitive disability; and the relationship between age of tobacco use initiation and cognitive disability., STUDY DESIGN: This cross-sectional study analyzed data from the 2019 National Youth Tobacco Survey (NYTS). Participants were a nationally representative sample of 19 018 students in grades 6-12. Estimates were calculated for ever use, current use, age of tobacco use initiation, and tobacco dependence. Associations between use patterns and cognitive disability status were examined using bivariate analyses and multivariable logistic regression., RESULTS: Compared with youths without cognitive disability, youths with cognitive disability had significantly greater odds of ever using any tobacco product (aOR, 1.49; 95% CI, 1.31-1.70), currently using any tobacco product (aOR, 1.41; 95% CI, 1.26-1.58), and currently using electronic cigarettes (e-cigarettes), cigarettes, cigars, hookahs, roll-your-own cigarettes, and heated tobacco products, specifically. They had higher prevalence and odds of reporting younger age of tobacco use initiation (aOR, 1.25; 95% CI, 1.10-1.43). Higher prevalence and odds of tobacco dependence were also observed among youths with cognitive disability compared with youths without cognitive disability (P < .001)., CONCLUSIONS: These findings reinforce the importance of developing early primary prevention efforts to reduce or delay tobacco use among adolescents with cognitive disability. They also suggest the need to address co-occurring disorders during tobacco cessation programs with this high-risk group. Copyright © 2022 Elsevier Inc. All rights reserved.
8034	Cerrai, Sonia, Benedetti, Elisa, Colasante, Emanuela, Scalese, Marco, Gorini, Giuseppe, Gallus, Silvano, Molinaro, Sabrina (2022) E-cigarette use and conventional cigarette smoking among European students: findings from the 2019 ESPAD survey. Addiction (Abingdon, England); 117<br><br>**Keywords:**<br>Adolescent Cigarette Smoking/ep [Epidemiology] *Cigarette Smoking Cross-Sectional Studies *Electronic Nicotine Delivery Systems Female Humans Male Students Surveys and Questionnaires Vaping/ep [Epidemiology] *Vaping<br><br>**Abstract:** BACKGROUND AND AIMS: Representative data on e-cigarette use among European adolescents are scant. This study reports current vaping and tobacco smoking individual and country-specific correlates among European students., DESIGN: Cross-sectional survey: 2019 European School Survey Project on Alcohol and Other Drugs (ESPAD) collecting data on risk behaviours on a representative sample of 16-year-old students., SETTING: A total of 35 European countries, 25-30 with Tobacco Control Scale (TCS) and TCS components PARTICIPANTS: A total of 99 648 students (49.1% males) turning 16

	years in the survey year., MEASUREMENTS: Data on current cigarette and e-cigarette use were gathered through a self-administered questionnaire which also collected socio-demographics and individual and family characteristics. ESPAD data were integrated with country-level data on TCS and selected TCS parameters to assess their association with the prevalence of current cigarette and e-cigarette use., FINDINGS: Of the 99 648 participating students, 12.4% were current e-cigarette users, from 5.5% in Serbia to 41.4% in Monaco; 19.3% current smokers, from 5.1% in Iceland to 32.4% in Italy. Compared with non-users, current e-cigarette users less frequently came from an average well-off family [odds ratio (OR) = 0.85, 95% confidence interval (CI) = 0.80-0.90] and lived in countries with higher cigarette prices (OR = 0.71, 95% CI = 0.50-0.99), restrictive measures on tobacco advertising and promotion (OR = 0.79, 95% CI = 0.63-0.99) and intermediate implementation of tobacco control measures (OR = 0.57, 95% CI = 0.38-0.85). Current vapers were more frequently life ever smokers (OR = 7.31, 95% CI = 6.47-8.25), were early smokers (OR = 4.35, 95% CI = 3.66-5.17), males (OR = 1.61, 95% CI = 1.55-1.67), from non-traditional families (OR = 1.43, 95% CI = 1.34-1.53), with relatively low parental education (OR = 1.15, 95% CI = 1.10-1.20). Compared with non-smokers, current smokers had similar family characteristics to vapers, and were less likely to live in countries with higher cigarette prices (OR = 0.70, 95% CI = 0.49-0.99) and higher spending on public anti-tobacco media campaigns (OR = 0.23, 95% CI = 0.10-0.50)., CONCLUSIONS: E-cigarette use among European adolescents is associated with weaker tobacco control measures, particularly on tobacco price, advertising and promotion. Besides preventing tobacco smoking, the adoption of governmental tobacco control policies in European countries also seems to contribute to the prevention of vaping among adolescents. Copyright © 2022 Society for the Study of Addiction.
8054	Chang, Joanne T., Mayer, Margaret, Jackson, Rebecca A., Rostron, Brian L., Coleman, Blair, Lee, Terrence, Ambrose, Bridget K., Chang, Cindy M. (2023) Characteristics and Patterns of Cigarette Smoking and Vaping By Past-Year Smokers Who Reported Using Electronic Nicotine Delivery System to Help Quit Smoking in the Past Year: Findings From the 2018-2019 Tobacco Use Supplement to the Current Population Survey. Nicotine & tobacco research : official journal of the Society for Research on Nicotine and Tobacco; 25<br><br>**Keywords:**<br>Adult Humans United States/ep [Epidemiology] Cigarette Smoking/ep [Epidemiology] *Cigarette Smoking Smokers Tobacco Smoking Cessation/mt [Methods] *Smoking Cessation *Electronic Nicotine Delivery Systems Vaping/ep [Epidemiology] *Vaping Prospective Studies Menthol Surveys and Questionnaires *Tobacco Products<br><br>**Abstract:** INTRODUCTION: Cigarette smoking has declined, while electronic nicotine delivery system (ENDS) use has increased in the United States. Understanding the role of ENDS in adult smoking quit attempts and cessation is important for estimating their population health impact., AIMS AND METHODS: We used data from 2018 to 2019 tobacco use supplement to the current population survey to examine demographic characteristics and ENDS use patterns among adult ENDS users who reported quitting smoking in the past year by trying to switch to ENDS ("switchers") and smokers who did or did not make a quit attempt in the past year. chi2 tests of proportions and t-tests were used to compare characteristics between groups., RESULTS: In 2018-2019, about three-quarters of switchers reported daily use of ENDS compared to only one-third of dual users with a recent quit attempt by trying to switch to ENDS. Compared to dual users who made a quit attempt by trying to switch to ENDS, switchers were more likely to use menthol/mint-flavored ENDS exclusively (5.6% vs. 13.1%; p = .004) but less likely to use tobacco-flavored ENDS exclusively (21.2% vs. 13.7%; p = .01)., CONCLUSIONS: ENDS users who quit smoking in the past year and reported trying to quit by switching to ENDS were more likely to use menthol/mint flavors exclusively and use ENDS daily compared to dual users who made a quit attempt by trying to switch to ENDS. Longer-term prospective data may better clarify the role of ENDS in smoking quit attempts and cessation., IMPLICATIONS: This study provides information on patterns of ENDS use in former smokers and current smokers who tried to quit smoking by switching to ENDS in a national sample of U.S. adults.

	These results can inform policy actions concerning ENDS products. Copyright Published by Oxford University Press on behalf of the Society for Research on Nicotine and Tobacco 2022.
8077	Chen, Ruifeng, Pierce, John P., Leas, Eric C., Benmarhnia, Tarik, Strong, David R., White, Martha M., Stone, Matthew, Trinidad, Dennis R., McMenamin, Sara B., Messer, Karen (2022) Effectiveness of e-cigarettes as aids for smoking cessation: evidence from the PATH Study cohort, 2017-2019. Tobacco control;<br><br>**Keywords:**<br><br><br>**Abstract:** OBJECTIVE: To assess the effectiveness of e-cigarettes in smoking cessation in the USA from 2017 to 2019, given the 2017 increase in high nicotine e-cigarette sales., METHODS: In 2017, the PATH Cohort Study included data on 3578 previous year smokers with a recent quit attempt and 1323 recent former smokers. Respondents reported e-cigarettes or other products used to quit cigarettes and many covariates associated with e-cigarette use. Study outcomes were 12+ months of cigarette abstinence and tobacco abstinence in 2019. We report weighted unadjusted estimates and use propensity score matched analyses with 1500 bootstrap samples to estimate adjusted risk differences (aRD)., RESULTS: In 2017, 12.6% (95% CI 11.3% to 13.9%) of recent quit attempters used e-cigarettes to help with their quit attempt, a decline from previous years. Cigarette abstinence for e-cigarette users (9.9%, 95% CI 6.6% to 13.2%) was lower than for no product use (18.6%, 95% CI 16.0% to 21.2%), and the aRD for e-cigarettes versus pharmaceutical aids was -7.3% (95% CI -14.4 to -0.4) and for e-cigarettes versus any other method was -7.7% (95% CI -12.2 to -3.2). Only 2.2% (95% CI 0.0% to 4.4%) of recent former smokers switched to a high nicotine e-cigarette. Subjects who switched to e-cigarettes appeared to have a higher relapse rate than those who did not switch to e-cigarettes or other tobacco, although the difference was not statistically significant., CONCLUSIONS: Sales increases in high nicotine e-cigarettes in 2017 did not translate to more smokers using these e-cigarettes to quit smoking. On average, using e-cigarettes for cessation in 2017 did not improve successful quitting or prevent relapse. Copyright © Author(s) (or their employer(s)) 2022. Re-use permitted under CC BY-NC. No commercial re-use. See rights and permissions. Published by BMJ.
8087	Chen-Sankey, Julia, Bover-Manderski, Michelle T. (2022) Importance of a Balanced Public Health Approach When Assessing Recent Patterns in the Prevalence of Adult e-Cigarette Use in the US. JAMA network open; 5<br><br>**Keywords:**<br>Adult *Electronic Nicotine Delivery Systems Humans Prevalence Public Health Smoking/ep [Epidemiology] Vaping/ep [Epidemiology] *Vaping<br><br>**Abstract:**
8092	Chen-Sankey, Julia, Lopez, Michael, Elhabashy, Maryam, Broun, Aaron (2023) How the Other Half Sees It: E-cigarette Advertising Exposure and Subsequent E-cigarette Use Among U.S. Young Adult Cigarette Smokers. Nicotine & tobacco research : official journal of the Society for Research on Nicotine and Tobacco; 25<br><br>**Keywords:**<br>Humans Young Adult Smokers *Electronic Nicotine Delivery Systems Advertising Vaping/ep [Epidemiology] *Vaping *Smoking Cessation *Tobacco Products Tobacco<br><br>**Abstract:** INTRODUCTION: E-cigarette advertising may benefit young adult cigarette smokers in transitioning to using e-cigarettes. We assessed whether e-cigarette advertising exposure was associated with subsequent e-cigarette use among young adult cigarette smokers., AIMS AND METHODS: Data were from Waves 4 (2016-2018) and 5 (2018-2019) of the nationally representative Population Assessment of

	Tobacco and Health (PATH) study adult survey. Respondents were young adult established cigarette smokers at Wave 4 (18-34 years; n = 3391) and a subsample of those who tried to quit smoking cigarettes completely in the past year at Wave 5 (n = 1235). Multivariable logistic regressions were used to examine the associations between e-cigarette advertising exposure (by channel of exposure) and subsequent past-year e-cigarette use in general and e-cigarette use to quit smoking cigarettes, controlling for covariates., RESULTS: At Wave 5, 43.4% of smokers reported past-year use of e-cigarettes; and 14.8% of smokers who tried to completely quit smoking reported past-year use of e-cigarettes to quit. E-cigarette advertising exposure was associated with subsequent past-year e-cigarette use (adjusted odds ratio [AOR] = 1.53, p < .0001, 95% confidence interval [CI] = 1.27, 1.86) and past-year use to quit smoking cigarettes (AOR = 1.65, p < .01, 95% CI = 1.19, 2.29). Advertising exposure through brick-and-mortar stores or websites/social media was similarly associated with both e-cigarette use behaviors., DISCUSSION: Exposure to e-cigarette advertising among U.S. young adult established cigarette smokers may be associated with subsequent e-cigarette use and use to quit smoking. More research is needed to understand the features of e-cigarette advertising (eg, discounts, flavors, smoker-targeted claims) that may shape perception and behavior related to e-cigarette use among young adult smokers., IMPLICATIONS: Little is known about the associations between e-cigarette advertising exposure and e-cigarette use among young adult cigarette smokers who may benefit from switching to e-cigarettes. This study found that e-cigarette advertising exposure was positively associated with (1) subsequent e-cigarette use among U.S. young adult established cigarette smokers and (2) subsequent e-cigarette use to quit smoking cigarettes among those who tried to completely quit in the past year. These observed associations were driven by smokers who did not currently use e-cigarettes at baseline. E-cigarette advertising exposure through brick-and-mortar stores or websites/social media was also positively associated with subsequent e-cigarette use behaviors. Copyright © The Author(s) 2022. Published by Oxford University Press on behalf of the Society for Research on Nicotine and Tobacco. All rights reserved. For permissions, please e-mail: journals.permissions@oup.com.
8113	Chulasai, Phantara, Chinwong, Surarong, Vientong, Purida, Hall, John J., Chinwong, Dujrudee (2022) Electronic Cigarette Use and Other Factors Associated with Cigarette Smoking among Thai Undergraduate Students. Healthcare (Basel, Switzerland); 10<br><br>**Keywords:**<br><br><br>**Abstract:** The prevalence of smoking among young adults in Thailand has gradually increased. Therefore, this study aimed to identify factors associated with cigarette smoking among undergraduate students. This cross-sectional study used a self-administered, anonymous online questionnaire to gather data from undergraduate students across four universities in Chiang Mai Province, Thailand. All 1126 participants were an average age of 21.30 years old (SD 1.48). The findings revealed seven factors significantly associated with cigarette smoking (p < 0.05), including male sex, having no medical conditions, consuming alcohol daily and consuming alcohol in the past, having brothers or sisters who smoked cigarettes, having a father or mother who smoked cigarettes, having parents who considered smoking acceptable and having parents who had uncertain concerns about smoking, and had or have used electronic cigarettes (e-cigarettes). These associated factors could be useful in implementing appropriate tobacco-control programs to prevent cigarette smoking among undergraduate students. Relevant organizations, universities and healthcare professionals should communicate correct and appropriate information about the illness and diseases caused by using tobacco products to strengthen the correct perceptions of the harms of cigarette smoking and e-cigarette use among undergraduate students. Furthermore, smoke-free policies should be monitored and strictly enforced, particularly in university areas.
8118	Cichonska, D., Maj, A., Ciesielska, A., Ossowska, A., Suligowska, K., Kusiak, A. (2022) Analysis of electronic cigarettes usage and traditional cigarettes smoking among 14 years old adolescents in Poland. Journal of Clinical Periodontology; 49

	**Keywords:**<br>adolescent *cigarette smoking conference abstract *electronic cigarette female health human major clinical study male *Poland primary school questionnaire smoking tobacco nicotine<br><br>**Abstract:** Background and Aim: Electronic cigarettes are nicotine delivery devices, which are nowadays gaining more popularity among not only traditional cigarettes smokers, but also people who have never smoked before, including young adults and adolescents. This study reports the analysis of electronic cigarettes usage and traditional cigarettes smoking among 14 years old adolescents in Poland and include intention to use electronic cigarettes and smoke traditional cigarettes in future. Method(s): The data collection took place in October and November 2021. The study included 178 students of age 14 from 8th grade of primary schools in Northern Poland, who were asked to answer seven questions concerning usage of electronic cigarettes and traditional cigarettes smoking. Result(s): Among the adolescents responding to our questionnaire traditional cigarettes smoking and electronic cigarettes usage have been declared by 17 (9.55%) participants. Only electronic cigarettes have been used by 6 (3.37%) participants. Smoking only traditional cigarettes has not been reported. Contact with both types of cigarettes have been declared by 11 (6.18%) adolescents. Among the adolescents who have never had contact with nicotine the willingness to future traditional cigarettes smoking and electronic cigarettes usage have been declared by 5 (2.81%) of them. Among the participants who have had contact with nicotine the willingness to future traditional cigarettes smoking and electronic cigarettes usage have been declared by 6 (35.29%) of them. The 96.7% of adolescents responding to our questionnaire are aware of the adverse effects of smoking on oral health. Conclusion(s): The usage of e-cigarettes and traditional cigarettes smoking is a problem that affects adolescents in Poland. Adolescents who have already had experience with electronic cigarettes or tobacco more often declare their willingness to use them in the future.
8126	Coats, Ellen M., Farrelly, Matthew C., Henes, Amy L., Pikowski, Jessica M., Brown, Elizabeth M., Nonnemaker, James M. (2022) Youth vaping beliefs and behaviors: evidence from New York. Health education research; 37<br><br>**Keywords:**<br>Adolescent *Cannabis Cross-Sectional Studies *Electronic Nicotine Delivery Systems Flavoring Agents Humans New York *Tobacco Products United States *Vaping<br><br>**Abstract:** Current use of vaping products has increased in recent years among youth in the United States. We conducted cross-sectional surveys of vaping product users aged 15-17 in New York in 2017 and 2019 to assess vaping freqency, reported nicotine content of vaping products used, risk perceptions of vaping and openness to vaping cannabis (2019 survey only). Between 2017 and 2019, the proportion of youth vapers who were frequent vaping product users increased from 16.8% to 26.2% (P < 0.05). The proportion of youth vapers who usually used high-nicotine vaping products also increased, from 12.6% to 40.0% (P < 0.05). In both years, the use of high-nicotine vaping products was positively associated with frequent use (P < 0.05). The perceived likelihood of harm from vaping increased (P < 0.05), but respondents' perception of harm from using tobacco-flavored vaping products remained higher than that from using menthol/mint or sweet flavors. In 2019, 60.6% of respondents reported having tried vaping cannabis. Results suggest shifts in youth vaping behavior toward more frequent use and use of higher nicotine vaping products, support previous findings about youth misperceptions about health risks of flavored vaping products and highlight openness to vaping cannabis among youth vaping product users.<br>Copyright © The Author(s) 2022. Published by Oxford University Press. All rights reserved. For permissions, please e-mail: journals.permissions@oup.com.
8151	Cooper, Maria, Day, Hannah R., Ren, Chunfeng, Oniyide, Olusola, Corey, Catherine G., Ambrose, Bridget K., Michael Cummings, K., Sargent, James, Niaura, Ray, Pierce, John P., Kaufman, Annette, Choi, Kelvin, Goniewicz, Maciej L., Stanton, Cassandra A., Villanti, Andrea, Kasza, Karin, Bansal-Travers, Maansi, Silveira, Marushka L., Kimmel, Heather L., Hull, Lynn C., Koblitz, Amber, Poonai, Karl, Paredes, Antonio,

	Taylor, Kristie, Borek, Nicolette, Hyland, Andrew J. (2022) Correlates of tobacco product initiation among youth and young adults between waves 1-4 of the population assessment of tobacco and Health (PATH) study (2013-2018). Addictive behaviors; 134<br><br>**Keywords:**<br><br>**Abstract:** INTRODUCTION: While risk factors for cigarette smoking among youth and young adults are well-documented, less is known about the correlates of initiation of other tobacco products. This study aims to provide estimates and correlates of initiation among U.S. youth and young adults., METHODS: Data on youth aged 12-17 (n = 10,072) and young adults aged 18-24 (N = 5,727) who provided information on cigarettes, electronic nicotine delivery systems (ENDS), cigars, pipe, hookah and smokeless tobacco use in Wave 1 (W1: 2013-2014)-Wave 4 (W4: 2016-2018) of the nationally-representative PATH Study were used to calculate ever use initiation and correlates of initiation by W4., RESULTS: Nearly 6 million youth and 2.5 million young adults used tobacco for the first time between W1-W4. Approximately one quarter of youth and young adult ENDS never users initiated ENDS between W1-W4 of the PATH Study. Among youth, use of other tobacco products, ever substance use, and high externalizing problems were associated with initiation of most products. Among young adults, use of other tobacco products and ever substance use were associated with initiation of most products. In both youth and young adults, Hispanics were more likely to initiate hookah use than their non-Hispanic White counterparts. While male sex was a risk factor for most tobacco product initiation across both age groups, it was not associated with hookah initiation., CONCLUSIONS: Cigarette and non-cigarette products shared many correlates of initiation, although there are noteworthy demographic differences. Findings can help tailor product specific interventions to reach populations at risk during preliminary stages of use. Copyright Published by Elsevier Ltd.
8155	Copp, Sebastian R., Wilson, Maria N., Asbridge, Mark (2022) Smoking Susceptibility in Canadian Adolescent Electronic-Cigarette Users. Substance use & misuse; 57<br><br>**Keywords:**<br>Adolescent Canada/ep [Epidemiology] *Electronic Nicotine Delivery Systems Electronics Humans Smoking/ep [Epidemiology] *Substance-Related Disorders Tobacco *Tobacco Products<br><br>**Abstract:** Background: E-cigarette use is increasing among Canadian youth, with experimentation especially prevalent among never-smoking youth. Among this group, there is concern e-cigarette use contributes to future initiation of smoking through a gateway effect. However, e-cigarette use and smoking share many common risk factors; a postulated mechanism to explain the apparent causal pathway from e-cigarette use to smoking initiation in previously smoking-naive youth. A better understanding of the relationships between smoking susceptibility and e-cigarette use among never-smoking youth is needed. Purpose/objectives: The primary aim of this study was to gain a deeper understanding of the risk factors associated with smoking susceptibility in youth who have recently used e-cigarettes. Methods: This study used data (n = 40,363) from the 2018/2019 Canadian Student Tobacco Alcohol and Drug Use Survey (CSTADS) to compare the risk factor profiles of susceptible and non-susceptible never-smoking e-cigarette users, as well as susceptible and non-susceptible never-smoking youth who have never used an e-cigarette. Results: E-cigarette use, independent of susceptibility status, was associated with a sociodemographic and behavioral risk factor profile likely to confer a higher risk of initiating smoking. Among e-cigarette users, smoking susceptibility was associated with more smoking risk factors. Conclusions/importance: Study findings support a common risk-factor model, rather than e-cigarette use itself, to explain differences in the likelihood of smoking initiation among e-cigarette users. E-cigarette use and smoking initiation may be interchangeable outcomes amongst those with smoking risk factors. The risks of e-cigarette use, and their regulatory status, need to be balanced with their potential as harm reduction tools.

8185	Czoli, Christine D., Luongo, Gabriella, Mischki, Trevor (2022) Prevalence trends and factors associated with vaping in Ontario (2015 to 2018) and Quebec (2017 to 2019), Canada. Health reports; 33<br><br>**Keywords:**<br>Adolescent Canada/ep [Epidemiology] *Cannabis Humans Ontario/ep [Epidemiology] Prevalence Quebec/ep [Epidemiology] Vaping/ep [Epidemiology] *Vaping<br><br>**Abstract:** Background: Robust surveillance of vaping product use (with or without nicotine) in Canada has been limited by the use of multiple tools with varying designs and content. The objective of the current study was to examine trends over time in vaping prevalence and to examine associated factors using data from the Canadian Community Health Survey (CCHS)., Data and methods: Trends in the prevalence of past-30-day vaping over time were examined using data available from optional modules of the CCHS for Ontario from 2015 to 2018 and for Quebec from 2017 to 2019. Multiple logistic regression models were used to examine correlates of vaping in Quebec (2019) and Ontario (2018)., Results: Vaping increased in Quebec from 3.4% (233,000) in 2017 to 4.2% (296,000) in 2018 and 4.7% (333,000) in 2019. In Ontario, vaping remained stable in the years 2015 (3.1% or 357,000), 2016 (2.7% or 311,000) and 2017 (2.7% or 309,000), then increased in 2018 (3.4% or 404,000). Increases in vaping in both provinces were driven by youth. Vaping was significantly associated with young age and cigarette smoking in both provinces, as well as with cannabis use in the past 12 months among Quebec respondents., Interpretation: In both provinces, increases in youth vaping were observed in recent years, and this is consistent with national trends. Study findings further our understanding of vaping behaviour and highlight the utility of the CCHS as an additional tool for surveillance of vaping product use among Canadians.
8193	Dai, Hongying, Benowitz, Neal L., Achutan, Chandran, Farazi, Paraskevi A., Degarege, Abraham, Khan, Ali S. (2022) Exposure to Toxicants Associated With Use and Transitions Between Cigarettes, e-Cigarettes, and No Tobacco. JAMA network open; 5<br><br>**Keywords:**<br>Adolescent Adult Aged Aged, 80 and over *Biomarkers/ur [Urine] *Carcinogens/to [Toxicity] Cohort Studies *Electronic Nicotine Delivery Systems/sn [Statistics & Numerical Data] Female Humans Inhalation Exposure Longitudinal Studies Male Middle Aged *Nicotine/ur [Urine] *Non-Smokers/sn [Statistics & Numerical Data] *Smokers/sn [Statistics & Numerical Data] *Tobacco Use/ae [Adverse Effects] United States Young Adult<br><br>**Abstract:** Importance: Transitions between e-cigarettes and cigarettes are common among tobacco users, but empirical evidence on the health outcomes of switching tobacco products is scarce., Objectives: To examine changes in urinary biomarkers between baseline and 1-year follow-up among adult tobacco users switching between e-cigarettes and cigarettes., Design, Setting, and Participants: This cohort study used data from wave 1 (baseline, September 2013 to December 2014) and wave 2 (1-year follow-up, October 2014 to October 2015) of the Population Assessment of Tobacco and Health Study. A subset of the probability sample of US adults who voluntarily provided biospecimens at 2 waves was analyzed. Participants were divided into 3 mutually exclusive groups at baseline: exclusive cigarette smokers, exclusive e-cigarette users, and dual users. Data analysis was performed in 2021., Exposures: Harmful and potentially harmful constituents included nicotine metabolites, tobacco-specific nitrosamines (TSNAs; including 4-(methylnitrosamino)-1-(3-pyridyl)-1-butanol [NNAL]), metals, polycyclic aromatic hydrocarbons (PAHs), and volatile organic compounds (VOCs)., Main Outcomes and Measures: Within-participant changes in 55 urinary biomarkers of exposure (BOEs) to harmful and potentially harmful constituents were examined using multivariable regression models., Results: Among 3211 participants (55.6% women, 68.3% White, 13.2% Black, and 11.8% Hispanic) at baseline, 21.9% of exclusive cigarette users, 42.8% of exclusive e-cigarette users, and 62.1% of dual users changed product use at follow-up (all percentages are weighted). There was a significant reduction in urine concentrations of TSNAs, PAHs, and VOCs when users transitioned from exclusive cigarette to exclusive e-cigarette use,

	with a 92% decrease in NNAL, from a mean of 168.4 pg/mg creatinine (95% CI, 102.3-277.1 pg/mg creatinine) to 12.9 pg/mg creatinine (95% CI, 6.4-25.7 pg/mg creatinine; P < .001). A similar panel of BOEs decreased when dual users transitioned to exclusive e-cigarette use; NNAL levels decreased by 96%, from a mean of 143.4 pg/mg creatinine (95% CI, 86.7-237.0 pg/mg creatinine) to 6.3 pg/mg creatinine (95% CI, 3.5-11.4 pg/mg creatinine; P < .001). Nicotine metabolites, TSNAs, PAHs, and VOCs significantly increased when baseline exclusive e-cigarette users transitioned to exclusive cigarette use or dual use. Switching from exclusive cigarette use to dual use was not associated with significant decreases in BOEs., Conclusions and Relevance: This national cohort study provides evidence on the potential harm reduction associated with transitioning from exclusive cigarette use or dual use to exclusive e-cigarette use. e-Cigarettes tend to supplement cigarettes through dual use instead of cessation at the population level. Continuous monitoring of BOE at the population level and assessment of BOE change by product transition are warranted, as well as defined adverse health outcomes.
8196	Dai, Hongying Daisy, Leventhal, Adam (2022) Changes in electronic cigarette use among U.S. Adults by cigarette smoking status, socciodemographics, and subjective depression, 2019-2020. Preventive medicine reports; 30<br><br>**Keywords:**<br><br>**Abstract:** E-cigarette use increased from 2017 to 2019 and then declined in 2020 among U.S. adults. It is important to understand whether the decline differed by smoking status and sociodemographic groups and whether daily e-cigarette use has subsequently changed. This study estimated e-cigarette use prevalence in 2019 and 2020 by analyzing the pooled National Health Interview Survey (n = 63,565) in 2022. Multivariable logistics regressions were performed to examine biannual change in current and daily e-cigarette use, overall and stratified by cigarette smoking status, sociodemographic factors, and subjective depression frequency. Among US adults, change in current e-cigarette use during 2019-2020 differed by smoking status (cigarette smoking status x year, p =.01) and race/ethnicity (race/ethnicity x year, p =.03). Current e-cigarette use prevalence decreased among current cigarette smokers (11.7 % to 8.0 %, p = 0.0001) but not among former or never smokers, and among Non-Hispanic White adults (5.1 % to 4.2 %, p = 0.04) and Non-Hispanic Black adults (3.4 % to 1.6 %, p = 0.0003) but not among other racial and ethnic groups. Current e-cigarette use also declined among those 25-64 years old but not among young adults 18-24 years old. Daily e-cigarette use did not significantly change during 2019-2020 in the overall sample (2.3 % to 2.0 %, p = 0.10), but declined among adults who were Non-Hispanic Black (2.4 % to 0.9 %, p = 0.0007), aged 35-44 years, and lived below the federal poverty level. Adults with monthly depressive episodes (vs without) continued to have a higher prevalence of current (7.0 % vs 3.0 %) and daily e-cigarette use (3.6 % vs 1.6 %) in 2020. Continuous monitoring of cross-population differences in adult e-cigarette use is warranted. Copyright © 2022 Published by Elsevier Inc.
8239	Delnevo, Cristine D. (2023) e-Cigarette and Cigarette Use Among Youth: Gateway or Common Liability?. JAMA network open; 6<br><br>**Keywords:**<br>Humans Adolescent *Electronic Nicotine Delivery Systems Cigarette Smoking/ep [Epidemiology] *Cigarette Smoking Vaping/ep [Epidemiology] *Vaping *Tobacco Products<br><br>**Abstract:**
8269	Do, Elizabeth K., Tulsiani, Shreya, Vallone, Donna M., Hair, Elizabeth C. (2022) Transitions in Frequent to Daily Tobacco and Nicotine Use among Youth and Young Adults. Substance use & misuse; 57<br><br>**Keywords:**<br>Adolescent *Electronic Nicotine Delivery Systems Humans Nicotine Tobacco *Tobacco Products Tobacco

	Use/ep [Epidemiology] Tobacco Use Disorder/ep [Epidemiology] *Tobacco Use Disorder Young Adult<br><br>**Abstract:** Objective: To examine use and frequency patterns across e-cigarettes, cigarettes, and little cigars, cigarillos, and cigars (LCCs) over time. Methods: Data were obtained from the Truth Longitudinal Cohort (TLC), a nationally representative longitudinal cohort of youth and young adults. Latent class analysis was conducted to classify participants (n = 5274) into subgroups based upon frequency of use of cigarettes, e-cigarettes, and little cigars, cigarillos, and cigars (LCCs) in the past 30 days. Latent transition analysis was used to estimate the probability of use pattern transitions across 23 months (February 2018 to December 2019), adjusted for the effects of gender, race/ethnicity, financial situation, sensation seeking, parent education, and household smoking. Findings: Findings reveal four groups of tobacco product users: (1) frequent to daily cigarette users (9%), (2) frequent to daily cigarette and LCC users (3%), (3) frequent to daily e-cigarette users (10%), and (4) former or noncurrent tobacco users (78%). Although most respondents (69-94%) retained their initial user patterns during the observation period, results also indicate shifts between user groups. Notably, 14% of frequent to daily cigarette and LCC users transitioned to frequent to daily cigarette use, while 6% of frequent to daily cigarette and LCC users, 9% of frequent to daily cigarette users, and 4% of former or noncurrent tobacco users transitioned to frequent to daily e-cigarette use. Conclusion: Although most frequent to daily tobacco users stay with their primary product, there are transitions between frequent to daily cigarette, e-cigarette, and LCC use. Transition patterns may influence risk for nicotine addiction among youth and young adults. Thus, policies focused on preventing and reducing all tobacco use are needed to curb the risk of nicotine addiction among youth and young adults.
8274	Dobbs, Page D., Hodges, Elise J., Dunlap, Chris M., Cheney, Marshall K. (2022) Potential risk factors for cigarette use among a sample of college JUUL users. Journal of American college health : J of ACH; 70<br><br>**Keywords:**<br>Cross-Sectional Studies *Electronic Nicotine Delivery Systems Humans Risk Factors Students *Tobacco Products Universities *Vaping<br><br>**Abstract:** Objective: To examine the relationship between demographics, other tobacco use, and JUUL dependency on combustible cigarette use among college JUUL users. Participants: Undergraduates (n = 595) at a large southwestern university who used JUUL weekly completed a cross-sectional online survey in March 2019. Methods: Logistic regressions examined associations between covariates and ever use/past 30-day use of cigarettes. Results: As age increased, odds of ever trying a cigarette increased (AOR = 1.87; 95% CI = 1.45-2.41); however, as age of JUUL initiation increased, odds of ever (AOR = 0.80; 95% CI = 0.65-0.99) or past 30-day (AOR = 0.78; 95% CI = 0.63-0.97) cigarette use decreased. Those moderately (AOR = 2.03; 95% CI = 1.07-3.82) or highly (AOR = 8.01; 95% CI = 3.08-20.83) dependent on JUUL were more likely to have tried cigarettes than those not dependent. However, dependence was not associated with past 30-day use. Conclusions: JUUL users may not transition to combustible cigarettes during college. Longitudinal studies are needed to examine transitions in JUUL users' tobacco product use after college.
8294	Du, A., Mittal, A., Merz, W. W., Myers, M., Crotty Alexander, L. E., Doran, N. (2022) Impulsivity-Related Personality Traits as Predictors of E-cigarette Use Among Young Adults Over Time. American Journal of Respiratory and Critical Care Medicine; 205<br><br>**Keywords:**<br>adult alcohol consumption cigarette smoking conference abstract controlled study *electronic cigarette female human *impulsiveness incidence major clinical study male *personality public health sensation seeking vaping young adult cannabis<br><br>**Abstract:** Introduction: E-cigarette use among teenagers and young adults has been a growing concern as research about the adverse health effects of e-cigarettes increases. It is important to identify factors that

	may increase risk of vaping initiation and frequency of use in order identify possible intervention targets for public health. One potential risk factor known to increase risk for other substance use is impulsivity. This study tested the hypothesis that impulsivity prospectively predicts vaping over time. Method(s): Active e-cigarette users (n=137; 51.8% male; Mean age 20 years at baseline) completed 8 waves of assessment over 21 months (2018-2020). The short form of the UPPS-P scale was used at baseline to measure impulsivity. Frequency of e-cigarette, cigarette, marijuana and alcohol use was calculated at each wave. Result(s): We observed positive associations between e-cigarette use and lack of premeditation [Incidence Rate Ratio (IRR)=1.06] and sensation seeking (IRR=1.09), ps < .05. Vaping frequency was inversely associated with negative urgency (IRR=0.95, p < .05). Positive urgency and lack of perseverance were not associated with frequency of vaping. Conclusion(s): As hypothesized, the participants with higher sensation seeking and lack of premeditation at baseline used e-cigarettes more frequently across 8 waves of assessment over 21 months. Unexpectedly, e-cigarette use was unrelated to positive urgency and was inversely associated with negative urgency. Results are consistent with prior literature that has shown cigarette use to be associated with sensation seeking, premeditation, and urgency while ecigarette use has been associated with sensation seeking and premeditation, with less relation to urgency. Our findings suggest that young adults higher in certain aspects of impulsivity may be at risk for more frequent e-cigarette use. Thus, vaping interventions for young adults should address these factors to ensure the greatest impact on public health.
8298	Duan, Zongshuan, Wang, Yu, Spears, Claire A., Self-Brown, Shannon R., Weaver, Scott R., Zheng, Pinpin, Eriksen, Michael P., Huang, Jidong (2022) Role of Mental Health in the Association Between E-Cigarettes and Cannabis Use. American journal of preventive medicine; 62<br><br>**Keywords:**<br>Adolescent *Cannabis Child *Electronic Nicotine Delivery Systems Humans Longitudinal Studies Mental Health Vaping/ep [Epidemiology] *Vaping<br><br>**Abstract:** INTRODUCTION: E-cigarette use may be associated prospectively with subsequent cannabis use among U.S. adolescents. However, it remains unclear whether this association differs by individual mental health status. This longitudinal study examines effect modifications by mental health status., METHODS: The first 4 waves (2013-2017) of the Population Assessment of Tobacco and Health study were analyzed in 2020. Adolescents (aged 12-17 years) who reported never using cannabis at baseline waves were included. Waves 1-3 were each considered as baseline for their 12-month follow-up waves. Generalized estimating equations were used to evaluate the effect modification of internalizing mental health and externalizing mental health problems on the associations between baseline past 30-day e-cigarette use and past 30-day cannabis use at follow-up, controlling for individual characteristics and state recreational cannabis laws., RESULTS: Baseline e-cigarette use was significantly associated with cannabis use at follow-up (AOR=4.81, 95% CI=2.93, 7.90). Adolescents with high severity of internalizing mental health/externalizing mental health problems were significantly more likely to initiate cannabis use. However, current e-cigarette users who reported high severity of internalizing mental health symptoms were less likely to initiate cannabis use (AOR=2.51, 95% CI=0.92, 6.83) than those who reported low severity of internalizing mental health problems (AOR=8.84, 95% CI=4.19, 18.65). There were no differences by the severity of externalizing mental health problems., CONCLUSIONS: Baseline e-cigarette use and endorsement of severe internalizing mental health/externalizing mental health problems were significantly associated with subsequent cannabis use among U.S. adolescents. Efforts to reduce youth vaping and improve youth mental health could help curb cannabis initiation. Tailored interventions may be warranted for e-cigarette-using adolescents with internalizing mental health problems. Copyright © 2021 American Journal of Preventive Medicine. Published by Elsevier Inc. All rights reserved.
8299	Duan, Zongshuan, Wang, Yu, Weaver, Scott R., Spears, Claire A., Zheng, Pinpin, Self-Brown, Shannon R., Eriksen, Michael P., Huang, Jidong (2022) Effect modification of legalizing recreational cannabis use on the association between e-cigarette use and future cannabis use among US adolescents. Drug and alcohol

	dependence; 233<br><br>**Keywords:**<br>Adolescent Adult Analgesics Cannabinoid Receptor Agonists *Cannabis *Electronic Nicotine Delivery Systems *Hallucinogens Humans Legislation, Drug United States/ep [Epidemiology] Vaping/ep [Epidemiology] *Vaping<br><br>**Abstract:** BACKGROUND: Many states have legalized recreational cannabis use for adults. However, no study has examined how this policy may interact with youth vaping to influence cannabis use among US adolescents. This study investigates whether the association between baseline e-cigarette use and subsequent cannabis use differs by state recreational cannabis legalization status., METHODS: This study analyzed data from the first four waves (2013-2018) of the Population Assessment of Tobacco and Health Study, a nationally representative longitudinal survey. The study sample included adolescents (aged 12-17) who reported never used cannabis at baseline. Generalized estimating equations were used to analyze the effect modification of state recreational cannabis law on the association between baseline e-cigarette use and cannabis use at 12-month follow-up, controlling for individual characteristics., RESULTS: Among adolescents who have never used cannabis at baseline, baseline past-30-day e-cigarette use was significantly associated with past-30-day cannabis use at 12-month follow-up (aOR=5.92, 95% CI: 3.52-9.95). This association was different by state recreational cannabis legalization status, as indicated by the significant interaction term. Subgroup analysis showed that the aOR was 18.39 (95% CI: 4.25-79.68) for adolescents living in states that legalized adult recreational cannabis use and 5.09 (95% CI: 2.86-9.07) for adolescents living in states without such laws., CONCLUSIONS: E-cigarette use is associated with cannabis initiation among youth. This association is stronger among those living in states that legalized adult recreational cannabis use. Further examination of the impact of e-cigarette use on cannabis initiation in relation to state cannabis laws is warranted. Copyright © 2022 Elsevier B.V. All rights reserved.
8314	Dwumfour-Poku, Ismail (2023) The impact of electronic cigarettes use on traditional cigarette uses among U.S. adolescents. Dissertation Abstracts International: Section B: The Sciences and Engineering; 84<br><br>**Keywords:**<br>*High School Students *Nicotine *Tobacco Smoking *Electronic Cigarettes Neighborhoods Public Health Risk Factors<br><br>**Abstract:** The increased usage of e-cigarettes among adolescents in the United States (U.S.) is a major public health concern. According to the Centers for Disease Control and Prevention (CDC), nicotine, an active ingredient in e-cigarettes, is the most addictive drug in use today. The purpose of the current study was to explore the relationship between smoking of e- cigarettes and the risk of smoking traditional cigarettes among middle and high school students in Grades 6 through 12 in the U.S., and to determine if demographic factors contribute to this predisposition. This, quantitative cross-sectional study used the social ecological model as the framework to guide the design. The study used secondary datasets from the 2011 National Youth Tobacco Survey with a sample size of 15,801 of U.S. middle and high school students. Chi-square and logistic regression analysis were used to determine if there was a relationship between e-cigarettes use and traditional cigarettes use. Results indicated a statistically significant relationship between e-cigarettes use and traditional cigarettes (p = .0001), and the odds of smoking traditional cigarettes is 13 times higher for those who smoke e-cigarettes (AOR = 13.384; CI = 11.04-16.263; p = .0001). The potential positive social change impact of this study is a better understanding of youth smoking behavior. This information provides further evidence that can be used towards the development of more effective intervention programs that focus specifically on middle and high school students who smoke e-cigarettes. The results of this study will help stakeholders such as local municipalities and community leaders focus more resources on low socioeconomic and at-risk neighborhoods where improvement is much needed. (PsycInfo Database Record (c) 2022 APA, all rights reserved)

8315	Dyer, Maddy L., Khouja, Jasmine N., Jackson, Abigail R., Havill, Michelle A., Dockrell, Martin J., Munafo, Marcus R., Attwood, Angela S. (2023) Effects of electronic cigarette e-liquid flavouring on cigarette craving. Tobacco control; 32<br><br>**Keywords:**<br>Adult Adolescent Humans *Electronic Nicotine Delivery Systems Nicotine/pd [Pharmacology] Craving *Tobacco Products Smoking Flavoring Agents<br><br>**Abstract:** BACKGROUND: E-liquid flavour restrictions may discourage electronic cigarette (e-cigarette) uptake among youth. However, possible unintended consequences may include reduced appeal and effectiveness of e-cigarettes for smoking cessation. Non-tobacco flavours appear to be important for smoking cessation, but how and why are currently unclear., METHODS: We conducted an experimental study in a UK sample of adult daily smokers using an independent groups design (N=84). Participants were randomised to use an e-cigarette with nicotine-containing fruit/sweet-flavoured e-liquid (blackcurrant, strawberry, vanilla, caramel) or unflavoured e-liquid for 1 week. The primary outcomes were average, peak and cue-elicited cigarette craving (the latter was assessed using a cue exposure task). The secondary outcomes were smoking lapse occurrence, enjoyment of the e-cigarette, ease of transitioning from smoking to using an e-cigarette, intentions to continue using an e-cigarette, intentions and motivation to quit smoking, return to smoking, and continuation of e-cigarette use., RESULTS: E-liquid flavouring did not appear to have an effect on average cigarette craving (b 0.18, 95% CI -0.44 to 0.79, p=0.57), peak cigarette craving (b -0.12, 95% CI -0.59 to 0.35, p=0.62) or cue-elicited cigarette craving (b -0.21, 95% CI -3.86 to 3.43, p=0.91). We did not find evidence of a difference in secondary outcomes., CONCLUSIONS: We did not find evidence to suggest that nicotine-containing fruit/sweet-flavoured and unflavoured e-liquids have different effects on cigarette cravings after 1 week of use. Further research is needed to establish if differences emerge over longer periods of exposure and extend to smoking cessation outcomes. Copyright © Author(s) (or their employer(s)) 2023. Re-use permitted under CC BY. Published by BMJ.
8371	Fearon, Ian M., Seltzer, Ryan G. N., Houser, Trisha L., Tope, Andrew, Cahours, Xavier, Verron, Thomas, Malt, Layla, Nahde, Thomas, O'Connell, Grant, Nides, Mitchell (2023) Curiosity and intentions to use myblu e-cigarettes and an examination of the 'gateway' theory: Data from cross-sectional nationally representative surveys. Drug testing and analysis;<br><br>**Keywords:**<br><br>**Abstract:** Encouraging adult smokers who are uninterested or unwilling to quit, and would otherwise continue to smoke, to transition to potentially less harmful nicotine products such as electronic nicotine delivery systems (ENDS) may positively impact population health. However, counterbalancing this benefit is the societal concern that ENDS may be used by never smokers and youth and serve as a 'gateway' into cigarette smoking. Data were analysed from two independent surveys of the prevalence and perceptions of myblu ENDS use in the United States. Total sample size was 22,232 young adults and 23,264 adults. Being curious to use myblu was 1.6-2.0 times more likely in young adult current smokers than young adult never smokers. This likelihood was 2.8 times greater for adult current smokers compared with adult never smokers in the perceptions survey, while in the prevalence survey, there was no difference between adult current and never smokers. Intentions to use myblu were significantly greater in young adult current smokers compared with young adult never smokers in both surveys and in adults in the prevalence survey. In all surveys and age cohorts, 124 of 45,496 participants (0.1% of the total survey population) reported first using myblu prior to smoking cigarettes and went on to become established smokers. Curiosity and intentions to use myblu were generally higher in current smokers compared with never smokers. There was minimal evidence to suggest the existence of a 'gateway' effect to established

	cigarette smoking among never-smoking myblu users. Copyright © 2023 The Authors. Drug Testing and Analysis published by John Wiley & Sons Ltd.
8402	Folayan, Morenike Oluwatoyin, Alade, Omolola, Adeyemo, Yewande, Sabbagh, Heba Jafar, Oyapero, Afolabi, Oziegbe, Elizabeth O., Popoola, Bamidele Olubukola, Quritum, Maryam, El Tantawi, Maha (2022) Differences in risk indicators associated with electronic cigarette use and tobacco smoking among adolescents and young people in Nigeria. BMJ open respiratory research; 9<br><br>**Keywords:**<br>Adolescent COVID-19/ep [Epidemiology] *COVID-19 *Electronic Nicotine Delivery Systems HIV Infections/ep [Epidemiology] *HIV Infections Humans Male Nigeria/ep [Epidemiology] Risk Factors Smoke Smoking/ep [Epidemiology] Tobacco Smoking/ep [Epidemiology] Vaping/ep [Epidemiology] *Vaping<br><br>**Abstract:** INTRODUCTION: The study determined the proportion of adolescents and young persons (AYP) in Nigeria who use e-cigarettes and smoke tobacco; and identified factors associated with the use of e-cigarettes and tobacco smoking., METHODS: AYP aged 11-23 years were recruited to participate in an online survey. The independent variables were respondents' health, HIV and COVID-19 status and their level of anxiety. The dependent variables were tobacco smoking and use of e-cigarettes. Binary logistic regression was used to determine the associations between the dependent and independent variables after adjusting for confounders (age, sex, educational level and vulnerability status)., RESULTS: There were 2206 respondents of which 568 (19.8%) used e-cigarettes and 787 (27.4%) smoked tobacco. Individual (18-23 years, having a health condition, high anxiety and being vulnerable) and familial (having father, mother, siblings or friends who used e-cigarettes) factors were associated with both the use of e-cigarettes and tobacco smoking. Tobacco smoking was a significant risk indicator for e-cigarettes use and vice versa. COVID-19 infection (adjusted OR, AOR: 3.602) and living with HIV (AOR: 1.898) were associated with higher odds of using e-cigarettes. Males (AOR: 1.577), 15-17 years (AOR: 6.621) and moderate anxiety (AOR: 2.500) were associated with higher odds of tobacco smoking. AYP with health conditions had higher odds of using e-cigarettes (AOR: 1.514) while AYP with moderate anxiety had lower odds of using e-cigarettes (AOR: 0.627)., CONCLUSION: The proportion of AYP in Nigeria who used e-cigarettes and smoked tobacco is high. Public health interventions that target the common risk factors for e-cigarettes use and tobacco smoking among AYP in Nigeria are urgently needed. Copyright © Author(s) (or their employer(s)) 2022. Re-use permitted under CC BY-NC. No commercial re-use. See rights and permissions. Published by BMJ.
8422	Friedman, Abigail S., Pesko, Michael F. (2022) Young adult responses to taxes on cigarettes and electronic nicotine delivery systems. Addiction (Abingdon, England); 117<br><br>**Keywords:**<br>Young Adult Humans United States Adult Adolescent *Electronic Nicotine Delivery Systems Smoking *Tobacco Products Taxes Vaping/ep [Epidemiology] *Vaping<br><br>**Abstract:** BACKGROUND AND AIMS: Although over half of United States states have passed taxes on electronic nicotine delivery systems (ENDS), recent evidence links ENDS tax rates to increases in smoking, suggesting potentially substantive health costs. Overall health implications will depend on how these taxes affect transitions from experimentation to regular smoking and vaping. Current analyses have not assessed ENDS tax rates' effects in young adulthood (ages 18-25). This study measures the relationship between ENDS and cigarette tax rates and ENDS use and smoking in young adulthood, a key period for initiation of regular tobacco use., DESIGN: Observational study of data from the Current Population Survey's 2010-2019 Tobacco Use Supplements., SETTING: The United States., PARTICIPANTS/CASES: A total of 38 906 18 to 25 year-olds MEASUREMENTS: Multivariable linear regressions estimated two-way fixed effects analyses to assess ENDS and cigarette tax rates' relationships to recent and daily smoking and vaping, adjusting for an array of potential sociodemographic and policy confounders along with state and year fixed effects., FINDINGS: A increase in ENDS taxes yielded significant reductions in young adults'

	daily vaping (beta ^ = -0.025; 95% CI, -0.037, -0.014) alongside increases in recent smoking (beta ^ = 0.037; 95% CI, 0.013, 0.061), primarily reflecting greater dual use (beta ^ =2.078; 95% CI, 0.890, 4.852; P = 0.09). A cigarette tax increase yielded 2.1 and 2.5 percentage point increases in recent and daily vaping, with 95% CIs of (0.004, 0.038) and (0.018, 0.032) respectively., CONCLUSIONS: In the United States, higher ENDS tax rates are associated with decreased ENDS use but increased cigarette smoking among 18- to 25-year-olds, with associations reversed for cigarette taxes. Copyright © 2022 The Authors. Addiction published by John Wiley & Sons Ltd on behalf of Society for the Study of Addiction.
8423	Friedman, Abigail S., Warner, Kenneth E. (2022) The E-Cigarette Flavor Debate - Promoting Adolescent and Adult Welfare. The New England journal of medicine; 386<br><br>**Keywords:**<br>Adolescent Adult *Electronic Nicotine Delivery Systems Flavoring Agents Humans *Tobacco Products Vaping/ae [Adverse Effects] *Vaping<br><br>**Abstract:**
8439	Gagne, Thierry, Lee, Joseph G. L., O'Loughlin, Jennifer (2021) Cigarette smoking and E-cigarette use among young adults in the United States: Findings from the 2016-18 behavioral risk factor surveillance system. Addictive behaviors; 113<br><br>**Keywords:**<br>Adolescent Adult Behavioral Risk Factor Surveillance System Cigarette Smoking/ep [Epidemiology] *Cigarette Smoking *Electronic Nicotine Delivery Systems Humans *Smoking Cessation United States/ep [Epidemiology] *Vaping Young Adult<br><br>**Abstract:** Because there are few proven smoking cessation approaches for young adults, it is critical to consider the potential of e-cigarettes as an option. Evidence from 2012 to 2013 in the United States (U.S.) suggested that current e-cigarette use was associated with a higher probability of daily smoking and a lower probability of quitting among young adults. This study examines the associations between e-cigarette use frequency and cigarette smoking in more recent data. Data on e-cigarette use were available for 32,656 current smokers and 9,565 past-year quitters ages 18-34 in the 2016-2018 U.S. Behavioral Risk Factor Surveillance System. Participants were categorized as "daily", "non-daily", "former", or "never" e-cigarette users. Adjusted prevalence ratios were estimated to test the associations between e-cigarette use and each of daily smoking and past-year cessation. With e-cigarette never-users as the reference: 1) daily e-cigarette users were 30% (95%CI 0.61-0.79) less likely to be daily smokers; non-daily and former e-cigarette users were 8% (95%CI 1.02-1.15) and 16% (95%CI 1.11-1.21) more likely to be daily smokers, respectively; 2) daily e-cigarette users were 54% (95%CI 1.38-1.73) more likely to have quit in the past year; non-daily and former e-cigarette users were 50% (95%CI 0.42-0.59) and 21% (95%CI 0.73-0.86) less likely to have quit in the past year, respectively. The findings suggest that non-daily e-cigarette use was associated with greater frequency of smoking and less quitting whereas daily e-cigarette use was associated with a lower frequency of smoking and more quitting among young adults in 2016-18. Copyright © 2020 Elsevier Ltd. All rights reserved.
8450	Gali, Kathleen, Kastaun, Sabrina, Pischke, Claudia R., Kotz, Daniel (2022) Trends and consumption patterns in the use of e-cigarettes among adolescents and young adults in Germany (the DEBRA study). Addictive behaviors; 133<br><br>**Keywords:**<br>Adolescent Cross-Sectional Studies *Electronic Nicotine Delivery Systems Germany/ep [Epidemiology] Humans Male Nicotine Tobacco Vaping/ep [Epidemiology] *Vaping Young Adult<br><br>**Abstract:** INTRODUCTION: Considering the growing popularity and rapid evolution of e-cigarettes, we

	examined e-cigarette use and tobacco smoking trends, and e-cigarette consumption patterns (i.e., device type, nicotine level) among adolescents and young adults in Germany., METHODS: Data from 26 waves of the German Study on Tobacco Use (DEBRA), a repeated cross-sectional nationwide household survey, were used to explore trends in the prevalence of ever e-cigarette use and tobacco smoking in a sample of adolescents (aged 14-17 years, N = 1,396) and young adults (aged 18-24 years, N = 4,685) between June/July 2016 and Aug/Sept 2020. Among current e-cigarette users (N = 208), consumption patterns were examined. Associations with e-cigarette use were analysed using multivariable regression., RESULTS: Adolescent e-cigarette use increased from 9.2% in 2016 to 16.5% in 2017, decreased in 2018 to 8.3% and then gradually increased to 13.4% by 2020. Adolescent tobacco smoking followed a similar trend. Young adult e-cigarette use rates remained relatively stable at 19.1% on average, while tobacco smoking slowly declined (2016: 44.7%, 2020: 38.5%). More than half of current e-cigarette users used refillable-style e-cigarettes (59.4% adolescents; 68.4% young adults) and also smoked tobacco (62.5% adolescents; 79.4% young adults). About 41.6% of adolescents and 56.0% of young adults vaped with nicotine. Among young adults, men (OR = 1.5; 95%CI: 1.3-1.8) and former (OR = 9.6; 95%CI: 1.1-13.1) and current (OR = 10.7; 95%CI: 8.7-13.2) tobacco smokers were more likely to have vaped., CONCLUSION: E-cigarette use continues to rise in German adolescents and young adults requiring targeted health interventions and campaigns aimed at preventing and/or reducing use in this population. Copyright © 2022 Elsevier Ltd. All rights reserved.
8453	Gallegos-Carrillo, Katia, Barrientos-Gutierrez, Inti, Arillo-Santillan, Edna, Rodriguez-Bolanos, Rosibel, Cruz-Jimenez, Lizeth, Desiree, Vidana-Perez, Cho, Yoo Jin, Thrasher, James F. (2022) Transitions between tobacco products: Correlates of changes in cigarette smoking and e-cigarette use among exclusive adult smokers and dual users in Mexico. Preventive medicine reports; 29<br><br>**Keywords:**<br>**Abstract:** This study examined transitions in tobacco products use among Mexican smokers and dual users. Data were analyzed from exclusive smokers (n = 2,946) and dual users (n = 1,643) recruited from an online consumer research panel and surveyed every-four months from November 2018 to April 2021. For exclusive smokers, estimated transitions were (time "t + 1" four months after prior survey): a) remain as exclusive smokers; b) dual use; c) exclusive e-cigarette use or quit both products. Among dual users, transitions analyzed were: a) remain as dual user; b) exclusive smoker; c) exclusive e-cigarette user or quit both products. Multinomial models regressed transitions at "t + 1" on time "t" for control variables. Most exclusive smokers (81%) remained as such, 12.6% transitioned to dual use, 2.3% to exclusive e-cigarette use, and 4% quitted both products. Exclusive smokers were more likely to transition to dual use if they recently attempted to quit (AOR = 1.45) or had partners/family or friends who used e-cigarettes (AOR = 2.47 & 2.56 respectively). Most dual users (74.8%) remained as dual users, 20.4% transitioned to exclusive smoking, 1.6% transitioned to exclusive e-cigarette use, and 3.2% quitted both products. Dual users were more likely to transition to exclusive smoking if they had lower educational attainment, recently attempted to quit e-cigarettes (AOR = 1.70). Having friends who use e-cigarettes (AOR = 0.29) and higher smoking dependence (AOR = 0.55) were associated with a lower likelihood of quitting. Recent quit attempts and e-cigarette use among close social network members may explain the short-term transitions, though longer follow-up is needed to assess sustained smoking cessation. Copyright © 2022 The Authors.
8456	Gallus, Silvano, Stival, Chiara, McKee, Martin, Carreras, Giulia, Gorini, Giuseppe, Odone, Anna, van den Brandt, Piet A., Pacifici, Roberta, Lugo, Alessandra (2022) Impact of electronic cigarette and heated tobacco product on conventional smoking: an Italian prospective cohort study conducted during the COVID-19 pandemic. Tobacco control;<br><br>**Keywords:**<br>**Abstract:** OBJECTIVE: Debate continues about whether electronic cigarettes (e-cigarettes) and heated tobacco products (HTP) reduce or increase the probability of smoking, with many studies compromised by stated or unstated conflicts of interest. We undertook a longitudinal study in Italy., METHODS: 3185

	Italian participants aged 18-74 years provided baseline (April-May) and follow-up (November-December) responses in 2020, reporting smoking status and use of e-cigarettes and HTP. We tracked transitions over that period and reported risk ratios (RR) and corresponding 95% CIs for changes in smoking in relation to baseline use of e-cigarettes and HTPs., RESULTS: Never cigarette smokers who used e-cigarettes at baseline were much more likely to start smoking (compared with never users, RR 8.78; 95% CI: 5.65 to 13.65) and current HTP users (RR 5.80; 95% CI: 3.65 to 9.20). Among ex-smokers, relapse (17.2%) at follow-up was more likely among e-cigarette (RR 4.25; 95% CI: 2.40 to 7.52) and HTP users (RR 3.32; 95% CI: 2.05 to 5.37). Among current smokers at baseline, those who had continued smoking at follow-up were 85.4% overall. These were more frequently current novel product users (compared with non-users, RR 1.10; 95% CI: 1.02 to 1.19 for e-cigarette users; RR 1.17; 95% CI: 1.10 to 1.23 for HTP users)., CONCLUSIONS: Both e-cigarette and HTP use predict starting smoking and relapse, and appear to reduce smoking cessation. Due to the limited sample size within specific strata, the association with quitting smoking should be confirmed by larger prospective studies. These findings do not support the use of e-cigarettes and HTPs in tobacco control as a consumer product, at least in Italy. Copyright © Author(s) (or their employer(s)) 2022. No commercial re-use. See rights and permissions. Published by BMJ.
8469	Gardner, Lauren A., Rowe, Amy-Leigh, Stockings, Emily, Champion, Katrina E., Hides, Leanne, McBride, Nyanda, Allsop, Steve, O'Dean, Siobhan, Sunderland, Matthew, Lee, Yong Yi, Mihalopoulos, Cathy, Freeman, Becky, Leung, Janni, McRobbie, Hayden, Stapinski, Lexine, Lee, Nicole, Thornton, Louise, Debenham, Jennifer, Teesson, Maree, Newton, Nicola C. (2023) Study protocol of the Our Futures Vaping Trial: a cluster randomised controlled trial of a school-based eHealth intervention to prevent e-cigarette use among adolescents. BMC public health; 23<br><br>**Keywords:**<br>Humans Adolescent *Electronic Nicotine Delivery Systems Vaping/pc [Prevention & Control] *Vaping Australia Quality of Life Schools Randomized Controlled Trials as Topic<br><br>**Abstract:** BACKGROUND: Effective and scalable prevention approaches are urgently needed to address the rapidly increasing rates of e-cigarette use among adolescents. School-based eHealth interventions can be an efficient, effective, and economical approach, yet there are none targeting e-cigarettes within Australia. This paper describes the protocol of the OurFutures Vaping Trial which aims to evaluate the efficacy and cost-effectiveness of the first school-based eHealth intervention targeting e-cigarettes in Australia., METHODS: A two-arm cluster randomised controlled trial will be conducted among Year 7 and 8 students (aged 12-14 years) in 42 secondary schools across New South Wales, Western Australia and Queensland, Australia. Using stratified block randomisation, schools will be assigned to either the OurFutures Vaping Program intervention group or an active control group (health education as usual). The intervention consists of four web-based cartoon lessons and accompanying activities delivered during health education over a four-week period. Whilst primarily focused on e-cigarette use, the program simultaneously addresses tobacco cigarette use. Students will complete online self-report surveys at baseline, post-intervention, 6-, 12-, 24-, and 36-months after baseline. The primary outcome is the uptake of e-cigarette use at 12-month follow-up. Secondary outcomes include the uptake of tobacco smoking, frequency/quantity of e-cigarettes use and tobacco smoking, intentions to use e-cigarettes/tobacco cigarettes, knowledge about e-cigarettes/tobacco cigarettes, motives and attitudes relating to e-cigarettes, self-efficacy to resist peer pressure and refuse e-cigarettes, mental health, quality of life, and resource utilisation. Generalized mixed effects regression will investigate whether receiving the intervention reduces the likelihood of primary and secondary outcomes. Cost-effectiveness and the effect on primary and secondary outcomes will also be examined over the longer-term., DISCUSSION: If effective, the intervention will be readily accessible to schools via the OurFutures platform and has the potential to make substantial health and economic impact. Without such intervention, young Australians will be the first generation to use nicotine at higher rates than previous generations, thereby undoing decades of effective tobacco control., TRIAL REGISTRATION: The trial has been prospectively registered

	with the Australian and New Zealand Clinical Trials Registry (ACTRN12623000022662; date registered: 10/01/2023). Copyright © 2023. The Author(s).
8492	Glantz, Stanton, Jeffers, Abra, Winickoff, Jonathan P. (2022) Nicotine Addiction and Intensity of e-Cigarette Use by Adolescents in the US, 2014 to 2021. JAMA network open; 5<br><br>**Keywords:**<br>Adolescent Male Female Humans Vaping/ep [Epidemiology] *Vaping *Electronic Nicotine Delivery Systems Tobacco Use Disorder/ep [Epidemiology] *Tobacco Use Disorder Nicotine Cross-Sectional Studies<br><br>**Abstract:** Importance: As e-cigarettes have become more effective at delivering the addictive drug nicotine, they have become the dominant form of tobacco use by US adolescents., Objective: To measure intensity of use of e-cigarettes, cigarettes, and other tobacco products among US adolescents and their dependence level over time., Design, Setting, and Participants: This survey study analyzed the cross-sectional National Youth Tobacco Surveys from 2014 to 2021. Confirmatory analysis was conducted using Youth Behavioral Risk Factor Surveillance System from 2015 to 2019. The surveys were administered to national probability samples of US students in grades 6 to 12., Exposures: Use of e-cigarettes and other tobacco products before and after the introduction of e-cigarettes delivering high levels of nicotine., Main Outcomes and Measures: First tobacco product used, age at initiation of use, intensity of use (days per month), and nicotine addiction (measured as time after waking to first use of any tobacco product)., Results: A total of 151 573 respondents were included in the analysis (51.1% male and 48.9% female; mean [SEM] age, 14.57 [0.03] years). Prevalence of e-cigarette use peaked in 2019 and then declined. Between 2014 and 2021, the age at initiation of e-cigarette use decreased, and intensity of use and addiction increased. By 2017, e-cigarettes became the most common first product used (77.0%). Age at initiation of use did not change for cigarettes or other tobacco products, and changes in intensity of use were minimal. By 2019, more e-cigarette users were using their first tobacco product within 5 minutes of waking than for cigarettes and all other products combined. Median e-cigarette use also increased from 3 to 5 d/mo in 2014 to 2018 to 6 to 9 d/mo in 2019 to 2020 and 10 to 19 d/mo in 2021., Conclusions and Relevance: The changes detected in this survey study may reflect the higher levels of nicotine delivery and addiction liability of modern e-cigarettes that use protonated nicotine to make nicotine easier to inhale. The increasing intensity of use of modern e-cigarettes highlights the clinical need to address youth addiction to these new high-nicotine products over the course of many clinical encounters. In addition, stronger regulation, including comprehensive bans on the sale of flavored tobacco products, should be implemented.
8502	Goldberg Scott, Shauna, Feigelson, Heather S., Powers, John David, Clennin, Morgan N., Lyons, Jason A., Gray, Mark T., Vachani, Anil, Burnett-Hartman, Andrea N. (2023) Demographic, Clinical, and Behavioral Factors Associated With Electronic Nicotine Delivery Systems Use in a Large Cohort in the United States. Tobacco use insights; 16<br><br>**Keywords:**<br>**Abstract:** Introduction: Our primary purpose is to understand comorbidities and health outcomes associated with electronic nicotine delivery systems (ENDS) use., Methods: Study participants were Kaiser Permanente (KP) members from eight US regions who joined the Kaiser Permanente Research Bank (KPRB) from September 2015 through December 2019 and completed a questionnaire assessing demographic and behavioral factors, including ENDS and traditional cigarette use. Medical history and health outcomes were obtained from electronic health records. We used multinomial logistic regression to estimate odd ratios (ORs) and 95% confidence intervals (CIs) of current and former ENDS use according to member characteristics, behavioral factors, and clinical history. We used Cox regression to estimate hazard ratios (HRs) and 95% CIs comparing risk of health outcomes according to ENDS use., Results: Of 119 593 participants, 1594 (1%) reported current ENDS use and 5603 (5%) reported past ENDS use. ENDS users were more likely to be younger, male, gay or lesbian, and American Indian / Alaskan Native or

	Asian. After adjustment for confounding, current ENDS use was associated with current traditional cigarette use (OR = 39.55; CI:33.44-46.77), current marijuana use (OR = 6.72; CI:5.61-8.05), history of lung cancer (OR = 2.64; CI:1.42-4.92), non-stroke cerebral vascular disease (OR = 1.55; CI:1.21-1.99), and chronic obstructive pulmonary disease (OR = 2.16; CI:1.77-2.63). Current ENDS use was also associated with increased risk of emergency room (ER) visits (HR = 1.17; CI: 1.05-1.30) and death (HR = 1.84; CI:1.02-3.32)., Conclusions: Concurrent traditional cigarette use, marijuana use, and comorbidities were prevalent among those who used ENDS, and current ENDS use was associated with an increased risk of ER visits and death. Additional research focused on health risks associated with concurrent ENDS and traditional cigarette use in those with underlying comorbidities is needed. Copyright © The Author(s) 2023.
8515	Graham, Amanda L., Cha, Sarah, Papandonatos, George D., Amato, Michael S., Jacobs, Megan A., Abroms, Lorien C., Berg, Carla J. (2022) E-cigarette and combusted tobacco abstinence among young adults: Secondary analyses from a U.S.-based randomized controlled trial of vaping cessation. Preventive medicine; 165<br><br>**Keywords:**<br>Humans Young Adult *Electronic Nicotine Delivery Systems *Smoking Cessation Tobacco *Tobacco Products Vaping/pc [Prevention & Control] *Vaping<br><br>**Abstract:** OBJECTIVE: To examine patterns of abstinence from e-cigarettes, combusted tobacco products (CTPs), both, or neither among young adults enrolled in a U.S.-based randomized trial of a text message vaping cessation intervention., METHODS: At baseline, 1829 young adult e-cigarette users were categorized as Exclusive E-cigarette Users (no past 30-day CTP use; n = 1036, 56.6%) or Dual Users (past 30-day CTP use; n = 793, 43.4%). Four groups were defined at 7-months: 1) Dual Abstinent, 2) Exclusive Vaping, 3) Exclusive CTP Use, and 4) Dual Users. The proportion of participants who were Dual Abstinent was the outcome of interest., RESULTS: At follow-up, 22.1% (95% CI: 20.3, 24.1) of participants were Dual Abstinent, 44.8% (95% CI: 42.5, 47.1) reported Exclusive Vaping, 6.3% (95% CI: 5.2, 7.5) reported Exclusive CTP Use, and 26.8% (95% CI: 24.8, 28.9) were Dual Users. A higher proportion of participants randomized to Intervention were Dual Abstinent (25.9%, 95% CI 23.1, 28.9) compared to Control (18.5%, 95% CI 16.0, 21.1; p = .0002). Analyses of treatment effects on dual abstinence by baseline tobacco product use favored Intervention over Control among both Exclusive E-cigarette Users (p = .019) and Dual Users (p = .0014)., CONCLUSION: A text message vaping cessation intervention was effective in promoting dual abstinence from e-cigarettes and CTPs among young adults. The advantage of treatment over control was equivalent for Exclusive E-cigarette Users and Dual Users. Rates of dual abstinence were higher among exclusive vapers than dual users, signaling the need for more research to optimize cessation programs for poly-tobacco users. Copyright © 2022 The Authors. Published by Elsevier Inc. All rights reserved.
8559	Hahn, Andrew W., Ruderman, Stephanie A., Nance, Robin M., Whitney, Bridget W., Eltonsy, Sherif, Haidar, Lara, Delaney, Joseph A. C., Drumright, Lydia N., Ma, Jimmy, Mayer, Kenneth H., Cleirigh, Conall O., Napravnik, Sonia, Eron, Joseph J., Christopoulos, Katerina, Bamford, Laura, Cachay, Edward, Jacobson, Jeffrey M., Willig, Amanda, Cropsey, Karen, Chander, Geetanjali, Crane, Heidi M., Fredericksen, Rob J. (2023) Vaporized Nicotine (E-Cigarette) and Tobacco Smoking Among People With HIV: Use Patterns and Associations With Depression and Panic Symptoms. Journal of acquired immune deficiency syndromes (1999); 92<br><br>**Keywords:**<br>Humans Nicotine/ae [Adverse Effects] *Electronic Nicotine Delivery Systems Depression/ep [Epidemiology] HIV Infections/co [Complications] HIV Infections/dt [Drug Therapy] *HIV Infections Tobacco Smoking<br><br>**Abstract:** BACKGROUND: Vaporized nicotine (VN) use is increasing among people with HIV (PWH). We

	examined demographics, patterns of use, depression, and panic symptoms associated with VN and combustible cigarette (CC) use among PWH., METHODS: We analyzed VN use among PWH in care at 7 US sites. PWH completed a set of patient-reported outcomes, including substance use and mental health. We categorized VN use as never vs. ever with the frequency of use (days/month) and CC use as never, former, or current. We used relative risk regression to associate VN and CC use, depression, and panic symptoms. Linear regression estimated each relationship with VN frequency. Models were adjusted for demographics., RESULTS: Among 7431 PWH, 812 (11%) reported ever-using VN, and 264 (4%) reported daily use. Half (51%) of VN users concurrently used CC. VN users were more likely than those without use to be younger, to be White, and to report ever-using CC. PWH reporting former CC use reported >=8.5 more days per month of VN use compared with never CC use [95% confidence interval (95% CI): 5.5 to 11.5 days/month] or current CC use (95% CI: 6.6 to 10.5 days/month). Depression (relative risk: 1.20 [95% CI: 1.02 to 1.42]) and panic disorder (1.71 [95% CI: 1.43 to 2.05]) were more common among PWH ever-using VN. Depression was common among PWH using VN (27%) and CC (22%), as was panic disorder (21% for VN and 16% for CC)., CONCLUSION: Our study elucidated demographic associations with VN use among PWH, revealed the overlap of VN and CC use, and associations with depression/panic symptoms, suggesting roles of VN in self-medication and CC substitution, warranting further longitudinal/qualitative research. Copyright © 2022 Wolters Kluwer Health, Inc. All rights reserved.
8560	Hair, Elizabeth C., Kreslake, Jennifer M., Rath, Jessica Miller, Pitzer, Lindsay, Bennett, Morgane, Vallone, Donna (2023) Early evidence of the associations between an anti-e-cigarette mass media campaign and e-cigarette knowledge and attitudes: results from a cross-sectional study of youth and young adults. Tobacco control; 32<br><br>**Keywords:**<br>Humans Adolescent Young Adult Cross-Sectional Studies *Electronic Nicotine Delivery Systems *Tobacco Products Mass Media Attitude<br><br>**Abstract:** OBJECTIVE: Antismoking mass media campaigns have contributed to significant declines in combustible tobacco use among young people. This study evaluates a national anti-e-cigarette campaign to determine its association with knowledge, attitudes and beliefs in the context of increasing e-cigarette use in the USA., METHODS: A national sample of respondents aged 15-24 years (n=8421) was drawn from a repeated cross-sectional online panel survey (220 participants/week) (October 2018 to December 2019). Self-reported exposure to the truth anti-e-cigarette campaign was measured according to level of ad awareness. Outcomes were subjective knowledge of campaign-targeted facts about e-cigarettes and attitudinal constructs about perceived e-cigarette harm, social unacceptability and anti-industry sentiments. Covariates included respondent demographics, current e-cigarette use and cigarette use, parental smoking, sensation seeking, mental health and growth in e-cigarette sales., RESULTS: Ad awareness was associated with knowledge that e-cigarette users are more likely to start smoking (low OR: 1.28, 95% CI 1.14 to 1.44; high OR: 1.88, 95% CI 1.66 to 2.13) and of the nicotine content of JUUL compared with cigarettes (low OR: 1.63, 95% CI 1.45 to 1.82; high OR: 2.50, 95% CI 2.21 to 2.84). High ad awareness was associated with knowledge that the long-term health effects of JUUL use are unknown (OR: 1.88, 95% CI 1.57 to 2.28). High ad awareness was associated with significantly higher perceived product harm (OR: 1.35, 95% CI 1.18 to 1.54), social unacceptability (OR: 1.32, 95% CI 1.15 to 1.53) and anti-industry attitudes (OR: 1.40, 95% CI 1.21 to 1.62), compared with respondents with no awareness., CONCLUSIONS: Young people with awareness of anti-e-cigarette ads demonstrate higher levels of campaign-targeted knowledge, attitudes and beliefs. Future campaign evaluation priorities include measuring the campaign effects on e-cigarette use behaviours. Copyright © Author(s) (or their employer(s)) 2023. No commercial re-use. See rights and permissions. Published by BMJ.
8580	Han, Dae-Hee, Elam, Kit K., Quinn, Patrick D., Huang, Chunfeng, Seo, Dong-Chul (2023) Within-person associations of escalated electronic nicotine delivery systems use with cigarette, alcohol, marijuana and drug use behaviors among US young adults. Addiction (Abingdon, England); 118

	**Keywords:**<br>Humans Young Adult United States/ep [Epidemiology] *Cannabis *Electronic Nicotine Delivery Systems Cross-Sectional Studies Binge Drinking/ep [Epidemiology] *Binge Drinking *Tobacco Products Substance-Related Disorders/ep [Epidemiology] *Substance-Related Disorders Tobacco Ethanol *Hallucinogens Vaping/ep [Epidemiology] *Vaping<br><br>**Abstract:** AIMS: Most extant evidence has addressed between-person differences, short-term or cross-sectional associations of electronic nicotine delivery systems (ENDS) use with other substance use, the majority focusing on current rather than escalated use. The present study aimed to examine within-person changes in escalated ENDS use and their associations with individual and combined substance use over a 6-year period., DESIGN, SETTING AND PARTICIPANTS: This study used a longitudinal cohort design with US young adults. A generalized linear mixed-model approach was employed to fit a series of weighted logistic regression models. Data were drawn from waves 1-5 of the Population Assessment of Tobacco and Health (PATH) study in the United States. Of the 9110 young adults at baseline, aged 18-24 years, a total of 5042 individuals had matched data across all five waves of assessments., MEASUREMENTS: Escalated ENDS use was computed by subtracting the number of days of ENDS use within the past 30 days at wave w - 1 from that at wave w and coded as 1 = escalated, if the value was greater than zero (otherwise, coded as 0 = not escalated)., FINDINGS: Escalated ENDS use gradually decreased over time, with the lowest prevalence at wave 4 (4.0%) but sharply increasing at wave 5 (8.4%). Escalated ENDS use was associated with increased odds of using each substance (binge drinking, marijuana use, marijuana vaping, prescription and illicit drugs) and different combinations of polysubstance use between cigarette smoking, binge drinking and marijuana use (Ps < 0.05). In addition, sweet/fruit flavor use (versus menthol/mint) was associated with increased likelihood of reporting co-use of cigarettes and marijuana., CONCLUSIONS: In the United States, the prevalence of young adults using electronic nicotine delivery systems appears to have increased steadily between 2013 and 2019, although the rate of increase may have started to accelerate in recent years. Escalated electronic nicotine delivery systems use and time-lagged established electronic nicotine delivery systems use appear to be prospectively associated with individual and combined substance use, particularly between cigarettes, alcohol and marijuana. Among established electronic nicotine delivery systems users, sweet/fruit flavor appears to be associated with increased risk of co-using cigarettes and marijuana. Copyright © 2022 The Authors. Addiction published by John Wiley & Sons Ltd on behalf of Society for the Study of Addiction.
8582	Han, Dae-Hee, Lee, Shin Hyung, Seo, Dong-Chul (2022) Within-Person Longitudinal Associations Between Electronic Nicotine Delivery Systems Use and Smoking Cessation Efforts Among US Continuing Adult Cigarette Smokers. Nicotine & tobacco research : official journal of the Society for Research on Nicotine and Tobacco; 24<br><br>**Keywords:**<br>Adult *Electronic Nicotine Delivery Systems Humans Smokers Smoking *Smoking Cessation *Tobacco Products<br><br>**Abstract:** OBJECTIVES: Prior studies that examined the role of electronic nicotine delivery systems (ENDS) use in smoking cessation have shown divergent conclusions. This study examined the time-course of ENDS-associated smoking abstinent behaviors among continuing cigarette smokers who were willing but unable to quit smoking., METHODS: Data were drawn from the four waves of the Population Assessment of Tobacco and Health Study. Of the 1684 smokers who tried to quit smoking completely at Wave 1, a total of 1094 who were continuing smokers until Wave 4 and were not lost to follow-ups comprised the sample. Using generalized linear mixed modeling, we fitted weighted negative binomial regression models to examine within-person associations of ENDS use with quit attempts and number of days abstinent from smoking., RESULTS: Quit attempt frequency and smoking abstinent days were highest at Wave 1, dropped at Wave 2, and then either increased a little or remained stagnant in later waves. ENDS use to quit smoking was associated with more frequent quit attempts (aIRR = 1.67, 95% CI = 1.42-1.95)

	and more abstinent days (aIRR = 3.28, 95% CI = 2.43-4.44), and the magnitude of such associations became stronger over time. ENDS use was associated with becoming a nondaily smoker among baseline daily smokers., CONCLUSIONS: ENDS use among continuing smokers may help increase the number of quit attempts and smoking abstinent days. Given that the study sample is continuing smokers who failed in complete smoking cessation, future research would be desirable that evaluates whether such abstinent behaviors sustain and manifest harm reduction with improved health outcomes., IMPLICATIONS: Smokers increasingly adopt electronic nicotine delivery systems (ENDS) to quit smoking. However, the association of ENDS use with smoking cessation efforts among continuing cigarette smokers who were willing but unable to quit smoking is largely unknown. This study found that quit attempts and smoking abstinent days increased with ENDS use to quit smoking by following up with population-representative continuing smokers. Additionally, this study assessed whether cigarette smokers' abstinent behaviors vary with their use of ENDS by estimating within-person associations with frequent assessments of both exposures and outcomes in a long-term perspective. Copyright © The Author(s) 2021. Published by Oxford University Press on behalf of the Society for Research on Nicotine and Tobacco. All rights reserved. For permissions, please e-mail: journals.permissions@oup.com.
8590	Hanafin, Joan, Sunday, Salome, Clancy, Luke (2021) Friends and family matter Most: a trend analysis of increasing e-cigarette use among Irish teenagers and socio-demographic, personal, peer and familial associations. BMC public health; 21<br><br>**Keywords:**<br>Adolescent Child Demography *Electronic Nicotine Delivery Systems Female Friends Humans Male Smoking/ep [Epidemiology] *Vaping<br><br>**Abstract:** BACKGROUND: E-cigarette ever-use and current-use among teenagers has increased worldwide, including in Ireland., METHODS: We use data from two Irish waves (2015, 2019) of the European School Survey Project on Alcohol and other Drugs (ESPAD) to investigate gender and teenage e-cigarette use (n = 3421 16-year-olds). Using chi-square analyses, we report changes in e-cigarette ever-use, current-use, and associated variables. Using multivariable logistic regression, we analyse the increase in e-cigarette use and socio-demographic, personal, peer and familial associations, focusing on gender differences., RESULTS: E-cigarette ever-use increased from 23% in 2015 to 37% in 2019, and current-use from 10 to 18%. Compared with 2015, the odds in 2019, of becoming both an e-cigarette ever-user and current-user, were significantly higher for girls than boys (ever-use: AOR 2.67 vs 2.04; current-use: AOR 3.11 vs 1.96). Smoking and e-cigarette use are linked but never-smokers who try e-cigarettes rose significantly from 33 to 67% and those using e-cigarettes to quit smoking decreased significantly from 17 to 3%. Almost two-thirds of respondents (66%) in 2019 said that their reason for trying e-cigarettes was "out of curiosity". Peer smoking is significantly associated with likelihood of e-cigarette ever-use (AOR 6.52) and current-use (AOR 5.45). If "Most/All friends smoke", odds were significantly higher for boys than for girls (ever-use AOR 7.07 vs 6.23; current-use AOR 5.90 vs 5.31). Less parental monitoring is significantly associated with greater e-cigarette ever-use (AOR 3.96) and current-use (4.48), and having parents who usually don't know where their child is on Saturday nights was also associated with significantly higher odds for boys than for girls (ever-use AOR 5.42 vs 3.33; current-use AOR 5.50 vs 3.50)., CONCLUSION: Respondents had significantly higher odds of being e-cigarette ever- and current-users in 2019 compared with 2015. Use is higher among boys but girls are increasingly at risk. Two-thirds had never smoked cigarettes at first e-cigarette use; two-thirds used out of curiosity but few (3%) for smoking cessation. The most prominent risk factors for e-cigarette use were peer- and parent-related, especially so for boys. Interventions that take account of friend and family influences may provide mechanisms for preventing an increasing risk of nicotine addiction. Copyright © 2021. The Author(s).
8592	Hanafin, J., Sunday, S., Clancy, L. (2022) E-cigarettes in Ireland - prevalence, motivations, and relationship with tobacco. European Respiratory Journal; 60

	**Keywords:**<br>adolescence adolescent child conference abstract controlled study *electronic cigarette friend human *Ireland Irish (citizen) law longitudinal study major clinical study male *motivation *prevalence random sample secondary analysis smoking smoking cessation *tobacco tobacco control tobacco dependence alcohol<br><br>**Abstract:** Adolescent e-cigarette use is increasing worldwide amid concerns about: identified and as yet unknown harms; nicotine addiction; being a "gateway" drug; and renormalisation of smoking. This study aimed to establish, from available data, the changing prevalence of Irish adolescents' e-cigarette use, reasons for use, and relationship with tobacco at first use. We analysed five Irish health datasets with questions on e-cigarette use among 16 and 17 year olds: ECIGS-TFRI (2014, N=817); SILNE-R (2016, N=2051); ESPAD (European Schools Project on Alcohol and Drugs) 2015 (N=1508), 2019 (3556); GUI (Growing Up in Ireland), Ireland's national longitudinal study (N=6216). All datasets comprised stratified random samples in school-based settings. Secondary analysis was carried out, using descriptive statistical techniques. Prevalence of e-cigarette ever-use increased from 23% in 2014 to 39% in 2019, and was very high among boys (46% by 2019). By 2019, more adolescents tried e-cigarettes (39%) than smoking (32%). By 2019, curiosity (66%) and friends (29%) were the two main reasons adolescents said they first used e-cigarettes, with only 3% saying it was to quit smoking Those saying they had never used tobacco when they first tried e-cigarettes increased from 32% in 2015 to 68% in 2019. Teenage e-cigarette use has risen rapidly in Ireland, with boys more at risk. The majority were not smokers when they started using e-cigarettes, pointing to new routes into nicotine addiction. They were not used by adolescents for smoking cessation. In addition to new regulation and legislation for e-cigarettes, current tobacco control regulations for young people should be extended to include e-cigarettes.
8606	Harlow, Alyssa F., Stokes, Andrew C., Brooks, Daniel R., Benjamin, Emelia J., Barrington-Trimis, Jessica L., Ross, Craig S. (2022) e-Cigarette Use and Combustible Cigarette Smoking Initiation Among Youth: Accounting for Time-Varying Exposure and Time-Dependent Confounding. Epidemiology (Cambridge, Mass.); 33<br><br>**Keywords:**<br>Adolescent Cigarette Smoking/ep [Epidemiology] *Cigarette Smoking *Electronic Nicotine Delivery Systems Humans Tobacco *Tobacco Products Vaping/ep [Epidemiology] *Vaping<br><br>**Abstract:** BACKGROUND: Youth e-cigarette use is associated with the initiation of combustible cigarette smoking, but prior studies have rarely accounted for time-varying measures of e-cigarette exposure or time-dependent confounding of e-cigarette use and smoking initiation., METHODS: Using five waves of the Population Assessment of Tobacco and Health (2013-2019), we estimated marginal structural models with inverse probability of treatment and censoring weights to examine the association between time-varying e-cigarette initiation and subsequent cigarette smoking initiation among e-cigarette- and cigarette-naive youth (12-17 years) at baseline. Time-dependent confounders used as predictors in inverse probability weights included tobacco-related attitudes or beliefs, mental health symptoms, substance use, and tobacco-marketing exposure., RESULTS: Among 9,584 youth at baseline, those who initiated e-cigarettes were 2.4 times as likely to subsequently initiate cigarette smoking as youth who did not initiate e-cigarettes (risk ratio = 2.4, 95% confidence interval [CI] = 2.1, 2.7), after accounting for time-dependent confounding and selection bias. Among youth who initiated e-cigarettes, more frequent vaping was associated with greater risk of smoking initiation (risk ratio >=3 days/month = 1.8, 95% CI = 1.4, 2.2; 1-2 days/month = 1.2; 95% CI = 0.93, 1.6 vs. 0 days/month). Weighted marginal structural model estimates were moderately attenuated compared with unweighted estimates adjusted for baseline-only confounders. At the US population level, we estimated over half a million youth initiated cigarette smoking because of prior e-cigarette use over follow-up., CONCLUSIONS: The association between youth vaping and combustible cigarette smoking persisted after accounting for time-dependent confounding. We estimate that e-cigarette use accounts for a considerable share of cigarette initiation among US

	youth. See video abstract at, http://links.lww.com/EDE/B937. Copyright © 2022 Wolters Kluwer Health, Inc. All rights reserved.
8610	Harrell, Melissa B., Mantey, Dale S., Chen, Baojiang, Kelder, Steven H., Barrington-Trimis, Jessica (2022) Impact of the e-cigarette era on cigarette smoking among youth in the United States: A population-level study. Preventive medicine; 164<br><br>**Keywords:**<br>Adolescent Humans United States/ep [Epidemiology] *Electronic Nicotine Delivery Systems Cigarette Smoking/ep [Epidemiology] *Cigarette Smoking Tobacco *Tobacco Products Schools Prevalence<br><br>**Abstract:** To examine and compare trends in past 30-day cigarette smoking among adolescents in the US from 2002 to 2019, before and after the onset of the "e-cigarette era" in 2014. Using National Youth Tobacco Survey (NYTS) data from 2002 to 2019, we modeled the prevalence of past 30-day cigarette smoking over time. A total of n = 274,551 (weighted N = 340,403,754) middle and high school students were included in this study. Interrupted time series analyses were used to examine changes in cigarette smoking over time and compare trends in cigarette smoking pre- and post-2014. Models were applied to the full sample and stratified by middle (6th-8th grade) and high school (9th-12th grade). The observed number of current adolescent cigarette smokers post-2014 was compared to the predicted number, given trends in cigarette smoking prevalence observed pre-2014. Among all students, past 30-day cigarette smoking declined by approximately 0.75% per year from 2002 to 2013 ($p < 0.001$). Following a significant drop in prevalence from 2013 to 2014 (1.64%; $p < 0.001$), the decline in past 30-day cigarette smoking slowed significantly to approximately 0.37% per year ($p < 0.001$), from 2015 to 2019. We estimate that the onset of the "e-cigarette era" in 2014 corresponded to over 1.66 million (95% CI: 1.57 m - 1.75 m) more past 30-day cigarette smokers from 2015 to 2019, cumulatively. The rate of decline in past 30-day cigarette smoking prevalence among adolescents observed since 2002 slowed with the onset of the "e-cigarette era" in 2014, providing evidence at a population-level for the "gateway effect." Copyright © 2022. Published by Elsevier Inc.
8636	Hedman, L., Backman, H., Stridsman, C., Lindberg, A., Ronmark, E. (2021) Most users quit e-cigarettes without succeeding to quit smoking. European Respiratory Journal; 58<br><br>**Keywords:**<br>adult aged conference abstract *electronic cigarette ex-smoker fear female follow up friend human longitudinal study major clinical study male non-smoker obstructive lung disease questionnaire smoking *smoking cessation smoking habit Sweden telephone interview tobacco nicotine<br><br>**Abstract:** Background: Whether e-cigarette use contributes to successful smoking cessation remains controversial. Aim(s): To study e-cigarette (EC) initiation and quitting in relation to smoking habits in a longitudinal study. Method(s): Within the Obstructive Lung Disease in Northern Sweden (OLIN) studies, 168 individuals reported EC use in a postal questionnaire survey (PQ) about respiratory health in 2016. In 2020, these individuals were invited to telephone interviews and 63 (38%) participated (52% women, 20-77y, mean 44y). Information about past and current EC, tobacco and other nicotine product use were collected and reasons for initiating or quitting EC recorded. Result(s): In the PQ, 5 (8%) of the EC users were non-smokers, 19 (30%) former smokers, and 39 (62%) smokers. At follow-up, 9 (15%) used EC daily, 22 (33%) occasionally, and 32 (52%) had quit. Of the 9 daily EC users, 1 was a smoker while 8 were former smokers, where of 6 were former smokers also in the PQ. Of the 32 that quit EC, 15 (47%) were still smokers, 8 (25%) had quit smoking since the PQ, and 1 had re-started smoking. The most common reason for EC initiation was to quit smoking (n=32; where of 16 former smokers and 16 still smokers) followed by curiosity (n=17), being less harmful than cigarettes (n=14), recommended by friends (n=11), and to reduce the number of cigarettes/day (n=10). The most common reasons to quit EC was trouble with the device (n=13), fear of health effects (n=9), did not want to use them anymore (n=9), and did not help

	them quit smoking (n=6). Conclusion(s): In this 4-year follow-up of EC users, more than half had quit using EC but without succeeding to quit smoking.
8638	Heiden, Brendan T., Baker, Timothy B., Smock, Nina, Pham, Giang, Chen, Jingling, Bierut, Laura J., Chen, Li-Shiun (2022) Assessment of formal tobacco treatment and smoking cessation in dual users of cigarettes and e-cigarettes. Thorax;<br><br>**Keywords:**<br>**Abstract:** BACKGROUND: The utility of electronic cigarettes ('e-cigarettes') as a smoking cessation adjunct remains unclear. Similarly, it is unclear if formal tobacco treatment (pharmacotherapy and/or behavioural support) augments smoking cessation in individuals who use both cigarettes and e-cigarettes., METHODS: We performed a longitudinal cohort study of adult outpatients evaluated in our tertiary care medical centre (6/2018-6/2020). E-cigarette use, smoking status and formal tobacco treatment (deterrent pharmacotherapy and/or behavioural support) were assessed in 6-month blocks (eg, cohort 1 (C1)=6/2018-12/2018, C2=1/2019-6/2019 and so on) using our electronic health record. We assessed the relationship between e-cigarette use (either with or without formal tobacco treatment) and point prevalence of smoking cessation at 6 and 12 months., RESULTS: 111 823 unique patients were included in the study. The prevalence of dual use of cigarettes and e-cigarettes increased significantly over the study period (C1=0.8%; C2=1.1%; C3=1.8%; C4=2.3%; p<0.001). The prevalence of smoking cessation at 12 months was higher among e-cigarette users (20.8%) compared with non-users (16.8%) (risk difference, 4.0% (95% CI 2.5% to 5.5%); adjusted relative risk (aRR) 1.354, 95% CI 1.252 to 1.464, p<0.0001). Further, among dual users of cigarettes and e-cigarettes, the prevalence of smoking cessation at 12 months was higher among individuals who received tobacco treatment (29.1%) compared with individuals who did not receive tobacco treatment (19.6%) (risk difference, 9.5% (95% CI, 4.6% to 14.4%); aRR 1.238, 95% CI 1.071 to 1.432, p=0.004)., INTERPRETATION: These results suggest that dual users of cigarettes and e-cigarettes benefit from formal tobacco treatment. Clinicians should consider offering formal tobacco treatment to such patients, though future trials are needed. Copyright © Author(s) (or their employer(s)) 2022. No commercial re-use. See rights and permissions. Published by BMJ.
8656	Heris, Christina, Scully, Maree, Chamberlain, Catherine, White, Victoria (2022) E-cigarette use and the relationship to smoking among Aboriginal and Torres Strait Islander and non-Indigenous Australian Secondary Students, 2017. Australian and New Zealand journal of public health; 46<br><br>**Keywords:**<br>Adolescent Humans Vaping/ep [Epidemiology] *Vaping *Electronic Nicotine Delivery Systems Native Hawaiian or Other Pacific Islander Community-Based Participatory Research Australia/ep [Epidemiology] Prospective Studies Longitudinal Studies Smoking/ep [Epidemiology] Students<br><br>**Abstract:** OBJECTIVE: Estimate the prevalence of e-cigarette use (vaping) among Aboriginal and Torres Strait Islander adolescents and explore the relationship between vaping and tobacco use., METHODS: N=18,199 students aged 12-17 years (n=1,097 Aboriginal and Torres Strait Islander) participating in the 2017 Australian Secondary Students' Alcohol and Drug (ASSAD) Survey self-reported their e-cigarette and lifetime, past month and past week smoking behaviours., RESULTS: Twenty-two per cent of Aboriginal and Torres Strait Islander students (14% all) reported having ever used e-cigarettes. Significantly greater odds of e-cigarette use among Aboriginal and Torres Strait Islander students was observed overall, but not among regular (past month/week) smokers. There were significant associations between e-cigarette use and any level of smoking for all students (p<0.001), with no variation by Indigenous status. While e-cigarette use was more common among smokers, 28% of Aboriginal and Torres Strait Islander ever-vapers (35% all ever-vapers) were never smokers., CONCLUSIONS: There is substantial prevalence of e-cigarette ever-use among Australian secondary students, including Aboriginal and Torres Strait Islander students, and a strong relationship with tobacco use., IMPLICATIONS FOR PUBLIC HEALTH: Policies facilitating e-cigarette access must not undercut tobacco control efforts for adolescents, particularly

	Aboriginal and Torres Strait Islander people who continue to experience higher smoking rates. Copyright © 2022 The Authors.
8705	Hu, S. Sean, Wang, Teresa W., Homa, David M., Tsai, James, Neff, Linda (2022) Cigarettes, Smokeless Tobacco, and E-Cigarettes: State-Specific Use Patterns Among U.S. Adults, 2017-2018. American journal of preventive medicine; 62<br><br>**Keywords:**<br>Adolescent Adult *Electronic Nicotine Delivery Systems Humans Tobacco *Tobacco Products Tobacco Use/ep [Epidemiology] Tobacco Use Disorder/ep [Epidemiology] *Tobacco Use Disorder *Tobacco, Smokeless United States/ep [Epidemiology]<br><br>**Abstract:** INTRODUCTION: State-level monitoring of changes in tobacco product use can inform tobacco control policy and practice. This study examines the state-specific prevalence of current cigarette smoking, smokeless tobacco use, and E-cigarette use and related cigarette quitting behaviors among E-cigarette users during 2017-2018., METHODS: Data from the 2017 and 2018 Behavioral Risk Factor Surveillance System were used to assess state-specific current use of cigarettes, smokeless tobacco, and E-cigarettes among adults aged >=18 years. Analyzed in 2021, state-specific tobacco product estimates and relative percentage changes between 2017 and 2018 were computed for U.S. states and the District of Columbia. Chi-square tests captured subgroup differences, and logistic regression assessed changes over time., RESULTS: Prevalence of adult current cigarette smoking and smokeless tobacco use varied across states and remained relatively stable during 2017-2018, whereas the prevalence of adult E-cigarette use significantly increased during 2017-2018 among 19 of 36 states that collected Behavioral Risk Factor Surveillance System E-cigarette data in 2018. In all states and the District of Columbia during 2017-2018 combined, the percentage of current cigarette smoking among current E-cigarette users was higher than that of never cigarette smoking; the percentage of attempting to quit cigarette smoking in the past year among dual users of cigarettes and E-cigarettes was >50%., CONCLUSIONS: During 2017-2018, the prevalence of adult current cigarette smoking and smokeless tobacco use varied across states and remained relatively stable, whereas adult E-cigarette use prevalence significantly increased. Comprehensive state-based tobacco prevention and control efforts are warranted to reduce the morbidity and mortality attributed to the use of all tobacco products, including E-cigarettes, among U.S. adults. Copyright Published by Elsevier Inc.
8709	Huang, Siyi, Chen, Qiushi, Griffin, Paul, Liu, Guodong, Azagba, Sunday (2023) Longitudinal transitions in tobacco use in youth and young adults: A latent transition analysis of the population assessment of tobacco and health study from Wave 1 to 5. Addictive behaviors; 138<br><br>**Keywords:**<br>Humans Male Adolescent Young Adult Female Tobacco *Electronic Nicotine Delivery Systems Tobacco Use/ep [Epidemiology] Tobacco Use/px [Psychology] Smoking/px [Psychology] *Tobacco Products Longitudinal Studies<br><br>**Abstract:** BACKGROUND: Tobacco use by youth and young adults can lead to significant long-term health problems. We aim to understand transitions in tobacco use patterns among these groups and the factors that affect transition patterns., METHODS: Using the five waves of data from the nationally representative Population Assessment of Tobacco and Health (PATH) Study (2013-2019), we conducted latent class analysis and latent transition analysis to understand tobacco use classes and the longitudinal transitions between classes. We also adjusted for covariates, including demographics, individual behaviors, household environment, and psychosocial factors, to capture their effects on class transition probabilities., RESULTS: Three tobacco use behaviors were identified: non-current user (C1), moderate e-cigarette user (C2), and poly-tobacco user (C3). At baseline (Wave 1), 94.4% of participants were classified as C1, 3.2% as C2, and 2.4% as C3, and the distribution shifted towards C2 and C3 over time. Progression to the next class represented the most common transitions (14.1% C2 to C3, 10.7% C1 to C2),

	while the direct progression from C1 to C3 was rare (0.6%). Being male, White, adult, living in smoking-allowed households, past-year alcohol use, drug use, internalizing problems, and social media follower of tobacco brands were associated with a faster progression to poly-tobacco use., CONCLUSIONS: The transition patterns implied that e-cigarette use might be an intermediate progression from non-current use into poly-tobacco use. Individual behaviors, household environment, and psychosocial factors are associated with elevated risks of progression. The findings may inform tobacco prevention and cessation policies among youth and young adults. Copyright © 2022 Elsevier Ltd. All rights reserved.
8731	Irvine, Dylan S., McGarity-Shipley, Ellen, Lee, Eun-Young, Janssen, Ian, Leatherdale, Scott T. (2022) Longitudinal Associations Between e-Cigarette Use, Cigarette Smoking, Physical Activity, and Recreational Screen Time in Canadian Adolescents. Nicotine & tobacco research : official journal of the Society for Research on Nicotine and Tobacco; 24<br><br>**Keywords:**<br>Adolescent Canada/ep [Epidemiology] Cigarette Smoking/ep [Epidemiology] *Cigarette Smoking *Electronic Nicotine Delivery Systems Exercise Humans Longitudinal Studies Screen Time *Vaping<br><br>**Abstract:** INTRODUCTION: This study examined longitudinal associations between e-cigarette use, cigarette smoking, physical activity, and recreational screen time (ST) in a cohort of Canadian adolescents (ages 14-17 years; grades 9-12)., AIMS AND METHODS: Data from 5951 adolescents who participated in COMPASS Year 4 (2015-2016; baseline) and Year 6 (2017-2018; follow-up) were used. Exposures included e-cigarette use and cigarette smoking. Outcomes included cutpoints for moderate- to vigorous-physical activity (MVPA; >=60 min/d), muscular strengthening exercises (MSE; >=3 time/wk), participation in sport (SP; intramural or competitive), and recreational screen time (ST; <=430 min/day). Generalized linear mixed models were performed., RESULTS: e-Cigarette use (16.6% vs. 39.2%), cigarette smoking (0.9% vs. 4.7%), and dual use (0.8% vs. 4.1%) increased from baseline to follow-up. SP (70.8% vs. 61.3%) and the prevalence of meeting MVPA (49.8% vs. 42.1%) and MSE cutpoints (54.0% vs. 45.3%) decreased from baseline to follow-up. Recreational ST remained similar from baseline to follow-up. New e-cigarette use at follow-up was associated with maintenance of SP and meeting MVPA and MSE cutpoints, but also with increased ST. New cigarette smoking at follow-up was associated with maintaining high ST and low SP. Cigarette smoking at baseline and follow-up was associated with maintaining high ST, low MSE, and low SP. Cigarette smoking cessation at follow-up was associated with increasing MVPA and MSE, decreasing ST, and maintaining low SP., CONCLUSION: Given the clustering and co-occurring unhealthy behavioral patterns, intervention strategies to promote healthy lifestyles should take a holistic approach, by targeting multiple behavioral changes simultaneously., IMPLICATIONS: This investigation highlighted that, unhealthy behaviors, particularly e-cigarette use, cigarette smoking, and excessive use of screens, tend to co-occur among Canadian adolescents. Therefore, intervention strategies to promote healthy lifestyles should take a holistic approach, by targeting multiple behavioral changes simultaneously particularly in school and community settings. As an exception, new and stable e-cigarette use appears to co-occur with achieving sufficient levels of physical activity. Increasing awareness about the risk of e-cigarette use may target population groups that are physically and socially active (eg, athletes, sport teams). Copyright © The Author(s) 2021. Published by Oxford University Press on behalf of the Society for Research on Nicotine and Tobacco. All rights reserved. For permissions, please e-mail: journals.permissions@oup.com.
8765	Jebai, Rime, Osibogun, Olatokunbo, Li, Wei, Gautam, Prem, Bursac, Zoran, Ward, Kenneth D., Maziak, Wasim (2022) Temporal Trends in Tobacco Product Use Among US Middle and High School Students: National Youth Tobacco Survey, 2011-2020. Public health reports (Washington, D.C. : 1974);<br><br>**Keywords:**<br>**Abstract:** OBJECTIVES: Despite significant declines in cigarette smoking during the past decade, other tobacco products gained popularity among middle and high school students. This study examined temporal trends in exclusive and concurrent use of tobacco products among middle and high school students in the United States from 2011 through 2020., METHODS: We used multiple annual datasets

	from the National Youth Tobacco Survey from 2011 through 2020 (N = 193 350) to examine trends of current (past 30 days) exclusive, dual, and poly use of tobacco products (ie, cigarettes, electronic cigarettes [e-cigarettes], cigars, hookahs, and smokeless tobacco). We used joinpoint regression models to calculate log-linear trends in annual percentage change (APC)., RESULTS: During 2011-2020, exclusive use of any tobacco product decreased significantly, except for e-cigarettes, which increased significantly at an APC of 226.8% during 2011-2014 and 14.6% during 2014-2020. This increase was more pronounced among high school students (APC = 336.6% [2011-2014] and 15.7% [2014-2020]) than among middle school students (APC = 10.4% [2014-2020]) and among male students (APC = 252.8% [2011-2014] and 14.8% [2014-2020]) than among female students (APC = 13.6% [2014-2020]). During 2011-2020, we also found upward trends in dual use of e-cigarettes and cigarettes (APC = 17.3%). Poly use of e-cigarettes, cigarettes, and any other tobacco products increased significantly at an APC of 57.1% during 2011-2014., CONCLUSIONS: The emergence of new tobacco products such as e-cigarettes in the US market has shifted the landscape of tobacco use among adolescents in the last decade toward poly product use, in which e-cigarettes are a prominent component. Our findings underscore the increasing complexity of tobacco use among adolescents in the United States and the need for strong policies and regulations adapted to evolving trends in cigarette and noncigarette tobacco products.
8842	Kechter, Afton, Simpson, Kelsey A., Ceasar, Rachel Carmen, Schiff, Sara J., Yamaguchi, Naosuke, Bluthenthal, Ricky N., Smiley, Sabrina L., Barrington-Trimis, Jessica L. (2022) Trajectories of Nicotine Use Leading to Dual and Cyclical Tobacco Product Use in Young Adults. Nicotine & tobacco research : official journal of the Society for Research on Nicotine and Tobacco; 24<br><br>**Keywords:**<br>Adolescent Adult *Electronic Nicotine Delivery Systems Female Humans Male Nicotine Smoking Cessation/mt [Methods] *Smoking Cessation *Tobacco Products *Tobacco Use Disorder United States *Vaping Young Adult<br><br>**Abstract:** INTRODUCTION: Young adult never-smokers who vape are at elevated risk of initiating cigarettes, while young adults who smoke often begin vaping to substitute or reduce cigarette use. Reasons underlying different use patterns of tobacco products are not well-understood., AIMS AND METHODS: We conducted 1-on-1 qualitative interviews with young adults (N = 62) who vape in Los Angeles, California from June 2018 to June 2019. Participants were 18-25 years old (79% male; racially/ethnically diverse) and self-reported vaping >=1x/week. We used a semi-structured interview guide and applied thematic analysis method to analyze data., RESULTS: Young adults initiated vaping due to peer socialization and e-liquid flavor novelty. They often reported vaping (after first smoking) due to a belief that e-cigarettes are healthier, social pressure to quit smoking, and convenience of use. Participants reported smoking (after first vaping) when traveling outside of the United States where vaping products were less accessible, and cigarettes were normative. Many of the personal narratives described patterns of dual and cyclical use, which was often attributed to nicotine dependence and cost, or described as dependent upon the current environment (eg, at a party)., CONCLUSIONS: The current study characterizes nicotine use trajectories and reasons why young adults vape, and smoke cigarettes. Dual and cyclical use of both e-cigarettes and cigarettes was common; this pattern of use should be considered in policy and prevention work to address nicotine dependence among young people., IMPLICATIONS: We display findings from the current study in a model depicting common trajectories of nicotine use, along with reasons for initiation, transitions between products, and dual/cyclical e-cigarette and combustible cigarette use. Copyright © The Author(s) 2021. Published by Oxford University Press on behalf of the Society for Research on Nicotine and Tobacco. All rights reserved. For permissions, please e-mail: journals.permissions@oup.com.
8848	Keller-Hamilton, Brittney Lee (2022) Application of the social ecological model to reduce tobacco use among adolescents. Dissertation Abstracts International: Section B: The Sciences and Engineering; 83<br><br>**Keywords:**

	*Costs and Cost Analysis *Policy Making *Risk Factors *Tobacco Smoking *Social Ecology Epidemiology Neighborhoods Retailing<br><br>**Abstract:** Background. Tobacco use remains the leading preventable cause of death in the United States. Adolescence presents a critical window for preventing tobacco use, as most adults started using tobacco before reaching age 18. One framework that is useful for understanding adolescent tobacco use is the social ecological model, which defines risk and protective factors for health behaviors, like tobacco use, at multiple levels of influence. This research studied risk factors for tobacco use at the individual, community, and policy levels. In a cohort of adolescent boys, we evaluated whether e-cigarette use increased risk of initiating cigarette or smokeless tobacco (SLT) use (individual level) and whether perceived neighborhood quality was associated with initiation of emerging and traditional tobacco products (community level). In a sample of Columbus, Ohio, tobacco retailers, we evaluated whether implementation of Tobacco 21 (T21) policy was associated with a change in trajectories of tobacco marketing practices (policy level). Methods. The adolescent cohort included N=1220 boys who were 11- to 16-years-old at enrollment and were followed every six months for two years. All boys lived in urban Franklin County, Ohio, or one of nine Appalachia Ohio counties. On each survey, boys reported their use of tobacco products, as well as risk and protective factors for tobacco use. The sample of tobacco retailers included N=74 to N=103 stores that held a cigarette dealer license. Stores were audited by trained fieldworkers in 2014, 2016, 2017, 2018, and 2019 (T21 enforcement began between the 2017 and 2018 audits). Fieldworkers recorded the prices of cigarette packs, availability of flavored products, count of products advertised outside stores, and count of products with interior or exterior price promotions.To estimate risk of initiating cigarette and SLT use after using e-cigarettes, we conducted a propensity score match in 25 multiple imputation datasets. Risk ratios (RRs) and standard errors were estimated in each dataset and results were combined. To estimate risk of initiating emerging (i.e., e-cigarettes and hookah) and traditional (i.e., cigarettes, cigars, and SLT) tobacco products, we ran Cox proportional hazards regression models that included indicators of perceived safety, social disorder, physical disorder, and cohesion for youths' neighborhoods at age 10 in five multiple imputation datasets. To evaluate whether trajectories of retailer marketing practices changed post-T21 implementation, we used mixed effects regression models. Results. In our analysis of tobacco use risk factors at the individual level, e-cigarette use more than doubled the risk of starting to smoke cigarettes (RR=2.71; 95% CI: 1.89, 3.87) and use SLT (RR=2.42; 95% CI:1.73, 3.38) among adolescent boys. At the community level, living in a physically disordered neighborhood at age 10 was associated with greater risk of initiating traditional tobacco products (hazard ratio = 2.01; 95% CI: 1.09, 3.79). At the policy level, implementation of T21 was associated with a change in the trajectories of the price of Marlboro Reds (per-year price increase of .25 pre-T21 vs. .08 post-T21; p=0.008) and count of price-promoted tobacco products (21% decrease per year pre-T21 vs. 29% increase per year post-T21; p=0.004). Discussion. At a time when the landscape of tobacco products used by adolescents is changing rapidly, this research advanced our understanding of risk factors for tobacco use at the individual, community, and policy levels of the social ecological model. Findings can be used to support interventions and policies to reduce tobacco use among adolescents. Results also provide directions for future research that would improve understanding of the associations we observed. (PsycInfo Database Record (c) 2022 APA, all rights reserved)
8851	Kelly, Brian C., Vuolo, Mike, Maggs, Jennifer, Staff, Jeremy (2023) E-cigarette use among early adolescent tobacco cigarette smokers: testing the disruption and entrenchment hypotheses in two longitudinal cohorts. Tobacco control;<br><br>**Keywords:**<br>**Abstract:** OBJECTIVE: Using longitudinal data from two large-scale cohorts in the UK and USA, we examine whether e-cigarette use steers adolescent early smokers away from tobacco cigarettes (disruption hypothesis) or deepens early patterns of tobacco smoking (entrenchment hypothesis) in comparison with early smokers who do not use e-cigarettes., METHODS: Youth who smoked tobacco cigarettes by early adolescence (before age 15) were selected from the ongoing UK Millennium Cohort

	Study (n=1090) and the US Population Assessment of Tobacco and Health (n=803) study. In regression models, the focal predictor was lifetime use of an e-cigarette by early adolescence and the primary outcome was current tobacco use by late adolescence (before age 18). Logistic and multinomial models controlled for early adolescent risk factors and sociodemographic background, and were weighted for attrition and adjusted for complex survey designs., RESULTS: Among youth who were early cigarette smokers, 57% of UK and 58% of US youth also used e-cigarettes. The odds of later adolescent smoking among early smoking youth were significantly higher among e-cigarette users relative to those who had not used e-cigarettes (adjusted OR (AORUK)=1.45; AORUSA=2.19). In both samples, multinomial models indicated that early smoking youth who used e-cigarettes were more likely to be frequent smokers relative to not smoking (AORUK=2.01; AORUSA=5.11) and infrequent smoking (AORUK=1.67; AORUSA=2.11)., CONCLUSIONS: Despite national differences in e-cigarette regulation and marketing, there is evidence e-cigarette use among early adolescent smokers in the UK and USA leads to higher odds of any smoking and more frequent tobacco cigarette use later in adolescence. Copyright © Author(s) (or their employer(s)) 2023. No commercial re-use. See rights and permissions. Published by BMJ.
8883	Kittaneh, Ahmad A., Lechner, William V. (2022) Associations Between Internalizing and Externalizing Symptoms on Subsequent Tobacco Product Initiation and Sustained Use as a Function of Sex Among Adolescents Ages 12-17. Journal of studies on alcohol and drugs; 83<br><br>**Keywords:**<br>Adolescent Male Female Humans Child *Electronic Nicotine Delivery Systems Tobacco Longitudinal Studies *Tobacco Products Tobacco Use/ep [Epidemiology] Tobacco Use/px [Psychology]<br><br>**Abstract:** OBJECTIVE: Previous studies have found positive associations between internalizing and externalizing symptoms and the initiation of tobacco use among adolescents. However, few studies have examined the relationship between these symptoms and sustained tobacco use patterns or how they may vary as a function of sex. The current study examined whether these symptoms predict initiation and sustained use of multiple tobacco products within a longitudinal cohort design., METHOD: Waves 1-3 of the Population Assessment of Tobacco and Health Study (2013-2016) were used to assess relationships between internalizing and externalizing symptoms, sex, and tobacco use categories (cigarettes, e-cigarettes, poly-tobacco use, other tobacco use) in 6,170 adolescents (M age = 13.42, SD = 1.10) who were tobacco naive at Wave 1., RESULTS: Internalizing and externalizing symptoms predicted initiation of several tobacco categories at the 2-year follow-up. Examining sustained use patterns revealed a more consistent relationship with externalizing symptoms across tobacco categories, whereas associations with internalizing symptoms were limited. Sex moderated the relationship between elevated internalizing symptoms and increased odds of subsequent e-cigarette and poly-tobacco initiation, with stronger relationships observed among females. In addition, the relationship between elevated externalizing symptoms and sustained other tobacco use was stronger in females as compared with males., CONCLUSIONS: Although both internalizing and externalizing symptoms predict tobacco initiation across several product categories, externalizing symptoms may reflect a more reliable signal of sustained tobacco use in adolescents. Mental health symptoms and subsequent tobacco use differed by sex, with females demonstrating stronger relationships for several products.
8888	Klemperer, Elias M., Bunn, Janice Y., Palmer, Amanda M., Smith, Tracy T., Toll, Benjamin A., Cummings, K. Michael, Carpenter, Matthew J. (2023) E-cigarette cessation and transitions in combusted tobacco smoking status: longitudinal findings from the US FDA PATH Study. Addiction (Abingdon, England);<br><br>**Keywords:**<br><br>**Abstract:** BACKGROUND AND AIMS: Electronic nicotine delivery systems (ENDS) can help people to quit smoking combusted tobacco products (CTPs), but most current and former smokers who use ENDS also intend to quit ENDS. This analysis measured whether ENDS cessation among current and former CTP

	smokers is associated with changes in CTP smoking or abstinence., DESIGN: Regression analysis of a nationally representative cohort from waves 4 (W4) and 5 (W5) of the Population Assessment of Tobacco and Health Study (December 2016-November 2019)., SETTING: United States., PARTICIPANTS: Adults (n = 1525) who reported W4 current or former use of >= 1 CTP and either currently using ENDS or quitting ENDS in the past year were included., MEASUREMENTS: Logistic regressions were performed separately among W4 current and former CTP smokers, controlling for demographic and tobacco use characteristics. First, we analyzed proximal outcomes by testing the association between ENDS quit status and CTP abstinence, both occurring during W5. Next, we analyzed long-term outcomes by testing W4 ENDS quit status as a predictor of CTP abstinence at W5, approximately 1 year later., FINDINGS: Among W4 current CTP smokers, there was no evidence that CTP smoking abstinence differed between those quitting or continuing using ENDS, both in our proximal [adjusted odds ratio (aOR) = 1.37, 95% confidence interval (CI) = 0.90, 2.10] and long-term (aOR = 0.90, 95% CI = 0.52, 1.53) analyses. Among former CTP smokers, quitting ENDS was associated with less CTP abstinence in our proximal analysis (aOR = 0.42, 95% CI = 0.20, 0.89), but there was no evidence that CTP smoking abstinence differed between those quitting or continuing using ENDS in our long-term analysis (aOR = 0.86, 95% CI = 0.44, 1.67)., CONCLUSIONS: There is no evidence that ENDS cessation is associated with CTP abstinence among current smokers, although mixed findings among former smokers indicate a possible risk for relapse to smoking associated with quitting ENDS. Copyright © 2023 The Authors. Addiction published by John Wiley & Sons Ltd on behalf of Society for the Study of Addiction.
8898	Kong, Grace, Chaffee, Benjamin W., Wu, Ran, Krishnan-Sarin, Suchitra, Liu, Feifei, Leventhal, Adam M., McConnell, Rob, Barrington-Trimis, Jessica (2022) E-cigarette device type and combustible tobacco use: Results from a pooled analysis of 10,482 youth. Drug and alcohol dependence; 232<br><br>**Keywords:**<br>Adolescent Adult Cross-Sectional Studies *Electronic Nicotine Delivery Systems Humans *Tobacco Products Tobacco Use/ep [Epidemiology] Vaping/ep [Epidemiology] *Vaping Young Adult<br><br>**Abstract:** INTRODUCTION: To understand whether using a certain e-cigarette device is more strongly associated with risk of combustible tobacco use among youth., METHODS: We conducted multivariable logistic regression analyses using cross-sectional data from 4 samples of youth in Connecticut and California (N = 10,482; ages 13-24), separately for each study using the total sample and the sample of past-month e-cigarette users, to understand the association between e-cigarette device type and past-month combustible tobacco use, while controlling for age, sex, race/ethnicity, socioeconomic status, school, and past-month marijuana use. Then, we conducted meta-analyses to calculate pooled associations for adolescents, young adults, and all individuals combined., RESULTS: Among the total sample, combustible tobacco use was associated with any e-cigarette device use (vs. no e-cigarette use) in the pooled analysis across all studies. Among past-month e-cigarette users, combustible tobacco use across all studies was 15.8%- 61.5%. Pooled associations among past-month e-cigarette users showed that using disposable devices (vs. pods; AOR=2.83, 95% CI: 1.73-4.61) and multiple devices most frequently (vs. pods; AOR=2.13, 95% CI: 1.16-3.90) was associated with greater odds of combustible tobacco use. Pooled associations also found that using multiple devices (vs. a single device) in the past month was associated with greater odds of combustible tobacco use (AOR 2.33, 95% CI: 1.74, 3.14)., DISCUSSION: Using disposable e-cigarettes and multiple devices is associated with greater likelihood of combustible tobacco use among e-cigarette using youth. Future research should elucidate the trajectory of e-cigarette device used and combustible tobacco use among youth to inform prevention and product regulation. Copyright © 2022 Elsevier B.V. All rights reserved.
8920	Krishnan, Nandita (2022) Applying social cognitive theory to identify predictors of electronic nicotine product use trajectories and cessation. Dissertation Abstracts International: Section B: The Sciences and Engineering; 83<br><br>**Keywords:**

	*Harm Reduction *Intervention *Nicotine *Smoking Cessation *Tobacco Smoking Electronic Cigarettes<br><br>**Abstract:** Electronic nicotine products (ENPs) are considered less harmful than cigarettes and could contribute to harm reduction if they facilitate sustained smoking cessation. On the other hand, ENPs may impede harm reduction and smoking cessation efforts if they are concurrently used with cigarettes for an extended duration, lead to smoking relapse among former smokers, or contribute to cigarette smoking initiation among never smokers. Additionally, ENPs have independent health risks and their long-term effects are unknown.In this dissertation, Population Assessment of Tobacco and Health (PATH) study data was analyzed to identify trajectories of ENP and cigarette use among adult ENP users. Informed by Social Cognitive Theory (SCT), cognitive, behavioral, and socio-environmental predictors of: 1) ENP and cigarette trajectories; 2) ENP quit attempts and cessation; 3) combined ENP and cigarette smoking abstinence; and 4) exclusive ENP use, were examined among ENP users.Across wave 1 to wave 5, two trajectories of ENP use (declining, consistent) and four trajectories of cigarette use (abstinent, occasional, declining, consistent) were identified. The majority of ENP users (52.3%) belonged to the joint trajectory characterized by declining ENP use and consistent cigarette use. Tobacco and ENP use characteristics emerged as significant predictors of group membership. Higher nicotine dependence was associated with membership in more harmful trajectories. Daily ENP use was associated with membership in less harmful cigarette trajectories but the consistent (vs declining) ENP trajectory. Socio-demographic characteristics also differed across trajectories.Wave 3 predictors of wave 4 ENP quit attempts, cessation, combined ENP and smoking abstinence, and exclusive ENP use included several SCT-based factors. Cognitive factors included interest in quitting ENPs, self-efficacy to quit ENPs, and perceived risk of ENPs; behavioral factors included cigarette smoking status, and use of non-combustibles and marijuana. Socio-environmental factors were household ENP rules and exposure to ENP advertising. However, there were differences in the magnitude and direction of the relationships between these variables and each of the outcomes examined. Findings of this dissertation indicate that SCT offers a useful framework for guiding ENP cessation and harm reduction interventions. ENPs do not appear to be displacing cigarette smoking at the population level, which underscores the need to promote smoking cessation and abstinence among ENP users. Interventions also need to address co-occurring use of other tobacco products and marijuana. Different cognitive factors may need to be targeted based on intervention objectives (harm reduction vs harm prevention) and outcomes (quit attempts vs cessation) of interest. Promoting supportive physical and social environments, through household vaping restrictions and limiting exposure to ENP advertising may also facilitate ENP cessation and harm reduction. (PsycInfo Database Record (c) 2022 APA, all rights reserved)
8922	Krishnan, Nandita, Berg, Carla J., Elmi, Angelo F., Klemperer, Elias M., Sherman, Scott E., Abroms, Lorien C. (2022) Trajectories of ENDS and cigarette use among dual users: analysis of waves 1 to 5 of the PATH Study. Tobacco control;<br><br>**Keywords:**<br><br>**Abstract:** INTRODUCTION: Concurrent electronic nicotine delivery system (ENDS) and cigarette (dual) use is harmful. Identifying longitudinal trajectories of ENDS and cigarette use among dual users can help to determine the public health impact of ENDS and inform tobacco control policies and interventions., OBJECTIVES: (1) To identify independent and joint trajectories of ENDS and cigarette use among wave (W) 1 adult dual users across W1 to W5 of the Population Assessment of Tobacco and Health (PATH) Study; and (2) identify W1 predictors of ENDS and cigarette joint trajectory group membership., METHODS: We used group-based trajectory modelling to estimate independent and joint trajectories of ENDS and cigarette use from wave 1 (W1; 2013-2014) to wave 5 (W5; 2018-2019) among W1 adult established dual users of ENDS and cigarettes (n=545) from the PATH Study. We used multinomial logistic regression to identify W1 predictors of joint trajectories., RESULTS: Two ENDS (early quitters=66.0%, stable users=34.0%) and three cigarette (stable users=55.2%, gradual quitters=27.3%, early

	quitters=17.5%) trajectories of W1 were identified. In joint trajectory analysis, 41.6% of participants were early ENDS quitters and stable cigarette users; 14.8% early ENDS quitters and gradual cigarette quitters; 14.6% stable ENDS users and stable cigarette users; 11.2% stable ENDS users and gradual cigarette quitters; 10.3% early ENDS quitters and early cigarette quitters; and 7.4% stable ENDS users and early cigarette quitters. Cigarette and ENDS use frequency, nicotine dependence, cannabis use and other non-combusted tobacco product use predicted trajectory group membership (p values <0.05)., CONCLUSIONS: Most dual users maintained long-term cigarette smoking or dual use, highlighting the need to address cessation of both products. Continued monitoring of trajectories and their predictors is needed, given ongoing changes to the ENDS marketplace. Copyright © Author(s) (or their employer(s)) 2022. No commercial re-use. See rights and permissions. Published by BMJ.
8931	Kurtulus, Serif, Can, Remziye (2022) Use of e-Cigarettes and Tobacco Products Among Youth in Turkey. The Eurasian journal of medicine; 54<br><br>**Keywords:**<br><br><br>**Abstract:** OBJECTIVE: The aim of this study was to determine high school students' views about tobacco products and e-cigarettes and e-cigarette smoking prevalence in one of Turkey's western provinces., MATERIALS AND METHODS: This cross-sectional study included 286 students. The data collection form consisted of 18 questions related to sociodemographic features and tobacco and e-cigarette use., RESULTS: Of the 286 participants, 32.2% reported having used a tobacco product in the past month, 1.02% reported having used e-cigarettes, and 15.2% have tried e-cigarettes at least once. Of those who tried e-cigarettes, 19.2% of them did so because of curiosity. A positive correlation was found between e-cigarette trial and tobacco use, with age. Smoking/e-cigarette use status was significant with "e-cigarettes are harmful" (P=.034), "e-cigarette smoke is harmful" (P = .003), and "selling and advertising e-cigarettes is prohibited" (P =.043)., CONCLUSION: This study determined that 3 out of 10 youths used tobacco products, and 1 used e-cigarettes. There is a need to raise awareness among high school students and youth regarding the dangers of e-ciga- rettes and tobacco products.
8978	Lee, P. N., Fry, J. S. (2022) Investigating the effect of e-cigarette use on quitting smoking in adults aged 25 years or more using the PATH study. F1000Research; 9<br><br>**Keywords:**<br>adult anxiety article body mass confounding variable female follow up health status human logistic regression analysis male nicotine replacement therapy observational study *population health prevalence quality of life risk factor sensitivity analysis smokeless tobacco *smoking smoking cessation snus systematic review tobacco tobacco dependence *electronic cigarette *population assessment of tobacco and health<br><br>**Abstract:** Background: The evidence on harms and benefits of e-cigarettes partly concerns whether their use encourages smokers to quit. We addressed this using data from the nationally representative PATH study, with detailed accounting for potential confounding variables. Method(s): We considered adults aged 25+. Our original analyses, reported in version 1 of this paper, used data for Waves 1 to 3, separate analyses considering Waves 1 to 2, 2 to 3 and 1 to 3. These related baseline ever e-cigarette use (or e-product use at Wave 2) to quitting at follow-up, adjusting for confounders derived from 55 candidates. Sensitivity analyses omitted ever other product users, linked quitting to current e-cigarette use, and used values of some predictors modified using follow-up data. Additional analyses used data for Waves 1 to 4, separately considering sustained, delayed and temporary quitting during Waves 1 to 3, 2 to 4 and 1 to 4. Sensitivity analyses considered 30-day quitting, restricted attention to smokers attempting to quit, and considered ever smokeless tobacco or snus use. Result(s): In the original analyses, unadjusted odds ratios (ORs) of quitting smoking for ever e-cigarette use were 1.29 (95% CI 1.01-1.66), 1.52 (1.26-1.83) and 1.47 (1.19-1.82) for the Wave 1 to 2, 2 to 3, and 1 to 3 analyses. These reduced after adjustment, to 1.23

	(0.94-1.61), 1.51 (1.24-1.85) and 1.39 (1.11-1.74). Quitting rates remained elevated in users in all sensitivity analyses. The additional analyses found associations of e-cigarette use with sustained, delayed and temporary quitting, associations little affected by considering 30-day quitting, and only slightly reduced restricting attention to quit attempters. Ever use of smokeless tobacco or snus also predicted increased quitting. Conclusion(s): As does most evidence from clinical trials, other analyses of PATH, and other epidemiological studies, our results suggest using e-cigarettes helps adult smokers to quit.Copyright © 2022 Lee PN and Fry JS.
8979	Lee, P. N., Fry, J. S., Forey, B. A., Coombs, K. J., Thornton, A. J. (2021) Cigarette consumption in adult dual users of cigarettes and e-cigarettes: a review of the evidence, including new results from the PATH study. F1000Research; 9<br><br>**Keywords:**<br>adult alcohol consumption article case report *cigarette smoking clinical article cross-sectional study demographics *electronic cigarette female human male prospective study quantitative analysis *smoking tobacco<br><br>**Abstract:** Background: Modelling disease risk from e-cigarette use requires knowing how much e-cigarette uptake affects a smoker's cigarette consumption. From Waves 1-3 of the US PATH study and other evidence, we sought answers to three questions: (Q1) Does consumption differ between dual users and exclusive smokers? (Q2) Does initiating e-cigarettes affect cigarette consumption? (Q3) Is baseline consumption related to later initiation of e-cigarettes? Methods: Consumption data from PATH were corrected for clear recording errors, with e-cigarette use classified as established or every day, and adjustment made for demographics, use of alcohol, drugs and other tobacco products, and age of starting smoking. Searches identified other studies published since 2008, with Q1 answered from cross-sectional studies, and Q2 and Q3 also from prospective studies. Results: (Q1) In PATH, consumption in current every day e-cigarette users was 2-3 cigarettes per day lower in dual users, with no difference seen in established users. From 31 published studies, consumption was no lower in dual users for current or ever e-cigarette users. (Q2) In PATH study consistent decreases in consumption occurred in those becoming every day dual users with increases seen in those reverting to exclusive smoking. Thirteen published studies consistently showed reduced consumption in those becoming dual users. (Q3) Three studies consistently showed greater consumption in smokers subsequently initiating e-cigarettes, but no significant difference was seen for PATH. Conclusion(s): Assessment is complicated by the few every day e-cigarette users in PATH, and between-study variability in quantifying e-cigarette use and presentation of results. As taking up e-cigarettes is associated with reducing consumption, finding no clear cross-sectional difference between dual users and exclusive smokers suggests smokers taking up e-cigarettes had higher consumption initially, limited evidence supporting this. Given the much lower disease risk of e-cigarettes vs. cigarettes, smokers becoming dual users should somewhat reduce their disease risk.Copyright: © 2021 Lee PN et al.
8997	Levy, David T., Gartner, Coral, Liber, Alex C., Sanchez-Romero, Luz Maria, Yuan, Zhe, Li, Yameng, Cummings, K. Michael, Borland, Ron (2023) The Australia Smoking and Vaping Model: The Potential Impact of Increasing Access to Nicotine Vaping Products. Nicotine & tobacco research : official journal of the Society for Research on Nicotine and Tobacco; 25<br><br>**Keywords:**<br>Humans Vaping/ep [Epidemiology] *Vaping Nicotine Australia/ep [Epidemiology] Smoking *Smoking Cessation *Electronic Nicotine Delivery Systems<br><br>**Abstract:** BACKGROUND: We model the potential impact of relaxing current nicotine vaping product (NVP) restrictions on public health in Australia., AIMS AND METHODS: A Restricted NVP Scenario was first developed to project current smoking and vaping rates, where a U.S. smoking model was calibrated to recent Australian trends. To model less restrictive NVP policies, a Permissive NVP Scenario applied rates

	of switching from smoking to vaping, initiation into NVP and cigarette use, and cessation from smoking and vaping based on U.S. trends. The model measures vaping risk relative to the excess mortality rate of smoking. The public health impacts are measured as the difference between smoking- and vaping-attributable deaths (SVADs) and life years lost (LYLs) in the Restricted and Permissive NVP Scenarios. Sensitivity analysis is conducted regarding the NVP excess risk and other factors., RESULTS: Assuming an NVP excess risk of 5% that of smoking, 104.2 thousand SVADs (7.7% reduction) and 2.05 million LYLs (17.3% reduction) are averted during 2017-2080 in the Permissive NVP Scenario compared to the Restricted NVP Scenario. Assuming 40% NVP excess risk, 70 thousand SVADs and 1.2 million LYLs are averted. The impact is sensitive to the rate at which smokers switch to NVPs and quit smoking, and relatively insensitive to the smoking initiation and NVP initiation and cessation rates., CONCLUSIONS: The model suggests the potential for public health gains to be achieved by relaxing NVP access regulations. However, the model would benefit from better information regarding the impact of NVPs on smoking under a relaxation of current restrictions., IMPLICATIONS: Australia has implemented a strong array of cigarette-oriented policies, but has restricted access to NVPs. The Smoking and Vaping Model offers a framework for modeling hypothetical policy scenarios. The Australian model shows the potential for public health gains by maintaining cigarette-oriented policies while relaxing the current restrictive NVP policy. Modeling results under a permissive NVP policy are particularly sensitive to the estimated rates of smoking cessation and switching to vaping, which are not well established and will likely depend on past and future cigarette-oriented policies and the specific NVP policies implemented in Australia. Copyright © The Author(s) 2022. Published by Oxford University Press on behalf of the Society for Research on Nicotine and Tobacco.
9003	Li, Dongmei, Ossip, Deborah J., Bansal-Travers, Maansi, Xie, Zidian (2022) Impact of the FDA flavour enforcement policy on flavoured electronic cigarette use behaviour changes. Tobacco control; 31<br><br>**Keywords:**<br>Adult United States Humans *Electronic Nicotine Delivery Systems *Vaping Menthol United States Food and Drug Administration Flavoring Agents *Tobacco Products Policy<br><br>**Abstract:** INTRODUCTION: This study aims to investigate electronic cigarette (e-cigarette) use behaviour changes after the implementation of the US Food and Drug Administration (FDA) restriction on the sale of all unauthorised flavoured cartridge-based e-cigarettes other than tobacco and menthol flavour on 6 February 2020, as well as factors associated with these changes., METHODS: Through Amazon's Mechanical Turk service, 3533 current adult flavoured e-cigarette users (who were not exclusive tobacco-flavoured or menthol-flavoured e-cigarette users) were recruited for an online survey from 8 July to 29 July 2021. Multiple logistic regression models were used to identify significant factors associated with quitting e-cigarette use, switching to other flavoured electronic nicotine delivery system (ENDS) products, switching to combustible tobacco products, switching to menthol-flavoured e-cigarettes and switching to tobacco-flavoured e-cigarettes., RESULTS: Resulting from the FDA flavour enforcement policy, the top four e-cigarette use behaviour changes were: (1) switching to other flavoured ENDS products such as the tank system or disposable e-cigarettes (29.24%), (2) switching to menthol-flavoured pod systems (18.09%), (3) switching to combustible tobacco products (14.12%) and (4) switching to tobacco-flavoured pod systems (12.03%). There were 4.9% participants who indicated that they quit e-cigarette use. Overall, multiple factors, especially past 30-day use of certain flavours, were associated with different behaviour changes., CONCLUSIONS: The implementation of the FDA flavour enforcement policy on cartridge-based e-cigarette was associated with significant e-cigarette behaviour changes, with multiple factors being associated with these changes. These results provide important information for future regulations of flavoured e-cigarette products. Copyright © Author(s) (or their employer(s)) 2022. Re-use permitted under CC BY-NC. No commercial re-use. See rights and permissions. Published by BMJ.
9014	Li, Xiaoyin, Holahan, Carole K., Loukas, Alexandra, Holahan, Charles J., Pasch, Keryn E., Marti, C. Nathan (2023) Alternative Tobacco Use and Cigarette Smoking Transitions among College Students in Texas. Substance use & misuse; 58

	**Keywords:**<br>Young Adult Humans Cigarette Smoking/ep [Epidemiology] *Cigarette Smoking Texas/ep [Epidemiology] Universities Tobacco Use *Tobacco Products Risk Factors Students *Electronic Nicotine Delivery Systems Adenosine Triphosphate<br><br>**Abstract:** Background: High prevalence of very light cigarette smoking and use of alternative tobacco products (ATPs; i.e. electronic nicotine delivery systems [ENDS], cigars, and hookah) among young adult college students are causes for concern. The purpose of this study is to examine transitions in cigarette smoking (never vs. non-current vs. very light vs. heavier) among college students across 2.5 years and determine if the use of ATPs is related to these transitions. Methods: This study used six waves of data across 2.5 years from Project M-PACT. Participants who were 18-25 years of age at baseline were included in this study (n = 4,806). Cigarette smoking state was categorized as never smoking, non-current smoking [0 cigarettes smoked per day (cpd) in past month], very light smoking (< =5 cpd in past month), and heavier smoking (>5 in past month). Multi-state Markov models were used to examine temporal transitions in the four smoking states and examine the association of time-varying current ATP use with transitions in smoking states. Results: The probabilities of remaining in a smoking state decreased over time. The time-varying current ATP use was significantly related to increased odds of transitioning from never smoking to non-current smoking, from never smoking to very light smoking, and from non-current to very light smoking. Conclusions: Findings highlight the need to prevent ATP use among college students and in turn inhibit initiation and escalation of cigarette smoking.
9069	Loukas, Alexandra, Marti, C. Nathan, Harrell, Melissa B. (2022) Electronic nicotine delivery systems use predicts transitions in cigarette smoking among young adults. Drug and alcohol dependence; 231<br><br>**Keywords:**<br>Adult Cigarette Smoking/ep [Epidemiology] *Cigarette Smoking *Electronic Nicotine Delivery Systems Female Humans Longitudinal Studies Male *Tobacco Use Disorder Universities *Vaping Young Adult<br><br>**Abstract:** BACKGROUND: Although cigarette smoking during young adulthood is characterized by volatility, few studies examine if use of electronic nicotine delivery systems (ENDS) impacts transitions in cigarette use behaviors across this developmental period. The purpose of this longitudinal study was to examine the role of ENDS use on three transitions in cigarette smoking among young adults; initiation, desistance, and re-uptake., METHODS: Participants were 5029 18-29-year-olds (64.2% female) enrolled in one of 24 Texas colleges at baseline and involved in an eight-wave, 4.5-year study. A multi-state, continuous time Markov model was used to assess the role of current/past 30-day and ever ENDS use on three transitions, spanning at least six months 1) never to current smoking (initiation); 2) current to non-current smoking (desistance); and 3) non-current to current smoking (re-uptake). The model also contained time-invariant socio-demographic, and time-varying intrapersonal (other tobacco use, nicotine dependence, sensation seeking, depressive symptoms) and interpersonal (peer cigarette use) covariates., RESULTS: Both current and ever ENDS use increased the probability of transitioning from never to current cigarette use (initiation) and decreased the probability of transitioning from current to non-current use (desistance). Current, but not ever, ENDS use also increased the probability of transitioning from non-current to current use (re-uptake). Adjustment for socio-demographic, intrapersonal, and interpersonal covariates did not alter these findings., DISCUSSION: ENDS use in young adulthood increases the risk for cigarette smoking behaviors across the continuum of uptake and progression. Prevention and cessation efforts targeting both ENDS and cigarette use during young adulthood are needed. Copyright © 2022 Elsevier B.V. All rights reserved.
9070	Loukas, Alexandra, Marti, C. Nathan, Harrell, Melissa B., Pasch, Keryn E., Wilkinson, Anna V. (2022) Electronic nicotine delivery systems (ENDS) dependence among Texas Young Adults, 2014-2019: Increasing trajectory after the 2017 surge in vape pod popularity. Drug and alcohol dependence; 241

	**Keywords:**<br>Young Adult Female Humans Male *Electronic Nicotine Delivery Systems Nicotine *Vaping *Tobacco Use Disorder Universities *Tobacco Products<br><br>**Abstract:** INTRODUCTION: Use of electronic nicotine delivery systems (ENDS) increased significantly after 2017. The increase is attributed to a surge in popularity of vape pods containing nicotine salts, which are high nicotine concentration ENDS that may heighten risk for dependence. However, little is known about changes in ENDS dependence before and after the 2017 surge. We examined the trajectory of ENDS dependence among young adults from 2014 to 2019., METHODS: Participants were 1700 18-25-year-olds (57.6 % female) from 24 Texas colleges who reported past 30-day ENDS use in at least one of eight study waves. ENDS dependence was assessed at all waves with one item, use of ENDS within 30 min of waking. A discontinuous growth curve model was fit to test the hypothesis that the ENDS dependence trajectory would increase only after 2017, from 2018 to 2019. The model included socio-demographic and cigarette dependence covariates RESULTS: The proportion of young adults reporting ENDS dependence ranged from 2.3 % in 2014 to 8.2 % in 2019. Results from the discontinuous growth curve model indicated that the ENDS dependence trajectory was flat from fall 2014 to spring 2017. However, the post-2017 trajectory, from spring 2018 to spring 2019, was positive and significant indicating that the proportion of young adults reporting ENDS dependence increased after 2017., CONCLUSIONS: The increase in ENDS dependence after 2017 likely resulted from the surge in popularity of vape pods containing nicotine salts. These vape pods are a disruptive technology that may result in more young adults developing nicotine dependence symptoms than do earlier generation devices. Copyright © 2022 Elsevier B.V. All rights reserved.
9079	Lund, Ingeborg, Saebo, Gunnar (2023) Vaping among Norwegians who smoke or formerly smoked: reasons, patterns of use, and smoking cessation activity. Harm reduction journal; 20<br><br>**Keywords:**<br>Adult Humans Smoking Cessation/mt [Methods] *Smoking Cessation Vaping/ep [Epidemiology] *Vaping *Electronic Nicotine Delivery Systems Norway/ep [Epidemiology] *Tobacco Use Disorder<br><br>**Abstract:** BACKGROUND: The majority of Norwegians who use e-cigarettes are adults who have smoked. Little is known about vaping reasons and -patterns in this group. The aim of this paper was to study vaping prevalence, patterns, and motivations among adults who smoke. Furthermore, to investigate smoking intensity and smoking cessation behaviour differences between those who vape and those who do not., METHODS: This study was based on two separate Norwegian samples: People who had ever smoked, from 2017 (N = 2099), and people who currently smoked and recent quitters, from 2018/2019 (N = 1336). Measures of vape frequencies, vape motives, and smoking cessation behaviours were utilised in descriptive analyses of relationships between vaping and smoking behaviour., RESULTS: Less than 1 in 10 in the ever-smoked group, 1 in 5 of the currently smoked or recently quit group, were currently vaping. Ever trial rates for vaping were much higher at 1 in 3 in the ever-smoked group, and 1 in 2 in the currently smoked or recently quit group. Dual use with combustible cigarettes was common, but people who smoked tended to use e-cigarettes less frequently while those who formerly smoked tended to use them more frequently. Both quitting attempts and smoking intensity reduction were positively associated with vaping, and the most common reasons for e-cigarette use were reported to be desires to reduce harm, to stop smoking, or to reduce smoking intensity., CONCLUSION: The results indicate that Norwegians who smoke tend to see e-cigarettes as a tool to reduce or completely stop smoking. The predominance of use-motivations related to reducing harm points at the importance of conveying correct information about relative harmfulness of tobacco- and nicotine products. Copyright © 2023. The Author(s).
9120	Maldonado Viasus, Diana C., Perdomo, Gustavo A., Garcia Duran, Maria C., Pulido, Adriana C., Hernandez, Manuela Luchau, Borrero Roldan, Elizabeth, Botero, M. Catalina, Moreno, Sergio M. (2022) ENDS consumption in students of higher education: Phenomenon on the rise during the COVID-19 pandemic.

	Tobacco induced diseases; 20<br><br>**Keywords:**<br><br><br>**Abstract:** INTRODUCTION: The COVID-19 pandemic triggered transformations in the population's lifestyles, including electronic nicotine delivery system (ENDS) consumption. The aim of the study was to determine associations between ENDS consumption habits and lifestyles among higher education students in Bogota, Colombia., METHODS: This study employed a cross-sectional analytical design, based on a self-administered online survey, conducted in 2021, among students aged 18-59 years. The sample size was 3985 students. Statistical analysis was done through frequency studies, hypothesis testing and a multivariate-penalized logistic regression model (firthlogit), based on the Akaike information criterion (AIC)., RESULTS: A total of 3573 students completed the survey, 61.5% were female, 55.3% were young (aged 18-26 years), and 44.6% were adults (aged 27-59 years). The prevalence of ENDS use during the pandemic was 7.3%. Age was negatively associated with ENDS use, as young people had a higher likelihood of using these devices. The likelihood of ENDS use was negative among females (OR=0.38; 95% CI: 0.2-0.5). In contrast, it was positive in students with a history of psychoactive substance abuse and/or dependence (OR=3.59; 95% CI: 1.0-12.0), students who had tried conventional cigarettes (OR=5.12; 95% CI: 3.0-8.5), participants who smoked tobacco during the pandemic (OR=3.15; 95% CI: 2.3-4.2), those who studied virtually (OR=1.52; 95% CI: 1.0-2.3), participants who lived with other ENDS users (OR=3.86; 95% CI: 2.8-5.2) and students with negative perception of impacts on their mental health (OR=1.48; 95% CI: 1.1-1.9)., CONCLUSIONS: Being male, aged <26 years, having a history of substance use, having tried conventional cigarettes, pandemic tobacco use, and having lived with other ENDS users, were the main factors associated with pandemic ENDS use. Studying the consumption habits of students in response to lifestyle changes, is fundamental for the formulation of strategies to reduce the development of addictive behaviors, especially in young students during the pandemic. Copyright © 2022 Maldonado Viasus D.C. et al.
9137	Mantey, Dale S., Omega-Njemnobi, Onyinye, Ruiz, Felisa A., Chen, Baojiang, Springer, Andrew E., Kelder, Steven H. (2023) Remote Versus In-Person Learning During COVID-19: Comparison of E-Cigarette Susceptibility and Ever Use Among a Diverse Cohort of 6th-Grade Students in Texas. Nicotine & tobacco research : official journal of the Society for Research on Nicotine and Tobacco; 25<br><br>**Keywords:**<br>Adolescent Humans Texas/ep [Epidemiology] *Electronic Nicotine Delivery Systems RNA, Viral COVID-19/ep [Epidemiology] *COVID-19 SARS-CoV-2 Students Disease Susceptibility<br><br>**Abstract:** BACKGROUND: In response to SARS-CoV2 (COVID-19), school districts incorporated remote learning as a mitigation strategy. This study examines the association between classroom setting (ie, on-campus versus remote) and e-cigarette susceptibility or ever use among a sample of Texas public middle school students., METHODS: Data from n = 985 students enrolled in the CATCH My Breath E-Cigarette Prevention Program trial were collected in Spring 2021. Participants were 6th-grade students in urban Texas. E-cigarette use was examined using the "at-risk" definition described by FDA, indicating either: (1) susceptible never user; or (2) experimental ever use. A multilevel, logistic regression model examined the association between classroom setting and e-cigarette susceptibility/ever use. Covariates included sex, race/ethnicity, academic achievement, household e-cigarette use, perceived school connectedness, and school-level economic status. Models account for nesting within school district. Analyses stratified by race/ethnicity were also conducted., RESULTS: Overall, 36.3% of the sample were susceptible never users or ever e-cigarette users. The sample was comprised of 55.0% on-campus and 45.0% remote learners. On-campus learners had greater odds of reporting e-cigarette susceptibility or ever use (aOR: 1.45; p = .014). These findings were observed among Latino (aOR: 1.77; p = .026) and White (aOR: 2.10; p = .099) but not African American/Black (aOR: 0.86; p = .728) youth., CONCLUSIONS: On-campus learning during

	the Spring 2021 semester was associated with greater risk for e-cigarette susceptibility or ever use among a diverse sample of 6th-grade students. E-cigarette susceptibility and ever use is a risk factor for progression to long-term e-cigarette use in later adolescence., IMPLICATIONS: As school districts prepare to return to on-campus learning in 2022, a focused approach to e-cigarette prevention may be needed to prevent widespread e-cigarette initiation and continued use. Further, study findings demonstrate a need for further research on the school environment as a determinant of e-cigarette use. Copyright © The Author(s) 2022. Published by Oxford University Press on behalf of the Society for Research on Nicotine and Tobacco. All rights reserved. For permissions, please e-mail: journals.permissions@oup.com.
9138	Mapanga, Witness, Craig, Ashleigh, Mtintsilana, Asanda, Dlamini, Siphiwe N., Du Toit, Justin, Ware, Lisa J., Norris, Shane A. (2023) The Effects of COVID-19 Pandemic Lockdowns on Alcohol Consumption and Tobacco Smoking Behaviour in South Africa: A National Survey. European addiction research;<br><br>**Keywords:**<br><br><br>**Abstract:** INTRODUCTION: During the first phase of the coronavirus (COVID-19) pandemic lockdowns in South Africa (SA), both alcohol and tobacco were considered non-essential goods and their sales were initially prohibited and further restricted to certain days and timeframes. This study investigates self-reported changes in alcohol consumption and tobacco smoking behaviour in the general population during the COVID-19 pandemic lockdowns in SA., METHODS: A cross-sectional national survey was conducted in October 2021 (before the Omicron wave 4 and while SA was in low-level lockdown) among 3,402 nationally representative respondents (weighted to 39,640,674) aged 18 years and older. Alcohol consumption and tobacco use were assessed from the beginning of the lockdown towards the end of March 2020 until October 2021 using the WHO-AUDIT and the US Centre for Disease Control (CDC) Global Adult Tobacco Survey questionnaires, respectively., RESULTS: Among those that drank alcohol (33.2%), 31.4% were classified as having a drinking problem that could be hazardous or harmful and 18.9% had severe alcohol use disorder during the COVID-19 lockdowns. Twenty-two per cent (22.0%) of those that reported alcohol consumption reported that the COVID-19 pandemic lockdowns changed their alcohol consumption habits, with 38.1% reporting a decreased intake or quitting altogether. Among the one in five respondents (19.2%) who had ever smoked, most reported smoking at the time of the survey (82.6%) with many classified as light smokers (87.8%; <=10 cigarettes/day). Almost a third (27.2%) of those smoking reported that the COVID-19 pandemic lockdowns had changed their use of tobacco products or vaping, with 60.0% reporting a reduction/quitting tobacco use. Given that sales were restricted this indicates that people could still get hold of tobacco products. Heavy smoking was associated with older age (p = 0.02), those classified as wealthy (p < 0.001), those who started or increased tobacco smoking during the pandemic lockdowns (p = 0.01) and residential provinces (p = 0.04)., CONCLUSION: Given restrictions on the sale of alcohol and tobacco in SA between 27 March and August 17, 2020, during the pandemic, respondents reported an overall decline in alcohol consumption and tobacco use which might suggest that the regulatory restrictive strategies on sales had some effect but may be inadequate, especially during times where individuals are likely to experience high-stress levels. These changes in alcohol consumption and tobacco use were different from what was reported in several European countries, possibly due to differences in the restrictions imposed in SA when compared to these European countries. Copyright © 2023 S. Karger AG, Basel.
9148	Martinelli, Thomas, Candel, Math J. J. M., de Vries, Hein, Talhout, Reinskje, Knapen, Vera, van Schayck, Constant P., Nagelhout, Gera E. (2023) Exploring the gateway hypothesis of e-cigarettes and tobacco: a prospective replication study among adolescents in the Netherlands and Flanders. Tobacco control; 32<br><br>**Keywords:**<br>Humans Adolescent Tobacco *Electronic Nicotine Delivery Systems Prospective Studies Netherlands/ep [Epidemiology] Longitudinal Studies *Adolescent Behavior Vaping/ep [Epidemiology] Vaping/ae [Adverse Effects] *Vaping

	**Abstract:** BACKGROUND: Studies demonstrated that adolescent e-cigarette use is associated with subsequent tobacco smoking, commonly referred to as the gateway effect. However, most studies only investigated gateways from e-cigarettes to tobacco smoking. This study replicates a cornerstone study revealing a positive association between both adolescent e-cigarette use and subsequent tobacco use; and tobacco and subsequent e-cigarette use in the Netherlands and Flanders., DESIGN: The longitudinal design included baseline (n=2839) and 6-month (n=1276) and 12-month (n=1025) follow-up surveys among a school-based cohort (mean age: 13.62). Ten high schools were recruited as a convenience sample. The analyses involved (1) associations of baseline e-cigarette use and subsequent tobacco smoking among never smokers; (2) associations of e-cigarette use frequency at baseline and tobacco smoking frequency at follow-up; and (3) the association of baseline tobacco smoking and subsequent e-cigarette use among non-users of e-cigarettes., FINDINGS: Consistent with prior findings, baseline e-cigarette use was associated with higher odds of tobacco smoking at 6-month (OR=1.89; 95% CI 1.05 to 3.37) and 12-month (OR=5.63; 95% CI 3.04 to 10.42) follow-ups. More frequent use of e-cigarettes at baseline was associated with more frequent smoking at follow-ups. Baseline tobacco smoking was associated with subsequent e-cigarette use (OR=3.10; 95% CI 1.58 to 6.06 at both follow-ups)., CONCLUSION: Our study replicated the positive relation between e-cigarette use and tobacco smoking in both directions for adolescents. This may mean that the gateway works in two directions, that e-cigarette and tobacco use share common risk factors, or that both mechanisms apply. Copyright © Author(s) (or their employer(s)) 2023. Re-use permitted under CC BY. Published by BMJ.
9151	Martinez-Loredo, Victor, Gonzalez-Roz, Alba, Dawkins, Lynne, Singh, Desmond, Murphy, James G., MacKillop, James (2022) Is E-cigarette Use Associated With Persistence or Discontinuation of Combustible Cigarettes? A 24-Month Longitudinal Investigation in Young Adult Binge Drinkers. Nicotine & tobacco research : official journal of the Society for Research on Nicotine and Tobacco; 24<br><br>**Keywords:**<br>Adult *Electronic Nicotine Delivery Systems Female Humans Male Smokers *Tobacco Products Tobacco Use United States Vaping/ep [Epidemiology] *Vaping Young Adult<br><br>**Abstract:** INTRODUCTION: It remains unclear whether electronic cigarette (e-cigarette) use promotes persistent combustible tobacco use or smoking discontinuation over time. Alcohol use is associated with a greater risk of adverse health effects of tobacco, and higher likelihood of e-cigarette use, making drinkers a high-priority subpopulation., AIMS AND METHODS: This study examined longitudinal patterns of combustible tobacco and e-cigarette use over 24 months in young adult binge drinkers. A pooled dataset of 1002 (58.5% female; M age = 22.14) binge drinkers from the United States (60%) and Canada (40%) was used. The primary outcomes were past month combustible tobacco and e-cigarette use. Nicotine dependence was measured using the Fagerstrom Test of Cigarette Dependence. Alcohol severity was measured using the Young Adult Alcohol Consequences Questionnaire. Latent transition analysis (LTA) was used to identify patterns of cigarette smoking and e-cigarette use over 24 months., RESULTS: The LTA yielded a four-class solution: (1) e-cigarettes-only users (prevalence over time: 7.75%-10.10%), (2) dual-product users (2.61%-9.89%), (3) combustible-only smokers (8.12%-20.70%), and (4) nonusers (61.66%-80.06%). Dual-product users predominantly transitioned to complete abstinence or exclusively e-cigarette use. In combustible-only smokers, the most common transition was to abstinence, followed by persistence of combustible-only status. At 24 months, 63% of e-cigarettes-only users transitioned to abstinence, with 37% continuing e-cigarettes-only use and 0% transitioning to dual or combustible cigarette use., CONCLUSIONS: Dual-product use in young adult binge drinkers was associated with discontinuation of combustible tobacco over time, and e-cigarette-only use was not associated with subsequent combustible tobacco use., IMPLICATIONS: These findings suggest that concurrent or exclusive e-cigarette use is not a risk factor for the persistence or development of combustible tobacco use in this subpopulation, with dual-product use reflecting a transitional pattern away from combustible use, toward discontinuation. Copyright © The Author(s) 2022. Published by Oxford University Press on behalf

	of the Society for Research on Nicotine and Tobacco. All rights reserved. For permissions, please e-mail: journals.permissions@oup.com.
9157	Mason, Tyler B., Tackett, Alayna P., Kechter, Afton, Leventhal, Adam M. (2022) Prospective Associations of Tobacco Weight Control Beliefs with E-Cigarette Use Patterns in the PATH Study. Substance use & misuse; 57<br><br>**Keywords:**<br>Adolescent *Electronic Nicotine Delivery Systems Humans Longitudinal Studies Tobacco *Tobacco Products Tobacco Use/ep [Epidemiology] Vaping/ep [Epidemiology] *Vaping<br><br>**Abstract:** INTRODUCTION: Some adolescents report using e-cigarettes (EC) for weight-related reasons, but longitudinal studies are lacking. This study examined associations between tobacco weight control beliefs and body mass index (BMI) with EC use patterns over one year., METHODS: Data from Waves 1 and 2 (September 2013 to October 2015) of the Population Assessment of Tobacco and Health (PATH) study were used. Questions about tobacco weight control beliefs, EC use patterns (never, ever, never to current, ever to current, current to current), cigarette and other tobacco product use, demographics, and BMI were examined among adolescent respondents across Wave 1 and Wave 2., RESULTS: Most adolescents were never EC users (85.8%). Prevalence of EC use patterns was low across categories of use (0.6%-5.3%). Higher BMI was associated with transition from ever but not current use at Wave 1 to current use at Wave 2. Greater baseline tobacco weight control beliefs and increases in tobacco weight control beliefs were associated with most EC use patterns compared to never use., CONCLUSIONS: Greater tobacco weight control beliefs were risk factors for e-cigarette initiation and maintenance among a nationally representative sample of adolescents. BMI was minimally associated with e-cigarette use patterns. Additional studies are needed to replicate and further examine these preliminary prospective associations between weight control beliefs and EC use.
9159	Mat Salleh, Rawaida, Baharom, Nizam, Siau, Ching Sin, Chan, Caryn Mei Hsien, Amit, Noh, Sia, Pei Yin, Wee, Lei Hum (2023) E-Cigarette Users' Profiles and Their Association with Identified Impacts of COVID-19 on Vaping among Young Adults in Malaysia. Healthcare (Basel, Switzerland); 11<br><br>**Keywords:**<br><br>**Abstract:** Electronic cigarettes (ECs) users' profiles and behaviors during the COVID-19 pandemic remain unclear. This cross-sectional study aimed to explore Malaysian EC users' profiles and their associations with related behaviors during the pandemic. The EC users (N = 351) were recruited from an official national vape entity. Respondents were predominantly of Malay ethnicity (90.6%), aged 31 to 35 years (27.6%), males (97.7%), married (68.7%), from Malaysia's west region states (63.5%) and tertiary educated (69.2%). The majority (80.3%) were non-dual users, and most purchased their vaping products online (77.2%), liked that they can vape while working at home (83.8%) and vaped more because of boredom (55.3%), had low and moderate nicotine addiction levels (94.9%), had low motivation level to quit EC use (92.6%) and were more likely to perceive that vaping did not increase the chances of complications from COVID-19. Respondents with moderate to high addiction levels had twice the odds of checking on their current EC supplies, whilst respondents with low motivation to quit had higher odds of using their tank/pod until the last drop and distancing from others when vaping. EC users should be encouraged to quit EC use, especially during the COVID-19 pandemic.
9166	Mattingly, Delvon T., Zavala-Arciniega, Luis, Hirschtick, Jana L., Meza, Rafael, Levy, David T., Fleischer, Nancy L. (2021) Trends in Exclusive, Dual and Polytobacco Use among U.S. Adults, 2014-2019: Results from Two Nationally Representative Surveys. International journal of environmental research and public health; 18

	**Keywords:**<br>Adult *Electronic Nicotine Delivery Systems Humans *Tobacco Products Tobacco Use/ep [Epidemiology] *Tobacco Use Disorder *Tobacco, Smokeless United States/ep [Epidemiology]<br><br>**Abstract:** Although increases in the variety of tobacco products available to consumers have led to investigations of dual/polytobacco use patterns, few studies have documented trends in these patterns over time. We used data from the 2014/2015 and 2018/2019 Tobacco Use Supplement to the Current Population Survey (TUS-CPS) and the 2015-2019 National Health Interview Survey (NHIS) to estimate trends in the following use patterns: exclusive use of cigarettes, electronic nicotine delivery systems (ENDS), other combustibles (cigars/cigarillos/little filtered cigars and traditional pipes/hookah), and smokeless tobacco (four categories); dual use (two product groups) of each product group with cigarettes (three categories); polyuse with cigarettes (all four product groups; one category); and dual/polyuse without cigarettes (one category). We estimated trends in product use patterns overall and by age, sex, and race/ethnicity using two-sample tests for differences in linear proportions. From 2014/2015 to 2018/2019, exclusive ENDS use increased, whereas cigarettes and ENDS dual use decreased. Furthermore, polyuse with cigarettes decreased, whereas dual/polyuse without cigarettes increased, with trends varying by age, sex, and race/ethnicity. Our findings suggest that patterns of dual/polyuse with and without cigarettes have changed in recent years, indicating the need for further surveillance of concurrent tobacco product use patterns.
9182	McLeish, Alison C., Hart, Joy L., Walker, Kandi L. (2023) Long-term impact of the COVID-19 pandemic on use behavior and risk perceptions of college student E-cigarette users. Journal of American college health : J of ACH;<br><br>**Keywords:**<br><br>**Abstract:** Objective: Work examining the long-term impact of the COVID-19 pandemic on e-cigarette use among college students is limited. Therefore, the current study examined differences in college student e-cigarette users' changes in use behavior and risk perceptions as the pandemic continues. Participants: 129 undergraduate current e-cigarette users (Mage = 19.68, SD = 1.85; 72.1% female; 85.3% White). Methods: Participants completed an online survey between October 2020 and April 2021. Results: In terms of changes in frequency of e-cigarette use, 30.5% of participants increased their use and 23.4% decreased their use. Greater e-cigarette dependence and anxiety were associated with increased use. Nearly half of e-cigarette users reported increased motivation to quit, and 32.5% had made at least one quit attempt. Conclusions: A substantial number of students increased their e-cigarette use as a result of the COVID-19 pandemic. Cessation efforts targeting increased anxiety and dependence may be useful in this population.
9183	McLeish, Alison C., Hart, Joy L., Wood, Lindsey A., Walker, Kandi L. (2022) Differences in young adults' perceptions of e-cigarettes by history of use. Journal of American college health : J of ACH;<br><br>**Keywords:**<br><br>**Abstract:** Objective: The current study examined group differences in college students' views on e-cigarettes in terms of safety, popularity, health risks, social acceptability, efficacy as a cessation aid, and promoting combustible cigarette use. Participants: 337 undergraduates (Mage = 20.84, SD = 3.79; 72.1% female; 68.5% White) divided into three groups: Never Users (n = 178), Ever Users (n = 108), and Current Users (n = 51). Methods: Participants completed questionnaires for course credit. Results: Compared to Ever and Never Users, Current Users believed e-cigarettes were safer, healthier, a useful tool to quit smoking, did not lead to combustible cigarette smoking, and that nicotine was less dangerous. Current Users also were less likely to endorse boosting social image as a motive for use. Conclusions: Public

	health campaigns could benefit from targeting current users' positive beliefs about e-cigarettes to reduce the prevalence of e-cigarette use among college students.
9189	Meienberg, A., Mayr, M., Vischer, A., Zellweger, M. J., Burkard, T. (2021) Smoking prevention in adolescents: A cross-sectional and qualitative evaluation of a newly implemented prevention program in Switzerland. BMJ Open; 11<br><br>**Keywords:**<br>adolescent *adolescent smoking article attitude to health awareness cigarette smoking cross-sectional study female heart infarction human lifestyle lung cancer male marketing mental stress peer pressure program acceptability qualitative research self report smoking habit *smoking prevention socioeconomics student Switzerland tobacco vaping vascular disease water pipe smoking multimedia<br><br>**Abstract:** Objectives In 2014, a hospital-based smoking prevention programme Nichtrauchen ist clever!' (NIC!) for adolescents aged 12-14 was initiated. The aim of the study was to evaluate participants' smoking behaviour and to explore the acceptance of the programme, and participants' awareness on smoking-related diseases and factors that promote smoking initiation. Design We performed a cross-sectional survey to evaluate participant's acceptance of the NIC! program and their smoking habits. A total of 1658 participants completed the survey between January 2016 and December 2019. A qualitative approach, including analysing feedback from students gathered at 77 prevention events between June 2014 and December 2019, was used to assess their view on reasons for smoking initiation and their knowledge about smoking-related diseases. Results Twenty-six per cent (429/1658) have already tried tobacco products (so called triers), specifically cigarettes, electronic (e)-cigarettes and shisha. The use of e-cigarettes was most popular among triers 58% (252/429). Eighty-eight per cent of participants (1408/1604) reported they had acquired good or excellent knowledge about smoking, and 89% (1439/1617) of participants stated that they intend not to smoke in the future. Particularly lung cancer and cancer in general, heart - and vascular diseases were frequently mentioned to be smoking related; where, on the other hand, a large number of relevant smoking-associated diseases were mentioned irregularly. Peer pressure, stress and coolness were identified as reasons for smoking initiation, whereas the influence of marketing and multimedia, as well as socioeconomic - and lifestyle-related factors on smoking behaviour was barely noticed. Conclusion NIC! had a high acceptance among the participants and a large number of students reported relevant gain of knowledge. We identified important knowledge-gaps relating to smoking initiation and smoking-related diseases, helping to improve further smoking prevention approaches.Copyright © Author(s) (or their employer(s)) 2021. Re-use permitted under CC BY-NC. No commercial re-use. See rights and permissions. Published by BMJ.
9204	Merz, William, Magraner, Jose, Gunge, Deepti, Advani, Ira, Crotty Alexander, Laura E., Oren, Eyal (2022) Electronic cigarette use and perceptions during COVID-19. Respiratory medicine; 200<br><br>**Keywords:**<br>COVID-19/ep [Epidemiology] *COVID-19 COVID-19 Testing *Electronic Nicotine Delivery Systems Humans Nicotine/ae [Adverse Effects] Pandemics SARS-CoV-2 *Tobacco Products Vaping/ep [Epidemiology] *Vaping<br><br>**Abstract:** This study was designed to examine how the COVID-19 pandemic changed e-cigarette user habits and risk perceptions. A nationally distributed 52-item questionnaire assessed nicotine e-cigarette use, perceptions, COVID-19 diagnosis, demographic data, and vaping habits among respondents aged 16-96 years (n = 565). Questions were developed in-house to assess vaping habits of users and risk perceptions of nicotine containing e-cigarette users and non-users both before and during the COVID-19 pandemic. Seventy-six percent of non-users believed that e-cigarette use would lead to worse COVID-19 symptoms, compared to 40% of e-cigarette users (P < 0.001). Twenty-eight percent of non-users also believed that e-cigarette users were more likely to be infected with SARS-CoV-2, versus 11% of e-cigarette users (P < 0.001). Fifty-eight percent of e-cigarette users described themselves as making no

	change in their e-cigarette usage, 10% decreased e-cigarette use, and 32% increased e-cigarette use during the pandemic. Twenty-five percent of users switched to vaping non-socially during the pandemic (P < 0.001). Sixty-seven percent of e-cigarette users replied that they would decrease or stop vaping if diagnosed with COVID and 31% said they would continue (P < 0.001). These findings reveal there are large differences in risk perception of e-cigarette use between users and non-users. Additionally, our findings characterize the habits of e-cigarette users during the COVID-19 pandemic, revealing users report steady to increased use, more caution in social settings, and would reduce usage if diagnosed with COVID-19. Copyright © 2022 The Author(s). Published by Elsevier Ltd.. All rights reserved.
9223	Mirbolouk, Mohammadhassan, Boakye, Ellen, Obisesan, Olufunmilayo, Osei, Albert D., Dzaye, Omar, Osuji, Ngozi, Erhabor, John, Stokes, Andrew C., El-Shahawy, Omar, Rodriguez, Carlos J., Hirsch, Glenn A., Benjamin, Emelia J., DeFilippis, Andrew P., Marie Robertson, Rose, Bhatnagar, Aruni, Blaha, Michael J. (2022) E-cigarette use among high school students in the United States prior to the COVID-19 pandemic: Trends, correlates, and sources of acquisition. Preventive medicine reports; 29<br><br>**Keywords:**<br><br>**Abstract:** Detailed description of the prevalence and sources of e-cigarettes among youth is needed to inform effective regulatory policies. We used the Youth Risk Behavior Surveillance System data (2015-2019) to assess trends in current (past-30-day-use) and frequent (>=10 days in past-30-days) e-cigarette use among United States high schoolers before the COVID-19 pandemic. First, we assessed trends overall and then stratified by participants' sociodemographic characteristics, use of other tobacco products, and experiences of psychosocial stress. We also evaluated past year quit attempts and the changing sources of e-cigarettes. Our sample size was 41,021 (15,356-2015; 12,873-2017; 12,792-2019). The prevalence of current e-cigarette use increased from 24.0% (95%CI:21.9%-26.3%) in 2015 to 32.7% (30.4%-35.1%) in 2019. The proportion of current users who reported frequent use also increased significantly from 22.6% (20.4%-24.8%) to 45.4% (42.7%-48.2%). Thus, an increasing proportion of US high school students who use e-cigarettes reported frequent use, indicating greater nicotine dependence. The increase in current and frequent e-cigarette use was more pronounced in youth who reported other substance use and psychosocial stressors such as bullying. Between 2017 and 2019, there was a decline in the proportion of youth who bought e-cigarettes online (6.9% to 3.2%) or from convenience stores (22.0% to 16.6%). Conversely, there was an increase in the proportion who borrowed (34.5% to 40.1%) or purchased e-cigarettes through other people (10.7% to 18.0%), indicating that most youth are evading age-related restrictions by obtaining e-cigarettes from other people. Finally, a considerable proportion of youth tobacco users are making quit attempts; 47.6% (45.1%-50.1%) in 2019. Copyright © 2022 The Authors.
9268	Moustafa, Afaf F., Rodriguez, Daniel, Pianin, Stephen H., Testa, Shannon M., Audrain-McGovern, Janet E. (2022) Dual Use of Nicotine and Cannabis Through Vaping Among Adolescents. American journal of preventive medicine; 63<br><br>**Keywords:**<br>Adolescent *Cannabis *Electronic Nicotine Delivery Systems Humans Nicotine Philadelphia/ep [Epidemiology] Prospective Studies Vaping/ep [Epidemiology] *Vaping<br><br>**Abstract:** INTRODUCTION: This study seeks to identify adolescent nicotine and cannabis vaping patterns and the characteristics of those adolescents who comprised each pattern., METHODS: This prospective longitudinal survey study measured the relationship between nicotine and cannabis vaping among 1,835 adolescents from 4 public high schools outside of Philadelphia, Pennsylvania. Adolescents completed in-classroom surveys, including questions of lifetime and past 30-day nicotine and cannabis vaping, at Wave 1 (fall 2016, ninth grade) and 6-month intervals for the following 36 months (fall 2019, 12th grade). Data were analyzed in 2021., RESULTS: A sequential processes growth mixture model revealed 4 latent conjoint classes of nicotine and cannabis vaping: early, declining dual use (Class 1: n=259); rapidly

	increasing dual use (Class 2: n=128); later, slower dual use (Class 3: n=313); and no use (Class 4: n=1,136). Increased odds of belonging to Class 1 and Class 2 versus belonging to Class 4 were significantly associated with cigarette smoking (OR=3.71, OR=2.21), alcohol use (OR=2.55, OR=4.39), peer vaping (OR=1.24, OR=1.20), sensation seeking (OR=1.03, OR=1.11), positive E-cigarette expectations (OR=1.21, OR=1.17), and cigar smoking (OR=2.39 Class 2 only). Increased odds of belonging to Class 3 versus Class 4 were significantly associated with alcohol use (OR=1.66), perceived benefits of E-cigarette use (OR=1.03), positive E-cigarette expectations (OR=1.08), depressive symptoms (OR=1.02), and sensation seeking (OR=1.03)., CONCLUSIONS: From middle to late adolescence, vaping of nicotine and cannabis develop in close parallel. Regulatory policy and prevention interventions should consider the interplay between these 2 substances during this period of adolescence. Copyright © 2022 American Journal of Preventive Medicine. Published by Elsevier Inc. All rights reserved.
9278	Mus, Sophia, Monzon, Jose, Islam, Farahnaz, Thrasher, James F., Barnoya, Joaquin (2023) First tobacco product tried and current use of cigarettes and electronic cigarettes among adolescents from Guatemala City. Salud publica de Mexico; 65<br><br>**Keywords:**<br>Humans Adolescent *Electronic Nicotine Delivery Systems Tobacco Guatemala Cross-Sectional Studies *Tobacco Products Flavoring Agents Tobacco Use<br><br>**Abstract:** OBJECTIVE: To assess how first experimenting with ciga-rettes or e-cigarettes is associated with current use in Gua-temala, a middle-income country with weak tobacco control and no e-cigarette regulations., MATERIALS AND METHODS: We surveyed students from private schools in Guatemala City, limiting analyses to ever users (n=1 026). Multinomial logistic models regressed current product use on first product used, adjusting for sociodemographics and friends and family use., RESULTS: The most common first product used was e-cigarettes (56%), followed by flavored cigarettes (24%) and regular cigarettes (20%). At the time of the survey, 4% were exclusive smokers, 37% were exclusive e-cigarette users, 18% dual users, and 40% had ever tried either but were not current users. Compared to those who first tried cigarettes, students who first tried e-cigarettes were less likely to be current smokers (RR=0.19 [CI: 0.11,0.31]) or dual users (RR=0.26 [CI: 0.14,0.49]) and students who first tried flavored cigarettes were more likely to be current smokers (RR=1.66 [CI=1.13,2.42])., CONCLUSIONS: In our sample, Guatemalan adolescents from private schools more frequently experiment and continue to use e-cigarettes than cigarettes. There is urgent need to implement e-cigarette restrictions in addition to tobacco control policy implementation.
9304	Nguyen, Nhung, Koester, Kimberly A., Kim, Minji, Watkins, Shannon Lea, Ling, Pamela M. (2023) "I'm both smoking and vaping": a longitudinal qualitative study of US young adults who tried to quit smoking cigarettes by using electronic cigarettes. Tobacco control;<br><br>**Keywords:**<br><br>**Abstract:** OBJECTIVE: To describe how young adults use electronic cigarettes (electronic nicotine delivery systems (ENDS)) for smoking cessation and reasons why they may or may not successfully quit smoking., METHODS: Longitudinal qualitative data were collected annually from 2017 to 2019 for 25 young adult tobacco users (aged 18-29 years) in California (USA) who used ENDS to quit/reduce smoking. Thematic and trajectory analyses were used to identify key within-person and between-person changes in tobacco/nicotine use over time., RESULTS: Five types of tobacco use transition were identified among baseline dual users of cigarettes and ENDS: sustained dual use without reduced smoking (n=8), transition to exclusive daily ENDS use (n=6), sustained dual use with reduced smoking (n=5), transition back to exclusive smoking (n=4) and transition to neither smoking nor vaping (n=2). Participants ' ENDS use behaviour varied over time in terms of vaping quantity and device characteristics (eg, changing nicotine concentrations/flavours, switching between multiple devices). Three themes that related to successfully

	replacing cigarettes with ENDS were perceived positive physical effects, perceived satisfaction and enjoyment and context changes. Four themes for unsuccessful replacement were perceived negative physical discomforts, perceived addictiveness and harm, unsatisfactory substitution for cigarettes and device malfunction., CONCLUSIONS: Young adults' experiences with using ENDS as a smoking cessation aid were highly variable. Adequate nicotine delivery and perceived safety and benefits contributed to successfully reducing or quitting cigarettes. Providing behavioural counselling and standardising ENDS products may enhance cessation for young adults. Copyright © Author(s) (or their employer(s)) 2023. No commercial re-use. See rights and permissions. Published by BMJ.
9316	Noar, Seth M., Gottfredson, Nisha C., Kieu, Talia, Rohde, Jacob A., Hall, Marissa G., Ma, Haijing, Fendinger, Nicholas J., Brewer, Noel T. (2022) Impact of Vaping Prevention Advertisements on US Adolescents: A Randomized Clinical Trial. JAMA network open; 5<br><br>**Keywords:**<br>Adolescent Advertising *Cigarette Smoking *Electronic Nicotine Delivery Systems Female Humans Male Tobacco Use Vaping/pc [Prevention & Control] *Vaping<br><br>**Abstract:** Importance: Understanding whether prevention advertisements reduce susceptibility to vaping is important owing to concerning levels of adolescent vaping., Objective: To examine whether vaping prevention advertisements from the US Food and Drug Administration (FDA) national Real Cost campaign lead to lower susceptibility to vaping among adolescents., Design, Setting, and Participants: For this 3-group randomized clinical trial with parallel assignment, participants were US adolescents aged 13 to 17 years who were susceptible to vaping or current e-cigarette users, recruited from online panels. Adolescents were randomized to 1 of 2 Real Cost vaping prevention trial groups (health harms- or addiction-themed advertisements) or to a control group (investigator-created neutral videos about vaping). Adolescents completed 4 weekly online surveys at visits 1 to 4 over a 3-week period. Data were analyzed from December 1, 2021, to August 25, 2022., Interventions: Adolescents saw 3 randomly ordered 30-second video advertisements online at each of 3 weekly study visits (visits 1, 2, and 3)., Main Outcomes and Measures: The primary trial outcome was susceptibility to vaping. Surveys also assessed susceptibility to smoking cigarettes to examine any spillover effects of vaping prevention advertisements on smoking outcomes. Both susceptibility measures had 3 items and ranged from 1 (indicating not susceptible) to 4 (indicating highly susceptible). The primary analyses compared Real Cost groups (combined) with the control group, while exploratory analyses compared the Real Cost groups with each other., Results: Participants were 1514 adolescents (1140 [75.3%] boys; mean [SD] age, 15.22 [1.18] years), including 504 randomized to the Real Cost health harms group, 506 randomized to the Real Cost addiction group, and 504 randomized to the control group. Adolescents in the Real Cost groups (combined) had lower susceptibility to vaping at visit 4 than those in the control group (b = -0.21; 95% CI, -0.32 to -0.10). The Real Cost groups did not differ from one another on susceptibility to vaping (visit 4: b = -0.05; 95% CI, -0.17 to 0.07). Adolescents in the Real Cost groups (combined) also had lower susceptibility to smoking cigarettes than those in the control group (b = -0.21; 95% CI, -0.32 to -0.10). For both vaping and smoking, Real Cost groups had less positive attitudes (vaping: b = -0.27; 95% CI, -0.40 to -0.14; smoking: b = -0.23; 95% CI, -0.39 to -0.08) compared with the control group., Conclusions and Relevance: These findings suggest that vaping prevention advertisements from the FDA Real Cost campaign led to lower adolescent susceptibility to vaping and had beneficial spillover effects on cigarette smoking outcomes. Tobacco prevention campaigns can help reduce youth tobacco use., Trial Registration: ClinicalTrials.gov Identifier: NCT04836455.
9370	Orton, Sophie, Taylor, Lauren, Laing, Libby, Lewis, Sarah, Ussher, Michael, Coleman, Tim, Cooper, Sue (2022) Are E-cigarettes associated with postpartum return to smoking? Secondary analyses of a UK pregnancy longitudinal cohort. BMJ open; 12<br><br>**Keywords:**<br>*Electronic Nicotine Delivery Systems Female Humans Postpartum Period Pregnancy Smoking/ep

	[Epidemiology] *Smoking Cessation United Kingdom/ep [Epidemiology]<br><br>**Abstract:** OBJECTIVES: Postpartum return to smoking (PPRS) is an important public health problem. E-cigarette (EC) use has increased in recent years, and in a contemporary UK pregnancy cohort, we investigated factors, including ECs use, associated with PPRS., DESIGN: Secondary analyses of a longitudinal cohort survey with questionnaires at baseline (8-26 weeks' gestation), late pregnancy (34-36 weeks) and 3 months after delivery., SETTING: 17 hospitals in England and Scotland in 2017., PARTICIPANTS: The cohort recruited 750 women who were current or recent ex-smokers and/or EC users. A subgroup of women reported being abstinent from smoking in late pregnancy (n=162, 21.6%), and of these 137 (84.6%) completed the postpartum questionnaire and were included in analyses., OUTCOME MEASURES: Demographics, smoking behaviours and beliefs, views and experience of ECs and infant feeding., RESULTS: 35.8% (95% CI 28% to 44%) of women reported PPRS. EC use in pregnancy (adjusted OR 0.34, 95% CI 0.13 to 0.85) and breast feeding (adjusted OR 0.06, 95% CI 0.02 to 0.24) were inversely associated with PPRS, while household member smoking at 3 months post partum was positively associated with PPRS (adjusted OR 11.1, 95% CI 2.47 to 50.2)., CONCLUSION: EC use in pregnancy could influence PPRS. Further research is needed to confirm this and investigate whether ECs could be used to prevent PPRS. Copyright © Author(s) (or their employer(s)) 2022. Re-use permitted under CC BY. Published by BMJ.
9371	Osibogun, Olatokunbo, Bursac, Zoran, Maziak, Wasim (2022) Longitudinal transition outcomes among adult dual users of e-cigarettes and cigarettes with the intention to quit in the United States: PATH Study (2013-2018). Preventive medicine reports; 26<br><br>**Keywords:**<br><br>**Abstract:** Many adult dual users of e-cigarettes and cigarettes in the United States report using e-cigarettes with the intention to quit (ITQ) smoking. This study examined transition outcomes among adult dual users of e-cigarettes and cigarettes with the ITQ compared to mono cigarette smokers with ITQ. We conducted a longitudinal analysis of 3,542 adults aged >= 18 years with data from Waves 1 and 4 of the United States Population Assessment of Tobacco and Health study (2013-2018) between May 2021 and January 2022. Current dual users (e-cigarettes and cigarettes use on >= 20 days in the past month) with the ITQ were compared to current mono cigarette smokers with the ITQ for transition outcomes (cessation, mono e-cigarette, mono cigarette and dual use) three years later. We conducted multinomial logistic regression modeling adjusting for potential confounders and reported the adjusted relative risk ratios (aRRR) with 95% confidence intervals (CI) for the transition outcomes. Approximately 10.7% (7.8-14.3) of dual users with the ITQ (in 2013) reported cessation (no past-month use of any tobacco) three years later, compared to 16.1% (14.6-17.7) of mono cigarette smokers. Dual users were 83% and 79% less likely to transition to cessation (aRRR: 0.17, 95% CI:0.09-0.32) or mono cigarette use (0.21, 0.14-0.32), respectively, compared to mono cigarette smokers. Our findings show that in a real-world scenario, dual e-cigarette and cigarette use may hinder rather than facilitate smoking cessation among those interested in quitting. This needs consideration when assessing the population impact of e-cigarettes and their role in harm reduction. Copyright © 2022 The Authors.
9372	Osibogun, Olatokunbo, Chapman, Simon, Peters, Matthew, Bursac, Zoran, Maziak, Wasim (2022) E-cigarette Transitions Among US Youth and Adults: Results from the Population Assessment of Tobacco and Health Study (2013-2018). Journal of prevention (2022); 43<br><br>**Keywords:**<br>Adolescent Adult Cross-Sectional Studies *Electronic Nicotine Delivery Systems Humans Tobacco *Tobacco Products United States/ep [Epidemiology] Vaping/ep [Epidemiology] *Vaping Young Adult<br><br>**Abstract:** Currently, the debate surrounding the regulation of e-cigarettes focuses mainly on the size of e-

	cigarettes' potentially beneficial effects (i.e., adult cessation) versus their unwarranted effects (i.e., initiation among tobacco-naive adolescents). Therefore, we investigated the relative scale of e-cigarette use transitions in the United States. We reported cross-sectional weighted prevalence estimates of past-month e-cigarette use by ever cigarette use from Waves 1-4 of the Population Assessment of Tobacco and Health study (2013-2018) among youth (12-17 years) and adults (>= 18 years). We also examined past-month e-cigarette mono and dual transitions related to cigarette smoking and reported the longitudinal weighted prevalence across waves. Among youth new e-cigarette users, the proportion of never-cigarette smokers increased from 24.1 in Wave 1 (n = 418) to 51.4% in Wave 4 (n = 310) (p < 0.0001 for trend). Of youth past-month e-cigarette mono-users in Wave 1 (n = 151), 15.2% transitioned to cigarette mono-use and 8.2% dual-use at Wave 2 or 3 or 4, compared to 60.2% no tobacco use and 16.4% e-cigarette mono-use. Among young adult past-month dual-users (18-24 years; n = 684), 22.6% transitioned to no tobacco use, 60.1% continued cigarette use, 11.4% dual use, and 5.9% e-cigarette mono-use. Among adult dual-users >= 25 years old (n = 1560), 13.6% transitioned to no tobacco use, 71.3% cigarette mono-use, 9.0% dual-use, and 6.1% e-cigarette mono-use. Transition to cigarette mono-use and continued dual-use were common among adult past-month e-cigarette users, while e-cigarette initiation was common among youth never-cigarette smokers. These findings contrast with data from other countries showing limited evidence of e-cigarette initiation among youth never cigarette smokers. Both e-cigarette and cigarette use should be addressed in youth and adults, given the potential for dual use in both populations. Copyright © 2022. The Author(s), under exclusive licence to Springer Science+Business Media, LLC, part of Springer Nature.
9405	Parnham, Jennie C., Vrinten, Charlotte, Rado, Marta K., Bottle, Alex, Filippidis, Filippos T., Laverty, Anthony A. (2023) Multistate transition modelling of e-cigarette use and cigarette smoking among youth in the UK. Tobacco control;<br><br>**Keywords:**<br><br><br>**Abstract:** INTRODUCTION: E-cigarette use remains a controversial topic, with questions over how people transition between e-cigarette use and cigarette smoking. This paper examined transitions into and out of nicotine product use in a representative sample of UK youth., METHODS: We used Markov multistate transition probability models on data from 10 229 participants (10-25 years old) in the UK Household Longitudinal Study (2015-2021). We used four product use states ('never', 'non-current use', 'e-cigarette only' and 'smoking and dual use') and estimated likelihood of transitions according to sociodemographic characteristics., RESULTS: Among participants who had never used nicotine products, most were still non-users a year later (92.9% probability; 95% CI 92.6%, 93.2%); a small proportion transitioned to using e-cigarettes only (4.0%; 95% CI 3.7%, 4.2%) and cigarettes (2.2%; 95% CI 2.0%, 2.4%). Those aged 14-17 years were the most likely to start using a nicotine product. E-cigarette use was less persistent overtime than cigarette smoking, with a 59.1% probability (95% CI 56.9%, 61.0%) of e-cigarette users still using after 1 year compared with 73.8% (95% CI 72.1%, 75.4%) for cigarette smoking. However, there was a 14% probability (95% CI 12.8%, 16.2%) that e-cigarette users went onto smoke cigarettes after 1 year, rising to 25% (95% CI 23%, 27%) after 3 years., CONCLUSION: This study found that although overall nicotine product use was relatively rare, participants were more likely to experiment with e-cigarette use than cigarette smoking. This was mostly not persistent over time; however, approximately one in seven transitioned to cigarette smoking. Regulators should aim to deter all nicotine product use among children. Copyright © Author(s) (or their employer(s)) 2023. Re-use permitted under CC BY. Published by BMJ.
9406	Pascoe, M., Nagia, S., Atal, N., Gadway, H., Huizenga, K., Bailey, B. (2023) Have e-cigarettes created a new crop of young adult substance users? Overlap between e-cigarette, traditional cigarette, and alcohol use. Journal of Substance Use; 28<br><br>**Keywords:**

	adult *alcohol consumption alcoholic beverage American Indian article behavioral risk factor surveillance system binge drinking *cigarette smoking demographics educational status European American female high school human major clinical study male nonhuman tobacco United States young adult *electronic cigarette<br><br>**Abstract:** Background: Electronic cigarette use has increased among U.S. young adults since the early 2000s, but it is unclear whether use overlaps with traditional substance use or has produced a new at-risk population. Our goal was to compare trends in use of different legal substances and examine the association between e-cigarette use, demographic factors, and the use of other substances. Method(s): Data from the nationally-representative 2016-2019 U.S. Behavioral Risk Factor Surveillance System (BRFSS) from adults aged 18-24 were used (N = 64,604). Result(s): Over the study period, e-cigarette use increased significantly, but traditional cigarette smoking and alcohol consumption decreased. Those who had ever used e-cigarettes were significantly more likely to be single, White or Native American, and report high school being their highest level of education. The majority of current daily e-cigarette users reported never having smoked traditional cigarettes (62.3%), not being a current traditional cigarette smoker (75.9%), and not being a binge drinker (59.3%). Conclusion(s): As young adult e-cigarette use has increased, users are largely individuals who are not otherwise engaging in legal substance use. Future studies should examine factors leading to uptake of e-cigarette use among young adults in order to address this growing public health concern.Copyright © 2021 Taylor & Francis Group, LLC.
9410	Patanavanich, Roengrudee, Worawattanakul, Methavee, Glantz, Stanton (2022) Longitudinal bidirectional association between youth electronic cigarette use and tobacco cigarette smoking initiation in Thailand. Tobacco control;<br><br>**Keywords:**<br><br>**Abstract:** INTRODUCTION: This study quantifies the longitudinal association between e-cigarette use and subsequent conventional cigarette initiation and vice versa among Thai youths., METHODS: Data from a longitudinal survey of 6045 Thai seventh grade students with baseline in 2019 and the 12-month follow-up in 2020 were analysed using complex survey multivariate logistic regressions to assess whether e-cigarette use was associated with subsequent cigarette smoking (ever, current and dual product users at follow-up) among baseline never smokers., RESULTS: Consistent with prior findings from other countries, among those who had never smoked cigarettes at baseline, ever e-cigarette users were more likely to try cigarette smoking (adjusted OR 4.44; 95% CI 2.23 to 8.86; p<0.001), or become dual users (adjusted OR 5.31; 95% CI 2.63 to 10.74; p<0.001) 1 year later. Baseline current e-cigarette users were more likely to become ever smokers (adjusted OR 5.37; 95% CI 1.82 to 15.90; p=0.005), current smokers (OR 3.92; 95% CI 1.69 to 9.14; p=0.003) and dual product users (adjusted OR 6.96; 95% CI 1.54 to 31.38; p=0.015) at the 12-month follow-up than non-e-cigarette users. Similarly, among never e-cigarette users at baseline, ever cigarette smoking were more likely to try e-cigarettes (adjusted OR 3.38; 95% CI 1.66 to 6.88; p=0.002), currently use e-cigarettes (adjusted OR 2.75; 95% CI 1.47 to 5.13; p=0.003) and currently use both e-cigarettes and cigarettes (adjusted OR 4.87; 95% CI 2.92 to 8.13; p<0.001) at the follow-up than never smokers. Among never e-cigarette users at baseline, current-cigarette smoking were more likely to try e-cigarettes (adjusted OR 6.21; 95% CI 2.58 to 14.95; p<0.001), currently use e-cigarettes (adjusted OR 2.80; 95% CI 1.27 to 6.14; p=0.014) and currently use both e-cigarettes and cigarettes (adjusted OR 7.70; 95% CI 3.45 to 17.19; p<0.001) at the follow-up than never smokers., CONCLUSIONS: This longitudinal study in Asian low-income and middle-income countries supports the prospective association of youth e-cigarette use with subsequent smoking initiation and youth cigarette use with subsequent e-cigarette initiation that is similar to that observed in high-income Western countries. Copyright © Author(s) (or their employer(s)) 2022. No commercial re-use. See rights and permissions. Published by BMJ.
9411	Patel, Minal, Simard, Bethany J., Benson, Adam F., Donovan, Emily M., Pitzer, Lindsay (2023) Measuring the impact of state and local Tobacco 21 policies in the United States: A longitudinal study of youth and

	young adults ages 15-21. Nicotine & tobacco research : official journal of the Society for Research on Nicotine and Tobacco; 25<br><br>**Keywords:**<br>Humans Adolescent Young Adult United States/ep [Epidemiology] Smoking/px [Psychology] *Electronic Nicotine Delivery Systems Tobacco Longitudinal Studies *Tobacco Products Policy<br><br>**Abstract:** INTRODUCTION: Tobacco 21 (T21) policies, which prohibit tobacco sales to individuals under 21, aim to reduce youth tobacco use by limiting youth access to these products. Little, however, is known about the longitudinal effect of T21 policies on youth tobacco use behaviors at the national level., METHODS: Participants aged 15-21 years from a longitudinal study conducted between May 2014 and May 2019 (n = 13,990) were matched to geocoded T21 policies. Generalized linear mixed models examined the association of direct and bordering T21 policy exposure and cigarette and e-cigarette use and intention to use, accounting for individual characteristics., RESULTS: Controlling for sociodemographic and psychosocial covariates, there were statistically significant positive associations between T21 exposure and e-cigarette use (OR = 1.45 [1.03,2.06], p < .003) and intention to use e-cigarettes (OR = 1.54 [1.05,2.26], p < .027). We found no association between T21 policy exposure and cigarette use or intention to use cigarettes. Furthermore, exposure to T21 policies did not significantly modify the relationship between age and either cigarette outcome., CONCLUSIONS: This is the first longitudinal study to evaluate state and local T21 policies at the national level. Our analyses demonstrate that existing T21 policies are not sufficient to reduce youth tobacco use and intentions to use, and suggest that T21 policies need to be a part of a comprehensive tobacco policy landscape. Our findings suggest further research is warranted on state and local T21 policy enforcement and implementation, including how T21 may differentially impact cigarette and e-cigarette use, and may have implications for the federal T21 policy., IMPLICATIONS: This research evaluates state and local T21 policies in the United States longitudinally, finding that T21 policies are not sufficient to stem e-cigarette use among adolescents and young adults. These findings support further policy action and suggest that local and state T21 policies are not sufficient to reduce tobacco use and rather, need to be part of a broader, more comprehensive set of tobacco control policies. Further research on enforcement and implementation challenges of T21 policies and the impacts of the new federal T21 policy is warranted. Copyright © The Author(s) 2022. Published by Oxford University Press on behalf of the Society for Research on Nicotine and Tobacco. All rights reserved. For permissions, please e-mail: journals.permissions@oup.com.
[truncated: 502,113 more chars]
